# Supplementary material for: Hip stabilization in an australopithecine-like hip: the influence of shape on muscle activation
Source: Biol Open. 2025 Jun 20;14(6):bio061931. doi: 10.1242/bio.061931 (PMC12208404; doi:10.1242/bio.061931)
Supplement: Supplementary information [file biolopen-14-061931-s1.pdf]

Supplemental Information

Fig. S1. Kinematic comparison to previous work

Joint angles are shown in the following five figures. All trials for the ADL human-like configuration are shown with blue dashed lines. The average is shown with a solid blue line while the values from Winter [74] at natural cadence are shown with a solid black line (where applicable).

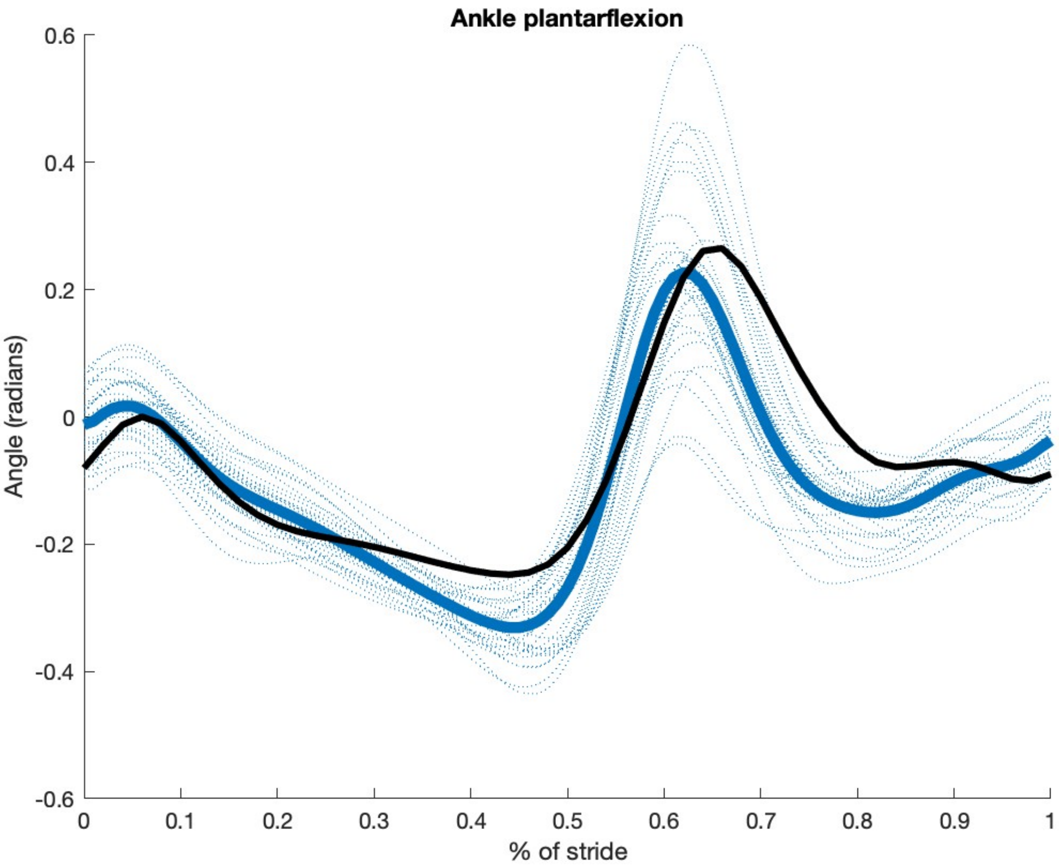

Fig. S1A

Biology Open • Supplementary information

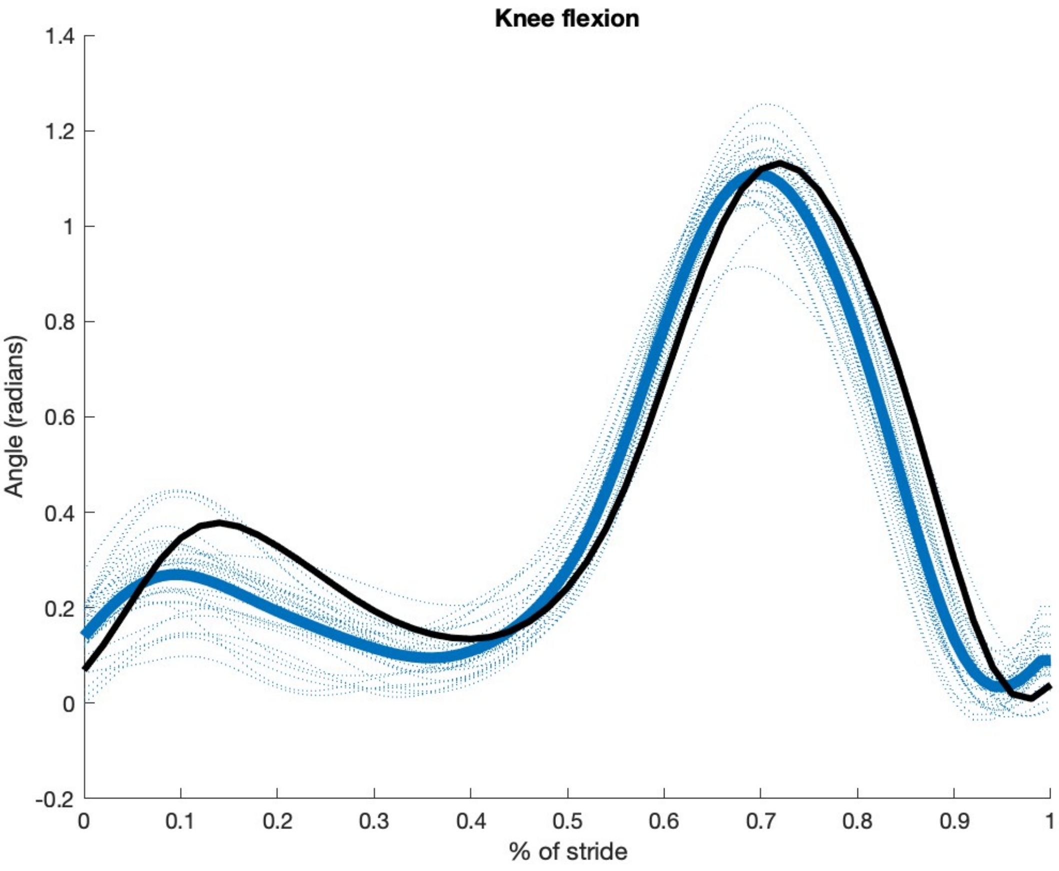

Fig. S1B

Biology Open • Supplementary information

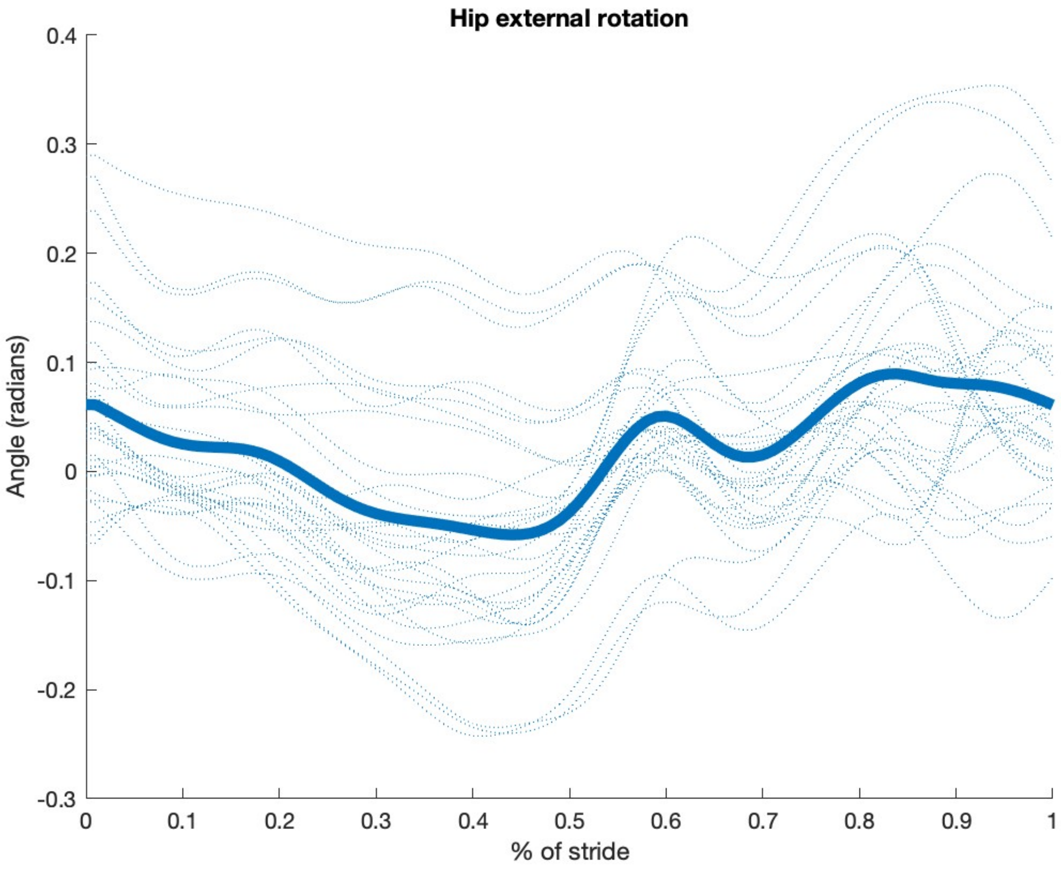

Fig. S1C

Biology Open • Supplementary information

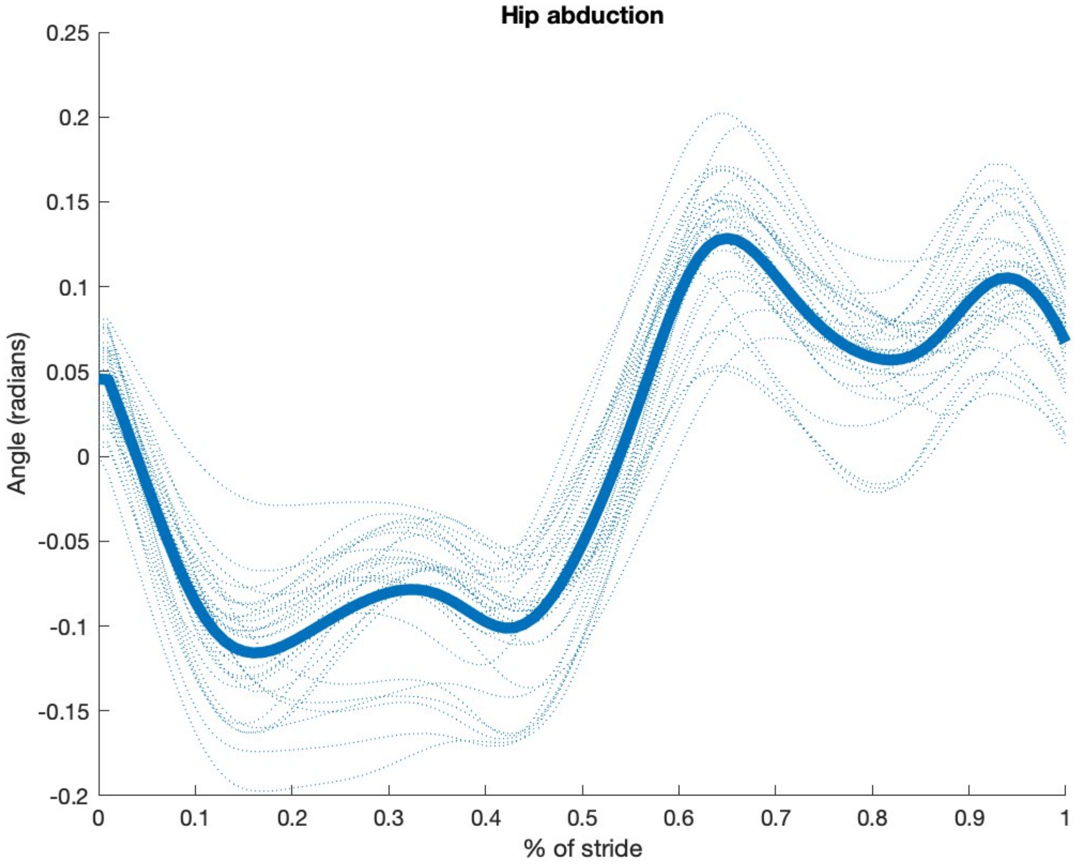

Fig. S1D

Biology Open • Supplementary information

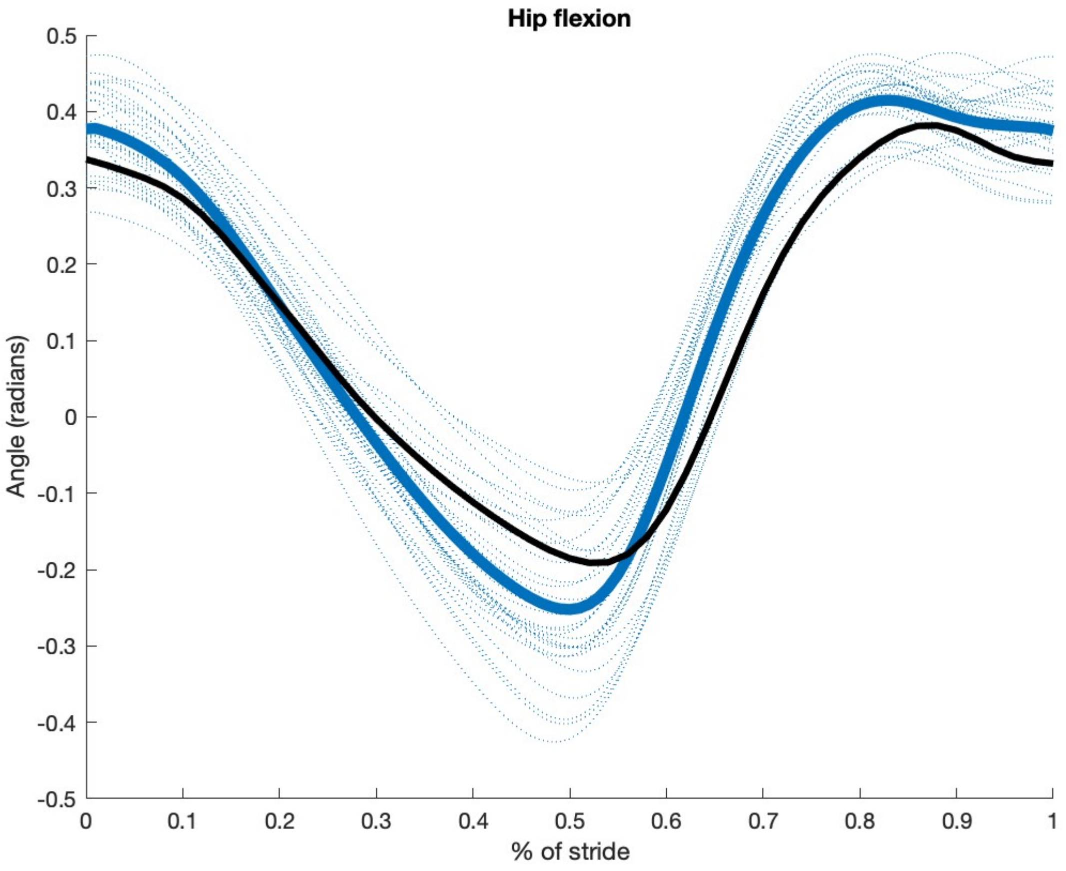
Hip flexion
0.5
0.4
0.3
0.2
0.1
0
-0.1
-0.2
-0.3
-0.4
-0.5
Angle (radians)
0
0.1
0.2
0.3
0.4
0.5
0.6
0.7
0.8
0.9
1
% of stride

Fig. S1E

Fig. S2. Gluteal muscle activation compared to previous work

Activation of the gluteal muscles compared to previously published data [64]. All trials for the ADL human-like configuration are shown with blue dashed lines. The average is shown with a solid blue line while the values from Sylvester et al. are shown with a solid black line.

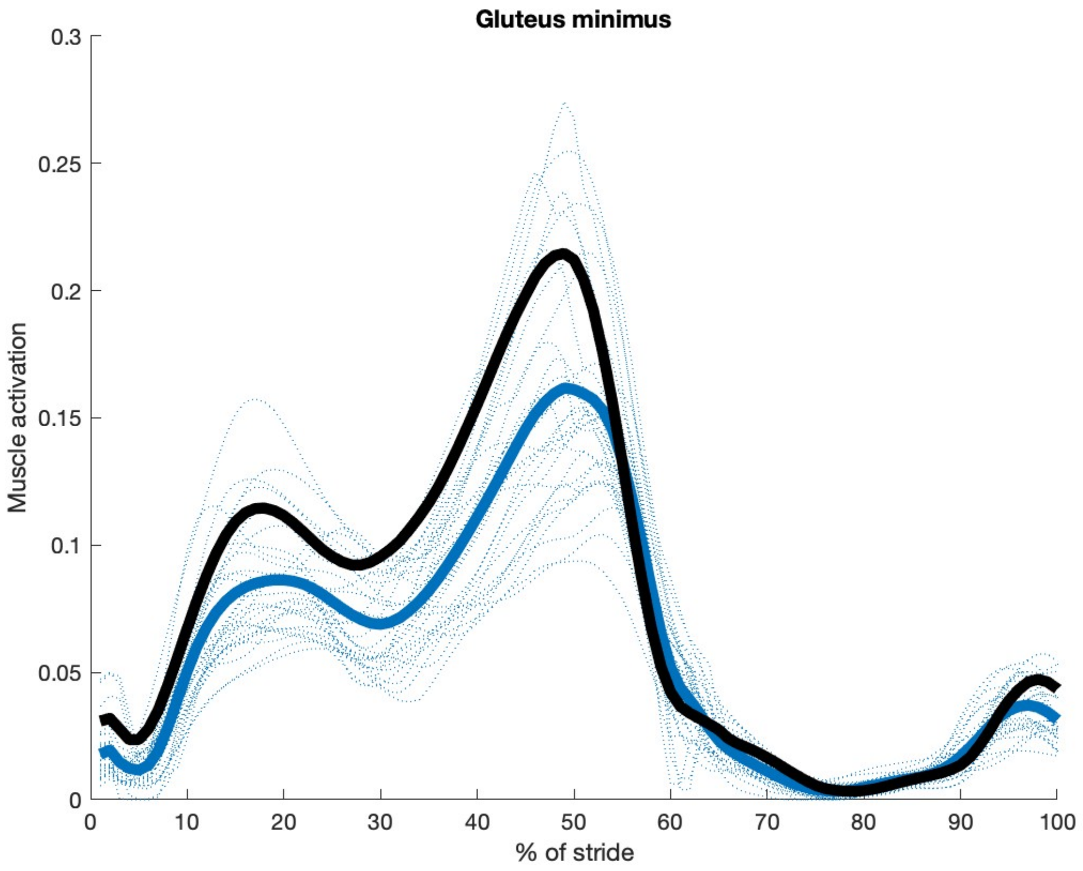

Fig. S2A

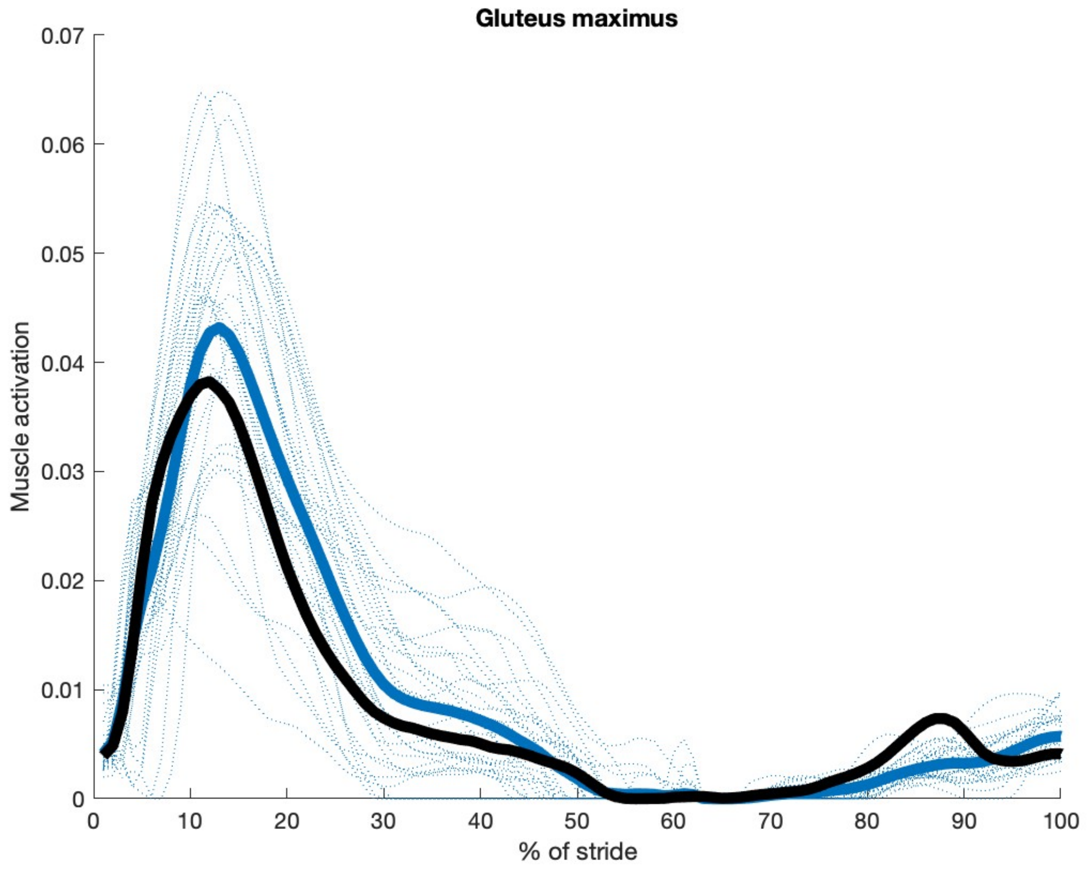

Fig. S2B

Biology Open • Supplementary information

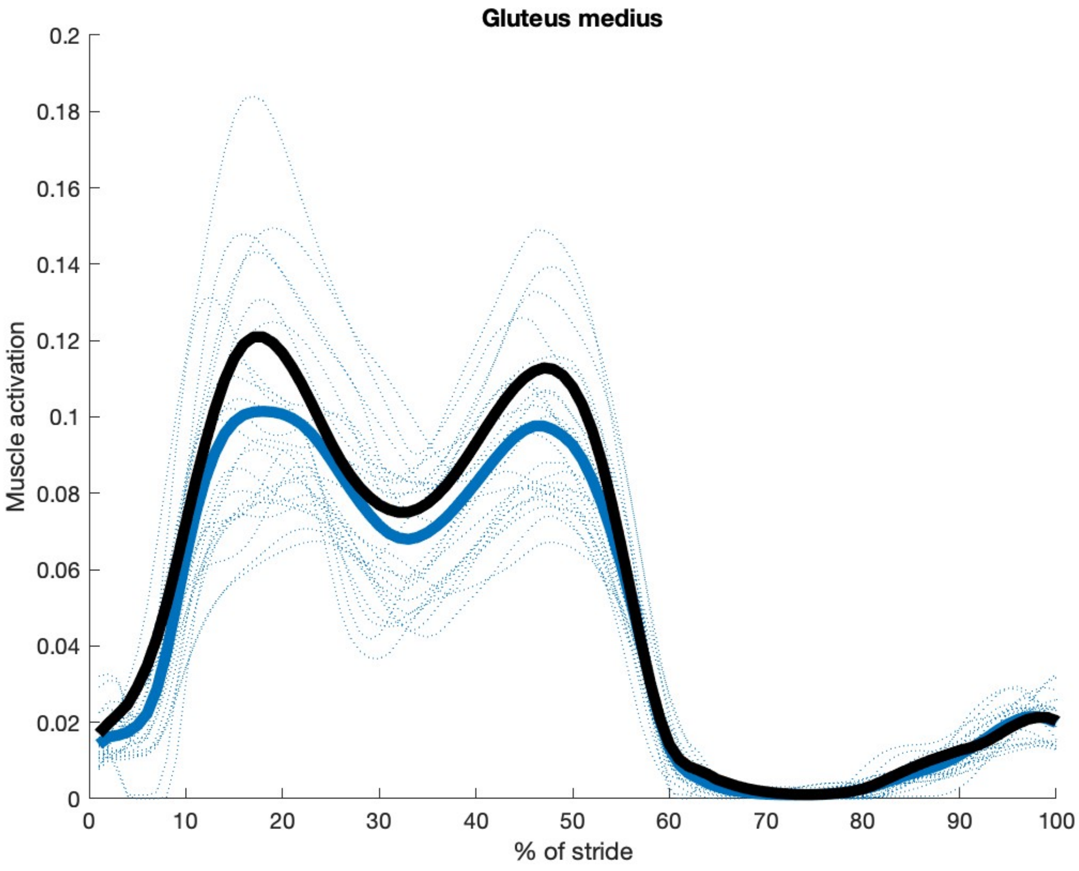

Fig. S2C

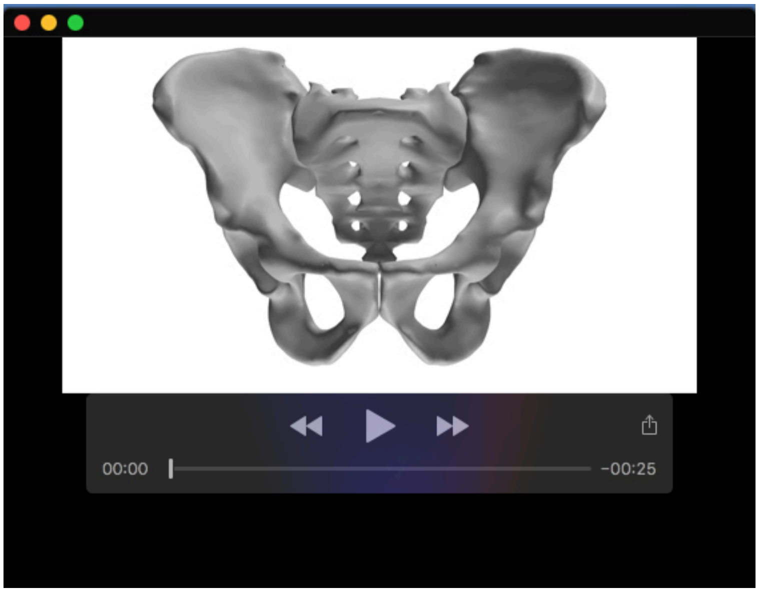

Movie 1.

Biology Open • Supplementary information

Fig. S3. Muscle element moment arms

The moment arms over which forces act about joints can be useful in visualizing and evaluating the relative effects of various parts of a structure (such as the hip) in static analyses. Consequently, important early research used them in comparisons among forms. Inverse dynamics solutions such as the one utilized in this study do not explicitly calculate moment arms. AnyBody has implemented a method of back-calculating moment arms and we provide this information for all lower limb muscle elements and the three degrees of rotation about the hip for both the modern human and australopithecine-like ADL models in spreadsheet formatted files. Moment arms are calculated for each muscle element moving through an idealized range of motion: 20 deg of extension to 90 deg of flexion; 30 deg of adduction to 50 deg of abduction; 40 deg of internal rotation to 40 deg of internal rotation.

Additional muscle activation plots

As with Figure 5 in the main text, all trials for the ADL human-like configuration are shown with blue dashed lines. The average for the ADL human-like (blue) and ADL australopithecine-like (gold) are shown with solid lines.

Group A muscles arise on the pelvis and insert on the femur proximal to the lesser trochanter (or proximal to the femoral shaft). They include (AnyBody muscle identifier): Iliacus, ObturatorExternus, ObturatorInternus, QuadratusFemoris, Piriformis, GemellusInferior, GemellusSuperior, GluteusMaximus, GluteusMedius, GluteusMinimus. The gluteal muscle activations are shown in Figure 5. Plots for the other Group A muscles are shown below.

Group B muscles cross the hip joint but arise either superiorly to the pelvis (PsoasMajor) or insert distally to the lesser trochanter. This latter set includes: AdductorBrevis, AdductorLongus, AdductorMagnus, BicepsFemorisCaputLongum, Sartorius, RectusFemoris, Gracilis, Semitendinosus, Semimembranosus, TensorFasciaeLatae. Plots for the Group B muscles are shown below.

Group C muscles are lower limb muscles, but do not cross the hip. They include: BicepsFemorisCaputBreve, ExtensorDigitorumLongus, ExtensorHallucisLongus, FlexorDigitorumLongus, FlexorHallucisLongus, GastrocnemiusLateralis, GastrocnemiusMedialis, Pectineus, PeroneusBrevis, PeroneusLongus, Plantaris, Popliteus, SoleusLateralis, SoleusMedialis, TibialisAnterior, TibialisPosterior, VastusIntermedius, VastusLateralis, VastusMedialis. We do not provide plots of muscle activation for these muscles.

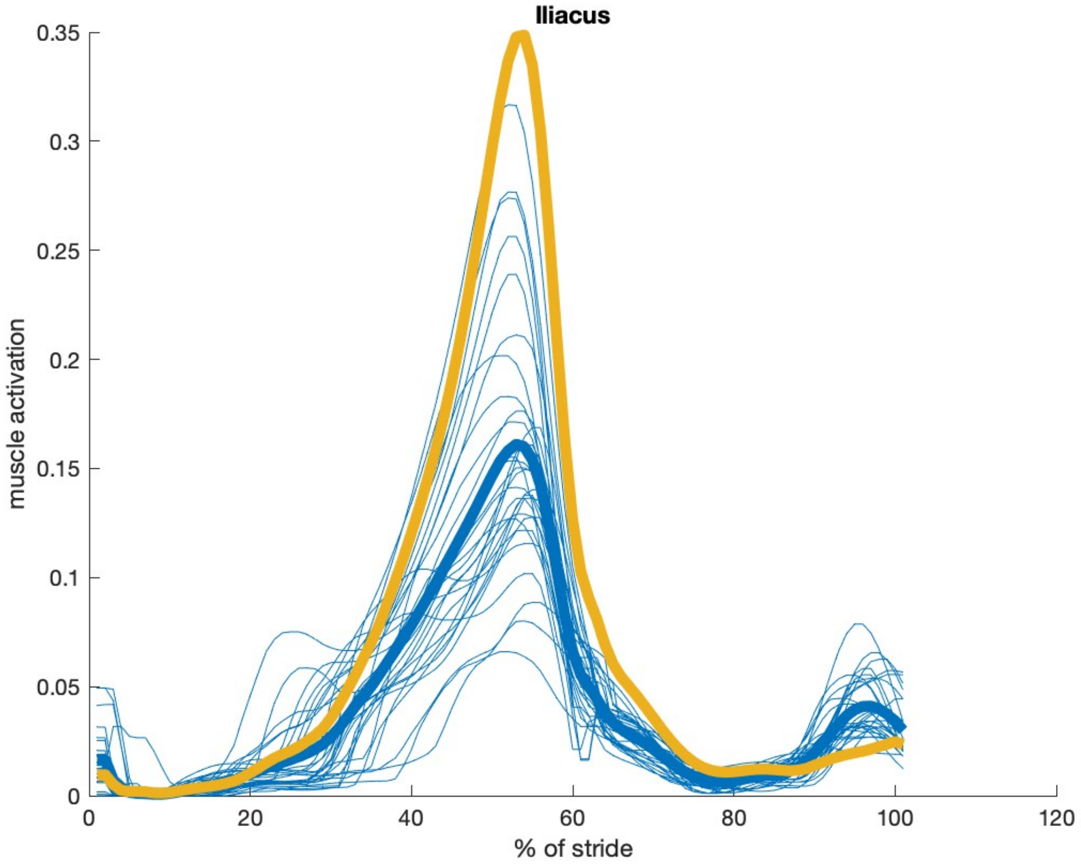
Iliacus
0.35
0.3
0.25
0.2
0.15
0.1
0.05
0
muscle activation
0
20
40
60
80
100
120
% of stride

Fig. S3A.

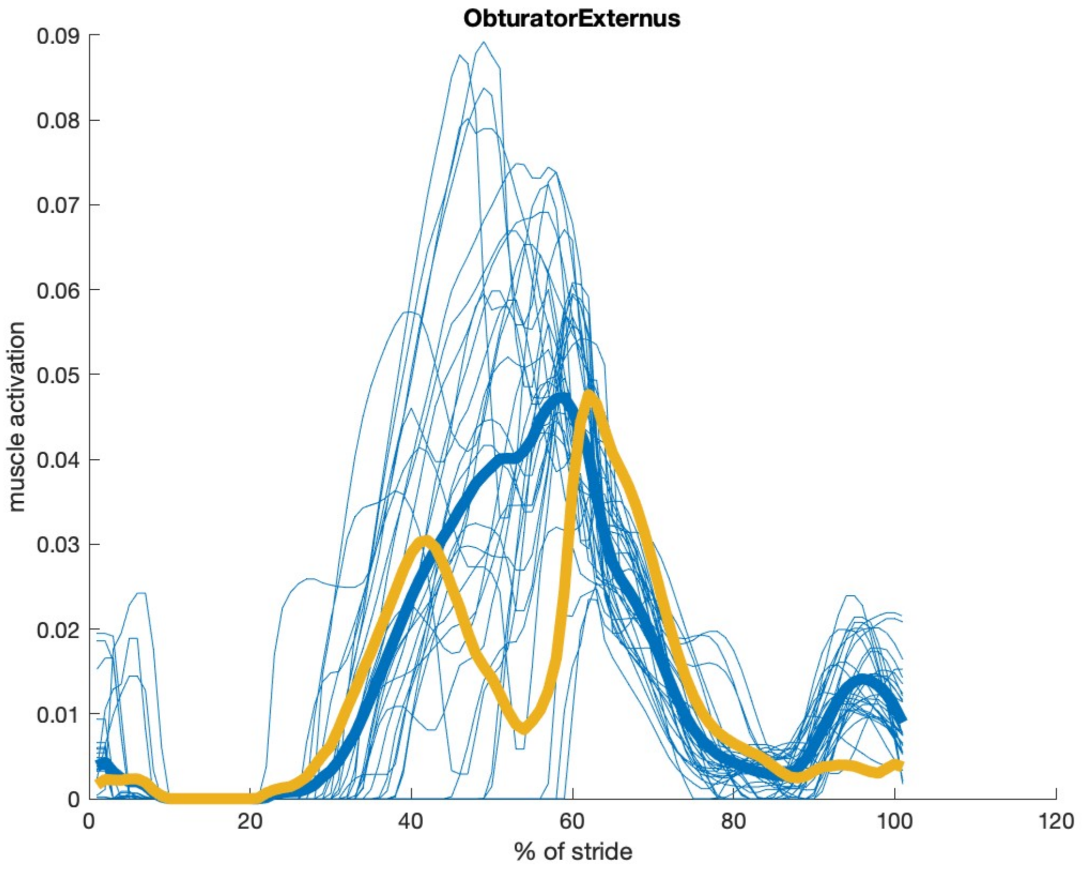

Fig. S3B.

Biology Open • Supplementary information

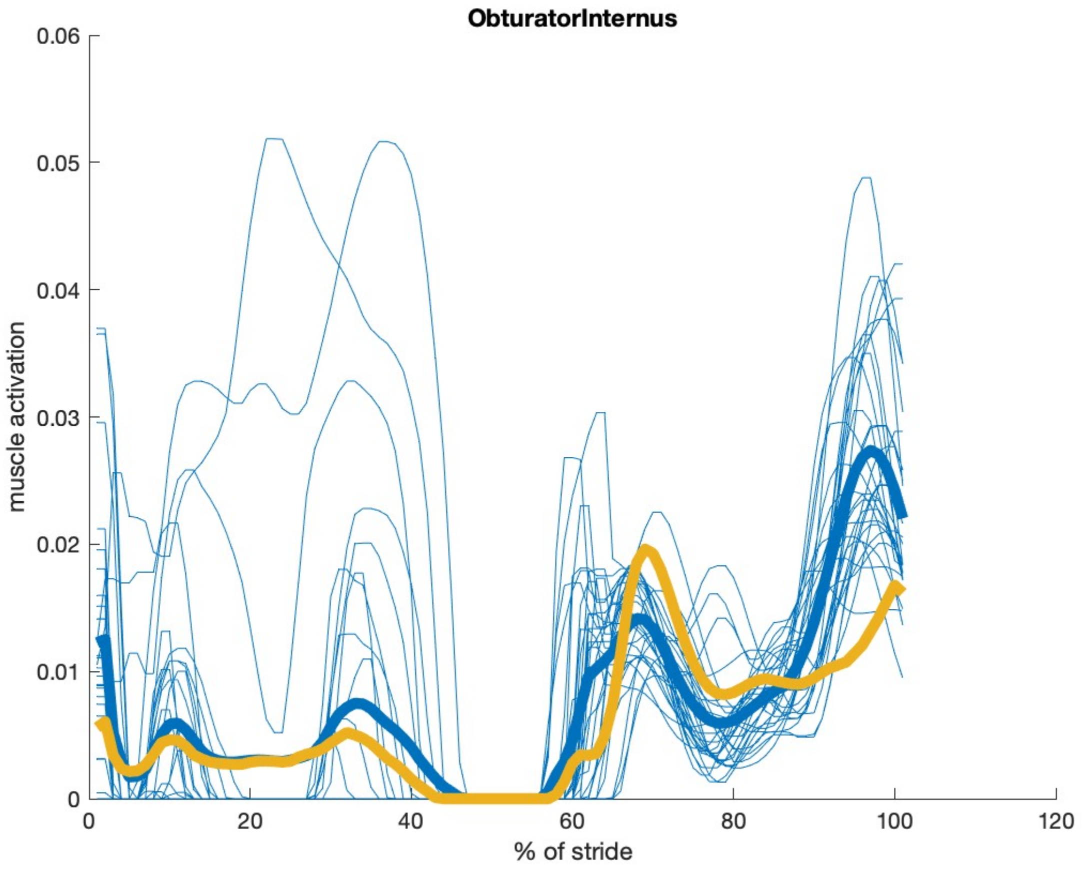

Fig. S3C.

Biology Open • Supplementary information

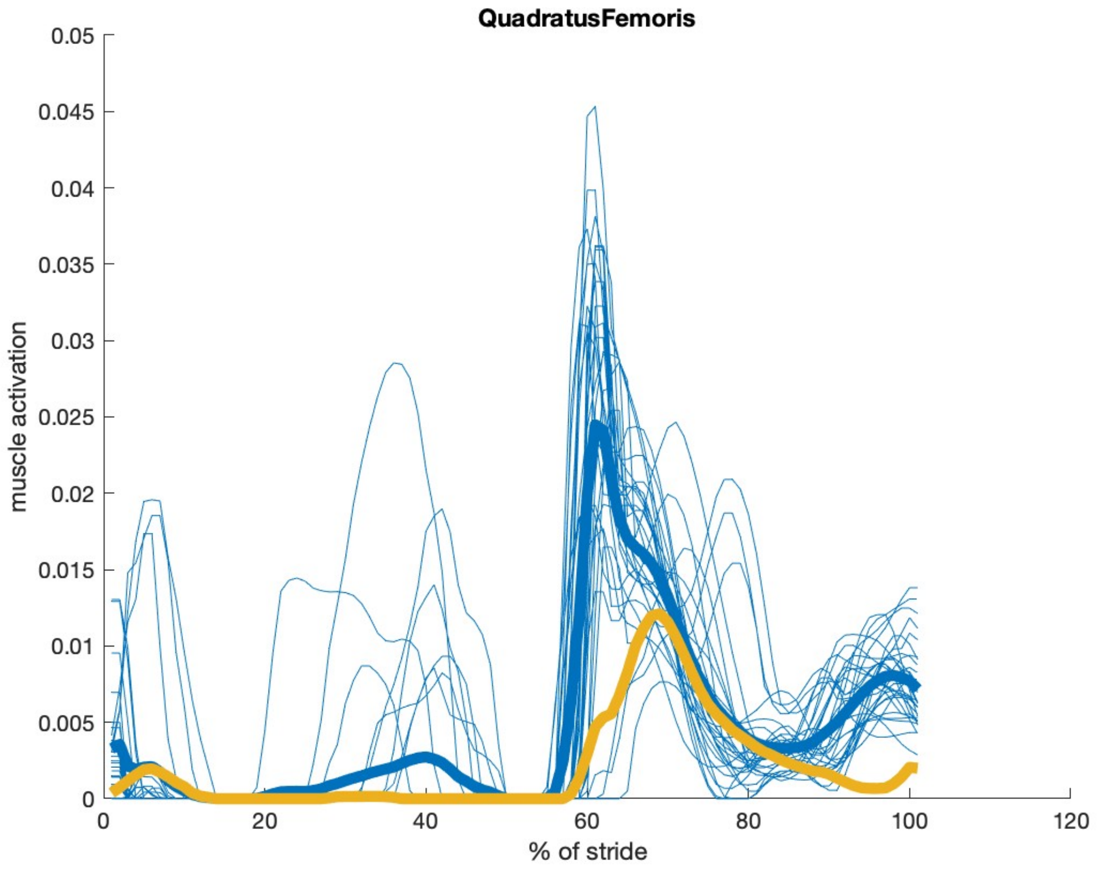

Fig. S3D.

Biology Open • Supplementary information

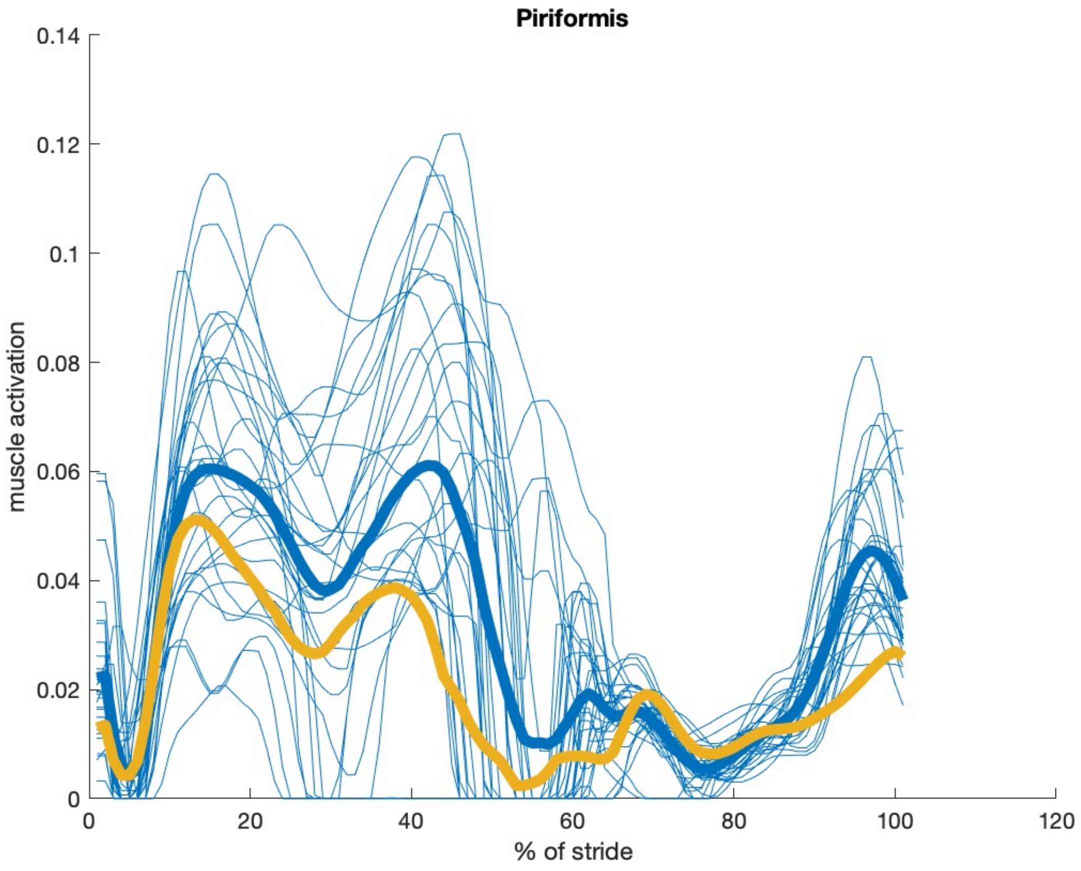
Piriformis
muscle activation
% of stride
0.14
0.12
0.1
0.08
0.06
0.04
0.02
0
0
20
40
60
80
100
120

Fig. S3E.

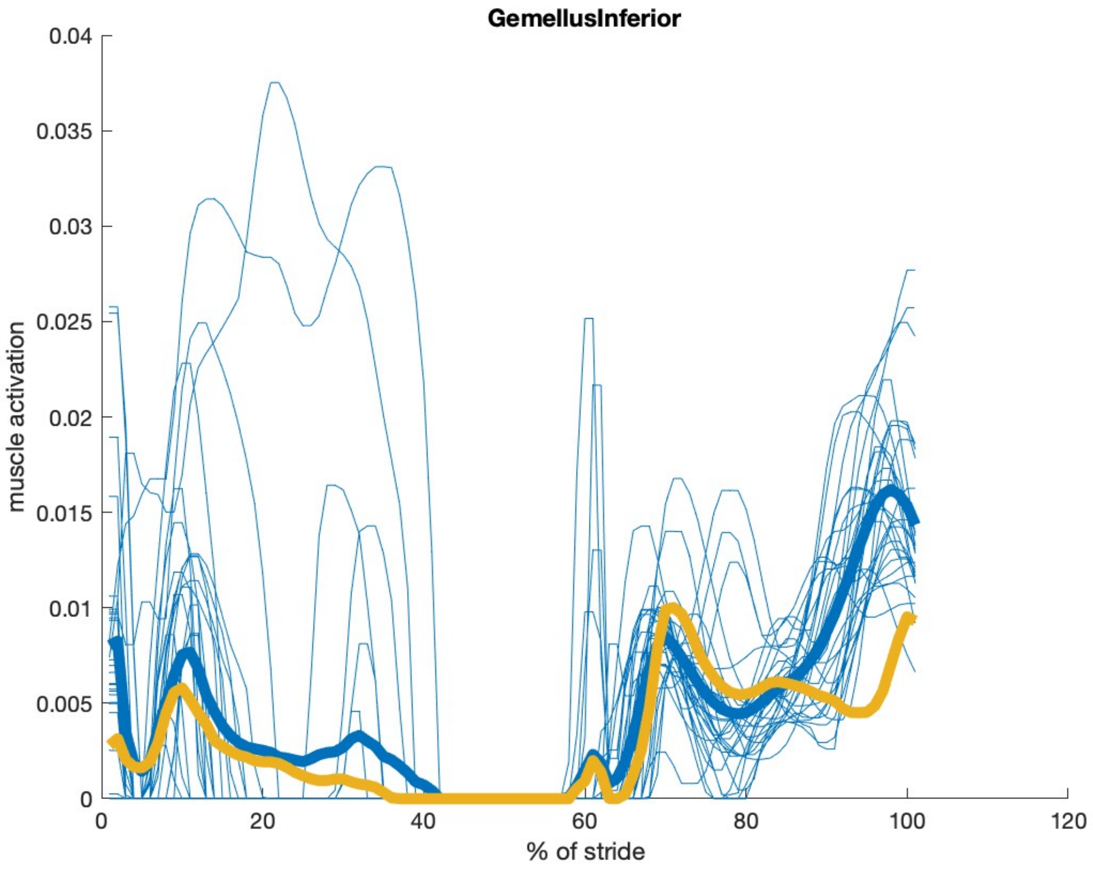

Fig. S3F.

Biology Open • Supplementary information

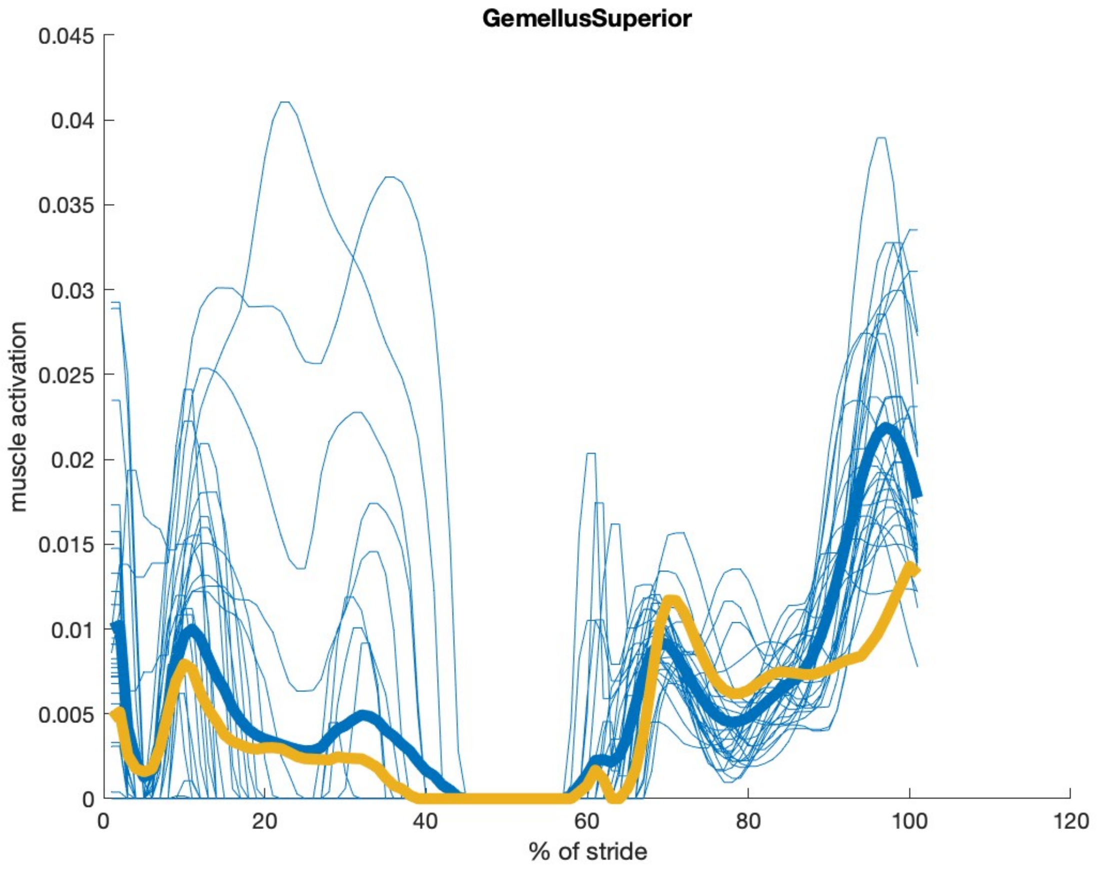

Fig. S3G.

Biology Open • Supplementary information

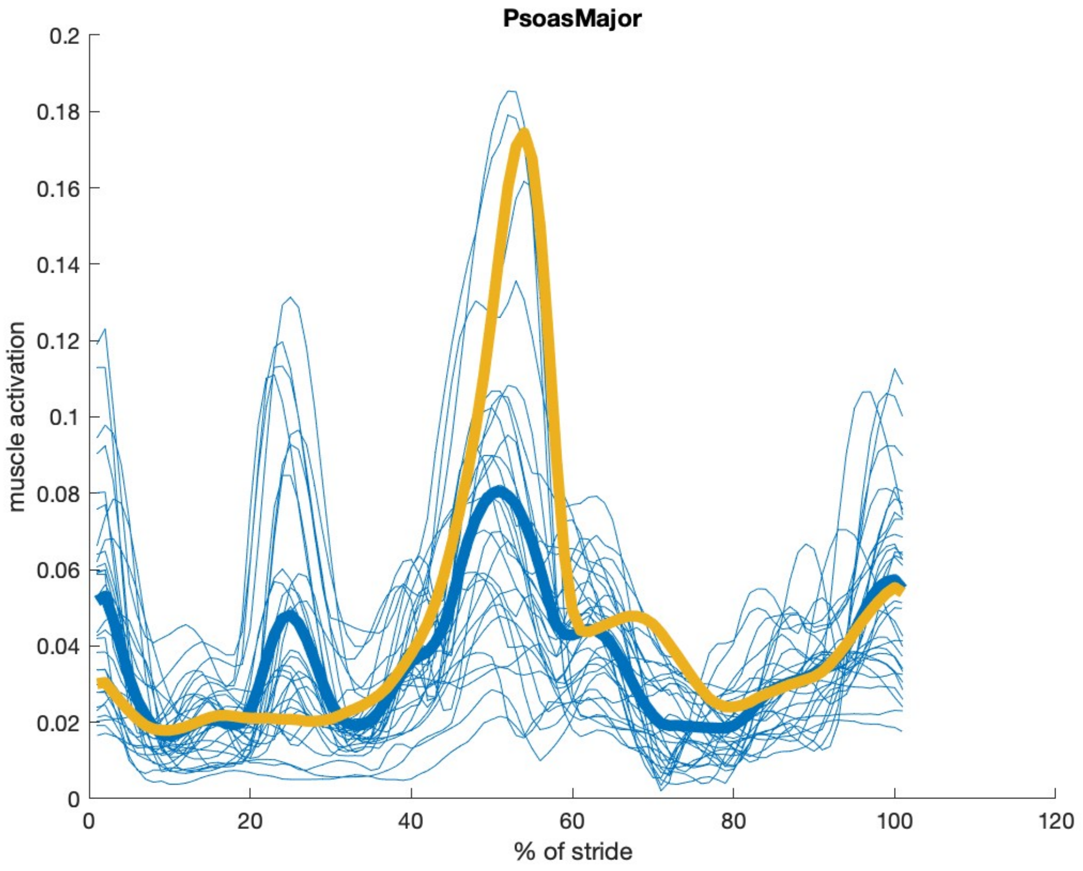

Fig. S3H.

Biology Open • Supplementary information

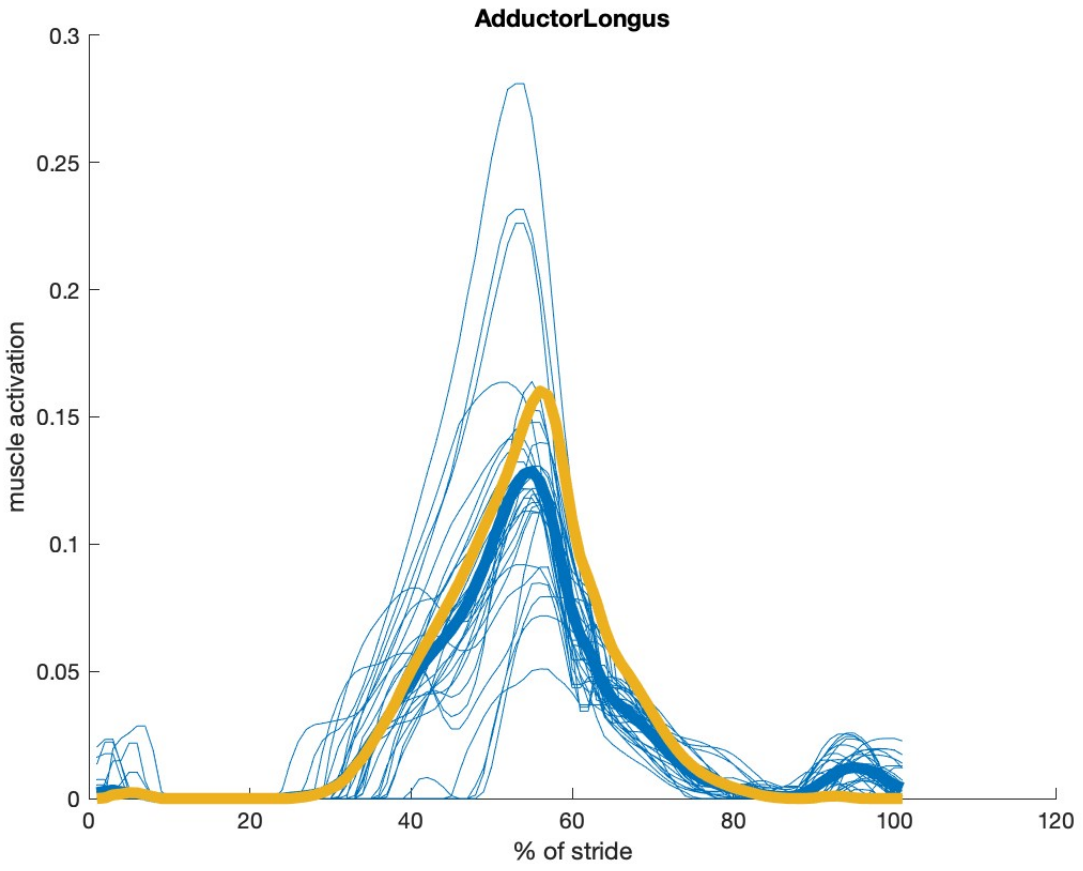

Fig. S3I.

Biology Open • Supplementary information

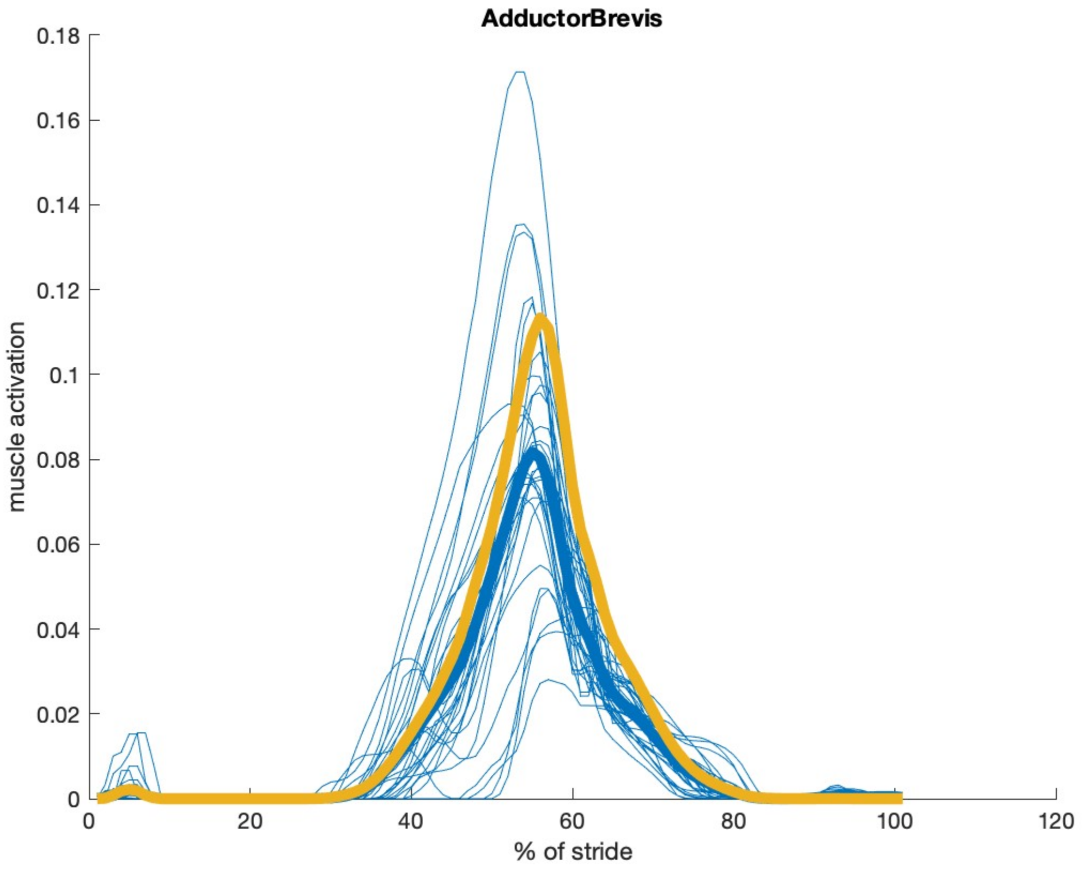

Fig. S3J.

Biology Open • Supplementary information

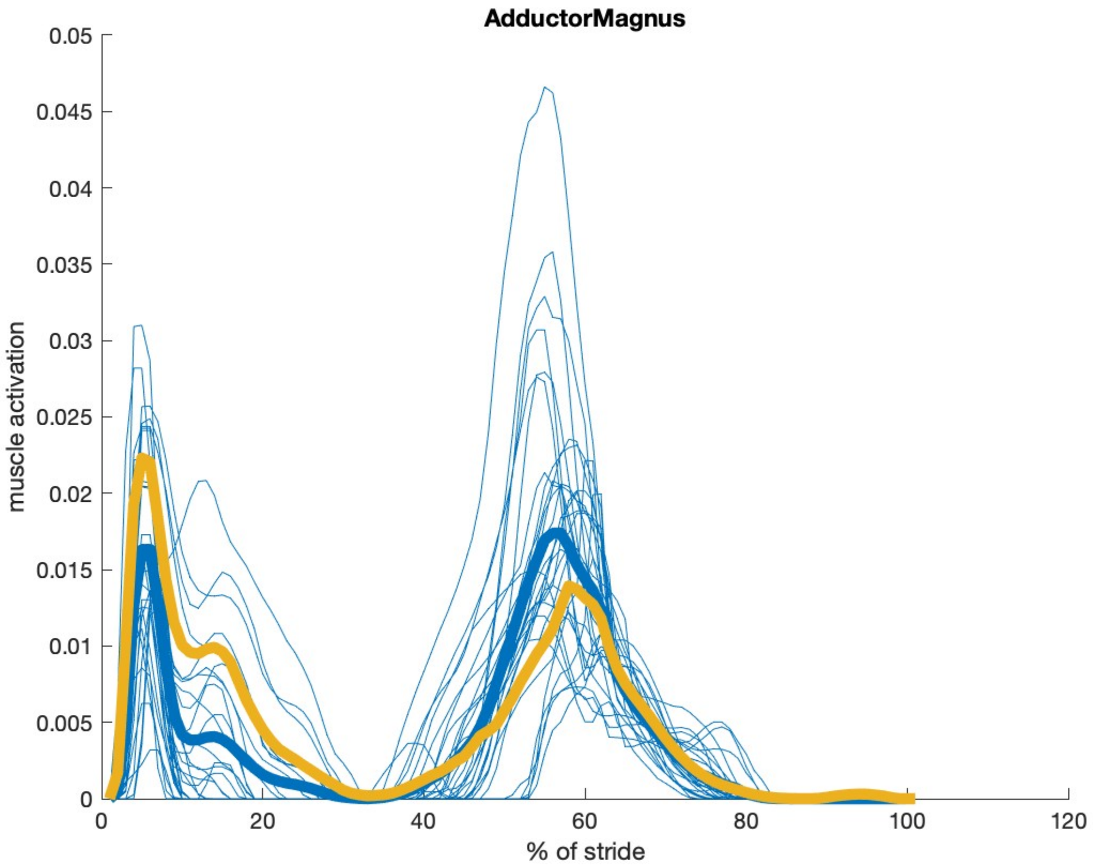

Fig. S3K.

Biology Open • Supplementary information

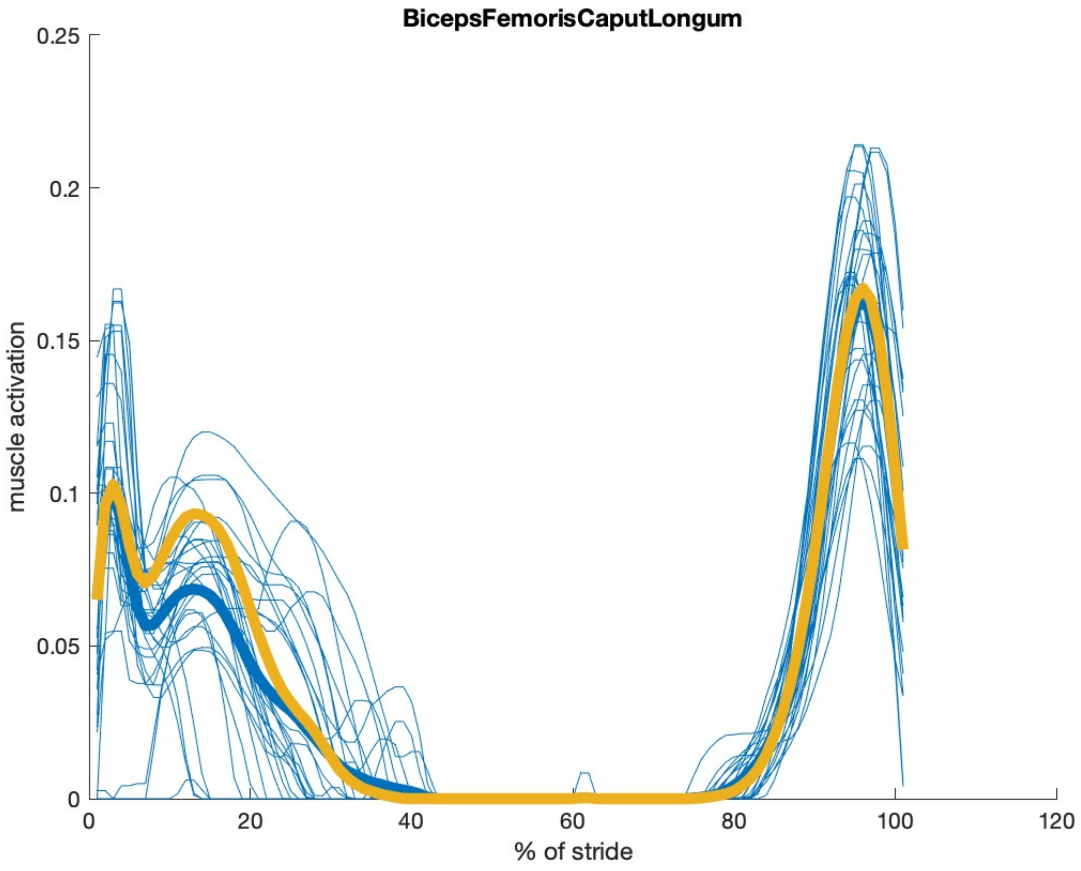

Fig. S3L.

Biology Open • Supplementary information

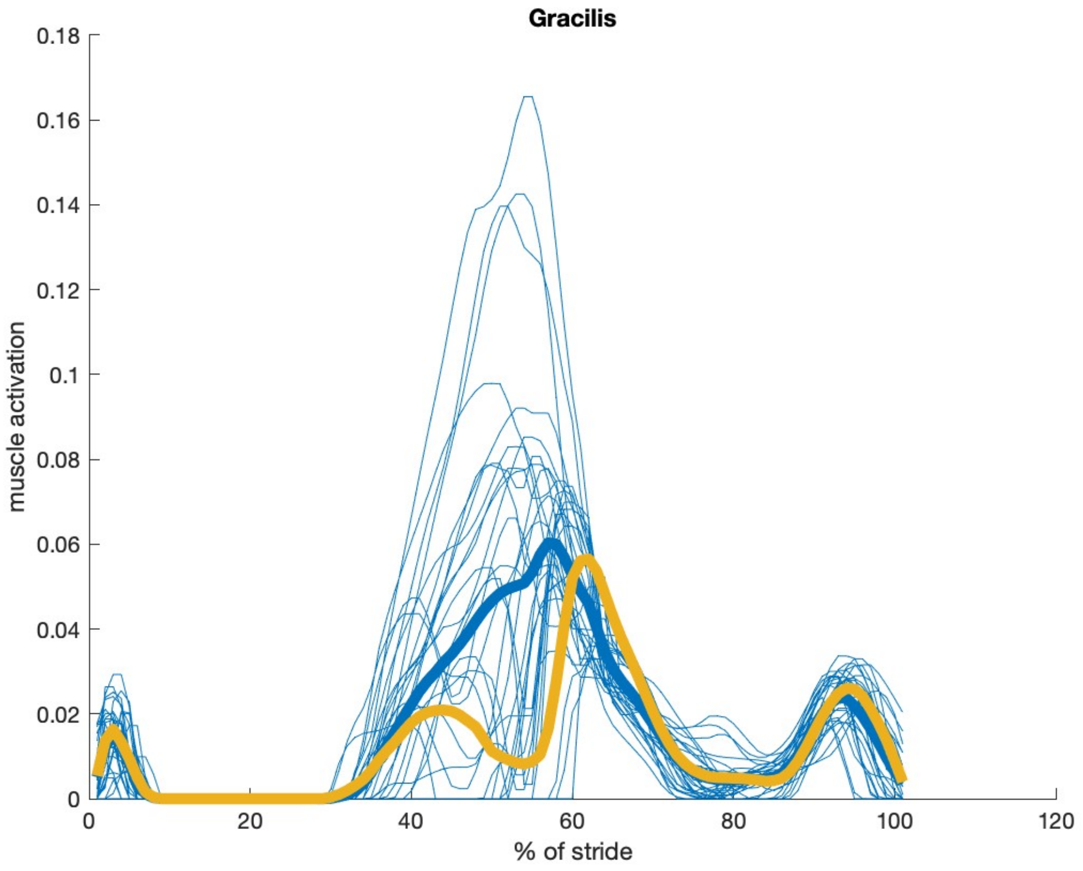

Fig. S3M.

Biology Open • Supplementary information

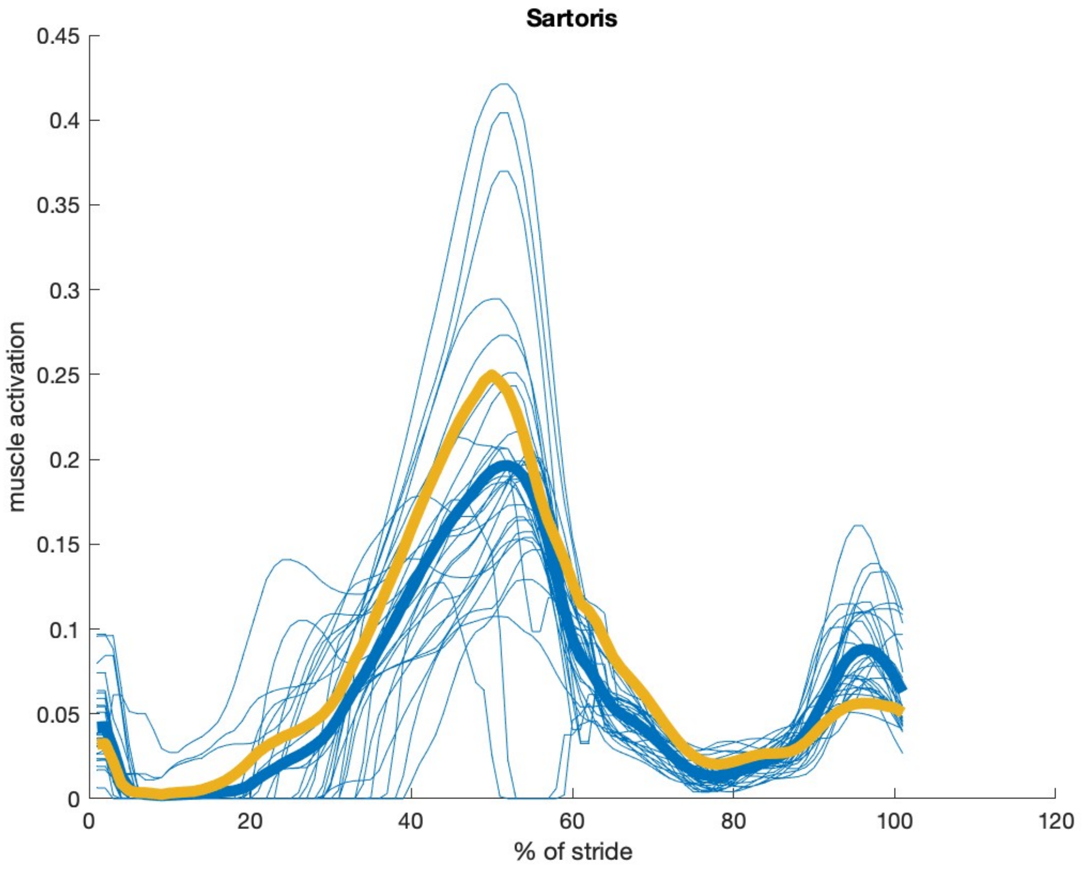

Fig. S3N.

Biology Open • Supplementary information

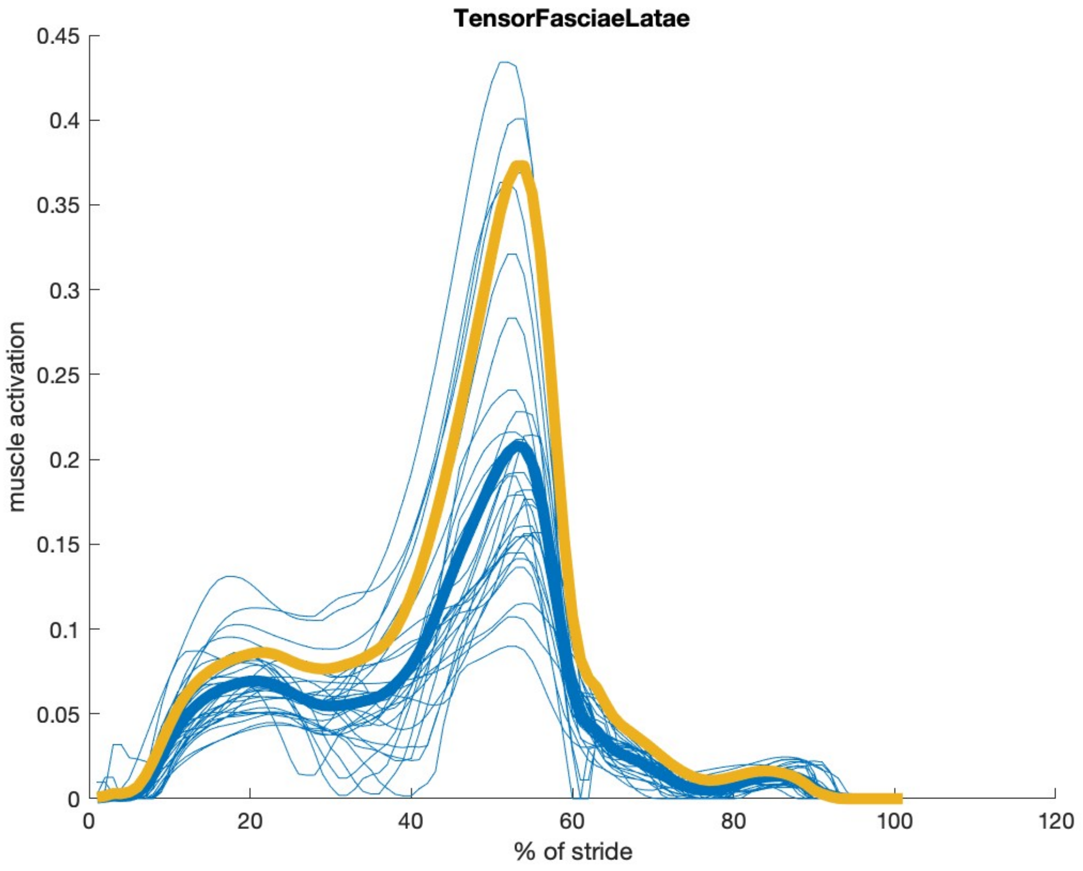
TensorFasciaeLatae
muscle activation
% of stride
0
0.05
0.1
0.15
0.2
0.25
0.3
0.35
0.4
0.45
0
20
40
60
80
100
120

Fig. S3O.

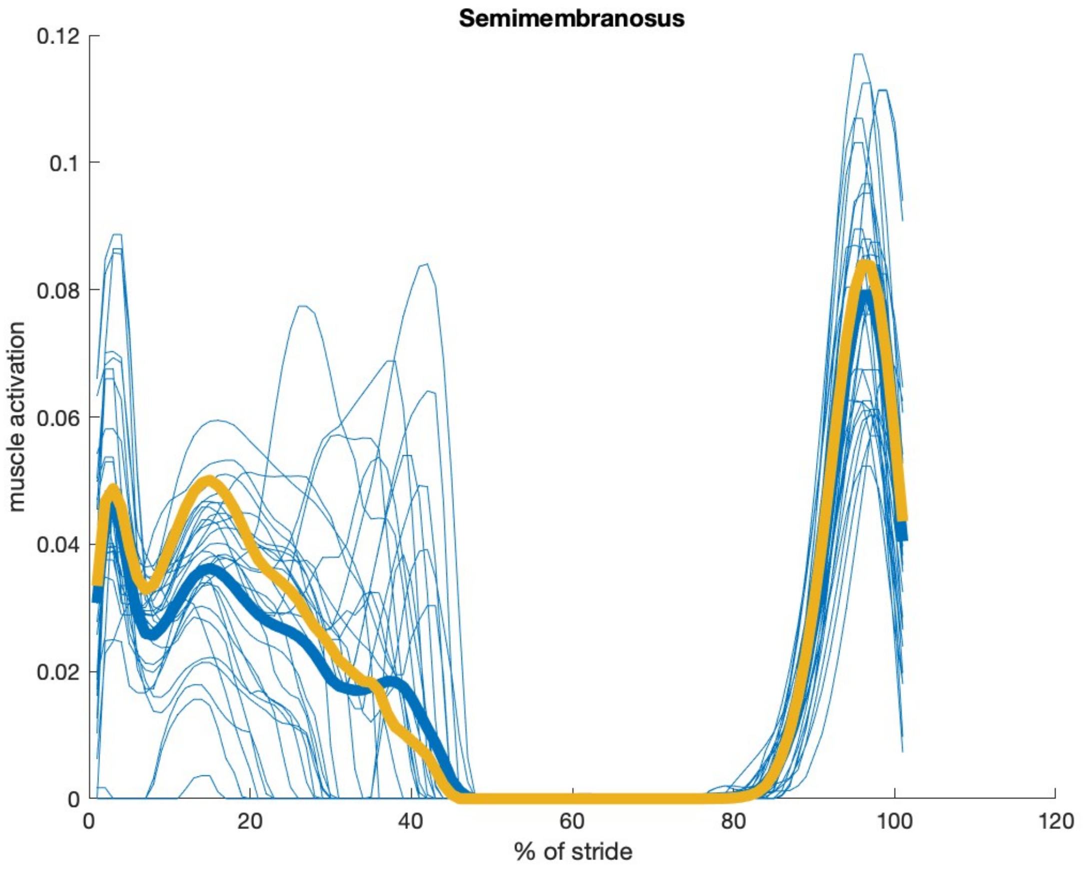

Fig. S3P.

Biology Open • Supplementary information

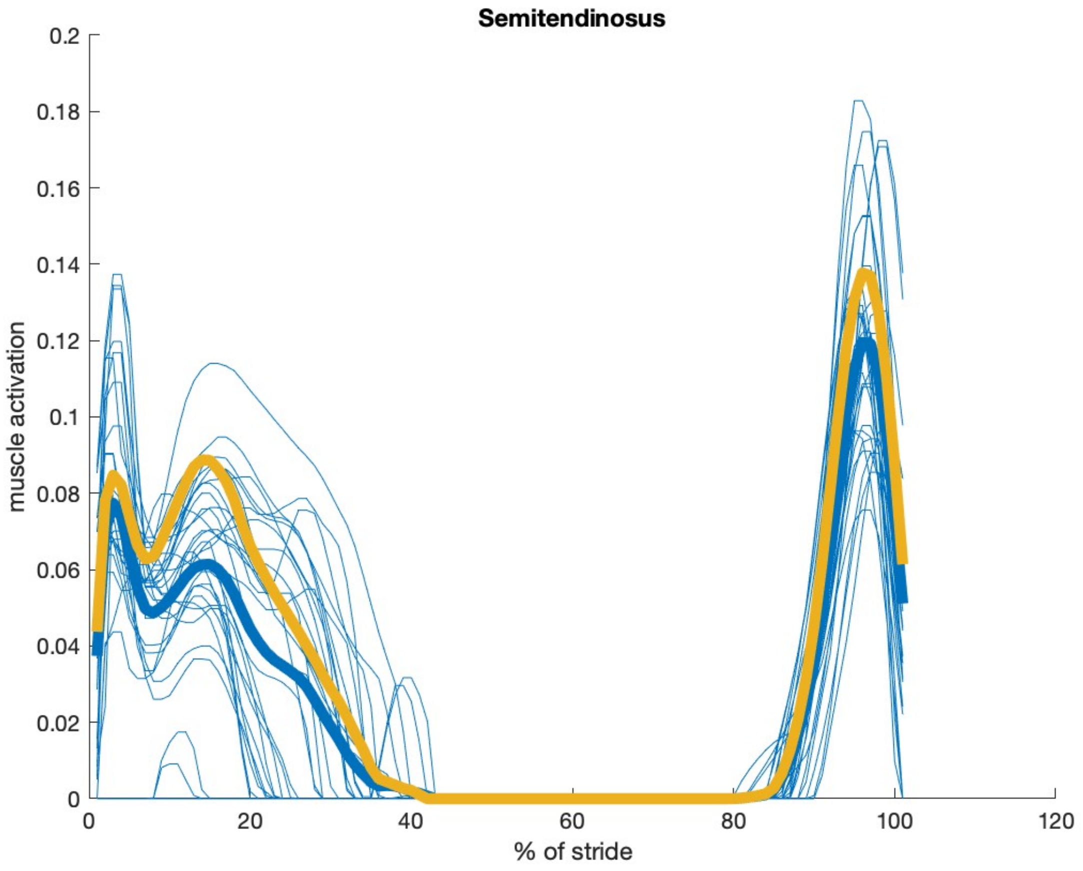

Fig. S3Q.

Biology Open • Supplementary information

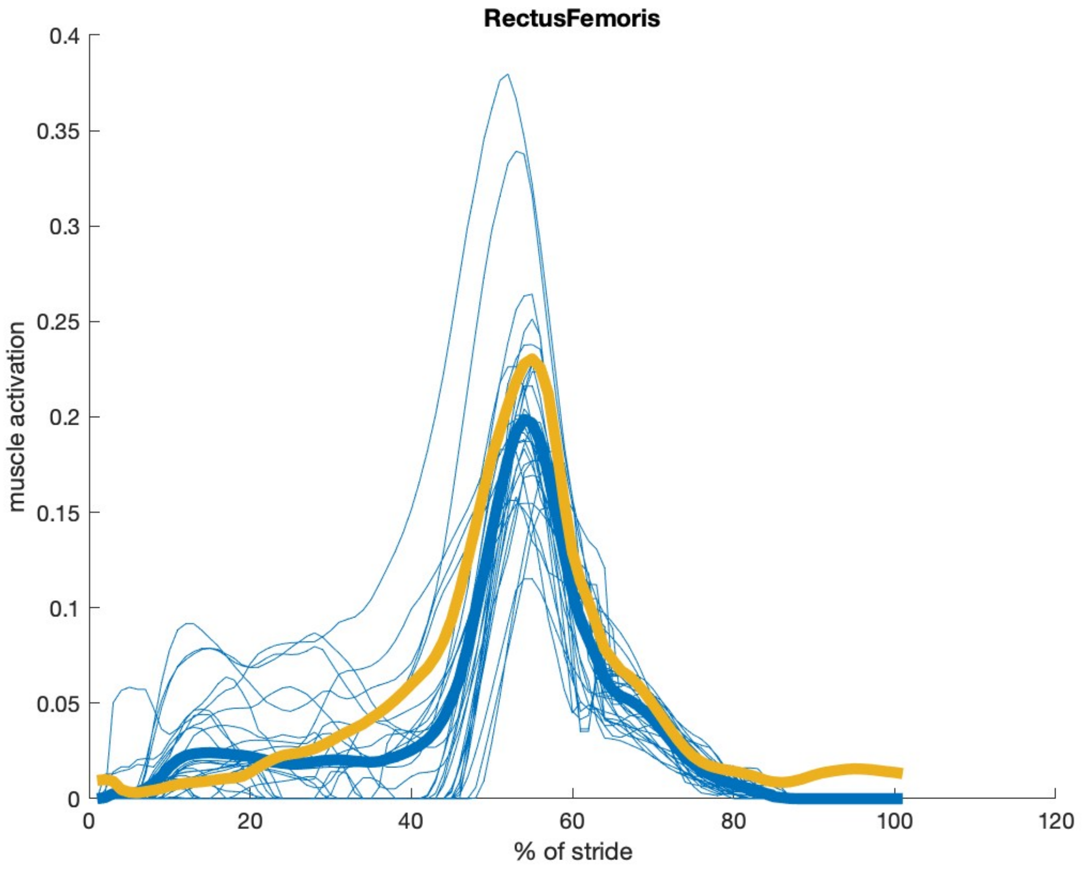

Fig. S3R.

Biology Open • Supplementary information

Anybody Scaling Files

ScalingFunctionTLEMLucyFemur_Mirrored_2014013

```
AnyFolder ScalingFunctionTLEMLucyFemur_Mirrored = {
  AnyFolder Left = {
          AnyFolder Thigh = {
        AnyMatrix AMirroring = {
        {1,0,0},
        {0,1,0},
        {0,0,-1}
        };
          AnyFunTransform3DRBF RBFTransform = {
                  RBFDef.Type = RBF_ThinPlate;
                  PolynomDegree = 1;
                  Points0 = ({
                          {0.0000000,0.0000000,0.0000000},
                          {-0.0000000,-0.3616821,0.0000000},
                          {-0.0097563,-0.3678799,0.0012967},
                          {-0.0000000,-0.3660632,0.0408203},
                          {-0.0000000,-0.3573010,-0.0408203},
                          {0.0161460,-0.0072838,0.0601290},
                          {0.0220217,-0.0203698,0.0463848},
                          {0.0123977,-0.0241932,0.0668573},
                          {0.0006898,0.0018121,0.0538181},
                          {0.0122809,-0.0068668,0.0414535},
                          {-0.0058991,-0.0138188,0.0648412},
                          {0.0211469,-0.0380855,0.0538111},
                          {0.0172133,-0.0317342,0.0328381},
                          {0.0177311,-0.0131946,0.0256176},
                          {-0.0110079,-0.0308867,0.0632370},
                          {0.0039435,-0.0395977,0.0638271},
                          {-0.0041683,-0.0187472,0.0413214},
                          {-0.0177999,-0.0021535,0.0496084},
                          {0.0001603,-0.0053126,0.0279593},
                          {-0.0211600,-0.0216243,0.0497147},
                          {0.0096163,-0.0568663,0.0545719},
                          {0.0162112,-0.0515013,0.0395230},
                          {0.0093533,-0.0433265,0.0187387},
                          {0.0119398,-0.0238469,0.0118403},
                          {0.0210707,-0.0048848,0.0065878},
                          {0.0125013,0.0064094,0.0177702},
                          {-0.0191523,-0.0356053,0.0382429},
                          {-0.0115821,-0.0474937,0.0537648},
                          {-0.0100073,-0.0236371,0.0267322},
                          {-0.0078925,0.0107098,0.0182833},
```

Biology Open • Supplementary information

{-0.0134217,-0.0055356,0.0182000},
{-0.0090061,-0.0665700,0.0475484},
{0.0071095,-0.0761976,0.0520035},
{0.0182119,-0.0704149,0.0386546},
{0.0073764,-0.0609481,0.0221006},
{-0.0122984,-0.0439585,0.0198428},
{-0.0032253,-0.0306792,0.0110995},
{-0.0012975,-0.0198451,-0.0052592},
{0.0140405,-0.0161125,-0.0071649},
{0.0189222,-0.0014898,-0.0127226},
{0.0181642,0.0132812,-0.0011050},
{0.0045119,0.0200001,0.0091189},
{-0.0176761,-0.0541983,0.0348587},
{-0.0136548,-0.0142015,0.0054311},
{-0.0135510,0.0182940,0.0017175},
{-0.0223865,0.0023399,0.0028018},
{-0.0086455,-0.0738247,0.0283843},
{-0.0050356,-0.0877749,0.0442195},
{0.0160135,-0.0919319,0.0465120},
{0.0128290,-0.0785810,0.0232431},
{-0.0168328,-0.0616327,0.0155917},
{-0.0172369,-0.0094567,-0.0119502},
{0.0002090,-0.0093641,-0.0209779},
{0.0064827,0.0081538,-0.0198453},
{0.0010777,0.0210422,-0.0084721},
{-0.0129485,0.0085451,-0.0162212},
{-0.0002322,-0.0924033,0.0245644},
{-0.0028175,-0.1052263,0.0418294},
{0.0172322,-0.1111444,0.0445760},
{0.0203259,-0.0957409,0.0271449},
{0.0057102,-0.1088933,0.0195113},
{-0.0010852,-0.1230776,0.0363132},
{0.0182727,-0.1303312,0.0422884},
{0.0242891,-0.1161703,0.0265054},
{0.0094531,-0.1268584,0.0162294},
{0.0014244,-0.1413293,0.0331653},
{0.0207271,-0.1496427,0.0394157},
{0.0267562,-0.1360142,0.0236798},
{0.0113839,-0.1461651,0.0136024},
{0.0030845,-0.1595094,0.0297156},
{0.0209528,-0.1691386,0.0369775},
{0.0283958,-0.1540209,0.0213795},
{0.0129499,-0.1649012,0.0113589},
{0.0016490,-0.1783108,0.0216287},
{0.0147105,-0.1880678,0.0328614},
{0.0298491,-0.1859865,0.0248551},

Biology Open • Supplementary information

{0.0286645,-0.1714377,0.0169488},
{0.0165897,-0.1861006,0.0082595},
{0.0014000,-0.1973476,0.0183740},
{0.0114301,-0.2109792,0.0286157},
{0.0267422,-0.2037622,0.0296951},
{0.0288630,-0.2018335,0.0130127},
{0.0145471,-0.2083505,0.0062833},
{0.0011300,-0.2198071,0.0155602},
{0.0126610,-0.2321235,0.0275354},
{0.0275647,-0.2219268,0.0265185},
{0.0286645,-0.2230253,0.0098701},
{0.0130434,-0.2304822,0.0039177},
{0.0022984,-0.2409585,0.0149650},
{0.0101315,-0.2534740,0.0252125},
{0.0279485,-0.2428555,0.0234276},
{0.0276684,-0.2421559,0.0058691},
{0.0101250,-0.2529527,0.0018983},
{0.0021376,-0.2667831,0.0107073},
{0.0114309,-0.2734905,0.0249960},
{0.0273923,-0.2634953,0.0216324},
{0.0278429,-0.2592546,0.0037692},
{0.0159101,-0.2712985,-0.0033988},
{0.0036708,-0.2863280,0.0004644},
{0.0023115,-0.2884756,0.0187554},
{0.0231385,-0.2879051,0.0224853},
{0.0304009,-0.2799973,0.0083549},
{0.0238189,-0.2917246,-0.0052462},
{0.0064904,-0.3027687,-0.0070587},
{-0.0003492,-0.3064690,0.0093014},
{0.0122179,-0.3045269,0.0243511},
{0.0301837,-0.3030127,0.0114462},
{0.0234182,-0.3107336,-0.0085198},
{0.0046893,-0.3214995,-0.0123881},
{-0.0022352,-0.3264905,0.0043340},
{0.0016841,-0.3202756,0.0217839},
{0.0229722,-0.3196887,0.0215083},
{0.0292438,-0.3228042,0.0051430},
{0.0221641,-0.3296190,-0.0132361},
{0.0100459,-0.3417597,-0.0244560},
{-0.0053009,-0.3375737,-0.0137705},
{-0.0064432,-0.3487307,0.0028676},
{-0.0059346,-0.3407801,0.0202938},
{0.0105130,-0.3365976,0.0264175},
{0.0282695,-0.3386249,0.0180307},
{0.0270402,-0.3431844,-0.0001487},
{0.0257040,-0.3506950,-0.0181851},

Biology Open • Supplementary information

{0.0178948,-0.3636114,-0.0302838},
{0.0043149,-0.3544109,-0.0381949},
{-0.0095210,-0.3461482,-0.0295031},
{-0.0164526,-0.3542895,-0.0126666},
{-0.0060896,-0.3690127,-0.0033061},
{-0.0176523,-0.3612686,0.0110109},
{-0.0153310,-0.3546943,0.0299634},
{0.0021329,-0.3508021,0.0350763},
{0.0210690,-0.3548628,0.0279835},
{0.0356594,-0.3558162,0.0159356},
{0.0275718,-0.3617450,-0.0018607},
{0.0298503,-0.3741530,-0.0163721},
{0.0175320,-0.3855300,-0.0277515},
{0.0025604,-0.3740099,-0.0397137},
{-0.0136727,-0.3624016,-0.0402102},
{-0.0277647,-0.3567472,-0.0284947},
{-0.0275446,-0.3699630,-0.0120829},
{-0.0175846,-0.3859355,-0.0177922},
{0.0001572,-0.3856873,-0.0118050},
{0.0030262,-0.3834856,0.0075553},
{-0.0144114,-0.3810887,0.0140304},
{-0.0247706,-0.3701239,0.0285134},
{-0.0044804,-0.3677201,0.0404632},
{0.0133183,-0.3712274,0.0334208},
{0.0324861,-0.3723417,0.0245668},
{0.0271451,-0.3766027,0.0072441},
{0.0167985,-0.3822816,-0.0065349},
{-0.0001454,-0.3910823,-0.0303010},
{-0.0144998,-0.3834154,-0.0384137},
{-0.0301321,-0.3750841,-0.0321336},
{0.0196491,-0.3864804,0.0210853},
{0.0015726,-0.3888034,0.0276569},
{-0.0146652,-0.3847848,0.0336913},
{-0.0014588,-0.0619951,0.0219778},
{-0.0118435,-0.0550626,0.0139639},
{0.0129812,-0.0349966,0.0673128},
{-0.0162907,-0.0105666,0.0597739},
{-0.0012885,-0.0520816,0.0562989},
{-0.0090599,-0.0631298,0.0172224},
{-0.0000222,-0.0771663,0.0231897},
{-0.0068148,0.0023885,-0.0160086},
{-0.0180544,-0.0425409,0.0446585},
{0.0020525,-0.0249904,0.0691549},
{-0.0157097,-0.0662315,0.0238393},
{-0.0010518,-0.0510686,0.0199353},
{-0.0208874,-0.0152865,0.0500034},

Biology Open • Supplementary information

{0.0168316,-0.0280857,0.0666680},
{-0.0187392,-0.0282333,0.0403282},
{0.0191622,-0.0351633,0.0622707},
{0.0001216,-0.0706289,0.0524049},
{-0.0169862,-0.0399062,0.0311827},
{-0.0185737,-0.0533066,0.0151224},
{-0.0219058,-0.0528723,0.0222263},
{-0.0028271,-0.0391620,0.0148452},
{0.0117900,-0.0553425,0.0235348},
{0.0219835,-0.0106400,0.0396338},
{-0.0025366,-0.0401046,0.0625273},
{0.0141017,-0.0018767,0.0198108},
{-0.0078770,-0.0473929,0.0173542},
{-0.0065351,-0.0766431,0.0470258},
{0.0065585,0.0145593,0.0161638},
{-0.0118588,0.0172875,-0.0085064},
{-0.0074087,-0.0627500,0.0506700},
{0.0071940,-0.0780068,0.0213362},
{0.0126736,-0.0708176,0.0244108},
{0.0074694,-0.0436450,0.0604196},
{0.0154186,-0.0139471,0.0064312},
{-0.0053535,-0.0213210,0.0040094},
{-0.0088733,0.0002018,0.0205402},
{-0.0112471,-0.0542608,0.0500266},
{-0.0061625,-0.0248673,0.0653244},
{0.0093695,-0.0065826,0.0614157},
{0.0138015,-0.0467322,0.0284333},
{-0.0020999,-0.0186885,-0.0116392},
{-0.0185640,-0.0225591,0.0566779},
{0.0175798,0.0124360,-0.0068467},
{-0.0039962,0.0217127,0.0053055},
{0.0014975,-0.0784591,0.0514056},
{-0.0172997,-0.0386971,0.0525252},
{0.0196540,-0.0268271,0.0416806},
{0.0135296,-0.0406504,0.0236882},
{0.0182471,0.0095959,0.0087906},
{0.0218667,0.0016804,0.0058793},
{0.0224975,-0.0022652,-0.0022994},
{0.0166750,-0.0191391,0.0236436},
{0.0119785,-0.0328723,0.0195376},
{0.0187631,-0.0133469,0.0308741},
{-0.0187543,-0.0461463,0.0257491},
{0.0039834,-0.0373928,0.0683089},
{0.0157029,-0.0423169,0.0385226},
{0.0164003,-0.0087069,0.0131181},
{0.0008253,0.0033370,-0.0212576},

Biology Open • Supplementary information

{0.0101707,-0.0009724,-0.0205688},
{0.0102948,-0.0642683,0.0524532},
{0.0152217,-0.0557944,0.0508978},
{0.0172973,-0.0565707,0.0419468},
{0.0167723,-0.0635334,0.0369834},
{-0.0047397,-0.0695325,0.0241398},
{-0.0152913,0.0035778,0.0161461},
{0.0091697,0.0009340,0.0575160},
{0.0196686,-0.0089533,0.0500006},
{0.0183998,-0.0212943,0.0622968},
{-0.0114340,-0.0316810,0.0250940},
{-0.0206442,-0.0092599,-0.0028425},
{0.0166868,-0.0784295,0.0276925},
{-0.0069678,-0.0054521,-0.0207011},
{0.0049490,0.0151109,-0.0160093},
{-0.0084025,-0.0318937,0.0162788},
{-0.0173679,0.0095594,-0.0108509},
{-0.0218912,0.0063457,-0.0020698},
{-0.0106547,-0.0668750,0.0397749},
{0.0136098,-0.0170666,-0.0018890},
{0.0141599,-0.0441405,0.0587594},
{0.0093255,-0.0183218,-0.0083008},
{0.0106367,-0.0128276,-0.0154269},
{0.0037350,-0.0549365,0.0209786},
{0.0087662,-0.0472902,0.0195496},
{0.0016839,-0.0708213,0.0221325},
{-0.0202977,-0.0530540,0.0301270},
{-0.0068584,0.0021781,0.0530002},
{-0.0144817,-0.0297218,0.0605833},
{0.0197015,-0.0165217,0.0560654},
{0.0156609,-0.0705363,0.0477736},
{0.0207876,-0.0221433,0.0330733},
{0.0092324,-0.0300767,0.0713160},
{-0.0203392,-0.0034488,0.0097332},
{0.0156978,-0.0143438,0.0629053},
{0.0143267,-0.0563519,0.0317489},
{-0.0161685,-0.0465391,0.0188591},
{-0.0196397,-0.0309875,0.0546496},
{0.0154804,-0.0380422,0.0305433},
{-0.0077774,-0.0351303,0.0637765},
{0.0063980,-0.0086419,-0.0201654},
{0.0030146,-0.0151094,-0.0165974},
{-0.0210080,-0.0096030,0.0507855},
{-0.0105684,-0.0411377,0.0586146},
{0.0180619,-0.0498960,0.0469835},
{-0.0192318,0.0116108,0.0024070},

{-0.0165651,0.0083622,0.0126870},
{-0.0014333,-0.0627138,0.0534746},
{0.0145127,-0.0080178,0.0353667},
{0.0148374,-0.0012228,0.0566181},
{0.0153834,-0.0062427,0.0444727},
{-0.0155164,-0.0132103,-0.0096398},
{0.0068685,-0.0221022,0.0011547},
{0.0003332,-0.0458500,0.0183716},
{0.0205582,0.0040956,-0.0082969},
{0.0175752,0.0009340,-0.0148407},
{0.0142357,-0.0063623,-0.0169332},
{-0.0147285,0.0023412,-0.0164678},
{-0.0158781,-0.0064451,-0.0151346},
{0.0175951,0.0133456,0.0037559},
{0.0117660,0.0189899,-0.0030690},
{0.0096040,0.0151827,-0.0137450},
{0.0096565,-0.0215194,0.0662734},
{-0.0006793,-0.0074494,0.0631218},
{0.0007819,-0.0004519,0.0600781},
{0.0065377,-0.0125433,0.0654199},
{0.0090461,0.0073096,0.0188711},
{0.0151418,-0.0168302,0.0146465},
{0.0025166,-0.0231863,0.0688894},
{-0.0097891,-0.0152077,0.0253086},
{-0.0109149,-0.0177935,0.0119825},
{-0.0072079,-0.0099373,0.0272725},
{-0.0183727,-0.0467429,0.0356276},
{-0.0126871,-0.0584555,0.0428141},
{-0.0057054,-0.0273042,0.0103554},
{0.0215142,-0.0159693,0.0474531},
{0.0128492,-0.0070016,0.0287645},
{0.0052134,-0.0017047,0.0247463},
{-0.0024284,-0.0707498,0.0513049},
{-0.0036140,0.0220593,-0.0040484},
{-0.0002405,-0.0121253,0.0390050},
{-0.0119811,-0.0156737,0.0015445},
{0.0142082,-0.0279792,0.0246553},
{0.0122244,-0.0262013,0.0169829},
{-0.0146264,-0.0038855,0.0549384},
{0.0207450,-0.0325771,0.0496412},
{-0.0155040,-0.0497560,0.0437087},
{-0.0081119,-0.0756450,0.0387365},
{0.0039684,-0.0062047,0.0313900},
{-0.0023726,-0.0038892,-0.0220521},
{-0.0136977,-0.0339839,0.0602619},
{-0.0035399,0.0089081,-0.0188894},

{-0.0049820,0.0176571,-0.0132254},
{0.0193902,-0.0803191,0.0360429},
{0.0040648,-0.0697109,0.0218205},
{0.0170470,-0.0818610,0.0456892},
{0.0104175,0.0188635,0.0064965},
{0.0135206,0.0092549,0.0158079},
{0.0023933,0.0160498,-0.0157404},
{0.0164753,-0.0118197,-0.0096995},
{0.0202584,-0.0066858,-0.0082383},
{-0.0098401,-0.0226001,0.0314028},
{-0.0130937,-0.0309622,0.0320764},
{-0.0201510,-0.0613297,0.0222264},
{-0.0010389,0.0036055,0.0209003},
{0.0042025,0.0073392,0.0199393},
{-0.0021630,-0.0038531,0.0251898},
{-0.0007845,-0.0293574,0.0675788},
{0.0070027,-0.0406327,0.0624565},
{-0.0124363,-0.0183001,0.0627966},
{-0.0120485,-0.0256613,0.0628493},
{0.0101200,0.0197695,0.0039334},
{0.0167636,0.0148266,0.0014155},
{-0.0074981,-0.0106930,0.0632797},
{0.0142533,-0.0503445,0.0351954},
{-0.0146107,-0.0460953,0.0506120},
{-0.0161117,-0.0471301,0.0178900},
{0.0112149,-0.0792793,0.0501460},
{-0.0099060,-0.0230794,0.0186951},
{-0.0114543,-0.0153049,0.0183675},
{-0.0042882,0.0161879,0.0151423},
{0.0014582,-0.0638572,0.0541080},
{0.0048554,-0.0582603,0.0553815},
{-0.0017077,-0.0572302,0.0546635},
{0.0166090,-0.0697169,0.0323038},
{-0.0208812,-0.0164273,0.0474354},
{0.0130454,-0.0241864,0.0178294},
{0.0053123,-0.0483770,0.0582251},
{0.0112225,-0.0361429,0.0673300},
{0.0085729,-0.0771256,0.0216100},
{-0.0070783,-0.0488033,0.0560869},
{0.0182083,-0.0411024,0.0454236},
{-0.0119590,-0.0403809,0.0213546},
{0.0233409,-0.0185535,0.0366343},
{-0.0046347,-0.0571211,0.0208243},
{0.0183166,-0.0006966,0.0140116},
{0.0061913,0.0053512,0.0201003},
{-0.0002364,-0.0310828,0.0680351},

Biology Open • Supplementary information

{-0.0170773,0.0139270,-0.0054987},
{0.0110805,0.0156034,0.0117010},
{-0.0124628,0.0164667,0.0094550},
{-0.0209014,-0.0073407,-0.0051969},
{-0.0210131,0.0010693,-0.0086891},
{-0.0041596,-0.0172332,0.0663041},
{-0.0216595,-0.0322172,0.0451540},
{0.0051908,-0.0426014,0.0613013},
{0.0150003,-0.0416136,0.0606257},
{-0.0135090,-0.0674234,0.0236425},
{0.0146416,0.0073691,0.0160052},
{0.0195615,-0.0112412,-0.0010333},
{-0.0198115,-0.0098244,0.0044735},
{-0.0157054,-0.0521236,0.0395039},
{-0.0081381,-0.0173108,-0.0046152},
{-0.0218324,-0.0262661,0.0471115},
{0.0051446,-0.0707257,0.0530555},
{0.0179359,-0.0471493,0.0528134},
{-0.0005591,-0.0317983,0.0113632},
{0.0071707,-0.0305943,0.0121186},
{0.0206239,-0.0061367,0.0066642},
{0.0187057,-0.0745868,0.0420207},
{-0.0095784,-0.0163113,0.0259524},
{-0.0012181,0.0127661,0.0186855},
{0.0059420,-0.0630691,0.0220087},
{0.0069408,-0.0573878,0.0552614},
{0.0150782,-0.0132884,-0.0101793},
{0.0005533,-0.0078574,0.0633775},
{-0.0088284,-0.0146399,-0.0147837},
{0.0167444,-0.0651105,0.0470347},
{0.0082666,-0.0181741,-0.0095133},
{-0.0175162,-0.0637444,0.0177069},
{0.0153795,-0.0360767,0.0287608},
{0.0162363,-0.0018864,0.0548412},
{0.0052731,-0.0075919,0.0392990},
{-0.0022288,-0.0556644,0.0549909},
{-0.0156238,-0.0550038,0.0377688},
{-0.0204137,-0.0100550,0.0536429},
{-0.0093683,0.0122429,-0.0162013},
{-0.0202927,0.0049166,0.0083887},
{0.0074706,-0.0627945,0.0222133},
{0.0035034,-0.0376103,0.0672421},
{-0.0190752,-0.0194129,0.0555530},
{0.0061758,-0.0074961,-0.0207741},
{0.0192011,-0.0273063,0.0588233},
{0.0130796,-0.0134383,0.0638611},

Biology Open • Supplementary information

{0.0120421,0.0019566,-0.0194140},
{0.0154152,-0.0065294,0.0179581},
{-0.0157335,0.0101219,0.0123981},
{0.0110376,-0.0488274,0.0567911},
{-0.0055170,-0.0343676,0.0647140},
{0.0216204,-0.0273988,0.0512041},
{0.0161331,-0.0135875,-0.0075501},
{-0.0184643,-0.0455633,0.0360087},
{-0.0091978,-0.0682413,0.0460847},
{0.0034304,-0.0471677,0.0588866},
{-0.0048025,-0.0384316,0.0149400},
{0.0118505,0.0109735,-0.0159161},
{-0.0027036,-0.0204189,-0.0015094},
{-0.0117607,-0.0692759,0.0292171},
{-0.0107080,-0.0033455,-0.0190487},
{-0.0042548,0.0214947,-0.0056846},
{-0.0097315,-0.0208529,0.0131997},
{-0.0178958,-0.0627218,0.0171551},
{-0.0068692,-0.0688818,0.0233316},
{0.0012083,0.0120695,-0.0186179},
{0.0215073,-0.0236027,0.0503069},
{-0.0060262,-0.0534931,0.0189523},
{0.0179951,-0.0336785,0.0397267},
{0.0190509,-0.0284972,0.0336394},
{-0.0169188,-0.0133609,-0.0014203},
{0.0135722,0.0136491,0.0114695},
{-0.0150549,-0.0123230,0.0067722},
{-0.0148411,-0.0311945,0.0355412},
{0.0185490,-0.0794042,0.0431266},
{0.0193568,-0.0087326,0.0575704},
{0.0201026,-0.0144353,0.0531586},
{0.0025583,-0.0248211,0.0045714},
{0.0132689,-0.0214866,0.0652899},
{-0.0028669,-0.0099311,0.0309271},
{-0.0219487,-0.0054893,-0.0021853},
{0.0026180,-0.0203806,-0.0040620},
{-0.0116230,-0.0668518,0.0370152},
{-0.0133007,-0.0264159,0.0619176},
{0.0186167,0.0050491,-0.0120059},
{-0.0191359,-0.0591690,0.0288149},
{-0.0178121,-0.0041834,0.0141448},
{0.0123077,-0.0063648,0.0279743},
{-0.0157729,-0.0374010,0.0315335},
{0.0149328,-0.0659100,0.0290095},
{0.0006205,0.0021977,-0.0214613},
{0.0170484,-0.0715687,0.0325980},

{0.0068499,0.0061192,0.0196608},
{-0.0167075,0.0020024,0.0150382},
{0.0059801,-0.0393117,0.0167489},
{-0.0072506,-0.0171067,0.0338751},
{0.0202363,-0.0251596,0.0420997},
{-0.0199311,-0.0601255,0.0171522},
{0.0216641,0.0053410,-0.0027841},
{-0.0197665,-0.0606125,0.0252972},
{0.0129986,-0.0214528,0.0098002},
{0.0201151,-0.0100929,0.0460501},
{0.0104137,0.0017765,-0.0203073},
{0.0166557,-0.0064123,0.0140197},
{-0.0124120,-0.0596549,0.0422637},
{0.0014885,0.0202590,0.0096781},
{0.0028720,0.0020207,-0.0219044},
{0.0376095,-0.3675427,0.0214537},
{0.0049949,0.0206417,-0.0079017},
{-0.0191723,-0.0407590,0.0391310},
{-0.0316406,-0.3638215,-0.0274841},
{0.0021392,0.0226920,0.0000985},
{0.0168435,-0.0229382,0.0264788},
{-0.0157304,-0.0005437,-0.0161456},
{0.0203722,-0.0299750,0.0562762},
{-0.0171077,0.0134146,0.0059615},
{0.0175519,-0.0370653,0.0413374},
{0.0154657,-0.0405127,0.0348980},
{-0.0215172,-0.0323502,0.0481925},
{0.0177998,-0.0515776,0.0444709},
{-0.0183804,-0.0448316,0.0275139},
{0.0033607,-0.0502028,0.0575894},
{0.0010325,-0.0479377,0.0191141},
{0.0224852,-0.0181821,0.0451780},
{0.0084089,-0.0720538,0.0521031},
{-0.0070880,-0.0772912,0.0288023},
{-0.0223712,-0.0028990,0.0024047},
{0.0173136,-0.0120017,0.0204612},
{0.0137772,-0.0623537,0.0276621},
{-0.0100390,-0.0231180,0.0292222},
{-0.0196395,-0.3730207,0.0364730},
{-0.0094445,-0.0526688,0.0528111},
{-0.0115309,-0.3679776,-0.0104995},
{0.0129812,-0.0410477,0.0224072},
{0.0117394,0.0154849,-0.0117191},
{-0.0245208,-0.3649412,0.0295111},
{0.0147476,-0.0502966,0.0364899},
{0.0006132,-0.0446771,0.0600524},

Biology Open • Supplementary information

{-0.0118123,0.0191139,0.0031977},
{-0.0017185,-0.0163303,0.0664216},
{-0.0085098,-0.0074564,0.0247745},
{0.0197459,-0.0086852,0.0430460},
{0.0187064,-0.0223532,0.0600966},
{-0.0206552,-0.0099712,0.0525024},
{0.0207525,0.0084646,-0.0024846},
{-0.0094915,-0.0325446,0.0184441},
{-0.0014464,-0.0140050,-0.0179819},
{-0.0122725,-0.0573444,0.0463125},
{-0.0106397,0.0177211,0.0094669},
{0.0111981,0.0191236,-0.0041491},
{-0.0162790,-0.0256279,0.0592687},
{-0.0060120,-0.3653959,0.0413411},
{-0.0135007,-0.3703638,-0.0387476},
{-0.0134258,-0.3703142,-0.0379467},
{-0.0133509,-0.3702645,-0.0371458},
{-0.0132760,-0.3702148,-0.0363450},
{-0.0132011,-0.3701651,-0.0355441},
{-0.0131262,-0.3701154,-0.0347432},
{-0.0130513,-0.3700658,-0.0339423},
{-0.0129765,-0.3700161,-0.0331414},
{-0.0129016,-0.3699664,-0.0323405},
{-0.0128267,-0.3699167,-0.0315396},
{-0.0127518,-0.3698670,-0.0307388},
{-0.0126769,-0.3698174,-0.0299379},
{-0.0126020,-0.3697677,-0.0291370},
{-0.0125271,-0.3697180,-0.0283361},
{-0.0124523,-0.3696683,-0.0275352},
{-0.0123774,-0.3696187,-0.0267343},
{-0.0123025,-0.3695690,-0.0259334},
{-0.0122276,-0.3695193,-0.0251325},
{-0.0121527,-0.3694696,-0.0243317},
{-0.0120778,-0.3694199,-0.0235308},
{-0.0120029,-0.3693703,-0.0227299},
{-0.0119280,-0.3693206,-0.0219290},
{-0.0118532,-0.3692709,-0.0211281},
{-0.0117783,-0.3692212,-0.0203272},
{-0.0117034,-0.3691715,-0.0195263},
{-0.0116285,-0.3691219,-0.0187255},
{-0.0115536,-0.3690722,-0.0179246},
{-0.0114787,-0.3690225,-0.0171237},
{-0.0114038,-0.3689728,-0.0163228},
{-0.0113289,-0.3689231,-0.0155219},
{-0.0112541,-0.3688735,-0.0147210},
{-0.0111792,-0.3688238,-0.0139201},

{-0.0111043,-0.3687741,-0.0131192},
{-0.0110294,-0.3687244,-0.0123184},
{-0.0109545,-0.3686748,-0.0115175},
{-0.0108796,-0.3686251,-0.0107166},
{-0.0108047,-0.3685754,-0.0099157},
{-0.0107298,-0.3685257,-0.0091148},
{-0.0106550,-0.3684760,-0.0083139},
{-0.0105801,-0.3684264,-0.0075130},
{-0.0105052,-0.3683767,-0.0067121},
{-0.0104303,-0.3683270,-0.0059113},
{-0.0103554,-0.3682773,-0.0051104},
{-0.0102805,-0.3682276,-0.0043095},
{-0.0102056,-0.3681780,-0.0035086},
{-0.0101307,-0.3681283,-0.0027077},
{-0.0100559,-0.3680786,-0.0019068},
{-0.0099810,-0.3680289,-0.0011059},
{-0.0099061,-0.3679792,-0.0003051},
{-0.0098312,-0.3679296,0.0004958},
{-0.0097563,-0.3678799,0.0012967},
{-0.0096814,-0.3678302,0.0020976},
{-0.0096065,-0.3677805,0.0028985},
{-0.0095317,-0.3677308,0.0036994},
{-0.0094568,-0.3676812,0.0045003},
{-0.0093819,-0.3676315,0.0053012},
{-0.0093070,-0.3675818,0.0061020},
{-0.0092321,-0.3675321,0.0069029},
{-0.0091572,-0.3674825,0.0077038},
{-0.0090823,-0.3674328,0.0085047},
{-0.0090074,-0.3673831,0.0093056},
{-0.0089326,-0.3673334,0.0101065},
{-0.0088577,-0.3672837,0.0109074},
{-0.0087828,-0.3672341,0.0117082},
{-0.0087079,-0.3671844,0.0125091},
{-0.0086330,-0.3671347,0.0133100},
{-0.0085581,-0.3670850,0.0141109},
{-0.0084832,-0.3670353,0.0149118},
{-0.0084083,-0.3669857,0.0157127},
{-0.0083335,-0.3669360,0.0165136},
{-0.0082586,-0.3668863,0.0173145},
{-0.0081837,-0.3668366,0.0181153},
{-0.0081088,-0.3667869,0.0189162},
{-0.0080339,-0.3667373,0.0197171},
{-0.0079590,-0.3666876,0.0205180},
{-0.0078841,-0.3666379,0.0213189},
{-0.0078092,-0.3665882,0.0221198},
{-0.0077344,-0.3665386,0.0229207},

Biology Open • Supplementary information

{-0.0076595,-0.3664889,0.0237215},
{-0.0075846,-0.3664392,0.0245224},
{-0.0075097,-0.3663895,0.0253233},
{-0.0074348,-0.3663398,0.0261242},
{-0.0073599,-0.3662902,0.0269251},
{-0.0072850,-0.3662405,0.0277260},
{-0.0072101,-0.3661908,0.0285269},
{-0.0071353,-0.3661411,0.0293278},
{-0.0070604,-0.3660914,0.0301286},
{-0.0069855,-0.3660418,0.0309295},
{-0.0069106,-0.3659921,0.0317304},
{-0.0068357,-0.3659424,0.0325313},
{-0.0067608,-0.3658927,0.0333322},
{-0.0066859,-0.3658430,0.0341331},
{-0.0066111,-0.3657934,0.0349340},
{-0.0065362,-0.3657437,0.0357348},
{-0.0064613,-0.3656940,0.0365357},
{-0.0063864,-0.3656443,0.0373366},
{-0.0063115,-0.3655947,0.0381375},
{-0.0062366,-0.3655450,0.0389384},
{-0.0061617,-0.3654953,0.0397393},
{-0.0060868,-0.3654456,0.0405402}
}* .AMirroring);
Points1 = ({
{0.0000000,0.0000000,0.0000000},
{-0.0000000,-0.4027836,0.0000000},
{-0.0092973,-0.4096858,0.0012357},
{-0.0000000,-0.4076626,0.0388999},
{-0.0000000,-0.3979047,-0.0388999},
{0.0138406,-0.0090516,0.0558018},
{0.0200827,-0.0235413,0.0439186},
{0.0096519,-0.0267018,0.0608753},
{0.0001560,-0.0004164,0.0496718},
{0.0112087,-0.0071597,0.0387099},
{-0.0059089,-0.0146640,0.0631350},
{0.0168096,-0.0405309,0.0503041},
{0.0150607,-0.0351936,0.0323243},
{0.0142258,-0.0151538,0.0244551},
{-0.0101224,-0.0336577,0.0602407},
{0.0033533,-0.0440599,0.0592468},
{-0.0030700,-0.0210978,0.0401842},
{-0.0124617,-0.0045279,0.0468007},
{-0.0000089,-0.0044416,0.0271534},
{-0.0122367,-0.0240260,0.0481094},
{0.0100535,-0.0637681,0.0528501},
{0.0159885,-0.0566508,0.0376507},

{0.0083346,-0.0478354,0.0188791},
{0.0096387,-0.0254757,0.0123163},
{0.0182644,-0.0057799,0.0056298},
{0.0103352,0.0063725,0.0157115},
{-0.0121733,-0.0400764,0.0358028},
{-0.0100003,-0.0520971,0.0508685},
{-0.0091806,-0.0268175,0.0245777},
{-0.0060953,0.0112178,0.0157402},
{-0.0113117,-0.0061715,0.0155160},
{-0.0074032,-0.0736756,0.0441138},
{0.0062648,-0.0845411,0.0512458},
{0.0170258,-0.0792487,0.0363197},
{0.0082631,-0.0682460,0.0207443},
{-0.0116967,-0.0484572,0.0203871},
{-0.0041740,-0.0344444,0.0100732},
{-0.0004415,-0.0209057,-0.0047422},
{0.0127196,-0.0163849,-0.0064387},
{0.0171868,-0.0018835,-0.0109960},
{0.0168519,0.0136467,-0.0011970},
{0.0031147,0.0207097,0.0088920},
{-0.0141432,-0.0601526,0.0334530},
{-0.0117504,-0.0131443,0.0057260},
{-0.0120271,0.0178560,0.0015880},
{-0.0183473,0.0024754,0.0031330},
{-0.0067502,-0.0816063,0.0273949},
{-0.0042715,-0.0978470,0.0415390},
{0.0144938,-0.1027625,0.0442775},
{0.0123723,-0.0878002,0.0215096},
{-0.0100432,-0.0651775,0.0211758},
{-0.0144115,-0.0093022,-0.0101012},
{0.0006526,-0.0096068,-0.0184608},
{0.0059279,0.0081700,-0.0169925},
{0.0016679,0.0211336,-0.0080282},
{-0.0104330,0.0088416,-0.0143647},
{0.0004393,-0.1029392,0.0230382},
{-0.0018451,-0.1170317,0.0396092},
{0.0156088,-0.1239112,0.0422435},
{0.0186180,-0.1065240,0.0260243},
{0.0061907,-0.1209464,0.0191095},
{0.0000137,-0.1366534,0.0346842},
{0.0167071,-0.1450961,0.0400616},
{0.0222202,-0.1292962,0.0255485},
{0.0096311,-0.1407837,0.0163264},
{0.0023178,-0.1568870,0.0318495},
{0.0190108,-0.1665138,0.0373701},
{0.0245381,-0.1513035,0.0229666},

Biology Open • Supplementary information

{0.0113746,-0.1622210,0.0139309},
{0.0038116,-0.1770975,0.0286605},
{0.0193102,-0.1881708,0.0350799},
{0.0260791,-0.1713140,0.0208088},
{0.0127429,-0.1830923,0.0117883},
{0.0026201,-0.1979627,0.0212102},
{0.0138967,-0.2091374,0.0313009},
{0.0273640,-0.2069636,0.0238265},
{0.0264244,-0.1906681,0.0166819},
{0.0159229,-0.2067746,0.0087720},
{0.0023329,-0.2191978,0.0181105},
{0.0110072,-0.2346347,0.0273242},
{0.0245724,-0.2267813,0.0282061},
{0.0266761,-0.2245388,0.0128870},
{0.0140626,-0.2315886,0.0068001},
{0.0019698,-0.2442894,0.0153596},
{0.0120544,-0.2582645,0.0262070},
{0.0253664,-0.2470404,0.0251903},
{0.0265447,-0.2481774,0.0098483},
{0.0126537,-0.2563000,0.0044389},
{0.0028636,-0.2679624,0.0146362},
{0.0097435,-0.2820872,0.0239789},
{0.0257822,-0.2703904,0.0222380},
{0.0257085,-0.2695159,0.0060201},
{0.0099393,-0.2813983,0.0023809},
{0.0025528,-0.2968419,0.0104930},
{0.0108702,-0.3044680,0.0237002},
{0.0253680,-0.2934196,0.0204975},
{0.0259142,-0.2886104,0.0039387},
{0.0151468,-0.3019492,-0.0027641},
{0.0038366,-0.3186990,0.0007464},
{0.0024870,-0.3211394,0.0178843},
{0.0216323,-0.3206310,0.0212765},
{0.0283123,-0.3118095,0.0080532},
{0.0224286,-0.3248240,-0.0047389},
{0.0063223,-0.3371008,-0.0065390},
{-0.0001644,-0.3412429,0.0089199},
{0.0115964,-0.3391236,0.0231160},
{0.0283880,-0.3374742,0.0108843},
{0.0222100,-0.3460352,-0.0080283},
{0.0044687,-0.3580347,-0.0118053},
{-0.0021300,-0.3635929,0.0041301},
{0.0016048,-0.3566718,0.0207591},
{0.0218791,-0.3560202,0.0204907},
{0.0278605,-0.3594886,0.0049020},
{0.0211214,-0.3670769,-0.0126134},

Biology Open • Supplementary information

{0.0095733,-0.3805973,-0.0233055},
{-0.0050515,-0.3759356,-0.0131227},
{-0.0061400,-0.3883604,0.0027327},
{-0.0056554,-0.3795064,0.0193391},
{0.0100184,-0.3748485,0.0251747},
{0.0269405,-0.3771063,0.0171825},
{0.0257690,-0.3821838,-0.0001417},
{0.0244929,-0.3905479,-0.0173287},
{0.0170530,-0.4049322,-0.0288592},
{0.0041119,-0.3946862,-0.0363951},
{-0.0090731,-0.3854844,-0.0281152},
{-0.0156786,-0.3945510,-0.0120707},
{-0.0058031,-0.4109473,-0.0031506},
{-0.0168219,-0.4023231,0.0104929},
{-0.0146098,-0.3950018,0.0285538},
{0.0020325,-0.3906672,0.0334262},
{0.0200778,-0.3951894,0.0266671},
{0.0339809,-0.3962512,0.0151859},
{0.0262757,-0.4028538,-0.0017731},
{0.0284460,-0.4166717,-0.0156019},
{0.0167082,-0.4293416,-0.0264459},
{0.0024399,-0.4165124,-0.0378454},
{-0.0130304,-0.4035849,-0.0383195},
{-0.0264585,-0.3972879,-0.0271551},
{-0.0262488,-0.4120056,-0.0115145},
{-0.0167564,-0.4297932,-0.0169552},
{0.0001498,-0.4295168,-0.0112496},
{0.0028838,-0.4270649,0.0071999},
{-0.0137334,-0.4243956,0.0133703},
{-0.0236053,-0.4121848,0.0271720},
{-0.0042697,-0.4095078,0.0385596},
{0.0126917,-0.4134137,0.0318485},
{0.0309588,-0.4146546,0.0234111},
{0.0258681,-0.4193999,0.0069033},
{0.0160082,-0.4257241,-0.0062274},
{-0.0001385,-0.4355248,-0.0288765},
{-0.0138176,-0.4269867,-0.0366066},
{-0.0287145,-0.4177087,-0.0306219},
{0.0187237,-0.4304000,0.0200934},
{0.0014986,-0.4329870,0.0263568},
{-0.0139744,-0.4285118,0.0321063},
{-0.0008062,-0.0700636,0.0184992},
{-0.0096648,-0.0609918,0.0200609},
{0.0092127,-0.0357787,0.0600361},
{-0.0132930,-0.0145381,0.0553378},
{-0.0016295,-0.0595979,0.0556326},

{-0.0058964,-0.0669269,0.0218265},
{-0.0013206,-0.0859956,0.0199313},
{-0.0065476,0.0017767,-0.0165156},
{-0.0116728,-0.0467752,0.0399757},
{0.0030952,-0.0278817,0.0636529},
{-0.0095173,-0.0691372,0.0249649},
{-0.0009995,-0.0568230,0.0178263},
{-0.0123421,-0.0178447,0.0493252},
{0.0127530,-0.0315567,0.0584055},
{-0.0098034,-0.0313431,0.0385506},
{0.0133865,-0.0360694,0.0567704},
{-0.0008347,-0.0784972,0.0521442},
{-0.0138820,-0.0459545,0.0303519},
{-0.0130798,-0.0582730,0.0213519},
{-0.0152314,-0.0569950,0.0225858},
{-0.0023235,-0.0433137,0.0131796},
{0.0101458,-0.0607182,0.0231946},
{0.0203262,-0.0138877,0.0384112},
{-0.0029272,-0.0461407,0.0601236},
{0.0127083,-0.0029408,0.0167124},
{-0.0083035,-0.0509771,0.0183540},
{-0.0055976,-0.0847444,0.0430478},
{0.0052355,0.0154759,0.0127315},
{-0.0106123,0.0178057,-0.0058364},
{-0.0066280,-0.0704037,0.0460779},
{0.0073627,-0.0885506,0.0188226},
{0.0121422,-0.0776785,0.0235624},
{0.0072750,-0.0493120,0.0570166},
{0.0135617,-0.0131801,0.0073590},
{-0.0054276,-0.0245760,0.0044384},
{-0.0067551,-0.0010137,0.0194661},
{-0.0094904,-0.0586949,0.0474140},
{-0.0058211,-0.0273082,0.0637136},
{0.0079061,-0.0066308,0.0581914},
{0.0127882,-0.0527138,0.0287084},
{-0.0018863,-0.0189126,-0.0116788},
{-0.0129557,-0.0255058,0.0529043},
{0.0150544,0.0138767,-0.0067969},
{-0.0038140,0.0224871,0.0035410},
{0.0005934,-0.0886810,0.0505759},
{-0.0122087,-0.0412285,0.0478970},
{0.0186655,-0.0291539,0.0406710},
{0.0108882,-0.0447520,0.0231212},
{0.0167526,0.0108318,0.0069200},
{0.0193068,0.0021693,0.0064636},
{0.0202102,-0.0020646,-0.0032716},

Biology Open • Supplementary information

{0.0129461,-0.0209672,0.0215692},
{0.0107461,-0.0360914,0.0205937},
{0.0152512,-0.0153012,0.0308556},
{-0.0163093,-0.0510484,0.0253644},
{0.0028221,-0.0399056,0.0617647},
{0.0157784,-0.0470024,0.0367571},
{0.0136382,-0.0105412,0.0126947},
{0.0009631,0.0045713,-0.0185774},
{0.0081394,-0.0007942,-0.0184361},
{0.0097486,-0.0701436,0.0513601},
{0.0139229,-0.0633414,0.0487901},
{0.0163533,-0.0615420,0.0405695},
{0.0165403,-0.0712448,0.0343091},
{-0.0044048,-0.0777329,0.0234801},
{-0.0123751,0.0038811,0.0133500},
{0.0072008,-0.0011662,0.0527180},
{0.0179163,-0.0097162,0.0480538},
{0.0139684,-0.0245575,0.0574996},
{-0.0111089,-0.0371276,0.0241959},
{-0.0171233,-0.0077754,-0.0024395},
{0.0152353,-0.0858390,0.0261527},
{-0.0065034,-0.0059565,-0.0181463},
{0.0038346,0.0146195,-0.0141376},
{-0.0087067,-0.0342662,0.0152651},
{-0.0138585,0.0106922,-0.0099330},
{-0.0188094,0.0051420,-0.0015853},
{-0.0085015,-0.0726318,0.0377290},
{0.0108876,-0.0180490,-0.0009051},
{0.0117179,-0.0470703,0.0547444},
{0.0081345,-0.0191369,-0.0085755},
{0.0094270,-0.0144123,-0.0131434},
{0.0040366,-0.0615142,0.0180780},
{0.0084048,-0.0524749,0.0204826},
{0.0011487,-0.0786677,0.0191501},
{-0.0170992,-0.0580418,0.0288094},
{-0.0056845,-0.0014070,0.0497758},
{-0.0123261,-0.0318505,0.0567765},
{0.0168795,-0.0179472,0.0516479},
{0.0141222,-0.0795978,0.0460602},
{0.0176919,-0.0248270,0.0334781},
{0.0078707,-0.0329393,0.0634993},
{-0.0160302,-0.0033608,0.0094796},
{0.0135936,-0.0166928,0.0574698},
{0.0145997,-0.0633664,0.0311347},
{-0.0143402,-0.0518687,0.0215098},
{-0.0132592,-0.0345163,0.0502450},

Biology Open • Supplementary information

{0.0131107,-0.0430710,0.0291931},
{-0.0073783,-0.0400754,0.0614790},
{0.0062655,-0.0080105,-0.0180043},
{0.0039068,-0.0159613,-0.0144786},
{-0.0135981,-0.0111603,0.0491823},
{-0.0098636,-0.0463256,0.0553539},
{0.0164808,-0.0559421,0.0451633},
{-0.0159585,0.0115581,0.0014988},
{-0.0130548,0.0094926,0.0111350},
{-0.0018004,-0.0703909,0.0523862},
{0.0125579,-0.0096141,0.0325999},
{0.0131284,-0.0036272,0.0526974},
{0.0144379,-0.0059457,0.0412969},
{-0.0120983,-0.0139299,-0.0082270},
{0.0054223,-0.0227362,0.0022510},
{0.0003493,-0.0507822,0.0163070},
{0.0189628,0.0032420,-0.0072684},
{0.0162536,0.0016821,-0.0126029},
{0.0121219,-0.0079895,-0.0151155},
{-0.0124406,0.0014217,-0.0147887},
{-0.0138914,-0.0061914,-0.0126721},
{0.0167683,0.0137061,0.0030695},
{0.0100195,0.0197631,-0.0021971},
{0.0085524,0.0143774,-0.0126649},
{0.0076423,-0.0234060,0.0617994},
{-0.0006738,-0.0089409,0.0609527},
{-0.0001572,-0.0021667,0.0549319},
{0.0051031,-0.0142126,0.0623547},
{0.0079683,0.0076145,0.0158602},
{0.0116953,-0.0180299,0.0150398},
{0.0037382,-0.0261939,0.0634059},
{-0.0086608,-0.0179037,0.0234219},
{-0.0097069,-0.0180950,0.0116921},
{-0.0061524,-0.0103629,0.0261536},
{-0.0143224,-0.0525572,0.0342145},
{-0.0107913,-0.0635359,0.0396028},
{-0.0065867,-0.0301567,0.0090534},
{0.0196877,-0.0191772,0.0446936},
{0.0116336,-0.0087732,0.0267664},
{0.0041110,-0.0009808,0.0234502},
{-0.0034088,-0.0788256,0.0509882},
{-0.0022874,0.0227456,-0.0031598},
{-0.0010872,-0.0137076,0.0378093},
{-0.0104020,-0.0148970,0.0023806},
{0.0123306,-0.0298961,0.0247815},
{0.0098252,-0.0281965,0.0165584},

Biology Open • Supplementary information

{-0.0104691,-0.0072156,0.0508958},
{0.0178246,-0.0344038,0.0463600},
{-0.0114876,-0.0555988,0.0401577},
{-0.0071094,-0.0840340,0.0364067},
{0.0041233,-0.0054521,0.0298169},
{-0.0028438,-0.0047854,-0.0192955},
{-0.0120029,-0.0361063,0.0563504},
{-0.0028643,0.0092495,-0.0163119},
{-0.0041129,0.0172447,-0.0120463},
{0.0177123,-0.0892024,0.0350986},
{0.0035146,-0.0769270,0.0183940},
{0.0156677,-0.0920358,0.0431475},
{0.0091254,0.0199544,0.0052002},
{0.0111779,0.0095126,0.0140865},
{0.0010457,0.0156732,-0.0138238},
{0.0153016,-0.0121542,-0.0085503},
{0.0180200,-0.0079500,-0.0073324},
{-0.0082797,-0.0253340,0.0294045},
{-0.0103770,-0.0360256,0.0310818},
{-0.0127548,-0.0643466,0.0248637},
{-0.0013473,0.0027760,0.0194192},
{0.0039527,0.0076383,0.0167534},
{-0.0023938,-0.0029760,0.0248086},
{0.0002443,-0.0321530,0.0629250},
{0.0065647,-0.0453257,0.0579106},
{-0.0111179,-0.0207245,0.0606634},
{-0.0110922,-0.0274837,0.0599195},
{0.0085881,0.0211477,0.0025601},
{0.0162452,0.0147715,0.0013584},
{-0.0067685,-0.0113962,0.0617639},
{0.0144620,-0.0555081,0.0332307},
{-0.0117863,-0.0496679,0.0464545},
{-0.0142553,-0.0526724,0.0212788},
{0.0104063,-0.0880431,0.0489254},
{-0.0093036,-0.0263609,0.0171187},
{-0.0095896,-0.0160526,0.0164597},
{-0.0042019,0.0167037,0.0122153},
{0.0008490,-0.0711739,0.0540885},
{0.0055887,-0.0656190,0.0542547},
{-0.0021433,-0.0652937,0.0537665},
{0.0157280,-0.0769416,0.0300315},
{-0.0118199,-0.0191031,0.0473272},
{0.0101245,-0.0259556,0.0166633},
{0.0048904,-0.0548167,0.0566416},
{0.0078795,-0.0366348,0.0602204},
{0.0090509,-0.0879922,0.0191883},

Biology Open • Supplementary information

{-0.0064310,-0.0552132,0.0549952},
{0.0170073,-0.0446732,0.0425054},
{-0.0113073,-0.0446549,0.0204437},
{0.0201001,-0.0215090,0.0361597},
{-0.0038219,-0.0635722,0.0196114},
{0.0157917,-0.0020760,0.0123358},
{0.0058547,0.0056326,0.0168977},
{0.0008687,-0.0338031,0.0628112},
{-0.0140595,0.0142966,-0.0049006},
{0.0100609,0.0163303,0.0098135},
{-0.0101213,0.0166620,0.0078743},
{-0.0176498,-0.0057182,-0.0049859},
{-0.0177550,0.0005289,-0.0071521},
{-0.0046483,-0.0181927,0.0640849},
{-0.0124716,-0.0356978,0.0420182},
{0.0052990,-0.0487094,0.0578833},
{0.0117940,-0.0432599,0.0557172},
{-0.0082908,-0.0709620,0.0245052},
{0.0119810,0.0073633,0.0145064},
{0.0172935,-0.0111088,-0.0010829},
{-0.0164318,-0.0083465,0.0048270},
{-0.0121172,-0.0583874,0.0372345},
{-0.0069363,-0.0174757,-0.0035835},
{-0.0122697,-0.0285778,0.0450380},
{0.0044134,-0.0776432,0.0525935},
{0.0151518,-0.0517550,0.0495824},
{-0.0016459,-0.0361321,0.0105430},
{0.0059260,-0.0331806,0.0125668},
{0.0178074,-0.0072234,0.0055262},
{0.0171944,-0.0843958,0.0398683},
{-0.0086631,-0.0196167,0.0240026},
{-0.0008336,0.0134253,0.0153615},
{0.0070233,-0.0707158,0.0202323},
{0.0083418,-0.0646213,0.0536390},
{0.0142483,-0.0134306,-0.0090045},
{0.0005274,-0.0094284,0.0614070},
{-0.0067827,-0.0152738,-0.0135008},
{0.0150777,-0.0730064,0.0447861},
{0.0072929,-0.0190703,-0.0098542},
{-0.0100685,-0.0667516,0.0221236},
{0.0126266,-0.0407090,0.0276459},
{0.0147177,-0.0044283,0.0513124},
{0.0044997,-0.0079072,0.0374031},
{-0.0028797,-0.0639258,0.0542974},
{-0.0121436,-0.0616149,0.0362605},
{-0.0137089,-0.0118224,0.0513466},

{-0.0074542,0.0126941,-0.0139205},
{-0.0162835,0.0059859,0.0077432},
{0.0088656,-0.0704379,0.0210929},
{0.0023652,-0.0402116,0.0608977},
{-0.0128060,-0.0224148,0.0522331},
{0.0061072,-0.0063397,-0.0186705},
{0.0146093,-0.0291952,0.0536915},
{0.0113454,-0.0154695,0.0589163},
{0.0098814,0.0021361,-0.0171970},
{0.0129988,-0.0083612,0.0163314},
{-0.0122711,0.0114018,0.0111073},
{0.0098233,-0.0540960,0.0546906},
{-0.0051831,-0.0392844,0.0622617},
{0.0184368,-0.0297259,0.0475508},
{0.0150270,-0.0135308,-0.0066604},
{-0.0141493,-0.0513287,0.0346907},
{-0.0071607,-0.0753662,0.0430094},
{0.0030339,-0.0537844,0.0573440},
{-0.0041859,-0.0423204,0.0133368},
{0.0105600,0.0109218,-0.0139047},
{-0.0020323,-0.0220721,-0.0008034},
{-0.0086928,-0.0748641,0.0287653},
{-0.0097939,-0.0035761,-0.0167225},
{-0.0027652,0.0221278,-0.0044485},
{-0.0090914,-0.0221683,0.0126277},
{-0.0101854,-0.0657640,0.0219547},
{-0.0056392,-0.0762738,0.0236372},
{0.0009038,0.0120999,-0.0159073},
{0.0188192,-0.0265200,0.0468194},
{-0.0055908,-0.0591851,0.0189674},
{0.0167205,-0.0370454,0.0378381},
{0.0166963,-0.0314024,0.0337116},
{-0.0136192,-0.0122006,-0.0009343},
{0.0125875,0.0142953,0.0096749},
{-0.0126980,-0.0111272,0.0066450},
{-0.0101476,-0.0358894,0.0343018},
{0.0170355,-0.0894538,0.0407177},
{0.0167732,-0.0105331,0.0534497},
{0.0178034,-0.0158597,0.0495172},
{0.0012920,-0.0269842,0.0048200},
{0.0102391,-0.0237953,0.0603023},
{-0.0026470,-0.0097168,0.0301373},
{-0.0182938,-0.0043216,-0.0016686},
{0.0026170,-0.0213946,-0.0036566},
{-0.0091901,-0.0724027,0.0354698},
{-0.0120882,-0.0279670,0.0584715},

Biology Open • Supplementary information

{0.0169783,0.0052803,-0.0103295},
{-0.0140165,-0.0633449,0.0289120},
{-0.0144548,-0.0042715,0.0122770},
{0.0113823,-0.0081745,0.0259614},
{-0.0127451,-0.0435743,0.0307505},
{0.0142796,-0.0727714,0.0276256},
{0.0007301,0.0034256,-0.0188199},
{0.0160201,-0.0787802,0.0301427},
{0.0065877,0.0064859,0.0161909},
{-0.0135560,0.0022967,0.0124315},
{0.0055517,-0.0436167,0.0159366},
{-0.0061447,-0.0193327,0.0326623},
{0.0195018,-0.0273892,0.0412577},
{-0.0122475,-0.0635951,0.0220758},
{0.0197916,0.0051952,-0.0029239},
{-0.0131685,-0.0637636,0.0268987},
{0.0106910,-0.0227228,0.0107238},
{0.0187336,-0.0118767,0.0440138},
{0.0082214,0.0019760,-0.0181062},
{0.0140032,-0.0084344,0.0131252},
{-0.0107651,-0.0645418,0.0389780},
{0.0004398,0.0209104,0.0089366},
{0.0026979,0.0033544,-0.0191283},
{0.0358316,-0.4093113,0.0204407},
{0.0048560,0.0207198,-0.0075735},
{-0.0129749,-0.0456861,0.0363015},
{-0.0301531,-0.4051661,-0.0261921},
{0.0022257,0.0234407,-0.0000311},
{0.0138046,-0.0249048,0.0258859},
{-0.0135597,-0.0011167,-0.0141459},
{0.0159009,-0.0314486,0.0515995},
{-0.0140759,0.0136495,0.0046176},
{0.0165773,-0.0406842,0.0389945},
{0.0147947,-0.0453872,0.0334473},
{-0.0127487,-0.0358005,0.0445752},
{0.0166461,-0.0574868,0.0429004},
{-0.0160676,-0.0499553,0.0267723},
{0.0029357,-0.0570726,0.0566141},
{0.0010447,-0.0532410,0.0169076},
{0.0205671,-0.0216939,0.0428867},
{0.0075935,-0.0792751,0.0513187},
{-0.0057131,-0.0860662,0.0272021},
{-0.0182276,-0.0022336,0.0029734},
{0.0138163,-0.0139058,0.0192220},
{0.0132884,-0.0690973,0.0267461},
{-0.0089206,-0.0256161,0.0267871},

{-0.0187146,-0.4154108,0.0347562},
{-0.0083043,-0.0578517,0.0507901},
{-0.0109884,-0.4097946,-0.0100056},
{0.0102413,-0.0448518,0.0218010},
{0.0104085,0.0150542,-0.0108947},
{-0.0233673,-0.4064132,0.0281228},
{0.0150018,-0.0551008,0.0340882},
{0.0002861,-0.0513804,0.0582573},
{-0.0105306,0.0187595,0.0027682},
{-0.0023656,-0.0173131,0.0639490},
{-0.0069936,-0.0076406,0.0234694},
{0.0185781,-0.0106847,0.0410797},
{0.0142273,-0.0253938,0.0556540},
{-0.0135473,-0.0113087,0.0506343},
{0.0189881,0.0085394,-0.0027311},
{-0.0096044,-0.0353752,0.0174421},
{-0.0003227,-0.0146434,-0.0159932},
{-0.0103232,-0.0624488,0.0429687},
{-0.0086265,0.0177551,0.0078016},
{0.0093513,0.0198473,-0.0029195},
{-0.0128323,-0.0280341,0.0554505},
{-0.0057291,-0.4069195,0.0393962},
{-0.0128655,-0.4124520,-0.0369248},
{-0.0127942,-0.4123967,-0.0361615},
{-0.0127228,-0.4123413,-0.0353983},
{-0.0126514,-0.4122860,-0.0346351},
{-0.0125801,-0.4122307,-0.0338719},
{-0.0125087,-0.4121754,-0.0331087},
{-0.0124374,-0.4121200,-0.0323455},
{-0.0123660,-0.4120647,-0.0315823},
{-0.0122946,-0.4120094,-0.0308191},
{-0.0122233,-0.4119541,-0.0300559},
{-0.0121519,-0.4118987,-0.0292927},
{-0.0120805,-0.4118434,-0.0285295},
{-0.0120092,-0.4117881,-0.0277662},
{-0.0119378,-0.4117328,-0.0270030},
{-0.0118664,-0.4116774,-0.0262398},
{-0.0117951,-0.4116221,-0.0254766},
{-0.0117237,-0.4115668,-0.0247134},
{-0.0116524,-0.4115115,-0.0239502},
{-0.0115810,-0.4114561,-0.0231870},
{-0.0115096,-0.4114008,-0.0224238},
{-0.0114383,-0.4113455,-0.0216606},
{-0.0113669,-0.4112902,-0.0208974},
{-0.0112955,-0.4112348,-0.0201342},
{-0.0112242,-0.4111795,-0.0193709},

Biology Open • Supplementary information

{-0.0111528,-0.4111242,-0.0186077},
{-0.0110814,-0.4110689,-0.0178445},
{-0.0110101,-0.4110135,-0.0170813},
{-0.0109387,-0.4109582,-0.0163181},
{-0.0108673,-0.4109029,-0.0155549},
{-0.0107960,-0.4108476,-0.0147917},
{-0.0107246,-0.4107922,-0.0140285},
{-0.0106533,-0.4107369,-0.0132653},
{-0.0105819,-0.4106816,-0.0125021},
{-0.0105105,-0.4106263,-0.0117388},
{-0.0104392,-0.4105709,-0.0109756},
{-0.0103678,-0.4105156,-0.0102124},
{-0.0102964,-0.4104603,-0.0094492},
{-0.0102251,-0.4104050,-0.0086860},
{-0.0101537,-0.4103497,-0.0079228},
{-0.0100823,-0.4102943,-0.0071596},
{-0.0100110,-0.4102390,-0.0063964},
{-0.0099396,-0.4101837,-0.0056332},
{-0.0098682,-0.4101284,-0.0048700},
{-0.0097969,-0.4100730,-0.0041068},
{-0.0097255,-0.4100177,-0.0033435},
{-0.0096542,-0.4099624,-0.0025803},
{-0.0095828,-0.4099071,-0.0018171},
{-0.0095114,-0.4098517,-0.0010539},
{-0.0094401,-0.4097964,-0.0002907},
{-0.0093687,-0.4097411,0.0004725},
{-0.0092973,-0.4096858,0.0012357},
{-0.0092260,-0.4096304,0.0019989},
{-0.0091546,-0.4095751,0.0027621},
{-0.0090832,-0.4095198,0.0035253},
{-0.0090119,-0.4094645,0.0042886},
{-0.0089405,-0.4094091,0.0050518},
{-0.0088691,-0.4093538,0.0058150},
{-0.0087978,-0.4092985,0.0065782},
{-0.0087264,-0.4092432,0.0073414},
{-0.0086551,-0.4091878,0.0081046},
{-0.0085837,-0.4091325,0.0088678},
{-0.0085123,-0.4090772,0.0096310},
{-0.0084410,-0.4090219,0.0103942},
{-0.0083696,-0.4089665,0.0111574},
{-0.0082982,-0.4089112,0.0119206},
{-0.0082269,-0.4088559,0.0126839},
{-0.0081555,-0.4088006,0.0134471},
{-0.0080841,-0.4087452,0.0142103},
{-0.0080128,-0.4086899,0.0149735},
{-0.0079414,-0.4086346,0.0157367},

Biology Open • Supplementary information

```
                    {-0.0078701,-0.4085793,0.0164999},
                    {-0.0077987,-0.4085239,0.0172631},
                    {-0.0077273,-0.4084686,0.0180263},
                    {-0.0076560,-0.4084133,0.0187895},
                    {-0.0075846,-0.4083580,0.0195527},
                    {-0.0075132,-0.4083026,0.0203159},
                    {-0.0074419,-0.4082473,0.0210792},
                    {-0.0073705,-0.4081920,0.0218424},
                    {-0.0072991,-0.4081367,0.0226056},
                    {-0.0072278,-0.4080813,0.0233688},
                    {-0.0071564,-0.4080260,0.0241320},
                    {-0.0070850,-0.4079707,0.0248952},
                    {-0.0070137,-0.4079154,0.0256584},
                    {-0.0069423,-0.4078600,0.0264216},
                    {-0.0068710,-0.4078047,0.0271848},
                    {-0.0067996,-0.4077494,0.0279480},
                    {-0.0067282,-0.4076941,0.0287113},
                    {-0.0066569,-0.4076387,0.0294745},
                    {-0.0065855,-0.4075834,0.0302377},
                    {-0.0065141,-0.4075281,0.0310009},
                    {-0.0064428,-0.4074728,0.0317641},
                    {-0.0063714,-0.4074174,0.0325273},
                    {-0.0063000,-0.4073621,0.0332905},
                    {-0.0062287,-0.4073068,0.0340537},
                    {-0.0061573,-0.4072515,0.0348169},
                    {-0.0060859,-0.4071962,0.0355801},
                    {-0.0060146,-0.4071408,0.0363433},
                    {-0.0059432,-0.4070855,0.0371066},
                    {-0.0058719,-0.4070302,0.0378698},
                    {-0.0058005,-0.4069749,0.0386330}
                    }* .AMirroring);
            BoundingBoxOnOff = Off;
      };
      AnyFunTransform3DIdentity ScaleFunction = {
            PreTransforms = {&.RBFTransform};
};
};

};
};
```

Biology Open • Supplementary information

ScalingFunctionTLEMLucyFemur_2014013

```
AnyFolder ScalingFunctionTLEMLucyFemur = {
  AnyFolder Right = {
        AnyFolder Thigh = {
        AnyFunTransform3DRBF RBFTransform = {
              RBFDef.Type = RBF_ThinPlate;
              PolynomDegree = 1;
              Points0 = {
                    {0.0000000,0.0000000,0.0000000},
                    {-0.0000000,-0.3616821,0.0000000},
                    {-0.0097563,-0.3678799,0.0012967},
                    {-0.0000000,-0.3660632,0.0408203},
                    {-0.0000000,-0.3573010,-0.0408203},
                    {0.0161460,-0.0072838,0.0601290},
                    {0.0220217,-0.0203698,0.0463848},
                    {0.0123977,-0.0241932,0.0668573},
                    {0.0006898,0.0018121,0.0538181},
                    {0.0122809,-0.0068668,0.0414535},
                    {-0.0058991,-0.0138188,0.0648412},
                    {0.0211469,-0.0380855,0.0538111},
                    {0.0172133,-0.0317342,0.0328381},
                    {0.0177311,-0.0131946,0.0256176},
                    {-0.0110079,-0.0308867,0.0632370},
                    {0.0039435,-0.0395977,0.0638271},
                    {-0.0041683,-0.0187472,0.0413214},
                    {-0.0177999,-0.0021535,0.0496084},
                    {0.0001603,-0.0053126,0.0279593},
                    {-0.0211600,-0.0216243,0.0497147},
                    {0.0096163,-0.0568663,0.0545719},
                    {0.0162112,-0.0515013,0.0395230},
                    {0.0093533,-0.0433265,0.0187387},
                    {0.0119398,-0.0238469,0.0118403},
                    {0.0210707,-0.0048848,0.0065878},
                    {0.0125013,0.0064094,0.0177702},
                    {-0.0191523,-0.0356053,0.0382429},
                    {-0.0115821,-0.0474937,0.0537648},
                    {-0.0100073,-0.0236371,0.0267322},
                    {-0.0078925,0.0107098,0.0182833},
                    {-0.0134217,-0.0055356,0.0182000},
                    {-0.0090061,-0.0665700,0.0475484},
                    {0.0071095,-0.0761976,0.0520035},
                    {0.0182119,-0.0704149,0.0386546},
                    {0.0073764,-0.0609481,0.0221006},
                    {-0.0122984,-0.0439585,0.0198428},
                    {-0.0032253,-0.0306792,0.0110995},
```

Biology Open • Supplementary information

{-0.0012975,-0.0198451,-0.0052592},
{0.0140405,-0.0161125,-0.0071649},
{0.0189222,-0.0014898,-0.0127226},
{0.0181642,0.0132812,-0.0011050},
{0.0045119,0.0200001,0.0091189},
{-0.0176761,-0.0541983,0.0348587},
{-0.0136548,-0.0142015,0.0054311},
{-0.0135510,0.0182940,0.0017175},
{-0.0223865,0.0023399,0.0028018},
{-0.0086455,-0.0738247,0.0283843},
{-0.0050356,-0.0877749,0.0442195},
{0.0160135,-0.0919319,0.0465120},
{0.0128290,-0.0785810,0.0232431},
{-0.0168328,-0.0616327,0.0155917},
{-0.0172369,-0.0094567,-0.0119502},
{0.0002090,-0.0093641,-0.0209779},
{0.0064827,0.0081538,-0.0198453},
{0.0010777,0.0210422,-0.0084721},
{-0.0129485,0.0085451,-0.0162212},
{-0.0002322,-0.0924033,0.0245644},
{-0.0028175,-0.1052263,0.0418294},
{0.0172322,-0.1111444,0.0445760},
{0.0203259,-0.0957409,0.0271449},
{0.0057102,-0.1088933,0.0195113},
{-0.0010852,-0.1230776,0.0363132},
{0.0182727,-0.1303312,0.0422884},
{0.0242891,-0.1161703,0.0265054},
{0.0094531,-0.1268584,0.0162294},
{0.0014244,-0.1413293,0.0331653},
{0.0207271,-0.1496427,0.0394157},
{0.0267562,-0.1360142,0.0236798},
{0.0113839,-0.1461651,0.0136024},
{0.0030845,-0.1595094,0.0297156},
{0.0209528,-0.1691386,0.0369775},
{0.0283958,-0.1540209,0.0213795},
{0.0129499,-0.1649012,0.0113589},
{0.0016490,-0.1783108,0.0216287},
{0.0147105,-0.1880678,0.0328614},
{0.0298491,-0.1859865,0.0248551},
{0.0286645,-0.1714377,0.0169488},
{0.0165897,-0.1861006,0.0082595},
{0.0014000,-0.1973476,0.0183740},
{0.0114301,-0.2109792,0.0286157},
{0.0267422,-0.2037622,0.0296951},
{0.0288630,-0.2018335,0.0130127},
{0.0145471,-0.2083505,0.0062833},

Biology Open • Supplementary information

{0.0011300,-0.2198071,0.0155602},
{0.0126610,-0.2321235,0.0275354},
{0.0275647,-0.2219268,0.0265185},
{0.0286645,-0.2230253,0.0098701},
{0.0130434,-0.2304822,0.0039177},
{0.0022984,-0.2409585,0.0149650},
{0.0101315,-0.2534740,0.0252125},
{0.0279485,-0.2428555,0.0234276},
{0.0276684,-0.2421559,0.0058691},
{0.0101250,-0.2529527,0.0018983},
{0.0021376,-0.2667831,0.0107073},
{0.0114309,-0.2734905,0.0249960},
{0.0273923,-0.2634953,0.0216324},
{0.0278429,-0.2592546,0.0037692},
{0.0159101,-0.2712985,-0.0033988},
{0.0036708,-0.2863280,0.0004644},
{0.0023115,-0.2884756,0.0187554},
{0.0231385,-0.2879051,0.0224853},
{0.0304009,-0.2799973,0.0083549},
{0.0238189,-0.2917246,-0.0052462},
{0.0064904,-0.3027687,-0.0070587},
{-0.0003492,-0.3064690,0.0093014},
{0.0122179,-0.3045269,0.0243511},
{0.0301837,-0.3030127,0.0114462},
{0.0234182,-0.3107336,-0.0085198},
{0.0046893,-0.3214995,-0.0123881},
{-0.0022352,-0.3264905,0.0043340},
{0.0016841,-0.3202756,0.0217839},
{0.0229722,-0.3196887,0.0215083},
{0.0292438,-0.3228042,0.0051430},
{0.0221641,-0.3296190,-0.0132361},
{0.0100459,-0.3417597,-0.0244560},
{-0.0053009,-0.3375737,-0.0137705},
{-0.0064432,-0.3487307,0.0028676},
{-0.0059346,-0.3407801,0.0202938},
{0.0105130,-0.3365976,0.0264175},
{0.0282695,-0.3386249,0.0180307},
{0.0270402,-0.3431844,-0.0001487},
{0.0257040,-0.3506950,-0.0181851},
{0.0178948,-0.3636114,-0.0302838},
{0.0043149,-0.3544109,-0.0381949},
{-0.0095210,-0.3461482,-0.0295031},
{-0.0164526,-0.3542895,-0.0126666},
{-0.0060896,-0.3690127,-0.0033061},
{-0.0176523,-0.3612686,0.0110109},
{-0.0153310,-0.3546943,0.0299634},

Biology Open • Supplementary information

{0.0021329,-0.3508021,0.0350763},
{0.0210690,-0.3548628,0.0279835},
{0.0356594,-0.3558162,0.0159356},
{0.0275718,-0.3617450,-0.0018607},
{0.0298503,-0.3741530,-0.0163721},
{0.0175320,-0.3855300,-0.0277515},
{0.0025604,-0.3740099,-0.0397137},
{-0.0136727,-0.3624016,-0.0402102},
{-0.0277647,-0.3567472,-0.0284947},
{-0.0275446,-0.3699630,-0.0120829},
{-0.0175846,-0.3859355,-0.0177922},
{0.0001572,-0.3856873,-0.0118050},
{0.0030262,-0.3834856,0.0075553},
{-0.0144114,-0.3810887,0.0140304},
{-0.0247706,-0.3701239,0.0285134},
{-0.0044804,-0.3677201,0.0404632},
{0.0133183,-0.3712274,0.0334208},
{0.0324861,-0.3723417,0.0245668},
{0.0271451,-0.3766027,0.0072441},
{0.0167985,-0.3822816,-0.0065349},
{-0.0001454,-0.3910823,-0.0303010},
{-0.0144998,-0.3834154,-0.0384137},
{-0.0301321,-0.3750841,-0.0321336},
{0.0196491,-0.3864804,0.0210853},
{0.0015726,-0.3888034,0.0276569},
{-0.0146652,-0.3847848,0.0336913},
{-0.0014588,-0.0619951,0.0219778},
{-0.0118435,-0.0550626,0.0139639},
{0.0129812,-0.0349966,0.0673128},
{-0.0162907,-0.0105666,0.0597739},
{-0.0012885,-0.0520816,0.0562989},
{-0.0090599,-0.0631298,0.0172224},
{-0.0000222,-0.0771663,0.0231897},
{-0.0068148,0.0023885,-0.0160086},
{-0.0180544,-0.0425409,0.0446585},
{0.0020525,-0.0249904,0.0691549},
{-0.0157097,-0.0662315,0.0238393},
{-0.0010518,-0.0510686,0.0199353},
{-0.0208874,-0.0152865,0.0500034},
{0.0168316,-0.0280857,0.0666680},
{-0.0187392,-0.0282333,0.0403282},
{0.0191622,-0.0351633,0.0622707},
{0.0001216,-0.0706289,0.0524049},
{-0.0169862,-0.0399062,0.0311827},
{-0.0185737,-0.0533066,0.0151224},
{-0.0219058,-0.0528723,0.0222263},

Biology Open • Supplementary information

{-0.0028271,-0.0391620,0.0148452},
{0.0117900,-0.0553425,0.0235348},
{0.0219835,-0.0106400,0.0396338},
{-0.0025366,-0.0401046,0.0625273},
{0.0141017,-0.0018767,0.0198108},
{-0.0078770,-0.0473929,0.0173542},
{-0.0065351,-0.0766431,0.0470258},
{0.0065585,0.0145593,0.0161638},
{-0.0118588,0.0172875,-0.0085064},
{-0.0074087,-0.0627500,0.0506700},
{0.0071940,-0.0780068,0.0213362},
{0.0126736,-0.0708176,0.0244108},
{0.0074694,-0.0436450,0.0604196},
{0.0154186,-0.0139471,0.0064312},
{-0.0053535,-0.0213210,0.0040094},
{-0.0088733,0.0002018,0.0205402},
{-0.0112471,-0.0542608,0.0500266},
{-0.0061625,-0.0248673,0.0653244},
{0.0093695,-0.0065826,0.0614157},
{0.0138015,-0.0467322,0.0284333},
{-0.0020999,-0.0186885,-0.0116392},
{-0.0185640,-0.0225591,0.0566779},
{0.0175798,0.0124360,-0.0068467},
{-0.0039962,0.0217127,0.0053055},
{0.0014975,-0.0784591,0.0514056},
{-0.0172997,-0.0386971,0.0525252},
{0.0196540,-0.0268271,0.0416806},
{0.0135296,-0.0406504,0.0236882},
{0.0182471,0.0095959,0.0087906},
{0.0218667,0.0016804,0.0058793},
{0.0224975,-0.0022652,-0.0022994},
{0.0166750,-0.0191391,0.0236436},
{0.0119785,-0.0328723,0.0195376},
{0.0187631,-0.0133469,0.0308741},
{-0.0187543,-0.0461463,0.0257491},
{0.0039834,-0.0373928,0.0683089},
{0.0157029,-0.0423169,0.0385226},
{0.0164003,-0.0087069,0.0131181},
{0.0008253,0.0033370,-0.0212576},
{0.0101707,-0.0009724,-0.0205688},
{0.0102948,-0.0642683,0.0524532},
{0.0152217,-0.0557944,0.0508978},
{0.0172973,-0.0565707,0.0419468},
{0.0167723,-0.0635334,0.0369834},
{-0.0047397,-0.0695325,0.0241398},
{-0.0152913,0.0035778,0.0161461},

{0.0091697,0.0009340,0.0575160},
{0.0196686,-0.0089533,0.0500006},
{0.0183998,-0.0212943,0.0622968},
{-0.0114340,-0.0316810,0.0250940},
{-0.0206442,-0.0092599,-0.0028425},
{0.0166868,-0.0784295,0.0276925},
{-0.0069678,-0.0054521,-0.0207011},
{0.0049490,0.0151109,-0.0160093},
{-0.0084025,-0.0318937,0.0162788},
{-0.0173679,0.0095594,-0.0108509},
{-0.0218912,0.0063457,-0.0020698},
{-0.0106547,-0.0668750,0.0397749},
{0.0136098,-0.0170666,-0.0018890},
{0.0141599,-0.0441405,0.0587594},
{0.0093255,-0.0183218,-0.0083008},
{0.0106367,-0.0128276,-0.0154269},
{0.0037350,-0.0549365,0.0209786},
{0.0087662,-0.0472902,0.0195496},
{0.0016839,-0.0708213,0.0221325},
{-0.0202977,-0.0530540,0.0301270},
{-0.0068584,0.0021781,0.0530002},
{-0.0144817,-0.0297218,0.0605833},
{0.0197015,-0.0165217,0.0560654},
{0.0156609,-0.0705363,0.0477736},
{0.0207876,-0.0221433,0.0330733},
{0.0092324,-0.0300767,0.0713160},
{-0.0203392,-0.0034488,0.0097332},
{0.0156978,-0.0143438,0.0629053},
{0.0143267,-0.0563519,0.0317489},
{-0.0161685,-0.0465391,0.0188591},
{-0.0196397,-0.0309875,0.0546496},
{0.0154804,-0.0380422,0.0305433},
{-0.0077774,-0.0351303,0.0637765},
{0.0063980,-0.0086419,-0.0201654},
{0.0030146,-0.0151094,-0.0165974},
{-0.0210080,-0.0096030,0.0507855},
{-0.0105684,-0.0411377,0.0586146},
{0.0180619,-0.0498960,0.0469835},
{-0.0192318,0.0116108,0.0024070},
{-0.0165651,0.0083622,0.0126870},
{-0.0014333,-0.0627138,0.0534746},
{0.0145127,-0.0080178,0.0353667},
{0.0148374,-0.0012228,0.0566181},
{0.0153834,-0.0062427,0.0444727},
{-0.0155164,-0.0132103,-0.0096398},
{0.0068685,-0.0221022,0.0011547},

{0.0003332,-0.0458500,0.0183716},
{0.0205582,0.0040956,-0.0082969},
{0.0175752,0.0009340,-0.0148407},
{0.0142357,-0.0063623,-0.0169332},
{-0.0147285,0.0023412,-0.0164678},
{-0.0158781,-0.0064451,-0.0151346},
{0.0175951,0.0133456,0.0037559},
{0.0117660,0.0189899,-0.0030690},
{0.0096040,0.0151827,-0.0137450},
{0.0096565,-0.0215194,0.0662734},
{-0.0006793,-0.0074494,0.0631218},
{0.0007819,-0.0004519,0.0600781},
{0.0065377,-0.0125433,0.0654199},
{0.0090461,0.0073096,0.0188711},
{0.0151418,-0.0168302,0.0146465},
{0.0025166,-0.0231863,0.0688894},
{-0.0097891,-0.0152077,0.0253086},
{-0.0109149,-0.0177935,0.0119825},
{-0.0072079,-0.0099373,0.0272725},
{-0.0183727,-0.0467429,0.0356276},
{-0.0126871,-0.0584555,0.0428141},
{-0.0057054,-0.0273042,0.0103554},
{0.0215142,-0.0159693,0.0474531},
{0.0128492,-0.0070016,0.0287645},
{0.0052134,-0.0017047,0.0247463},
{-0.0024284,-0.0707498,0.0513049},
{-0.0036140,0.0220593,-0.0040484},
{-0.0002405,-0.0121253,0.0390050},
{-0.0119811,-0.0156737,0.0015445},
{0.0142082,-0.0279792,0.0246553},
{0.0122244,-0.0262013,0.0169829},
{-0.0146264,-0.0038855,0.0549384},
{0.0207450,-0.0325771,0.0496412},
{-0.0155040,-0.0497560,0.0437087},
{-0.0081119,-0.0756450,0.0387365},
{0.0039684,-0.0062047,0.0313900},
{-0.0023726,-0.0038892,-0.0220521},
{-0.0136977,-0.0339839,0.0602619},
{-0.0035399,0.0089081,-0.0188894},
{-0.0049820,0.0176571,-0.0132254},
{0.0193902,-0.0803191,0.0360429},
{0.0040648,-0.0697109,0.0218205},
{0.0170470,-0.0818610,0.0456892},
{0.0104175,0.0188635,0.0064965},
{0.0135206,0.0092549,0.0158079},
{0.0023933,0.0160498,-0.0157404},

Biology Open • Supplementary information

{0.0164753,-0.0118197,-0.0096995},
{0.0202584,-0.0066858,-0.0082383},
{-0.0098401,-0.0226001,0.0314028},
{-0.0130937,-0.0309622,0.0320764},
{-0.0201510,-0.0613297,0.0222264},
{-0.0010389,0.0036055,0.0209003},
{0.0042025,0.0073392,0.0199393},
{-0.0021630,-0.0038531,0.0251898},
{-0.0007845,-0.0293574,0.0675788},
{0.0070027,-0.0406327,0.0624565},
{-0.0124363,-0.0183001,0.0627966},
{-0.0120485,-0.0256613,0.0628493},
{0.0101200,0.0197695,0.0039334},
{0.0167636,0.0148266,0.0014155},
{-0.0074981,-0.0106930,0.0632797},
{0.0142533,-0.0503445,0.0351954},
{-0.0146107,-0.0460953,0.0506120},
{-0.0161117,-0.0471301,0.0178900},
{0.0112149,-0.0792793,0.0501460},
{-0.0099060,-0.0230794,0.0186951},
{-0.0114543,-0.0153049,0.0183675},
{-0.0042882,0.0161879,0.0151423},
{0.0014582,-0.0638572,0.0541080},
{0.0048554,-0.0582603,0.0553815},
{-0.0017077,-0.0572302,0.0546635},
{0.0166090,-0.0697169,0.0323038},
{-0.0208812,-0.0164273,0.0474354},
{0.0130454,-0.0241864,0.0178294},
{0.0053123,-0.0483770,0.0582251},
{0.0112225,-0.0361429,0.0673300},
{0.0085729,-0.0771256,0.0216100},
{-0.0070783,-0.0488033,0.0560869},
{0.0182083,-0.0411024,0.0454236},
{-0.0119590,-0.0403809,0.0213546},
{0.0233409,-0.0185535,0.0366343},
{-0.0046347,-0.0571211,0.0208243},
{0.0183166,-0.0006966,0.0140116},
{0.0061913,0.0053512,0.0201003},
{-0.0002364,-0.0310828,0.0680351},
{-0.0170773,0.0139270,-0.0054987},
{0.0110805,0.0156034,0.0117010},
{-0.0124628,0.0164667,0.0094550},
{-0.0209014,-0.0073407,-0.0051969},
{-0.0210131,0.0010693,-0.0086891},
{-0.0041596,-0.0172332,0.0663041},
{-0.0216595,-0.0322172,0.0451540},

{0.0051908,-0.0426014,0.0613013},
{0.0150003,-0.0416136,0.0606257},
{-0.0135090,-0.0674234,0.0236425},
{0.0146416,0.0073691,0.0160052},
{0.0195615,-0.0112412,-0.0010333},
{-0.0198115,-0.0098244,0.0044735},
{-0.0157054,-0.0521236,0.0395039},
{-0.0081381,-0.0173108,-0.0046152},
{-0.0218324,-0.0262661,0.0471115},
{0.0051446,-0.0707257,0.0530555},
{0.0179359,-0.0471493,0.0528134},
{-0.0005591,-0.0317983,0.0113632},
{0.0071707,-0.0305943,0.0121186},
{0.0206239,-0.0061367,0.0066642},
{0.0187057,-0.0745868,0.0420207},
{-0.0095784,-0.0163113,0.0259524},
{-0.0012181,0.0127661,0.0186855},
{0.0059420,-0.0630691,0.0220087},
{0.0069408,-0.0573878,0.0552614},
{0.0150782,-0.0132884,-0.0101793},
{0.0005533,-0.0078574,0.0633775},
{-0.0088284,-0.0146399,-0.0147837},
{0.0167444,-0.0651105,0.0470347},
{0.0082666,-0.0181741,-0.0095133},
{-0.0175162,-0.0637444,0.0177069},
{0.0153795,-0.0360767,0.0287608},
{0.0162363,-0.0018864,0.0548412},
{0.0052731,-0.0075919,0.0392990},
{-0.0022288,-0.0556644,0.0549909},
{-0.0156238,-0.0550038,0.0377688},
{-0.0204137,-0.0100550,0.0536429},
{-0.0093683,0.0122429,-0.0162013},
{-0.0202927,0.0049166,0.0083887},
{0.0074706,-0.0627945,0.0222133},
{0.0035034,-0.0376103,0.0672421},
{-0.0190752,-0.0194129,0.0555530},
{0.0061758,-0.0074961,-0.0207741},
{0.0192011,-0.0273063,0.0588233},
{0.0130796,-0.0134383,0.0638611},
{0.0120421,0.0019566,-0.0194140},
{0.0154152,-0.0065294,0.0179581},
{-0.0157335,0.0101219,0.0123981},
{0.0110376,-0.0488274,0.0567911},
{-0.0055170,-0.0343676,0.0647140},
{0.0216204,-0.0273988,0.0512041},
{0.0161331,-0.0135875,-0.0075501},

Biology Open • Supplementary information

{-0.0184643,-0.0455633,0.0360087},
{-0.0091978,-0.0682413,0.0460847},
{0.0034304,-0.0471677,0.0588866},
{-0.0048025,-0.0384316,0.0149400},
{0.0118505,0.0109735,-0.0159161},
{-0.0027036,-0.0204189,-0.0015094},
{-0.0117607,-0.0692759,0.0292171},
{-0.0107080,-0.0033455,-0.0190487},
{-0.0042548,0.0214947,-0.0056846},
{-0.0097315,-0.0208529,0.0131997},
{-0.0178958,-0.0627218,0.0171551},
{-0.0068692,-0.0688818,0.0233316},
{0.0012083,0.0120695,-0.0186179},
{0.0215073,-0.0236027,0.0503069},
{-0.0060262,-0.0534931,0.0189523},
{0.0179951,-0.0336785,0.0397267},
{0.0190509,-0.0284972,0.0336394},
{-0.0169188,-0.0133609,-0.0014203},
{0.0135722,0.0136491,0.0114695},
{-0.0150549,-0.0123230,0.0067722},
{-0.0148411,-0.0311945,0.0355412},
{0.0185490,-0.0794042,0.0431266},
{0.0193568,-0.0087326,0.0575704},
{0.0201026,-0.0144353,0.0531586},
{0.0025583,-0.0248211,0.0045714},
{0.0132689,-0.0214866,0.0652899},
{-0.0028669,-0.0099311,0.0309271},
{-0.0219487,-0.0054893,-0.0021853},
{0.0026180,-0.0203806,-0.0040620},
{-0.0116230,-0.0668518,0.0370152},
{-0.0133007,-0.0264159,0.0619176},
{0.0186167,0.0050491,-0.0120059},
{-0.0191359,-0.0591690,0.0288149},
{-0.0178121,-0.0041834,0.0141448},
{0.0123077,-0.0063648,0.0279743},
{-0.0157729,-0.0374010,0.0315335},
{0.0149328,-0.0659100,0.0290095},
{0.0006205,0.0021977,-0.0214613},
{0.0170484,-0.0715687,0.0325980},
{0.0068499,0.0061192,0.0196608},
{-0.0167075,0.0020024,0.0150382},
{0.0059801,-0.0393117,0.0167489},
{-0.0072506,-0.0171067,0.0338751},
{0.0202363,-0.0251596,0.0420997},
{-0.0199311,-0.0601255,0.0171522},
{0.0216641,0.0053410,-0.0027841},

{-0.0197665,-0.0606125,0.0252972},
{0.0129986,-0.0214528,0.0098002},
{0.0201151,-0.0100929,0.0460501},
{0.0104137,0.0017765,-0.0203073},
{0.0166557,-0.0064123,0.0140197},
{-0.0124120,-0.0596549,0.0422637},
{0.0014885,0.0202590,0.0096781},
{0.0028720,0.0020207,-0.0219044},
{0.0376095,-0.3675427,0.0214537},
{0.0049949,0.0206417,-0.0079017},
{-0.0191723,-0.0407590,0.0391310},
{-0.0316406,-0.3638215,-0.0274841},
{0.0021392,0.0226920,0.0000985},
{0.0168435,-0.0229382,0.0264788},
{-0.0157304,-0.0005437,-0.0161456},
{0.0203722,-0.0299750,0.0562762},
{-0.0171077,0.0134146,0.0059615},
{0.0175519,-0.0370653,0.0413374},
{0.0154657,-0.0405127,0.0348980},
{-0.0215172,-0.0323502,0.0481925},
{0.0177998,-0.0515776,0.0444709},
{-0.0183804,-0.0448316,0.0275139},
{0.0033607,-0.0502028,0.0575894},
{0.0010325,-0.0479377,0.0191141},
{0.0224852,-0.0181821,0.0451780},
{0.0084089,-0.0720538,0.0521031},
{-0.0070880,-0.0772912,0.0288023},
{-0.0223712,-0.0028990,0.0024047},
{0.0173136,-0.0120017,0.0204612},
{0.0137772,-0.0623537,0.0276621},
{-0.0100390,-0.0231180,0.0292222},
{-0.0196395,-0.3730207,0.0364730},
{-0.0094445,-0.0526688,0.0528111},
{-0.0115309,-0.3679776,-0.0104995},
{0.0129812,-0.0410477,0.0224072},
{0.0117394,0.0154849,-0.0117191},
{-0.0245208,-0.3649412,0.0295111},
{0.0147476,-0.0502966,0.0364899},
{0.0006132,-0.0446771,0.0600524},
{-0.0118123,0.0191139,0.0031977},
{-0.0017185,-0.0163303,0.0664216},
{-0.0085098,-0.0074564,0.0247745},
{0.0197459,-0.0086852,0.0430460},
{0.0187064,-0.0223532,0.0600966},
{-0.0206552,-0.0099712,0.0525024},
{0.0207525,0.0084646,-0.0024846},

Biology Open • Supplementary information

{-0.0094915,-0.0325446,0.0184441},
{-0.0014464,-0.0140050,-0.0179819},
{-0.0122725,-0.0573444,0.0463125},
{-0.0106397,0.0177211,0.0094669},
{0.0111981,0.0191236,-0.0041491},
{-0.0162790,-0.0256279,0.0592687},
{-0.0060120,-0.3653959,0.0413411},
{-0.0135007,-0.3703638,-0.0387476},
{-0.0134258,-0.3703142,-0.0379467},
{-0.0133509,-0.3702645,-0.0371458},
{-0.0132760,-0.3702148,-0.0363450},
{-0.0132011,-0.3701651,-0.0355441},
{-0.0131262,-0.3701154,-0.0347432},
{-0.0130513,-0.3700658,-0.0339423},
{-0.0129765,-0.3700161,-0.0331414},
{-0.0129016,-0.3699664,-0.0323405},
{-0.0128267,-0.3699167,-0.0315396},
{-0.0127518,-0.3698670,-0.0307388},
{-0.0126769,-0.3698174,-0.0299379},
{-0.0126020,-0.3697677,-0.0291370},
{-0.0125271,-0.3697180,-0.0283361},
{-0.0124523,-0.3696683,-0.0275352},
{-0.0123774,-0.3696187,-0.0267343},
{-0.0123025,-0.3695690,-0.0259334},
{-0.0122276,-0.3695193,-0.0251325},
{-0.0121527,-0.3694696,-0.0243317},
{-0.0120778,-0.3694199,-0.0235308},
{-0.0120029,-0.3693703,-0.0227299},
{-0.0119280,-0.3693206,-0.0219290},
{-0.0118532,-0.3692709,-0.0211281},
{-0.0117783,-0.3692212,-0.0203272},
{-0.0117034,-0.3691715,-0.0195263},
{-0.0116285,-0.3691219,-0.0187255},
{-0.0115536,-0.3690722,-0.0179246},
{-0.0114787,-0.3690225,-0.0171237},
{-0.0114038,-0.3689728,-0.0163228},
{-0.0113289,-0.3689231,-0.0155219},
{-0.0112541,-0.3688735,-0.0147210},
{-0.0111792,-0.3688238,-0.0139201},
{-0.0111043,-0.3687741,-0.0131192},
{-0.0110294,-0.3687244,-0.0123184},
{-0.0109545,-0.3686748,-0.0115175},
{-0.0108796,-0.3686251,-0.0107166},
{-0.0108047,-0.3685754,-0.0099157},
{-0.0107298,-0.3685257,-0.0091148},
{-0.0106550,-0.3684760,-0.0083139},

{-0.0105801,-0.3684264,-0.0075130},
{-0.0105052,-0.3683767,-0.0067121},
{-0.0104303,-0.3683270,-0.0059113},
{-0.0103554,-0.3682773,-0.0051104},
{-0.0102805,-0.3682276,-0.0043095},
{-0.0102056,-0.3681780,-0.0035086},
{-0.0101307,-0.3681283,-0.0027077},
{-0.0100559,-0.3680786,-0.0019068},
{-0.0099810,-0.3680289,-0.0011059},
{-0.0099061,-0.3679792,-0.0003051},
{-0.0098312,-0.3679296,0.0004958},
{-0.0097563,-0.3678799,0.0012967},
{-0.0096814,-0.3678302,0.0020976},
{-0.0096065,-0.3677805,0.0028985},
{-0.0095317,-0.3677308,0.0036994},
{-0.0094568,-0.3676812,0.0045003},
{-0.0093819,-0.3676315,0.0053012},
{-0.0093070,-0.3675818,0.0061020},
{-0.0092321,-0.3675321,0.0069029},
{-0.0091572,-0.3674825,0.0077038},
{-0.0090823,-0.3674328,0.0085047},
{-0.0090074,-0.3673831,0.0093056},
{-0.0089326,-0.3673334,0.0101065},
{-0.0088577,-0.3672837,0.0109074},
{-0.0087828,-0.3672341,0.0117082},
{-0.0087079,-0.3671844,0.0125091},
{-0.0086330,-0.3671347,0.0133100},
{-0.0085581,-0.3670850,0.0141109},
{-0.0084832,-0.3670353,0.0149118},
{-0.0084083,-0.3669857,0.0157127},
{-0.0083335,-0.3669360,0.0165136},
{-0.0082586,-0.3668863,0.0173145},
{-0.0081837,-0.3668366,0.0181153},
{-0.0081088,-0.3667869,0.0189162},
{-0.0080339,-0.3667373,0.0197171},
{-0.0079590,-0.3666876,0.0205180},
{-0.0078841,-0.3666379,0.0213189},
{-0.0078092,-0.3665882,0.0221198},
{-0.0077344,-0.3665386,0.0229207},
{-0.0076595,-0.3664889,0.0237215},
{-0.0075846,-0.3664392,0.0245224},
{-0.0075097,-0.3663895,0.0253233},
{-0.0074348,-0.3663398,0.0261242},
{-0.0073599,-0.3662902,0.0269251},
{-0.0072850,-0.3662405,0.0277260},
{-0.0072101,-0.3661908,0.0285269},

{-0.0071353,-0.3661411,0.0293278},
{-0.0070604,-0.3660914,0.0301286},
{-0.0069855,-0.3660418,0.0309295},
{-0.0069106,-0.3659921,0.0317304},
{-0.0068357,-0.3659424,0.0325313},
{-0.0067608,-0.3658927,0.0333322},
{-0.0066859,-0.3658430,0.0341331},
{-0.0066111,-0.3657934,0.0349340},
{-0.0065362,-0.3657437,0.0357348},
{-0.0064613,-0.3656940,0.0365357},
{-0.0063864,-0.3656443,0.0373366},
{-0.0063115,-0.3655947,0.0381375},
{-0.0062366,-0.3655450,0.0389384},
{-0.0061617,-0.3654953,0.0397393},
{-0.0060868,-0.3654456,0.0405402}
};
Points1 = {
{0.0000000,0.0000000,0.0000000},
{-0.0000000,-0.4027836,0.0000000},
{-0.0092973,-0.4096858,0.0012357},
{-0.0000000,-0.4076626,0.0388999},
{-0.0000000,-0.3979047,-0.0388999},
{0.0138406,-0.0090516,0.0558018},
{0.0200827,-0.0235413,0.0439186},
{0.0096519,-0.0267018,0.0608753},
{0.0001560,-0.0004164,0.0496718},
{0.0112087,-0.0071597,0.0387099},
{-0.0059089,-0.0146640,0.0631350},
{0.0168096,-0.0405309,0.0503041},
{0.0150607,-0.0351936,0.0323243},
{0.0142258,-0.0151538,0.0244551},
{-0.0101224,-0.0336577,0.0602407},
{0.0033533,-0.0440599,0.0592468},
{-0.0030700,-0.0210978,0.0401842},
{-0.0124617,-0.0045279,0.0468007},
{-0.0000089,-0.0044416,0.0271534},
{-0.0122367,-0.0240260,0.0481094},
{0.0100535,-0.0637681,0.0528501},
{0.0159885,-0.0566508,0.0376507},
{0.0083346,-0.0478354,0.0188791},
{0.0096387,-0.0254757,0.0123163},
{0.0182644,-0.0057799,0.0056298},
{0.0103352,0.0063725,0.0157115},
{-0.0121733,-0.0400764,0.0358028},
{-0.0100003,-0.0520971,0.0508685},
{-0.0091806,-0.0268175,0.0245777},

{-0.0060953,0.0112178,0.0157402},
{-0.0113117,-0.0061715,0.0155160},
{-0.0074032,-0.0736756,0.0441138},
{0.0062648,-0.0845411,0.0512458},
{0.0170258,-0.0792487,0.0363197},
{0.0082631,-0.0682460,0.0207443},
{-0.0116967,-0.0484572,0.0203871},
{-0.0041740,-0.0344444,0.0100732},
{-0.0004415,-0.0209057,-0.0047422},
{0.0127196,-0.0163849,-0.0064387},
{0.0171868,-0.0018835,-0.0109960},
{0.0168519,0.0136467,-0.0011970},
{0.0031147,0.0207097,0.0088920},
{-0.0141432,-0.0601526,0.0334530},
{-0.0117504,-0.0131443,0.0057260},
{-0.0120271,0.0178560,0.0015880},
{-0.0183473,0.0024754,0.0031330},
{-0.0067502,-0.0816063,0.0273949},
{-0.0042715,-0.0978470,0.0415390},
{0.0144938,-0.1027625,0.0442775},
{0.0123723,-0.0878002,0.0215096},
{-0.0100432,-0.0651775,0.0211758},
{-0.0144115,-0.0093022,-0.0101012},
{0.0006526,-0.0096068,-0.0184608},
{0.0059279,0.0081700,-0.0169925},
{0.0016679,0.0211336,-0.0080282},
{-0.0104330,0.0088416,-0.0143647},
{0.0004393,-0.1029392,0.0230382},
{-0.0018451,-0.1170317,0.0396092},
{0.0156088,-0.1239112,0.0422435},
{0.0186180,-0.1065240,0.0260243},
{0.0061907,-0.1209464,0.0191095},
{0.0000137,-0.1366534,0.0346842},
{0.0167071,-0.1450961,0.0400616},
{0.0222202,-0.1292962,0.0255485},
{0.0096311,-0.1407837,0.0163264},
{0.0023178,-0.1568870,0.0318495},
{0.0190108,-0.1665138,0.0373701},
{0.0245381,-0.1513035,0.0229666},
{0.0113746,-0.1622210,0.0139309},
{0.0038116,-0.1770975,0.0286605},
{0.0193102,-0.1881708,0.0350799},
{0.0260791,-0.1713140,0.0208088},
{0.0127429,-0.1830923,0.0117883},
{0.0026201,-0.1979627,0.0212102},
{0.0138967,-0.2091374,0.0313009},

Biology Open • Supplementary information

{0.0273640,-0.2069636,0.0238265},
{0.0264244,-0.1906681,0.0166819},
{0.0159229,-0.2067746,0.0087720},
{0.0023329,-0.2191978,0.0181105},
{0.0110072,-0.2346347,0.0273242},
{0.0245724,-0.2267813,0.0282061},
{0.0266761,-0.2245388,0.0128870},
{0.0140626,-0.2315886,0.0068001},
{0.0019698,-0.2442894,0.0153596},
{0.0120544,-0.2582645,0.0262070},
{0.0253664,-0.2470404,0.0251903},
{0.0265447,-0.2481774,0.0098483},
{0.0126537,-0.2563000,0.0044389},
{0.0028636,-0.2679624,0.0146362},
{0.0097435,-0.2820872,0.0239789},
{0.0257822,-0.2703904,0.0222380},
{0.0257085,-0.2695159,0.0060201},
{0.0099393,-0.2813983,0.0023809},
{0.0025528,-0.2968419,0.0104930},
{0.0108702,-0.3044680,0.0237002},
{0.0253680,-0.2934196,0.0204975},
{0.0259142,-0.2886104,0.0039387},
{0.0151468,-0.3019492,-0.0027641},
{0.0038366,-0.3186990,0.0007464},
{0.0024870,-0.3211394,0.0178843},
{0.0216323,-0.3206310,0.0212765},
{0.0283123,-0.3118095,0.0080532},
{0.0224286,-0.3248240,-0.0047389},
{0.0063223,-0.3371008,-0.0065390},
{-0.0001644,-0.3412429,0.0089199},
{0.0115964,-0.3391236,0.0231160},
{0.0283880,-0.3374742,0.0108843},
{0.0222100,-0.3460352,-0.0080283},
{0.0044687,-0.3580347,-0.0118053},
{-0.0021300,-0.3635929,0.0041301},
{0.0016048,-0.3566718,0.0207591},
{0.0218791,-0.3560202,0.0204907},
{0.0278605,-0.3594886,0.0049020},
{0.0211214,-0.3670769,-0.0126134},
{0.0095733,-0.3805973,-0.0233055},
{-0.0050515,-0.3759356,-0.0131227},
{-0.0061400,-0.3883604,0.0027327},
{-0.0056554,-0.3795064,0.0193391},
{0.0100184,-0.3748485,0.0251747},
{0.0269405,-0.3771063,0.0171825},
{0.0257690,-0.3821838,-0.0001417},

Biology Open • Supplementary information

{0.0244929,-0.3905479,-0.0173287},
{0.0170530,-0.4049322,-0.0288592},
{0.0041119,-0.3946862,-0.0363951},
{-0.0090731,-0.3854844,-0.0281152},
{-0.0156786,-0.3945510,-0.0120707},
{-0.0058031,-0.4109473,-0.0031506},
{-0.0168219,-0.4023231,0.0104929},
{-0.0146098,-0.3950018,0.0285538},
{0.0020325,-0.3906672,0.0334262},
{0.0200778,-0.3951894,0.0266671},
{0.0339809,-0.3962512,0.0151859},
{0.0262757,-0.4028538,-0.0017731},
{0.0284460,-0.4166717,-0.0156019},
{0.0167082,-0.4293416,-0.0264459},
{0.0024399,-0.4165124,-0.0378454},
{-0.0130304,-0.4035849,-0.0383195},
{-0.0264585,-0.3972879,-0.0271551},
{-0.0262488,-0.4120056,-0.0115145},
{-0.0167564,-0.4297932,-0.0169552},
{0.0001498,-0.4295168,-0.0112496},
{0.0028838,-0.4270649,0.0071999},
{-0.0137334,-0.4243956,0.0133703},
{-0.0236053,-0.4121848,0.0271720},
{-0.0042697,-0.4095078,0.0385596},
{0.0126917,-0.4134137,0.0318485},
{0.0309588,-0.4146546,0.0234111},
{0.0258681,-0.4193999,0.0069033},
{0.0160082,-0.4257241,-0.0062274},
{-0.0001385,-0.4355248,-0.0288765},
{-0.0138176,-0.4269867,-0.0366066},
{-0.0287145,-0.4177087,-0.0306219},
{0.0187237,-0.4304000,0.0200934},
{0.0014986,-0.4329870,0.0263568},
{-0.0139744,-0.4285118,0.0321063},
{-0.0008062,-0.0700636,0.0184992},
{-0.0096648,-0.0609918,0.0200609},
{0.0092127,-0.0357787,0.0600361},
{-0.0132930,-0.0145381,0.0553378},
{-0.0016295,-0.0595979,0.0556326},
{-0.0058964,-0.0669269,0.0218265},
{-0.0013206,-0.0859956,0.0199313},
{-0.0065476,0.0017767,-0.0165156},
{-0.0116728,-0.0467752,0.0399757},
{0.0030952,-0.0278817,0.0636529},
{-0.0095173,-0.0691372,0.0249649},
{-0.0009995,-0.0568230,0.0178263},

Biology Open • Supplementary information

{-0.0123421,-0.0178447,0.0493252},
{0.0127530,-0.0315567,0.0584055},
{-0.0098034,-0.0313431,0.0385506},
{0.0133865,-0.0360694,0.0567704},
{-0.0008347,-0.0784972,0.0521442},
{-0.0138820,-0.0459545,0.0303519},
{-0.0130798,-0.0582730,0.0213519},
{-0.0152314,-0.0569950,0.0225858},
{-0.0023235,-0.0433137,0.0131796},
{0.0101458,-0.0607182,0.0231946},
{0.0203262,-0.0138877,0.0384112},
{-0.0029272,-0.0461407,0.0601236},
{0.0127083,-0.0029408,0.0167124},
{-0.0083035,-0.0509771,0.0183540},
{-0.0055976,-0.0847444,0.0430478},
{0.0052355,0.0154759,0.0127315},
{-0.0106123,0.0178057,-0.0058364},
{-0.0066280,-0.0704037,0.0460779},
{0.0073627,-0.0885506,0.0188226},
{0.0121422,-0.0776785,0.0235624},
{0.0072750,-0.0493120,0.0570166},
{0.0135617,-0.0131801,0.0073590},
{-0.0054276,-0.0245760,0.0044384},
{-0.0067551,-0.0010137,0.0194661},
{-0.0094904,-0.0586949,0.0474140},
{-0.0058211,-0.0273082,0.0637136},
{0.0079061,-0.0066308,0.0581914},
{0.0127882,-0.0527138,0.0287084},
{-0.0018863,-0.0189126,-0.0116788},
{-0.0129557,-0.0255058,0.0529043},
{0.0150544,0.0138767,-0.0067969},
{-0.0038140,0.0224871,0.0035410},
{0.0005934,-0.0886810,0.0505759},
{-0.0122087,-0.0412285,0.0478970},
{0.0186655,-0.0291539,0.0406710},
{0.0108882,-0.0447520,0.0231212},
{0.0167526,0.0108318,0.0069200},
{0.0193068,0.0021693,0.0064636},
{0.0202102,-0.0020646,-0.0032716},
{0.0129461,-0.0209672,0.0215692},
{0.0107461,-0.0360914,0.0205937},
{0.0152512,-0.0153012,0.0308556},
{-0.0163093,-0.0510484,0.0253644},
{0.0028221,-0.0399056,0.0617647},
{0.0157784,-0.0470024,0.0367571},
{0.0136382,-0.0105412,0.0126947},

Biology Open • Supplementary information

{0.0009631,0.0045713,-0.0185774},
{0.0081394,-0.0007942,-0.0184361},
{0.0097486,-0.0701436,0.0513601},
{0.0139229,-0.0633414,0.0487901},
{0.0163533,-0.0615420,0.0405695},
{0.0165403,-0.0712448,0.0343091},
{-0.0044048,-0.0777329,0.0234801},
{-0.0123751,0.0038811,0.0133500},
{0.0072008,-0.0011662,0.0527180},
{0.0179163,-0.0097162,0.0480538},
{0.0139684,-0.0245575,0.0574996},
{-0.0111089,-0.0371276,0.0241959},
{-0.0171233,-0.0077754,-0.0024395},
{0.0152353,-0.0858390,0.0261527},
{-0.0065034,-0.0059565,-0.0181463},
{0.0038346,0.0146195,-0.0141376},
{-0.0087067,-0.0342662,0.0152651},
{-0.0138585,0.0106922,-0.0099330},
{-0.0188094,0.0051420,-0.0015853},
{-0.0085015,-0.0726318,0.0377290},
{0.0108876,-0.0180490,-0.0009051},
{0.0117179,-0.0470703,0.0547444},
{0.0081345,-0.0191369,-0.0085755},
{0.0094270,-0.0144123,-0.0131434},
{0.0040366,-0.0615142,0.0180780},
{0.0084048,-0.0524749,0.0204826},
{0.0011487,-0.0786677,0.0191501},
{-0.0170992,-0.0580418,0.0288094},
{-0.0056845,-0.0014070,0.0497758},
{-0.0123261,-0.0318505,0.0567765},
{0.0168795,-0.0179472,0.0516479},
{0.0141222,-0.0795978,0.0460602},
{0.0176919,-0.0248270,0.0334781},
{0.0078707,-0.0329393,0.0634993},
{-0.0160302,-0.0033608,0.0094796},
{0.0135936,-0.0166928,0.0574698},
{0.0145997,-0.0633664,0.0311347},
{-0.0143402,-0.0518687,0.0215098},
{-0.0132592,-0.0345163,0.0502450},
{0.0131107,-0.0430710,0.0291931},
{-0.0073783,-0.0400754,0.0614790},
{0.0062655,-0.0080105,-0.0180043},
{0.0039068,-0.0159613,-0.0144786},
{-0.0135981,-0.0111603,0.0491823},
{-0.0098636,-0.0463256,0.0553539},
{0.0164808,-0.0559421,0.0451633},

Biology Open • Supplementary information

{-0.0159585,0.0115581,0.0014988},
{-0.0130548,0.0094926,0.0111350},
{-0.0018004,-0.0703909,0.0523862},
{0.0125579,-0.0096141,0.0325999},
{0.0131284,-0.0036272,0.0526974},
{0.0144379,-0.0059457,0.0412969},
{-0.0120983,-0.0139299,-0.0082270},
{0.0054223,-0.0227362,0.0022510},
{0.0003493,-0.0507822,0.0163070},
{0.0189628,0.0032420,-0.0072684},
{0.0162536,0.0016821,-0.0126029},
{0.0121219,-0.0079895,-0.0151155},
{-0.0124406,0.0014217,-0.0147887},
{-0.0138914,-0.0061914,-0.0126721},
{0.0167683,0.0137061,0.0030695},
{0.0100195,0.0197631,-0.0021971},
{0.0085524,0.0143774,-0.0126649},
{0.0076423,-0.0234060,0.0617994},
{-0.0006738,-0.0089409,0.0609527},
{-0.0001572,-0.0021667,0.0549319},
{0.0051031,-0.0142126,0.0623547},
{0.0079683,0.0076145,0.0158602},
{0.0116953,-0.0180299,0.0150398},
{0.0037382,-0.0261939,0.0634059},
{-0.0086608,-0.0179037,0.0234219},
{-0.0097069,-0.0180950,0.0116921},
{-0.0061524,-0.0103629,0.0261536},
{-0.0143224,-0.0525572,0.0342145},
{-0.0107913,-0.0635359,0.0396028},
{-0.0065867,-0.0301567,0.0090534},
{0.0196877,-0.0191772,0.0446936},
{0.0116336,-0.0087732,0.0267664},
{0.0041110,-0.0009808,0.0234502},
{-0.0034088,-0.0788256,0.0509882},
{-0.0022874,0.0227456,-0.0031598},
{-0.0010872,-0.0137076,0.0378093},
{-0.0104020,-0.0148970,0.0023806},
{0.0123306,-0.0298961,0.0247815},
{0.0098252,-0.0281965,0.0165584},
{-0.0104691,-0.0072156,0.0508958},
{0.0178246,-0.0344038,0.0463600},
{-0.0114876,-0.0555988,0.0401577},
{-0.0071094,-0.0840340,0.0364067},
{0.0041233,-0.0054521,0.0298169},
{-0.0028438,-0.0047854,-0.0192955},
{-0.0120029,-0.0361063,0.0563504},

Biology Open • Supplementary information

{-0.0028643,0.0092495,-0.0163119},
{-0.0041129,0.0172447,-0.0120463},
{0.0177123,-0.0892024,0.0350986},
{0.0035146,-0.0769270,0.0183940},
{0.0156677,-0.0920358,0.0431475},
{0.0091254,0.0199544,0.0052002},
{0.0111779,0.0095126,0.0140865},
{0.0010457,0.0156732,-0.0138238},
{0.0153016,-0.0121542,-0.0085503},
{0.0180200,-0.0079500,-0.0073324},
{-0.0082797,-0.0253340,0.0294045},
{-0.0103770,-0.0360256,0.0310818},
{-0.0127548,-0.0643466,0.0248637},
{-0.0013473,0.0027760,0.0194192},
{0.0039527,0.0076383,0.0167534},
{-0.0023938,-0.0029760,0.0248086},
{0.0002443,-0.0321530,0.0629250},
{0.0065647,-0.0453257,0.0579106},
{-0.0111179,-0.0207245,0.0606634},
{-0.0110922,-0.0274837,0.0599195},
{0.0085881,0.0211477,0.0025601},
{0.0162452,0.0147715,0.0013584},
{-0.0067685,-0.0113962,0.0617639},
{0.0144620,-0.0555081,0.0332307},
{-0.0117863,-0.0496679,0.0464545},
{-0.0142553,-0.0526724,0.0212788},
{0.0104063,-0.0880431,0.0489254},
{-0.0093036,-0.0263609,0.0171187},
{-0.0095896,-0.0160526,0.0164597},
{-0.0042019,0.0167037,0.0122153},
{0.0008490,-0.0711739,0.0540885},
{0.0055887,-0.0656190,0.0542547},
{-0.0021433,-0.0652937,0.0537665},
{0.0157280,-0.0769416,0.0300315},
{-0.0118199,-0.0191031,0.0473272},
{0.0101245,-0.0259556,0.0166633},
{0.0048904,-0.0548167,0.0566416},
{0.0078795,-0.0366348,0.0602204},
{0.0090509,-0.0879922,0.0191883},
{-0.0064310,-0.0552132,0.0549952},
{0.0170073,-0.0446732,0.0425054},
{-0.0113073,-0.0446549,0.0204437},
{0.0201001,-0.0215090,0.0361597},
{-0.0038219,-0.0635722,0.0196114},
{0.0157917,-0.0020760,0.0123358},
{0.0058547,0.0056326,0.0168977},

{0.0008687,-0.0338031,0.0628112},
{-0.0140595,0.0142966,-0.0049006},
{0.0100609,0.0163303,0.0098135},
{-0.0101213,0.0166620,0.0078743},
{-0.0176498,-0.0057182,-0.0049859},
{-0.0177550,0.0005289,-0.0071521},
{-0.0046483,-0.0181927,0.0640849},
{-0.0124716,-0.0356978,0.0420182},
{0.0052990,-0.0487094,0.0578833},
{0.0117940,-0.0432599,0.0557172},
{-0.0082908,-0.0709620,0.0245052},
{0.0119810,0.0073633,0.0145064},
{0.0172935,-0.0111088,-0.0010829},
{-0.0164318,-0.0083465,0.0048270},
{-0.0121172,-0.0583874,0.0372345},
{-0.0069363,-0.0174757,-0.0035835},
{-0.0122697,-0.0285778,0.0450380},
{0.0044134,-0.0776432,0.0525935},
{0.0151518,-0.0517550,0.0495824},
{-0.0016459,-0.0361321,0.0105430},
{0.0059260,-0.0331806,0.0125668},
{0.0178074,-0.0072234,0.0055262},
{0.0171944,-0.0843958,0.0398683},
{-0.0086631,-0.0196167,0.0240026},
{-0.0008336,0.0134253,0.0153615},
{0.0070233,-0.0707158,0.0202323},
{0.0083418,-0.0646213,0.0536390},
{0.0142483,-0.0134306,-0.0090045},
{0.0005274,-0.0094284,0.0614070},
{-0.0067827,-0.0152738,-0.0135008},
{0.0150777,-0.0730064,0.0447861},
{0.0072929,-0.0190703,-0.0098542},
{-0.0100685,-0.0667516,0.0221236},
{0.0126266,-0.0407090,0.0276459},
{0.0147177,-0.0044283,0.0513124},
{0.0044997,-0.0079072,0.0374031},
{-0.0028797,-0.0639258,0.0542974},
{-0.0121436,-0.0616149,0.0362605},
{-0.0137089,-0.0118224,0.0513466},
{-0.0074542,0.0126941,-0.0139205},
{-0.0162835,0.0059859,0.0077432},
{0.0088656,-0.0704379,0.0210929},
{0.0023652,-0.0402116,0.0608977},
{-0.0128060,-0.0224148,0.0522331},
{0.0061072,-0.0063397,-0.0186705},
{0.0146093,-0.0291952,0.0536915},

{0.0113454,-0.0154695,0.0589163},
{0.0098814,0.0021361,-0.0171970},
{0.0129988,-0.0083612,0.0163314},
{-0.0122711,0.0114018,0.0111073},
{0.0098233,-0.0540960,0.0546906},
{-0.0051831,-0.0392844,0.0622617},
{0.0184368,-0.0297259,0.0475508},
{0.0150270,-0.0135308,-0.0066604},
{-0.0141493,-0.0513287,0.0346907},
{-0.0071607,-0.0753662,0.0430094},
{0.0030339,-0.0537844,0.0573440},
{-0.0041859,-0.0423204,0.0133368},
{0.0105600,0.0109218,-0.0139047},
{-0.0020323,-0.0220721,-0.0008034},
{-0.0086928,-0.0748641,0.0287653},
{-0.0097939,-0.0035761,-0.0167225},
{-0.0027652,0.0221278,-0.0044485},
{-0.0090914,-0.0221683,0.0126277},
{-0.0101854,-0.0657640,0.0219547},
{-0.0056392,-0.0762738,0.0236372},
{0.0009038,0.0120999,-0.0159073},
{0.0188192,-0.0265200,0.0468194},
{-0.0055908,-0.0591851,0.0189674},
{0.0167205,-0.0370454,0.0378381},
{0.0166963,-0.0314024,0.0337116},
{-0.0136192,-0.0122006,-0.0009343},
{0.0125875,0.0142953,0.0096749},
{-0.0126980,-0.0111272,0.0066450},
{-0.0101476,-0.0358894,0.0343018},
{0.0170355,-0.0894538,0.0407177},
{0.0167732,-0.0105331,0.0534497},
{0.0178034,-0.0158597,0.0495172},
{0.0012920,-0.0269842,0.0048200},
{0.0102391,-0.0237953,0.0603023},
{-0.0026470,-0.0097168,0.0301373},
{-0.0182938,-0.0043216,-0.0016686},
{0.0026170,-0.0213946,-0.0036566},
{-0.0091901,-0.0724027,0.0354698},
{-0.0120882,-0.0279670,0.0584715},
{0.0169783,0.0052803,-0.0103295},
{-0.0140165,-0.0633449,0.0289120},
{-0.0144548,-0.0042715,0.0122770},
{0.0113823,-0.0081745,0.0259614},
{-0.0127451,-0.0435743,0.0307505},
{0.0142796,-0.0727714,0.0276256},
{0.0007301,0.0034256,-0.0188199},

{0.0160201,-0.0787802,0.0301427},
{0.0065877,0.0064859,0.0161909},
{-0.0135560,0.0022967,0.0124315},
{0.0055517,-0.0436167,0.0159366},
{-0.0061447,-0.0193327,0.0326623},
{0.0195018,-0.0273892,0.0412577},
{-0.0122475,-0.0635951,0.0220758},
{0.0197916,0.0051952,-0.0029239},
{-0.0131685,-0.0637636,0.0268987},
{0.0106910,-0.0227228,0.0107238},
{0.0187336,-0.0118767,0.0440138},
{0.0082214,0.0019760,-0.0181062},
{0.0140032,-0.0084344,0.0131252},
{-0.0107651,-0.0645418,0.0389780},
{0.0004398,0.0209104,0.0089366},
{0.0026979,0.0033544,-0.0191283},
{0.0358316,-0.4093113,0.0204407},
{0.0048560,0.0207198,-0.0075735},
{-0.0129749,-0.0456861,0.0363015},
{-0.0301531,-0.4051661,-0.0261921},
{0.0022257,0.0234407,-0.0000311},
{0.0138046,-0.0249048,0.0258859},
{-0.0135597,-0.0011167,-0.0141459},
{0.0159009,-0.0314486,0.0515995},
{-0.0140759,0.0136495,0.0046176},
{0.0165773,-0.0406842,0.0389945},
{0.0147947,-0.0453872,0.0334473},
{-0.0127487,-0.0358005,0.0445752},
{0.0166461,-0.0574868,0.0429004},
{-0.0160676,-0.0499553,0.0267723},
{0.0029357,-0.0570726,0.0566141},
{0.0010447,-0.0532410,0.0169076},
{0.0205671,-0.0216939,0.0428867},
{0.0075935,-0.0792751,0.0513187},
{-0.0057131,-0.0860662,0.0272021},
{-0.0182276,-0.0022336,0.0029734},
{0.0138163,-0.0139058,0.0192220},
{0.0132884,-0.0690973,0.0267461},
{-0.0089206,-0.0256161,0.0267871},
{-0.0187146,-0.4154108,0.0347562},
{-0.0083043,-0.0578517,0.0507901},
{-0.0109884,-0.4097946,-0.0100056},
{0.0102413,-0.0448518,0.0218010},
{0.0104085,0.0150542,-0.0108947},
{-0.0233673,-0.4064132,0.0281228},
{0.0150018,-0.0551008,0.0340882},

Biology Open • Supplementary information

{0.0002861,-0.0513804,0.0582573},
{-0.0105306,0.0187595,0.0027682},
{-0.0023656,-0.0173131,0.0639490},
{-0.0069936,-0.0076406,0.0234694},
{0.0185781,-0.0106847,0.0410797},
{0.0142273,-0.0253938,0.0556540},
{-0.0135473,-0.0113087,0.0506343},
{0.0189881,0.0085394,-0.0027311},
{-0.0096044,-0.0353752,0.0174421},
{-0.0003227,-0.0146434,-0.0159932},
{-0.0103232,-0.0624488,0.0429687},
{-0.0086265,0.0177551,0.0078016},
{0.0093513,0.0198473,-0.0029195},
{-0.0128323,-0.0280341,0.0554505},
{-0.0057291,-0.4069195,0.0393962},
{-0.0128655,-0.4124520,-0.0369248},
{-0.0127942,-0.4123967,-0.0361615},
{-0.0127228,-0.4123413,-0.0353983},
{-0.0126514,-0.4122860,-0.0346351},
{-0.0125801,-0.4122307,-0.0338719},
{-0.0125087,-0.4121754,-0.0331087},
{-0.0124374,-0.4121200,-0.0323455},
{-0.0123660,-0.4120647,-0.0315823},
{-0.0122946,-0.4120094,-0.0308191},
{-0.0122233,-0.4119541,-0.0300559},
{-0.0121519,-0.4118987,-0.0292927},
{-0.0120805,-0.4118434,-0.0285295},
{-0.0120092,-0.4117881,-0.0277662},
{-0.0119378,-0.4117328,-0.0270030},
{-0.0118664,-0.4116774,-0.0262398},
{-0.0117951,-0.4116221,-0.0254766},
{-0.0117237,-0.4115668,-0.0247134},
{-0.0116524,-0.4115115,-0.0239502},
{-0.0115810,-0.4114561,-0.0231870},
{-0.0115096,-0.4114008,-0.0224238},
{-0.0114383,-0.4113455,-0.0216606},
{-0.0113669,-0.4112902,-0.0208974},
{-0.0112955,-0.4112348,-0.0201342},
{-0.0112242,-0.4111795,-0.0193709},
{-0.0111528,-0.4111242,-0.0186077},
{-0.0110814,-0.4110689,-0.0178445},
{-0.0110101,-0.4110135,-0.0170813},
{-0.0109387,-0.4109582,-0.0163181},
{-0.0108673,-0.4109029,-0.0155549},
{-0.0107960,-0.4108476,-0.0147917},
{-0.0107246,-0.4107922,-0.0140285},

Biology Open • Supplementary information

{-0.0106533,-0.4107369,-0.0132653},
{-0.0105819,-0.4106816,-0.0125021},
{-0.0105105,-0.4106263,-0.0117388},
{-0.0104392,-0.4105709,-0.0109756},
{-0.0103678,-0.4105156,-0.0102124},
{-0.0102964,-0.4104603,-0.0094492},
{-0.0102251,-0.4104050,-0.0086860},
{-0.0101537,-0.4103497,-0.0079228},
{-0.0100823,-0.4102943,-0.0071596},
{-0.0100110,-0.4102390,-0.0063964},
{-0.0099396,-0.4101837,-0.0056332},
{-0.0098682,-0.4101284,-0.0048700},
{-0.0097969,-0.4100730,-0.0041068},
{-0.0097255,-0.4100177,-0.0033435},
{-0.0096542,-0.4099624,-0.0025803},
{-0.0095828,-0.4099071,-0.0018171},
{-0.0095114,-0.4098517,-0.0010539},
{-0.0094401,-0.4097964,-0.0002907},
{-0.0093687,-0.4097411,0.0004725},
{-0.0092973,-0.4096858,0.0012357},
{-0.0092260,-0.4096304,0.0019989},
{-0.0091546,-0.4095751,0.0027621},
{-0.0090832,-0.4095198,0.0035253},
{-0.0090119,-0.4094645,0.0042886},
{-0.0089405,-0.4094091,0.0050518},
{-0.0088691,-0.4093538,0.0058150},
{-0.0087978,-0.4092985,0.0065782},
{-0.0087264,-0.4092432,0.0073414},
{-0.0086551,-0.4091878,0.0081046},
{-0.0085837,-0.4091325,0.0088678},
{-0.0085123,-0.4090772,0.0096310},
{-0.0084410,-0.4090219,0.0103942},
{-0.0083696,-0.4089665,0.0111574},
{-0.0082982,-0.4089112,0.0119206},
{-0.0082269,-0.4088559,0.0126839},
{-0.0081555,-0.4088006,0.0134471},
{-0.0080841,-0.4087452,0.0142103},
{-0.0080128,-0.4086899,0.0149735},
{-0.0079414,-0.4086346,0.0157367},
{-0.0078701,-0.4085793,0.0164999},
{-0.0077987,-0.4085239,0.0172631},
{-0.0077273,-0.4084686,0.0180263},
{-0.0076560,-0.4084133,0.0187895},
{-0.0075846,-0.4083580,0.0195527},
{-0.0075132,-0.4083026,0.0203159},
{-0.0074419,-0.4082473,0.0210792},

Biology Open • Supplementary information

```
                    {-0.0073705,-0.4081920,0.0218424},
                    {-0.0072991,-0.4081367,0.0226056},
                    {-0.0072278,-0.4080813,0.0233688},
                    {-0.0071564,-0.4080260,0.0241320},
                    {-0.0070850,-0.4079707,0.0248952},
                    {-0.0070137,-0.4079154,0.0256584},
                    {-0.0069423,-0.4078600,0.0264216},
                    {-0.0068710,-0.4078047,0.0271848},
                    {-0.0067996,-0.4077494,0.0279480},
                    {-0.0067282,-0.4076941,0.0287113},
                    {-0.0066569,-0.4076387,0.0294745},
                    {-0.0065855,-0.4075834,0.0302377},
                    {-0.0065141,-0.4075281,0.0310009},
                    {-0.0064428,-0.4074728,0.0317641},
                    {-0.0063714,-0.4074174,0.0325273},
                    {-0.0063000,-0.4073621,0.0332905},
                    {-0.0062287,-0.4073068,0.0340537},
                    {-0.0061573,-0.4072515,0.0348169},
                    {-0.0060859,-0.4071962,0.0355801},
                    {-0.0060146,-0.4071408,0.0363433},
                    {-0.0059432,-0.4070855,0.0371066},
                    {-0.0058719,-0.4070302,0.0378698},
                    {-0.0058005,-0.4069749,0.0386330}
                    };
            BoundingBoxOnOff = Off;
        };
        AnyFunTransform3DIdentity ScaleFunction = {
            PreTransforms = {&.RBFTransform};
};
};

};
};
```

Biology Open • Supplementary information

ScalingFunctionTLEMLucyPelvis_2014013

```
AnyFolder ScalingFunctionTLEMLucyPelvis = {
AnyFolder Pelvis = {
	AnyFunTransform3DRBF RBFTransform = {
		RBFDef.Type = RBF_ThinPlate;
		PolynomDegree = 1;
		Points0 = {
			{0.0000000,0.0000000,0.1177000},
			{-0.0000000,-0.0832729,0.0191000},
			{-0.0508179,-0.0694062,0.0815920},
			{0.0000000,0.0000000,-0.1177000},
			{-0.0000000,-0.0832729,-0.0191000},
			{-0.0508179,-0.0694062,-0.0815920},
			{0.0000000,0.0000000,0.0000000},
			{-0.1164020,-0.0039449,0.0456760},
			{-0.1164020,-0.0039449,-0.0456760},
			{-0.1092909,-0.0992949,0.0515590},
			{-0.1092909,-0.0992949,-0.0515590},
			{-0.0733749,0.0757445,0.0915590},
			{-0.0733749,0.0757445,-0.0915590},
			{-0.1241738,0.0316243,0.0458050},
			{-0.1241738,0.0316243,-0.0458050},
			{-0.1108008,0.0563490,0.0555690},
			{-0.1108008,0.0563490,-0.0555690},
			{-0.0545356,0.0481653,0.1291690},
			{-0.0545356,0.0481653,-0.1291690},
			{-0.0360236,0.0456352,0.1316250},
			{-0.0360236,0.0456352,-0.1316250},
			{-0.0804664,-0.0228481,0.0672140},
			{-0.0804664,-0.0228481,-0.0672140},
			{-0.1004070,-0.0623213,0.0494990},
			{-0.1004070,-0.0623213,-0.0494990},
			{-0.0174901,-0.0357786,0.0977400},
			{-0.0174901,-0.0357786,-0.0977400},
			{-0.0665950,-0.1308418,0.0278190},
			{-0.0665950,-0.1308418,-0.0278190},
			{-0.0962739,-0.1257274,0.0551670},
			{-0.0962739,-0.1257274,-0.0551670},
			{-0.0393340,-0.1196098,0.0159820},
			{-0.0393340,-0.1196098,-0.0159820},
			{-0.0585979,-0.0148191,0.0651020},
			{-0.0585979,-0.0148191,-0.0651020},
			{-0.0238420,-0.0800479,0.0351680},
			{-0.0238420,-0.0800479,-0.0351680},
			{-0.0234553,-0.0576924,0.0715330},
```

Biology Open • Supplementary information

{-0.0234553,-0.0576924,-0.0715330},
{-0.0472319,-0.0833276,0.0513790},
{-0.0472319,-0.0833276,-0.0513790},
{-0.0327175,-0.0506481,0.0520240},
{-0.0327175,-0.0506481,-0.0520240},
{-0.0746024,-0.0627219,0.0928110},
{-0.0746024,-0.0627219,-0.0928110},
{-0.0119676,-0.0928080,0.0076900},
{-0.0119676,-0.0928080,-0.0076900},
{-0.0263299,-0.0760728,0.0667080},
{-0.0263299,-0.0760728,-0.0667080},
{-0.0498842,0.0119450,0.1070630},
{-0.0498842,0.0119450,-0.1070630},
{-0.0553874,0.0627474,0.1172390},
{-0.0553874,0.0627474,-0.1172390},
{-0.0786774,0.0073556,0.0741800},
{-0.0786774,0.0073556,-0.0741800},
{-0.0742762,0.0474272,0.0542330},
{-0.0742762,0.0474272,-0.0542330},
{-0.0634121,0.0117185,0.0618430},
{-0.0634121,0.0117185,-0.0618430},
{-0.0813084,0.0007821,0.0508130},
{-0.0813084,0.0007821,-0.0508130},
{-0.0496423,-0.0444458,0.0983920},
{-0.0496423,-0.0444458,-0.0983920},
{-0.0480005,-0.0338999,0.0625080},
{-0.0480005,-0.0338999,-0.0625080},
{-0.0428000,-0.0273188,0.0973490},
{-0.0428000,-0.0273188,-0.0973490},
{-0.0548383,-0.0518766,0.0624050},
{-0.0548383,-0.0518766,-0.0624050},
{-0.0696448,-0.1052827,0.0436200},
{-0.0696448,-0.1052827,-0.0436200},
{-0.0739608,-0.0803177,0.0785950},
{-0.0739608,-0.0803177,-0.0785950},
{-0.0586066,-0.0884764,0.0653190},
{-0.0586066,-0.0884764,-0.0653190},
{-0.1017003,0.0085215,0.0624150},
{-0.1017003,0.0085215,-0.0624150},
{-0.0126252,0.0252852,0.1292530},
{-0.0126252,0.0252852,-0.1292530},
{-0.0030963,0.0150156,0.1262640},
{-0.0030963,0.0150156,-0.1262640},
{-0.0621797,0.0048743,0.0605920},
{-0.0621797,0.0048743,-0.0605920},
{-0.0870082,-0.0684280,0.0489900},

Biology Open • Supplementary information

{-0.0870082,-0.0684280,-0.0489900},
{-0.0916211,-0.0478344,0.0575290},
{-0.0916211,-0.0478344,-0.0575290},
{-0.0428573,-0.0467026,0.0985250},
{-0.0428573,-0.0467026,-0.0985250},
{-0.0483869,-0.0455474,0.0848580},
{-0.0483869,-0.0455474,-0.0848580},
{-0.0203929,-0.0681811,0.0426070},
{-0.0203929,-0.0681811,-0.0426070},
{-0.0271973,-0.0807786,0.0491650},
{-0.0271973,-0.0807786,-0.0491650},
{-0.0325935,-0.1026813,0.0148280},
{-0.0325935,-0.1026813,-0.0148280},
{-0.0273900,-0.1059173,0.0048750},
{-0.0273900,-0.1059173,-0.0048750},
{-0.0015971,-0.0778770,0.0065530},
{-0.0015971,-0.0778770,-0.0065530},
{-0.1034983,-0.0140026,0.0521800},
{-0.1034983,-0.0140026,-0.0521800},
{-0.1141160,0.0077734,0.0497600},
{-0.1141160,0.0077734,-0.0497600},
{-0.1123797,0.0244808,0.0350160},
{-0.1123797,0.0244808,-0.0350160},
{-0.0984470,-0.0648210,0.0457100},
{-0.0984470,-0.0648210,-0.0457100},
{-0.0814332,-0.0704479,0.0806410},
{-0.0814332,-0.0704479,-0.0806410},
{-0.0540218,-0.0805070,0.0547320},
{-0.0540218,-0.0805070,-0.0547320},
{-0.0674356,-0.0763171,0.0651080},
{-0.0674356,-0.0763171,-0.0651080},
{-0.0602364,-0.0751827,0.0490600},
{-0.0602364,-0.0751827,-0.0490600},
{-0.0604272,-0.0801096,0.0617610},
{-0.0604272,-0.0801096,-0.0617610},
{-0.0655251,0.0632155,0.1093750},
{-0.0655251,0.0632155,-0.1093750},
{-0.0665046,0.0014289,0.0572170},
{-0.0665046,0.0014289,-0.0572170},
{-0.1053786,0.0347803,0.0071580},
{-0.1053786,0.0347803,-0.0071580},
{-0.1321649,-0.0069725,0.0058340},
{-0.1321649,-0.0069725,-0.0058340},
{-0.1232735,-0.0434903,0.0301110},
{-0.1232735,-0.0434903,-0.0301110},
{-0.0497024,0.0261862,0.0084640},

{-0.0497024,0.0261862,-0.0084640},
{-0.0887827,0.0446048,0.0327030},
{-0.0887827,0.0446048,-0.0327030},
{-0.1153998,-0.0126010,0.0290730},
{-0.1153998,-0.0126010,-0.0290730},
{-0.0899939,0.0093882,0.0208440},
{-0.0899939,0.0093882,-0.0208440},
{-0.1312654,-0.0574898,0.0094690},
{-0.1312654,-0.0574898,-0.0094690},
{-0.0996029,0.0042806,0.0105620},
{-0.0996029,0.0042806,-0.0105620},
{-0.1226177,-0.0324069,0.0331630},
{-0.1226177,-0.0324069,-0.0331630},
{-0.1097808,-0.0051619,0.0102520},
{-0.1097808,-0.0051619,-0.0102520}
};
Points1 = {
{0.0000000,0.0000000,0.1097492},
{-0.0000006,-0.0805613,0.0159809},
{-0.0275205,-0.0625580,0.0763734},
{0.0000000,0.0000000,-0.1097492},
{-0.0000006,-0.0805613,-0.0159809},
{-0.0275205,-0.0625580,-0.0763734},
{0.0000000,0.0000000,0.0000000},
{-0.0751438,-0.0263084,0.0371758},
{-0.0751438,-0.0263084,-0.0371758},
{-0.0712246,-0.0884765,0.0542792},
{-0.0712246,-0.0884765,-0.0542792},
{-0.0488982,0.0370006,0.0800438},
{-0.0488982,0.0370006,-0.0800438},
{-0.0793299,-0.0065910,0.0329602},
{-0.0793299,-0.0065910,-0.0329602},
{-0.0720126,0.0138591,0.0437634},
{-0.0720126,0.0138591,-0.0437634},
{-0.0301876,0.0283998,0.1136434},
{-0.0301876,0.0283998,-0.1136434},
{-0.0196727,0.0286866,0.1173741},
{-0.0196727,0.0286866,-0.1173741},
{-0.0531818,-0.0356966,0.0602587},
{-0.0531818,-0.0356966,-0.0602587},
{-0.0664456,-0.0652543,0.0557422},
{-0.0664456,-0.0652543,-0.0557422},
{-0.0099019,-0.0349782,0.0823959},
{-0.0099019,-0.0349782,-0.0823959},
{-0.0460213,-0.1114220,0.0299560},
{-0.0460213,-0.1114220,-0.0299560},

Biology Open • Supplementary information

{-0.0622570,-0.1032898,0.0570830},
{-0.0622570,-0.1032898,-0.0570830},
{-0.0258920,-0.1067782,0.0167127},
{-0.0258920,-0.1067782,-0.0167127},
{-0.0413806,-0.0254405,0.0582245},
{-0.0413806,-0.0254405,-0.0582245},
{-0.0131565,-0.0734553,0.0301868},
{-0.0131565,-0.0734553,-0.0301868},
{-0.0138452,-0.0522131,0.0647187},
{-0.0138452,-0.0522131,-0.0647187},
{-0.0257049,-0.0711031,0.0496567},
{-0.0257049,-0.0711031,-0.0496567},
{-0.0215182,-0.0524152,0.0503653},
{-0.0215182,-0.0524152,-0.0503653},
{-0.0436326,-0.0586769,0.0803276},
{-0.0436326,-0.0586769,-0.0803276},
{-0.0062945,-0.0893747,0.0068615},
{-0.0062945,-0.0893747,-0.0068615},
{-0.0147121,-0.0629146,0.0589619},
{-0.0147121,-0.0629146,-0.0589619},
{-0.0318694,-0.0008837,0.0934721},
{-0.0318694,-0.0008837,-0.0934721},
{-0.0340154,0.0353180,0.1036826},
{-0.0340154,0.0353180,-0.1036826},
{-0.0540325,-0.0126847,0.0610688},
{-0.0540325,-0.0126847,-0.0610688},
{-0.0517458,0.0124604,0.0440085},
{-0.0517458,0.0124604,-0.0440085},
{-0.0469840,-0.0091072,0.0495166},
{-0.0469840,-0.0091072,-0.0495166},
{-0.0565596,-0.0205324,0.0411122},
{-0.0565596,-0.0205324,-0.0411122},
{-0.0273928,-0.0449500,0.0830964},
{-0.0273928,-0.0449500,-0.0830964},
{-0.0315623,-0.0386896,0.0593840},
{-0.0315623,-0.0386896,-0.0593840},
{-0.0254835,-0.0310697,0.0834215},
{-0.0254835,-0.0310697,-0.0834215},
{-0.0326078,-0.0520144,0.0608479},
{-0.0326078,-0.0520144,-0.0608479},
{-0.0440850,-0.0912740,0.0466471},
{-0.0440850,-0.0912740,-0.0466471},
{-0.0429387,-0.0717403,0.0730665},
{-0.0429387,-0.0717403,-0.0730665},
{-0.0327393,-0.0780466,0.0651399},
{-0.0327393,-0.0780466,-0.0651399},

{-0.0669515,-0.0153526,0.0506383},
{-0.0669515,-0.0153526,-0.0506383},
{-0.0065508,0.0169072,0.1176731},
{-0.0065508,0.0169072,-0.1176731},
{-0.0011771,0.0106602,0.1162254},
{-0.0011771,0.0106602,-0.1162254},
{-0.0467530,-0.0136596,0.0483032},
{-0.0467530,-0.0136596,-0.0483032},
{-0.0558929,-0.0676314,0.0553713},
{-0.0558929,-0.0676314,-0.0553713},
{-0.0593104,-0.0541056,0.0578967},
{-0.0593104,-0.0541056,-0.0578967},
{-0.0226046,-0.0466222,0.0826950},
{-0.0226046,-0.0466222,-0.0826950},
{-0.0276762,-0.0459527,0.0743175},
{-0.0276762,-0.0459527,-0.0743175},
{-0.0116794,-0.0650464,0.0365741},
{-0.0116794,-0.0650464,-0.0365741},
{-0.0148749,-0.0709789,0.0433350},
{-0.0148749,-0.0709789,-0.0433350},
{-0.0186151,-0.0944211,0.0152263},
{-0.0186151,-0.0944211,-0.0152263},
{-0.0138182,-0.0973961,0.0059481},
{-0.0138182,-0.0973961,-0.0059481},
{-0.0015996,-0.0794215,0.0055197},
{-0.0015996,-0.0794215,-0.0055197},
{-0.0672046,-0.0314788,0.0462008},
{-0.0672046,-0.0314788,-0.0462008},
{-0.0739834,-0.0173547,0.0391678},
{-0.0739834,-0.0173547,-0.0391678},
{-0.0726343,-0.0104260,0.0251826},
{-0.0726343,-0.0104260,-0.0251826},
{-0.0655187,-0.0669368,0.0545657},
{-0.0655187,-0.0669368,-0.0545657},
{-0.0486372,-0.0655564,0.0743660},
{-0.0486372,-0.0655564,-0.0743660},
{-0.0291055,-0.0683849,0.0539226},
{-0.0291055,-0.0683849,-0.0539226},
{-0.0386815,-0.0699299,0.0650384},
{-0.0386815,-0.0699299,-0.0650384},
{-0.0345956,-0.0680912,0.0508260},
{-0.0345956,-0.0680912,-0.0508260},
{-0.0335136,-0.0723389,0.0627383},
{-0.0335136,-0.0723389,-0.0627383},
{-0.0421291,0.0323153,0.0963164},
{-0.0421291,0.0323153,-0.0963164},

Biology Open • Supplementary information

```
				{-0.0497149,-0.0170924,0.0448689},
				{-0.0497149,-0.0170924,-0.0448689},
				{-0.0687601,-0.0054038,0.0050608},
				{-0.0687601,-0.0054038,-0.0050608},
				{-0.0840367,-0.0341151,0.0040271},
				{-0.0840367,-0.0341151,-0.0040271},
				{-0.0832060,-0.0590624,0.0189049},
				{-0.0832060,-0.0590624,-0.0189049},
				{-0.0375090,-0.0061744,0.0068785},
				{-0.0375090,-0.0061744,-0.0068785},
				{-0.0601617,0.0055440,0.0249240},
				{-0.0601617,0.0055440,-0.0249240},
				{-0.0745313,-0.0355116,0.0227865},
				{-0.0745313,-0.0355116,-0.0227865},
				{-0.0600944,-0.0205359,0.0161298},
				{-0.0600944,-0.0205359,-0.0161298},
				{-0.0886916,-0.0691077,0.0059086},
				{-0.0886916,-0.0691077,-0.0059086},
				{-0.0639921,-0.0263699,0.0080936},
				{-0.0639921,-0.0263699,-0.0080936},
				{-0.0812733,-0.0502575,0.0240681},
				{-0.0812733,-0.0502575,-0.0240681},
				{-0.0691103,-0.0334595,0.0077551},
				{-0.0691103,-0.0334595,-0.0077551}
				};
			BoundingBoxOnOff = Off;
		};
		AnyFunTransform3DIdentity ScaleFunction = {
			PreTransforms = {&.RBFTransform};
};
};
AnyFolder Sacrum = {
		AnyFunTransform3DRBF RBFTransform = {
			RBFDef.Type = RBF_ThinPlate;
			PolynomDegree = 1;
			Points0 = {
				{0.0000000,0.0000000,0.1177000},
				{-0.0000000,-0.0832729,0.0191000},
				{-0.0508179,-0.0694062,0.0815920},
				{0.0000000,0.0000000,-0.1177000},
				{-0.0000000,-0.0832729,-0.0191000},
				{-0.0508179,-0.0694062,-0.0815920},
				{0.0000000,0.0000000,0.0000000},
				{-0.1164020,-0.0039449,0.0456760},
				{-0.1164020,-0.0039449,-0.0456760},
				{-0.1092909,-0.0992949,0.0515590},
```

Biology Open • Supplementary information

{-0.1092909,-0.0992949,-0.0515590},
{-0.0733749,0.0757445,0.0915590},
{-0.0733749,0.0757445,-0.0915590},
{-0.1241738,0.0316243,0.0458050},
{-0.1241738,0.0316243,-0.0458050},
{-0.1108008,0.0563490,0.0555690},
{-0.1108008,0.0563490,-0.0555690},
{-0.0545356,0.0481653,0.1291690},
{-0.0545356,0.0481653,-0.1291690},
{-0.0360236,0.0456352,0.1316250},
{-0.0360236,0.0456352,-0.1316250},
{-0.0804664,-0.0228481,0.0672140},
{-0.0804664,-0.0228481,-0.0672140},
{-0.1004070,-0.0623213,0.0494990},
{-0.1004070,-0.0623213,-0.0494990},
{-0.0174901,-0.0357786,0.0977400},
{-0.0174901,-0.0357786,-0.0977400},
{-0.0665950,-0.1308418,0.0278190},
{-0.0665950,-0.1308418,-0.0278190},
{-0.0962739,-0.1257274,0.0551670},
{-0.0962739,-0.1257274,-0.0551670},
{-0.0393340,-0.1196098,0.0159820},
{-0.0393340,-0.1196098,-0.0159820},
{-0.0585979,-0.0148191,0.0651020},
{-0.0585979,-0.0148191,-0.0651020},
{-0.0238420,-0.0800479,0.0351680},
{-0.0238420,-0.0800479,-0.0351680},
{-0.0234553,-0.0576924,0.0715330},
{-0.0234553,-0.0576924,-0.0715330},
{-0.0472319,-0.0833276,0.0513790},
{-0.0472319,-0.0833276,-0.0513790},
{-0.0327175,-0.0506481,0.0520240},
{-0.0327175,-0.0506481,-0.0520240},
{-0.0746024,-0.0627219,0.0928110},
{-0.0746024,-0.0627219,-0.0928110},
{-0.0119676,-0.0928080,0.0076900},
{-0.0119676,-0.0928080,-0.0076900},
{-0.0263299,-0.0760728,0.0667080},
{-0.0263299,-0.0760728,-0.0667080},
{-0.0498842,0.0119450,0.1070630},
{-0.0498842,0.0119450,-0.1070630},
{-0.0553874,0.0627474,0.1172390},
{-0.0553874,0.0627474,-0.1172390},
{-0.0786774,0.0073556,0.0741800},
{-0.0786774,0.0073556,-0.0741800},
{-0.0742762,0.0474272,0.0542330},

Biology Open • Supplementary information

{-0.0742762,0.0474272,-0.0542330},
{-0.0634121,0.0117185,0.0618430},
{-0.0634121,0.0117185,-0.0618430},
{-0.0813084,0.0007821,0.0508130},
{-0.0813084,0.0007821,-0.0508130},
{-0.0496423,-0.0444458,0.0983920},
{-0.0496423,-0.0444458,-0.0983920},
{-0.0480005,-0.0338999,0.0625080},
{-0.0480005,-0.0338999,-0.0625080},
{-0.0428000,-0.0273188,0.0973490},
{-0.0428000,-0.0273188,-0.0973490},
{-0.0548383,-0.0518766,0.0624050},
{-0.0548383,-0.0518766,-0.0624050},
{-0.0696448,-0.1052827,0.0436200},
{-0.0696448,-0.1052827,-0.0436200},
{-0.0739608,-0.0803177,0.0785950},
{-0.0739608,-0.0803177,-0.0785950},
{-0.0586066,-0.0884764,0.0653190},
{-0.0586066,-0.0884764,-0.0653190},
{-0.1017003,0.0085215,0.0624150},
{-0.1017003,0.0085215,-0.0624150},
{-0.0126252,0.0252852,0.1292530},
{-0.0126252,0.0252852,-0.1292530},
{-0.0030963,0.0150156,0.1262640},
{-0.0030963,0.0150156,-0.1262640},
{-0.0621797,0.0048743,0.0605920},
{-0.0621797,0.0048743,-0.0605920},
{-0.0870082,-0.0684280,0.0489900},
{-0.0870082,-0.0684280,-0.0489900},
{-0.0916211,-0.0478344,0.0575290},
{-0.0916211,-0.0478344,-0.0575290},
{-0.0428573,-0.0467026,0.0985250},
{-0.0428573,-0.0467026,-0.0985250},
{-0.0483869,-0.0455474,0.0848580},
{-0.0483869,-0.0455474,-0.0848580},
{-0.0203929,-0.0681811,0.0426070},
{-0.0203929,-0.0681811,-0.0426070},
{-0.0271973,-0.0807786,0.0491650},
{-0.0271973,-0.0807786,-0.0491650},
{-0.0325935,-0.1026813,0.0148280},
{-0.0325935,-0.1026813,-0.0148280},
{-0.0273900,-0.1059173,0.0048750},
{-0.0273900,-0.1059173,-0.0048750},
{-0.0015971,-0.0778770,0.0065530},
{-0.0015971,-0.0778770,-0.0065530},
{-0.1034983,-0.0140026,0.0521800},

```
        {-0.1034983,-0.0140026,-0.0521800},
        {-0.1141160,0.0077734,0.0497600},
        {-0.1141160,0.0077734,-0.0497600},
        {-0.1123797,0.0244808,0.0350160},
        {-0.1123797,0.0244808,-0.0350160},
        {-0.0984470,-0.0648210,0.0457100},
        {-0.0984470,-0.0648210,-0.0457100},
        {-0.0814332,-0.0704479,0.0806410},
        {-0.0814332,-0.0704479,-0.0806410},
        {-0.0540218,-0.0805070,0.0547320},
        {-0.0540218,-0.0805070,-0.0547320},
        {-0.0674356,-0.0763171,0.0651080},
        {-0.0674356,-0.0763171,-0.0651080},
        {-0.0602364,-0.0751827,0.0490600},
        {-0.0602364,-0.0751827,-0.0490600},
        {-0.0604272,-0.0801096,0.0617610},
        {-0.0604272,-0.0801096,-0.0617610},
        {-0.0655251,0.0632155,0.1093750},
        {-0.0655251,0.0632155,-0.1093750},
        {-0.0665046,0.0014289,0.0572170},
        {-0.0665046,0.0014289,-0.0572170},
        {-0.1053786,0.0347803,0.0071580},
        {-0.1053786,0.0347803,-0.0071580},
        {-0.1321649,-0.0069725,0.0058340},
        {-0.1321649,-0.0069725,-0.0058340},
        {-0.1232735,-0.0434903,0.0301110},
        {-0.1232735,-0.0434903,-0.0301110},
        {-0.0497024,0.0261862,0.0084640},
        {-0.0497024,0.0261862,-0.0084640},
        {-0.0887827,0.0446048,0.0327030},
        {-0.0887827,0.0446048,-0.0327030},
        {-0.1153998,-0.0126010,0.0290730},
        {-0.1153998,-0.0126010,-0.0290730},
        {-0.0899939,0.0093882,0.0208440},
        {-0.0899939,0.0093882,-0.0208440},
        {-0.1312654,-0.0574898,0.0094690},
        {-0.1312654,-0.0574898,-0.0094690},
        {-0.0996029,0.0042806,0.0105620},
        {-0.0996029,0.0042806,-0.0105620},
        {-0.1226177,-0.0324069,0.0331630},
        {-0.1226177,-0.0324069,-0.0331630},
        {-0.1097808,-0.0051619,0.0102520},
        {-0.1097808,-0.0051619,-0.0102520}
        };
Points1 = {
        {0.0000000,0.0000000,0.1097492},
```

Biology Open • Supplementary information

{-0.0000006,-0.0805613,0.0159809},
{-0.0275205,-0.0625580,0.0763734},
{0.0000000,0.0000000,-0.1097492},
{-0.0000006,-0.0805613,-0.0159809},
{-0.0275205,-0.0625580,-0.0763734},
{0.0000000,0.0000000,0.0000000},
{-0.0751438,-0.0263084,0.0371758},
{-0.0751438,-0.0263084,-0.0371758},
{-0.0712246,-0.0884765,0.0542792},
{-0.0712246,-0.0884765,-0.0542792},
{-0.0488982,0.0370006,0.0800438},
{-0.0488982,0.0370006,-0.0800438},
{-0.0793299,-0.0065910,0.0329602},
{-0.0793299,-0.0065910,-0.0329602},
{-0.0720126,0.0138591,0.0437634},
{-0.0720126,0.0138591,-0.0437634},
{-0.0301876,0.0283998,0.1136434},
{-0.0301876,0.0283998,-0.1136434},
{-0.0196727,0.0286866,0.1173741},
{-0.0196727,0.0286866,-0.1173741},
{-0.0531818,-0.0356966,0.0602587},
{-0.0531818,-0.0356966,-0.0602587},
{-0.0664456,-0.0652543,0.0557422},
{-0.0664456,-0.0652543,-0.0557422},
{-0.0099019,-0.0349782,0.0823959},
{-0.0099019,-0.0349782,-0.0823959},
{-0.0460213,-0.1114220,0.0299560},
{-0.0460213,-0.1114220,-0.0299560},
{-0.0622570,-0.1032898,0.0570830},
{-0.0622570,-0.1032898,-0.0570830},
{-0.0258920,-0.1067782,0.0167127},
{-0.0258920,-0.1067782,-0.0167127},
{-0.0413806,-0.0254405,0.0582245},
{-0.0413806,-0.0254405,-0.0582245},
{-0.0131565,-0.0734553,0.0301868},
{-0.0131565,-0.0734553,-0.0301868},
{-0.0138452,-0.0522131,0.0647187},
{-0.0138452,-0.0522131,-0.0647187},
{-0.0257049,-0.0711031,0.0496567},
{-0.0257049,-0.0711031,-0.0496567},
{-0.0215182,-0.0524152,0.0503653},
{-0.0215182,-0.0524152,-0.0503653},
{-0.0436326,-0.0586769,0.0803276},
{-0.0436326,-0.0586769,-0.0803276},
{-0.0062945,-0.0893747,0.0068615},
{-0.0062945,-0.0893747,-0.0068615},

Biology Open • Supplementary information

{-0.0147121,-0.0629146,0.0589619},
{-0.0147121,-0.0629146,-0.0589619},
{-0.0318694,-0.0008837,0.0934721},
{-0.0318694,-0.0008837,-0.0934721},
{-0.0340154,0.0353180,0.1036826},
{-0.0340154,0.0353180,-0.1036826},
{-0.0540325,-0.0126847,0.0610688},
{-0.0540325,-0.0126847,-0.0610688},
{-0.0517458,0.0124604,0.0440085},
{-0.0517458,0.0124604,-0.0440085},
{-0.0469840,-0.0091072,0.0495166},
{-0.0469840,-0.0091072,-0.0495166},
{-0.0565596,-0.0205324,0.0411122},
{-0.0565596,-0.0205324,-0.0411122},
{-0.0273928,-0.0449500,0.0830964},
{-0.0273928,-0.0449500,-0.0830964},
{-0.0315623,-0.0386896,0.0593840},
{-0.0315623,-0.0386896,-0.0593840},
{-0.0254835,-0.0310697,0.0834215},
{-0.0254835,-0.0310697,-0.0834215},
{-0.0326078,-0.0520144,0.0608479},
{-0.0326078,-0.0520144,-0.0608479},
{-0.0440850,-0.0912740,0.0466471},
{-0.0440850,-0.0912740,-0.0466471},
{-0.0429387,-0.0717403,0.0730665},
{-0.0429387,-0.0717403,-0.0730665},
{-0.0327393,-0.0780466,0.0651399},
{-0.0327393,-0.0780466,-0.0651399},
{-0.0669515,-0.0153526,0.0506383},
{-0.0669515,-0.0153526,-0.0506383},
{-0.0065508,0.0169072,0.1176731},
{-0.0065508,0.0169072,-0.1176731},
{-0.0011771,0.0106602,0.1162254},
{-0.0011771,0.0106602,-0.1162254},
{-0.0467530,-0.0136596,0.0483032},
{-0.0467530,-0.0136596,-0.0483032},
{-0.0558929,-0.0676314,0.0553713},
{-0.0558929,-0.0676314,-0.0553713},
{-0.0593104,-0.0541056,0.0578967},
{-0.0593104,-0.0541056,-0.0578967},
{-0.0226046,-0.0466222,0.0826950},
{-0.0226046,-0.0466222,-0.0826950},
{-0.0276762,-0.0459527,0.0743175},
{-0.0276762,-0.0459527,-0.0743175},
{-0.0116794,-0.0650464,0.0365741},
{-0.0116794,-0.0650464,-0.0365741},

{-0.0148749,-0.0709789,0.0433350},
{-0.0148749,-0.0709789,-0.0433350},
{-0.0186151,-0.0944211,0.0152263},
{-0.0186151,-0.0944211,-0.0152263},
{-0.0138182,-0.0973961,0.0059481},
{-0.0138182,-0.0973961,-0.0059481},
{-0.0015996,-0.0794215,0.0055197},
{-0.0015996,-0.0794215,-0.0055197},
{-0.0672046,-0.0314788,0.0462008},
{-0.0672046,-0.0314788,-0.0462008},
{-0.0739834,-0.0173547,0.0391678},
{-0.0739834,-0.0173547,-0.0391678},
{-0.0726343,-0.0104260,0.0251826},
{-0.0726343,-0.0104260,-0.0251826},
{-0.0655187,-0.0669368,0.0545657},
{-0.0655187,-0.0669368,-0.0545657},
{-0.0486372,-0.0655564,0.0743660},
{-0.0486372,-0.0655564,-0.0743660},
{-0.0291055,-0.0683849,0.0539226},
{-0.0291055,-0.0683849,-0.0539226},
{-0.0386815,-0.0699299,0.0650384},
{-0.0386815,-0.0699299,-0.0650384},
{-0.0345956,-0.0680912,0.0508260},
{-0.0345956,-0.0680912,-0.0508260},
{-0.0335136,-0.0723389,0.0627383},
{-0.0335136,-0.0723389,-0.0627383},
{-0.0421291,0.0323153,0.0963164},
{-0.0421291,0.0323153,-0.0963164},
{-0.0497149,-0.0170924,0.0448689},
{-0.0497149,-0.0170924,-0.0448689},
{-0.0687601,-0.0054038,0.0050608},
{-0.0687601,-0.0054038,-0.0050608},
{-0.0840367,-0.0341151,0.0040271},
{-0.0840367,-0.0341151,-0.0040271},
{-0.0832060,-0.0590624,0.0189049},
{-0.0832060,-0.0590624,-0.0189049},
{-0.0375090,-0.0061744,0.0068785},
{-0.0375090,-0.0061744,-0.0068785},
{-0.0601617,0.0055440,0.0249240},
{-0.0601617,0.0055440,-0.0249240},
{-0.0745313,-0.0355116,0.0227865},
{-0.0745313,-0.0355116,-0.0227865},
{-0.0600944,-0.0205359,0.0161298},
{-0.0600944,-0.0205359,-0.0161298},
{-0.0886916,-0.0691077,0.0059086},
{-0.0886916,-0.0691077,-0.0059086},

```
                    {-0.0639921,-0.0263699,0.0080936},
                    {-0.0639921,-0.0263699,-0.0080936},
                    {-0.0812733,-0.0502575,0.0240681},
                    {-0.0812733,-0.0502575,-0.0240681},
                    {-0.0691103,-0.0334595,0.0077551},
                    {-0.0691103,-0.0334595,-0.0077551}
                    };
            BoundingBoxOnOff = Off;
        };
        AnyFunTransform3DIdentity ScaleFunction = {
            PreTransforms = {&.RBFTransform};
};
};

};
```

Biology Open • Supplementary information

ScalingFunctionTLEMLucyFemur_Mirrored_2014001

```
AnyFolder ScalingFunctionTLEMLucyFemur = {
  AnyFolder Right = {
    AnyFolder Thigh = {
        AnyFunTransform3DRBF RBFTransform = {
            RBFDef.Type = RBF_ThinPlate;
            PolynomDegree = 1;
            Points0 = {
                {0.0000000,0.0000000,0.0000000},
                {-0.0000000,-0.3616821,0.0000000},
                {-0.0097563,-0.3678799,0.0012967},
                {-0.0000000,-0.3660632,0.0408203},
                {-0.0000000,-0.3573010,-0.0408203},
                {0.0161460,-0.0072838,0.0601290},
                {0.0220217,-0.0203698,0.0463848},
                {0.0123977,-0.0241932,0.0668573},
                {0.0006898,0.0018121,0.0538181},
                {0.0122809,-0.0068668,0.0414535},
                {-0.0058991,-0.0138188,0.0648412},
                {0.0211469,-0.0380855,0.0538111},
                {0.0172133,-0.0317342,0.0328381},
                {0.0177311,-0.0131946,0.0256176},
                {-0.0110079,-0.0308867,0.0632370},
                {0.0039435,-0.0395977,0.0638271},
                {-0.0041683,-0.0187472,0.0413214},
                {-0.0177999,-0.0021535,0.0496084},
                {0.0001603,-0.0053126,0.0279593},
                {-0.0211600,-0.0216243,0.0497147},
                {0.0096163,-0.0568663,0.0545719},
                {0.0162112,-0.0515013,0.0395230},
                {0.0093533,-0.0433265,0.0187387},
                {0.0119398,-0.0238469,0.0118403},
                {0.0210707,-0.0048848,0.0065878},
                {0.0125013,0.0064094,0.0177702},
                {-0.0191523,-0.0356053,0.0382429},
                {-0.0115821,-0.0474937,0.0537648},
                {-0.0100073,-0.0236371,0.0267322},
                {-0.0078925,0.0107098,0.0182833},
                {-0.0134217,-0.0055356,0.0182000},
                {-0.0090061,-0.0665700,0.0475484},
                {0.0071095,-0.0761976,0.0520035},
                {0.0182119,-0.0704149,0.0386546},
                {0.0073764,-0.0609481,0.0221006},
                {-0.0122984,-0.0439585,0.0198428},
                {-0.0032253,-0.0306792,0.0110995},
```

Biology Open • Supplementary information

{-0.0012975,-0.0198451,-0.0052592},
{0.0140405,-0.0161125,-0.0071649},
{0.0189222,-0.0014898,-0.0127226},
{0.0181642,0.0132812,-0.0011050},
{0.0045119,0.0200001,0.0091189},
{-0.0176761,-0.0541983,0.0348587},
{-0.0136548,-0.0142015,0.0054311},
{-0.0135510,0.0182940,0.0017175},
{-0.0223865,0.0023399,0.0028018},
{-0.0086455,-0.0738247,0.0283843},
{-0.0050356,-0.0877749,0.0442195},
{0.0160135,-0.0919319,0.0465120},
{0.0128290,-0.0785810,0.0232431},
{-0.0168328,-0.0616327,0.0155917},
{-0.0172369,-0.0094567,-0.0119502},
{0.0002090,-0.0093641,-0.0209779},
{0.0064827,0.0081538,-0.0198453},
{0.0010777,0.0210422,-0.0084721},
{-0.0129485,0.0085451,-0.0162212},
{-0.0002322,-0.0924033,0.0245644},
{-0.0028175,-0.1052263,0.0418294},
{0.0172322,-0.1111444,0.0445760},
{0.0203259,-0.0957409,0.0271449},
{0.0057102,-0.1088933,0.0195113},
{-0.0010852,-0.1230776,0.0363132},
{0.0182727,-0.1303312,0.0422884},
{0.0242891,-0.1161703,0.0265054},
{0.0094531,-0.1268584,0.0162294},
{0.0014244,-0.1413293,0.0331653},
{0.0207271,-0.1496427,0.0394157},
{0.0267562,-0.1360142,0.0236798},
{0.0113839,-0.1461651,0.0136024},
{0.0030845,-0.1595094,0.0297156},
{0.0209528,-0.1691386,0.0369775},
{0.0283958,-0.1540209,0.0213795},
{0.0129499,-0.1649012,0.0113589},
{0.0016490,-0.1783108,0.0216287},
{0.0147105,-0.1880678,0.0328614},
{0.0298491,-0.1859865,0.0248551},
{0.0286645,-0.1714377,0.0169488},
{0.0165897,-0.1861006,0.0082595},
{0.0014000,-0.1973476,0.0183740},
{0.0114301,-0.2109792,0.0286157},
{0.0267422,-0.2037622,0.0296951},
{0.0288630,-0.2018335,0.0130127},
{0.0145471,-0.2083505,0.0062833},

Biology Open • Supplementary information

{0.0011300,-0.2198071,0.0155602},
{0.0126610,-0.2321235,0.0275354},
{0.0275647,-0.2219268,0.0265185},
{0.0286645,-0.2230253,0.0098701},
{0.0130434,-0.2304822,0.0039177},
{0.0022984,-0.2409585,0.0149650},
{0.0101315,-0.2534740,0.0252125},
{0.0279485,-0.2428555,0.0234276},
{0.0276684,-0.2421559,0.0058691},
{0.0101250,-0.2529527,0.0018983},
{0.0021376,-0.2667831,0.0107073},
{0.0114309,-0.2734905,0.0249960},
{0.0273923,-0.2634953,0.0216324},
{0.0278429,-0.2592546,0.0037692},
{0.0159101,-0.2712985,-0.0033988},
{0.0036708,-0.2863280,0.0004644},
{0.0023115,-0.2884756,0.0187554},
{0.0231385,-0.2879051,0.0224853},
{0.0304009,-0.2799973,0.0083549},
{0.0238189,-0.2917246,-0.0052462},
{0.0064904,-0.3027687,-0.0070587},
{-0.0003492,-0.3064690,0.0093014},
{0.0122179,-0.3045269,0.0243511},
{0.0301837,-0.3030127,0.0114462},
{0.0234182,-0.3107336,-0.0085198},
{0.0046893,-0.3214995,-0.0123881},
{-0.0022352,-0.3264905,0.0043340},
{0.0016841,-0.3202756,0.0217839},
{0.0229722,-0.3196887,0.0215083},
{0.0292438,-0.3228042,0.0051430},
{0.0221641,-0.3296190,-0.0132361},
{0.0100459,-0.3417597,-0.0244560},
{-0.0053009,-0.3375737,-0.0137705},
{-0.0064432,-0.3487307,0.0028676},
{-0.0059346,-0.3407801,0.0202938},
{0.0105130,-0.3365976,0.0264175},
{0.0282695,-0.3386249,0.0180307},
{0.0270402,-0.3431844,-0.0001487},
{0.0257040,-0.3506950,-0.0181851},
{0.0178948,-0.3636114,-0.0302838},
{0.0043149,-0.3544109,-0.0381949},
{-0.0095210,-0.3461482,-0.0295031},
{-0.0164526,-0.3542895,-0.0126666},
{-0.0060896,-0.3690127,-0.0033061},
{-0.0176523,-0.3612686,0.0110109},
{-0.0153310,-0.3546943,0.0299634},

{0.0021329,-0.3508021,0.0350763},
{0.0210690,-0.3548628,0.0279835},
{0.0356594,-0.3558162,0.0159356},
{0.0275718,-0.3617450,-0.0018607},
{0.0298503,-0.3741530,-0.0163721},
{0.0175320,-0.3855300,-0.0277515},
{0.0025604,-0.3740099,-0.0397137},
{-0.0136727,-0.3624016,-0.0402102},
{-0.0277647,-0.3567472,-0.0284947},
{-0.0275446,-0.3699630,-0.0120829},
{-0.0175846,-0.3859355,-0.0177922},
{0.0001572,-0.3856873,-0.0118050},
{0.0030262,-0.3834856,0.0075553},
{-0.0144114,-0.3810887,0.0140304},
{-0.0247706,-0.3701239,0.0285134},
{-0.0044804,-0.3677201,0.0404632},
{0.0133183,-0.3712274,0.0334208},
{0.0324861,-0.3723417,0.0245668},
{0.0271451,-0.3766027,0.0072441},
{0.0167985,-0.3822816,-0.0065349},
{-0.0001454,-0.3910823,-0.0303010},
{-0.0144998,-0.3834154,-0.0384137},
{-0.0301321,-0.3750841,-0.0321336},
{0.0196491,-0.3864804,0.0210853},
{0.0015726,-0.3888034,0.0276569},
{-0.0146652,-0.3847848,0.0336913},
{-0.0014588,-0.0619951,0.0219778},
{-0.0118435,-0.0550626,0.0139639},
{0.0129812,-0.0349966,0.0673128},
{-0.0162907,-0.0105666,0.0597739},
{-0.0012885,-0.0520816,0.0562989},
{-0.0090599,-0.0631298,0.0172224},
{-0.0000222,-0.0771663,0.0231897},
{-0.0068148,0.0023885,-0.0160086},
{-0.0180544,-0.0425409,0.0446585},
{0.0020525,-0.0249904,0.0691549},
{-0.0157097,-0.0662315,0.0238393},
{-0.0010518,-0.0510686,0.0199353},
{-0.0208874,-0.0152865,0.0500034},
{0.0168316,-0.0280857,0.0666680},
{-0.0187392,-0.0282333,0.0403282},
{0.0191622,-0.0351633,0.0622707},
{0.0001216,-0.0706289,0.0524049},
{-0.0169862,-0.0399062,0.0311827},
{-0.0185737,-0.0533066,0.0151224},
{-0.0219058,-0.0528723,0.0222263},

Biology Open • Supplementary information

{-0.0028271,-0.0391620,0.0148452},
{0.0117900,-0.0553425,0.0235348},
{0.0219835,-0.0106400,0.0396338},
{-0.0025366,-0.0401046,0.0625273},
{0.0141017,-0.0018767,0.0198108},
{-0.0078770,-0.0473929,0.0173542},
{-0.0065351,-0.0766431,0.0470258},
{0.0065585,0.0145593,0.0161638},
{-0.0118588,0.0172875,-0.0085064},
{-0.0074087,-0.0627500,0.0506700},
{0.0071940,-0.0780068,0.0213362},
{0.0126736,-0.0708176,0.0244108},
{0.0074694,-0.0436450,0.0604196},
{0.0154186,-0.0139471,0.0064312},
{-0.0053535,-0.0213210,0.0040094},
{-0.0088733,0.0002018,0.0205402},
{-0.0112471,-0.0542608,0.0500266},
{-0.0061625,-0.0248673,0.0653244},
{0.0093695,-0.0065826,0.0614157},
{0.0138015,-0.0467322,0.0284333},
{-0.0020999,-0.0186885,-0.0116392},
{-0.0185640,-0.0225591,0.0566779},
{0.0175798,0.0124360,-0.0068467},
{-0.0039962,0.0217127,0.0053055},
{0.0014975,-0.0784591,0.0514056},
{-0.0172997,-0.0386971,0.0525252},
{0.0196540,-0.0268271,0.0416806},
{0.0135296,-0.0406504,0.0236882},
{0.0182471,0.0095959,0.0087906},
{0.0218667,0.0016804,0.0058793},
{0.0224975,-0.0022652,-0.0022994},
{0.0166750,-0.0191391,0.0236436},
{0.0119785,-0.0328723,0.0195376},
{0.0187631,-0.0133469,0.0308741},
{-0.0187543,-0.0461463,0.0257491},
{0.0039834,-0.0373928,0.0683089},
{0.0157029,-0.0423169,0.0385226},
{0.0164003,-0.0087069,0.0131181},
{0.0008253,0.0033370,-0.0212576},
{0.0101707,-0.0009724,-0.0205688},
{0.0102948,-0.0642683,0.0524532},
{0.0152217,-0.0557944,0.0508978},
{0.0172973,-0.0565707,0.0419468},
{0.0167723,-0.0635334,0.0369834},
{-0.0047397,-0.0695325,0.0241398},
{-0.0152913,0.0035778,0.0161461},

{0.0091697,0.0009340,0.0575160},
{0.0196686,-0.0089533,0.0500006},
{0.0183998,-0.0212943,0.0622968},
{-0.0114340,-0.0316810,0.0250940},
{-0.0206442,-0.0092599,-0.0028425},
{0.0166868,-0.0784295,0.0276925},
{-0.0069678,-0.0054521,-0.0207011},
{0.0049490,0.0151109,-0.0160093},
{-0.0084025,-0.0318937,0.0162788},
{-0.0173679,0.0095594,-0.0108509},
{-0.0218912,0.0063457,-0.0020698},
{-0.0106547,-0.0668750,0.0397749},
{0.0136098,-0.0170666,-0.0018890},
{0.0141599,-0.0441405,0.0587594},
{0.0093255,-0.0183218,-0.0083008},
{0.0106367,-0.0128276,-0.0154269},
{0.0037350,-0.0549365,0.0209786},
{0.0087662,-0.0472902,0.0195496},
{0.0016839,-0.0708213,0.0221325},
{-0.0202977,-0.0530540,0.0301270},
{-0.0068584,0.0021781,0.0530002},
{-0.0144817,-0.0297218,0.0605833},
{0.0197015,-0.0165217,0.0560654},
{0.0156609,-0.0705363,0.0477736},
{0.0207876,-0.0221433,0.0330733},
{0.0092324,-0.0300767,0.0713160},
{-0.0203392,-0.0034488,0.0097332},
{0.0156978,-0.0143438,0.0629053},
{0.0143267,-0.0563519,0.0317489},
{-0.0161685,-0.0465391,0.0188591},
{-0.0196397,-0.0309875,0.0546496},
{0.0154804,-0.0380422,0.0305433},
{-0.0077774,-0.0351303,0.0637765},
{0.0063980,-0.0086419,-0.0201654},
{0.0030146,-0.0151094,-0.0165974},
{-0.0210080,-0.0096030,0.0507855},
{-0.0105684,-0.0411377,0.0586146},
{0.0180619,-0.0498960,0.0469835},
{-0.0192318,0.0116108,0.0024070},
{-0.0165651,0.0083622,0.0126870},
{-0.0014333,-0.0627138,0.0534746},
{0.0145127,-0.0080178,0.0353667},
{0.0148374,-0.0012228,0.0566181},
{0.0153834,-0.0062427,0.0444727},
{-0.0155164,-0.0132103,-0.0096398},
{0.0068685,-0.0221022,0.0011547},

Biology Open • Supplementary information

{0.0003332,-0.0458500,0.0183716},
{0.0205582,0.0040956,-0.0082969},
{0.0175752,0.0009340,-0.0148407},
{0.0142357,-0.0063623,-0.0169332},
{-0.0147285,0.0023412,-0.0164678},
{-0.0158781,-0.0064451,-0.0151346},
{0.0175951,0.0133456,0.0037559},
{0.0117660,0.0189899,-0.0030690},
{0.0096040,0.0151827,-0.0137450},
{0.0096565,-0.0215194,0.0662734},
{-0.0006793,-0.0074494,0.0631218},
{0.0007819,-0.0004519,0.0600781},
{0.0065377,-0.0125433,0.0654199},
{0.0090461,0.0073096,0.0188711},
{0.0151418,-0.0168302,0.0146465},
{0.0025166,-0.0231863,0.0688894},
{-0.0097891,-0.0152077,0.0253086},
{-0.0109149,-0.0177935,0.0119825},
{-0.0072079,-0.0099373,0.0272725},
{-0.0183727,-0.0467429,0.0356276},
{-0.0126871,-0.0584555,0.0428141},
{-0.0057054,-0.0273042,0.0103554},
{0.0215142,-0.0159693,0.0474531},
{0.0128492,-0.0070016,0.0287645},
{0.0052134,-0.0017047,0.0247463},
{-0.0024284,-0.0707498,0.0513049},
{-0.0036140,0.0220593,-0.0040484},
{-0.0002405,-0.0121253,0.0390050},
{-0.0119811,-0.0156737,0.0015445},
{0.0142082,-0.0279792,0.0246553},
{0.0122244,-0.0262013,0.0169829},
{-0.0146264,-0.0038855,0.0549384},
{0.0207450,-0.0325771,0.0496412},
{-0.0155040,-0.0497560,0.0437087},
{-0.0081119,-0.0756450,0.0387365},
{0.0039684,-0.0062047,0.0313900},
{-0.0023726,-0.0038892,-0.0220521},
{-0.0136977,-0.0339839,0.0602619},
{-0.0035399,0.0089081,-0.0188894},
{-0.0049820,0.0176571,-0.0132254},
{0.0193902,-0.0803191,0.0360429},
{0.0040648,-0.0697109,0.0218205},
{0.0170470,-0.0818610,0.0456892},
{0.0104175,0.0188635,0.0064965},
{0.0135206,0.0092549,0.0158079},
{0.0023933,0.0160498,-0.0157404},

{0.0164753,-0.0118197,-0.0096995},
{0.0202584,-0.0066858,-0.0082383},
{-0.0098401,-0.0226001,0.0314028},
{-0.0130937,-0.0309622,0.0320764},
{-0.0201510,-0.0613297,0.0222264},
{-0.0010389,0.0036055,0.0209003},
{0.0042025,0.0073392,0.0199393},
{-0.0021630,-0.0038531,0.0251898},
{-0.0007845,-0.0293574,0.0675788},
{0.0070027,-0.0406327,0.0624565},
{-0.0124363,-0.0183001,0.0627966},
{-0.0120485,-0.0256613,0.0628493},
{0.0101200,0.0197695,0.0039334},
{0.0167636,0.0148266,0.0014155},
{-0.0074981,-0.0106930,0.0632797},
{0.0142533,-0.0503445,0.0351954},
{-0.0146107,-0.0460953,0.0506120},
{-0.0161117,-0.0471301,0.0178900},
{0.0112149,-0.0792793,0.0501460},
{-0.0099060,-0.0230794,0.0186951},
{-0.0114543,-0.0153049,0.0183675},
{-0.0042882,0.0161879,0.0151423},
{0.0014582,-0.0638572,0.0541080},
{0.0048554,-0.0582603,0.0553815},
{-0.0017077,-0.0572302,0.0546635},
{0.0166090,-0.0697169,0.0323038},
{-0.0208812,-0.0164273,0.0474354},
{0.0130454,-0.0241864,0.0178294},
{0.0053123,-0.0483770,0.0582251},
{0.0112225,-0.0361429,0.0673300},
{0.0085729,-0.0771256,0.0216100},
{-0.0070783,-0.0488033,0.0560869},
{0.0182083,-0.0411024,0.0454236},
{-0.0119590,-0.0403809,0.0213546},
{0.0233409,-0.0185535,0.0366343},
{-0.0046347,-0.0571211,0.0208243},
{0.0183166,-0.0006966,0.0140116},
{0.0061913,0.0053512,0.0201003},
{-0.0002364,-0.0310828,0.0680351},
{-0.0170773,0.0139270,-0.0054987},
{0.0110805,0.0156034,0.0117010},
{-0.0124628,0.0164667,0.0094550},
{-0.0209014,-0.0073407,-0.0051969},
{-0.0210131,0.0010693,-0.0086891},
{-0.0041596,-0.0172332,0.0663041},
{-0.0216595,-0.0322172,0.0451540},

Biology Open • Supplementary information

{0.0051908,-0.0426014,0.0613013},
{0.0150003,-0.0416136,0.0606257},
{-0.0135090,-0.0674234,0.0236425},
{0.0146416,0.0073691,0.0160052},
{0.0195615,-0.0112412,-0.0010333},
{-0.0198115,-0.0098244,0.0044735},
{-0.0157054,-0.0521236,0.0395039},
{-0.0081381,-0.0173108,-0.0046152},
{-0.0218324,-0.0262661,0.0471115},
{0.0051446,-0.0707257,0.0530555},
{0.0179359,-0.0471493,0.0528134},
{-0.0005591,-0.0317983,0.0113632},
{0.0071707,-0.0305943,0.0121186},
{0.0206239,-0.0061367,0.0066642},
{0.0187057,-0.0745868,0.0420207},
{-0.0095784,-0.0163113,0.0259524},
{-0.0012181,0.0127661,0.0186855},
{0.0059420,-0.0630691,0.0220087},
{0.0069408,-0.0573878,0.0552614},
{0.0150782,-0.0132884,-0.0101793},
{0.0005533,-0.0078574,0.0633775},
{-0.0088284,-0.0146399,-0.0147837},
{0.0167444,-0.0651105,0.0470347},
{0.0082666,-0.0181741,-0.0095133},
{-0.0175162,-0.0637444,0.0177069},
{0.0153795,-0.0360767,0.0287608},
{0.0162363,-0.0018864,0.0548412},
{0.0052731,-0.0075919,0.0392990},
{-0.0022288,-0.0556644,0.0549909},
{-0.0156238,-0.0550038,0.0377688},
{-0.0204137,-0.0100550,0.0536429},
{-0.0093683,0.0122429,-0.0162013},
{-0.0202927,0.0049166,0.0083887},
{0.0074706,-0.0627945,0.0222133},
{0.0035034,-0.0376103,0.0672421},
{-0.0190752,-0.0194129,0.0555530},
{0.0061758,-0.0074961,-0.0207741},
{0.0192011,-0.0273063,0.0588233},
{0.0130796,-0.0134383,0.0638611},
{0.0120421,0.0019566,-0.0194140},
{0.0154152,-0.0065294,0.0179581},
{-0.0157335,0.0101219,0.0123981},
{0.0110376,-0.0488274,0.0567911},
{-0.0055170,-0.0343676,0.0647140},
{0.0216204,-0.0273988,0.0512041},
{0.0161331,-0.0135875,-0.0075501},

{-0.0184643,-0.0455633,0.0360087},
{-0.0091978,-0.0682413,0.0460847},
{0.0034304,-0.0471677,0.0588866},
{-0.0048025,-0.0384316,0.0149400},
{0.0118505,0.0109735,-0.0159161},
{-0.0027036,-0.0204189,-0.0015094},
{-0.0117607,-0.0692759,0.0292171},
{-0.0107080,-0.0033455,-0.0190487},
{-0.0042548,0.0214947,-0.0056846},
{-0.0097315,-0.0208529,0.0131997},
{-0.0178958,-0.0627218,0.0171551},
{-0.0068692,-0.0688818,0.0233316},
{0.0012083,0.0120695,-0.0186179},
{0.0215073,-0.0236027,0.0503069},
{-0.0060262,-0.0534931,0.0189523},
{0.0179951,-0.0336785,0.0397267},
{0.0190509,-0.0284972,0.0336394},
{-0.0169188,-0.0133609,-0.0014203},
{0.0135722,0.0136491,0.0114695},
{-0.0150549,-0.0123230,0.0067722},
{-0.0148411,-0.0311945,0.0355412},
{0.0185490,-0.0794042,0.0431266},
{0.0193568,-0.0087326,0.0575704},
{0.0201026,-0.0144353,0.0531586},
{0.0025583,-0.0248211,0.0045714},
{0.0132689,-0.0214866,0.0652899},
{-0.0028669,-0.0099311,0.0309271},
{-0.0219487,-0.0054893,-0.0021853},
{0.0026180,-0.0203806,-0.0040620},
{-0.0116230,-0.0668518,0.0370152},
{-0.0133007,-0.0264159,0.0619176},
{0.0186167,0.0050491,-0.0120059},
{-0.0191359,-0.0591690,0.0288149},
{-0.0178121,-0.0041834,0.0141448},
{0.0123077,-0.0063648,0.0279743},
{-0.0157729,-0.0374010,0.0315335},
{0.0149328,-0.0659100,0.0290095},
{0.0006205,0.0021977,-0.0214613},
{0.0170484,-0.0715687,0.0325980},
{0.0068499,0.0061192,0.0196608},
{-0.0167075,0.0020024,0.0150382},
{0.0059801,-0.0393117,0.0167489},
{-0.0072506,-0.0171067,0.0338751},
{0.0202363,-0.0251596,0.0420997},
{-0.0199311,-0.0601255,0.0171522},
{0.0216641,0.0053410,-0.0027841},

Biology Open • Supplementary information

{-0.0197665,-0.0606125,0.0252972},
{0.0129986,-0.0214528,0.0098002},
{0.0201151,-0.0100929,0.0460501},
{0.0104137,0.0017765,-0.0203073},
{0.0166557,-0.0064123,0.0140197},
{-0.0124120,-0.0596549,0.0422637},
{0.0014885,0.0202590,0.0096781},
{0.0028720,0.0020207,-0.0219044},
{0.0376095,-0.3675427,0.0214537},
{0.0049949,0.0206417,-0.0079017},
{-0.0191723,-0.0407590,0.0391310},
{-0.0316406,-0.3638215,-0.0274841},
{0.0021392,0.0226920,0.0000985},
{0.0168435,-0.0229382,0.0264788},
{-0.0157304,-0.0005437,-0.0161456},
{0.0203722,-0.0299750,0.0562762},
{-0.0171077,0.0134146,0.0059615},
{0.0175519,-0.0370653,0.0413374},
{0.0154657,-0.0405127,0.0348980},
{-0.0215172,-0.0323502,0.0481925},
{0.0177998,-0.0515776,0.0444709},
{-0.0183804,-0.0448316,0.0275139},
{0.0033607,-0.0502028,0.0575894},
{0.0010325,-0.0479377,0.0191141},
{0.0224852,-0.0181821,0.0451780},
{0.0084089,-0.0720538,0.0521031},
{-0.0070880,-0.0772912,0.0288023},
{-0.0223712,-0.0028990,0.0024047},
{0.0173136,-0.0120017,0.0204612},
{0.0137772,-0.0623537,0.0276621},
{-0.0100390,-0.0231180,0.0292222},
{-0.0196395,-0.3730207,0.0364730},
{-0.0094445,-0.0526688,0.0528111},
{-0.0115309,-0.3679776,-0.0104995},
{0.0129812,-0.0410477,0.0224072},
{0.0117394,0.0154849,-0.0117191},
{-0.0245208,-0.3649412,0.0295111},
{0.0147476,-0.0502966,0.0364899},
{0.0006132,-0.0446771,0.0600524},
{-0.0118123,0.0191139,0.0031977},
{-0.0017185,-0.0163303,0.0664216},
{-0.0085098,-0.0074564,0.0247745},
{0.0197459,-0.0086852,0.0430460},
{0.0187064,-0.0223532,0.0600966},
{-0.0206552,-0.0099712,0.0525024},
{0.0207525,0.0084646,-0.0024846},

Biology Open • Supplementary information

{-0.0094915,-0.0325446,0.0184441},
{-0.0014464,-0.0140050,-0.0179819},
{-0.0122725,-0.0573444,0.0463125},
{-0.0106397,0.0177211,0.0094669},
{0.0111981,0.0191236,-0.0041491},
{-0.0162790,-0.0256279,0.0592687},
{-0.0060120,-0.3653959,0.0413411},
{-0.0135007,-0.3703638,-0.0387476},
{-0.0134258,-0.3703142,-0.0379467},
{-0.0133509,-0.3702645,-0.0371458},
{-0.0132760,-0.3702148,-0.0363450},
{-0.0132011,-0.3701651,-0.0355441},
{-0.0131262,-0.3701154,-0.0347432},
{-0.0130513,-0.3700658,-0.0339423},
{-0.0129765,-0.3700161,-0.0331414},
{-0.0129016,-0.3699664,-0.0323405},
{-0.0128267,-0.3699167,-0.0315396},
{-0.0127518,-0.3698670,-0.0307388},
{-0.0126769,-0.3698174,-0.0299379},
{-0.0126020,-0.3697677,-0.0291370},
{-0.0125271,-0.3697180,-0.0283361},
{-0.0124523,-0.3696683,-0.0275352},
{-0.0123774,-0.3696187,-0.0267343},
{-0.0123025,-0.3695690,-0.0259334},
{-0.0122276,-0.3695193,-0.0251325},
{-0.0121527,-0.3694696,-0.0243317},
{-0.0120778,-0.3694199,-0.0235308},
{-0.0120029,-0.3693703,-0.0227299},
{-0.0119280,-0.3693206,-0.0219290},
{-0.0118532,-0.3692709,-0.0211281},
{-0.0117783,-0.3692212,-0.0203272},
{-0.0117034,-0.3691715,-0.0195263},
{-0.0116285,-0.3691219,-0.0187255},
{-0.0115536,-0.3690722,-0.0179246},
{-0.0114787,-0.3690225,-0.0171237},
{-0.0114038,-0.3689728,-0.0163228},
{-0.0113289,-0.3689231,-0.0155219},
{-0.0112541,-0.3688735,-0.0147210},
{-0.0111792,-0.3688238,-0.0139201},
{-0.0111043,-0.3687741,-0.0131192},
{-0.0110294,-0.3687244,-0.0123184},
{-0.0109545,-0.3686748,-0.0115175},
{-0.0108796,-0.3686251,-0.0107166},
{-0.0108047,-0.3685754,-0.0099157},
{-0.0107298,-0.3685257,-0.0091148},
{-0.0106550,-0.3684760,-0.0083139},

Biology Open • Supplementary information

{-0.0105801,-0.3684264,-0.0075130},
{-0.0105052,-0.3683767,-0.0067121},
{-0.0104303,-0.3683270,-0.0059113},
{-0.0103554,-0.3682773,-0.0051104},
{-0.0102805,-0.3682276,-0.0043095},
{-0.0102056,-0.3681780,-0.0035086},
{-0.0101307,-0.3681283,-0.0027077},
{-0.0100559,-0.3680786,-0.0019068},
{-0.0099810,-0.3680289,-0.0011059},
{-0.0099061,-0.3679792,-0.0003051},
{-0.0098312,-0.3679296,0.0004958},
{-0.0097563,-0.3678799,0.0012967},
{-0.0096814,-0.3678302,0.0020976},
{-0.0096065,-0.3677805,0.0028985},
{-0.0095317,-0.3677308,0.0036994},
{-0.0094568,-0.3676812,0.0045003},
{-0.0093819,-0.3676315,0.0053012},
{-0.0093070,-0.3675818,0.0061020},
{-0.0092321,-0.3675321,0.0069029},
{-0.0091572,-0.3674825,0.0077038},
{-0.0090823,-0.3674328,0.0085047},
{-0.0090074,-0.3673831,0.0093056},
{-0.0089326,-0.3673334,0.0101065},
{-0.0088577,-0.3672837,0.0109074},
{-0.0087828,-0.3672341,0.0117082},
{-0.0087079,-0.3671844,0.0125091},
{-0.0086330,-0.3671347,0.0133100},
{-0.0085581,-0.3670850,0.0141109},
{-0.0084832,-0.3670353,0.0149118},
{-0.0084083,-0.3669857,0.0157127},
{-0.0083335,-0.3669360,0.0165136},
{-0.0082586,-0.3668863,0.0173145},
{-0.0081837,-0.3668366,0.0181153},
{-0.0081088,-0.3667869,0.0189162},
{-0.0080339,-0.3667373,0.0197171},
{-0.0079590,-0.3666876,0.0205180},
{-0.0078841,-0.3666379,0.0213189},
{-0.0078092,-0.3665882,0.0221198},
{-0.0077344,-0.3665386,0.0229207},
{-0.0076595,-0.3664889,0.0237215},
{-0.0075846,-0.3664392,0.0245224},
{-0.0075097,-0.3663895,0.0253233},
{-0.0074348,-0.3663398,0.0261242},
{-0.0073599,-0.3662902,0.0269251},
{-0.0072850,-0.3662405,0.0277260},
{-0.0072101,-0.3661908,0.0285269},

```
        {-0.0071353,-0.3661411,0.0293278},
        {-0.0070604,-0.3660914,0.0301286},
        {-0.0069855,-0.3660418,0.0309295},
        {-0.0069106,-0.3659921,0.0317304},
        {-0.0068357,-0.3659424,0.0325313},
        {-0.0067608,-0.3658927,0.0333322},
        {-0.0066859,-0.3658430,0.0341331},
        {-0.0066111,-0.3657934,0.0349340},
        {-0.0065362,-0.3657437,0.0357348},
        {-0.0064613,-0.3656940,0.0365357},
        {-0.0063864,-0.3656443,0.0373366},
        {-0.0063115,-0.3655947,0.0381375},
        {-0.0062366,-0.3655450,0.0389384},
        {-0.0061617,-0.3654953,0.0397393},
        {-0.0060868,-0.3654456,0.0405402}
        };
Points1 = {
        {0.0000000,0.0000000,0.0000000},
        {-0.0000000,-0.3844239,0.0000000},
        {-0.0090962,-0.3910114,0.0012090},
        {-0.0000000,-0.3890805,0.0380584},
        {-0.0000000,-0.3797674,-0.0380584},
        {0.0135412,-0.0086390,0.0545946},
        {0.0196483,-0.0224683,0.0429684},
        {0.0094431,-0.0254847,0.0595583},
        {0.0001526,-0.0003974,0.0485973},
        {0.0109662,-0.0068334,0.0378725},
        {-0.0057810,-0.0139956,0.0617691},
        {0.0164460,-0.0386834,0.0492159},
        {0.0147348,-0.0335894,0.0316250},
        {0.0139180,-0.0144630,0.0239261},
        {-0.0099034,-0.0321235,0.0589375},
        {0.0032807,-0.0420515,0.0579651},
        {-0.0030036,-0.0201361,0.0393148},
        {-0.0121921,-0.0043215,0.0457883},
        {-0.0000087,-0.0042392,0.0265660},
        {-0.0119720,-0.0229308,0.0470686},
        {0.0098360,-0.0608614,0.0517067},
        {0.0156426,-0.0540685,0.0368361},
        {0.0081543,-0.0456550,0.0184707},
        {0.0094302,-0.0243144,0.0120498},
        {0.0178693,-0.0055164,0.0055080},
        {0.0101116,0.0060820,0.0153716},
        {-0.0119099,-0.0382496,0.0350282},
        {-0.0097840,-0.0497224,0.0497680},
        {-0.0089820,-0.0255951,0.0240460},
```

{-0.0059634,0.0107064,0.0153997},
{-0.0110670,-0.0058902,0.0151803},
{-0.0072431,-0.0703173,0.0431595},
{0.0061293,-0.0806876,0.0501372},
{0.0166575,-0.0756364,0.0355340},
{0.0080844,-0.0651352,0.0202955},
{-0.0114436,-0.0462484,0.0199460},
{-0.0040837,-0.0328744,0.0098553},
{-0.0004320,-0.0199527,-0.0046396},
{0.0124444,-0.0156380,-0.0062994},
{0.0168150,-0.0017977,-0.0107581},
{0.0164873,0.0130246,-0.0011711},
{0.0030473,0.0197657,0.0086996},
{-0.0138372,-0.0574107,0.0327293},
{-0.0114962,-0.0125451,0.0056021},
{-0.0117669,0.0170421,0.0015537},
{-0.0179504,0.0023626,0.0030653},
{-0.0066042,-0.0778865,0.0268022},
{-0.0041791,-0.0933870,0.0406404},
{0.0141802,-0.0980784,0.0433196},
{0.0121047,-0.0837981,0.0210443},
{-0.0098260,-0.0622066,0.0207177},
{-0.0140997,-0.0088782,-0.0098827},
{0.0006385,-0.0091689,-0.0180615},
{0.0057996,0.0077976,-0.0166249},
{0.0016318,0.0201703,-0.0078545},
{-0.0102073,0.0084386,-0.0140539},
{0.0004298,-0.0982470,0.0225398},
{-0.0018052,-0.1116971,0.0387523},
{0.0152711,-0.1182631,0.0413296},
{0.0182152,-0.1016684,0.0254613},
{0.0060568,-0.1154334,0.0186961},
{0.0000134,-0.1304245,0.0339339},
{0.0163456,-0.1384823,0.0391950},
{0.0217395,-0.1234026,0.0249958},
{0.0094227,-0.1343665,0.0159732},
{0.0022676,-0.1497358,0.0311605},
{0.0185995,-0.1589238,0.0365616},
{0.0240072,-0.1444068,0.0224697},
{0.0111285,-0.1548267,0.0136295},
{0.0037291,-0.1690250,0.0280404},
{0.0188925,-0.1795936,0.0343210},
{0.0255149,-0.1635052,0.0203586},
{0.0124673,-0.1747466,0.0115332},
{0.0025634,-0.1889392,0.0207514},
{0.0135960,-0.1996045,0.0306237},

Biology Open • Supplementary information

{0.0267720,-0.1975298,0.0233111},
{0.0258527,-0.1819770,0.0163210},
{0.0155784,-0.1973494,0.0085822},
{0.0022824,-0.2092063,0.0177187},
{0.0107691,-0.2239396,0.0267330},
{0.0240408,-0.2164442,0.0275959},
{0.0260990,-0.2143039,0.0126082},
{0.0137584,-0.2210323,0.0066529},
{0.0019272,-0.2331542,0.0150273},
{0.0117936,-0.2464923,0.0256401},
{0.0248176,-0.2357798,0.0246453},
{0.0259704,-0.2368650,0.0096352},
{0.0123799,-0.2446174,0.0043429},
{0.0028017,-0.2557481,0.0143195},
{0.0095327,-0.2692291,0.0234601},
{0.0252244,-0.2580655,0.0217569},
{0.0251523,-0.2572308,0.0058898},
{0.0097243,-0.2685715,0.0023294},
{0.0024975,-0.2833112,0.0102660},
{0.0106350,-0.2905897,0.0231875},
{0.0248192,-0.2800449,0.0200541},
{0.0253535,-0.2754549,0.0038535},
{0.0148192,-0.2881857,-0.0027043},
{0.0037536,-0.3041721,0.0007302},
{0.0024332,-0.3065012,0.0174974},
{0.0211643,-0.3060160,0.0208162},
{0.0276998,-0.2975966,0.0078790},
{0.0219434,-0.3100179,-0.0046363},
{0.0061855,-0.3217350,-0.0063975},
{-0.0001608,-0.3256884,0.0087270},
{0.0113456,-0.3236657,0.0226159},
{0.0277738,-0.3220914,0.0106489},
{0.0217295,-0.3302622,-0.0078546},
{0.0043720,-0.3417147,-0.0115499},
{-0.0020839,-0.3470196,0.0040407},
{0.0015701,-0.3404139,0.0203100},
{0.0214057,-0.3397921,0.0200474},
{0.0272577,-0.3431024,0.0047959},
{0.0206645,-0.3503447,-0.0123405},
{0.0093662,-0.3632489,-0.0228013},
{-0.0049422,-0.3587997,-0.0128388},
{-0.0060072,-0.3706581,0.0026736},
{-0.0055331,-0.3622077,0.0189207},
{0.0098017,-0.3577621,0.0246301},
{0.0263577,-0.3599170,0.0168107},
{0.0252116,-0.3647631,-0.0001387},

{0.0239630,-0.3727459,-0.0169538},
{0.0166841,-0.3864745,-0.0282348},
{0.0040230,-0.3766956,-0.0356078},
{-0.0088768,-0.3679133,-0.0275069},
{-0.0153394,-0.3765665,-0.0118095},
{-0.0056776,-0.3922154,-0.0030824},
{-0.0164580,-0.3839844,0.0102659},
{-0.0142937,-0.3769968,0.0279361},
{0.0019886,-0.3728598,0.0327030},
{0.0196435,-0.3771759,0.0260902},
{0.0332458,-0.3781893,0.0148574},
{0.0257073,-0.3844909,-0.0017348},
{0.0278306,-0.3976790,-0.0152644},
{0.0163467,-0.4097714,-0.0258738},
{0.0023871,-0.3975269,-0.0370267},
{-0.0127485,-0.3851887,-0.0374905},
{-0.0258861,-0.3791787,-0.0265677},
{-0.0256809,-0.3932256,-0.0112654},
{-0.0163939,-0.4102024,-0.0165883},
{0.0001466,-0.4099386,-0.0110062},
{0.0028214,-0.4075984,0.0070441},
{-0.0134363,-0.4050508,0.0130811},
{-0.0230946,-0.3933966,0.0265842},
{-0.0041773,-0.3908416,0.0377255},
{0.0124171,-0.3945694,0.0311595},
{0.0302890,-0.3957538,0.0229046},
{0.0253085,-0.4002828,0.0067539},
{0.0156619,-0.4063187,-0.0060927},
{-0.0001355,-0.4156727,-0.0282518},
{-0.0135187,-0.4075238,-0.0358146},
{-0.0280933,-0.3986687,-0.0299595},
{0.0183187,-0.4107815,0.0196587},
{0.0014662,-0.4132505,0.0257866},
{-0.0136720,-0.4089793,0.0314117},
{-0.0007887,-0.0668699,0.0180990},
{-0.0094557,-0.0582117,0.0196269},
{0.0090134,-0.0341478,0.0587373},
{-0.0130054,-0.0138754,0.0541406},
{-0.0015942,-0.0568813,0.0544290},
{-0.0057688,-0.0638763,0.0213544},
{-0.0012920,-0.0820757,0.0195001},
{-0.0064060,0.0016957,-0.0161583},
{-0.0114202,-0.0446431,0.0391109},
{0.0030282,-0.0266108,0.0622759},
{-0.0093114,-0.0659858,0.0244248},
{-0.0009779,-0.0542329,0.0174407},

Biology Open • Supplementary information

{-0.0120751,-0.0170313,0.0482581},
{0.0124771,-0.0301183,0.0571420},
{-0.0095913,-0.0299145,0.0377167},
{0.0130969,-0.0344253,0.0555422},
{-0.0008166,-0.0749192,0.0510161},
{-0.0135817,-0.0438598,0.0296953},
{-0.0127968,-0.0556168,0.0208900},
{-0.0149019,-0.0543970,0.0220972},
{-0.0022733,-0.0413394,0.0128944},
{0.0099263,-0.0579505,0.0226928},
{0.0198865,-0.0132547,0.0375802},
{-0.0028638,-0.0440375,0.0588229},
{0.0124334,-0.0028068,0.0163509},
{-0.0081238,-0.0486534,0.0179570},
{-0.0054765,-0.0808815,0.0421165},
{0.0051223,0.0147705,0.0124561},
{-0.0103827,0.0169941,-0.0057102},
{-0.0064846,-0.0671946,0.0450811},
{0.0072034,-0.0845142,0.0184154},
{0.0118795,-0.0741378,0.0230526},
{0.0071176,-0.0470642,0.0557831},
{0.0132683,-0.0125793,0.0071998},
{-0.0053102,-0.0234557,0.0043424},
{-0.0066089,-0.0009675,0.0190450},
{-0.0092851,-0.0560195,0.0463883},
{-0.0056952,-0.0260635,0.0623352},
{0.0077351,-0.0063286,0.0569325},
{0.0125115,-0.0503110,0.0280874},
{-0.0018455,-0.0180506,-0.0114262},
{-0.0126754,-0.0243432,0.0517598},
{0.0147287,0.0132441,-0.0066498},
{-0.0037315,0.0214621,0.0034644},
{0.0005805,-0.0846388,0.0494817},
{-0.0119445,-0.0393492,0.0468608},
{0.0182617,-0.0278250,0.0397911},
{0.0106526,-0.0427121,0.0226210},
{0.0163902,0.0103381,0.0067703},
{0.0188891,0.0020704,0.0063238},
{0.0197730,-0.0019705,-0.0032008},
{0.0126660,-0.0200115,0.0211026},
{0.0105136,-0.0344463,0.0201481},
{0.0149213,-0.0146037,0.0301881},
{-0.0159564,-0.0487215,0.0248157},
{0.0027610,-0.0380866,0.0604285},
{0.0154371,-0.0448599,0.0359619},
{0.0133431,-0.0100607,0.0124200},

Biology Open • Supplementary information

{0.0009423,0.0043629,-0.0181755},
{0.0079634,-0.0007580,-0.0180373},
{0.0095377,-0.0669463,0.0502489},
{0.0136217,-0.0604541,0.0477345},
{0.0159995,-0.0587368,0.0396918},
{0.0161825,-0.0679973,0.0335669},
{-0.0043095,-0.0741897,0.0229721},
{-0.0121074,0.0037042,0.0130612},
{0.0070450,-0.0011130,0.0515776},
{0.0175287,-0.0092733,0.0470143},
{0.0136662,-0.0234382,0.0562557},
{-0.0108685,-0.0354352,0.0236725},
{-0.0167529,-0.0074210,-0.0023867},
{0.0149057,-0.0819262,0.0255869},
{-0.0063627,-0.0056850,-0.0177537},
{0.0037516,0.0139531,-0.0138318},
{-0.0085184,-0.0327042,0.0149349},
{-0.0135587,0.0102048,-0.0097181},
{-0.0184025,0.0049076,-0.0015510},
{-0.0083176,-0.0693211,0.0369128},
{0.0106521,-0.0172263,-0.0008855},
{0.0114644,-0.0449247,0.0535601},
{0.0079585,-0.0182646,-0.0083900},
{0.0092230,-0.0137554,-0.0128590},
{0.0039493,-0.0587102,0.0176869},
{0.0082230,-0.0500830,0.0200395},
{0.0011238,-0.0750818,0.0187358},
{-0.0167293,-0.0553962,0.0281862},
{-0.0055616,-0.0013429,0.0486990},
{-0.0120595,-0.0303987,0.0555482},
{0.0165144,-0.0171292,0.0505306},
{0.0138167,-0.0759696,0.0450637},
{0.0173092,-0.0236953,0.0327538},
{0.0077005,-0.0314379,0.0621256},
{-0.0156834,-0.0032076,0.0092745},
{0.0132996,-0.0159319,0.0562265},
{0.0142838,-0.0604781,0.0304611},
{-0.0140300,-0.0495044,0.0210445},
{-0.0129724,-0.0329429,0.0491580},
{0.0128270,-0.0411078,0.0285616},
{-0.0072187,-0.0382487,0.0601490},
{0.0061300,-0.0076454,-0.0176148},
{0.0038223,-0.0152338,-0.0141654},
{-0.0133040,-0.0106516,0.0481183},
{-0.0096502,-0.0442140,0.0541564},
{0.0161242,-0.0533922,0.0441862},

Biology Open • Supplementary information

{-0.0156133,0.0110312,0.0014663},
{-0.0127723,0.0090599,0.0108941},
{-0.0017614,-0.0671824,0.0512529},
{0.0122862,-0.0091759,0.0318947},
{0.0128444,-0.0034619,0.0515574},
{0.0141256,-0.0056746,0.0404035},
{-0.0118366,-0.0132950,-0.0080490},
{0.0053050,-0.0216999,0.0022023},
{0.0003418,-0.0484675,0.0159542},
{0.0185526,0.0030942,-0.0071111},
{0.0159020,0.0016054,-0.0123302},
{0.0118597,-0.0076253,-0.0147885},
{-0.0121715,0.0013569,-0.0144687},
{-0.0135909,-0.0059092,-0.0123980},
{0.0164055,0.0130813,0.0030031},
{0.0098027,0.0188623,-0.0021496},
{0.0083674,0.0137220,-0.0123909},
{0.0074769,-0.0223391,0.0604625},
{-0.0006593,-0.0085333,0.0596341},
{-0.0001538,-0.0020680,0.0537435},
{0.0049927,-0.0135648,0.0610058},
{0.0077959,0.0072674,0.0155171},
{0.0114423,-0.0172081,0.0147144},
{0.0036573,-0.0250000,0.0620342},
{-0.0084734,-0.0170876,0.0229152},
{-0.0094969,-0.0172702,0.0114391},
{-0.0060193,-0.0098906,0.0255878},
{-0.0140126,-0.0501615,0.0334743},
{-0.0105578,-0.0606398,0.0387460},
{-0.0064442,-0.0287821,0.0088575},
{0.0192618,-0.0183031,0.0437267},
{0.0113819,-0.0083733,0.0261874},
{0.0040221,-0.0009361,0.0229429},
{-0.0033351,-0.0752326,0.0498851},
{-0.0022379,0.0217088,-0.0030914},
{-0.0010637,-0.0130828,0.0369914},
{-0.0101770,-0.0142180,0.0023291},
{0.0120638,-0.0285334,0.0242454},
{0.0096126,-0.0269112,0.0162002},
{-0.0102426,-0.0068867,0.0497947},
{0.0174390,-0.0328356,0.0453571},
{-0.0112391,-0.0530645,0.0392889},
{-0.0069556,-0.0802035,0.0356191},
{0.0040341,-0.0052035,0.0291718},
{-0.0027823,-0.0045673,-0.0188781},
{-0.0117433,-0.0344605,0.0551314},

Biology Open • Supplementary information

{-0.0028024,0.0088279,-0.0159590},
{-0.0040239,0.0164587,-0.0117857},
{0.0173291,-0.0851364,0.0343393},
{0.0034386,-0.0734205,0.0179961},
{0.0153287,-0.0878406,0.0422141},
{0.0089280,0.0190448,0.0050877},
{0.0109361,0.0090790,0.0137818},
{0.0010231,0.0149588,-0.0135247},
{0.0149706,-0.0116002,-0.0083653},
{0.0176302,-0.0075876,-0.0071738},
{-0.0081006,-0.0241792,0.0287684},
{-0.0101525,-0.0343835,0.0304094},
{-0.0124789,-0.0614135,0.0243258},
{-0.0013182,0.0026495,0.0189991},
{0.0038672,0.0072902,0.0163910},
{-0.0023420,-0.0028404,0.0242719},
{0.0002390,-0.0306874,0.0615637},
{0.0064227,-0.0432597,0.0566578},
{-0.0108774,-0.0197799,0.0593510},
{-0.0108523,-0.0262309,0.0586232},
{0.0084023,0.0201837,0.0025047},
{0.0158937,0.0140981,0.0013290},
{-0.0066221,-0.0108767,0.0604277},
{0.0141491,-0.0529779,0.0325118},
{-0.0115313,-0.0474039,0.0454495},
{-0.0139469,-0.0502715,0.0208185},
{0.0101811,-0.0840299,0.0478670},
{-0.0091023,-0.0251593,0.0167484},
{-0.0093821,-0.0153209,0.0161037},
{-0.0041110,0.0159423,0.0119511},
{0.0008306,-0.0679297,0.0529183},
{0.0054678,-0.0626280,0.0530810},
{-0.0020970,-0.0623174,0.0526033},
{0.0153877,-0.0734345,0.0293818},
{-0.0115642,-0.0182323,0.0463033},
{0.0099054,-0.0247725,0.0163028},
{0.0047846,-0.0523180,0.0554163},
{0.0077091,-0.0349649,0.0589176},
{0.0088551,-0.0839814,0.0187731},
{-0.0062918,-0.0526965,0.0538054},
{0.0166394,-0.0426369,0.0415858},
{-0.0110627,-0.0426195,0.0200014},
{0.0196653,-0.0205286,0.0353775},
{-0.0037392,-0.0606745,0.0191871},
{0.0154501,-0.0019814,0.0120689},
{0.0057281,0.0053758,0.0165321},

Biology Open • Supplementary information

{0.0008500,-0.0322623,0.0614523},
{-0.0137553,0.0136450,-0.0047946},
{0.0098432,0.0155860,0.0096012},
{-0.0099024,0.0159025,0.0077040},
{-0.0172680,-0.0054575,-0.0048780},
{-0.0173709,0.0005048,-0.0069973},
{-0.0045478,-0.0173635,0.0626985},
{-0.0122018,-0.0340707,0.0411092},
{0.0051844,-0.0464891,0.0566311},
{0.0115389,-0.0412880,0.0545119},
{-0.0081114,-0.0677274,0.0239750},
{0.0117218,0.0070277,0.0141925},
{0.0169194,-0.0106024,-0.0010595},
{-0.0160763,-0.0079660,0.0047226},
{-0.0118551,-0.0557260,0.0364289},
{-0.0067862,-0.0166791,-0.0035060},
{-0.0120043,-0.0272752,0.0440636},
{0.0043179,-0.0741040,0.0514557},
{0.0148240,-0.0493959,0.0485098},
{-0.0016103,-0.0344851,0.0103149},
{0.0057978,-0.0316682,0.0122949},
{0.0174222,-0.0068941,0.0054067},
{0.0168224,-0.0805489,0.0390058},
{-0.0084757,-0.0187225,0.0234833},
{-0.0008156,0.0128134,0.0150291},
{0.0068713,-0.0674924,0.0197946},
{0.0081614,-0.0616758,0.0524786},
{0.0139401,-0.0128184,-0.0088097},
{0.0005159,-0.0089987,0.0600785},
{-0.0066359,-0.0145776,-0.0132087},
{0.0147515,-0.0696786,0.0438172},
{0.0071352,-0.0182011,-0.0096410},
{-0.0098507,-0.0637090,0.0216450},
{0.0123535,-0.0388534,0.0270478},
{0.0143993,-0.0042265,0.0502024},
{0.0044023,-0.0075467,0.0365939},
{-0.0028174,-0.0610119,0.0531227},
{-0.0118809,-0.0588063,0.0354761},
{-0.0134123,-0.0112835,0.0502358},
{-0.0072929,0.0121154,-0.0136193},
{-0.0159312,0.0057130,0.0075757},
{0.0086738,-0.0672272,0.0206366},
{0.0023140,-0.0383787,0.0595802},
{-0.0125290,-0.0213931,0.0511031},
{0.0059751,-0.0060507,-0.0182666},
{0.0142933,-0.0278644,0.0525299},

{0.0111000,-0.0147643,0.0576417},
{0.0096677,0.0020387,-0.0168250},
{0.0127175,-0.0079801,0.0159781},
{-0.0120056,0.0108821,0.0108670},
{0.0096108,-0.0516302,0.0535074},
{-0.0050709,-0.0374938,0.0609148},
{0.0180380,-0.0283709,0.0465221},
{0.0147019,-0.0129140,-0.0065163},
{-0.0138432,-0.0489891,0.0339402},
{-0.0070058,-0.0719309,0.0420789},
{0.0029682,-0.0513328,0.0561035},
{-0.0040954,-0.0403913,0.0130483},
{0.0103315,0.0104239,-0.0136039},
{-0.0019883,-0.0210660,-0.0007860},
{-0.0085048,-0.0714517,0.0281430},
{-0.0095821,-0.0034131,-0.0163607},
{-0.0027054,0.0211192,-0.0043523},
{-0.0088947,-0.0211578,0.0123545},
{-0.0099651,-0.0627664,0.0214797},
{-0.0055172,-0.0727971,0.0231259},
{0.0008843,0.0115484,-0.0155632},
{0.0184120,-0.0253112,0.0458065},
{-0.0054699,-0.0564873,0.0185570},
{0.0163588,-0.0353568,0.0370195},
{0.0163351,-0.0299710,0.0329823},
{-0.0133245,-0.0116445,-0.0009141},
{0.0123152,0.0136437,0.0094656},
{-0.0124233,-0.0106200,0.0065013},
{-0.0099280,-0.0342535,0.0335597},
{0.0166670,-0.0853763,0.0398368},
{0.0164103,-0.0100530,0.0522934},
{0.0174182,-0.0151368,0.0484459},
{0.0012641,-0.0257542,0.0047157},
{0.0100176,-0.0227107,0.0589978},
{-0.0025898,-0.0092739,0.0294853},
{-0.0178980,-0.0041246,-0.0016325},
{0.0025603,-0.0204194,-0.0035775},
{-0.0089913,-0.0691025,0.0347025},
{-0.0118267,-0.0266922,0.0572066},
{0.0166110,0.0050397,-0.0101060},
{-0.0137133,-0.0604575,0.0282866},
{-0.0141421,-0.0040768,0.0120114},
{0.0111361,-0.0078018,0.0253998},
{-0.0124694,-0.0415881,0.0300852},
{0.0139706,-0.0694544,0.0270280},
{0.0007143,0.0032695,-0.0184127},

Biology Open • Supplementary information

{0.0156736,-0.0751892,0.0294906},
{0.0064452,0.0061902,0.0158407},
{-0.0132627,0.0021920,0.0121625},
{0.0054316,-0.0416285,0.0155919},
{-0.0060117,-0.0184515,0.0319557},
{0.0190799,-0.0261407,0.0403651},
{-0.0119826,-0.0606963,0.0215982},
{0.0193634,0.0049584,-0.0028606},
{-0.0128836,-0.0608571,0.0263167},
{0.0104597,-0.0216870,0.0104918},
{0.0183283,-0.0113353,0.0430617},
{0.0080435,0.0018860,-0.0177145},
{0.0137002,-0.0080500,0.0128413},
{-0.0105322,-0.0615999,0.0381348},
{0.0004302,0.0199572,0.0087433},
{0.0026396,0.0032015,-0.0187145},
{0.0350564,-0.3906540,0.0199985},
{0.0047509,0.0197753,-0.0074096},
{-0.0126942,-0.0436037,0.0355161},
{-0.0295007,-0.3866978,-0.0256255},
{0.0021775,0.0223723,-0.0000304},
{0.0135059,-0.0237696,0.0253259},
{-0.0132663,-0.0010658,-0.0138399},
{0.0155569,-0.0300151,0.0504833},
{-0.0137713,0.0130274,0.0045177},
{0.0162187,-0.0388298,0.0381509},
{0.0144746,-0.0433184,0.0327237},
{-0.0124729,-0.0341686,0.0436108},
{0.0162860,-0.0548665,0.0419723},
{-0.0157200,-0.0476782,0.0261931},
{0.0028722,-0.0544711,0.0553893},
{0.0010221,-0.0508142,0.0165418},
{0.0201222,-0.0207050,0.0419589},
{0.0074292,-0.0756616,0.0502085},
{-0.0055895,-0.0821432,0.0266136},
{-0.0178333,-0.0021318,0.0029091},
{0.0135174,-0.0132719,0.0188061},
{0.0130009,-0.0659477,0.0261675},
{-0.0087276,-0.0244484,0.0262076},
{-0.0183097,-0.3964755,0.0340043},
{-0.0081247,-0.0552147,0.0496913},
{-0.0107507,-0.3911153,-0.0097891},
{0.0100197,-0.0428073,0.0213294},
{0.0101833,0.0143680,-0.0106590},
{-0.0228617,-0.3878880,0.0275144},
{0.0146773,-0.0525892,0.0333508},

Biology Open • Supplementary information

{0.0002799,-0.0490384,0.0569970},
{-0.0103028,0.0179044,0.0027083},
{-0.0023145,-0.0165239,0.0625655},
{-0.0068423,-0.0072924,0.0229617},
{0.0181761,-0.0101977,0.0401910},
{0.0139195,-0.0242363,0.0544500},
{-0.0132542,-0.0107932,0.0495389},
{0.0185773,0.0081501,-0.0026720},
{-0.0093966,-0.0337627,0.0170648},
{-0.0003157,-0.0139759,-0.0156472},
{-0.0100999,-0.0596023,0.0420391},
{-0.0084399,0.0169458,0.0076329},
{0.0091490,0.0189426,-0.0028563},
{-0.0125547,-0.0267563,0.0542509},
{-0.0056052,-0.3883713,0.0385439},
{-0.0125872,-0.3936516,-0.0361259},
{-0.0125174,-0.3935988,-0.0353792},
{-0.0124476,-0.3935460,-0.0346325},
{-0.0123778,-0.3934932,-0.0338858},
{-0.0123079,-0.3934404,-0.0331391},
{-0.0122381,-0.3933876,-0.0323924},
{-0.0121683,-0.3933348,-0.0316458},
{-0.0120985,-0.3932820,-0.0308991},
{-0.0120287,-0.3932292,-0.0301524},
{-0.0119588,-0.3931764,-0.0294057},
{-0.0118890,-0.3931235,-0.0286590},
{-0.0118192,-0.3930707,-0.0279123},
{-0.0117494,-0.3930179,-0.0271656},
{-0.0116795,-0.3929651,-0.0264189},
{-0.0116097,-0.3929123,-0.0256722},
{-0.0115399,-0.3928595,-0.0249255},
{-0.0114701,-0.3928067,-0.0241788},
{-0.0114003,-0.3927539,-0.0234321},
{-0.0113304,-0.3927011,-0.0226854},
{-0.0112606,-0.3926483,-0.0219387},
{-0.0111908,-0.3925955,-0.0211920},
{-0.0111210,-0.3925427,-0.0204453},
{-0.0110512,-0.3924899,-0.0196986},
{-0.0109813,-0.3924371,-0.0189519},
{-0.0109115,-0.3923843,-0.0182052},
{-0.0108417,-0.3923315,-0.0174585},
{-0.0107719,-0.3922787,-0.0167118},
{-0.0107021,-0.3922259,-0.0159651},
{-0.0106322,-0.3921731,-0.0152184},
{-0.0105624,-0.3921203,-0.0144717},
{-0.0104926,-0.3920675,-0.0137250},

{-0.0104228,-0.3920147,-0.0129783},
{-0.0103530,-0.3919619,-0.0122316},
{-0.0102831,-0.3919091,-0.0114849},
{-0.0102133,-0.3918563,-0.0107382},
{-0.0101435,-0.3918035,-0.0099915},
{-0.0100737,-0.3917507,-0.0092448},
{-0.0100039,-0.3916979,-0.0084981},
{-0.0099340,-0.3916451,-0.0077514},
{-0.0098642,-0.3915923,-0.0070047},
{-0.0097944,-0.3915395,-0.0062580},
{-0.0097246,-0.3914867,-0.0055113},
{-0.0096548,-0.3914339,-0.0047646},
{-0.0095849,-0.3913811,-0.0040179},
{-0.0095151,-0.3913283,-0.0032712},
{-0.0094453,-0.3912755,-0.0025245},
{-0.0093755,-0.3912226,-0.0017778},
{-0.0093057,-0.3911698,-0.0010311},
{-0.0092358,-0.3911170,-0.0002844},
{-0.0091660,-0.3910642,0.0004623},
{-0.0090962,-0.3910114,0.0012090},
{-0.0090264,-0.3909586,0.0019557},
{-0.0089566,-0.3909058,0.0027024},
{-0.0088867,-0.3908530,0.0034491},
{-0.0088169,-0.3908002,0.0041958},
{-0.0087471,-0.3907474,0.0049425},
{-0.0086773,-0.3906946,0.0056892},
{-0.0086075,-0.3906418,0.0064359},
{-0.0085376,-0.3905890,0.0071826},
{-0.0084678,-0.3905362,0.0079293},
{-0.0083980,-0.3904834,0.0086760},
{-0.0083282,-0.3904306,0.0094227},
{-0.0082584,-0.3903778,0.0101694},
{-0.0081885,-0.3903250,0.0109161},
{-0.0081187,-0.3902722,0.0116628},
{-0.0080489,-0.3902194,0.0124095},
{-0.0079791,-0.3901666,0.0131562},
{-0.0079093,-0.3901138,0.0139029},
{-0.0078394,-0.3900610,0.0146496},
{-0.0077696,-0.3900082,0.0153963},
{-0.0076998,-0.3899554,0.0161429},
{-0.0076300,-0.3899026,0.0168896},
{-0.0075602,-0.3898498,0.0176363},
{-0.0074903,-0.3897970,0.0183830},
{-0.0074205,-0.3897442,0.0191297},
{-0.0073507,-0.3896914,0.0198764},
{-0.0072809,-0.3896386,0.0206231},

Biology Open • Supplementary information

```
                    {-0.0072111,-0.3895858,0.0213698},
                    {-0.0071412,-0.3895330,0.0221165},
                    {-0.0070714,-0.3894802,0.0228632},
                    {-0.0070016,-0.3894274,0.0236099},
                    {-0.0069318,-0.3893746,0.0243566},
                    {-0.0068619,-0.3893217,0.0251033},
                    {-0.0067921,-0.3892689,0.0258500},
                    {-0.0067223,-0.3892161,0.0265967},
                    {-0.0066525,-0.3891633,0.0273434},
                    {-0.0065827,-0.3891105,0.0280901},
                    {-0.0065128,-0.3890577,0.0288368},
                    {-0.0064430,-0.3890049,0.0295835},
                    {-0.0063732,-0.3889521,0.0303302},
                    {-0.0063034,-0.3888993,0.0310769},
                    {-0.0062336,-0.3888465,0.0318236},
                    {-0.0061637,-0.3887937,0.0325703},
                    {-0.0060939,-0.3887409,0.0333170},
                    {-0.0060241,-0.3886881,0.0340637},
                    {-0.0059543,-0.3886353,0.0348104},
                    {-0.0058845,-0.3885825,0.0355571},
                    {-0.0058146,-0.3885297,0.0363038},
                    {-0.0057448,-0.3884769,0.0370505},
                    {-0.0056750,-0.3884241,0.0377972}
                    };
            BoundingBoxOnOff = Off;
        };
        AnyFunTransform3DIdentity ScaleFunction = {
            PreTransforms = {&.RBFTransform};
};
};
};

};
```

Biology Open • Supplementary information

ScalingFunctionTLEMLucyPelvis_2014001

```
// This file defines a new scaling function to make the pelvis (os cox) like Lucy's using
TPS

AnyFolder ScalingFunctionTLEMLucyPelvis = {
 AnyFolder Pelvis = {     // this is really the Os Cox only
// this transforms pelvis
   AnyFunTransform3DRBF RBFTransform = {
     RBFDef.Type = RBF_ThinPlate;
     PolynomDegree = 1;
     Points0 = {  // TLEM2
       {0.0000000,0.0000000,0.1177000},
       {-0.0000000,-0.0832729,0.0191000},
       {-0.0508179,-0.0694062,0.0815920},
       {0.0000000,0.0000000,-0.1177000},
       {-0.0000000,-0.0832729,-0.0191000},
       {-0.0508179,-0.0694062,-0.0815920},
       {0.0000000,0.0000000,0.0000000},
       {-0.1164020,-0.0039449,0.0456760},
       {-0.1164020,-0.0039449,-0.0456760},
       {-0.1092909,-0.0992949,0.0515590},
       {-0.1092909,-0.0992949,-0.0515590},
       {-0.0733749,0.0757445,0.0915590},
       {-0.0733749,0.0757445,-0.0915590},
       {-0.1241738,0.0316243,0.0458050},
       {-0.1241738,0.0316243,-0.0458050},
       {-0.1108008,0.0563490,0.0555690},
       {-0.1108008,0.0563490,-0.0555690},
       {-0.0545356,0.0481653,0.1291690},
       {-0.0545356,0.0481653,-0.1291690},
       {-0.0360236,0.0456352,0.1316250},
       {-0.0360236,0.0456352,-0.1316250},
       {-0.0804664,-0.0228481,0.0672140},
       {-0.0804664,-0.0228481,-0.0672140},
       {-0.1004070,-0.0623213,0.0494990},
       {-0.1004070,-0.0623213,-0.0494990},
       {-0.0174901,-0.0357786,0.0977400},
       {-0.0174901,-0.0357786,-0.0977400},
       {-0.0665950,-0.1308418,0.0278190},
       {-0.0665950,-0.1308418,-0.0278190},
       {-0.0962739,-0.1257274,0.0551670},
       {-0.0962739,-0.1257274,-0.0551670},
       {-0.0393340,-0.1196098,0.0159820},
       {-0.0393340,-0.1196098,-0.0159820},
       {-0.0585979,-0.0148191,0.0651020},
```

Biology Open • Supplementary information

{-0.0585979,-0.0148191,-0.0651020},
{-0.0238420,-0.0800479,0.0351680},
{-0.0238420,-0.0800479,-0.0351680},
{-0.0234553,-0.0576924,0.0715330},
{-0.0234553,-0.0576924,-0.0715330},
{-0.0472319,-0.0833276,0.0513790},
{-0.0472319,-0.0833276,-0.0513790},
{-0.0327175,-0.0506481,0.0520240},
{-0.0327175,-0.0506481,-0.0520240},
{-0.0746024,-0.0627219,0.0928110},
{-0.0746024,-0.0627219,-0.0928110},
{-0.0119676,-0.0928080,0.0076900},
{-0.0119676,-0.0928080,-0.0076900},
{-0.0263299,-0.0760728,0.0667080},
{-0.0263299,-0.0760728,-0.0667080},
{-0.0498842,0.0119450,0.1070630},
{-0.0498842,0.0119450,-0.1070630},
{-0.0553874,0.0627474,0.1172390},
{-0.0553874,0.0627474,-0.1172390},
{-0.0786774,0.0073556,0.0741800},
{-0.0786774,0.0073556,-0.0741800},
{-0.0742762,0.0474272,0.0542330},
{-0.0742762,0.0474272,-0.0542330},
{-0.0634121,0.0117185,0.0618430},
{-0.0634121,0.0117185,-0.0618430},
{-0.0813084,0.0007821,0.0508130},
{-0.0813084,0.0007821,-0.0508130},
{-0.0496423,-0.0444458,0.0983920},
{-0.0496423,-0.0444458,-0.0983920},
{-0.0480005,-0.0338999,0.0625080},
{-0.0480005,-0.0338999,-0.0625080},
{-0.0428000,-0.0273188,0.0973490},
{-0.0428000,-0.0273188,-0.0973490},
{-0.0548383,-0.0518766,0.0624050},
{-0.0548383,-0.0518766,-0.0624050},
{-0.0696448,-0.1052827,0.0436200},
{-0.0696448,-0.1052827,-0.0436200},
{-0.0739608,-0.0803177,0.0785950},
{-0.0739608,-0.0803177,-0.0785950},
{-0.0586066,-0.0884764,0.0653190},
{-0.0586066,-0.0884764,-0.0653190},
{-0.1017003,0.0085215,0.0624150},
{-0.1017003,0.0085215,-0.0624150},
{-0.0126252,0.0252852,0.1292530},
{-0.0126252,0.0252852,-0.1292530},
{-0.0030963,0.0150156,0.1262640},

Biology Open • Supplementary information

{-0.0030963,0.0150156,-0.1262640},
{-0.0621797,0.0048743,0.0605920},
{-0.0621797,0.0048743,-0.0605920},
{-0.0870082,-0.0684280,0.0489900},
{-0.0870082,-0.0684280,-0.0489900},
{-0.0916211,-0.0478344,0.0575290},
{-0.0916211,-0.0478344,-0.0575290},
{-0.0428573,-0.0467026,0.0985250},
{-0.0428573,-0.0467026,-0.0985250},
{-0.0483869,-0.0455474,0.0848580},
{-0.0483869,-0.0455474,-0.0848580},
{-0.0203929,-0.0681811,0.0426070},
{-0.0203929,-0.0681811,-0.0426070},
{-0.0271973,-0.0807786,0.0491650},
{-0.0271973,-0.0807786,-0.0491650},
{-0.0325935,-0.1026813,0.0148280},
{-0.0325935,-0.1026813,-0.0148280},
{-0.0273900,-0.1059173,0.0048750},
{-0.0273900,-0.1059173,-0.0048750},
{-0.0015971,-0.0778770,0.0065530},
{-0.0015971,-0.0778770,-0.0065530},
{-0.1034983,-0.0140026,0.0521800},
{-0.1034983,-0.0140026,-0.0521800},
{-0.1141160,0.0077734,0.0497600},
{-0.1141160,0.0077734,-0.0497600},
{-0.1123797,0.0244808,0.0350160},
{-0.1123797,0.0244808,-0.0350160},
{-0.0984470,-0.0648210,0.0457100},
{-0.0984470,-0.0648210,-0.0457100},
{-0.0814332,-0.0704479,0.0806410},
{-0.0814332,-0.0704479,-0.0806410},
{-0.0540218,-0.0805070,0.0547320},
{-0.0540218,-0.0805070,-0.0547320},
{-0.0674356,-0.0763171,0.0651080},
{-0.0674356,-0.0763171,-0.0651080},
{-0.0602364,-0.0751827,0.0490600},
{-0.0602364,-0.0751827,-0.0490600},
{-0.0604272,-0.0801096,0.0617610},
{-0.0604272,-0.0801096,-0.0617610},
{-0.0655251,0.0632155,0.1093750},
{-0.0655251,0.0632155,-0.1093750},
{-0.0665046,0.0014289,0.0572170},
{-0.0665046,0.0014289,-0.0572170},
{-0.1053786,0.0347803,0.0071580},
{-0.1053786,0.0347803,-0.0071580},
{-0.1321649,-0.0069725,0.0058340},

Biology Open • Supplementary information

```
      {-0.1321649,-0.0069725,-0.0058340},
      {-0.1232735,-0.0434903,0.0301110},
      {-0.1232735,-0.0434903,-0.0301110},
      {-0.0497024,0.0261862,0.0084640},
      {-0.0497024,0.0261862,-0.0084640},
      {-0.0887827,0.0446048,0.0327030},
      {-0.0887827,0.0446048,-0.0327030},
      {-0.1153998,-0.0126010,0.0290730},
      {-0.1153998,-0.0126010,-0.0290730},
      {-0.0899939,0.0093882,0.0208440},
      {-0.0899939,0.0093882,-0.0208440},
      {-0.1312654,-0.0574898,0.0094690},
      {-0.1312654,-0.0574898,-0.0094690},
      {-0.0996029,0.0042806,0.0105620},
      {-0.0996029,0.0042806,-0.0105620},
      {-0.1226177,-0.0324069,0.0331630},
      {-0.1226177,-0.0324069,-0.0331630},
      {-0.1097808,-0.0051619,0.0102520},
      {-0.1097808,-0.0051619,-0.0102520}
    }; // close TLEM2 pelvis
//
//
//
    Points1 = { //Lucy pelvis
      {0.0000000,0.0000000,0.1222020},
      {-0.0000007,-0.0897023,0.0177942},
      {-0.0306432,-0.0696562,0.0850392},
      {0.0000000,0.0000000,-0.1222020},
      {-0.0000007,-0.0897023,-0.0177942},
      {-0.0306432,-0.0696562,-0.0850392},
      {0.0000000,0.0000000,0.0000000},
      {-0.0836701,-0.0292935,0.0413940},
      {-0.0836701,-0.0292935,-0.0413940},
      {-0.0793062,-0.0985156,0.0604380},
      {-0.0793062,-0.0985156,-0.0604380},
      {-0.0544464,0.0411989,0.0891260},
      {-0.0544464,0.0411989,-0.0891260},
      {-0.0883311,-0.0073389,0.0367000},
      {-0.0883311,-0.0073389,-0.0367000},
      {-0.0801836,0.0154316,0.0487290},
      {-0.0801836,0.0154316,-0.0487290},
      {-0.0336129,0.0316222,0.1265380},
      {-0.0336129,0.0316222,-0.1265380},
      {-0.0219049,0.0319415,0.1306920},
      {-0.0219049,0.0319415,-0.1306920},
      {-0.0592161,-0.0397469,0.0670960},
```

{-0.0592161,-0.0397469,-0.0670960},
{-0.0739849,-0.0726584,0.0620670},
{-0.0739849,-0.0726584,-0.0620670},
{-0.0110254,-0.0389470,0.0917450},
{-0.0110254,-0.0389470,-0.0917450},
{-0.0512431,-0.1240646,0.0333550},
{-0.0512431,-0.1240646,-0.0333550},
{-0.0693210,-0.1150097,0.0635600},
{-0.0693210,-0.1150097,-0.0635600},
{-0.0288298,-0.1188939,0.0186090},
{-0.0288298,-0.1188939,-0.0186090},
{-0.0460759,-0.0283271,0.0648310},
{-0.0460759,-0.0283271,-0.0648310},
{-0.0146493,-0.0817899,0.0336120},
{-0.0146493,-0.0817899,-0.0336120},
{-0.0154162,-0.0581374,0.0720620},
{-0.0154162,-0.0581374,-0.0720620},
{-0.0286215,-0.0791708,0.0552910},
{-0.0286215,-0.0791708,-0.0552910},
{-0.0239598,-0.0583625,0.0560800},
{-0.0239598,-0.0583625,-0.0560800},
{-0.0485834,-0.0653347,0.0894420},
{-0.0485834,-0.0653347,-0.0894420},
{-0.0070087,-0.0995157,0.0076400},
{-0.0070087,-0.0995157,-0.0076400},
{-0.0163814,-0.0700533,0.0656520},
{-0.0163814,-0.0700533,-0.0656520},
{-0.0354855,-0.0009839,0.1040780},
{-0.0354855,-0.0009839,-0.1040780},
{-0.0378749,0.0393254,0.1154470},
{-0.0378749,0.0393254,-0.1154470},
{-0.0601633,-0.0141240,0.0679980},
{-0.0601633,-0.0141240,-0.0679980},
{-0.0576172,0.0138742,0.0490020},
{-0.0576172,0.0138742,-0.0490020},
{-0.0523150,-0.0101405,0.0551350},
{-0.0523150,-0.0101405,-0.0551350},
{-0.0629772,-0.0228622,0.0457770},
{-0.0629772,-0.0228622,-0.0457770},
{-0.0305010,-0.0500503,0.0925250},
{-0.0305010,-0.0500503,-0.0925250},
{-0.0351435,-0.0430796,0.0661220},
{-0.0351435,-0.0430796,-0.0661220},
{-0.0283750,-0.0345951,0.0928870},
{-0.0283750,-0.0345951,-0.0928870},
{-0.0363077,-0.0579162,0.0677520},

Biology Open • Supplementary information

{-0.0363077,-0.0579162,-0.0677520},
{-0.0490871,-0.1016305,0.0519400},
{-0.0490871,-0.1016305,-0.0519400},
{-0.0478107,-0.0798804,0.0813570},
{-0.0478107,-0.0798804,-0.0813570},
{-0.0364541,-0.0869022,0.0725310},
{-0.0364541,-0.0869022,-0.0725310},
{-0.0745482,-0.0170946,0.0563840},
{-0.0745482,-0.0170946,-0.0563840},
{-0.0072941,0.0188256,0.1310250},
{-0.0072941,0.0188256,-0.1310250},
{-0.0013107,0.0118697,0.1294130},
{-0.0013107,0.0118697,-0.1294130},
{-0.0520579,-0.0152095,0.0537840},
{-0.0520579,-0.0152095,-0.0537840},
{-0.0622348,-0.0753052,0.0616540},
{-0.0622348,-0.0753052,-0.0616540},
{-0.0660401,-0.0602447,0.0644660},
{-0.0660401,-0.0602447,-0.0644660},
{-0.0251694,-0.0519123,0.0920780},
{-0.0251694,-0.0519123,-0.0920780},
{-0.0308165,-0.0511667,0.0827500},
{-0.0308165,-0.0511667,-0.0827500},
{-0.0130046,-0.0724270,0.0407240},
{-0.0130046,-0.0724270,-0.0407240},
{-0.0165627,-0.0790326,0.0482520},
{-0.0165627,-0.0790326,-0.0482520},
{-0.0207273,-0.1051347,0.0169540},
{-0.0207273,-0.1051347,-0.0169540},
{-0.0153861,-0.1084472,0.0066230},
{-0.0153861,-0.1084472,-0.0066230},
{-0.0017811,-0.0884332,0.0061460},
{-0.0017811,-0.0884332,-0.0061460},
{-0.0748300,-0.0350506,0.0514430},
{-0.0748300,-0.0350506,-0.0514430},
{-0.0823780,-0.0193239,0.0436120},
{-0.0823780,-0.0193239,-0.0436120},
{-0.0808758,-0.0116090,0.0280400},
{-0.0808758,-0.0116090,-0.0280400},
{-0.0729528,-0.0745319,0.0607570},
{-0.0729528,-0.0745319,-0.0607570},
{-0.0541558,-0.0729948,0.0828040},
{-0.0541558,-0.0729948,-0.0828040},
{-0.0324080,-0.0761442,0.0600410},
{-0.0324080,-0.0761442,-0.0600410},
{-0.0430705,-0.0778645,0.0724180},

Biology Open • Supplementary information

```
        {-0.0430705,-0.0778645,-0.0724180},
        {-0.0385211,-0.0758172,0.0565930},
        {-0.0385211,-0.0758172,-0.0565930},
        {-0.0373163,-0.0805468,0.0698570},
        {-0.0373163,-0.0805468,-0.0698570},
        {-0.0469093,0.0359819,0.1072450},
        {-0.0469093,0.0359819,-0.1072450},
        {-0.0553559,-0.0190318,0.0499600},
        {-0.0553559,-0.0190318,-0.0499600},
        {-0.0765620,-0.0060170,0.0056350},
        {-0.0765620,-0.0060170,-0.0056350},
        {-0.0935720,-0.0379860,0.0044840},
        {-0.0935720,-0.0379860,-0.0044840},
        {-0.0926470,-0.0657640,0.0210500},
        {-0.0926470,-0.0657640,-0.0210500},
        {-0.0417650,-0.0068750,0.0076590},
        {-0.0417650,-0.0068750,-0.0076590},
        {-0.0669880,0.0061730,0.0277520},
        {-0.0669880,0.0061730,-0.0277520},
        {-0.0829880,-0.0395410,0.0253720},
        {-0.0829880,-0.0395410,-0.0253720},
        {-0.0669130,-0.0228660,0.0179600},
        {-0.0669130,-0.0228660,-0.0179600},
        {-0.0987550,-0.0769490,0.0065790},
        {-0.0987550,-0.0769490,-0.0065790},
        {-0.0712530,-0.0293620,0.0090120},
        {-0.0712530,-0.0293620,-0.0090120},
        {-0.0904950,-0.0559600,0.0267990},
        {-0.0904950,-0.0559600,-0.0267990},
        {-0.0769520,-0.0372560,0.0086350},
        {-0.0769520,-0.0372560,-0.0086350}
      }; // close Lucy
      BoundingBoxOnOff = Off;
    };
// this is the identity transform and is needed because the pelvis is unilateral (doesn't
need a mirrored transform)
    AnyFunTransform3DIdentity ScaleFunction = {
      PreTransforms = {&.RBFTransform};
      };
  }; // close pelvis (Os Cox) folder

//
//
//
 AnyFolder Sacrum = {     // this is the sacrum only
// this transforms the sacrum
```

Biology Open • Supplementary information

```
AnyFunTransform3DRBF RBFTransform = {
  RBFDef.Type = RBF_ThinPlate;
  PolynomDegree = 1;
  Points0 = {   // TLEM2
    {0.0000000,0.0000000,0.1177000},
    {-0.0000000,-0.0832729,0.0191000},
    {-0.0508179,-0.0694062,0.0815920},
    {0.0000000,0.0000000,-0.1177000},
    {-0.0000000,-0.0832729,-0.0191000},
    {-0.0508179,-0.0694062,-0.0815920},
    {0.0000000,0.0000000,0.0000000},
    {-0.1164020,-0.0039449,0.0456760},
    {-0.1164020,-0.0039449,-0.0456760},
    {-0.1092909,-0.0992949,0.0515590},
    {-0.1092909,-0.0992949,-0.0515590},
    {-0.0733749,0.0757445,0.0915590},
    {-0.0733749,0.0757445,-0.0915590},
    {-0.1241738,0.0316243,0.0458050},
    {-0.1241738,0.0316243,-0.0458050},
    {-0.1108008,0.0563490,0.0555690},
    {-0.1108008,0.0563490,-0.0555690},
    {-0.0545356,0.0481653,0.1291690},
    {-0.0545356,0.0481653,-0.1291690},
    {-0.0360236,0.0456352,0.1316250},
    {-0.0360236,0.0456352,-0.1316250},
    {-0.0804664,-0.0228481,0.0672140},
    {-0.0804664,-0.0228481,-0.0672140},
    {-0.1004070,-0.0623213,0.0494990},
    {-0.1004070,-0.0623213,-0.0494990},
    {-0.0174901,-0.0357786,0.0977400},
    {-0.0174901,-0.0357786,-0.0977400},
    {-0.0665950,-0.1308418,0.0278190},
    {-0.0665950,-0.1308418,-0.0278190},
    {-0.0962739,-0.1257274,0.0551670},
    {-0.0962739,-0.1257274,-0.0551670},
    {-0.0393340,-0.1196098,0.0159820},
    {-0.0393340,-0.1196098,-0.0159820},
    {-0.0585979,-0.0148191,0.0651020},
    {-0.0585979,-0.0148191,-0.0651020},
    {-0.0238420,-0.0800479,0.0351680},
    {-0.0238420,-0.0800479,-0.0351680},
    {-0.0234553,-0.0576924,0.0715330},
    {-0.0234553,-0.0576924,-0.0715330},
    {-0.0472319,-0.0833276,0.0513790},
    {-0.0472319,-0.0833276,-0.0513790},
    {-0.0327175,-0.0506481,0.0520240},
```

Biology Open • Supplementary information

{-0.0327175,-0.0506481,-0.0520240},
{-0.0746024,-0.0627219,0.0928110},
{-0.0746024,-0.0627219,-0.0928110},
{-0.0119676,-0.0928080,0.0076900},
{-0.0119676,-0.0928080,-0.0076900},
{-0.0263299,-0.0760728,0.0667080},
{-0.0263299,-0.0760728,-0.0667080},
{-0.0498842,0.0119450,0.1070630},
{-0.0498842,0.0119450,-0.1070630},
{-0.0553874,0.0627474,0.1172390},
{-0.0553874,0.0627474,-0.1172390},
{-0.0786774,0.0073556,0.0741800},
{-0.0786774,0.0073556,-0.0741800},
{-0.0742762,0.0474272,0.0542330},
{-0.0742762,0.0474272,-0.0542330},
{-0.0634121,0.0117185,0.0618430},
{-0.0634121,0.0117185,-0.0618430},
{-0.0813084,0.0007821,0.0508130},
{-0.0813084,0.0007821,-0.0508130},
{-0.0496423,-0.0444458,0.0983920},
{-0.0496423,-0.0444458,-0.0983920},
{-0.0480005,-0.0338999,0.0625080},
{-0.0480005,-0.0338999,-0.0625080},
{-0.0428000,-0.0273188,0.0973490},
{-0.0428000,-0.0273188,-0.0973490},
{-0.0548383,-0.0518766,0.0624050},
{-0.0548383,-0.0518766,-0.0624050},
{-0.0696448,-0.1052827,0.0436200},
{-0.0696448,-0.1052827,-0.0436200},
{-0.0739608,-0.0803177,0.0785950},
{-0.0739608,-0.0803177,-0.0785950},
{-0.0586066,-0.0884764,0.0653190},
{-0.0586066,-0.0884764,-0.0653190},
{-0.1017003,0.0085215,0.0624150},
{-0.1017003,0.0085215,-0.0624150},
{-0.0126252,0.0252852,0.1292530},
{-0.0126252,0.0252852,-0.1292530},
{-0.0030963,0.0150156,0.1262640},
{-0.0030963,0.0150156,-0.1262640},
{-0.0621797,0.0048743,0.0605920},
{-0.0621797,0.0048743,-0.0605920},
{-0.0870082,-0.0684280,0.0489900},
{-0.0870082,-0.0684280,-0.0489900},
{-0.0916211,-0.0478344,0.0575290},
{-0.0916211,-0.0478344,-0.0575290},
{-0.0428573,-0.0467026,0.0985250},

Biology Open • Supplementary information

{-0.0428573,-0.0467026,-0.0985250},
{-0.0483869,-0.0455474,0.0848580},
{-0.0483869,-0.0455474,-0.0848580},
{-0.0203929,-0.0681811,0.0426070},
{-0.0203929,-0.0681811,-0.0426070},
{-0.0271973,-0.0807786,0.0491650},
{-0.0271973,-0.0807786,-0.0491650},
{-0.0325935,-0.1026813,0.0148280},
{-0.0325935,-0.1026813,-0.0148280},
{-0.0273900,-0.1059173,0.0048750},
{-0.0273900,-0.1059173,-0.0048750},
{-0.0015971,-0.0778770,0.0065530},
{-0.0015971,-0.0778770,-0.0065530},
{-0.1034983,-0.0140026,0.0521800},
{-0.1034983,-0.0140026,-0.0521800},
{-0.1141160,0.0077734,0.0497600},
{-0.1141160,0.0077734,-0.0497600},
{-0.1123797,0.0244808,0.0350160},
{-0.1123797,0.0244808,-0.0350160},
{-0.0984470,-0.0648210,0.0457100},
{-0.0984470,-0.0648210,-0.0457100},
{-0.0814332,-0.0704479,0.0806410},
{-0.0814332,-0.0704479,-0.0806410},
{-0.0540218,-0.0805070,0.0547320},
{-0.0540218,-0.0805070,-0.0547320},
{-0.0674356,-0.0763171,0.0651080},
{-0.0674356,-0.0763171,-0.0651080},
{-0.0602364,-0.0751827,0.0490600},
{-0.0602364,-0.0751827,-0.0490600},
{-0.0604272,-0.0801096,0.0617610},
{-0.0604272,-0.0801096,-0.0617610},
{-0.0655251,0.0632155,0.1093750},
{-0.0655251,0.0632155,-0.1093750},
{-0.0665046,0.0014289,0.0572170},
{-0.0665046,0.0014289,-0.0572170},
{-0.1053786,0.0347803,0.0071580},
{-0.1053786,0.0347803,-0.0071580},
{-0.1321649,-0.0069725,0.0058340},
{-0.1321649,-0.0069725,-0.0058340},
{-0.1232735,-0.0434903,0.0301110},
{-0.1232735,-0.0434903,-0.0301110},
{-0.0497024,0.0261862,0.0084640},
{-0.0497024,0.0261862,-0.0084640},
{-0.0887827,0.0446048,0.0327030},
{-0.0887827,0.0446048,-0.0327030},
{-0.1153998,-0.0126010,0.0290730},

Biology Open • Supplementary information

```
        {-0.1153998,-0.0126010,-0.0290730},
        {-0.0899939,0.0093882,0.0208440},
        {-0.0899939,0.0093882,-0.0208440},
        {-0.1312654,-0.0574898,0.0094690},
        {-0.1312654,-0.0574898,-0.0094690},
        {-0.0996029,0.0042806,0.0105620},
        {-0.0996029,0.0042806,-0.0105620},
        {-0.1226177,-0.0324069,0.0331630},
        {-0.1226177,-0.0324069,-0.0331630},
        {-0.1097808,-0.0051619,0.0102520},
        {-0.1097808,-0.0051619,-0.0102520}
    }; // close TLEM2
//
    Points1 = {    // Lucy
        {0.0000000,0.0000000,0.1222020},
        {-0.0000007,-0.0897023,0.0177942},
        {-0.0306432,-0.0696562,0.0850392},
        {0.0000000,0.0000000,-0.1222020},
        {-0.0000007,-0.0897023,-0.0177942},
        {-0.0306432,-0.0696562,-0.0850392},
        {0.0000000,0.0000000,0.0000000},
        {-0.0836701,-0.0292935,0.0413940},
        {-0.0836701,-0.0292935,-0.0413940},
        {-0.0793062,-0.0985156,0.0604380},
        {-0.0793062,-0.0985156,-0.0604380},
        {-0.0544464,0.0411989,0.0891260},
        {-0.0544464,0.0411989,-0.0891260},
        {-0.0883311,-0.0073389,0.0367000},
        {-0.0883311,-0.0073389,-0.0367000},
        {-0.0801836,0.0154316,0.0487290},
        {-0.0801836,0.0154316,-0.0487290},
        {-0.0336129,0.0316222,0.1265380},
        {-0.0336129,0.0316222,-0.1265380},
        {-0.0219049,0.0319415,0.1306920},
        {-0.0219049,0.0319415,-0.1306920},
        {-0.0592161,-0.0397469,0.0670960},
        {-0.0592161,-0.0397469,-0.0670960},
        {-0.0739849,-0.0726584,0.0620670},
        {-0.0739849,-0.0726584,-0.0620670},
        {-0.0110254,-0.0389470,0.0917450},
        {-0.0110254,-0.0389470,-0.0917450},
        {-0.0512431,-0.1240646,0.0333550},
        {-0.0512431,-0.1240646,-0.0333550},
        {-0.0693210,-0.1150097,0.0635600},
        {-0.0693210,-0.1150097,-0.0635600},
        {-0.0288298,-0.1188939,0.0186090},
```

Biology Open • Supplementary information

{-0.0288298,-0.1188939,-0.0186090},
{-0.0460759,-0.0283271,0.0648310},
{-0.0460759,-0.0283271,-0.0648310},
{-0.0146493,-0.0817899,0.0336120},
{-0.0146493,-0.0817899,-0.0336120},
{-0.0154162,-0.0581374,0.0720620},
{-0.0154162,-0.0581374,-0.0720620},
{-0.0286215,-0.0791708,0.0552910},
{-0.0286215,-0.0791708,-0.0552910},
{-0.0239598,-0.0583625,0.0560800},
{-0.0239598,-0.0583625,-0.0560800},
{-0.0485834,-0.0653347,0.0894420},
{-0.0485834,-0.0653347,-0.0894420},
{-0.0070087,-0.0995157,0.0076400},
{-0.0070087,-0.0995157,-0.0076400},
{-0.0163814,-0.0700533,0.0656520},
{-0.0163814,-0.0700533,-0.0656520},
{-0.0354855,-0.0009839,0.1040780},
{-0.0354855,-0.0009839,-0.1040780},
{-0.0378749,0.0393254,0.1154470},
{-0.0378749,0.0393254,-0.1154470},
{-0.0601633,-0.0141240,0.0679980},
{-0.0601633,-0.0141240,-0.0679980},
{-0.0576172,0.0138742,0.0490020},
{-0.0576172,0.0138742,-0.0490020},
{-0.0523150,-0.0101405,0.0551350},
{-0.0523150,-0.0101405,-0.0551350},
{-0.0629772,-0.0228622,0.0457770},
{-0.0629772,-0.0228622,-0.0457770},
{-0.0305010,-0.0500503,0.0925250},
{-0.0305010,-0.0500503,-0.0925250},
{-0.0351435,-0.0430796,0.0661220},
{-0.0351435,-0.0430796,-0.0661220},
{-0.0283750,-0.0345951,0.0928870},
{-0.0283750,-0.0345951,-0.0928870},
{-0.0363077,-0.0579162,0.0677520},
{-0.0363077,-0.0579162,-0.0677520},
{-0.0490871,-0.1016305,0.0519400},
{-0.0490871,-0.1016305,-0.0519400},
{-0.0478107,-0.0798804,0.0813570},
{-0.0478107,-0.0798804,-0.0813570},
{-0.0364541,-0.0869022,0.0725310},
{-0.0364541,-0.0869022,-0.0725310},
{-0.0745482,-0.0170946,0.0563840},
{-0.0745482,-0.0170946,-0.0563840},
{-0.0072941,0.0188256,0.1310250},

Biology Open • Supplementary information

{-0.0072941,0.0188256,-0.1310250},
{-0.0013107,0.0118697,0.1294130},
{-0.0013107,0.0118697,-0.1294130},
{-0.0520579,-0.0152095,0.0537840},
{-0.0520579,-0.0152095,-0.0537840},
{-0.0622348,-0.0753052,0.0616540},
{-0.0622348,-0.0753052,-0.0616540},
{-0.0660401,-0.0602447,0.0644660},
{-0.0660401,-0.0602447,-0.0644660},
{-0.0251694,-0.0519123,0.0920780},
{-0.0251694,-0.0519123,-0.0920780},
{-0.0308165,-0.0511667,0.0827500},
{-0.0308165,-0.0511667,-0.0827500},
{-0.0130046,-0.0724270,0.0407240},
{-0.0130046,-0.0724270,-0.0407240},
{-0.0165627,-0.0790326,0.0482520},
{-0.0165627,-0.0790326,-0.0482520},
{-0.0207273,-0.1051347,0.0169540},
{-0.0207273,-0.1051347,-0.0169540},
{-0.0153861,-0.1084472,0.0066230},
{-0.0153861,-0.1084472,-0.0066230},
{-0.0017811,-0.0884332,0.0061460},
{-0.0017811,-0.0884332,-0.0061460},
{-0.0748300,-0.0350506,0.0514430},
{-0.0748300,-0.0350506,-0.0514430},
{-0.0823780,-0.0193239,0.0436120},
{-0.0823780,-0.0193239,-0.0436120},
{-0.0808758,-0.0116090,0.0280400},
{-0.0808758,-0.0116090,-0.0280400},
{-0.0729528,-0.0745319,0.0607570},
{-0.0729528,-0.0745319,-0.0607570},
{-0.0541558,-0.0729948,0.0828040},
{-0.0541558,-0.0729948,-0.0828040},
{-0.0324080,-0.0761442,0.0600410},
{-0.0324080,-0.0761442,-0.0600410},
{-0.0430705,-0.0778645,0.0724180},
{-0.0430705,-0.0778645,-0.0724180},
{-0.0385211,-0.0758172,0.0565930},
{-0.0385211,-0.0758172,-0.0565930},
{-0.0373163,-0.0805468,0.0698570},
{-0.0373163,-0.0805468,-0.0698570},
{-0.0469093,0.0359819,0.1072450},
{-0.0469093,0.0359819,-0.1072450},
{-0.0553559,-0.0190318,0.0499600},
{-0.0553559,-0.0190318,-0.0499600},
{-0.0765620,-0.0060170,0.0056350},

Biology Open • Supplementary information

```
        {-0.0765620,-0.0060170,-0.0056350},
        {-0.0935720,-0.0379860,0.0044840},
        {-0.0935720,-0.0379860,-0.0044840},
        {-0.0926470,-0.0657640,0.0210500},
        {-0.0926470,-0.0657640,-0.0210500},
        {-0.0417650,-0.0068750,0.0076590},
        {-0.0417650,-0.0068750,-0.0076590},
        {-0.0669880,0.0061730,0.0277520},
        {-0.0669880,0.0061730,-0.0277520},
        {-0.0829880,-0.0395410,0.0253720},
        {-0.0829880,-0.0395410,-0.0253720},
        {-0.0669130,-0.0228660,0.0179600},
        {-0.0669130,-0.0228660,-0.0179600},
        {-0.0987550,-0.0769490,0.0065790},
        {-0.0987550,-0.0769490,-0.0065790},
        {-0.0712530,-0.0293620,0.0090120},
        {-0.0712530,-0.0293620,-0.0090120},
        {-0.0904950,-0.0559600,0.0267990},
        {-0.0904950,-0.0559600,-0.0267990},
        {-0.0769520,-0.0372560,0.0086350},
        {-0.0769520,-0.0372560,-0.0086350}
      };  // Lucy
      BoundingBoxOnOff = Off;
    };
// this is the identity transform and is needed because the pelvis is unilateral (doesn't need a mirrored transform)
    AnyFunTransform3DIdentity ScaleFunction = {
      PreTransforms = {&.RBFTransform};
    };
  }; // close sacrum  folder
 }; // close scaling function
```

ScalingFunctionTLEMLucyFemur_2014001

```
AnyFolder ScalingFunctionTLEMLucyFemur = {
  AnyFolder Right = {
    AnyFolder Thigh = {
        AnyFunTransform3DRBF RBFTransform = {
            RBFDef.Type = RBF_ThinPlate;
            PolynomDegree = 1;
            Points0 = {
                {0.0000000,0.0000000,0.0000000},
                {-0.0000000,-0.3616821,0.0000000},
                {-0.0097563,-0.3678799,0.0012967},
                {-0.0000000,-0.3660632,0.0408203},
                {-0.0000000,-0.3573010,-0.0408203},
                {0.0161460,-0.0072838,0.0601290},
                {0.0220217,-0.0203698,0.0463848},
                {0.0123977,-0.0241932,0.0668573},
                {0.0006898,0.0018121,0.0538181},
                {0.0122809,-0.0068668,0.0414535},
                {-0.0058991,-0.0138188,0.0648412},
                {0.0211469,-0.0380855,0.0538111},
                {0.0172133,-0.0317342,0.0328381},
                {0.0177311,-0.0131946,0.0256176},
                {-0.0110079,-0.0308867,0.0632370},
                {0.0039435,-0.0395977,0.0638271},
                {-0.0041683,-0.0187472,0.0413214},
                {-0.0177999,-0.0021535,0.0496084},
                {0.0001603,-0.0053126,0.0279593},
                {-0.0211600,-0.0216243,0.0497147},
                {0.0096163,-0.0568663,0.0545719},
                {0.0162112,-0.0515013,0.0395230},
                {0.0093533,-0.0433265,0.0187387},
                {0.0119398,-0.0238469,0.0118403},
                {0.0210707,-0.0048848,0.0065878},
                {0.0125013,0.0064094,0.0177702},
                {-0.0191523,-0.0356053,0.0382429},
                {-0.0115821,-0.0474937,0.0537648},
                {-0.0100073,-0.0236371,0.0267322},
                {-0.0078925,0.0107098,0.0182833},
                {-0.0134217,-0.0055356,0.0182000},
                {-0.0090061,-0.0665700,0.0475484},
                {0.0071095,-0.0761976,0.0520035},
                {0.0182119,-0.0704149,0.0386546},
                {0.0073764,-0.0609481,0.0221006},
                {-0.0122984,-0.0439585,0.0198428},
                {-0.0032253,-0.0306792,0.0110995},
```

{-0.0012975,-0.0198451,-0.0052592},
{0.0140405,-0.0161125,-0.0071649},
{0.0189222,-0.0014898,-0.0127226},
{0.0181642,0.0132812,-0.0011050},
{0.0045119,0.0200001,0.0091189},
{-0.0176761,-0.0541983,0.0348587},
{-0.0136548,-0.0142015,0.0054311},
{-0.0135510,0.0182940,0.0017175},
{-0.0223865,0.0023399,0.0028018},
{-0.0086455,-0.0738247,0.0283843},
{-0.0050356,-0.0877749,0.0442195},
{0.0160135,-0.0919319,0.0465120},
{0.0128290,-0.0785810,0.0232431},
{-0.0168328,-0.0616327,0.0155917},
{-0.0172369,-0.0094567,-0.0119502},
{0.0002090,-0.0093641,-0.0209779},
{0.0064827,0.0081538,-0.0198453},
{0.0010777,0.0210422,-0.0084721},
{-0.0129485,0.0085451,-0.0162212},
{-0.0002322,-0.0924033,0.0245644},
{-0.0028175,-0.1052263,0.0418294},
{0.0172322,-0.1111444,0.0445760},
{0.0203259,-0.0957409,0.0271449},
{0.0057102,-0.1088933,0.0195113},
{-0.0010852,-0.1230776,0.0363132},
{0.0182727,-0.1303312,0.0422884},
{0.0242891,-0.1161703,0.0265054},
{0.0094531,-0.1268584,0.0162294},
{0.0014244,-0.1413293,0.0331653},
{0.0207271,-0.1496427,0.0394157},
{0.0267562,-0.1360142,0.0236798},
{0.0113839,-0.1461651,0.0136024},
{0.0030845,-0.1595094,0.0297156},
{0.0209528,-0.1691386,0.0369775},
{0.0283958,-0.1540209,0.0213795},
{0.0129499,-0.1649012,0.0113589},
{0.0016490,-0.1783108,0.0216287},
{0.0147105,-0.1880678,0.0328614},
{0.0298491,-0.1859865,0.0248551},
{0.0286645,-0.1714377,0.0169488},
{0.0165897,-0.1861006,0.0082595},
{0.0014000,-0.1973476,0.0183740},
{0.0114301,-0.2109792,0.0286157},
{0.0267422,-0.2037622,0.0296951},
{0.0288630,-0.2018335,0.0130127},
{0.0145471,-0.2083505,0.0062833},

Biology Open • Supplementary information

{0.0011300,-0.2198071,0.0155602},
{0.0126610,-0.2321235,0.0275354},
{0.0275647,-0.2219268,0.0265185},
{0.0286645,-0.2230253,0.0098701},
{0.0130434,-0.2304822,0.0039177},
{0.0022984,-0.2409585,0.0149650},
{0.0101315,-0.2534740,0.0252125},
{0.0279485,-0.2428555,0.0234276},
{0.0276684,-0.2421559,0.0058691},
{0.0101250,-0.2529527,0.0018983},
{0.0021376,-0.2667831,0.0107073},
{0.0114309,-0.2734905,0.0249960},
{0.0273923,-0.2634953,0.0216324},
{0.0278429,-0.2592546,0.0037692},
{0.0159101,-0.2712985,-0.0033988},
{0.0036708,-0.2863280,0.0004644},
{0.0023115,-0.2884756,0.0187554},
{0.0231385,-0.2879051,0.0224853},
{0.0304009,-0.2799973,0.0083549},
{0.0238189,-0.2917246,-0.0052462},
{0.0064904,-0.3027687,-0.0070587},
{-0.0003492,-0.3064690,0.0093014},
{0.0122179,-0.3045269,0.0243511},
{0.0301837,-0.3030127,0.0114462},
{0.0234182,-0.3107336,-0.0085198},
{0.0046893,-0.3214995,-0.0123881},
{-0.0022352,-0.3264905,0.0043340},
{0.0016841,-0.3202756,0.0217839},
{0.0229722,-0.3196887,0.0215083},
{0.0292438,-0.3228042,0.0051430},
{0.0221641,-0.3296190,-0.0132361},
{0.0100459,-0.3417597,-0.0244560},
{-0.0053009,-0.3375737,-0.0137705},
{-0.0064432,-0.3487307,0.0028676},
{-0.0059346,-0.3407801,0.0202938},
{0.0105130,-0.3365976,0.0264175},
{0.0282695,-0.3386249,0.0180307},
{0.0270402,-0.3431844,-0.0001487},
{0.0257040,-0.3506950,-0.0181851},
{0.0178948,-0.3636114,-0.0302838},
{0.0043149,-0.3544109,-0.0381949},
{-0.0095210,-0.3461482,-0.0295031},
{-0.0164526,-0.3542895,-0.0126666},
{-0.0060896,-0.3690127,-0.0033061},
{-0.0176523,-0.3612686,0.0110109},
{-0.0153310,-0.3546943,0.0299634},

Biology Open • Supplementary information

{0.0021329,-0.3508021,0.0350763},
{0.0210690,-0.3548628,0.0279835},
{0.0356594,-0.3558162,0.0159356},
{0.0275718,-0.3617450,-0.0018607},
{0.0298503,-0.3741530,-0.0163721},
{0.0175320,-0.3855300,-0.0277515},
{0.0025604,-0.3740099,-0.0397137},
{-0.0136727,-0.3624016,-0.0402102},
{-0.0277647,-0.3567472,-0.0284947},
{-0.0275446,-0.3699630,-0.0120829},
{-0.0175846,-0.3859355,-0.0177922},
{0.0001572,-0.3856873,-0.0118050},
{0.0030262,-0.3834856,0.0075553},
{-0.0144114,-0.3810887,0.0140304},
{-0.0247706,-0.3701239,0.0285134},
{-0.0044804,-0.3677201,0.0404632},
{0.0133183,-0.3712274,0.0334208},
{0.0324861,-0.3723417,0.0245668},
{0.0271451,-0.3766027,0.0072441},
{0.0167985,-0.3822816,-0.0065349},
{-0.0001454,-0.3910823,-0.0303010},
{-0.0144998,-0.3834154,-0.0384137},
{-0.0301321,-0.3750841,-0.0321336},
{0.0196491,-0.3864804,0.0210853},
{0.0015726,-0.3888034,0.0276569},
{-0.0146652,-0.3847848,0.0336913},
{-0.0014588,-0.0619951,0.0219778},
{-0.0118435,-0.0550626,0.0139639},
{0.0129812,-0.0349966,0.0673128},
{-0.0162907,-0.0105666,0.0597739},
{-0.0012885,-0.0520816,0.0562989},
{-0.0090599,-0.0631298,0.0172224},
{-0.0000222,-0.0771663,0.0231897},
{-0.0068148,0.0023885,-0.0160086},
{-0.0180544,-0.0425409,0.0446585},
{0.0020525,-0.0249904,0.0691549},
{-0.0157097,-0.0662315,0.0238393},
{-0.0010518,-0.0510686,0.0199353},
{-0.0208874,-0.0152865,0.0500034},
{0.0168316,-0.0280857,0.0666680},
{-0.0187392,-0.0282333,0.0403282},
{0.0191622,-0.0351633,0.0622707},
{0.0001216,-0.0706289,0.0524049},
{-0.0169862,-0.0399062,0.0311827},
{-0.0185737,-0.0533066,0.0151224},
{-0.0219058,-0.0528723,0.0222263},

Biology Open • Supplementary information

{-0.0028271,-0.0391620,0.0148452},
{0.0117900,-0.0553425,0.0235348},
{0.0219835,-0.0106400,0.0396338},
{-0.0025366,-0.0401046,0.0625273},
{0.0141017,-0.0018767,0.0198108},
{-0.0078770,-0.0473929,0.0173542},
{-0.0065351,-0.0766431,0.0470258},
{0.0065585,0.0145593,0.0161638},
{-0.0118588,0.0172875,-0.0085064},
{-0.0074087,-0.0627500,0.0506700},
{0.0071940,-0.0780068,0.0213362},
{0.0126736,-0.0708176,0.0244108},
{0.0074694,-0.0436450,0.0604196},
{0.0154186,-0.0139471,0.0064312},
{-0.0053535,-0.0213210,0.0040094},
{-0.0088733,0.0002018,0.0205402},
{-0.0112471,-0.0542608,0.0500266},
{-0.0061625,-0.0248673,0.0653244},
{0.0093695,-0.0065826,0.0614157},
{0.0138015,-0.0467322,0.0284333},
{-0.0020999,-0.0186885,-0.0116392},
{-0.0185640,-0.0225591,0.0566779},
{0.0175798,0.0124360,-0.0068467},
{-0.0039962,0.0217127,0.0053055},
{0.0014975,-0.0784591,0.0514056},
{-0.0172997,-0.0386971,0.0525252},
{0.0196540,-0.0268271,0.0416806},
{0.0135296,-0.0406504,0.0236882},
{0.0182471,0.0095959,0.0087906},
{0.0218667,0.0016804,0.0058793},
{0.0224975,-0.0022652,-0.0022994},
{0.0166750,-0.0191391,0.0236436},
{0.0119785,-0.0328723,0.0195376},
{0.0187631,-0.0133469,0.0308741},
{-0.0187543,-0.0461463,0.0257491},
{0.0039834,-0.0373928,0.0683089},
{0.0157029,-0.0423169,0.0385226},
{0.0164003,-0.0087069,0.0131181},
{0.0008253,0.0033370,-0.0212576},
{0.0101707,-0.0009724,-0.0205688},
{0.0102948,-0.0642683,0.0524532},
{0.0152217,-0.0557944,0.0508978},
{0.0172973,-0.0565707,0.0419468},
{0.0167723,-0.0635334,0.0369834},
{-0.0047397,-0.0695325,0.0241398},
{-0.0152913,0.0035778,0.0161461},

Biology Open • Supplementary information

{0.0091697,0.0009340,0.0575160},
{0.0196686,-0.0089533,0.0500006},
{0.0183998,-0.0212943,0.0622968},
{-0.0114340,-0.0316810,0.0250940},
{-0.0206442,-0.0092599,-0.0028425},
{0.0166868,-0.0784295,0.0276925},
{-0.0069678,-0.0054521,-0.0207011},
{0.0049490,0.0151109,-0.0160093},
{-0.0084025,-0.0318937,0.0162788},
{-0.0173679,0.0095594,-0.0108509},
{-0.0218912,0.0063457,-0.0020698},
{-0.0106547,-0.0668750,0.0397749},
{0.0136098,-0.0170666,-0.0018890},
{0.0141599,-0.0441405,0.0587594},
{0.0093255,-0.0183218,-0.0083008},
{0.0106367,-0.0128276,-0.0154269},
{0.0037350,-0.0549365,0.0209786},
{0.0087662,-0.0472902,0.0195496},
{0.0016839,-0.0708213,0.0221325},
{-0.0202977,-0.0530540,0.0301270},
{-0.0068584,0.0021781,0.0530002},
{-0.0144817,-0.0297218,0.0605833},
{0.0197015,-0.0165217,0.0560654},
{0.0156609,-0.0705363,0.0477736},
{0.0207876,-0.0221433,0.0330733},
{0.0092324,-0.0300767,0.0713160},
{-0.0203392,-0.0034488,0.0097332},
{0.0156978,-0.0143438,0.0629053},
{0.0143267,-0.0563519,0.0317489},
{-0.0161685,-0.0465391,0.0188591},
{-0.0196397,-0.0309875,0.0546496},
{0.0154804,-0.0380422,0.0305433},
{-0.0077774,-0.0351303,0.0637765},
{0.0063980,-0.0086419,-0.0201654},
{0.0030146,-0.0151094,-0.0165974},
{-0.0210080,-0.0096030,0.0507855},
{-0.0105684,-0.0411377,0.0586146},
{0.0180619,-0.0498960,0.0469835},
{-0.0192318,0.0116108,0.0024070},
{-0.0165651,0.0083622,0.0126870},
{-0.0014333,-0.0627138,0.0534746},
{0.0145127,-0.0080178,0.0353667},
{0.0148374,-0.0012228,0.0566181},
{0.0153834,-0.0062427,0.0444727},
{-0.0155164,-0.0132103,-0.0096398},
{0.0068685,-0.0221022,0.0011547},

{0.0003332,-0.0458500,0.0183716},
{0.0205582,0.0040956,-0.0082969},
{0.0175752,0.0009340,-0.0148407},
{0.0142357,-0.0063623,-0.0169332},
{-0.0147285,0.0023412,-0.0164678},
{-0.0158781,-0.0064451,-0.0151346},
{0.0175951,0.0133456,0.0037559},
{0.0117660,0.0189899,-0.0030690},
{0.0096040,0.0151827,-0.0137450},
{0.0096565,-0.0215194,0.0662734},
{-0.0006793,-0.0074494,0.0631218},
{0.0007819,-0.0004519,0.0600781},
{0.0065377,-0.0125433,0.0654199},
{0.0090461,0.0073096,0.0188711},
{0.0151418,-0.0168302,0.0146465},
{0.0025166,-0.0231863,0.0688894},
{-0.0097891,-0.0152077,0.0253086},
{-0.0109149,-0.0177935,0.0119825},
{-0.0072079,-0.0099373,0.0272725},
{-0.0183727,-0.0467429,0.0356276},
{-0.0126871,-0.0584555,0.0428141},
{-0.0057054,-0.0273042,0.0103554},
{0.0215142,-0.0159693,0.0474531},
{0.0128492,-0.0070016,0.0287645},
{0.0052134,-0.0017047,0.0247463},
{-0.0024284,-0.0707498,0.0513049},
{-0.0036140,0.0220593,-0.0040484},
{-0.0002405,-0.0121253,0.0390050},
{-0.0119811,-0.0156737,0.0015445},
{0.0142082,-0.0279792,0.0246553},
{0.0122244,-0.0262013,0.0169829},
{-0.0146264,-0.0038855,0.0549384},
{0.0207450,-0.0325771,0.0496412},
{-0.0155040,-0.0497560,0.0437087},
{-0.0081119,-0.0756450,0.0387365},
{0.0039684,-0.0062047,0.0313900},
{-0.0023726,-0.0038892,-0.0220521},
{-0.0136977,-0.0339839,0.0602619},
{-0.0035399,0.0089081,-0.0188894},
{-0.0049820,0.0176571,-0.0132254},
{0.0193902,-0.0803191,0.0360429},
{0.0040648,-0.0697109,0.0218205},
{0.0170470,-0.0818610,0.0456892},
{0.0104175,0.0188635,0.0064965},
{0.0135206,0.0092549,0.0158079},
{0.0023933,0.0160498,-0.0157404},

Biology Open • Supplementary information

{0.0164753,-0.0118197,-0.0096995},
{0.0202584,-0.0066858,-0.0082383},
{-0.0098401,-0.0226001,0.0314028},
{-0.0130937,-0.0309622,0.0320764},
{-0.0201510,-0.0613297,0.0222264},
{-0.0010389,0.0036055,0.0209003},
{0.0042025,0.0073392,0.0199393},
{-0.0021630,-0.0038531,0.0251898},
{-0.0007845,-0.0293574,0.0675788},
{0.0070027,-0.0406327,0.0624565},
{-0.0124363,-0.0183001,0.0627966},
{-0.0120485,-0.0256613,0.0628493},
{0.0101200,0.0197695,0.0039334},
{0.0167636,0.0148266,0.0014155},
{-0.0074981,-0.0106930,0.0632797},
{0.0142533,-0.0503445,0.0351954},
{-0.0146107,-0.0460953,0.0506120},
{-0.0161117,-0.0471301,0.0178900},
{0.0112149,-0.0792793,0.0501460},
{-0.0099060,-0.0230794,0.0186951},
{-0.0114543,-0.0153049,0.0183675},
{-0.0042882,0.0161879,0.0151423},
{0.0014582,-0.0638572,0.0541080},
{0.0048554,-0.0582603,0.0553815},
{-0.0017077,-0.0572302,0.0546635},
{0.0166090,-0.0697169,0.0323038},
{-0.0208812,-0.0164273,0.0474354},
{0.0130454,-0.0241864,0.0178294},
{0.0053123,-0.0483770,0.0582251},
{0.0112225,-0.0361429,0.0673300},
{0.0085729,-0.0771256,0.0216100},
{-0.0070783,-0.0488033,0.0560869},
{0.0182083,-0.0411024,0.0454236},
{-0.0119590,-0.0403809,0.0213546},
{0.0233409,-0.0185535,0.0366343},
{-0.0046347,-0.0571211,0.0208243},
{0.0183166,-0.0006966,0.0140116},
{0.0061913,0.0053512,0.0201003},
{-0.0002364,-0.0310828,0.0680351},
{-0.0170773,0.0139270,-0.0054987},
{0.0110805,0.0156034,0.0117010},
{-0.0124628,0.0164667,0.0094550},
{-0.0209014,-0.0073407,-0.0051969},
{-0.0210131,0.0010693,-0.0086891},
{-0.0041596,-0.0172332,0.0663041},
{-0.0216595,-0.0322172,0.0451540},

Biology Open • Supplementary information

{0.0051908,-0.0426014,0.0613013},
{0.0150003,-0.0416136,0.0606257},
{-0.0135090,-0.0674234,0.0236425},
{0.0146416,0.0073691,0.0160052},
{0.0195615,-0.0112412,-0.0010333},
{-0.0198115,-0.0098244,0.0044735},
{-0.0157054,-0.0521236,0.0395039},
{-0.0081381,-0.0173108,-0.0046152},
{-0.0218324,-0.0262661,0.0471115},
{0.0051446,-0.0707257,0.0530555},
{0.0179359,-0.0471493,0.0528134},
{-0.0005591,-0.0317983,0.0113632},
{0.0071707,-0.0305943,0.0121186},
{0.0206239,-0.0061367,0.0066642},
{0.0187057,-0.0745868,0.0420207},
{-0.0095784,-0.0163113,0.0259524},
{-0.0012181,0.0127661,0.0186855},
{0.0059420,-0.0630691,0.0220087},
{0.0069408,-0.0573878,0.0552614},
{0.0150782,-0.0132884,-0.0101793},
{0.0005533,-0.0078574,0.0633775},
{-0.0088284,-0.0146399,-0.0147837},
{0.0167444,-0.0651105,0.0470347},
{0.0082666,-0.0181741,-0.0095133},
{-0.0175162,-0.0637444,0.0177069},
{0.0153795,-0.0360767,0.0287608},
{0.0162363,-0.0018864,0.0548412},
{0.0052731,-0.0075919,0.0392990},
{-0.0022288,-0.0556644,0.0549909},
{-0.0156238,-0.0550038,0.0377688},
{-0.0204137,-0.0100550,0.0536429},
{-0.0093683,0.0122429,-0.0162013},
{-0.0202927,0.0049166,0.0083887},
{0.0074706,-0.0627945,0.0222133},
{0.0035034,-0.0376103,0.0672421},
{-0.0190752,-0.0194129,0.0555530},
{0.0061758,-0.0074961,-0.0207741},
{0.0192011,-0.0273063,0.0588233},
{0.0130796,-0.0134383,0.0638611},
{0.0120421,0.0019566,-0.0194140},
{0.0154152,-0.0065294,0.0179581},
{-0.0157335,0.0101219,0.0123981},
{0.0110376,-0.0488274,0.0567911},
{-0.0055170,-0.0343676,0.0647140},
{0.0216204,-0.0273988,0.0512041},
{0.0161331,-0.0135875,-0.0075501},

Biology Open • Supplementary information

{-0.0184643,-0.0455633,0.0360087},
{-0.0091978,-0.0682413,0.0460847},
{0.0034304,-0.0471677,0.0588866},
{-0.0048025,-0.0384316,0.0149400},
{0.0118505,0.0109735,-0.0159161},
{-0.0027036,-0.0204189,-0.0015094},
{-0.0117607,-0.0692759,0.0292171},
{-0.0107080,-0.0033455,-0.0190487},
{-0.0042548,0.0214947,-0.0056846},
{-0.0097315,-0.0208529,0.0131997},
{-0.0178958,-0.0627218,0.0171551},
{-0.0068692,-0.0688818,0.0233316},
{0.0012083,0.0120695,-0.0186179},
{0.0215073,-0.0236027,0.0503069},
{-0.0060262,-0.0534931,0.0189523},
{0.0179951,-0.0336785,0.0397267},
{0.0190509,-0.0284972,0.0336394},
{-0.0169188,-0.0133609,-0.0014203},
{0.0135722,0.0136491,0.0114695},
{-0.0150549,-0.0123230,0.0067722},
{-0.0148411,-0.0311945,0.0355412},
{0.0185490,-0.0794042,0.0431266},
{0.0193568,-0.0087326,0.0575704},
{0.0201026,-0.0144353,0.0531586},
{0.0025583,-0.0248211,0.0045714},
{0.0132689,-0.0214866,0.0652899},
{-0.0028669,-0.0099311,0.0309271},
{-0.0219487,-0.0054893,-0.0021853},
{0.0026180,-0.0203806,-0.0040620},
{-0.0116230,-0.0668518,0.0370152},
{-0.0133007,-0.0264159,0.0619176},
{0.0186167,0.0050491,-0.0120059},
{-0.0191359,-0.0591690,0.0288149},
{-0.0178121,-0.0041834,0.0141448},
{0.0123077,-0.0063648,0.0279743},
{-0.0157729,-0.0374010,0.0315335},
{0.0149328,-0.0659100,0.0290095},
{0.0006205,0.0021977,-0.0214613},
{0.0170484,-0.0715687,0.0325980},
{0.0068499,0.0061192,0.0196608},
{-0.0167075,0.0020024,0.0150382},
{0.0059801,-0.0393117,0.0167489},
{-0.0072506,-0.0171067,0.0338751},
{0.0202363,-0.0251596,0.0420997},
{-0.0199311,-0.0601255,0.0171522},
{0.0216641,0.0053410,-0.0027841},

Biology Open • Supplementary information

{-0.0197665,-0.0606125,0.0252972},
{0.0129986,-0.0214528,0.0098002},
{0.0201151,-0.0100929,0.0460501},
{0.0104137,0.0017765,-0.0203073},
{0.0166557,-0.0064123,0.0140197},
{-0.0124120,-0.0596549,0.0422637},
{0.0014885,0.0202590,0.0096781},
{0.0028720,0.0020207,-0.0219044},
{0.0376095,-0.3675427,0.0214537},
{0.0049949,0.0206417,-0.0079017},
{-0.0191723,-0.0407590,0.0391310},
{-0.0316406,-0.3638215,-0.0274841},
{0.0021392,0.0226920,0.0000985},
{0.0168435,-0.0229382,0.0264788},
{-0.0157304,-0.0005437,-0.0161456},
{0.0203722,-0.0299750,0.0562762},
{-0.0171077,0.0134146,0.0059615},
{0.0175519,-0.0370653,0.0413374},
{0.0154657,-0.0405127,0.0348980},
{-0.0215172,-0.0323502,0.0481925},
{0.0177998,-0.0515776,0.0444709},
{-0.0183804,-0.0448316,0.0275139},
{0.0033607,-0.0502028,0.0575894},
{0.0010325,-0.0479377,0.0191141},
{0.0224852,-0.0181821,0.0451780},
{0.0084089,-0.0720538,0.0521031},
{-0.0070880,-0.0772912,0.0288023},
{-0.0223712,-0.0028990,0.0024047},
{0.0173136,-0.0120017,0.0204612},
{0.0137772,-0.0623537,0.0276621},
{-0.0100390,-0.0231180,0.0292222},
{-0.0196395,-0.3730207,0.0364730},
{-0.0094445,-0.0526688,0.0528111},
{-0.0115309,-0.3679776,-0.0104995},
{0.0129812,-0.0410477,0.0224072},
{0.0117394,0.0154849,-0.0117191},
{-0.0245208,-0.3649412,0.0295111},
{0.0147476,-0.0502966,0.0364899},
{0.0006132,-0.0446771,0.0600524},
{-0.0118123,0.0191139,0.0031977},
{-0.0017185,-0.0163303,0.0664216},
{-0.0085098,-0.0074564,0.0247745},
{0.0197459,-0.0086852,0.0430460},
{0.0187064,-0.0223532,0.0600966},
{-0.0206552,-0.0099712,0.0525024},
{0.0207525,0.0084646,-0.0024846},

Biology Open • Supplementary information

{-0.0094915,-0.0325446,0.0184441},
{-0.0014464,-0.0140050,-0.0179819},
{-0.0122725,-0.0573444,0.0463125},
{-0.0106397,0.0177211,0.0094669},
{0.0111981,0.0191236,-0.0041491},
{-0.0162790,-0.0256279,0.0592687},
{-0.0060120,-0.3653959,0.0413411},
{-0.0135007,-0.3703638,-0.0387476},
{-0.0134258,-0.3703142,-0.0379467},
{-0.0133509,-0.3702645,-0.0371458},
{-0.0132760,-0.3702148,-0.0363450},
{-0.0132011,-0.3701651,-0.0355441},
{-0.0131262,-0.3701154,-0.0347432},
{-0.0130513,-0.3700658,-0.0339423},
{-0.0129765,-0.3700161,-0.0331414},
{-0.0129016,-0.3699664,-0.0323405},
{-0.0128267,-0.3699167,-0.0315396},
{-0.0127518,-0.3698670,-0.0307388},
{-0.0126769,-0.3698174,-0.0299379},
{-0.0126020,-0.3697677,-0.0291370},
{-0.0125271,-0.3697180,-0.0283361},
{-0.0124523,-0.3696683,-0.0275352},
{-0.0123774,-0.3696187,-0.0267343},
{-0.0123025,-0.3695690,-0.0259334},
{-0.0122276,-0.3695193,-0.0251325},
{-0.0121527,-0.3694696,-0.0243317},
{-0.0120778,-0.3694199,-0.0235308},
{-0.0120029,-0.3693703,-0.0227299},
{-0.0119280,-0.3693206,-0.0219290},
{-0.0118532,-0.3692709,-0.0211281},
{-0.0117783,-0.3692212,-0.0203272},
{-0.0117034,-0.3691715,-0.0195263},
{-0.0116285,-0.3691219,-0.0187255},
{-0.0115536,-0.3690722,-0.0179246},
{-0.0114787,-0.3690225,-0.0171237},
{-0.0114038,-0.3689728,-0.0163228},
{-0.0113289,-0.3689231,-0.0155219},
{-0.0112541,-0.3688735,-0.0147210},
{-0.0111792,-0.3688238,-0.0139201},
{-0.0111043,-0.3687741,-0.0131192},
{-0.0110294,-0.3687244,-0.0123184},
{-0.0109545,-0.3686748,-0.0115175},
{-0.0108796,-0.3686251,-0.0107166},
{-0.0108047,-0.3685754,-0.0099157},
{-0.0107298,-0.3685257,-0.0091148},
{-0.0106550,-0.3684760,-0.0083139},

Biology Open • Supplementary information

{-0.0105801,-0.3684264,-0.0075130},
{-0.0105052,-0.3683767,-0.0067121},
{-0.0104303,-0.3683270,-0.0059113},
{-0.0103554,-0.3682773,-0.0051104},
{-0.0102805,-0.3682276,-0.0043095},
{-0.0102056,-0.3681780,-0.0035086},
{-0.0101307,-0.3681283,-0.0027077},
{-0.0100559,-0.3680786,-0.0019068},
{-0.0099810,-0.3680289,-0.0011059},
{-0.0099061,-0.3679792,-0.0003051},
{-0.0098312,-0.3679296,0.0004958},
{-0.0097563,-0.3678799,0.0012967},
{-0.0096814,-0.3678302,0.0020976},
{-0.0096065,-0.3677805,0.0028985},
{-0.0095317,-0.3677308,0.0036994},
{-0.0094568,-0.3676812,0.0045003},
{-0.0093819,-0.3676315,0.0053012},
{-0.0093070,-0.3675818,0.0061020},
{-0.0092321,-0.3675321,0.0069029},
{-0.0091572,-0.3674825,0.0077038},
{-0.0090823,-0.3674328,0.0085047},
{-0.0090074,-0.3673831,0.0093056},
{-0.0089326,-0.3673334,0.0101065},
{-0.0088577,-0.3672837,0.0109074},
{-0.0087828,-0.3672341,0.0117082},
{-0.0087079,-0.3671844,0.0125091},
{-0.0086330,-0.3671347,0.0133100},
{-0.0085581,-0.3670850,0.0141109},
{-0.0084832,-0.3670353,0.0149118},
{-0.0084083,-0.3669857,0.0157127},
{-0.0083335,-0.3669360,0.0165136},
{-0.0082586,-0.3668863,0.0173145},
{-0.0081837,-0.3668366,0.0181153},
{-0.0081088,-0.3667869,0.0189162},
{-0.0080339,-0.3667373,0.0197171},
{-0.0079590,-0.3666876,0.0205180},
{-0.0078841,-0.3666379,0.0213189},
{-0.0078092,-0.3665882,0.0221198},
{-0.0077344,-0.3665386,0.0229207},
{-0.0076595,-0.3664889,0.0237215},
{-0.0075846,-0.3664392,0.0245224},
{-0.0075097,-0.3663895,0.0253233},
{-0.0074348,-0.3663398,0.0261242},
{-0.0073599,-0.3662902,0.0269251},
{-0.0072850,-0.3662405,0.0277260},
{-0.0072101,-0.3661908,0.0285269},

```
		{-0.0071353,-0.3661411,0.0293278},
		{-0.0070604,-0.3660914,0.0301286},
		{-0.0069855,-0.3660418,0.0309295},
		{-0.0069106,-0.3659921,0.0317304},
		{-0.0068357,-0.3659424,0.0325313},
		{-0.0067608,-0.3658927,0.0333322},
		{-0.0066859,-0.3658430,0.0341331},
		{-0.0066111,-0.3657934,0.0349340},
		{-0.0065362,-0.3657437,0.0357348},
		{-0.0064613,-0.3656940,0.0365357},
		{-0.0063864,-0.3656443,0.0373366},
		{-0.0063115,-0.3655947,0.0381375},
		{-0.0062366,-0.3655450,0.0389384},
		{-0.0061617,-0.3654953,0.0397393},
		{-0.0060868,-0.3654456,0.0405402}
		};
	Points1 = {
		{0.0000000,0.0000000,0.0000000},
		{-0.0000000,-0.3844239,0.0000000},
		{-0.0090962,-0.3910114,0.0012090},
		{-0.0000000,-0.3890805,0.0380584},
		{-0.0000000,-0.3797674,-0.0380584},
		{0.0135412,-0.0086390,0.0545946},
		{0.0196483,-0.0224683,0.0429684},
		{0.0094431,-0.0254847,0.0595583},
		{0.0001526,-0.0003974,0.0485973},
		{0.0109662,-0.0068334,0.0378725},
		{-0.0057810,-0.0139956,0.0617691},
		{0.0164460,-0.0386834,0.0492159},
		{0.0147348,-0.0335894,0.0316250},
		{0.0139180,-0.0144630,0.0239261},
		{-0.0099034,-0.0321235,0.0589375},
		{0.0032807,-0.0420515,0.0579651},
		{-0.0030036,-0.0201361,0.0393148},
		{-0.0121921,-0.0043215,0.0457883},
		{-0.0000087,-0.0042392,0.0265660},
		{-0.0119720,-0.0229308,0.0470686},
		{0.0098360,-0.0608614,0.0517067},
		{0.0156426,-0.0540685,0.0368361},
		{0.0081543,-0.0456550,0.0184707},
		{0.0094302,-0.0243144,0.0120498},
		{0.0178693,-0.0055164,0.0055080},
		{0.0101116,0.0060820,0.0153716},
		{-0.0119099,-0.0382496,0.0350282},
		{-0.0097840,-0.0497224,0.0497680},
		{-0.0089820,-0.0255951,0.0240460},
```

Biology Open • Supplementary information

{-0.0059634,0.0107064,0.0153997},
{-0.0110670,-0.0058902,0.0151803},
{-0.0072431,-0.0703173,0.0431595},
{0.0061293,-0.0806876,0.0501372},
{0.0166575,-0.0756364,0.0355340},
{0.0080844,-0.0651352,0.0202955},
{-0.0114436,-0.0462484,0.0199460},
{-0.0040837,-0.0328744,0.0098553},
{-0.0004320,-0.0199527,-0.0046396},
{0.0124444,-0.0156380,-0.0062994},
{0.0168150,-0.0017977,-0.0107581},
{0.0164873,0.0130246,-0.0011711},
{0.0030473,0.0197657,0.0086996},
{-0.0138372,-0.0574107,0.0327293},
{-0.0114962,-0.0125451,0.0056021},
{-0.0117669,0.0170421,0.0015537},
{-0.0179504,0.0023626,0.0030653},
{-0.0066042,-0.0778865,0.0268022},
{-0.0041791,-0.0933870,0.0406404},
{0.0141802,-0.0980784,0.0433196},
{0.0121047,-0.0837981,0.0210443},
{-0.0098260,-0.0622066,0.0207177},
{-0.0140997,-0.0088782,-0.0098827},
{0.0006385,-0.0091689,-0.0180615},
{0.0057996,0.0077976,-0.0166249},
{0.0016318,0.0201703,-0.0078545},
{-0.0102073,0.0084386,-0.0140539},
{0.0004298,-0.0982470,0.0225398},
{-0.0018052,-0.1116971,0.0387523},
{0.0152711,-0.1182631,0.0413296},
{0.0182152,-0.1016684,0.0254613},
{0.0060568,-0.1154334,0.0186961},
{0.0000134,-0.1304245,0.0339339},
{0.0163456,-0.1384823,0.0391950},
{0.0217395,-0.1234026,0.0249958},
{0.0094227,-0.1343665,0.0159732},
{0.0022676,-0.1497358,0.0311605},
{0.0185995,-0.1589238,0.0365616},
{0.0240072,-0.1444068,0.0224697},
{0.0111285,-0.1548267,0.0136295},
{0.0037291,-0.1690250,0.0280404},
{0.0188925,-0.1795936,0.0343210},
{0.0255149,-0.1635052,0.0203586},
{0.0124673,-0.1747466,0.0115332},
{0.0025634,-0.1889392,0.0207514},
{0.0135960,-0.1996045,0.0306237},

Biology Open • Supplementary information

{0.0267720,-0.1975298,0.0233111},
{0.0258527,-0.1819770,0.0163210},
{0.0155784,-0.1973494,0.0085822},
{0.0022824,-0.2092063,0.0177187},
{0.0107691,-0.2239396,0.0267330},
{0.0240408,-0.2164442,0.0275959},
{0.0260990,-0.2143039,0.0126082},
{0.0137584,-0.2210323,0.0066529},
{0.0019272,-0.2331542,0.0150273},
{0.0117936,-0.2464923,0.0256401},
{0.0248176,-0.2357798,0.0246453},
{0.0259704,-0.2368650,0.0096352},
{0.0123799,-0.2446174,0.0043429},
{0.0028017,-0.2557481,0.0143195},
{0.0095327,-0.2692291,0.0234601},
{0.0252244,-0.2580655,0.0217569},
{0.0251523,-0.2572308,0.0058898},
{0.0097243,-0.2685715,0.0023294},
{0.0024975,-0.2833112,0.0102660},
{0.0106350,-0.2905897,0.0231875},
{0.0248192,-0.2800449,0.0200541},
{0.0253535,-0.2754549,0.0038535},
{0.0148192,-0.2881857,-0.0027043},
{0.0037536,-0.3041721,0.0007302},
{0.0024332,-0.3065012,0.0174974},
{0.0211643,-0.3060160,0.0208162},
{0.0276998,-0.2975966,0.0078790},
{0.0219434,-0.3100179,-0.0046363},
{0.0061855,-0.3217350,-0.0063975},
{-0.0001608,-0.3256884,0.0087270},
{0.0113456,-0.3236657,0.0226159},
{0.0277738,-0.3220914,0.0106489},
{0.0217295,-0.3302622,-0.0078546},
{0.0043720,-0.3417147,-0.0115499},
{-0.0020839,-0.3470196,0.0040407},
{0.0015701,-0.3404139,0.0203100},
{0.0214057,-0.3397921,0.0200474},
{0.0272577,-0.3431024,0.0047959},
{0.0206645,-0.3503447,-0.0123405},
{0.0093662,-0.3632489,-0.0228013},
{-0.0049422,-0.3587997,-0.0128388},
{-0.0060072,-0.3706581,0.0026736},
{-0.0055331,-0.3622077,0.0189207},
{0.0098017,-0.3577621,0.0246301},
{0.0263577,-0.3599170,0.0168107},
{0.0252116,-0.3647631,-0.0001387},

{0.0239630,-0.3727459,-0.0169538},
{0.0166841,-0.3864745,-0.0282348},
{0.0040230,-0.3766956,-0.0356078},
{-0.0088768,-0.3679133,-0.0275069},
{-0.0153394,-0.3765665,-0.0118095},
{-0.0056776,-0.3922154,-0.0030824},
{-0.0164580,-0.3839844,0.0102659},
{-0.0142937,-0.3769968,0.0279361},
{0.0019886,-0.3728598,0.0327030},
{0.0196435,-0.3771759,0.0260902},
{0.0332458,-0.3781893,0.0148574},
{0.0257073,-0.3844909,-0.0017348},
{0.0278306,-0.3976790,-0.0152644},
{0.0163467,-0.4097714,-0.0258738},
{0.0023871,-0.3975269,-0.0370267},
{-0.0127485,-0.3851887,-0.0374905},
{-0.0258861,-0.3791787,-0.0265677},
{-0.0256809,-0.3932256,-0.0112654},
{-0.0163939,-0.4102024,-0.0165883},
{0.0001466,-0.4099386,-0.0110062},
{0.0028214,-0.4075984,0.0070441},
{-0.0134363,-0.4050508,0.0130811},
{-0.0230946,-0.3933966,0.0265842},
{-0.0041773,-0.3908416,0.0377255},
{0.0124171,-0.3945694,0.0311595},
{0.0302890,-0.3957538,0.0229046},
{0.0253085,-0.4002828,0.0067539},
{0.0156619,-0.4063187,-0.0060927},
{-0.0001355,-0.4156727,-0.0282518},
{-0.0135187,-0.4075238,-0.0358146},
{-0.0280933,-0.3986687,-0.0299595},
{0.0183187,-0.4107815,0.0196587},
{0.0014662,-0.4132505,0.0257866},
{-0.0136720,-0.4089793,0.0314117},
{-0.0007887,-0.0668699,0.0180990},
{-0.0094557,-0.0582117,0.0196269},
{0.0090134,-0.0341478,0.0587373},
{-0.0130054,-0.0138754,0.0541406},
{-0.0015942,-0.0568813,0.0544290},
{-0.0057688,-0.0638763,0.0213544},
{-0.0012920,-0.0820757,0.0195001},
{-0.0064060,0.0016957,-0.0161583},
{-0.0114202,-0.0446431,0.0391109},
{0.0030282,-0.0266108,0.0622759},
{-0.0093114,-0.0659858,0.0244248},
{-0.0009779,-0.0542329,0.0174407},

Biology Open • Supplementary information

{-0.0120751,-0.0170313,0.0482581},
{0.0124771,-0.0301183,0.0571420},
{-0.0095913,-0.0299145,0.0377167},
{0.0130969,-0.0344253,0.0555422},
{-0.0008166,-0.0749192,0.0510161},
{-0.0135817,-0.0438598,0.0296953},
{-0.0127968,-0.0556168,0.0208900},
{-0.0149019,-0.0543970,0.0220972},
{-0.0022733,-0.0413394,0.0128944},
{0.0099263,-0.0579505,0.0226928},
{0.0198865,-0.0132547,0.0375802},
{-0.0028638,-0.0440375,0.0588229},
{0.0124334,-0.0028068,0.0163509},
{-0.0081238,-0.0486534,0.0179570},
{-0.0054765,-0.0808815,0.0421165},
{0.0051223,0.0147705,0.0124561},
{-0.0103827,0.0169941,-0.0057102},
{-0.0064846,-0.0671946,0.0450811},
{0.0072034,-0.0845142,0.0184154},
{0.0118795,-0.0741378,0.0230526},
{0.0071176,-0.0470642,0.0557831},
{0.0132683,-0.0125793,0.0071998},
{-0.0053102,-0.0234557,0.0043424},
{-0.0066089,-0.0009675,0.0190450},
{-0.0092851,-0.0560195,0.0463883},
{-0.0056952,-0.0260635,0.0623352},
{0.0077351,-0.0063286,0.0569325},
{0.0125115,-0.0503110,0.0280874},
{-0.0018455,-0.0180506,-0.0114262},
{-0.0126754,-0.0243432,0.0517598},
{0.0147287,0.0132441,-0.0066498},
{-0.0037315,0.0214621,0.0034644},
{0.0005805,-0.0846388,0.0494817},
{-0.0119445,-0.0393492,0.0468608},
{0.0182617,-0.0278250,0.0397911},
{0.0106526,-0.0427121,0.0226210},
{0.0163902,0.0103381,0.0067703},
{0.0188891,0.0020704,0.0063238},
{0.0197730,-0.0019705,-0.0032008},
{0.0126660,-0.0200115,0.0211026},
{0.0105136,-0.0344463,0.0201481},
{0.0149213,-0.0146037,0.0301881},
{-0.0159564,-0.0487215,0.0248157},
{0.0027610,-0.0380866,0.0604285},
{0.0154371,-0.0448599,0.0359619},
{0.0133431,-0.0100607,0.0124200},

{0.0009423,0.0043629,-0.0181755},
{0.0079634,-0.0007580,-0.0180373},
{0.0095377,-0.0669463,0.0502489},
{0.0136217,-0.0604541,0.0477345},
{0.0159995,-0.0587368,0.0396918},
{0.0161825,-0.0679973,0.0335669},
{-0.0043095,-0.0741897,0.0229721},
{-0.0121074,0.0037042,0.0130612},
{0.0070450,-0.0011130,0.0515776},
{0.0175287,-0.0092733,0.0470143},
{0.0136662,-0.0234382,0.0562557},
{-0.0108685,-0.0354352,0.0236725},
{-0.0167529,-0.0074210,-0.0023867},
{0.0149057,-0.0819262,0.0255869},
{-0.0063627,-0.0056850,-0.0177537},
{0.0037516,0.0139531,-0.0138318},
{-0.0085184,-0.0327042,0.0149349},
{-0.0135587,0.0102048,-0.0097181},
{-0.0184025,0.0049076,-0.0015510},
{-0.0083176,-0.0693211,0.0369128},
{0.0106521,-0.0172263,-0.0008855},
{0.0114644,-0.0449247,0.0535601},
{0.0079585,-0.0182646,-0.0083900},
{0.0092230,-0.0137554,-0.0128590},
{0.0039493,-0.0587102,0.0176869},
{0.0082230,-0.0500830,0.0200395},
{0.0011238,-0.0750818,0.0187358},
{-0.0167293,-0.0553962,0.0281862},
{-0.0055616,-0.0013429,0.0486990},
{-0.0120595,-0.0303987,0.0555482},
{0.0165144,-0.0171292,0.0505306},
{0.0138167,-0.0759696,0.0450637},
{0.0173092,-0.0236953,0.0327538},
{0.0077005,-0.0314379,0.0621256},
{-0.0156834,-0.0032076,0.0092745},
{0.0132996,-0.0159319,0.0562265},
{0.0142838,-0.0604781,0.0304611},
{-0.0140300,-0.0495044,0.0210445},
{-0.0129724,-0.0329429,0.0491580},
{0.0128270,-0.0411078,0.0285616},
{-0.0072187,-0.0382487,0.0601490},
{0.0061300,-0.0076454,-0.0176148},
{0.0038223,-0.0152338,-0.0141654},
{-0.0133040,-0.0106516,0.0481183},
{-0.0096502,-0.0442140,0.0541564},
{0.0161242,-0.0533922,0.0441862},

{-0.0156133,0.0110312,0.0014663},
{-0.0127723,0.0090599,0.0108941},
{-0.0017614,-0.0671824,0.0512529},
{0.0122862,-0.0091759,0.0318947},
{0.0128444,-0.0034619,0.0515574},
{0.0141256,-0.0056746,0.0404035},
{-0.0118366,-0.0132950,-0.0080490},
{0.0053050,-0.0216999,0.0022023},
{0.0003418,-0.0484675,0.0159542},
{0.0185526,0.0030942,-0.0071111},
{0.0159020,0.0016054,-0.0123302},
{0.0118597,-0.0076253,-0.0147885},
{-0.0121715,0.0013569,-0.0144687},
{-0.0135909,-0.0059092,-0.0123980},
{0.0164055,0.0130813,0.0030031},
{0.0098027,0.0188623,-0.0021496},
{0.0083674,0.0137220,-0.0123909},
{0.0074769,-0.0223391,0.0604625},
{-0.0006593,-0.0085333,0.0596341},
{-0.0001538,-0.0020680,0.0537435},
{0.0049927,-0.0135648,0.0610058},
{0.0077959,0.0072674,0.0155171},
{0.0114423,-0.0172081,0.0147144},
{0.0036573,-0.0250000,0.0620342},
{-0.0084734,-0.0170876,0.0229152},
{-0.0094969,-0.0172702,0.0114391},
{-0.0060193,-0.0098906,0.0255878},
{-0.0140126,-0.0501615,0.0334743},
{-0.0105578,-0.0606398,0.0387460},
{-0.0064442,-0.0287821,0.0088575},
{0.0192618,-0.0183031,0.0437267},
{0.0113819,-0.0083733,0.0261874},
{0.0040221,-0.0009361,0.0229429},
{-0.0033351,-0.0752326,0.0498851},
{-0.0022379,0.0217088,-0.0030914},
{-0.0010637,-0.0130828,0.0369914},
{-0.0101770,-0.0142180,0.0023291},
{0.0120638,-0.0285334,0.0242454},
{0.0096126,-0.0269112,0.0162002},
{-0.0102426,-0.0068867,0.0497947},
{0.0174390,-0.0328356,0.0453571},
{-0.0112391,-0.0530645,0.0392889},
{-0.0069556,-0.0802035,0.0356191},
{0.0040341,-0.0052035,0.0291718},
{-0.0027823,-0.0045673,-0.0188781},
{-0.0117433,-0.0344605,0.0551314},

{-0.0028024,0.0088279,-0.0159590},
{-0.0040239,0.0164587,-0.0117857},
{0.0173291,-0.0851364,0.0343393},
{0.0034386,-0.0734205,0.0179961},
{0.0153287,-0.0878406,0.0422141},
{0.0089280,0.0190448,0.0050877},
{0.0109361,0.0090790,0.0137818},
{0.0010231,0.0149588,-0.0135247},
{0.0149706,-0.0116002,-0.0083653},
{0.0176302,-0.0075876,-0.0071738},
{-0.0081006,-0.0241792,0.0287684},
{-0.0101525,-0.0343835,0.0304094},
{-0.0124789,-0.0614135,0.0243258},
{-0.0013182,0.0026495,0.0189991},
{0.0038672,0.0072902,0.0163910},
{-0.0023420,-0.0028404,0.0242719},
{0.0002390,-0.0306874,0.0615637},
{0.0064227,-0.0432597,0.0566578},
{-0.0108774,-0.0197799,0.0593510},
{-0.0108523,-0.0262309,0.0586232},
{0.0084023,0.0201837,0.0025047},
{0.0158937,0.0140981,0.0013290},
{-0.0066221,-0.0108767,0.0604277},
{0.0141491,-0.0529779,0.0325118},
{-0.0115313,-0.0474039,0.0454495},
{-0.0139469,-0.0502715,0.0208185},
{0.0101811,-0.0840299,0.0478670},
{-0.0091023,-0.0251593,0.0167484},
{-0.0093821,-0.0153209,0.0161037},
{-0.0041110,0.0159423,0.0119511},
{0.0008306,-0.0679297,0.0529183},
{0.0054678,-0.0626280,0.0530810},
{-0.0020970,-0.0623174,0.0526033},
{0.0153877,-0.0734345,0.0293818},
{-0.0115642,-0.0182323,0.0463033},
{0.0099054,-0.0247725,0.0163028},
{0.0047846,-0.0523180,0.0554163},
{0.0077091,-0.0349649,0.0589176},
{0.0088551,-0.0839814,0.0187731},
{-0.0062918,-0.0526965,0.0538054},
{0.0166394,-0.0426369,0.0415858},
{-0.0110627,-0.0426195,0.0200014},
{0.0196653,-0.0205286,0.0353775},
{-0.0037392,-0.0606745,0.0191871},
{0.0154501,-0.0019814,0.0120689},
{0.0057281,0.0053758,0.0165321},

Biology Open • Supplementary information

{0.0008500,-0.0322623,0.0614523},
{-0.0137553,0.0136450,-0.0047946},
{0.0098432,0.0155860,0.0096012},
{-0.0099024,0.0159025,0.0077040},
{-0.0172680,-0.0054575,-0.0048780},
{-0.0173709,0.0005048,-0.0069973},
{-0.0045478,-0.0173635,0.0626985},
{-0.0122018,-0.0340707,0.0411092},
{0.0051844,-0.0464891,0.0566311},
{0.0115389,-0.0412880,0.0545119},
{-0.0081114,-0.0677274,0.0239750},
{0.0117218,0.0070277,0.0141925},
{0.0169194,-0.0106024,-0.0010595},
{-0.0160763,-0.0079660,0.0047226},
{-0.0118551,-0.0557260,0.0364289},
{-0.0067862,-0.0166791,-0.0035060},
{-0.0120043,-0.0272752,0.0440636},
{0.0043179,-0.0741040,0.0514557},
{0.0148240,-0.0493959,0.0485098},
{-0.0016103,-0.0344851,0.0103149},
{0.0057978,-0.0316682,0.0122949},
{0.0174222,-0.0068941,0.0054067},
{0.0168224,-0.0805489,0.0390058},
{-0.0084757,-0.0187225,0.0234833},
{-0.0008156,0.0128134,0.0150291},
{0.0068713,-0.0674924,0.0197946},
{0.0081614,-0.0616758,0.0524786},
{0.0139401,-0.0128184,-0.0088097},
{0.0005159,-0.0089987,0.0600785},
{-0.0066359,-0.0145776,-0.0132087},
{0.0147515,-0.0696786,0.0438172},
{0.0071352,-0.0182011,-0.0096410},
{-0.0098507,-0.0637090,0.0216450},
{0.0123535,-0.0388534,0.0270478},
{0.0143993,-0.0042265,0.0502024},
{0.0044023,-0.0075467,0.0365939},
{-0.0028174,-0.0610119,0.0531227},
{-0.0118809,-0.0588063,0.0354761},
{-0.0134123,-0.0112835,0.0502358},
{-0.0072929,0.0121154,-0.0136193},
{-0.0159312,0.0057130,0.0075757},
{0.0086738,-0.0672272,0.0206366},
{0.0023140,-0.0383787,0.0595802},
{-0.0125290,-0.0213931,0.0511031},
{0.0059751,-0.0060507,-0.0182666},
{0.0142933,-0.0278644,0.0525299},

{0.0111000,-0.0147643,0.0576417},
{0.0096677,0.0020387,-0.0168250},
{0.0127175,-0.0079801,0.0159781},
{-0.0120056,0.0108821,0.0108670},
{0.0096108,-0.0516302,0.0535074},
{-0.0050709,-0.0374938,0.0609148},
{0.0180380,-0.0283709,0.0465221},
{0.0147019,-0.0129140,-0.0065163},
{-0.0138432,-0.0489891,0.0339402},
{-0.0070058,-0.0719309,0.0420789},
{0.0029682,-0.0513328,0.0561035},
{-0.0040954,-0.0403913,0.0130483},
{0.0103315,0.0104239,-0.0136039},
{-0.0019883,-0.0210660,-0.0007860},
{-0.0085048,-0.0714517,0.0281430},
{-0.0095821,-0.0034131,-0.0163607},
{-0.0027054,0.0211192,-0.0043523},
{-0.0088947,-0.0211578,0.0123545},
{-0.0099651,-0.0627664,0.0214797},
{-0.0055172,-0.0727971,0.0231259},
{0.0008843,0.0115484,-0.0155632},
{0.0184120,-0.0253112,0.0458065},
{-0.0054699,-0.0564873,0.0185570},
{0.0163588,-0.0353568,0.0370195},
{0.0163351,-0.0299710,0.0329823},
{-0.0133245,-0.0116445,-0.0009141},
{0.0123152,0.0136437,0.0094656},
{-0.0124233,-0.0106200,0.0065013},
{-0.0099280,-0.0342535,0.0335597},
{0.0166670,-0.0853763,0.0398368},
{0.0164103,-0.0100530,0.0522934},
{0.0174182,-0.0151368,0.0484459},
{0.0012641,-0.0257542,0.0047157},
{0.0100176,-0.0227107,0.0589978},
{-0.0025898,-0.0092739,0.0294853},
{-0.0178980,-0.0041246,-0.0016325},
{0.0025603,-0.0204194,-0.0035775},
{-0.0089913,-0.0691025,0.0347025},
{-0.0118267,-0.0266922,0.0572066},
{0.0166110,0.0050397,-0.0101060},
{-0.0137133,-0.0604575,0.0282866},
{-0.0141421,-0.0040768,0.0120114},
{0.0111361,-0.0078018,0.0253998},
{-0.0124694,-0.0415881,0.0300852},
{0.0139706,-0.0694544,0.0270280},
{0.0007143,0.0032695,-0.0184127},

{0.0156736,-0.0751892,0.0294906},
{0.0064452,0.0061902,0.0158407},
{-0.0132627,0.0021920,0.0121625},
{0.0054316,-0.0416285,0.0155919},
{-0.0060117,-0.0184515,0.0319557},
{0.0190799,-0.0261407,0.0403651},
{-0.0119826,-0.0606963,0.0215982},
{0.0193634,0.0049584,-0.0028606},
{-0.0128836,-0.0608571,0.0263167},
{0.0104597,-0.0216870,0.0104918},
{0.0183283,-0.0113353,0.0430617},
{0.0080435,0.0018860,-0.0177145},
{0.0137002,-0.0080500,0.0128413},
{-0.0105322,-0.0615999,0.0381348},
{0.0004302,0.0199572,0.0087433},
{0.0026396,0.0032015,-0.0187145},
{0.0350564,-0.3906540,0.0199985},
{0.0047509,0.0197753,-0.0074096},
{-0.0126942,-0.0436037,0.0355161},
{-0.0295007,-0.3866978,-0.0256255},
{0.0021775,0.0223723,-0.0000304},
{0.0135059,-0.0237696,0.0253259},
{-0.0132663,-0.0010658,-0.0138399},
{0.0155569,-0.0300151,0.0504833},
{-0.0137713,0.0130274,0.0045177},
{0.0162187,-0.0388298,0.0381509},
{0.0144746,-0.0433184,0.0327237},
{-0.0124729,-0.0341686,0.0436108},
{0.0162860,-0.0548665,0.0419723},
{-0.0157200,-0.0476782,0.0261931},
{0.0028722,-0.0544711,0.0553893},
{0.0010221,-0.0508142,0.0165418},
{0.0201222,-0.0207050,0.0419589},
{0.0074292,-0.0756616,0.0502085},
{-0.0055895,-0.0821432,0.0266136},
{-0.0178333,-0.0021318,0.0029091},
{0.0135174,-0.0132719,0.0188061},
{0.0130009,-0.0659477,0.0261675},
{-0.0087276,-0.0244484,0.0262076},
{-0.0183097,-0.3964755,0.0340043},
{-0.0081247,-0.0552147,0.0496913},
{-0.0107507,-0.3911153,-0.0097891},
{0.0100197,-0.0428073,0.0213294},
{0.0101833,0.0143680,-0.0106590},
{-0.0228617,-0.3878880,0.0275144},
{0.0146773,-0.0525892,0.0333508},

Biology Open • Supplementary information

{0.0002799,-0.0490384,0.0569970},
{-0.0103028,0.0179044,0.0027083},
{-0.0023145,-0.0165239,0.0625655},
{-0.0068423,-0.0072924,0.0229617},
{0.0181761,-0.0101977,0.0401910},
{0.0139195,-0.0242363,0.0544500},
{-0.0132542,-0.0107932,0.0495389},
{0.0185773,0.0081501,-0.0026720},
{-0.0093966,-0.0337627,0.0170648},
{-0.0003157,-0.0139759,-0.0156472},
{-0.0100999,-0.0596023,0.0420391},
{-0.0084399,0.0169458,0.0076329},
{0.0091490,0.0189426,-0.0028563},
{-0.0125547,-0.0267563,0.0542509},
{-0.0056052,-0.3883713,0.0385439},
{-0.0125872,-0.3936516,-0.0361259},
{-0.0125174,-0.3935988,-0.0353792},
{-0.0124476,-0.3935460,-0.0346325},
{-0.0123778,-0.3934932,-0.0338858},
{-0.0123079,-0.3934404,-0.0331391},
{-0.0122381,-0.3933876,-0.0323924},
{-0.0121683,-0.3933348,-0.0316458},
{-0.0120985,-0.3932820,-0.0308991},
{-0.0120287,-0.3932292,-0.0301524},
{-0.0119588,-0.3931764,-0.0294057},
{-0.0118890,-0.3931235,-0.0286590},
{-0.0118192,-0.3930707,-0.0279123},
{-0.0117494,-0.3930179,-0.0271656},
{-0.0116795,-0.3929651,-0.0264189},
{-0.0116097,-0.3929123,-0.0256722},
{-0.0115399,-0.3928595,-0.0249255},
{-0.0114701,-0.3928067,-0.0241788},
{-0.0114003,-0.3927539,-0.0234321},
{-0.0113304,-0.3927011,-0.0226854},
{-0.0112606,-0.3926483,-0.0219387},
{-0.0111908,-0.3925955,-0.0211920},
{-0.0111210,-0.3925427,-0.0204453},
{-0.0110512,-0.3924899,-0.0196986},
{-0.0109813,-0.3924371,-0.0189519},
{-0.0109115,-0.3923843,-0.0182052},
{-0.0108417,-0.3923315,-0.0174585},
{-0.0107719,-0.3922787,-0.0167118},
{-0.0107021,-0.3922259,-0.0159651},
{-0.0106322,-0.3921731,-0.0152184},
{-0.0105624,-0.3921203,-0.0144717},
{-0.0104926,-0.3920675,-0.0137250},

{-0.0104228,-0.3920147,-0.0129783},
{-0.0103530,-0.3919619,-0.0122316},
{-0.0102831,-0.3919091,-0.0114849},
{-0.0102133,-0.3918563,-0.0107382},
{-0.0101435,-0.3918035,-0.0099915},
{-0.0100737,-0.3917507,-0.0092448},
{-0.0100039,-0.3916979,-0.0084981},
{-0.0099340,-0.3916451,-0.0077514},
{-0.0098642,-0.3915923,-0.0070047},
{-0.0097944,-0.3915395,-0.0062580},
{-0.0097246,-0.3914867,-0.0055113},
{-0.0096548,-0.3914339,-0.0047646},
{-0.0095849,-0.3913811,-0.0040179},
{-0.0095151,-0.3913283,-0.0032712},
{-0.0094453,-0.3912755,-0.0025245},
{-0.0093755,-0.3912226,-0.0017778},
{-0.0093057,-0.3911698,-0.0010311},
{-0.0092358,-0.3911170,-0.0002844},
{-0.0091660,-0.3910642,0.0004623},
{-0.0090962,-0.3910114,0.0012090},
{-0.0090264,-0.3909586,0.0019557},
{-0.0089566,-0.3909058,0.0027024},
{-0.0088867,-0.3908530,0.0034491},
{-0.0088169,-0.3908002,0.0041958},
{-0.0087471,-0.3907474,0.0049425},
{-0.0086773,-0.3906946,0.0056892},
{-0.0086075,-0.3906418,0.0064359},
{-0.0085376,-0.3905890,0.0071826},
{-0.0084678,-0.3905362,0.0079293},
{-0.0083980,-0.3904834,0.0086760},
{-0.0083282,-0.3904306,0.0094227},
{-0.0082584,-0.3903778,0.0101694},
{-0.0081885,-0.3903250,0.0109161},
{-0.0081187,-0.3902722,0.0116628},
{-0.0080489,-0.3902194,0.0124095},
{-0.0079791,-0.3901666,0.0131562},
{-0.0079093,-0.3901138,0.0139029},
{-0.0078394,-0.3900610,0.0146496},
{-0.0077696,-0.3900082,0.0153963},
{-0.0076998,-0.3899554,0.0161429},
{-0.0076300,-0.3899026,0.0168896},
{-0.0075602,-0.3898498,0.0176363},
{-0.0074903,-0.3897970,0.0183830},
{-0.0074205,-0.3897442,0.0191297},
{-0.0073507,-0.3896914,0.0198764},
{-0.0072809,-0.3896386,0.0206231},

Biology Open • Supplementary information

```
                    {-0.0072111,-0.3895858,0.0213698},
                    {-0.0071412,-0.3895330,0.0221165},
                    {-0.0070714,-0.3894802,0.0228632},
                    {-0.0070016,-0.3894274,0.0236099},
                    {-0.0069318,-0.3893746,0.0243566},
                    {-0.0068619,-0.3893217,0.0251033},
                    {-0.0067921,-0.3892689,0.0258500},
                    {-0.0067223,-0.3892161,0.0265967},
                    {-0.0066525,-0.3891633,0.0273434},
                    {-0.0065827,-0.3891105,0.0280901},
                    {-0.0065128,-0.3890577,0.0288368},
                    {-0.0064430,-0.3890049,0.0295835},
                    {-0.0063732,-0.3889521,0.0303302},
                    {-0.0063034,-0.3888993,0.0310769},
                    {-0.0062336,-0.3888465,0.0318236},
                    {-0.0061637,-0.3887937,0.0325703},
                    {-0.0060939,-0.3887409,0.0333170},
                    {-0.0060241,-0.3886881,0.0340637},
                    {-0.0059543,-0.3886353,0.0348104},
                    {-0.0058845,-0.3885825,0.0355571},
                    {-0.0058146,-0.3885297,0.0363038},
                    {-0.0057448,-0.3884769,0.0370505},
                    {-0.0056750,-0.3884241,0.0377972}
                    };
            BoundingBoxOnOff = Off;
        };
        AnyFunTransform3DIdentity ScaleFunction = {
            PreTransforms = {&.RBFTransform};
};
};
};

};
```

Biology Open • Supplementary information

ScalingFunctionTLEMLucyFemur_Mirrored_2014014

```
AnyFolder ScalingFunctionTLEMLucyFemur_Mirrored = {
  AnyFolder Left = {
    AnyFolder Thigh = {
      AnyMatrix AMirroring = {
        {1,0,0},
        {0,1,0},
        {0,0,-1}
      };
          AnyFunTransform3DRBF RBFTransform = {
                    RBFDef.Type = RBF_ThinPlate;
                    PolynomDegree = 1;
                    Points0 = ({
                              {0.0000000,0.0000000,0.0000000},
                              {-0.0000000,-0.3616821,0.0000000},
                              {-0.0097563,-0.3678799,0.0012967},
                              {-0.0000000,-0.3660632,0.0408203},
                              {-0.0000000,-0.3573010,-0.0408203},
                              {0.0161460,-0.0072838,0.0601290},
                              {0.0220217,-0.0203698,0.0463848},
                              {0.0123977,-0.0241932,0.0668573},
                              {0.0006898,0.0018121,0.0538181},
                              {0.0122809,-0.0068668,0.0414535},
                              {-0.0058991,-0.0138188,0.0648412},
                              {0.0211469,-0.0380855,0.0538111},
                              {0.0172133,-0.0317342,0.0328381},
                              {0.0177311,-0.0131946,0.0256176},
                              {-0.0110079,-0.0308867,0.0632370},
                              {0.0039435,-0.0395977,0.0638271},
                              {-0.0041683,-0.0187472,0.0413214},
                              {-0.0177999,-0.0021535,0.0496084},
                              {0.0001603,-0.0053126,0.0279593},
                              {-0.0211600,-0.0216243,0.0497147},
                              {0.0096163,-0.0568663,0.0545719},
                              {0.0162112,-0.0515013,0.0395230},
                              {0.0093533,-0.0433265,0.0187387},
                              {0.0119398,-0.0238469,0.0118403},
                              {0.0210707,-0.0048848,0.0065878},
                              {0.0125013,0.0064094,0.0177702},
                              {-0.0191523,-0.0356053,0.0382429},
                              {-0.0115821,-0.0474937,0.0537648},
                              {-0.0100073,-0.0236371,0.0267322},
                              {-0.0078925,0.0107098,0.0182833},
                              {-0.0134217,-0.0055356,0.0182000},
                              {-0.0090061,-0.0665700,0.0475484},
```

Biology Open • Supplementary information

{0.0071095,-0.0761976,0.0520035},
{0.0182119,-0.0704149,0.0386546},
{0.0073764,-0.0609481,0.0221006},
{-0.0122984,-0.0439585,0.0198428},
{-0.0032253,-0.0306792,0.0110995},
{-0.0012975,-0.0198451,-0.0052592},
{0.0140405,-0.0161125,-0.0071649},
{0.0189222,-0.0014898,-0.0127226},
{0.0181642,0.0132812,-0.0011050},
{0.0045119,0.0200001,0.0091189},
{-0.0176761,-0.0541983,0.0348587},
{-0.0136548,-0.0142015,0.0054311},
{-0.0135510,0.0182940,0.0017175},
{-0.0223865,0.0023399,0.0028018},
{-0.0086455,-0.0738247,0.0283843},
{-0.0050356,-0.0877749,0.0442195},
{0.0160135,-0.0919319,0.0465120},
{0.0128290,-0.0785810,0.0232431},
{-0.0168328,-0.0616327,0.0155917},
{-0.0172369,-0.0094567,-0.0119502},
{0.0002090,-0.0093641,-0.0209779},
{0.0064827,0.0081538,-0.0198453},
{0.0010777,0.0210422,-0.0084721},
{-0.0129485,0.0085451,-0.0162212},
{-0.0002322,-0.0924033,0.0245644},
{-0.0028175,-0.1052263,0.0418294},
{0.0172322,-0.1111444,0.0445760},
{0.0203259,-0.0957409,0.0271449},
{0.0057102,-0.1088933,0.0195113},
{-0.0010852,-0.1230776,0.0363132},
{0.0182727,-0.1303312,0.0422884},
{0.0242891,-0.1161703,0.0265054},
{0.0094531,-0.1268584,0.0162294},
{0.0014244,-0.1413293,0.0331653},
{0.0207271,-0.1496427,0.0394157},
{0.0267562,-0.1360142,0.0236798},
{0.0113839,-0.1461651,0.0136024},
{0.0030845,-0.1595094,0.0297156},
{0.0209528,-0.1691386,0.0369775},
{0.0283958,-0.1540209,0.0213795},
{0.0129499,-0.1649012,0.0113589},
{0.0016490,-0.1783108,0.0216287},
{0.0147105,-0.1880678,0.0328614},
{0.0298491,-0.1859865,0.0248551},
{0.0286645,-0.1714377,0.0169488},
{0.0165897,-0.1861006,0.0082595},

Biology Open • Supplementary information

{0.0014000,-0.1973476,0.0183740},
{0.0114301,-0.2109792,0.0286157},
{0.0267422,-0.2037622,0.0296951},
{0.0288630,-0.2018335,0.0130127},
{0.0145471,-0.2083505,0.0062833},
{0.0011300,-0.2198071,0.0155602},
{0.0126610,-0.2321235,0.0275354},
{0.0275647,-0.2219268,0.0265185},
{0.0286645,-0.2230253,0.0098701},
{0.0130434,-0.2304822,0.0039177},
{0.0022984,-0.2409585,0.0149650},
{0.0101315,-0.2534740,0.0252125},
{0.0279485,-0.2428555,0.0234276},
{0.0276684,-0.2421559,0.0058691},
{0.0101250,-0.2529527,0.0018983},
{0.0021376,-0.2667831,0.0107073},
{0.0114309,-0.2734905,0.0249960},
{0.0273923,-0.2634953,0.0216324},
{0.0278429,-0.2592546,0.0037692},
{0.0159101,-0.2712985,-0.0033988},
{0.0036708,-0.2863280,0.0004644},
{0.0023115,-0.2884756,0.0187554},
{0.0231385,-0.2879051,0.0224853},
{0.0304009,-0.2799973,0.0083549},
{0.0238189,-0.2917246,-0.0052462},
{0.0064904,-0.3027687,-0.0070587},
{-0.0003492,-0.3064690,0.0093014},
{0.0122179,-0.3045269,0.0243511},
{0.0301837,-0.3030127,0.0114462},
{0.0234182,-0.3107336,-0.0085198},
{0.0046893,-0.3214995,-0.0123881},
{-0.0022352,-0.3264905,0.0043340},
{0.0016841,-0.3202756,0.0217839},
{0.0229722,-0.3196887,0.0215083},
{0.0292438,-0.3228042,0.0051430},
{0.0221641,-0.3296190,-0.0132361},
{0.0100459,-0.3417597,-0.0244560},
{-0.0053009,-0.3375737,-0.0137705},
{-0.0064432,-0.3487307,0.0028676},
{-0.0059346,-0.3407801,0.0202938},
{0.0105130,-0.3365976,0.0264175},
{0.0282695,-0.3386249,0.0180307},
{0.0270402,-0.3431844,-0.0001487},
{0.0257040,-0.3506950,-0.0181851},
{0.0178948,-0.3636114,-0.0302838},
{0.0043149,-0.3544109,-0.0381949},

Biology Open • Supplementary information

{-0.0095210,-0.3461482,-0.0295031},
{-0.0164526,-0.3542895,-0.0126666},
{-0.0060896,-0.3690127,-0.0033061},
{-0.0176523,-0.3612686,0.0110109},
{-0.0153310,-0.3546943,0.0299634},
{0.0021329,-0.3508021,0.0350763},
{0.0210690,-0.3548628,0.0279835},
{0.0356594,-0.3558162,0.0159356},
{0.0275718,-0.3617450,-0.0018607},
{0.0298503,-0.3741530,-0.0163721},
{0.0175320,-0.3855300,-0.0277515},
{0.0025604,-0.3740099,-0.0397137},
{-0.0136727,-0.3624016,-0.0402102},
{-0.0277647,-0.3567472,-0.0284947},
{-0.0275446,-0.3699630,-0.0120829},
{-0.0175846,-0.3859355,-0.0177922},
{0.0001572,-0.3856873,-0.0118050},
{0.0030262,-0.3834856,0.0075553},
{-0.0144114,-0.3810887,0.0140304},
{-0.0247706,-0.3701239,0.0285134},
{-0.0044804,-0.3677201,0.0404632},
{0.0133183,-0.3712274,0.0334208},
{0.0324861,-0.3723417,0.0245668},
{0.0271451,-0.3766027,0.0072441},
{0.0167985,-0.3822816,-0.0065349},
{-0.0001454,-0.3910823,-0.0303010},
{-0.0144998,-0.3834154,-0.0384137},
{-0.0301321,-0.3750841,-0.0321336},
{0.0196491,-0.3864804,0.0210853},
{0.0015726,-0.3888034,0.0276569},
{-0.0146652,-0.3847848,0.0336913},
{-0.0014588,-0.0619951,0.0219778},
{-0.0118435,-0.0550626,0.0139639},
{0.0129812,-0.0349966,0.0673128},
{-0.0162907,-0.0105666,0.0597739},
{-0.0012885,-0.0520816,0.0562989},
{-0.0090599,-0.0631298,0.0172224},
{-0.0000222,-0.0771663,0.0231897},
{-0.0068148,0.0023885,-0.0160086},
{-0.0180544,-0.0425409,0.0446585},
{0.0020525,-0.0249904,0.0691549},
{-0.0157097,-0.0662315,0.0238393},
{-0.0010518,-0.0510686,0.0199353},
{-0.0208874,-0.0152865,0.0500034},
{0.0168316,-0.0280857,0.0666680},
{-0.0187392,-0.0282333,0.0403282},

{0.0191622,-0.0351633,0.0622707},
{0.0001216,-0.0706289,0.0524049},
{-0.0169862,-0.0399062,0.0311827},
{-0.0185737,-0.0533066,0.0151224},
{-0.0219058,-0.0528723,0.0222263},
{-0.0028271,-0.0391620,0.0148452},
{0.0117900,-0.0553425,0.0235348},
{0.0219835,-0.0106400,0.0396338},
{-0.0025366,-0.0401046,0.0625273},
{0.0141017,-0.0018767,0.0198108},
{-0.0078770,-0.0473929,0.0173542},
{-0.0065351,-0.0766431,0.0470258},
{0.0065585,0.0145593,0.0161638},
{-0.0118588,0.0172875,-0.0085064},
{-0.0074087,-0.0627500,0.0506700},
{0.0071940,-0.0780068,0.0213362},
{0.0126736,-0.0708176,0.0244108},
{0.0074694,-0.0436450,0.0604196},
{0.0154186,-0.0139471,0.0064312},
{-0.0053535,-0.0213210,0.0040094},
{-0.0088733,0.0002018,0.0205402},
{-0.0112471,-0.0542608,0.0500266},
{-0.0061625,-0.0248673,0.0653244},
{0.0093695,-0.0065826,0.0614157},
{0.0138015,-0.0467322,0.0284333},
{-0.0020999,-0.0186885,-0.0116392},
{-0.0185640,-0.0225591,0.0566779},
{0.0175798,0.0124360,-0.0068467},
{-0.0039962,0.0217127,0.0053055},
{0.0014975,-0.0784591,0.0514056},
{-0.0172997,-0.0386971,0.0525252},
{0.0196540,-0.0268271,0.0416806},
{0.0135296,-0.0406504,0.0236882},
{0.0182471,0.0095959,0.0087906},
{0.0218667,0.0016804,0.0058793},
{0.0224975,-0.0022652,-0.0022994},
{0.0166750,-0.0191391,0.0236436},
{0.0119785,-0.0328723,0.0195376},
{0.0187631,-0.0133469,0.0308741},
{-0.0187543,-0.0461463,0.0257491},
{0.0039834,-0.0373928,0.0683089},
{0.0157029,-0.0423169,0.0385226},
{0.0164003,-0.0087069,0.0131181},
{0.0008253,0.0033370,-0.0212576},
{0.0101707,-0.0009724,-0.0205688},
{0.0102948,-0.0642683,0.0524532},

Biology Open • Supplementary information

{0.0152217,-0.0557944,0.0508978},
{0.0172973,-0.0565707,0.0419468},
{0.0167723,-0.0635334,0.0369834},
{-0.0047397,-0.0695325,0.0241398},
{-0.0152913,0.0035778,0.0161461},
{0.0091697,0.0009340,0.0575160},
{0.0196686,-0.0089533,0.0500006},
{0.0183998,-0.0212943,0.0622968},
{-0.0114340,-0.0316810,0.0250940},
{-0.0206442,-0.0092599,-0.0028425},
{0.0166868,-0.0784295,0.0276925},
{-0.0069678,-0.0054521,-0.0207011},
{0.0049490,0.0151109,-0.0160093},
{-0.0084025,-0.0318937,0.0162788},
{-0.0173679,0.0095594,-0.0108509},
{-0.0218912,0.0063457,-0.0020698},
{-0.0106547,-0.0668750,0.0397749},
{0.0136098,-0.0170666,-0.0018890},
{0.0141599,-0.0441405,0.0587594},
{0.0093255,-0.0183218,-0.0083008},
{0.0106367,-0.0128276,-0.0154269},
{0.0037350,-0.0549365,0.0209786},
{0.0087662,-0.0472902,0.0195496},
{0.0016839,-0.0708213,0.0221325},
{-0.0202977,-0.0530540,0.0301270},
{-0.0068584,0.0021781,0.0530002},
{-0.0144817,-0.0297218,0.0605833},
{0.0197015,-0.0165217,0.0560654},
{0.0156609,-0.0705363,0.0477736},
{0.0207876,-0.0221433,0.0330733},
{0.0092324,-0.0300767,0.0713160},
{-0.0203392,-0.0034488,0.0097332},
{0.0156978,-0.0143438,0.0629053},
{0.0143267,-0.0563519,0.0317489},
{-0.0161685,-0.0465391,0.0188591},
{-0.0196397,-0.0309875,0.0546496},
{0.0154804,-0.0380422,0.0305433},
{-0.0077774,-0.0351303,0.0637765},
{0.0063980,-0.0086419,-0.0201654},
{0.0030146,-0.0151094,-0.0165974},
{-0.0210080,-0.0096030,0.0507855},
{-0.0105684,-0.0411377,0.0586146},
{0.0180619,-0.0498960,0.0469835},
{-0.0192318,0.0116108,0.0024070},
{-0.0165651,0.0083622,0.0126870},
{-0.0014333,-0.0627138,0.0534746},

Biology Open • Supplementary information

{0.0145127,-0.0080178,0.0353667},
{0.0148374,-0.0012228,0.0566181},
{0.0153834,-0.0062427,0.0444727},
{-0.0155164,-0.0132103,-0.0096398},
{0.0068685,-0.0221022,0.0011547},
{0.0003332,-0.0458500,0.0183716},
{0.0205582,0.0040956,-0.0082969},
{0.0175752,0.0009340,-0.0148407},
{0.0142357,-0.0063623,-0.0169332},
{-0.0147285,0.0023412,-0.0164678},
{-0.0158781,-0.0064451,-0.0151346},
{0.0175951,0.0133456,0.0037559},
{0.0117660,0.0189899,-0.0030690},
{0.0096040,0.0151827,-0.0137450},
{0.0096565,-0.0215194,0.0662734},
{-0.0006793,-0.0074494,0.0631218},
{0.0007819,-0.0004519,0.0600781},
{0.0065377,-0.0125433,0.0654199},
{0.0090461,0.0073096,0.0188711},
{0.0151418,-0.0168302,0.0146465},
{0.0025166,-0.0231863,0.0688894},
{-0.0097891,-0.0152077,0.0253086},
{-0.0109149,-0.0177935,0.0119825},
{-0.0072079,-0.0099373,0.0272725},
{-0.0183727,-0.0467429,0.0356276},
{-0.0126871,-0.0584555,0.0428141},
{-0.0057054,-0.0273042,0.0103554},
{0.0215142,-0.0159693,0.0474531},
{0.0128492,-0.0070016,0.0287645},
{0.0052134,-0.0017047,0.0247463},
{-0.0024284,-0.0707498,0.0513049},
{-0.0036140,0.0220593,-0.0040484},
{-0.0002405,-0.0121253,0.0390050},
{-0.0119811,-0.0156737,0.0015445},
{0.0142082,-0.0279792,0.0246553},
{0.0122244,-0.0262013,0.0169829},
{-0.0146264,-0.0038855,0.0549384},
{0.0207450,-0.0325771,0.0496412},
{-0.0155040,-0.0497560,0.0437087},
{-0.0081119,-0.0756450,0.0387365},
{0.0039684,-0.0062047,0.0313900},
{-0.0023726,-0.0038892,-0.0220521},
{-0.0136977,-0.0339839,0.0602619},
{-0.0035399,0.0089081,-0.0188894},
{-0.0049820,0.0176571,-0.0132254},
{0.0193902,-0.0803191,0.0360429},

Biology Open • Supplementary information

{0.0040648,-0.0697109,0.0218205},
{0.0170470,-0.0818610,0.0456892},
{0.0104175,0.0188635,0.0064965},
{0.0135206,0.0092549,0.0158079},
{0.0023933,0.0160498,-0.0157404},
{0.0164753,-0.0118197,-0.0096995},
{0.0202584,-0.0066858,-0.0082383},
{-0.0098401,-0.0226001,0.0314028},
{-0.0130937,-0.0309622,0.0320764},
{-0.0201510,-0.0613297,0.0222264},
{-0.0010389,0.0036055,0.0209003},
{0.0042025,0.0073392,0.0199393},
{-0.0021630,-0.0038531,0.0251898},
{-0.0007845,-0.0293574,0.0675788},
{0.0070027,-0.0406327,0.0624565},
{-0.0124363,-0.0183001,0.0627966},
{-0.0120485,-0.0256613,0.0628493},
{0.0101200,0.0197695,0.0039334},
{0.0167636,0.0148266,0.0014155},
{-0.0074981,-0.0106930,0.0632797},
{0.0142533,-0.0503445,0.0351954},
{-0.0146107,-0.0460953,0.0506120},
{-0.0161117,-0.0471301,0.0178900},
{0.0112149,-0.0792793,0.0501460},
{-0.0099060,-0.0230794,0.0186951},
{-0.0114543,-0.0153049,0.0183675},
{-0.0042882,0.0161879,0.0151423},
{0.0014582,-0.0638572,0.0541080},
{0.0048554,-0.0582603,0.0553815},
{-0.0017077,-0.0572302,0.0546635},
{0.0166090,-0.0697169,0.0323038},
{-0.0208812,-0.0164273,0.0474354},
{0.0130454,-0.0241864,0.0178294},
{0.0053123,-0.0483770,0.0582251},
{0.0112225,-0.0361429,0.0673300},
{0.0085729,-0.0771256,0.0216100},
{-0.0070783,-0.0488033,0.0560869},
{0.0182083,-0.0411024,0.0454236},
{-0.0119590,-0.0403809,0.0213546},
{0.0233409,-0.0185535,0.0366343},
{-0.0046347,-0.0571211,0.0208243},
{0.0183166,-0.0006966,0.0140116},
{0.0061913,0.0053512,0.0201003},
{-0.0002364,-0.0310828,0.0680351},
{-0.0170773,0.0139270,-0.0054987},
{0.0110805,0.0156034,0.0117010},

Biology Open • Supplementary information

{-0.0124628,0.0164667,0.0094550},
{-0.0209014,-0.0073407,-0.0051969},
{-0.0210131,0.0010693,-0.0086891},
{-0.0041596,-0.0172332,0.0663041},
{-0.0216595,-0.0322172,0.0451540},
{0.0051908,-0.0426014,0.0613013},
{0.0150003,-0.0416136,0.0606257},
{-0.0135090,-0.0674234,0.0236425},
{0.0146416,0.0073691,0.0160052},
{0.0195615,-0.0112412,-0.0010333},
{-0.0198115,-0.0098244,0.0044735},
{-0.0157054,-0.0521236,0.0395039},
{-0.0081381,-0.0173108,-0.0046152},
{-0.0218324,-0.0262661,0.0471115},
{0.0051446,-0.0707257,0.0530555},
{0.0179359,-0.0471493,0.0528134},
{-0.0005591,-0.0317983,0.0113632},
{0.0071707,-0.0305943,0.0121186},
{0.0206239,-0.0061367,0.0066642},
{0.0187057,-0.0745868,0.0420207},
{-0.0095784,-0.0163113,0.0259524},
{-0.0012181,0.0127661,0.0186855},
{0.0059420,-0.0630691,0.0220087},
{0.0069408,-0.0573878,0.0552614},
{0.0150782,-0.0132884,-0.0101793},
{0.0005533,-0.0078574,0.0633775},
{-0.0088284,-0.0146399,-0.0147837},
{0.0167444,-0.0651105,0.0470347},
{0.0082666,-0.0181741,-0.0095133},
{-0.0175162,-0.0637444,0.0177069},
{0.0153795,-0.0360767,0.0287608},
{0.0162363,-0.0018864,0.0548412},
{0.0052731,-0.0075919,0.0392990},
{-0.0022288,-0.0556644,0.0549909},
{-0.0156238,-0.0550038,0.0377688},
{-0.0204137,-0.0100550,0.0536429},
{-0.0093683,0.0122429,-0.0162013},
{-0.0202927,0.0049166,0.0083887},
{0.0074706,-0.0627945,0.0222133},
{0.0035034,-0.0376103,0.0672421},
{-0.0190752,-0.0194129,0.0555530},
{0.0061758,-0.0074961,-0.0207741},
{0.0192011,-0.0273063,0.0588233},
{0.0130796,-0.0134383,0.0638611},
{0.0120421,0.0019566,-0.0194140},
{0.0154152,-0.0065294,0.0179581},

Biology Open • Supplementary information

{-0.0157335,0.0101219,0.0123981},
{0.0110376,-0.0488274,0.0567911},
{-0.0055170,-0.0343676,0.0647140},
{0.0216204,-0.0273988,0.0512041},
{0.0161331,-0.0135875,-0.0075501},
{-0.0184643,-0.0455633,0.0360087},
{-0.0091978,-0.0682413,0.0460847},
{0.0034304,-0.0471677,0.0588866},
{-0.0048025,-0.0384316,0.0149400},
{0.0118505,0.0109735,-0.0159161},
{-0.0027036,-0.0204189,-0.0015094},
{-0.0117607,-0.0692759,0.0292171},
{-0.0107080,-0.0033455,-0.0190487},
{-0.0042548,0.0214947,-0.0056846},
{-0.0097315,-0.0208529,0.0131997},
{-0.0178958,-0.0627218,0.0171551},
{-0.0068692,-0.0688818,0.0233316},
{0.0012083,0.0120695,-0.0186179},
{0.0215073,-0.0236027,0.0503069},
{-0.0060262,-0.0534931,0.0189523},
{0.0179951,-0.0336785,0.0397267},
{0.0190509,-0.0284972,0.0336394},
{-0.0169188,-0.0133609,-0.0014203},
{0.0135722,0.0136491,0.0114695},
{-0.0150549,-0.0123230,0.0067722},
{-0.0148411,-0.0311945,0.0355412},
{0.0185490,-0.0794042,0.0431266},
{0.0193568,-0.0087326,0.0575704},
{0.0201026,-0.0144353,0.0531586},
{0.0025583,-0.0248211,0.0045714},
{0.0132689,-0.0214866,0.0652899},
{-0.0028669,-0.0099311,0.0309271},
{-0.0219487,-0.0054893,-0.0021853},
{0.0026180,-0.0203806,-0.0040620},
{-0.0116230,-0.0668518,0.0370152},
{-0.0133007,-0.0264159,0.0619176},
{0.0186167,0.0050491,-0.0120059},
{-0.0191359,-0.0591690,0.0288149},
{-0.0178121,-0.0041834,0.0141448},
{0.0123077,-0.0063648,0.0279743},
{-0.0157729,-0.0374010,0.0315335},
{0.0149328,-0.0659100,0.0290095},
{0.0006205,0.0021977,-0.0214613},
{0.0170484,-0.0715687,0.0325980},
{0.0068499,0.0061192,0.0196608},
{-0.0167075,0.0020024,0.0150382},

Biology Open • Supplementary information

{0.0059801,-0.0393117,0.0167489},
{-0.0072506,-0.0171067,0.0338751},
{0.0202363,-0.0251596,0.0420997},
{-0.0199311,-0.0601255,0.0171522},
{0.0216641,0.0053410,-0.0027841},
{-0.0197665,-0.0606125,0.0252972},
{0.0129986,-0.0214528,0.0098002},
{0.0201151,-0.0100929,0.0460501},
{0.0104137,0.0017765,-0.0203073},
{0.0166557,-0.0064123,0.0140197},
{-0.0124120,-0.0596549,0.0422637},
{0.0014885,0.0202590,0.0096781},
{0.0028720,0.0020207,-0.0219044},
{0.0376095,-0.3675427,0.0214537},
{0.0049949,0.0206417,-0.0079017},
{-0.0191723,-0.0407590,0.0391310},
{-0.0316406,-0.3638215,-0.0274841},
{0.0021392,0.0226920,0.0000985},
{0.0168435,-0.0229382,0.0264788},
{-0.0157304,-0.0005437,-0.0161456},
{0.0203722,-0.0299750,0.0562762},
{-0.0171077,0.0134146,0.0059615},
{0.0175519,-0.0370653,0.0413374},
{0.0154657,-0.0405127,0.0348980},
{-0.0215172,-0.0323502,0.0481925},
{0.0177998,-0.0515776,0.0444709},
{-0.0183804,-0.0448316,0.0275139},
{0.0033607,-0.0502028,0.0575894},
{0.0010325,-0.0479377,0.0191141},
{0.0224852,-0.0181821,0.0451780},
{0.0084089,-0.0720538,0.0521031},
{-0.0070880,-0.0772912,0.0288023},
{-0.0223712,-0.0028990,0.0024047},
{0.0173136,-0.0120017,0.0204612},
{0.0137772,-0.0623537,0.0276621},
{-0.0100390,-0.0231180,0.0292222},
{-0.0196395,-0.3730207,0.0364730},
{-0.0094445,-0.0526688,0.0528111},
{-0.0115309,-0.3679776,-0.0104995},
{0.0129812,-0.0410477,0.0224072},
{0.0117394,0.0154849,-0.0117191},
{-0.0245208,-0.3649412,0.0295111},
{0.0147476,-0.0502966,0.0364899},
{0.0006132,-0.0446771,0.0600524},
{-0.0118123,0.0191139,0.0031977},
{-0.0017185,-0.0163303,0.0664216},

Biology Open • Supplementary information

{-0.0085098,-0.0074564,0.0247745},
{0.0197459,-0.0086852,0.0430460},
{0.0187064,-0.0223532,0.0600966},
{-0.0206552,-0.0099712,0.0525024},
{0.0207525,0.0084646,-0.0024846},
{-0.0094915,-0.0325446,0.0184441},
{-0.0014464,-0.0140050,-0.0179819},
{-0.0122725,-0.0573444,0.0463125},
{-0.0106397,0.0177211,0.0094669},
{0.0111981,0.0191236,-0.0041491},
{-0.0162790,-0.0256279,0.0592687},
{-0.0060120,-0.3653959,0.0413411},
{-0.0135007,-0.3703638,-0.0387476},
{-0.0134258,-0.3703142,-0.0379467},
{-0.0133509,-0.3702645,-0.0371458},
{-0.0132760,-0.3702148,-0.0363450},
{-0.0132011,-0.3701651,-0.0355441},
{-0.0131262,-0.3701154,-0.0347432},
{-0.0130513,-0.3700658,-0.0339423},
{-0.0129765,-0.3700161,-0.0331414},
{-0.0129016,-0.3699664,-0.0323405},
{-0.0128267,-0.3699167,-0.0315396},
{-0.0127518,-0.3698670,-0.0307388},
{-0.0126769,-0.3698174,-0.0299379},
{-0.0126020,-0.3697677,-0.0291370},
{-0.0125271,-0.3697180,-0.0283361},
{-0.0124523,-0.3696683,-0.0275352},
{-0.0123774,-0.3696187,-0.0267343},
{-0.0123025,-0.3695690,-0.0259334},
{-0.0122276,-0.3695193,-0.0251325},
{-0.0121527,-0.3694696,-0.0243317},
{-0.0120778,-0.3694199,-0.0235308},
{-0.0120029,-0.3693703,-0.0227299},
{-0.0119280,-0.3693206,-0.0219290},
{-0.0118532,-0.3692709,-0.0211281},
{-0.0117783,-0.3692212,-0.0203272},
{-0.0117034,-0.3691715,-0.0195263},
{-0.0116285,-0.3691219,-0.0187255},
{-0.0115536,-0.3690722,-0.0179246},
{-0.0114787,-0.3690225,-0.0171237},
{-0.0114038,-0.3689728,-0.0163228},
{-0.0113289,-0.3689231,-0.0155219},
{-0.0112541,-0.3688735,-0.0147210},
{-0.0111792,-0.3688238,-0.0139201},
{-0.0111043,-0.3687741,-0.0131192},
{-0.0110294,-0.3687244,-0.0123184},

Biology Open • Supplementary information

{-0.0109545,-0.3686748,-0.0115175},
{-0.0108796,-0.3686251,-0.0107166},
{-0.0108047,-0.3685754,-0.0099157},
{-0.0107298,-0.3685257,-0.0091148},
{-0.0106550,-0.3684760,-0.0083139},
{-0.0105801,-0.3684264,-0.0075130},
{-0.0105052,-0.3683767,-0.0067121},
{-0.0104303,-0.3683270,-0.0059113},
{-0.0103554,-0.3682773,-0.0051104},
{-0.0102805,-0.3682276,-0.0043095},
{-0.0102056,-0.3681780,-0.0035086},
{-0.0101307,-0.3681283,-0.0027077},
{-0.0100559,-0.3680786,-0.0019068},
{-0.0099810,-0.3680289,-0.0011059},
{-0.0099061,-0.3679792,-0.0003051},
{-0.0098312,-0.3679296,0.0004958},
{-0.0097563,-0.3678799,0.0012967},
{-0.0096814,-0.3678302,0.0020976},
{-0.0096065,-0.3677805,0.0028985},
{-0.0095317,-0.3677308,0.0036994},
{-0.0094568,-0.3676812,0.0045003},
{-0.0093819,-0.3676315,0.0053012},
{-0.0093070,-0.3675818,0.0061020},
{-0.0092321,-0.3675321,0.0069029},
{-0.0091572,-0.3674825,0.0077038},
{-0.0090823,-0.3674328,0.0085047},
{-0.0090074,-0.3673831,0.0093056},
{-0.0089326,-0.3673334,0.0101065},
{-0.0088577,-0.3672837,0.0109074},
{-0.0087828,-0.3672341,0.0117082},
{-0.0087079,-0.3671844,0.0125091},
{-0.0086330,-0.3671347,0.0133100},
{-0.0085581,-0.3670850,0.0141109},
{-0.0084832,-0.3670353,0.0149118},
{-0.0084083,-0.3669857,0.0157127},
{-0.0083335,-0.3669360,0.0165136},
{-0.0082586,-0.3668863,0.0173145},
{-0.0081837,-0.3668366,0.0181153},
{-0.0081088,-0.3667869,0.0189162},
{-0.0080339,-0.3667373,0.0197171},
{-0.0079590,-0.3666876,0.0205180},
{-0.0078841,-0.3666379,0.0213189},
{-0.0078092,-0.3665882,0.0221198},
{-0.0077344,-0.3665386,0.0229207},
{-0.0076595,-0.3664889,0.0237215},
{-0.0075846,-0.3664392,0.0245224},

Biology Open • Supplementary information

```
        {-0.0075097,-0.3663895,0.0253233},
        {-0.0074348,-0.3663398,0.0261242},
        {-0.0073599,-0.3662902,0.0269251},
        {-0.0072850,-0.3662405,0.0277260},
        {-0.0072101,-0.3661908,0.0285269},
        {-0.0071353,-0.3661411,0.0293278},
        {-0.0070604,-0.3660914,0.0301286},
        {-0.0069855,-0.3660418,0.0309295},
        {-0.0069106,-0.3659921,0.0317304},
        {-0.0068357,-0.3659424,0.0325313},
        {-0.0067608,-0.3658927,0.0333322},
        {-0.0066859,-0.3658430,0.0341331},
        {-0.0066111,-0.3657934,0.0349340},
        {-0.0065362,-0.3657437,0.0357348},
        {-0.0064613,-0.3656940,0.0365357},
        {-0.0063864,-0.3656443,0.0373366},
        {-0.0063115,-0.3655947,0.0381375},
        {-0.0062366,-0.3655450,0.0389384},
        {-0.0061617,-0.3654953,0.0397393},
        {-0.0060868,-0.3654456,0.0405402}
}* .AMirroring);
 Points1 = ({
        {0.0000000,0.0000000,0.0000000},
        {-0.0000000,-0.3844239,0.0000000},
        {-0.0090962,-0.3910114,0.0012090},
        {-0.0000000,-0.3890805,0.0380584},
        {-0.0000000,-0.3797674,-0.0380584},
        {0.0135412,-0.0086390,0.0545946},
        {0.0196483,-0.0224683,0.0429684},
        {0.0094431,-0.0254847,0.0595583},
        {0.0001526,-0.0003974,0.0485973},
        {0.0109662,-0.0068334,0.0378725},
        {-0.0057810,-0.0139956,0.0617691},
        {0.0164460,-0.0386834,0.0492159},
        {0.0147348,-0.0335894,0.0316250},
        {0.0139180,-0.0144630,0.0239261},
        {-0.0099034,-0.0321235,0.0589375},
        {0.0032807,-0.0420515,0.0579651},
        {-0.0030036,-0.0201361,0.0393148},
        {-0.0121921,-0.0043215,0.0457883},
        {-0.0000087,-0.0042392,0.0265660},
        {-0.0119720,-0.0229308,0.0470686},
        {0.0098360,-0.0608614,0.0517067},
        {0.0156426,-0.0540685,0.0368361},
        {0.0081543,-0.0456550,0.0184707},
        {0.0094302,-0.0243144,0.0120498},
```

Biology Open • Supplementary information

{0.0178693,-0.0055164,0.0055080},
{0.0101116,0.0060820,0.0153716},
{-0.0119099,-0.0382496,0.0350282},
{-0.0097840,-0.0497224,0.0497680},
{-0.0089820,-0.0255951,0.0240460},
{-0.0059634,0.0107064,0.0153997},
{-0.0110670,-0.0058902,0.0151803},
{-0.0072431,-0.0703173,0.0431595},
{0.0061293,-0.0806876,0.0501372},
{0.0166575,-0.0756364,0.0355340},
{0.0080844,-0.0651352,0.0202955},
{-0.0114436,-0.0462484,0.0199460},
{-0.0040837,-0.0328744,0.0098553},
{-0.0004320,-0.0199527,-0.0046396},
{0.0124444,-0.0156380,-0.0062994},
{0.0168150,-0.0017977,-0.0107581},
{0.0164873,0.0130246,-0.0011711},
{0.0030473,0.0197657,0.0086996},
{-0.0138372,-0.0574107,0.0327293},
{-0.0114962,-0.0125451,0.0056021},
{-0.0117669,0.0170421,0.0015537},
{-0.0179504,0.0023626,0.0030653},
{-0.0066042,-0.0778865,0.0268022},
{-0.0041791,-0.0933870,0.0406404},
{0.0141802,-0.0980784,0.0433196},
{0.0121047,-0.0837981,0.0210443},
{-0.0098260,-0.0622066,0.0207177},
{-0.0140997,-0.0088782,-0.0098827},
{0.0006385,-0.0091689,-0.0180615},
{0.0057996,0.0077976,-0.0166249},
{0.0016318,0.0201703,-0.0078545},
{-0.0102073,0.0084386,-0.0140539},
{0.0004298,-0.0982470,0.0225398},
{-0.0018052,-0.1116971,0.0387523},
{0.0152711,-0.1182631,0.0413296},
{0.0182152,-0.1016684,0.0254613},
{0.0060568,-0.1154334,0.0186961},
{0.0000134,-0.1304245,0.0339339},
{0.0163456,-0.1384823,0.0391950},
{0.0217395,-0.1234026,0.0249958},
{0.0094227,-0.1343665,0.0159732},
{0.0022676,-0.1497358,0.0311605},
{0.0185995,-0.1589238,0.0365616},
{0.0240072,-0.1444068,0.0224697},
{0.0111285,-0.1548267,0.0136295},
{0.0037291,-0.1690250,0.0280404},

Biology Open • Supplementary information

{0.0188925,-0.1795936,0.0343210},
{0.0255149,-0.1635052,0.0203586},
{0.0124673,-0.1747466,0.0115332},
{0.0025634,-0.1889392,0.0207514},
{0.0135960,-0.1996045,0.0306237},
{0.0267720,-0.1975298,0.0233111},
{0.0258527,-0.1819770,0.0163210},
{0.0155784,-0.1973494,0.0085822},
{0.0022824,-0.2092063,0.0177187},
{0.0107691,-0.2239396,0.0267330},
{0.0240408,-0.2164442,0.0275959},
{0.0260990,-0.2143039,0.0126082},
{0.0137584,-0.2210323,0.0066529},
{0.0019272,-0.2331542,0.0150273},
{0.0117936,-0.2464923,0.0256401},
{0.0248176,-0.2357798,0.0246453},
{0.0259704,-0.2368650,0.0096352},
{0.0123799,-0.2446174,0.0043429},
{0.0028017,-0.2557481,0.0143195},
{0.0095327,-0.2692291,0.0234601},
{0.0252244,-0.2580655,0.0217569},
{0.0251523,-0.2572308,0.0058898},
{0.0097243,-0.2685715,0.0023294},
{0.0024975,-0.2833112,0.0102660},
{0.0106350,-0.2905897,0.0231875},
{0.0248192,-0.2800449,0.0200541},
{0.0253535,-0.2754549,0.0038535},
{0.0148192,-0.2881857,-0.0027043},
{0.0037536,-0.3041721,0.0007302},
{0.0024332,-0.3065012,0.0174974},
{0.0211643,-0.3060160,0.0208162},
{0.0276998,-0.2975966,0.0078790},
{0.0219434,-0.3100179,-0.0046363},
{0.0061855,-0.3217350,-0.0063975},
{-0.0001608,-0.3256884,0.0087270},
{0.0113456,-0.3236657,0.0226159},
{0.0277738,-0.3220914,0.0106489},
{0.0217295,-0.3302622,-0.0078546},
{0.0043720,-0.3417147,-0.0115499},
{-0.0020839,-0.3470196,0.0040407},
{0.0015701,-0.3404139,0.0203100},
{0.0214057,-0.3397921,0.0200474},
{0.0272577,-0.3431024,0.0047959},
{0.0206645,-0.3503447,-0.0123405},
{0.0093662,-0.3632489,-0.0228013},
{-0.0049422,-0.3587997,-0.0128388},

Biology Open • Supplementary information

{-0.0060072,-0.3706581,0.0026736},
{-0.0055331,-0.3622077,0.0189207},
{0.0098017,-0.3577621,0.0246301},
{0.0263577,-0.3599170,0.0168107},
{0.0252116,-0.3647631,-0.0001387},
{0.0239630,-0.3727459,-0.0169538},
{0.0166841,-0.3864745,-0.0282348},
{0.0040230,-0.3766956,-0.0356078},
{-0.0088768,-0.3679133,-0.0275069},
{-0.0153394,-0.3765665,-0.0118095},
{-0.0056776,-0.3922154,-0.0030824},
{-0.0164580,-0.3839844,0.0102659},
{-0.0142937,-0.3769968,0.0279361},
{0.0019886,-0.3728598,0.0327030},
{0.0196435,-0.3771759,0.0260902},
{0.0332458,-0.3781893,0.0148574},
{0.0257073,-0.3844909,-0.0017348},
{0.0278306,-0.3976790,-0.0152644},
{0.0163467,-0.4097714,-0.0258738},
{0.0023871,-0.3975269,-0.0370267},
{-0.0127485,-0.3851887,-0.0374905},
{-0.0258861,-0.3791787,-0.0265677},
{-0.0256809,-0.3932256,-0.0112654},
{-0.0163939,-0.4102024,-0.0165883},
{0.0001466,-0.4099386,-0.0110062},
{0.0028214,-0.4075984,0.0070441},
{-0.0134363,-0.4050508,0.0130811},
{-0.0230946,-0.3933966,0.0265842},
{-0.0041773,-0.3908416,0.0377255},
{0.0124171,-0.3945694,0.0311595},
{0.0302890,-0.3957538,0.0229046},
{0.0253085,-0.4002828,0.0067539},
{0.0156619,-0.4063187,-0.0060927},
{-0.0001355,-0.4156727,-0.0282518},
{-0.0135187,-0.4075238,-0.0358146},
{-0.0280933,-0.3986687,-0.0299595},
{0.0183187,-0.4107815,0.0196587},
{0.0014662,-0.4132505,0.0257866},
{-0.0136720,-0.4089793,0.0314117},
{-0.0007887,-0.0668699,0.0180990},
{-0.0094557,-0.0582117,0.0196269},
{0.0090134,-0.0341478,0.0587373},
{-0.0130054,-0.0138754,0.0541406},
{-0.0015942,-0.0568813,0.0544290},
{-0.0057688,-0.0638763,0.0213544},
{-0.0012920,-0.0820757,0.0195001},

Biology Open • Supplementary information

{-0.0064060,0.0016957,-0.0161583},
{-0.0114202,-0.0446431,0.0391109},
{0.0030282,-0.0266108,0.0622759},
{-0.0093114,-0.0659858,0.0244248},
{-0.0009779,-0.0542329,0.0174407},
{-0.0120751,-0.0170313,0.0482581},
{0.0124771,-0.0301183,0.0571420},
{-0.0095913,-0.0299145,0.0377167},
{0.0130969,-0.0344253,0.0555422},
{-0.0008166,-0.0749192,0.0510161},
{-0.0135817,-0.0438598,0.0296953},
{-0.0127968,-0.0556168,0.0208900},
{-0.0149019,-0.0543970,0.0220972},
{-0.0022733,-0.0413394,0.0128944},
{0.0099263,-0.0579505,0.0226928},
{0.0198865,-0.0132547,0.0375802},
{-0.0028638,-0.0440375,0.0588229},
{0.0124334,-0.0028068,0.0163509},
{-0.0081238,-0.0486534,0.0179570},
{-0.0054765,-0.0808815,0.0421165},
{0.0051223,0.0147705,0.0124561},
{-0.0103827,0.0169941,-0.0057102},
{-0.0064846,-0.0671946,0.0450811},
{0.0072034,-0.0845142,0.0184154},
{0.0118795,-0.0741378,0.0230526},
{0.0071176,-0.0470642,0.0557831},
{0.0132683,-0.0125793,0.0071998},
{-0.0053102,-0.0234557,0.0043424},
{-0.0066089,-0.0009675,0.0190450},
{-0.0092851,-0.0560195,0.0463883},
{-0.0056952,-0.0260635,0.0623352},
{0.0077351,-0.0063286,0.0569325},
{0.0125115,-0.0503110,0.0280874},
{-0.0018455,-0.0180506,-0.0114262},
{-0.0126754,-0.0243432,0.0517598},
{0.0147287,0.0132441,-0.0066498},
{-0.0037315,0.0214621,0.0034644},
{0.0005805,-0.0846388,0.0494817},
{-0.0119445,-0.0393492,0.0468608},
{0.0182617,-0.0278250,0.0397911},
{0.0106526,-0.0427121,0.0226210},
{0.0163902,0.0103381,0.0067703},
{0.0188891,0.0020704,0.0063238},
{0.0197730,-0.0019705,-0.0032008},
{0.0126660,-0.0200115,0.0211026},
{0.0105136,-0.0344463,0.0201481},

Biology Open • Supplementary information

{0.0149213,-0.0146037,0.0301881},
{-0.0159564,-0.0487215,0.0248157},
{0.0027610,-0.0380866,0.0604285},
{0.0154371,-0.0448599,0.0359619},
{0.0133431,-0.0100607,0.0124200},
{0.0009423,0.0043629,-0.0181755},
{0.0079634,-0.0007580,-0.0180373},
{0.0095377,-0.0669463,0.0502489},
{0.0136217,-0.0604541,0.0477345},
{0.0159995,-0.0587368,0.0396918},
{0.0161825,-0.0679973,0.0335669},
{-0.0043095,-0.0741897,0.0229721},
{-0.0121074,0.0037042,0.0130612},
{0.0070450,-0.0011130,0.0515776},
{0.0175287,-0.0092733,0.0470143},
{0.0136662,-0.0234382,0.0562557},
{-0.0108685,-0.0354352,0.0236725},
{-0.0167529,-0.0074210,-0.0023867},
{0.0149057,-0.0819262,0.0255869},
{-0.0063627,-0.0056850,-0.0177537},
{0.0037516,0.0139531,-0.0138318},
{-0.0085184,-0.0327042,0.0149349},
{-0.0135587,0.0102048,-0.0097181},
{-0.0184025,0.0049076,-0.0015510},
{-0.0083176,-0.0693211,0.0369128},
{0.0106521,-0.0172263,-0.0008855},
{0.0114644,-0.0449247,0.0535601},
{0.0079585,-0.0182646,-0.0083900},
{0.0092230,-0.0137554,-0.0128590},
{0.0039493,-0.0587102,0.0176869},
{0.0082230,-0.0500830,0.0200395},
{0.0011238,-0.0750818,0.0187358},
{-0.0167293,-0.0553962,0.0281862},
{-0.0055616,-0.0013429,0.0486990},
{-0.0120595,-0.0303987,0.0555482},
{0.0165144,-0.0171292,0.0505306},
{0.0138167,-0.0759696,0.0450637},
{0.0173092,-0.0236953,0.0327538},
{0.0077005,-0.0314379,0.0621256},
{-0.0156834,-0.0032076,0.0092745},
{0.0132996,-0.0159319,0.0562265},
{0.0142838,-0.0604781,0.0304611},
{-0.0140300,-0.0495044,0.0210445},
{-0.0129724,-0.0329429,0.0491580},
{0.0128270,-0.0411078,0.0285616},
{-0.0072187,-0.0382487,0.0601490},

Biology Open • Supplementary information

{0.0061300,-0.0076454,-0.0176148},
{0.0038223,-0.0152338,-0.0141654},
{-0.0133040,-0.0106516,0.0481183},
{-0.0096502,-0.0442140,0.0541564},
{0.0161242,-0.0533922,0.0441862},
{-0.0156133,0.0110312,0.0014663},
{-0.0127723,0.0090599,0.0108941},
{-0.0017614,-0.0671824,0.0512529},
{0.0122862,-0.0091759,0.0318947},
{0.0128444,-0.0034619,0.0515574},
{0.0141256,-0.0056746,0.0404035},
{-0.0118366,-0.0132950,-0.0080490},
{0.0053050,-0.0216999,0.0022023},
{0.0003418,-0.0484675,0.0159542},
{0.0185526,0.0030942,-0.0071111},
{0.0159020,0.0016054,-0.0123302},
{0.0118597,-0.0076253,-0.0147885},
{-0.0121715,0.0013569,-0.0144687},
{-0.0135909,-0.0059092,-0.0123980},
{0.0164055,0.0130813,0.0030031},
{0.0098027,0.0188623,-0.0021496},
{0.0083674,0.0137220,-0.0123909},
{0.0074769,-0.0223391,0.0604625},
{-0.0006593,-0.0085333,0.0596341},
{-0.0001538,-0.0020680,0.0537435},
{0.0049927,-0.0135648,0.0610058},
{0.0077959,0.0072674,0.0155171},
{0.0114423,-0.0172081,0.0147144},
{0.0036573,-0.0250000,0.0620342},
{-0.0084734,-0.0170876,0.0229152},
{-0.0094969,-0.0172702,0.0114391},
{-0.0060193,-0.0098906,0.0255878},
{-0.0140126,-0.0501615,0.0334743},
{-0.0105578,-0.0606398,0.0387460},
{-0.0064442,-0.0287821,0.0088575},
{0.0192618,-0.0183031,0.0437267},
{0.0113819,-0.0083733,0.0261874},
{0.0040221,-0.0009361,0.0229429},
{-0.0033351,-0.0752326,0.0498851},
{-0.0022379,0.0217088,-0.0030914},
{-0.0010637,-0.0130828,0.0369914},
{-0.0101770,-0.0142180,0.0023291},
{0.0120638,-0.0285334,0.0242454},
{0.0096126,-0.0269112,0.0162002},
{-0.0102426,-0.0068867,0.0497947},
{0.0174390,-0.0328356,0.0453571},

Biology Open • Supplementary information

{-0.0112391,-0.0530645,0.0392889},
{-0.0069556,-0.0802035,0.0356191},
{0.0040341,-0.0052035,0.0291718},
{-0.0027823,-0.0045673,-0.0188781},
{-0.0117433,-0.0344605,0.0551314},
{-0.0028024,0.0088279,-0.0159590},
{-0.0040239,0.0164587,-0.0117857},
{0.0173291,-0.0851364,0.0343393},
{0.0034386,-0.0734205,0.0179961},
{0.0153287,-0.0878406,0.0422141},
{0.0089280,0.0190448,0.0050877},
{0.0109361,0.0090790,0.0137818},
{0.0010231,0.0149588,-0.0135247},
{0.0149706,-0.0116002,-0.0083653},
{0.0176302,-0.0075876,-0.0071738},
{-0.0081006,-0.0241792,0.0287684},
{-0.0101525,-0.0343835,0.0304094},
{-0.0124789,-0.0614135,0.0243258},
{-0.0013182,0.0026495,0.0189991},
{0.0038672,0.0072902,0.0163910},
{-0.0023420,-0.0028404,0.0242719},
{0.0002390,-0.0306874,0.0615637},
{0.0064227,-0.0432597,0.0566578},
{-0.0108774,-0.0197799,0.0593510},
{-0.0108523,-0.0262309,0.0586232},
{0.0084023,0.0201837,0.0025047},
{0.0158937,0.0140981,0.0013290},
{-0.0066221,-0.0108767,0.0604277},
{0.0141491,-0.0529779,0.0325118},
{-0.0115313,-0.0474039,0.0454495},
{-0.0139469,-0.0502715,0.0208185},
{0.0101811,-0.0840299,0.0478670},
{-0.0091023,-0.0251593,0.0167484},
{-0.0093821,-0.0153209,0.0161037},
{-0.0041110,0.0159423,0.0119511},
{0.0008306,-0.0679297,0.0529183},
{0.0054678,-0.0626280,0.0530810},
{-0.0020970,-0.0623174,0.0526033},
{0.0153877,-0.0734345,0.0293818},
{-0.0115642,-0.0182323,0.0463033},
{0.0099054,-0.0247725,0.0163028},
{0.0047846,-0.0523180,0.0554163},
{0.0077091,-0.0349649,0.0589176},
{0.0088551,-0.0839814,0.0187731},
{-0.0062918,-0.0526965,0.0538054},
{0.0166394,-0.0426369,0.0415858},

Biology Open • Supplementary information

{-0.0110627,-0.0426195,0.0200014},
{0.0196653,-0.0205286,0.0353775},
{-0.0037392,-0.0606745,0.0191871},
{0.0154501,-0.0019814,0.0120689},
{0.0057281,0.0053758,0.0165321},
{0.0008500,-0.0322623,0.0614523},
{-0.0137553,0.0136450,-0.0047946},
{0.0098432,0.0155860,0.0096012},
{-0.0099024,0.0159025,0.0077040},
{-0.0172680,-0.0054575,-0.0048780},
{-0.0173709,0.0005048,-0.0069973},
{-0.0045478,-0.0173635,0.0626985},
{-0.0122018,-0.0340707,0.0411092},
{0.0051844,-0.0464891,0.0566311},
{0.0115389,-0.0412880,0.0545119},
{-0.0081114,-0.0677274,0.0239750},
{0.0117218,0.0070277,0.0141925},
{0.0169194,-0.0106024,-0.0010595},
{-0.0160763,-0.0079660,0.0047226},
{-0.0118551,-0.0557260,0.0364289},
{-0.0067862,-0.0166791,-0.0035060},
{-0.0120043,-0.0272752,0.0440636},
{0.0043179,-0.0741040,0.0514557},
{0.0148240,-0.0493959,0.0485098},
{-0.0016103,-0.0344851,0.0103149},
{0.0057978,-0.0316682,0.0122949},
{0.0174222,-0.0068941,0.0054067},
{0.0168224,-0.0805489,0.0390058},
{-0.0084757,-0.0187225,0.0234833},
{-0.0008156,0.0128134,0.0150291},
{0.0068713,-0.0674924,0.0197946},
{0.0081614,-0.0616758,0.0524786},
{0.0139401,-0.0128184,-0.0088097},
{0.0005159,-0.0089987,0.0600785},
{-0.0066359,-0.0145776,-0.0132087},
{0.0147515,-0.0696786,0.0438172},
{0.0071352,-0.0182011,-0.0096410},
{-0.0098507,-0.0637090,0.0216450},
{0.0123535,-0.0388534,0.0270478},
{0.0143993,-0.0042265,0.0502024},
{0.0044023,-0.0075467,0.0365939},
{-0.0028174,-0.0610119,0.0531227},
{-0.0118809,-0.0588063,0.0354761},
{-0.0134123,-0.0112835,0.0502358},
{-0.0072929,0.0121154,-0.0136193},
{-0.0159312,0.0057130,0.0075757},

Biology Open • Supplementary information

{0.0086738,-0.0672272,0.0206366},
{0.0023140,-0.0383787,0.0595802},
{-0.0125290,-0.0213931,0.0511031},
{0.0059751,-0.0060507,-0.0182666},
{0.0142933,-0.0278644,0.0525299},
{0.0111000,-0.0147643,0.0576417},
{0.0096677,0.0020387,-0.0168250},
{0.0127175,-0.0079801,0.0159781},
{-0.0120056,0.0108821,0.0108670},
{0.0096108,-0.0516302,0.0535074},
{-0.0050709,-0.0374938,0.0609148},
{0.0180380,-0.0283709,0.0465221},
{0.0147019,-0.0129140,-0.0065163},
{-0.0138432,-0.0489891,0.0339402},
{-0.0070058,-0.0719309,0.0420789},
{0.0029682,-0.0513328,0.0561035},
{-0.0040954,-0.0403913,0.0130483},
{0.0103315,0.0104239,-0.0136039},
{-0.0019883,-0.0210660,-0.0007860},
{-0.0085048,-0.0714517,0.0281430},
{-0.0095821,-0.0034131,-0.0163607},
{-0.0027054,0.0211192,-0.0043523},
{-0.0088947,-0.0211578,0.0123545},
{-0.0099651,-0.0627664,0.0214797},
{-0.0055172,-0.0727971,0.0231259},
{0.0008843,0.0115484,-0.0155632},
{0.0184120,-0.0253112,0.0458065},
{-0.0054699,-0.0564873,0.0185570},
{0.0163588,-0.0353568,0.0370195},
{0.0163351,-0.0299710,0.0329823},
{-0.0133245,-0.0116445,-0.0009141},
{0.0123152,0.0136437,0.0094656},
{-0.0124233,-0.0106200,0.0065013},
{-0.0099280,-0.0342535,0.0335597},
{0.0166670,-0.0853763,0.0398368},
{0.0164103,-0.0100530,0.0522934},
{0.0174182,-0.0151368,0.0484459},
{0.0012641,-0.0257542,0.0047157},
{0.0100176,-0.0227107,0.0589978},
{-0.0025898,-0.0092739,0.0294853},
{-0.0178980,-0.0041246,-0.0016325},
{0.0025603,-0.0204194,-0.0035775},
{-0.0089913,-0.0691025,0.0347025},
{-0.0118267,-0.0266922,0.0572066},
{0.0166110,0.0050397,-0.0101060},
{-0.0137133,-0.0604575,0.0282866},

Biology Open • Supplementary information

{-0.0141421,-0.0040768,0.0120114},
{0.0111361,-0.0078018,0.0253998},
{-0.0124694,-0.0415881,0.0300852},
{0.0139706,-0.0694544,0.0270280},
{0.0007143,0.0032695,-0.0184127},
{0.0156736,-0.0751892,0.0294906},
{0.0064452,0.0061902,0.0158407},
{-0.0132627,0.0021920,0.0121625},
{0.0054316,-0.0416285,0.0155919},
{-0.0060117,-0.0184515,0.0319557},
{0.0190799,-0.0261407,0.0403651},
{-0.0119826,-0.0606963,0.0215982},
{0.0193634,0.0049584,-0.0028606},
{-0.0128836,-0.0608571,0.0263167},
{0.0104597,-0.0216870,0.0104918},
{0.0183283,-0.0113353,0.0430617},
{0.0080435,0.0018860,-0.0177145},
{0.0137002,-0.0080500,0.0128413},
{-0.0105322,-0.0615999,0.0381348},
{0.0004302,0.0199572,0.0087433},
{0.0026396,0.0032015,-0.0187145},
{0.0350564,-0.3906540,0.0199985},
{0.0047509,0.0197753,-0.0074096},
{-0.0126942,-0.0436037,0.0355161},
{-0.0295007,-0.3866978,-0.0256255},
{0.0021775,0.0223723,-0.0000304},
{0.0135059,-0.0237696,0.0253259},
{-0.0132663,-0.0010658,-0.0138399},
{0.0155569,-0.0300151,0.0504833},
{-0.0137713,0.0130274,0.0045177},
{0.0162187,-0.0388298,0.0381509},
{0.0144746,-0.0433184,0.0327237},
{-0.0124729,-0.0341686,0.0436108},
{0.0162860,-0.0548665,0.0419723},
{-0.0157200,-0.0476782,0.0261931},
{0.0028722,-0.0544711,0.0553893},
{0.0010221,-0.0508142,0.0165418},
{0.0201222,-0.0207050,0.0419589},
{0.0074292,-0.0756616,0.0502085},
{-0.0055895,-0.0821432,0.0266136},
{-0.0178333,-0.0021318,0.0029091},
{0.0135174,-0.0132719,0.0188061},
{0.0130009,-0.0659477,0.0261675},
{-0.0087276,-0.0244484,0.0262076},
{-0.0183097,-0.3964755,0.0340043},
{-0.0081247,-0.0552147,0.0496913},

Biology Open • Supplementary information

{-0.0107507,-0.3911153,-0.0097891},
{0.0100197,-0.0428073,0.0213294},
{0.0101833,0.0143680,-0.0106590},
{-0.0228617,-0.3878880,0.0275144},
{0.0146773,-0.0525892,0.0333508},
{0.0002799,-0.0490384,0.0569970},
{-0.0103028,0.0179044,0.0027083},
{-0.0023145,-0.0165239,0.0625655},
{-0.0068423,-0.0072924,0.0229617},
{0.0181761,-0.0101977,0.0401910},
{0.0139195,-0.0242363,0.0544500},
{-0.0132542,-0.0107932,0.0495389},
{0.0185773,0.0081501,-0.0026720},
{-0.0093966,-0.0337627,0.0170648},
{-0.0003157,-0.0139759,-0.0156472},
{-0.0100999,-0.0596023,0.0420391},
{-0.0084399,0.0169458,0.0076329},
{0.0091490,0.0189426,-0.0028563},
{-0.0125547,-0.0267563,0.0542509},
{-0.0056052,-0.3883713,0.0385439},
{-0.0125872,-0.3936516,-0.0361259},
{-0.0125174,-0.3935988,-0.0353792},
{-0.0124476,-0.3935460,-0.0346325},
{-0.0123778,-0.3934932,-0.0338858},
{-0.0123079,-0.3934404,-0.0331391},
{-0.0122381,-0.3933876,-0.0323924},
{-0.0121683,-0.3933348,-0.0316458},
{-0.0120985,-0.3932820,-0.0308991},
{-0.0120287,-0.3932292,-0.0301524},
{-0.0119588,-0.3931764,-0.0294057},
{-0.0118890,-0.3931235,-0.0286590},
{-0.0118192,-0.3930707,-0.0279123},
{-0.0117494,-0.3930179,-0.0271656},
{-0.0116795,-0.3929651,-0.0264189},
{-0.0116097,-0.3929123,-0.0256722},
{-0.0115399,-0.3928595,-0.0249255},
{-0.0114701,-0.3928067,-0.0241788},
{-0.0114003,-0.3927539,-0.0234321},
{-0.0113304,-0.3927011,-0.0226854},
{-0.0112606,-0.3926483,-0.0219387},
{-0.0111908,-0.3925955,-0.0211920},
{-0.0111210,-0.3925427,-0.0204453},
{-0.0110512,-0.3924899,-0.0196986},
{-0.0109813,-0.3924371,-0.0189519},
{-0.0109115,-0.3923843,-0.0182052},
{-0.0108417,-0.3923315,-0.0174585},

Biology Open • Supplementary information

{-0.0107719,-0.3922787,-0.0167118},
{-0.0107021,-0.3922259,-0.0159651},
{-0.0106322,-0.3921731,-0.0152184},
{-0.0105624,-0.3921203,-0.0144717},
{-0.0104926,-0.3920675,-0.0137250},
{-0.0104228,-0.3920147,-0.0129783},
{-0.0103530,-0.3919619,-0.0122316},
{-0.0102831,-0.3919091,-0.0114849},
{-0.0102133,-0.3918563,-0.0107382},
{-0.0101435,-0.3918035,-0.0099915},
{-0.0100737,-0.3917507,-0.0092448},
{-0.0100039,-0.3916979,-0.0084981},
{-0.0099340,-0.3916451,-0.0077514},
{-0.0098642,-0.3915923,-0.0070047},
{-0.0097944,-0.3915395,-0.0062580},
{-0.0097246,-0.3914867,-0.0055113},
{-0.0096548,-0.3914339,-0.0047646},
{-0.0095849,-0.3913811,-0.0040179},
{-0.0095151,-0.3913283,-0.0032712},
{-0.0094453,-0.3912755,-0.0025245},
{-0.0093755,-0.3912226,-0.0017778},
{-0.0093057,-0.3911698,-0.0010311},
{-0.0092358,-0.3911170,-0.0002844},
{-0.0091660,-0.3910642,0.0004623},
{-0.0090962,-0.3910114,0.0012090},
{-0.0090264,-0.3909586,0.0019557},
{-0.0089566,-0.3909058,0.0027024},
{-0.0088867,-0.3908530,0.0034491},
{-0.0088169,-0.3908002,0.0041958},
{-0.0087471,-0.3907474,0.0049425},
{-0.0086773,-0.3906946,0.0056892},
{-0.0086075,-0.3906418,0.0064359},
{-0.0085376,-0.3905890,0.0071826},
{-0.0084678,-0.3905362,0.0079293},
{-0.0083980,-0.3904834,0.0086760},
{-0.0083282,-0.3904306,0.0094227},
{-0.0082584,-0.3903778,0.0101694},
{-0.0081885,-0.3903250,0.0109161},
{-0.0081187,-0.3902722,0.0116628},
{-0.0080489,-0.3902194,0.0124095},
{-0.0079791,-0.3901666,0.0131562},
{-0.0079093,-0.3901138,0.0139029},
{-0.0078394,-0.3900610,0.0146496},
{-0.0077696,-0.3900082,0.0153963},
{-0.0076998,-0.3899554,0.0161429},
{-0.0076300,-0.3899026,0.0168896},

Biology Open • Supplementary information

```
{-0.0075602,-0.3898498,0.0176363},
{-0.0074903,-0.3897970,0.0183830},
{-0.0074205,-0.3897442,0.0191297},
{-0.0073507,-0.3896914,0.0198764},
{-0.0072809,-0.3896386,0.0206231},
{-0.0072111,-0.3895858,0.0213698},
{-0.0071412,-0.3895330,0.0221165},
{-0.0070714,-0.3894802,0.0228632},
{-0.0070016,-0.3894274,0.0236099},
{-0.0069318,-0.3893746,0.0243566},
{-0.0068619,-0.3893217,0.0251033},
{-0.0067921,-0.3892689,0.0258500},
{-0.0067223,-0.3892161,0.0265967},
{-0.0066525,-0.3891633,0.0273434},
{-0.0065827,-0.3891105,0.0280901},
{-0.0065128,-0.3890577,0.0288368},
{-0.0064430,-0.3890049,0.0295835},
{-0.0063732,-0.3889521,0.0303302},
{-0.0063034,-0.3888993,0.0310769},
{-0.0062336,-0.3888465,0.0318236},
{-0.0061637,-0.3887937,0.0325703},
{-0.0060939,-0.3887409,0.0333170},
{-0.0060241,-0.3886881,0.0340637},
{-0.0059543,-0.3886353,0.0348104},
{-0.0058845,-0.3885825,0.0355571},
{-0.0058146,-0.3885297,0.0363038},
{-0.0057448,-0.3884769,0.0370505},
{-0.0056750,-0.3884241,0.0377972}
}* .AMirroring);
          BoundingBoxOnOff = Off;
     };
     AnyFunTransform3DIdentity ScaleFunction = {
          PreTransforms = {&.RBFTransform};
   };
  };
 };
};
```

Biology Open • Supplementary information

ScalingFunctionTLEMLucyPelvis_2014014

```
AnyFolder ScalingFunctionTLEMLucyFemur = {
  AnyFolder Right = {
          AnyFolder Thigh = {
          AnyFunTransform3DRBF RBFTransform = {
                  RBFDef.Type = RBF_ThinPlate;
                  PolynomDegree = 1;
                  Points0 = {
                          {0.0000000,0.0000000,0.0000000},
                          {-0.0000000,-0.3616821,0.0000000},
                          {-0.0097563,-0.3678799,0.0012967},
                          {-0.0000000,-0.3660632,0.0408203},
                          {-0.0000000,-0.3573010,-0.0408203},
                          {0.0161460,-0.0072838,0.0601290},
                          {0.0220217,-0.0203698,0.0463848},
                          {0.0123977,-0.0241932,0.0668573},
                          {0.0006898,0.0018121,0.0538181},
                          {0.0122809,-0.0068668,0.0414535},
                          {-0.0058991,-0.0138188,0.0648412},
                          {0.0211469,-0.0380855,0.0538111},
                          {0.0172133,-0.0317342,0.0328381},
                          {0.0177311,-0.0131946,0.0256176},
                          {-0.0110079,-0.0308867,0.0632370},
                          {0.0039435,-0.0395977,0.0638271},
                          {-0.0041683,-0.0187472,0.0413214},
                          {-0.0177999,-0.0021535,0.0496084},
                          {0.0001603,-0.0053126,0.0279593},
                          {-0.0211600,-0.0216243,0.0497147},
                          {0.0096163,-0.0568663,0.0545719},
                          {0.0162112,-0.0515013,0.0395230},
                          {0.0093533,-0.0433265,0.0187387},
                          {0.0119398,-0.0238469,0.0118403},
                          {0.0210707,-0.0048848,0.0065878},
                          {0.0125013,0.0064094,0.0177702},
                          {-0.0191523,-0.0356053,0.0382429},
                          {-0.0115821,-0.0474937,0.0537648},
                          {-0.0100073,-0.0236371,0.0267322},
                          {-0.0078925,0.0107098,0.0182833},
                          {-0.0134217,-0.0055356,0.0182000},
                          {-0.0090061,-0.0665700,0.0475484},
                          {0.0071095,-0.0761976,0.0520035},
                          {0.0182119,-0.0704149,0.0386546},
                          {0.0073764,-0.0609481,0.0221006},
                          {-0.0122984,-0.0439585,0.0198428},
                          {-0.0032253,-0.0306792,0.0110995},
```

{-0.0012975,-0.0198451,-0.0052592},
{0.0140405,-0.0161125,-0.0071649},
{0.0189222,-0.0014898,-0.0127226},
{0.0181642,0.0132812,-0.0011050},
{0.0045119,0.0200001,0.0091189},
{-0.0176761,-0.0541983,0.0348587},
{-0.0136548,-0.0142015,0.0054311},
{-0.0135510,0.0182940,0.0017175},
{-0.0223865,0.0023399,0.0028018},
{-0.0086455,-0.0738247,0.0283843},
{-0.0050356,-0.0877749,0.0442195},
{0.0160135,-0.0919319,0.0465120},
{0.0128290,-0.0785810,0.0232431},
{-0.0168328,-0.0616327,0.0155917},
{-0.0172369,-0.0094567,-0.0119502},
{0.0002090,-0.0093641,-0.0209779},
{0.0064827,0.0081538,-0.0198453},
{0.0010777,0.0210422,-0.0084721},
{-0.0129485,0.0085451,-0.0162212},
{-0.0002322,-0.0924033,0.0245644},
{-0.0028175,-0.1052263,0.0418294},
{0.0172322,-0.1111444,0.0445760},
{0.0203259,-0.0957409,0.0271449},
{0.0057102,-0.1088933,0.0195113},
{-0.0010852,-0.1230776,0.0363132},
{0.0182727,-0.1303312,0.0422884},
{0.0242891,-0.1161703,0.0265054},
{0.0094531,-0.1268584,0.0162294},
{0.0014244,-0.1413293,0.0331653},
{0.0207271,-0.1496427,0.0394157},
{0.0267562,-0.1360142,0.0236798},
{0.0113839,-0.1461651,0.0136024},
{0.0030845,-0.1595094,0.0297156},
{0.0209528,-0.1691386,0.0369775},
{0.0283958,-0.1540209,0.0213795},
{0.0129499,-0.1649012,0.0113589},
{0.0016490,-0.1783108,0.0216287},
{0.0147105,-0.1880678,0.0328614},
{0.0298491,-0.1859865,0.0248551},
{0.0286645,-0.1714377,0.0169488},
{0.0165897,-0.1861006,0.0082595},
{0.0014000,-0.1973476,0.0183740},
{0.0114301,-0.2109792,0.0286157},
{0.0267422,-0.2037622,0.0296951},
{0.0288630,-0.2018335,0.0130127},
{0.0145471,-0.2083505,0.0062833},

{0.0011300,-0.2198071,0.0155602},
{0.0126610,-0.2321235,0.0275354},
{0.0275647,-0.2219268,0.0265185},
{0.0286645,-0.2230253,0.0098701},
{0.0130434,-0.2304822,0.0039177},
{0.0022984,-0.2409585,0.0149650},
{0.0101315,-0.2534740,0.0252125},
{0.0279485,-0.2428555,0.0234276},
{0.0276684,-0.2421559,0.0058691},
{0.0101250,-0.2529527,0.0018983},
{0.0021376,-0.2667831,0.0107073},
{0.0114309,-0.2734905,0.0249960},
{0.0273923,-0.2634953,0.0216324},
{0.0278429,-0.2592546,0.0037692},
{0.0159101,-0.2712985,-0.0033988},
{0.0036708,-0.2863280,0.0004644},
{0.0023115,-0.2884756,0.0187554},
{0.0231385,-0.2879051,0.0224853},
{0.0304009,-0.2799973,0.0083549},
{0.0238189,-0.2917246,-0.0052462},
{0.0064904,-0.3027687,-0.0070587},
{-0.0003492,-0.3064690,0.0093014},
{0.0122179,-0.3045269,0.0243511},
{0.0301837,-0.3030127,0.0114462},
{0.0234182,-0.3107336,-0.0085198},
{0.0046893,-0.3214995,-0.0123881},
{-0.0022352,-0.3264905,0.0043340},
{0.0016841,-0.3202756,0.0217839},
{0.0229722,-0.3196887,0.0215083},
{0.0292438,-0.3228042,0.0051430},
{0.0221641,-0.3296190,-0.0132361},
{0.0100459,-0.3417597,-0.0244560},
{-0.0053009,-0.3375737,-0.0137705},
{-0.0064432,-0.3487307,0.0028676},
{-0.0059346,-0.3407801,0.0202938},
{0.0105130,-0.3365976,0.0264175},
{0.0282695,-0.3386249,0.0180307},
{0.0270402,-0.3431844,-0.0001487},
{0.0257040,-0.3506950,-0.0181851},
{0.0178948,-0.3636114,-0.0302838},
{0.0043149,-0.3544109,-0.0381949},
{-0.0095210,-0.3461482,-0.0295031},
{-0.0164526,-0.3542895,-0.0126666},
{-0.0060896,-0.3690127,-0.0033061},
{-0.0176523,-0.3612686,0.0110109},
{-0.0153310,-0.3546943,0.0299634},

Biology Open • Supplementary information

{0.0021329,-0.3508021,0.0350763},
{0.0210690,-0.3548628,0.0279835},
{0.0356594,-0.3558162,0.0159356},
{0.0275718,-0.3617450,-0.0018607},
{0.0298503,-0.3741530,-0.0163721},
{0.0175320,-0.3855300,-0.0277515},
{0.0025604,-0.3740099,-0.0397137},
{-0.0136727,-0.3624016,-0.0402102},
{-0.0277647,-0.3567472,-0.0284947},
{-0.0275446,-0.3699630,-0.0120829},
{-0.0175846,-0.3859355,-0.0177922},
{0.0001572,-0.3856873,-0.0118050},
{0.0030262,-0.3834856,0.0075553},
{-0.0144114,-0.3810887,0.0140304},
{-0.0247706,-0.3701239,0.0285134},
{-0.0044804,-0.3677201,0.0404632},
{0.0133183,-0.3712274,0.0334208},
{0.0324861,-0.3723417,0.0245668},
{0.0271451,-0.3766027,0.0072441},
{0.0167985,-0.3822816,-0.0065349},
{-0.0001454,-0.3910823,-0.0303010},
{-0.0144998,-0.3834154,-0.0384137},
{-0.0301321,-0.3750841,-0.0321336},
{0.0196491,-0.3864804,0.0210853},
{0.0015726,-0.3888034,0.0276569},
{-0.0146652,-0.3847848,0.0336913},
{-0.0014588,-0.0619951,0.0219778},
{-0.0118435,-0.0550626,0.0139639},
{0.0129812,-0.0349966,0.0673128},
{-0.0162907,-0.0105666,0.0597739},
{-0.0012885,-0.0520816,0.0562989},
{-0.0090599,-0.0631298,0.0172224},
{-0.0000222,-0.0771663,0.0231897},
{-0.0068148,0.0023885,-0.0160086},
{-0.0180544,-0.0425409,0.0446585},
{0.0020525,-0.0249904,0.0691549},
{-0.0157097,-0.0662315,0.0238393},
{-0.0010518,-0.0510686,0.0199353},
{-0.0208874,-0.0152865,0.0500034},
{0.0168316,-0.0280857,0.0666680},
{-0.0187392,-0.0282333,0.0403282},
{0.0191622,-0.0351633,0.0622707},
{0.0001216,-0.0706289,0.0524049},
{-0.0169862,-0.0399062,0.0311827},
{-0.0185737,-0.0533066,0.0151224},
{-0.0219058,-0.0528723,0.0222263},

Biology Open • Supplementary information

{-0.0028271,-0.0391620,0.0148452},
{0.0117900,-0.0553425,0.0235348},
{0.0219835,-0.0106400,0.0396338},
{-0.0025366,-0.0401046,0.0625273},
{0.0141017,-0.0018767,0.0198108},
{-0.0078770,-0.0473929,0.0173542},
{-0.0065351,-0.0766431,0.0470258},
{0.0065585,0.0145593,0.0161638},
{-0.0118588,0.0172875,-0.0085064},
{-0.0074087,-0.0627500,0.0506700},
{0.0071940,-0.0780068,0.0213362},
{0.0126736,-0.0708176,0.0244108},
{0.0074694,-0.0436450,0.0604196},
{0.0154186,-0.0139471,0.0064312},
{-0.0053535,-0.0213210,0.0040094},
{-0.0088733,0.0002018,0.0205402},
{-0.0112471,-0.0542608,0.0500266},
{-0.0061625,-0.0248673,0.0653244},
{0.0093695,-0.0065826,0.0614157},
{0.0138015,-0.0467322,0.0284333},
{-0.0020999,-0.0186885,-0.0116392},
{-0.0185640,-0.0225591,0.0566779},
{0.0175798,0.0124360,-0.0068467},
{-0.0039962,0.0217127,0.0053055},
{0.0014975,-0.0784591,0.0514056},
{-0.0172997,-0.0386971,0.0525252},
{0.0196540,-0.0268271,0.0416806},
{0.0135296,-0.0406504,0.0236882},
{0.0182471,0.0095959,0.0087906},
{0.0218667,0.0016804,0.0058793},
{0.0224975,-0.0022652,-0.0022994},
{0.0166750,-0.0191391,0.0236436},
{0.0119785,-0.0328723,0.0195376},
{0.0187631,-0.0133469,0.0308741},
{-0.0187543,-0.0461463,0.0257491},
{0.0039834,-0.0373928,0.0683089},
{0.0157029,-0.0423169,0.0385226},
{0.0164003,-0.0087069,0.0131181},
{0.0008253,0.0033370,-0.0212576},
{0.0101707,-0.0009724,-0.0205688},
{0.0102948,-0.0642683,0.0524532},
{0.0152217,-0.0557944,0.0508978},
{0.0172973,-0.0565707,0.0419468},
{0.0167723,-0.0635334,0.0369834},
{-0.0047397,-0.0695325,0.0241398},
{-0.0152913,0.0035778,0.0161461},

{0.0091697,0.0009340,0.0575160},
{0.0196686,-0.0089533,0.0500006},
{0.0183998,-0.0212943,0.0622968},
{-0.0114340,-0.0316810,0.0250940},
{-0.0206442,-0.0092599,-0.0028425},
{0.0166868,-0.0784295,0.0276925},
{-0.0069678,-0.0054521,-0.0207011},
{0.0049490,0.0151109,-0.0160093},
{-0.0084025,-0.0318937,0.0162788},
{-0.0173679,0.0095594,-0.0108509},
{-0.0218912,0.0063457,-0.0020698},
{-0.0106547,-0.0668750,0.0397749},
{0.0136098,-0.0170666,-0.0018890},
{0.0141599,-0.0441405,0.0587594},
{0.0093255,-0.0183218,-0.0083008},
{0.0106367,-0.0128276,-0.0154269},
{0.0037350,-0.0549365,0.0209786},
{0.0087662,-0.0472902,0.0195496},
{0.0016839,-0.0708213,0.0221325},
{-0.0202977,-0.0530540,0.0301270},
{-0.0068584,0.0021781,0.0530002},
{-0.0144817,-0.0297218,0.0605833},
{0.0197015,-0.0165217,0.0560654},
{0.0156609,-0.0705363,0.0477736},
{0.0207876,-0.0221433,0.0330733},
{0.0092324,-0.0300767,0.0713160},
{-0.0203392,-0.0034488,0.0097332},
{0.0156978,-0.0143438,0.0629053},
{0.0143267,-0.0563519,0.0317489},
{-0.0161685,-0.0465391,0.0188591},
{-0.0196397,-0.0309875,0.0546496},
{0.0154804,-0.0380422,0.0305433},
{-0.0077774,-0.0351303,0.0637765},
{0.0063980,-0.0086419,-0.0201654},
{0.0030146,-0.0151094,-0.0165974},
{-0.0210080,-0.0096030,0.0507855},
{-0.0105684,-0.0411377,0.0586146},
{0.0180619,-0.0498960,0.0469835},
{-0.0192318,0.0116108,0.0024070},
{-0.0165651,0.0083622,0.0126870},
{-0.0014333,-0.0627138,0.0534746},
{0.0145127,-0.0080178,0.0353667},
{0.0148374,-0.0012228,0.0566181},
{0.0153834,-0.0062427,0.0444727},
{-0.0155164,-0.0132103,-0.0096398},
{0.0068685,-0.0221022,0.0011547},

Biology Open • Supplementary information

{0.0003332,-0.0458500,0.0183716},
{0.0205582,0.0040956,-0.0082969},
{0.0175752,0.0009340,-0.0148407},
{0.0142357,-0.0063623,-0.0169332},
{-0.0147285,0.0023412,-0.0164678},
{-0.0158781,-0.0064451,-0.0151346},
{0.0175951,0.0133456,0.0037559},
{0.0117660,0.0189899,-0.0030690},
{0.0096040,0.0151827,-0.0137450},
{0.0096565,-0.0215194,0.0662734},
{-0.0006793,-0.0074494,0.0631218},
{0.0007819,-0.0004519,0.0600781},
{0.0065377,-0.0125433,0.0654199},
{0.0090461,0.0073096,0.0188711},
{0.0151418,-0.0168302,0.0146465},
{0.0025166,-0.0231863,0.0688894},
{-0.0097891,-0.0152077,0.0253086},
{-0.0109149,-0.0177935,0.0119825},
{-0.0072079,-0.0099373,0.0272725},
{-0.0183727,-0.0467429,0.0356276},
{-0.0126871,-0.0584555,0.0428141},
{-0.0057054,-0.0273042,0.0103554},
{0.0215142,-0.0159693,0.0474531},
{0.0128492,-0.0070016,0.0287645},
{0.0052134,-0.0017047,0.0247463},
{-0.0024284,-0.0707498,0.0513049},
{-0.0036140,0.0220593,-0.0040484},
{-0.0002405,-0.0121253,0.0390050},
{-0.0119811,-0.0156737,0.0015445},
{0.0142082,-0.0279792,0.0246553},
{0.0122244,-0.0262013,0.0169829},
{-0.0146264,-0.0038855,0.0549384},
{0.0207450,-0.0325771,0.0496412},
{-0.0155040,-0.0497560,0.0437087},
{-0.0081119,-0.0756450,0.0387365},
{0.0039684,-0.0062047,0.0313900},
{-0.0023726,-0.0038892,-0.0220521},
{-0.0136977,-0.0339839,0.0602619},
{-0.0035399,0.0089081,-0.0188894},
{-0.0049820,0.0176571,-0.0132254},
{0.0193902,-0.0803191,0.0360429},
{0.0040648,-0.0697109,0.0218205},
{0.0170470,-0.0818610,0.0456892},
{0.0104175,0.0188635,0.0064965},
{0.0135206,0.0092549,0.0158079},
{0.0023933,0.0160498,-0.0157404},

Biology Open • Supplementary information

{0.0164753,-0.0118197,-0.0096995},
{0.0202584,-0.0066858,-0.0082383},
{-0.0098401,-0.0226001,0.0314028},
{-0.0130937,-0.0309622,0.0320764},
{-0.0201510,-0.0613297,0.0222264},
{-0.0010389,0.0036055,0.0209003},
{0.0042025,0.0073392,0.0199393},
{-0.0021630,-0.0038531,0.0251898},
{-0.0007845,-0.0293574,0.0675788},
{0.0070027,-0.0406327,0.0624565},
{-0.0124363,-0.0183001,0.0627966},
{-0.0120485,-0.0256613,0.0628493},
{0.0101200,0.0197695,0.0039334},
{0.0167636,0.0148266,0.0014155},
{-0.0074981,-0.0106930,0.0632797},
{0.0142533,-0.0503445,0.0351954},
{-0.0146107,-0.0460953,0.0506120},
{-0.0161117,-0.0471301,0.0178900},
{0.0112149,-0.0792793,0.0501460},
{-0.0099060,-0.0230794,0.0186951},
{-0.0114543,-0.0153049,0.0183675},
{-0.0042882,0.0161879,0.0151423},
{0.0014582,-0.0638572,0.0541080},
{0.0048554,-0.0582603,0.0553815},
{-0.0017077,-0.0572302,0.0546635},
{0.0166090,-0.0697169,0.0323038},
{-0.0208812,-0.0164273,0.0474354},
{0.0130454,-0.0241864,0.0178294},
{0.0053123,-0.0483770,0.0582251},
{0.0112225,-0.0361429,0.0673300},
{0.0085729,-0.0771256,0.0216100},
{-0.0070783,-0.0488033,0.0560869},
{0.0182083,-0.0411024,0.0454236},
{-0.0119590,-0.0403809,0.0213546},
{0.0233409,-0.0185535,0.0366343},
{-0.0046347,-0.0571211,0.0208243},
{0.0183166,-0.0006966,0.0140116},
{0.0061913,0.0053512,0.0201003},
{-0.0002364,-0.0310828,0.0680351},
{-0.0170773,0.0139270,-0.0054987},
{0.0110805,0.0156034,0.0117010},
{-0.0124628,0.0164667,0.0094550},
{-0.0209014,-0.0073407,-0.0051969},
{-0.0210131,0.0010693,-0.0086891},
{-0.0041596,-0.0172332,0.0663041},
{-0.0216595,-0.0322172,0.0451540},

Biology Open • Supplementary information

{0.0051908,-0.0426014,0.0613013},
{0.0150003,-0.0416136,0.0606257},
{-0.0135090,-0.0674234,0.0236425},
{0.0146416,0.0073691,0.0160052},
{0.0195615,-0.0112412,-0.0010333},
{-0.0198115,-0.0098244,0.0044735},
{-0.0157054,-0.0521236,0.0395039},
{-0.0081381,-0.0173108,-0.0046152},
{-0.0218324,-0.0262661,0.0471115},
{0.0051446,-0.0707257,0.0530555},
{0.0179359,-0.0471493,0.0528134},
{-0.0005591,-0.0317983,0.0113632},
{0.0071707,-0.0305943,0.0121186},
{0.0206239,-0.0061367,0.0066642},
{0.0187057,-0.0745868,0.0420207},
{-0.0095784,-0.0163113,0.0259524},
{-0.0012181,0.0127661,0.0186855},
{0.0059420,-0.0630691,0.0220087},
{0.0069408,-0.0573878,0.0552614},
{0.0150782,-0.0132884,-0.0101793},
{0.0005533,-0.0078574,0.0633775},
{-0.0088284,-0.0146399,-0.0147837},
{0.0167444,-0.0651105,0.0470347},
{0.0082666,-0.0181741,-0.0095133},
{-0.0175162,-0.0637444,0.0177069},
{0.0153795,-0.0360767,0.0287608},
{0.0162363,-0.0018864,0.0548412},
{0.0052731,-0.0075919,0.0392990},
{-0.0022288,-0.0556644,0.0549909},
{-0.0156238,-0.0550038,0.0377688},
{-0.0204137,-0.0100550,0.0536429},
{-0.0093683,0.0122429,-0.0162013},
{-0.0202927,0.0049166,0.0083887},
{0.0074706,-0.0627945,0.0222133},
{0.0035034,-0.0376103,0.0672421},
{-0.0190752,-0.0194129,0.0555530},
{0.0061758,-0.0074961,-0.0207741},
{0.0192011,-0.0273063,0.0588233},
{0.0130796,-0.0134383,0.0638611},
{0.0120421,0.0019566,-0.0194140},
{0.0154152,-0.0065294,0.0179581},
{-0.0157335,0.0101219,0.0123981},
{0.0110376,-0.0488274,0.0567911},
{-0.0055170,-0.0343676,0.0647140},
{0.0216204,-0.0273988,0.0512041},
{0.0161331,-0.0135875,-0.0075501},

{-0.0184643,-0.0455633,0.0360087},
{-0.0091978,-0.0682413,0.0460847},
{0.0034304,-0.0471677,0.0588866},
{-0.0048025,-0.0384316,0.0149400},
{0.0118505,0.0109735,-0.0159161},
{-0.0027036,-0.0204189,-0.0015094},
{-0.0117607,-0.0692759,0.0292171},
{-0.0107080,-0.0033455,-0.0190487},
{-0.0042548,0.0214947,-0.0056846},
{-0.0097315,-0.0208529,0.0131997},
{-0.0178958,-0.0627218,0.0171551},
{-0.0068692,-0.0688818,0.0233316},
{0.0012083,0.0120695,-0.0186179},
{0.0215073,-0.0236027,0.0503069},
{-0.0060262,-0.0534931,0.0189523},
{0.0179951,-0.0336785,0.0397267},
{0.0190509,-0.0284972,0.0336394},
{-0.0169188,-0.0133609,-0.0014203},
{0.0135722,0.0136491,0.0114695},
{-0.0150549,-0.0123230,0.0067722},
{-0.0148411,-0.0311945,0.0355412},
{0.0185490,-0.0794042,0.0431266},
{0.0193568,-0.0087326,0.0575704},
{0.0201026,-0.0144353,0.0531586},
{0.0025583,-0.0248211,0.0045714},
{0.0132689,-0.0214866,0.0652899},
{-0.0028669,-0.0099311,0.0309271},
{-0.0219487,-0.0054893,-0.0021853},
{0.0026180,-0.0203806,-0.0040620},
{-0.0116230,-0.0668518,0.0370152},
{-0.0133007,-0.0264159,0.0619176},
{0.0186167,0.0050491,-0.0120059},
{-0.0191359,-0.0591690,0.0288149},
{-0.0178121,-0.0041834,0.0141448},
{0.0123077,-0.0063648,0.0279743},
{-0.0157729,-0.0374010,0.0315335},
{0.0149328,-0.0659100,0.0290095},
{0.0006205,0.0021977,-0.0214613},
{0.0170484,-0.0715687,0.0325980},
{0.0068499,0.0061192,0.0196608},
{-0.0167075,0.0020024,0.0150382},
{0.0059801,-0.0393117,0.0167489},
{-0.0072506,-0.0171067,0.0338751},
{0.0202363,-0.0251596,0.0420997},
{-0.0199311,-0.0601255,0.0171522},
{0.0216641,0.0053410,-0.0027841},

{-0.0197665,-0.0606125,0.0252972},
{0.0129986,-0.0214528,0.0098002},
{0.0201151,-0.0100929,0.0460501},
{0.0104137,0.0017765,-0.0203073},
{0.0166557,-0.0064123,0.0140197},
{-0.0124120,-0.0596549,0.0422637},
{0.0014885,0.0202590,0.0096781},
{0.0028720,0.0020207,-0.0219044},
{0.0376095,-0.3675427,0.0214537},
{0.0049949,0.0206417,-0.0079017},
{-0.0191723,-0.0407590,0.0391310},
{-0.0316406,-0.3638215,-0.0274841},
{0.0021392,0.0226920,0.0000985},
{0.0168435,-0.0229382,0.0264788},
{-0.0157304,-0.0005437,-0.0161456},
{0.0203722,-0.0299750,0.0562762},
{-0.0171077,0.0134146,0.0059615},
{0.0175519,-0.0370653,0.0413374},
{0.0154657,-0.0405127,0.0348980},
{-0.0215172,-0.0323502,0.0481925},
{0.0177998,-0.0515776,0.0444709},
{-0.0183804,-0.0448316,0.0275139},
{0.0033607,-0.0502028,0.0575894},
{0.0010325,-0.0479377,0.0191141},
{0.0224852,-0.0181821,0.0451780},
{0.0084089,-0.0720538,0.0521031},
{-0.0070880,-0.0772912,0.0288023},
{-0.0223712,-0.0028990,0.0024047},
{0.0173136,-0.0120017,0.0204612},
{0.0137772,-0.0623537,0.0276621},
{-0.0100390,-0.0231180,0.0292222},
{-0.0196395,-0.3730207,0.0364730},
{-0.0094445,-0.0526688,0.0528111},
{-0.0115309,-0.3679776,-0.0104995},
{0.0129812,-0.0410477,0.0224072},
{0.0117394,0.0154849,-0.0117191},
{-0.0245208,-0.3649412,0.0295111},
{0.0147476,-0.0502966,0.0364899},
{0.0006132,-0.0446771,0.0600524},
{-0.0118123,0.0191139,0.0031977},
{-0.0017185,-0.0163303,0.0664216},
{-0.0085098,-0.0074564,0.0247745},
{0.0197459,-0.0086852,0.0430460},
{0.0187064,-0.0223532,0.0600966},
{-0.0206552,-0.0099712,0.0525024},
{0.0207525,0.0084646,-0.0024846},

Biology Open • Supplementary information

{-0.0094915,-0.0325446,0.0184441},
{-0.0014464,-0.0140050,-0.0179819},
{-0.0122725,-0.0573444,0.0463125},
{-0.0106397,0.0177211,0.0094669},
{0.0111981,0.0191236,-0.0041491},
{-0.0162790,-0.0256279,0.0592687},
{-0.0060120,-0.3653959,0.0413411},
{-0.0135007,-0.3703638,-0.0387476},
{-0.0134258,-0.3703142,-0.0379467},
{-0.0133509,-0.3702645,-0.0371458},
{-0.0132760,-0.3702148,-0.0363450},
{-0.0132011,-0.3701651,-0.0355441},
{-0.0131262,-0.3701154,-0.0347432},
{-0.0130513,-0.3700658,-0.0339423},
{-0.0129765,-0.3700161,-0.0331414},
{-0.0129016,-0.3699664,-0.0323405},
{-0.0128267,-0.3699167,-0.0315396},
{-0.0127518,-0.3698670,-0.0307388},
{-0.0126769,-0.3698174,-0.0299379},
{-0.0126020,-0.3697677,-0.0291370},
{-0.0125271,-0.3697180,-0.0283361},
{-0.0124523,-0.3696683,-0.0275352},
{-0.0123774,-0.3696187,-0.0267343},
{-0.0123025,-0.3695690,-0.0259334},
{-0.0122276,-0.3695193,-0.0251325},
{-0.0121527,-0.3694696,-0.0243317},
{-0.0120778,-0.3694199,-0.0235308},
{-0.0120029,-0.3693703,-0.0227299},
{-0.0119280,-0.3693206,-0.0219290},
{-0.0118532,-0.3692709,-0.0211281},
{-0.0117783,-0.3692212,-0.0203272},
{-0.0117034,-0.3691715,-0.0195263},
{-0.0116285,-0.3691219,-0.0187255},
{-0.0115536,-0.3690722,-0.0179246},
{-0.0114787,-0.3690225,-0.0171237},
{-0.0114038,-0.3689728,-0.0163228},
{-0.0113289,-0.3689231,-0.0155219},
{-0.0112541,-0.3688735,-0.0147210},
{-0.0111792,-0.3688238,-0.0139201},
{-0.0111043,-0.3687741,-0.0131192},
{-0.0110294,-0.3687244,-0.0123184},
{-0.0109545,-0.3686748,-0.0115175},
{-0.0108796,-0.3686251,-0.0107166},
{-0.0108047,-0.3685754,-0.0099157},
{-0.0107298,-0.3685257,-0.0091148},
{-0.0106550,-0.3684760,-0.0083139},

Biology Open • Supplementary information

{-0.0105801,-0.3684264,-0.0075130},
{-0.0105052,-0.3683767,-0.0067121},
{-0.0104303,-0.3683270,-0.0059113},
{-0.0103554,-0.3682773,-0.0051104},
{-0.0102805,-0.3682276,-0.0043095},
{-0.0102056,-0.3681780,-0.0035086},
{-0.0101307,-0.3681283,-0.0027077},
{-0.0100559,-0.3680786,-0.0019068},
{-0.0099810,-0.3680289,-0.0011059},
{-0.0099061,-0.3679792,-0.0003051},
{-0.0098312,-0.3679296,0.0004958},
{-0.0097563,-0.3678799,0.0012967},
{-0.0096814,-0.3678302,0.0020976},
{-0.0096065,-0.3677805,0.0028985},
{-0.0095317,-0.3677308,0.0036994},
{-0.0094568,-0.3676812,0.0045003},
{-0.0093819,-0.3676315,0.0053012},
{-0.0093070,-0.3675818,0.0061020},
{-0.0092321,-0.3675321,0.0069029},
{-0.0091572,-0.3674825,0.0077038},
{-0.0090823,-0.3674328,0.0085047},
{-0.0090074,-0.3673831,0.0093056},
{-0.0089326,-0.3673334,0.0101065},
{-0.0088577,-0.3672837,0.0109074},
{-0.0087828,-0.3672341,0.0117082},
{-0.0087079,-0.3671844,0.0125091},
{-0.0086330,-0.3671347,0.0133100},
{-0.0085581,-0.3670850,0.0141109},
{-0.0084832,-0.3670353,0.0149118},
{-0.0084083,-0.3669857,0.0157127},
{-0.0083335,-0.3669360,0.0165136},
{-0.0082586,-0.3668863,0.0173145},
{-0.0081837,-0.3668366,0.0181153},
{-0.0081088,-0.3667869,0.0189162},
{-0.0080339,-0.3667373,0.0197171},
{-0.0079590,-0.3666876,0.0205180},
{-0.0078841,-0.3666379,0.0213189},
{-0.0078092,-0.3665882,0.0221198},
{-0.0077344,-0.3665386,0.0229207},
{-0.0076595,-0.3664889,0.0237215},
{-0.0075846,-0.3664392,0.0245224},
{-0.0075097,-0.3663895,0.0253233},
{-0.0074348,-0.3663398,0.0261242},
{-0.0073599,-0.3662902,0.0269251},
{-0.0072850,-0.3662405,0.0277260},
{-0.0072101,-0.3661908,0.0285269},

{-0.0071353,-0.3661411,0.0293278},
{-0.0070604,-0.3660914,0.0301286},
{-0.0069855,-0.3660418,0.0309295},
{-0.0069106,-0.3659921,0.0317304},
{-0.0068357,-0.3659424,0.0325313},
{-0.0067608,-0.3658927,0.0333322},
{-0.0066859,-0.3658430,0.0341331},
{-0.0066111,-0.3657934,0.0349340},
{-0.0065362,-0.3657437,0.0357348},
{-0.0064613,-0.3656940,0.0365357},
{-0.0063864,-0.3656443,0.0373366},
{-0.0063115,-0.3655947,0.0381375},
{-0.0062366,-0.3655450,0.0389384},
{-0.0061617,-0.3654953,0.0397393},
{-0.0060868,-0.3654456,0.0405402}
};
Points1 = {
{0.0000000,0.0000000,0.0000000},
{-0.0000000,-0.4635220,0.0000000},
{-0.0099443,-0.4714649,0.0013217},
{-0.0000000,-0.4691367,0.0416068},
{-0.0000000,-0.4579073,-0.0416068},
{0.0148038,-0.0104165,0.0596848},
{0.0214802,-0.0270913,0.0469747},
{0.0103235,-0.0307284,0.0651113},
{0.0001668,-0.0004792,0.0531283},
{0.0119887,-0.0082394,0.0414036},
{-0.0063200,-0.0168753,0.0675283},
{0.0179793,-0.0466428,0.0538046},
{0.0161087,-0.0405007,0.0345736},
{0.0152157,-0.0174389,0.0261569},
{-0.0108267,-0.0387332,0.0644327},
{0.0035866,-0.0507039,0.0633696},
{-0.0032836,-0.0242793,0.0429804},
{-0.0133289,-0.0052107,0.0500574},
{-0.0000095,-0.0051114,0.0290429},
{-0.0130883,-0.0276490,0.0514572},
{0.0107531,-0.0733841,0.0565277},
{0.0171010,-0.0651935,0.0402706},
{0.0089145,-0.0550488,0.0201929},
{0.0103094,-0.0293173,0.0131733},
{0.0195353,-0.0066515,0.0060215},
{0.0110544,0.0073334,0.0168048},
{-0.0130204,-0.0461198,0.0382942},
{-0.0106962,-0.0599532,0.0544082},
{-0.0098194,-0.0308615,0.0262879},

Biology Open • Supplementary information

{-0.0065194,0.0129094,0.0168355},
{-0.0120988,-0.0071021,0.0165957},
{-0.0079184,-0.0847856,0.0471835},
{0.0067008,-0.0972896,0.0548118},
{0.0182106,-0.0911992,0.0388471},
{0.0088381,-0.0785372,0.0221878},
{-0.0125106,-0.0557644,0.0218057},
{-0.0044644,-0.0396385,0.0107741},
{-0.0004723,-0.0240582,-0.0050722},
{0.0136047,-0.0188556,-0.0068868},
{0.0183828,-0.0021676,-0.0117612},
{0.0180245,0.0157045,-0.0012803},
{0.0033314,0.0238326,0.0095108},
{-0.0151274,-0.0692234,0.0357809},
{-0.0125681,-0.0151264,0.0061244},
{-0.0128640,0.0205487,0.0016985},
{-0.0196240,0.0028487,0.0033511},
{-0.0072200,-0.0939122,0.0293012},
{-0.0045688,-0.1126020,0.0444296},
{0.0155023,-0.1182588,0.0473586},
{0.0132333,-0.1010401,0.0230064},
{-0.0107421,-0.0750060,0.0226494},
{-0.0154143,-0.0107050,-0.0108041},
{0.0006980,-0.0110554,-0.0197454},
{0.0063404,0.0094021,-0.0181749},
{0.0017840,0.0243204,-0.0085868},
{-0.0111590,0.0101749,-0.0153642},
{0.0004699,-0.1184620,0.0246413},
{-0.0019735,-0.1346796,0.0423654},
{0.0166950,-0.1425966,0.0451830},
{0.0199136,-0.1225874,0.0278352},
{0.0066215,-0.1391847,0.0204392},
{0.0000146,-0.1572603,0.0370978},
{0.0178697,-0.1669761,0.0428494},
{0.0237665,-0.1487936,0.0273263},
{0.0103013,-0.1620134,0.0174624},
{0.0024791,-0.1805450,0.0340658},
{0.0203336,-0.1916235,0.0399705},
{0.0262456,-0.1741195,0.0245647},
{0.0121661,-0.1866834,0.0149003},
{0.0040768,-0.2038032,0.0306548},
{0.0206540,-0.2165463,0.0375210},
{0.0278938,-0.1971476,0.0222568},
{0.0136297,-0.2107020,0.0126085},
{0.0028024,-0.2278148,0.0226862},
{0.0148637,-0.2406746,0.0334790},

{0.0292682,-0.2381730,0.0254845},
{0.0282631,-0.2194201,0.0178427},
{0.0170309,-0.2379555,0.0093824},
{0.0024952,-0.2522521,0.0193707},
{0.0117731,-0.2700168,0.0292255},
{0.0262823,-0.2609791,0.0301688},
{0.0285324,-0.2583985,0.0137838},
{0.0150412,-0.2665114,0.0072732},
{0.0021069,-0.2811273,0.0164284},
{0.0128932,-0.2972099,0.0280307},
{0.0271315,-0.2842932,0.0269431},
{0.0283918,-0.2856017,0.0105336},
{0.0135342,-0.2949492,0.0047478},
{0.0030629,-0.3083702,0.0156546},
{0.0104215,-0.3246250,0.0256475},
{0.0275763,-0.3111644,0.0237855},
{0.0274974,-0.3101579,0.0064390},
{0.0106310,-0.3238321,0.0025466},
{0.0027304,-0.3416046,0.0112232},
{0.0116266,-0.3503807,0.0253494},
{0.0271332,-0.3376662,0.0219239},
{0.0277174,-0.3321318,0.0042128},
{0.0162009,-0.3474821,-0.0029564},
{0.0041036,-0.3667577,0.0007983},
{0.0026601,-0.3695661,0.0191288},
{0.0231376,-0.3689810,0.0227570},
{0.0302824,-0.3588293,0.0086136},
{0.0239893,-0.3738063,-0.0050686},
{0.0067623,-0.3879344,-0.0069940},
{-0.0001758,-0.3927011,0.0095407},
{0.0124034,-0.3902623,0.0247246},
{0.0303634,-0.3883641,0.0116417},
{0.0237555,-0.3982161,-0.0085870},
{0.0047796,-0.4120251,-0.0126267},
{-0.0022782,-0.4184214,0.0044175},
{0.0017165,-0.4104566,0.0222037},
{0.0234015,-0.4097068,0.0219166},
{0.0297992,-0.4136982,0.0052431},
{0.0225912,-0.4224308,-0.0134911},
{0.0102395,-0.4379900,-0.0249272},
{-0.0054030,-0.4326254,-0.0140358},
{-0.0065673,-0.4469238,0.0029228},
{-0.0060490,-0.4367346,0.0206848},
{0.0107155,-0.4313743,0.0269265},
{0.0288152,-0.4339726,0.0183781},
{0.0275622,-0.4398158,-0.0001516},

Biology Open • Supplementary information

{0.0261972,-0.4494412,-0.0185345},
{0.0182396,-0.4659945,-0.0308673},
{0.0043981,-0.4542035,-0.0389277},
{-0.0097044,-0.4436141,-0.0300716},
{-0.0167696,-0.4540479,-0.0129106},
{-0.0062069,-0.4729167,-0.0033698},
{-0.0179925,-0.4629921,0.0112230},
{-0.0156264,-0.4545667,0.0305408},
{0.0021740,-0.4495785,0.0357522},
{0.0214750,-0.4547826,0.0285227},
{0.0363455,-0.4560045,0.0162426},
{0.0281041,-0.4636027,-0.0018965},
{0.0304255,-0.4795043,-0.0166876},
{0.0178708,-0.4940849,-0.0282862},
{0.0026097,-0.4793210,-0.0404789},
{-0.0139372,-0.4644441,-0.0409860},
{-0.0282997,-0.4571976,-0.0290448},
{-0.0280753,-0.4741346,-0.0123157},
{-0.0179224,-0.4946046,-0.0181350},
{0.0001602,-0.4942865,-0.0120324},
{0.0030845,-0.4914648,0.0077009},
{-0.0146891,-0.4883929,0.0143007},
{-0.0252479,-0.4743408,0.0290628},
{-0.0045668,-0.4712601,0.0412429},
{0.0135749,-0.4757550,0.0340648},
{0.0331131,-0.4771830,0.0250402},
{0.0276682,-0.4826439,0.0073837},
{0.0171222,-0.4899218,-0.0066608},
{-0.0001482,-0.5012005,-0.0308859},
{-0.0147792,-0.4913748,-0.0391539},
{-0.0307126,-0.4806977,-0.0327528},
{0.0200266,-0.4953028,0.0214916},
{0.0016029,-0.4982799,0.0281909},
{-0.0149468,-0.4931298,0.0343405},
{-0.0008623,-0.0806289,0.0197865},
{-0.0103373,-0.0701892,0.0214569},
{0.0098538,-0.0411740,0.0642138},
{-0.0142180,-0.0167304,0.0591885},
{-0.0017429,-0.0685850,0.0595038},
{-0.0063067,-0.0770193,0.0233454},
{-0.0014125,-0.0989634,0.0213183},
{-0.0070033,0.0020446,-0.0176649},
{-0.0124850,-0.0538287,0.0427574},
{0.0033106,-0.0320862,0.0680823},
{-0.0101795,-0.0795629,0.0267021},
{-0.0010690,-0.0653917,0.0190668},

Biology Open • Supplementary information

{-0.0132009,-0.0205356,0.0527575},
{0.0136404,-0.0363154,0.0624697},
{-0.0104856,-0.0360696,0.0412332},
{0.0143180,-0.0415086,0.0607208},
{-0.0008927,-0.0903343,0.0557727},
{-0.0148480,-0.0528842,0.0324640},
{-0.0139899,-0.0670604,0.0228377},
{-0.0162913,-0.0655896,0.0241575},
{-0.0024852,-0.0498452,0.0140967},
{0.0108518,-0.0698743,0.0248086},
{0.0217406,-0.0159819,0.0410841},
{-0.0031309,-0.0530986,0.0643073},
{0.0135926,-0.0033843,0.0178754},
{-0.0088813,-0.0586643,0.0196312},
{-0.0059871,-0.0975235,0.0460433},
{0.0055999,0.0178096,0.0136174},
{-0.0113507,0.0204907,-0.0062426},
{-0.0070892,-0.0810203,0.0492843},
{0.0078750,-0.1019037,0.0201324},
{0.0129871,-0.0893922,0.0252020},
{0.0077813,-0.0567480,0.0609841},
{0.0145054,-0.0151676,0.0078711},
{-0.0058053,-0.0282819,0.0047472},
{-0.0072251,-0.0011666,0.0208207},
{-0.0101508,-0.0675459,0.0507134},
{-0.0062262,-0.0314262,0.0681471},
{0.0084563,-0.0076307,0.0622407},
{0.0136781,-0.0606628,0.0307062},
{-0.0020176,-0.0217646,-0.0124915},
{-0.0138572,-0.0293520,0.0565857},
{0.0161020,0.0159692,-0.0072699},
{-0.0040794,0.0258781,0.0037874},
{0.0006347,-0.1020538,0.0540952},
{-0.0130582,-0.0474456,0.0512300},
{0.0199643,-0.0335502,0.0435011},
{0.0116459,-0.0515005,0.0247301},
{0.0179184,0.0124652,0.0074015},
{0.0206503,0.0024964,0.0069134},
{0.0216165,-0.0023760,-0.0034992},
{0.0138469,-0.0241290,0.0230701},
{0.0114939,-0.0415338,0.0220267},
{0.0163125,-0.0176085,0.0330027},
{-0.0174441,-0.0587463,0.0271294},
{0.0030184,-0.0459233,0.0660627},
{0.0168764,-0.0540902,0.0393149},
{0.0145872,-0.0121308,0.0135780},

Biology Open • Supplementary information

{0.0010302,0.0052606,-0.0198701},
{0.0087058,-0.0009140,-0.0197190},
{0.0104270,-0.0807210,0.0549340},
{0.0148917,-0.0728930,0.0521852},
{0.0174912,-0.0708224,0.0433925},
{0.0176913,-0.0819882,0.0366966},
{-0.0047113,-0.0894547,0.0251140},
{-0.0132362,0.0044664,0.0142790},
{0.0077019,-0.0013420,0.0563865},
{0.0191630,-0.0111814,0.0513977},
{0.0149404,-0.0282607,0.0615008},
{-0.0118819,-0.0427263,0.0258796},
{-0.0183148,-0.0089479,-0.0026092},
{0.0162954,-0.0987832,0.0279726},
{-0.0069559,-0.0068547,-0.0194090},
{0.0041014,0.0168241,-0.0151214},
{-0.0093126,-0.0394334,0.0163274},
{-0.0148229,0.0123045,-0.0106242},
{-0.0201183,0.0059174,-0.0016956},
{-0.0090931,-0.0835844,0.0403545},
{0.0116452,-0.0207707,-0.0009681},
{0.0125333,-0.0541683,0.0585539},
{0.0087005,-0.0220227,-0.0091723},
{0.0100830,-0.0165857,-0.0140580},
{0.0043175,-0.0707903,0.0193360},
{0.0089897,-0.0603879,0.0219080},
{0.0012286,-0.0905305,0.0204827},
{-0.0182891,-0.0667944,0.0308142},
{-0.0060801,-0.0016192,0.0532395},
{-0.0131838,-0.0366534,0.0607273},
{0.0180541,-0.0206536,0.0552419},
{0.0151049,-0.0916009,0.0492653},
{0.0189230,-0.0285708,0.0358077},
{0.0084184,-0.0379064,0.0679180},
{-0.0171457,-0.0038676,0.0101392},
{0.0145396,-0.0192100,0.0614689},
{0.0156156,-0.0729219,0.0333012},
{-0.0153381,-0.0596903,0.0230066},
{-0.0141819,-0.0397212,0.0537413},
{0.0140230,-0.0495660,0.0312246},
{-0.0078918,-0.0461187,0.0657571},
{0.0067015,-0.0092184,-0.0192571},
{0.0041787,-0.0183683,-0.0154861},
{-0.0145444,-0.0128432,0.0526047},
{-0.0105500,-0.0533114,0.0592057},
{0.0176276,-0.0643780,0.0483060},

{-0.0170690,0.0133010,0.0016030},
{-0.0139632,0.0109240,0.0119099},
{-0.0019257,-0.0810056,0.0560315},
{0.0134317,-0.0110639,0.0348684},
{0.0140419,-0.0041742,0.0563644},
{0.0154426,-0.0068422,0.0441705},
{-0.0129402,-0.0160305,-0.0087995},
{0.0057996,-0.0261648,0.0024076},
{0.0003737,-0.0584400,0.0174417},
{0.0202824,0.0037308,-0.0077741},
{0.0173847,0.0019357,-0.0134799},
{0.0129654,-0.0091943,-0.0161673},
{-0.0133063,0.0016361,-0.0158178},
{-0.0148581,-0.0071251,-0.0135539},
{0.0179351,0.0157729,0.0032831},
{0.0107167,0.0227433,-0.0023500},
{0.0091475,0.0165454,-0.0135462},
{0.0081741,-0.0269355,0.0660998},
{-0.0007207,-0.0102891,0.0651942},
{-0.0001681,-0.0024935,0.0587544},
{0.0054582,-0.0163558,0.0666937},
{0.0085228,0.0087627,0.0169638},
{0.0125091,-0.0207488,0.0160863},
{0.0039983,-0.0301439,0.0678181},
{-0.0092634,-0.0206036,0.0250518},
{-0.0103824,-0.0208237,0.0125057},
{-0.0065806,-0.0119256,0.0279735},
{-0.0153191,-0.0604826,0.0365954},
{-0.0115422,-0.0731169,0.0423586},
{-0.0070451,-0.0347042,0.0096834},
{0.0210577,-0.0220691,0.0478037},
{0.0124431,-0.0100961,0.0286290},
{0.0043971,-0.0011288,0.0250820},
{-0.0036460,-0.0907122,0.0545362},
{-0.0024466,0.0261756,-0.0033797},
{-0.0011628,-0.0157747,0.0404403},
{-0.0111259,-0.0171434,0.0025463},
{0.0131886,-0.0344043,0.0265060},
{0.0105089,-0.0324484,0.0177106},
{-0.0111976,-0.0083036,0.0544374},
{0.0190649,-0.0395918,0.0495861},
{-0.0122870,-0.0639829,0.0429521},
{-0.0076041,-0.0967060,0.0389401},
{0.0044102,-0.0062742,0.0318917},
{-0.0030417,-0.0055071,-0.0206382},
{-0.0128382,-0.0415510,0.0602716},

Biology Open • Supplementary information

{-0.0030636,0.0106443,-0.0174469},
{-0.0043991,0.0198452,-0.0128846},
{0.0189448,-0.1026538,0.0375409},
{0.0037592,-0.0885273,0.0196740},
{0.0167579,-0.1059145,0.0461500},
{0.0097605,0.0229634,0.0055620},
{0.0119557,0.0109470,0.0150667},
{0.0011185,0.0180367,-0.0147857},
{0.0163664,-0.0139870,-0.0091453},
{0.0192740,-0.0091488,-0.0078427},
{-0.0088559,-0.0291543,0.0314507},
{-0.0110991,-0.0414582,0.0332446},
{-0.0136423,-0.0740498,0.0265938},
{-0.0014411,0.0031946,0.0207705},
{0.0042277,0.0087902,0.0179192},
{-0.0025604,-0.0034248,0.0265349},
{0.0002613,-0.0370016,0.0673037},
{0.0070215,-0.0521606,0.0619404},
{-0.0118916,-0.0238497,0.0648847},
{-0.0118641,-0.0316281,0.0640891},
{0.0091857,0.0243367,0.0027382},
{0.0173756,0.0169989,0.0014529},
{-0.0072395,-0.0131147,0.0660618},
{0.0154683,-0.0638785,0.0355430},
{-0.0126065,-0.0571576,0.0496871},
{-0.0152472,-0.0606152,0.0227595},
{0.0111304,-0.1013197,0.0523300},
{-0.0099510,-0.0303360,0.0183100},
{-0.0102569,-0.0184732,0.0176051},
{-0.0044943,0.0192226,0.0130653},
{0.0009080,-0.0819067,0.0578523},
{0.0059776,-0.0755142,0.0580301},
{-0.0022925,-0.0751397,0.0575079},
{0.0168224,-0.0885441,0.0321212},
{-0.0126424,-0.0219838,0.0506205},
{0.0108290,-0.0298696,0.0178229},
{0.0052307,-0.0630828,0.0605831},
{0.0084278,-0.0421592,0.0644109},
{0.0096807,-0.1012611,0.0205235},
{-0.0068785,-0.0635392,0.0588221},
{0.0181908,-0.0514098,0.0454632},
{-0.0120941,-0.0513887,0.0218663},
{0.0214988,-0.0247525,0.0386759},
{-0.0040878,-0.0731587,0.0209761},
{0.0168906,-0.0023891,0.0131942},
{0.0062621,0.0064819,0.0180735},

Biology Open • Supplementary information

{0.0009292,-0.0389005,0.0671820},
{-0.0150378,0.0164525,-0.0052416},
{0.0107610,0.0187929,0.0104964},
{-0.0108256,0.0191746,0.0084223},
{-0.0188780,-0.0065805,-0.0053328},
{-0.0189905,0.0006087,-0.0076497},
{-0.0049718,-0.0209361,0.0685443},
{-0.0133395,-0.0410810,0.0449421},
{0.0056677,-0.0560546,0.0619112},
{0.0126147,-0.0497833,0.0595944},
{-0.0088677,-0.0816628,0.0262104},
{0.0128147,0.0084737,0.0155158},
{0.0184969,-0.0127839,-0.0011583},
{-0.0175752,-0.0096051,0.0051629},
{-0.0129604,-0.0671920,0.0398254},
{-0.0074190,-0.0201109,-0.0038329},
{-0.0131235,-0.0328872,0.0481720},
{0.0047205,-0.0893515,0.0562532},
{0.0162061,-0.0595595,0.0530326},
{-0.0017605,-0.0415806,0.0112767},
{0.0063383,-0.0381842,0.0134413},
{0.0190466,-0.0083126,0.0059108},
{0.0183909,-0.0971224,0.0426426},
{-0.0092660,-0.0225748,0.0256729},
{-0.0008916,0.0154498,0.0164304},
{0.0075120,-0.0813795,0.0216402},
{0.0089223,-0.0743660,0.0573715},
{0.0152398,-0.0154559,-0.0096311},
{0.0005641,-0.0108502,0.0656801},
{-0.0072546,-0.0175771,-0.0144402},
{0.0161268,-0.0840155,0.0479026},
{0.0078004,-0.0219461,-0.0105399},
{-0.0107691,-0.0768176,0.0236631},
{0.0135053,-0.0468478,0.0295696},
{0.0157418,-0.0050961,0.0548831},
{0.0048128,-0.0090995,0.0400058},
{-0.0030801,-0.0735656,0.0580757},
{-0.0129886,-0.0709062,0.0387838},
{-0.0146628,-0.0136052,0.0549196},
{-0.0079729,0.0146083,-0.0148892},
{-0.0174166,0.0068885,0.0082820},
{0.0094825,-0.0810597,0.0225606},
{0.0025297,-0.0462754,0.0651353},
{-0.0136971,-0.0257949,0.0558678},
{0.0065322,-0.0072957,-0.0199698},
{0.0156260,-0.0335977,0.0574277},

Biology Open • Supplementary information

{0.0121349,-0.0178022,0.0630160},
{0.0105691,0.0024582,-0.0183937},
{0.0139033,-0.0096221,0.0174678},
{-0.0131250,0.0131212,0.0118803},
{0.0105069,-0.0622534,0.0584963},
{-0.0055437,-0.0452084,0.0665942},
{0.0197198,-0.0342085,0.0508596},
{0.0160727,-0.0155712,-0.0071238},
{-0.0151338,-0.0590689,0.0371047},
{-0.0076590,-0.0867312,0.0460022},
{0.0032450,-0.0618949,0.0613344},
{-0.0044772,-0.0487021,0.0142649},
{0.0112948,0.0125687,-0.0148723},
{-0.0021737,-0.0254005,-0.0008593},
{-0.0092977,-0.0861534,0.0307670},
{-0.0104755,-0.0041153,-0.0178861},
{-0.0029576,0.0254646,-0.0047581},
{-0.0097240,-0.0255112,0.0135064},
{-0.0108942,-0.0756810,0.0234824},
{-0.0060316,-0.0877756,0.0252821},
{0.0009667,0.0139245,-0.0170143},
{0.0201287,-0.0305191,0.0500773},
{-0.0059799,-0.0681100,0.0202872},
{0.0178840,-0.0426317,0.0404711},
{0.0178581,-0.0361378,0.0360575},
{-0.0145669,-0.0140404,-0.0009993},
{0.0134634,0.0164510,0.0103481},
{-0.0135816,-0.0128051,0.0071074},
{-0.0108537,-0.0413014,0.0366887},
{0.0182209,-0.1029431,0.0435511},
{0.0179403,-0.0121215,0.0571691},
{0.0190423,-0.0182513,0.0529629},
{0.0013819,-0.0310533,0.0051554},
{0.0109516,-0.0273836,0.0644985},
{-0.0028312,-0.0111821,0.0322345},
{-0.0195668,-0.0049732,-0.0017847},
{0.0027991,-0.0246209,-0.0039111},
{-0.0098297,-0.0833208,0.0379380},
{-0.0129294,-0.0321843,0.0625403},
{0.0181597,0.0060766,-0.0110482},
{-0.0149919,-0.0728971,0.0309239},
{-0.0154607,-0.0049156,0.0131313},
{0.0121743,-0.0094071,0.0277680},
{-0.0136320,-0.0501452,0.0328903},
{0.0152732,-0.0837451,0.0295480},
{0.0007809,0.0039422,-0.0201295},

Biology Open • Supplementary information

{0.0171349,-0.0906600,0.0322402},
{0.0070462,0.0074639,0.0173176},
{-0.0144993,0.0026431,0.0132965},
{0.0059381,-0.0501939,0.0170456},
{-0.0065723,-0.0222480,0.0349351},
{0.0208589,-0.0315194,0.0441286},
{-0.0130998,-0.0731850,0.0236120},
{0.0211688,0.0059787,-0.0031273},
{-0.0140848,-0.0733789,0.0287704},
{0.0114350,-0.0261493,0.0114700},
{0.0200372,-0.0136676,0.0470766},
{0.0087934,0.0022740,-0.0193661},
{0.0149776,-0.0097063,0.0140386},
{-0.0115142,-0.0742745,0.0416903},
{0.0004704,0.0240636,0.0095585},
{0.0028857,0.0038602,-0.0204594},
{0.0383249,-0.4710340,0.0218631},
{0.0051939,0.0238443,-0.0081005},
{-0.0138777,-0.0525754,0.0388275},
{-0.0322513,-0.4662637,-0.0280147},
{0.0023806,0.0269755,-0.0000332},
{0.0147652,-0.0286603,0.0276871},
{-0.0145032,-0.0012851,-0.0151303},
{0.0170074,-0.0361909,0.0551902},
{-0.0150553,0.0157078,0.0049389},
{0.0177309,-0.0468193,0.0417080},
{0.0158242,-0.0522314,0.0357748},
{-0.0136358,-0.0411991,0.0476770},
{0.0178045,-0.0661556,0.0458856},
{-0.0171857,-0.0574884,0.0286352},
{0.0031400,-0.0656790,0.0605536},
{0.0011174,-0.0612696,0.0180841},
{0.0219983,-0.0249652,0.0458711},
{0.0081219,-0.0912295,0.0548897},
{-0.0061107,-0.0990447,0.0290950},
{-0.0194960,-0.0025704,0.0031803},
{0.0147777,-0.0160027,0.0205595},
{0.0142131,-0.0795170,0.0286072},
{-0.0095414,-0.0294789,0.0286511},
{-0.0200169,-0.4780533,0.0371747},
{-0.0088822,-0.0665755,0.0543244},
{-0.0117531,-0.4715902,-0.0107018},
{0.0109539,-0.0516153,0.0233181},
{0.0111328,0.0173243,-0.0116528},
{-0.0249933,-0.4676988,0.0300797},
{0.0160458,-0.0634098,0.0364603},

Biology Open • Supplementary information

{0.0003060,-0.0591284,0.0623112},
{-0.0112634,0.0215884,0.0029608},
{-0.0025303,-0.0199239,0.0683990},
{-0.0074802,-0.0087928,0.0251026},
{0.0198708,-0.0122959,0.0439383},
{0.0152173,-0.0292231,0.0595268},
{-0.0144900,-0.0130140,0.0541578},
{0.0203094,0.0098271,-0.0029211},
{-0.0102727,-0.0407096,0.0186558},
{-0.0003452,-0.0168515,-0.0171061},
{-0.0110416,-0.0718659,0.0459587},
{-0.0092268,0.0204326,0.0083445},
{0.0100020,0.0228402,-0.0031226},
{-0.0137252,-0.0322616,0.0593091},
{-0.0061278,-0.4682816,0.0421376},
{-0.0137608,-0.4746483,-0.0394942},
{-0.0136845,-0.4745846,-0.0386779},
{-0.0136081,-0.4745210,-0.0378616},
{-0.0135318,-0.4744573,-0.0370453},
{-0.0134555,-0.4743936,-0.0362289},
{-0.0133792,-0.4743300,-0.0354126},
{-0.0133028,-0.4742663,-0.0345963},
{-0.0132265,-0.4742026,-0.0337800},
{-0.0131502,-0.4741390,-0.0329637},
{-0.0130738,-0.4740753,-0.0321473},
{-0.0129975,-0.4740116,-0.0313310},
{-0.0129212,-0.4739480,-0.0305147},
{-0.0128448,-0.4738843,-0.0296984},
{-0.0127685,-0.4738206,-0.0288821},
{-0.0126922,-0.4737570,-0.0280658},
{-0.0126159,-0.4736933,-0.0272494},
{-0.0125395,-0.4736296,-0.0264331},
{-0.0124632,-0.4735660,-0.0256168},
{-0.0123869,-0.4735023,-0.0248005},
{-0.0123105,-0.4734386,-0.0239842},
{-0.0122342,-0.4733749,-0.0231678},
{-0.0121579,-0.4733113,-0.0223515},
{-0.0120815,-0.4732476,-0.0215352},
{-0.0120052,-0.4731839,-0.0207189},
{-0.0119289,-0.4731203,-0.0199026},
{-0.0118526,-0.4730566,-0.0190863},
{-0.0117762,-0.4729929,-0.0182699},
{-0.0116999,-0.4729293,-0.0174536},
{-0.0116236,-0.4728656,-0.0166373},
{-0.0115472,-0.4728019,-0.0158210},
{-0.0114709,-0.4727383,-0.0150047},

Biology Open • Supplementary information

{-0.0113946,-0.4726746,-0.0141883},
{-0.0113182,-0.4726109,-0.0133720},
{-0.0112419,-0.4725473,-0.0125557},
{-0.0111656,-0.4724836,-0.0117394},
{-0.0110892,-0.4724199,-0.0109231},
{-0.0110129,-0.4723563,-0.0101068},
{-0.0109366,-0.4722926,-0.0092904},
{-0.0108603,-0.4722289,-0.0084741},
{-0.0107839,-0.4721653,-0.0076578},
{-0.0107076,-0.4721016,-0.0068415},
{-0.0106313,-0.4720379,-0.0060252},
{-0.0105549,-0.4719743,-0.0052088},
{-0.0104786,-0.4719106,-0.0043925},
{-0.0104023,-0.4718469,-0.0035762},
{-0.0103259,-0.4717833,-0.0027599},
{-0.0102496,-0.4717196,-0.0019436},
{-0.0101733,-0.4716559,-0.0011273},
{-0.0100970,-0.4715923,-0.0003109},
{-0.0100206,-0.4715286,0.0005054},
{-0.0099443,-0.4714649,0.0013217},
{-0.0098680,-0.4714013,0.0021380},
{-0.0097916,-0.4713376,0.0029543},
{-0.0097153,-0.4712739,0.0037707},
{-0.0096390,-0.4712103,0.0045870},
{-0.0095626,-0.4711466,0.0054033},
{-0.0094863,-0.4710829,0.0062196},
{-0.0094100,-0.4710193,0.0070359},
{-0.0093337,-0.4709556,0.0078522},
{-0.0092573,-0.4708919,0.0086686},
{-0.0091810,-0.4708283,0.0094849},
{-0.0091047,-0.4707646,0.0103012},
{-0.0090283,-0.4707009,0.0111175},
{-0.0089520,-0.4706373,0.0119338},
{-0.0088757,-0.4705736,0.0127502},
{-0.0087993,-0.4705099,0.0135665},
{-0.0087230,-0.4704463,0.0143828},
{-0.0086467,-0.4703826,0.0151991},
{-0.0085704,-0.4703189,0.0160154},
{-0.0084940,-0.4702553,0.0168317},
{-0.0084177,-0.4701916,0.0176481},
{-0.0083414,-0.4701279,0.0184644},
{-0.0082650,-0.4700642,0.0192807},
{-0.0081887,-0.4700006,0.0200970},
{-0.0081124,-0.4699369,0.0209133},
{-0.0080360,-0.4698732,0.0217297},
{-0.0079597,-0.4698096,0.0225460},

Biology Open • Supplementary information

```
                    {-0.0078834,-0.4697459,0.0233623},
                    {-0.0078071,-0.4696822,0.0241786},
                    {-0.0077307,-0.4696186,0.0249949},
                    {-0.0076544,-0.4695549,0.0258112},
                    {-0.0075781,-0.4694912,0.0266276},
                    {-0.0075017,-0.4694276,0.0274439},
                    {-0.0074254,-0.4693639,0.0282602},
                    {-0.0073491,-0.4693002,0.0290765},
                    {-0.0072727,-0.4692366,0.0298928},
                    {-0.0071964,-0.4691729,0.0307092},
                    {-0.0071201,-0.4691092,0.0315255},
                    {-0.0070438,-0.4690456,0.0323418},
                    {-0.0069674,-0.4689819,0.0331581},
                    {-0.0068911,-0.4689182,0.0339744},
                    {-0.0068148,-0.4688546,0.0347907},
                    {-0.0067384,-0.4687909,0.0356071},
                    {-0.0066621,-0.4687272,0.0364234},
                    {-0.0065858,-0.4686636,0.0372397},
                    {-0.0065094,-0.4685999,0.0380560},
                    {-0.0064331,-0.4685362,0.0388723},
                    {-0.0063568,-0.4684726,0.0396887},
                    {-0.0062805,-0.4684089,0.0405050},
                    {-0.0062041,-0.4683452,0.0413213}
                    };
            BoundingBoxOnOff = Off;
        };
        AnyFunTransform3DIdentity ScaleFunction = {
            PreTransforms = {&.RBFTransform};
};
};

};
};
```

Biology Open • Supplementary information

ScalingFunctionTLEMLucyFemur_2014014

```
AnyFolder ScalingFunctionTLEMLucyFemur = {
  AnyFolder Right = {
        AnyFolder Thigh = {
        AnyFunTransform3DRBF RBFTransform = {
                RBFDef.Type = RBF_ThinPlate;
                PolynomDegree = 1;
                Points0 = {
                        {0.0000000,0.0000000,0.0000000},
                        {-0.0000000,-0.3616821,0.0000000},
                        {-0.0097563,-0.3678799,0.0012967},
                        {-0.0000000,-0.3660632,0.0408203},
                        {-0.0000000,-0.3573010,-0.0408203},
                        {0.0161460,-0.0072838,0.0601290},
                        {0.0220217,-0.0203698,0.0463848},
                        {0.0123977,-0.0241932,0.0668573},
                        {0.0006898,0.0018121,0.0538181},
                        {0.0122809,-0.0068668,0.0414535},
                        {-0.0058991,-0.0138188,0.0648412},
                        {0.0211469,-0.0380855,0.0538111},
                        {0.0172133,-0.0317342,0.0328381},
                        {0.0177311,-0.0131946,0.0256176},
                        {-0.0110079,-0.0308867,0.0632370},
                        {0.0039435,-0.0395977,0.0638271},
                        {-0.0041683,-0.0187472,0.0413214},
                        {-0.0177999,-0.0021535,0.0496084},
                        {0.0001603,-0.0053126,0.0279593},
                        {-0.0211600,-0.0216243,0.0497147},
                        {0.0096163,-0.0568663,0.0545719},
                        {0.0162112,-0.0515013,0.0395230},
                        {0.0093533,-0.0433265,0.0187387},
                        {0.0119398,-0.0238469,0.0118403},
                        {0.0210707,-0.0048848,0.0065878},
                        {0.0125013,0.0064094,0.0177702},
                        {-0.0191523,-0.0356053,0.0382429},
                        {-0.0115821,-0.0474937,0.0537648},
                        {-0.0100073,-0.0236371,0.0267322},
                        {-0.0078925,0.0107098,0.0182833},
                        {-0.0134217,-0.0055356,0.0182000},
                        {-0.0090061,-0.0665700,0.0475484},
                        {0.0071095,-0.0761976,0.0520035},
                        {0.0182119,-0.0704149,0.0386546},
                        {0.0073764,-0.0609481,0.0221006},
                        {-0.0122984,-0.0439585,0.0198428},
                        {-0.0032253,-0.0306792,0.0110995},
```

Biology Open • Supplementary information

{-0.0012975,-0.0198451,-0.0052592},
{0.0140405,-0.0161125,-0.0071649},
{0.0189222,-0.0014898,-0.0127226},
{0.0181642,0.0132812,-0.0011050},
{0.0045119,0.0200001,0.0091189},
{-0.0176761,-0.0541983,0.0348587},
{-0.0136548,-0.0142015,0.0054311},
{-0.0135510,0.0182940,0.0017175},
{-0.0223865,0.0023399,0.0028018},
{-0.0086455,-0.0738247,0.0283843},
{-0.0050356,-0.0877749,0.0442195},
{0.0160135,-0.0919319,0.0465120},
{0.0128290,-0.0785810,0.0232431},
{-0.0168328,-0.0616327,0.0155917},
{-0.0172369,-0.0094567,-0.0119502},
{0.0002090,-0.0093641,-0.0209779},
{0.0064827,0.0081538,-0.0198453},
{0.0010777,0.0210422,-0.0084721},
{-0.0129485,0.0085451,-0.0162212},
{-0.0002322,-0.0924033,0.0245644},
{-0.0028175,-0.1052263,0.0418294},
{0.0172322,-0.1111444,0.0445760},
{0.0203259,-0.0957409,0.0271449},
{0.0057102,-0.1088933,0.0195113},
{-0.0010852,-0.1230776,0.0363132},
{0.0182727,-0.1303312,0.0422884},
{0.0242891,-0.1161703,0.0265054},
{0.0094531,-0.1268584,0.0162294},
{0.0014244,-0.1413293,0.0331653},
{0.0207271,-0.1496427,0.0394157},
{0.0267562,-0.1360142,0.0236798},
{0.0113839,-0.1461651,0.0136024},
{0.0030845,-0.1595094,0.0297156},
{0.0209528,-0.1691386,0.0369775},
{0.0283958,-0.1540209,0.0213795},
{0.0129499,-0.1649012,0.0113589},
{0.0016490,-0.1783108,0.0216287},
{0.0147105,-0.1880678,0.0328614},
{0.0298491,-0.1859865,0.0248551},
{0.0286645,-0.1714377,0.0169488},
{0.0165897,-0.1861006,0.0082595},
{0.0014000,-0.1973476,0.0183740},
{0.0114301,-0.2109792,0.0286157},
{0.0267422,-0.2037622,0.0296951},
{0.0288630,-0.2018335,0.0130127},
{0.0145471,-0.2083505,0.0062833},

Biology Open • Supplementary information

{0.0011300,-0.2198071,0.0155602},
{0.0126610,-0.2321235,0.0275354},
{0.0275647,-0.2219268,0.0265185},
{0.0286645,-0.2230253,0.0098701},
{0.0130434,-0.2304822,0.0039177},
{0.0022984,-0.2409585,0.0149650},
{0.0101315,-0.2534740,0.0252125},
{0.0279485,-0.2428555,0.0234276},
{0.0276684,-0.2421559,0.0058691},
{0.0101250,-0.2529527,0.0018983},
{0.0021376,-0.2667831,0.0107073},
{0.0114309,-0.2734905,0.0249960},
{0.0273923,-0.2634953,0.0216324},
{0.0278429,-0.2592546,0.0037692},
{0.0159101,-0.2712985,-0.0033988},
{0.0036708,-0.2863280,0.0004644},
{0.0023115,-0.2884756,0.0187554},
{0.0231385,-0.2879051,0.0224853},
{0.0304009,-0.2799973,0.0083549},
{0.0238189,-0.2917246,-0.0052462},
{0.0064904,-0.3027687,-0.0070587},
{-0.0003492,-0.3064690,0.0093014},
{0.0122179,-0.3045269,0.0243511},
{0.0301837,-0.3030127,0.0114462},
{0.0234182,-0.3107336,-0.0085198},
{0.0046893,-0.3214995,-0.0123881},
{-0.0022352,-0.3264905,0.0043340},
{0.0016841,-0.3202756,0.0217839},
{0.0229722,-0.3196887,0.0215083},
{0.0292438,-0.3228042,0.0051430},
{0.0221641,-0.3296190,-0.0132361},
{0.0100459,-0.3417597,-0.0244560},
{-0.0053009,-0.3375737,-0.0137705},
{-0.0064432,-0.3487307,0.0028676},
{-0.0059346,-0.3407801,0.0202938},
{0.0105130,-0.3365976,0.0264175},
{0.0282695,-0.3386249,0.0180307},
{0.0270402,-0.3431844,-0.0001487},
{0.0257040,-0.3506950,-0.0181851},
{0.0178948,-0.3636114,-0.0302838},
{0.0043149,-0.3544109,-0.0381949},
{-0.0095210,-0.3461482,-0.0295031},
{-0.0164526,-0.3542895,-0.0126666},
{-0.0060896,-0.3690127,-0.0033061},
{-0.0176523,-0.3612686,0.0110109},
{-0.0153310,-0.3546943,0.0299634},

Biology Open • Supplementary information

{0.0021329,-0.3508021,0.0350763},
{0.0210690,-0.3548628,0.0279835},
{0.0356594,-0.3558162,0.0159356},
{0.0275718,-0.3617450,-0.0018607},
{0.0298503,-0.3741530,-0.0163721},
{0.0175320,-0.3855300,-0.0277515},
{0.0025604,-0.3740099,-0.0397137},
{-0.0136727,-0.3624016,-0.0402102},
{-0.0277647,-0.3567472,-0.0284947},
{-0.0275446,-0.3699630,-0.0120829},
{-0.0175846,-0.3859355,-0.0177922},
{0.0001572,-0.3856873,-0.0118050},
{0.0030262,-0.3834856,0.0075553},
{-0.0144114,-0.3810887,0.0140304},
{-0.0247706,-0.3701239,0.0285134},
{-0.0044804,-0.3677201,0.0404632},
{0.0133183,-0.3712274,0.0334208},
{0.0324861,-0.3723417,0.0245668},
{0.0271451,-0.3766027,0.0072441},
{0.0167985,-0.3822816,-0.0065349},
{-0.0001454,-0.3910823,-0.0303010},
{-0.0144998,-0.3834154,-0.0384137},
{-0.0301321,-0.3750841,-0.0321336},
{0.0196491,-0.3864804,0.0210853},
{0.0015726,-0.3888034,0.0276569},
{-0.0146652,-0.3847848,0.0336913},
{-0.0014588,-0.0619951,0.0219778},
{-0.0118435,-0.0550626,0.0139639},
{0.0129812,-0.0349966,0.0673128},
{-0.0162907,-0.0105666,0.0597739},
{-0.0012885,-0.0520816,0.0562989},
{-0.0090599,-0.0631298,0.0172224},
{-0.0000222,-0.0771663,0.0231897},
{-0.0068148,0.0023885,-0.0160086},
{-0.0180544,-0.0425409,0.0446585},
{0.0020525,-0.0249904,0.0691549},
{-0.0157097,-0.0662315,0.0238393},
{-0.0010518,-0.0510686,0.0199353},
{-0.0208874,-0.0152865,0.0500034},
{0.0168316,-0.0280857,0.0666680},
{-0.0187392,-0.0282333,0.0403282},
{0.0191622,-0.0351633,0.0622707},
{0.0001216,-0.0706289,0.0524049},
{-0.0169862,-0.0399062,0.0311827},
{-0.0185737,-0.0533066,0.0151224},
{-0.0219058,-0.0528723,0.0222263},

Biology Open • Supplementary information

{-0.0028271,-0.0391620,0.0148452},
{0.0117900,-0.0553425,0.0235348},
{0.0219835,-0.0106400,0.0396338},
{-0.0025366,-0.0401046,0.0625273},
{0.0141017,-0.0018767,0.0198108},
{-0.0078770,-0.0473929,0.0173542},
{-0.0065351,-0.0766431,0.0470258},
{0.0065585,0.0145593,0.0161638},
{-0.0118588,0.0172875,-0.0085064},
{-0.0074087,-0.0627500,0.0506700},
{0.0071940,-0.0780068,0.0213362},
{0.0126736,-0.0708176,0.0244108},
{0.0074694,-0.0436450,0.0604196},
{0.0154186,-0.0139471,0.0064312},
{-0.0053535,-0.0213210,0.0040094},
{-0.0088733,0.0002018,0.0205402},
{-0.0112471,-0.0542608,0.0500266},
{-0.0061625,-0.0248673,0.0653244},
{0.0093695,-0.0065826,0.0614157},
{0.0138015,-0.0467322,0.0284333},
{-0.0020999,-0.0186885,-0.0116392},
{-0.0185640,-0.0225591,0.0566779},
{0.0175798,0.0124360,-0.0068467},
{-0.0039962,0.0217127,0.0053055},
{0.0014975,-0.0784591,0.0514056},
{-0.0172997,-0.0386971,0.0525252},
{0.0196540,-0.0268271,0.0416806},
{0.0135296,-0.0406504,0.0236882},
{0.0182471,0.0095959,0.0087906},
{0.0218667,0.0016804,0.0058793},
{0.0224975,-0.0022652,-0.0022994},
{0.0166750,-0.0191391,0.0236436},
{0.0119785,-0.0328723,0.0195376},
{0.0187631,-0.0133469,0.0308741},
{-0.0187543,-0.0461463,0.0257491},
{0.0039834,-0.0373928,0.0683089},
{0.0157029,-0.0423169,0.0385226},
{0.0164003,-0.0087069,0.0131181},
{0.0008253,0.0033370,-0.0212576},
{0.0101707,-0.0009724,-0.0205688},
{0.0102948,-0.0642683,0.0524532},
{0.0152217,-0.0557944,0.0508978},
{0.0172973,-0.0565707,0.0419468},
{0.0167723,-0.0635334,0.0369834},
{-0.0047397,-0.0695325,0.0241398},
{-0.0152913,0.0035778,0.0161461},

{0.0091697,0.0009340,0.0575160},
{0.0196686,-0.0089533,0.0500006},
{0.0183998,-0.0212943,0.0622968},
{-0.0114340,-0.0316810,0.0250940},
{-0.0206442,-0.0092599,-0.0028425},
{0.0166868,-0.0784295,0.0276925},
{-0.0069678,-0.0054521,-0.0207011},
{0.0049490,0.0151109,-0.0160093},
{-0.0084025,-0.0318937,0.0162788},
{-0.0173679,0.0095594,-0.0108509},
{-0.0218912,0.0063457,-0.0020698},
{-0.0106547,-0.0668750,0.0397749},
{0.0136098,-0.0170666,-0.0018890},
{0.0141599,-0.0441405,0.0587594},
{0.0093255,-0.0183218,-0.0083008},
{0.0106367,-0.0128276,-0.0154269},
{0.0037350,-0.0549365,0.0209786},
{0.0087662,-0.0472902,0.0195496},
{0.0016839,-0.0708213,0.0221325},
{-0.0202977,-0.0530540,0.0301270},
{-0.0068584,0.0021781,0.0530002},
{-0.0144817,-0.0297218,0.0605833},
{0.0197015,-0.0165217,0.0560654},
{0.0156609,-0.0705363,0.0477736},
{0.0207876,-0.0221433,0.0330733},
{0.0092324,-0.0300767,0.0713160},
{-0.0203392,-0.0034488,0.0097332},
{0.0156978,-0.0143438,0.0629053},
{0.0143267,-0.0563519,0.0317489},
{-0.0161685,-0.0465391,0.0188591},
{-0.0196397,-0.0309875,0.0546496},
{0.0154804,-0.0380422,0.0305433},
{-0.0077774,-0.0351303,0.0637765},
{0.0063980,-0.0086419,-0.0201654},
{0.0030146,-0.0151094,-0.0165974},
{-0.0210080,-0.0096030,0.0507855},
{-0.0105684,-0.0411377,0.0586146},
{0.0180619,-0.0498960,0.0469835},
{-0.0192318,0.0116108,0.0024070},
{-0.0165651,0.0083622,0.0126870},
{-0.0014333,-0.0627138,0.0534746},
{0.0145127,-0.0080178,0.0353667},
{0.0148374,-0.0012228,0.0566181},
{0.0153834,-0.0062427,0.0444727},
{-0.0155164,-0.0132103,-0.0096398},
{0.0068685,-0.0221022,0.0011547},

Biology Open • Supplementary information

{0.0003332,-0.0458500,0.0183716},
{0.0205582,0.0040956,-0.0082969},
{0.0175752,0.0009340,-0.0148407},
{0.0142357,-0.0063623,-0.0169332},
{-0.0147285,0.0023412,-0.0164678},
{-0.0158781,-0.0064451,-0.0151346},
{0.0175951,0.0133456,0.0037559},
{0.0117660,0.0189899,-0.0030690},
{0.0096040,0.0151827,-0.0137450},
{0.0096565,-0.0215194,0.0662734},
{-0.0006793,-0.0074494,0.0631218},
{0.0007819,-0.0004519,0.0600781},
{0.0065377,-0.0125433,0.0654199},
{0.0090461,0.0073096,0.0188711},
{0.0151418,-0.0168302,0.0146465},
{0.0025166,-0.0231863,0.0688894},
{-0.0097891,-0.0152077,0.0253086},
{-0.0109149,-0.0177935,0.0119825},
{-0.0072079,-0.0099373,0.0272725},
{-0.0183727,-0.0467429,0.0356276},
{-0.0126871,-0.0584555,0.0428141},
{-0.0057054,-0.0273042,0.0103554},
{0.0215142,-0.0159693,0.0474531},
{0.0128492,-0.0070016,0.0287645},
{0.0052134,-0.0017047,0.0247463},
{-0.0024284,-0.0707498,0.0513049},
{-0.0036140,0.0220593,-0.0040484},
{-0.0002405,-0.0121253,0.0390050},
{-0.0119811,-0.0156737,0.0015445},
{0.0142082,-0.0279792,0.0246553},
{0.0122244,-0.0262013,0.0169829},
{-0.0146264,-0.0038855,0.0549384},
{0.0207450,-0.0325771,0.0496412},
{-0.0155040,-0.0497560,0.0437087},
{-0.0081119,-0.0756450,0.0387365},
{0.0039684,-0.0062047,0.0313900},
{-0.0023726,-0.0038892,-0.0220521},
{-0.0136977,-0.0339839,0.0602619},
{-0.0035399,0.0089081,-0.0188894},
{-0.0049820,0.0176571,-0.0132254},
{0.0193902,-0.0803191,0.0360429},
{0.0040648,-0.0697109,0.0218205},
{0.0170470,-0.0818610,0.0456892},
{0.0104175,0.0188635,0.0064965},
{0.0135206,0.0092549,0.0158079},
{0.0023933,0.0160498,-0.0157404},

Biology Open • Supplementary information

{0.0164753,-0.0118197,-0.0096995},
{0.0202584,-0.0066858,-0.0082383},
{-0.0098401,-0.0226001,0.0314028},
{-0.0130937,-0.0309622,0.0320764},
{-0.0201510,-0.0613297,0.0222264},
{-0.0010389,0.0036055,0.0209003},
{0.0042025,0.0073392,0.0199393},
{-0.0021630,-0.0038531,0.0251898},
{-0.0007845,-0.0293574,0.0675788},
{0.0070027,-0.0406327,0.0624565},
{-0.0124363,-0.0183001,0.0627966},
{-0.0120485,-0.0256613,0.0628493},
{0.0101200,0.0197695,0.0039334},
{0.0167636,0.0148266,0.0014155},
{-0.0074981,-0.0106930,0.0632797},
{0.0142533,-0.0503445,0.0351954},
{-0.0146107,-0.0460953,0.0506120},
{-0.0161117,-0.0471301,0.0178900},
{0.0112149,-0.0792793,0.0501460},
{-0.0099060,-0.0230794,0.0186951},
{-0.0114543,-0.0153049,0.0183675},
{-0.0042882,0.0161879,0.0151423},
{0.0014582,-0.0638572,0.0541080},
{0.0048554,-0.0582603,0.0553815},
{-0.0017077,-0.0572302,0.0546635},
{0.0166090,-0.0697169,0.0323038},
{-0.0208812,-0.0164273,0.0474354},
{0.0130454,-0.0241864,0.0178294},
{0.0053123,-0.0483770,0.0582251},
{0.0112225,-0.0361429,0.0673300},
{0.0085729,-0.0771256,0.0216100},
{-0.0070783,-0.0488033,0.0560869},
{0.0182083,-0.0411024,0.0454236},
{-0.0119590,-0.0403809,0.0213546},
{0.0233409,-0.0185535,0.0366343},
{-0.0046347,-0.0571211,0.0208243},
{0.0183166,-0.0006966,0.0140116},
{0.0061913,0.0053512,0.0201003},
{-0.0002364,-0.0310828,0.0680351},
{-0.0170773,0.0139270,-0.0054987},
{0.0110805,0.0156034,0.0117010},
{-0.0124628,0.0164667,0.0094550},
{-0.0209014,-0.0073407,-0.0051969},
{-0.0210131,0.0010693,-0.0086891},
{-0.0041596,-0.0172332,0.0663041},
{-0.0216595,-0.0322172,0.0451540},

{0.0051908,-0.0426014,0.0613013},
{0.0150003,-0.0416136,0.0606257},
{-0.0135090,-0.0674234,0.0236425},
{0.0146416,0.0073691,0.0160052},
{0.0195615,-0.0112412,-0.0010333},
{-0.0198115,-0.0098244,0.0044735},
{-0.0157054,-0.0521236,0.0395039},
{-0.0081381,-0.0173108,-0.0046152},
{-0.0218324,-0.0262661,0.0471115},
{0.0051446,-0.0707257,0.0530555},
{0.0179359,-0.0471493,0.0528134},
{-0.0005591,-0.0317983,0.0113632},
{0.0071707,-0.0305943,0.0121186},
{0.0206239,-0.0061367,0.0066642},
{0.0187057,-0.0745868,0.0420207},
{-0.0095784,-0.0163113,0.0259524},
{-0.0012181,0.0127661,0.0186855},
{0.0059420,-0.0630691,0.0220087},
{0.0069408,-0.0573878,0.0552614},
{0.0150782,-0.0132884,-0.0101793},
{0.0005533,-0.0078574,0.0633775},
{-0.0088284,-0.0146399,-0.0147837},
{0.0167444,-0.0651105,0.0470347},
{0.0082666,-0.0181741,-0.0095133},
{-0.0175162,-0.0637444,0.0177069},
{0.0153795,-0.0360767,0.0287608},
{0.0162363,-0.0018864,0.0548412},
{0.0052731,-0.0075919,0.0392990},
{-0.0022288,-0.0556644,0.0549909},
{-0.0156238,-0.0550038,0.0377688},
{-0.0204137,-0.0100550,0.0536429},
{-0.0093683,0.0122429,-0.0162013},
{-0.0202927,0.0049166,0.0083887},
{0.0074706,-0.0627945,0.0222133},
{0.0035034,-0.0376103,0.0672421},
{-0.0190752,-0.0194129,0.0555530},
{0.0061758,-0.0074961,-0.0207741},
{0.0192011,-0.0273063,0.0588233},
{0.0130796,-0.0134383,0.0638611},
{0.0120421,0.0019566,-0.0194140},
{0.0154152,-0.0065294,0.0179581},
{-0.0157335,0.0101219,0.0123981},
{0.0110376,-0.0488274,0.0567911},
{-0.0055170,-0.0343676,0.0647140},
{0.0216204,-0.0273988,0.0512041},
{0.0161331,-0.0135875,-0.0075501},

Biology Open • Supplementary information

{-0.0184643,-0.0455633,0.0360087},
{-0.0091978,-0.0682413,0.0460847},
{0.0034304,-0.0471677,0.0588866},
{-0.0048025,-0.0384316,0.0149400},
{0.0118505,0.0109735,-0.0159161},
{-0.0027036,-0.0204189,-0.0015094},
{-0.0117607,-0.0692759,0.0292171},
{-0.0107080,-0.0033455,-0.0190487},
{-0.0042548,0.0214947,-0.0056846},
{-0.0097315,-0.0208529,0.0131997},
{-0.0178958,-0.0627218,0.0171551},
{-0.0068692,-0.0688818,0.0233316},
{0.0012083,0.0120695,-0.0186179},
{0.0215073,-0.0236027,0.0503069},
{-0.0060262,-0.0534931,0.0189523},
{0.0179951,-0.0336785,0.0397267},
{0.0190509,-0.0284972,0.0336394},
{-0.0169188,-0.0133609,-0.0014203},
{0.0135722,0.0136491,0.0114695},
{-0.0150549,-0.0123230,0.0067722},
{-0.0148411,-0.0311945,0.0355412},
{0.0185490,-0.0794042,0.0431266},
{0.0193568,-0.0087326,0.0575704},
{0.0201026,-0.0144353,0.0531586},
{0.0025583,-0.0248211,0.0045714},
{0.0132689,-0.0214866,0.0652899},
{-0.0028669,-0.0099311,0.0309271},
{-0.0219487,-0.0054893,-0.0021853},
{0.0026180,-0.0203806,-0.0040620},
{-0.0116230,-0.0668518,0.0370152},
{-0.0133007,-0.0264159,0.0619176},
{0.0186167,0.0050491,-0.0120059},
{-0.0191359,-0.0591690,0.0288149},
{-0.0178121,-0.0041834,0.0141448},
{0.0123077,-0.0063648,0.0279743},
{-0.0157729,-0.0374010,0.0315335},
{0.0149328,-0.0659100,0.0290095},
{0.0006205,0.0021977,-0.0214613},
{0.0170484,-0.0715687,0.0325980},
{0.0068499,0.0061192,0.0196608},
{-0.0167075,0.0020024,0.0150382},
{0.0059801,-0.0393117,0.0167489},
{-0.0072506,-0.0171067,0.0338751},
{0.0202363,-0.0251596,0.0420997},
{-0.0199311,-0.0601255,0.0171522},
{0.0216641,0.0053410,-0.0027841},

{-0.0197665,-0.0606125,0.0252972},
{0.0129986,-0.0214528,0.0098002},
{0.0201151,-0.0100929,0.0460501},
{0.0104137,0.0017765,-0.0203073},
{0.0166557,-0.0064123,0.0140197},
{-0.0124120,-0.0596549,0.0422637},
{0.0014885,0.0202590,0.0096781},
{0.0028720,0.0020207,-0.0219044},
{0.0376095,-0.3675427,0.0214537},
{0.0049949,0.0206417,-0.0079017},
{-0.0191723,-0.0407590,0.0391310},
{-0.0316406,-0.3638215,-0.0274841},
{0.0021392,0.0226920,0.0000985},
{0.0168435,-0.0229382,0.0264788},
{-0.0157304,-0.0005437,-0.0161456},
{0.0203722,-0.0299750,0.0562762},
{-0.0171077,0.0134146,0.0059615},
{0.0175519,-0.0370653,0.0413374},
{0.0154657,-0.0405127,0.0348980},
{-0.0215172,-0.0323502,0.0481925},
{0.0177998,-0.0515776,0.0444709},
{-0.0183804,-0.0448316,0.0275139},
{0.0033607,-0.0502028,0.0575894},
{0.0010325,-0.0479377,0.0191141},
{0.0224852,-0.0181821,0.0451780},
{0.0084089,-0.0720538,0.0521031},
{-0.0070880,-0.0772912,0.0288023},
{-0.0223712,-0.0028990,0.0024047},
{0.0173136,-0.0120017,0.0204612},
{0.0137772,-0.0623537,0.0276621},
{-0.0100390,-0.0231180,0.0292222},
{-0.0196395,-0.3730207,0.0364730},
{-0.0094445,-0.0526688,0.0528111},
{-0.0115309,-0.3679776,-0.0104995},
{0.0129812,-0.0410477,0.0224072},
{0.0117394,0.0154849,-0.0117191},
{-0.0245208,-0.3649412,0.0295111},
{0.0147476,-0.0502966,0.0364899},
{0.0006132,-0.0446771,0.0600524},
{-0.0118123,0.0191139,0.0031977},
{-0.0017185,-0.0163303,0.0664216},
{-0.0085098,-0.0074564,0.0247745},
{0.0197459,-0.0086852,0.0430460},
{0.0187064,-0.0223532,0.0600966},
{-0.0206552,-0.0099712,0.0525024},
{0.0207525,0.0084646,-0.0024846},

Biology Open • Supplementary information

{-0.0094915,-0.0325446,0.0184441},
{-0.0014464,-0.0140050,-0.0179819},
{-0.0122725,-0.0573444,0.0463125},
{-0.0106397,0.0177211,0.0094669},
{0.0111981,0.0191236,-0.0041491},
{-0.0162790,-0.0256279,0.0592687},
{-0.0060120,-0.3653959,0.0413411},
{-0.0135007,-0.3703638,-0.0387476},
{-0.0134258,-0.3703142,-0.0379467},
{-0.0133509,-0.3702645,-0.0371458},
{-0.0132760,-0.3702148,-0.0363450},
{-0.0132011,-0.3701651,-0.0355441},
{-0.0131262,-0.3701154,-0.0347432},
{-0.0130513,-0.3700658,-0.0339423},
{-0.0129765,-0.3700161,-0.0331414},
{-0.0129016,-0.3699664,-0.0323405},
{-0.0128267,-0.3699167,-0.0315396},
{-0.0127518,-0.3698670,-0.0307388},
{-0.0126769,-0.3698174,-0.0299379},
{-0.0126020,-0.3697677,-0.0291370},
{-0.0125271,-0.3697180,-0.0283361},
{-0.0124523,-0.3696683,-0.0275352},
{-0.0123774,-0.3696187,-0.0267343},
{-0.0123025,-0.3695690,-0.0259334},
{-0.0122276,-0.3695193,-0.0251325},
{-0.0121527,-0.3694696,-0.0243317},
{-0.0120778,-0.3694199,-0.0235308},
{-0.0120029,-0.3693703,-0.0227299},
{-0.0119280,-0.3693206,-0.0219290},
{-0.0118532,-0.3692709,-0.0211281},
{-0.0117783,-0.3692212,-0.0203272},
{-0.0117034,-0.3691715,-0.0195263},
{-0.0116285,-0.3691219,-0.0187255},
{-0.0115536,-0.3690722,-0.0179246},
{-0.0114787,-0.3690225,-0.0171237},
{-0.0114038,-0.3689728,-0.0163228},
{-0.0113289,-0.3689231,-0.0155219},
{-0.0112541,-0.3688735,-0.0147210},
{-0.0111792,-0.3688238,-0.0139201},
{-0.0111043,-0.3687741,-0.0131192},
{-0.0110294,-0.3687244,-0.0123184},
{-0.0109545,-0.3686748,-0.0115175},
{-0.0108796,-0.3686251,-0.0107166},
{-0.0108047,-0.3685754,-0.0099157},
{-0.0107298,-0.3685257,-0.0091148},
{-0.0106550,-0.3684760,-0.0083139},

Biology Open • Supplementary information

{-0.0105801,-0.3684264,-0.0075130},
{-0.0105052,-0.3683767,-0.0067121},
{-0.0104303,-0.3683270,-0.0059113},
{-0.0103554,-0.3682773,-0.0051104},
{-0.0102805,-0.3682276,-0.0043095},
{-0.0102056,-0.3681780,-0.0035086},
{-0.0101307,-0.3681283,-0.0027077},
{-0.0100559,-0.3680786,-0.0019068},
{-0.0099810,-0.3680289,-0.0011059},
{-0.0099061,-0.3679792,-0.0003051},
{-0.0098312,-0.3679296,0.0004958},
{-0.0097563,-0.3678799,0.0012967},
{-0.0096814,-0.3678302,0.0020976},
{-0.0096065,-0.3677805,0.0028985},
{-0.0095317,-0.3677308,0.0036994},
{-0.0094568,-0.3676812,0.0045003},
{-0.0093819,-0.3676315,0.0053012},
{-0.0093070,-0.3675818,0.0061020},
{-0.0092321,-0.3675321,0.0069029},
{-0.0091572,-0.3674825,0.0077038},
{-0.0090823,-0.3674328,0.0085047},
{-0.0090074,-0.3673831,0.0093056},
{-0.0089326,-0.3673334,0.0101065},
{-0.0088577,-0.3672837,0.0109074},
{-0.0087828,-0.3672341,0.0117082},
{-0.0087079,-0.3671844,0.0125091},
{-0.0086330,-0.3671347,0.0133100},
{-0.0085581,-0.3670850,0.0141109},
{-0.0084832,-0.3670353,0.0149118},
{-0.0084083,-0.3669857,0.0157127},
{-0.0083335,-0.3669360,0.0165136},
{-0.0082586,-0.3668863,0.0173145},
{-0.0081837,-0.3668366,0.0181153},
{-0.0081088,-0.3667869,0.0189162},
{-0.0080339,-0.3667373,0.0197171},
{-0.0079590,-0.3666876,0.0205180},
{-0.0078841,-0.3666379,0.0213189},
{-0.0078092,-0.3665882,0.0221198},
{-0.0077344,-0.3665386,0.0229207},
{-0.0076595,-0.3664889,0.0237215},
{-0.0075846,-0.3664392,0.0245224},
{-0.0075097,-0.3663895,0.0253233},
{-0.0074348,-0.3663398,0.0261242},
{-0.0073599,-0.3662902,0.0269251},
{-0.0072850,-0.3662405,0.0277260},
{-0.0072101,-0.3661908,0.0285269},

{-0.0071353,-0.3661411,0.0293278},
{-0.0070604,-0.3660914,0.0301286},
{-0.0069855,-0.3660418,0.0309295},
{-0.0069106,-0.3659921,0.0317304},
{-0.0068357,-0.3659424,0.0325313},
{-0.0067608,-0.3658927,0.0333322},
{-0.0066859,-0.3658430,0.0341331},
{-0.0066111,-0.3657934,0.0349340},
{-0.0065362,-0.3657437,0.0357348},
{-0.0064613,-0.3656940,0.0365357},
{-0.0063864,-0.3656443,0.0373366},
{-0.0063115,-0.3655947,0.0381375},
{-0.0062366,-0.3655450,0.0389384},
{-0.0061617,-0.3654953,0.0397393},
{-0.0060868,-0.3654456,0.0405402}
};
Points1 = {
{0.0000000,0.0000000,0.0000000},
{-0.0000000,-0.4635220,0.0000000},
{-0.0099443,-0.4714649,0.0013217},
{-0.0000000,-0.4691367,0.0416068},
{-0.0000000,-0.4579073,-0.0416068},
{0.0148038,-0.0104165,0.0596848},
{0.0214802,-0.0270913,0.0469747},
{0.0103235,-0.0307284,0.0651113},
{0.0001668,-0.0004792,0.0531283},
{0.0119887,-0.0082394,0.0414036},
{-0.0063200,-0.0168753,0.0675283},
{0.0179793,-0.0466428,0.0538046},
{0.0161087,-0.0405007,0.0345736},
{0.0152157,-0.0174389,0.0261569},
{-0.0108267,-0.0387332,0.0644327},
{0.0035866,-0.0507039,0.0633696},
{-0.0032836,-0.0242793,0.0429804},
{-0.0133289,-0.0052107,0.0500574},
{-0.0000095,-0.0051114,0.0290429},
{-0.0130883,-0.0276490,0.0514572},
{0.0107531,-0.0733841,0.0565277},
{0.0171010,-0.0651935,0.0402706},
{0.0089145,-0.0550488,0.0201929},
{0.0103094,-0.0293173,0.0131733},
{0.0195353,-0.0066515,0.0060215},
{0.0110544,0.0073334,0.0168048},
{-0.0130204,-0.0461198,0.0382942},
{-0.0106962,-0.0599532,0.0544082},
{-0.0098194,-0.0308615,0.0262879},

Biology Open • Supplementary information

{-0.0065194,0.0129094,0.0168355},
{-0.0120988,-0.0071021,0.0165957},
{-0.0079184,-0.0847856,0.0471835},
{0.0067008,-0.0972896,0.0548118},
{0.0182106,-0.0911992,0.0388471},
{0.0088381,-0.0785372,0.0221878},
{-0.0125106,-0.0557644,0.0218057},
{-0.0044644,-0.0396385,0.0107741},
{-0.0004723,-0.0240582,-0.0050722},
{0.0136047,-0.0188556,-0.0068868},
{0.0183828,-0.0021676,-0.0117612},
{0.0180245,0.0157045,-0.0012803},
{0.0033314,0.0238326,0.0095108},
{-0.0151274,-0.0692234,0.0357809},
{-0.0125681,-0.0151264,0.0061244},
{-0.0128640,0.0205487,0.0016985},
{-0.0196240,0.0028487,0.0033511},
{-0.0072200,-0.0939122,0.0293012},
{-0.0045688,-0.1126020,0.0444296},
{0.0155023,-0.1182588,0.0473586},
{0.0132333,-0.1010401,0.0230064},
{-0.0107421,-0.0750060,0.0226494},
{-0.0154143,-0.0107050,-0.0108041},
{0.0006980,-0.0110554,-0.0197454},
{0.0063404,0.0094021,-0.0181749},
{0.0017840,0.0243204,-0.0085868},
{-0.0111590,0.0101749,-0.0153642},
{0.0004699,-0.1184620,0.0246413},
{-0.0019735,-0.1346796,0.0423654},
{0.0166950,-0.1425966,0.0451830},
{0.0199136,-0.1225874,0.0278352},
{0.0066215,-0.1391847,0.0204392},
{0.0000146,-0.1572603,0.0370978},
{0.0178697,-0.1669761,0.0428494},
{0.0237665,-0.1487936,0.0273263},
{0.0103013,-0.1620134,0.0174624},
{0.0024791,-0.1805450,0.0340658},
{0.0203336,-0.1916235,0.0399705},
{0.0262456,-0.1741195,0.0245647},
{0.0121661,-0.1866834,0.0149003},
{0.0040768,-0.2038032,0.0306548},
{0.0206540,-0.2165463,0.0375210},
{0.0278938,-0.1971476,0.0222568},
{0.0136297,-0.2107020,0.0126085},
{0.0028024,-0.2278148,0.0226862},
{0.0148637,-0.2406746,0.0334790},

{0.0292682,-0.2381730,0.0254845},
{0.0282631,-0.2194201,0.0178427},
{0.0170309,-0.2379555,0.0093824},
{0.0024952,-0.2522521,0.0193707},
{0.0117731,-0.2700168,0.0292255},
{0.0262823,-0.2609791,0.0301688},
{0.0285324,-0.2583985,0.0137838},
{0.0150412,-0.2665114,0.0072732},
{0.0021069,-0.2811273,0.0164284},
{0.0128932,-0.2972099,0.0280307},
{0.0271315,-0.2842932,0.0269431},
{0.0283918,-0.2856017,0.0105336},
{0.0135342,-0.2949492,0.0047478},
{0.0030629,-0.3083702,0.0156546},
{0.0104215,-0.3246250,0.0256475},
{0.0275763,-0.3111644,0.0237855},
{0.0274974,-0.3101579,0.0064390},
{0.0106310,-0.3238321,0.0025466},
{0.0027304,-0.3416046,0.0112232},
{0.0116266,-0.3503807,0.0253494},
{0.0271332,-0.3376662,0.0219239},
{0.0277174,-0.3321318,0.0042128},
{0.0162009,-0.3474821,-0.0029564},
{0.0041036,-0.3667577,0.0007983},
{0.0026601,-0.3695661,0.0191288},
{0.0231376,-0.3689810,0.0227570},
{0.0302824,-0.3588293,0.0086136},
{0.0239893,-0.3738063,-0.0050686},
{0.0067623,-0.3879344,-0.0069940},
{-0.0001758,-0.3927011,0.0095407},
{0.0124034,-0.3902623,0.0247246},
{0.0303634,-0.3883641,0.0116417},
{0.0237555,-0.3982161,-0.0085870},
{0.0047796,-0.4120251,-0.0126267},
{-0.0022782,-0.4184214,0.0044175},
{0.0017165,-0.4104566,0.0222037},
{0.0234015,-0.4097068,0.0219166},
{0.0297992,-0.4136982,0.0052431},
{0.0225912,-0.4224308,-0.0134911},
{0.0102395,-0.4379900,-0.0249272},
{-0.0054030,-0.4326254,-0.0140358},
{-0.0065673,-0.4469238,0.0029228},
{-0.0060490,-0.4367346,0.0206848},
{0.0107155,-0.4313743,0.0269265},
{0.0288152,-0.4339726,0.0183781},
{0.0275622,-0.4398158,-0.0001516},

Biology Open • Supplementary information

{0.0261972,-0.4494412,-0.0185345},
{0.0182396,-0.4659945,-0.0308673},
{0.0043981,-0.4542035,-0.0389277},
{-0.0097044,-0.4436141,-0.0300716},
{-0.0167696,-0.4540479,-0.0129106},
{-0.0062069,-0.4729167,-0.0033698},
{-0.0179925,-0.4629921,0.0112230},
{-0.0156264,-0.4545667,0.0305408},
{0.0021740,-0.4495785,0.0357522},
{0.0214750,-0.4547826,0.0285227},
{0.0363455,-0.4560045,0.0162426},
{0.0281041,-0.4636027,-0.0018965},
{0.0304255,-0.4795043,-0.0166876},
{0.0178708,-0.4940849,-0.0282862},
{0.0026097,-0.4793210,-0.0404789},
{-0.0139372,-0.4644441,-0.0409860},
{-0.0282997,-0.4571976,-0.0290448},
{-0.0280753,-0.4741346,-0.0123157},
{-0.0179224,-0.4946046,-0.0181350},
{0.0001602,-0.4942865,-0.0120324},
{0.0030845,-0.4914648,0.0077009},
{-0.0146891,-0.4883929,0.0143007},
{-0.0252479,-0.4743408,0.0290628},
{-0.0045668,-0.4712601,0.0412429},
{0.0135749,-0.4757550,0.0340648},
{0.0331131,-0.4771830,0.0250402},
{0.0276682,-0.4826439,0.0073837},
{0.0171222,-0.4899218,-0.0066608},
{-0.0001482,-0.5012005,-0.0308859},
{-0.0147792,-0.4913748,-0.0391539},
{-0.0307126,-0.4806977,-0.0327528},
{0.0200266,-0.4953028,0.0214916},
{0.0016029,-0.4982799,0.0281909},
{-0.0149468,-0.4931298,0.0343405},
{-0.0008623,-0.0806289,0.0197865},
{-0.0103373,-0.0701892,0.0214569},
{0.0098538,-0.0411740,0.0642138},
{-0.0142180,-0.0167304,0.0591885},
{-0.0017429,-0.0685850,0.0595038},
{-0.0063067,-0.0770193,0.0233454},
{-0.0014125,-0.0989634,0.0213183},
{-0.0070033,0.0020446,-0.0176649},
{-0.0124850,-0.0538287,0.0427574},
{0.0033106,-0.0320862,0.0680823},
{-0.0101795,-0.0795629,0.0267021},
{-0.0010690,-0.0653917,0.0190668},

Biology Open • Supplementary information

{-0.0132009,-0.0205356,0.0527575},
{0.0136404,-0.0363154,0.0624697},
{-0.0104856,-0.0360696,0.0412332},
{0.0143180,-0.0415086,0.0607208},
{-0.0008927,-0.0903343,0.0557727},
{-0.0148480,-0.0528842,0.0324640},
{-0.0139899,-0.0670604,0.0228377},
{-0.0162913,-0.0655896,0.0241575},
{-0.0024852,-0.0498452,0.0140967},
{0.0108518,-0.0698743,0.0248086},
{0.0217406,-0.0159819,0.0410841},
{-0.0031309,-0.0530986,0.0643073},
{0.0135926,-0.0033843,0.0178754},
{-0.0088813,-0.0586643,0.0196312},
{-0.0059871,-0.0975235,0.0460433},
{0.0055999,0.0178096,0.0136174},
{-0.0113507,0.0204907,-0.0062426},
{-0.0070892,-0.0810203,0.0492843},
{0.0078750,-0.1019037,0.0201324},
{0.0129871,-0.0893922,0.0252020},
{0.0077813,-0.0567480,0.0609841},
{0.0145054,-0.0151676,0.0078711},
{-0.0058053,-0.0282819,0.0047472},
{-0.0072251,-0.0011666,0.0208207},
{-0.0101508,-0.0675459,0.0507134},
{-0.0062262,-0.0314262,0.0681471},
{0.0084563,-0.0076307,0.0622407},
{0.0136781,-0.0606628,0.0307062},
{-0.0020176,-0.0217646,-0.0124915},
{-0.0138572,-0.0293520,0.0565857},
{0.0161020,0.0159692,-0.0072699},
{-0.0040794,0.0258781,0.0037874},
{0.0006347,-0.1020538,0.0540952},
{-0.0130582,-0.0474456,0.0512300},
{0.0199643,-0.0335502,0.0435011},
{0.0116459,-0.0515005,0.0247301},
{0.0179184,0.0124652,0.0074015},
{0.0206503,0.0024964,0.0069134},
{0.0216165,-0.0023760,-0.0034992},
{0.0138469,-0.0241290,0.0230701},
{0.0114939,-0.0415338,0.0220267},
{0.0163125,-0.0176085,0.0330027},
{-0.0174441,-0.0587463,0.0271294},
{0.0030184,-0.0459233,0.0660627},
{0.0168764,-0.0540902,0.0393149},
{0.0145872,-0.0121308,0.0135780},

Biology Open • Supplementary information

{0.0010302,0.0052606,-0.0198701},
{0.0087058,-0.0009140,-0.0197190},
{0.0104270,-0.0807210,0.0549340},
{0.0148917,-0.0728930,0.0521852},
{0.0174912,-0.0708224,0.0433925},
{0.0176913,-0.0819882,0.0366966},
{-0.0047113,-0.0894547,0.0251140},
{-0.0132362,0.0044664,0.0142790},
{0.0077019,-0.0013420,0.0563865},
{0.0191630,-0.0111814,0.0513977},
{0.0149404,-0.0282607,0.0615008},
{-0.0118819,-0.0427263,0.0258796},
{-0.0183148,-0.0089479,-0.0026092},
{0.0162954,-0.0987832,0.0279726},
{-0.0069559,-0.0068547,-0.0194090},
{0.0041014,0.0168241,-0.0151214},
{-0.0093126,-0.0394334,0.0163274},
{-0.0148229,0.0123045,-0.0106242},
{-0.0201183,0.0059174,-0.0016956},
{-0.0090931,-0.0835844,0.0403545},
{0.0116452,-0.0207707,-0.0009681},
{0.0125333,-0.0541683,0.0585539},
{0.0087005,-0.0220227,-0.0091723},
{0.0100830,-0.0165857,-0.0140580},
{0.0043175,-0.0707903,0.0193360},
{0.0089897,-0.0603879,0.0219080},
{0.0012286,-0.0905305,0.0204827},
{-0.0182891,-0.0667944,0.0308142},
{-0.0060801,-0.0016192,0.0532395},
{-0.0131838,-0.0366534,0.0607273},
{0.0180541,-0.0206536,0.0552419},
{0.0151049,-0.0916009,0.0492653},
{0.0189230,-0.0285708,0.0358077},
{0.0084184,-0.0379064,0.0679180},
{-0.0171457,-0.0038676,0.0101392},
{0.0145396,-0.0192100,0.0614689},
{0.0156156,-0.0729219,0.0333012},
{-0.0153381,-0.0596903,0.0230066},
{-0.0141819,-0.0397212,0.0537413},
{0.0140230,-0.0495660,0.0312246},
{-0.0078918,-0.0461187,0.0657571},
{0.0067015,-0.0092184,-0.0192571},
{0.0041787,-0.0183683,-0.0154861},
{-0.0145444,-0.0128432,0.0526047},
{-0.0105500,-0.0533114,0.0592057},
{0.0176276,-0.0643780,0.0483060},

Biology Open • Supplementary information

{-0.0170690,0.0133010,0.0016030},
{-0.0139632,0.0109240,0.0119099},
{-0.0019257,-0.0810056,0.0560315},
{0.0134317,-0.0110639,0.0348684},
{0.0140419,-0.0041742,0.0563644},
{0.0154426,-0.0068422,0.0441705},
{-0.0129402,-0.0160305,-0.0087995},
{0.0057996,-0.0261648,0.0024076},
{0.0003737,-0.0584400,0.0174417},
{0.0202824,0.0037308,-0.0077741},
{0.0173847,0.0019357,-0.0134799},
{0.0129654,-0.0091943,-0.0161673},
{-0.0133063,0.0016361,-0.0158178},
{-0.0148581,-0.0071251,-0.0135539},
{0.0179351,0.0157729,0.0032831},
{0.0107167,0.0227433,-0.0023500},
{0.0091475,0.0165454,-0.0135462},
{0.0081741,-0.0269355,0.0660998},
{-0.0007207,-0.0102891,0.0651942},
{-0.0001681,-0.0024935,0.0587544},
{0.0054582,-0.0163558,0.0666937},
{0.0085228,0.0087627,0.0169638},
{0.0125091,-0.0207488,0.0160863},
{0.0039983,-0.0301439,0.0678181},
{-0.0092634,-0.0206036,0.0250518},
{-0.0103824,-0.0208237,0.0125057},
{-0.0065806,-0.0119256,0.0279735},
{-0.0153191,-0.0604826,0.0365954},
{-0.0115422,-0.0731169,0.0423586},
{-0.0070451,-0.0347042,0.0096834},
{0.0210577,-0.0220691,0.0478037},
{0.0124431,-0.0100961,0.0286290},
{0.0043971,-0.0011288,0.0250820},
{-0.0036460,-0.0907122,0.0545362},
{-0.0024466,0.0261756,-0.0033797},
{-0.0011628,-0.0157747,0.0404403},
{-0.0111259,-0.0171434,0.0025463},
{0.0131886,-0.0344043,0.0265060},
{0.0105089,-0.0324484,0.0177106},
{-0.0111976,-0.0083036,0.0544374},
{0.0190649,-0.0395918,0.0495861},
{-0.0122870,-0.0639829,0.0429521},
{-0.0076041,-0.0967060,0.0389401},
{0.0044102,-0.0062742,0.0318917},
{-0.0030417,-0.0055071,-0.0206382},
{-0.0128382,-0.0415510,0.0602716},

{-0.0030636,0.0106443,-0.0174469},
{-0.0043991,0.0198452,-0.0128846},
{0.0189448,-0.1026538,0.0375409},
{0.0037592,-0.0885273,0.0196740},
{0.0167579,-0.1059145,0.0461500},
{0.0097605,0.0229634,0.0055620},
{0.0119557,0.0109470,0.0150667},
{0.0011185,0.0180367,-0.0147857},
{0.0163664,-0.0139870,-0.0091453},
{0.0192740,-0.0091488,-0.0078427},
{-0.0088559,-0.0291543,0.0314507},
{-0.0110991,-0.0414582,0.0332446},
{-0.0136423,-0.0740498,0.0265938},
{-0.0014411,0.0031946,0.0207705},
{0.0042277,0.0087902,0.0179192},
{-0.0025604,-0.0034248,0.0265349},
{0.0002613,-0.0370016,0.0673037},
{0.0070215,-0.0521606,0.0619404},
{-0.0118916,-0.0238497,0.0648847},
{-0.0118641,-0.0316281,0.0640891},
{0.0091857,0.0243367,0.0027382},
{0.0173756,0.0169989,0.0014529},
{-0.0072395,-0.0131147,0.0660618},
{0.0154683,-0.0638785,0.0355430},
{-0.0126065,-0.0571576,0.0496871},
{-0.0152472,-0.0606152,0.0227595},
{0.0111304,-0.1013197,0.0523300},
{-0.0099510,-0.0303360,0.0183100},
{-0.0102569,-0.0184732,0.0176051},
{-0.0044943,0.0192226,0.0130653},
{0.0009080,-0.0819067,0.0578523},
{0.0059776,-0.0755142,0.0580301},
{-0.0022925,-0.0751397,0.0575079},
{0.0168224,-0.0885441,0.0321212},
{-0.0126424,-0.0219838,0.0506205},
{0.0108290,-0.0298696,0.0178229},
{0.0052307,-0.0630828,0.0605831},
{0.0084278,-0.0421592,0.0644109},
{0.0096807,-0.1012611,0.0205235},
{-0.0068785,-0.0635392,0.0588221},
{0.0181908,-0.0514098,0.0454632},
{-0.0120941,-0.0513887,0.0218663},
{0.0214988,-0.0247525,0.0386759},
{-0.0040878,-0.0731587,0.0209761},
{0.0168906,-0.0023891,0.0131942},
{0.0062621,0.0064819,0.0180735},

Biology Open • Supplementary information

{0.0009292,-0.0389005,0.0671820},
{-0.0150378,0.0164525,-0.0052416},
{0.0107610,0.0187929,0.0104964},
{-0.0108256,0.0191746,0.0084223},
{-0.0188780,-0.0065805,-0.0053328},
{-0.0189905,0.0006087,-0.0076497},
{-0.0049718,-0.0209361,0.0685443},
{-0.0133395,-0.0410810,0.0449421},
{0.0056677,-0.0560546,0.0619112},
{0.0126147,-0.0497833,0.0595944},
{-0.0088677,-0.0816628,0.0262104},
{0.0128147,0.0084737,0.0155158},
{0.0184969,-0.0127839,-0.0011583},
{-0.0175752,-0.0096051,0.0051629},
{-0.0129604,-0.0671920,0.0398254},
{-0.0074190,-0.0201109,-0.0038329},
{-0.0131235,-0.0328872,0.0481720},
{0.0047205,-0.0893515,0.0562532},
{0.0162061,-0.0595595,0.0530326},
{-0.0017605,-0.0415806,0.0112767},
{0.0063383,-0.0381842,0.0134413},
{0.0190466,-0.0083126,0.0059108},
{0.0183909,-0.0971224,0.0426426},
{-0.0092660,-0.0225748,0.0256729},
{-0.0008916,0.0154498,0.0164304},
{0.0075120,-0.0813795,0.0216402},
{0.0089223,-0.0743660,0.0573715},
{0.0152398,-0.0154559,-0.0096311},
{0.0005641,-0.0108502,0.0656801},
{-0.0072546,-0.0175771,-0.0144402},
{0.0161268,-0.0840155,0.0479026},
{0.0078004,-0.0219461,-0.0105399},
{-0.0107691,-0.0768176,0.0236631},
{0.0135053,-0.0468478,0.0295696},
{0.0157418,-0.0050961,0.0548831},
{0.0048128,-0.0090995,0.0400058},
{-0.0030801,-0.0735656,0.0580757},
{-0.0129886,-0.0709062,0.0387838},
{-0.0146628,-0.0136052,0.0549196},
{-0.0079729,0.0146083,-0.0148892},
{-0.0174166,0.0068885,0.0082820},
{0.0094825,-0.0810597,0.0225606},
{0.0025297,-0.0462754,0.0651353},
{-0.0136971,-0.0257949,0.0558678},
{0.0065322,-0.0072957,-0.0199698},
{0.0156260,-0.0335977,0.0574277},

Biology Open • Supplementary information

{0.0121349,-0.0178022,0.0630160},
{0.0105691,0.0024582,-0.0183937},
{0.0139033,-0.0096221,0.0174678},
{-0.0131250,0.0131212,0.0118803},
{0.0105069,-0.0622534,0.0584963},
{-0.0055437,-0.0452084,0.0665942},
{0.0197198,-0.0342085,0.0508596},
{0.0160727,-0.0155712,-0.0071238},
{-0.0151338,-0.0590689,0.0371047},
{-0.0076590,-0.0867312,0.0460022},
{0.0032450,-0.0618949,0.0613344},
{-0.0044772,-0.0487021,0.0142649},
{0.0112948,0.0125687,-0.0148723},
{-0.0021737,-0.0254005,-0.0008593},
{-0.0092977,-0.0861534,0.0307670},
{-0.0104755,-0.0041153,-0.0178861},
{-0.0029576,0.0254646,-0.0047581},
{-0.0097240,-0.0255112,0.0135064},
{-0.0108942,-0.0756810,0.0234824},
{-0.0060316,-0.0877756,0.0252821},
{0.0009667,0.0139245,-0.0170143},
{0.0201287,-0.0305191,0.0500773},
{-0.0059799,-0.0681100,0.0202872},
{0.0178840,-0.0426317,0.0404711},
{0.0178581,-0.0361378,0.0360575},
{-0.0145669,-0.0140404,-0.0009993},
{0.0134634,0.0164510,0.0103481},
{-0.0135816,-0.0128051,0.0071074},
{-0.0108537,-0.0413014,0.0366887},
{0.0182209,-0.1029431,0.0435511},
{0.0179403,-0.0121215,0.0571691},
{0.0190423,-0.0182513,0.0529629},
{0.0013819,-0.0310533,0.0051554},
{0.0109516,-0.0273836,0.0644985},
{-0.0028312,-0.0111821,0.0322345},
{-0.0195668,-0.0049732,-0.0017847},
{0.0027991,-0.0246209,-0.0039111},
{-0.0098297,-0.0833208,0.0379380},
{-0.0129294,-0.0321843,0.0625403},
{0.0181597,0.0060766,-0.0110482},
{-0.0149919,-0.0728971,0.0309239},
{-0.0154607,-0.0049156,0.0131313},
{0.0121743,-0.0094071,0.0277680},
{-0.0136320,-0.0501452,0.0328903},
{0.0152732,-0.0837451,0.0295480},
{0.0007809,0.0039422,-0.0201295},

Biology Open • Supplementary information

{0.0171349,-0.0906600,0.0322402},
{0.0070462,0.0074639,0.0173176},
{-0.0144993,0.0026431,0.0132965},
{0.0059381,-0.0501939,0.0170456},
{-0.0065723,-0.0222480,0.0349351},
{0.0208589,-0.0315194,0.0441286},
{-0.0130998,-0.0731850,0.0236120},
{0.0211688,0.0059787,-0.0031273},
{-0.0140848,-0.0733789,0.0287704},
{0.0114350,-0.0261493,0.0114700},
{0.0200372,-0.0136676,0.0470766},
{0.0087934,0.0022740,-0.0193661},
{0.0149776,-0.0097063,0.0140386},
{-0.0115142,-0.0742745,0.0416903},
{0.0004704,0.0240636,0.0095585},
{0.0028857,0.0038602,-0.0204594},
{0.0383249,-0.4710340,0.0218631},
{0.0051939,0.0238443,-0.0081005},
{-0.0138777,-0.0525754,0.0388275},
{-0.0322513,-0.4662637,-0.0280147},
{0.0023806,0.0269755,-0.0000332},
{0.0147652,-0.0286603,0.0276871},
{-0.0145032,-0.0012851,-0.0151303},
{0.0170074,-0.0361909,0.0551902},
{-0.0150553,0.0157078,0.0049389},
{0.0177309,-0.0468193,0.0417080},
{0.0158242,-0.0522314,0.0357748},
{-0.0136358,-0.0411991,0.0476770},
{0.0178045,-0.0661556,0.0458856},
{-0.0171857,-0.0574884,0.0286352},
{0.0031400,-0.0656790,0.0605536},
{0.0011174,-0.0612696,0.0180841},
{0.0219983,-0.0249652,0.0458711},
{0.0081219,-0.0912295,0.0548897},
{-0.0061107,-0.0990447,0.0290950},
{-0.0194960,-0.0025704,0.0031803},
{0.0147777,-0.0160027,0.0205595},
{0.0142131,-0.0795170,0.0286072},
{-0.0095414,-0.0294789,0.0286511},
{-0.0200169,-0.4780533,0.0371747},
{-0.0088822,-0.0665755,0.0543244},
{-0.0117531,-0.4715902,-0.0107018},
{0.0109539,-0.0516153,0.0233181},
{0.0111328,0.0173243,-0.0116528},
{-0.0249933,-0.4676988,0.0300797},
{0.0160458,-0.0634098,0.0364603},

{0.0003060,-0.0591284,0.0623112},
{-0.0112634,0.0215884,0.0029608},
{-0.0025303,-0.0199239,0.0683990},
{-0.0074802,-0.0087928,0.0251026},
{0.0198708,-0.0122959,0.0439383},
{0.0152173,-0.0292231,0.0595268},
{-0.0144900,-0.0130140,0.0541578},
{0.0203094,0.0098271,-0.0029211},
{-0.0102727,-0.0407096,0.0186558},
{-0.0003452,-0.0168515,-0.0171061},
{-0.0110416,-0.0718659,0.0459587},
{-0.0092268,0.0204326,0.0083445},
{0.0100020,0.0228402,-0.0031226},
{-0.0137252,-0.0322616,0.0593091},
{-0.0061278,-0.4682816,0.0421376},
{-0.0137608,-0.4746483,-0.0394942},
{-0.0136845,-0.4745846,-0.0386779},
{-0.0136081,-0.4745210,-0.0378616},
{-0.0135318,-0.4744573,-0.0370453},
{-0.0134555,-0.4743936,-0.0362289},
{-0.0133792,-0.4743300,-0.0354126},
{-0.0133028,-0.4742663,-0.0345963},
{-0.0132265,-0.4742026,-0.0337800},
{-0.0131502,-0.4741390,-0.0329637},
{-0.0130738,-0.4740753,-0.0321473},
{-0.0129975,-0.4740116,-0.0313310},
{-0.0129212,-0.4739480,-0.0305147},
{-0.0128448,-0.4738843,-0.0296984},
{-0.0127685,-0.4738206,-0.0288821},
{-0.0126922,-0.4737570,-0.0280658},
{-0.0126159,-0.4736933,-0.0272494},
{-0.0125395,-0.4736296,-0.0264331},
{-0.0124632,-0.4735660,-0.0256168},
{-0.0123869,-0.4735023,-0.0248005},
{-0.0123105,-0.4734386,-0.0239842},
{-0.0122342,-0.4733749,-0.0231678},
{-0.0121579,-0.4733113,-0.0223515},
{-0.0120815,-0.4732476,-0.0215352},
{-0.0120052,-0.4731839,-0.0207189},
{-0.0119289,-0.4731203,-0.0199026},
{-0.0118526,-0.4730566,-0.0190863},
{-0.0117762,-0.4729929,-0.0182699},
{-0.0116999,-0.4729293,-0.0174536},
{-0.0116236,-0.4728656,-0.0166373},
{-0.0115472,-0.4728019,-0.0158210},
{-0.0114709,-0.4727383,-0.0150047},

Biology Open • Supplementary information

{-0.0113946,-0.4726746,-0.0141883},
{-0.0113182,-0.4726109,-0.0133720},
{-0.0112419,-0.4725473,-0.0125557},
{-0.0111656,-0.4724836,-0.0117394},
{-0.0110892,-0.4724199,-0.0109231},
{-0.0110129,-0.4723563,-0.0101068},
{-0.0109366,-0.4722926,-0.0092904},
{-0.0108603,-0.4722289,-0.0084741},
{-0.0107839,-0.4721653,-0.0076578},
{-0.0107076,-0.4721016,-0.0068415},
{-0.0106313,-0.4720379,-0.0060252},
{-0.0105549,-0.4719743,-0.0052088},
{-0.0104786,-0.4719106,-0.0043925},
{-0.0104023,-0.4718469,-0.0035762},
{-0.0103259,-0.4717833,-0.0027599},
{-0.0102496,-0.4717196,-0.0019436},
{-0.0101733,-0.4716559,-0.0011273},
{-0.0100970,-0.4715923,-0.0003109},
{-0.0100206,-0.4715286,0.0005054},
{-0.0099443,-0.4714649,0.0013217},
{-0.0098680,-0.4714013,0.0021380},
{-0.0097916,-0.4713376,0.0029543},
{-0.0097153,-0.4712739,0.0037707},
{-0.0096390,-0.4712103,0.0045870},
{-0.0095626,-0.4711466,0.0054033},
{-0.0094863,-0.4710829,0.0062196},
{-0.0094100,-0.4710193,0.0070359},
{-0.0093337,-0.4709556,0.0078522},
{-0.0092573,-0.4708919,0.0086686},
{-0.0091810,-0.4708283,0.0094849},
{-0.0091047,-0.4707646,0.0103012},
{-0.0090283,-0.4707009,0.0111175},
{-0.0089520,-0.4706373,0.0119338},
{-0.0088757,-0.4705736,0.0127502},
{-0.0087993,-0.4705099,0.0135665},
{-0.0087230,-0.4704463,0.0143828},
{-0.0086467,-0.4703826,0.0151991},
{-0.0085704,-0.4703189,0.0160154},
{-0.0084940,-0.4702553,0.0168317},
{-0.0084177,-0.4701916,0.0176481},
{-0.0083414,-0.4701279,0.0184644},
{-0.0082650,-0.4700642,0.0192807},
{-0.0081887,-0.4700006,0.0200970},
{-0.0081124,-0.4699369,0.0209133},
{-0.0080360,-0.4698732,0.0217297},
{-0.0079597,-0.4698096,0.0225460},

Biology Open • Supplementary information

```
                    {-0.0078834,-0.4697459,0.0233623},
                    {-0.0078071,-0.4696822,0.0241786},
                    {-0.0077307,-0.4696186,0.0249949},
                    {-0.0076544,-0.4695549,0.0258112},
                    {-0.0075781,-0.4694912,0.0266276},
                    {-0.0075017,-0.4694276,0.0274439},
                    {-0.0074254,-0.4693639,0.0282602},
                    {-0.0073491,-0.4693002,0.0290765},
                    {-0.0072727,-0.4692366,0.0298928},
                    {-0.0071964,-0.4691729,0.0307092},
                    {-0.0071201,-0.4691092,0.0315255},
                    {-0.0070438,-0.4690456,0.0323418},
                    {-0.0069674,-0.4689819,0.0331581},
                    {-0.0068911,-0.4689182,0.0339744},
                    {-0.0068148,-0.4688546,0.0347907},
                    {-0.0067384,-0.4687909,0.0356071},
                    {-0.0066621,-0.4687272,0.0364234},
                    {-0.0065858,-0.4686636,0.0372397},
                    {-0.0065094,-0.4685999,0.0380560},
                    {-0.0064331,-0.4685362,0.0388723},
                    {-0.0063568,-0.4684726,0.0396887},
                    {-0.0062805,-0.4684089,0.0405050},
                    {-0.0062041,-0.4683452,0.0413213}
                    };
            BoundingBoxOnOff = Off;
        };
        AnyFunTransform3DIdentity ScaleFunction = {
            PreTransforms = {&.RBFTransform};
};
};

};
};
```

Biology Open • Supplementary information

ScalingFunctionTLEMLucyFemur_Mirrored_2014016

```
AnyFolder ScalingFunctionTLEMLucyFemur_Mirrored = {
  AnyFolder Left = {
        AnyFolder Thigh = {
      AnyMatrix AMirroring = {
      {1,0,0},
      {0,1,0},
      {0,0,-1}
      };
        AnyFunTransform3DRBF RBFTransform = {
                RBFDef.Type = RBF_ThinPlate;
                PolynomDegree = 1;
                Points0 = ({
                        {0.0000000,0.0000000,0.0000000},
                        {-0.0000000,-0.3616821,0.0000000},
                        {-0.0097563,-0.3678799,0.0012967},
                        {-0.0000000,-0.3660632,0.0408203},
                        {-0.0000000,-0.3573010,-0.0408203},
                        {0.0161460,-0.0072838,0.0601290},
                        {0.0220217,-0.0203698,0.0463848},
                        {0.0123977,-0.0241932,0.0668573},
                        {0.0006898,0.0018121,0.0538181},
                        {0.0122809,-0.0068668,0.0414535},
                        {-0.0058991,-0.0138188,0.0648412},
                        {0.0211469,-0.0380855,0.0538111},
                        {0.0172133,-0.0317342,0.0328381},
                        {0.0177311,-0.0131946,0.0256176},
                        {-0.0110079,-0.0308867,0.0632370},
                        {0.0039435,-0.0395977,0.0638271},
                        {-0.0041683,-0.0187472,0.0413214},
                        {-0.0177999,-0.0021535,0.0496084},
                        {0.0001603,-0.0053126,0.0279593},
                        {-0.0211600,-0.0216243,0.0497147},
                        {0.0096163,-0.0568663,0.0545719},
                        {0.0162112,-0.0515013,0.0395230},
                        {0.0093533,-0.0433265,0.0187387},
                        {0.0119398,-0.0238469,0.0118403},
                        {0.0210707,-0.0048848,0.0065878},
                        {0.0125013,0.0064094,0.0177702},
                        {-0.0191523,-0.0356053,0.0382429},
                        {-0.0115821,-0.0474937,0.0537648},
                        {-0.0100073,-0.0236371,0.0267322},
                        {-0.0078925,0.0107098,0.0182833},
                        {-0.0134217,-0.0055356,0.0182000},
                        {-0.0090061,-0.0665700,0.0475484},
```

{0.0071095,-0.0761976,0.0520035},
{0.0182119,-0.0704149,0.0386546},
{0.0073764,-0.0609481,0.0221006},
{-0.0122984,-0.0439585,0.0198428},
{-0.0032253,-0.0306792,0.0110995},
{-0.0012975,-0.0198451,-0.0052592},
{0.0140405,-0.0161125,-0.0071649},
{0.0189222,-0.0014898,-0.0127226},
{0.0181642,0.0132812,-0.0011050},
{0.0045119,0.0200001,0.0091189},
{-0.0176761,-0.0541983,0.0348587},
{-0.0136548,-0.0142015,0.0054311},
{-0.0135510,0.0182940,0.0017175},
{-0.0223865,0.0023399,0.0028018},
{-0.0086455,-0.0738247,0.0283843},
{-0.0050356,-0.0877749,0.0442195},
{0.0160135,-0.0919319,0.0465120},
{0.0128290,-0.0785810,0.0232431},
{-0.0168328,-0.0616327,0.0155917},
{-0.0172369,-0.0094567,-0.0119502},
{0.0002090,-0.0093641,-0.0209779},
{0.0064827,0.0081538,-0.0198453},
{0.0010777,0.0210422,-0.0084721},
{-0.0129485,0.0085451,-0.0162212},
{-0.0002322,-0.0924033,0.0245644},
{-0.0028175,-0.1052263,0.0418294},
{0.0172322,-0.1111444,0.0445760},
{0.0203259,-0.0957409,0.0271449},
{0.0057102,-0.1088933,0.0195113},
{-0.0010852,-0.1230776,0.0363132},
{0.0182727,-0.1303312,0.0422884},
{0.0242891,-0.1161703,0.0265054},
{0.0094531,-0.1268584,0.0162294},
{0.0014244,-0.1413293,0.0331653},
{0.0207271,-0.1496427,0.0394157},
{0.0267562,-0.1360142,0.0236798},
{0.0113839,-0.1461651,0.0136024},
{0.0030845,-0.1595094,0.0297156},
{0.0209528,-0.1691386,0.0369775},
{0.0283958,-0.1540209,0.0213795},
{0.0129499,-0.1649012,0.0113589},
{0.0016490,-0.1783108,0.0216287},
{0.0147105,-0.1880678,0.0328614},
{0.0298491,-0.1859865,0.0248551},
{0.0286645,-0.1714377,0.0169488},
{0.0165897,-0.1861006,0.0082595},

Biology Open • Supplementary information

{0.0014000,-0.1973476,0.0183740},
{0.0114301,-0.2109792,0.0286157},
{0.0267422,-0.2037622,0.0296951},
{0.0288630,-0.2018335,0.0130127},
{0.0145471,-0.2083505,0.0062833},
{0.0011300,-0.2198071,0.0155602},
{0.0126610,-0.2321235,0.0275354},
{0.0275647,-0.2219268,0.0265185},
{0.0286645,-0.2230253,0.0098701},
{0.0130434,-0.2304822,0.0039177},
{0.0022984,-0.2409585,0.0149650},
{0.0101315,-0.2534740,0.0252125},
{0.0279485,-0.2428555,0.0234276},
{0.0276684,-0.2421559,0.0058691},
{0.0101250,-0.2529527,0.0018983},
{0.0021376,-0.2667831,0.0107073},
{0.0114309,-0.2734905,0.0249960},
{0.0273923,-0.2634953,0.0216324},
{0.0278429,-0.2592546,0.0037692},
{0.0159101,-0.2712985,-0.0033988},
{0.0036708,-0.2863280,0.0004644},
{0.0023115,-0.2884756,0.0187554},
{0.0231385,-0.2879051,0.0224853},
{0.0304009,-0.2799973,0.0083549},
{0.0238189,-0.2917246,-0.0052462},
{0.0064904,-0.3027687,-0.0070587},
{-0.0003492,-0.3064690,0.0093014},
{0.0122179,-0.3045269,0.0243511},
{0.0301837,-0.3030127,0.0114462},
{0.0234182,-0.3107336,-0.0085198},
{0.0046893,-0.3214995,-0.0123881},
{-0.0022352,-0.3264905,0.0043340},
{0.0016841,-0.3202756,0.0217839},
{0.0229722,-0.3196887,0.0215083},
{0.0292438,-0.3228042,0.0051430},
{0.0221641,-0.3296190,-0.0132361},
{0.0100459,-0.3417597,-0.0244560},
{-0.0053009,-0.3375737,-0.0137705},
{-0.0064432,-0.3487307,0.0028676},
{-0.0059346,-0.3407801,0.0202938},
{0.0105130,-0.3365976,0.0264175},
{0.0282695,-0.3386249,0.0180307},
{0.0270402,-0.3431844,-0.0001487},
{0.0257040,-0.3506950,-0.0181851},
{0.0178948,-0.3636114,-0.0302838},
{0.0043149,-0.3544109,-0.0381949},

Biology Open • Supplementary information

{-0.0095210,-0.3461482,-0.0295031},
{-0.0164526,-0.3542895,-0.0126666},
{-0.0060896,-0.3690127,-0.0033061},
{-0.0176523,-0.3612686,0.0110109},
{-0.0153310,-0.3546943,0.0299634},
{0.0021329,-0.3508021,0.0350763},
{0.0210690,-0.3548628,0.0279835},
{0.0356594,-0.3558162,0.0159356},
{0.0275718,-0.3617450,-0.0018607},
{0.0298503,-0.3741530,-0.0163721},
{0.0175320,-0.3855300,-0.0277515},
{0.0025604,-0.3740099,-0.0397137},
{-0.0136727,-0.3624016,-0.0402102},
{-0.0277647,-0.3567472,-0.0284947},
{-0.0275446,-0.3699630,-0.0120829},
{-0.0175846,-0.3859355,-0.0177922},
{0.0001572,-0.3856873,-0.0118050},
{0.0030262,-0.3834856,0.0075553},
{-0.0144114,-0.3810887,0.0140304},
{-0.0247706,-0.3701239,0.0285134},
{-0.0044804,-0.3677201,0.0404632},
{0.0133183,-0.3712274,0.0334208},
{0.0324861,-0.3723417,0.0245668},
{0.0271451,-0.3766027,0.0072441},
{0.0167985,-0.3822816,-0.0065349},
{-0.0001454,-0.3910823,-0.0303010},
{-0.0144998,-0.3834154,-0.0384137},
{-0.0301321,-0.3750841,-0.0321336},
{0.0196491,-0.3864804,0.0210853},
{0.0015726,-0.3888034,0.0276569},
{-0.0146652,-0.3847848,0.0336913},
{-0.0014588,-0.0619951,0.0219778},
{-0.0118435,-0.0550626,0.0139639},
{0.0129812,-0.0349966,0.0673128},
{-0.0162907,-0.0105666,0.0597739},
{-0.0012885,-0.0520816,0.0562989},
{-0.0090599,-0.0631298,0.0172224},
{-0.0000222,-0.0771663,0.0231897},
{-0.0068148,0.0023885,-0.0160086},
{-0.0180544,-0.0425409,0.0446585},
{0.0020525,-0.0249904,0.0691549},
{-0.0157097,-0.0662315,0.0238393},
{-0.0010518,-0.0510686,0.0199353},
{-0.0208874,-0.0152865,0.0500034},
{0.0168316,-0.0280857,0.0666680},
{-0.0187392,-0.0282333,0.0403282},

Biology Open • Supplementary information

{0.0191622,-0.0351633,0.0622707},
{0.0001216,-0.0706289,0.0524049},
{-0.0169862,-0.0399062,0.0311827},
{-0.0185737,-0.0533066,0.0151224},
{-0.0219058,-0.0528723,0.0222263},
{-0.0028271,-0.0391620,0.0148452},
{0.0117900,-0.0553425,0.0235348},
{0.0219835,-0.0106400,0.0396338},
{-0.0025366,-0.0401046,0.0625273},
{0.0141017,-0.0018767,0.0198108},
{-0.0078770,-0.0473929,0.0173542},
{-0.0065351,-0.0766431,0.0470258},
{0.0065585,0.0145593,0.0161638},
{-0.0118588,0.0172875,-0.0085064},
{-0.0074087,-0.0627500,0.0506700},
{0.0071940,-0.0780068,0.0213362},
{0.0126736,-0.0708176,0.0244108},
{0.0074694,-0.0436450,0.0604196},
{0.0154186,-0.0139471,0.0064312},
{-0.0053535,-0.0213210,0.0040094},
{-0.0088733,0.0002018,0.0205402},
{-0.0112471,-0.0542608,0.0500266},
{-0.0061625,-0.0248673,0.0653244},
{0.0093695,-0.0065826,0.0614157},
{0.0138015,-0.0467322,0.0284333},
{-0.0020999,-0.0186885,-0.0116392},
{-0.0185640,-0.0225591,0.0566779},
{0.0175798,0.0124360,-0.0068467},
{-0.0039962,0.0217127,0.0053055},
{0.0014975,-0.0784591,0.0514056},
{-0.0172997,-0.0386971,0.0525252},
{0.0196540,-0.0268271,0.0416806},
{0.0135296,-0.0406504,0.0236882},
{0.0182471,0.0095959,0.0087906},
{0.0218667,0.0016804,0.0058793},
{0.0224975,-0.0022652,-0.0022994},
{0.0166750,-0.0191391,0.0236436},
{0.0119785,-0.0328723,0.0195376},
{0.0187631,-0.0133469,0.0308741},
{-0.0187543,-0.0461463,0.0257491},
{0.0039834,-0.0373928,0.0683089},
{0.0157029,-0.0423169,0.0385226},
{0.0164003,-0.0087069,0.0131181},
{0.0008253,0.0033370,-0.0212576},
{0.0101707,-0.0009724,-0.0205688},
{0.0102948,-0.0642683,0.0524532},

Biology Open • Supplementary information

{0.0152217,-0.0557944,0.0508978},
{0.0172973,-0.0565707,0.0419468},
{0.0167723,-0.0635334,0.0369834},
{-0.0047397,-0.0695325,0.0241398},
{-0.0152913,0.0035778,0.0161461},
{0.0091697,0.0009340,0.0575160},
{0.0196686,-0.0089533,0.0500006},
{0.0183998,-0.0212943,0.0622968},
{-0.0114340,-0.0316810,0.0250940},
{-0.0206442,-0.0092599,-0.0028425},
{0.0166868,-0.0784295,0.0276925},
{-0.0069678,-0.0054521,-0.0207011},
{0.0049490,0.0151109,-0.0160093},
{-0.0084025,-0.0318937,0.0162788},
{-0.0173679,0.0095594,-0.0108509},
{-0.0218912,0.0063457,-0.0020698},
{-0.0106547,-0.0668750,0.0397749},
{0.0136098,-0.0170666,-0.0018890},
{0.0141599,-0.0441405,0.0587594},
{0.0093255,-0.0183218,-0.0083008},
{0.0106367,-0.0128276,-0.0154269},
{0.0037350,-0.0549365,0.0209786},
{0.0087662,-0.0472902,0.0195496},
{0.0016839,-0.0708213,0.0221325},
{-0.0202977,-0.0530540,0.0301270},
{-0.0068584,0.0021781,0.0530002},
{-0.0144817,-0.0297218,0.0605833},
{0.0197015,-0.0165217,0.0560654},
{0.0156609,-0.0705363,0.0477736},
{0.0207876,-0.0221433,0.0330733},
{0.0092324,-0.0300767,0.0713160},
{-0.0203392,-0.0034488,0.0097332},
{0.0156978,-0.0143438,0.0629053},
{0.0143267,-0.0563519,0.0317489},
{-0.0161685,-0.0465391,0.0188591},
{-0.0196397,-0.0309875,0.0546496},
{0.0154804,-0.0380422,0.0305433},
{-0.0077774,-0.0351303,0.0637765},
{0.0063980,-0.0086419,-0.0201654},
{0.0030146,-0.0151094,-0.0165974},
{-0.0210080,-0.0096030,0.0507855},
{-0.0105684,-0.0411377,0.0586146},
{0.0180619,-0.0498960,0.0469835},
{-0.0192318,0.0116108,0.0024070},
{-0.0165651,0.0083622,0.0126870},
{-0.0014333,-0.0627138,0.0534746},

Biology Open • Supplementary information

{0.0145127,-0.0080178,0.0353667},
{0.0148374,-0.0012228,0.0566181},
{0.0153834,-0.0062427,0.0444727},
{-0.0155164,-0.0132103,-0.0096398},
{0.0068685,-0.0221022,0.0011547},
{0.0003332,-0.0458500,0.0183716},
{0.0205582,0.0040956,-0.0082969},
{0.0175752,0.0009340,-0.0148407},
{0.0142357,-0.0063623,-0.0169332},
{-0.0147285,0.0023412,-0.0164678},
{-0.0158781,-0.0064451,-0.0151346},
{0.0175951,0.0133456,0.0037559},
{0.0117660,0.0189899,-0.0030690},
{0.0096040,0.0151827,-0.0137450},
{0.0096565,-0.0215194,0.0662734},
{-0.0006793,-0.0074494,0.0631218},
{0.0007819,-0.0004519,0.0600781},
{0.0065377,-0.0125433,0.0654199},
{0.0090461,0.0073096,0.0188711},
{0.0151418,-0.0168302,0.0146465},
{0.0025166,-0.0231863,0.0688894},
{-0.0097891,-0.0152077,0.0253086},
{-0.0109149,-0.0177935,0.0119825},
{-0.0072079,-0.0099373,0.0272725},
{-0.0183727,-0.0467429,0.0356276},
{-0.0126871,-0.0584555,0.0428141},
{-0.0057054,-0.0273042,0.0103554},
{0.0215142,-0.0159693,0.0474531},
{0.0128492,-0.0070016,0.0287645},
{0.0052134,-0.0017047,0.0247463},
{-0.0024284,-0.0707498,0.0513049},
{-0.0036140,0.0220593,-0.0040484},
{-0.0002405,-0.0121253,0.0390050},
{-0.0119811,-0.0156737,0.0015445},
{0.0142082,-0.0279792,0.0246553},
{0.0122244,-0.0262013,0.0169829},
{-0.0146264,-0.0038855,0.0549384},
{0.0207450,-0.0325771,0.0496412},
{-0.0155040,-0.0497560,0.0437087},
{-0.0081119,-0.0756450,0.0387365},
{0.0039684,-0.0062047,0.0313900},
{-0.0023726,-0.0038892,-0.0220521},
{-0.0136977,-0.0339839,0.0602619},
{-0.0035399,0.0089081,-0.0188894},
{-0.0049820,0.0176571,-0.0132254},
{0.0193902,-0.0803191,0.0360429},

Biology Open • Supplementary information

{0.0040648,-0.0697109,0.0218205},
{0.0170470,-0.0818610,0.0456892},
{0.0104175,0.0188635,0.0064965},
{0.0135206,0.0092549,0.0158079},
{0.0023933,0.0160498,-0.0157404},
{0.0164753,-0.0118197,-0.0096995},
{0.0202584,-0.0066858,-0.0082383},
{-0.0098401,-0.0226001,0.0314028},
{-0.0130937,-0.0309622,0.0320764},
{-0.0201510,-0.0613297,0.0222264},
{-0.0010389,0.0036055,0.0209003},
{0.0042025,0.0073392,0.0199393},
{-0.0021630,-0.0038531,0.0251898},
{-0.0007845,-0.0293574,0.0675788},
{0.0070027,-0.0406327,0.0624565},
{-0.0124363,-0.0183001,0.0627966},
{-0.0120485,-0.0256613,0.0628493},
{0.0101200,0.0197695,0.0039334},
{0.0167636,0.0148266,0.0014155},
{-0.0074981,-0.0106930,0.0632797},
{0.0142533,-0.0503445,0.0351954},
{-0.0146107,-0.0460953,0.0506120},
{-0.0161117,-0.0471301,0.0178900},
{0.0112149,-0.0792793,0.0501460},
{-0.0099060,-0.0230794,0.0186951},
{-0.0114543,-0.0153049,0.0183675},
{-0.0042882,0.0161879,0.0151423},
{0.0014582,-0.0638572,0.0541080},
{0.0048554,-0.0582603,0.0553815},
{-0.0017077,-0.0572302,0.0546635},
{0.0166090,-0.0697169,0.0323038},
{-0.0208812,-0.0164273,0.0474354},
{0.0130454,-0.0241864,0.0178294},
{0.0053123,-0.0483770,0.0582251},
{0.0112225,-0.0361429,0.0673300},
{0.0085729,-0.0771256,0.0216100},
{-0.0070783,-0.0488033,0.0560869},
{0.0182083,-0.0411024,0.0454236},
{-0.0119590,-0.0403809,0.0213546},
{0.0233409,-0.0185535,0.0366343},
{-0.0046347,-0.0571211,0.0208243},
{0.0183166,-0.0006966,0.0140116},
{0.0061913,0.0053512,0.0201003},
{-0.0002364,-0.0310828,0.0680351},
{-0.0170773,0.0139270,-0.0054987},
{0.0110805,0.0156034,0.0117010},

Biology Open • Supplementary information

{-0.0124628,0.0164667,0.0094550},
{-0.0209014,-0.0073407,-0.0051969},
{-0.0210131,0.0010693,-0.0086891},
{-0.0041596,-0.0172332,0.0663041},
{-0.0216595,-0.0322172,0.0451540},
{0.0051908,-0.0426014,0.0613013},
{0.0150003,-0.0416136,0.0606257},
{-0.0135090,-0.0674234,0.0236425},
{0.0146416,0.0073691,0.0160052},
{0.0195615,-0.0112412,-0.0010333},
{-0.0198115,-0.0098244,0.0044735},
{-0.0157054,-0.0521236,0.0395039},
{-0.0081381,-0.0173108,-0.0046152},
{-0.0218324,-0.0262661,0.0471115},
{0.0051446,-0.0707257,0.0530555},
{0.0179359,-0.0471493,0.0528134},
{-0.0005591,-0.0317983,0.0113632},
{0.0071707,-0.0305943,0.0121186},
{0.0206239,-0.0061367,0.0066642},
{0.0187057,-0.0745868,0.0420207},
{-0.0095784,-0.0163113,0.0259524},
{-0.0012181,0.0127661,0.0186855},
{0.0059420,-0.0630691,0.0220087},
{0.0069408,-0.0573878,0.0552614},
{0.0150782,-0.0132884,-0.0101793},
{0.0005533,-0.0078574,0.0633775},
{-0.0088284,-0.0146399,-0.0147837},
{0.0167444,-0.0651105,0.0470347},
{0.0082666,-0.0181741,-0.0095133},
{-0.0175162,-0.0637444,0.0177069},
{0.0153795,-0.0360767,0.0287608},
{0.0162363,-0.0018864,0.0548412},
{0.0052731,-0.0075919,0.0392990},
{-0.0022288,-0.0556644,0.0549909},
{-0.0156238,-0.0550038,0.0377688},
{-0.0204137,-0.0100550,0.0536429},
{-0.0093683,0.0122429,-0.0162013},
{-0.0202927,0.0049166,0.0083887},
{0.0074706,-0.0627945,0.0222133},
{0.0035034,-0.0376103,0.0672421},
{-0.0190752,-0.0194129,0.0555530},
{0.0061758,-0.0074961,-0.0207741},
{0.0192011,-0.0273063,0.0588233},
{0.0130796,-0.0134383,0.0638611},
{0.0120421,0.0019566,-0.0194140},
{0.0154152,-0.0065294,0.0179581},

{-0.0157335,0.0101219,0.0123981},
{0.0110376,-0.0488274,0.0567911},
{-0.0055170,-0.0343676,0.0647140},
{0.0216204,-0.0273988,0.0512041},
{0.0161331,-0.0135875,-0.0075501},
{-0.0184643,-0.0455633,0.0360087},
{-0.0091978,-0.0682413,0.0460847},
{0.0034304,-0.0471677,0.0588866},
{-0.0048025,-0.0384316,0.0149400},
{0.0118505,0.0109735,-0.0159161},
{-0.0027036,-0.0204189,-0.0015094},
{-0.0117607,-0.0692759,0.0292171},
{-0.0107080,-0.0033455,-0.0190487},
{-0.0042548,0.0214947,-0.0056846},
{-0.0097315,-0.0208529,0.0131997},
{-0.0178958,-0.0627218,0.0171551},
{-0.0068692,-0.0688818,0.0233316},
{0.0012083,0.0120695,-0.0186179},
{0.0215073,-0.0236027,0.0503069},
{-0.0060262,-0.0534931,0.0189523},
{0.0179951,-0.0336785,0.0397267},
{0.0190509,-0.0284972,0.0336394},
{-0.0169188,-0.0133609,-0.0014203},
{0.0135722,0.0136491,0.0114695},
{-0.0150549,-0.0123230,0.0067722},
{-0.0148411,-0.0311945,0.0355412},
{0.0185490,-0.0794042,0.0431266},
{0.0193568,-0.0087326,0.0575704},
{0.0201026,-0.0144353,0.0531586},
{0.0025583,-0.0248211,0.0045714},
{0.0132689,-0.0214866,0.0652899},
{-0.0028669,-0.0099311,0.0309271},
{-0.0219487,-0.0054893,-0.0021853},
{0.0026180,-0.0203806,-0.0040620},
{-0.0116230,-0.0668518,0.0370152},
{-0.0133007,-0.0264159,0.0619176},
{0.0186167,0.0050491,-0.0120059},
{-0.0191359,-0.0591690,0.0288149},
{-0.0178121,-0.0041834,0.0141448},
{0.0123077,-0.0063648,0.0279743},
{-0.0157729,-0.0374010,0.0315335},
{0.0149328,-0.0659100,0.0290095},
{0.0006205,0.0021977,-0.0214613},
{0.0170484,-0.0715687,0.0325980},
{0.0068499,0.0061192,0.0196608},
{-0.0167075,0.0020024,0.0150382},

Biology Open • Supplementary information

{0.0059801,-0.0393117,0.0167489},
{-0.0072506,-0.0171067,0.0338751},
{0.0202363,-0.0251596,0.0420997},
{-0.0199311,-0.0601255,0.0171522},
{0.0216641,0.0053410,-0.0027841},
{-0.0197665,-0.0606125,0.0252972},
{0.0129986,-0.0214528,0.0098002},
{0.0201151,-0.0100929,0.0460501},
{0.0104137,0.0017765,-0.0203073},
{0.0166557,-0.0064123,0.0140197},
{-0.0124120,-0.0596549,0.0422637},
{0.0014885,0.0202590,0.0096781},
{0.0028720,0.0020207,-0.0219044},
{0.0376095,-0.3675427,0.0214537},
{0.0049949,0.0206417,-0.0079017},
{-0.0191723,-0.0407590,0.0391310},
{-0.0316406,-0.3638215,-0.0274841},
{0.0021392,0.0226920,0.0000985},
{0.0168435,-0.0229382,0.0264788},
{-0.0157304,-0.0005437,-0.0161456},
{0.0203722,-0.0299750,0.0562762},
{-0.0171077,0.0134146,0.0059615},
{0.0175519,-0.0370653,0.0413374},
{0.0154657,-0.0405127,0.0348980},
{-0.0215172,-0.0323502,0.0481925},
{0.0177998,-0.0515776,0.0444709},
{-0.0183804,-0.0448316,0.0275139},
{0.0033607,-0.0502028,0.0575894},
{0.0010325,-0.0479377,0.0191141},
{0.0224852,-0.0181821,0.0451780},
{0.0084089,-0.0720538,0.0521031},
{-0.0070880,-0.0772912,0.0288023},
{-0.0223712,-0.0028990,0.0024047},
{0.0173136,-0.0120017,0.0204612},
{0.0137772,-0.0623537,0.0276621},
{-0.0100390,-0.0231180,0.0292222},
{-0.0196395,-0.3730207,0.0364730},
{-0.0094445,-0.0526688,0.0528111},
{-0.0115309,-0.3679776,-0.0104995},
{0.0129812,-0.0410477,0.0224072},
{0.0117394,0.0154849,-0.0117191},
{-0.0245208,-0.3649412,0.0295111},
{0.0147476,-0.0502966,0.0364899},
{0.0006132,-0.0446771,0.0600524},
{-0.0118123,0.0191139,0.0031977},
{-0.0017185,-0.0163303,0.0664216},

Biology Open • Supplementary information

{-0.0085098,-0.0074564,0.0247745},
{0.0197459,-0.0086852,0.0430460},
{0.0187064,-0.0223532,0.0600966},
{-0.0206552,-0.0099712,0.0525024},
{0.0207525,0.0084646,-0.0024846},
{-0.0094915,-0.0325446,0.0184441},
{-0.0014464,-0.0140050,-0.0179819},
{-0.0122725,-0.0573444,0.0463125},
{-0.0106397,0.0177211,0.0094669},
{0.0111981,0.0191236,-0.0041491},
{-0.0162790,-0.0256279,0.0592687},
{-0.0060120,-0.3653959,0.0413411},
{-0.0135007,-0.3703638,-0.0387476},
{-0.0134258,-0.3703142,-0.0379467},
{-0.0133509,-0.3702645,-0.0371458},
{-0.0132760,-0.3702148,-0.0363450},
{-0.0132011,-0.3701651,-0.0355441},
{-0.0131262,-0.3701154,-0.0347432},
{-0.0130513,-0.3700658,-0.0339423},
{-0.0129765,-0.3700161,-0.0331414},
{-0.0129016,-0.3699664,-0.0323405},
{-0.0128267,-0.3699167,-0.0315396},
{-0.0127518,-0.3698670,-0.0307388},
{-0.0126769,-0.3698174,-0.0299379},
{-0.0126020,-0.3697677,-0.0291370},
{-0.0125271,-0.3697180,-0.0283361},
{-0.0124523,-0.3696683,-0.0275352},
{-0.0123774,-0.3696187,-0.0267343},
{-0.0123025,-0.3695690,-0.0259334},
{-0.0122276,-0.3695193,-0.0251325},
{-0.0121527,-0.3694696,-0.0243317},
{-0.0120778,-0.3694199,-0.0235308},
{-0.0120029,-0.3693703,-0.0227299},
{-0.0119280,-0.3693206,-0.0219290},
{-0.0118532,-0.3692709,-0.0211281},
{-0.0117783,-0.3692212,-0.0203272},
{-0.0117034,-0.3691715,-0.0195263},
{-0.0116285,-0.3691219,-0.0187255},
{-0.0115536,-0.3690722,-0.0179246},
{-0.0114787,-0.3690225,-0.0171237},
{-0.0114038,-0.3689728,-0.0163228},
{-0.0113289,-0.3689231,-0.0155219},
{-0.0112541,-0.3688735,-0.0147210},
{-0.0111792,-0.3688238,-0.0139201},
{-0.0111043,-0.3687741,-0.0131192},
{-0.0110294,-0.3687244,-0.0123184},

Biology Open • Supplementary information

{-0.0109545,-0.3686748,-0.0115175},
{-0.0108796,-0.3686251,-0.0107166},
{-0.0108047,-0.3685754,-0.0099157},
{-0.0107298,-0.3685257,-0.0091148},
{-0.0106550,-0.3684760,-0.0083139},
{-0.0105801,-0.3684264,-0.0075130},
{-0.0105052,-0.3683767,-0.0067121},
{-0.0104303,-0.3683270,-0.0059113},
{-0.0103554,-0.3682773,-0.0051104},
{-0.0102805,-0.3682276,-0.0043095},
{-0.0102056,-0.3681780,-0.0035086},
{-0.0101307,-0.3681283,-0.0027077},
{-0.0100559,-0.3680786,-0.0019068},
{-0.0099810,-0.3680289,-0.0011059},
{-0.0099061,-0.3679792,-0.0003051},
{-0.0098312,-0.3679296,0.0004958},
{-0.0097563,-0.3678799,0.0012967},
{-0.0096814,-0.3678302,0.0020976},
{-0.0096065,-0.3677805,0.0028985},
{-0.0095317,-0.3677308,0.0036994},
{-0.0094568,-0.3676812,0.0045003},
{-0.0093819,-0.3676315,0.0053012},
{-0.0093070,-0.3675818,0.0061020},
{-0.0092321,-0.3675321,0.0069029},
{-0.0091572,-0.3674825,0.0077038},
{-0.0090823,-0.3674328,0.0085047},
{-0.0090074,-0.3673831,0.0093056},
{-0.0089326,-0.3673334,0.0101065},
{-0.0088577,-0.3672837,0.0109074},
{-0.0087828,-0.3672341,0.0117082},
{-0.0087079,-0.3671844,0.0125091},
{-0.0086330,-0.3671347,0.0133100},
{-0.0085581,-0.3670850,0.0141109},
{-0.0084832,-0.3670353,0.0149118},
{-0.0084083,-0.3669857,0.0157127},
{-0.0083335,-0.3669360,0.0165136},
{-0.0082586,-0.3668863,0.0173145},
{-0.0081837,-0.3668366,0.0181153},
{-0.0081088,-0.3667869,0.0189162},
{-0.0080339,-0.3667373,0.0197171},
{-0.0079590,-0.3666876,0.0205180},
{-0.0078841,-0.3666379,0.0213189},
{-0.0078092,-0.3665882,0.0221198},
{-0.0077344,-0.3665386,0.0229207},
{-0.0076595,-0.3664889,0.0237215},
{-0.0075846,-0.3664392,0.0245224},

{-0.0075097,-0.3663895,0.0253233},
{-0.0074348,-0.3663398,0.0261242},
{-0.0073599,-0.3662902,0.0269251},
{-0.0072850,-0.3662405,0.0277260},
{-0.0072101,-0.3661908,0.0285269},
{-0.0071353,-0.3661411,0.0293278},
{-0.0070604,-0.3660914,0.0301286},
{-0.0069855,-0.3660418,0.0309295},
{-0.0069106,-0.3659921,0.0317304},
{-0.0068357,-0.3659424,0.0325313},
{-0.0067608,-0.3658927,0.0333322},
{-0.0066859,-0.3658430,0.0341331},
{-0.0066111,-0.3657934,0.0349340},
{-0.0065362,-0.3657437,0.0357348},
{-0.0064613,-0.3656940,0.0365357},
{-0.0063864,-0.3656443,0.0373366},
{-0.0063115,-0.3655947,0.0381375},
{-0.0062366,-0.3655450,0.0389384},
{-0.0061617,-0.3654953,0.0397393},
{-0.0060868,-0.3654456,0.0405402}
}* .AMirroring);
Points1 = ({
{0.0000000,0.0000000,0.0000000},
{-0.0000000,-0.4374563,0.0000000},
{-0.0094266,-0.4449526,0.0012529},
{-0.0000000,-0.4427553,0.0394409},
{-0.0000000,-0.4321573,-0.0394409},
{0.0140331,-0.0098308,0.0565778},
{0.0203620,-0.0255678,0.0445293},
{0.0097861,-0.0290004,0.0617219},
{0.0001582,-0.0004522,0.0503626},
{0.0113646,-0.0077761,0.0392483},
{-0.0059910,-0.0159263,0.0640130},
{0.0170434,-0.0440199,0.0510037},
{0.0152701,-0.0382232,0.0327739},
{0.0144236,-0.0164583,0.0247953},
{-0.0102631,-0.0365551,0.0610785},
{0.0033999,-0.0478527,0.0600708},
{-0.0031127,-0.0229140,0.0407430},
{-0.0126350,-0.0049177,0.0474516},
{-0.0000090,-0.0048240,0.0275310},
{-0.0124069,-0.0260942,0.0487785},
{0.0101933,-0.0692574,0.0535851},
{0.0162108,-0.0615274,0.0381743},
{0.0084505,-0.0519532,0.0191417},
{0.0097727,-0.0276687,0.0124876},

{0.0185184,-0.0062774,0.0057081},
{0.0104789,0.0069210,0.0159300},
{-0.0123426,-0.0435263,0.0363007},
{-0.0101394,-0.0565818,0.0515759},
{-0.0093082,-0.0291261,0.0249195},
{-0.0061800,0.0121834,0.0159591},
{-0.0114690,-0.0067027,0.0157318},
{-0.0075062,-0.0800178,0.0447273},
{0.0063519,-0.0918186,0.0519585},
{0.0172626,-0.0860707,0.0368248},
{0.0083781,-0.0741208,0.0210328},
{-0.0118593,-0.0526285,0.0206706},
{-0.0042320,-0.0374095,0.0102133},
{-0.0004477,-0.0227053,-0.0048082},
{0.0128965,-0.0177953,-0.0065283},
{0.0174258,-0.0020457,-0.0111489},
{0.0170862,0.0148214,-0.0012137},
{0.0031580,0.0224924,0.0090157},
{-0.0143399,-0.0653307,0.0339183},
{-0.0119138,-0.0142758,0.0058056},
{-0.0121943,0.0193931,0.0016101},
{-0.0186025,0.0026885,0.0031766},
{-0.0068441,-0.0886311,0.0277759},
{-0.0043309,-0.1062699,0.0421167},
{0.0146953,-0.1116086,0.0448933},
{0.0125444,-0.0953582,0.0218087},
{-0.0101829,-0.0707882,0.0214703},
{-0.0146119,-0.0101030,-0.0102417},
{0.0006616,-0.0104337,-0.0187176},
{0.0060103,0.0088733,-0.0172288},
{0.0016911,0.0229528,-0.0081398},
{-0.0105781,0.0096027,-0.0145644},
{0.0004454,-0.1118004,0.0233586},
{-0.0018708,-0.1271060,0.0401600},
{0.0158259,-0.1345778,0.0428309},
{0.0188769,-0.1156938,0.0263862},
{0.0062768,-0.1313577,0.0193752},
{0.0000139,-0.1484169,0.0351666},
{0.0169394,-0.1575863,0.0406188},
{0.0225293,-0.1404263,0.0259038},
{0.0097650,-0.1529028,0.0165534},
{0.0023500,-0.1703923,0.0322925},
{0.0192752,-0.1808478,0.0378898},
{0.0248794,-0.1643281,0.0232860},
{0.0115328,-0.1761854,0.0141246},
{0.0038646,-0.1923425,0.0290591},

Biology Open • Supplementary information

{0.0195788,-0.2043690,0.0355678},
{0.0264418,-0.1860612,0.0210982},
{0.0129202,-0.1988534,0.0119522},
{0.0026565,-0.2150039,0.0215052},
{0.0140899,-0.2271405,0.0317362},
{0.0277446,-0.2247796,0.0241579},
{0.0267919,-0.2070813,0.0169139},
{0.0161443,-0.2245743,0.0088940},
{0.0023653,-0.2380669,0.0183624},
{0.0111603,-0.2548327,0.0277042},
{0.0249141,-0.2463032,0.0285983},
{0.0270471,-0.2438677,0.0130662},
{0.0142582,-0.2515244,0.0068946},
{0.0019972,-0.2653184,0.0155732},
{0.0122220,-0.2804966,0.0265715},
{0.0257192,-0.2683062,0.0255406},
{0.0269139,-0.2695411,0.0099852},
{0.0128296,-0.2783630,0.0045006},
{0.0029034,-0.2910293,0.0148397},
{0.0098790,-0.3063700,0.0243124},
{0.0261408,-0.2936663,0.0225473},
{0.0260660,-0.2927165,0.0061038},
{0.0100776,-0.3056217,0.0024140},
{0.0025883,-0.3223948,0.0106390},
{0.0110214,-0.3306774,0.0240298},
{0.0257208,-0.3186779,0.0207826},
{0.0262745,-0.3134547,0.0039935},
{0.0153575,-0.3279418,-0.0028025},
{0.0038900,-0.3461335,0.0007567},
{0.0025216,-0.3487839,0.0181330},
{0.0219331,-0.3482317,0.0215724},
{0.0287061,-0.3386509,0.0081652},
{0.0227405,-0.3527857,-0.0048048},
{0.0064102,-0.3661193,-0.0066299},
{-0.0001667,-0.3706180,0.0090440},
{0.0117577,-0.3683163,0.0234375},
{0.0287828,-0.3665248,0.0110357},
{0.0225189,-0.3758228,-0.0081400},
{0.0045308,-0.3888552,-0.0119694},
{-0.0021596,-0.3948919,0.0041875},
{0.0016272,-0.3873750,0.0210478},
{0.0221833,-0.3866673,0.0207757},
{0.0282479,-0.3904343,0.0049701},
{0.0214151,-0.3986758,-0.0127888},
{0.0097065,-0.4133601,-0.0236296},
{-0.0051218,-0.4082971,-0.0133052},

Biology Open • Supplementary information

{-0.0062254,-0.4217915,0.0027707},
{-0.0057341,-0.4121753,0.0196081},
{0.0101577,-0.4071164,0.0255248},
{0.0273152,-0.4095686,0.0174214},
{0.0261274,-0.4150832,-0.0001437},
{0.0248335,-0.4241673,-0.0175697},
{0.0172901,-0.4397898,-0.0292605},
{0.0041691,-0.4286618,-0.0369013},
{-0.0091992,-0.4186679,-0.0285062},
{-0.0158966,-0.4285149,-0.0122385},
{-0.0058838,-0.4463227,-0.0031944},
{-0.0170558,-0.4369562,0.0106388},
{-0.0148130,-0.4290045,0.0289509},
{0.0020608,-0.4242969,0.0338910},
{0.0203571,-0.4292084,0.0270379},
{0.0344535,-0.4303615,0.0153971},
{0.0266411,-0.4375325,-0.0017978},
{0.0288416,-0.4525399,-0.0158189},
{0.0169405,-0.4663005,-0.0268137},
{0.0024738,-0.4523669,-0.0383717},
{-0.0132117,-0.4383265,-0.0388524},
{-0.0268265,-0.4314875,-0.0275328},
{-0.0266138,-0.4474721,-0.0116746},
{-0.0169895,-0.4667910,-0.0171910},
{0.0001519,-0.4664908,-0.0114060},
{0.0029239,-0.4638277,0.0073000},
{-0.0139244,-0.4609286,0.0135562},
{-0.0239336,-0.4476667,0.0275499},
{-0.0043290,-0.4447593,0.0390959},
{0.0128682,-0.4490014,0.0322915},
{0.0313893,-0.4503491,0.0237367},
{0.0262279,-0.4555029,0.0069993},
{0.0162308,-0.4623715,-0.0063140},
{-0.0001405,-0.4730159,-0.0292780},
{-0.0140098,-0.4637429,-0.0371157},
{-0.0291138,-0.4536662,-0.0310478},
{0.0189841,-0.4674499,0.0203728},
{0.0015194,-0.4702596,0.0267233},
{-0.0141687,-0.4653992,0.0325528},
{-0.0008174,-0.0760948,0.0187565},
{-0.0097992,-0.0662421,0.0203399},
{0.0093408,-0.0388586,0.0608710},
{-0.0134778,-0.0157896,0.0561074},
{-0.0016521,-0.0647282,0.0564063},
{-0.0059784,-0.0726882,0.0221301},
{-0.0013390,-0.0933983,0.0202085},

{-0.0066387,0.0019297,-0.0167453},
{-0.0118351,-0.0508017,0.0405316},
{0.0031383,-0.0302818,0.0645382},
{-0.0096496,-0.0750887,0.0253121},
{-0.0010134,-0.0617145,0.0180743},
{-0.0125137,-0.0193808,0.0500112},
{0.0129304,-0.0342732,0.0592178},
{-0.0099397,-0.0340412,0.0390868},
{0.0135727,-0.0391744,0.0575599},
{-0.0008463,-0.0852545,0.0528694},
{-0.0140750,-0.0499104,0.0307740},
{-0.0132617,-0.0632893,0.0216489},
{-0.0154433,-0.0619012,0.0228999},
{-0.0023558,-0.0470422,0.0133629},
{0.0102869,-0.0659450,0.0235172},
{0.0206089,-0.0150832,0.0389454},
{-0.0029679,-0.0501126,0.0609597},
{0.0128851,-0.0031940,0.0169448},
{-0.0084190,-0.0553653,0.0186093},
{-0.0056754,-0.0920394,0.0436464},
{0.0053084,0.0168081,0.0129086},
{-0.0107599,0.0193385,-0.0059176},
{-0.0067202,-0.0764642,0.0467187},
{0.0074651,-0.0961732,0.0190844},
{0.0123111,-0.0843653,0.0238901},
{0.0073762,-0.0535569,0.0578095},
{0.0137503,-0.0143146,0.0074614},
{-0.0055031,-0.0266915,0.0045001},
{-0.0068490,-0.0011010,0.0197368},
{-0.0096224,-0.0637475,0.0480734},
{-0.0059021,-0.0296590,0.0645996},
{0.0080161,-0.0072016,0.0590007},
{0.0129660,-0.0572515,0.0291077},
{-0.0019126,-0.0205407,-0.0118412},
{-0.0131358,-0.0277014,0.0536401},
{0.0152638,0.0150712,-0.0068914},
{-0.0038670,0.0244229,0.0035903},
{0.0006016,-0.0963149,0.0512792},
{-0.0123785,-0.0447775,0.0485631},
{0.0189250,-0.0316636,0.0412366},
{0.0110396,-0.0486044,0.0234427},
{0.0169856,0.0117642,0.0070162},
{0.0195753,0.0023561,0.0065535},
{0.0204912,-0.0022424,-0.0033171},
{0.0131261,-0.0227721,0.0218692},
{0.0108955,-0.0391982,0.0208801},

Biology Open • Supplementary information

{0.0154633,-0.0166183,0.0312847},
{-0.0165361,-0.0554427,0.0257171},
{0.0028613,-0.0433408,0.0626237},
{0.0159978,-0.0510484,0.0372683},
{0.0138278,-0.0114486,0.0128712},
{0.0009765,0.0049648,-0.0188357},
{0.0082526,-0.0008626,-0.0186925},
{0.0098842,-0.0761817,0.0520743},
{0.0141165,-0.0687940,0.0494686},
{0.0165807,-0.0668397,0.0411337},
{0.0167704,-0.0773777,0.0347863},
{-0.0044661,-0.0844243,0.0238066},
{-0.0125472,0.0042152,0.0135357},
{0.0073010,-0.0012666,0.0534512},
{0.0181655,-0.0105526,0.0487221},
{0.0141627,-0.0266715,0.0582993},
{-0.0112634,-0.0403236,0.0245324},
{-0.0173614,-0.0084447,-0.0024734},
{0.0154472,-0.0932282,0.0265164},
{-0.0065938,-0.0064693,-0.0183986},
{0.0038879,0.0158780,-0.0143342},
{-0.0088278,-0.0372159,0.0154774},
{-0.0140512,0.0116126,-0.0100712},
{-0.0190710,0.0055846,-0.0016073},
{-0.0086197,-0.0788841,0.0382538},
{0.0110390,-0.0196027,-0.0009177},
{0.0118808,-0.0511222,0.0555058},
{0.0082476,-0.0207843,-0.0086948},
{0.0095581,-0.0156530,-0.0133261},
{0.0040928,-0.0668095,0.0183294},
{0.0085217,-0.0569921,0.0207675},
{0.0011647,-0.0854396,0.0194164},
{-0.0173370,-0.0630382,0.0292101},
{-0.0057636,-0.0015281,0.0504681},
{-0.0124975,-0.0345923,0.0575661},
{0.0171143,-0.0194922,0.0523662},
{0.0143186,-0.0864498,0.0467007},
{0.0179380,-0.0269641,0.0339437},
{0.0079802,-0.0357748,0.0643824},
{-0.0162532,-0.0036501,0.0096114},
{0.0137827,-0.0181297,0.0582691},
{0.0148027,-0.0688212,0.0315677},
{-0.0145396,-0.0563337,0.0218089},
{-0.0134436,-0.0374875,0.0509437},
{0.0132930,-0.0467787,0.0295991},
{-0.0074809,-0.0435252,0.0623340},

Biology Open • Supplementary information

{0.0063526,-0.0087001,-0.0182546},
{0.0039611,-0.0173353,-0.0146800},
{-0.0137873,-0.0121210,0.0498663},
{-0.0100008,-0.0503134,0.0561237},
{0.0167100,-0.0607578,0.0457914},
{-0.0161804,0.0125530,0.0015196},
{-0.0132363,0.0103097,0.0112899},
{-0.0018254,-0.0764503,0.0531147},
{0.0127325,-0.0104417,0.0330533},
{0.0133110,-0.0039395,0.0534303},
{0.0146387,-0.0064575,0.0418712},
{-0.0122665,-0.0151291,-0.0083414},
{0.0054977,-0.0246934,0.0022823},
{0.0003542,-0.0551537,0.0165338},
{0.0192266,0.0035210,-0.0073694},
{0.0164797,0.0018269,-0.0127782},
{0.0122905,-0.0086772,-0.0153257},
{-0.0126137,0.0015441,-0.0149943},
{-0.0140846,-0.0067244,-0.0128484},
{0.0170015,0.0148859,0.0031122},
{0.0101588,0.0214644,-0.0022277},
{0.0086714,0.0156150,-0.0128410},
{0.0077486,-0.0254208,0.0626589},
{-0.0006832,-0.0097105,0.0618004},
{-0.0001594,-0.0023532,0.0556958},
{0.0051741,-0.0154361,0.0632219},
{0.0080791,0.0082700,0.0160808},
{0.0118579,-0.0195820,0.0152489},
{0.0037901,-0.0284488,0.0642877},
{-0.0087812,-0.0194449,0.0237477},
{-0.0098419,-0.0196527,0.0118547},
{-0.0062380,-0.0112550,0.0265173},
{-0.0145216,-0.0570814,0.0346904},
{-0.0109413,-0.0690052,0.0401535},
{-0.0066783,-0.0327527,0.0091793},
{0.0199615,-0.0208280,0.0453152},
{0.0117954,-0.0095284,0.0271387},
{0.0041682,-0.0010653,0.0237763},
{-0.0034562,-0.0856111,0.0516973},
{-0.0023192,0.0247036,-0.0032037},
{-0.0011023,-0.0148876,0.0383351},
{-0.0105467,-0.0161794,0.0024137},
{0.0125021,-0.0324696,0.0251262},
{0.0099618,-0.0306237,0.0167887},
{-0.0106147,-0.0078367,0.0516036},
{0.0180725,-0.0373654,0.0470048},

{-0.0116474,-0.0603849,0.0407162},
{-0.0072082,-0.0912678,0.0369130},
{0.0041806,-0.0059214,0.0302316},
{-0.0028834,-0.0051974,-0.0195638},
{-0.0121699,-0.0392144,0.0571341},
{-0.0029042,0.0100457,-0.0165387},
{-0.0041701,0.0187292,-0.0122138},
{0.0179586,-0.0968812,0.0355867},
{0.0035635,-0.0835491,0.0186498},
{0.0158856,-0.0999585,0.0437476},
{0.0092524,0.0216721,0.0052725},
{0.0113334,0.0103314,0.0142824},
{0.0010602,0.0170224,-0.0140160},
{0.0155144,-0.0132005,-0.0086692},
{0.0182706,-0.0086343,-0.0074344},
{-0.0083949,-0.0275148,0.0298135},
{-0.0105213,-0.0391268,0.0315141},
{-0.0129322,-0.0698857,0.0252094},
{-0.0013661,0.0030150,0.0196893},
{0.0040076,0.0082959,0.0169864},
{-0.0024271,-0.0032322,0.0251536},
{0.0002477,-0.0349208,0.0638001},
{0.0066560,-0.0492274,0.0587160},
{-0.0112726,-0.0225085,0.0615070},
{-0.0112465,-0.0298495,0.0607528},
{0.0087076,0.0229681,0.0025957},
{0.0164711,0.0160430,0.0013773},
{-0.0068626,-0.0123772,0.0626229},
{0.0146631,-0.0602864,0.0336928},
{-0.0119502,-0.0539434,0.0471005},
{-0.0144535,-0.0572065,0.0215748},
{0.0105510,-0.0956220,0.0496059},
{-0.0094330,-0.0286301,0.0173568},
{-0.0097229,-0.0174344,0.0166887},
{-0.0042603,0.0181416,0.0123852},
{0.0008608,-0.0773008,0.0548407},
{0.0056665,-0.0712677,0.0550092},
{-0.0021731,-0.0709143,0.0545142},
{0.0159467,-0.0835650,0.0304491},
{-0.0119843,-0.0207475,0.0479854},
{0.0102653,-0.0281899,0.0168951},
{0.0049584,-0.0595354,0.0574294},
{0.0079891,-0.0397885,0.0610579},
{0.0091768,-0.0955668,0.0194551},
{-0.0065204,-0.0599661,0.0557600},
{0.0172438,-0.0485188,0.0430965},

Biology Open • Supplementary information

{-0.0114645,-0.0484989,0.0207280},
{0.0203797,-0.0233606,0.0366626},
{-0.0038750,-0.0690447,0.0198841},
{0.0160113,-0.0022547,0.0125073},
{0.0059362,0.0061174,0.0171327},
{0.0008808,-0.0367129,0.0636847},
{-0.0142550,0.0155273,-0.0049687},
{0.0102008,0.0177361,0.0099500},
{-0.0102621,0.0180963,0.0079838},
{-0.0178953,-0.0062104,-0.0050552},
{-0.0180019,0.0005744,-0.0072515},
{-0.0047130,-0.0197588,0.0649762},
{-0.0126451,-0.0387708,0.0426025},
{0.0053727,-0.0529024,0.0586883},
{0.0119580,-0.0469838,0.0564921},
{-0.0084061,-0.0770706,0.0248460},
{0.0121476,0.0079972,0.0147081},
{0.0175340,-0.0120650,-0.0010980},
{-0.0166603,-0.0090650,0.0048941},
{-0.0122857,-0.0634135,0.0377523},
{-0.0070328,-0.0189800,-0.0036334},
{-0.0124404,-0.0310379,0.0456643},
{0.0044747,-0.0843269,0.0533249},
{0.0153625,-0.0562102,0.0502720},
{-0.0016688,-0.0392424,0.0106897},
{0.0060084,-0.0360369,0.0127416},
{0.0180551,-0.0078452,0.0056031},
{0.0174335,-0.0916608,0.0404228},
{-0.0087836,-0.0213053,0.0243364},
{-0.0008452,0.0145810,0.0155751},
{0.0071209,-0.0768032,0.0205137},
{0.0084578,-0.0701841,0.0543850},
{0.0144465,-0.0145867,-0.0091297},
{0.0005347,-0.0102401,0.0622610},
{-0.0068770,-0.0165886,-0.0136885},
{0.0152873,-0.0792910,0.0454090},
{0.0073944,-0.0207119,-0.0099912},
{-0.0102085,-0.0724978,0.0224313},
{0.0128022,-0.0442134,0.0280303},
{0.0149223,-0.0048095,0.0520261},
{0.0045623,-0.0085878,0.0379233},
{-0.0029198,-0.0694287,0.0550525},
{-0.0123125,-0.0669188,0.0367648},
{-0.0138995,-0.0128401,0.0520607},
{-0.0075579,0.0137868,-0.0141141},
{-0.0165100,0.0065011,0.0078509},

Biology Open • Supplementary information

{0.0089889,-0.0765014,0.0213862},
{0.0023981,-0.0436731,0.0617446},
{-0.0129841,-0.0243444,0.0529595},
{0.0061922,-0.0068855,-0.0189302},
{0.0148125,-0.0317084,0.0544382},
{0.0115032,-0.0168011,0.0597357},
{0.0100189,0.0023200,-0.0174362},
{0.0131795,-0.0090810,0.0165585},
{-0.0124417,0.0123833,0.0112618},
{0.0099599,-0.0587527,0.0554512},
{-0.0052552,-0.0426661,0.0631276},
{0.0186932,-0.0322848,0.0482121},
{0.0152360,-0.0146955,-0.0067530},
{-0.0143460,-0.0557473,0.0351732},
{-0.0072603,-0.0818540,0.0436075},
{0.0030761,-0.0584143,0.0581415},
{-0.0042441,-0.0459634,0.0135223},
{0.0107068,0.0118619,-0.0140981},
{-0.0020605,-0.0239721,-0.0008146},
{-0.0088137,-0.0813086,0.0291654},
{-0.0099301,-0.0038839,-0.0169551},
{-0.0028036,0.0240326,-0.0045104},
{-0.0092178,-0.0240766,0.0128033},
{-0.0103271,-0.0714252,0.0222600},
{-0.0057177,-0.0828397,0.0239660},
{0.0009164,0.0131415,-0.0161286},
{0.0190809,-0.0288029,0.0474705},
{-0.0056686,-0.0642799,0.0192312},
{0.0169530,-0.0402343,0.0383643},
{0.0169285,-0.0341056,0.0341804},
{-0.0138086,-0.0132509,-0.0009473},
{0.0127625,0.0155259,0.0098094},
{-0.0128746,-0.0120850,0.0067374},
{-0.0102887,-0.0389788,0.0347788},
{0.0172724,-0.0971542,0.0412840},
{0.0170064,-0.0114398,0.0541931},
{0.0180510,-0.0172250,0.0502058},
{0.0013100,-0.0293071,0.0048870},
{0.0103815,-0.0258437,0.0611410},
{-0.0026838,-0.0105533,0.0305564},
{-0.0185482,-0.0046936,-0.0016918},
{0.0026533,-0.0232363,-0.0037075},
{-0.0093180,-0.0786353,0.0359631},
{-0.0122563,-0.0303745,0.0592847},
{0.0172144,0.0057349,-0.0104731},
{-0.0142115,-0.0687978,0.0293141},

Biology Open • Supplementary information

{-0.0146559,-0.0046392,0.0124477},
{0.0115406,-0.0088781,0.0263225},
{-0.0129224,-0.0473253,0.0311781},
{0.0144782,-0.0790358,0.0280098},
{0.0007403,0.0037205,-0.0190816},
{0.0162429,-0.0855618,0.0305619},
{0.0066794,0.0070442,0.0164161},
{-0.0137445,0.0024944,0.0126043},
{0.0056290,-0.0473713,0.0161583},
{-0.0062301,-0.0209969,0.0331165},
{0.0197730,-0.0297469,0.0418314},
{-0.0124178,-0.0690695,0.0223828},
{0.0200669,0.0056425,-0.0029645},
{-0.0133516,-0.0692525,0.0272727},
{0.0108397,-0.0246788,0.0108730},
{0.0189941,-0.0128990,0.0446259},
{0.0083357,0.0021462,-0.0183580},
{0.0141979,-0.0091605,0.0133078},
{-0.0109148,-0.0700977,0.0395201},
{0.0004459,0.0227104,0.0090609},
{0.0027355,0.0036432,-0.0193944},
{0.0363299,-0.4445459,0.0207249},
{0.0049235,0.0225034,-0.0076788},
{-0.0131553,-0.0496189,0.0368063},
{-0.0305724,-0.4400438,-0.0265564},
{0.0022567,0.0254586,-0.0000315},
{0.0139966,-0.0270486,0.0262459},
{-0.0137482,-0.0012128,-0.0143426},
{0.0161220,-0.0341557,0.0523172},
{-0.0142716,0.0148245,0.0046818},
{0.0168079,-0.0441864,0.0395368},
{0.0150004,-0.0492943,0.0339125},
{-0.0129260,-0.0388823,0.0451951},
{0.0168776,-0.0624354,0.0434970},
{-0.0162911,-0.0542556,0.0271446},
{0.0029765,-0.0619856,0.0574014},
{0.0010592,-0.0578242,0.0171427},
{0.0208532,-0.0235613,0.0434832},
{0.0076991,-0.0860993,0.0520324},
{-0.0057926,-0.0934750,0.0275804},
{-0.0184811,-0.0024259,0.0030148},
{0.0140084,-0.0151028,0.0194893},
{0.0134732,-0.0750454,0.0271181},
{-0.0090447,-0.0278212,0.0271597},
{-0.0189749,-0.4511705,0.0352395},
{-0.0084198,-0.0628317,0.0514965},

{-0.0111412,-0.4450708,-0.0101447},
{0.0103837,-0.0487127,0.0221042},
{0.0105533,0.0163501,-0.0110462},
{-0.0236922,-0.4413982,0.0285139},
{0.0152105,-0.0598440,0.0345623},
{0.0002901,-0.0558033,0.0590675},
{-0.0106771,0.0203744,0.0028067},
{-0.0023985,-0.0188035,0.0648384},
{-0.0070908,-0.0082984,0.0237958},
{0.0188364,-0.0116045,0.0416510},
{0.0144252,-0.0275798,0.0564280},
{-0.0137357,-0.0122822,0.0513385},
{0.0192522,0.0092744,-0.0027691},
{-0.0097379,-0.0384203,0.0176847},
{-0.0003272,-0.0159039,-0.0162156},
{-0.0104668,-0.0678246,0.0435663},
{-0.0087464,0.0192836,0.0079101},
{0.0094814,0.0215558,-0.0029601},
{-0.0130107,-0.0304474,0.0562217},
{-0.0058088,-0.4419482,0.0399441},
{-0.0130445,-0.4479569,-0.0374383},
{-0.0129721,-0.4478968,-0.0366645},
{-0.0128998,-0.4478367,-0.0358906},
{-0.0128274,-0.4477767,-0.0351168},
{-0.0127550,-0.4477166,-0.0343430},
{-0.0126827,-0.4476565,-0.0335692},
{-0.0126103,-0.4475964,-0.0327953},
{-0.0125380,-0.4475363,-0.0320215},
{-0.0124656,-0.4474762,-0.0312477},
{-0.0123933,-0.4474161,-0.0304739},
{-0.0123209,-0.4473560,-0.0297000},
{-0.0122485,-0.4472960,-0.0289262},
{-0.0121762,-0.4472359,-0.0281524},
{-0.0121038,-0.4471758,-0.0273786},
{-0.0120315,-0.4471157,-0.0266048},
{-0.0119591,-0.4470556,-0.0258309},
{-0.0118868,-0.4469955,-0.0250571},
{-0.0118144,-0.4469354,-0.0242833},
{-0.0117420,-0.4468753,-0.0235095},
{-0.0116697,-0.4468153,-0.0227356},
{-0.0115973,-0.4467552,-0.0219618},
{-0.0115250,-0.4466951,-0.0211880},
{-0.0114526,-0.4466350,-0.0204142},
{-0.0113803,-0.4465749,-0.0196403},
{-0.0113079,-0.4465148,-0.0188665},
{-0.0112356,-0.4464547,-0.0180927},

{-0.0111632,-0.4463947,-0.0173189},
{-0.0110908,-0.4463346,-0.0165450},
{-0.0110185,-0.4462745,-0.0157712},
{-0.0109461,-0.4462144,-0.0149974},
{-0.0108738,-0.4461543,-0.0142236},
{-0.0108014,-0.4460942,-0.0134497},
{-0.0107291,-0.4460341,-0.0126759},
{-0.0106567,-0.4459740,-0.0119021},
{-0.0105843,-0.4459140,-0.0111283},
{-0.0105120,-0.4458539,-0.0103545},
{-0.0104396,-0.4457938,-0.0095806},
{-0.0103673,-0.4457337,-0.0088068},
{-0.0102949,-0.4456736,-0.0080330},
{-0.0102226,-0.4456135,-0.0072592},
{-0.0101502,-0.4455534,-0.0064853},
{-0.0100778,-0.4454933,-0.0057115},
{-0.0100055,-0.4454333,-0.0049377},
{-0.0099331,-0.4453732,-0.0041639},
{-0.0098608,-0.4453131,-0.0033900},
{-0.0097884,-0.4452530,-0.0026162},
{-0.0097161,-0.4451929,-0.0018424},
{-0.0096437,-0.4451328,-0.0010686},
{-0.0095713,-0.4450727,-0.0002947},
{-0.0094990,-0.4450126,0.0004791},
{-0.0094266,-0.4449526,0.0012529},
{-0.0093543,-0.4448925,0.0020267},
{-0.0092819,-0.4448324,0.0028005},
{-0.0092096,-0.4447723,0.0035744},
{-0.0091372,-0.4447122,0.0043482},
{-0.0090649,-0.4446521,0.0051220},
{-0.0089925,-0.4445920,0.0058958},
{-0.0089201,-0.4445320,0.0066697},
{-0.0088478,-0.4444719,0.0074435},
{-0.0087754,-0.4444118,0.0082173},
{-0.0087031,-0.4443517,0.0089911},
{-0.0086307,-0.4442916,0.0097650},
{-0.0085584,-0.4442315,0.0105388},
{-0.0084860,-0.4441714,0.0113126},
{-0.0084136,-0.4441113,0.0120864},
{-0.0083413,-0.4440513,0.0128603},
{-0.0082689,-0.4439912,0.0136341},
{-0.0081966,-0.4439311,0.0144079},
{-0.0081242,-0.4438710,0.0151817},
{-0.0080519,-0.4438109,0.0159555},
{-0.0079795,-0.4437508,0.0167294},
{-0.0079071,-0.4436907,0.0175032},

```
                              {-0.0078348,-0.4436306,0.0182770},
                              {-0.0077624,-0.4435706,0.0190508},
                              {-0.0076901,-0.4435105,0.0198247},
                              {-0.0076177,-0.4434504,0.0205985},
                              {-0.0075454,-0.4433903,0.0213723},
                              {-0.0074730,-0.4433302,0.0221461},
                              {-0.0074006,-0.4432701,0.0229200},
                              {-0.0073283,-0.4432100,0.0236938},
                              {-0.0072559,-0.4431500,0.0244676},
                              {-0.0071836,-0.4430899,0.0252414},
                              {-0.0071112,-0.4430298,0.0260153},
                              {-0.0070389,-0.4429697,0.0267891},
                              {-0.0069665,-0.4429096,0.0275629},
                              {-0.0068942,-0.4428495,0.0283367},
                              {-0.0068218,-0.4427894,0.0291105},
                              {-0.0067494,-0.4427293,0.0298844},
                              {-0.0066771,-0.4426693,0.0306582},
                              {-0.0066047,-0.4426092,0.0314320},
                              {-0.0065324,-0.4425491,0.0322058},
                              {-0.0064600,-0.4424890,0.0329797},
                              {-0.0063877,-0.4424289,0.0337535},
                              {-0.0063153,-0.4423688,0.0345273},
                              {-0.0062429,-0.4423087,0.0353011},
                              {-0.0061706,-0.4422486,0.0360750},
                              {-0.0060982,-0.4421886,0.0368488},
                              {-0.0060259,-0.4421285,0.0376226},
                              {-0.0059535,-0.4420684,0.0383964},
                              {-0.0058812,-0.4420083,0.0391703}
                              }* .AMirroring);
                    BoundingBoxOnOff = Off;
          };
          AnyFunTransform3DIdentity ScaleFunction = {
                    PreTransforms = {&.RBFTransform};
};
};

};
};
```

Biology Open • Supplementary information

ScalingFunctionTLEMLucyPelvis_2014016

```
AnyFolder ScalingFunctionTLEMLucyPelvis = {
AnyFolder Pelvis = {
	AnyFunTransform3DRBF RBFTransform = {
		RBFDef.Type = RBF_ThinPlate;
		PolynomDegree = 1;
		Points0 = {
			{0.0000000,0.0000000,0.1177000},
			{-0.0000000,-0.0832729,0.0191000},
			{-0.0508179,-0.0694062,0.0815920},
			{0.0000000,0.0000000,-0.1177000},
			{-0.0000000,-0.0832729,-0.0191000},
			{-0.0508179,-0.0694062,-0.0815920},
			{0.0000000,0.0000000,0.0000000},
			{-0.1164020,-0.0039449,0.0456760},
			{-0.1164020,-0.0039449,-0.0456760},
			{-0.1092909,-0.0992949,0.0515590},
			{-0.1092909,-0.0992949,-0.0515590},
			{-0.0733749,0.0757445,0.0915590},
			{-0.0733749,0.0757445,-0.0915590},
			{-0.1241738,0.0316243,0.0458050},
			{-0.1241738,0.0316243,-0.0458050},
			{-0.1108008,0.0563490,0.0555690},
			{-0.1108008,0.0563490,-0.0555690},
			{-0.0545356,0.0481653,0.1291690},
			{-0.0545356,0.0481653,-0.1291690},
			{-0.0360236,0.0456352,0.1316250},
			{-0.0360236,0.0456352,-0.1316250},
			{-0.0804664,-0.0228481,0.0672140},
			{-0.0804664,-0.0228481,-0.0672140},
			{-0.1004070,-0.0623213,0.0494990},
			{-0.1004070,-0.0623213,-0.0494990},
			{-0.0174901,-0.0357786,0.0977400},
			{-0.0174901,-0.0357786,-0.0977400},
			{-0.0665950,-0.1308418,0.0278190},
			{-0.0665950,-0.1308418,-0.0278190},
			{-0.0962739,-0.1257274,0.0551670},
			{-0.0962739,-0.1257274,-0.0551670},
			{-0.0393340,-0.1196098,0.0159820},
			{-0.0393340,-0.1196098,-0.0159820},
			{-0.0585979,-0.0148191,0.0651020},
			{-0.0585979,-0.0148191,-0.0651020},
			{-0.0238420,-0.0800479,0.0351680},
			{-0.0238420,-0.0800479,-0.0351680},
			{-0.0234553,-0.0576924,0.0715330},
```

Biology Open • Supplementary information

{-0.0234553,-0.0576924,-0.0715330},
{-0.0472319,-0.0833276,0.0513790},
{-0.0472319,-0.0833276,-0.0513790},
{-0.0327175,-0.0506481,0.0520240},
{-0.0327175,-0.0506481,-0.0520240},
{-0.0746024,-0.0627219,0.0928110},
{-0.0746024,-0.0627219,-0.0928110},
{-0.0119676,-0.0928080,0.0076900},
{-0.0119676,-0.0928080,-0.0076900},
{-0.0263299,-0.0760728,0.0667080},
{-0.0263299,-0.0760728,-0.0667080},
{-0.0498842,0.0119450,0.1070630},
{-0.0498842,0.0119450,-0.1070630},
{-0.0553874,0.0627474,0.1172390},
{-0.0553874,0.0627474,-0.1172390},
{-0.0786774,0.0073556,0.0741800},
{-0.0786774,0.0073556,-0.0741800},
{-0.0742762,0.0474272,0.0542330},
{-0.0742762,0.0474272,-0.0542330},
{-0.0634121,0.0117185,0.0618430},
{-0.0634121,0.0117185,-0.0618430},
{-0.0813084,0.0007821,0.0508130},
{-0.0813084,0.0007821,-0.0508130},
{-0.0496423,-0.0444458,0.0983920},
{-0.0496423,-0.0444458,-0.0983920},
{-0.0480005,-0.0338999,0.0625080},
{-0.0480005,-0.0338999,-0.0625080},
{-0.0428000,-0.0273188,0.0973490},
{-0.0428000,-0.0273188,-0.0973490},
{-0.0548383,-0.0518766,0.0624050},
{-0.0548383,-0.0518766,-0.0624050},
{-0.0696448,-0.1052827,0.0436200},
{-0.0696448,-0.1052827,-0.0436200},
{-0.0739608,-0.0803177,0.0785950},
{-0.0739608,-0.0803177,-0.0785950},
{-0.0586066,-0.0884764,0.0653190},
{-0.0586066,-0.0884764,-0.0653190},
{-0.1017003,0.0085215,0.0624150},
{-0.1017003,0.0085215,-0.0624150},
{-0.0126252,0.0252852,0.1292530},
{-0.0126252,0.0252852,-0.1292530},
{-0.0030963,0.0150156,0.1262640},
{-0.0030963,0.0150156,-0.1262640},
{-0.0621797,0.0048743,0.0605920},
{-0.0621797,0.0048743,-0.0605920},
{-0.0870082,-0.0684280,0.0489900},

{-0.0870082,-0.0684280,-0.0489900},
{-0.0916211,-0.0478344,0.0575290},
{-0.0916211,-0.0478344,-0.0575290},
{-0.0428573,-0.0467026,0.0985250},
{-0.0428573,-0.0467026,-0.0985250},
{-0.0483869,-0.0455474,0.0848580},
{-0.0483869,-0.0455474,-0.0848580},
{-0.0203929,-0.0681811,0.0426070},
{-0.0203929,-0.0681811,-0.0426070},
{-0.0271973,-0.0807786,0.0491650},
{-0.0271973,-0.0807786,-0.0491650},
{-0.0325935,-0.1026813,0.0148280},
{-0.0325935,-0.1026813,-0.0148280},
{-0.0273900,-0.1059173,0.0048750},
{-0.0273900,-0.1059173,-0.0048750},
{-0.0015971,-0.0778770,0.0065530},
{-0.0015971,-0.0778770,-0.0065530},
{-0.1034983,-0.0140026,0.0521800},
{-0.1034983,-0.0140026,-0.0521800},
{-0.1141160,0.0077734,0.0497600},
{-0.1141160,0.0077734,-0.0497600},
{-0.1123797,0.0244808,0.0350160},
{-0.1123797,0.0244808,-0.0350160},
{-0.0984470,-0.0648210,0.0457100},
{-0.0984470,-0.0648210,-0.0457100},
{-0.0814332,-0.0704479,0.0806410},
{-0.0814332,-0.0704479,-0.0806410},
{-0.0540218,-0.0805070,0.0547320},
{-0.0540218,-0.0805070,-0.0547320},
{-0.0674356,-0.0763171,0.0651080},
{-0.0674356,-0.0763171,-0.0651080},
{-0.0602364,-0.0751827,0.0490600},
{-0.0602364,-0.0751827,-0.0490600},
{-0.0604272,-0.0801096,0.0617610},
{-0.0604272,-0.0801096,-0.0617610},
{-0.0655251,0.0632155,0.1093750},
{-0.0655251,0.0632155,-0.1093750},
{-0.0665046,0.0014289,0.0572170},
{-0.0665046,0.0014289,-0.0572170},
{-0.1053786,0.0347803,0.0071580},
{-0.1053786,0.0347803,-0.0071580},
{-0.1321649,-0.0069725,0.0058340},
{-0.1321649,-0.0069725,-0.0058340},
{-0.1232735,-0.0434903,0.0301110},
{-0.1232735,-0.0434903,-0.0301110},
{-0.0497024,0.0261862,0.0084640},

Biology Open • Supplementary information

```
        {-0.0497024,0.0261862,-0.0084640},
        {-0.0887827,0.0446048,0.0327030},
        {-0.0887827,0.0446048,-0.0327030},
        {-0.1153998,-0.0126010,0.0290730},
        {-0.1153998,-0.0126010,-0.0290730},
        {-0.0899939,0.0093882,0.0208440},
        {-0.0899939,0.0093882,-0.0208440},
        {-0.1312654,-0.0574898,0.0094690},
        {-0.1312654,-0.0574898,-0.0094690},
        {-0.0996029,0.0042806,0.0105620},
        {-0.0996029,0.0042806,-0.0105620},
        {-0.1226177,-0.0324069,0.0331630},
        {-0.1226177,-0.0324069,-0.0331630},
        {-0.1097808,-0.0051619,0.0102520},
        {-0.1097808,-0.0051619,-0.0102520}
        };
Points1 = {
        {0.0000000,0.0000000,0.1348305},
        {-0.0000008,-0.0989722,0.0196331},
        {-0.0338099,-0.0768545,0.0938272},
        {0.0000000,0.0000000,-0.1348305},
        {-0.0000008,-0.0989722,-0.0196331},
        {-0.0338099,-0.0768545,-0.0938272},
        {0.0000000,0.0000000,0.0000000},
        {-0.0923166,-0.0323207,0.0456717},
        {-0.0923166,-0.0323207,-0.0456717},
        {-0.0875017,-0.1086963,0.0666837},
        {-0.0875017,-0.1086963,-0.0666837},
        {-0.0600730,0.0454564,0.0983364},
        {-0.0600730,0.0454564,-0.0983364},
        {-0.0974593,-0.0080973,0.0404926},
        {-0.0974593,-0.0080973,-0.0404926},
        {-0.0884698,0.0170264,0.0537647},
        {-0.0884698,0.0170264,-0.0537647},
        {-0.0370864,0.0348901,0.1396146},
        {-0.0370864,0.0348901,-0.1396146},
        {-0.0241686,0.0352424,0.1441978},
        {-0.0241686,0.0352424,-0.1441978},
        {-0.0653355,-0.0438544,0.0740298},
        {-0.0653355,-0.0438544,-0.0740298},
        {-0.0816306,-0.0801670,0.0684811},
        {-0.0816306,-0.0801670,-0.0684811},
        {-0.0121648,-0.0429718,0.1012260},
        {-0.0121648,-0.0429718,-0.1012260},
        {-0.0565386,-0.1368855,0.0368019},
        {-0.0565386,-0.1368855,-0.0368019},
```

Biology Open • Supplementary information

{-0.0764847,-0.1268949,0.0701284},
{-0.0764847,-0.1268949,-0.0701284},
{-0.0318092,-0.1311805,0.0205321},
{-0.0318092,-0.1311805,-0.0205321},
{-0.0508374,-0.0312545,0.0715307},
{-0.0508374,-0.0312545,-0.0715307},
{-0.0161632,-0.0902422,0.0370855},
{-0.0161632,-0.0902422,-0.0370855},
{-0.0170093,-0.0641454,0.0795090},
{-0.0170093,-0.0641454,-0.0795090},
{-0.0315793,-0.0873524,0.0610048},
{-0.0315793,-0.0873524,-0.0610048},
{-0.0264358,-0.0643938,0.0618754},
{-0.0264358,-0.0643938,-0.0618754},
{-0.0536040,-0.0720865,0.0986850},
{-0.0536040,-0.0720865,-0.0986850},
{-0.0077330,-0.1097997,0.0084295},
{-0.0077330,-0.1097997,-0.0084295},
{-0.0180743,-0.0772926,0.0724365},
{-0.0180743,-0.0772926,-0.0724365},
{-0.0391526,-0.0010856,0.1148335},
{-0.0391526,-0.0010856,-0.1148335},
{-0.0417890,0.0433893,0.1273774},
{-0.0417890,0.0433893,-0.1273774},
{-0.0663807,-0.0155836,0.0750250},
{-0.0663807,-0.0155836,-0.0750250},
{-0.0635714,0.0153080,0.0540659},
{-0.0635714,0.0153080,-0.0540659},
{-0.0577213,-0.0111884,0.0608327},
{-0.0577213,-0.0111884,-0.0608327},
{-0.0694853,-0.0252248,0.0505076},
{-0.0694853,-0.0252248,-0.0505076},
{-0.0336530,-0.0552225,0.1020866},
{-0.0336530,-0.0552225,-0.1020866},
{-0.0387753,-0.0475314,0.0729551},
{-0.0387753,-0.0475314,-0.0729551},
{-0.0313073,-0.0381702,0.1024860},
{-0.0313073,-0.0381702,-0.1024860},
{-0.0400598,-0.0639014,0.0747536},
{-0.0400598,-0.0639014,-0.0747536},
{-0.0541598,-0.1121331,0.0573075},
{-0.0541598,-0.1121331,-0.0573075},
{-0.0527516,-0.0881353,0.0897645},
{-0.0527516,-0.0881353,-0.0897645},
{-0.0402213,-0.0958827,0.0800264},
{-0.0402213,-0.0958827,-0.0800264},

{-0.0822521,-0.0188612,0.0622108},
{-0.0822521,-0.0188612,-0.0622108},
{-0.0080479,0.0207710,0.1445652},
{-0.0080479,0.0207710,-0.1445652},
{-0.0014461,0.0130964,0.1427867},
{-0.0014461,0.0130964,-0.1427867},
{-0.0574376,-0.0167812,0.0593421},
{-0.0574376,-0.0167812,-0.0593421},
{-0.0686662,-0.0830874,0.0680254},
{-0.0686662,-0.0830874,-0.0680254},
{-0.0728647,-0.0664705,0.0711280},
{-0.0728647,-0.0664705,-0.0711280},
{-0.0277705,-0.0572769,0.1015934},
{-0.0277705,-0.0572769,-0.1015934},
{-0.0340011,-0.0564543,0.0913015},
{-0.0340011,-0.0564543,-0.0913015},
{-0.0143485,-0.0799117,0.0449325},
{-0.0143485,-0.0799117,-0.0449325},
{-0.0182743,-0.0871999,0.0532384},
{-0.0182743,-0.0871999,-0.0532384},
{-0.0228693,-0.1159994,0.0187060},
{-0.0228693,-0.1159994,-0.0187060},
{-0.0169761,-0.1196543,0.0073074},
{-0.0169761,-0.1196543,-0.0073074},
{-0.0019652,-0.0975719,0.0067811},
{-0.0019652,-0.0975719,-0.0067811},
{-0.0825630,-0.0386727,0.0567592},
{-0.0825630,-0.0386727,-0.0567592},
{-0.0908911,-0.0213208,0.0481189},
{-0.0908911,-0.0213208,-0.0481189},
{-0.0892336,-0.0128087,0.0309377},
{-0.0892336,-0.0128087,-0.0309377},
{-0.0804918,-0.0822341,0.0670357},
{-0.0804918,-0.0822341,-0.0670357},
{-0.0597523,-0.0805381,0.0913610},
{-0.0597523,-0.0805381,-0.0913610},
{-0.0357570,-0.0840130,0.0662457},
{-0.0357570,-0.0840130,-0.0662457},
{-0.0475214,-0.0859111,0.0799017},
{-0.0475214,-0.0859111,-0.0799017},
{-0.0425019,-0.0836523,0.0624414},
{-0.0425019,-0.0836523,-0.0624414},
{-0.0411725,-0.0888706,0.0770761},
{-0.0411725,-0.0888706,-0.0770761},
{-0.0517570,0.0397003,0.1183278},
{-0.0517570,0.0397003,-0.1183278},

```
                {-0.0610764,-0.0209986,0.0551229},
                {-0.0610764,-0.0209986,-0.0551229},
                {-0.0844740,-0.0066388,0.0062173},
                {-0.0844740,-0.0066388,-0.0062173},
                {-0.1032418,-0.0419115,0.0049474},
                {-0.1032418,-0.0419115,-0.0049474},
                {-0.1022212,-0.0725601,0.0232253},
                {-0.1022212,-0.0725601,-0.0232253},
                {-0.0460810,-0.0075855,0.0084505},
                {-0.0460810,-0.0075855,-0.0084505},
                {-0.0739106,0.0068109,0.0306199},
                {-0.0739106,0.0068109,-0.0306199},
                {-0.0915641,-0.0436272,0.0279940},
                {-0.0915641,-0.0436272,-0.0279940},
                {-0.0738279,-0.0252290,0.0198160},
                {-0.0738279,-0.0252290,-0.0198160},
                {-0.1089604,-0.0849010,0.0072589},
                {-0.1089604,-0.0849010,-0.0072589},
                {-0.0786164,-0.0323963,0.0099433},
                {-0.0786164,-0.0323963,-0.0099433},
                {-0.0998468,-0.0617430,0.0295684},
                {-0.0998468,-0.0617430,-0.0295684},
                {-0.0849043,-0.0411061,0.0095273},
                {-0.0849043,-0.0411061,-0.0095273}
                };
            BoundingBoxOnOff = Off;
        };
        AnyFunTransform3DIdentity ScaleFunction = {
            PreTransforms = {&.RBFTransform};
};
};
AnyFolder Sacrum = {
        AnyFunTransform3DRBF RBFTransform = {
            RBFDef.Type = RBF_ThinPlate;
            PolynomDegree = 1;
            Points0 = {
                {0.0000000,0.0000000,0.1177000},
                {-0.0000000,-0.0832729,0.0191000},
                {-0.0508179,-0.0694062,0.0815920},
                {0.0000000,0.0000000,-0.1177000},
                {-0.0000000,-0.0832729,-0.0191000},
                {-0.0508179,-0.0694062,-0.0815920},
                {0.0000000,0.0000000,0.0000000},
                {-0.1164020,-0.0039449,0.0456760},
                {-0.1164020,-0.0039449,-0.0456760},
                {-0.1092909,-0.0992949,0.0515590},
```

{-0.1092909,-0.0992949,-0.0515590},
{-0.0733749,0.0757445,0.0915590},
{-0.0733749,0.0757445,-0.0915590},
{-0.1241738,0.0316243,0.0458050},
{-0.1241738,0.0316243,-0.0458050},
{-0.1108008,0.0563490,0.0555690},
{-0.1108008,0.0563490,-0.0555690},
{-0.0545356,0.0481653,0.1291690},
{-0.0545356,0.0481653,-0.1291690},
{-0.0360236,0.0456352,0.1316250},
{-0.0360236,0.0456352,-0.1316250},
{-0.0804664,-0.0228481,0.0672140},
{-0.0804664,-0.0228481,-0.0672140},
{-0.1004070,-0.0623213,0.0494990},
{-0.1004070,-0.0623213,-0.0494990},
{-0.0174901,-0.0357786,0.0977400},
{-0.0174901,-0.0357786,-0.0977400},
{-0.0665950,-0.1308418,0.0278190},
{-0.0665950,-0.1308418,-0.0278190},
{-0.0962739,-0.1257274,0.0551670},
{-0.0962739,-0.1257274,-0.0551670},
{-0.0393340,-0.1196098,0.0159820},
{-0.0393340,-0.1196098,-0.0159820},
{-0.0585979,-0.0148191,0.0651020},
{-0.0585979,-0.0148191,-0.0651020},
{-0.0238420,-0.0800479,0.0351680},
{-0.0238420,-0.0800479,-0.0351680},
{-0.0234553,-0.0576924,0.0715330},
{-0.0234553,-0.0576924,-0.0715330},
{-0.0472319,-0.0833276,0.0513790},
{-0.0472319,-0.0833276,-0.0513790},
{-0.0327175,-0.0506481,0.0520240},
{-0.0327175,-0.0506481,-0.0520240},
{-0.0746024,-0.0627219,0.0928110},
{-0.0746024,-0.0627219,-0.0928110},
{-0.0119676,-0.0928080,0.0076900},
{-0.0119676,-0.0928080,-0.0076900},
{-0.0263299,-0.0760728,0.0667080},
{-0.0263299,-0.0760728,-0.0667080},
{-0.0498842,0.0119450,0.1070630},
{-0.0498842,0.0119450,-0.1070630},
{-0.0553874,0.0627474,0.1172390},
{-0.0553874,0.0627474,-0.1172390},
{-0.0786774,0.0073556,0.0741800},
{-0.0786774,0.0073556,-0.0741800},
{-0.0742762,0.0474272,0.0542330},

Biology Open • Supplementary information

{-0.0742762,0.0474272,-0.0542330},
{-0.0634121,0.0117185,0.0618430},
{-0.0634121,0.0117185,-0.0618430},
{-0.0813084,0.0007821,0.0508130},
{-0.0813084,0.0007821,-0.0508130},
{-0.0496423,-0.0444458,0.0983920},
{-0.0496423,-0.0444458,-0.0983920},
{-0.0480005,-0.0338999,0.0625080},
{-0.0480005,-0.0338999,-0.0625080},
{-0.0428000,-0.0273188,0.0973490},
{-0.0428000,-0.0273188,-0.0973490},
{-0.0548383,-0.0518766,0.0624050},
{-0.0548383,-0.0518766,-0.0624050},
{-0.0696448,-0.1052827,0.0436200},
{-0.0696448,-0.1052827,-0.0436200},
{-0.0739608,-0.0803177,0.0785950},
{-0.0739608,-0.0803177,-0.0785950},
{-0.0586066,-0.0884764,0.0653190},
{-0.0586066,-0.0884764,-0.0653190},
{-0.1017003,0.0085215,0.0624150},
{-0.1017003,0.0085215,-0.0624150},
{-0.0126252,0.0252852,0.1292530},
{-0.0126252,0.0252852,-0.1292530},
{-0.0030963,0.0150156,0.1262640},
{-0.0030963,0.0150156,-0.1262640},
{-0.0621797,0.0048743,0.0605920},
{-0.0621797,0.0048743,-0.0605920},
{-0.0870082,-0.0684280,0.0489900},
{-0.0870082,-0.0684280,-0.0489900},
{-0.0916211,-0.0478344,0.0575290},
{-0.0916211,-0.0478344,-0.0575290},
{-0.0428573,-0.0467026,0.0985250},
{-0.0428573,-0.0467026,-0.0985250},
{-0.0483869,-0.0455474,0.0848580},
{-0.0483869,-0.0455474,-0.0848580},
{-0.0203929,-0.0681811,0.0426070},
{-0.0203929,-0.0681811,-0.0426070},
{-0.0271973,-0.0807786,0.0491650},
{-0.0271973,-0.0807786,-0.0491650},
{-0.0325935,-0.1026813,0.0148280},
{-0.0325935,-0.1026813,-0.0148280},
{-0.0273900,-0.1059173,0.0048750},
{-0.0273900,-0.1059173,-0.0048750},
{-0.0015971,-0.0778770,0.0065530},
{-0.0015971,-0.0778770,-0.0065530},
{-0.1034983,-0.0140026,0.0521800},

```
        {-0.1034983,-0.0140026,-0.0521800},
        {-0.1141160,0.0077734,0.0497600},
        {-0.1141160,0.0077734,-0.0497600},
        {-0.1123797,0.0244808,0.0350160},
        {-0.1123797,0.0244808,-0.0350160},
        {-0.0984470,-0.0648210,0.0457100},
        {-0.0984470,-0.0648210,-0.0457100},
        {-0.0814332,-0.0704479,0.0806410},
        {-0.0814332,-0.0704479,-0.0806410},
        {-0.0540218,-0.0805070,0.0547320},
        {-0.0540218,-0.0805070,-0.0547320},
        {-0.0674356,-0.0763171,0.0651080},
        {-0.0674356,-0.0763171,-0.0651080},
        {-0.0602364,-0.0751827,0.0490600},
        {-0.0602364,-0.0751827,-0.0490600},
        {-0.0604272,-0.0801096,0.0617610},
        {-0.0604272,-0.0801096,-0.0617610},
        {-0.0655251,0.0632155,0.1093750},
        {-0.0655251,0.0632155,-0.1093750},
        {-0.0665046,0.0014289,0.0572170},
        {-0.0665046,0.0014289,-0.0572170},
        {-0.1053786,0.0347803,0.0071580},
        {-0.1053786,0.0347803,-0.0071580},
        {-0.1321649,-0.0069725,0.0058340},
        {-0.1321649,-0.0069725,-0.0058340},
        {-0.1232735,-0.0434903,0.0301110},
        {-0.1232735,-0.0434903,-0.0301110},
        {-0.0497024,0.0261862,0.0084640},
        {-0.0497024,0.0261862,-0.0084640},
        {-0.0887827,0.0446048,0.0327030},
        {-0.0887827,0.0446048,-0.0327030},
        {-0.1153998,-0.0126010,0.0290730},
        {-0.1153998,-0.0126010,-0.0290730},
        {-0.0899939,0.0093882,0.0208440},
        {-0.0899939,0.0093882,-0.0208440},
        {-0.1312654,-0.0574898,0.0094690},
        {-0.1312654,-0.0574898,-0.0094690},
        {-0.0996029,0.0042806,0.0105620},
        {-0.0996029,0.0042806,-0.0105620},
        {-0.1226177,-0.0324069,0.0331630},
        {-0.1226177,-0.0324069,-0.0331630},
        {-0.1097808,-0.0051619,0.0102520},
        {-0.1097808,-0.0051619,-0.0102520}
        };
Points1 = {
        {0.0000000,0.0000000,0.1348305},
```

Biology Open • Supplementary information

{-0.0000008,-0.0989722,0.0196331},
{-0.0338099,-0.0768545,0.0938272},
{0.0000000,0.0000000,-0.1348305},
{-0.0000008,-0.0989722,-0.0196331},
{-0.0338099,-0.0768545,-0.0938272},
{0.0000000,0.0000000,0.0000000},
{-0.0923166,-0.0323207,0.0456717},
{-0.0923166,-0.0323207,-0.0456717},
{-0.0875017,-0.1086963,0.0666837},
{-0.0875017,-0.1086963,-0.0666837},
{-0.0600730,0.0454564,0.0983364},
{-0.0600730,0.0454564,-0.0983364},
{-0.0974593,-0.0080973,0.0404926},
{-0.0974593,-0.0080973,-0.0404926},
{-0.0884698,0.0170264,0.0537647},
{-0.0884698,0.0170264,-0.0537647},
{-0.0370864,0.0348901,0.1396146},
{-0.0370864,0.0348901,-0.1396146},
{-0.0241686,0.0352424,0.1441978},
{-0.0241686,0.0352424,-0.1441978},
{-0.0653355,-0.0438544,0.0740298},
{-0.0653355,-0.0438544,-0.0740298},
{-0.0816306,-0.0801670,0.0684811},
{-0.0816306,-0.0801670,-0.0684811},
{-0.0121648,-0.0429718,0.1012260},
{-0.0121648,-0.0429718,-0.1012260},
{-0.0565386,-0.1368855,0.0368019},
{-0.0565386,-0.1368855,-0.0368019},
{-0.0764847,-0.1268949,0.0701284},
{-0.0764847,-0.1268949,-0.0701284},
{-0.0318092,-0.1311805,0.0205321},
{-0.0318092,-0.1311805,-0.0205321},
{-0.0508374,-0.0312545,0.0715307},
{-0.0508374,-0.0312545,-0.0715307},
{-0.0161632,-0.0902422,0.0370855},
{-0.0161632,-0.0902422,-0.0370855},
{-0.0170093,-0.0641454,0.0795090},
{-0.0170093,-0.0641454,-0.0795090},
{-0.0315793,-0.0873524,0.0610048},
{-0.0315793,-0.0873524,-0.0610048},
{-0.0264358,-0.0643938,0.0618754},
{-0.0264358,-0.0643938,-0.0618754},
{-0.0536040,-0.0720865,0.0986850},
{-0.0536040,-0.0720865,-0.0986850},
{-0.0077330,-0.1097997,0.0084295},
{-0.0077330,-0.1097997,-0.0084295},

Biology Open • Supplementary information

{-0.0180743,-0.0772926,0.0724365},
{-0.0180743,-0.0772926,-0.0724365},
{-0.0391526,-0.0010856,0.1148335},
{-0.0391526,-0.0010856,-0.1148335},
{-0.0417890,0.0433893,0.1273774},
{-0.0417890,0.0433893,-0.1273774},
{-0.0663807,-0.0155836,0.0750250},
{-0.0663807,-0.0155836,-0.0750250},
{-0.0635714,0.0153080,0.0540659},
{-0.0635714,0.0153080,-0.0540659},
{-0.0577213,-0.0111884,0.0608327},
{-0.0577213,-0.0111884,-0.0608327},
{-0.0694853,-0.0252248,0.0505076},
{-0.0694853,-0.0252248,-0.0505076},
{-0.0336530,-0.0552225,0.1020866},
{-0.0336530,-0.0552225,-0.1020866},
{-0.0387753,-0.0475314,0.0729551},
{-0.0387753,-0.0475314,-0.0729551},
{-0.0313073,-0.0381702,0.1024860},
{-0.0313073,-0.0381702,-0.1024860},
{-0.0400598,-0.0639014,0.0747536},
{-0.0400598,-0.0639014,-0.0747536},
{-0.0541598,-0.1121331,0.0573075},
{-0.0541598,-0.1121331,-0.0573075},
{-0.0527516,-0.0881353,0.0897645},
{-0.0527516,-0.0881353,-0.0897645},
{-0.0402213,-0.0958827,0.0800264},
{-0.0402213,-0.0958827,-0.0800264},
{-0.0822521,-0.0188612,0.0622108},
{-0.0822521,-0.0188612,-0.0622108},
{-0.0080479,0.0207710,0.1445652},
{-0.0080479,0.0207710,-0.1445652},
{-0.0014461,0.0130964,0.1427867},
{-0.0014461,0.0130964,-0.1427867},
{-0.0574376,-0.0167812,0.0593421},
{-0.0574376,-0.0167812,-0.0593421},
{-0.0686662,-0.0830874,0.0680254},
{-0.0686662,-0.0830874,-0.0680254},
{-0.0728647,-0.0664705,0.0711280},
{-0.0728647,-0.0664705,-0.0711280},
{-0.0277705,-0.0572769,0.1015934},
{-0.0277705,-0.0572769,-0.1015934},
{-0.0340011,-0.0564543,0.0913015},
{-0.0340011,-0.0564543,-0.0913015},
{-0.0143485,-0.0799117,0.0449325},
{-0.0143485,-0.0799117,-0.0449325},

Biology Open • Supplementary information

{-0.0182743,-0.0871999,0.0532384},
{-0.0182743,-0.0871999,-0.0532384},
{-0.0228693,-0.1159994,0.0187060},
{-0.0228693,-0.1159994,-0.0187060},
{-0.0169761,-0.1196543,0.0073074},
{-0.0169761,-0.1196543,-0.0073074},
{-0.0019652,-0.0975719,0.0067811},
{-0.0019652,-0.0975719,-0.0067811},
{-0.0825630,-0.0386727,0.0567592},
{-0.0825630,-0.0386727,-0.0567592},
{-0.0908911,-0.0213208,0.0481189},
{-0.0908911,-0.0213208,-0.0481189},
{-0.0892336,-0.0128087,0.0309377},
{-0.0892336,-0.0128087,-0.0309377},
{-0.0804918,-0.0822341,0.0670357},
{-0.0804918,-0.0822341,-0.0670357},
{-0.0597523,-0.0805381,0.0913610},
{-0.0597523,-0.0805381,-0.0913610},
{-0.0357570,-0.0840130,0.0662457},
{-0.0357570,-0.0840130,-0.0662457},
{-0.0475214,-0.0859111,0.0799017},
{-0.0475214,-0.0859111,-0.0799017},
{-0.0425019,-0.0836523,0.0624414},
{-0.0425019,-0.0836523,-0.0624414},
{-0.0411725,-0.0888706,0.0770761},
{-0.0411725,-0.0888706,-0.0770761},
{-0.0517570,0.0397003,0.1183278},
{-0.0517570,0.0397003,-0.1183278},
{-0.0610764,-0.0209986,0.0551229},
{-0.0610764,-0.0209986,-0.0551229},
{-0.0844740,-0.0066388,0.0062173},
{-0.0844740,-0.0066388,-0.0062173},
{-0.1032418,-0.0419115,0.0049474},
{-0.1032418,-0.0419115,-0.0049474},
{-0.1022212,-0.0725601,0.0232253},
{-0.1022212,-0.0725601,-0.0232253},
{-0.0460810,-0.0075855,0.0084505},
{-0.0460810,-0.0075855,-0.0084505},
{-0.0739106,0.0068109,0.0306199},
{-0.0739106,0.0068109,-0.0306199},
{-0.0915641,-0.0436272,0.0279940},
{-0.0915641,-0.0436272,-0.0279940},
{-0.0738279,-0.0252290,0.0198160},
{-0.0738279,-0.0252290,-0.0198160},
{-0.1089604,-0.0849010,0.0072589},
{-0.1089604,-0.0849010,-0.0072589},

Biology Open • Supplementary information

```
                    {-0.0786164,-0.0323963,0.0099433},
                    {-0.0786164,-0.0323963,-0.0099433},
                    {-0.0998468,-0.0617430,0.0295684},
                    {-0.0998468,-0.0617430,-0.0295684},
                    {-0.0849043,-0.0411061,0.0095273},
                    {-0.0849043,-0.0411061,-0.0095273}
                    };
            BoundingBoxOnOff = Off;
        };
        AnyFunTransform3DIdentity ScaleFunction = {
            PreTransforms = {&.RBFTransform};
};
};

};
```

ScalingFunctionTLEMLucyFemur_2014016

```
AnyFolder ScalingFunctionTLEMLucyFemur = {
  AnyFolder Right = {
        AnyFolder Thigh = {
        AnyFunTransform3DRBF RBFTransform = {
                RBFDef.Type = RBF_ThinPlate;
                PolynomDegree = 1;
                Points0 = {
                        {0.0000000,0.0000000,0.0000000},
                        {-0.0000000,-0.3616821,0.0000000},
                        {-0.0097563,-0.3678799,0.0012967},
                        {-0.0000000,-0.3660632,0.0408203},
                        {-0.0000000,-0.3573010,-0.0408203},
                        {0.0161460,-0.0072838,0.0601290},
                        {0.0220217,-0.0203698,0.0463848},
                        {0.0123977,-0.0241932,0.0668573},
                        {0.0006898,0.0018121,0.0538181},
                        {0.0122809,-0.0068668,0.0414535},
                        {-0.0058991,-0.0138188,0.0648412},
                        {0.0211469,-0.0380855,0.0538111},
                        {0.0172133,-0.0317342,0.0328381},
                        {0.0177311,-0.0131946,0.0256176},
                        {-0.0110079,-0.0308867,0.0632370},
                        {0.0039435,-0.0395977,0.0638271},
                        {-0.0041683,-0.0187472,0.0413214},
                        {-0.0177999,-0.0021535,0.0496084},
                        {0.0001603,-0.0053126,0.0279593},
                        {-0.0211600,-0.0216243,0.0497147},
                        {0.0096163,-0.0568663,0.0545719},
                        {0.0162112,-0.0515013,0.0395230},
                        {0.0093533,-0.0433265,0.0187387},
                        {0.0119398,-0.0238469,0.0118403},
                        {0.0210707,-0.0048848,0.0065878},
                        {0.0125013,0.0064094,0.0177702},
                        {-0.0191523,-0.0356053,0.0382429},
                        {-0.0115821,-0.0474937,0.0537648},
                        {-0.0100073,-0.0236371,0.0267322},
                        {-0.0078925,0.0107098,0.0182833},
                        {-0.0134217,-0.0055356,0.0182000},
                        {-0.0090061,-0.0665700,0.0475484},
                        {0.0071095,-0.0761976,0.0520035},
                        {0.0182119,-0.0704149,0.0386546},
                        {0.0073764,-0.0609481,0.0221006},
                        {-0.0122984,-0.0439585,0.0198428},
                        {-0.0032253,-0.0306792,0.0110995},
```

Biology Open • Supplementary information

{-0.0012975,-0.0198451,-0.0052592},
{0.0140405,-0.0161125,-0.0071649},
{0.0189222,-0.0014898,-0.0127226},
{0.0181642,0.0132812,-0.0011050},
{0.0045119,0.0200001,0.0091189},
{-0.0176761,-0.0541983,0.0348587},
{-0.0136548,-0.0142015,0.0054311},
{-0.0135510,0.0182940,0.0017175},
{-0.0223865,0.0023399,0.0028018},
{-0.0086455,-0.0738247,0.0283843},
{-0.0050356,-0.0877749,0.0442195},
{0.0160135,-0.0919319,0.0465120},
{0.0128290,-0.0785810,0.0232431},
{-0.0168328,-0.0616327,0.0155917},
{-0.0172369,-0.0094567,-0.0119502},
{0.0002090,-0.0093641,-0.0209779},
{0.0064827,0.0081538,-0.0198453},
{0.0010777,0.0210422,-0.0084721},
{-0.0129485,0.0085451,-0.0162212},
{-0.0002322,-0.0924033,0.0245644},
{-0.0028175,-0.1052263,0.0418294},
{0.0172322,-0.1111444,0.0445760},
{0.0203259,-0.0957409,0.0271449},
{0.0057102,-0.1088933,0.0195113},
{-0.0010852,-0.1230776,0.0363132},
{0.0182727,-0.1303312,0.0422884},
{0.0242891,-0.1161703,0.0265054},
{0.0094531,-0.1268584,0.0162294},
{0.0014244,-0.1413293,0.0331653},
{0.0207271,-0.1496427,0.0394157},
{0.0267562,-0.1360142,0.0236798},
{0.0113839,-0.1461651,0.0136024},
{0.0030845,-0.1595094,0.0297156},
{0.0209528,-0.1691386,0.0369775},
{0.0283958,-0.1540209,0.0213795},
{0.0129499,-0.1649012,0.0113589},
{0.0016490,-0.1783108,0.0216287},
{0.0147105,-0.1880678,0.0328614},
{0.0298491,-0.1859865,0.0248551},
{0.0286645,-0.1714377,0.0169488},
{0.0165897,-0.1861006,0.0082595},
{0.0014000,-0.1973476,0.0183740},
{0.0114301,-0.2109792,0.0286157},
{0.0267422,-0.2037622,0.0296951},
{0.0288630,-0.2018335,0.0130127},
{0.0145471,-0.2083505,0.0062833},

{0.0011300,-0.2198071,0.0155602},
{0.0126610,-0.2321235,0.0275354},
{0.0275647,-0.2219268,0.0265185},
{0.0286645,-0.2230253,0.0098701},
{0.0130434,-0.2304822,0.0039177},
{0.0022984,-0.2409585,0.0149650},
{0.0101315,-0.2534740,0.0252125},
{0.0279485,-0.2428555,0.0234276},
{0.0276684,-0.2421559,0.0058691},
{0.0101250,-0.2529527,0.0018983},
{0.0021376,-0.2667831,0.0107073},
{0.0114309,-0.2734905,0.0249960},
{0.0273923,-0.2634953,0.0216324},
{0.0278429,-0.2592546,0.0037692},
{0.0159101,-0.2712985,-0.0033988},
{0.0036708,-0.2863280,0.0004644},
{0.0023115,-0.2884756,0.0187554},
{0.0231385,-0.2879051,0.0224853},
{0.0304009,-0.2799973,0.0083549},
{0.0238189,-0.2917246,-0.0052462},
{0.0064904,-0.3027687,-0.0070587},
{-0.0003492,-0.3064690,0.0093014},
{0.0122179,-0.3045269,0.0243511},
{0.0301837,-0.3030127,0.0114462},
{0.0234182,-0.3107336,-0.0085198},
{0.0046893,-0.3214995,-0.0123881},
{-0.0022352,-0.3264905,0.0043340},
{0.0016841,-0.3202756,0.0217839},
{0.0229722,-0.3196887,0.0215083},
{0.0292438,-0.3228042,0.0051430},
{0.0221641,-0.3296190,-0.0132361},
{0.0100459,-0.3417597,-0.0244560},
{-0.0053009,-0.3375737,-0.0137705},
{-0.0064432,-0.3487307,0.0028676},
{-0.0059346,-0.3407801,0.0202938},
{0.0105130,-0.3365976,0.0264175},
{0.0282695,-0.3386249,0.0180307},
{0.0270402,-0.3431844,-0.0001487},
{0.0257040,-0.3506950,-0.0181851},
{0.0178948,-0.3636114,-0.0302838},
{0.0043149,-0.3544109,-0.0381949},
{-0.0095210,-0.3461482,-0.0295031},
{-0.0164526,-0.3542895,-0.0126666},
{-0.0060896,-0.3690127,-0.0033061},
{-0.0176523,-0.3612686,0.0110109},
{-0.0153310,-0.3546943,0.0299634},

{0.0021329,-0.3508021,0.0350763},
{0.0210690,-0.3548628,0.0279835},
{0.0356594,-0.3558162,0.0159356},
{0.0275718,-0.3617450,-0.0018607},
{0.0298503,-0.3741530,-0.0163721},
{0.0175320,-0.3855300,-0.0277515},
{0.0025604,-0.3740099,-0.0397137},
{-0.0136727,-0.3624016,-0.0402102},
{-0.0277647,-0.3567472,-0.0284947},
{-0.0275446,-0.3699630,-0.0120829},
{-0.0175846,-0.3859355,-0.0177922},
{0.0001572,-0.3856873,-0.0118050},
{0.0030262,-0.3834856,0.0075553},
{-0.0144114,-0.3810887,0.0140304},
{-0.0247706,-0.3701239,0.0285134},
{-0.0044804,-0.3677201,0.0404632},
{0.0133183,-0.3712274,0.0334208},
{0.0324861,-0.3723417,0.0245668},
{0.0271451,-0.3766027,0.0072441},
{0.0167985,-0.3822816,-0.0065349},
{-0.0001454,-0.3910823,-0.0303010},
{-0.0144998,-0.3834154,-0.0384137},
{-0.0301321,-0.3750841,-0.0321336},
{0.0196491,-0.3864804,0.0210853},
{0.0015726,-0.3888034,0.0276569},
{-0.0146652,-0.3847848,0.0336913},
{-0.0014588,-0.0619951,0.0219778},
{-0.0118435,-0.0550626,0.0139639},
{0.0129812,-0.0349966,0.0673128},
{-0.0162907,-0.0105666,0.0597739},
{-0.0012885,-0.0520816,0.0562989},
{-0.0090599,-0.0631298,0.0172224},
{-0.0000222,-0.0771663,0.0231897},
{-0.0068148,0.0023885,-0.0160086},
{-0.0180544,-0.0425409,0.0446585},
{0.0020525,-0.0249904,0.0691549},
{-0.0157097,-0.0662315,0.0238393},
{-0.0010518,-0.0510686,0.0199353},
{-0.0208874,-0.0152865,0.0500034},
{0.0168316,-0.0280857,0.0666680},
{-0.0187392,-0.0282333,0.0403282},
{0.0191622,-0.0351633,0.0622707},
{0.0001216,-0.0706289,0.0524049},
{-0.0169862,-0.0399062,0.0311827},
{-0.0185737,-0.0533066,0.0151224},
{-0.0219058,-0.0528723,0.0222263},

Biology Open • Supplementary information

{-0.0028271,-0.0391620,0.0148452},
{0.0117900,-0.0553425,0.0235348},
{0.0219835,-0.0106400,0.0396338},
{-0.0025366,-0.0401046,0.0625273},
{0.0141017,-0.0018767,0.0198108},
{-0.0078770,-0.0473929,0.0173542},
{-0.0065351,-0.0766431,0.0470258},
{0.0065585,0.0145593,0.0161638},
{-0.0118588,0.0172875,-0.0085064},
{-0.0074087,-0.0627500,0.0506700},
{0.0071940,-0.0780068,0.0213362},
{0.0126736,-0.0708176,0.0244108},
{0.0074694,-0.0436450,0.0604196},
{0.0154186,-0.0139471,0.0064312},
{-0.0053535,-0.0213210,0.0040094},
{-0.0088733,0.0002018,0.0205402},
{-0.0112471,-0.0542608,0.0500266},
{-0.0061625,-0.0248673,0.0653244},
{0.0093695,-0.0065826,0.0614157},
{0.0138015,-0.0467322,0.0284333},
{-0.0020999,-0.0186885,-0.0116392},
{-0.0185640,-0.0225591,0.0566779},
{0.0175798,0.0124360,-0.0068467},
{-0.0039962,0.0217127,0.0053055},
{0.0014975,-0.0784591,0.0514056},
{-0.0172997,-0.0386971,0.0525252},
{0.0196540,-0.0268271,0.0416806},
{0.0135296,-0.0406504,0.0236882},
{0.0182471,0.0095959,0.0087906},
{0.0218667,0.0016804,0.0058793},
{0.0224975,-0.0022652,-0.0022994},
{0.0166750,-0.0191391,0.0236436},
{0.0119785,-0.0328723,0.0195376},
{0.0187631,-0.0133469,0.0308741},
{-0.0187543,-0.0461463,0.0257491},
{0.0039834,-0.0373928,0.0683089},
{0.0157029,-0.0423169,0.0385226},
{0.0164003,-0.0087069,0.0131181},
{0.0008253,0.0033370,-0.0212576},
{0.0101707,-0.0009724,-0.0205688},
{0.0102948,-0.0642683,0.0524532},
{0.0152217,-0.0557944,0.0508978},
{0.0172973,-0.0565707,0.0419468},
{0.0167723,-0.0635334,0.0369834},
{-0.0047397,-0.0695325,0.0241398},
{-0.0152913,0.0035778,0.0161461},

Biology Open • Supplementary information

{0.0091697,0.0009340,0.0575160},
{0.0196686,-0.0089533,0.0500006},
{0.0183998,-0.0212943,0.0622968},
{-0.0114340,-0.0316810,0.0250940},
{-0.0206442,-0.0092599,-0.0028425},
{0.0166868,-0.0784295,0.0276925},
{-0.0069678,-0.0054521,-0.0207011},
{0.0049490,0.0151109,-0.0160093},
{-0.0084025,-0.0318937,0.0162788},
{-0.0173679,0.0095594,-0.0108509},
{-0.0218912,0.0063457,-0.0020698},
{-0.0106547,-0.0668750,0.0397749},
{0.0136098,-0.0170666,-0.0018890},
{0.0141599,-0.0441405,0.0587594},
{0.0093255,-0.0183218,-0.0083008},
{0.0106367,-0.0128276,-0.0154269},
{0.0037350,-0.0549365,0.0209786},
{0.0087662,-0.0472902,0.0195496},
{0.0016839,-0.0708213,0.0221325},
{-0.0202977,-0.0530540,0.0301270},
{-0.0068584,0.0021781,0.0530002},
{-0.0144817,-0.0297218,0.0605833},
{0.0197015,-0.0165217,0.0560654},
{0.0156609,-0.0705363,0.0477736},
{0.0207876,-0.0221433,0.0330733},
{0.0092324,-0.0300767,0.0713160},
{-0.0203392,-0.0034488,0.0097332},
{0.0156978,-0.0143438,0.0629053},
{0.0143267,-0.0563519,0.0317489},
{-0.0161685,-0.0465391,0.0188591},
{-0.0196397,-0.0309875,0.0546496},
{0.0154804,-0.0380422,0.0305433},
{-0.0077774,-0.0351303,0.0637765},
{0.0063980,-0.0086419,-0.0201654},
{0.0030146,-0.0151094,-0.0165974},
{-0.0210080,-0.0096030,0.0507855},
{-0.0105684,-0.0411377,0.0586146},
{0.0180619,-0.0498960,0.0469835},
{-0.0192318,0.0116108,0.0024070},
{-0.0165651,0.0083622,0.0126870},
{-0.0014333,-0.0627138,0.0534746},
{0.0145127,-0.0080178,0.0353667},
{0.0148374,-0.0012228,0.0566181},
{0.0153834,-0.0062427,0.0444727},
{-0.0155164,-0.0132103,-0.0096398},
{0.0068685,-0.0221022,0.0011547},

Biology Open • Supplementary information

{0.0003332,-0.0458500,0.0183716},
{0.0205582,0.0040956,-0.0082969},
{0.0175752,0.0009340,-0.0148407},
{0.0142357,-0.0063623,-0.0169332},
{-0.0147285,0.0023412,-0.0164678},
{-0.0158781,-0.0064451,-0.0151346},
{0.0175951,0.0133456,0.0037559},
{0.0117660,0.0189899,-0.0030690},
{0.0096040,0.0151827,-0.0137450},
{0.0096565,-0.0215194,0.0662734},
{-0.0006793,-0.0074494,0.0631218},
{0.0007819,-0.0004519,0.0600781},
{0.0065377,-0.0125433,0.0654199},
{0.0090461,0.0073096,0.0188711},
{0.0151418,-0.0168302,0.0146465},
{0.0025166,-0.0231863,0.0688894},
{-0.0097891,-0.0152077,0.0253086},
{-0.0109149,-0.0177935,0.0119825},
{-0.0072079,-0.0099373,0.0272725},
{-0.0183727,-0.0467429,0.0356276},
{-0.0126871,-0.0584555,0.0428141},
{-0.0057054,-0.0273042,0.0103554},
{0.0215142,-0.0159693,0.0474531},
{0.0128492,-0.0070016,0.0287645},
{0.0052134,-0.0017047,0.0247463},
{-0.0024284,-0.0707498,0.0513049},
{-0.0036140,0.0220593,-0.0040484},
{-0.0002405,-0.0121253,0.0390050},
{-0.0119811,-0.0156737,0.0015445},
{0.0142082,-0.0279792,0.0246553},
{0.0122244,-0.0262013,0.0169829},
{-0.0146264,-0.0038855,0.0549384},
{0.0207450,-0.0325771,0.0496412},
{-0.0155040,-0.0497560,0.0437087},
{-0.0081119,-0.0756450,0.0387365},
{0.0039684,-0.0062047,0.0313900},
{-0.0023726,-0.0038892,-0.0220521},
{-0.0136977,-0.0339839,0.0602619},
{-0.0035399,0.0089081,-0.0188894},
{-0.0049820,0.0176571,-0.0132254},
{0.0193902,-0.0803191,0.0360429},
{0.0040648,-0.0697109,0.0218205},
{0.0170470,-0.0818610,0.0456892},
{0.0104175,0.0188635,0.0064965},
{0.0135206,0.0092549,0.0158079},
{0.0023933,0.0160498,-0.0157404},

{0.0164753,-0.0118197,-0.0096995},
{0.0202584,-0.0066858,-0.0082383},
{-0.0098401,-0.0226001,0.0314028},
{-0.0130937,-0.0309622,0.0320764},
{-0.0201510,-0.0613297,0.0222264},
{-0.0010389,0.0036055,0.0209003},
{0.0042025,0.0073392,0.0199393},
{-0.0021630,-0.0038531,0.0251898},
{-0.0007845,-0.0293574,0.0675788},
{0.0070027,-0.0406327,0.0624565},
{-0.0124363,-0.0183001,0.0627966},
{-0.0120485,-0.0256613,0.0628493},
{0.0101200,0.0197695,0.0039334},
{0.0167636,0.0148266,0.0014155},
{-0.0074981,-0.0106930,0.0632797},
{0.0142533,-0.0503445,0.0351954},
{-0.0146107,-0.0460953,0.0506120},
{-0.0161117,-0.0471301,0.0178900},
{0.0112149,-0.0792793,0.0501460},
{-0.0099060,-0.0230794,0.0186951},
{-0.0114543,-0.0153049,0.0183675},
{-0.0042882,0.0161879,0.0151423},
{0.0014582,-0.0638572,0.0541080},
{0.0048554,-0.0582603,0.0553815},
{-0.0017077,-0.0572302,0.0546635},
{0.0166090,-0.0697169,0.0323038},
{-0.0208812,-0.0164273,0.0474354},
{0.0130454,-0.0241864,0.0178294},
{0.0053123,-0.0483770,0.0582251},
{0.0112225,-0.0361429,0.0673300},
{0.0085729,-0.0771256,0.0216100},
{-0.0070783,-0.0488033,0.0560869},
{0.0182083,-0.0411024,0.0454236},
{-0.0119590,-0.0403809,0.0213546},
{0.0233409,-0.0185535,0.0366343},
{-0.0046347,-0.0571211,0.0208243},
{0.0183166,-0.0006966,0.0140116},
{0.0061913,0.0053512,0.0201003},
{-0.0002364,-0.0310828,0.0680351},
{-0.0170773,0.0139270,-0.0054987},
{0.0110805,0.0156034,0.0117010},
{-0.0124628,0.0164667,0.0094550},
{-0.0209014,-0.0073407,-0.0051969},
{-0.0210131,0.0010693,-0.0086891},
{-0.0041596,-0.0172332,0.0663041},
{-0.0216595,-0.0322172,0.0451540},

Biology Open • Supplementary information

{0.0051908,-0.0426014,0.0613013},
{0.0150003,-0.0416136,0.0606257},
{-0.0135090,-0.0674234,0.0236425},
{0.0146416,0.0073691,0.0160052},
{0.0195615,-0.0112412,-0.0010333},
{-0.0198115,-0.0098244,0.0044735},
{-0.0157054,-0.0521236,0.0395039},
{-0.0081381,-0.0173108,-0.0046152},
{-0.0218324,-0.0262661,0.0471115},
{0.0051446,-0.0707257,0.0530555},
{0.0179359,-0.0471493,0.0528134},
{-0.0005591,-0.0317983,0.0113632},
{0.0071707,-0.0305943,0.0121186},
{0.0206239,-0.0061367,0.0066642},
{0.0187057,-0.0745868,0.0420207},
{-0.0095784,-0.0163113,0.0259524},
{-0.0012181,0.0127661,0.0186855},
{0.0059420,-0.0630691,0.0220087},
{0.0069408,-0.0573878,0.0552614},
{0.0150782,-0.0132884,-0.0101793},
{0.0005533,-0.0078574,0.0633775},
{-0.0088284,-0.0146399,-0.0147837},
{0.0167444,-0.0651105,0.0470347},
{0.0082666,-0.0181741,-0.0095133},
{-0.0175162,-0.0637444,0.0177069},
{0.0153795,-0.0360767,0.0287608},
{0.0162363,-0.0018864,0.0548412},
{0.0052731,-0.0075919,0.0392990},
{-0.0022288,-0.0556644,0.0549909},
{-0.0156238,-0.0550038,0.0377688},
{-0.0204137,-0.0100550,0.0536429},
{-0.0093683,0.0122429,-0.0162013},
{-0.0202927,0.0049166,0.0083887},
{0.0074706,-0.0627945,0.0222133},
{0.0035034,-0.0376103,0.0672421},
{-0.0190752,-0.0194129,0.0555530},
{0.0061758,-0.0074961,-0.0207741},
{0.0192011,-0.0273063,0.0588233},
{0.0130796,-0.0134383,0.0638611},
{0.0120421,0.0019566,-0.0194140},
{0.0154152,-0.0065294,0.0179581},
{-0.0157335,0.0101219,0.0123981},
{0.0110376,-0.0488274,0.0567911},
{-0.0055170,-0.0343676,0.0647140},
{0.0216204,-0.0273988,0.0512041},
{0.0161331,-0.0135875,-0.0075501},

Biology Open • Supplementary information

{-0.0184643,-0.0455633,0.0360087},
{-0.0091978,-0.0682413,0.0460847},
{0.0034304,-0.0471677,0.0588866},
{-0.0048025,-0.0384316,0.0149400},
{0.0118505,0.0109735,-0.0159161},
{-0.0027036,-0.0204189,-0.0015094},
{-0.0117607,-0.0692759,0.0292171},
{-0.0107080,-0.0033455,-0.0190487},
{-0.0042548,0.0214947,-0.0056846},
{-0.0097315,-0.0208529,0.0131997},
{-0.0178958,-0.0627218,0.0171551},
{-0.0068692,-0.0688818,0.0233316},
{0.0012083,0.0120695,-0.0186179},
{0.0215073,-0.0236027,0.0503069},
{-0.0060262,-0.0534931,0.0189523},
{0.0179951,-0.0336785,0.0397267},
{0.0190509,-0.0284972,0.0336394},
{-0.0169188,-0.0133609,-0.0014203},
{0.0135722,0.0136491,0.0114695},
{-0.0150549,-0.0123230,0.0067722},
{-0.0148411,-0.0311945,0.0355412},
{0.0185490,-0.0794042,0.0431266},
{0.0193568,-0.0087326,0.0575704},
{0.0201026,-0.0144353,0.0531586},
{0.0025583,-0.0248211,0.0045714},
{0.0132689,-0.0214866,0.0652899},
{-0.0028669,-0.0099311,0.0309271},
{-0.0219487,-0.0054893,-0.0021853},
{0.0026180,-0.0203806,-0.0040620},
{-0.0116230,-0.0668518,0.0370152},
{-0.0133007,-0.0264159,0.0619176},
{0.0186167,0.0050491,-0.0120059},
{-0.0191359,-0.0591690,0.0288149},
{-0.0178121,-0.0041834,0.0141448},
{0.0123077,-0.0063648,0.0279743},
{-0.0157729,-0.0374010,0.0315335},
{0.0149328,-0.0659100,0.0290095},
{0.0006205,0.0021977,-0.0214613},
{0.0170484,-0.0715687,0.0325980},
{0.0068499,0.0061192,0.0196608},
{-0.0167075,0.0020024,0.0150382},
{0.0059801,-0.0393117,0.0167489},
{-0.0072506,-0.0171067,0.0338751},
{0.0202363,-0.0251596,0.0420997},
{-0.0199311,-0.0601255,0.0171522},
{0.0216641,0.0053410,-0.0027841},

Biology Open • Supplementary information

{-0.0197665,-0.0606125,0.0252972},
{0.0129986,-0.0214528,0.0098002},
{0.0201151,-0.0100929,0.0460501},
{0.0104137,0.0017765,-0.0203073},
{0.0166557,-0.0064123,0.0140197},
{-0.0124120,-0.0596549,0.0422637},
{0.0014885,0.0202590,0.0096781},
{0.0028720,0.0020207,-0.0219044},
{0.0376095,-0.3675427,0.0214537},
{0.0049949,0.0206417,-0.0079017},
{-0.0191723,-0.0407590,0.0391310},
{-0.0316406,-0.3638215,-0.0274841},
{0.0021392,0.0226920,0.0000985},
{0.0168435,-0.0229382,0.0264788},
{-0.0157304,-0.0005437,-0.0161456},
{0.0203722,-0.0299750,0.0562762},
{-0.0171077,0.0134146,0.0059615},
{0.0175519,-0.0370653,0.0413374},
{0.0154657,-0.0405127,0.0348980},
{-0.0215172,-0.0323502,0.0481925},
{0.0177998,-0.0515776,0.0444709},
{-0.0183804,-0.0448316,0.0275139},
{0.0033607,-0.0502028,0.0575894},
{0.0010325,-0.0479377,0.0191141},
{0.0224852,-0.0181821,0.0451780},
{0.0084089,-0.0720538,0.0521031},
{-0.0070880,-0.0772912,0.0288023},
{-0.0223712,-0.0028990,0.0024047},
{0.0173136,-0.0120017,0.0204612},
{0.0137772,-0.0623537,0.0276621},
{-0.0100390,-0.0231180,0.0292222},
{-0.0196395,-0.3730207,0.0364730},
{-0.0094445,-0.0526688,0.0528111},
{-0.0115309,-0.3679776,-0.0104995},
{0.0129812,-0.0410477,0.0224072},
{0.0117394,0.0154849,-0.0117191},
{-0.0245208,-0.3649412,0.0295111},
{0.0147476,-0.0502966,0.0364899},
{0.0006132,-0.0446771,0.0600524},
{-0.0118123,0.0191139,0.0031977},
{-0.0017185,-0.0163303,0.0664216},
{-0.0085098,-0.0074564,0.0247745},
{0.0197459,-0.0086852,0.0430460},
{0.0187064,-0.0223532,0.0600966},
{-0.0206552,-0.0099712,0.0525024},
{0.0207525,0.0084646,-0.0024846},

{-0.0094915,-0.0325446,0.0184441},
{-0.0014464,-0.0140050,-0.0179819},
{-0.0122725,-0.0573444,0.0463125},
{-0.0106397,0.0177211,0.0094669},
{0.0111981,0.0191236,-0.0041491},
{-0.0162790,-0.0256279,0.0592687},
{-0.0060120,-0.3653959,0.0413411},
{-0.0135007,-0.3703638,-0.0387476},
{-0.0134258,-0.3703142,-0.0379467},
{-0.0133509,-0.3702645,-0.0371458},
{-0.0132760,-0.3702148,-0.0363450},
{-0.0132011,-0.3701651,-0.0355441},
{-0.0131262,-0.3701154,-0.0347432},
{-0.0130513,-0.3700658,-0.0339423},
{-0.0129765,-0.3700161,-0.0331414},
{-0.0129016,-0.3699664,-0.0323405},
{-0.0128267,-0.3699167,-0.0315396},
{-0.0127518,-0.3698670,-0.0307388},
{-0.0126769,-0.3698174,-0.0299379},
{-0.0126020,-0.3697677,-0.0291370},
{-0.0125271,-0.3697180,-0.0283361},
{-0.0124523,-0.3696683,-0.0275352},
{-0.0123774,-0.3696187,-0.0267343},
{-0.0123025,-0.3695690,-0.0259334},
{-0.0122276,-0.3695193,-0.0251325},
{-0.0121527,-0.3694696,-0.0243317},
{-0.0120778,-0.3694199,-0.0235308},
{-0.0120029,-0.3693703,-0.0227299},
{-0.0119280,-0.3693206,-0.0219290},
{-0.0118532,-0.3692709,-0.0211281},
{-0.0117783,-0.3692212,-0.0203272},
{-0.0117034,-0.3691715,-0.0195263},
{-0.0116285,-0.3691219,-0.0187255},
{-0.0115536,-0.3690722,-0.0179246},
{-0.0114787,-0.3690225,-0.0171237},
{-0.0114038,-0.3689728,-0.0163228},
{-0.0113289,-0.3689231,-0.0155219},
{-0.0112541,-0.3688735,-0.0147210},
{-0.0111792,-0.3688238,-0.0139201},
{-0.0111043,-0.3687741,-0.0131192},
{-0.0110294,-0.3687244,-0.0123184},
{-0.0109545,-0.3686748,-0.0115175},
{-0.0108796,-0.3686251,-0.0107166},
{-0.0108047,-0.3685754,-0.0099157},
{-0.0107298,-0.3685257,-0.0091148},
{-0.0106550,-0.3684760,-0.0083139},

Biology Open • Supplementary information

{-0.0105801,-0.3684264,-0.0075130},
{-0.0105052,-0.3683767,-0.0067121},
{-0.0104303,-0.3683270,-0.0059113},
{-0.0103554,-0.3682773,-0.0051104},
{-0.0102805,-0.3682276,-0.0043095},
{-0.0102056,-0.3681780,-0.0035086},
{-0.0101307,-0.3681283,-0.0027077},
{-0.0100559,-0.3680786,-0.0019068},
{-0.0099810,-0.3680289,-0.0011059},
{-0.0099061,-0.3679792,-0.0003051},
{-0.0098312,-0.3679296,0.0004958},
{-0.0097563,-0.3678799,0.0012967},
{-0.0096814,-0.3678302,0.0020976},
{-0.0096065,-0.3677805,0.0028985},
{-0.0095317,-0.3677308,0.0036994},
{-0.0094568,-0.3676812,0.0045003},
{-0.0093819,-0.3676315,0.0053012},
{-0.0093070,-0.3675818,0.0061020},
{-0.0092321,-0.3675321,0.0069029},
{-0.0091572,-0.3674825,0.0077038},
{-0.0090823,-0.3674328,0.0085047},
{-0.0090074,-0.3673831,0.0093056},
{-0.0089326,-0.3673334,0.0101065},
{-0.0088577,-0.3672837,0.0109074},
{-0.0087828,-0.3672341,0.0117082},
{-0.0087079,-0.3671844,0.0125091},
{-0.0086330,-0.3671347,0.0133100},
{-0.0085581,-0.3670850,0.0141109},
{-0.0084832,-0.3670353,0.0149118},
{-0.0084083,-0.3669857,0.0157127},
{-0.0083335,-0.3669360,0.0165136},
{-0.0082586,-0.3668863,0.0173145},
{-0.0081837,-0.3668366,0.0181153},
{-0.0081088,-0.3667869,0.0189162},
{-0.0080339,-0.3667373,0.0197171},
{-0.0079590,-0.3666876,0.0205180},
{-0.0078841,-0.3666379,0.0213189},
{-0.0078092,-0.3665882,0.0221198},
{-0.0077344,-0.3665386,0.0229207},
{-0.0076595,-0.3664889,0.0237215},
{-0.0075846,-0.3664392,0.0245224},
{-0.0075097,-0.3663895,0.0253233},
{-0.0074348,-0.3663398,0.0261242},
{-0.0073599,-0.3662902,0.0269251},
{-0.0072850,-0.3662405,0.0277260},
{-0.0072101,-0.3661908,0.0285269},

```
{-0.0071353,-0.3661411,0.0293278},
{-0.0070604,-0.3660914,0.0301286},
{-0.0069855,-0.3660418,0.0309295},
{-0.0069106,-0.3659921,0.0317304},
{-0.0068357,-0.3659424,0.0325313},
{-0.0067608,-0.3658927,0.0333322},
{-0.0066859,-0.3658430,0.0341331},
{-0.0066111,-0.3657934,0.0349340},
{-0.0065362,-0.3657437,0.0357348},
{-0.0064613,-0.3656940,0.0365357},
{-0.0063864,-0.3656443,0.0373366},
{-0.0063115,-0.3655947,0.0381375},
{-0.0062366,-0.3655450,0.0389384},
{-0.0061617,-0.3654953,0.0397393},
{-0.0060868,-0.3654456,0.0405402}
};
Points1 = {
{0.0000000,0.0000000,0.0000000},
{-0.0000000,-0.4374563,0.0000000},
{-0.0094266,-0.4449526,0.0012529},
{-0.0000000,-0.4427553,0.0394409},
{-0.0000000,-0.4321573,-0.0394409},
{0.0140331,-0.0098308,0.0565778},
{0.0203620,-0.0255678,0.0445293},
{0.0097861,-0.0290004,0.0617219},
{0.0001582,-0.0004522,0.0503626},
{0.0113646,-0.0077761,0.0392483},
{-0.0059910,-0.0159263,0.0640130},
{0.0170434,-0.0440199,0.0510037},
{0.0152701,-0.0382232,0.0327739},
{0.0144236,-0.0164583,0.0247953},
{-0.0102631,-0.0365551,0.0610785},
{0.0033999,-0.0478527,0.0600708},
{-0.0031127,-0.0229140,0.0407430},
{-0.0126350,-0.0049177,0.0474516},
{-0.0000090,-0.0048240,0.0275310},
{-0.0124069,-0.0260942,0.0487785},
{0.0101933,-0.0692574,0.0535851},
{0.0162108,-0.0615274,0.0381743},
{0.0084505,-0.0519532,0.0191417},
{0.0097727,-0.0276687,0.0124876},
{0.0185184,-0.0062774,0.0057081},
{0.0104789,0.0069210,0.0159300},
{-0.0123426,-0.0435263,0.0363007},
{-0.0101394,-0.0565818,0.0515759},
{-0.0093082,-0.0291261,0.0249195},
```

Biology Open • Supplementary information

{-0.0061800,0.0121834,0.0159591},
{-0.0114690,-0.0067027,0.0157318},
{-0.0075062,-0.0800178,0.0447273},
{0.0063519,-0.0918186,0.0519585},
{0.0172626,-0.0860707,0.0368248},
{0.0083781,-0.0741208,0.0210328},
{-0.0118593,-0.0526285,0.0206706},
{-0.0042320,-0.0374095,0.0102133},
{-0.0004477,-0.0227053,-0.0048082},
{0.0128965,-0.0177953,-0.0065283},
{0.0174258,-0.0020457,-0.0111489},
{0.0170862,0.0148214,-0.0012137},
{0.0031580,0.0224924,0.0090157},
{-0.0143399,-0.0653307,0.0339183},
{-0.0119138,-0.0142758,0.0058056},
{-0.0121943,0.0193931,0.0016101},
{-0.0186025,0.0026885,0.0031766},
{-0.0068441,-0.0886311,0.0277759},
{-0.0043309,-0.1062699,0.0421167},
{0.0146953,-0.1116086,0.0448933},
{0.0125444,-0.0953582,0.0218087},
{-0.0101829,-0.0707882,0.0214703},
{-0.0146119,-0.0101030,-0.0102417},
{0.0006616,-0.0104337,-0.0187176},
{0.0060103,0.0088733,-0.0172288},
{0.0016911,0.0229528,-0.0081398},
{-0.0105781,0.0096027,-0.0145644},
{0.0004454,-0.1118004,0.0233586},
{-0.0018708,-0.1271060,0.0401600},
{0.0158259,-0.1345778,0.0428309},
{0.0188769,-0.1156938,0.0263862},
{0.0062768,-0.1313577,0.0193752},
{0.0000139,-0.1484169,0.0351666},
{0.0169394,-0.1575863,0.0406188},
{0.0225293,-0.1404263,0.0259038},
{0.0097650,-0.1529028,0.0165534},
{0.0023500,-0.1703923,0.0322925},
{0.0192752,-0.1808478,0.0378898},
{0.0248794,-0.1643281,0.0232860},
{0.0115328,-0.1761854,0.0141246},
{0.0038646,-0.1923425,0.0290591},
{0.0195788,-0.2043690,0.0355678},
{0.0264418,-0.1860612,0.0210982},
{0.0129202,-0.1988534,0.0119522},
{0.0026565,-0.2150039,0.0215052},
{0.0140899,-0.2271405,0.0317362},

Biology Open • Supplementary information

{0.0277446,-0.2247796,0.0241579},
{0.0267919,-0.2070813,0.0169139},
{0.0161443,-0.2245743,0.0088940},
{0.0023653,-0.2380669,0.0183624},
{0.0111603,-0.2548327,0.0277042},
{0.0249141,-0.2463032,0.0285983},
{0.0270471,-0.2438677,0.0130662},
{0.0142582,-0.2515244,0.0068946},
{0.0019972,-0.2653184,0.0155732},
{0.0122220,-0.2804966,0.0265715},
{0.0257192,-0.2683062,0.0255406},
{0.0269139,-0.2695411,0.0099852},
{0.0128296,-0.2783630,0.0045006},
{0.0029034,-0.2910293,0.0148397},
{0.0098790,-0.3063700,0.0243124},
{0.0261408,-0.2936663,0.0225473},
{0.0260660,-0.2927165,0.0061038},
{0.0100776,-0.3056217,0.0024140},
{0.0025883,-0.3223948,0.0106390},
{0.0110214,-0.3306774,0.0240298},
{0.0257208,-0.3186779,0.0207826},
{0.0262745,-0.3134547,0.0039935},
{0.0153575,-0.3279418,-0.0028025},
{0.0038900,-0.3461335,0.0007567},
{0.0025216,-0.3487839,0.0181330},
{0.0219331,-0.3482317,0.0215724},
{0.0287061,-0.3386509,0.0081652},
{0.0227405,-0.3527857,-0.0048048},
{0.0064102,-0.3661193,-0.0066299},
{-0.0001667,-0.3706180,0.0090440},
{0.0117577,-0.3683163,0.0234375},
{0.0287828,-0.3665248,0.0110357},
{0.0225189,-0.3758228,-0.0081400},
{0.0045308,-0.3888552,-0.0119694},
{-0.0021596,-0.3948919,0.0041875},
{0.0016272,-0.3873750,0.0210478},
{0.0221833,-0.3866673,0.0207757},
{0.0282479,-0.3904343,0.0049701},
{0.0214151,-0.3986758,-0.0127888},
{0.0097065,-0.4133601,-0.0236296},
{-0.0051218,-0.4082971,-0.0133052},
{-0.0062254,-0.4217915,0.0027707},
{-0.0057341,-0.4121753,0.0196081},
{0.0101577,-0.4071164,0.0255248},
{0.0273152,-0.4095686,0.0174214},
{0.0261274,-0.4150832,-0.0001437},

Biology Open • Supplementary information

{0.0248335,-0.4241673,-0.0175697},
{0.0172901,-0.4397898,-0.0292605},
{0.0041691,-0.4286618,-0.0369013},
{-0.0091992,-0.4186679,-0.0285062},
{-0.0158966,-0.4285149,-0.0122385},
{-0.0058838,-0.4463227,-0.0031944},
{-0.0170558,-0.4369562,0.0106388},
{-0.0148130,-0.4290045,0.0289509},
{0.0020608,-0.4242969,0.0338910},
{0.0203571,-0.4292084,0.0270379},
{0.0344535,-0.4303615,0.0153971},
{0.0266411,-0.4375325,-0.0017978},
{0.0288416,-0.4525399,-0.0158189},
{0.0169405,-0.4663005,-0.0268137},
{0.0024738,-0.4523669,-0.0383717},
{-0.0132117,-0.4383265,-0.0388524},
{-0.0268265,-0.4314875,-0.0275328},
{-0.0266138,-0.4474721,-0.0116746},
{-0.0169895,-0.4667910,-0.0171910},
{0.0001519,-0.4664908,-0.0114060},
{0.0029239,-0.4638277,0.0073000},
{-0.0139244,-0.4609286,0.0135562},
{-0.0239336,-0.4476667,0.0275499},
{-0.0043290,-0.4447593,0.0390959},
{0.0128682,-0.4490014,0.0322915},
{0.0313893,-0.4503491,0.0237367},
{0.0262279,-0.4555029,0.0069993},
{0.0162308,-0.4623715,-0.0063140},
{-0.0001405,-0.4730159,-0.0292780},
{-0.0140098,-0.4637429,-0.0371157},
{-0.0291138,-0.4536662,-0.0310478},
{0.0189841,-0.4674499,0.0203728},
{0.0015194,-0.4702596,0.0267233},
{-0.0141687,-0.4653992,0.0325528},
{-0.0008174,-0.0760948,0.0187565},
{-0.0097992,-0.0662421,0.0203399},
{0.0093408,-0.0388586,0.0608710},
{-0.0134778,-0.0157896,0.0561074},
{-0.0016521,-0.0647282,0.0564063},
{-0.0059784,-0.0726882,0.0221301},
{-0.0013390,-0.0933983,0.0202085},
{-0.0066387,0.0019297,-0.0167453},
{-0.0118351,-0.0508017,0.0405316},
{0.0031383,-0.0302818,0.0645382},
{-0.0096496,-0.0750887,0.0253121},
{-0.0010134,-0.0617145,0.0180743},

{-0.0125137,-0.0193808,0.0500112},
{0.0129304,-0.0342732,0.0592178},
{-0.0099397,-0.0340412,0.0390868},
{0.0135727,-0.0391744,0.0575599},
{-0.0008463,-0.0852545,0.0528694},
{-0.0140750,-0.0499104,0.0307740},
{-0.0132617,-0.0632893,0.0216489},
{-0.0154433,-0.0619012,0.0228999},
{-0.0023558,-0.0470422,0.0133629},
{0.0102869,-0.0659450,0.0235172},
{0.0206089,-0.0150832,0.0389454},
{-0.0029679,-0.0501126,0.0609597},
{0.0128851,-0.0031940,0.0169448},
{-0.0084190,-0.0553653,0.0186093},
{-0.0056754,-0.0920394,0.0436464},
{0.0053084,0.0168081,0.0129086},
{-0.0107599,0.0193385,-0.0059176},
{-0.0067202,-0.0764642,0.0467187},
{0.0074651,-0.0961732,0.0190844},
{0.0123111,-0.0843653,0.0238901},
{0.0073762,-0.0535569,0.0578095},
{0.0137503,-0.0143146,0.0074614},
{-0.0055031,-0.0266915,0.0045001},
{-0.0068490,-0.0011010,0.0197368},
{-0.0096224,-0.0637475,0.0480734},
{-0.0059021,-0.0296590,0.0645996},
{0.0080161,-0.0072016,0.0590007},
{0.0129660,-0.0572515,0.0291077},
{-0.0019126,-0.0205407,-0.0118412},
{-0.0131358,-0.0277014,0.0536401},
{0.0152638,0.0150712,-0.0068914},
{-0.0038670,0.0244229,0.0035903},
{0.0006016,-0.0963149,0.0512792},
{-0.0123785,-0.0447775,0.0485631},
{0.0189250,-0.0316636,0.0412366},
{0.0110396,-0.0486044,0.0234427},
{0.0169856,0.0117642,0.0070162},
{0.0195753,0.0023561,0.0065535},
{0.0204912,-0.0022424,-0.0033171},
{0.0131261,-0.0227721,0.0218692},
{0.0108955,-0.0391982,0.0208801},
{0.0154633,-0.0166183,0.0312847},
{-0.0165361,-0.0554427,0.0257171},
{0.0028613,-0.0433408,0.0626237},
{0.0159978,-0.0510484,0.0372683},
{0.0138278,-0.0114486,0.0128712},

Biology Open • Supplementary information

{0.0009765,0.0049648,-0.0188357},
{0.0082526,-0.0008626,-0.0186925},
{0.0098842,-0.0761817,0.0520743},
{0.0141165,-0.0687940,0.0494686},
{0.0165807,-0.0668397,0.0411337},
{0.0167704,-0.0773777,0.0347863},
{-0.0044661,-0.0844243,0.0238066},
{-0.0125472,0.0042152,0.0135357},
{0.0073010,-0.0012666,0.0534512},
{0.0181655,-0.0105526,0.0487221},
{0.0141627,-0.0266715,0.0582993},
{-0.0112634,-0.0403236,0.0245324},
{-0.0173614,-0.0084447,-0.0024734},
{0.0154472,-0.0932282,0.0265164},
{-0.0065938,-0.0064693,-0.0183986},
{0.0038879,0.0158780,-0.0143342},
{-0.0088278,-0.0372159,0.0154774},
{-0.0140512,0.0116126,-0.0100712},
{-0.0190710,0.0055846,-0.0016073},
{-0.0086197,-0.0788841,0.0382538},
{0.0110390,-0.0196027,-0.0009177},
{0.0118808,-0.0511222,0.0555058},
{0.0082476,-0.0207843,-0.0086948},
{0.0095581,-0.0156530,-0.0133261},
{0.0040928,-0.0668095,0.0183294},
{0.0085217,-0.0569921,0.0207675},
{0.0011647,-0.0854396,0.0194164},
{-0.0173370,-0.0630382,0.0292101},
{-0.0057636,-0.0015281,0.0504681},
{-0.0124975,-0.0345923,0.0575661},
{0.0171143,-0.0194922,0.0523662},
{0.0143186,-0.0864498,0.0467007},
{0.0179380,-0.0269641,0.0339437},
{0.0079802,-0.0357748,0.0643824},
{-0.0162532,-0.0036501,0.0096114},
{0.0137827,-0.0181297,0.0582691},
{0.0148027,-0.0688212,0.0315677},
{-0.0145396,-0.0563337,0.0218089},
{-0.0134436,-0.0374875,0.0509437},
{0.0132930,-0.0467787,0.0295991},
{-0.0074809,-0.0435252,0.0623340},
{0.0063526,-0.0087001,-0.0182546},
{0.0039611,-0.0173353,-0.0146800},
{-0.0137873,-0.0121210,0.0498663},
{-0.0100008,-0.0503134,0.0561237},
{0.0167100,-0.0607578,0.0457914},

Biology Open • Supplementary information

{-0.0161804,0.0125530,0.0015196},
{-0.0132363,0.0103097,0.0112899},
{-0.0018254,-0.0764503,0.0531147},
{0.0127325,-0.0104417,0.0330533},
{0.0133110,-0.0039395,0.0534303},
{0.0146387,-0.0064575,0.0418712},
{-0.0122665,-0.0151291,-0.0083414},
{0.0054977,-0.0246934,0.0022823},
{0.0003542,-0.0551537,0.0165338},
{0.0192266,0.0035210,-0.0073694},
{0.0164797,0.0018269,-0.0127782},
{0.0122905,-0.0086772,-0.0153257},
{-0.0126137,0.0015441,-0.0149943},
{-0.0140846,-0.0067244,-0.0128484},
{0.0170015,0.0148859,0.0031122},
{0.0101588,0.0214644,-0.0022277},
{0.0086714,0.0156150,-0.0128410},
{0.0077486,-0.0254208,0.0626589},
{-0.0006832,-0.0097105,0.0618004},
{-0.0001594,-0.0023532,0.0556958},
{0.0051741,-0.0154361,0.0632219},
{0.0080791,0.0082700,0.0160808},
{0.0118579,-0.0195820,0.0152489},
{0.0037901,-0.0284488,0.0642877},
{-0.0087812,-0.0194449,0.0237477},
{-0.0098419,-0.0196527,0.0118547},
{-0.0062380,-0.0112550,0.0265173},
{-0.0145216,-0.0570814,0.0346904},
{-0.0109413,-0.0690052,0.0401535},
{-0.0066783,-0.0327527,0.0091793},
{0.0199615,-0.0208280,0.0453152},
{0.0117954,-0.0095284,0.0271387},
{0.0041682,-0.0010653,0.0237763},
{-0.0034562,-0.0856111,0.0516973},
{-0.0023192,0.0247036,-0.0032037},
{-0.0011023,-0.0148876,0.0383351},
{-0.0105467,-0.0161794,0.0024137},
{0.0125021,-0.0324696,0.0251262},
{0.0099618,-0.0306237,0.0167887},
{-0.0106147,-0.0078367,0.0516036},
{0.0180725,-0.0373654,0.0470048},
{-0.0116474,-0.0603849,0.0407162},
{-0.0072082,-0.0912678,0.0369130},
{0.0041806,-0.0059214,0.0302316},
{-0.0028834,-0.0051974,-0.0195638},
{-0.0121699,-0.0392144,0.0571341},

Biology Open • Supplementary information

{-0.0029042,0.0100457,-0.0165387},
{-0.0041701,0.0187292,-0.0122138},
{0.0179586,-0.0968812,0.0355867},
{0.0035635,-0.0835491,0.0186498},
{0.0158856,-0.0999585,0.0437476},
{0.0092524,0.0216721,0.0052725},
{0.0113334,0.0103314,0.0142824},
{0.0010602,0.0170224,-0.0140160},
{0.0155144,-0.0132005,-0.0086692},
{0.0182706,-0.0086343,-0.0074344},
{-0.0083949,-0.0275148,0.0298135},
{-0.0105213,-0.0391268,0.0315141},
{-0.0129322,-0.0698857,0.0252094},
{-0.0013661,0.0030150,0.0196893},
{0.0040076,0.0082959,0.0169864},
{-0.0024271,-0.0032322,0.0251536},
{0.0002477,-0.0349208,0.0638001},
{0.0066560,-0.0492274,0.0587160},
{-0.0112726,-0.0225085,0.0615070},
{-0.0112465,-0.0298495,0.0607528},
{0.0087076,0.0229681,0.0025957},
{0.0164711,0.0160430,0.0013773},
{-0.0068626,-0.0123772,0.0626229},
{0.0146631,-0.0602864,0.0336928},
{-0.0119502,-0.0539434,0.0471005},
{-0.0144535,-0.0572065,0.0215748},
{0.0105510,-0.0956220,0.0496059},
{-0.0094330,-0.0286301,0.0173568},
{-0.0097229,-0.0174344,0.0166887},
{-0.0042603,0.0181416,0.0123852},
{0.0008608,-0.0773008,0.0548407},
{0.0056665,-0.0712677,0.0550092},
{-0.0021731,-0.0709143,0.0545142},
{0.0159467,-0.0835650,0.0304491},
{-0.0119843,-0.0207475,0.0479854},
{0.0102653,-0.0281899,0.0168951},
{0.0049584,-0.0595354,0.0574294},
{0.0079891,-0.0397885,0.0610579},
{0.0091768,-0.0955668,0.0194551},
{-0.0065204,-0.0599661,0.0557600},
{0.0172438,-0.0485188,0.0430965},
{-0.0114645,-0.0484989,0.0207280},
{0.0203797,-0.0233606,0.0366626},
{-0.0038750,-0.0690447,0.0198841},
{0.0160113,-0.0022547,0.0125073},
{0.0059362,0.0061174,0.0171327},

Biology Open • Supplementary information

{0.0008808,-0.0367129,0.0636847},
{-0.0142550,0.0155273,-0.0049687},
{0.0102008,0.0177361,0.0099500},
{-0.0102621,0.0180963,0.0079838},
{-0.0178953,-0.0062104,-0.0050552},
{-0.0180019,0.0005744,-0.0072515},
{-0.0047130,-0.0197588,0.0649762},
{-0.0126451,-0.0387708,0.0426025},
{0.0053727,-0.0529024,0.0586883},
{0.0119580,-0.0469838,0.0564921},
{-0.0084061,-0.0770706,0.0248460},
{0.0121476,0.0079972,0.0147081},
{0.0175340,-0.0120650,-0.0010980},
{-0.0166603,-0.0090650,0.0048941},
{-0.0122857,-0.0634135,0.0377523},
{-0.0070328,-0.0189800,-0.0036334},
{-0.0124404,-0.0310379,0.0456643},
{0.0044747,-0.0843269,0.0533249},
{0.0153625,-0.0562102,0.0502720},
{-0.0016688,-0.0392424,0.0106897},
{0.0060084,-0.0360369,0.0127416},
{0.0180551,-0.0078452,0.0056031},
{0.0174335,-0.0916608,0.0404228},
{-0.0087836,-0.0213053,0.0243364},
{-0.0008452,0.0145810,0.0155751},
{0.0071209,-0.0768032,0.0205137},
{0.0084578,-0.0701841,0.0543850},
{0.0144465,-0.0145867,-0.0091297},
{0.0005347,-0.0102401,0.0622610},
{-0.0068770,-0.0165886,-0.0136885},
{0.0152873,-0.0792910,0.0454090},
{0.0073944,-0.0207119,-0.0099912},
{-0.0102085,-0.0724978,0.0224313},
{0.0128022,-0.0442134,0.0280303},
{0.0149223,-0.0048095,0.0520261},
{0.0045623,-0.0085878,0.0379233},
{-0.0029198,-0.0694287,0.0550525},
{-0.0123125,-0.0669188,0.0367648},
{-0.0138995,-0.0128401,0.0520607},
{-0.0075579,0.0137868,-0.0141141},
{-0.0165100,0.0065011,0.0078509},
{0.0089889,-0.0765014,0.0213862},
{0.0023981,-0.0436731,0.0617446},
{-0.0129841,-0.0243444,0.0529595},
{0.0061922,-0.0068855,-0.0189302},
{0.0148125,-0.0317084,0.0544382},

Biology Open • Supplementary information

{0.0115032,-0.0168011,0.0597357},
{0.0100189,0.0023200,-0.0174362},
{0.0131795,-0.0090810,0.0165585},
{-0.0124417,0.0123833,0.0112618},
{0.0099599,-0.0587527,0.0554512},
{-0.0052552,-0.0426661,0.0631276},
{0.0186932,-0.0322848,0.0482121},
{0.0152360,-0.0146955,-0.0067530},
{-0.0143460,-0.0557473,0.0351732},
{-0.0072603,-0.0818540,0.0436075},
{0.0030761,-0.0584143,0.0581415},
{-0.0042441,-0.0459634,0.0135223},
{0.0107068,0.0118619,-0.0140981},
{-0.0020605,-0.0239721,-0.0008146},
{-0.0088137,-0.0813086,0.0291654},
{-0.0099301,-0.0038839,-0.0169551},
{-0.0028036,0.0240326,-0.0045104},
{-0.0092178,-0.0240766,0.0128033},
{-0.0103271,-0.0714252,0.0222600},
{-0.0057177,-0.0828397,0.0239660},
{0.0009164,0.0131415,-0.0161286},
{0.0190809,-0.0288029,0.0474705},
{-0.0056686,-0.0642799,0.0192312},
{0.0169530,-0.0402343,0.0383643},
{0.0169285,-0.0341056,0.0341804},
{-0.0138086,-0.0132509,-0.0009473},
{0.0127625,0.0155259,0.0098094},
{-0.0128746,-0.0120850,0.0067374},
{-0.0102887,-0.0389788,0.0347788},
{0.0172724,-0.0971542,0.0412840},
{0.0170064,-0.0114398,0.0541931},
{0.0180510,-0.0172250,0.0502058},
{0.0013100,-0.0293071,0.0048870},
{0.0103815,-0.0258437,0.0611410},
{-0.0026838,-0.0105533,0.0305564},
{-0.0185482,-0.0046936,-0.0016918},
{0.0026533,-0.0232363,-0.0037075},
{-0.0093180,-0.0786353,0.0359631},
{-0.0122563,-0.0303745,0.0592847},
{0.0172144,0.0057349,-0.0104731},
{-0.0142115,-0.0687978,0.0293141},
{-0.0146559,-0.0046392,0.0124477},
{0.0115406,-0.0088781,0.0263225},
{-0.0129224,-0.0473253,0.0311781},
{0.0144782,-0.0790358,0.0280098},
{0.0007403,0.0037205,-0.0190816},

Biology Open • Supplementary information

{0.0162429,-0.0855618,0.0305619},
{0.0066794,0.0070442,0.0164161},
{-0.0137445,0.0024944,0.0126043},
{0.0056290,-0.0473713,0.0161583},
{-0.0062301,-0.0209969,0.0331165},
{0.0197730,-0.0297469,0.0418314},
{-0.0124178,-0.0690695,0.0223828},
{0.0200669,0.0056425,-0.0029645},
{-0.0133516,-0.0692525,0.0272727},
{0.0108397,-0.0246788,0.0108730},
{0.0189941,-0.0128990,0.0446259},
{0.0083357,0.0021462,-0.0183580},
{0.0141979,-0.0091605,0.0133078},
{-0.0109148,-0.0700977,0.0395201},
{0.0004459,0.0227104,0.0090609},
{0.0027355,0.0036432,-0.0193944},
{0.0363299,-0.4445459,0.0207249},
{0.0049235,0.0225034,-0.0076788},
{-0.0131553,-0.0496189,0.0368063},
{-0.0305724,-0.4400438,-0.0265564},
{0.0022567,0.0254586,-0.0000315},
{0.0139966,-0.0270486,0.0262459},
{-0.0137482,-0.0012128,-0.0143426},
{0.0161220,-0.0341557,0.0523172},
{-0.0142716,0.0148245,0.0046818},
{0.0168079,-0.0441864,0.0395368},
{0.0150004,-0.0492943,0.0339125},
{-0.0129260,-0.0388823,0.0451951},
{0.0168776,-0.0624354,0.0434970},
{-0.0162911,-0.0542556,0.0271446},
{0.0029765,-0.0619856,0.0574014},
{0.0010592,-0.0578242,0.0171427},
{0.0208532,-0.0235613,0.0434832},
{0.0076991,-0.0860993,0.0520324},
{-0.0057926,-0.0934750,0.0275804},
{-0.0184811,-0.0024259,0.0030148},
{0.0140084,-0.0151028,0.0194893},
{0.0134732,-0.0750454,0.0271181},
{-0.0090447,-0.0278212,0.0271597},
{-0.0189749,-0.4511705,0.0352395},
{-0.0084198,-0.0628317,0.0514965},
{-0.0111412,-0.4450708,-0.0101447},
{0.0103837,-0.0487127,0.0221042},
{0.0105533,0.0163501,-0.0110462},
{-0.0236922,-0.4413982,0.0285139},
{0.0152105,-0.0598440,0.0345623},

Biology Open • Supplementary information

{0.0002901,-0.0558033,0.0590675},
{-0.0106771,0.0203744,0.0028067},
{-0.0023985,-0.0188035,0.0648384},
{-0.0070908,-0.0082984,0.0237958},
{0.0188364,-0.0116045,0.0416510},
{0.0144252,-0.0275798,0.0564280},
{-0.0137357,-0.0122822,0.0513385},
{0.0192522,0.0092744,-0.0027691},
{-0.0097379,-0.0384203,0.0176847},
{-0.0003272,-0.0159039,-0.0162156},
{-0.0104668,-0.0678246,0.0435663},
{-0.0087464,0.0192836,0.0079101},
{0.0094814,0.0215558,-0.0029601},
{-0.0130107,-0.0304474,0.0562217},
{-0.0058088,-0.4419482,0.0399441},
{-0.0130445,-0.4479569,-0.0374383},
{-0.0129721,-0.4478968,-0.0366645},
{-0.0128998,-0.4478367,-0.0358906},
{-0.0128274,-0.4477767,-0.0351168},
{-0.0127550,-0.4477166,-0.0343430},
{-0.0126827,-0.4476565,-0.0335692},
{-0.0126103,-0.4475964,-0.0327953},
{-0.0125380,-0.4475363,-0.0320215},
{-0.0124656,-0.4474762,-0.0312477},
{-0.0123933,-0.4474161,-0.0304739},
{-0.0123209,-0.4473560,-0.0297000},
{-0.0122485,-0.4472960,-0.0289262},
{-0.0121762,-0.4472359,-0.0281524},
{-0.0121038,-0.4471758,-0.0273786},
{-0.0120315,-0.4471157,-0.0266048},
{-0.0119591,-0.4470556,-0.0258309},
{-0.0118868,-0.4469955,-0.0250571},
{-0.0118144,-0.4469354,-0.0242833},
{-0.0117420,-0.4468753,-0.0235095},
{-0.0116697,-0.4468153,-0.0227356},
{-0.0115973,-0.4467552,-0.0219618},
{-0.0115250,-0.4466951,-0.0211880},
{-0.0114526,-0.4466350,-0.0204142},
{-0.0113803,-0.4465749,-0.0196403},
{-0.0113079,-0.4465148,-0.0188665},
{-0.0112356,-0.4464547,-0.0180927},
{-0.0111632,-0.4463947,-0.0173189},
{-0.0110908,-0.4463346,-0.0165450},
{-0.0110185,-0.4462745,-0.0157712},
{-0.0109461,-0.4462144,-0.0149974},
{-0.0108738,-0.4461543,-0.0142236},

Biology Open • Supplementary information

{-0.0108014,-0.4460942,-0.0134497},
{-0.0107291,-0.4460341,-0.0126759},
{-0.0106567,-0.4459740,-0.0119021},
{-0.0105843,-0.4459140,-0.0111283},
{-0.0105120,-0.4458539,-0.0103545},
{-0.0104396,-0.4457938,-0.0095806},
{-0.0103673,-0.4457337,-0.0088068},
{-0.0102949,-0.4456736,-0.0080330},
{-0.0102226,-0.4456135,-0.0072592},
{-0.0101502,-0.4455534,-0.0064853},
{-0.0100778,-0.4454933,-0.0057115},
{-0.0100055,-0.4454333,-0.0049377},
{-0.0099331,-0.4453732,-0.0041639},
{-0.0098608,-0.4453131,-0.0033900},
{-0.0097884,-0.4452530,-0.0026162},
{-0.0097161,-0.4451929,-0.0018424},
{-0.0096437,-0.4451328,-0.0010686},
{-0.0095713,-0.4450727,-0.0002947},
{-0.0094990,-0.4450126,0.0004791},
{-0.0094266,-0.4449526,0.0012529},
{-0.0093543,-0.4448925,0.0020267},
{-0.0092819,-0.4448324,0.0028005},
{-0.0092096,-0.4447723,0.0035744},
{-0.0091372,-0.4447122,0.0043482},
{-0.0090649,-0.4446521,0.0051220},
{-0.0089925,-0.4445920,0.0058958},
{-0.0089201,-0.4445320,0.0066697},
{-0.0088478,-0.4444719,0.0074435},
{-0.0087754,-0.4444118,0.0082173},
{-0.0087031,-0.4443517,0.0089911},
{-0.0086307,-0.4442916,0.0097650},
{-0.0085584,-0.4442315,0.0105388},
{-0.0084860,-0.4441714,0.0113126},
{-0.0084136,-0.4441113,0.0120864},
{-0.0083413,-0.4440513,0.0128603},
{-0.0082689,-0.4439912,0.0136341},
{-0.0081966,-0.4439311,0.0144079},
{-0.0081242,-0.4438710,0.0151817},
{-0.0080519,-0.4438109,0.0159555},
{-0.0079795,-0.4437508,0.0167294},
{-0.0079071,-0.4436907,0.0175032},
{-0.0078348,-0.4436306,0.0182770},
{-0.0077624,-0.4435706,0.0190508},
{-0.0076901,-0.4435105,0.0198247},
{-0.0076177,-0.4434504,0.0205985},
{-0.0075454,-0.4433903,0.0213723},

```
                    {-0.0074730,-0.4433302,0.0221461},
                    {-0.0074006,-0.4432701,0.0229200},
                    {-0.0073283,-0.4432100,0.0236938},
                    {-0.0072559,-0.4431500,0.0244676},
                    {-0.0071836,-0.4430899,0.0252414},
                    {-0.0071112,-0.4430298,0.0260153},
                    {-0.0070389,-0.4429697,0.0267891},
                    {-0.0069665,-0.4429096,0.0275629},
                    {-0.0068942,-0.4428495,0.0283367},
                    {-0.0068218,-0.4427894,0.0291105},
                    {-0.0067494,-0.4427293,0.0298844},
                    {-0.0066771,-0.4426693,0.0306582},
                    {-0.0066047,-0.4426092,0.0314320},
                    {-0.0065324,-0.4425491,0.0322058},
                    {-0.0064600,-0.4424890,0.0329797},
                    {-0.0063877,-0.4424289,0.0337535},
                    {-0.0063153,-0.4423688,0.0345273},
                    {-0.0062429,-0.4423087,0.0353011},
                    {-0.0061706,-0.4422486,0.0360750},
                    {-0.0060982,-0.4421886,0.0368488},
                    {-0.0060259,-0.4421285,0.0376226},
                    {-0.0059535,-0.4420684,0.0383964},
                    {-0.0058812,-0.4420083,0.0391703}
                    };
            BoundingBoxOnOff = Off;
        };
        AnyFunTransform3DIdentity ScaleFunction = {
            PreTransforms = {&.RBFTransform};
};
};

};
};
```

Biology Open • Supplementary information

ScalingFunctionTLEMLucyFemur_Mirrored_2014019

```
AnyFolder ScalingFunctionTLEMLucyFemur_Mirrored = {
  AnyFolder Left = {
    AnyFolder Thigh = {
      AnyMatrix AMirroring = {
        {1,0,0},
        {0,1,0},
        {0,0,-1}
      };
           AnyFunTransform3DRBF RBFTransform = {
                      RBFDef.Type = RBF_ThinPlate;
                      PolynomDegree = 1;
                      Points0 = ({
                                 {0.0000000,0.0000000,0.0000000},
                                 {-0.0000000,-0.3616821,0.0000000},
                                 {-0.0097563,-0.3678799,0.0012967},
                                 {-0.0000000,-0.3660632,0.0408203},
                                 {-0.0000000,-0.3573010,-0.0408203},
                                 {0.0161460,-0.0072838,0.0601290},
                                 {0.0220217,-0.0203698,0.0463848},
                                 {0.0123977,-0.0241932,0.0668573},
                                 {0.0006898,0.0018121,0.0538181},
                                 {0.0122809,-0.0068668,0.0414535},
                                 {-0.0058991,-0.0138188,0.0648412},
                                 {0.0211469,-0.0380855,0.0538111},
                                 {0.0172133,-0.0317342,0.0328381},
                                 {0.0177311,-0.0131946,0.0256176},
                                 {-0.0110079,-0.0308867,0.0632370},
                                 {0.0039435,-0.0395977,0.0638271},
                                 {-0.0041683,-0.0187472,0.0413214},
                                 {-0.0177999,-0.0021535,0.0496084},
                                 {0.0001603,-0.0053126,0.0279593},
                                 {-0.0211600,-0.0216243,0.0497147},
                                 {0.0096163,-0.0568663,0.0545719},
                                 {0.0162112,-0.0515013,0.0395230},
                                 {0.0093533,-0.0433265,0.0187387},
                                 {0.0119398,-0.0238469,0.0118403},
                                 {0.0210707,-0.0048848,0.0065878},
                                 {0.0125013,0.0064094,0.0177702},
                                 {-0.0191523,-0.0356053,0.0382429},
                                 {-0.0115821,-0.0474937,0.0537648},
                                 {-0.0100073,-0.0236371,0.0267322},
                                 {-0.0078925,0.0107098,0.0182833},
                                 {-0.0134217,-0.0055356,0.0182000},
                                 {-0.0090061,-0.0665700,0.0475484},
```

Biology Open • Supplementary information

{0.0071095,-0.0761976,0.0520035},
{0.0182119,-0.0704149,0.0386546},
{0.0073764,-0.0609481,0.0221006},
{-0.0122984,-0.0439585,0.0198428},
{-0.0032253,-0.0306792,0.0110995},
{-0.0012975,-0.0198451,-0.0052592},
{0.0140405,-0.0161125,-0.0071649},
{0.0189222,-0.0014898,-0.0127226},
{0.0181642,0.0132812,-0.0011050},
{0.0045119,0.0200001,0.0091189},
{-0.0176761,-0.0541983,0.0348587},
{-0.0136548,-0.0142015,0.0054311},
{-0.0135510,0.0182940,0.0017175},
{-0.0223865,0.0023399,0.0028018},
{-0.0086455,-0.0738247,0.0283843},
{-0.0050356,-0.0877749,0.0442195},
{0.0160135,-0.0919319,0.0465120},
{0.0128290,-0.0785810,0.0232431},
{-0.0168328,-0.0616327,0.0155917},
{-0.0172369,-0.0094567,-0.0119502},
{0.0002090,-0.0093641,-0.0209779},
{0.0064827,0.0081538,-0.0198453},
{0.0010777,0.0210422,-0.0084721},
{-0.0129485,0.0085451,-0.0162212},
{-0.0002322,-0.0924033,0.0245644},
{-0.0028175,-0.1052263,0.0418294},
{0.0172322,-0.1111444,0.0445760},
{0.0203259,-0.0957409,0.0271449},
{0.0057102,-0.1088933,0.0195113},
{-0.0010852,-0.1230776,0.0363132},
{0.0182727,-0.1303312,0.0422884},
{0.0242891,-0.1161703,0.0265054},
{0.0094531,-0.1268584,0.0162294},
{0.0014244,-0.1413293,0.0331653},
{0.0207271,-0.1496427,0.0394157},
{0.0267562,-0.1360142,0.0236798},
{0.0113839,-0.1461651,0.0136024},
{0.0030845,-0.1595094,0.0297156},
{0.0209528,-0.1691386,0.0369775},
{0.0283958,-0.1540209,0.0213795},
{0.0129499,-0.1649012,0.0113589},
{0.0016490,-0.1783108,0.0216287},
{0.0147105,-0.1880678,0.0328614},
{0.0298491,-0.1859865,0.0248551},
{0.0286645,-0.1714377,0.0169488},
{0.0165897,-0.1861006,0.0082595},

Biology Open • Supplementary information

{0.0014000,-0.1973476,0.0183740},
{0.0114301,-0.2109792,0.0286157},
{0.0267422,-0.2037622,0.0296951},
{0.0288630,-0.2018335,0.0130127},
{0.0145471,-0.2083505,0.0062833},
{0.0011300,-0.2198071,0.0155602},
{0.0126610,-0.2321235,0.0275354},
{0.0275647,-0.2219268,0.0265185},
{0.0286645,-0.2230253,0.0098701},
{0.0130434,-0.2304822,0.0039177},
{0.0022984,-0.2409585,0.0149650},
{0.0101315,-0.2534740,0.0252125},
{0.0279485,-0.2428555,0.0234276},
{0.0276684,-0.2421559,0.0058691},
{0.0101250,-0.2529527,0.0018983},
{0.0021376,-0.2667831,0.0107073},
{0.0114309,-0.2734905,0.0249960},
{0.0273923,-0.2634953,0.0216324},
{0.0278429,-0.2592546,0.0037692},
{0.0159101,-0.2712985,-0.0033988},
{0.0036708,-0.2863280,0.0004644},
{0.0023115,-0.2884756,0.0187554},
{0.0231385,-0.2879051,0.0224853},
{0.0304009,-0.2799973,0.0083549},
{0.0238189,-0.2917246,-0.0052462},
{0.0064904,-0.3027687,-0.0070587},
{-0.0003492,-0.3064690,0.0093014},
{0.0122179,-0.3045269,0.0243511},
{0.0301837,-0.3030127,0.0114462},
{0.0234182,-0.3107336,-0.0085198},
{0.0046893,-0.3214995,-0.0123881},
{-0.0022352,-0.3264905,0.0043340},
{0.0016841,-0.3202756,0.0217839},
{0.0229722,-0.3196887,0.0215083},
{0.0292438,-0.3228042,0.0051430},
{0.0221641,-0.3296190,-0.0132361},
{0.0100459,-0.3417597,-0.0244560},
{-0.0053009,-0.3375737,-0.0137705},
{-0.0064432,-0.3487307,0.0028676},
{-0.0059346,-0.3407801,0.0202938},
{0.0105130,-0.3365976,0.0264175},
{0.0282695,-0.3386249,0.0180307},
{0.0270402,-0.3431844,-0.0001487},
{0.0257040,-0.3506950,-0.0181851},
{0.0178948,-0.3636114,-0.0302838},
{0.0043149,-0.3544109,-0.0381949},

Biology Open • Supplementary information

{-0.0095210,-0.3461482,-0.0295031},
{-0.0164526,-0.3542895,-0.0126666},
{-0.0060896,-0.3690127,-0.0033061},
{-0.0176523,-0.3612686,0.0110109},
{-0.0153310,-0.3546943,0.0299634},
{0.0021329,-0.3508021,0.0350763},
{0.0210690,-0.3548628,0.0279835},
{0.0356594,-0.3558162,0.0159356},
{0.0275718,-0.3617450,-0.0018607},
{0.0298503,-0.3741530,-0.0163721},
{0.0175320,-0.3855300,-0.0277515},
{0.0025604,-0.3740099,-0.0397137},
{-0.0136727,-0.3624016,-0.0402102},
{-0.0277647,-0.3567472,-0.0284947},
{-0.0275446,-0.3699630,-0.0120829},
{-0.0175846,-0.3859355,-0.0177922},
{0.0001572,-0.3856873,-0.0118050},
{0.0030262,-0.3834856,0.0075553},
{-0.0144114,-0.3810887,0.0140304},
{-0.0247706,-0.3701239,0.0285134},
{-0.0044804,-0.3677201,0.0404632},
{0.0133183,-0.3712274,0.0334208},
{0.0324861,-0.3723417,0.0245668},
{0.0271451,-0.3766027,0.0072441},
{0.0167985,-0.3822816,-0.0065349},
{-0.0001454,-0.3910823,-0.0303010},
{-0.0144998,-0.3834154,-0.0384137},
{-0.0301321,-0.3750841,-0.0321336},
{0.0196491,-0.3864804,0.0210853},
{0.0015726,-0.3888034,0.0276569},
{-0.0146652,-0.3847848,0.0336913},
{-0.0014588,-0.0619951,0.0219778},
{-0.0118435,-0.0550626,0.0139639},
{0.0129812,-0.0349966,0.0673128},
{-0.0162907,-0.0105666,0.0597739},
{-0.0012885,-0.0520816,0.0562989},
{-0.0090599,-0.0631298,0.0172224},
{-0.0000222,-0.0771663,0.0231897},
{-0.0068148,0.0023885,-0.0160086},
{-0.0180544,-0.0425409,0.0446585},
{0.0020525,-0.0249904,0.0691549},
{-0.0157097,-0.0662315,0.0238393},
{-0.0010518,-0.0510686,0.0199353},
{-0.0208874,-0.0152865,0.0500034},
{0.0168316,-0.0280857,0.0666680},
{-0.0187392,-0.0282333,0.0403282},

Biology Open • Supplementary information

{0.0191622,-0.0351633,0.0622707},
{0.0001216,-0.0706289,0.0524049},
{-0.0169862,-0.0399062,0.0311827},
{-0.0185737,-0.0533066,0.0151224},
{-0.0219058,-0.0528723,0.0222263},
{-0.0028271,-0.0391620,0.0148452},
{0.0117900,-0.0553425,0.0235348},
{0.0219835,-0.0106400,0.0396338},
{-0.0025366,-0.0401046,0.0625273},
{0.0141017,-0.0018767,0.0198108},
{-0.0078770,-0.0473929,0.0173542},
{-0.0065351,-0.0766431,0.0470258},
{0.0065585,0.0145593,0.0161638},
{-0.0118588,0.0172875,-0.0085064},
{-0.0074087,-0.0627500,0.0506700},
{0.0071940,-0.0780068,0.0213362},
{0.0126736,-0.0708176,0.0244108},
{0.0074694,-0.0436450,0.0604196},
{0.0154186,-0.0139471,0.0064312},
{-0.0053535,-0.0213210,0.0040094},
{-0.0088733,0.0002018,0.0205402},
{-0.0112471,-0.0542608,0.0500266},
{-0.0061625,-0.0248673,0.0653244},
{0.0093695,-0.0065826,0.0614157},
{0.0138015,-0.0467322,0.0284333},
{-0.0020999,-0.0186885,-0.0116392},
{-0.0185640,-0.0225591,0.0566779},
{0.0175798,0.0124360,-0.0068467},
{-0.0039962,0.0217127,0.0053055},
{0.0014975,-0.0784591,0.0514056},
{-0.0172997,-0.0386971,0.0525252},
{0.0196540,-0.0268271,0.0416806},
{0.0135296,-0.0406504,0.0236882},
{0.0182471,0.0095959,0.0087906},
{0.0218667,0.0016804,0.0058793},
{0.0224975,-0.0022652,-0.0022994},
{0.0166750,-0.0191391,0.0236436},
{0.0119785,-0.0328723,0.0195376},
{0.0187631,-0.0133469,0.0308741},
{-0.0187543,-0.0461463,0.0257491},
{0.0039834,-0.0373928,0.0683089},
{0.0157029,-0.0423169,0.0385226},
{0.0164003,-0.0087069,0.0131181},
{0.0008253,0.0033370,-0.0212576},
{0.0101707,-0.0009724,-0.0205688},
{0.0102948,-0.0642683,0.0524532},

Biology Open • Supplementary information

{0.0152217,-0.0557944,0.0508978},
{0.0172973,-0.0565707,0.0419468},
{0.0167723,-0.0635334,0.0369834},
{-0.0047397,-0.0695325,0.0241398},
{-0.0152913,0.0035778,0.0161461},
{0.0091697,0.0009340,0.0575160},
{0.0196686,-0.0089533,0.0500006},
{0.0183998,-0.0212943,0.0622968},
{-0.0114340,-0.0316810,0.0250940},
{-0.0206442,-0.0092599,-0.0028425},
{0.0166868,-0.0784295,0.0276925},
{-0.0069678,-0.0054521,-0.0207011},
{0.0049490,0.0151109,-0.0160093},
{-0.0084025,-0.0318937,0.0162788},
{-0.0173679,0.0095594,-0.0108509},
{-0.0218912,0.0063457,-0.0020698},
{-0.0106547,-0.0668750,0.0397749},
{0.0136098,-0.0170666,-0.0018890},
{0.0141599,-0.0441405,0.0587594},
{0.0093255,-0.0183218,-0.0083008},
{0.0106367,-0.0128276,-0.0154269},
{0.0037350,-0.0549365,0.0209786},
{0.0087662,-0.0472902,0.0195496},
{0.0016839,-0.0708213,0.0221325},
{-0.0202977,-0.0530540,0.0301270},
{-0.0068584,0.0021781,0.0530002},
{-0.0144817,-0.0297218,0.0605833},
{0.0197015,-0.0165217,0.0560654},
{0.0156609,-0.0705363,0.0477736},
{0.0207876,-0.0221433,0.0330733},
{0.0092324,-0.0300767,0.0713160},
{-0.0203392,-0.0034488,0.0097332},
{0.0156978,-0.0143438,0.0629053},
{0.0143267,-0.0563519,0.0317489},
{-0.0161685,-0.0465391,0.0188591},
{-0.0196397,-0.0309875,0.0546496},
{0.0154804,-0.0380422,0.0305433},
{-0.0077774,-0.0351303,0.0637765},
{0.0063980,-0.0086419,-0.0201654},
{0.0030146,-0.0151094,-0.0165974},
{-0.0210080,-0.0096030,0.0507855},
{-0.0105684,-0.0411377,0.0586146},
{0.0180619,-0.0498960,0.0469835},
{-0.0192318,0.0116108,0.0024070},
{-0.0165651,0.0083622,0.0126870},
{-0.0014333,-0.0627138,0.0534746},

Biology Open • Supplementary information

{0.0145127,-0.0080178,0.0353667},
{0.0148374,-0.0012228,0.0566181},
{0.0153834,-0.0062427,0.0444727},
{-0.0155164,-0.0132103,-0.0096398},
{0.0068685,-0.0221022,0.0011547},
{0.0003332,-0.0458500,0.0183716},
{0.0205582,0.0040956,-0.0082969},
{0.0175752,0.0009340,-0.0148407},
{0.0142357,-0.0063623,-0.0169332},
{-0.0147285,0.0023412,-0.0164678},
{-0.0158781,-0.0064451,-0.0151346},
{0.0175951,0.0133456,0.0037559},
{0.0117660,0.0189899,-0.0030690},
{0.0096040,0.0151827,-0.0137450},
{0.0096565,-0.0215194,0.0662734},
{-0.0006793,-0.0074494,0.0631218},
{0.0007819,-0.0004519,0.0600781},
{0.0065377,-0.0125433,0.0654199},
{0.0090461,0.0073096,0.0188711},
{0.0151418,-0.0168302,0.0146465},
{0.0025166,-0.0231863,0.0688894},
{-0.0097891,-0.0152077,0.0253086},
{-0.0109149,-0.0177935,0.0119825},
{-0.0072079,-0.0099373,0.0272725},
{-0.0183727,-0.0467429,0.0356276},
{-0.0126871,-0.0584555,0.0428141},
{-0.0057054,-0.0273042,0.0103554},
{0.0215142,-0.0159693,0.0474531},
{0.0128492,-0.0070016,0.0287645},
{0.0052134,-0.0017047,0.0247463},
{-0.0024284,-0.0707498,0.0513049},
{-0.0036140,0.0220593,-0.0040484},
{-0.0002405,-0.0121253,0.0390050},
{-0.0119811,-0.0156737,0.0015445},
{0.0142082,-0.0279792,0.0246553},
{0.0122244,-0.0262013,0.0169829},
{-0.0146264,-0.0038855,0.0549384},
{0.0207450,-0.0325771,0.0496412},
{-0.0155040,-0.0497560,0.0437087},
{-0.0081119,-0.0756450,0.0387365},
{0.0039684,-0.0062047,0.0313900},
{-0.0023726,-0.0038892,-0.0220521},
{-0.0136977,-0.0339839,0.0602619},
{-0.0035399,0.0089081,-0.0188894},
{-0.0049820,0.0176571,-0.0132254},
{0.0193902,-0.0803191,0.0360429},

{0.0040648,-0.0697109,0.0218205},
{0.0170470,-0.0818610,0.0456892},
{0.0104175,0.0188635,0.0064965},
{0.0135206,0.0092549,0.0158079},
{0.0023933,0.0160498,-0.0157404},
{0.0164753,-0.0118197,-0.0096995},
{0.0202584,-0.0066858,-0.0082383},
{-0.0098401,-0.0226001,0.0314028},
{-0.0130937,-0.0309622,0.0320764},
{-0.0201510,-0.0613297,0.0222264},
{-0.0010389,0.0036055,0.0209003},
{0.0042025,0.0073392,0.0199393},
{-0.0021630,-0.0038531,0.0251898},
{-0.0007845,-0.0293574,0.0675788},
{0.0070027,-0.0406327,0.0624565},
{-0.0124363,-0.0183001,0.0627966},
{-0.0120485,-0.0256613,0.0628493},
{0.0101200,0.0197695,0.0039334},
{0.0167636,0.0148266,0.0014155},
{-0.0074981,-0.0106930,0.0632797},
{0.0142533,-0.0503445,0.0351954},
{-0.0146107,-0.0460953,0.0506120},
{-0.0161117,-0.0471301,0.0178900},
{0.0112149,-0.0792793,0.0501460},
{-0.0099060,-0.0230794,0.0186951},
{-0.0114543,-0.0153049,0.0183675},
{-0.0042882,0.0161879,0.0151423},
{0.0014582,-0.0638572,0.0541080},
{0.0048554,-0.0582603,0.0553815},
{-0.0017077,-0.0572302,0.0546635},
{0.0166090,-0.0697169,0.0323038},
{-0.0208812,-0.0164273,0.0474354},
{0.0130454,-0.0241864,0.0178294},
{0.0053123,-0.0483770,0.0582251},
{0.0112225,-0.0361429,0.0673300},
{0.0085729,-0.0771256,0.0216100},
{-0.0070783,-0.0488033,0.0560869},
{0.0182083,-0.0411024,0.0454236},
{-0.0119590,-0.0403809,0.0213546},
{0.0233409,-0.0185535,0.0366343},
{-0.0046347,-0.0571211,0.0208243},
{0.0183166,-0.0006966,0.0140116},
{0.0061913,0.0053512,0.0201003},
{-0.0002364,-0.0310828,0.0680351},
{-0.0170773,0.0139270,-0.0054987},
{0.0110805,0.0156034,0.0117010},

{-0.0124628,0.0164667,0.0094550},
{-0.0209014,-0.0073407,-0.0051969},
{-0.0210131,0.0010693,-0.0086891},
{-0.0041596,-0.0172332,0.0663041},
{-0.0216595,-0.0322172,0.0451540},
{0.0051908,-0.0426014,0.0613013},
{0.0150003,-0.0416136,0.0606257},
{-0.0135090,-0.0674234,0.0236425},
{0.0146416,0.0073691,0.0160052},
{0.0195615,-0.0112412,-0.0010333},
{-0.0198115,-0.0098244,0.0044735},
{-0.0157054,-0.0521236,0.0395039},
{-0.0081381,-0.0173108,-0.0046152},
{-0.0218324,-0.0262661,0.0471115},
{0.0051446,-0.0707257,0.0530555},
{0.0179359,-0.0471493,0.0528134},
{-0.0005591,-0.0317983,0.0113632},
{0.0071707,-0.0305943,0.0121186},
{0.0206239,-0.0061367,0.0066642},
{0.0187057,-0.0745868,0.0420207},
{-0.0095784,-0.0163113,0.0259524},
{-0.0012181,0.0127661,0.0186855},
{0.0059420,-0.0630691,0.0220087},
{0.0069408,-0.0573878,0.0552614},
{0.0150782,-0.0132884,-0.0101793},
{0.0005533,-0.0078574,0.0633775},
{-0.0088284,-0.0146399,-0.0147837},
{0.0167444,-0.0651105,0.0470347},
{0.0082666,-0.0181741,-0.0095133},
{-0.0175162,-0.0637444,0.0177069},
{0.0153795,-0.0360767,0.0287608},
{0.0162363,-0.0018864,0.0548412},
{0.0052731,-0.0075919,0.0392990},
{-0.0022288,-0.0556644,0.0549909},
{-0.0156238,-0.0550038,0.0377688},
{-0.0204137,-0.0100550,0.0536429},
{-0.0093683,0.0122429,-0.0162013},
{-0.0202927,0.0049166,0.0083887},
{0.0074706,-0.0627945,0.0222133},
{0.0035034,-0.0376103,0.0672421},
{-0.0190752,-0.0194129,0.0555530},
{0.0061758,-0.0074961,-0.0207741},
{0.0192011,-0.0273063,0.0588233},
{0.0130796,-0.0134383,0.0638611},
{0.0120421,0.0019566,-0.0194140},
{0.0154152,-0.0065294,0.0179581},

{-0.0157335,0.0101219,0.0123981},
{0.0110376,-0.0488274,0.0567911},
{-0.0055170,-0.0343676,0.0647140},
{0.0216204,-0.0273988,0.0512041},
{0.0161331,-0.0135875,-0.0075501},
{-0.0184643,-0.0455633,0.0360087},
{-0.0091978,-0.0682413,0.0460847},
{0.0034304,-0.0471677,0.0588866},
{-0.0048025,-0.0384316,0.0149400},
{0.0118505,0.0109735,-0.0159161},
{-0.0027036,-0.0204189,-0.0015094},
{-0.0117607,-0.0692759,0.0292171},
{-0.0107080,-0.0033455,-0.0190487},
{-0.0042548,0.0214947,-0.0056846},
{-0.0097315,-0.0208529,0.0131997},
{-0.0178958,-0.0627218,0.0171551},
{-0.0068692,-0.0688818,0.0233316},
{0.0012083,0.0120695,-0.0186179},
{0.0215073,-0.0236027,0.0503069},
{-0.0060262,-0.0534931,0.0189523},
{0.0179951,-0.0336785,0.0397267},
{0.0190509,-0.0284972,0.0336394},
{-0.0169188,-0.0133609,-0.0014203},
{0.0135722,0.0136491,0.0114695},
{-0.0150549,-0.0123230,0.0067722},
{-0.0148411,-0.0311945,0.0355412},
{0.0185490,-0.0794042,0.0431266},
{0.0193568,-0.0087326,0.0575704},
{0.0201026,-0.0144353,0.0531586},
{0.0025583,-0.0248211,0.0045714},
{0.0132689,-0.0214866,0.0652899},
{-0.0028669,-0.0099311,0.0309271},
{-0.0219487,-0.0054893,-0.0021853},
{0.0026180,-0.0203806,-0.0040620},
{-0.0116230,-0.0668518,0.0370152},
{-0.0133007,-0.0264159,0.0619176},
{0.0186167,0.0050491,-0.0120059},
{-0.0191359,-0.0591690,0.0288149},
{-0.0178121,-0.0041834,0.0141448},
{0.0123077,-0.0063648,0.0279743},
{-0.0157729,-0.0374010,0.0315335},
{0.0149328,-0.0659100,0.0290095},
{0.0006205,0.0021977,-0.0214613},
{0.0170484,-0.0715687,0.0325980},
{0.0068499,0.0061192,0.0196608},
{-0.0167075,0.0020024,0.0150382},

Biology Open • Supplementary information

{0.0059801,-0.0393117,0.0167489},
{-0.0072506,-0.0171067,0.0338751},
{0.0202363,-0.0251596,0.0420997},
{-0.0199311,-0.0601255,0.0171522},
{0.0216641,0.0053410,-0.0027841},
{-0.0197665,-0.0606125,0.0252972},
{0.0129986,-0.0214528,0.0098002},
{0.0201151,-0.0100929,0.0460501},
{0.0104137,0.0017765,-0.0203073},
{0.0166557,-0.0064123,0.0140197},
{-0.0124120,-0.0596549,0.0422637},
{0.0014885,0.0202590,0.0096781},
{0.0028720,0.0020207,-0.0219044},
{0.0376095,-0.3675427,0.0214537},
{0.0049949,0.0206417,-0.0079017},
{-0.0191723,-0.0407590,0.0391310},
{-0.0316406,-0.3638215,-0.0274841},
{0.0021392,0.0226920,0.0000985},
{0.0168435,-0.0229382,0.0264788},
{-0.0157304,-0.0005437,-0.0161456},
{0.0203722,-0.0299750,0.0562762},
{-0.0171077,0.0134146,0.0059615},
{0.0175519,-0.0370653,0.0413374},
{0.0154657,-0.0405127,0.0348980},
{-0.0215172,-0.0323502,0.0481925},
{0.0177998,-0.0515776,0.0444709},
{-0.0183804,-0.0448316,0.0275139},
{0.0033607,-0.0502028,0.0575894},
{0.0010325,-0.0479377,0.0191141},
{0.0224852,-0.0181821,0.0451780},
{0.0084089,-0.0720538,0.0521031},
{-0.0070880,-0.0772912,0.0288023},
{-0.0223712,-0.0028990,0.0024047},
{0.0173136,-0.0120017,0.0204612},
{0.0137772,-0.0623537,0.0276621},
{-0.0100390,-0.0231180,0.0292222},
{-0.0196395,-0.3730207,0.0364730},
{-0.0094445,-0.0526688,0.0528111},
{-0.0115309,-0.3679776,-0.0104995},
{0.0129812,-0.0410477,0.0224072},
{0.0117394,0.0154849,-0.0117191},
{-0.0245208,-0.3649412,0.0295111},
{0.0147476,-0.0502966,0.0364899},
{0.0006132,-0.0446771,0.0600524},
{-0.0118123,0.0191139,0.0031977},
{-0.0017185,-0.0163303,0.0664216},

Biology Open • Supplementary information

{-0.0085098,-0.0074564,0.0247745},
{0.0197459,-0.0086852,0.0430460},
{0.0187064,-0.0223532,0.0600966},
{-0.0206552,-0.0099712,0.0525024},
{0.0207525,0.0084646,-0.0024846},
{-0.0094915,-0.0325446,0.0184441},
{-0.0014464,-0.0140050,-0.0179819},
{-0.0122725,-0.0573444,0.0463125},
{-0.0106397,0.0177211,0.0094669},
{0.0111981,0.0191236,-0.0041491},
{-0.0162790,-0.0256279,0.0592687},
{-0.0060120,-0.3653959,0.0413411},
{-0.0135007,-0.3703638,-0.0387476},
{-0.0134258,-0.3703142,-0.0379467},
{-0.0133509,-0.3702645,-0.0371458},
{-0.0132760,-0.3702148,-0.0363450},
{-0.0132011,-0.3701651,-0.0355441},
{-0.0131262,-0.3701154,-0.0347432},
{-0.0130513,-0.3700658,-0.0339423},
{-0.0129765,-0.3700161,-0.0331414},
{-0.0129016,-0.3699664,-0.0323405},
{-0.0128267,-0.3699167,-0.0315396},
{-0.0127518,-0.3698670,-0.0307388},
{-0.0126769,-0.3698174,-0.0299379},
{-0.0126020,-0.3697677,-0.0291370},
{-0.0125271,-0.3697180,-0.0283361},
{-0.0124523,-0.3696683,-0.0275352},
{-0.0123774,-0.3696187,-0.0267343},
{-0.0123025,-0.3695690,-0.0259334},
{-0.0122276,-0.3695193,-0.0251325},
{-0.0121527,-0.3694696,-0.0243317},
{-0.0120778,-0.3694199,-0.0235308},
{-0.0120029,-0.3693703,-0.0227299},
{-0.0119280,-0.3693206,-0.0219290},
{-0.0118532,-0.3692709,-0.0211281},
{-0.0117783,-0.3692212,-0.0203272},
{-0.0117034,-0.3691715,-0.0195263},
{-0.0116285,-0.3691219,-0.0187255},
{-0.0115536,-0.3690722,-0.0179246},
{-0.0114787,-0.3690225,-0.0171237},
{-0.0114038,-0.3689728,-0.0163228},
{-0.0113289,-0.3689231,-0.0155219},
{-0.0112541,-0.3688735,-0.0147210},
{-0.0111792,-0.3688238,-0.0139201},
{-0.0111043,-0.3687741,-0.0131192},
{-0.0110294,-0.3687244,-0.0123184},

{-0.0109545,-0.3686748,-0.0115175},
{-0.0108796,-0.3686251,-0.0107166},
{-0.0108047,-0.3685754,-0.0099157},
{-0.0107298,-0.3685257,-0.0091148},
{-0.0106550,-0.3684760,-0.0083139},
{-0.0105801,-0.3684264,-0.0075130},
{-0.0105052,-0.3683767,-0.0067121},
{-0.0104303,-0.3683270,-0.0059113},
{-0.0103554,-0.3682773,-0.0051104},
{-0.0102805,-0.3682276,-0.0043095},
{-0.0102056,-0.3681780,-0.0035086},
{-0.0101307,-0.3681283,-0.0027077},
{-0.0100559,-0.3680786,-0.0019068},
{-0.0099810,-0.3680289,-0.0011059},
{-0.0099061,-0.3679792,-0.0003051},
{-0.0098312,-0.3679296,0.0004958},
{-0.0097563,-0.3678799,0.0012967},
{-0.0096814,-0.3678302,0.0020976},
{-0.0096065,-0.3677805,0.0028985},
{-0.0095317,-0.3677308,0.0036994},
{-0.0094568,-0.3676812,0.0045003},
{-0.0093819,-0.3676315,0.0053012},
{-0.0093070,-0.3675818,0.0061020},
{-0.0092321,-0.3675321,0.0069029},
{-0.0091572,-0.3674825,0.0077038},
{-0.0090823,-0.3674328,0.0085047},
{-0.0090074,-0.3673831,0.0093056},
{-0.0089326,-0.3673334,0.0101065},
{-0.0088577,-0.3672837,0.0109074},
{-0.0087828,-0.3672341,0.0117082},
{-0.0087079,-0.3671844,0.0125091},
{-0.0086330,-0.3671347,0.0133100},
{-0.0085581,-0.3670850,0.0141109},
{-0.0084832,-0.3670353,0.0149118},
{-0.0084083,-0.3669857,0.0157127},
{-0.0083335,-0.3669360,0.0165136},
{-0.0082586,-0.3668863,0.0173145},
{-0.0081837,-0.3668366,0.0181153},
{-0.0081088,-0.3667869,0.0189162},
{-0.0080339,-0.3667373,0.0197171},
{-0.0079590,-0.3666876,0.0205180},
{-0.0078841,-0.3666379,0.0213189},
{-0.0078092,-0.3665882,0.0221198},
{-0.0077344,-0.3665386,0.0229207},
{-0.0076595,-0.3664889,0.0237215},
{-0.0075846,-0.3664392,0.0245224},

Biology Open • Supplementary information

{-0.0075097,-0.3663895,0.0253233},
{-0.0074348,-0.3663398,0.0261242},
{-0.0073599,-0.3662902,0.0269251},
{-0.0072850,-0.3662405,0.0277260},
{-0.0072101,-0.3661908,0.0285269},
{-0.0071353,-0.3661411,0.0293278},
{-0.0070604,-0.3660914,0.0301286},
{-0.0069855,-0.3660418,0.0309295},
{-0.0069106,-0.3659921,0.0317304},
{-0.0068357,-0.3659424,0.0325313},
{-0.0067608,-0.3658927,0.0333322},
{-0.0066859,-0.3658430,0.0341331},
{-0.0066111,-0.3657934,0.0349340},
{-0.0065362,-0.3657437,0.0357348},
{-0.0064613,-0.3656940,0.0365357},
{-0.0063864,-0.3656443,0.0373366},
{-0.0063115,-0.3655947,0.0381375},
{-0.0062366,-0.3655450,0.0389384},
{-0.0061617,-0.3654953,0.0397393},
{-0.0060868,-0.3654456,0.0405402}
}* .AMirroring);
Points1 = ({
{0.0000000,0.0000000,0.0000000},
{-0.0000000,-0.4251555,0.0000000},
{-0.0096030,-0.4324410,0.0012763},
{-0.0000000,-0.4303055,0.0401789},
{-0.0000000,-0.4200055,-0.0401789},
{0.0142957,-0.0095543,0.0576365},
{0.0207430,-0.0248489,0.0453625},
{0.0099692,-0.0281849,0.0628768},
{0.0001611,-0.0004395,0.0513050},
{0.0115772,-0.0075574,0.0399826},
{-0.0061031,-0.0154785,0.0652108},
{0.0173623,-0.0427821,0.0519580},
{0.0155558,-0.0371484,0.0333871},
{0.0146935,-0.0159955,0.0252592},
{-0.0104552,-0.0355272,0.0622214},
{0.0034635,-0.0465071,0.0611948},
{-0.0031709,-0.0222696,0.0415054},
{-0.0128714,-0.0047794,0.0483395},
{-0.0000091,-0.0046883,0.0280461},
{-0.0126391,-0.0253605,0.0496912},
{0.0103840,-0.0673100,0.0545877},
{0.0165141,-0.0597973,0.0388886},
{0.0086086,-0.0504923,0.0194999},
{0.0099556,-0.0268907,0.0127212},

{0.0188649,-0.0061009,0.0058149},
{0.0106750,0.0067264,0.0162281},
{-0.0125735,-0.0423024,0.0369799},
{-0.0103291,-0.0549908,0.0525409},
{-0.0094824,-0.0283071,0.0253857},
{-0.0062957,0.0118408,0.0162577},
{-0.0116836,-0.0065143,0.0160262},
{-0.0076466,-0.0777678,0.0455642},
{0.0064708,-0.0892368,0.0529307},
{0.0175856,-0.0836504,0.0375139},
{0.0085348,-0.0720366,0.0214263},
{-0.0120812,-0.0511487,0.0210574},
{-0.0043112,-0.0363576,0.0104044},
{-0.0004560,-0.0220668,-0.0048982},
{0.0131378,-0.0172949,-0.0066504},
{0.0177519,-0.0019882,-0.0113575},
{0.0174059,0.0144047,-0.0012364},
{0.0032171,0.0218599,0.0091844},
{-0.0146082,-0.0634936,0.0345529},
{-0.0121368,-0.0138744,0.0059142},
{-0.0124225,0.0188478,0.0016402},
{-0.0189506,0.0026129,0.0032360},
{-0.0069722,-0.0861389,0.0282956},
{-0.0044120,-0.1032818,0.0429048},
{0.0149703,-0.1084703,0.0457333},
{0.0127791,-0.0926769,0.0222168},
{-0.0103734,-0.0687977,0.0218721},
{-0.0148853,-0.0098189,-0.0104333},
{0.0006740,-0.0101404,-0.0190678},
{0.0061228,0.0086238,-0.0175512},
{0.0017228,0.0223074,-0.0082921},
{-0.0107760,0.0093327,-0.0148370},
{0.0004537,-0.1086567,0.0237957},
{-0.0019058,-0.1235320,0.0409115},
{0.0161220,-0.1307936,0.0436324},
{0.0192302,-0.1124406,0.0268799},
{0.0063943,-0.1276641,0.0197378},
{0.0000141,-0.1442436,0.0358246},
{0.0172564,-0.1531552,0.0413788},
{0.0229508,-0.1364777,0.0263885},
{0.0099477,-0.1486033,0.0168631},
{0.0023940,-0.1656010,0.0328967},
{0.0196358,-0.1757625,0.0385987},
{0.0253449,-0.1597074,0.0237217},
{0.0117486,-0.1712313,0.0143889},
{0.0039369,-0.1869341,0.0296028},

Biology Open • Supplementary information

{0.0199451,-0.1986224,0.0362333},
{0.0269365,-0.1808293,0.0214930},
{0.0131619,-0.1932618,0.0121758},
{0.0027062,-0.2089582,0.0219076},
{0.0143536,-0.2207535,0.0323300},
{0.0282637,-0.2184590,0.0246099},
{0.0272932,-0.2012584,0.0172303},
{0.0164464,-0.2182595,0.0090604},
{0.0024096,-0.2313727,0.0187059},
{0.0113691,-0.2476670,0.0282225},
{0.0253803,-0.2393774,0.0291334},
{0.0275532,-0.2370104,0.0133107},
{0.0145250,-0.2444518,0.0070236},
{0.0020346,-0.2578580,0.0158646},
{0.0124507,-0.2726093,0.0270687},
{0.0262004,-0.2607617,0.0260185},
{0.0274174,-0.2619619,0.0101721},
{0.0130697,-0.2705357,0.0045848},
{0.0029578,-0.2828459,0.0151174},
{0.0100639,-0.2977552,0.0247673},
{0.0266299,-0.2854088,0.0229692},
{0.0265537,-0.2844856,0.0062180},
{0.0102661,-0.2970280,0.0024592},
{0.0026367,-0.3133294,0.0108380},
{0.0112276,-0.3213791,0.0244795},
{0.0262020,-0.3097170,0.0211714},
{0.0267662,-0.3046407,0.0040682},
{0.0156449,-0.3187204,-0.0028549},
{0.0039628,-0.3364006,0.0007709},
{0.0025688,-0.3389765,0.0184723},
{0.0223435,-0.3384398,0.0219760},
{0.0292432,-0.3291284,0.0083180},
{0.0231660,-0.3428657,-0.0048947},
{0.0065302,-0.3558244,-0.0067540},
{-0.0001698,-0.3601966,0.0092132},
{0.0119777,-0.3579596,0.0238760},
{0.0293213,-0.3562185,0.0112422},
{0.0229403,-0.3652550,-0.0082923},
{0.0046156,-0.3779211,-0.0121934},
{-0.0022000,-0.3837880,0.0042659},
{0.0016576,-0.3764824,0.0214416},
{0.0225984,-0.3757946,0.0211644},
{0.0287765,-0.3794557,0.0050631},
{0.0218158,-0.3874654,-0.0130281},
{0.0098881,-0.4017368,-0.0240717},
{-0.0052176,-0.3968162,-0.0135541},

Biology Open • Supplementary information

{-0.0063419,-0.4099311,0.0028225},
{-0.0058414,-0.4005854,0.0199749},
{0.0103478,-0.3956687,0.0260024},
{0.0278263,-0.3980519,0.0177474},
{0.0266163,-0.4034115,-0.0001464},
{0.0252982,-0.4122401,-0.0178984},
{0.0176137,-0.4274234,-0.0298080},
{0.0042471,-0.4166083,-0.0375918},
{-0.0093714,-0.4068954,-0.0290396},
{-0.0161941,-0.4164656,-0.0124675},
{-0.0059939,-0.4337725,-0.0032542},
{-0.0173750,-0.4246694,0.0108378},
{-0.0150902,-0.4169414,0.0294926},
{0.0020994,-0.4123661,0.0345252},
{0.0207380,-0.4171395,0.0275438},
{0.0350981,-0.4182602,0.0156852},
{0.0271396,-0.4252295,-0.0018314},
{0.0293813,-0.4398149,-0.0161149},
{0.0172575,-0.4531886,-0.0273154},
{0.0025201,-0.4396468,-0.0390897},
{-0.0134589,-0.4260013,-0.0395794},
{-0.0273285,-0.4193545,-0.0280480},
{-0.0271118,-0.4348897,-0.0118931},
{-0.0173073,-0.4536653,-0.0175126},
{0.0001547,-0.4533735,-0.0116195},
{0.0029786,-0.4507854,0.0074366},
{-0.0141849,-0.4479678,0.0138099},
{-0.0243814,-0.4350788,0.0280654},
{-0.0044100,-0.4322531,0.0398274},
{0.0131090,-0.4363760,0.0328957},
{0.0319767,-0.4376858,0.0241808},
{0.0267186,-0.4426946,0.0071303},
{0.0165345,-0.4493701,-0.0064322},
{-0.0001431,-0.4597152,-0.0298259},
{-0.0142719,-0.4507029,-0.0378101},
{-0.0296586,-0.4409095,-0.0316287},
{0.0193393,-0.4543057,0.0207540},
{0.0015479,-0.4570364,0.0272234},
{-0.0144338,-0.4523126,0.0331619},
{-0.0008327,-0.0739551,0.0191074},
{-0.0099825,-0.0643795,0.0207205},
{0.0095156,-0.0377659,0.0620100},
{-0.0137300,-0.0153456,0.0571572},
{-0.0016831,-0.0629081,0.0574617},
{-0.0060903,-0.0706443,0.0225442},
{-0.0013640,-0.0907720,0.0205866},

{-0.0067629,0.0018754,-0.0170586},
{-0.0120565,-0.0493732,0.0412900},
{0.0031970,-0.0294303,0.0657458},
{-0.0098302,-0.0729773,0.0257857},
{-0.0010324,-0.0599791,0.0184125},
{-0.0127479,-0.0188359,0.0509469},
{0.0131723,-0.0333095,0.0603258},
{-0.0101257,-0.0330840,0.0398181},
{0.0138266,-0.0380728,0.0586369},
{-0.0008621,-0.0828572,0.0538586},
{-0.0143384,-0.0485069,0.0313499},
{-0.0135098,-0.0615097,0.0220539},
{-0.0157322,-0.0601606,0.0233284},
{-0.0023999,-0.0457195,0.0136129},
{0.0104794,-0.0640907,0.0239572},
{0.0209945,-0.0146591,0.0396741},
{-0.0030234,-0.0487035,0.0621004},
{0.0131262,-0.0031042,0.0172619},
{-0.0085765,-0.0538085,0.0189575},
{-0.0057816,-0.0894513,0.0444631},
{0.0054077,0.0163355,0.0131501},
{-0.0109612,0.0187947,-0.0060283},
{-0.0068459,-0.0743141,0.0475929},
{0.0076047,-0.0934689,0.0194415},
{0.0125414,-0.0819930,0.0243371},
{0.0075142,-0.0520509,0.0588912},
{0.0140076,-0.0139121,0.0076010},
{-0.0056060,-0.0259410,0.0045843},
{-0.0069772,-0.0010700,0.0201061},
{-0.0098024,-0.0619550,0.0489729},
{-0.0060125,-0.0288250,0.0658084},
{0.0081661,-0.0069991,0.0601046},
{0.0132087,-0.0556416,0.0296523},
{-0.0019484,-0.0199631,-0.0120628},
{-0.0133816,-0.0269225,0.0546438},
{0.0155494,0.0146474,-0.0070204},
{-0.0039394,0.0237361,0.0036575},
{0.0006129,-0.0936066,0.0522387},
{-0.0126101,-0.0435184,0.0494718},
{0.0192791,-0.0307732,0.0420082},
{0.0112462,-0.0472377,0.0238814},
{0.0173034,0.0114334,0.0071475},
{0.0199416,0.0022898,0.0066762},
{0.0208747,-0.0021793,-0.0033791},
{0.0133717,-0.0221318,0.0222784},
{0.0110994,-0.0380960,0.0212708},

{0.0157526,-0.0161510,0.0318701},
{-0.0168455,-0.0538837,0.0261983},
{0.0029148,-0.0421221,0.0637955},
{0.0162972,-0.0496130,0.0379656},
{0.0140866,-0.0111267,0.0131121},
{0.0009948,0.0048252,-0.0191881},
{0.0084071,-0.0008383,-0.0190423},
{0.0100691,-0.0740396,0.0530487},
{0.0143806,-0.0668595,0.0503942},
{0.0168909,-0.0649603,0.0419033},
{0.0170841,-0.0752019,0.0354372},
{-0.0045497,-0.0820504,0.0242521},
{-0.0127820,0.0040967,0.0137890},
{0.0074376,-0.0012309,0.0544513},
{0.0185054,-0.0102559,0.0496338},
{0.0144277,-0.0259215,0.0593901},
{-0.0114741,-0.0391897,0.0249915},
{-0.0176863,-0.0082073,-0.0025197},
{0.0157362,-0.0906067,0.0270126},
{-0.0067172,-0.0062874,-0.0187429},
{0.0039607,0.0154315,-0.0146024},
{-0.0089930,-0.0361694,0.0157670},
{-0.0143142,0.0112861,-0.0102596},
{-0.0194279,0.0054276,-0.0016374},
{-0.0087810,-0.0766660,0.0389695},
{0.0112456,-0.0190515,-0.0009348},
{0.0121031,-0.0496847,0.0565443},
{0.0084019,-0.0201998,-0.0088575},
{0.0097369,-0.0152128,-0.0135755},
{0.0041693,-0.0649308,0.0186724},
{0.0086812,-0.0553895,0.0211561},
{0.0011865,-0.0830371,0.0197797},
{-0.0176614,-0.0612657,0.0297566},
{-0.0058714,-0.0014852,0.0514124},
{-0.0127314,-0.0336196,0.0586432},
{0.0174345,-0.0189441,0.0533461},
{0.0145866,-0.0840189,0.0475746},
{0.0182736,-0.0262059,0.0345788},
{0.0081295,-0.0347689,0.0655871},
{-0.0165573,-0.0035475,0.0097913},
{0.0140406,-0.0176199,0.0593594},
{0.0150797,-0.0668860,0.0321583},
{-0.0148117,-0.0547496,0.0222170},
{-0.0136952,-0.0364334,0.0518969},
{0.0135417,-0.0454633,0.0301530},
{-0.0076209,-0.0423014,0.0635003},

Biology Open • Supplementary information

{0.0064715,-0.0084554,-0.0185962},
{0.0040352,-0.0168479,-0.0149546},
{-0.0140452,-0.0117802,0.0507994},
{-0.0101879,-0.0488987,0.0571738},
{0.0170226,-0.0590493,0.0466482},
{-0.0164832,0.0122000,0.0015480},
{-0.0134840,0.0100198,0.0115011},
{-0.0018596,-0.0743006,0.0541086},
{0.0129708,-0.0101481,0.0336718},
{0.0135600,-0.0038287,0.0544300},
{0.0149126,-0.0062759,0.0426546},
{-0.0124961,-0.0147037,-0.0084975},
{0.0056005,-0.0239991,0.0023250},
{0.0003608,-0.0536028,0.0168432},
{0.0195863,0.0034220,-0.0075073},
{0.0167880,0.0017755,-0.0130173},
{0.0125205,-0.0084333,-0.0156125},
{-0.0128497,0.0015007,-0.0152749},
{-0.0143481,-0.0065353,-0.0130888},
{0.0173196,0.0144673,0.0031705},
{0.0103489,0.0208608,-0.0022694},
{0.0088336,0.0151759,-0.0130813},
{0.0078935,-0.0247060,0.0638313},
{-0.0006960,-0.0094375,0.0629568},
{-0.0001624,-0.0022871,0.0567379},
{0.0052709,-0.0150020,0.0644049},
{0.0082303,0.0080374,0.0163817},
{0.0120798,-0.0190314,0.0155343},
{0.0038611,-0.0276488,0.0654906},
{-0.0089455,-0.0188982,0.0241920},
{-0.0100261,-0.0191001,0.0120765},
{-0.0063547,-0.0109385,0.0270135},
{-0.0147933,-0.0554764,0.0353395},
{-0.0111461,-0.0670649,0.0409048},
{-0.0068033,-0.0318317,0.0093511},
{0.0203350,-0.0202424,0.0461631},
{0.0120161,-0.0092605,0.0276465},
{0.0042462,-0.0010353,0.0242212},
{-0.0035209,-0.0832038,0.0526646},
{-0.0023626,0.0240090,-0.0032637},
{-0.0011229,-0.0144690,0.0390524},
{-0.0107440,-0.0157244,0.0024589},
{0.0127360,-0.0315566,0.0255963},
{0.0101482,-0.0297626,0.0171028},
{-0.0108133,-0.0076163,0.0525692},
{0.0184106,-0.0363147,0.0478843},

Biology Open • Supplementary information

{-0.0118653,-0.0586869,0.0414780},
{-0.0073431,-0.0887015,0.0376037},
{0.0042588,-0.0057549,0.0307972},
{-0.0029373,-0.0050512,-0.0199299},
{-0.0123976,-0.0381118,0.0582032},
{-0.0029585,0.0097633,-0.0168482},
{-0.0042481,0.0182025,-0.0124424},
{0.0182947,-0.0941570,0.0362526},
{0.0036301,-0.0811998,0.0189988},
{0.0161828,-0.0971478,0.0445661},
{0.0094255,0.0210627,0.0053711},
{0.0115454,0.0100409,0.0145496},
{0.0010801,0.0165438,-0.0142783},
{0.0158047,-0.0128293,-0.0088314},
{0.0186125,-0.0083915,-0.0075735},
{-0.0085519,-0.0267411,0.0303713},
{-0.0107182,-0.0380266,0.0321037},
{-0.0131742,-0.0679206,0.0256811},
{-0.0013916,0.0029302,0.0200577},
{0.0040826,0.0080626,0.0173042},
{-0.0024725,-0.0031413,0.0256242},
{0.0002523,-0.0339389,0.0649938},
{0.0067805,-0.0478432,0.0598146},
{-0.0114835,-0.0218756,0.0626579},
{-0.0114569,-0.0290102,0.0618896},
{0.0088705,0.0223223,0.0026442},
{0.0167793,0.0155919,0.0014030},
{-0.0069910,-0.0120291,0.0637946},
{0.0149375,-0.0585912,0.0343232},
{-0.0121738,-0.0524266,0.0479818},
{-0.0147240,-0.0555980,0.0219784},
{0.0107484,-0.0929333,0.0505340},
{-0.0096095,-0.0278250,0.0176816},
{-0.0099049,-0.0169442,0.0170009},
{-0.0043400,0.0176315,0.0126169},
{0.0008769,-0.0751272,0.0558668},
{0.0057725,-0.0692637,0.0560385},
{-0.0022138,-0.0689203,0.0555342},
{0.0162451,-0.0812152,0.0310188},
{-0.0122085,-0.0201641,0.0488832},
{0.0104573,-0.0273972,0.0172112},
{0.0050512,-0.0578613,0.0585039},
{0.0081386,-0.0386696,0.0622004},
{0.0093485,-0.0928796,0.0198191},
{-0.0066424,-0.0582799,0.0568033},
{0.0175665,-0.0471545,0.0439029},

{-0.0116790,-0.0471352,0.0211158},
{0.0207610,-0.0227037,0.0373486},
{-0.0039475,-0.0671032,0.0202562},
{0.0163109,-0.0021913,0.0127414},
{0.0060472,0.0059454,0.0174532},
{0.0008973,-0.0356806,0.0648763},
{-0.0145217,0.0150907,-0.0050617},
{0.0103917,0.0172374,0.0101362},
{-0.0104541,0.0175875,0.0081332},
{-0.0182301,-0.0060358,-0.0051498},
{-0.0183388,0.0005583,-0.0073872},
{-0.0048011,-0.0192032,0.0661919},
{-0.0128817,-0.0376806,0.0433997},
{0.0054732,-0.0514149,0.0597865},
{0.0121818,-0.0456627,0.0575491},
{-0.0085634,-0.0749034,0.0253109},
{0.0123749,0.0077723,0.0149833},
{0.0178621,-0.0117258,-0.0011185},
{-0.0169720,-0.0088101,0.0049857},
{-0.0125156,-0.0616304,0.0384587},
{-0.0071644,-0.0184463,-0.0037014},
{-0.0126731,-0.0301651,0.0465187},
{0.0045585,-0.0819557,0.0543227},
{0.0156500,-0.0546297,0.0512126},
{-0.0017001,-0.0381389,0.0108897},
{0.0061208,-0.0350236,0.0129800},
{0.0183929,-0.0076246,0.0057079},
{0.0177597,-0.0890834,0.0411791},
{-0.0089480,-0.0207062,0.0247918},
{-0.0008610,0.0141710,0.0158665},
{0.0072542,-0.0746436,0.0208976},
{0.0086161,-0.0682106,0.0554026},
{0.0147168,-0.0141766,-0.0093005},
{0.0005447,-0.0099521,0.0634260},
{-0.0070057,-0.0161222,-0.0139446},
{0.0155734,-0.0770614,0.0462586},
{0.0075327,-0.0201295,-0.0101782},
{-0.0103995,-0.0704592,0.0228510},
{0.0130418,-0.0429701,0.0285548},
{0.0152016,-0.0046743,0.0529995},
{0.0046476,-0.0083463,0.0386328},
{-0.0029744,-0.0674764,0.0560826},
{-0.0125429,-0.0650371,0.0374527},
{-0.0141596,-0.0124791,0.0530348},
{-0.0076993,0.0133991,-0.0143782},
{-0.0168189,0.0063183,0.0079978},

Biology Open • Supplementary information

{0.0091571,-0.0743503,0.0217864},
{0.0024429,-0.0424451,0.0628999},
{-0.0132271,-0.0236598,0.0539505},
{0.0063080,-0.0066918,-0.0192844},
{0.0150897,-0.0308168,0.0554568},
{0.0117184,-0.0163287,0.0608534},
{0.0102063,0.0022547,-0.0177624},
{0.0134261,-0.0088256,0.0168683},
{-0.0126745,0.0120351,0.0114725},
{0.0101463,-0.0571006,0.0564887},
{-0.0053535,-0.0414664,0.0643088},
{0.0190430,-0.0313770,0.0491142},
{0.0155211,-0.0142823,-0.0068794},
{-0.0146145,-0.0541797,0.0358313},
{-0.0073961,-0.0795523,0.0444234},
{0.0031336,-0.0567718,0.0592294},
{-0.0043236,-0.0446710,0.0137753},
{0.0109072,0.0115284,-0.0143619},
{-0.0020991,-0.0232980,-0.0008298},
{-0.0089786,-0.0790223,0.0297111},
{-0.0101159,-0.0037747,-0.0172723},
{-0.0028561,0.0233569,-0.0045948},
{-0.0093903,-0.0233996,0.0130429},
{-0.0105203,-0.0694168,0.0226765},
{-0.0058246,-0.0805103,0.0244144},
{0.0009336,0.0127720,-0.0164303},
{0.0194379,-0.0279930,0.0483587},
{-0.0057746,-0.0624724,0.0195910},
{0.0172702,-0.0391030,0.0390821},
{0.0172452,-0.0331466,0.0348200},
{-0.0140669,-0.0128783,-0.0009650},
{0.0130013,0.0150894,0.0099930},
{-0.0131155,-0.0117452,0.0068635},
{-0.0104812,-0.0378828,0.0354295},
{0.0175956,-0.0944224,0.0420564},
{0.0173246,-0.0111182,0.0552071},
{0.0183887,-0.0167406,0.0511452},
{0.0013345,-0.0284830,0.0049784},
{0.0105758,-0.0251170,0.0622850},
{-0.0027341,-0.0102565,0.0311282},
{-0.0188953,-0.0045616,-0.0017235},
{0.0027030,-0.0225830,-0.0037768},
{-0.0094923,-0.0764242,0.0366360},
{-0.0124856,-0.0295204,0.0603940},
{0.0175365,0.0055736,-0.0106691},
{-0.0144774,-0.0668633,0.0298626},

Biology Open • Supplementary information

{-0.0149301,-0.0045087,0.0126807},
{0.0117565,-0.0086285,0.0268150},
{-0.0131642,-0.0459946,0.0317615},
{0.0147491,-0.0768134,0.0285339},
{0.0007541,0.0036159,-0.0194386},
{0.0165468,-0.0831559,0.0311338},
{0.0068043,0.0068461,0.0167233},
{-0.0140017,0.0024243,0.0128402},
{0.0057343,-0.0460393,0.0164606},
{-0.0063467,-0.0204065,0.0337362},
{0.0201430,-0.0289105,0.0426142},
{-0.0126502,-0.0671274,0.0228016},
{0.0204423,0.0054838,-0.0030200},
{-0.0136014,-0.0673052,0.0277830},
{0.0110425,-0.0239849,0.0110764},
{0.0193495,-0.0125363,0.0454609},
{0.0084917,0.0020858,-0.0187015},
{0.0144636,-0.0089029,0.0135568},
{-0.0111190,-0.0681267,0.0402595},
{0.0004542,0.0220718,0.0092305},
{0.0027866,0.0035407,-0.0197572},
{0.0370096,-0.4320457,0.0211127},
{0.0050156,0.0218706,-0.0078225},
{-0.0134015,-0.0482237,0.0374950},
{-0.0311445,-0.4276703,-0.0270533},
{0.0022989,0.0247427,-0.0000321},
{0.0142584,-0.0262881,0.0267369},
{-0.0140055,-0.0011787,-0.0146110},
{0.0164237,-0.0331953,0.0532961},
{-0.0145386,0.0144077,0.0047694},
{0.0171224,-0.0429439,0.0402766},
{0.0152811,-0.0479082,0.0345470},
{-0.0131678,-0.0377890,0.0460407},
{0.0171934,-0.0606798,0.0443109},
{-0.0165959,-0.0527300,0.0276525},
{0.0030322,-0.0602426,0.0584754},
{0.0010790,-0.0561982,0.0174635},
{0.0212434,-0.0228988,0.0442968},
{0.0078432,-0.0836783,0.0530060},
{-0.0059010,-0.0908466,0.0280964},
{-0.0188269,-0.0023576,0.0030712},
{0.0142705,-0.0146782,0.0198540},
{0.0137253,-0.0729352,0.0276255},
{-0.0092139,-0.0270389,0.0276679},
{-0.0193299,-0.4384840,0.0358989},
{-0.0085774,-0.0610649,0.0524600},

Biology Open • Supplementary information

{-0.0113497,-0.4325559,-0.0103345},
{0.0105780,-0.0473430,0.0225178},
{0.0107507,0.0158903,-0.0112529},
{-0.0241355,-0.4289866,0.0290474},
{0.0154951,-0.0581613,0.0352090},
{0.0002955,-0.0542342,0.0601727},
{-0.0108769,0.0198015,0.0028592},
{-0.0024434,-0.0182747,0.0660515},
{-0.0072235,-0.0080650,0.0242411},
{0.0191889,-0.0112782,0.0424304},
{0.0146951,-0.0268043,0.0574839},
{-0.0139927,-0.0119368,0.0522991},
{0.0196124,0.0090137,-0.0028209},
{-0.0099202,-0.0373400,0.0180156},
{-0.0003333,-0.0154567,-0.0165191},
{-0.0106627,-0.0659174,0.0443814},
{-0.0089101,0.0187413,0.0080582},
{0.0096588,0.0209497,-0.0030155},
{-0.0132542,-0.0295912,0.0572737},
{-0.0059175,-0.4295211,0.0406915},
{-0.0132885,-0.4353608,-0.0381388},
{-0.0132148,-0.4353024,-0.0373505},
{-0.0131411,-0.4352440,-0.0365622},
{-0.0130674,-0.4351856,-0.0357739},
{-0.0129937,-0.4351272,-0.0349856},
{-0.0129200,-0.4350689,-0.0341973},
{-0.0128463,-0.4350105,-0.0334090},
{-0.0127726,-0.4349521,-0.0326207},
{-0.0126989,-0.4348937,-0.0318324},
{-0.0126251,-0.4348353,-0.0310441},
{-0.0125514,-0.4347769,-0.0302558},
{-0.0124777,-0.4347185,-0.0294675},
{-0.0124040,-0.4346601,-0.0286792},
{-0.0123303,-0.4346017,-0.0278909},
{-0.0122566,-0.4345433,-0.0271026},
{-0.0121829,-0.4344849,-0.0263143},
{-0.0121092,-0.4344265,-0.0255259},
{-0.0120355,-0.4343681,-0.0247376},
{-0.0119618,-0.4343097,-0.0239493},
{-0.0118880,-0.4342513,-0.0231610},
{-0.0118143,-0.4341929,-0.0223727},
{-0.0117406,-0.4341345,-0.0215844},
{-0.0116669,-0.4340761,-0.0207961},
{-0.0115932,-0.4340177,-0.0200078},
{-0.0115195,-0.4339593,-0.0192195},
{-0.0114458,-0.4339009,-0.0184312},

Biology Open • Supplementary information

{-0.0113721,-0.4338425,-0.0176429},
{-0.0112984,-0.4337841,-0.0168546},
{-0.0112246,-0.4337257,-0.0160663},
{-0.0111509,-0.4336673,-0.0152780},
{-0.0110772,-0.4336089,-0.0144897},
{-0.0110035,-0.4335505,-0.0137014},
{-0.0109298,-0.4334921,-0.0129131},
{-0.0108561,-0.4334337,-0.0121248},
{-0.0107824,-0.4333753,-0.0113365},
{-0.0107087,-0.4333169,-0.0105482},
{-0.0106350,-0.4332585,-0.0097599},
{-0.0105613,-0.4332001,-0.0089716},
{-0.0104875,-0.4331417,-0.0081833},
{-0.0104138,-0.4330833,-0.0073950},
{-0.0103401,-0.4330249,-0.0066067},
{-0.0102664,-0.4329665,-0.0058184},
{-0.0101927,-0.4329081,-0.0050301},
{-0.0101190,-0.4328497,-0.0042418},
{-0.0100453,-0.4327914,-0.0034535},
{-0.0099716,-0.4327330,-0.0026652},
{-0.0098979,-0.4326746,-0.0018769},
{-0.0098241,-0.4326162,-0.0010886},
{-0.0097504,-0.4325578,-0.0003003},
{-0.0096767,-0.4324994,0.0004880},
{-0.0096030,-0.4324410,0.0012763},
{-0.0095293,-0.4323826,0.0020646},
{-0.0094556,-0.4323242,0.0028529},
{-0.0093819,-0.4322658,0.0036413},
{-0.0093082,-0.4322074,0.0044296},
{-0.0092345,-0.4321490,0.0052179},
{-0.0091608,-0.4320906,0.0060062},
{-0.0090870,-0.4320322,0.0067945},
{-0.0090133,-0.4319738,0.0075828},
{-0.0089396,-0.4319154,0.0083711},
{-0.0088659,-0.4318570,0.0091594},
{-0.0087922,-0.4317986,0.0099477},
{-0.0087185,-0.4317402,0.0107360},
{-0.0086448,-0.4316818,0.0115243},
{-0.0085711,-0.4316234,0.0123126},
{-0.0084974,-0.4315650,0.0131009},
{-0.0084236,-0.4315066,0.0138892},
{-0.0083499,-0.4314482,0.0146775},
{-0.0082762,-0.4313898,0.0154658},
{-0.0082025,-0.4313314,0.0162541},
{-0.0081288,-0.4312730,0.0170424},
{-0.0080551,-0.4312146,0.0178307},

Biology Open • Supplementary information

```
                {-0.0079814,-0.4311562,0.0186190},
                {-0.0079077,-0.4310978,0.0194073},
                {-0.0078340,-0.4310394,0.0201956},
                {-0.0077603,-0.4309810,0.0209839},
                {-0.0076865,-0.4309226,0.0217722},
                {-0.0076128,-0.4308642,0.0225605},
                {-0.0075391,-0.4308058,0.0233488},
                {-0.0074654,-0.4307474,0.0241371},
                {-0.0073917,-0.4306890,0.0249254},
                {-0.0073180,-0.4306306,0.0257137},
                {-0.0072443,-0.4305722,0.0265020},
                {-0.0071706,-0.4305139,0.0272903},
                {-0.0070969,-0.4304555,0.0280786},
                {-0.0070231,-0.4303971,0.0288669},
                {-0.0069494,-0.4303387,0.0296552},
                {-0.0068757,-0.4302803,0.0304435},
                {-0.0068020,-0.4302219,0.0312318},
                {-0.0067283,-0.4301635,0.0320201},
                {-0.0066546,-0.4301051,0.0328084},
                {-0.0065809,-0.4300467,0.0335968},
                {-0.0065072,-0.4299883,0.0343851},
                {-0.0064335,-0.4299299,0.0351734},
                {-0.0063598,-0.4298715,0.0359617},
                {-0.0062860,-0.4298131,0.0367500},
                {-0.0062123,-0.4297547,0.0375383},
                {-0.0061386,-0.4296963,0.0383266},
                {-0.0060649,-0.4296379,0.0391149},
                {-0.0059912,-0.4295795,0.0399032}
                }* .AMirroring);
            BoundingBoxOnOff = Off;
        };
        AnyFunTransform3DIdentity ScaleFunction = {
            PreTransforms = {&.RBFTransform};
      };
};
};
};
```

Biology Open • Supplementary information

ScalingFunctionTLEMLucyPelvis_2014019

```
AnyFolder ScalingFunctionTLEMLucyPelvis = {
AnyFolder Pelvis = {
	AnyFunTransform3DRBF RBFTransform = {
		RBFDef.Type = RBF_ThinPlate;
		PolynomDegree = 1;
		Points0 = {
			{0.0000000,0.0000000,0.1177000},
			{-0.0000000,-0.0832729,0.0191000},
			{-0.0508179,-0.0694062,0.0815920},
			{0.0000000,0.0000000,-0.1177000},
			{-0.0000000,-0.0832729,-0.0191000},
			{-0.0508179,-0.0694062,-0.0815920},
			{0.0000000,0.0000000,0.0000000},
			{-0.1164020,-0.0039449,0.0456760},
			{-0.1164020,-0.0039449,-0.0456760},
			{-0.1092909,-0.0992949,0.0515590},
			{-0.1092909,-0.0992949,-0.0515590},
			{-0.0733749,0.0757445,0.0915590},
			{-0.0733749,0.0757445,-0.0915590},
			{-0.1241738,0.0316243,0.0458050},
			{-0.1241738,0.0316243,-0.0458050},
			{-0.1108008,0.0563490,0.0555690},
			{-0.1108008,0.0563490,-0.0555690},
			{-0.0545356,0.0481653,0.1291690},
			{-0.0545356,0.0481653,-0.1291690},
			{-0.0360236,0.0456352,0.1316250},
			{-0.0360236,0.0456352,-0.1316250},
			{-0.0804664,-0.0228481,0.0672140},
			{-0.0804664,-0.0228481,-0.0672140},
			{-0.1004070,-0.0623213,0.0494990},
			{-0.1004070,-0.0623213,-0.0494990},
			{-0.0174901,-0.0357786,0.0977400},
			{-0.0174901,-0.0357786,-0.0977400},
			{-0.0665950,-0.1308418,0.0278190},
			{-0.0665950,-0.1308418,-0.0278190},
			{-0.0962739,-0.1257274,0.0551670},
			{-0.0962739,-0.1257274,-0.0551670},
			{-0.0393340,-0.1196098,0.0159820},
			{-0.0393340,-0.1196098,-0.0159820},
			{-0.0585979,-0.0148191,0.0651020},
			{-0.0585979,-0.0148191,-0.0651020},
			{-0.0238420,-0.0800479,0.0351680},
			{-0.0238420,-0.0800479,-0.0351680},
			{-0.0234553,-0.0576924,0.0715330},
```

Biology Open • Supplementary information

{-0.0234553,-0.0576924,-0.0715330},
{-0.0472319,-0.0833276,0.0513790},
{-0.0472319,-0.0833276,-0.0513790},
{-0.0327175,-0.0506481,0.0520240},
{-0.0327175,-0.0506481,-0.0520240},
{-0.0746024,-0.0627219,0.0928110},
{-0.0746024,-0.0627219,-0.0928110},
{-0.0119676,-0.0928080,0.0076900},
{-0.0119676,-0.0928080,-0.0076900},
{-0.0263299,-0.0760728,0.0667080},
{-0.0263299,-0.0760728,-0.0667080},
{-0.0498842,0.0119450,0.1070630},
{-0.0498842,0.0119450,-0.1070630},
{-0.0553874,0.0627474,0.1172390},
{-0.0553874,0.0627474,-0.1172390},
{-0.0786774,0.0073556,0.0741800},
{-0.0786774,0.0073556,-0.0741800},
{-0.0742762,0.0474272,0.0542330},
{-0.0742762,0.0474272,-0.0542330},
{-0.0634121,0.0117185,0.0618430},
{-0.0634121,0.0117185,-0.0618430},
{-0.0813084,0.0007821,0.0508130},
{-0.0813084,0.0007821,-0.0508130},
{-0.0496423,-0.0444458,0.0983920},
{-0.0496423,-0.0444458,-0.0983920},
{-0.0480005,-0.0338999,0.0625080},
{-0.0480005,-0.0338999,-0.0625080},
{-0.0428000,-0.0273188,0.0973490},
{-0.0428000,-0.0273188,-0.0973490},
{-0.0548383,-0.0518766,0.0624050},
{-0.0548383,-0.0518766,-0.0624050},
{-0.0696448,-0.1052827,0.0436200},
{-0.0696448,-0.1052827,-0.0436200},
{-0.0739608,-0.0803177,0.0785950},
{-0.0739608,-0.0803177,-0.0785950},
{-0.0586066,-0.0884764,0.0653190},
{-0.0586066,-0.0884764,-0.0653190},
{-0.1017003,0.0085215,0.0624150},
{-0.1017003,0.0085215,-0.0624150},
{-0.0126252,0.0252852,0.1292530},
{-0.0126252,0.0252852,-0.1292530},
{-0.0030963,0.0150156,0.1262640},
{-0.0030963,0.0150156,-0.1262640},
{-0.0621797,0.0048743,0.0605920},
{-0.0621797,0.0048743,-0.0605920},
{-0.0870082,-0.0684280,0.0489900},

Biology Open • Supplementary information

{-0.0870082,-0.0684280,-0.0489900},
{-0.0916211,-0.0478344,0.0575290},
{-0.0916211,-0.0478344,-0.0575290},
{-0.0428573,-0.0467026,0.0985250},
{-0.0428573,-0.0467026,-0.0985250},
{-0.0483869,-0.0455474,0.0848580},
{-0.0483869,-0.0455474,-0.0848580},
{-0.0203929,-0.0681811,0.0426070},
{-0.0203929,-0.0681811,-0.0426070},
{-0.0271973,-0.0807786,0.0491650},
{-0.0271973,-0.0807786,-0.0491650},
{-0.0325935,-0.1026813,0.0148280},
{-0.0325935,-0.1026813,-0.0148280},
{-0.0273900,-0.1059173,0.0048750},
{-0.0273900,-0.1059173,-0.0048750},
{-0.0015971,-0.0778770,0.0065530},
{-0.0015971,-0.0778770,-0.0065530},
{-0.1034983,-0.0140026,0.0521800},
{-0.1034983,-0.0140026,-0.0521800},
{-0.1141160,0.0077734,0.0497600},
{-0.1141160,0.0077734,-0.0497600},
{-0.1123797,0.0244808,0.0350160},
{-0.1123797,0.0244808,-0.0350160},
{-0.0984470,-0.0648210,0.0457100},
{-0.0984470,-0.0648210,-0.0457100},
{-0.0814332,-0.0704479,0.0806410},
{-0.0814332,-0.0704479,-0.0806410},
{-0.0540218,-0.0805070,0.0547320},
{-0.0540218,-0.0805070,-0.0547320},
{-0.0674356,-0.0763171,0.0651080},
{-0.0674356,-0.0763171,-0.0651080},
{-0.0602364,-0.0751827,0.0490600},
{-0.0602364,-0.0751827,-0.0490600},
{-0.0604272,-0.0801096,0.0617610},
{-0.0604272,-0.0801096,-0.0617610},
{-0.0655251,0.0632155,0.1093750},
{-0.0655251,0.0632155,-0.1093750},
{-0.0665046,0.0014289,0.0572170},
{-0.0665046,0.0014289,-0.0572170},
{-0.1053786,0.0347803,0.0071580},
{-0.1053786,0.0347803,-0.0071580},
{-0.1321649,-0.0069725,0.0058340},
{-0.1321649,-0.0069725,-0.0058340},
{-0.1232735,-0.0434903,0.0301110},
{-0.1232735,-0.0434903,-0.0301110},
{-0.0497024,0.0261862,0.0084640},

```
    {-0.0497024,0.0261862,-0.0084640},
    {-0.0887827,0.0446048,0.0327030},
    {-0.0887827,0.0446048,-0.0327030},
    {-0.1153998,-0.0126010,0.0290730},
    {-0.1153998,-0.0126010,-0.0290730},
    {-0.0899939,0.0093882,0.0208440},
    {-0.0899939,0.0093882,-0.0208440},
    {-0.1312654,-0.0574898,0.0094690},
    {-0.1312654,-0.0574898,-0.0094690},
    {-0.0996029,0.0042806,0.0105620},
    {-0.0996029,0.0042806,-0.0105620},
    {-0.1226177,-0.0324069,0.0331630},
    {-0.1226177,-0.0324069,-0.0331630},
    {-0.1097808,-0.0051619,0.0102520},
    {-0.1097808,-0.0051619,-0.0102520}
    };
Points1 = {
    {0.0000000,0.0000000,0.1191082},
    {-0.0000007,-0.0874313,0.0173437},
    {-0.0298674,-0.0678926,0.0828862},
    {0.0000000,0.0000000,-0.1191082},
    {-0.0000007,-0.0874313,-0.0173437},
    {-0.0298674,-0.0678926,-0.0828862},
    {0.0000000,0.0000000,0.0000000},
    {-0.0815518,-0.0285519,0.0403460},
    {-0.0815518,-0.0285519,-0.0403460},
    {-0.0772983,-0.0960214,0.0589079},
    {-0.0772983,-0.0960214,-0.0589079},
    {-0.0530680,0.0401558,0.0868696},
    {-0.0530680,0.0401558,-0.0868696},
    {-0.0860948,-0.0071531,0.0357709},
    {-0.0860948,-0.0071531,-0.0357709},
    {-0.0781536,0.0150409,0.0474953},
    {-0.0781536,0.0150409,-0.0474953},
    {-0.0327619,0.0308216,0.1233344},
    {-0.0327619,0.0308216,-0.1233344},
    {-0.0213503,0.0311328,0.1273832},
    {-0.0213503,0.0311328,-0.1273832},
    {-0.0577169,-0.0387406,0.0653973},
    {-0.0577169,-0.0387406,-0.0653973},
    {-0.0721118,-0.0708189,0.0604956},
    {-0.0721118,-0.0708189,-0.0604956},
    {-0.0107463,-0.0379610,0.0894223},
    {-0.0107463,-0.0379610,-0.0894223},
    {-0.0499458,-0.1209236,0.0325105},
    {-0.0499458,-0.1209236,-0.0325105},
```

Biology Open • Supplementary information

{-0.0675660,-0.1120979,0.0619508},
{-0.0675660,-0.1120979,-0.0619508},
{-0.0281000,-0.1158838,0.0181379},
{-0.0281000,-0.1158838,-0.0181379},
{-0.0449094,-0.0276099,0.0631896},
{-0.0449094,-0.0276099,-0.0631896},
{-0.0142784,-0.0797192,0.0327610},
{-0.0142784,-0.0797192,-0.0327610},
{-0.0150259,-0.0566656,0.0702376},
{-0.0150259,-0.0566656,-0.0702376},
{-0.0278969,-0.0771664,0.0538912},
{-0.0278969,-0.0771664,-0.0538912},
{-0.0233532,-0.0568849,0.0546602},
{-0.0233532,-0.0568849,-0.0546602},
{-0.0473534,-0.0636806,0.0871776},
{-0.0473534,-0.0636806,-0.0871776},
{-0.0068313,-0.0969962,0.0074466},
{-0.0068313,-0.0969962,-0.0074466},
{-0.0159667,-0.0682797,0.0639899},
{-0.0159667,-0.0682797,-0.0639899},
{-0.0345871,-0.0009590,0.1014430},
{-0.0345871,-0.0009590,-0.1014430},
{-0.0369160,0.0383298,0.1125242},
{-0.0369160,0.0383298,-0.1125242},
{-0.0586402,-0.0137664,0.0662765},
{-0.0586402,-0.0137664,-0.0662765},
{-0.0561584,0.0135230,0.0477614},
{-0.0561584,0.0135230,-0.0477614},
{-0.0509905,-0.0098838,0.0537391},
{-0.0509905,-0.0098838,-0.0537391},
{-0.0613828,-0.0222834,0.0446180},
{-0.0613828,-0.0222834,-0.0446180},
{-0.0297288,-0.0487831,0.0901825},
{-0.0297288,-0.0487831,-0.0901825},
{-0.0342538,-0.0419889,0.0644480},
{-0.0342538,-0.0419889,-0.0644480},
{-0.0276566,-0.0337192,0.0905353},
{-0.0276566,-0.0337192,-0.0905353},
{-0.0353885,-0.0564500,0.0660367},
{-0.0353885,-0.0564500,-0.0660367},
{-0.0478444,-0.0990574,0.0506250},
{-0.0478444,-0.0990574,-0.0506250},
{-0.0466003,-0.0778580,0.0792973},
{-0.0466003,-0.0778580,-0.0792973},
{-0.0355312,-0.0847020,0.0706947},
{-0.0355312,-0.0847020,-0.0706947},

{-0.0726608,-0.0166618,0.0549565},
{-0.0726608,-0.0166618,-0.0549565},
{-0.0071095,0.0183490,0.1277078},
{-0.0071095,0.0183490,-0.1277078},
{-0.0012775,0.0115692,0.1261366},
{-0.0012775,0.0115692,-0.1261366},
{-0.0507399,-0.0148244,0.0524223},
{-0.0507399,-0.0148244,-0.0524223},
{-0.0606592,-0.0733987,0.0600931},
{-0.0606592,-0.0733987,-0.0600931},
{-0.0643681,-0.0587195,0.0628339},
{-0.0643681,-0.0587195,-0.0628339},
{-0.0245322,-0.0505980,0.0897468},
{-0.0245322,-0.0505980,-0.0897468},
{-0.0300363,-0.0498713,0.0806550},
{-0.0300363,-0.0498713,-0.0806550},
{-0.0126754,-0.0705933,0.0396930},
{-0.0126754,-0.0705933,-0.0396930},
{-0.0161434,-0.0770317,0.0470304},
{-0.0161434,-0.0770317,-0.0470304},
{-0.0202025,-0.1024729,0.0165248},
{-0.0202025,-0.1024729,-0.0165248},
{-0.0149965,-0.1057016,0.0064553},
{-0.0149965,-0.1057016,-0.0064553},
{-0.0017360,-0.0861943,0.0059904},
{-0.0017360,-0.0861943,-0.0059904},
{-0.0729355,-0.0341632,0.0501406},
{-0.0729355,-0.0341632,-0.0501406},
{-0.0802924,-0.0188347,0.0425079},
{-0.0802924,-0.0188347,-0.0425079},
{-0.0788283,-0.0113151,0.0273301},
{-0.0788283,-0.0113151,-0.0273301},
{-0.0711058,-0.0726449,0.0592188},
{-0.0711058,-0.0726449,-0.0592188},
{-0.0527847,-0.0711467,0.0807076},
{-0.0527847,-0.0711467,-0.0807076},
{-0.0315875,-0.0742164,0.0585209},
{-0.0315875,-0.0742164,-0.0585209},
{-0.0419800,-0.0758932,0.0705846},
{-0.0419800,-0.0758932,-0.0705846},
{-0.0375458,-0.0738977,0.0551602},
{-0.0375458,-0.0738977,-0.0551602},
{-0.0363715,-0.0785076,0.0680884},
{-0.0363715,-0.0785076,-0.0680884},
{-0.0457217,0.0350710,0.1045298},
{-0.0457217,0.0350710,-0.1045298},

Biology Open • Supplementary information

```
                    {-0.0539544,-0.0185500,0.0486951},
                    {-0.0539544,-0.0185500,-0.0486951},
                    {-0.0746237,-0.0058647,0.0054923},
                    {-0.0746237,-0.0058647,-0.0054923},
                    {-0.0912030,-0.0370243,0.0043705},
                    {-0.0912030,-0.0370243,-0.0043705},
                    {-0.0903014,-0.0640990,0.0205171},
                    {-0.0903014,-0.0640990,-0.0205171},
                    {-0.0407076,-0.0067009,0.0074651},
                    {-0.0407076,-0.0067009,-0.0074651},
                    {-0.0652920,0.0060167,0.0270494},
                    {-0.0652920,0.0060167,-0.0270494},
                    {-0.0808870,-0.0385399,0.0247296},
                    {-0.0808870,-0.0385399,-0.0247296},
                    {-0.0652189,-0.0222871,0.0175053},
                    {-0.0652189,-0.0222871,-0.0175053},
                    {-0.0962548,-0.0750009,0.0064124},
                    {-0.0962548,-0.0750009,-0.0064124},
                    {-0.0694491,-0.0286186,0.0087838},
                    {-0.0694491,-0.0286186,-0.0087838},
                    {-0.0882039,-0.0545432,0.0261205},
                    {-0.0882039,-0.0545432,-0.0261205},
                    {-0.0750038,-0.0363128,0.0084164},
                    {-0.0750038,-0.0363128,-0.0084164}
                    };
            BoundingBoxOnOff = Off;
        };
        AnyFunTransform3DIdentity ScaleFunction = {
            PreTransforms = {&.RBFTransform};
};
};
AnyFolder Sacrum = {
        AnyFunTransform3DRBF RBFTransform = {
            RBFDef.Type = RBF_ThinPlate;
            PolynomDegree = 1;
            Points0 = {
                    {0.0000000,0.0000000,0.1177000},
                    {-0.0000000,-0.0832729,0.0191000},
                    {-0.0508179,-0.0694062,0.0815920},
                    {0.0000000,0.0000000,-0.1177000},
                    {-0.0000000,-0.0832729,-0.0191000},
                    {-0.0508179,-0.0694062,-0.0815920},
                    {0.0000000,0.0000000,0.0000000},
                    {-0.1164020,-0.0039449,0.0456760},
                    {-0.1164020,-0.0039449,-0.0456760},
                    {-0.1092909,-0.0992949,0.0515590},
```

Biology Open • Supplementary information

{-0.1092909,-0.0992949,-0.0515590},
{-0.0733749,0.0757445,0.0915590},
{-0.0733749,0.0757445,-0.0915590},
{-0.1241738,0.0316243,0.0458050},
{-0.1241738,0.0316243,-0.0458050},
{-0.1108008,0.0563490,0.0555690},
{-0.1108008,0.0563490,-0.0555690},
{-0.0545356,0.0481653,0.1291690},
{-0.0545356,0.0481653,-0.1291690},
{-0.0360236,0.0456352,0.1316250},
{-0.0360236,0.0456352,-0.1316250},
{-0.0804664,-0.0228481,0.0672140},
{-0.0804664,-0.0228481,-0.0672140},
{-0.1004070,-0.0623213,0.0494990},
{-0.1004070,-0.0623213,-0.0494990},
{-0.0174901,-0.0357786,0.0977400},
{-0.0174901,-0.0357786,-0.0977400},
{-0.0665950,-0.1308418,0.0278190},
{-0.0665950,-0.1308418,-0.0278190},
{-0.0962739,-0.1257274,0.0551670},
{-0.0962739,-0.1257274,-0.0551670},
{-0.0393340,-0.1196098,0.0159820},
{-0.0393340,-0.1196098,-0.0159820},
{-0.0585979,-0.0148191,0.0651020},
{-0.0585979,-0.0148191,-0.0651020},
{-0.0238420,-0.0800479,0.0351680},
{-0.0238420,-0.0800479,-0.0351680},
{-0.0234553,-0.0576924,0.0715330},
{-0.0234553,-0.0576924,-0.0715330},
{-0.0472319,-0.0833276,0.0513790},
{-0.0472319,-0.0833276,-0.0513790},
{-0.0327175,-0.0506481,0.0520240},
{-0.0327175,-0.0506481,-0.0520240},
{-0.0746024,-0.0627219,0.0928110},
{-0.0746024,-0.0627219,-0.0928110},
{-0.0119676,-0.0928080,0.0076900},
{-0.0119676,-0.0928080,-0.0076900},
{-0.0263299,-0.0760728,0.0667080},
{-0.0263299,-0.0760728,-0.0667080},
{-0.0498842,0.0119450,0.1070630},
{-0.0498842,0.0119450,-0.1070630},
{-0.0553874,0.0627474,0.1172390},
{-0.0553874,0.0627474,-0.1172390},
{-0.0786774,0.0073556,0.0741800},
{-0.0786774,0.0073556,-0.0741800},
{-0.0742762,0.0474272,0.0542330},

{-0.0742762,0.0474272,-0.0542330},
{-0.0634121,0.0117185,0.0618430},
{-0.0634121,0.0117185,-0.0618430},
{-0.0813084,0.0007821,0.0508130},
{-0.0813084,0.0007821,-0.0508130},
{-0.0496423,-0.0444458,0.0983920},
{-0.0496423,-0.0444458,-0.0983920},
{-0.0480005,-0.0338999,0.0625080},
{-0.0480005,-0.0338999,-0.0625080},
{-0.0428000,-0.0273188,0.0973490},
{-0.0428000,-0.0273188,-0.0973490},
{-0.0548383,-0.0518766,0.0624050},
{-0.0548383,-0.0518766,-0.0624050},
{-0.0696448,-0.1052827,0.0436200},
{-0.0696448,-0.1052827,-0.0436200},
{-0.0739608,-0.0803177,0.0785950},
{-0.0739608,-0.0803177,-0.0785950},
{-0.0586066,-0.0884764,0.0653190},
{-0.0586066,-0.0884764,-0.0653190},
{-0.1017003,0.0085215,0.0624150},
{-0.1017003,0.0085215,-0.0624150},
{-0.0126252,0.0252852,0.1292530},
{-0.0126252,0.0252852,-0.1292530},
{-0.0030963,0.0150156,0.1262640},
{-0.0030963,0.0150156,-0.1262640},
{-0.0621797,0.0048743,0.0605920},
{-0.0621797,0.0048743,-0.0605920},
{-0.0870082,-0.0684280,0.0489900},
{-0.0870082,-0.0684280,-0.0489900},
{-0.0916211,-0.0478344,0.0575290},
{-0.0916211,-0.0478344,-0.0575290},
{-0.0428573,-0.0467026,0.0985250},
{-0.0428573,-0.0467026,-0.0985250},
{-0.0483869,-0.0455474,0.0848580},
{-0.0483869,-0.0455474,-0.0848580},
{-0.0203929,-0.0681811,0.0426070},
{-0.0203929,-0.0681811,-0.0426070},
{-0.0271973,-0.0807786,0.0491650},
{-0.0271973,-0.0807786,-0.0491650},
{-0.0325935,-0.1026813,0.0148280},
{-0.0325935,-0.1026813,-0.0148280},
{-0.0273900,-0.1059173,0.0048750},
{-0.0273900,-0.1059173,-0.0048750},
{-0.0015971,-0.0778770,0.0065530},
{-0.0015971,-0.0778770,-0.0065530},
{-0.1034983,-0.0140026,0.0521800},

Biology Open • Supplementary information

{-0.1034983,-0.0140026,-0.0521800},
{-0.1141160,0.0077734,0.0497600},
{-0.1141160,0.0077734,-0.0497600},
{-0.1123797,0.0244808,0.0350160},
{-0.1123797,0.0244808,-0.0350160},
{-0.0984470,-0.0648210,0.0457100},
{-0.0984470,-0.0648210,-0.0457100},
{-0.0814332,-0.0704479,0.0806410},
{-0.0814332,-0.0704479,-0.0806410},
{-0.0540218,-0.0805070,0.0547320},
{-0.0540218,-0.0805070,-0.0547320},
{-0.0674356,-0.0763171,0.0651080},
{-0.0674356,-0.0763171,-0.0651080},
{-0.0602364,-0.0751827,0.0490600},
{-0.0602364,-0.0751827,-0.0490600},
{-0.0604272,-0.0801096,0.0617610},
{-0.0604272,-0.0801096,-0.0617610},
{-0.0655251,0.0632155,0.1093750},
{-0.0655251,0.0632155,-0.1093750},
{-0.0665046,0.0014289,0.0572170},
{-0.0665046,0.0014289,-0.0572170},
{-0.1053786,0.0347803,0.0071580},
{-0.1053786,0.0347803,-0.0071580},
{-0.1321649,-0.0069725,0.0058340},
{-0.1321649,-0.0069725,-0.0058340},
{-0.1232735,-0.0434903,0.0301110},
{-0.1232735,-0.0434903,-0.0301110},
{-0.0497024,0.0261862,0.0084640},
{-0.0497024,0.0261862,-0.0084640},
{-0.0887827,0.0446048,0.0327030},
{-0.0887827,0.0446048,-0.0327030},
{-0.1153998,-0.0126010,0.0290730},
{-0.1153998,-0.0126010,-0.0290730},
{-0.0899939,0.0093882,0.0208440},
{-0.0899939,0.0093882,-0.0208440},
{-0.1312654,-0.0574898,0.0094690},
{-0.1312654,-0.0574898,-0.0094690},
{-0.0996029,0.0042806,0.0105620},
{-0.0996029,0.0042806,-0.0105620},
{-0.1226177,-0.0324069,0.0331630},
{-0.1226177,-0.0324069,-0.0331630},
{-0.1097808,-0.0051619,0.0102520},
{-0.1097808,-0.0051619,-0.0102520}
};
Points1 = {
{0.0000000,0.0000000,0.1191082},

{-0.0000007,-0.0874313,0.0173437},
{-0.0298674,-0.0678926,0.0828862},
{0.0000000,0.0000000,-0.1191082},
{-0.0000007,-0.0874313,-0.0173437},
{-0.0298674,-0.0678926,-0.0828862},
{0.0000000,0.0000000,0.0000000},
{-0.0815518,-0.0285519,0.0403460},
{-0.0815518,-0.0285519,-0.0403460},
{-0.0772983,-0.0960214,0.0589079},
{-0.0772983,-0.0960214,-0.0589079},
{-0.0530680,0.0401558,0.0868696},
{-0.0530680,0.0401558,-0.0868696},
{-0.0860948,-0.0071531,0.0357709},
{-0.0860948,-0.0071531,-0.0357709},
{-0.0781536,0.0150409,0.0474953},
{-0.0781536,0.0150409,-0.0474953},
{-0.0327619,0.0308216,0.1233344},
{-0.0327619,0.0308216,-0.1233344},
{-0.0213503,0.0311328,0.1273832},
{-0.0213503,0.0311328,-0.1273832},
{-0.0577169,-0.0387406,0.0653973},
{-0.0577169,-0.0387406,-0.0653973},
{-0.0721118,-0.0708189,0.0604956},
{-0.0721118,-0.0708189,-0.0604956},
{-0.0107463,-0.0379610,0.0894223},
{-0.0107463,-0.0379610,-0.0894223},
{-0.0499458,-0.1209236,0.0325105},
{-0.0499458,-0.1209236,-0.0325105},
{-0.0675660,-0.1120979,0.0619508},
{-0.0675660,-0.1120979,-0.0619508},
{-0.0281000,-0.1158838,0.0181379},
{-0.0281000,-0.1158838,-0.0181379},
{-0.0449094,-0.0276099,0.0631896},
{-0.0449094,-0.0276099,-0.0631896},
{-0.0142784,-0.0797192,0.0327610},
{-0.0142784,-0.0797192,-0.0327610},
{-0.0150259,-0.0566656,0.0702376},
{-0.0150259,-0.0566656,-0.0702376},
{-0.0278969,-0.0771664,0.0538912},
{-0.0278969,-0.0771664,-0.0538912},
{-0.0233532,-0.0568849,0.0546602},
{-0.0233532,-0.0568849,-0.0546602},
{-0.0473534,-0.0636806,0.0871776},
{-0.0473534,-0.0636806,-0.0871776},
{-0.0068313,-0.0969962,0.0074466},
{-0.0068313,-0.0969962,-0.0074466},

Biology Open • Supplementary information

{-0.0159667,-0.0682797,0.0639899},
{-0.0159667,-0.0682797,-0.0639899},
{-0.0345871,-0.0009590,0.1014430},
{-0.0345871,-0.0009590,-0.1014430},
{-0.0369160,0.0383298,0.1125242},
{-0.0369160,0.0383298,-0.1125242},
{-0.0586402,-0.0137664,0.0662765},
{-0.0586402,-0.0137664,-0.0662765},
{-0.0561584,0.0135230,0.0477614},
{-0.0561584,0.0135230,-0.0477614},
{-0.0509905,-0.0098838,0.0537391},
{-0.0509905,-0.0098838,-0.0537391},
{-0.0613828,-0.0222834,0.0446180},
{-0.0613828,-0.0222834,-0.0446180},
{-0.0297288,-0.0487831,0.0901825},
{-0.0297288,-0.0487831,-0.0901825},
{-0.0342538,-0.0419889,0.0644480},
{-0.0342538,-0.0419889,-0.0644480},
{-0.0276566,-0.0337192,0.0905353},
{-0.0276566,-0.0337192,-0.0905353},
{-0.0353885,-0.0564500,0.0660367},
{-0.0353885,-0.0564500,-0.0660367},
{-0.0478444,-0.0990574,0.0506250},
{-0.0478444,-0.0990574,-0.0506250},
{-0.0466003,-0.0778580,0.0792973},
{-0.0466003,-0.0778580,-0.0792973},
{-0.0355312,-0.0847020,0.0706947},
{-0.0355312,-0.0847020,-0.0706947},
{-0.0726608,-0.0166618,0.0549565},
{-0.0726608,-0.0166618,-0.0549565},
{-0.0071095,0.0183490,0.1277078},
{-0.0071095,0.0183490,-0.1277078},
{-0.0012775,0.0115692,0.1261366},
{-0.0012775,0.0115692,-0.1261366},
{-0.0507399,-0.0148244,0.0524223},
{-0.0507399,-0.0148244,-0.0524223},
{-0.0606592,-0.0733987,0.0600931},
{-0.0606592,-0.0733987,-0.0600931},
{-0.0643681,-0.0587195,0.0628339},
{-0.0643681,-0.0587195,-0.0628339},
{-0.0245322,-0.0505980,0.0897468},
{-0.0245322,-0.0505980,-0.0897468},
{-0.0300363,-0.0498713,0.0806550},
{-0.0300363,-0.0498713,-0.0806550},
{-0.0126754,-0.0705933,0.0396930},
{-0.0126754,-0.0705933,-0.0396930},

Biology Open • Supplementary information

{-0.0161434,-0.0770317,0.0470304},
{-0.0161434,-0.0770317,-0.0470304},
{-0.0202025,-0.1024729,0.0165248},
{-0.0202025,-0.1024729,-0.0165248},
{-0.0149965,-0.1057016,0.0064553},
{-0.0149965,-0.1057016,-0.0064553},
{-0.0017360,-0.0861943,0.0059904},
{-0.0017360,-0.0861943,-0.0059904},
{-0.0729355,-0.0341632,0.0501406},
{-0.0729355,-0.0341632,-0.0501406},
{-0.0802924,-0.0188347,0.0425079},
{-0.0802924,-0.0188347,-0.0425079},
{-0.0788283,-0.0113151,0.0273301},
{-0.0788283,-0.0113151,-0.0273301},
{-0.0711058,-0.0726449,0.0592188},
{-0.0711058,-0.0726449,-0.0592188},
{-0.0527847,-0.0711467,0.0807076},
{-0.0527847,-0.0711467,-0.0807076},
{-0.0315875,-0.0742164,0.0585209},
{-0.0315875,-0.0742164,-0.0585209},
{-0.0419800,-0.0758932,0.0705846},
{-0.0419800,-0.0758932,-0.0705846},
{-0.0375458,-0.0738977,0.0551602},
{-0.0375458,-0.0738977,-0.0551602},
{-0.0363715,-0.0785076,0.0680884},
{-0.0363715,-0.0785076,-0.0680884},
{-0.0457217,0.0350710,0.1045298},
{-0.0457217,0.0350710,-0.1045298},
{-0.0539544,-0.0185500,0.0486951},
{-0.0539544,-0.0185500,-0.0486951},
{-0.0746237,-0.0058647,0.0054923},
{-0.0746237,-0.0058647,-0.0054923},
{-0.0912030,-0.0370243,0.0043705},
{-0.0912030,-0.0370243,-0.0043705},
{-0.0903014,-0.0640990,0.0205171},
{-0.0903014,-0.0640990,-0.0205171},
{-0.0407076,-0.0067009,0.0074651},
{-0.0407076,-0.0067009,-0.0074651},
{-0.0652920,0.0060167,0.0270494},
{-0.0652920,0.0060167,-0.0270494},
{-0.0808870,-0.0385399,0.0247296},
{-0.0808870,-0.0385399,-0.0247296},
{-0.0652189,-0.0222871,0.0175053},
{-0.0652189,-0.0222871,-0.0175053},
{-0.0962548,-0.0750009,0.0064124},
{-0.0962548,-0.0750009,-0.0064124},

```
                    {-0.0694491,-0.0286186,0.0087838},
                    {-0.0694491,-0.0286186,-0.0087838},
                    {-0.0882039,-0.0545432,0.0261205},
                    {-0.0882039,-0.0545432,-0.0261205},
                    {-0.0750038,-0.0363128,0.0084164},
                    {-0.0750038,-0.0363128,-0.0084164}
                    };
            BoundingBoxOnOff = Off;
        };
        AnyFunTransform3DIdentity ScaleFunction = {
            PreTransforms = {&.RBFTransform};
};
};

};
```

Biology Open • Supplementary information

ScalingFunctionTLEMLucyFemur_2014019

```
AnyFolder ScalingFunctionTLEMLucyFemur = {
  AnyFolder Right = {
    AnyFolder Thigh = {
        AnyFunTransform3DRBF RBFTransform = {
            RBFDef.Type = RBF_ThinPlate;
            PolynomDegree = 1;
            Points0 = {
                {0.0000000,0.0000000,0.0000000},
                {-0.0000000,-0.3616821,0.0000000},
                {-0.0097563,-0.3678799,0.0012967},
                {-0.0000000,-0.3660632,0.0408203},
                {-0.0000000,-0.3573010,-0.0408203},
                {0.0161460,-0.0072838,0.0601290},
                {0.0220217,-0.0203698,0.0463848},
                {0.0123977,-0.0241932,0.0668573},
                {0.0006898,0.0018121,0.0538181},
                {0.0122809,-0.0068668,0.0414535},
                {-0.0058991,-0.0138188,0.0648412},
                {0.0211469,-0.0380855,0.0538111},
                {0.0172133,-0.0317342,0.0328381},
                {0.0177311,-0.0131946,0.0256176},
                {-0.0110079,-0.0308867,0.0632370},
                {0.0039435,-0.0395977,0.0638271},
                {-0.0041683,-0.0187472,0.0413214},
                {-0.0177999,-0.0021535,0.0496084},
                {0.0001603,-0.0053126,0.0279593},
                {-0.0211600,-0.0216243,0.0497147},
                {0.0096163,-0.0568663,0.0545719},
                {0.0162112,-0.0515013,0.0395230},
                {0.0093533,-0.0433265,0.0187387},
                {0.0119398,-0.0238469,0.0118403},
                {0.0210707,-0.0048848,0.0065878},
                {0.0125013,0.0064094,0.0177702},
                {-0.0191523,-0.0356053,0.0382429},
                {-0.0115821,-0.0474937,0.0537648},
                {-0.0100073,-0.0236371,0.0267322},
                {-0.0078925,0.0107098,0.0182833},
                {-0.0134217,-0.0055356,0.0182000},
                {-0.0090061,-0.0665700,0.0475484},
                {0.0071095,-0.0761976,0.0520035},
                {0.0182119,-0.0704149,0.0386546},
                {0.0073764,-0.0609481,0.0221006},
                {-0.0122984,-0.0439585,0.0198428},
                {-0.0032253,-0.0306792,0.0110995},
```

{-0.0012975,-0.0198451,-0.0052592},
{0.0140405,-0.0161125,-0.0071649},
{0.0189222,-0.0014898,-0.0127226},
{0.0181642,0.0132812,-0.0011050},
{0.0045119,0.0200001,0.0091189},
{-0.0176761,-0.0541983,0.0348587},
{-0.0136548,-0.0142015,0.0054311},
{-0.0135510,0.0182940,0.0017175},
{-0.0223865,0.0023399,0.0028018},
{-0.0086455,-0.0738247,0.0283843},
{-0.0050356,-0.0877749,0.0442195},
{0.0160135,-0.0919319,0.0465120},
{0.0128290,-0.0785810,0.0232431},
{-0.0168328,-0.0616327,0.0155917},
{-0.0172369,-0.0094567,-0.0119502},
{0.0002090,-0.0093641,-0.0209779},
{0.0064827,0.0081538,-0.0198453},
{0.0010777,0.0210422,-0.0084721},
{-0.0129485,0.0085451,-0.0162212},
{-0.0002322,-0.0924033,0.0245644},
{-0.0028175,-0.1052263,0.0418294},
{0.0172322,-0.1111444,0.0445760},
{0.0203259,-0.0957409,0.0271449},
{0.0057102,-0.1088933,0.0195113},
{-0.0010852,-0.1230776,0.0363132},
{0.0182727,-0.1303312,0.0422884},
{0.0242891,-0.1161703,0.0265054},
{0.0094531,-0.1268584,0.0162294},
{0.0014244,-0.1413293,0.0331653},
{0.0207271,-0.1496427,0.0394157},
{0.0267562,-0.1360142,0.0236798},
{0.0113839,-0.1461651,0.0136024},
{0.0030845,-0.1595094,0.0297156},
{0.0209528,-0.1691386,0.0369775},
{0.0283958,-0.1540209,0.0213795},
{0.0129499,-0.1649012,0.0113589},
{0.0016490,-0.1783108,0.0216287},
{0.0147105,-0.1880678,0.0328614},
{0.0298491,-0.1859865,0.0248551},
{0.0286645,-0.1714377,0.0169488},
{0.0165897,-0.1861006,0.0082595},
{0.0014000,-0.1973476,0.0183740},
{0.0114301,-0.2109792,0.0286157},
{0.0267422,-0.2037622,0.0296951},
{0.0288630,-0.2018335,0.0130127},
{0.0145471,-0.2083505,0.0062833},

{0.0011300,-0.2198071,0.0155602},
{0.0126610,-0.2321235,0.0275354},
{0.0275647,-0.2219268,0.0265185},
{0.0286645,-0.2230253,0.0098701},
{0.0130434,-0.2304822,0.0039177},
{0.0022984,-0.2409585,0.0149650},
{0.0101315,-0.2534740,0.0252125},
{0.0279485,-0.2428555,0.0234276},
{0.0276684,-0.2421559,0.0058691},
{0.0101250,-0.2529527,0.0018983},
{0.0021376,-0.2667831,0.0107073},
{0.0114309,-0.2734905,0.0249960},
{0.0273923,-0.2634953,0.0216324},
{0.0278429,-0.2592546,0.0037692},
{0.0159101,-0.2712985,-0.0033988},
{0.0036708,-0.2863280,0.0004644},
{0.0023115,-0.2884756,0.0187554},
{0.0231385,-0.2879051,0.0224853},
{0.0304009,-0.2799973,0.0083549},
{0.0238189,-0.2917246,-0.0052462},
{0.0064904,-0.3027687,-0.0070587},
{-0.0003492,-0.3064690,0.0093014},
{0.0122179,-0.3045269,0.0243511},
{0.0301837,-0.3030127,0.0114462},
{0.0234182,-0.3107336,-0.0085198},
{0.0046893,-0.3214995,-0.0123881},
{-0.0022352,-0.3264905,0.0043340},
{0.0016841,-0.3202756,0.0217839},
{0.0229722,-0.3196887,0.0215083},
{0.0292438,-0.3228042,0.0051430},
{0.0221641,-0.3296190,-0.0132361},
{0.0100459,-0.3417597,-0.0244560},
{-0.0053009,-0.3375737,-0.0137705},
{-0.0064432,-0.3487307,0.0028676},
{-0.0059346,-0.3407801,0.0202938},
{0.0105130,-0.3365976,0.0264175},
{0.0282695,-0.3386249,0.0180307},
{0.0270402,-0.3431844,-0.0001487},
{0.0257040,-0.3506950,-0.0181851},
{0.0178948,-0.3636114,-0.0302838},
{0.0043149,-0.3544109,-0.0381949},
{-0.0095210,-0.3461482,-0.0295031},
{-0.0164526,-0.3542895,-0.0126666},
{-0.0060896,-0.3690127,-0.0033061},
{-0.0176523,-0.3612686,0.0110109},
{-0.0153310,-0.3546943,0.0299634},

Biology Open • Supplementary information

{0.0021329,-0.3508021,0.0350763},
{0.0210690,-0.3548628,0.0279835},
{0.0356594,-0.3558162,0.0159356},
{0.0275718,-0.3617450,-0.0018607},
{0.0298503,-0.3741530,-0.0163721},
{0.0175320,-0.3855300,-0.0277515},
{0.0025604,-0.3740099,-0.0397137},
{-0.0136727,-0.3624016,-0.0402102},
{-0.0277647,-0.3567472,-0.0284947},
{-0.0275446,-0.3699630,-0.0120829},
{-0.0175846,-0.3859355,-0.0177922},
{0.0001572,-0.3856873,-0.0118050},
{0.0030262,-0.3834856,0.0075553},
{-0.0144114,-0.3810887,0.0140304},
{-0.0247706,-0.3701239,0.0285134},
{-0.0044804,-0.3677201,0.0404632},
{0.0133183,-0.3712274,0.0334208},
{0.0324861,-0.3723417,0.0245668},
{0.0271451,-0.3766027,0.0072441},
{0.0167985,-0.3822816,-0.0065349},
{-0.0001454,-0.3910823,-0.0303010},
{-0.0144998,-0.3834154,-0.0384137},
{-0.0301321,-0.3750841,-0.0321336},
{0.0196491,-0.3864804,0.0210853},
{0.0015726,-0.3888034,0.0276569},
{-0.0146652,-0.3847848,0.0336913},
{-0.0014588,-0.0619951,0.0219778},
{-0.0118435,-0.0550626,0.0139639},
{0.0129812,-0.0349966,0.0673128},
{-0.0162907,-0.0105666,0.0597739},
{-0.0012885,-0.0520816,0.0562989},
{-0.0090599,-0.0631298,0.0172224},
{-0.0000222,-0.0771663,0.0231897},
{-0.0068148,0.0023885,-0.0160086},
{-0.0180544,-0.0425409,0.0446585},
{0.0020525,-0.0249904,0.0691549},
{-0.0157097,-0.0662315,0.0238393},
{-0.0010518,-0.0510686,0.0199353},
{-0.0208874,-0.0152865,0.0500034},
{0.0168316,-0.0280857,0.0666680},
{-0.0187392,-0.0282333,0.0403282},
{0.0191622,-0.0351633,0.0622707},
{0.0001216,-0.0706289,0.0524049},
{-0.0169862,-0.0399062,0.0311827},
{-0.0185737,-0.0533066,0.0151224},
{-0.0219058,-0.0528723,0.0222263},

Biology Open • Supplementary information

{-0.0028271,-0.0391620,0.0148452},
{0.0117900,-0.0553425,0.0235348},
{0.0219835,-0.0106400,0.0396338},
{-0.0025366,-0.0401046,0.0625273},
{0.0141017,-0.0018767,0.0198108},
{-0.0078770,-0.0473929,0.0173542},
{-0.0065351,-0.0766431,0.0470258},
{0.0065585,0.0145593,0.0161638},
{-0.0118588,0.0172875,-0.0085064},
{-0.0074087,-0.0627500,0.0506700},
{0.0071940,-0.0780068,0.0213362},
{0.0126736,-0.0708176,0.0244108},
{0.0074694,-0.0436450,0.0604196},
{0.0154186,-0.0139471,0.0064312},
{-0.0053535,-0.0213210,0.0040094},
{-0.0088733,0.0002018,0.0205402},
{-0.0112471,-0.0542608,0.0500266},
{-0.0061625,-0.0248673,0.0653244},
{0.0093695,-0.0065826,0.0614157},
{0.0138015,-0.0467322,0.0284333},
{-0.0020999,-0.0186885,-0.0116392},
{-0.0185640,-0.0225591,0.0566779},
{0.0175798,0.0124360,-0.0068467},
{-0.0039962,0.0217127,0.0053055},
{0.0014975,-0.0784591,0.0514056},
{-0.0172997,-0.0386971,0.0525252},
{0.0196540,-0.0268271,0.0416806},
{0.0135296,-0.0406504,0.0236882},
{0.0182471,0.0095959,0.0087906},
{0.0218667,0.0016804,0.0058793},
{0.0224975,-0.0022652,-0.0022994},
{0.0166750,-0.0191391,0.0236436},
{0.0119785,-0.0328723,0.0195376},
{0.0187631,-0.0133469,0.0308741},
{-0.0187543,-0.0461463,0.0257491},
{0.0039834,-0.0373928,0.0683089},
{0.0157029,-0.0423169,0.0385226},
{0.0164003,-0.0087069,0.0131181},
{0.0008253,0.0033370,-0.0212576},
{0.0101707,-0.0009724,-0.0205688},
{0.0102948,-0.0642683,0.0524532},
{0.0152217,-0.0557944,0.0508978},
{0.0172973,-0.0565707,0.0419468},
{0.0167723,-0.0635334,0.0369834},
{-0.0047397,-0.0695325,0.0241398},
{-0.0152913,0.0035778,0.0161461},

Biology Open • Supplementary information

{0.0091697,0.0009340,0.0575160},
{0.0196686,-0.0089533,0.0500006},
{0.0183998,-0.0212943,0.0622968},
{-0.0114340,-0.0316810,0.0250940},
{-0.0206442,-0.0092599,-0.0028425},
{0.0166868,-0.0784295,0.0276925},
{-0.0069678,-0.0054521,-0.0207011},
{0.0049490,0.0151109,-0.0160093},
{-0.0084025,-0.0318937,0.0162788},
{-0.0173679,0.0095594,-0.0108509},
{-0.0218912,0.0063457,-0.0020698},
{-0.0106547,-0.0668750,0.0397749},
{0.0136098,-0.0170666,-0.0018890},
{0.0141599,-0.0441405,0.0587594},
{0.0093255,-0.0183218,-0.0083008},
{0.0106367,-0.0128276,-0.0154269},
{0.0037350,-0.0549365,0.0209786},
{0.0087662,-0.0472902,0.0195496},
{0.0016839,-0.0708213,0.0221325},
{-0.0202977,-0.0530540,0.0301270},
{-0.0068584,0.0021781,0.0530002},
{-0.0144817,-0.0297218,0.0605833},
{0.0197015,-0.0165217,0.0560654},
{0.0156609,-0.0705363,0.0477736},
{0.0207876,-0.0221433,0.0330733},
{0.0092324,-0.0300767,0.0713160},
{-0.0203392,-0.0034488,0.0097332},
{0.0156978,-0.0143438,0.0629053},
{0.0143267,-0.0563519,0.0317489},
{-0.0161685,-0.0465391,0.0188591},
{-0.0196397,-0.0309875,0.0546496},
{0.0154804,-0.0380422,0.0305433},
{-0.0077774,-0.0351303,0.0637765},
{0.0063980,-0.0086419,-0.0201654},
{0.0030146,-0.0151094,-0.0165974},
{-0.0210080,-0.0096030,0.0507855},
{-0.0105684,-0.0411377,0.0586146},
{0.0180619,-0.0498960,0.0469835},
{-0.0192318,0.0116108,0.0024070},
{-0.0165651,0.0083622,0.0126870},
{-0.0014333,-0.0627138,0.0534746},
{0.0145127,-0.0080178,0.0353667},
{0.0148374,-0.0012228,0.0566181},
{0.0153834,-0.0062427,0.0444727},
{-0.0155164,-0.0132103,-0.0096398},
{0.0068685,-0.0221022,0.0011547},

Biology Open • Supplementary information

{0.0003332,-0.0458500,0.0183716},
{0.0205582,0.0040956,-0.0082969},
{0.0175752,0.0009340,-0.0148407},
{0.0142357,-0.0063623,-0.0169332},
{-0.0147285,0.0023412,-0.0164678},
{-0.0158781,-0.0064451,-0.0151346},
{0.0175951,0.0133456,0.0037559},
{0.0117660,0.0189899,-0.0030690},
{0.0096040,0.0151827,-0.0137450},
{0.0096565,-0.0215194,0.0662734},
{-0.0006793,-0.0074494,0.0631218},
{0.0007819,-0.0004519,0.0600781},
{0.0065377,-0.0125433,0.0654199},
{0.0090461,0.0073096,0.0188711},
{0.0151418,-0.0168302,0.0146465},
{0.0025166,-0.0231863,0.0688894},
{-0.0097891,-0.0152077,0.0253086},
{-0.0109149,-0.0177935,0.0119825},
{-0.0072079,-0.0099373,0.0272725},
{-0.0183727,-0.0467429,0.0356276},
{-0.0126871,-0.0584555,0.0428141},
{-0.0057054,-0.0273042,0.0103554},
{0.0215142,-0.0159693,0.0474531},
{0.0128492,-0.0070016,0.0287645},
{0.0052134,-0.0017047,0.0247463},
{-0.0024284,-0.0707498,0.0513049},
{-0.0036140,0.0220593,-0.0040484},
{-0.0002405,-0.0121253,0.0390050},
{-0.0119811,-0.0156737,0.0015445},
{0.0142082,-0.0279792,0.0246553},
{0.0122244,-0.0262013,0.0169829},
{-0.0146264,-0.0038855,0.0549384},
{0.0207450,-0.0325771,0.0496412},
{-0.0155040,-0.0497560,0.0437087},
{-0.0081119,-0.0756450,0.0387365},
{0.0039684,-0.0062047,0.0313900},
{-0.0023726,-0.0038892,-0.0220521},
{-0.0136977,-0.0339839,0.0602619},
{-0.0035399,0.0089081,-0.0188894},
{-0.0049820,0.0176571,-0.0132254},
{0.0193902,-0.0803191,0.0360429},
{0.0040648,-0.0697109,0.0218205},
{0.0170470,-0.0818610,0.0456892},
{0.0104175,0.0188635,0.0064965},
{0.0135206,0.0092549,0.0158079},
{0.0023933,0.0160498,-0.0157404},

Biology Open • Supplementary information

{0.0164753,-0.0118197,-0.0096995},
{0.0202584,-0.0066858,-0.0082383},
{-0.0098401,-0.0226001,0.0314028},
{-0.0130937,-0.0309622,0.0320764},
{-0.0201510,-0.0613297,0.0222264},
{-0.0010389,0.0036055,0.0209003},
{0.0042025,0.0073392,0.0199393},
{-0.0021630,-0.0038531,0.0251898},
{-0.0007845,-0.0293574,0.0675788},
{0.0070027,-0.0406327,0.0624565},
{-0.0124363,-0.0183001,0.0627966},
{-0.0120485,-0.0256613,0.0628493},
{0.0101200,0.0197695,0.0039334},
{0.0167636,0.0148266,0.0014155},
{-0.0074981,-0.0106930,0.0632797},
{0.0142533,-0.0503445,0.0351954},
{-0.0146107,-0.0460953,0.0506120},
{-0.0161117,-0.0471301,0.0178900},
{0.0112149,-0.0792793,0.0501460},
{-0.0099060,-0.0230794,0.0186951},
{-0.0114543,-0.0153049,0.0183675},
{-0.0042882,0.0161879,0.0151423},
{0.0014582,-0.0638572,0.0541080},
{0.0048554,-0.0582603,0.0553815},
{-0.0017077,-0.0572302,0.0546635},
{0.0166090,-0.0697169,0.0323038},
{-0.0208812,-0.0164273,0.0474354},
{0.0130454,-0.0241864,0.0178294},
{0.0053123,-0.0483770,0.0582251},
{0.0112225,-0.0361429,0.0673300},
{0.0085729,-0.0771256,0.0216100},
{-0.0070783,-0.0488033,0.0560869},
{0.0182083,-0.0411024,0.0454236},
{-0.0119590,-0.0403809,0.0213546},
{0.0233409,-0.0185535,0.0366343},
{-0.0046347,-0.0571211,0.0208243},
{0.0183166,-0.0006966,0.0140116},
{0.0061913,0.0053512,0.0201003},
{-0.0002364,-0.0310828,0.0680351},
{-0.0170773,0.0139270,-0.0054987},
{0.0110805,0.0156034,0.0117010},
{-0.0124628,0.0164667,0.0094550},
{-0.0209014,-0.0073407,-0.0051969},
{-0.0210131,0.0010693,-0.0086891},
{-0.0041596,-0.0172332,0.0663041},
{-0.0216595,-0.0322172,0.0451540},

Biology Open • Supplementary information

{0.0051908,-0.0426014,0.0613013},
{0.0150003,-0.0416136,0.0606257},
{-0.0135090,-0.0674234,0.0236425},
{0.0146416,0.0073691,0.0160052},
{0.0195615,-0.0112412,-0.0010333},
{-0.0198115,-0.0098244,0.0044735},
{-0.0157054,-0.0521236,0.0395039},
{-0.0081381,-0.0173108,-0.0046152},
{-0.0218324,-0.0262661,0.0471115},
{0.0051446,-0.0707257,0.0530555},
{0.0179359,-0.0471493,0.0528134},
{-0.0005591,-0.0317983,0.0113632},
{0.0071707,-0.0305943,0.0121186},
{0.0206239,-0.0061367,0.0066642},
{0.0187057,-0.0745868,0.0420207},
{-0.0095784,-0.0163113,0.0259524},
{-0.0012181,0.0127661,0.0186855},
{0.0059420,-0.0630691,0.0220087},
{0.0069408,-0.0573878,0.0552614},
{0.0150782,-0.0132884,-0.0101793},
{0.0005533,-0.0078574,0.0633775},
{-0.0088284,-0.0146399,-0.0147837},
{0.0167444,-0.0651105,0.0470347},
{0.0082666,-0.0181741,-0.0095133},
{-0.0175162,-0.0637444,0.0177069},
{0.0153795,-0.0360767,0.0287608},
{0.0162363,-0.0018864,0.0548412},
{0.0052731,-0.0075919,0.0392990},
{-0.0022288,-0.0556644,0.0549909},
{-0.0156238,-0.0550038,0.0377688},
{-0.0204137,-0.0100550,0.0536429},
{-0.0093683,0.0122429,-0.0162013},
{-0.0202927,0.0049166,0.0083887},
{0.0074706,-0.0627945,0.0222133},
{0.0035034,-0.0376103,0.0672421},
{-0.0190752,-0.0194129,0.0555530},
{0.0061758,-0.0074961,-0.0207741},
{0.0192011,-0.0273063,0.0588233},
{0.0130796,-0.0134383,0.0638611},
{0.0120421,0.0019566,-0.0194140},
{0.0154152,-0.0065294,0.0179581},
{-0.0157335,0.0101219,0.0123981},
{0.0110376,-0.0488274,0.0567911},
{-0.0055170,-0.0343676,0.0647140},
{0.0216204,-0.0273988,0.0512041},
{0.0161331,-0.0135875,-0.0075501},

Biology Open • Supplementary information

{-0.0184643,-0.0455633,0.0360087},
{-0.0091978,-0.0682413,0.0460847},
{0.0034304,-0.0471677,0.0588866},
{-0.0048025,-0.0384316,0.0149400},
{0.0118505,0.0109735,-0.0159161},
{-0.0027036,-0.0204189,-0.0015094},
{-0.0117607,-0.0692759,0.0292171},
{-0.0107080,-0.0033455,-0.0190487},
{-0.0042548,0.0214947,-0.0056846},
{-0.0097315,-0.0208529,0.0131997},
{-0.0178958,-0.0627218,0.0171551},
{-0.0068692,-0.0688818,0.0233316},
{0.0012083,0.0120695,-0.0186179},
{0.0215073,-0.0236027,0.0503069},
{-0.0060262,-0.0534931,0.0189523},
{0.0179951,-0.0336785,0.0397267},
{0.0190509,-0.0284972,0.0336394},
{-0.0169188,-0.0133609,-0.0014203},
{0.0135722,0.0136491,0.0114695},
{-0.0150549,-0.0123230,0.0067722},
{-0.0148411,-0.0311945,0.0355412},
{0.0185490,-0.0794042,0.0431266},
{0.0193568,-0.0087326,0.0575704},
{0.0201026,-0.0144353,0.0531586},
{0.0025583,-0.0248211,0.0045714},
{0.0132689,-0.0214866,0.0652899},
{-0.0028669,-0.0099311,0.0309271},
{-0.0219487,-0.0054893,-0.0021853},
{0.0026180,-0.0203806,-0.0040620},
{-0.0116230,-0.0668518,0.0370152},
{-0.0133007,-0.0264159,0.0619176},
{0.0186167,0.0050491,-0.0120059},
{-0.0191359,-0.0591690,0.0288149},
{-0.0178121,-0.0041834,0.0141448},
{0.0123077,-0.0063648,0.0279743},
{-0.0157729,-0.0374010,0.0315335},
{0.0149328,-0.0659100,0.0290095},
{0.0006205,0.0021977,-0.0214613},
{0.0170484,-0.0715687,0.0325980},
{0.0068499,0.0061192,0.0196608},
{-0.0167075,0.0020024,0.0150382},
{0.0059801,-0.0393117,0.0167489},
{-0.0072506,-0.0171067,0.0338751},
{0.0202363,-0.0251596,0.0420997},
{-0.0199311,-0.0601255,0.0171522},
{0.0216641,0.0053410,-0.0027841},

{-0.0197665,-0.0606125,0.0252972},
{0.0129986,-0.0214528,0.0098002},
{0.0201151,-0.0100929,0.0460501},
{0.0104137,0.0017765,-0.0203073},
{0.0166557,-0.0064123,0.0140197},
{-0.0124120,-0.0596549,0.0422637},
{0.0014885,0.0202590,0.0096781},
{0.0028720,0.0020207,-0.0219044},
{0.0376095,-0.3675427,0.0214537},
{0.0049949,0.0206417,-0.0079017},
{-0.0191723,-0.0407590,0.0391310},
{-0.0316406,-0.3638215,-0.0274841},
{0.0021392,0.0226920,0.0000985},
{0.0168435,-0.0229382,0.0264788},
{-0.0157304,-0.0005437,-0.0161456},
{0.0203722,-0.0299750,0.0562762},
{-0.0171077,0.0134146,0.0059615},
{0.0175519,-0.0370653,0.0413374},
{0.0154657,-0.0405127,0.0348980},
{-0.0215172,-0.0323502,0.0481925},
{0.0177998,-0.0515776,0.0444709},
{-0.0183804,-0.0448316,0.0275139},
{0.0033607,-0.0502028,0.0575894},
{0.0010325,-0.0479377,0.0191141},
{0.0224852,-0.0181821,0.0451780},
{0.0084089,-0.0720538,0.0521031},
{-0.0070880,-0.0772912,0.0288023},
{-0.0223712,-0.0028990,0.0024047},
{0.0173136,-0.0120017,0.0204612},
{0.0137772,-0.0623537,0.0276621},
{-0.0100390,-0.0231180,0.0292222},
{-0.0196395,-0.3730207,0.0364730},
{-0.0094445,-0.0526688,0.0528111},
{-0.0115309,-0.3679776,-0.0104995},
{0.0129812,-0.0410477,0.0224072},
{0.0117394,0.0154849,-0.0117191},
{-0.0245208,-0.3649412,0.0295111},
{0.0147476,-0.0502966,0.0364899},
{0.0006132,-0.0446771,0.0600524},
{-0.0118123,0.0191139,0.0031977},
{-0.0017185,-0.0163303,0.0664216},
{-0.0085098,-0.0074564,0.0247745},
{0.0197459,-0.0086852,0.0430460},
{0.0187064,-0.0223532,0.0600966},
{-0.0206552,-0.0099712,0.0525024},
{0.0207525,0.0084646,-0.0024846},

Biology Open • Supplementary information

{-0.0094915,-0.0325446,0.0184441},
{-0.0014464,-0.0140050,-0.0179819},
{-0.0122725,-0.0573444,0.0463125},
{-0.0106397,0.0177211,0.0094669},
{0.0111981,0.0191236,-0.0041491},
{-0.0162790,-0.0256279,0.0592687},
{-0.0060120,-0.3653959,0.0413411},
{-0.0135007,-0.3703638,-0.0387476},
{-0.0134258,-0.3703142,-0.0379467},
{-0.0133509,-0.3702645,-0.0371458},
{-0.0132760,-0.3702148,-0.0363450},
{-0.0132011,-0.3701651,-0.0355441},
{-0.0131262,-0.3701154,-0.0347432},
{-0.0130513,-0.3700658,-0.0339423},
{-0.0129765,-0.3700161,-0.0331414},
{-0.0129016,-0.3699664,-0.0323405},
{-0.0128267,-0.3699167,-0.0315396},
{-0.0127518,-0.3698670,-0.0307388},
{-0.0126769,-0.3698174,-0.0299379},
{-0.0126020,-0.3697677,-0.0291370},
{-0.0125271,-0.3697180,-0.0283361},
{-0.0124523,-0.3696683,-0.0275352},
{-0.0123774,-0.3696187,-0.0267343},
{-0.0123025,-0.3695690,-0.0259334},
{-0.0122276,-0.3695193,-0.0251325},
{-0.0121527,-0.3694696,-0.0243317},
{-0.0120778,-0.3694199,-0.0235308},
{-0.0120029,-0.3693703,-0.0227299},
{-0.0119280,-0.3693206,-0.0219290},
{-0.0118532,-0.3692709,-0.0211281},
{-0.0117783,-0.3692212,-0.0203272},
{-0.0117034,-0.3691715,-0.0195263},
{-0.0116285,-0.3691219,-0.0187255},
{-0.0115536,-0.3690722,-0.0179246},
{-0.0114787,-0.3690225,-0.0171237},
{-0.0114038,-0.3689728,-0.0163228},
{-0.0113289,-0.3689231,-0.0155219},
{-0.0112541,-0.3688735,-0.0147210},
{-0.0111792,-0.3688238,-0.0139201},
{-0.0111043,-0.3687741,-0.0131192},
{-0.0110294,-0.3687244,-0.0123184},
{-0.0109545,-0.3686748,-0.0115175},
{-0.0108796,-0.3686251,-0.0107166},
{-0.0108047,-0.3685754,-0.0099157},
{-0.0107298,-0.3685257,-0.0091148},
{-0.0106550,-0.3684760,-0.0083139},

{-0.0105801,-0.3684264,-0.0075130},
{-0.0105052,-0.3683767,-0.0067121},
{-0.0104303,-0.3683270,-0.0059113},
{-0.0103554,-0.3682773,-0.0051104},
{-0.0102805,-0.3682276,-0.0043095},
{-0.0102056,-0.3681780,-0.0035086},
{-0.0101307,-0.3681283,-0.0027077},
{-0.0100559,-0.3680786,-0.0019068},
{-0.0099810,-0.3680289,-0.0011059},
{-0.0099061,-0.3679792,-0.0003051},
{-0.0098312,-0.3679296,0.0004958},
{-0.0097563,-0.3678799,0.0012967},
{-0.0096814,-0.3678302,0.0020976},
{-0.0096065,-0.3677805,0.0028985},
{-0.0095317,-0.3677308,0.0036994},
{-0.0094568,-0.3676812,0.0045003},
{-0.0093819,-0.3676315,0.0053012},
{-0.0093070,-0.3675818,0.0061020},
{-0.0092321,-0.3675321,0.0069029},
{-0.0091572,-0.3674825,0.0077038},
{-0.0090823,-0.3674328,0.0085047},
{-0.0090074,-0.3673831,0.0093056},
{-0.0089326,-0.3673334,0.0101065},
{-0.0088577,-0.3672837,0.0109074},
{-0.0087828,-0.3672341,0.0117082},
{-0.0087079,-0.3671844,0.0125091},
{-0.0086330,-0.3671347,0.0133100},
{-0.0085581,-0.3670850,0.0141109},
{-0.0084832,-0.3670353,0.0149118},
{-0.0084083,-0.3669857,0.0157127},
{-0.0083335,-0.3669360,0.0165136},
{-0.0082586,-0.3668863,0.0173145},
{-0.0081837,-0.3668366,0.0181153},
{-0.0081088,-0.3667869,0.0189162},
{-0.0080339,-0.3667373,0.0197171},
{-0.0079590,-0.3666876,0.0205180},
{-0.0078841,-0.3666379,0.0213189},
{-0.0078092,-0.3665882,0.0221198},
{-0.0077344,-0.3665386,0.0229207},
{-0.0076595,-0.3664889,0.0237215},
{-0.0075846,-0.3664392,0.0245224},
{-0.0075097,-0.3663895,0.0253233},
{-0.0074348,-0.3663398,0.0261242},
{-0.0073599,-0.3662902,0.0269251},
{-0.0072850,-0.3662405,0.0277260},
{-0.0072101,-0.3661908,0.0285269},

```
{-0.0071353,-0.3661411,0.0293278},
{-0.0070604,-0.3660914,0.0301286},
{-0.0069855,-0.3660418,0.0309295},
{-0.0069106,-0.3659921,0.0317304},
{-0.0068357,-0.3659424,0.0325313},
{-0.0067608,-0.3658927,0.0333322},
{-0.0066859,-0.3658430,0.0341331},
{-0.0066111,-0.3657934,0.0349340},
{-0.0065362,-0.3657437,0.0357348},
{-0.0064613,-0.3656940,0.0365357},
{-0.0063864,-0.3656443,0.0373366},
{-0.0063115,-0.3655947,0.0381375},
{-0.0062366,-0.3655450,0.0389384},
{-0.0061617,-0.3654953,0.0397393},
{-0.0060868,-0.3654456,0.0405402}
};
Points1 = {
{0.0000000,0.0000000,0.0000000},
{-0.0000000,-0.4251555,0.0000000},
{-0.0096030,-0.4324410,0.0012763},
{-0.0000000,-0.4303055,0.0401789},
{-0.0000000,-0.4200055,-0.0401789},
{0.0142957,-0.0095543,0.0576365},
{0.0207430,-0.0248489,0.0453625},
{0.0099692,-0.0281849,0.0628768},
{0.0001611,-0.0004395,0.0513050},
{0.0115772,-0.0075574,0.0399826},
{-0.0061031,-0.0154785,0.0652108},
{0.0173623,-0.0427821,0.0519580},
{0.0155558,-0.0371484,0.0333871},
{0.0146935,-0.0159955,0.0252592},
{-0.0104552,-0.0355272,0.0622214},
{0.0034635,-0.0465071,0.0611948},
{-0.0031709,-0.0222696,0.0415054},
{-0.0128714,-0.0047794,0.0483395},
{-0.0000091,-0.0046883,0.0280461},
{-0.0126391,-0.0253605,0.0496912},
{0.0103840,-0.0673100,0.0545877},
{0.0165141,-0.0597973,0.0388886},
{0.0086086,-0.0504923,0.0194999},
{0.0099556,-0.0268907,0.0127212},
{0.0188649,-0.0061009,0.0058149},
{0.0106750,0.0067264,0.0162281},
{-0.0125735,-0.0423024,0.0369799},
{-0.0103291,-0.0549908,0.0525409},
{-0.0094824,-0.0283071,0.0253857},
```

Biology Open • Supplementary information

{-0.0062957,0.0118408,0.0162577},
{-0.0116836,-0.0065143,0.0160262},
{-0.0076466,-0.0777678,0.0455642},
{0.0064708,-0.0892368,0.0529307},
{0.0175856,-0.0836504,0.0375139},
{0.0085348,-0.0720366,0.0214263},
{-0.0120812,-0.0511487,0.0210574},
{-0.0043112,-0.0363576,0.0104044},
{-0.0004560,-0.0220668,-0.0048982},
{0.0131378,-0.0172949,-0.0066504},
{0.0177519,-0.0019882,-0.0113575},
{0.0174059,0.0144047,-0.0012364},
{0.0032171,0.0218599,0.0091844},
{-0.0146082,-0.0634936,0.0345529},
{-0.0121368,-0.0138744,0.0059142},
{-0.0124225,0.0188478,0.0016402},
{-0.0189506,0.0026129,0.0032360},
{-0.0069722,-0.0861389,0.0282956},
{-0.0044120,-0.1032818,0.0429048},
{0.0149703,-0.1084703,0.0457333},
{0.0127791,-0.0926769,0.0222168},
{-0.0103734,-0.0687977,0.0218721},
{-0.0148853,-0.0098189,-0.0104333},
{0.0006740,-0.0101404,-0.0190678},
{0.0061228,0.0086238,-0.0175512},
{0.0017228,0.0223074,-0.0082921},
{-0.0107760,0.0093327,-0.0148370},
{0.0004537,-0.1086567,0.0237957},
{-0.0019058,-0.1235320,0.0409115},
{0.0161220,-0.1307936,0.0436324},
{0.0192302,-0.1124406,0.0268799},
{0.0063943,-0.1276641,0.0197378},
{0.0000141,-0.1442436,0.0358246},
{0.0172564,-0.1531552,0.0413788},
{0.0229508,-0.1364777,0.0263885},
{0.0099477,-0.1486033,0.0168631},
{0.0023940,-0.1656010,0.0328967},
{0.0196358,-0.1757625,0.0385987},
{0.0253449,-0.1597074,0.0237217},
{0.0117486,-0.1712313,0.0143889},
{0.0039369,-0.1869341,0.0296028},
{0.0199451,-0.1986224,0.0362333},
{0.0269365,-0.1808293,0.0214930},
{0.0131619,-0.1932618,0.0121758},
{0.0027062,-0.2089582,0.0219076},
{0.0143536,-0.2207535,0.0323300},

Biology Open • Supplementary information

{0.0282637,-0.2184590,0.0246099},
{0.0272932,-0.2012584,0.0172303},
{0.0164464,-0.2182595,0.0090604},
{0.0024096,-0.2313727,0.0187059},
{0.0113691,-0.2476670,0.0282225},
{0.0253803,-0.2393774,0.0291334},
{0.0275532,-0.2370104,0.0133107},
{0.0145250,-0.2444518,0.0070236},
{0.0020346,-0.2578580,0.0158646},
{0.0124507,-0.2726093,0.0270687},
{0.0262004,-0.2607617,0.0260185},
{0.0274174,-0.2619619,0.0101721},
{0.0130697,-0.2705357,0.0045848},
{0.0029578,-0.2828459,0.0151174},
{0.0100639,-0.2977552,0.0247673},
{0.0266299,-0.2854088,0.0229692},
{0.0265537,-0.2844856,0.0062180},
{0.0102661,-0.2970280,0.0024592},
{0.0026367,-0.3133294,0.0108380},
{0.0112276,-0.3213791,0.0244795},
{0.0262020,-0.3097170,0.0211714},
{0.0267662,-0.3046407,0.0040682},
{0.0156449,-0.3187204,-0.0028549},
{0.0039628,-0.3364006,0.0007709},
{0.0025688,-0.3389765,0.0184723},
{0.0223435,-0.3384398,0.0219760},
{0.0292432,-0.3291284,0.0083180},
{0.0231660,-0.3428657,-0.0048947},
{0.0065302,-0.3558244,-0.0067540},
{-0.0001698,-0.3601966,0.0092132},
{0.0119777,-0.3579596,0.0238760},
{0.0293213,-0.3562185,0.0112422},
{0.0229403,-0.3652550,-0.0082923},
{0.0046156,-0.3779211,-0.0121934},
{-0.0022000,-0.3837880,0.0042659},
{0.0016576,-0.3764824,0.0214416},
{0.0225984,-0.3757946,0.0211644},
{0.0287765,-0.3794557,0.0050631},
{0.0218158,-0.3874654,-0.0130281},
{0.0098881,-0.4017368,-0.0240717},
{-0.0052176,-0.3968162,-0.0135541},
{-0.0063419,-0.4099311,0.0028225},
{-0.0058414,-0.4005854,0.0199749},
{0.0103478,-0.3956687,0.0260024},
{0.0278263,-0.3980519,0.0177474},
{0.0266163,-0.4034115,-0.0001464},

Biology Open • Supplementary information

{0.0252982,-0.4122401,-0.0178984},
{0.0176137,-0.4274234,-0.0298080},
{0.0042471,-0.4166083,-0.0375918},
{-0.0093714,-0.4068954,-0.0290396},
{-0.0161941,-0.4164656,-0.0124675},
{-0.0059939,-0.4337725,-0.0032542},
{-0.0173750,-0.4246694,0.0108378},
{-0.0150902,-0.4169414,0.0294926},
{0.0020994,-0.4123661,0.0345252},
{0.0207380,-0.4171395,0.0275438},
{0.0350981,-0.4182602,0.0156852},
{0.0271396,-0.4252295,-0.0018314},
{0.0293813,-0.4398149,-0.0161149},
{0.0172575,-0.4531886,-0.0273154},
{0.0025201,-0.4396468,-0.0390897},
{-0.0134589,-0.4260013,-0.0395794},
{-0.0273285,-0.4193545,-0.0280480},
{-0.0271118,-0.4348897,-0.0118931},
{-0.0173073,-0.4536653,-0.0175126},
{0.0001547,-0.4533735,-0.0116195},
{0.0029786,-0.4507854,0.0074366},
{-0.0141849,-0.4479678,0.0138099},
{-0.0243814,-0.4350788,0.0280654},
{-0.0044100,-0.4322531,0.0398274},
{0.0131090,-0.4363760,0.0328957},
{0.0319767,-0.4376858,0.0241808},
{0.0267186,-0.4426946,0.0071303},
{0.0165345,-0.4493701,-0.0064322},
{-0.0001431,-0.4597152,-0.0298259},
{-0.0142719,-0.4507029,-0.0378101},
{-0.0296586,-0.4409095,-0.0316287},
{0.0193393,-0.4543057,0.0207540},
{0.0015479,-0.4570364,0.0272234},
{-0.0144338,-0.4523126,0.0331619},
{-0.0008327,-0.0739551,0.0191074},
{-0.0099825,-0.0643795,0.0207205},
{0.0095156,-0.0377659,0.0620100},
{-0.0137300,-0.0153456,0.0571572},
{-0.0016831,-0.0629081,0.0574617},
{-0.0060903,-0.0706443,0.0225442},
{-0.0013640,-0.0907720,0.0205866},
{-0.0067629,0.0018754,-0.0170586},
{-0.0120565,-0.0493732,0.0412900},
{0.0031970,-0.0294303,0.0657458},
{-0.0098302,-0.0729773,0.0257857},
{-0.0010324,-0.0599791,0.0184125},

Biology Open • Supplementary information

{-0.0127479,-0.0188359,0.0509469},
{0.0131723,-0.0333095,0.0603258},
{-0.0101257,-0.0330840,0.0398181},
{0.0138266,-0.0380728,0.0586369},
{-0.0008621,-0.0828572,0.0538586},
{-0.0143384,-0.0485069,0.0313499},
{-0.0135098,-0.0615097,0.0220539},
{-0.0157322,-0.0601606,0.0233284},
{-0.0023999,-0.0457195,0.0136129},
{0.0104794,-0.0640907,0.0239572},
{0.0209945,-0.0146591,0.0396741},
{-0.0030234,-0.0487035,0.0621004},
{0.0131262,-0.0031042,0.0172619},
{-0.0085765,-0.0538085,0.0189575},
{-0.0057816,-0.0894513,0.0444631},
{0.0054077,0.0163355,0.0131501},
{-0.0109612,0.0187947,-0.0060283},
{-0.0068459,-0.0743141,0.0475929},
{0.0076047,-0.0934689,0.0194415},
{0.0125414,-0.0819930,0.0243371},
{0.0075142,-0.0520509,0.0588912},
{0.0140076,-0.0139121,0.0076010},
{-0.0056060,-0.0259410,0.0045843},
{-0.0069772,-0.0010700,0.0201061},
{-0.0098024,-0.0619550,0.0489729},
{-0.0060125,-0.0288250,0.0658084},
{0.0081661,-0.0069991,0.0601046},
{0.0132087,-0.0556416,0.0296523},
{-0.0019484,-0.0199631,-0.0120628},
{-0.0133816,-0.0269225,0.0546438},
{0.0155494,0.0146474,-0.0070204},
{-0.0039394,0.0237361,0.0036575},
{0.0006129,-0.0936066,0.0522387},
{-0.0126101,-0.0435184,0.0494718},
{0.0192791,-0.0307732,0.0420082},
{0.0112462,-0.0472377,0.0238814},
{0.0173034,0.0114334,0.0071475},
{0.0199416,0.0022898,0.0066762},
{0.0208747,-0.0021793,-0.0033791},
{0.0133717,-0.0221318,0.0222784},
{0.0110994,-0.0380960,0.0212708},
{0.0157526,-0.0161510,0.0318701},
{-0.0168455,-0.0538837,0.0261983},
{0.0029148,-0.0421221,0.0637955},
{0.0162972,-0.0496130,0.0379656},
{0.0140866,-0.0111267,0.0131121},

Biology Open • Supplementary information

{0.0009948,0.0048252,-0.0191881},
{0.0084071,-0.0008383,-0.0190423},
{0.0100691,-0.0740396,0.0530487},
{0.0143806,-0.0668595,0.0503942},
{0.0168909,-0.0649603,0.0419033},
{0.0170841,-0.0752019,0.0354372},
{-0.0045497,-0.0820504,0.0242521},
{-0.0127820,0.0040967,0.0137890},
{0.0074376,-0.0012309,0.0544513},
{0.0185054,-0.0102559,0.0496338},
{0.0144277,-0.0259215,0.0593901},
{-0.0114741,-0.0391897,0.0249915},
{-0.0176863,-0.0082073,-0.0025197},
{0.0157362,-0.0906067,0.0270126},
{-0.0067172,-0.0062874,-0.0187429},
{0.0039607,0.0154315,-0.0146024},
{-0.0089930,-0.0361694,0.0157670},
{-0.0143142,0.0112861,-0.0102596},
{-0.0194279,0.0054276,-0.0016374},
{-0.0087810,-0.0766660,0.0389695},
{0.0112456,-0.0190515,-0.0009348},
{0.0121031,-0.0496847,0.0565443},
{0.0084019,-0.0201998,-0.0088575},
{0.0097369,-0.0152128,-0.0135755},
{0.0041693,-0.0649308,0.0186724},
{0.0086812,-0.0553895,0.0211561},
{0.0011865,-0.0830371,0.0197797},
{-0.0176614,-0.0612657,0.0297566},
{-0.0058714,-0.0014852,0.0514124},
{-0.0127314,-0.0336196,0.0586432},
{0.0174345,-0.0189441,0.0533461},
{0.0145866,-0.0840189,0.0475746},
{0.0182736,-0.0262059,0.0345788},
{0.0081295,-0.0347689,0.0655871},
{-0.0165573,-0.0035475,0.0097913},
{0.0140406,-0.0176199,0.0593594},
{0.0150797,-0.0668860,0.0321583},
{-0.0148117,-0.0547496,0.0222170},
{-0.0136952,-0.0364334,0.0518969},
{0.0135417,-0.0454633,0.0301530},
{-0.0076209,-0.0423014,0.0635003},
{0.0064715,-0.0084554,-0.0185962},
{0.0040352,-0.0168479,-0.0149546},
{-0.0140452,-0.0117802,0.0507994},
{-0.0101879,-0.0488987,0.0571738},
{0.0170226,-0.0590493,0.0466482},

Biology Open • Supplementary information

{-0.0164832,0.0122000,0.0015480},
{-0.0134840,0.0100198,0.0115011},
{-0.0018596,-0.0743006,0.0541086},
{0.0129708,-0.0101481,0.0336718},
{0.0135600,-0.0038287,0.0544300},
{0.0149126,-0.0062759,0.0426546},
{-0.0124961,-0.0147037,-0.0084975},
{0.0056005,-0.0239991,0.0023250},
{0.0003608,-0.0536028,0.0168432},
{0.0195863,0.0034220,-0.0075073},
{0.0167880,0.0017755,-0.0130173},
{0.0125205,-0.0084333,-0.0156125},
{-0.0128497,0.0015007,-0.0152749},
{-0.0143481,-0.0065353,-0.0130888},
{0.0173196,0.0144673,0.0031705},
{0.0103489,0.0208608,-0.0022694},
{0.0088336,0.0151759,-0.0130813},
{0.0078935,-0.0247060,0.0638313},
{-0.0006960,-0.0094375,0.0629568},
{-0.0001624,-0.0022871,0.0567379},
{0.0052709,-0.0150020,0.0644049},
{0.0082303,0.0080374,0.0163817},
{0.0120798,-0.0190314,0.0155343},
{0.0038611,-0.0276488,0.0654906},
{-0.0089455,-0.0188982,0.0241920},
{-0.0100261,-0.0191001,0.0120765},
{-0.0063547,-0.0109385,0.0270135},
{-0.0147933,-0.0554764,0.0353395},
{-0.0111461,-0.0670649,0.0409048},
{-0.0068033,-0.0318317,0.0093511},
{0.0203350,-0.0202424,0.0461631},
{0.0120161,-0.0092605,0.0276465},
{0.0042462,-0.0010353,0.0242212},
{-0.0035209,-0.0832038,0.0526646},
{-0.0023626,0.0240090,-0.0032637},
{-0.0011229,-0.0144690,0.0390524},
{-0.0107440,-0.0157244,0.0024589},
{0.0127360,-0.0315566,0.0255963},
{0.0101482,-0.0297626,0.0171028},
{-0.0108133,-0.0076163,0.0525692},
{0.0184106,-0.0363147,0.0478843},
{-0.0118653,-0.0586869,0.0414780},
{-0.0073431,-0.0887015,0.0376037},
{0.0042588,-0.0057549,0.0307972},
{-0.0029373,-0.0050512,-0.0199299},
{-0.0123976,-0.0381118,0.0582032},

{-0.0029585,0.0097633,-0.0168482},
{-0.0042481,0.0182025,-0.0124424},
{0.0182947,-0.0941570,0.0362526},
{0.0036301,-0.0811998,0.0189988},
{0.0161828,-0.0971478,0.0445661},
{0.0094255,0.0210627,0.0053711},
{0.0115454,0.0100409,0.0145496},
{0.0010801,0.0165438,-0.0142783},
{0.0158047,-0.0128293,-0.0088314},
{0.0186125,-0.0083915,-0.0075735},
{-0.0085519,-0.0267411,0.0303713},
{-0.0107182,-0.0380266,0.0321037},
{-0.0131742,-0.0679206,0.0256811},
{-0.0013916,0.0029302,0.0200577},
{0.0040826,0.0080626,0.0173042},
{-0.0024725,-0.0031413,0.0256242},
{0.0002523,-0.0339389,0.0649938},
{0.0067805,-0.0478432,0.0598146},
{-0.0114835,-0.0218756,0.0626579},
{-0.0114569,-0.0290102,0.0618896},
{0.0088705,0.0223223,0.0026442},
{0.0167793,0.0155919,0.0014030},
{-0.0069910,-0.0120291,0.0637946},
{0.0149375,-0.0585912,0.0343232},
{-0.0121738,-0.0524266,0.0479818},
{-0.0147240,-0.0555980,0.0219784},
{0.0107484,-0.0929333,0.0505340},
{-0.0096095,-0.0278250,0.0176816},
{-0.0099049,-0.0169442,0.0170009},
{-0.0043400,0.0176315,0.0126169},
{0.0008769,-0.0751272,0.0558668},
{0.0057725,-0.0692637,0.0560385},
{-0.0022138,-0.0689203,0.0555342},
{0.0162451,-0.0812152,0.0310188},
{-0.0122085,-0.0201641,0.0488832},
{0.0104573,-0.0273972,0.0172112},
{0.0050512,-0.0578613,0.0585039},
{0.0081386,-0.0386696,0.0622004},
{0.0093485,-0.0928796,0.0198191},
{-0.0066424,-0.0582799,0.0568033},
{0.0175665,-0.0471545,0.0439029},
{-0.0116790,-0.0471352,0.0211158},
{0.0207610,-0.0227037,0.0373486},
{-0.0039475,-0.0671032,0.0202562},
{0.0163109,-0.0021913,0.0127414},
{0.0060472,0.0059454,0.0174532},

Biology Open • Supplementary information

{0.0008973,-0.0356806,0.0648763},
{-0.0145217,0.0150907,-0.0050617},
{0.0103917,0.0172374,0.0101362},
{-0.0104541,0.0175875,0.0081332},
{-0.0182301,-0.0060358,-0.0051498},
{-0.0183388,0.0005583,-0.0073872},
{-0.0048011,-0.0192032,0.0661919},
{-0.0128817,-0.0376806,0.0433997},
{0.0054732,-0.0514149,0.0597865},
{0.0121818,-0.0456627,0.0575491},
{-0.0085634,-0.0749034,0.0253109},
{0.0123749,0.0077723,0.0149833},
{0.0178621,-0.0117258,-0.0011185},
{-0.0169720,-0.0088101,0.0049857},
{-0.0125156,-0.0616304,0.0384587},
{-0.0071644,-0.0184463,-0.0037014},
{-0.0126731,-0.0301651,0.0465187},
{0.0045585,-0.0819557,0.0543227},
{0.0156500,-0.0546297,0.0512126},
{-0.0017001,-0.0381389,0.0108897},
{0.0061208,-0.0350236,0.0129800},
{0.0183929,-0.0076246,0.0057079},
{0.0177597,-0.0890834,0.0411791},
{-0.0089480,-0.0207062,0.0247918},
{-0.0008610,0.0141710,0.0158665},
{0.0072542,-0.0746436,0.0208976},
{0.0086161,-0.0682106,0.0554026},
{0.0147168,-0.0141766,-0.0093005},
{0.0005447,-0.0099521,0.0634260},
{-0.0070057,-0.0161222,-0.0139446},
{0.0155734,-0.0770614,0.0462586},
{0.0075327,-0.0201295,-0.0101782},
{-0.0103995,-0.0704592,0.0228510},
{0.0130418,-0.0429701,0.0285548},
{0.0152016,-0.0046743,0.0529995},
{0.0046476,-0.0083463,0.0386328},
{-0.0029744,-0.0674764,0.0560826},
{-0.0125429,-0.0650371,0.0374527},
{-0.0141596,-0.0124791,0.0530348},
{-0.0076993,0.0133991,-0.0143782},
{-0.0168189,0.0063183,0.0079978},
{0.0091571,-0.0743503,0.0217864},
{0.0024429,-0.0424451,0.0628999},
{-0.0132271,-0.0236598,0.0539505},
{0.0063080,-0.0066918,-0.0192844},
{0.0150897,-0.0308168,0.0554568},

Biology Open • Supplementary information

{0.0117184,-0.0163287,0.0608534},
{0.0102063,0.0022547,-0.0177624},
{0.0134261,-0.0088256,0.0168683},
{-0.0126745,0.0120351,0.0114725},
{0.0101463,-0.0571006,0.0564887},
{-0.0053535,-0.0414664,0.0643088},
{0.0190430,-0.0313770,0.0491142},
{0.0155211,-0.0142823,-0.0068794},
{-0.0146145,-0.0541797,0.0358313},
{-0.0073961,-0.0795523,0.0444234},
{0.0031336,-0.0567718,0.0592294},
{-0.0043236,-0.0446710,0.0137753},
{0.0109072,0.0115284,-0.0143619},
{-0.0020991,-0.0232980,-0.0008298},
{-0.0089786,-0.0790223,0.0297111},
{-0.0101159,-0.0037747,-0.0172723},
{-0.0028561,0.0233569,-0.0045948},
{-0.0093903,-0.0233996,0.0130429},
{-0.0105203,-0.0694168,0.0226765},
{-0.0058246,-0.0805103,0.0244144},
{0.0009336,0.0127720,-0.0164303},
{0.0194379,-0.0279930,0.0483587},
{-0.0057746,-0.0624724,0.0195910},
{0.0172702,-0.0391030,0.0390821},
{0.0172452,-0.0331466,0.0348200},
{-0.0140669,-0.0128783,-0.0009650},
{0.0130013,0.0150894,0.0099930},
{-0.0131155,-0.0117452,0.0068635},
{-0.0104812,-0.0378828,0.0354295},
{0.0175956,-0.0944224,0.0420564},
{0.0173246,-0.0111182,0.0552071},
{0.0183887,-0.0167406,0.0511452},
{0.0013345,-0.0284830,0.0049784},
{0.0105758,-0.0251170,0.0622850},
{-0.0027341,-0.0102565,0.0311282},
{-0.0188953,-0.0045616,-0.0017235},
{0.0027030,-0.0225830,-0.0037768},
{-0.0094923,-0.0764242,0.0366360},
{-0.0124856,-0.0295204,0.0603940},
{0.0175365,0.0055736,-0.0106691},
{-0.0144774,-0.0668633,0.0298626},
{-0.0149301,-0.0045087,0.0126807},
{0.0117565,-0.0086285,0.0268150},
{-0.0131642,-0.0459946,0.0317615},
{0.0147491,-0.0768134,0.0285339},
{0.0007541,0.0036159,-0.0194386},

{0.0165468,-0.0831559,0.0311338},
{0.0068043,0.0068461,0.0167233},
{-0.0140017,0.0024243,0.0128402},
{0.0057343,-0.0460393,0.0164606},
{-0.0063467,-0.0204065,0.0337362},
{0.0201430,-0.0289105,0.0426142},
{-0.0126502,-0.0671274,0.0228016},
{0.0204423,0.0054838,-0.0030200},
{-0.0136014,-0.0673052,0.0277830},
{0.0110425,-0.0239849,0.0110764},
{0.0193495,-0.0125363,0.0454609},
{0.0084917,0.0020858,-0.0187015},
{0.0144636,-0.0089029,0.0135568},
{-0.0111190,-0.0681267,0.0402595},
{0.0004542,0.0220718,0.0092305},
{0.0027866,0.0035407,-0.0197572},
{0.0370096,-0.4320457,0.0211127},
{0.0050156,0.0218706,-0.0078225},
{-0.0134015,-0.0482237,0.0374950},
{-0.0311445,-0.4276703,-0.0270533},
{0.0022989,0.0247427,-0.0000321},
{0.0142584,-0.0262881,0.0267369},
{-0.0140055,-0.0011787,-0.0146110},
{0.0164237,-0.0331953,0.0532961},
{-0.0145386,0.0144077,0.0047694},
{0.0171224,-0.0429439,0.0402766},
{0.0152811,-0.0479082,0.0345470},
{-0.0131678,-0.0377890,0.0460407},
{0.0171934,-0.0606798,0.0443109},
{-0.0165959,-0.0527300,0.0276525},
{0.0030322,-0.0602426,0.0584754},
{0.0010790,-0.0561982,0.0174635},
{0.0212434,-0.0228988,0.0442968},
{0.0078432,-0.0836783,0.0530060},
{-0.0059010,-0.0908466,0.0280964},
{-0.0188269,-0.0023576,0.0030712},
{0.0142705,-0.0146782,0.0198540},
{0.0137253,-0.0729352,0.0276255},
{-0.0092139,-0.0270389,0.0276679},
{-0.0193299,-0.4384840,0.0358989},
{-0.0085774,-0.0610649,0.0524600},
{-0.0113497,-0.4325559,-0.0103345},
{0.0105780,-0.0473430,0.0225178},
{0.0107507,0.0158903,-0.0112529},
{-0.0241355,-0.4289866,0.0290474},
{0.0154951,-0.0581613,0.0352090},

Biology Open • Supplementary information

{0.0002955,-0.0542342,0.0601727},
{-0.0108769,0.0198015,0.0028592},
{-0.0024434,-0.0182747,0.0660515},
{-0.0072235,-0.0080650,0.0242411},
{0.0191889,-0.0112782,0.0424304},
{0.0146951,-0.0268043,0.0574839},
{-0.0139927,-0.0119368,0.0522991},
{0.0196124,0.0090137,-0.0028209},
{-0.0099202,-0.0373400,0.0180156},
{-0.0003333,-0.0154567,-0.0165191},
{-0.0106627,-0.0659174,0.0443814},
{-0.0089101,0.0187413,0.0080582},
{0.0096588,0.0209497,-0.0030155},
{-0.0132542,-0.0295912,0.0572737},
{-0.0059175,-0.4295211,0.0406915},
{-0.0132885,-0.4353608,-0.0381388},
{-0.0132148,-0.4353024,-0.0373505},
{-0.0131411,-0.4352440,-0.0365622},
{-0.0130674,-0.4351856,-0.0357739},
{-0.0129937,-0.4351272,-0.0349856},
{-0.0129200,-0.4350689,-0.0341973},
{-0.0128463,-0.4350105,-0.0334090},
{-0.0127726,-0.4349521,-0.0326207},
{-0.0126989,-0.4348937,-0.0318324},
{-0.0126251,-0.4348353,-0.0310441},
{-0.0125514,-0.4347769,-0.0302558},
{-0.0124777,-0.4347185,-0.0294675},
{-0.0124040,-0.4346601,-0.0286792},
{-0.0123303,-0.4346017,-0.0278909},
{-0.0122566,-0.4345433,-0.0271026},
{-0.0121829,-0.4344849,-0.0263143},
{-0.0121092,-0.4344265,-0.0255259},
{-0.0120355,-0.4343681,-0.0247376},
{-0.0119618,-0.4343097,-0.0239493},
{-0.0118880,-0.4342513,-0.0231610},
{-0.0118143,-0.4341929,-0.0223727},
{-0.0117406,-0.4341345,-0.0215844},
{-0.0116669,-0.4340761,-0.0207961},
{-0.0115932,-0.4340177,-0.0200078},
{-0.0115195,-0.4339593,-0.0192195},
{-0.0114458,-0.4339009,-0.0184312},
{-0.0113721,-0.4338425,-0.0176429},
{-0.0112984,-0.4337841,-0.0168546},
{-0.0112246,-0.4337257,-0.0160663},
{-0.0111509,-0.4336673,-0.0152780},
{-0.0110772,-0.4336089,-0.0144897},

Biology Open • Supplementary information

{-0.0110035,-0.4335505,-0.0137014},
{-0.0109298,-0.4334921,-0.0129131},
{-0.0108561,-0.4334337,-0.0121248},
{-0.0107824,-0.4333753,-0.0113365},
{-0.0107087,-0.4333169,-0.0105482},
{-0.0106350,-0.4332585,-0.0097599},
{-0.0105613,-0.4332001,-0.0089716},
{-0.0104875,-0.4331417,-0.0081833},
{-0.0104138,-0.4330833,-0.0073950},
{-0.0103401,-0.4330249,-0.0066067},
{-0.0102664,-0.4329665,-0.0058184},
{-0.0101927,-0.4329081,-0.0050301},
{-0.0101190,-0.4328497,-0.0042418},
{-0.0100453,-0.4327914,-0.0034535},
{-0.0099716,-0.4327330,-0.0026652},
{-0.0098979,-0.4326746,-0.0018769},
{-0.0098241,-0.4326162,-0.0010886},
{-0.0097504,-0.4325578,-0.0003003},
{-0.0096767,-0.4324994,0.0004880},
{-0.0096030,-0.4324410,0.0012763},
{-0.0095293,-0.4323826,0.0020646},
{-0.0094556,-0.4323242,0.0028529},
{-0.0093819,-0.4322658,0.0036413},
{-0.0093082,-0.4322074,0.0044296},
{-0.0092345,-0.4321490,0.0052179},
{-0.0091608,-0.4320906,0.0060062},
{-0.0090870,-0.4320322,0.0067945},
{-0.0090133,-0.4319738,0.0075828},
{-0.0089396,-0.4319154,0.0083711},
{-0.0088659,-0.4318570,0.0091594},
{-0.0087922,-0.4317986,0.0099477},
{-0.0087185,-0.4317402,0.0107360},
{-0.0086448,-0.4316818,0.0115243},
{-0.0085711,-0.4316234,0.0123126},
{-0.0084974,-0.4315650,0.0131009},
{-0.0084236,-0.4315066,0.0138892},
{-0.0083499,-0.4314482,0.0146775},
{-0.0082762,-0.4313898,0.0154658},
{-0.0082025,-0.4313314,0.0162541},
{-0.0081288,-0.4312730,0.0170424},
{-0.0080551,-0.4312146,0.0178307},
{-0.0079814,-0.4311562,0.0186190},
{-0.0079077,-0.4310978,0.0194073},
{-0.0078340,-0.4310394,0.0201956},
{-0.0077603,-0.4309810,0.0209839},
{-0.0076865,-0.4309226,0.0217722},

Biology Open • Supplementary information

```
                {-0.0076128,-0.4308642,0.0225605},
                {-0.0075391,-0.4308058,0.0233488},
                {-0.0074654,-0.4307474,0.0241371},
                {-0.0073917,-0.4306890,0.0249254},
                {-0.0073180,-0.4306306,0.0257137},
                {-0.0072443,-0.4305722,0.0265020},
                {-0.0071706,-0.4305139,0.0272903},
                {-0.0070969,-0.4304555,0.0280786},
                {-0.0070231,-0.4303971,0.0288669},
                {-0.0069494,-0.4303387,0.0296552},
                {-0.0068757,-0.4302803,0.0304435},
                {-0.0068020,-0.4302219,0.0312318},
                {-0.0067283,-0.4301635,0.0320201},
                {-0.0066546,-0.4301051,0.0328084},
                {-0.0065809,-0.4300467,0.0335968},
                {-0.0065072,-0.4299883,0.0343851},
                {-0.0064335,-0.4299299,0.0351734},
                {-0.0063598,-0.4298715,0.0359617},
                {-0.0062860,-0.4298131,0.0367500},
                {-0.0062123,-0.4297547,0.0375383},
                {-0.0061386,-0.4296963,0.0383266},
                {-0.0060649,-0.4296379,0.0391149},
                {-0.0059912,-0.4295795,0.0399032}
                };
            BoundingBoxOnOff = Off;
        };
        AnyFunTransform3DIdentity ScaleFunction = {
            PreTransforms = {&.RBFTransform};
    };
};
};
};
```

Biology Open • Supplementary information

ScalingFunctionTLEMLucyFemur_Mirrored_2014030

```
AnyFolder ScalingFunctionTLEMLucyFemur_Mirrored = {
 AnyFolder Left = {
        AnyFolder Thigh = {
     AnyMatrix AMirroring = {
      {1,0,0},
      {0,1,0},
      {0,0,-1}
     };
        AnyFunTransform3DRBF RBFTransform = {
                 RBFDef.Type = RBF_ThinPlate;
                 PolynomDegree = 1;
                 Points0 = ({
                          {0.0000000,0.0000000,0.0000000},
                          {-0.0000000,-0.3616821,0.0000000},
                          {-0.0097563,-0.3678799,0.0012967},
                          {-0.0000000,-0.3660632,0.0408203},
                          {-0.0000000,-0.3573010,-0.0408203},
                          {0.0161460,-0.0072838,0.0601290},
                          {0.0220217,-0.0203698,0.0463848},
                          {0.0123977,-0.0241932,0.0668573},
                          {0.0006898,0.0018121,0.0538181},
                          {0.0122809,-0.0068668,0.0414535},
                          {-0.0058991,-0.0138188,0.0648412},
                          {0.0211469,-0.0380855,0.0538111},
                          {0.0172133,-0.0317342,0.0328381},
                          {0.0177311,-0.0131946,0.0256176},
                          {-0.0110079,-0.0308867,0.0632370},
                          {0.0039435,-0.0395977,0.0638271},
                          {-0.0041683,-0.0187472,0.0413214},
                          {-0.0177999,-0.0021535,0.0496084},
                          {0.0001603,-0.0053126,0.0279593},
                          {-0.0211600,-0.0216243,0.0497147},
                          {0.0096163,-0.0568663,0.0545719},
                          {0.0162112,-0.0515013,0.0395230},
                          {0.0093533,-0.0433265,0.0187387},
                          {0.0119398,-0.0238469,0.0118403},
                          {0.0210707,-0.0048848,0.0065878},
                          {0.0125013,0.0064094,0.0177702},
                          {-0.0191523,-0.0356053,0.0382429},
                          {-0.0115821,-0.0474937,0.0537648},
                          {-0.0100073,-0.0236371,0.0267322},
                          {-0.0078925,0.0107098,0.0182833},
                          {-0.0134217,-0.0055356,0.0182000},
                          {-0.0090061,-0.0665700,0.0475484},
```

{0.0071095,-0.0761976,0.0520035},
{0.0182119,-0.0704149,0.0386546},
{0.0073764,-0.0609481,0.0221006},
{-0.0122984,-0.0439585,0.0198428},
{-0.0032253,-0.0306792,0.0110995},
{-0.0012975,-0.0198451,-0.0052592},
{0.0140405,-0.0161125,-0.0071649},
{0.0189222,-0.0014898,-0.0127226},
{0.0181642,0.0132812,-0.0011050},
{0.0045119,0.0200001,0.0091189},
{-0.0176761,-0.0541983,0.0348587},
{-0.0136548,-0.0142015,0.0054311},
{-0.0135510,0.0182940,0.0017175},
{-0.0223865,0.0023399,0.0028018},
{-0.0086455,-0.0738247,0.0283843},
{-0.0050356,-0.0877749,0.0442195},
{0.0160135,-0.0919319,0.0465120},
{0.0128290,-0.0785810,0.0232431},
{-0.0168328,-0.0616327,0.0155917},
{-0.0172369,-0.0094567,-0.0119502},
{0.0002090,-0.0093641,-0.0209779},
{0.0064827,0.0081538,-0.0198453},
{0.0010777,0.0210422,-0.0084721},
{-0.0129485,0.0085451,-0.0162212},
{-0.0002322,-0.0924033,0.0245644},
{-0.0028175,-0.1052263,0.0418294},
{0.0172322,-0.1111444,0.0445760},
{0.0203259,-0.0957409,0.0271449},
{0.0057102,-0.1088933,0.0195113},
{-0.0010852,-0.1230776,0.0363132},
{0.0182727,-0.1303312,0.0422884},
{0.0242891,-0.1161703,0.0265054},
{0.0094531,-0.1268584,0.0162294},
{0.0014244,-0.1413293,0.0331653},
{0.0207271,-0.1496427,0.0394157},
{0.0267562,-0.1360142,0.0236798},
{0.0113839,-0.1461651,0.0136024},
{0.0030845,-0.1595094,0.0297156},
{0.0209528,-0.1691386,0.0369775},
{0.0283958,-0.1540209,0.0213795},
{0.0129499,-0.1649012,0.0113589},
{0.0016490,-0.1783108,0.0216287},
{0.0147105,-0.1880678,0.0328614},
{0.0298491,-0.1859865,0.0248551},
{0.0286645,-0.1714377,0.0169488},
{0.0165897,-0.1861006,0.0082595},

Biology Open • Supplementary information

{0.0014000,-0.1973476,0.0183740},
{0.0114301,-0.2109792,0.0286157},
{0.0267422,-0.2037622,0.0296951},
{0.0288630,-0.2018335,0.0130127},
{0.0145471,-0.2083505,0.0062833},
{0.0011300,-0.2198071,0.0155602},
{0.0126610,-0.2321235,0.0275354},
{0.0275647,-0.2219268,0.0265185},
{0.0286645,-0.2230253,0.0098701},
{0.0130434,-0.2304822,0.0039177},
{0.0022984,-0.2409585,0.0149650},
{0.0101315,-0.2534740,0.0252125},
{0.0279485,-0.2428555,0.0234276},
{0.0276684,-0.2421559,0.0058691},
{0.0101250,-0.2529527,0.0018983},
{0.0021376,-0.2667831,0.0107073},
{0.0114309,-0.2734905,0.0249960},
{0.0273923,-0.2634953,0.0216324},
{0.0278429,-0.2592546,0.0037692},
{0.0159101,-0.2712985,-0.0033988},
{0.0036708,-0.2863280,0.0004644},
{0.0023115,-0.2884756,0.0187554},
{0.0231385,-0.2879051,0.0224853},
{0.0304009,-0.2799973,0.0083549},
{0.0238189,-0.2917246,-0.0052462},
{0.0064904,-0.3027687,-0.0070587},
{-0.0003492,-0.3064690,0.0093014},
{0.0122179,-0.3045269,0.0243511},
{0.0301837,-0.3030127,0.0114462},
{0.0234182,-0.3107336,-0.0085198},
{0.0046893,-0.3214995,-0.0123881},
{-0.0022352,-0.3264905,0.0043340},
{0.0016841,-0.3202756,0.0217839},
{0.0229722,-0.3196887,0.0215083},
{0.0292438,-0.3228042,0.0051430},
{0.0221641,-0.3296190,-0.0132361},
{0.0100459,-0.3417597,-0.0244560},
{-0.0053009,-0.3375737,-0.0137705},
{-0.0064432,-0.3487307,0.0028676},
{-0.0059346,-0.3407801,0.0202938},
{0.0105130,-0.3365976,0.0264175},
{0.0282695,-0.3386249,0.0180307},
{0.0270402,-0.3431844,-0.0001487},
{0.0257040,-0.3506950,-0.0181851},
{0.0178948,-0.3636114,-0.0302838},
{0.0043149,-0.3544109,-0.0381949},

{-0.0095210,-0.3461482,-0.0295031},
{-0.0164526,-0.3542895,-0.0126666},
{-0.0060896,-0.3690127,-0.0033061},
{-0.0176523,-0.3612686,0.0110109},
{-0.0153310,-0.3546943,0.0299634},
{0.0021329,-0.3508021,0.0350763},
{0.0210690,-0.3548628,0.0279835},
{0.0356594,-0.3558162,0.0159356},
{0.0275718,-0.3617450,-0.0018607},
{0.0298503,-0.3741530,-0.0163721},
{0.0175320,-0.3855300,-0.0277515},
{0.0025604,-0.3740099,-0.0397137},
{-0.0136727,-0.3624016,-0.0402102},
{-0.0277647,-0.3567472,-0.0284947},
{-0.0275446,-0.3699630,-0.0120829},
{-0.0175846,-0.3859355,-0.0177922},
{0.0001572,-0.3856873,-0.0118050},
{0.0030262,-0.3834856,0.0075553},
{-0.0144114,-0.3810887,0.0140304},
{-0.0247706,-0.3701239,0.0285134},
{-0.0044804,-0.3677201,0.0404632},
{0.0133183,-0.3712274,0.0334208},
{0.0324861,-0.3723417,0.0245668},
{0.0271451,-0.3766027,0.0072441},
{0.0167985,-0.3822816,-0.0065349},
{-0.0001454,-0.3910823,-0.0303010},
{-0.0144998,-0.3834154,-0.0384137},
{-0.0301321,-0.3750841,-0.0321336},
{0.0196491,-0.3864804,0.0210853},
{0.0015726,-0.3888034,0.0276569},
{-0.0146652,-0.3847848,0.0336913},
{-0.0014588,-0.0619951,0.0219778},
{-0.0118435,-0.0550626,0.0139639},
{0.0129812,-0.0349966,0.0673128},
{-0.0162907,-0.0105666,0.0597739},
{-0.0012885,-0.0520816,0.0562989},
{-0.0090599,-0.0631298,0.0172224},
{-0.0000222,-0.0771663,0.0231897},
{-0.0068148,0.0023885,-0.0160086},
{-0.0180544,-0.0425409,0.0446585},
{0.0020525,-0.0249904,0.0691549},
{-0.0157097,-0.0662315,0.0238393},
{-0.0010518,-0.0510686,0.0199353},
{-0.0208874,-0.0152865,0.0500034},
{0.0168316,-0.0280857,0.0666680},
{-0.0187392,-0.0282333,0.0403282},

Biology Open • Supplementary information

{0.0191622,-0.0351633,0.0622707},
{0.0001216,-0.0706289,0.0524049},
{-0.0169862,-0.0399062,0.0311827},
{-0.0185737,-0.0533066,0.0151224},
{-0.0219058,-0.0528723,0.0222263},
{-0.0028271,-0.0391620,0.0148452},
{0.0117900,-0.0553425,0.0235348},
{0.0219835,-0.0106400,0.0396338},
{-0.0025366,-0.0401046,0.0625273},
{0.0141017,-0.0018767,0.0198108},
{-0.0078770,-0.0473929,0.0173542},
{-0.0065351,-0.0766431,0.0470258},
{0.0065585,0.0145593,0.0161638},
{-0.0118588,0.0172875,-0.0085064},
{-0.0074087,-0.0627500,0.0506700},
{0.0071940,-0.0780068,0.0213362},
{0.0126736,-0.0708176,0.0244108},
{0.0074694,-0.0436450,0.0604196},
{0.0154186,-0.0139471,0.0064312},
{-0.0053535,-0.0213210,0.0040094},
{-0.0088733,0.0002018,0.0205402},
{-0.0112471,-0.0542608,0.0500266},
{-0.0061625,-0.0248673,0.0653244},
{0.0093695,-0.0065826,0.0614157},
{0.0138015,-0.0467322,0.0284333},
{-0.0020999,-0.0186885,-0.0116392},
{-0.0185640,-0.0225591,0.0566779},
{0.0175798,0.0124360,-0.0068467},
{-0.0039962,0.0217127,0.0053055},
{0.0014975,-0.0784591,0.0514056},
{-0.0172997,-0.0386971,0.0525252},
{0.0196540,-0.0268271,0.0416806},
{0.0135296,-0.0406504,0.0236882},
{0.0182471,0.0095959,0.0087906},
{0.0218667,0.0016804,0.0058793},
{0.0224975,-0.0022652,-0.0022994},
{0.0166750,-0.0191391,0.0236436},
{0.0119785,-0.0328723,0.0195376},
{0.0187631,-0.0133469,0.0308741},
{-0.0187543,-0.0461463,0.0257491},
{0.0039834,-0.0373928,0.0683089},
{0.0157029,-0.0423169,0.0385226},
{0.0164003,-0.0087069,0.0131181},
{0.0008253,0.0033370,-0.0212576},
{0.0101707,-0.0009724,-0.0205688},
{0.0102948,-0.0642683,0.0524532},

Biology Open • Supplementary information

{0.0152217,-0.0557944,0.0508978},
{0.0172973,-0.0565707,0.0419468},
{0.0167723,-0.0635334,0.0369834},
{-0.0047397,-0.0695325,0.0241398},
{-0.0152913,0.0035778,0.0161461},
{0.0091697,0.0009340,0.0575160},
{0.0196686,-0.0089533,0.0500006},
{0.0183998,-0.0212943,0.0622968},
{-0.0114340,-0.0316810,0.0250940},
{-0.0206442,-0.0092599,-0.0028425},
{0.0166868,-0.0784295,0.0276925},
{-0.0069678,-0.0054521,-0.0207011},
{0.0049490,0.0151109,-0.0160093},
{-0.0084025,-0.0318937,0.0162788},
{-0.0173679,0.0095594,-0.0108509},
{-0.0218912,0.0063457,-0.0020698},
{-0.0106547,-0.0668750,0.0397749},
{0.0136098,-0.0170666,-0.0018890},
{0.0141599,-0.0441405,0.0587594},
{0.0093255,-0.0183218,-0.0083008},
{0.0106367,-0.0128276,-0.0154269},
{0.0037350,-0.0549365,0.0209786},
{0.0087662,-0.0472902,0.0195496},
{0.0016839,-0.0708213,0.0221325},
{-0.0202977,-0.0530540,0.0301270},
{-0.0068584,0.0021781,0.0530002},
{-0.0144817,-0.0297218,0.0605833},
{0.0197015,-0.0165217,0.0560654},
{0.0156609,-0.0705363,0.0477736},
{0.0207876,-0.0221433,0.0330733},
{0.0092324,-0.0300767,0.0713160},
{-0.0203392,-0.0034488,0.0097332},
{0.0156978,-0.0143438,0.0629053},
{0.0143267,-0.0563519,0.0317489},
{-0.0161685,-0.0465391,0.0188591},
{-0.0196397,-0.0309875,0.0546496},
{0.0154804,-0.0380422,0.0305433},
{-0.0077774,-0.0351303,0.0637765},
{0.0063980,-0.0086419,-0.0201654},
{0.0030146,-0.0151094,-0.0165974},
{-0.0210080,-0.0096030,0.0507855},
{-0.0105684,-0.0411377,0.0586146},
{0.0180619,-0.0498960,0.0469835},
{-0.0192318,0.0116108,0.0024070},
{-0.0165651,0.0083622,0.0126870},
{-0.0014333,-0.0627138,0.0534746},

Biology Open • Supplementary information

{0.0145127,-0.0080178,0.0353667},
{0.0148374,-0.0012228,0.0566181},
{0.0153834,-0.0062427,0.0444727},
{-0.0155164,-0.0132103,-0.0096398},
{0.0068685,-0.0221022,0.0011547},
{0.0003332,-0.0458500,0.0183716},
{0.0205582,0.0040956,-0.0082969},
{0.0175752,0.0009340,-0.0148407},
{0.0142357,-0.0063623,-0.0169332},
{-0.0147285,0.0023412,-0.0164678},
{-0.0158781,-0.0064451,-0.0151346},
{0.0175951,0.0133456,0.0037559},
{0.0117660,0.0189899,-0.0030690},
{0.0096040,0.0151827,-0.0137450},
{0.0096565,-0.0215194,0.0662734},
{-0.0006793,-0.0074494,0.0631218},
{0.0007819,-0.0004519,0.0600781},
{0.0065377,-0.0125433,0.0654199},
{0.0090461,0.0073096,0.0188711},
{0.0151418,-0.0168302,0.0146465},
{0.0025166,-0.0231863,0.0688894},
{-0.0097891,-0.0152077,0.0253086},
{-0.0109149,-0.0177935,0.0119825},
{-0.0072079,-0.0099373,0.0272725},
{-0.0183727,-0.0467429,0.0356276},
{-0.0126871,-0.0584555,0.0428141},
{-0.0057054,-0.0273042,0.0103554},
{0.0215142,-0.0159693,0.0474531},
{0.0128492,-0.0070016,0.0287645},
{0.0052134,-0.0017047,0.0247463},
{-0.0024284,-0.0707498,0.0513049},
{-0.0036140,0.0220593,-0.0040484},
{-0.0002405,-0.0121253,0.0390050},
{-0.0119811,-0.0156737,0.0015445},
{0.0142082,-0.0279792,0.0246553},
{0.0122244,-0.0262013,0.0169829},
{-0.0146264,-0.0038855,0.0549384},
{0.0207450,-0.0325771,0.0496412},
{-0.0155040,-0.0497560,0.0437087},
{-0.0081119,-0.0756450,0.0387365},
{0.0039684,-0.0062047,0.0313900},
{-0.0023726,-0.0038892,-0.0220521},
{-0.0136977,-0.0339839,0.0602619},
{-0.0035399,0.0089081,-0.0188894},
{-0.0049820,0.0176571,-0.0132254},
{0.0193902,-0.0803191,0.0360429},

Biology Open • Supplementary information

{0.0040648,-0.0697109,0.0218205},
{0.0170470,-0.0818610,0.0456892},
{0.0104175,0.0188635,0.0064965},
{0.0135206,0.0092549,0.0158079},
{0.0023933,0.0160498,-0.0157404},
{0.0164753,-0.0118197,-0.0096995},
{0.0202584,-0.0066858,-0.0082383},
{-0.0098401,-0.0226001,0.0314028},
{-0.0130937,-0.0309622,0.0320764},
{-0.0201510,-0.0613297,0.0222264},
{-0.0010389,0.0036055,0.0209003},
{0.0042025,0.0073392,0.0199393},
{-0.0021630,-0.0038531,0.0251898},
{-0.0007845,-0.0293574,0.0675788},
{0.0070027,-0.0406327,0.0624565},
{-0.0124363,-0.0183001,0.0627966},
{-0.0120485,-0.0256613,0.0628493},
{0.0101200,0.0197695,0.0039334},
{0.0167636,0.0148266,0.0014155},
{-0.0074981,-0.0106930,0.0632797},
{0.0142533,-0.0503445,0.0351954},
{-0.0146107,-0.0460953,0.0506120},
{-0.0161117,-0.0471301,0.0178900},
{0.0112149,-0.0792793,0.0501460},
{-0.0099060,-0.0230794,0.0186951},
{-0.0114543,-0.0153049,0.0183675},
{-0.0042882,0.0161879,0.0151423},
{0.0014582,-0.0638572,0.0541080},
{0.0048554,-0.0582603,0.0553815},
{-0.0017077,-0.0572302,0.0546635},
{0.0166090,-0.0697169,0.0323038},
{-0.0208812,-0.0164273,0.0474354},
{0.0130454,-0.0241864,0.0178294},
{0.0053123,-0.0483770,0.0582251},
{0.0112225,-0.0361429,0.0673300},
{0.0085729,-0.0771256,0.0216100},
{-0.0070783,-0.0488033,0.0560869},
{0.0182083,-0.0411024,0.0454236},
{-0.0119590,-0.0403809,0.0213546},
{0.0233409,-0.0185535,0.0366343},
{-0.0046347,-0.0571211,0.0208243},
{0.0183166,-0.0006966,0.0140116},
{0.0061913,0.0053512,0.0201003},
{-0.0002364,-0.0310828,0.0680351},
{-0.0170773,0.0139270,-0.0054987},
{0.0110805,0.0156034,0.0117010},

{-0.0124628,0.0164667,0.0094550},
{-0.0209014,-0.0073407,-0.0051969},
{-0.0210131,0.0010693,-0.0086891},
{-0.0041596,-0.0172332,0.0663041},
{-0.0216595,-0.0322172,0.0451540},
{0.0051908,-0.0426014,0.0613013},
{0.0150003,-0.0416136,0.0606257},
{-0.0135090,-0.0674234,0.0236425},
{0.0146416,0.0073691,0.0160052},
{0.0195615,-0.0112412,-0.0010333},
{-0.0198115,-0.0098244,0.0044735},
{-0.0157054,-0.0521236,0.0395039},
{-0.0081381,-0.0173108,-0.0046152},
{-0.0218324,-0.0262661,0.0471115},
{0.0051446,-0.0707257,0.0530555},
{0.0179359,-0.0471493,0.0528134},
{-0.0005591,-0.0317983,0.0113632},
{0.0071707,-0.0305943,0.0121186},
{0.0206239,-0.0061367,0.0066642},
{0.0187057,-0.0745868,0.0420207},
{-0.0095784,-0.0163113,0.0259524},
{-0.0012181,0.0127661,0.0186855},
{0.0059420,-0.0630691,0.0220087},
{0.0069408,-0.0573878,0.0552614},
{0.0150782,-0.0132884,-0.0101793},
{0.0005533,-0.0078574,0.0633775},
{-0.0088284,-0.0146399,-0.0147837},
{0.0167444,-0.0651105,0.0470347},
{0.0082666,-0.0181741,-0.0095133},
{-0.0175162,-0.0637444,0.0177069},
{0.0153795,-0.0360767,0.0287608},
{0.0162363,-0.0018864,0.0548412},
{0.0052731,-0.0075919,0.0392990},
{-0.0022288,-0.0556644,0.0549909},
{-0.0156238,-0.0550038,0.0377688},
{-0.0204137,-0.0100550,0.0536429},
{-0.0093683,0.0122429,-0.0162013},
{-0.0202927,0.0049166,0.0083887},
{0.0074706,-0.0627945,0.0222133},
{0.0035034,-0.0376103,0.0672421},
{-0.0190752,-0.0194129,0.0555530},
{0.0061758,-0.0074961,-0.0207741},
{0.0192011,-0.0273063,0.0588233},
{0.0130796,-0.0134383,0.0638611},
{0.0120421,0.0019566,-0.0194140},
{0.0154152,-0.0065294,0.0179581},

Biology Open • Supplementary information

{-0.0157335,0.0101219,0.0123981},
{0.0110376,-0.0488274,0.0567911},
{-0.0055170,-0.0343676,0.0647140},
{0.0216204,-0.0273988,0.0512041},
{0.0161331,-0.0135875,-0.0075501},
{-0.0184643,-0.0455633,0.0360087},
{-0.0091978,-0.0682413,0.0460847},
{0.0034304,-0.0471677,0.0588866},
{-0.0048025,-0.0384316,0.0149400},
{0.0118505,0.0109735,-0.0159161},
{-0.0027036,-0.0204189,-0.0015094},
{-0.0117607,-0.0692759,0.0292171},
{-0.0107080,-0.0033455,-0.0190487},
{-0.0042548,0.0214947,-0.0056846},
{-0.0097315,-0.0208529,0.0131997},
{-0.0178958,-0.0627218,0.0171551},
{-0.0068692,-0.0688818,0.0233316},
{0.0012083,0.0120695,-0.0186179},
{0.0215073,-0.0236027,0.0503069},
{-0.0060262,-0.0534931,0.0189523},
{0.0179951,-0.0336785,0.0397267},
{0.0190509,-0.0284972,0.0336394},
{-0.0169188,-0.0133609,-0.0014203},
{0.0135722,0.0136491,0.0114695},
{-0.0150549,-0.0123230,0.0067722},
{-0.0148411,-0.0311945,0.0355412},
{0.0185490,-0.0794042,0.0431266},
{0.0193568,-0.0087326,0.0575704},
{0.0201026,-0.0144353,0.0531586},
{0.0025583,-0.0248211,0.0045714},
{0.0132689,-0.0214866,0.0652899},
{-0.0028669,-0.0099311,0.0309271},
{-0.0219487,-0.0054893,-0.0021853},
{0.0026180,-0.0203806,-0.0040620},
{-0.0116230,-0.0668518,0.0370152},
{-0.0133007,-0.0264159,0.0619176},
{0.0186167,0.0050491,-0.0120059},
{-0.0191359,-0.0591690,0.0288149},
{-0.0178121,-0.0041834,0.0141448},
{0.0123077,-0.0063648,0.0279743},
{-0.0157729,-0.0374010,0.0315335},
{0.0149328,-0.0659100,0.0290095},
{0.0006205,0.0021977,-0.0214613},
{0.0170484,-0.0715687,0.0325980},
{0.0068499,0.0061192,0.0196608},
{-0.0167075,0.0020024,0.0150382},

Biology Open • Supplementary information

{0.0059801,-0.0393117,0.0167489},
{-0.0072506,-0.0171067,0.0338751},
{0.0202363,-0.0251596,0.0420997},
{-0.0199311,-0.0601255,0.0171522},
{0.0216641,0.0053410,-0.0027841},
{-0.0197665,-0.0606125,0.0252972},
{0.0129986,-0.0214528,0.0098002},
{0.0201151,-0.0100929,0.0460501},
{0.0104137,0.0017765,-0.0203073},
{0.0166557,-0.0064123,0.0140197},
{-0.0124120,-0.0596549,0.0422637},
{0.0014885,0.0202590,0.0096781},
{0.0028720,0.0020207,-0.0219044},
{0.0376095,-0.3675427,0.0214537},
{0.0049949,0.0206417,-0.0079017},
{-0.0191723,-0.0407590,0.0391310},
{-0.0316406,-0.3638215,-0.0274841},
{0.0021392,0.0226920,0.0000985},
{0.0168435,-0.0229382,0.0264788},
{-0.0157304,-0.0005437,-0.0161456},
{0.0203722,-0.0299750,0.0562762},
{-0.0171077,0.0134146,0.0059615},
{0.0175519,-0.0370653,0.0413374},
{0.0154657,-0.0405127,0.0348980},
{-0.0215172,-0.0323502,0.0481925},
{0.0177998,-0.0515776,0.0444709},
{-0.0183804,-0.0448316,0.0275139},
{0.0033607,-0.0502028,0.0575894},
{0.0010325,-0.0479377,0.0191141},
{0.0224852,-0.0181821,0.0451780},
{0.0084089,-0.0720538,0.0521031},
{-0.0070880,-0.0772912,0.0288023},
{-0.0223712,-0.0028990,0.0024047},
{0.0173136,-0.0120017,0.0204612},
{0.0137772,-0.0623537,0.0276621},
{-0.0100390,-0.0231180,0.0292222},
{-0.0196395,-0.3730207,0.0364730},
{-0.0094445,-0.0526688,0.0528111},
{-0.0115309,-0.3679776,-0.0104995},
{0.0129812,-0.0410477,0.0224072},
{0.0117394,0.0154849,-0.0117191},
{-0.0245208,-0.3649412,0.0295111},
{0.0147476,-0.0502966,0.0364899},
{0.0006132,-0.0446771,0.0600524},
{-0.0118123,0.0191139,0.0031977},
{-0.0017185,-0.0163303,0.0664216},

Biology Open • Supplementary information

{-0.0085098,-0.0074564,0.0247745},
{0.0197459,-0.0086852,0.0430460},
{0.0187064,-0.0223532,0.0600966},
{-0.0206552,-0.0099712,0.0525024},
{0.0207525,0.0084646,-0.0024846},
{-0.0094915,-0.0325446,0.0184441},
{-0.0014464,-0.0140050,-0.0179819},
{-0.0122725,-0.0573444,0.0463125},
{-0.0106397,0.0177211,0.0094669},
{0.0111981,0.0191236,-0.0041491},
{-0.0162790,-0.0256279,0.0592687},
{-0.0060120,-0.3653959,0.0413411},
{-0.0135007,-0.3703638,-0.0387476},
{-0.0134258,-0.3703142,-0.0379467},
{-0.0133509,-0.3702645,-0.0371458},
{-0.0132760,-0.3702148,-0.0363450},
{-0.0132011,-0.3701651,-0.0355441},
{-0.0131262,-0.3701154,-0.0347432},
{-0.0130513,-0.3700658,-0.0339423},
{-0.0129765,-0.3700161,-0.0331414},
{-0.0129016,-0.3699664,-0.0323405},
{-0.0128267,-0.3699167,-0.0315396},
{-0.0127518,-0.3698670,-0.0307388},
{-0.0126769,-0.3698174,-0.0299379},
{-0.0126020,-0.3697677,-0.0291370},
{-0.0125271,-0.3697180,-0.0283361},
{-0.0124523,-0.3696683,-0.0275352},
{-0.0123774,-0.3696187,-0.0267343},
{-0.0123025,-0.3695690,-0.0259334},
{-0.0122276,-0.3695193,-0.0251325},
{-0.0121527,-0.3694696,-0.0243317},
{-0.0120778,-0.3694199,-0.0235308},
{-0.0120029,-0.3693703,-0.0227299},
{-0.0119280,-0.3693206,-0.0219290},
{-0.0118532,-0.3692709,-0.0211281},
{-0.0117783,-0.3692212,-0.0203272},
{-0.0117034,-0.3691715,-0.0195263},
{-0.0116285,-0.3691219,-0.0187255},
{-0.0115536,-0.3690722,-0.0179246},
{-0.0114787,-0.3690225,-0.0171237},
{-0.0114038,-0.3689728,-0.0163228},
{-0.0113289,-0.3689231,-0.0155219},
{-0.0112541,-0.3688735,-0.0147210},
{-0.0111792,-0.3688238,-0.0139201},
{-0.0111043,-0.3687741,-0.0131192},
{-0.0110294,-0.3687244,-0.0123184},

Biology Open • Supplementary information

{-0.0109545,-0.3686748,-0.0115175},
{-0.0108796,-0.3686251,-0.0107166},
{-0.0108047,-0.3685754,-0.0099157},
{-0.0107298,-0.3685257,-0.0091148},
{-0.0106550,-0.3684760,-0.0083139},
{-0.0105801,-0.3684264,-0.0075130},
{-0.0105052,-0.3683767,-0.0067121},
{-0.0104303,-0.3683270,-0.0059113},
{-0.0103554,-0.3682773,-0.0051104},
{-0.0102805,-0.3682276,-0.0043095},
{-0.0102056,-0.3681780,-0.0035086},
{-0.0101307,-0.3681283,-0.0027077},
{-0.0100559,-0.3680786,-0.0019068},
{-0.0099810,-0.3680289,-0.0011059},
{-0.0099061,-0.3679792,-0.0003051},
{-0.0098312,-0.3679296,0.0004958},
{-0.0097563,-0.3678799,0.0012967},
{-0.0096814,-0.3678302,0.0020976},
{-0.0096065,-0.3677805,0.0028985},
{-0.0095317,-0.3677308,0.0036994},
{-0.0094568,-0.3676812,0.0045003},
{-0.0093819,-0.3676315,0.0053012},
{-0.0093070,-0.3675818,0.0061020},
{-0.0092321,-0.3675321,0.0069029},
{-0.0091572,-0.3674825,0.0077038},
{-0.0090823,-0.3674328,0.0085047},
{-0.0090074,-0.3673831,0.0093056},
{-0.0089326,-0.3673334,0.0101065},
{-0.0088577,-0.3672837,0.0109074},
{-0.0087828,-0.3672341,0.0117082},
{-0.0087079,-0.3671844,0.0125091},
{-0.0086330,-0.3671347,0.0133100},
{-0.0085581,-0.3670850,0.0141109},
{-0.0084832,-0.3670353,0.0149118},
{-0.0084083,-0.3669857,0.0157127},
{-0.0083335,-0.3669360,0.0165136},
{-0.0082586,-0.3668863,0.0173145},
{-0.0081837,-0.3668366,0.0181153},
{-0.0081088,-0.3667869,0.0189162},
{-0.0080339,-0.3667373,0.0197171},
{-0.0079590,-0.3666876,0.0205180},
{-0.0078841,-0.3666379,0.0213189},
{-0.0078092,-0.3665882,0.0221198},
{-0.0077344,-0.3665386,0.0229207},
{-0.0076595,-0.3664889,0.0237215},
{-0.0075846,-0.3664392,0.0245224},

Biology Open • Supplementary information

```
{-0.0075097,-0.3663895,0.0253233},
{-0.0074348,-0.3663398,0.0261242},
{-0.0073599,-0.3662902,0.0269251},
{-0.0072850,-0.3662405,0.0277260},
{-0.0072101,-0.3661908,0.0285269},
{-0.0071353,-0.3661411,0.0293278},
{-0.0070604,-0.3660914,0.0301286},
{-0.0069855,-0.3660418,0.0309295},
{-0.0069106,-0.3659921,0.0317304},
{-0.0068357,-0.3659424,0.0325313},
{-0.0067608,-0.3658927,0.0333322},
{-0.0066859,-0.3658430,0.0341331},
{-0.0066111,-0.3657934,0.0349340},
{-0.0065362,-0.3657437,0.0357348},
{-0.0064613,-0.3656940,0.0365357},
{-0.0063864,-0.3656443,0.0373366},
{-0.0063115,-0.3655947,0.0381375},
{-0.0062366,-0.3655450,0.0389384},
{-0.0061617,-0.3654953,0.0397393},
{-0.0060868,-0.3654456,0.0405402}
}* .AMirroring);
Points1 = ({
{0.0000000,0.0000000,0.0000000},
{-0.0000000,-0.4659265,0.0000000},
{-0.0104358,-0.4739106,0.0013870},
{-0.0000000,-0.4715703,0.0436633},
{-0.0000000,-0.4602826,-0.0436633},
{0.0155354,-0.0104706,0.0626348},
{0.0225419,-0.0272318,0.0492964},
{0.0108338,-0.0308878,0.0683295},
{0.0001751,-0.0004817,0.0557542},
{0.0125812,-0.0082821,0.0434500},
{-0.0066324,-0.0169628,0.0708659},
{0.0188680,-0.0468848,0.0564639},
{0.0169048,-0.0407108,0.0362825},
{0.0159677,-0.0175294,0.0274497},
{-0.0113619,-0.0389341,0.0676173},
{0.0037639,-0.0509670,0.0665017},
{-0.0034459,-0.0244052,0.0451047},
{-0.0139876,-0.0052377,0.0525315},
{-0.0000099,-0.0051379,0.0304783},
{-0.0137351,-0.0277925,0.0540005},
{0.0112846,-0.0737648,0.0593216},
{0.0179463,-0.0655317,0.0422610},
{0.0093551,-0.0553344,0.0211909},
{0.0108190,-0.0294694,0.0138244},
```

Biology Open • Supplementary information

{0.0205009,-0.0066860,0.0063191},
{0.0116007,0.0073715,0.0176354},
{-0.0136639,-0.0463590,0.0401869},
{-0.0112249,-0.0602642,0.0570973},
{-0.0103047,-0.0310216,0.0275872},
{-0.0068417,0.0129763,0.0176676},
{-0.0126968,-0.0071390,0.0174160},
{-0.0083098,-0.0852254,0.0495156},
{0.0070319,-0.0977943,0.0575209},
{0.0191106,-0.0916722,0.0407671},
{0.0092750,-0.0789446,0.0232845},
{-0.0131289,-0.0560537,0.0228835},
{-0.0046851,-0.0398441,0.0113067},
{-0.0004956,-0.0241830,-0.0053229},
{0.0142771,-0.0189535,-0.0072272},
{0.0192914,-0.0021788,-0.0123425},
{0.0189154,0.0157860,-0.0013436},
{0.0034961,0.0239562,0.0099808},
{-0.0158750,-0.0695825,0.0375494},
{-0.0131893,-0.0152049,0.0064271},
{-0.0134998,0.0206552,0.0017825},
{-0.0205940,0.0028635,0.0035167},
{-0.0075768,-0.0943994,0.0307494},
{-0.0047946,-0.1131861,0.0466255},
{0.0162685,-0.1188722,0.0496994},
{0.0138873,-0.1015643,0.0241435},
{-0.0112730,-0.0753951,0.0237689},
{-0.0161762,-0.0107605,-0.0113381},
{0.0007325,-0.0111128,-0.0207214},
{0.0066537,0.0094508,-0.0190732},
{0.0018722,0.0244466,-0.0090112},
{-0.0117105,0.0102276,-0.0161236},
{0.0004931,-0.1190765,0.0258592},
{-0.0020710,-0.1353783,0.0444594},
{0.0175201,-0.1433363,0.0474162},
{0.0208978,-0.1232233,0.0292110},
{0.0069488,-0.1399067,0.0214494},
{0.0000154,-0.1580761,0.0389313},
{0.0187529,-0.1678422,0.0449672},
{0.0249411,-0.1495654,0.0286769},
{0.0108104,-0.1628538,0.0183255},
{0.0026016,-0.1814816,0.0357496},
{0.0213387,-0.1926176,0.0419461},
{0.0275428,-0.1750228,0.0257788},
{0.0127674,-0.1876518,0.0156367},
{0.0042783,-0.2048604,0.0321700},

Biology Open • Supplementary information

{0.0216748,-0.2176696,0.0393755},
{0.0292725,-0.1981703,0.0233569},
{0.0143033,-0.2117950,0.0132317},
{0.0029409,-0.2289966,0.0238075},
{0.0155983,-0.2419230,0.0351337},
{0.0307148,-0.2394085,0.0267441},
{0.0296601,-0.2205584,0.0187246},
{0.0178726,-0.2391899,0.0098461},
{0.0026186,-0.2535606,0.0203281},
{0.0123550,-0.2714175,0.0306700},
{0.0275813,-0.2623329,0.0316599},
{0.0299426,-0.2597389,0.0144650},
{0.0157846,-0.2678939,0.0076327},
{0.0022110,-0.2825857,0.0172404},
{0.0135304,-0.2987517,0.0294161},
{0.0284725,-0.2857679,0.0282748},
{0.0297951,-0.2870832,0.0110542},
{0.0142031,-0.2964792,0.0049824},
{0.0032143,-0.3099698,0.0164284},
{0.0109366,-0.3263089,0.0269151},
{0.0289393,-0.3127785,0.0249611},
{0.0288565,-0.3117668,0.0067572},
{0.0111564,-0.3255120,0.0026724},
{0.0028653,-0.3433766,0.0117779},
{0.0122012,-0.3521983,0.0266023},
{0.0284743,-0.3394178,0.0230075},
{0.0290874,-0.3338547,0.0044210},
{0.0170016,-0.3492846,-0.0031025},
{0.0043064,-0.3686602,0.0008378},
{0.0027916,-0.3714832,0.0200743},
{0.0242812,-0.3708951,0.0238818},
{0.0317792,-0.3606907,0.0090394},
{0.0251750,-0.3757454,-0.0053191},
{0.0070965,-0.3899468,-0.0073397},
{-0.0001845,-0.3947382,0.0100122},
{0.0130164,-0.3922867,0.0259466},
{0.0318641,-0.3903787,0.0122171},
{0.0249297,-0.4002818,-0.0090114},
{0.0050158,-0.4141624,-0.0132508},
{-0.0023908,-0.4205919,0.0046358},
{0.0018014,-0.4125858,0.0233011},
{0.0245582,-0.4118321,0.0229998},
{0.0312720,-0.4158443,0.0055022},
{0.0237077,-0.4246221,-0.0141579},
{0.0107456,-0.4402620,-0.0261593},
{-0.0056701,-0.4348696,-0.0147295},

Biology Open • Supplementary information

{-0.0068919,-0.4492421,0.0030673},
{-0.0063479,-0.4390001,0.0217072},
{0.0112452,-0.4336120,0.0282574},
{0.0302394,-0.4362238,0.0192865},
{0.0289245,-0.4420973,-0.0001591},
{0.0274921,-0.4517726,-0.0194506},
{0.0191411,-0.4684118,-0.0323930},
{0.0046155,-0.4565596,-0.0408518},
{-0.0101841,-0.4459153,-0.0315579},
{-0.0175985,-0.4564032,-0.0135487},
{-0.0065137,-0.4753699,-0.0035364},
{-0.0188817,-0.4653938,0.0117777},
{-0.0163988,-0.4569247,0.0320503},
{0.0022814,-0.4519106,0.0375192},
{0.0225364,-0.4571417,0.0299325},
{0.0381419,-0.4583699,0.0170454},
{0.0294932,-0.4660076,-0.0019902},
{0.0319293,-0.4819917,-0.0175124},
{0.0187541,-0.4966479,-0.0296843},
{0.0027387,-0.4818074,-0.0424796},
{-0.0146260,-0.4668534,-0.0430118},
{-0.0296984,-0.4595692,-0.0304803},
{-0.0294630,-0.4765942,-0.0129244},
{-0.0188083,-0.4971703,-0.0190313},
{0.0001681,-0.4968505,-0.0126271},
{0.0032369,-0.4940142,0.0080815},
{-0.0154151,-0.4909264,0.0150075},
{-0.0264958,-0.4768014,0.0304993},
{-0.0047925,-0.4737047,0.0432813},
{0.0142458,-0.4782229,0.0357484},
{0.0347497,-0.4796584,0.0262778},
{0.0290357,-0.4851476,0.0077486},
{0.0179684,-0.4924632,-0.0069900},
{-0.0001555,-0.5038004,-0.0324124},
{-0.0155096,-0.4939238,-0.0410891},
{-0.0322306,-0.4831913,-0.0343716},
{0.0210165,-0.4978721,0.0225538},
{0.0016821,-0.5008647,0.0295842},
{-0.0156855,-0.4956879,0.0360378},
{-0.0009049,-0.0810472,0.0207645},
{-0.0108482,-0.0705533,0.0225174},
{0.0103408,-0.0413876,0.0673876},
{-0.0149207,-0.0168172,0.0621139},
{-0.0018290,-0.0689408,0.0624448},
{-0.0066184,-0.0774188,0.0244992},
{-0.0014823,-0.0994767,0.0223719},

Biology Open • Supplementary information

{-0.0073494,0.0020552,-0.0185380},
{-0.0131021,-0.0541079,0.0448707},
{0.0034742,-0.0322526,0.0714473},
{-0.0106827,-0.0799756,0.0280219},
{-0.0011219,-0.0657309,0.0200092},
{-0.0138534,-0.0206422,0.0553651},
{0.0143146,-0.0365037,0.0655574},
{-0.0110038,-0.0362567,0.0432712},
{0.0150257,-0.0417239,0.0637220},
{-0.0009369,-0.0908029,0.0585293},
{-0.0155818,-0.0531586,0.0340686},
{-0.0146814,-0.0674082,0.0239665},
{-0.0170965,-0.0659298,0.0253515},
{-0.0026080,-0.0501038,0.0147934},
{0.0113882,-0.0702368,0.0260348},
{0.0228152,-0.0160648,0.0431147},
{-0.0032856,-0.0533740,0.0674858},
{0.0142645,-0.0034019,0.0187589},
{-0.0093203,-0.0589686,0.0206015},
{-0.0062830,-0.0980294,0.0483190},
{0.0058766,0.0179020,0.0142905},
{-0.0119118,0.0205970,-0.0065511},
{-0.0074396,-0.0814406,0.0517202},
{0.0082642,-0.1024323,0.0211275},
{0.0136290,-0.0898559,0.0264476},
{0.0081658,-0.0570424,0.0639983},
{0.0152223,-0.0152462,0.0082602},
{-0.0060922,-0.0284286,0.0049819},
{-0.0075822,-0.0011726,0.0218497},
{-0.0106525,-0.0678963,0.0532199},
{-0.0065339,-0.0315892,0.0715154},
{0.0088743,-0.0076703,0.0653170},
{0.0143541,-0.0609775,0.0322238},
{-0.0021173,-0.0218775,-0.0131089},
{-0.0145421,-0.0295043,0.0593825},
{0.0168979,0.0160521,-0.0076292},
{-0.0042810,0.0260123,0.0039746},
{0.0006660,-0.1025832,0.0567689},
{-0.0137036,-0.0476917,0.0537620},
{0.0209511,-0.0337243,0.0456512},
{0.0122215,-0.0517676,0.0259524},
{0.0188040,0.0125299,0.0077674},
{0.0216710,0.0025094,0.0072551},
{0.0226849,-0.0023883,-0.0036722},
{0.0145313,-0.0242542,0.0242104},
{0.0120620,-0.0417493,0.0231154},

Biology Open • Supplementary information

{0.0171187,-0.0176999,0.0346339},
{-0.0183063,-0.0590510,0.0284703},
{0.0031676,-0.0461615,0.0693279},
{0.0177105,-0.0543707,0.0412581},
{0.0153082,-0.0121937,0.0142491},
{0.0010811,0.0052879,-0.0208522},
{0.0091361,-0.0009187,-0.0206937},
{0.0109423,-0.0811397,0.0576491},
{0.0156278,-0.0732711,0.0547644},
{0.0183557,-0.0711897,0.0455372},
{0.0185657,-0.0824135,0.0385103},
{-0.0049442,-0.0899188,0.0263553},
{-0.0138904,0.0044896,0.0149848},
{0.0080826,-0.0013490,0.0591734},
{0.0201102,-0.0112394,0.0539381},
{0.0156789,-0.0284073,0.0645405},
{-0.0124692,-0.0429479,0.0271588},
{-0.0192201,-0.0089943,-0.0027382},
{0.0171009,-0.0992956,0.0293551},
{-0.0072997,-0.0068903,-0.0203683},
{0.0043041,0.0169113,-0.0158688},
{-0.0097729,-0.0396379,0.0171344},
{-0.0155555,0.0123683,-0.0111493},
{-0.0211127,0.0059481,-0.0017794},
{-0.0095425,-0.0840180,0.0423490},
{0.0122208,-0.0208784,-0.0010159},
{0.0131527,-0.0544493,0.0614479},
{0.0091305,-0.0221369,-0.0096256},
{0.0105813,-0.0166717,-0.0147528},
{0.0045309,-0.0711575,0.0202917},
{0.0094340,-0.0607012,0.0229908},
{0.0012893,-0.0910001,0.0214950},
{-0.0191930,-0.0671408,0.0323372},
{-0.0063806,-0.0016276,0.0558709},
{-0.0138355,-0.0368436,0.0637288},
{0.0189465,-0.0207608,0.0579723},
{0.0158515,-0.0920761,0.0517003},
{0.0198583,-0.0287190,0.0375775},
{0.0088345,-0.0381031,0.0712749},
{-0.0179931,-0.0038877,0.0106404},
{0.0152582,-0.0193096,0.0645071},
{0.0163874,-0.0733001,0.0349471},
{-0.0160962,-0.0599999,0.0241437},
{-0.0148828,-0.0399272,0.0563975},
{0.0147161,-0.0498231,0.0327679},
{-0.0082818,-0.0463579,0.0690072},

Biology Open • Supplementary information

{0.0070327,-0.0092663,-0.0202089},
{0.0043852,-0.0184635,-0.0162515},
{-0.0152633,-0.0129098,0.0552047},
{-0.0110714,-0.0535879,0.0621320},
{0.0184989,-0.0647120,0.0506936},
{-0.0179126,0.0133700,0.0016823},
{-0.0146533,0.0109807,0.0124985},
{-0.0020208,-0.0814258,0.0588009},
{0.0140956,-0.0111213,0.0365918},
{0.0147360,-0.0041959,0.0591503},
{0.0162058,-0.0068777,0.0463537},
{-0.0135797,-0.0161137,-0.0092344},
{0.0060862,-0.0263005,0.0025266},
{0.0003921,-0.0587432,0.0183038},
{0.0212849,0.0037502,-0.0081584},
{0.0182439,0.0019458,-0.0141461},
{0.0136063,-0.0092420,-0.0169664},
{-0.0139640,0.0016446,-0.0165996},
{-0.0155924,-0.0071620,-0.0142238},
{0.0188216,0.0158547,0.0034454},
{0.0112464,0.0228613,-0.0024662},
{0.0095997,0.0166312,-0.0142157},
{0.0085781,-0.0270752,0.0693668},
{-0.0007564,-0.0103425,0.0684165},
{-0.0001764,-0.0025064,0.0616583},
{0.0057280,-0.0164407,0.0699901},
{0.0089440,0.0088082,0.0178023},
{0.0131274,-0.0208564,0.0168814},
{0.0041959,-0.0303002,0.0711700},
{-0.0097213,-0.0207104,0.0262900},
{-0.0108955,-0.0209317,0.0131238},
{-0.0069058,-0.0119875,0.0293561},
{-0.0160762,-0.0607963,0.0384041},
{-0.0121126,-0.0734962,0.0444522},
{-0.0073933,-0.0348843,0.0101620},
{0.0220985,-0.0221836,0.0501664},
{0.0130581,-0.0101485,0.0300440},
{0.0046144,-0.0011346,0.0263217},
{-0.0038262,-0.0911828,0.0572317},
{-0.0025675,0.0263114,-0.0035467},
{-0.0012203,-0.0158565,0.0424391},
{-0.0116758,-0.0172323,0.0026721},
{0.0138405,-0.0345828,0.0278160},
{0.0110283,-0.0326167,0.0185860},
{-0.0117510,-0.0083467,0.0571280},
{0.0200072,-0.0397971,0.0520369},

{-0.0128943,-0.0643148,0.0450750},
{-0.0079799,-0.0972077,0.0408647},
{0.0046282,-0.0063068,0.0334680},
{-0.0031921,-0.0055356,-0.0216583},
{-0.0134727,-0.0417666,0.0632506},
{-0.0032151,0.0106995,-0.0183093},
{-0.0046165,0.0199481,-0.0135214},
{0.0198812,-0.1031863,0.0393964},
{0.0039450,-0.0889866,0.0206464},
{0.0175862,-0.1064639,0.0484310},
{0.0102429,0.0230826,0.0058369},
{0.0125467,0.0110038,0.0158114},
{0.0011737,0.0181303,-0.0155165},
{0.0171753,-0.0140596,-0.0095973},
{0.0202266,-0.0091963,-0.0082303},
{-0.0092936,-0.0293055,0.0330051},
{-0.0116477,-0.0416732,0.0348878},
{-0.0143166,-0.0744339,0.0279082},
{-0.0015123,0.0032112,0.0217971},
{0.0044367,0.0088358,0.0188049},
{-0.0026869,-0.0034426,0.0278464},
{0.0002742,-0.0371935,0.0706302},
{0.0073685,-0.0524312,0.0650018},
{-0.0124794,-0.0239734,0.0680917},
{-0.0124505,-0.0317922,0.0672567},
{0.0096397,0.0244629,0.0028735},
{0.0182344,0.0170871,0.0015247},
{-0.0075973,-0.0131827,0.0693269},
{0.0162329,-0.0642099,0.0372998},
{-0.0132296,-0.0574541,0.0521429},
{-0.0160008,-0.0609296,0.0238844},
{0.0116805,-0.1018452,0.0549164},
{-0.0104428,-0.0304934,0.0192150},
{-0.0107638,-0.0185691,0.0184753},
{-0.0047164,0.0193223,0.0137111},
{0.0009529,-0.0823316,0.0607116},
{0.0062731,-0.0759059,0.0608982},
{-0.0024058,-0.0755295,0.0603502},
{0.0176539,-0.0890035,0.0337088},
{-0.0132673,-0.0220978,0.0531224},
{0.0113642,-0.0300245,0.0187038},
{0.0054893,-0.0634101,0.0635775},
{0.0088444,-0.0423779,0.0675945},
{0.0101592,-0.1017864,0.0215379},
{-0.0072184,-0.0638688,0.0617294},
{0.0190899,-0.0516765,0.0477102},

{-0.0126919,-0.0516553,0.0229470},
{0.0225614,-0.0248809,0.0405875},
{-0.0042899,-0.0735382,0.0220128},
{0.0177254,-0.0024015,0.0138463},
{0.0065716,0.0065156,0.0189668},
{0.0009751,-0.0391023,0.0705025},
{-0.0157811,0.0165379,-0.0055007},
{0.0112929,0.0188904,0.0110152},
{-0.0113607,0.0192741,0.0088385},
{-0.0198111,-0.0066146,-0.0055964},
{-0.0199291,0.0006118,-0.0080278},
{-0.0052175,-0.0210447,0.0719322},
{-0.0139988,-0.0412941,0.0471634},
{0.0059479,-0.0563454,0.0649712},
{0.0132382,-0.0500416,0.0625399},
{-0.0093060,-0.0820864,0.0275058},
{0.0134481,0.0085177,0.0162827},
{0.0194111,-0.0128502,-0.0012155},
{-0.0184438,-0.0096549,0.0054181},
{-0.0136010,-0.0675406,0.0417938},
{-0.0077857,-0.0202153,-0.0040224},
{-0.0137722,-0.0330578,0.0505529},
{0.0049538,-0.0898150,0.0590336},
{0.0170071,-0.0598685,0.0556538},
{-0.0018475,-0.0417963,0.0118340},
{0.0066516,-0.0383822,0.0141056},
{0.0199879,-0.0083558,0.0062029},
{0.0192999,-0.0976262,0.0447502},
{-0.0097239,-0.0226919,0.0269418},
{-0.0009357,0.0155300,0.0172425},
{0.0078833,-0.0818016,0.0227098},
{0.0093633,-0.0747518,0.0602072},
{0.0159930,-0.0155361,-0.0101071},
{0.0005919,-0.0109065,0.0689263},
{-0.0076132,-0.0176682,-0.0151539},
{0.0169239,-0.0844513,0.0502702},
{0.0081860,-0.0220599,-0.0110608},
{-0.0113014,-0.0772160,0.0248327},
{0.0141728,-0.0470908,0.0310311},
{0.0165199,-0.0051225,0.0575957},
{0.0050507,-0.0091467,0.0419831},
{-0.0032324,-0.0739472,0.0609461},
{-0.0136306,-0.0712740,0.0407007},
{-0.0153875,-0.0136758,0.0576340},
{-0.0083670,0.0146841,-0.0156251},
{-0.0182774,0.0069242,0.0086914},

Biology Open • Supplementary information

{0.0099512,-0.0814802,0.0236757},
{0.0026548,-0.0465154,0.0683546},
{-0.0143741,-0.0259287,0.0586291},
{0.0068551,-0.0073336,-0.0209568},
{0.0163983,-0.0337720,0.0602661},
{0.0127347,-0.0178946,0.0661306},
{0.0110914,0.0024709,-0.0193028},
{0.0145905,-0.0096720,0.0183312},
{-0.0137737,0.0131892,0.0124674},
{0.0110262,-0.0625764,0.0613875},
{-0.0058177,-0.0454429,0.0698857},
{0.0206944,-0.0343859,0.0533734},
{0.0168671,-0.0156519,-0.0074759},
{-0.0158818,-0.0593754,0.0389386},
{-0.0080375,-0.0871811,0.0482759},
{0.0034054,-0.0622160,0.0643659},
{-0.0046985,-0.0489548,0.0149699},
{0.0118531,0.0126339,-0.0156074},
{-0.0022811,-0.0255322,-0.0009018},
{-0.0097573,-0.0866003,0.0322876},
{-0.0109932,-0.0041367,-0.0187702},
{-0.0031038,0.0255967,-0.0049932},
{-0.0102047,-0.0256435,0.0141740},
{-0.0114326,-0.0760736,0.0246430},
{-0.0063298,-0.0882310,0.0265316},
{0.0010145,0.0139968,-0.0178552},
{0.0211236,-0.0306774,0.0525525},
{-0.0062754,-0.0684633,0.0212899},
{0.0187679,-0.0428528,0.0424714},
{0.0187407,-0.0363252,0.0378396},
{-0.0152868,-0.0141133,-0.0010487},
{0.0141288,0.0165364,0.0108596},
{-0.0142529,-0.0128716,0.0074587},
{-0.0113901,-0.0415156,0.0385020},
{0.0191215,-0.1034772,0.0457036},
{0.0188271,-0.0121844,0.0599947},
{0.0199834,-0.0183460,0.0555806},
{0.0014503,-0.0312144,0.0054102},
{0.0114929,-0.0275256,0.0676864},
{-0.0029712,-0.0112401,0.0338277},
{-0.0205339,-0.0049990,-0.0018729},
{0.0029374,-0.0247486,-0.0041044},
{-0.0103155,-0.0837530,0.0398132},
{-0.0135684,-0.0323513,0.0656314},
{0.0190573,0.0061081,-0.0115943},
{-0.0157329,-0.0732752,0.0324523},

{-0.0162249,-0.0049411,0.0137803},
{0.0127761,-0.0094559,0.0291404},
{-0.0143058,-0.0504053,0.0345159},
{0.0160281,-0.0841795,0.0310084},
{0.0008195,0.0039627,-0.0211244},
{0.0179818,-0.0911303,0.0338337},
{0.0073944,0.0075026,0.0181735},
{-0.0152159,0.0026568,0.0139537},
{0.0062316,-0.0504543,0.0178881},
{-0.0068971,-0.0223634,0.0366618},
{0.0218898,-0.0316829,0.0463097},
{-0.0137472,-0.0735647,0.0247790},
{0.0222151,0.0060097,-0.0032819},
{-0.0147810,-0.0737595,0.0301924},
{0.0120002,-0.0262850,0.0120370},
{0.0210276,-0.0137385,0.0494034},
{0.0092281,0.0022858,-0.0203233},
{0.0157179,-0.0097566,0.0147324},
{-0.0120833,-0.0746598,0.0437509},
{0.0004936,0.0241884,0.0100309},
{0.0030283,0.0038803,-0.0214706},
{0.0402192,-0.4734774,0.0229436},
{0.0054506,0.0239680,-0.0085008},
{-0.0145637,-0.0528482,0.0407466},
{-0.0338453,-0.4686824,-0.0293994},
{0.0024982,0.0271154,-0.0000349},
{0.0154950,-0.0288090,0.0290556},
{-0.0152201,-0.0012918,-0.0158781},
{0.0178480,-0.0363786,0.0579180},
{-0.0157995,0.0157893,0.0051830},
{0.0186072,-0.0470621,0.0437694},
{0.0166063,-0.0525024,0.0375430},
{-0.0143098,-0.0414128,0.0500334},
{0.0186845,-0.0664988,0.0481535},
{-0.0180351,-0.0577866,0.0300506},
{0.0032952,-0.0660197,0.0635465},
{0.0011726,-0.0615874,0.0189780},
{0.0230856,-0.0250947,0.0481383},
{0.0085233,-0.0917027,0.0576027},
{-0.0064127,-0.0995585,0.0305330},
{-0.0204596,-0.0025837,0.0033375},
{0.0155081,-0.0160857,0.0215757},
{0.0149156,-0.0799295,0.0300212},
{-0.0100130,-0.0296318,0.0300672},
{-0.0210062,-0.4805332,0.0390121},
{-0.0093212,-0.0669209,0.0570094},

{-0.0123340,-0.4740365,-0.0112308},
{0.0114953,-0.0518830,0.0244706},
{0.0116830,0.0174141,-0.0122287},
{-0.0262286,-0.4701250,0.0315665},
{0.0168388,-0.0637387,0.0382624},
{0.0003212,-0.0594351,0.0653910},
{-0.0118201,0.0217004,0.0031072},
{-0.0026553,-0.0200272,0.0717796},
{-0.0078499,-0.0088384,0.0263433},
{0.0208530,-0.0123597,0.0461100},
{0.0159694,-0.0293747,0.0624689},
{-0.0152062,-0.0130815,0.0568346},
{0.0213132,0.0098780,-0.0030655},
{-0.0107804,-0.0409208,0.0195779},
{-0.0003622,-0.0169390,-0.0179516},
{-0.0115873,-0.0722387,0.0482302},
{-0.0096828,0.0205386,0.0087570},
{0.0104964,0.0229587,-0.0032770},
{-0.0144036,-0.0324289,0.0622405},
{-0.0064307,-0.4707107,0.0442203},
{-0.0144409,-0.4771105,-0.0414462},
{-0.0143608,-0.4770465,-0.0405896},
{-0.0142807,-0.4769825,-0.0397329},
{-0.0142006,-0.4769185,-0.0388762},
{-0.0141205,-0.4768545,-0.0380196},
{-0.0140404,-0.4767905,-0.0371629},
{-0.0139603,-0.4767265,-0.0363062},
{-0.0138802,-0.4766625,-0.0354496},
{-0.0138001,-0.4765985,-0.0345929},
{-0.0137200,-0.4765345,-0.0337362},
{-0.0136399,-0.4764705,-0.0328796},
{-0.0135598,-0.4764065,-0.0320229},
{-0.0134797,-0.4763425,-0.0311663},
{-0.0133996,-0.4762785,-0.0303096},
{-0.0133195,-0.4762145,-0.0294529},
{-0.0132394,-0.4761505,-0.0285963},
{-0.0131593,-0.4760865,-0.0277396},
{-0.0130792,-0.4760225,-0.0268829},
{-0.0129991,-0.4759585,-0.0260263},
{-0.0129190,-0.4758945,-0.0251696},
{-0.0128389,-0.4758305,-0.0243129},
{-0.0127588,-0.4757665,-0.0234563},
{-0.0126787,-0.4757025,-0.0225996},
{-0.0125986,-0.4756385,-0.0217429},
{-0.0125185,-0.4755745,-0.0208863},
{-0.0124384,-0.4755105,-0.0200296},

Biology Open • Supplementary information

{-0.0123583,-0.4754465,-0.0191729},
{-0.0122782,-0.4753825,-0.0183163},
{-0.0121981,-0.4753185,-0.0174596},
{-0.0121180,-0.4752545,-0.0166029},
{-0.0120379,-0.4751905,-0.0157463},
{-0.0119578,-0.4751265,-0.0148896},
{-0.0118777,-0.4750626,-0.0140329},
{-0.0117975,-0.4749986,-0.0131763},
{-0.0117174,-0.4749346,-0.0123196},
{-0.0116373,-0.4748706,-0.0114630},
{-0.0115572,-0.4748066,-0.0106063},
{-0.0114771,-0.4747426,-0.0097496},
{-0.0113970,-0.4746786,-0.0088930},
{-0.0113169,-0.4746146,-0.0080363},
{-0.0112368,-0.4745506,-0.0071796},
{-0.0111567,-0.4744866,-0.0063230},
{-0.0110766,-0.4744226,-0.0054663},
{-0.0109965,-0.4743586,-0.0046096},
{-0.0109164,-0.4742946,-0.0037530},
{-0.0108363,-0.4742306,-0.0028963},
{-0.0107562,-0.4741666,-0.0020396},
{-0.0106761,-0.4741026,-0.0011830},
{-0.0105960,-0.4740386,-0.0003263},
{-0.0105159,-0.4739746,0.0005304},
{-0.0104358,-0.4739106,0.0013870},
{-0.0103557,-0.4738466,0.0022437},
{-0.0102756,-0.4737826,0.0031004},
{-0.0101955,-0.4737186,0.0039570},
{-0.0101154,-0.4736546,0.0048137},
{-0.0100353,-0.4735906,0.0056704},
{-0.0099552,-0.4735266,0.0065270},
{-0.0098751,-0.4734626,0.0073837},
{-0.0097950,-0.4733986,0.0082403},
{-0.0097149,-0.4733346,0.0090970},
{-0.0096348,-0.4732706,0.0099537},
{-0.0095547,-0.4732066,0.0108103},
{-0.0094746,-0.4731426,0.0116670},
{-0.0093945,-0.4730786,0.0125237},
{-0.0093144,-0.4730146,0.0133803},
{-0.0092343,-0.4729506,0.0142370},
{-0.0091542,-0.4728866,0.0150937},
{-0.0090741,-0.4728226,0.0159503},
{-0.0089940,-0.4727586,0.0168070},
{-0.0089138,-0.4726946,0.0176637},
{-0.0088337,-0.4726306,0.0185203},
{-0.0087536,-0.4725666,0.0193770},

Biology Open • Supplementary information

```
                    {-0.0086735,-0.4725026,0.0202337},
                    {-0.0085934,-0.4724387,0.0210903},
                    {-0.0085133,-0.4723747,0.0219470},
                    {-0.0084332,-0.4723107,0.0228037},
                    {-0.0083531,-0.4722467,0.0236603},
                    {-0.0082730,-0.4721827,0.0245170},
                    {-0.0081929,-0.4721187,0.0253737},
                    {-0.0081128,-0.4720547,0.0262303},
                    {-0.0080327,-0.4719907,0.0270870},
                    {-0.0079526,-0.4719267,0.0279437},
                    {-0.0078725,-0.4718627,0.0288003},
                    {-0.0077924,-0.4717987,0.0296570},
                    {-0.0077123,-0.4717347,0.0305136},
                    {-0.0076322,-0.4716707,0.0313703},
                    {-0.0075521,-0.4716067,0.0322270},
                    {-0.0074720,-0.4715427,0.0330836},
                    {-0.0073919,-0.4714787,0.0339403},
                    {-0.0073118,-0.4714147,0.0347970},
                    {-0.0072317,-0.4713507,0.0356536},
                    {-0.0071516,-0.4712867,0.0365103},
                    {-0.0070715,-0.4712227,0.0373670},
                    {-0.0069914,-0.4711587,0.0382236},
                    {-0.0069113,-0.4710947,0.0390803},
                    {-0.0068312,-0.4710307,0.0399370},
                    {-0.0067511,-0.4709667,0.0407936},
                    {-0.0066710,-0.4709027,0.0416503},
                    {-0.0065909,-0.4708387,0.0425070},
                    {-0.0065108,-0.4707747,0.0433636}
                    }* .AMirroring);
            BoundingBoxOnOff = Off;
        };
        AnyFunTransform3DIdentity ScaleFunction = {
            PreTransforms = {&.RBFTransform};
};
};

};
};
```

Biology Open • Supplementary information

ScalingFunctionTLEMLucyPelvis_2014030

```
AnyFolder ScalingFunctionTLEMLucyPelvis = {
AnyFolder Pelvis = {
	AnyFunTransform3DRBF RBFTransform = {
		RBFDef.Type = RBF_ThinPlate;
		PolynomDegree = 1;
		Points0 = {
			{0.0000000,0.0000000,0.1177000},
			{-0.0000000,-0.0832729,0.0191000},
			{-0.0508179,-0.0694062,0.0815920},
			{0.0000000,0.0000000,-0.1177000},
			{-0.0000000,-0.0832729,-0.0191000},
			{-0.0508179,-0.0694062,-0.0815920},
			{0.0000000,0.0000000,0.0000000},
			{-0.1164020,-0.0039449,0.0456760},
			{-0.1164020,-0.0039449,-0.0456760},
			{-0.1092909,-0.0992949,0.0515590},
			{-0.1092909,-0.0992949,-0.0515590},
			{-0.0733749,0.0757445,0.0915590},
			{-0.0733749,0.0757445,-0.0915590},
			{-0.1241738,0.0316243,0.0458050},
			{-0.1241738,0.0316243,-0.0458050},
			{-0.1108008,0.0563490,0.0555690},
			{-0.1108008,0.0563490,-0.0555690},
			{-0.0545356,0.0481653,0.1291690},
			{-0.0545356,0.0481653,-0.1291690},
			{-0.0360236,0.0456352,0.1316250},
			{-0.0360236,0.0456352,-0.1316250},
			{-0.0804664,-0.0228481,0.0672140},
			{-0.0804664,-0.0228481,-0.0672140},
			{-0.1004070,-0.0623213,0.0494990},
			{-0.1004070,-0.0623213,-0.0494990},
			{-0.0174901,-0.0357786,0.0977400},
			{-0.0174901,-0.0357786,-0.0977400},
			{-0.0665950,-0.1308418,0.0278190},
			{-0.0665950,-0.1308418,-0.0278190},
			{-0.0962739,-0.1257274,0.0551670},
			{-0.0962739,-0.1257274,-0.0551670},
			{-0.0393340,-0.1196098,0.0159820},
			{-0.0393340,-0.1196098,-0.0159820},
			{-0.0585979,-0.0148191,0.0651020},
			{-0.0585979,-0.0148191,-0.0651020},
			{-0.0238420,-0.0800479,0.0351680},
			{-0.0238420,-0.0800479,-0.0351680},
			{-0.0234553,-0.0576924,0.0715330},
```

Biology Open • Supplementary information

{-0.0234553,-0.0576924,-0.0715330},
{-0.0472319,-0.0833276,0.0513790},
{-0.0472319,-0.0833276,-0.0513790},
{-0.0327175,-0.0506481,0.0520240},
{-0.0327175,-0.0506481,-0.0520240},
{-0.0746024,-0.0627219,0.0928110},
{-0.0746024,-0.0627219,-0.0928110},
{-0.0119676,-0.0928080,0.0076900},
{-0.0119676,-0.0928080,-0.0076900},
{-0.0263299,-0.0760728,0.0667080},
{-0.0263299,-0.0760728,-0.0667080},
{-0.0498842,0.0119450,0.1070630},
{-0.0498842,0.0119450,-0.1070630},
{-0.0553874,0.0627474,0.1172390},
{-0.0553874,0.0627474,-0.1172390},
{-0.0786774,0.0073556,0.0741800},
{-0.0786774,0.0073556,-0.0741800},
{-0.0742762,0.0474272,0.0542330},
{-0.0742762,0.0474272,-0.0542330},
{-0.0634121,0.0117185,0.0618430},
{-0.0634121,0.0117185,-0.0618430},
{-0.0813084,0.0007821,0.0508130},
{-0.0813084,0.0007821,-0.0508130},
{-0.0496423,-0.0444458,0.0983920},
{-0.0496423,-0.0444458,-0.0983920},
{-0.0480005,-0.0338999,0.0625080},
{-0.0480005,-0.0338999,-0.0625080},
{-0.0428000,-0.0273188,0.0973490},
{-0.0428000,-0.0273188,-0.0973490},
{-0.0548383,-0.0518766,0.0624050},
{-0.0548383,-0.0518766,-0.0624050},
{-0.0696448,-0.1052827,0.0436200},
{-0.0696448,-0.1052827,-0.0436200},
{-0.0739608,-0.0803177,0.0785950},
{-0.0739608,-0.0803177,-0.0785950},
{-0.0586066,-0.0884764,0.0653190},
{-0.0586066,-0.0884764,-0.0653190},
{-0.1017003,0.0085215,0.0624150},
{-0.1017003,0.0085215,-0.0624150},
{-0.0126252,0.0252852,0.1292530},
{-0.0126252,0.0252852,-0.1292530},
{-0.0030963,0.0150156,0.1262640},
{-0.0030963,0.0150156,-0.1262640},
{-0.0621797,0.0048743,0.0605920},
{-0.0621797,0.0048743,-0.0605920},
{-0.0870082,-0.0684280,0.0489900},

{-0.0870082,-0.0684280,-0.0489900},
{-0.0916211,-0.0478344,0.0575290},
{-0.0916211,-0.0478344,-0.0575290},
{-0.0428573,-0.0467026,0.0985250},
{-0.0428573,-0.0467026,-0.0985250},
{-0.0483869,-0.0455474,0.0848580},
{-0.0483869,-0.0455474,-0.0848580},
{-0.0203929,-0.0681811,0.0426070},
{-0.0203929,-0.0681811,-0.0426070},
{-0.0271973,-0.0807786,0.0491650},
{-0.0271973,-0.0807786,-0.0491650},
{-0.0325935,-0.1026813,0.0148280},
{-0.0325935,-0.1026813,-0.0148280},
{-0.0273900,-0.1059173,0.0048750},
{-0.0273900,-0.1059173,-0.0048750},
{-0.0015971,-0.0778770,0.0065530},
{-0.0015971,-0.0778770,-0.0065530},
{-0.1034983,-0.0140026,0.0521800},
{-0.1034983,-0.0140026,-0.0521800},
{-0.1141160,0.0077734,0.0497600},
{-0.1141160,0.0077734,-0.0497600},
{-0.1123797,0.0244808,0.0350160},
{-0.1123797,0.0244808,-0.0350160},
{-0.0984470,-0.0648210,0.0457100},
{-0.0984470,-0.0648210,-0.0457100},
{-0.0814332,-0.0704479,0.0806410},
{-0.0814332,-0.0704479,-0.0806410},
{-0.0540218,-0.0805070,0.0547320},
{-0.0540218,-0.0805070,-0.0547320},
{-0.0674356,-0.0763171,0.0651080},
{-0.0674356,-0.0763171,-0.0651080},
{-0.0602364,-0.0751827,0.0490600},
{-0.0602364,-0.0751827,-0.0490600},
{-0.0604272,-0.0801096,0.0617610},
{-0.0604272,-0.0801096,-0.0617610},
{-0.0655251,0.0632155,0.1093750},
{-0.0655251,0.0632155,-0.1093750},
{-0.0665046,0.0014289,0.0572170},
{-0.0665046,0.0014289,-0.0572170},
{-0.1053786,0.0347803,0.0071580},
{-0.1053786,0.0347803,-0.0071580},
{-0.1321649,-0.0069725,0.0058340},
{-0.1321649,-0.0069725,-0.0058340},
{-0.1232735,-0.0434903,0.0301110},
{-0.1232735,-0.0434903,-0.0301110},
{-0.0497024,0.0261862,0.0084640},

```
        {-0.0497024,0.0261862,-0.0084640},
        {-0.0887827,0.0446048,0.0327030},
        {-0.0887827,0.0446048,-0.0327030},
        {-0.1153998,-0.0126010,0.0290730},
        {-0.1153998,-0.0126010,-0.0290730},
        {-0.0899939,0.0093882,0.0208440},
        {-0.0899939,0.0093882,-0.0208440},
        {-0.1312654,-0.0574898,0.0094690},
        {-0.1312654,-0.0574898,-0.0094690},
        {-0.0996029,0.0042806,0.0105620},
        {-0.0996029,0.0042806,-0.0105620},
        {-0.1226177,-0.0324069,0.0331630},
        {-0.1226177,-0.0324069,-0.0331630},
        {-0.1097808,-0.0051619,0.0102520},
        {-0.1097808,-0.0051619,-0.0102520}
        };
Points1 = {
        {0.0000000,0.0000000,0.1332421},
        {-0.0000008,-0.0978063,0.0194018},
        {-0.0334116,-0.0759491,0.0927219},
        {0.0000000,0.0000000,-0.1332421},
        {-0.0000008,-0.0978063,-0.0194018},
        {-0.0334116,-0.0759491,-0.0927219},
        {0.0000000,0.0000000,0.0000000},
        {-0.0912291,-0.0319400,0.0451337},
        {-0.0912291,-0.0319400,-0.0451337},
        {-0.0864709,-0.1074158,0.0658982},
        {-0.0864709,-0.1074158,-0.0658982},
        {-0.0593653,0.0449209,0.0971779},
        {-0.0593653,0.0449209,-0.0971779},
        {-0.0963112,-0.0080019,0.0400156},
        {-0.0963112,-0.0080019,-0.0400156},
        {-0.0874276,0.0168258,0.0531313},
        {-0.0874276,0.0168258,-0.0531313},
        {-0.0366496,0.0344791,0.1379698},
        {-0.0366496,0.0344791,-0.1379698},
        {-0.0238839,0.0348272,0.1424991},
        {-0.0238839,0.0348272,-0.1424991},
        {-0.0645658,-0.0433377,0.0731577},
        {-0.0645658,-0.0433377,-0.0731577},
        {-0.0806689,-0.0792226,0.0676743},
        {-0.0806689,-0.0792226,-0.0676743},
        {-0.0120215,-0.0424656,0.1000335},
        {-0.0120215,-0.0424656,-0.1000335},
        {-0.0558726,-0.1352730,0.0363684},
        {-0.0558726,-0.1352730,-0.0363684},
```

{-0.0755837,-0.1254000,0.0693022},
{-0.0755837,-0.1254000,-0.0693022},
{-0.0314344,-0.1296351,0.0202902},
{-0.0314344,-0.1296351,-0.0202902},
{-0.0502385,-0.0308863,0.0706880},
{-0.0502385,-0.0308863,-0.0706880},
{-0.0159728,-0.0891791,0.0366486},
{-0.0159728,-0.0891791,-0.0366486},
{-0.0168089,-0.0633898,0.0785723},
{-0.0168089,-0.0633898,-0.0785723},
{-0.0312073,-0.0863234,0.0602862},
{-0.0312073,-0.0863234,-0.0602862},
{-0.0261244,-0.0636352,0.0611464},
{-0.0261244,-0.0636352,-0.0611464},
{-0.0529726,-0.0712372,0.0975225},
{-0.0529726,-0.0712372,-0.0975225},
{-0.0076419,-0.1085062,0.0083302},
{-0.0076419,-0.1085062,-0.0083302},
{-0.0178614,-0.0763821,0.0715832},
{-0.0178614,-0.0763821,-0.0715832},
{-0.0386914,-0.0010728,0.1134807},
{-0.0386914,-0.0010728,-0.1134807},
{-0.0412967,0.0428782,0.1258768},
{-0.0412967,0.0428782,-0.1258768},
{-0.0655987,-0.0154000,0.0741411},
{-0.0655987,-0.0154000,-0.0741411},
{-0.0628225,0.0151277,0.0534290},
{-0.0628225,0.0151277,-0.0534290},
{-0.0570413,-0.0110566,0.0601161},
{-0.0570413,-0.0110566,-0.0601161},
{-0.0686667,-0.0249276,0.0499126},
{-0.0686667,-0.0249276,-0.0499126},
{-0.0332565,-0.0545720,0.1008840},
{-0.0332565,-0.0545720,-0.1008840},
{-0.0383185,-0.0469715,0.0720957},
{-0.0383185,-0.0469715,-0.0720957},
{-0.0309385,-0.0377205,0.1012787},
{-0.0309385,-0.0377205,-0.1012787},
{-0.0395879,-0.0631486,0.0738729},
{-0.0395879,-0.0631486,-0.0738729},
{-0.0535218,-0.1108121,0.0566324},
{-0.0535218,-0.1108121,-0.0566324},
{-0.0521301,-0.0870970,0.0887070},
{-0.0521301,-0.0870970,-0.0887070},
{-0.0397475,-0.0947532,0.0790837},
{-0.0397475,-0.0947532,-0.0790837},

{-0.0812831,-0.0186390,0.0614779},
{-0.0812831,-0.0186390,-0.0614779},
{-0.0079531,0.0205263,0.1428622},
{-0.0079531,0.0205263,-0.1428622},
{-0.0014291,0.0129421,0.1411046},
{-0.0014291,0.0129421,-0.1411046},
{-0.0567609,-0.0165836,0.0586430},
{-0.0567609,-0.0165836,-0.0586430},
{-0.0678573,-0.0821086,0.0672240},
{-0.0678573,-0.0821086,-0.0672240},
{-0.0720064,-0.0656874,0.0702901},
{-0.0720064,-0.0656874,-0.0702901},
{-0.0274433,-0.0566022,0.1003966},
{-0.0274433,-0.0566022,-0.1003966},
{-0.0336006,-0.0557893,0.0902259},
{-0.0336006,-0.0557893,-0.0902259},
{-0.0141795,-0.0789703,0.0444031},
{-0.0141795,-0.0789703,-0.0444031},
{-0.0180590,-0.0861726,0.0526112},
{-0.0180590,-0.0861726,-0.0526112},
{-0.0225999,-0.1146328,0.0184857},
{-0.0225999,-0.1146328,-0.0184857},
{-0.0167761,-0.1182447,0.0072213},
{-0.0167761,-0.1182447,-0.0072213},
{-0.0019420,-0.0964225,0.0067012},
{-0.0019420,-0.0964225,-0.0067012},
{-0.0815903,-0.0382171,0.0560905},
{-0.0815903,-0.0382171,-0.0560905},
{-0.0898203,-0.0210697,0.0475520},
{-0.0898203,-0.0210697,-0.0475520},
{-0.0881824,-0.0126578,0.0305732},
{-0.0881824,-0.0126578,-0.0305732},
{-0.0795436,-0.0812653,0.0662460},
{-0.0795436,-0.0812653,-0.0662460},
{-0.0590484,-0.0795893,0.0902848},
{-0.0590484,-0.0795893,-0.0902848},
{-0.0353358,-0.0830233,0.0654653},
{-0.0353358,-0.0830233,-0.0654653},
{-0.0469616,-0.0848991,0.0789605},
{-0.0469616,-0.0848991,-0.0789605},
{-0.0420012,-0.0826668,0.0617058},
{-0.0420012,-0.0826668,-0.0617058},
{-0.0406875,-0.0878237,0.0761681},
{-0.0406875,-0.0878237,-0.0761681},
{-0.0511473,0.0392327,0.1169338},
{-0.0511473,0.0392327,-0.1169338},

Biology Open • Supplementary information

```
                    {-0.0603569,-0.0207512,0.0544735},
                    {-0.0603569,-0.0207512,-0.0544735},
                    {-0.0834788,-0.0065606,0.0061441},
                    {-0.0834788,-0.0065606,-0.0061441},
                    {-0.1020256,-0.0414178,0.0048891},
                    {-0.1020256,-0.0414178,-0.0048891},
                    {-0.1010170,-0.0717053,0.0229517},
                    {-0.1010170,-0.0717053,-0.0229517},
                    {-0.0455382,-0.0074961,0.0083509},
                    {-0.0455382,-0.0074961,-0.0083509},
                    {-0.0730399,0.0067307,0.0302592},
                    {-0.0730399,0.0067307,-0.0302592},
                    {-0.0904854,-0.0431133,0.0276642},
                    {-0.0904854,-0.0431133,-0.0276642},
                    {-0.0729581,-0.0249318,0.0195826},
                    {-0.0729581,-0.0249318,-0.0195826},
                    {-0.1076768,-0.0839008,0.0071734},
                    {-0.1076768,-0.0839008,-0.0071734},
                    {-0.0776902,-0.0320147,0.0098262},
                    {-0.0776902,-0.0320147,-0.0098262},
                    {-0.0986706,-0.0610156,0.0292201},
                    {-0.0986706,-0.0610156,-0.0292201},
                    {-0.0839041,-0.0406218,0.0094151},
                    {-0.0839041,-0.0406218,-0.0094151}
                    };
            BoundingBoxOnOff = Off;
        };
        AnyFunTransform3DIdentity ScaleFunction = {
            PreTransforms = {&.RBFTransform};
};
};
AnyFolder Sacrum = {
        AnyFunTransform3DRBF RBFTransform = {
            RBFDef.Type = RBF_ThinPlate;
            PolynomDegree = 1;
            Points0 = {
                    {0.0000000,0.0000000,0.1177000},
                    {-0.0000000,-0.0832729,0.0191000},
                    {-0.0508179,-0.0694062,0.0815920},
                    {0.0000000,0.0000000,-0.1177000},
                    {-0.0000000,-0.0832729,-0.0191000},
                    {-0.0508179,-0.0694062,-0.0815920},
                    {0.0000000,0.0000000,0.0000000},
                    {-0.1164020,-0.0039449,0.0456760},
                    {-0.1164020,-0.0039449,-0.0456760},
                    {-0.1092909,-0.0992949,0.0515590},
```

Biology Open • Supplementary information

{-0.1092909,-0.0992949,-0.0515590},
{-0.0733749,0.0757445,0.0915590},
{-0.0733749,0.0757445,-0.0915590},
{-0.1241738,0.0316243,0.0458050},
{-0.1241738,0.0316243,-0.0458050},
{-0.1108008,0.0563490,0.0555690},
{-0.1108008,0.0563490,-0.0555690},
{-0.0545356,0.0481653,0.1291690},
{-0.0545356,0.0481653,-0.1291690},
{-0.0360236,0.0456352,0.1316250},
{-0.0360236,0.0456352,-0.1316250},
{-0.0804664,-0.0228481,0.0672140},
{-0.0804664,-0.0228481,-0.0672140},
{-0.1004070,-0.0623213,0.0494990},
{-0.1004070,-0.0623213,-0.0494990},
{-0.0174901,-0.0357786,0.0977400},
{-0.0174901,-0.0357786,-0.0977400},
{-0.0665950,-0.1308418,0.0278190},
{-0.0665950,-0.1308418,-0.0278190},
{-0.0962739,-0.1257274,0.0551670},
{-0.0962739,-0.1257274,-0.0551670},
{-0.0393340,-0.1196098,0.0159820},
{-0.0393340,-0.1196098,-0.0159820},
{-0.0585979,-0.0148191,0.0651020},
{-0.0585979,-0.0148191,-0.0651020},
{-0.0238420,-0.0800479,0.0351680},
{-0.0238420,-0.0800479,-0.0351680},
{-0.0234553,-0.0576924,0.0715330},
{-0.0234553,-0.0576924,-0.0715330},
{-0.0472319,-0.0833276,0.0513790},
{-0.0472319,-0.0833276,-0.0513790},
{-0.0327175,-0.0506481,0.0520240},
{-0.0327175,-0.0506481,-0.0520240},
{-0.0746024,-0.0627219,0.0928110},
{-0.0746024,-0.0627219,-0.0928110},
{-0.0119676,-0.0928080,0.0076900},
{-0.0119676,-0.0928080,-0.0076900},
{-0.0263299,-0.0760728,0.0667080},
{-0.0263299,-0.0760728,-0.0667080},
{-0.0498842,0.0119450,0.1070630},
{-0.0498842,0.0119450,-0.1070630},
{-0.0553874,0.0627474,0.1172390},
{-0.0553874,0.0627474,-0.1172390},
{-0.0786774,0.0073556,0.0741800},
{-0.0786774,0.0073556,-0.0741800},
{-0.0742762,0.0474272,0.0542330},

Biology Open • Supplementary information

{-0.0742762,0.0474272,-0.0542330},
{-0.0634121,0.0117185,0.0618430},
{-0.0634121,0.0117185,-0.0618430},
{-0.0813084,0.0007821,0.0508130},
{-0.0813084,0.0007821,-0.0508130},
{-0.0496423,-0.0444458,0.0983920},
{-0.0496423,-0.0444458,-0.0983920},
{-0.0480005,-0.0338999,0.0625080},
{-0.0480005,-0.0338999,-0.0625080},
{-0.0428000,-0.0273188,0.0973490},
{-0.0428000,-0.0273188,-0.0973490},
{-0.0548383,-0.0518766,0.0624050},
{-0.0548383,-0.0518766,-0.0624050},
{-0.0696448,-0.1052827,0.0436200},
{-0.0696448,-0.1052827,-0.0436200},
{-0.0739608,-0.0803177,0.0785950},
{-0.0739608,-0.0803177,-0.0785950},
{-0.0586066,-0.0884764,0.0653190},
{-0.0586066,-0.0884764,-0.0653190},
{-0.1017003,0.0085215,0.0624150},
{-0.1017003,0.0085215,-0.0624150},
{-0.0126252,0.0252852,0.1292530},
{-0.0126252,0.0252852,-0.1292530},
{-0.0030963,0.0150156,0.1262640},
{-0.0030963,0.0150156,-0.1262640},
{-0.0621797,0.0048743,0.0605920},
{-0.0621797,0.0048743,-0.0605920},
{-0.0870082,-0.0684280,0.0489900},
{-0.0870082,-0.0684280,-0.0489900},
{-0.0916211,-0.0478344,0.0575290},
{-0.0916211,-0.0478344,-0.0575290},
{-0.0428573,-0.0467026,0.0985250},
{-0.0428573,-0.0467026,-0.0985250},
{-0.0483869,-0.0455474,0.0848580},
{-0.0483869,-0.0455474,-0.0848580},
{-0.0203929,-0.0681811,0.0426070},
{-0.0203929,-0.0681811,-0.0426070},
{-0.0271973,-0.0807786,0.0491650},
{-0.0271973,-0.0807786,-0.0491650},
{-0.0325935,-0.1026813,0.0148280},
{-0.0325935,-0.1026813,-0.0148280},
{-0.0273900,-0.1059173,0.0048750},
{-0.0273900,-0.1059173,-0.0048750},
{-0.0015971,-0.0778770,0.0065530},
{-0.0015971,-0.0778770,-0.0065530},
{-0.1034983,-0.0140026,0.0521800},

```
	{-0.1034983,-0.0140026,-0.0521800},
	{-0.1141160,0.0077734,0.0497600},
	{-0.1141160,0.0077734,-0.0497600},
	{-0.1123797,0.0244808,0.0350160},
	{-0.1123797,0.0244808,-0.0350160},
	{-0.0984470,-0.0648210,0.0457100},
	{-0.0984470,-0.0648210,-0.0457100},
	{-0.0814332,-0.0704479,0.0806410},
	{-0.0814332,-0.0704479,-0.0806410},
	{-0.0540218,-0.0805070,0.0547320},
	{-0.0540218,-0.0805070,-0.0547320},
	{-0.0674356,-0.0763171,0.0651080},
	{-0.0674356,-0.0763171,-0.0651080},
	{-0.0602364,-0.0751827,0.0490600},
	{-0.0602364,-0.0751827,-0.0490600},
	{-0.0604272,-0.0801096,0.0617610},
	{-0.0604272,-0.0801096,-0.0617610},
	{-0.0655251,0.0632155,0.1093750},
	{-0.0655251,0.0632155,-0.1093750},
	{-0.0665046,0.0014289,0.0572170},
	{-0.0665046,0.0014289,-0.0572170},
	{-0.1053786,0.0347803,0.0071580},
	{-0.1053786,0.0347803,-0.0071580},
	{-0.1321649,-0.0069725,0.0058340},
	{-0.1321649,-0.0069725,-0.0058340},
	{-0.1232735,-0.0434903,0.0301110},
	{-0.1232735,-0.0434903,-0.0301110},
	{-0.0497024,0.0261862,0.0084640},
	{-0.0497024,0.0261862,-0.0084640},
	{-0.0887827,0.0446048,0.0327030},
	{-0.0887827,0.0446048,-0.0327030},
	{-0.1153998,-0.0126010,0.0290730},
	{-0.1153998,-0.0126010,-0.0290730},
	{-0.0899939,0.0093882,0.0208440},
	{-0.0899939,0.0093882,-0.0208440},
	{-0.1312654,-0.0574898,0.0094690},
	{-0.1312654,-0.0574898,-0.0094690},
	{-0.0996029,0.0042806,0.0105620},
	{-0.0996029,0.0042806,-0.0105620},
	{-0.1226177,-0.0324069,0.0331630},
	{-0.1226177,-0.0324069,-0.0331630},
	{-0.1097808,-0.0051619,0.0102520},
	{-0.1097808,-0.0051619,-0.0102520}
	};
Points1 = {
	{0.0000000,0.0000000,0.1332421},
```

Biology Open • Supplementary information

{-0.0000008,-0.0978063,0.0194018},
{-0.0334116,-0.0759491,0.0927219},
{0.0000000,0.0000000,-0.1332421},
{-0.0000008,-0.0978063,-0.0194018},
{-0.0334116,-0.0759491,-0.0927219},
{0.0000000,0.0000000,0.0000000},
{-0.0912291,-0.0319400,0.0451337},
{-0.0912291,-0.0319400,-0.0451337},
{-0.0864709,-0.1074158,0.0658982},
{-0.0864709,-0.1074158,-0.0658982},
{-0.0593653,0.0449209,0.0971779},
{-0.0593653,0.0449209,-0.0971779},
{-0.0963112,-0.0080019,0.0400156},
{-0.0963112,-0.0080019,-0.0400156},
{-0.0874276,0.0168258,0.0531313},
{-0.0874276,0.0168258,-0.0531313},
{-0.0366496,0.0344791,0.1379698},
{-0.0366496,0.0344791,-0.1379698},
{-0.0238839,0.0348272,0.1424991},
{-0.0238839,0.0348272,-0.1424991},
{-0.0645658,-0.0433377,0.0731577},
{-0.0645658,-0.0433377,-0.0731577},
{-0.0806689,-0.0792226,0.0676743},
{-0.0806689,-0.0792226,-0.0676743},
{-0.0120215,-0.0424656,0.1000335},
{-0.0120215,-0.0424656,-0.1000335},
{-0.0558726,-0.1352730,0.0363684},
{-0.0558726,-0.1352730,-0.0363684},
{-0.0755837,-0.1254000,0.0693022},
{-0.0755837,-0.1254000,-0.0693022},
{-0.0314344,-0.1296351,0.0202902},
{-0.0314344,-0.1296351,-0.0202902},
{-0.0502385,-0.0308863,0.0706880},
{-0.0502385,-0.0308863,-0.0706880},
{-0.0159728,-0.0891791,0.0366486},
{-0.0159728,-0.0891791,-0.0366486},
{-0.0168089,-0.0633898,0.0785723},
{-0.0168089,-0.0633898,-0.0785723},
{-0.0312073,-0.0863234,0.0602862},
{-0.0312073,-0.0863234,-0.0602862},
{-0.0261244,-0.0636352,0.0611464},
{-0.0261244,-0.0636352,-0.0611464},
{-0.0529726,-0.0712372,0.0975225},
{-0.0529726,-0.0712372,-0.0975225},
{-0.0076419,-0.1085062,0.0083302},
{-0.0076419,-0.1085062,-0.0083302},

Biology Open • Supplementary information

{-0.0178614,-0.0763821,0.0715832},
{-0.0178614,-0.0763821,-0.0715832},
{-0.0386914,-0.0010728,0.1134807},
{-0.0386914,-0.0010728,-0.1134807},
{-0.0412967,0.0428782,0.1258768},
{-0.0412967,0.0428782,-0.1258768},
{-0.0655987,-0.0154000,0.0741411},
{-0.0655987,-0.0154000,-0.0741411},
{-0.0628225,0.0151277,0.0534290},
{-0.0628225,0.0151277,-0.0534290},
{-0.0570413,-0.0110566,0.0601161},
{-0.0570413,-0.0110566,-0.0601161},
{-0.0686667,-0.0249276,0.0499126},
{-0.0686667,-0.0249276,-0.0499126},
{-0.0332565,-0.0545720,0.1008840},
{-0.0332565,-0.0545720,-0.1008840},
{-0.0383185,-0.0469715,0.0720957},
{-0.0383185,-0.0469715,-0.0720957},
{-0.0309385,-0.0377205,0.1012787},
{-0.0309385,-0.0377205,-0.1012787},
{-0.0395879,-0.0631486,0.0738729},
{-0.0395879,-0.0631486,-0.0738729},
{-0.0535218,-0.1108121,0.0566324},
{-0.0535218,-0.1108121,-0.0566324},
{-0.0521301,-0.0870970,0.0887070},
{-0.0521301,-0.0870970,-0.0887070},
{-0.0397475,-0.0947532,0.0790837},
{-0.0397475,-0.0947532,-0.0790837},
{-0.0812831,-0.0186390,0.0614779},
{-0.0812831,-0.0186390,-0.0614779},
{-0.0079531,0.0205263,0.1428622},
{-0.0079531,0.0205263,-0.1428622},
{-0.0014291,0.0129421,0.1411046},
{-0.0014291,0.0129421,-0.1411046},
{-0.0567609,-0.0165836,0.0586430},
{-0.0567609,-0.0165836,-0.0586430},
{-0.0678573,-0.0821086,0.0672240},
{-0.0678573,-0.0821086,-0.0672240},
{-0.0720064,-0.0656874,0.0702901},
{-0.0720064,-0.0656874,-0.0702901},
{-0.0274433,-0.0566022,0.1003966},
{-0.0274433,-0.0566022,-0.1003966},
{-0.0336006,-0.0557893,0.0902259},
{-0.0336006,-0.0557893,-0.0902259},
{-0.0141795,-0.0789703,0.0444031},
{-0.0141795,-0.0789703,-0.0444031},

Biology Open • Supplementary information

{-0.0180590,-0.0861726,0.0526112},
{-0.0180590,-0.0861726,-0.0526112},
{-0.0225999,-0.1146328,0.0184857},
{-0.0225999,-0.1146328,-0.0184857},
{-0.0167761,-0.1182447,0.0072213},
{-0.0167761,-0.1182447,-0.0072213},
{-0.0019420,-0.0964225,0.0067012},
{-0.0019420,-0.0964225,-0.0067012},
{-0.0815903,-0.0382171,0.0560905},
{-0.0815903,-0.0382171,-0.0560905},
{-0.0898203,-0.0210697,0.0475520},
{-0.0898203,-0.0210697,-0.0475520},
{-0.0881824,-0.0126578,0.0305732},
{-0.0881824,-0.0126578,-0.0305732},
{-0.0795436,-0.0812653,0.0662460},
{-0.0795436,-0.0812653,-0.0662460},
{-0.0590484,-0.0795893,0.0902848},
{-0.0590484,-0.0795893,-0.0902848},
{-0.0353358,-0.0830233,0.0654653},
{-0.0353358,-0.0830233,-0.0654653},
{-0.0469616,-0.0848991,0.0789605},
{-0.0469616,-0.0848991,-0.0789605},
{-0.0420012,-0.0826668,0.0617058},
{-0.0420012,-0.0826668,-0.0617058},
{-0.0406875,-0.0878237,0.0761681},
{-0.0406875,-0.0878237,-0.0761681},
{-0.0511473,0.0392327,0.1169338},
{-0.0511473,0.0392327,-0.1169338},
{-0.0603569,-0.0207512,0.0544735},
{-0.0603569,-0.0207512,-0.0544735},
{-0.0834788,-0.0065606,0.0061441},
{-0.0834788,-0.0065606,-0.0061441},
{-0.1020256,-0.0414178,0.0048891},
{-0.1020256,-0.0414178,-0.0048891},
{-0.1010170,-0.0717053,0.0229517},
{-0.1010170,-0.0717053,-0.0229517},
{-0.0455382,-0.0074961,0.0083509},
{-0.0455382,-0.0074961,-0.0083509},
{-0.0730399,0.0067307,0.0302592},
{-0.0730399,0.0067307,-0.0302592},
{-0.0904854,-0.0431133,0.0276642},
{-0.0904854,-0.0431133,-0.0276642},
{-0.0729581,-0.0249318,0.0195826},
{-0.0729581,-0.0249318,-0.0195826},
{-0.1076768,-0.0839008,0.0071734},
{-0.1076768,-0.0839008,-0.0071734},

Biology Open • Supplementary information

```
                    {-0.0776902,-0.0320147,0.0098262},
                    {-0.0776902,-0.0320147,-0.0098262},
                    {-0.0986706,-0.0610156,0.0292201},
                    {-0.0986706,-0.0610156,-0.0292201},
                    {-0.0839041,-0.0406218,0.0094151},
                    {-0.0839041,-0.0406218,-0.0094151}
                    };
            BoundingBoxOnOff = Off;
        };
        AnyFunTransform3DIdentity ScaleFunction = {
            PreTransforms = {&.RBFTransform};
};
};

};
```

Biology Open • Supplementary information

ScalingFunctionTLEMLucyFemur_2014030

```
AnyFolder ScalingFunctionTLEMLucyFemur = {
  AnyFolder Right = {
        AnyFolder Thigh = {
        AnyFunTransform3DRBF RBFTransform = {
              RBFDef.Type = RBF_ThinPlate;
              PolynomDegree = 1;
              Points0 = {
                    {0.0000000,0.0000000,0.0000000},
                    {-0.0000000,-0.3616821,0.0000000},
                    {-0.0097563,-0.3678799,0.0012967},
                    {-0.0000000,-0.3660632,0.0408203},
                    {-0.0000000,-0.3573010,-0.0408203},
                    {0.0161460,-0.0072838,0.0601290},
                    {0.0220217,-0.0203698,0.0463848},
                    {0.0123977,-0.0241932,0.0668573},
                    {0.0006898,0.0018121,0.0538181},
                    {0.0122809,-0.0068668,0.0414535},
                    {-0.0058991,-0.0138188,0.0648412},
                    {0.0211469,-0.0380855,0.0538111},
                    {0.0172133,-0.0317342,0.0328381},
                    {0.0177311,-0.0131946,0.0256176},
                    {-0.0110079,-0.0308867,0.0632370},
                    {0.0039435,-0.0395977,0.0638271},
                    {-0.0041683,-0.0187472,0.0413214},
                    {-0.0177999,-0.0021535,0.0496084},
                    {0.0001603,-0.0053126,0.0279593},
                    {-0.0211600,-0.0216243,0.0497147},
                    {0.0096163,-0.0568663,0.0545719},
                    {0.0162112,-0.0515013,0.0395230},
                    {0.0093533,-0.0433265,0.0187387},
                    {0.0119398,-0.0238469,0.0118403},
                    {0.0210707,-0.0048848,0.0065878},
                    {0.0125013,0.0064094,0.0177702},
                    {-0.0191523,-0.0356053,0.0382429},
                    {-0.0115821,-0.0474937,0.0537648},
                    {-0.0100073,-0.0236371,0.0267322},
                    {-0.0078925,0.0107098,0.0182833},
                    {-0.0134217,-0.0055356,0.0182000},
                    {-0.0090061,-0.0665700,0.0475484},
                    {0.0071095,-0.0761976,0.0520035},
                    {0.0182119,-0.0704149,0.0386546},
                    {0.0073764,-0.0609481,0.0221006},
                    {-0.0122984,-0.0439585,0.0198428},
                    {-0.0032253,-0.0306792,0.0110995},
```

Biology Open • Supplementary information

{-0.0012975,-0.0198451,-0.0052592},
{0.0140405,-0.0161125,-0.0071649},
{0.0189222,-0.0014898,-0.0127226},
{0.0181642,0.0132812,-0.0011050},
{0.0045119,0.0200001,0.0091189},
{-0.0176761,-0.0541983,0.0348587},
{-0.0136548,-0.0142015,0.0054311},
{-0.0135510,0.0182940,0.0017175},
{-0.0223865,0.0023399,0.0028018},
{-0.0086455,-0.0738247,0.0283843},
{-0.0050356,-0.0877749,0.0442195},
{0.0160135,-0.0919319,0.0465120},
{0.0128290,-0.0785810,0.0232431},
{-0.0168328,-0.0616327,0.0155917},
{-0.0172369,-0.0094567,-0.0119502},
{0.0002090,-0.0093641,-0.0209779},
{0.0064827,0.0081538,-0.0198453},
{0.0010777,0.0210422,-0.0084721},
{-0.0129485,0.0085451,-0.0162212},
{-0.0002322,-0.0924033,0.0245644},
{-0.0028175,-0.1052263,0.0418294},
{0.0172322,-0.1111444,0.0445760},
{0.0203259,-0.0957409,0.0271449},
{0.0057102,-0.1088933,0.0195113},
{-0.0010852,-0.1230776,0.0363132},
{0.0182727,-0.1303312,0.0422884},
{0.0242891,-0.1161703,0.0265054},
{0.0094531,-0.1268584,0.0162294},
{0.0014244,-0.1413293,0.0331653},
{0.0207271,-0.1496427,0.0394157},
{0.0267562,-0.1360142,0.0236798},
{0.0113839,-0.1461651,0.0136024},
{0.0030845,-0.1595094,0.0297156},
{0.0209528,-0.1691386,0.0369775},
{0.0283958,-0.1540209,0.0213795},
{0.0129499,-0.1649012,0.0113589},
{0.0016490,-0.1783108,0.0216287},
{0.0147105,-0.1880678,0.0328614},
{0.0298491,-0.1859865,0.0248551},
{0.0286645,-0.1714377,0.0169488},
{0.0165897,-0.1861006,0.0082595},
{0.0014000,-0.1973476,0.0183740},
{0.0114301,-0.2109792,0.0286157},
{0.0267422,-0.2037622,0.0296951},
{0.0288630,-0.2018335,0.0130127},
{0.0145471,-0.2083505,0.0062833},

{0.0011300,-0.2198071,0.0155602},
{0.0126610,-0.2321235,0.0275354},
{0.0275647,-0.2219268,0.0265185},
{0.0286645,-0.2230253,0.0098701},
{0.0130434,-0.2304822,0.0039177},
{0.0022984,-0.2409585,0.0149650},
{0.0101315,-0.2534740,0.0252125},
{0.0279485,-0.2428555,0.0234276},
{0.0276684,-0.2421559,0.0058691},
{0.0101250,-0.2529527,0.0018983},
{0.0021376,-0.2667831,0.0107073},
{0.0114309,-0.2734905,0.0249960},
{0.0273923,-0.2634953,0.0216324},
{0.0278429,-0.2592546,0.0037692},
{0.0159101,-0.2712985,-0.0033988},
{0.0036708,-0.2863280,0.0004644},
{0.0023115,-0.2884756,0.0187554},
{0.0231385,-0.2879051,0.0224853},
{0.0304009,-0.2799973,0.0083549},
{0.0238189,-0.2917246,-0.0052462},
{0.0064904,-0.3027687,-0.0070587},
{-0.0003492,-0.3064690,0.0093014},
{0.0122179,-0.3045269,0.0243511},
{0.0301837,-0.3030127,0.0114462},
{0.0234182,-0.3107336,-0.0085198},
{0.0046893,-0.3214995,-0.0123881},
{-0.0022352,-0.3264905,0.0043340},
{0.0016841,-0.3202756,0.0217839},
{0.0229722,-0.3196887,0.0215083},
{0.0292438,-0.3228042,0.0051430},
{0.0221641,-0.3296190,-0.0132361},
{0.0100459,-0.3417597,-0.0244560},
{-0.0053009,-0.3375737,-0.0137705},
{-0.0064432,-0.3487307,0.0028676},
{-0.0059346,-0.3407801,0.0202938},
{0.0105130,-0.3365976,0.0264175},
{0.0282695,-0.3386249,0.0180307},
{0.0270402,-0.3431844,-0.0001487},
{0.0257040,-0.3506950,-0.0181851},
{0.0178948,-0.3636114,-0.0302838},
{0.0043149,-0.3544109,-0.0381949},
{-0.0095210,-0.3461482,-0.0295031},
{-0.0164526,-0.3542895,-0.0126666},
{-0.0060896,-0.3690127,-0.0033061},
{-0.0176523,-0.3612686,0.0110109},
{-0.0153310,-0.3546943,0.0299634},

{0.0021329,-0.3508021,0.0350763},
{0.0210690,-0.3548628,0.0279835},
{0.0356594,-0.3558162,0.0159356},
{0.0275718,-0.3617450,-0.0018607},
{0.0298503,-0.3741530,-0.0163721},
{0.0175320,-0.3855300,-0.0277515},
{0.0025604,-0.3740099,-0.0397137},
{-0.0136727,-0.3624016,-0.0402102},
{-0.0277647,-0.3567472,-0.0284947},
{-0.0275446,-0.3699630,-0.0120829},
{-0.0175846,-0.3859355,-0.0177922},
{0.0001572,-0.3856873,-0.0118050},
{0.0030262,-0.3834856,0.0075553},
{-0.0144114,-0.3810887,0.0140304},
{-0.0247706,-0.3701239,0.0285134},
{-0.0044804,-0.3677201,0.0404632},
{0.0133183,-0.3712274,0.0334208},
{0.0324861,-0.3723417,0.0245668},
{0.0271451,-0.3766027,0.0072441},
{0.0167985,-0.3822816,-0.0065349},
{-0.0001454,-0.3910823,-0.0303010},
{-0.0144998,-0.3834154,-0.0384137},
{-0.0301321,-0.3750841,-0.0321336},
{0.0196491,-0.3864804,0.0210853},
{0.0015726,-0.3888034,0.0276569},
{-0.0146652,-0.3847848,0.0336913},
{-0.0014588,-0.0619951,0.0219778},
{-0.0118435,-0.0550626,0.0139639},
{0.0129812,-0.0349966,0.0673128},
{-0.0162907,-0.0105666,0.0597739},
{-0.0012885,-0.0520816,0.0562989},
{-0.0090599,-0.0631298,0.0172224},
{-0.0000222,-0.0771663,0.0231897},
{-0.0068148,0.0023885,-0.0160086},
{-0.0180544,-0.0425409,0.0446585},
{0.0020525,-0.0249904,0.0691549},
{-0.0157097,-0.0662315,0.0238393},
{-0.0010518,-0.0510686,0.0199353},
{-0.0208874,-0.0152865,0.0500034},
{0.0168316,-0.0280857,0.0666680},
{-0.0187392,-0.0282333,0.0403282},
{0.0191622,-0.0351633,0.0622707},
{0.0001216,-0.0706289,0.0524049},
{-0.0169862,-0.0399062,0.0311827},
{-0.0185737,-0.0533066,0.0151224},
{-0.0219058,-0.0528723,0.0222263},

Biology Open • Supplementary information

{-0.0028271,-0.0391620,0.0148452},
{0.0117900,-0.0553425,0.0235348},
{0.0219835,-0.0106400,0.0396338},
{-0.0025366,-0.0401046,0.0625273},
{0.0141017,-0.0018767,0.0198108},
{-0.0078770,-0.0473929,0.0173542},
{-0.0065351,-0.0766431,0.0470258},
{0.0065585,0.0145593,0.0161638},
{-0.0118588,0.0172875,-0.0085064},
{-0.0074087,-0.0627500,0.0506700},
{0.0071940,-0.0780068,0.0213362},
{0.0126736,-0.0708176,0.0244108},
{0.0074694,-0.0436450,0.0604196},
{0.0154186,-0.0139471,0.0064312},
{-0.0053535,-0.0213210,0.0040094},
{-0.0088733,0.0002018,0.0205402},
{-0.0112471,-0.0542608,0.0500266},
{-0.0061625,-0.0248673,0.0653244},
{0.0093695,-0.0065826,0.0614157},
{0.0138015,-0.0467322,0.0284333},
{-0.0020999,-0.0186885,-0.0116392},
{-0.0185640,-0.0225591,0.0566779},
{0.0175798,0.0124360,-0.0068467},
{-0.0039962,0.0217127,0.0053055},
{0.0014975,-0.0784591,0.0514056},
{-0.0172997,-0.0386971,0.0525252},
{0.0196540,-0.0268271,0.0416806},
{0.0135296,-0.0406504,0.0236882},
{0.0182471,0.0095959,0.0087906},
{0.0218667,0.0016804,0.0058793},
{0.0224975,-0.0022652,-0.0022994},
{0.0166750,-0.0191391,0.0236436},
{0.0119785,-0.0328723,0.0195376},
{0.0187631,-0.0133469,0.0308741},
{-0.0187543,-0.0461463,0.0257491},
{0.0039834,-0.0373928,0.0683089},
{0.0157029,-0.0423169,0.0385226},
{0.0164003,-0.0087069,0.0131181},
{0.0008253,0.0033370,-0.0212576},
{0.0101707,-0.0009724,-0.0205688},
{0.0102948,-0.0642683,0.0524532},
{0.0152217,-0.0557944,0.0508978},
{0.0172973,-0.0565707,0.0419468},
{0.0167723,-0.0635334,0.0369834},
{-0.0047397,-0.0695325,0.0241398},
{-0.0152913,0.0035778,0.0161461},

Biology Open • Supplementary information

{0.0091697,0.0009340,0.0575160},
{0.0196686,-0.0089533,0.0500006},
{0.0183998,-0.0212943,0.0622968},
{-0.0114340,-0.0316810,0.0250940},
{-0.0206442,-0.0092599,-0.0028425},
{0.0166868,-0.0784295,0.0276925},
{-0.0069678,-0.0054521,-0.0207011},
{0.0049490,0.0151109,-0.0160093},
{-0.0084025,-0.0318937,0.0162788},
{-0.0173679,0.0095594,-0.0108509},
{-0.0218912,0.0063457,-0.0020698},
{-0.0106547,-0.0668750,0.0397749},
{0.0136098,-0.0170666,-0.0018890},
{0.0141599,-0.0441405,0.0587594},
{0.0093255,-0.0183218,-0.0083008},
{0.0106367,-0.0128276,-0.0154269},
{0.0037350,-0.0549365,0.0209786},
{0.0087662,-0.0472902,0.0195496},
{0.0016839,-0.0708213,0.0221325},
{-0.0202977,-0.0530540,0.0301270},
{-0.0068584,0.0021781,0.0530002},
{-0.0144817,-0.0297218,0.0605833},
{0.0197015,-0.0165217,0.0560654},
{0.0156609,-0.0705363,0.0477736},
{0.0207876,-0.0221433,0.0330733},
{0.0092324,-0.0300767,0.0713160},
{-0.0203392,-0.0034488,0.0097332},
{0.0156978,-0.0143438,0.0629053},
{0.0143267,-0.0563519,0.0317489},
{-0.0161685,-0.0465391,0.0188591},
{-0.0196397,-0.0309875,0.0546496},
{0.0154804,-0.0380422,0.0305433},
{-0.0077774,-0.0351303,0.0637765},
{0.0063980,-0.0086419,-0.0201654},
{0.0030146,-0.0151094,-0.0165974},
{-0.0210080,-0.0096030,0.0507855},
{-0.0105684,-0.0411377,0.0586146},
{0.0180619,-0.0498960,0.0469835},
{-0.0192318,0.0116108,0.0024070},
{-0.0165651,0.0083622,0.0126870},
{-0.0014333,-0.0627138,0.0534746},
{0.0145127,-0.0080178,0.0353667},
{0.0148374,-0.0012228,0.0566181},
{0.0153834,-0.0062427,0.0444727},
{-0.0155164,-0.0132103,-0.0096398},
{0.0068685,-0.0221022,0.0011547},

Biology Open • Supplementary information

{0.0003332,-0.0458500,0.0183716},
{0.0205582,0.0040956,-0.0082969},
{0.0175752,0.0009340,-0.0148407},
{0.0142357,-0.0063623,-0.0169332},
{-0.0147285,0.0023412,-0.0164678},
{-0.0158781,-0.0064451,-0.0151346},
{0.0175951,0.0133456,0.0037559},
{0.0117660,0.0189899,-0.0030690},
{0.0096040,0.0151827,-0.0137450},
{0.0096565,-0.0215194,0.0662734},
{-0.0006793,-0.0074494,0.0631218},
{0.0007819,-0.0004519,0.0600781},
{0.0065377,-0.0125433,0.0654199},
{0.0090461,0.0073096,0.0188711},
{0.0151418,-0.0168302,0.0146465},
{0.0025166,-0.0231863,0.0688894},
{-0.0097891,-0.0152077,0.0253086},
{-0.0109149,-0.0177935,0.0119825},
{-0.0072079,-0.0099373,0.0272725},
{-0.0183727,-0.0467429,0.0356276},
{-0.0126871,-0.0584555,0.0428141},
{-0.0057054,-0.0273042,0.0103554},
{0.0215142,-0.0159693,0.0474531},
{0.0128492,-0.0070016,0.0287645},
{0.0052134,-0.0017047,0.0247463},
{-0.0024284,-0.0707498,0.0513049},
{-0.0036140,0.0220593,-0.0040484},
{-0.0002405,-0.0121253,0.0390050},
{-0.0119811,-0.0156737,0.0015445},
{0.0142082,-0.0279792,0.0246553},
{0.0122244,-0.0262013,0.0169829},
{-0.0146264,-0.0038855,0.0549384},
{0.0207450,-0.0325771,0.0496412},
{-0.0155040,-0.0497560,0.0437087},
{-0.0081119,-0.0756450,0.0387365},
{0.0039684,-0.0062047,0.0313900},
{-0.0023726,-0.0038892,-0.0220521},
{-0.0136977,-0.0339839,0.0602619},
{-0.0035399,0.0089081,-0.0188894},
{-0.0049820,0.0176571,-0.0132254},
{0.0193902,-0.0803191,0.0360429},
{0.0040648,-0.0697109,0.0218205},
{0.0170470,-0.0818610,0.0456892},
{0.0104175,0.0188635,0.0064965},
{0.0135206,0.0092549,0.0158079},
{0.0023933,0.0160498,-0.0157404},

Biology Open • Supplementary information

{0.0164753,-0.0118197,-0.0096995},
{0.0202584,-0.0066858,-0.0082383},
{-0.0098401,-0.0226001,0.0314028},
{-0.0130937,-0.0309622,0.0320764},
{-0.0201510,-0.0613297,0.0222264},
{-0.0010389,0.0036055,0.0209003},
{0.0042025,0.0073392,0.0199393},
{-0.0021630,-0.0038531,0.0251898},
{-0.0007845,-0.0293574,0.0675788},
{0.0070027,-0.0406327,0.0624565},
{-0.0124363,-0.0183001,0.0627966},
{-0.0120485,-0.0256613,0.0628493},
{0.0101200,0.0197695,0.0039334},
{0.0167636,0.0148266,0.0014155},
{-0.0074981,-0.0106930,0.0632797},
{0.0142533,-0.0503445,0.0351954},
{-0.0146107,-0.0460953,0.0506120},
{-0.0161117,-0.0471301,0.0178900},
{0.0112149,-0.0792793,0.0501460},
{-0.0099060,-0.0230794,0.0186951},
{-0.0114543,-0.0153049,0.0183675},
{-0.0042882,0.0161879,0.0151423},
{0.0014582,-0.0638572,0.0541080},
{0.0048554,-0.0582603,0.0553815},
{-0.0017077,-0.0572302,0.0546635},
{0.0166090,-0.0697169,0.0323038},
{-0.0208812,-0.0164273,0.0474354},
{0.0130454,-0.0241864,0.0178294},
{0.0053123,-0.0483770,0.0582251},
{0.0112225,-0.0361429,0.0673300},
{0.0085729,-0.0771256,0.0216100},
{-0.0070783,-0.0488033,0.0560869},
{0.0182083,-0.0411024,0.0454236},
{-0.0119590,-0.0403809,0.0213546},
{0.0233409,-0.0185535,0.0366343},
{-0.0046347,-0.0571211,0.0208243},
{0.0183166,-0.0006966,0.0140116},
{0.0061913,0.0053512,0.0201003},
{-0.0002364,-0.0310828,0.0680351},
{-0.0170773,0.0139270,-0.0054987},
{0.0110805,0.0156034,0.0117010},
{-0.0124628,0.0164667,0.0094550},
{-0.0209014,-0.0073407,-0.0051969},
{-0.0210131,0.0010693,-0.0086891},
{-0.0041596,-0.0172332,0.0663041},
{-0.0216595,-0.0322172,0.0451540},

Biology Open • Supplementary information

{0.0051908,-0.0426014,0.0613013},
{0.0150003,-0.0416136,0.0606257},
{-0.0135090,-0.0674234,0.0236425},
{0.0146416,0.0073691,0.0160052},
{0.0195615,-0.0112412,-0.0010333},
{-0.0198115,-0.0098244,0.0044735},
{-0.0157054,-0.0521236,0.0395039},
{-0.0081381,-0.0173108,-0.0046152},
{-0.0218324,-0.0262661,0.0471115},
{0.0051446,-0.0707257,0.0530555},
{0.0179359,-0.0471493,0.0528134},
{-0.0005591,-0.0317983,0.0113632},
{0.0071707,-0.0305943,0.0121186},
{0.0206239,-0.0061367,0.0066642},
{0.0187057,-0.0745868,0.0420207},
{-0.0095784,-0.0163113,0.0259524},
{-0.0012181,0.0127661,0.0186855},
{0.0059420,-0.0630691,0.0220087},
{0.0069408,-0.0573878,0.0552614},
{0.0150782,-0.0132884,-0.0101793},
{0.0005533,-0.0078574,0.0633775},
{-0.0088284,-0.0146399,-0.0147837},
{0.0167444,-0.0651105,0.0470347},
{0.0082666,-0.0181741,-0.0095133},
{-0.0175162,-0.0637444,0.0177069},
{0.0153795,-0.0360767,0.0287608},
{0.0162363,-0.0018864,0.0548412},
{0.0052731,-0.0075919,0.0392990},
{-0.0022288,-0.0556644,0.0549909},
{-0.0156238,-0.0550038,0.0377688},
{-0.0204137,-0.0100550,0.0536429},
{-0.0093683,0.0122429,-0.0162013},
{-0.0202927,0.0049166,0.0083887},
{0.0074706,-0.0627945,0.0222133},
{0.0035034,-0.0376103,0.0672421},
{-0.0190752,-0.0194129,0.0555530},
{0.0061758,-0.0074961,-0.0207741},
{0.0192011,-0.0273063,0.0588233},
{0.0130796,-0.0134383,0.0638611},
{0.0120421,0.0019566,-0.0194140},
{0.0154152,-0.0065294,0.0179581},
{-0.0157335,0.0101219,0.0123981},
{0.0110376,-0.0488274,0.0567911},
{-0.0055170,-0.0343676,0.0647140},
{0.0216204,-0.0273988,0.0512041},
{0.0161331,-0.0135875,-0.0075501},

{-0.0184643,-0.0455633,0.0360087},
{-0.0091978,-0.0682413,0.0460847},
{0.0034304,-0.0471677,0.0588866},
{-0.0048025,-0.0384316,0.0149400},
{0.0118505,0.0109735,-0.0159161},
{-0.0027036,-0.0204189,-0.0015094},
{-0.0117607,-0.0692759,0.0292171},
{-0.0107080,-0.0033455,-0.0190487},
{-0.0042548,0.0214947,-0.0056846},
{-0.0097315,-0.0208529,0.0131997},
{-0.0178958,-0.0627218,0.0171551},
{-0.0068692,-0.0688818,0.0233316},
{0.0012083,0.0120695,-0.0186179},
{0.0215073,-0.0236027,0.0503069},
{-0.0060262,-0.0534931,0.0189523},
{0.0179951,-0.0336785,0.0397267},
{0.0190509,-0.0284972,0.0336394},
{-0.0169188,-0.0133609,-0.0014203},
{0.0135722,0.0136491,0.0114695},
{-0.0150549,-0.0123230,0.0067722},
{-0.0148411,-0.0311945,0.0355412},
{0.0185490,-0.0794042,0.0431266},
{0.0193568,-0.0087326,0.0575704},
{0.0201026,-0.0144353,0.0531586},
{0.0025583,-0.0248211,0.0045714},
{0.0132689,-0.0214866,0.0652899},
{-0.0028669,-0.0099311,0.0309271},
{-0.0219487,-0.0054893,-0.0021853},
{0.0026180,-0.0203806,-0.0040620},
{-0.0116230,-0.0668518,0.0370152},
{-0.0133007,-0.0264159,0.0619176},
{0.0186167,0.0050491,-0.0120059},
{-0.0191359,-0.0591690,0.0288149},
{-0.0178121,-0.0041834,0.0141448},
{0.0123077,-0.0063648,0.0279743},
{-0.0157729,-0.0374010,0.0315335},
{0.0149328,-0.0659100,0.0290095},
{0.0006205,0.0021977,-0.0214613},
{0.0170484,-0.0715687,0.0325980},
{0.0068499,0.0061192,0.0196608},
{-0.0167075,0.0020024,0.0150382},
{0.0059801,-0.0393117,0.0167489},
{-0.0072506,-0.0171067,0.0338751},
{0.0202363,-0.0251596,0.0420997},
{-0.0199311,-0.0601255,0.0171522},
{0.0216641,0.0053410,-0.0027841},

{-0.0197665,-0.0606125,0.0252972},
{0.0129986,-0.0214528,0.0098002},
{0.0201151,-0.0100929,0.0460501},
{0.0104137,0.0017765,-0.0203073},
{0.0166557,-0.0064123,0.0140197},
{-0.0124120,-0.0596549,0.0422637},
{0.0014885,0.0202590,0.0096781},
{0.0028720,0.0020207,-0.0219044},
{0.0376095,-0.3675427,0.0214537},
{0.0049949,0.0206417,-0.0079017},
{-0.0191723,-0.0407590,0.0391310},
{-0.0316406,-0.3638215,-0.0274841},
{0.0021392,0.0226920,0.0000985},
{0.0168435,-0.0229382,0.0264788},
{-0.0157304,-0.0005437,-0.0161456},
{0.0203722,-0.0299750,0.0562762},
{-0.0171077,0.0134146,0.0059615},
{0.0175519,-0.0370653,0.0413374},
{0.0154657,-0.0405127,0.0348980},
{-0.0215172,-0.0323502,0.0481925},
{0.0177998,-0.0515776,0.0444709},
{-0.0183804,-0.0448316,0.0275139},
{0.0033607,-0.0502028,0.0575894},
{0.0010325,-0.0479377,0.0191141},
{0.0224852,-0.0181821,0.0451780},
{0.0084089,-0.0720538,0.0521031},
{-0.0070880,-0.0772912,0.0288023},
{-0.0223712,-0.0028990,0.0024047},
{0.0173136,-0.0120017,0.0204612},
{0.0137772,-0.0623537,0.0276621},
{-0.0100390,-0.0231180,0.0292222},
{-0.0196395,-0.3730207,0.0364730},
{-0.0094445,-0.0526688,0.0528111},
{-0.0115309,-0.3679776,-0.0104995},
{0.0129812,-0.0410477,0.0224072},
{0.0117394,0.0154849,-0.0117191},
{-0.0245208,-0.3649412,0.0295111},
{0.0147476,-0.0502966,0.0364899},
{0.0006132,-0.0446771,0.0600524},
{-0.0118123,0.0191139,0.0031977},
{-0.0017185,-0.0163303,0.0664216},
{-0.0085098,-0.0074564,0.0247745},
{0.0197459,-0.0086852,0.0430460},
{0.0187064,-0.0223532,0.0600966},
{-0.0206552,-0.0099712,0.0525024},
{0.0207525,0.0084646,-0.0024846},

{-0.0094915,-0.0325446,0.0184441},
{-0.0014464,-0.0140050,-0.0179819},
{-0.0122725,-0.0573444,0.0463125},
{-0.0106397,0.0177211,0.0094669},
{0.0111981,0.0191236,-0.0041491},
{-0.0162790,-0.0256279,0.0592687},
{-0.0060120,-0.3653959,0.0413411},
{-0.0135007,-0.3703638,-0.0387476},
{-0.0134258,-0.3703142,-0.0379467},
{-0.0133509,-0.3702645,-0.0371458},
{-0.0132760,-0.3702148,-0.0363450},
{-0.0132011,-0.3701651,-0.0355441},
{-0.0131262,-0.3701154,-0.0347432},
{-0.0130513,-0.3700658,-0.0339423},
{-0.0129765,-0.3700161,-0.0331414},
{-0.0129016,-0.3699664,-0.0323405},
{-0.0128267,-0.3699167,-0.0315396},
{-0.0127518,-0.3698670,-0.0307388},
{-0.0126769,-0.3698174,-0.0299379},
{-0.0126020,-0.3697677,-0.0291370},
{-0.0125271,-0.3697180,-0.0283361},
{-0.0124523,-0.3696683,-0.0275352},
{-0.0123774,-0.3696187,-0.0267343},
{-0.0123025,-0.3695690,-0.0259334},
{-0.0122276,-0.3695193,-0.0251325},
{-0.0121527,-0.3694696,-0.0243317},
{-0.0120778,-0.3694199,-0.0235308},
{-0.0120029,-0.3693703,-0.0227299},
{-0.0119280,-0.3693206,-0.0219290},
{-0.0118532,-0.3692709,-0.0211281},
{-0.0117783,-0.3692212,-0.0203272},
{-0.0117034,-0.3691715,-0.0195263},
{-0.0116285,-0.3691219,-0.0187255},
{-0.0115536,-0.3690722,-0.0179246},
{-0.0114787,-0.3690225,-0.0171237},
{-0.0114038,-0.3689728,-0.0163228},
{-0.0113289,-0.3689231,-0.0155219},
{-0.0112541,-0.3688735,-0.0147210},
{-0.0111792,-0.3688238,-0.0139201},
{-0.0111043,-0.3687741,-0.0131192},
{-0.0110294,-0.3687244,-0.0123184},
{-0.0109545,-0.3686748,-0.0115175},
{-0.0108796,-0.3686251,-0.0107166},
{-0.0108047,-0.3685754,-0.0099157},
{-0.0107298,-0.3685257,-0.0091148},
{-0.0106550,-0.3684760,-0.0083139},

Biology Open • Supplementary information

{-0.0105801,-0.3684264,-0.0075130},
{-0.0105052,-0.3683767,-0.0067121},
{-0.0104303,-0.3683270,-0.0059113},
{-0.0103554,-0.3682773,-0.0051104},
{-0.0102805,-0.3682276,-0.0043095},
{-0.0102056,-0.3681780,-0.0035086},
{-0.0101307,-0.3681283,-0.0027077},
{-0.0100559,-0.3680786,-0.0019068},
{-0.0099810,-0.3680289,-0.0011059},
{-0.0099061,-0.3679792,-0.0003051},
{-0.0098312,-0.3679296,0.0004958},
{-0.0097563,-0.3678799,0.0012967},
{-0.0096814,-0.3678302,0.0020976},
{-0.0096065,-0.3677805,0.0028985},
{-0.0095317,-0.3677308,0.0036994},
{-0.0094568,-0.3676812,0.0045003},
{-0.0093819,-0.3676315,0.0053012},
{-0.0093070,-0.3675818,0.0061020},
{-0.0092321,-0.3675321,0.0069029},
{-0.0091572,-0.3674825,0.0077038},
{-0.0090823,-0.3674328,0.0085047},
{-0.0090074,-0.3673831,0.0093056},
{-0.0089326,-0.3673334,0.0101065},
{-0.0088577,-0.3672837,0.0109074},
{-0.0087828,-0.3672341,0.0117082},
{-0.0087079,-0.3671844,0.0125091},
{-0.0086330,-0.3671347,0.0133100},
{-0.0085581,-0.3670850,0.0141109},
{-0.0084832,-0.3670353,0.0149118},
{-0.0084083,-0.3669857,0.0157127},
{-0.0083335,-0.3669360,0.0165136},
{-0.0082586,-0.3668863,0.0173145},
{-0.0081837,-0.3668366,0.0181153},
{-0.0081088,-0.3667869,0.0189162},
{-0.0080339,-0.3667373,0.0197171},
{-0.0079590,-0.3666876,0.0205180},
{-0.0078841,-0.3666379,0.0213189},
{-0.0078092,-0.3665882,0.0221198},
{-0.0077344,-0.3665386,0.0229207},
{-0.0076595,-0.3664889,0.0237215},
{-0.0075846,-0.3664392,0.0245224},
{-0.0075097,-0.3663895,0.0253233},
{-0.0074348,-0.3663398,0.0261242},
{-0.0073599,-0.3662902,0.0269251},
{-0.0072850,-0.3662405,0.0277260},
{-0.0072101,-0.3661908,0.0285269},

```
{-0.0071353,-0.3661411,0.0293278},
{-0.0070604,-0.3660914,0.0301286},
{-0.0069855,-0.3660418,0.0309295},
{-0.0069106,-0.3659921,0.0317304},
{-0.0068357,-0.3659424,0.0325313},
{-0.0067608,-0.3658927,0.0333322},
{-0.0066859,-0.3658430,0.0341331},
{-0.0066111,-0.3657934,0.0349340},
{-0.0065362,-0.3657437,0.0357348},
{-0.0064613,-0.3656940,0.0365357},
{-0.0063864,-0.3656443,0.0373366},
{-0.0063115,-0.3655947,0.0381375},
{-0.0062366,-0.3655450,0.0389384},
{-0.0061617,-0.3654953,0.0397393},
{-0.0060868,-0.3654456,0.0405402}
};
Points1 = {
{0.0000000,0.0000000,0.0000000},
{-0.0000000,-0.4659265,0.0000000},
{-0.0104358,-0.4739106,0.0013870},
{-0.0000000,-0.4715703,0.0436633},
{-0.0000000,-0.4602826,-0.0436633},
{0.0155354,-0.0104706,0.0626348},
{0.0225419,-0.0272318,0.0492964},
{0.0108338,-0.0308878,0.0683295},
{0.0001751,-0.0004817,0.0557542},
{0.0125812,-0.0082821,0.0434500},
{-0.0066324,-0.0169628,0.0708659},
{0.0188680,-0.0468848,0.0564639},
{0.0169048,-0.0407108,0.0362825},
{0.0159677,-0.0175294,0.0274497},
{-0.0113619,-0.0389341,0.0676173},
{0.0037639,-0.0509670,0.0665017},
{-0.0034459,-0.0244052,0.0451047},
{-0.0139876,-0.0052377,0.0525315},
{-0.0000099,-0.0051379,0.0304783},
{-0.0137351,-0.0277925,0.0540005},
{0.0112846,-0.0737648,0.0593216},
{0.0179463,-0.0655317,0.0422610},
{0.0093551,-0.0553344,0.0211909},
{0.0108190,-0.0294694,0.0138244},
{0.0205009,-0.0066860,0.0063191},
{0.0116007,0.0073715,0.0176354},
{-0.0136639,-0.0463590,0.0401869},
{-0.0112249,-0.0602642,0.0570973},
{-0.0103047,-0.0310216,0.0275872},
```

Biology Open • Supplementary information

{-0.0068417,0.0129763,0.0176676},
{-0.0126968,-0.0071390,0.0174160},
{-0.0083098,-0.0852254,0.0495156},
{0.0070319,-0.0977943,0.0575209},
{0.0191106,-0.0916722,0.0407671},
{0.0092750,-0.0789446,0.0232845},
{-0.0131289,-0.0560537,0.0228835},
{-0.0046851,-0.0398441,0.0113067},
{-0.0004956,-0.0241830,-0.0053229},
{0.0142771,-0.0189535,-0.0072272},
{0.0192914,-0.0021788,-0.0123425},
{0.0189154,0.0157860,-0.0013436},
{0.0034961,0.0239562,0.0099808},
{-0.0158750,-0.0695825,0.0375494},
{-0.0131893,-0.0152049,0.0064271},
{-0.0134998,0.0206552,0.0017825},
{-0.0205940,0.0028635,0.0035167},
{-0.0075768,-0.0943994,0.0307494},
{-0.0047946,-0.1131861,0.0466255},
{0.0162685,-0.1188722,0.0496994},
{0.0138873,-0.1015643,0.0241435},
{-0.0112730,-0.0753951,0.0237689},
{-0.0161762,-0.0107605,-0.0113381},
{0.0007325,-0.0111128,-0.0207214},
{0.0066537,0.0094508,-0.0190732},
{0.0018722,0.0244466,-0.0090112},
{-0.0117105,0.0102276,-0.0161236},
{0.0004931,-0.1190765,0.0258592},
{-0.0020710,-0.1353783,0.0444594},
{0.0175201,-0.1433363,0.0474162},
{0.0208978,-0.1232233,0.0292110},
{0.0069488,-0.1399067,0.0214494},
{0.0000154,-0.1580761,0.0389313},
{0.0187529,-0.1678422,0.0449672},
{0.0249411,-0.1495654,0.0286769},
{0.0108104,-0.1628538,0.0183255},
{0.0026016,-0.1814816,0.0357496},
{0.0213387,-0.1926176,0.0419461},
{0.0275428,-0.1750228,0.0257788},
{0.0127674,-0.1876518,0.0156367},
{0.0042783,-0.2048604,0.0321700},
{0.0216748,-0.2176696,0.0393755},
{0.0292725,-0.1981703,0.0233569},
{0.0143033,-0.2117950,0.0132317},
{0.0029409,-0.2289966,0.0238075},
{0.0155983,-0.2419230,0.0351337},

{0.0307148,-0.2394085,0.0267441},
{0.0296601,-0.2205584,0.0187246},
{0.0178726,-0.2391899,0.0098461},
{0.0026186,-0.2535606,0.0203281},
{0.0123550,-0.2714175,0.0306700},
{0.0275813,-0.2623329,0.0316599},
{0.0299426,-0.2597389,0.0144650},
{0.0157846,-0.2678939,0.0076327},
{0.0022110,-0.2825857,0.0172404},
{0.0135304,-0.2987517,0.0294161},
{0.0284725,-0.2857679,0.0282748},
{0.0297951,-0.2870832,0.0110542},
{0.0142031,-0.2964792,0.0049824},
{0.0032143,-0.3099698,0.0164284},
{0.0109366,-0.3263089,0.0269151},
{0.0289393,-0.3127785,0.0249611},
{0.0288565,-0.3117668,0.0067572},
{0.0111564,-0.3255120,0.0026724},
{0.0028653,-0.3433766,0.0117779},
{0.0122012,-0.3521983,0.0266023},
{0.0284743,-0.3394178,0.0230075},
{0.0290874,-0.3338547,0.0044210},
{0.0170016,-0.3492846,-0.0031025},
{0.0043064,-0.3686602,0.0008378},
{0.0027916,-0.3714832,0.0200743},
{0.0242812,-0.3708951,0.0238818},
{0.0317792,-0.3606907,0.0090394},
{0.0251750,-0.3757454,-0.0053191},
{0.0070965,-0.3899468,-0.0073397},
{-0.0001845,-0.3947382,0.0100122},
{0.0130164,-0.3922867,0.0259466},
{0.0318641,-0.3903787,0.0122171},
{0.0249297,-0.4002818,-0.0090114},
{0.0050158,-0.4141624,-0.0132508},
{-0.0023908,-0.4205919,0.0046358},
{0.0018014,-0.4125858,0.0233011},
{0.0245582,-0.4118321,0.0229998},
{0.0312720,-0.4158443,0.0055022},
{0.0237077,-0.4246221,-0.0141579},
{0.0107456,-0.4402620,-0.0261593},
{-0.0056701,-0.4348696,-0.0147295},
{-0.0068919,-0.4492421,0.0030673},
{-0.0063479,-0.4390001,0.0217072},
{0.0112452,-0.4336120,0.0282574},
{0.0302394,-0.4362238,0.0192865},
{0.0289245,-0.4420973,-0.0001591},

Biology Open • Supplementary information

{0.0274921,-0.4517726,-0.0194506},
{0.0191411,-0.4684118,-0.0323930},
{0.0046155,-0.4565596,-0.0408518},
{-0.0101841,-0.4459153,-0.0315579},
{-0.0175985,-0.4564032,-0.0135487},
{-0.0065137,-0.4753699,-0.0035364},
{-0.0188817,-0.4653938,0.0117777},
{-0.0163988,-0.4569247,0.0320503},
{0.0022814,-0.4519106,0.0375192},
{0.0225364,-0.4571417,0.0299325},
{0.0381419,-0.4583699,0.0170454},
{0.0294932,-0.4660076,-0.0019902},
{0.0319293,-0.4819917,-0.0175124},
{0.0187541,-0.4966479,-0.0296843},
{0.0027387,-0.4818074,-0.0424796},
{-0.0146260,-0.4668534,-0.0430118},
{-0.0296984,-0.4595692,-0.0304803},
{-0.0294630,-0.4765942,-0.0129244},
{-0.0188083,-0.4971703,-0.0190313},
{0.0001681,-0.4968505,-0.0126271},
{0.0032369,-0.4940142,0.0080815},
{-0.0154151,-0.4909264,0.0150075},
{-0.0264958,-0.4768014,0.0304993},
{-0.0047925,-0.4737047,0.0432813},
{0.0142458,-0.4782229,0.0357484},
{0.0347497,-0.4796584,0.0262778},
{0.0290357,-0.4851476,0.0077486},
{0.0179684,-0.4924632,-0.0069900},
{-0.0001555,-0.5038004,-0.0324124},
{-0.0155096,-0.4939238,-0.0410891},
{-0.0322306,-0.4831913,-0.0343716},
{0.0210165,-0.4978721,0.0225538},
{0.0016821,-0.5008647,0.0295842},
{-0.0156855,-0.4956879,0.0360378},
{-0.0009049,-0.0810472,0.0207645},
{-0.0108482,-0.0705533,0.0225174},
{0.0103408,-0.0413876,0.0673876},
{-0.0149207,-0.0168172,0.0621139},
{-0.0018290,-0.0689408,0.0624448},
{-0.0066184,-0.0774188,0.0244992},
{-0.0014823,-0.0994767,0.0223719},
{-0.0073494,0.0020552,-0.0185380},
{-0.0131021,-0.0541079,0.0448707},
{0.0034742,-0.0322526,0.0714473},
{-0.0106827,-0.0799756,0.0280219},
{-0.0011219,-0.0657309,0.0200092},

Biology Open • Supplementary information

{-0.0138534,-0.0206422,0.0553651},
{0.0143146,-0.0365037,0.0655574},
{-0.0110038,-0.0362567,0.0432712},
{0.0150257,-0.0417239,0.0637220},
{-0.0009369,-0.0908029,0.0585293},
{-0.0155818,-0.0531586,0.0340686},
{-0.0146814,-0.0674082,0.0239665},
{-0.0170965,-0.0659298,0.0253515},
{-0.0026080,-0.0501038,0.0147934},
{0.0113882,-0.0702368,0.0260348},
{0.0228152,-0.0160648,0.0431147},
{-0.0032856,-0.0533740,0.0674858},
{0.0142645,-0.0034019,0.0187589},
{-0.0093203,-0.0589686,0.0206015},
{-0.0062830,-0.0980294,0.0483190},
{0.0058766,0.0179020,0.0142905},
{-0.0119118,0.0205970,-0.0065511},
{-0.0074396,-0.0814406,0.0517202},
{0.0082642,-0.1024323,0.0211275},
{0.0136290,-0.0898559,0.0264476},
{0.0081658,-0.0570424,0.0639983},
{0.0152223,-0.0152462,0.0082602},
{-0.0060922,-0.0284286,0.0049819},
{-0.0075822,-0.0011726,0.0218497},
{-0.0106525,-0.0678963,0.0532199},
{-0.0065339,-0.0315892,0.0715154},
{0.0088743,-0.0076703,0.0653170},
{0.0143541,-0.0609775,0.0322238},
{-0.0021173,-0.0218775,-0.0131089},
{-0.0145421,-0.0295043,0.0593825},
{0.0168979,0.0160521,-0.0076292},
{-0.0042810,0.0260123,0.0039746},
{0.0006660,-0.1025832,0.0567689},
{-0.0137036,-0.0476917,0.0537620},
{0.0209511,-0.0337243,0.0456512},
{0.0122215,-0.0517676,0.0259524},
{0.0188040,0.0125299,0.0077674},
{0.0216710,0.0025094,0.0072551},
{0.0226849,-0.0023883,-0.0036722},
{0.0145313,-0.0242542,0.0242104},
{0.0120620,-0.0417493,0.0231154},
{0.0171187,-0.0176999,0.0346339},
{-0.0183063,-0.0590510,0.0284703},
{0.0031676,-0.0461615,0.0693279},
{0.0177105,-0.0543707,0.0412581},
{0.0153082,-0.0121937,0.0142491},

{0.0010811,0.0052879,-0.0208522},
{0.0091361,-0.0009187,-0.0206937},
{0.0109423,-0.0811397,0.0576491},
{0.0156278,-0.0732711,0.0547644},
{0.0183557,-0.0711897,0.0455372},
{0.0185657,-0.0824135,0.0385103},
{-0.0049442,-0.0899188,0.0263553},
{-0.0138904,0.0044896,0.0149848},
{0.0080826,-0.0013490,0.0591734},
{0.0201102,-0.0112394,0.0539381},
{0.0156789,-0.0284073,0.0645405},
{-0.0124692,-0.0429479,0.0271588},
{-0.0192201,-0.0089943,-0.0027382},
{0.0171009,-0.0992956,0.0293551},
{-0.0072997,-0.0068903,-0.0203683},
{0.0043041,0.0169113,-0.0158688},
{-0.0097729,-0.0396379,0.0171344},
{-0.0155555,0.0123683,-0.0111493},
{-0.0211127,0.0059481,-0.0017794},
{-0.0095425,-0.0840180,0.0423490},
{0.0122208,-0.0208784,-0.0010159},
{0.0131527,-0.0544493,0.0614479},
{0.0091305,-0.0221369,-0.0096256},
{0.0105813,-0.0166717,-0.0147528},
{0.0045309,-0.0711575,0.0202917},
{0.0094340,-0.0607012,0.0229908},
{0.0012893,-0.0910001,0.0214950},
{-0.0191930,-0.0671408,0.0323372},
{-0.0063806,-0.0016276,0.0558709},
{-0.0138355,-0.0368436,0.0637288},
{0.0189465,-0.0207608,0.0579723},
{0.0158515,-0.0920761,0.0517003},
{0.0198583,-0.0287190,0.0375775},
{0.0088345,-0.0381031,0.0712749},
{-0.0179931,-0.0038877,0.0106404},
{0.0152582,-0.0193096,0.0645071},
{0.0163874,-0.0733001,0.0349471},
{-0.0160962,-0.0599999,0.0241437},
{-0.0148828,-0.0399272,0.0563975},
{0.0147161,-0.0498231,0.0327679},
{-0.0082818,-0.0463579,0.0690072},
{0.0070327,-0.0092663,-0.0202089},
{0.0043852,-0.0184635,-0.0162515},
{-0.0152633,-0.0129098,0.0552047},
{-0.0110714,-0.0535879,0.0621320},
{0.0184989,-0.0647120,0.0506936},

Biology Open • Supplementary information

{-0.0179126,0.0133700,0.0016823},
{-0.0146533,0.0109807,0.0124985},
{-0.0020208,-0.0814258,0.0588009},
{0.0140956,-0.0111213,0.0365918},
{0.0147360,-0.0041959,0.0591503},
{0.0162058,-0.0068777,0.0463537},
{-0.0135797,-0.0161137,-0.0092344},
{0.0060862,-0.0263005,0.0025266},
{0.0003921,-0.0587432,0.0183038},
{0.0212849,0.0037502,-0.0081584},
{0.0182439,0.0019458,-0.0141461},
{0.0136063,-0.0092420,-0.0169664},
{-0.0139640,0.0016446,-0.0165996},
{-0.0155924,-0.0071620,-0.0142238},
{0.0188216,0.0158547,0.0034454},
{0.0112464,0.0228613,-0.0024662},
{0.0095997,0.0166312,-0.0142157},
{0.0085781,-0.0270752,0.0693668},
{-0.0007564,-0.0103425,0.0684165},
{-0.0001764,-0.0025064,0.0616583},
{0.0057280,-0.0164407,0.0699901},
{0.0089440,0.0088082,0.0178023},
{0.0131274,-0.0208564,0.0168814},
{0.0041959,-0.0303002,0.0711700},
{-0.0097213,-0.0207104,0.0262900},
{-0.0108955,-0.0209317,0.0131238},
{-0.0069058,-0.0119875,0.0293561},
{-0.0160762,-0.0607963,0.0384041},
{-0.0121126,-0.0734962,0.0444522},
{-0.0073933,-0.0348843,0.0101620},
{0.0220985,-0.0221836,0.0501664},
{0.0130581,-0.0101485,0.0300440},
{0.0046144,-0.0011346,0.0263217},
{-0.0038262,-0.0911828,0.0572317},
{-0.0025675,0.0263114,-0.0035467},
{-0.0012203,-0.0158565,0.0424391},
{-0.0116758,-0.0172323,0.0026721},
{0.0138405,-0.0345828,0.0278160},
{0.0110283,-0.0326167,0.0185860},
{-0.0117510,-0.0083467,0.0571280},
{0.0200072,-0.0397971,0.0520369},
{-0.0128943,-0.0643148,0.0450750},
{-0.0079799,-0.0972077,0.0408647},
{0.0046282,-0.0063068,0.0334680},
{-0.0031921,-0.0055356,-0.0216583},
{-0.0134727,-0.0417666,0.0632506},

{-0.0032151,0.0106995,-0.0183093},
{-0.0046165,0.0199481,-0.0135214},
{0.0198812,-0.1031863,0.0393964},
{0.0039450,-0.0889866,0.0206464},
{0.0175862,-0.1064639,0.0484310},
{0.0102429,0.0230826,0.0058369},
{0.0125467,0.0110038,0.0158114},
{0.0011737,0.0181303,-0.0155165},
{0.0171753,-0.0140596,-0.0095973},
{0.0202266,-0.0091963,-0.0082303},
{-0.0092936,-0.0293055,0.0330051},
{-0.0116477,-0.0416732,0.0348878},
{-0.0143166,-0.0744339,0.0279082},
{-0.0015123,0.0032112,0.0217971},
{0.0044367,0.0088358,0.0188049},
{-0.0026869,-0.0034426,0.0278464},
{0.0002742,-0.0371935,0.0706302},
{0.0073685,-0.0524312,0.0650018},
{-0.0124794,-0.0239734,0.0680917},
{-0.0124505,-0.0317922,0.0672567},
{0.0096397,0.0244629,0.0028735},
{0.0182344,0.0170871,0.0015247},
{-0.0075973,-0.0131827,0.0693269},
{0.0162329,-0.0642099,0.0372998},
{-0.0132296,-0.0574541,0.0521429},
{-0.0160008,-0.0609296,0.0238844},
{0.0116805,-0.1018452,0.0549164},
{-0.0104428,-0.0304934,0.0192150},
{-0.0107638,-0.0185691,0.0184753},
{-0.0047164,0.0193223,0.0137111},
{0.0009529,-0.0823316,0.0607116},
{0.0062731,-0.0759059,0.0608982},
{-0.0024058,-0.0755295,0.0603502},
{0.0176539,-0.0890035,0.0337088},
{-0.0132673,-0.0220978,0.0531224},
{0.0113642,-0.0300245,0.0187038},
{0.0054893,-0.0634101,0.0635775},
{0.0088444,-0.0423779,0.0675945},
{0.0101592,-0.1017864,0.0215379},
{-0.0072184,-0.0638688,0.0617294},
{0.0190899,-0.0516765,0.0477102},
{-0.0126919,-0.0516553,0.0229470},
{0.0225614,-0.0248809,0.0405875},
{-0.0042899,-0.0735382,0.0220128},
{0.0177254,-0.0024015,0.0138463},
{0.0065716,0.0065156,0.0189668},

Biology Open • Supplementary information

{0.0009751,-0.0391023,0.0705025},
{-0.0157811,0.0165379,-0.0055007},
{0.0112929,0.0188904,0.0110152},
{-0.0113607,0.0192741,0.0088385},
{-0.0198111,-0.0066146,-0.0055964},
{-0.0199291,0.0006118,-0.0080278},
{-0.0052175,-0.0210447,0.0719322},
{-0.0139988,-0.0412941,0.0471634},
{0.0059479,-0.0563454,0.0649712},
{0.0132382,-0.0500416,0.0625399},
{-0.0093060,-0.0820864,0.0275058},
{0.0134481,0.0085177,0.0162827},
{0.0194111,-0.0128502,-0.0012155},
{-0.0184438,-0.0096549,0.0054181},
{-0.0136010,-0.0675406,0.0417938},
{-0.0077857,-0.0202153,-0.0040224},
{-0.0137722,-0.0330578,0.0505529},
{0.0049538,-0.0898150,0.0590336},
{0.0170071,-0.0598685,0.0556538},
{-0.0018475,-0.0417963,0.0118340},
{0.0066516,-0.0383822,0.0141056},
{0.0199879,-0.0083558,0.0062029},
{0.0192999,-0.0976262,0.0447502},
{-0.0097239,-0.0226919,0.0269418},
{-0.0009357,0.0155300,0.0172425},
{0.0078833,-0.0818016,0.0227098},
{0.0093633,-0.0747518,0.0602072},
{0.0159930,-0.0155361,-0.0101071},
{0.0005919,-0.0109065,0.0689263},
{-0.0076132,-0.0176682,-0.0151539},
{0.0169239,-0.0844513,0.0502702},
{0.0081860,-0.0220599,-0.0110608},
{-0.0113014,-0.0772160,0.0248327},
{0.0141728,-0.0470908,0.0310311},
{0.0165199,-0.0051225,0.0575957},
{0.0050507,-0.0091467,0.0419831},
{-0.0032324,-0.0739472,0.0609461},
{-0.0136306,-0.0712740,0.0407007},
{-0.0153875,-0.0136758,0.0576340},
{-0.0083670,0.0146841,-0.0156251},
{-0.0182774,0.0069242,0.0086914},
{0.0099512,-0.0814802,0.0236757},
{0.0026548,-0.0465154,0.0683546},
{-0.0143741,-0.0259287,0.0586291},
{0.0068551,-0.0073336,-0.0209568},
{0.0163983,-0.0337720,0.0602661},

{0.0127347,-0.0178946,0.0661306},
{0.0110914,0.0024709,-0.0193028},
{0.0145905,-0.0096720,0.0183312},
{-0.0137737,0.0131892,0.0124674},
{0.0110262,-0.0625764,0.0613875},
{-0.0058177,-0.0454429,0.0698857},
{0.0206944,-0.0343859,0.0533734},
{0.0168671,-0.0156519,-0.0074759},
{-0.0158818,-0.0593754,0.0389386},
{-0.0080375,-0.0871811,0.0482759},
{0.0034054,-0.0622160,0.0643659},
{-0.0046985,-0.0489548,0.0149699},
{0.0118531,0.0126339,-0.0156074},
{-0.0022811,-0.0255322,-0.0009018},
{-0.0097573,-0.0866003,0.0322876},
{-0.0109932,-0.0041367,-0.0187702},
{-0.0031038,0.0255967,-0.0049932},
{-0.0102047,-0.0256435,0.0141740},
{-0.0114326,-0.0760736,0.0246430},
{-0.0063298,-0.0882310,0.0265316},
{0.0010145,0.0139968,-0.0178552},
{0.0211236,-0.0306774,0.0525525},
{-0.0062754,-0.0684633,0.0212899},
{0.0187679,-0.0428528,0.0424714},
{0.0187407,-0.0363252,0.0378396},
{-0.0152868,-0.0141133,-0.0010487},
{0.0141288,0.0165364,0.0108596},
{-0.0142529,-0.0128716,0.0074587},
{-0.0113901,-0.0415156,0.0385020},
{0.0191215,-0.1034772,0.0457036},
{0.0188271,-0.0121844,0.0599947},
{0.0199834,-0.0183460,0.0555806},
{0.0014503,-0.0312144,0.0054102},
{0.0114929,-0.0275256,0.0676864},
{-0.0029712,-0.0112401,0.0338277},
{-0.0205339,-0.0049990,-0.0018729},
{0.0029374,-0.0247486,-0.0041044},
{-0.0103155,-0.0837530,0.0398132},
{-0.0135684,-0.0323513,0.0656314},
{0.0190573,0.0061081,-0.0115943},
{-0.0157329,-0.0732752,0.0324523},
{-0.0162249,-0.0049411,0.0137803},
{0.0127761,-0.0094559,0.0291404},
{-0.0143058,-0.0504053,0.0345159},
{0.0160281,-0.0841795,0.0310084},
{0.0008195,0.0039627,-0.0211244},

{0.0179818,-0.0911303,0.0338337},
{0.0073944,0.0075026,0.0181735},
{-0.0152159,0.0026568,0.0139537},
{0.0062316,-0.0504543,0.0178881},
{-0.0068971,-0.0223634,0.0366618},
{0.0218898,-0.0316829,0.0463097},
{-0.0137472,-0.0735647,0.0247790},
{0.0222151,0.0060097,-0.0032819},
{-0.0147810,-0.0737595,0.0301924},
{0.0120002,-0.0262850,0.0120370},
{0.0210276,-0.0137385,0.0494034},
{0.0092281,0.0022858,-0.0203233},
{0.0157179,-0.0097566,0.0147324},
{-0.0120833,-0.0746598,0.0437509},
{0.0004936,0.0241884,0.0100309},
{0.0030283,0.0038803,-0.0214706},
{0.0402192,-0.4734774,0.0229436},
{0.0054506,0.0239680,-0.0085008},
{-0.0145637,-0.0528482,0.0407466},
{-0.0338453,-0.4686824,-0.0293994},
{0.0024982,0.0271154,-0.0000349},
{0.0154950,-0.0288090,0.0290556},
{-0.0152201,-0.0012918,-0.0158781},
{0.0178480,-0.0363786,0.0579180},
{-0.0157995,0.0157893,0.0051830},
{0.0186072,-0.0470621,0.0437694},
{0.0166063,-0.0525024,0.0375430},
{-0.0143098,-0.0414128,0.0500334},
{0.0186845,-0.0664988,0.0481535},
{-0.0180351,-0.0577866,0.0300506},
{0.0032952,-0.0660197,0.0635465},
{0.0011726,-0.0615874,0.0189780},
{0.0230856,-0.0250947,0.0481383},
{0.0085233,-0.0917027,0.0576027},
{-0.0064127,-0.0995585,0.0305330},
{-0.0204596,-0.0025837,0.0033375},
{0.0155081,-0.0160857,0.0215757},
{0.0149156,-0.0799295,0.0300212},
{-0.0100130,-0.0296318,0.0300672},
{-0.0210062,-0.4805332,0.0390121},
{-0.0093212,-0.0669209,0.0570094},
{-0.0123340,-0.4740365,-0.0112308},
{0.0114953,-0.0518830,0.0244706},
{0.0116830,0.0174141,-0.0122287},
{-0.0262286,-0.4701250,0.0315665},
{0.0168388,-0.0637387,0.0382624},

{0.0003212,-0.0594351,0.0653910},
{-0.0118201,0.0217004,0.0031072},
{-0.0026553,-0.0200272,0.0717796},
{-0.0078499,-0.0088384,0.0263433},
{0.0208530,-0.0123597,0.0461100},
{0.0159694,-0.0293747,0.0624689},
{-0.0152062,-0.0130815,0.0568346},
{0.0213132,0.0098780,-0.0030655},
{-0.0107804,-0.0409208,0.0195779},
{-0.0003622,-0.0169390,-0.0179516},
{-0.0115873,-0.0722387,0.0482302},
{-0.0096828,0.0205386,0.0087570},
{0.0104964,0.0229587,-0.0032770},
{-0.0144036,-0.0324289,0.0622405},
{-0.0064307,-0.4707107,0.0442203},
{-0.0144409,-0.4771105,-0.0414462},
{-0.0143608,-0.4770465,-0.0405896},
{-0.0142807,-0.4769825,-0.0397329},
{-0.0142006,-0.4769185,-0.0388762},
{-0.0141205,-0.4768545,-0.0380196},
{-0.0140404,-0.4767905,-0.0371629},
{-0.0139603,-0.4767265,-0.0363062},
{-0.0138802,-0.4766625,-0.0354496},
{-0.0138001,-0.4765985,-0.0345929},
{-0.0137200,-0.4765345,-0.0337362},
{-0.0136399,-0.4764705,-0.0328796},
{-0.0135598,-0.4764065,-0.0320229},
{-0.0134797,-0.4763425,-0.0311663},
{-0.0133996,-0.4762785,-0.0303096},
{-0.0133195,-0.4762145,-0.0294529},
{-0.0132394,-0.4761505,-0.0285963},
{-0.0131593,-0.4760865,-0.0277396},
{-0.0130792,-0.4760225,-0.0268829},
{-0.0129991,-0.4759585,-0.0260263},
{-0.0129190,-0.4758945,-0.0251696},
{-0.0128389,-0.4758305,-0.0243129},
{-0.0127588,-0.4757665,-0.0234563},
{-0.0126787,-0.4757025,-0.0225996},
{-0.0125986,-0.4756385,-0.0217429},
{-0.0125185,-0.4755745,-0.0208863},
{-0.0124384,-0.4755105,-0.0200296},
{-0.0123583,-0.4754465,-0.0191729},
{-0.0122782,-0.4753825,-0.0183163},
{-0.0121981,-0.4753185,-0.0174596},
{-0.0121180,-0.4752545,-0.0166029},
{-0.0120379,-0.4751905,-0.0157463},

{-0.0119578,-0.4751265,-0.0148896},
{-0.0118777,-0.4750626,-0.0140329},
{-0.0117975,-0.4749986,-0.0131763},
{-0.0117174,-0.4749346,-0.0123196},
{-0.0116373,-0.4748706,-0.0114630},
{-0.0115572,-0.4748066,-0.0106063},
{-0.0114771,-0.4747426,-0.0097496},
{-0.0113970,-0.4746786,-0.0088930},
{-0.0113169,-0.4746146,-0.0080363},
{-0.0112368,-0.4745506,-0.0071796},
{-0.0111567,-0.4744866,-0.0063230},
{-0.0110766,-0.4744226,-0.0054663},
{-0.0109965,-0.4743586,-0.0046096},
{-0.0109164,-0.4742946,-0.0037530},
{-0.0108363,-0.4742306,-0.0028963},
{-0.0107562,-0.4741666,-0.0020396},
{-0.0106761,-0.4741026,-0.0011830},
{-0.0105960,-0.4740386,-0.0003263},
{-0.0105159,-0.4739746,0.0005304},
{-0.0104358,-0.4739106,0.0013870},
{-0.0103557,-0.4738466,0.0022437},
{-0.0102756,-0.4737826,0.0031004},
{-0.0101955,-0.4737186,0.0039570},
{-0.0101154,-0.4736546,0.0048137},
{-0.0100353,-0.4735906,0.0056704},
{-0.0099552,-0.4735266,0.0065270},
{-0.0098751,-0.4734626,0.0073837},
{-0.0097950,-0.4733986,0.0082403},
{-0.0097149,-0.4733346,0.0090970},
{-0.0096348,-0.4732706,0.0099537},
{-0.0095547,-0.4732066,0.0108103},
{-0.0094746,-0.4731426,0.0116670},
{-0.0093945,-0.4730786,0.0125237},
{-0.0093144,-0.4730146,0.0133803},
{-0.0092343,-0.4729506,0.0142370},
{-0.0091542,-0.4728866,0.0150937},
{-0.0090741,-0.4728226,0.0159503},
{-0.0089940,-0.4727586,0.0168070},
{-0.0089138,-0.4726946,0.0176637},
{-0.0088337,-0.4726306,0.0185203},
{-0.0087536,-0.4725666,0.0193770},
{-0.0086735,-0.4725026,0.0202337},
{-0.0085934,-0.4724387,0.0210903},
{-0.0085133,-0.4723747,0.0219470},
{-0.0084332,-0.4723107,0.0228037},
{-0.0083531,-0.4722467,0.0236603},

```
                    {-0.0082730,-0.4721827,0.0245170},
                    {-0.0081929,-0.4721187,0.0253737},
                    {-0.0081128,-0.4720547,0.0262303},
                    {-0.0080327,-0.4719907,0.0270870},
                    {-0.0079526,-0.4719267,0.0279437},
                    {-0.0078725,-0.4718627,0.0288003},
                    {-0.0077924,-0.4717987,0.0296570},
                    {-0.0077123,-0.4717347,0.0305136},
                    {-0.0076322,-0.4716707,0.0313703},
                    {-0.0075521,-0.4716067,0.0322270},
                    {-0.0074720,-0.4715427,0.0330836},
                    {-0.0073919,-0.4714787,0.0339403},
                    {-0.0073118,-0.4714147,0.0347970},
                    {-0.0072317,-0.4713507,0.0356536},
                    {-0.0071516,-0.4712867,0.0365103},
                    {-0.0070715,-0.4712227,0.0373670},
                    {-0.0069914,-0.4711587,0.0382236},
                    {-0.0069113,-0.4710947,0.0390803},
                    {-0.0068312,-0.4710307,0.0399370},
                    {-0.0067511,-0.4709667,0.0407936},
                    {-0.0066710,-0.4709027,0.0416503},
                    {-0.0065909,-0.4708387,0.0425070},
                    {-0.0065108,-0.4707747,0.0433636}
                    };
            BoundingBoxOnOff = Off;
        };
        AnyFunTransform3DIdentity ScaleFunction = {
            PreTransforms = {&.RBFTransform};
};
};

};
};
```

Biology Open • Supplementary information

ScalingFunctionTLEMLucyFemur_Mirrored_2014031

```
AnyFolder ScalingFunctionTLEMLucyFemur_Mirrored = {
 AnyFolder Left = {
       AnyFolder Thigh = {
     AnyMatrix AMirroring = {
     {1,0,0},
     {0,1,0},
     {0,0,-1}
     };
       AnyFunTransform3DRBF RBFTransform = {
              RBFDef.Type = RBF_ThinPlate;
              PolynomDegree = 1;
              Points0 = ({
                     {0.0000000,0.0000000,0.0000000},
                     {-0.0000000,-0.3616821,0.0000000},
                     {-0.0097563,-0.3678799,0.0012967},
                     {-0.0000000,-0.3660632,0.0408203},
                     {-0.0000000,-0.3573010,-0.0408203},
                     {0.0161460,-0.0072838,0.0601290},
                     {0.0220217,-0.0203698,0.0463848},
                     {0.0123977,-0.0241932,0.0668573},
                     {0.0006898,0.0018121,0.0538181},
                     {0.0122809,-0.0068668,0.0414535},
                     {-0.0058991,-0.0138188,0.0648412},
                     {0.0211469,-0.0380855,0.0538111},
                     {0.0172133,-0.0317342,0.0328381},
                     {0.0177311,-0.0131946,0.0256176},
                     {-0.0110079,-0.0308867,0.0632370},
                     {0.0039435,-0.0395977,0.0638271},
                     {-0.0041683,-0.0187472,0.0413214},
                     {-0.0177999,-0.0021535,0.0496084},
                     {0.0001603,-0.0053126,0.0279593},
                     {-0.0211600,-0.0216243,0.0497147},
                     {0.0096163,-0.0568663,0.0545719},
                     {0.0162112,-0.0515013,0.0395230},
                     {0.0093533,-0.0433265,0.0187387},
                     {0.0119398,-0.0238469,0.0118403},
                     {0.0210707,-0.0048848,0.0065878},
                     {0.0125013,0.0064094,0.0177702},
                     {-0.0191523,-0.0356053,0.0382429},
                     {-0.0115821,-0.0474937,0.0537648},
                     {-0.0100073,-0.0236371,0.0267322},
                     {-0.0078925,0.0107098,0.0182833},
                     {-0.0134217,-0.0055356,0.0182000},
                     {-0.0090061,-0.0665700,0.0475484},
```

Biology Open • Supplementary information

{0.0071095,-0.0761976,0.0520035},
{0.0182119,-0.0704149,0.0386546},
{0.0073764,-0.0609481,0.0221006},
{-0.0122984,-0.0439585,0.0198428},
{-0.0032253,-0.0306792,0.0110995},
{-0.0012975,-0.0198451,-0.0052592},
{0.0140405,-0.0161125,-0.0071649},
{0.0189222,-0.0014898,-0.0127226},
{0.0181642,0.0132812,-0.0011050},
{0.0045119,0.0200001,0.0091189},
{-0.0176761,-0.0541983,0.0348587},
{-0.0136548,-0.0142015,0.0054311},
{-0.0135510,0.0182940,0.0017175},
{-0.0223865,0.0023399,0.0028018},
{-0.0086455,-0.0738247,0.0283843},
{-0.0050356,-0.0877749,0.0442195},
{0.0160135,-0.0919319,0.0465120},
{0.0128290,-0.0785810,0.0232431},
{-0.0168328,-0.0616327,0.0155917},
{-0.0172369,-0.0094567,-0.0119502},
{0.0002090,-0.0093641,-0.0209779},
{0.0064827,0.0081538,-0.0198453},
{0.0010777,0.0210422,-0.0084721},
{-0.0129485,0.0085451,-0.0162212},
{-0.0002322,-0.0924033,0.0245644},
{-0.0028175,-0.1052263,0.0418294},
{0.0172322,-0.1111444,0.0445760},
{0.0203259,-0.0957409,0.0271449},
{0.0057102,-0.1088933,0.0195113},
{-0.0010852,-0.1230776,0.0363132},
{0.0182727,-0.1303312,0.0422884},
{0.0242891,-0.1161703,0.0265054},
{0.0094531,-0.1268584,0.0162294},
{0.0014244,-0.1413293,0.0331653},
{0.0207271,-0.1496427,0.0394157},
{0.0267562,-0.1360142,0.0236798},
{0.0113839,-0.1461651,0.0136024},
{0.0030845,-0.1595094,0.0297156},
{0.0209528,-0.1691386,0.0369775},
{0.0283958,-0.1540209,0.0213795},
{0.0129499,-0.1649012,0.0113589},
{0.0016490,-0.1783108,0.0216287},
{0.0147105,-0.1880678,0.0328614},
{0.0298491,-0.1859865,0.0248551},
{0.0286645,-0.1714377,0.0169488},
{0.0165897,-0.1861006,0.0082595},

Biology Open • Supplementary information

{0.0014000,-0.1973476,0.0183740},
{0.0114301,-0.2109792,0.0286157},
{0.0267422,-0.2037622,0.0296951},
{0.0288630,-0.2018335,0.0130127},
{0.0145471,-0.2083505,0.0062833},
{0.0011300,-0.2198071,0.0155602},
{0.0126610,-0.2321235,0.0275354},
{0.0275647,-0.2219268,0.0265185},
{0.0286645,-0.2230253,0.0098701},
{0.0130434,-0.2304822,0.0039177},
{0.0022984,-0.2409585,0.0149650},
{0.0101315,-0.2534740,0.0252125},
{0.0279485,-0.2428555,0.0234276},
{0.0276684,-0.2421559,0.0058691},
{0.0101250,-0.2529527,0.0018983},
{0.0021376,-0.2667831,0.0107073},
{0.0114309,-0.2734905,0.0249960},
{0.0273923,-0.2634953,0.0216324},
{0.0278429,-0.2592546,0.0037692},
{0.0159101,-0.2712985,-0.0033988},
{0.0036708,-0.2863280,0.0004644},
{0.0023115,-0.2884756,0.0187554},
{0.0231385,-0.2879051,0.0224853},
{0.0304009,-0.2799973,0.0083549},
{0.0238189,-0.2917246,-0.0052462},
{0.0064904,-0.3027687,-0.0070587},
{-0.0003492,-0.3064690,0.0093014},
{0.0122179,-0.3045269,0.0243511},
{0.0301837,-0.3030127,0.0114462},
{0.0234182,-0.3107336,-0.0085198},
{0.0046893,-0.3214995,-0.0123881},
{-0.0022352,-0.3264905,0.0043340},
{0.0016841,-0.3202756,0.0217839},
{0.0229722,-0.3196887,0.0215083},
{0.0292438,-0.3228042,0.0051430},
{0.0221641,-0.3296190,-0.0132361},
{0.0100459,-0.3417597,-0.0244560},
{-0.0053009,-0.3375737,-0.0137705},
{-0.0064432,-0.3487307,0.0028676},
{-0.0059346,-0.3407801,0.0202938},
{0.0105130,-0.3365976,0.0264175},
{0.0282695,-0.3386249,0.0180307},
{0.0270402,-0.3431844,-0.0001487},
{0.0257040,-0.3506950,-0.0181851},
{0.0178948,-0.3636114,-0.0302838},
{0.0043149,-0.3544109,-0.0381949},

Biology Open • Supplementary information

{-0.0095210,-0.3461482,-0.0295031},
{-0.0164526,-0.3542895,-0.0126666},
{-0.0060896,-0.3690127,-0.0033061},
{-0.0176523,-0.3612686,0.0110109},
{-0.0153310,-0.3546943,0.0299634},
{0.0021329,-0.3508021,0.0350763},
{0.0210690,-0.3548628,0.0279835},
{0.0356594,-0.3558162,0.0159356},
{0.0275718,-0.3617450,-0.0018607},
{0.0298503,-0.3741530,-0.0163721},
{0.0175320,-0.3855300,-0.0277515},
{0.0025604,-0.3740099,-0.0397137},
{-0.0136727,-0.3624016,-0.0402102},
{-0.0277647,-0.3567472,-0.0284947},
{-0.0275446,-0.3699630,-0.0120829},
{-0.0175846,-0.3859355,-0.0177922},
{0.0001572,-0.3856873,-0.0118050},
{0.0030262,-0.3834856,0.0075553},
{-0.0144114,-0.3810887,0.0140304},
{-0.0247706,-0.3701239,0.0285134},
{-0.0044804,-0.3677201,0.0404632},
{0.0133183,-0.3712274,0.0334208},
{0.0324861,-0.3723417,0.0245668},
{0.0271451,-0.3766027,0.0072441},
{0.0167985,-0.3822816,-0.0065349},
{-0.0001454,-0.3910823,-0.0303010},
{-0.0144998,-0.3834154,-0.0384137},
{-0.0301321,-0.3750841,-0.0321336},
{0.0196491,-0.3864804,0.0210853},
{0.0015726,-0.3888034,0.0276569},
{-0.0146652,-0.3847848,0.0336913},
{-0.0014588,-0.0619951,0.0219778},
{-0.0118435,-0.0550626,0.0139639},
{0.0129812,-0.0349966,0.0673128},
{-0.0162907,-0.0105666,0.0597739},
{-0.0012885,-0.0520816,0.0562989},
{-0.0090599,-0.0631298,0.0172224},
{-0.0000222,-0.0771663,0.0231897},
{-0.0068148,0.0023885,-0.0160086},
{-0.0180544,-0.0425409,0.0446585},
{0.0020525,-0.0249904,0.0691549},
{-0.0157097,-0.0662315,0.0238393},
{-0.0010518,-0.0510686,0.0199353},
{-0.0208874,-0.0152865,0.0500034},
{0.0168316,-0.0280857,0.0666680},
{-0.0187392,-0.0282333,0.0403282},

Biology Open • Supplementary information

{0.0191622,-0.0351633,0.0622707},
{0.0001216,-0.0706289,0.0524049},
{-0.0169862,-0.0399062,0.0311827},
{-0.0185737,-0.0533066,0.0151224},
{-0.0219058,-0.0528723,0.0222263},
{-0.0028271,-0.0391620,0.0148452},
{0.0117900,-0.0553425,0.0235348},
{0.0219835,-0.0106400,0.0396338},
{-0.0025366,-0.0401046,0.0625273},
{0.0141017,-0.0018767,0.0198108},
{-0.0078770,-0.0473929,0.0173542},
{-0.0065351,-0.0766431,0.0470258},
{0.0065585,0.0145593,0.0161638},
{-0.0118588,0.0172875,-0.0085064},
{-0.0074087,-0.0627500,0.0506700},
{0.0071940,-0.0780068,0.0213362},
{0.0126736,-0.0708176,0.0244108},
{0.0074694,-0.0436450,0.0604196},
{0.0154186,-0.0139471,0.0064312},
{-0.0053535,-0.0213210,0.0040094},
{-0.0088733,0.0002018,0.0205402},
{-0.0112471,-0.0542608,0.0500266},
{-0.0061625,-0.0248673,0.0653244},
{0.0093695,-0.0065826,0.0614157},
{0.0138015,-0.0467322,0.0284333},
{-0.0020999,-0.0186885,-0.0116392},
{-0.0185640,-0.0225591,0.0566779},
{0.0175798,0.0124360,-0.0068467},
{-0.0039962,0.0217127,0.0053055},
{0.0014975,-0.0784591,0.0514056},
{-0.0172997,-0.0386971,0.0525252},
{0.0196540,-0.0268271,0.0416806},
{0.0135296,-0.0406504,0.0236882},
{0.0182471,0.0095959,0.0087906},
{0.0218667,0.0016804,0.0058793},
{0.0224975,-0.0022652,-0.0022994},
{0.0166750,-0.0191391,0.0236436},
{0.0119785,-0.0328723,0.0195376},
{0.0187631,-0.0133469,0.0308741},
{-0.0187543,-0.0461463,0.0257491},
{0.0039834,-0.0373928,0.0683089},
{0.0157029,-0.0423169,0.0385226},
{0.0164003,-0.0087069,0.0131181},
{0.0008253,0.0033370,-0.0212576},
{0.0101707,-0.0009724,-0.0205688},
{0.0102948,-0.0642683,0.0524532},

Biology Open • Supplementary information

{0.0152217,-0.0557944,0.0508978},
{0.0172973,-0.0565707,0.0419468},
{0.0167723,-0.0635334,0.0369834},
{-0.0047397,-0.0695325,0.0241398},
{-0.0152913,0.0035778,0.0161461},
{0.0091697,0.0009340,0.0575160},
{0.0196686,-0.0089533,0.0500006},
{0.0183998,-0.0212943,0.0622968},
{-0.0114340,-0.0316810,0.0250940},
{-0.0206442,-0.0092599,-0.0028425},
{0.0166868,-0.0784295,0.0276925},
{-0.0069678,-0.0054521,-0.0207011},
{0.0049490,0.0151109,-0.0160093},
{-0.0084025,-0.0318937,0.0162788},
{-0.0173679,0.0095594,-0.0108509},
{-0.0218912,0.0063457,-0.0020698},
{-0.0106547,-0.0668750,0.0397749},
{0.0136098,-0.0170666,-0.0018890},
{0.0141599,-0.0441405,0.0587594},
{0.0093255,-0.0183218,-0.0083008},
{0.0106367,-0.0128276,-0.0154269},
{0.0037350,-0.0549365,0.0209786},
{0.0087662,-0.0472902,0.0195496},
{0.0016839,-0.0708213,0.0221325},
{-0.0202977,-0.0530540,0.0301270},
{-0.0068584,0.0021781,0.0530002},
{-0.0144817,-0.0297218,0.0605833},
{0.0197015,-0.0165217,0.0560654},
{0.0156609,-0.0705363,0.0477736},
{0.0207876,-0.0221433,0.0330733},
{0.0092324,-0.0300767,0.0713160},
{-0.0203392,-0.0034488,0.0097332},
{0.0156978,-0.0143438,0.0629053},
{0.0143267,-0.0563519,0.0317489},
{-0.0161685,-0.0465391,0.0188591},
{-0.0196397,-0.0309875,0.0546496},
{0.0154804,-0.0380422,0.0305433},
{-0.0077774,-0.0351303,0.0637765},
{0.0063980,-0.0086419,-0.0201654},
{0.0030146,-0.0151094,-0.0165974},
{-0.0210080,-0.0096030,0.0507855},
{-0.0105684,-0.0411377,0.0586146},
{0.0180619,-0.0498960,0.0469835},
{-0.0192318,0.0116108,0.0024070},
{-0.0165651,0.0083622,0.0126870},
{-0.0014333,-0.0627138,0.0534746},

Biology Open • Supplementary information

{0.0145127,-0.0080178,0.0353667},
{0.0148374,-0.0012228,0.0566181},
{0.0153834,-0.0062427,0.0444727},
{-0.0155164,-0.0132103,-0.0096398},
{0.0068685,-0.0221022,0.0011547},
{0.0003332,-0.0458500,0.0183716},
{0.0205582,0.0040956,-0.0082969},
{0.0175752,0.0009340,-0.0148407},
{0.0142357,-0.0063623,-0.0169332},
{-0.0147285,0.0023412,-0.0164678},
{-0.0158781,-0.0064451,-0.0151346},
{0.0175951,0.0133456,0.0037559},
{0.0117660,0.0189899,-0.0030690},
{0.0096040,0.0151827,-0.0137450},
{0.0096565,-0.0215194,0.0662734},
{-0.0006793,-0.0074494,0.0631218},
{0.0007819,-0.0004519,0.0600781},
{0.0065377,-0.0125433,0.0654199},
{0.0090461,0.0073096,0.0188711},
{0.0151418,-0.0168302,0.0146465},
{0.0025166,-0.0231863,0.0688894},
{-0.0097891,-0.0152077,0.0253086},
{-0.0109149,-0.0177935,0.0119825},
{-0.0072079,-0.0099373,0.0272725},
{-0.0183727,-0.0467429,0.0356276},
{-0.0126871,-0.0584555,0.0428141},
{-0.0057054,-0.0273042,0.0103554},
{0.0215142,-0.0159693,0.0474531},
{0.0128492,-0.0070016,0.0287645},
{0.0052134,-0.0017047,0.0247463},
{-0.0024284,-0.0707498,0.0513049},
{-0.0036140,0.0220593,-0.0040484},
{-0.0002405,-0.0121253,0.0390050},
{-0.0119811,-0.0156737,0.0015445},
{0.0142082,-0.0279792,0.0246553},
{0.0122244,-0.0262013,0.0169829},
{-0.0146264,-0.0038855,0.0549384},
{0.0207450,-0.0325771,0.0496412},
{-0.0155040,-0.0497560,0.0437087},
{-0.0081119,-0.0756450,0.0387365},
{0.0039684,-0.0062047,0.0313900},
{-0.0023726,-0.0038892,-0.0220521},
{-0.0136977,-0.0339839,0.0602619},
{-0.0035399,0.0089081,-0.0188894},
{-0.0049820,0.0176571,-0.0132254},
{0.0193902,-0.0803191,0.0360429},

{0.0040648,-0.0697109,0.0218205},
{0.0170470,-0.0818610,0.0456892},
{0.0104175,0.0188635,0.0064965},
{0.0135206,0.0092549,0.0158079},
{0.0023933,0.0160498,-0.0157404},
{0.0164753,-0.0118197,-0.0096995},
{0.0202584,-0.0066858,-0.0082383},
{-0.0098401,-0.0226001,0.0314028},
{-0.0130937,-0.0309622,0.0320764},
{-0.0201510,-0.0613297,0.0222264},
{-0.0010389,0.0036055,0.0209003},
{0.0042025,0.0073392,0.0199393},
{-0.0021630,-0.0038531,0.0251898},
{-0.0007845,-0.0293574,0.0675788},
{0.0070027,-0.0406327,0.0624565},
{-0.0124363,-0.0183001,0.0627966},
{-0.0120485,-0.0256613,0.0628493},
{0.0101200,0.0197695,0.0039334},
{0.0167636,0.0148266,0.0014155},
{-0.0074981,-0.0106930,0.0632797},
{0.0142533,-0.0503445,0.0351954},
{-0.0146107,-0.0460953,0.0506120},
{-0.0161117,-0.0471301,0.0178900},
{0.0112149,-0.0792793,0.0501460},
{-0.0099060,-0.0230794,0.0186951},
{-0.0114543,-0.0153049,0.0183675},
{-0.0042882,0.0161879,0.0151423},
{0.0014582,-0.0638572,0.0541080},
{0.0048554,-0.0582603,0.0553815},
{-0.0017077,-0.0572302,0.0546635},
{0.0166090,-0.0697169,0.0323038},
{-0.0208812,-0.0164273,0.0474354},
{0.0130454,-0.0241864,0.0178294},
{0.0053123,-0.0483770,0.0582251},
{0.0112225,-0.0361429,0.0673300},
{0.0085729,-0.0771256,0.0216100},
{-0.0070783,-0.0488033,0.0560869},
{0.0182083,-0.0411024,0.0454236},
{-0.0119590,-0.0403809,0.0213546},
{0.0233409,-0.0185535,0.0366343},
{-0.0046347,-0.0571211,0.0208243},
{0.0183166,-0.0006966,0.0140116},
{0.0061913,0.0053512,0.0201003},
{-0.0002364,-0.0310828,0.0680351},
{-0.0170773,0.0139270,-0.0054987},
{0.0110805,0.0156034,0.0117010},

Biology Open • Supplementary information

{-0.0124628,0.0164667,0.0094550},
{-0.0209014,-0.0073407,-0.0051969},
{-0.0210131,0.0010693,-0.0086891},
{-0.0041596,-0.0172332,0.0663041},
{-0.0216595,-0.0322172,0.0451540},
{0.0051908,-0.0426014,0.0613013},
{0.0150003,-0.0416136,0.0606257},
{-0.0135090,-0.0674234,0.0236425},
{0.0146416,0.0073691,0.0160052},
{0.0195615,-0.0112412,-0.0010333},
{-0.0198115,-0.0098244,0.0044735},
{-0.0157054,-0.0521236,0.0395039},
{-0.0081381,-0.0173108,-0.0046152},
{-0.0218324,-0.0262661,0.0471115},
{0.0051446,-0.0707257,0.0530555},
{0.0179359,-0.0471493,0.0528134},
{-0.0005591,-0.0317983,0.0113632},
{0.0071707,-0.0305943,0.0121186},
{0.0206239,-0.0061367,0.0066642},
{0.0187057,-0.0745868,0.0420207},
{-0.0095784,-0.0163113,0.0259524},
{-0.0012181,0.0127661,0.0186855},
{0.0059420,-0.0630691,0.0220087},
{0.0069408,-0.0573878,0.0552614},
{0.0150782,-0.0132884,-0.0101793},
{0.0005533,-0.0078574,0.0633775},
{-0.0088284,-0.0146399,-0.0147837},
{0.0167444,-0.0651105,0.0470347},
{0.0082666,-0.0181741,-0.0095133},
{-0.0175162,-0.0637444,0.0177069},
{0.0153795,-0.0360767,0.0287608},
{0.0162363,-0.0018864,0.0548412},
{0.0052731,-0.0075919,0.0392990},
{-0.0022288,-0.0556644,0.0549909},
{-0.0156238,-0.0550038,0.0377688},
{-0.0204137,-0.0100550,0.0536429},
{-0.0093683,0.0122429,-0.0162013},
{-0.0202927,0.0049166,0.0083887},
{0.0074706,-0.0627945,0.0222133},
{0.0035034,-0.0376103,0.0672421},
{-0.0190752,-0.0194129,0.0555530},
{0.0061758,-0.0074961,-0.0207741},
{0.0192011,-0.0273063,0.0588233},
{0.0130796,-0.0134383,0.0638611},
{0.0120421,0.0019566,-0.0194140},
{0.0154152,-0.0065294,0.0179581},

Biology Open • Supplementary information

{-0.0157335,0.0101219,0.0123981},
{0.0110376,-0.0488274,0.0567911},
{-0.0055170,-0.0343676,0.0647140},
{0.0216204,-0.0273988,0.0512041},
{0.0161331,-0.0135875,-0.0075501},
{-0.0184643,-0.0455633,0.0360087},
{-0.0091978,-0.0682413,0.0460847},
{0.0034304,-0.0471677,0.0588866},
{-0.0048025,-0.0384316,0.0149400},
{0.0118505,0.0109735,-0.0159161},
{-0.0027036,-0.0204189,-0.0015094},
{-0.0117607,-0.0692759,0.0292171},
{-0.0107080,-0.0033455,-0.0190487},
{-0.0042548,0.0214947,-0.0056846},
{-0.0097315,-0.0208529,0.0131997},
{-0.0178958,-0.0627218,0.0171551},
{-0.0068692,-0.0688818,0.0233316},
{0.0012083,0.0120695,-0.0186179},
{0.0215073,-0.0236027,0.0503069},
{-0.0060262,-0.0534931,0.0189523},
{0.0179951,-0.0336785,0.0397267},
{0.0190509,-0.0284972,0.0336394},
{-0.0169188,-0.0133609,-0.0014203},
{0.0135722,0.0136491,0.0114695},
{-0.0150549,-0.0123230,0.0067722},
{-0.0148411,-0.0311945,0.0355412},
{0.0185490,-0.0794042,0.0431266},
{0.0193568,-0.0087326,0.0575704},
{0.0201026,-0.0144353,0.0531586},
{0.0025583,-0.0248211,0.0045714},
{0.0132689,-0.0214866,0.0652899},
{-0.0028669,-0.0099311,0.0309271},
{-0.0219487,-0.0054893,-0.0021853},
{0.0026180,-0.0203806,-0.0040620},
{-0.0116230,-0.0668518,0.0370152},
{-0.0133007,-0.0264159,0.0619176},
{0.0186167,0.0050491,-0.0120059},
{-0.0191359,-0.0591690,0.0288149},
{-0.0178121,-0.0041834,0.0141448},
{0.0123077,-0.0063648,0.0279743},
{-0.0157729,-0.0374010,0.0315335},
{0.0149328,-0.0659100,0.0290095},
{0.0006205,0.0021977,-0.0214613},
{0.0170484,-0.0715687,0.0325980},
{0.0068499,0.0061192,0.0196608},
{-0.0167075,0.0020024,0.0150382},

Biology Open • Supplementary information

{0.0059801,-0.0393117,0.0167489},
{-0.0072506,-0.0171067,0.0338751},
{0.0202363,-0.0251596,0.0420997},
{-0.0199311,-0.0601255,0.0171522},
{0.0216641,0.0053410,-0.0027841},
{-0.0197665,-0.0606125,0.0252972},
{0.0129986,-0.0214528,0.0098002},
{0.0201151,-0.0100929,0.0460501},
{0.0104137,0.0017765,-0.0203073},
{0.0166557,-0.0064123,0.0140197},
{-0.0124120,-0.0596549,0.0422637},
{0.0014885,0.0202590,0.0096781},
{0.0028720,0.0020207,-0.0219044},
{0.0376095,-0.3675427,0.0214537},
{0.0049949,0.0206417,-0.0079017},
{-0.0191723,-0.0407590,0.0391310},
{-0.0316406,-0.3638215,-0.0274841},
{0.0021392,0.0226920,0.0000985},
{0.0168435,-0.0229382,0.0264788},
{-0.0157304,-0.0005437,-0.0161456},
{0.0203722,-0.0299750,0.0562762},
{-0.0171077,0.0134146,0.0059615},
{0.0175519,-0.0370653,0.0413374},
{0.0154657,-0.0405127,0.0348980},
{-0.0215172,-0.0323502,0.0481925},
{0.0177998,-0.0515776,0.0444709},
{-0.0183804,-0.0448316,0.0275139},
{0.0033607,-0.0502028,0.0575894},
{0.0010325,-0.0479377,0.0191141},
{0.0224852,-0.0181821,0.0451780},
{0.0084089,-0.0720538,0.0521031},
{-0.0070880,-0.0772912,0.0288023},
{-0.0223712,-0.0028990,0.0024047},
{0.0173136,-0.0120017,0.0204612},
{0.0137772,-0.0623537,0.0276621},
{-0.0100390,-0.0231180,0.0292222},
{-0.0196395,-0.3730207,0.0364730},
{-0.0094445,-0.0526688,0.0528111},
{-0.0115309,-0.3679776,-0.0104995},
{0.0129812,-0.0410477,0.0224072},
{0.0117394,0.0154849,-0.0117191},
{-0.0245208,-0.3649412,0.0295111},
{0.0147476,-0.0502966,0.0364899},
{0.0006132,-0.0446771,0.0600524},
{-0.0118123,0.0191139,0.0031977},
{-0.0017185,-0.0163303,0.0664216},

Biology Open • Supplementary information

{-0.0085098,-0.0074564,0.0247745},
{0.0197459,-0.0086852,0.0430460},
{0.0187064,-0.0223532,0.0600966},
{-0.0206552,-0.0099712,0.0525024},
{0.0207525,0.0084646,-0.0024846},
{-0.0094915,-0.0325446,0.0184441},
{-0.0014464,-0.0140050,-0.0179819},
{-0.0122725,-0.0573444,0.0463125},
{-0.0106397,0.0177211,0.0094669},
{0.0111981,0.0191236,-0.0041491},
{-0.0162790,-0.0256279,0.0592687},
{-0.0060120,-0.3653959,0.0413411},
{-0.0135007,-0.3703638,-0.0387476},
{-0.0134258,-0.3703142,-0.0379467},
{-0.0133509,-0.3702645,-0.0371458},
{-0.0132760,-0.3702148,-0.0363450},
{-0.0132011,-0.3701651,-0.0355441},
{-0.0131262,-0.3701154,-0.0347432},
{-0.0130513,-0.3700658,-0.0339423},
{-0.0129765,-0.3700161,-0.0331414},
{-0.0129016,-0.3699664,-0.0323405},
{-0.0128267,-0.3699167,-0.0315396},
{-0.0127518,-0.3698670,-0.0307388},
{-0.0126769,-0.3698174,-0.0299379},
{-0.0126020,-0.3697677,-0.0291370},
{-0.0125271,-0.3697180,-0.0283361},
{-0.0124523,-0.3696683,-0.0275352},
{-0.0123774,-0.3696187,-0.0267343},
{-0.0123025,-0.3695690,-0.0259334},
{-0.0122276,-0.3695193,-0.0251325},
{-0.0121527,-0.3694696,-0.0243317},
{-0.0120778,-0.3694199,-0.0235308},
{-0.0120029,-0.3693703,-0.0227299},
{-0.0119280,-0.3693206,-0.0219290},
{-0.0118532,-0.3692709,-0.0211281},
{-0.0117783,-0.3692212,-0.0203272},
{-0.0117034,-0.3691715,-0.0195263},
{-0.0116285,-0.3691219,-0.0187255},
{-0.0115536,-0.3690722,-0.0179246},
{-0.0114787,-0.3690225,-0.0171237},
{-0.0114038,-0.3689728,-0.0163228},
{-0.0113289,-0.3689231,-0.0155219},
{-0.0112541,-0.3688735,-0.0147210},
{-0.0111792,-0.3688238,-0.0139201},
{-0.0111043,-0.3687741,-0.0131192},
{-0.0110294,-0.3687244,-0.0123184},

Biology Open • Supplementary information

{-0.0109545,-0.3686748,-0.0115175},
{-0.0108796,-0.3686251,-0.0107166},
{-0.0108047,-0.3685754,-0.0099157},
{-0.0107298,-0.3685257,-0.0091148},
{-0.0106550,-0.3684760,-0.0083139},
{-0.0105801,-0.3684264,-0.0075130},
{-0.0105052,-0.3683767,-0.0067121},
{-0.0104303,-0.3683270,-0.0059113},
{-0.0103554,-0.3682773,-0.0051104},
{-0.0102805,-0.3682276,-0.0043095},
{-0.0102056,-0.3681780,-0.0035086},
{-0.0101307,-0.3681283,-0.0027077},
{-0.0100559,-0.3680786,-0.0019068},
{-0.0099810,-0.3680289,-0.0011059},
{-0.0099061,-0.3679792,-0.0003051},
{-0.0098312,-0.3679296,0.0004958},
{-0.0097563,-0.3678799,0.0012967},
{-0.0096814,-0.3678302,0.0020976},
{-0.0096065,-0.3677805,0.0028985},
{-0.0095317,-0.3677308,0.0036994},
{-0.0094568,-0.3676812,0.0045003},
{-0.0093819,-0.3676315,0.0053012},
{-0.0093070,-0.3675818,0.0061020},
{-0.0092321,-0.3675321,0.0069029},
{-0.0091572,-0.3674825,0.0077038},
{-0.0090823,-0.3674328,0.0085047},
{-0.0090074,-0.3673831,0.0093056},
{-0.0089326,-0.3673334,0.0101065},
{-0.0088577,-0.3672837,0.0109074},
{-0.0087828,-0.3672341,0.0117082},
{-0.0087079,-0.3671844,0.0125091},
{-0.0086330,-0.3671347,0.0133100},
{-0.0085581,-0.3670850,0.0141109},
{-0.0084832,-0.3670353,0.0149118},
{-0.0084083,-0.3669857,0.0157127},
{-0.0083335,-0.3669360,0.0165136},
{-0.0082586,-0.3668863,0.0173145},
{-0.0081837,-0.3668366,0.0181153},
{-0.0081088,-0.3667869,0.0189162},
{-0.0080339,-0.3667373,0.0197171},
{-0.0079590,-0.3666876,0.0205180},
{-0.0078841,-0.3666379,0.0213189},
{-0.0078092,-0.3665882,0.0221198},
{-0.0077344,-0.3665386,0.0229207},
{-0.0076595,-0.3664889,0.0237215},
{-0.0075846,-0.3664392,0.0245224},

Biology Open • Supplementary information

{-0.0075097,-0.3663895,0.0253233},
{-0.0074348,-0.3663398,0.0261242},
{-0.0073599,-0.3662902,0.0269251},
{-0.0072850,-0.3662405,0.0277260},
{-0.0072101,-0.3661908,0.0285269},
{-0.0071353,-0.3661411,0.0293278},
{-0.0070604,-0.3660914,0.0301286},
{-0.0069855,-0.3660418,0.0309295},
{-0.0069106,-0.3659921,0.0317304},
{-0.0068357,-0.3659424,0.0325313},
{-0.0067608,-0.3658927,0.0333322},
{-0.0066859,-0.3658430,0.0341331},
{-0.0066111,-0.3657934,0.0349340},
{-0.0065362,-0.3657437,0.0357348},
{-0.0064613,-0.3656940,0.0365357},
{-0.0063864,-0.3656443,0.0373366},
{-0.0063115,-0.3655947,0.0381375},
{-0.0062366,-0.3655450,0.0389384},
{-0.0061617,-0.3654953,0.0397393},
{-0.0060868,-0.3654456,0.0405402}
}* .AMirroring);
Points1 = ({
{0.0000000,0.0000000,0.0000000},
{-0.0000000,-0.4260351,0.0000000},
{-0.0098081,-0.4333357,0.0013036},
{-0.0000000,-0.4311957,0.0410368},
{-0.0000000,-0.4208745,-0.0410368},
{0.0146010,-0.0095741,0.0588672},
{0.0211859,-0.0249003,0.0463312},
{0.0101821,-0.0282433,0.0642194},
{0.0001646,-0.0004404,0.0524005},
{0.0118244,-0.0075730,0.0408364},
{-0.0062334,-0.0155105,0.0666032},
{0.0177330,-0.0428706,0.0530675},
{0.0158880,-0.0372253,0.0341000},
{0.0150072,-0.0160286,0.0257986},
{-0.0106784,-0.0356007,0.0635500},
{0.0035375,-0.0466033,0.0625015},
{-0.0032386,-0.0223157,0.0423916},
{-0.0131463,-0.0047893,0.0493717},
{-0.0000093,-0.0046980,0.0286450},
{-0.0129090,-0.0254129,0.0507522},
{0.0106058,-0.0674492,0.0557533},
{0.0168668,-0.0599211,0.0397190},
{0.0087924,-0.0505968,0.0199162},
{0.0101682,-0.0269463,0.0129929},

Biology Open • Supplementary information

{0.0192677,-0.0061135,0.0059390},
{0.0109029,0.0067403,0.0165746},
{-0.0128420,-0.0423899,0.0377696},
{-0.0105497,-0.0551045,0.0536628},
{-0.0096849,-0.0283656,0.0259278},
{-0.0064301,0.0118653,0.0166049},
{-0.0119331,-0.0065277,0.0163684},
{-0.0078099,-0.0779286,0.0465371},
{0.0066090,-0.0894214,0.0540609},
{0.0179611,-0.0838235,0.0383149},
{0.0087171,-0.0721856,0.0218839},
{-0.0123392,-0.0512545,0.0215070},
{-0.0044033,-0.0364328,0.0106265},
{-0.0004658,-0.0221125,-0.0050027},
{0.0134184,-0.0173307,-0.0067924},
{0.0181309,-0.0019923,-0.0116001},
{0.0177776,0.0144345,-0.0012628},
{0.0032858,0.0219052,0.0093805},
{-0.0149201,-0.0636250,0.0352907},
{-0.0123959,-0.0139031,0.0060405},
{-0.0126878,0.0188868,0.0016752},
{-0.0193552,0.0026183,0.0033051},
{-0.0071211,-0.0863171,0.0288998},
{-0.0045062,-0.1034954,0.0438209},
{0.0152900,-0.1086947,0.0467099},
{0.0130520,-0.0928686,0.0226912},
{-0.0105949,-0.0689400,0.0223391},
{-0.0152032,-0.0098392,-0.0106561},
{0.0006884,-0.0101613,-0.0194749},
{0.0062535,0.0086417,-0.0179260},
{0.0017596,0.0223535,-0.0084692},
{-0.0110061,0.0093520,-0.0151538},
{0.0004634,-0.1088815,0.0243038},
{-0.0019465,-0.1237875,0.0417851},
{0.0164663,-0.1310642,0.0445640},
{0.0196408,-0.1126733,0.0274539},
{0.0065308,-0.1279282,0.0201592},
{0.0000144,-0.1445420,0.0365896},
{0.0176249,-0.1534720,0.0422624},
{0.0234409,-0.1367601,0.0269520},
{0.0101602,-0.1489107,0.0172232},
{0.0024451,-0.1659436,0.0335992},
{0.0200551,-0.1761262,0.0394229},
{0.0258861,-0.1600378,0.0242282},
{0.0119994,-0.1715856,0.0146961},
{0.0040210,-0.1873208,0.0302349},

Biology Open • Supplementary information

{0.0203710,-0.1990333,0.0370070},
{0.0275117,-0.1812035,0.0219519},
{0.0134430,-0.1936617,0.0124358},
{0.0027640,-0.2093905,0.0223754},
{0.0146601,-0.2212103,0.0330203},
{0.0288672,-0.2189110,0.0251354},
{0.0278760,-0.2016748,0.0175983},
{0.0167976,-0.2187111,0.0092539},
{0.0024610,-0.2318514,0.0191054},
{0.0116118,-0.2481795,0.0288252},
{0.0259222,-0.2398727,0.0297555},
{0.0281415,-0.2375008,0.0135949},
{0.0148351,-0.2449575,0.0071736},
{0.0020780,-0.2583914,0.0162034},
{0.0127165,-0.2731733,0.0276467},
{0.0267599,-0.2613012,0.0265741},
{0.0280029,-0.2625039,0.0103893},
{0.0133488,-0.2710954,0.0046827},
{0.0030209,-0.2834310,0.0154402},
{0.0102788,-0.2983712,0.0252961},
{0.0271985,-0.2859992,0.0234596},
{0.0271207,-0.2850742,0.0063508},
{0.0104853,-0.2976425,0.0025117},
{0.0026930,-0.3139776,0.0110695},
{0.0114673,-0.3220440,0.0250022},
{0.0267615,-0.3103578,0.0216235},
{0.0273377,-0.3052710,0.0041551},
{0.0159789,-0.3193798,-0.0029159},
{0.0040474,-0.3370965,0.0007874},
{0.0026237,-0.3396778,0.0188668},
{0.0228206,-0.3391400,0.0224453},
{0.0298676,-0.3298093,0.0084956},
{0.0236607,-0.3435751,-0.0049992},
{0.0066696,-0.3565606,-0.0068982},
{-0.0001734,-0.3609418,0.0094100},
{0.0122335,-0.3587002,0.0243859},
{0.0299474,-0.3569555,0.0114822},
{0.0234301,-0.3660107,-0.0084693},
{0.0047141,-0.3787029,-0.0124538},
{-0.0022470,-0.3845820,0.0043570},
{0.0016930,-0.3772613,0.0218995},
{0.0230810,-0.3765721,0.0216164},
{0.0293909,-0.3802408,0.0051713},
{0.0222817,-0.3882671,-0.0133063},
{0.0100992,-0.4025680,-0.0245857},
{-0.0053290,-0.3976372,-0.0138435},

Biology Open • Supplementary information

{-0.0064773,-0.4107793,0.0028828},
{-0.0059661,-0.4014141,0.0204015},
{0.0105687,-0.3964874,0.0265576},
{0.0284205,-0.3988755,0.0181264},
{0.0271846,-0.4042461,-0.0001495},
{0.0258384,-0.4130930,-0.0182806},
{0.0179898,-0.4283077,-0.0304445},
{0.0043378,-0.4174702,-0.0383945},
{-0.0095715,-0.4077373,-0.0296597},
{-0.0165399,-0.4173272,-0.0127338},
{-0.0061219,-0.4346700,-0.0033237},
{-0.0177460,-0.4255480,0.0110693},
{-0.0154124,-0.4178040,0.0301224},
{0.0021442,-0.4132192,0.0352624},
{0.0211808,-0.4180025,0.0281320},
{0.0358476,-0.4191256,0.0160201},
{0.0277191,-0.4261093,-0.0018705},
{0.0300087,-0.4407249,-0.0164590},
{0.0176260,-0.4541262,-0.0278987},
{0.0025739,-0.4405564,-0.0399244},
{-0.0137462,-0.4268826,-0.0404245},
{-0.0279120,-0.4202221,-0.0286469},
{-0.0276907,-0.4357894,-0.0121470},
{-0.0176769,-0.4546039,-0.0178866},
{0.0001580,-0.4543115,-0.0118676},
{0.0030422,-0.4517180,0.0075954},
{-0.0144878,-0.4488946,0.0141048},
{-0.0249020,-0.4359790,0.0286647},
{-0.0045042,-0.4331474,0.0406779},
{0.0133889,-0.4372788,0.0335981},
{0.0326595,-0.4385913,0.0246972},
{0.0272892,-0.4436105,0.0072825},
{0.0168876,-0.4502999,-0.0065695},
{-0.0001461,-0.4606664,-0.0304627},
{-0.0145767,-0.4516354,-0.0386175},
{-0.0302919,-0.4418217,-0.0323041},
{0.0197523,-0.4552456,0.0211972},
{0.0015809,-0.4579820,0.0278047},
{-0.0147420,-0.4532484,0.0338700},
{-0.0008505,-0.0741081,0.0195154},
{-0.0101957,-0.0645127,0.0211629},
{0.0097188,-0.0378441,0.0633341},
{-0.0140232,-0.0153774,0.0583777},
{-0.0017190,-0.0630383,0.0586887},
{-0.0062203,-0.0707904,0.0230256},
{-0.0013932,-0.0909598,0.0210262},

{-0.0069073,0.0018793,-0.0174229},
{-0.0123140,-0.0494754,0.0421717},
{0.0032652,-0.0294912,0.0671496},
{-0.0100401,-0.0731283,0.0263363},
{-0.0010544,-0.0601032,0.0188056},
{-0.0130201,-0.0188748,0.0520348},
{0.0134536,-0.0333784,0.0616140},
{-0.0103419,-0.0331525,0.0406684},
{0.0141219,-0.0381516,0.0598890},
{-0.0008805,-0.0830286,0.0550087},
{-0.0146446,-0.0486073,0.0320193},
{-0.0137983,-0.0616369,0.0225249},
{-0.0160682,-0.0602851,0.0238266},
{-0.0024512,-0.0458141,0.0139036},
{0.0107032,-0.0642233,0.0244688},
{0.0214428,-0.0146894,0.0405212},
{-0.0030880,-0.0488043,0.0634264},
{0.0134064,-0.0031106,0.0176305},
{-0.0087596,-0.0539198,0.0193623},
{-0.0059051,-0.0896364,0.0454125},
{0.0055232,0.0163693,0.0134309},
{-0.0111952,0.0188336,-0.0061570},
{-0.0069921,-0.0744679,0.0486092},
{0.0077671,-0.0936623,0.0198566},
{0.0128092,-0.0821627,0.0248567},
{0.0076747,-0.0521586,0.0601487},
{0.0143067,-0.0139409,0.0077633},
{-0.0057257,-0.0259946,0.0046822},
{-0.0071262,-0.0010722,0.0205354},
{-0.0100117,-0.0620832,0.0500186},
{-0.0061409,-0.0288847,0.0672136},
{0.0083405,-0.0070136,0.0613881},
{0.0134907,-0.0557568,0.0302855},
{-0.0019900,-0.0200044,-0.0123204},
{-0.0136674,-0.0269782,0.0558106},
{0.0158814,0.0146777,-0.0071703},
{-0.0040235,0.0237852,0.0037356},
{0.0006260,-0.0938003,0.0533542},
{-0.0128793,-0.0436085,0.0505282},
{0.0196908,-0.0308369,0.0429052},
{0.0114863,-0.0473354,0.0243913},
{0.0176729,0.0114571,0.0073001},
{0.0203674,0.0022946,0.0068187},
{0.0213204,-0.0021838,-0.0034513},
{0.0136572,-0.0221776,0.0227541},
{0.0113364,-0.0381748,0.0217249},

Biology Open • Supplementary information

{0.0160890,-0.0161845,0.0325506},
{-0.0172052,-0.0539952,0.0267577},
{0.0029771,-0.0422093,0.0651577},
{0.0166452,-0.0497157,0.0387763},
{0.0143874,-0.0111497,0.0133920},
{0.0010161,0.0048352,-0.0195979},
{0.0085866,-0.0008400,-0.0194489},
{0.0102841,-0.0741928,0.0541815},
{0.0146877,-0.0669979,0.0514703},
{0.0172516,-0.0650947,0.0427981},
{0.0174489,-0.0753575,0.0361938},
{-0.0046468,-0.0822202,0.0247699},
{-0.0130549,0.0041052,0.0140834},
{0.0075964,-0.0012335,0.0556140},
{0.0189005,-0.0102771,0.0506936},
{0.0147358,-0.0259752,0.0606583},
{-0.0117191,-0.0392708,0.0255251},
{-0.0180639,-0.0082243,-0.0025735},
{0.0160722,-0.0907942,0.0275894},
{-0.0068606,-0.0063004,-0.0191431},
{0.0040452,0.0154634,-0.0149142},
{-0.0091850,-0.0362442,0.0161037},
{-0.0146198,0.0113094,-0.0104787},
{-0.0198427,0.0054388,-0.0016724},
{-0.0089685,-0.0768246,0.0398016},
{0.0114857,-0.0190909,-0.0009548},
{0.0123616,-0.0497875,0.0577517},
{0.0085813,-0.0202416,-0.0090466},
{0.0099448,-0.0152443,-0.0138654},
{0.0042584,-0.0650652,0.0190711},
{0.0088665,-0.0555041,0.0216078},
{0.0012118,-0.0832089,0.0202021},
{-0.0180385,-0.0613924,0.0303920},
{-0.0059968,-0.0014882,0.0525102},
{-0.0130032,-0.0336891,0.0598954},
{0.0178068,-0.0189833,0.0544852},
{0.0148980,-0.0841928,0.0485904},
{0.0186638,-0.0262602,0.0353172},
{0.0083031,-0.0348408,0.0669876},
{-0.0169108,-0.0035548,0.0100003},
{0.0143404,-0.0176564,0.0606269},
{0.0154017,-0.0670244,0.0328450},
{-0.0151280,-0.0548629,0.0226914},
{-0.0139876,-0.0365088,0.0530051},
{0.0138309,-0.0455574,0.0307968},
{-0.0077837,-0.0423889,0.0648563},

Biology Open • Supplementary information

{0.0066097,-0.0084729,-0.0189933},
{0.0041214,-0.0168827,-0.0152740},
{-0.0143451,-0.0118045,0.0518841},
{-0.0104054,-0.0489999,0.0583947},
{0.0173861,-0.0591715,0.0476443},
{-0.0168352,0.0122253,0.0015811},
{-0.0137719,0.0100406,0.0117467},
{-0.0018993,-0.0744544,0.0552640},
{0.0132477,-0.0101691,0.0343908},
{0.0138496,-0.0038366,0.0555923},
{0.0152310,-0.0062889,0.0435655},
{-0.0127629,-0.0147341,-0.0086789},
{0.0057201,-0.0240487,0.0023746},
{0.0003685,-0.0537137,0.0172028},
{0.0200045,0.0034291,-0.0076676},
{0.0171465,0.0017792,-0.0132952},
{0.0127878,-0.0084507,-0.0159459},
{-0.0131241,0.0015038,-0.0156011},
{-0.0146545,-0.0065488,-0.0133683},
{0.0176895,0.0144973,0.0032382},
{0.0105699,0.0209040,-0.0023178},
{0.0090222,0.0152073,-0.0133606},
{0.0080621,-0.0247571,0.0651943},
{-0.0007109,-0.0094570,0.0643011},
{-0.0001658,-0.0022918,0.0579495},
{0.0053834,-0.0150331,0.0657801},
{0.0084060,0.0080541,0.0167315},
{0.0123377,-0.0190707,0.0158660},
{0.0039435,-0.0277060,0.0668890},
{-0.0091365,-0.0189373,0.0247086},
{-0.0102402,-0.0191396,0.0123343},
{-0.0064904,-0.0109612,0.0275903},
{-0.0151092,-0.0555911,0.0360941},
{-0.0113841,-0.0672036,0.0417783},
{-0.0069486,-0.0318976,0.0095507},
{0.0207692,-0.0202843,0.0471488},
{0.0122727,-0.0092796,0.0282368},
{0.0043368,-0.0010375,0.0247384},
{-0.0035961,-0.0833760,0.0537891},
{-0.0024131,0.0240586,-0.0033334},
{-0.0011469,-0.0144989,0.0398863},
{-0.0109735,-0.0157570,0.0025114},
{0.0130079,-0.0316219,0.0261429},
{0.0103649,-0.0298242,0.0174680},
{-0.0110442,-0.0076321,0.0536917},
{0.0188037,-0.0363898,0.0489068},

{-0.0121187,-0.0588083,0.0423637},
{-0.0074999,-0.0888850,0.0384067},
{0.0043498,-0.0057668,0.0314548},
{-0.0030001,-0.0050617,-0.0203555},
{-0.0126623,-0.0381906,0.0594460},
{-0.0030217,0.0097835,-0.0172079},
{-0.0043389,0.0182402,-0.0127080},
{0.0186853,-0.0943518,0.0370267},
{0.0037077,-0.0813678,0.0194045},
{0.0165284,-0.0973488,0.0455178},
{0.0096267,0.0211063,0.0054858},
{0.0117920,0.0100617,0.0148603},
{0.0011031,0.0165780,-0.0145832},
{0.0161422,-0.0128558,-0.0090200},
{0.0190099,-0.0084089,-0.0077352},
{-0.0087346,-0.0267964,0.0310198},
{-0.0109470,-0.0381053,0.0327892},
{-0.0134555,-0.0680611,0.0262295},
{-0.0014213,0.0029363,0.0204860},
{0.0041698,0.0080793,0.0176737},
{-0.0025253,-0.0031478,0.0261714},
{0.0002577,-0.0340091,0.0663817},
{0.0069253,-0.0479422,0.0610919},
{-0.0117287,-0.0219209,0.0639958},
{-0.0117016,-0.0290702,0.0632111},
{0.0090599,0.0223685,0.0027007},
{0.0171376,0.0156242,0.0014330},
{-0.0071403,-0.0120540,0.0651568},
{0.0152564,-0.0587124,0.0350561},
{-0.0124338,-0.0525350,0.0490064},
{-0.0150384,-0.0557130,0.0224477},
{0.0109779,-0.0931255,0.0516131},
{-0.0098147,-0.0278826,0.0180591},
{-0.0101164,-0.0169792,0.0173639},
{-0.0044327,0.0176680,0.0128863},
{0.0008956,-0.0752826,0.0570597},
{0.0058957,-0.0694070,0.0572351},
{-0.0022611,-0.0690629,0.0567201},
{0.0165920,-0.0813832,0.0316812},
{-0.0124692,-0.0202059,0.0499270},
{0.0106806,-0.0274539,0.0175787},
{0.0051591,-0.0579811,0.0597532},
{0.0083124,-0.0387496,0.0635286},
{0.0095481,-0.0930718,0.0202423},
{-0.0067842,-0.0584005,0.0580163},
{0.0179416,-0.0472521,0.0448404},

{-0.0119284,-0.0472327,0.0215667},
{0.0212043,-0.0227507,0.0381461},
{-0.0040318,-0.0672421,0.0206887},
{0.0166592,-0.0021959,0.0130134},
{0.0061764,0.0059577,0.0178259},
{0.0009165,-0.0357544,0.0662616},
{-0.0148318,0.0151219,-0.0051698},
{0.0106136,0.0172730,0.0103526},
{-0.0106773,0.0176239,0.0083069},
{-0.0186194,-0.0060483,-0.0052598},
{-0.0187303,0.0005594,-0.0075449},
{-0.0049037,-0.0192430,0.0676053},
{-0.0131568,-0.0377586,0.0443264},
{0.0055901,-0.0515212,0.0610631},
{0.0124419,-0.0457572,0.0587780},
{-0.0087462,-0.0750584,0.0258513},
{0.0126392,0.0077884,0.0153033},
{0.0182435,-0.0117500,-0.0011424},
{-0.0173344,-0.0088283,0.0050922},
{-0.0127829,-0.0617579,0.0392799},
{-0.0073173,-0.0184845,-0.0037804},
{-0.0129437,-0.0302275,0.0475121},
{0.0046558,-0.0821253,0.0554826},
{0.0159841,-0.0547427,0.0523062},
{-0.0017364,-0.0382178,0.0111222},
{0.0062515,-0.0350960,0.0132571},
{0.0187856,-0.0076404,0.0058298},
{0.0181390,-0.0892677,0.0420584},
{-0.0091390,-0.0207491,0.0253212},
{-0.0008794,0.0142003,0.0162053},
{0.0074091,-0.0747980,0.0213438},
{0.0088001,-0.0683517,0.0565856},
{0.0150310,-0.0142059,-0.0094991},
{0.0005563,-0.0099727,0.0647803},
{-0.0071553,-0.0161555,-0.0142424},
{0.0159059,-0.0772208,0.0472464},
{0.0076936,-0.0201712,-0.0103955},
{-0.0106216,-0.0706050,0.0233390},
{0.0133203,-0.0430590,0.0291645},
{0.0155262,-0.0046839,0.0541312},
{0.0047469,-0.0083636,0.0394578},
{-0.0030379,-0.0676160,0.0572801},
{-0.0128107,-0.0651717,0.0382525},
{-0.0144619,-0.0125049,0.0541672},
{-0.0078637,0.0134268,-0.0146852},
{-0.0171780,0.0063314,0.0081686},

Biology Open • Supplementary information

{0.0093526,-0.0745041,0.0222516},
{0.0024951,-0.0425329,0.0642430},
{-0.0135095,-0.0237088,0.0551025},
{0.0064427,-0.0067057,-0.0196962},
{0.0154119,-0.0308805,0.0566410},
{0.0119687,-0.0163625,0.0621528},
{0.0104243,0.0022594,-0.0181417},
{0.0137128,-0.0088439,0.0172285},
{-0.0129452,0.0120600,0.0117175},
{0.0103630,-0.0572188,0.0576950},
{-0.0054678,-0.0415522,0.0656820},
{0.0194496,-0.0314419,0.0501629},
{0.0158525,-0.0143119,-0.0070263},
{-0.0149265,-0.0542918,0.0365964},
{-0.0075540,-0.0797169,0.0453720},
{0.0032005,-0.0568892,0.0604941},
{-0.0044159,-0.0447634,0.0140694},
{0.0111401,0.0115522,-0.0146686},
{-0.0021439,-0.0233462,-0.0008475},
{-0.0091704,-0.0791858,0.0303455},
{-0.0103319,-0.0037825,-0.0176411},
{-0.0029171,0.0234052,-0.0046929},
{-0.0095908,-0.0234480,0.0133214},
{-0.0107450,-0.0695604,0.0231607},
{-0.0059490,-0.0806769,0.0249357},
{0.0009535,0.0127984,-0.0167812},
{0.0198530,-0.0280509,0.0493913},
{-0.0058979,-0.0626016,0.0200093},
{0.0176390,-0.0391839,0.0399166},
{0.0176135,-0.0332152,0.0355635},
{-0.0143673,-0.0129049,-0.0009856},
{0.0132790,0.0151206,0.0102064},
{-0.0133956,-0.0117695,0.0070100},
{-0.0107050,-0.0379612,0.0361861},
{0.0179713,-0.0946177,0.0429545},
{0.0176946,-0.0111412,0.0563859},
{0.0187814,-0.0167753,0.0522373},
{0.0013630,-0.0285419,0.0050847},
{0.0108016,-0.0251690,0.0636150},
{-0.0027924,-0.0102777,0.0317929},
{-0.0192987,-0.0045710,-0.0017603},
{0.0027607,-0.0226297,-0.0038575},
{-0.0096950,-0.0765823,0.0374183},
{-0.0127523,-0.0295814,0.0616836},
{0.0179110,0.0055852,-0.0108969},
{-0.0147865,-0.0670016,0.0305003},

Biology Open • Supplementary information

{-0.0152489,-0.0045181,0.0129514},
{0.0120076,-0.0086463,0.0273876},
{-0.0134453,-0.0460897,0.0324397},
{0.0150640,-0.0769723,0.0291432},
{0.0007702,0.0036234,-0.0198537},
{0.0169002,-0.0833279,0.0317986},
{0.0069496,0.0068603,0.0170804},
{-0.0143007,0.0024293,0.0131144},
{0.0058567,-0.0461345,0.0168121},
{-0.0064822,-0.0204487,0.0344565},
{0.0205731,-0.0289703,0.0435241},
{-0.0129203,-0.0672663,0.0232885},
{0.0208788,0.0054951,-0.0030845},
{-0.0138919,-0.0674444,0.0283763},
{0.0112783,-0.0240345,0.0113129},
{0.0197627,-0.0125623,0.0464317},
{0.0086730,0.0020901,-0.0191008},
{0.0147724,-0.0089213,0.0138462},
{-0.0113565,-0.0682676,0.0411192},
{0.0004639,0.0221175,0.0094276},
{0.0028461,0.0035481,-0.0201791},
{0.0377999,-0.4329396,0.0215635},
{0.0051227,0.0219159,-0.0079895},
{-0.0136876,-0.0483234,0.0382956},
{-0.0318095,-0.4285551,-0.0276310},
{0.0023480,0.0247939,-0.0000328},
{0.0145629,-0.0263425,0.0273079},
{-0.0143046,-0.0011812,-0.0149230},
{0.0167744,-0.0332640,0.0544341},
{-0.0148491,0.0144375,0.0048712},
{0.0174880,-0.0430328,0.0411366},
{0.0156074,-0.0480073,0.0352847},
{-0.0134490,-0.0378672,0.0470238},
{0.0175606,-0.0608054,0.0452570},
{-0.0169503,-0.0528391,0.0282430},
{0.0030970,-0.0603672,0.0597241},
{0.0011021,-0.0563145,0.0178364},
{0.0216970,-0.0229462,0.0452427},
{0.0080107,-0.0838514,0.0541378},
{-0.0060270,-0.0910346,0.0286964},
{-0.0192289,-0.0023625,0.0031368},
{0.0145752,-0.0147085,0.0202779},
{0.0140184,-0.0730861,0.0282154},
{-0.0094107,-0.0270948,0.0282586},
{-0.0197427,-0.4393912,0.0366655},
{-0.0087605,-0.0611913,0.0535802},

Biology Open • Supplementary information

{-0.0115920,-0.4334508,-0.0105552},
{0.0108039,-0.0474409,0.0229987},
{0.0109803,0.0159232,-0.0114931},
{-0.0246509,-0.4298741,0.0296677},
{0.0158259,-0.0582816,0.0359608},
{0.0003019,-0.0543464,0.0614576},
{-0.0111091,0.0198424,0.0029203},
{-0.0024956,-0.0183125,0.0674620},
{-0.0073777,-0.0080817,0.0247587},
{0.0195986,-0.0113015,0.0433364},
{0.0150089,-0.0268597,0.0587113},
{-0.0142915,-0.0119615,0.0534159},
{0.0200312,0.0090323,-0.0028811},
{-0.0101320,-0.0374173,0.0184003},
{-0.0003404,-0.0154887,-0.0168718},
{-0.0108903,-0.0660538,0.0453291},
{-0.0091004,0.0187801,0.0082302},
{0.0098650,0.0209930,-0.0030799},
{-0.0135372,-0.0296524,0.0584966},
{-0.0060438,-0.4304097,0.0415604},
{-0.0135723,-0.4362616,-0.0389532},
{-0.0134970,-0.4362030,-0.0381480},
{-0.0134217,-0.4361445,-0.0373429},
{-0.0133464,-0.4360860,-0.0365378},
{-0.0132712,-0.4360275,-0.0357326},
{-0.0131959,-0.4359690,-0.0349275},
{-0.0131206,-0.4359105,-0.0341224},
{-0.0130453,-0.4358519,-0.0333172},
{-0.0129700,-0.4357934,-0.0325121},
{-0.0128947,-0.4357349,-0.0317070},
{-0.0128194,-0.4356764,-0.0309018},
{-0.0127442,-0.4356179,-0.0300967},
{-0.0126689,-0.4355593,-0.0292915},
{-0.0125936,-0.4355008,-0.0284864},
{-0.0125183,-0.4354423,-0.0276813},
{-0.0124430,-0.4353838,-0.0268761},
{-0.0123677,-0.4353253,-0.0260710},
{-0.0122925,-0.4352668,-0.0252659},
{-0.0122172,-0.4352082,-0.0244607},
{-0.0121419,-0.4351497,-0.0236556},
{-0.0120666,-0.4350912,-0.0228505},
{-0.0119913,-0.4350327,-0.0220453},
{-0.0119160,-0.4349742,-0.0212402},
{-0.0118408,-0.4349156,-0.0204351},
{-0.0117655,-0.4348571,-0.0196299},
{-0.0116902,-0.4347986,-0.0188248},

Biology Open • Supplementary information

{-0.0116149,-0.4347401,-0.0180197},
{-0.0115396,-0.4346816,-0.0172145},
{-0.0114643,-0.4346231,-0.0164094},
{-0.0113890,-0.4345645,-0.0156042},
{-0.0113138,-0.4345060,-0.0147991},
{-0.0112385,-0.4344475,-0.0139940},
{-0.0111632,-0.4343890,-0.0131888},
{-0.0110879,-0.4343305,-0.0123837},
{-0.0110126,-0.4342719,-0.0115786},
{-0.0109373,-0.4342134,-0.0107734},
{-0.0108621,-0.4341549,-0.0099683},
{-0.0107868,-0.4340964,-0.0091632},
{-0.0107115,-0.4340379,-0.0083580},
{-0.0106362,-0.4339794,-0.0075529},
{-0.0105609,-0.4339208,-0.0067478},
{-0.0104856,-0.4338623,-0.0059426},
{-0.0104103,-0.4338038,-0.0051375},
{-0.0103351,-0.4337453,-0.0043324},
{-0.0102598,-0.4336868,-0.0035272},
{-0.0101845,-0.4336282,-0.0027221},
{-0.0101092,-0.4335697,-0.0019169},
{-0.0100339,-0.4335112,-0.0011118},
{-0.0099586,-0.4334527,-0.0003067},
{-0.0098834,-0.4333942,0.0004985},
{-0.0098081,-0.4333357,0.0013036},
{-0.0097328,-0.4332771,0.0021087},
{-0.0096575,-0.4332186,0.0029139},
{-0.0095822,-0.4331601,0.0037190},
{-0.0095069,-0.4331016,0.0045241},
{-0.0094316,-0.4330431,0.0053293},
{-0.0093564,-0.4329845,0.0061344},
{-0.0092811,-0.4329260,0.0069395},
{-0.0092058,-0.4328675,0.0077447},
{-0.0091305,-0.4328090,0.0085498},
{-0.0090552,-0.4327505,0.0093549},
{-0.0089799,-0.4326919,0.0101601},
{-0.0089047,-0.4326334,0.0109652},
{-0.0088294,-0.4325749,0.0117704},
{-0.0087541,-0.4325164,0.0125755},
{-0.0086788,-0.4324579,0.0133806},
{-0.0086035,-0.4323994,0.0141858},
{-0.0085282,-0.4323408,0.0149909},
{-0.0084530,-0.4322823,0.0157960},
{-0.0083777,-0.4322238,0.0166012},
{-0.0083024,-0.4321653,0.0174063},
{-0.0082271,-0.4321068,0.0182114},

Biology Open • Supplementary information

```
                    {-0.0081518,-0.4320482,0.0190166},
                    {-0.0080765,-0.4319897,0.0198217},
                    {-0.0080012,-0.4319312,0.0206268},
                    {-0.0079260,-0.4318727,0.0214320},
                    {-0.0078507,-0.4318142,0.0222371},
                    {-0.0077754,-0.4317557,0.0230423},
                    {-0.0077001,-0.4316971,0.0238474},
                    {-0.0076248,-0.4316386,0.0246525},
                    {-0.0075495,-0.4315801,0.0254577},
                    {-0.0074743,-0.4315216,0.0262628},
                    {-0.0073990,-0.4314631,0.0270679},
                    {-0.0073237,-0.4314045,0.0278731},
                    {-0.0072484,-0.4313460,0.0286782},
                    {-0.0071731,-0.4312875,0.0294833},
                    {-0.0070978,-0.4312290,0.0302885},
                    {-0.0070225,-0.4311705,0.0310936},
                    {-0.0069473,-0.4311120,0.0318987},
                    {-0.0068720,-0.4310534,0.0327039},
                    {-0.0067967,-0.4309949,0.0335090},
                    {-0.0067214,-0.4309364,0.0343141},
                    {-0.0066461,-0.4308779,0.0351193},
                    {-0.0065708,-0.4308194,0.0359244},
                    {-0.0064956,-0.4307608,0.0367296},
                    {-0.0064203,-0.4307023,0.0375347},
                    {-0.0063450,-0.4306438,0.0383398},
                    {-0.0062697,-0.4305853,0.0391450},
                    {-0.0061944,-0.4305268,0.0399501},
                    {-0.0061191,-0.4304683,0.0407552}
                    }* .AMirroring);
            BoundingBoxOnOff = Off;
        };
        AnyFunTransform3DIdentity ScaleFunction = {
            PreTransforms = {&.RBFTransform};
};
};

};
};
```

Biology Open • Supplementary information

ScalingFunctionTLEMLucyPelvis_2014031

```
AnyFolder ScalingFunctionTLEMLucyPelvis = {
AnyFolder Pelvis = {
	AnyFunTransform3DRBF RBFTransform = {
		RBFDef.Type = RBF_ThinPlate;
		PolynomDegree = 1;
		Points0 = {
			{0.0000000,0.0000000,0.1177000},
			{-0.0000000,-0.0832729,0.0191000},
			{-0.0508179,-0.0694062,0.0815920},
			{0.0000000,0.0000000,-0.1177000},
			{-0.0000000,-0.0832729,-0.0191000},
			{-0.0508179,-0.0694062,-0.0815920},
			{0.0000000,0.0000000,0.0000000},
			{-0.1164020,-0.0039449,0.0456760},
			{-0.1164020,-0.0039449,-0.0456760},
			{-0.1092909,-0.0992949,0.0515590},
			{-0.1092909,-0.0992949,-0.0515590},
			{-0.0733749,0.0757445,0.0915590},
			{-0.0733749,0.0757445,-0.0915590},
			{-0.1241738,0.0316243,0.0458050},
			{-0.1241738,0.0316243,-0.0458050},
			{-0.1108008,0.0563490,0.0555690},
			{-0.1108008,0.0563490,-0.0555690},
			{-0.0545356,0.0481653,0.1291690},
			{-0.0545356,0.0481653,-0.1291690},
			{-0.0360236,0.0456352,0.1316250},
			{-0.0360236,0.0456352,-0.1316250},
			{-0.0804664,-0.0228481,0.0672140},
			{-0.0804664,-0.0228481,-0.0672140},
			{-0.1004070,-0.0623213,0.0494990},
			{-0.1004070,-0.0623213,-0.0494990},
			{-0.0174901,-0.0357786,0.0977400},
			{-0.0174901,-0.0357786,-0.0977400},
			{-0.0665950,-0.1308418,0.0278190},
			{-0.0665950,-0.1308418,-0.0278190},
			{-0.0962739,-0.1257274,0.0551670},
			{-0.0962739,-0.1257274,-0.0551670},
			{-0.0393340,-0.1196098,0.0159820},
			{-0.0393340,-0.1196098,-0.0159820},
			{-0.0585979,-0.0148191,0.0651020},
			{-0.0585979,-0.0148191,-0.0651020},
			{-0.0238420,-0.0800479,0.0351680},
			{-0.0238420,-0.0800479,-0.0351680},
			{-0.0234553,-0.0576924,0.0715330},
```

Biology Open • Supplementary information

{-0.0234553,-0.0576924,-0.0715330},
{-0.0472319,-0.0833276,0.0513790},
{-0.0472319,-0.0833276,-0.0513790},
{-0.0327175,-0.0506481,0.0520240},
{-0.0327175,-0.0506481,-0.0520240},
{-0.0746024,-0.0627219,0.0928110},
{-0.0746024,-0.0627219,-0.0928110},
{-0.0119676,-0.0928080,0.0076900},
{-0.0119676,-0.0928080,-0.0076900},
{-0.0263299,-0.0760728,0.0667080},
{-0.0263299,-0.0760728,-0.0667080},
{-0.0498842,0.0119450,0.1070630},
{-0.0498842,0.0119450,-0.1070630},
{-0.0553874,0.0627474,0.1172390},
{-0.0553874,0.0627474,-0.1172390},
{-0.0786774,0.0073556,0.0741800},
{-0.0786774,0.0073556,-0.0741800},
{-0.0742762,0.0474272,0.0542330},
{-0.0742762,0.0474272,-0.0542330},
{-0.0634121,0.0117185,0.0618430},
{-0.0634121,0.0117185,-0.0618430},
{-0.0813084,0.0007821,0.0508130},
{-0.0813084,0.0007821,-0.0508130},
{-0.0496423,-0.0444458,0.0983920},
{-0.0496423,-0.0444458,-0.0983920},
{-0.0480005,-0.0338999,0.0625080},
{-0.0480005,-0.0338999,-0.0625080},
{-0.0428000,-0.0273188,0.0973490},
{-0.0428000,-0.0273188,-0.0973490},
{-0.0548383,-0.0518766,0.0624050},
{-0.0548383,-0.0518766,-0.0624050},
{-0.0696448,-0.1052827,0.0436200},
{-0.0696448,-0.1052827,-0.0436200},
{-0.0739608,-0.0803177,0.0785950},
{-0.0739608,-0.0803177,-0.0785950},
{-0.0586066,-0.0884764,0.0653190},
{-0.0586066,-0.0884764,-0.0653190},
{-0.1017003,0.0085215,0.0624150},
{-0.1017003,0.0085215,-0.0624150},
{-0.0126252,0.0252852,0.1292530},
{-0.0126252,0.0252852,-0.1292530},
{-0.0030963,0.0150156,0.1262640},
{-0.0030963,0.0150156,-0.1262640},
{-0.0621797,0.0048743,0.0605920},
{-0.0621797,0.0048743,-0.0605920},
{-0.0870082,-0.0684280,0.0489900},

{-0.0870082,-0.0684280,-0.0489900},
{-0.0916211,-0.0478344,0.0575290},
{-0.0916211,-0.0478344,-0.0575290},
{-0.0428573,-0.0467026,0.0985250},
{-0.0428573,-0.0467026,-0.0985250},
{-0.0483869,-0.0455474,0.0848580},
{-0.0483869,-0.0455474,-0.0848580},
{-0.0203929,-0.0681811,0.0426070},
{-0.0203929,-0.0681811,-0.0426070},
{-0.0271973,-0.0807786,0.0491650},
{-0.0271973,-0.0807786,-0.0491650},
{-0.0325935,-0.1026813,0.0148280},
{-0.0325935,-0.1026813,-0.0148280},
{-0.0273900,-0.1059173,0.0048750},
{-0.0273900,-0.1059173,-0.0048750},
{-0.0015971,-0.0778770,0.0065530},
{-0.0015971,-0.0778770,-0.0065530},
{-0.1034983,-0.0140026,0.0521800},
{-0.1034983,-0.0140026,-0.0521800},
{-0.1141160,0.0077734,0.0497600},
{-0.1141160,0.0077734,-0.0497600},
{-0.1123797,0.0244808,0.0350160},
{-0.1123797,0.0244808,-0.0350160},
{-0.0984470,-0.0648210,0.0457100},
{-0.0984470,-0.0648210,-0.0457100},
{-0.0814332,-0.0704479,0.0806410},
{-0.0814332,-0.0704479,-0.0806410},
{-0.0540218,-0.0805070,0.0547320},
{-0.0540218,-0.0805070,-0.0547320},
{-0.0674356,-0.0763171,0.0651080},
{-0.0674356,-0.0763171,-0.0651080},
{-0.0602364,-0.0751827,0.0490600},
{-0.0602364,-0.0751827,-0.0490600},
{-0.0604272,-0.0801096,0.0617610},
{-0.0604272,-0.0801096,-0.0617610},
{-0.0655251,0.0632155,0.1093750},
{-0.0655251,0.0632155,-0.1093750},
{-0.0665046,0.0014289,0.0572170},
{-0.0665046,0.0014289,-0.0572170},
{-0.1053786,0.0347803,0.0071580},
{-0.1053786,0.0347803,-0.0071580},
{-0.1321649,-0.0069725,0.0058340},
{-0.1321649,-0.0069725,-0.0058340},
{-0.1232735,-0.0434903,0.0301110},
{-0.1232735,-0.0434903,-0.0301110},
{-0.0497024,0.0261862,0.0084640},

```
{-0.0497024,0.0261862,-0.0084640},
{-0.0887827,0.0446048,0.0327030},
{-0.0887827,0.0446048,-0.0327030},
{-0.1153998,-0.0126010,0.0290730},
{-0.1153998,-0.0126010,-0.0290730},
{-0.0899939,0.0093882,0.0208440},
{-0.0899939,0.0093882,-0.0208440},
{-0.1312654,-0.0574898,0.0094690},
{-0.1312654,-0.0574898,-0.0094690},
{-0.0996029,0.0042806,0.0105620},
{-0.0996029,0.0042806,-0.0105620},
{-0.1226177,-0.0324069,0.0331630},
{-0.1226177,-0.0324069,-0.0331630},
{-0.1097808,-0.0051619,0.0102520},
{-0.1097808,-0.0051619,-0.0102520}
};
Points1 = {
{0.0000000,0.0000000,0.1146472},
{-0.0000007,-0.0841567,0.0166942},
{-0.0287487,-0.0653499,0.0797819},
{0.0000000,0.0000000,-0.1146472},
{-0.0000007,-0.0841567,-0.0166942},
{-0.0287487,-0.0653499,-0.0797819},
{0.0000000,0.0000000,0.0000000},
{-0.0784974,-0.0274825,0.0388349},
{-0.0784974,-0.0274825,-0.0388349},
{-0.0744033,-0.0924251,0.0567016},
{-0.0744033,-0.0924251,-0.0567016},
{-0.0510804,0.0386519,0.0836160},
{-0.0510804,0.0386519,-0.0836160},
{-0.0828703,-0.0068852,0.0344311},
{-0.0828703,-0.0068852,-0.0344311},
{-0.0752265,0.0144776,0.0457165},
{-0.0752265,0.0144776,-0.0457165},
{-0.0315348,0.0296673,0.1187151},
{-0.0315348,0.0296673,-0.1187151},
{-0.0205507,0.0299668,0.1226123},
{-0.0205507,0.0299668,-0.1226123},
{-0.0555552,-0.0372896,0.0629480},
{-0.0555552,-0.0372896,-0.0629480},
{-0.0694110,-0.0681665,0.0582299},
{-0.0694110,-0.0681665,-0.0582299},
{-0.0103438,-0.0365392,0.0860731},
{-0.0103438,-0.0365392,-0.0860731},
{-0.0480752,-0.1163946,0.0312929},
{-0.0480752,-0.1163946,-0.0312929},
```

Biology Open • Supplementary information

{-0.0650354,-0.1078995,0.0596306},
{-0.0650354,-0.1078995,-0.0596306},
{-0.0270475,-0.1115436,0.0174585},
{-0.0270475,-0.1115436,-0.0174585},
{-0.0432274,-0.0265759,0.0608230},
{-0.0432274,-0.0265759,-0.0608230},
{-0.0137436,-0.0767335,0.0315340},
{-0.0137436,-0.0767335,-0.0315340},
{-0.0144631,-0.0545433,0.0676070},
{-0.0144631,-0.0545433,-0.0676070},
{-0.0268521,-0.0742763,0.0518728},
{-0.0268521,-0.0742763,-0.0518728},
{-0.0224786,-0.0547544,0.0526130},
{-0.0224786,-0.0547544,-0.0526130},
{-0.0455798,-0.0612956,0.0839125},
{-0.0455798,-0.0612956,-0.0839125},
{-0.0065754,-0.0933634,0.0071677},
{-0.0065754,-0.0933634,-0.0071677},
{-0.0153687,-0.0657224,0.0615932},
{-0.0153687,-0.0657224,-0.0615932},
{-0.0332917,-0.0009231,0.0976437},
{-0.0332917,-0.0009231,-0.0976437},
{-0.0355334,0.0368942,0.1083098},
{-0.0355334,0.0368942,-0.1083098},
{-0.0564439,-0.0132508,0.0637942},
{-0.0564439,-0.0132508,-0.0637942},
{-0.0540551,0.0130165,0.0459726},
{-0.0540551,0.0130165,-0.0459726},
{-0.0490808,-0.0095136,0.0517264},
{-0.0490808,-0.0095136,-0.0517264},
{-0.0590838,-0.0214488,0.0429470},
{-0.0590838,-0.0214488,-0.0429470},
{-0.0286153,-0.0469561,0.0868049},
{-0.0286153,-0.0469561,-0.0868049},
{-0.0329709,-0.0404163,0.0620342},
{-0.0329709,-0.0404163,-0.0620342},
{-0.0266208,-0.0324563,0.0871445},
{-0.0266208,-0.0324563,-0.0871445},
{-0.0340631,-0.0543357,0.0635634},
{-0.0340631,-0.0543357,-0.0635634},
{-0.0460524,-0.0953474,0.0487290},
{-0.0460524,-0.0953474,-0.0487290},
{-0.0448550,-0.0749420,0.0763273},
{-0.0448550,-0.0749420,-0.0763273},
{-0.0342004,-0.0815297,0.0680470},
{-0.0342004,-0.0815297,-0.0680470},

Biology Open • Supplementary information

{-0.0699394,-0.0160378,0.0528982},
{-0.0699394,-0.0160378,-0.0528982},
{-0.0068432,0.0176617,0.1229247},
{-0.0068432,0.0176617,-0.1229247},
{-0.0012296,0.0111359,0.1214124},
{-0.0012296,0.0111359,-0.1214124},
{-0.0488395,-0.0142692,0.0504590},
{-0.0488395,-0.0142692,-0.0504590},
{-0.0583873,-0.0706497,0.0578424},
{-0.0583873,-0.0706497,-0.0578424},
{-0.0619573,-0.0565203,0.0604806},
{-0.0619573,-0.0565203,-0.0604806},
{-0.0236134,-0.0487029,0.0863855},
{-0.0236134,-0.0487029,-0.0863855},
{-0.0289114,-0.0480035,0.0776342},
{-0.0289114,-0.0480035,-0.0776342},
{-0.0122006,-0.0679494,0.0382063},
{-0.0122006,-0.0679494,-0.0382063},
{-0.0155387,-0.0741466,0.0452690},
{-0.0155387,-0.0741466,-0.0452690},
{-0.0194459,-0.0986350,0.0159059},
{-0.0194459,-0.0986350,-0.0159059},
{-0.0144349,-0.1017428,0.0062136},
{-0.0144349,-0.1017428,-0.0062136},
{-0.0016710,-0.0829660,0.0057660},
{-0.0016710,-0.0829660,-0.0057660},
{-0.0702038,-0.0328837,0.0482627},
{-0.0702038,-0.0328837,-0.0482627},
{-0.0772852,-0.0181292,0.0409158},
{-0.0772852,-0.0181292,-0.0409158},
{-0.0758759,-0.0108913,0.0263065},
{-0.0758759,-0.0108913,-0.0263065},
{-0.0684427,-0.0699242,0.0570009},
{-0.0684427,-0.0699242,-0.0570009},
{-0.0508078,-0.0684821,0.0776849},
{-0.0508078,-0.0684821,-0.0776849},
{-0.0304044,-0.0714368,0.0563291},
{-0.0304044,-0.0714368,-0.0563291},
{-0.0404078,-0.0730508,0.0679410},
{-0.0404078,-0.0730508,-0.0679410},
{-0.0361396,-0.0711300,0.0530943},
{-0.0361396,-0.0711300,-0.0530943},
{-0.0350093,-0.0755673,0.0655383},
{-0.0350093,-0.0755673,-0.0655383},
{-0.0440093,0.0337575,0.1006149},
{-0.0440093,0.0337575,-0.1006149},

Biology Open • Supplementary information

```
                    {-0.0519337,-0.0178552,0.0468714},
                    {-0.0519337,-0.0178552,-0.0468714},
                    {-0.0718288,-0.0056450,0.0052866},
                    {-0.0718288,-0.0056450,-0.0052866},
                    {-0.0877872,-0.0356376,0.0042068},
                    {-0.0877872,-0.0356376,-0.0042068},
                    {-0.0869194,-0.0616983,0.0197486},
                    {-0.0869194,-0.0616983,-0.0197486},
                    {-0.0391830,-0.0064500,0.0071855},
                    {-0.0391830,-0.0064500,-0.0071855},
                    {-0.0628466,0.0057914,0.0260363},
                    {-0.0628466,0.0057914,-0.0260363},
                    {-0.0778575,-0.0370965,0.0238034},
                    {-0.0778575,-0.0370965,-0.0238034},
                    {-0.0627763,-0.0214524,0.0168497},
                    {-0.0627763,-0.0214524,-0.0168497},
                    {-0.0926497,-0.0721918,0.0061723},
                    {-0.0926497,-0.0721918,-0.0061723},
                    {-0.0668480,-0.0275468,0.0084549},
                    {-0.0668480,-0.0275468,-0.0084549},
                    {-0.0849004,-0.0525004,0.0251422},
                    {-0.0849004,-0.0525004,-0.0251422},
                    {-0.0721947,-0.0349527,0.0081012},
                    {-0.0721947,-0.0349527,-0.0081012}
                    };
            BoundingBoxOnOff = Off;
        };
        AnyFunTransform3DIdentity ScaleFunction = {
            PreTransforms = {&.RBFTransform};
};
};
AnyFolder Sacrum = {
        AnyFunTransform3DRBF RBFTransform = {
            RBFDef.Type = RBF_ThinPlate;
            PolynomDegree = 1;
            Points0 = {
                    {0.0000000,0.0000000,0.1177000},
                    {-0.0000000,-0.0832729,0.0191000},
                    {-0.0508179,-0.0694062,0.0815920},
                    {0.0000000,0.0000000,-0.1177000},
                    {-0.0000000,-0.0832729,-0.0191000},
                    {-0.0508179,-0.0694062,-0.0815920},
                    {0.0000000,0.0000000,0.0000000},
                    {-0.1164020,-0.0039449,0.0456760},
                    {-0.1164020,-0.0039449,-0.0456760},
                    {-0.1092909,-0.0992949,0.0515590},
```

{-0.1092909,-0.0992949,-0.0515590},
{-0.0733749,0.0757445,0.0915590},
{-0.0733749,0.0757445,-0.0915590},
{-0.1241738,0.0316243,0.0458050},
{-0.1241738,0.0316243,-0.0458050},
{-0.1108008,0.0563490,0.0555690},
{-0.1108008,0.0563490,-0.0555690},
{-0.0545356,0.0481653,0.1291690},
{-0.0545356,0.0481653,-0.1291690},
{-0.0360236,0.0456352,0.1316250},
{-0.0360236,0.0456352,-0.1316250},
{-0.0804664,-0.0228481,0.0672140},
{-0.0804664,-0.0228481,-0.0672140},
{-0.1004070,-0.0623213,0.0494990},
{-0.1004070,-0.0623213,-0.0494990},
{-0.0174901,-0.0357786,0.0977400},
{-0.0174901,-0.0357786,-0.0977400},
{-0.0665950,-0.1308418,0.0278190},
{-0.0665950,-0.1308418,-0.0278190},
{-0.0962739,-0.1257274,0.0551670},
{-0.0962739,-0.1257274,-0.0551670},
{-0.0393340,-0.1196098,0.0159820},
{-0.0393340,-0.1196098,-0.0159820},
{-0.0585979,-0.0148191,0.0651020},
{-0.0585979,-0.0148191,-0.0651020},
{-0.0238420,-0.0800479,0.0351680},
{-0.0238420,-0.0800479,-0.0351680},
{-0.0234553,-0.0576924,0.0715330},
{-0.0234553,-0.0576924,-0.0715330},
{-0.0472319,-0.0833276,0.0513790},
{-0.0472319,-0.0833276,-0.0513790},
{-0.0327175,-0.0506481,0.0520240},
{-0.0327175,-0.0506481,-0.0520240},
{-0.0746024,-0.0627219,0.0928110},
{-0.0746024,-0.0627219,-0.0928110},
{-0.0119676,-0.0928080,0.0076900},
{-0.0119676,-0.0928080,-0.0076900},
{-0.0263299,-0.0760728,0.0667080},
{-0.0263299,-0.0760728,-0.0667080},
{-0.0498842,0.0119450,0.1070630},
{-0.0498842,0.0119450,-0.1070630},
{-0.0553874,0.0627474,0.1172390},
{-0.0553874,0.0627474,-0.1172390},
{-0.0786774,0.0073556,0.0741800},
{-0.0786774,0.0073556,-0.0741800},
{-0.0742762,0.0474272,0.0542330},

Biology Open • Supplementary information

{-0.0742762,0.0474272,-0.0542330},
{-0.0634121,0.0117185,0.0618430},
{-0.0634121,0.0117185,-0.0618430},
{-0.0813084,0.0007821,0.0508130},
{-0.0813084,0.0007821,-0.0508130},
{-0.0496423,-0.0444458,0.0983920},
{-0.0496423,-0.0444458,-0.0983920},
{-0.0480005,-0.0338999,0.0625080},
{-0.0480005,-0.0338999,-0.0625080},
{-0.0428000,-0.0273188,0.0973490},
{-0.0428000,-0.0273188,-0.0973490},
{-0.0548383,-0.0518766,0.0624050},
{-0.0548383,-0.0518766,-0.0624050},
{-0.0696448,-0.1052827,0.0436200},
{-0.0696448,-0.1052827,-0.0436200},
{-0.0739608,-0.0803177,0.0785950},
{-0.0739608,-0.0803177,-0.0785950},
{-0.0586066,-0.0884764,0.0653190},
{-0.0586066,-0.0884764,-0.0653190},
{-0.1017003,0.0085215,0.0624150},
{-0.1017003,0.0085215,-0.0624150},
{-0.0126252,0.0252852,0.1292530},
{-0.0126252,0.0252852,-0.1292530},
{-0.0030963,0.0150156,0.1262640},
{-0.0030963,0.0150156,-0.1262640},
{-0.0621797,0.0048743,0.0605920},
{-0.0621797,0.0048743,-0.0605920},
{-0.0870082,-0.0684280,0.0489900},
{-0.0870082,-0.0684280,-0.0489900},
{-0.0916211,-0.0478344,0.0575290},
{-0.0916211,-0.0478344,-0.0575290},
{-0.0428573,-0.0467026,0.0985250},
{-0.0428573,-0.0467026,-0.0985250},
{-0.0483869,-0.0455474,0.0848580},
{-0.0483869,-0.0455474,-0.0848580},
{-0.0203929,-0.0681811,0.0426070},
{-0.0203929,-0.0681811,-0.0426070},
{-0.0271973,-0.0807786,0.0491650},
{-0.0271973,-0.0807786,-0.0491650},
{-0.0325935,-0.1026813,0.0148280},
{-0.0325935,-0.1026813,-0.0148280},
{-0.0273900,-0.1059173,0.0048750},
{-0.0273900,-0.1059173,-0.0048750},
{-0.0015971,-0.0778770,0.0065530},
{-0.0015971,-0.0778770,-0.0065530},
{-0.1034983,-0.0140026,0.0521800},

```
        {-0.1034983,-0.0140026,-0.0521800},
        {-0.1141160,0.0077734,0.0497600},
        {-0.1141160,0.0077734,-0.0497600},
        {-0.1123797,0.0244808,0.0350160},
        {-0.1123797,0.0244808,-0.0350160},
        {-0.0984470,-0.0648210,0.0457100},
        {-0.0984470,-0.0648210,-0.0457100},
        {-0.0814332,-0.0704479,0.0806410},
        {-0.0814332,-0.0704479,-0.0806410},
        {-0.0540218,-0.0805070,0.0547320},
        {-0.0540218,-0.0805070,-0.0547320},
        {-0.0674356,-0.0763171,0.0651080},
        {-0.0674356,-0.0763171,-0.0651080},
        {-0.0602364,-0.0751827,0.0490600},
        {-0.0602364,-0.0751827,-0.0490600},
        {-0.0604272,-0.0801096,0.0617610},
        {-0.0604272,-0.0801096,-0.0617610},
        {-0.0655251,0.0632155,0.1093750},
        {-0.0655251,0.0632155,-0.1093750},
        {-0.0665046,0.0014289,0.0572170},
        {-0.0665046,0.0014289,-0.0572170},
        {-0.1053786,0.0347803,0.0071580},
        {-0.1053786,0.0347803,-0.0071580},
        {-0.1321649,-0.0069725,0.0058340},
        {-0.1321649,-0.0069725,-0.0058340},
        {-0.1232735,-0.0434903,0.0301110},
        {-0.1232735,-0.0434903,-0.0301110},
        {-0.0497024,0.0261862,0.0084640},
        {-0.0497024,0.0261862,-0.0084640},
        {-0.0887827,0.0446048,0.0327030},
        {-0.0887827,0.0446048,-0.0327030},
        {-0.1153998,-0.0126010,0.0290730},
        {-0.1153998,-0.0126010,-0.0290730},
        {-0.0899939,0.0093882,0.0208440},
        {-0.0899939,0.0093882,-0.0208440},
        {-0.1312654,-0.0574898,0.0094690},
        {-0.1312654,-0.0574898,-0.0094690},
        {-0.0996029,0.0042806,0.0105620},
        {-0.0996029,0.0042806,-0.0105620},
        {-0.1226177,-0.0324069,0.0331630},
        {-0.1226177,-0.0324069,-0.0331630},
        {-0.1097808,-0.0051619,0.0102520},
        {-0.1097808,-0.0051619,-0.0102520}
        };
Points1 = {
        {0.0000000,0.0000000,0.1146472},
```

{-0.0000007,-0.0841567,0.0166942},
{-0.0287487,-0.0653499,0.0797819},
{0.0000000,0.0000000,-0.1146472},
{-0.0000007,-0.0841567,-0.0166942},
{-0.0287487,-0.0653499,-0.0797819},
{0.0000000,0.0000000,0.0000000},
{-0.0784974,-0.0274825,0.0388349},
{-0.0784974,-0.0274825,-0.0388349},
{-0.0744033,-0.0924251,0.0567016},
{-0.0744033,-0.0924251,-0.0567016},
{-0.0510804,0.0386519,0.0836160},
{-0.0510804,0.0386519,-0.0836160},
{-0.0828703,-0.0068852,0.0344311},
{-0.0828703,-0.0068852,-0.0344311},
{-0.0752265,0.0144776,0.0457165},
{-0.0752265,0.0144776,-0.0457165},
{-0.0315348,0.0296673,0.1187151},
{-0.0315348,0.0296673,-0.1187151},
{-0.0205507,0.0299668,0.1226123},
{-0.0205507,0.0299668,-0.1226123},
{-0.0555552,-0.0372896,0.0629480},
{-0.0555552,-0.0372896,-0.0629480},
{-0.0694110,-0.0681665,0.0582299},
{-0.0694110,-0.0681665,-0.0582299},
{-0.0103438,-0.0365392,0.0860731},
{-0.0103438,-0.0365392,-0.0860731},
{-0.0480752,-0.1163946,0.0312929},
{-0.0480752,-0.1163946,-0.0312929},
{-0.0650354,-0.1078995,0.0596306},
{-0.0650354,-0.1078995,-0.0596306},
{-0.0270475,-0.1115436,0.0174585},
{-0.0270475,-0.1115436,-0.0174585},
{-0.0432274,-0.0265759,0.0608230},
{-0.0432274,-0.0265759,-0.0608230},
{-0.0137436,-0.0767335,0.0315340},
{-0.0137436,-0.0767335,-0.0315340},
{-0.0144631,-0.0545433,0.0676070},
{-0.0144631,-0.0545433,-0.0676070},
{-0.0268521,-0.0742763,0.0518728},
{-0.0268521,-0.0742763,-0.0518728},
{-0.0224786,-0.0547544,0.0526130},
{-0.0224786,-0.0547544,-0.0526130},
{-0.0455798,-0.0612956,0.0839125},
{-0.0455798,-0.0612956,-0.0839125},
{-0.0065754,-0.0933634,0.0071677},
{-0.0065754,-0.0933634,-0.0071677},

Biology Open • Supplementary information

{-0.0153687,-0.0657224,0.0615932},
{-0.0153687,-0.0657224,-0.0615932},
{-0.0332917,-0.0009231,0.0976437},
{-0.0332917,-0.0009231,-0.0976437},
{-0.0355334,0.0368942,0.1083098},
{-0.0355334,0.0368942,-0.1083098},
{-0.0564439,-0.0132508,0.0637942},
{-0.0564439,-0.0132508,-0.0637942},
{-0.0540551,0.0130165,0.0459726},
{-0.0540551,0.0130165,-0.0459726},
{-0.0490808,-0.0095136,0.0517264},
{-0.0490808,-0.0095136,-0.0517264},
{-0.0590838,-0.0214488,0.0429470},
{-0.0590838,-0.0214488,-0.0429470},
{-0.0286153,-0.0469561,0.0868049},
{-0.0286153,-0.0469561,-0.0868049},
{-0.0329709,-0.0404163,0.0620342},
{-0.0329709,-0.0404163,-0.0620342},
{-0.0266208,-0.0324563,0.0871445},
{-0.0266208,-0.0324563,-0.0871445},
{-0.0340631,-0.0543357,0.0635634},
{-0.0340631,-0.0543357,-0.0635634},
{-0.0460524,-0.0953474,0.0487290},
{-0.0460524,-0.0953474,-0.0487290},
{-0.0448550,-0.0749420,0.0763273},
{-0.0448550,-0.0749420,-0.0763273},
{-0.0342004,-0.0815297,0.0680470},
{-0.0342004,-0.0815297,-0.0680470},
{-0.0699394,-0.0160378,0.0528982},
{-0.0699394,-0.0160378,-0.0528982},
{-0.0068432,0.0176617,0.1229247},
{-0.0068432,0.0176617,-0.1229247},
{-0.0012296,0.0111359,0.1214124},
{-0.0012296,0.0111359,-0.1214124},
{-0.0488395,-0.0142692,0.0504590},
{-0.0488395,-0.0142692,-0.0504590},
{-0.0583873,-0.0706497,0.0578424},
{-0.0583873,-0.0706497,-0.0578424},
{-0.0619573,-0.0565203,0.0604806},
{-0.0619573,-0.0565203,-0.0604806},
{-0.0236134,-0.0487029,0.0863855},
{-0.0236134,-0.0487029,-0.0863855},
{-0.0289114,-0.0480035,0.0776342},
{-0.0289114,-0.0480035,-0.0776342},
{-0.0122006,-0.0679494,0.0382063},
{-0.0122006,-0.0679494,-0.0382063},

Biology Open • Supplementary information

{-0.0155387,-0.0741466,0.0452690},
{-0.0155387,-0.0741466,-0.0452690},
{-0.0194459,-0.0986350,0.0159059},
{-0.0194459,-0.0986350,-0.0159059},
{-0.0144349,-0.1017428,0.0062136},
{-0.0144349,-0.1017428,-0.0062136},
{-0.0016710,-0.0829660,0.0057660},
{-0.0016710,-0.0829660,-0.0057660},
{-0.0702038,-0.0328837,0.0482627},
{-0.0702038,-0.0328837,-0.0482627},
{-0.0772852,-0.0181292,0.0409158},
{-0.0772852,-0.0181292,-0.0409158},
{-0.0758759,-0.0108913,0.0263065},
{-0.0758759,-0.0108913,-0.0263065},
{-0.0684427,-0.0699242,0.0570009},
{-0.0684427,-0.0699242,-0.0570009},
{-0.0508078,-0.0684821,0.0776849},
{-0.0508078,-0.0684821,-0.0776849},
{-0.0304044,-0.0714368,0.0563291},
{-0.0304044,-0.0714368,-0.0563291},
{-0.0404078,-0.0730508,0.0679410},
{-0.0404078,-0.0730508,-0.0679410},
{-0.0361396,-0.0711300,0.0530943},
{-0.0361396,-0.0711300,-0.0530943},
{-0.0350093,-0.0755673,0.0655383},
{-0.0350093,-0.0755673,-0.0655383},
{-0.0440093,0.0337575,0.1006149},
{-0.0440093,0.0337575,-0.1006149},
{-0.0519337,-0.0178552,0.0468714},
{-0.0519337,-0.0178552,-0.0468714},
{-0.0718288,-0.0056450,0.0052866},
{-0.0718288,-0.0056450,-0.0052866},
{-0.0877872,-0.0356376,0.0042068},
{-0.0877872,-0.0356376,-0.0042068},
{-0.0869194,-0.0616983,0.0197486},
{-0.0869194,-0.0616983,-0.0197486},
{-0.0391830,-0.0064500,0.0071855},
{-0.0391830,-0.0064500,-0.0071855},
{-0.0628466,0.0057914,0.0260363},
{-0.0628466,0.0057914,-0.0260363},
{-0.0778575,-0.0370965,0.0238034},
{-0.0778575,-0.0370965,-0.0238034},
{-0.0627763,-0.0214524,0.0168497},
{-0.0627763,-0.0214524,-0.0168497},
{-0.0926497,-0.0721918,0.0061723},
{-0.0926497,-0.0721918,-0.0061723},

Biology Open • Supplementary information

```
                    {-0.0668480,-0.0275468,0.0084549},
                    {-0.0668480,-0.0275468,-0.0084549},
                    {-0.0849004,-0.0525004,0.0251422},
                    {-0.0849004,-0.0525004,-0.0251422},
                    {-0.0721947,-0.0349527,0.0081012},
                    {-0.0721947,-0.0349527,-0.0081012}
                    };
            BoundingBoxOnOff = Off;
        };
        AnyFunTransform3DIdentity ScaleFunction = {
            PreTransforms = {&.RBFTransform};
};
};

};
```

Biology Open • Supplementary information

ScalingFunctionTLEMLucyFemur_2014031

```
AnyFolder ScalingFunctionTLEMLucyFemur = {
  AnyFolder Right = {
        AnyFolder Thigh = {
        AnyFunTransform3DRBF RBFTransform = {
              RBFDef.Type = RBF_ThinPlate;
              PolynomDegree = 1;
              Points0 = {
                    {0.0000000,0.0000000,0.0000000},
                    {-0.0000000,-0.3616821,0.0000000},
                    {-0.0097563,-0.3678799,0.0012967},
                    {-0.0000000,-0.3660632,0.0408203},
                    {-0.0000000,-0.3573010,-0.0408203},
                    {0.0161460,-0.0072838,0.0601290},
                    {0.0220217,-0.0203698,0.0463848},
                    {0.0123977,-0.0241932,0.0668573},
                    {0.0006898,0.0018121,0.0538181},
                    {0.0122809,-0.0068668,0.0414535},
                    {-0.0058991,-0.0138188,0.0648412},
                    {0.0211469,-0.0380855,0.0538111},
                    {0.0172133,-0.0317342,0.0328381},
                    {0.0177311,-0.0131946,0.0256176},
                    {-0.0110079,-0.0308867,0.0632370},
                    {0.0039435,-0.0395977,0.0638271},
                    {-0.0041683,-0.0187472,0.0413214},
                    {-0.0177999,-0.0021535,0.0496084},
                    {0.0001603,-0.0053126,0.0279593},
                    {-0.0211600,-0.0216243,0.0497147},
                    {0.0096163,-0.0568663,0.0545719},
                    {0.0162112,-0.0515013,0.0395230},
                    {0.0093533,-0.0433265,0.0187387},
                    {0.0119398,-0.0238469,0.0118403},
                    {0.0210707,-0.0048848,0.0065878},
                    {0.0125013,0.0064094,0.0177702},
                    {-0.0191523,-0.0356053,0.0382429},
                    {-0.0115821,-0.0474937,0.0537648},
                    {-0.0100073,-0.0236371,0.0267322},
                    {-0.0078925,0.0107098,0.0182833},
                    {-0.0134217,-0.0055356,0.0182000},
                    {-0.0090061,-0.0665700,0.0475484},
                    {0.0071095,-0.0761976,0.0520035},
                    {0.0182119,-0.0704149,0.0386546},
                    {0.0073764,-0.0609481,0.0221006},
                    {-0.0122984,-0.0439585,0.0198428},
                    {-0.0032253,-0.0306792,0.0110995},
```

Biology Open • Supplementary information

{-0.0012975,-0.0198451,-0.0052592},
{0.0140405,-0.0161125,-0.0071649},
{0.0189222,-0.0014898,-0.0127226},
{0.0181642,0.0132812,-0.0011050},
{0.0045119,0.0200001,0.0091189},
{-0.0176761,-0.0541983,0.0348587},
{-0.0136548,-0.0142015,0.0054311},
{-0.0135510,0.0182940,0.0017175},
{-0.0223865,0.0023399,0.0028018},
{-0.0086455,-0.0738247,0.0283843},
{-0.0050356,-0.0877749,0.0442195},
{0.0160135,-0.0919319,0.0465120},
{0.0128290,-0.0785810,0.0232431},
{-0.0168328,-0.0616327,0.0155917},
{-0.0172369,-0.0094567,-0.0119502},
{0.0002090,-0.0093641,-0.0209779},
{0.0064827,0.0081538,-0.0198453},
{0.0010777,0.0210422,-0.0084721},
{-0.0129485,0.0085451,-0.0162212},
{-0.0002322,-0.0924033,0.0245644},
{-0.0028175,-0.1052263,0.0418294},
{0.0172322,-0.1111444,0.0445760},
{0.0203259,-0.0957409,0.0271449},
{0.0057102,-0.1088933,0.0195113},
{-0.0010852,-0.1230776,0.0363132},
{0.0182727,-0.1303312,0.0422884},
{0.0242891,-0.1161703,0.0265054},
{0.0094531,-0.1268584,0.0162294},
{0.0014244,-0.1413293,0.0331653},
{0.0207271,-0.1496427,0.0394157},
{0.0267562,-0.1360142,0.0236798},
{0.0113839,-0.1461651,0.0136024},
{0.0030845,-0.1595094,0.0297156},
{0.0209528,-0.1691386,0.0369775},
{0.0283958,-0.1540209,0.0213795},
{0.0129499,-0.1649012,0.0113589},
{0.0016490,-0.1783108,0.0216287},
{0.0147105,-0.1880678,0.0328614},
{0.0298491,-0.1859865,0.0248551},
{0.0286645,-0.1714377,0.0169488},
{0.0165897,-0.1861006,0.0082595},
{0.0014000,-0.1973476,0.0183740},
{0.0114301,-0.2109792,0.0286157},
{0.0267422,-0.2037622,0.0296951},
{0.0288630,-0.2018335,0.0130127},
{0.0145471,-0.2083505,0.0062833},

Biology Open • Supplementary information

{0.0011300,-0.2198071,0.0155602},
{0.0126610,-0.2321235,0.0275354},
{0.0275647,-0.2219268,0.0265185},
{0.0286645,-0.2230253,0.0098701},
{0.0130434,-0.2304822,0.0039177},
{0.0022984,-0.2409585,0.0149650},
{0.0101315,-0.2534740,0.0252125},
{0.0279485,-0.2428555,0.0234276},
{0.0276684,-0.2421559,0.0058691},
{0.0101250,-0.2529527,0.0018983},
{0.0021376,-0.2667831,0.0107073},
{0.0114309,-0.2734905,0.0249960},
{0.0273923,-0.2634953,0.0216324},
{0.0278429,-0.2592546,0.0037692},
{0.0159101,-0.2712985,-0.0033988},
{0.0036708,-0.2863280,0.0004644},
{0.0023115,-0.2884756,0.0187554},
{0.0231385,-0.2879051,0.0224853},
{0.0304009,-0.2799973,0.0083549},
{0.0238189,-0.2917246,-0.0052462},
{0.0064904,-0.3027687,-0.0070587},
{-0.0003492,-0.3064690,0.0093014},
{0.0122179,-0.3045269,0.0243511},
{0.0301837,-0.3030127,0.0114462},
{0.0234182,-0.3107336,-0.0085198},
{0.0046893,-0.3214995,-0.0123881},
{-0.0022352,-0.3264905,0.0043340},
{0.0016841,-0.3202756,0.0217839},
{0.0229722,-0.3196887,0.0215083},
{0.0292438,-0.3228042,0.0051430},
{0.0221641,-0.3296190,-0.0132361},
{0.0100459,-0.3417597,-0.0244560},
{-0.0053009,-0.3375737,-0.0137705},
{-0.0064432,-0.3487307,0.0028676},
{-0.0059346,-0.3407801,0.0202938},
{0.0105130,-0.3365976,0.0264175},
{0.0282695,-0.3386249,0.0180307},
{0.0270402,-0.3431844,-0.0001487},
{0.0257040,-0.3506950,-0.0181851},
{0.0178948,-0.3636114,-0.0302838},
{0.0043149,-0.3544109,-0.0381949},
{-0.0095210,-0.3461482,-0.0295031},
{-0.0164526,-0.3542895,-0.0126666},
{-0.0060896,-0.3690127,-0.0033061},
{-0.0176523,-0.3612686,0.0110109},
{-0.0153310,-0.3546943,0.0299634},

Biology Open • Supplementary information

{0.0021329,-0.3508021,0.0350763},
{0.0210690,-0.3548628,0.0279835},
{0.0356594,-0.3558162,0.0159356},
{0.0275718,-0.3617450,-0.0018607},
{0.0298503,-0.3741530,-0.0163721},
{0.0175320,-0.3855300,-0.0277515},
{0.0025604,-0.3740099,-0.0397137},
{-0.0136727,-0.3624016,-0.0402102},
{-0.0277647,-0.3567472,-0.0284947},
{-0.0275446,-0.3699630,-0.0120829},
{-0.0175846,-0.3859355,-0.0177922},
{0.0001572,-0.3856873,-0.0118050},
{0.0030262,-0.3834856,0.0075553},
{-0.0144114,-0.3810887,0.0140304},
{-0.0247706,-0.3701239,0.0285134},
{-0.0044804,-0.3677201,0.0404632},
{0.0133183,-0.3712274,0.0334208},
{0.0324861,-0.3723417,0.0245668},
{0.0271451,-0.3766027,0.0072441},
{0.0167985,-0.3822816,-0.0065349},
{-0.0001454,-0.3910823,-0.0303010},
{-0.0144998,-0.3834154,-0.0384137},
{-0.0301321,-0.3750841,-0.0321336},
{0.0196491,-0.3864804,0.0210853},
{0.0015726,-0.3888034,0.0276569},
{-0.0146652,-0.3847848,0.0336913},
{-0.0014588,-0.0619951,0.0219778},
{-0.0118435,-0.0550626,0.0139639},
{0.0129812,-0.0349966,0.0673128},
{-0.0162907,-0.0105666,0.0597739},
{-0.0012885,-0.0520816,0.0562989},
{-0.0090599,-0.0631298,0.0172224},
{-0.0000222,-0.0771663,0.0231897},
{-0.0068148,0.0023885,-0.0160086},
{-0.0180544,-0.0425409,0.0446585},
{0.0020525,-0.0249904,0.0691549},
{-0.0157097,-0.0662315,0.0238393},
{-0.0010518,-0.0510686,0.0199353},
{-0.0208874,-0.0152865,0.0500034},
{0.0168316,-0.0280857,0.0666680},
{-0.0187392,-0.0282333,0.0403282},
{0.0191622,-0.0351633,0.0622707},
{0.0001216,-0.0706289,0.0524049},
{-0.0169862,-0.0399062,0.0311827},
{-0.0185737,-0.0533066,0.0151224},
{-0.0219058,-0.0528723,0.0222263},

Biology Open • Supplementary information

{-0.0028271,-0.0391620,0.0148452},
{0.0117900,-0.0553425,0.0235348},
{0.0219835,-0.0106400,0.0396338},
{-0.0025366,-0.0401046,0.0625273},
{0.0141017,-0.0018767,0.0198108},
{-0.0078770,-0.0473929,0.0173542},
{-0.0065351,-0.0766431,0.0470258},
{0.0065585,0.0145593,0.0161638},
{-0.0118588,0.0172875,-0.0085064},
{-0.0074087,-0.0627500,0.0506700},
{0.0071940,-0.0780068,0.0213362},
{0.0126736,-0.0708176,0.0244108},
{0.0074694,-0.0436450,0.0604196},
{0.0154186,-0.0139471,0.0064312},
{-0.0053535,-0.0213210,0.0040094},
{-0.0088733,0.0002018,0.0205402},
{-0.0112471,-0.0542608,0.0500266},
{-0.0061625,-0.0248673,0.0653244},
{0.0093695,-0.0065826,0.0614157},
{0.0138015,-0.0467322,0.0284333},
{-0.0020999,-0.0186885,-0.0116392},
{-0.0185640,-0.0225591,0.0566779},
{0.0175798,0.0124360,-0.0068467},
{-0.0039962,0.0217127,0.0053055},
{0.0014975,-0.0784591,0.0514056},
{-0.0172997,-0.0386971,0.0525252},
{0.0196540,-0.0268271,0.0416806},
{0.0135296,-0.0406504,0.0236882},
{0.0182471,0.0095959,0.0087906},
{0.0218667,0.0016804,0.0058793},
{0.0224975,-0.0022652,-0.0022994},
{0.0166750,-0.0191391,0.0236436},
{0.0119785,-0.0328723,0.0195376},
{0.0187631,-0.0133469,0.0308741},
{-0.0187543,-0.0461463,0.0257491},
{0.0039834,-0.0373928,0.0683089},
{0.0157029,-0.0423169,0.0385226},
{0.0164003,-0.0087069,0.0131181},
{0.0008253,0.0033370,-0.0212576},
{0.0101707,-0.0009724,-0.0205688},
{0.0102948,-0.0642683,0.0524532},
{0.0152217,-0.0557944,0.0508978},
{0.0172973,-0.0565707,0.0419468},
{0.0167723,-0.0635334,0.0369834},
{-0.0047397,-0.0695325,0.0241398},
{-0.0152913,0.0035778,0.0161461},

{0.0091697,0.0009340,0.0575160},
{0.0196686,-0.0089533,0.0500006},
{0.0183998,-0.0212943,0.0622968},
{-0.0114340,-0.0316810,0.0250940},
{-0.0206442,-0.0092599,-0.0028425},
{0.0166868,-0.0784295,0.0276925},
{-0.0069678,-0.0054521,-0.0207011},
{0.0049490,0.0151109,-0.0160093},
{-0.0084025,-0.0318937,0.0162788},
{-0.0173679,0.0095594,-0.0108509},
{-0.0218912,0.0063457,-0.0020698},
{-0.0106547,-0.0668750,0.0397749},
{0.0136098,-0.0170666,-0.0018890},
{0.0141599,-0.0441405,0.0587594},
{0.0093255,-0.0183218,-0.0083008},
{0.0106367,-0.0128276,-0.0154269},
{0.0037350,-0.0549365,0.0209786},
{0.0087662,-0.0472902,0.0195496},
{0.0016839,-0.0708213,0.0221325},
{-0.0202977,-0.0530540,0.0301270},
{-0.0068584,0.0021781,0.0530002},
{-0.0144817,-0.0297218,0.0605833},
{0.0197015,-0.0165217,0.0560654},
{0.0156609,-0.0705363,0.0477736},
{0.0207876,-0.0221433,0.0330733},
{0.0092324,-0.0300767,0.0713160},
{-0.0203392,-0.0034488,0.0097332},
{0.0156978,-0.0143438,0.0629053},
{0.0143267,-0.0563519,0.0317489},
{-0.0161685,-0.0465391,0.0188591},
{-0.0196397,-0.0309875,0.0546496},
{0.0154804,-0.0380422,0.0305433},
{-0.0077774,-0.0351303,0.0637765},
{0.0063980,-0.0086419,-0.0201654},
{0.0030146,-0.0151094,-0.0165974},
{-0.0210080,-0.0096030,0.0507855},
{-0.0105684,-0.0411377,0.0586146},
{0.0180619,-0.0498960,0.0469835},
{-0.0192318,0.0116108,0.0024070},
{-0.0165651,0.0083622,0.0126870},
{-0.0014333,-0.0627138,0.0534746},
{0.0145127,-0.0080178,0.0353667},
{0.0148374,-0.0012228,0.0566181},
{0.0153834,-0.0062427,0.0444727},
{-0.0155164,-0.0132103,-0.0096398},
{0.0068685,-0.0221022,0.0011547},

Biology Open • Supplementary information

{0.0003332,-0.0458500,0.0183716},
{0.0205582,0.0040956,-0.0082969},
{0.0175752,0.0009340,-0.0148407},
{0.0142357,-0.0063623,-0.0169332},
{-0.0147285,0.0023412,-0.0164678},
{-0.0158781,-0.0064451,-0.0151346},
{0.0175951,0.0133456,0.0037559},
{0.0117660,0.0189899,-0.0030690},
{0.0096040,0.0151827,-0.0137450},
{0.0096565,-0.0215194,0.0662734},
{-0.0006793,-0.0074494,0.0631218},
{0.0007819,-0.0004519,0.0600781},
{0.0065377,-0.0125433,0.0654199},
{0.0090461,0.0073096,0.0188711},
{0.0151418,-0.0168302,0.0146465},
{0.0025166,-0.0231863,0.0688894},
{-0.0097891,-0.0152077,0.0253086},
{-0.0109149,-0.0177935,0.0119825},
{-0.0072079,-0.0099373,0.0272725},
{-0.0183727,-0.0467429,0.0356276},
{-0.0126871,-0.0584555,0.0428141},
{-0.0057054,-0.0273042,0.0103554},
{0.0215142,-0.0159693,0.0474531},
{0.0128492,-0.0070016,0.0287645},
{0.0052134,-0.0017047,0.0247463},
{-0.0024284,-0.0707498,0.0513049},
{-0.0036140,0.0220593,-0.0040484},
{-0.0002405,-0.0121253,0.0390050},
{-0.0119811,-0.0156737,0.0015445},
{0.0142082,-0.0279792,0.0246553},
{0.0122244,-0.0262013,0.0169829},
{-0.0146264,-0.0038855,0.0549384},
{0.0207450,-0.0325771,0.0496412},
{-0.0155040,-0.0497560,0.0437087},
{-0.0081119,-0.0756450,0.0387365},
{0.0039684,-0.0062047,0.0313900},
{-0.0023726,-0.0038892,-0.0220521},
{-0.0136977,-0.0339839,0.0602619},
{-0.0035399,0.0089081,-0.0188894},
{-0.0049820,0.0176571,-0.0132254},
{0.0193902,-0.0803191,0.0360429},
{0.0040648,-0.0697109,0.0218205},
{0.0170470,-0.0818610,0.0456892},
{0.0104175,0.0188635,0.0064965},
{0.0135206,0.0092549,0.0158079},
{0.0023933,0.0160498,-0.0157404},

Biology Open • Supplementary information

{0.0164753,-0.0118197,-0.0096995},
{0.0202584,-0.0066858,-0.0082383},
{-0.0098401,-0.0226001,0.0314028},
{-0.0130937,-0.0309622,0.0320764},
{-0.0201510,-0.0613297,0.0222264},
{-0.0010389,0.0036055,0.0209003},
{0.0042025,0.0073392,0.0199393},
{-0.0021630,-0.0038531,0.0251898},
{-0.0007845,-0.0293574,0.0675788},
{0.0070027,-0.0406327,0.0624565},
{-0.0124363,-0.0183001,0.0627966},
{-0.0120485,-0.0256613,0.0628493},
{0.0101200,0.0197695,0.0039334},
{0.0167636,0.0148266,0.0014155},
{-0.0074981,-0.0106930,0.0632797},
{0.0142533,-0.0503445,0.0351954},
{-0.0146107,-0.0460953,0.0506120},
{-0.0161117,-0.0471301,0.0178900},
{0.0112149,-0.0792793,0.0501460},
{-0.0099060,-0.0230794,0.0186951},
{-0.0114543,-0.0153049,0.0183675},
{-0.0042882,0.0161879,0.0151423},
{0.0014582,-0.0638572,0.0541080},
{0.0048554,-0.0582603,0.0553815},
{-0.0017077,-0.0572302,0.0546635},
{0.0166090,-0.0697169,0.0323038},
{-0.0208812,-0.0164273,0.0474354},
{0.0130454,-0.0241864,0.0178294},
{0.0053123,-0.0483770,0.0582251},
{0.0112225,-0.0361429,0.0673300},
{0.0085729,-0.0771256,0.0216100},
{-0.0070783,-0.0488033,0.0560869},
{0.0182083,-0.0411024,0.0454236},
{-0.0119590,-0.0403809,0.0213546},
{0.0233409,-0.0185535,0.0366343},
{-0.0046347,-0.0571211,0.0208243},
{0.0183166,-0.0006966,0.0140116},
{0.0061913,0.0053512,0.0201003},
{-0.0002364,-0.0310828,0.0680351},
{-0.0170773,0.0139270,-0.0054987},
{0.0110805,0.0156034,0.0117010},
{-0.0124628,0.0164667,0.0094550},
{-0.0209014,-0.0073407,-0.0051969},
{-0.0210131,0.0010693,-0.0086891},
{-0.0041596,-0.0172332,0.0663041},
{-0.0216595,-0.0322172,0.0451540},

{0.0051908,-0.0426014,0.0613013},
{0.0150003,-0.0416136,0.0606257},
{-0.0135090,-0.0674234,0.0236425},
{0.0146416,0.0073691,0.0160052},
{0.0195615,-0.0112412,-0.0010333},
{-0.0198115,-0.0098244,0.0044735},
{-0.0157054,-0.0521236,0.0395039},
{-0.0081381,-0.0173108,-0.0046152},
{-0.0218324,-0.0262661,0.0471115},
{0.0051446,-0.0707257,0.0530555},
{0.0179359,-0.0471493,0.0528134},
{-0.0005591,-0.0317983,0.0113632},
{0.0071707,-0.0305943,0.0121186},
{0.0206239,-0.0061367,0.0066642},
{0.0187057,-0.0745868,0.0420207},
{-0.0095784,-0.0163113,0.0259524},
{-0.0012181,0.0127661,0.0186855},
{0.0059420,-0.0630691,0.0220087},
{0.0069408,-0.0573878,0.0552614},
{0.0150782,-0.0132884,-0.0101793},
{0.0005533,-0.0078574,0.0633775},
{-0.0088284,-0.0146399,-0.0147837},
{0.0167444,-0.0651105,0.0470347},
{0.0082666,-0.0181741,-0.0095133},
{-0.0175162,-0.0637444,0.0177069},
{0.0153795,-0.0360767,0.0287608},
{0.0162363,-0.0018864,0.0548412},
{0.0052731,-0.0075919,0.0392990},
{-0.0022288,-0.0556644,0.0549909},
{-0.0156238,-0.0550038,0.0377688},
{-0.0204137,-0.0100550,0.0536429},
{-0.0093683,0.0122429,-0.0162013},
{-0.0202927,0.0049166,0.0083887},
{0.0074706,-0.0627945,0.0222133},
{0.0035034,-0.0376103,0.0672421},
{-0.0190752,-0.0194129,0.0555530},
{0.0061758,-0.0074961,-0.0207741},
{0.0192011,-0.0273063,0.0588233},
{0.0130796,-0.0134383,0.0638611},
{0.0120421,0.0019566,-0.0194140},
{0.0154152,-0.0065294,0.0179581},
{-0.0157335,0.0101219,0.0123981},
{0.0110376,-0.0488274,0.0567911},
{-0.0055170,-0.0343676,0.0647140},
{0.0216204,-0.0273988,0.0512041},
{0.0161331,-0.0135875,-0.0075501},

Biology Open • Supplementary information

{-0.0184643,-0.0455633,0.0360087},
{-0.0091978,-0.0682413,0.0460847},
{0.0034304,-0.0471677,0.0588866},
{-0.0048025,-0.0384316,0.0149400},
{0.0118505,0.0109735,-0.0159161},
{-0.0027036,-0.0204189,-0.0015094},
{-0.0117607,-0.0692759,0.0292171},
{-0.0107080,-0.0033455,-0.0190487},
{-0.0042548,0.0214947,-0.0056846},
{-0.0097315,-0.0208529,0.0131997},
{-0.0178958,-0.0627218,0.0171551},
{-0.0068692,-0.0688818,0.0233316},
{0.0012083,0.0120695,-0.0186179},
{0.0215073,-0.0236027,0.0503069},
{-0.0060262,-0.0534931,0.0189523},
{0.0179951,-0.0336785,0.0397267},
{0.0190509,-0.0284972,0.0336394},
{-0.0169188,-0.0133609,-0.0014203},
{0.0135722,0.0136491,0.0114695},
{-0.0150549,-0.0123230,0.0067722},
{-0.0148411,-0.0311945,0.0355412},
{0.0185490,-0.0794042,0.0431266},
{0.0193568,-0.0087326,0.0575704},
{0.0201026,-0.0144353,0.0531586},
{0.0025583,-0.0248211,0.0045714},
{0.0132689,-0.0214866,0.0652899},
{-0.0028669,-0.0099311,0.0309271},
{-0.0219487,-0.0054893,-0.0021853},
{0.0026180,-0.0203806,-0.0040620},
{-0.0116230,-0.0668518,0.0370152},
{-0.0133007,-0.0264159,0.0619176},
{0.0186167,0.0050491,-0.0120059},
{-0.0191359,-0.0591690,0.0288149},
{-0.0178121,-0.0041834,0.0141448},
{0.0123077,-0.0063648,0.0279743},
{-0.0157729,-0.0374010,0.0315335},
{0.0149328,-0.0659100,0.0290095},
{0.0006205,0.0021977,-0.0214613},
{0.0170484,-0.0715687,0.0325980},
{0.0068499,0.0061192,0.0196608},
{-0.0167075,0.0020024,0.0150382},
{0.0059801,-0.0393117,0.0167489},
{-0.0072506,-0.0171067,0.0338751},
{0.0202363,-0.0251596,0.0420997},
{-0.0199311,-0.0601255,0.0171522},
{0.0216641,0.0053410,-0.0027841},

{-0.0197665,-0.0606125,0.0252972},
{0.0129986,-0.0214528,0.0098002},
{0.0201151,-0.0100929,0.0460501},
{0.0104137,0.0017765,-0.0203073},
{0.0166557,-0.0064123,0.0140197},
{-0.0124120,-0.0596549,0.0422637},
{0.0014885,0.0202590,0.0096781},
{0.0028720,0.0020207,-0.0219044},
{0.0376095,-0.3675427,0.0214537},
{0.0049949,0.0206417,-0.0079017},
{-0.0191723,-0.0407590,0.0391310},
{-0.0316406,-0.3638215,-0.0274841},
{0.0021392,0.0226920,0.0000985},
{0.0168435,-0.0229382,0.0264788},
{-0.0157304,-0.0005437,-0.0161456},
{0.0203722,-0.0299750,0.0562762},
{-0.0171077,0.0134146,0.0059615},
{0.0175519,-0.0370653,0.0413374},
{0.0154657,-0.0405127,0.0348980},
{-0.0215172,-0.0323502,0.0481925},
{0.0177998,-0.0515776,0.0444709},
{-0.0183804,-0.0448316,0.0275139},
{0.0033607,-0.0502028,0.0575894},
{0.0010325,-0.0479377,0.0191141},
{0.0224852,-0.0181821,0.0451780},
{0.0084089,-0.0720538,0.0521031},
{-0.0070880,-0.0772912,0.0288023},
{-0.0223712,-0.0028990,0.0024047},
{0.0173136,-0.0120017,0.0204612},
{0.0137772,-0.0623537,0.0276621},
{-0.0100390,-0.0231180,0.0292222},
{-0.0196395,-0.3730207,0.0364730},
{-0.0094445,-0.0526688,0.0528111},
{-0.0115309,-0.3679776,-0.0104995},
{0.0129812,-0.0410477,0.0224072},
{0.0117394,0.0154849,-0.0117191},
{-0.0245208,-0.3649412,0.0295111},
{0.0147476,-0.0502966,0.0364899},
{0.0006132,-0.0446771,0.0600524},
{-0.0118123,0.0191139,0.0031977},
{-0.0017185,-0.0163303,0.0664216},
{-0.0085098,-0.0074564,0.0247745},
{0.0197459,-0.0086852,0.0430460},
{0.0187064,-0.0223532,0.0600966},
{-0.0206552,-0.0099712,0.0525024},
{0.0207525,0.0084646,-0.0024846},

Biology Open • Supplementary information

{-0.0094915,-0.0325446,0.0184441},
{-0.0014464,-0.0140050,-0.0179819},
{-0.0122725,-0.0573444,0.0463125},
{-0.0106397,0.0177211,0.0094669},
{0.0111981,0.0191236,-0.0041491},
{-0.0162790,-0.0256279,0.0592687},
{-0.0060120,-0.3653959,0.0413411},
{-0.0135007,-0.3703638,-0.0387476},
{-0.0134258,-0.3703142,-0.0379467},
{-0.0133509,-0.3702645,-0.0371458},
{-0.0132760,-0.3702148,-0.0363450},
{-0.0132011,-0.3701651,-0.0355441},
{-0.0131262,-0.3701154,-0.0347432},
{-0.0130513,-0.3700658,-0.0339423},
{-0.0129765,-0.3700161,-0.0331414},
{-0.0129016,-0.3699664,-0.0323405},
{-0.0128267,-0.3699167,-0.0315396},
{-0.0127518,-0.3698670,-0.0307388},
{-0.0126769,-0.3698174,-0.0299379},
{-0.0126020,-0.3697677,-0.0291370},
{-0.0125271,-0.3697180,-0.0283361},
{-0.0124523,-0.3696683,-0.0275352},
{-0.0123774,-0.3696187,-0.0267343},
{-0.0123025,-0.3695690,-0.0259334},
{-0.0122276,-0.3695193,-0.0251325},
{-0.0121527,-0.3694696,-0.0243317},
{-0.0120778,-0.3694199,-0.0235308},
{-0.0120029,-0.3693703,-0.0227299},
{-0.0119280,-0.3693206,-0.0219290},
{-0.0118532,-0.3692709,-0.0211281},
{-0.0117783,-0.3692212,-0.0203272},
{-0.0117034,-0.3691715,-0.0195263},
{-0.0116285,-0.3691219,-0.0187255},
{-0.0115536,-0.3690722,-0.0179246},
{-0.0114787,-0.3690225,-0.0171237},
{-0.0114038,-0.3689728,-0.0163228},
{-0.0113289,-0.3689231,-0.0155219},
{-0.0112541,-0.3688735,-0.0147210},
{-0.0111792,-0.3688238,-0.0139201},
{-0.0111043,-0.3687741,-0.0131192},
{-0.0110294,-0.3687244,-0.0123184},
{-0.0109545,-0.3686748,-0.0115175},
{-0.0108796,-0.3686251,-0.0107166},
{-0.0108047,-0.3685754,-0.0099157},
{-0.0107298,-0.3685257,-0.0091148},
{-0.0106550,-0.3684760,-0.0083139},

Biology Open • Supplementary information

{-0.0105801,-0.3684264,-0.0075130},
{-0.0105052,-0.3683767,-0.0067121},
{-0.0104303,-0.3683270,-0.0059113},
{-0.0103554,-0.3682773,-0.0051104},
{-0.0102805,-0.3682276,-0.0043095},
{-0.0102056,-0.3681780,-0.0035086},
{-0.0101307,-0.3681283,-0.0027077},
{-0.0100559,-0.3680786,-0.0019068},
{-0.0099810,-0.3680289,-0.0011059},
{-0.0099061,-0.3679792,-0.0003051},
{-0.0098312,-0.3679296,0.0004958},
{-0.0097563,-0.3678799,0.0012967},
{-0.0096814,-0.3678302,0.0020976},
{-0.0096065,-0.3677805,0.0028985},
{-0.0095317,-0.3677308,0.0036994},
{-0.0094568,-0.3676812,0.0045003},
{-0.0093819,-0.3676315,0.0053012},
{-0.0093070,-0.3675818,0.0061020},
{-0.0092321,-0.3675321,0.0069029},
{-0.0091572,-0.3674825,0.0077038},
{-0.0090823,-0.3674328,0.0085047},
{-0.0090074,-0.3673831,0.0093056},
{-0.0089326,-0.3673334,0.0101065},
{-0.0088577,-0.3672837,0.0109074},
{-0.0087828,-0.3672341,0.0117082},
{-0.0087079,-0.3671844,0.0125091},
{-0.0086330,-0.3671347,0.0133100},
{-0.0085581,-0.3670850,0.0141109},
{-0.0084832,-0.3670353,0.0149118},
{-0.0084083,-0.3669857,0.0157127},
{-0.0083335,-0.3669360,0.0165136},
{-0.0082586,-0.3668863,0.0173145},
{-0.0081837,-0.3668366,0.0181153},
{-0.0081088,-0.3667869,0.0189162},
{-0.0080339,-0.3667373,0.0197171},
{-0.0079590,-0.3666876,0.0205180},
{-0.0078841,-0.3666379,0.0213189},
{-0.0078092,-0.3665882,0.0221198},
{-0.0077344,-0.3665386,0.0229207},
{-0.0076595,-0.3664889,0.0237215},
{-0.0075846,-0.3664392,0.0245224},
{-0.0075097,-0.3663895,0.0253233},
{-0.0074348,-0.3663398,0.0261242},
{-0.0073599,-0.3662902,0.0269251},
{-0.0072850,-0.3662405,0.0277260},
{-0.0072101,-0.3661908,0.0285269},

{-0.0071353,-0.3661411,0.0293278},
{-0.0070604,-0.3660914,0.0301286},
{-0.0069855,-0.3660418,0.0309295},
{-0.0069106,-0.3659921,0.0317304},
{-0.0068357,-0.3659424,0.0325313},
{-0.0067608,-0.3658927,0.0333322},
{-0.0066859,-0.3658430,0.0341331},
{-0.0066111,-0.3657934,0.0349340},
{-0.0065362,-0.3657437,0.0357348},
{-0.0064613,-0.3656940,0.0365357},
{-0.0063864,-0.3656443,0.0373366},
{-0.0063115,-0.3655947,0.0381375},
{-0.0062366,-0.3655450,0.0389384},
{-0.0061617,-0.3654953,0.0397393},
{-0.0060868,-0.3654456,0.0405402}
};
Points1 = {
{0.0000000,0.0000000,0.0000000},
{-0.0000000,-0.4260351,0.0000000},
{-0.0098081,-0.4333357,0.0013036},
{-0.0000000,-0.4311957,0.0410368},
{-0.0000000,-0.4208745,-0.0410368},
{0.0146010,-0.0095741,0.0588672},
{0.0211859,-0.0249003,0.0463312},
{0.0101821,-0.0282433,0.0642194},
{0.0001646,-0.0004404,0.0524005},
{0.0118244,-0.0075730,0.0408364},
{-0.0062334,-0.0155105,0.0666032},
{0.0177330,-0.0428706,0.0530675},
{0.0158880,-0.0372253,0.0341000},
{0.0150072,-0.0160286,0.0257986},
{-0.0106784,-0.0356007,0.0635500},
{0.0035375,-0.0466033,0.0625015},
{-0.0032386,-0.0223157,0.0423916},
{-0.0131463,-0.0047893,0.0493717},
{-0.0000093,-0.0046980,0.0286450},
{-0.0129090,-0.0254129,0.0507522},
{0.0106058,-0.0674492,0.0557533},
{0.0168668,-0.0599211,0.0397190},
{0.0087924,-0.0505968,0.0199162},
{0.0101682,-0.0269463,0.0129929},
{0.0192677,-0.0061135,0.0059390},
{0.0109029,0.0067403,0.0165746},
{-0.0128420,-0.0423899,0.0377696},
{-0.0105497,-0.0551045,0.0536628},
{-0.0096849,-0.0283656,0.0259278},

{-0.0064301,0.0118653,0.0166049},
{-0.0119331,-0.0065277,0.0163684},
{-0.0078099,-0.0779286,0.0465371},
{0.0066090,-0.0894214,0.0540609},
{0.0179611,-0.0838235,0.0383149},
{0.0087171,-0.0721856,0.0218839},
{-0.0123392,-0.0512545,0.0215070},
{-0.0044033,-0.0364328,0.0106265},
{-0.0004658,-0.0221125,-0.0050027},
{0.0134184,-0.0173307,-0.0067924},
{0.0181309,-0.0019923,-0.0116001},
{0.0177776,0.0144345,-0.0012628},
{0.0032858,0.0219052,0.0093805},
{-0.0149201,-0.0636250,0.0352907},
{-0.0123959,-0.0139031,0.0060405},
{-0.0126878,0.0188868,0.0016752},
{-0.0193552,0.0026183,0.0033051},
{-0.0071211,-0.0863171,0.0288998},
{-0.0045062,-0.1034954,0.0438209},
{0.0152900,-0.1086947,0.0467099},
{0.0130520,-0.0928686,0.0226912},
{-0.0105949,-0.0689400,0.0223391},
{-0.0152032,-0.0098392,-0.0106561},
{0.0006884,-0.0101613,-0.0194749},
{0.0062535,0.0086417,-0.0179260},
{0.0017596,0.0223535,-0.0084692},
{-0.0110061,0.0093520,-0.0151538},
{0.0004634,-0.1088815,0.0243038},
{-0.0019465,-0.1237875,0.0417851},
{0.0164663,-0.1310642,0.0445640},
{0.0196408,-0.1126733,0.0274539},
{0.0065308,-0.1279282,0.0201592},
{0.0000144,-0.1445420,0.0365896},
{0.0176249,-0.1534720,0.0422624},
{0.0234409,-0.1367601,0.0269520},
{0.0101602,-0.1489107,0.0172232},
{0.0024451,-0.1659436,0.0335992},
{0.0200551,-0.1761262,0.0394229},
{0.0258861,-0.1600378,0.0242282},
{0.0119994,-0.1715856,0.0146961},
{0.0040210,-0.1873208,0.0302349},
{0.0203710,-0.1990333,0.0370070},
{0.0275117,-0.1812035,0.0219519},
{0.0134430,-0.1936617,0.0124358},
{0.0027640,-0.2093905,0.0223754},
{0.0146601,-0.2212103,0.0330203},

Biology Open • Supplementary information

{0.0288672,-0.2189110,0.0251354},
{0.0278760,-0.2016748,0.0175983},
{0.0167976,-0.2187111,0.0092539},
{0.0024610,-0.2318514,0.0191054},
{0.0116118,-0.2481795,0.0288252},
{0.0259222,-0.2398727,0.0297555},
{0.0281415,-0.2375008,0.0135949},
{0.0148351,-0.2449575,0.0071736},
{0.0020780,-0.2583914,0.0162034},
{0.0127165,-0.2731733,0.0276467},
{0.0267599,-0.2613012,0.0265741},
{0.0280029,-0.2625039,0.0103893},
{0.0133488,-0.2710954,0.0046827},
{0.0030209,-0.2834310,0.0154402},
{0.0102788,-0.2983712,0.0252961},
{0.0271985,-0.2859992,0.0234596},
{0.0271207,-0.2850742,0.0063508},
{0.0104853,-0.2976425,0.0025117},
{0.0026930,-0.3139776,0.0110695},
{0.0114673,-0.3220440,0.0250022},
{0.0267615,-0.3103578,0.0216235},
{0.0273377,-0.3052710,0.0041551},
{0.0159789,-0.3193798,-0.0029159},
{0.0040474,-0.3370965,0.0007874},
{0.0026237,-0.3396778,0.0188668},
{0.0228206,-0.3391400,0.0224453},
{0.0298676,-0.3298093,0.0084956},
{0.0236607,-0.3435751,-0.0049992},
{0.0066696,-0.3565606,-0.0068982},
{-0.0001734,-0.3609418,0.0094100},
{0.0122335,-0.3587002,0.0243859},
{0.0299474,-0.3569555,0.0114822},
{0.0234301,-0.3660107,-0.0084693},
{0.0047141,-0.3787029,-0.0124538},
{-0.0022470,-0.3845820,0.0043570},
{0.0016930,-0.3772613,0.0218995},
{0.0230810,-0.3765721,0.0216164},
{0.0293909,-0.3802408,0.0051713},
{0.0222817,-0.3882671,-0.0133063},
{0.0100992,-0.4025680,-0.0245857},
{-0.0053290,-0.3976372,-0.0138435},
{-0.0064773,-0.4107793,0.0028828},
{-0.0059661,-0.4014141,0.0204015},
{0.0105687,-0.3964874,0.0265576},
{0.0284205,-0.3988755,0.0181264},
{0.0271846,-0.4042461,-0.0001495},

Biology Open • Supplementary information

{0.0258384,-0.4130930,-0.0182806},
{0.0179898,-0.4283077,-0.0304445},
{0.0043378,-0.4174702,-0.0383945},
{-0.0095715,-0.4077373,-0.0296597},
{-0.0165399,-0.4173272,-0.0127338},
{-0.0061219,-0.4346700,-0.0033237},
{-0.0177460,-0.4255480,0.0110693},
{-0.0154124,-0.4178040,0.0301224},
{0.0021442,-0.4132192,0.0352624},
{0.0211808,-0.4180025,0.0281320},
{0.0358476,-0.4191256,0.0160201},
{0.0277191,-0.4261093,-0.0018705},
{0.0300087,-0.4407249,-0.0164590},
{0.0176260,-0.4541262,-0.0278987},
{0.0025739,-0.4405564,-0.0399244},
{-0.0137462,-0.4268826,-0.0404245},
{-0.0279120,-0.4202221,-0.0286469},
{-0.0276907,-0.4357894,-0.0121470},
{-0.0176769,-0.4546039,-0.0178866},
{0.0001580,-0.4543115,-0.0118676},
{0.0030422,-0.4517180,0.0075954},
{-0.0144878,-0.4488946,0.0141048},
{-0.0249020,-0.4359790,0.0286647},
{-0.0045042,-0.4331474,0.0406779},
{0.0133889,-0.4372788,0.0335981},
{0.0326595,-0.4385913,0.0246972},
{0.0272892,-0.4436105,0.0072825},
{0.0168876,-0.4502999,-0.0065695},
{-0.0001461,-0.4606664,-0.0304627},
{-0.0145767,-0.4516354,-0.0386175},
{-0.0302919,-0.4418217,-0.0323041},
{0.0197523,-0.4552456,0.0211972},
{0.0015809,-0.4579820,0.0278047},
{-0.0147420,-0.4532484,0.0338700},
{-0.0008505,-0.0741081,0.0195154},
{-0.0101957,-0.0645127,0.0211629},
{0.0097188,-0.0378441,0.0633341},
{-0.0140232,-0.0153774,0.0583777},
{-0.0017190,-0.0630383,0.0586887},
{-0.0062203,-0.0707904,0.0230256},
{-0.0013932,-0.0909598,0.0210262},
{-0.0069073,0.0018793,-0.0174229},
{-0.0123140,-0.0494754,0.0421717},
{0.0032652,-0.0294912,0.0671496},
{-0.0100401,-0.0731283,0.0263363},
{-0.0010544,-0.0601032,0.0188056},

Biology Open • Supplementary information

{-0.0130201,-0.0188748,0.0520348},
{0.0134536,-0.0333784,0.0616140},
{-0.0103419,-0.0331525,0.0406684},
{0.0141219,-0.0381516,0.0598890},
{-0.0008805,-0.0830286,0.0550087},
{-0.0146446,-0.0486073,0.0320193},
{-0.0137983,-0.0616369,0.0225249},
{-0.0160682,-0.0602851,0.0238266},
{-0.0024512,-0.0458141,0.0139036},
{0.0107032,-0.0642233,0.0244688},
{0.0214428,-0.0146894,0.0405212},
{-0.0030880,-0.0488043,0.0634264},
{0.0134064,-0.0031106,0.0176305},
{-0.0087596,-0.0539198,0.0193623},
{-0.0059051,-0.0896364,0.0454125},
{0.0055232,0.0163693,0.0134309},
{-0.0111952,0.0188336,-0.0061570},
{-0.0069921,-0.0744679,0.0486092},
{0.0077671,-0.0936623,0.0198566},
{0.0128092,-0.0821627,0.0248567},
{0.0076747,-0.0521586,0.0601487},
{0.0143067,-0.0139409,0.0077633},
{-0.0057257,-0.0259946,0.0046822},
{-0.0071262,-0.0010722,0.0205354},
{-0.0100117,-0.0620832,0.0500186},
{-0.0061409,-0.0288847,0.0672136},
{0.0083405,-0.0070136,0.0613881},
{0.0134907,-0.0557568,0.0302855},
{-0.0019900,-0.0200044,-0.0123204},
{-0.0136674,-0.0269782,0.0558106},
{0.0158814,0.0146777,-0.0071703},
{-0.0040235,0.0237852,0.0037356},
{0.0006260,-0.0938003,0.0533542},
{-0.0128793,-0.0436085,0.0505282},
{0.0196908,-0.0308369,0.0429052},
{0.0114863,-0.0473354,0.0243913},
{0.0176729,0.0114571,0.0073001},
{0.0203674,0.0022946,0.0068187},
{0.0213204,-0.0021838,-0.0034513},
{0.0136572,-0.0221776,0.0227541},
{0.0113364,-0.0381748,0.0217249},
{0.0160890,-0.0161845,0.0325506},
{-0.0172052,-0.0539952,0.0267577},
{0.0029771,-0.0422093,0.0651577},
{0.0166452,-0.0497157,0.0387763},
{0.0143874,-0.0111497,0.0133920},

Biology Open • Supplementary information

{0.0010161,0.0048352,-0.0195979},
{0.0085866,-0.0008400,-0.0194489},
{0.0102841,-0.0741928,0.0541815},
{0.0146877,-0.0669979,0.0514703},
{0.0172516,-0.0650947,0.0427981},
{0.0174489,-0.0753575,0.0361938},
{-0.0046468,-0.0822202,0.0247699},
{-0.0130549,0.0041052,0.0140834},
{0.0075964,-0.0012335,0.0556140},
{0.0189005,-0.0102771,0.0506936},
{0.0147358,-0.0259752,0.0606583},
{-0.0117191,-0.0392708,0.0255251},
{-0.0180639,-0.0082243,-0.0025735},
{0.0160722,-0.0907942,0.0275894},
{-0.0068606,-0.0063004,-0.0191431},
{0.0040452,0.0154634,-0.0149142},
{-0.0091850,-0.0362442,0.0161037},
{-0.0146198,0.0113094,-0.0104787},
{-0.0198427,0.0054388,-0.0016724},
{-0.0089685,-0.0768246,0.0398016},
{0.0114857,-0.0190909,-0.0009548},
{0.0123616,-0.0497875,0.0577517},
{0.0085813,-0.0202416,-0.0090466},
{0.0099448,-0.0152443,-0.0138654},
{0.0042584,-0.0650652,0.0190711},
{0.0088665,-0.0555041,0.0216078},
{0.0012118,-0.0832089,0.0202021},
{-0.0180385,-0.0613924,0.0303920},
{-0.0059968,-0.0014882,0.0525102},
{-0.0130032,-0.0336891,0.0598954},
{0.0178068,-0.0189833,0.0544852},
{0.0148980,-0.0841928,0.0485904},
{0.0186638,-0.0262602,0.0353172},
{0.0083031,-0.0348408,0.0669876},
{-0.0169108,-0.0035548,0.0100003},
{0.0143404,-0.0176564,0.0606269},
{0.0154017,-0.0670244,0.0328450},
{-0.0151280,-0.0548629,0.0226914},
{-0.0139876,-0.0365088,0.0530051},
{0.0138309,-0.0455574,0.0307968},
{-0.0077837,-0.0423889,0.0648563},
{0.0066097,-0.0084729,-0.0189933},
{0.0041214,-0.0168827,-0.0152740},
{-0.0143451,-0.0118045,0.0518841},
{-0.0104054,-0.0489999,0.0583947},
{0.0173861,-0.0591715,0.0476443},

Biology Open • Supplementary information

{-0.0168352,0.0122253,0.0015811},
{-0.0137719,0.0100406,0.0117467},
{-0.0018993,-0.0744544,0.0552640},
{0.0132477,-0.0101691,0.0343908},
{0.0138496,-0.0038366,0.0555923},
{0.0152310,-0.0062889,0.0435655},
{-0.0127629,-0.0147341,-0.0086789},
{0.0057201,-0.0240487,0.0023746},
{0.0003685,-0.0537137,0.0172028},
{0.0200045,0.0034291,-0.0076676},
{0.0171465,0.0017792,-0.0132952},
{0.0127878,-0.0084507,-0.0159459},
{-0.0131241,0.0015038,-0.0156011},
{-0.0146545,-0.0065488,-0.0133683},
{0.0176895,0.0144973,0.0032382},
{0.0105699,0.0209040,-0.0023178},
{0.0090222,0.0152073,-0.0133606},
{0.0080621,-0.0247571,0.0651943},
{-0.0007109,-0.0094570,0.0643011},
{-0.0001658,-0.0022918,0.0579495},
{0.0053834,-0.0150331,0.0657801},
{0.0084060,0.0080541,0.0167315},
{0.0123377,-0.0190707,0.0158660},
{0.0039435,-0.0277060,0.0668890},
{-0.0091365,-0.0189373,0.0247086},
{-0.0102402,-0.0191396,0.0123343},
{-0.0064904,-0.0109612,0.0275903},
{-0.0151092,-0.0555911,0.0360941},
{-0.0113841,-0.0672036,0.0417783},
{-0.0069486,-0.0318976,0.0095507},
{0.0207692,-0.0202843,0.0471488},
{0.0122727,-0.0092796,0.0282368},
{0.0043368,-0.0010375,0.0247384},
{-0.0035961,-0.0833760,0.0537891},
{-0.0024131,0.0240586,-0.0033334},
{-0.0011469,-0.0144989,0.0398863},
{-0.0109735,-0.0157570,0.0025114},
{0.0130079,-0.0316219,0.0261429},
{0.0103649,-0.0298242,0.0174680},
{-0.0110442,-0.0076321,0.0536917},
{0.0188037,-0.0363898,0.0489068},
{-0.0121187,-0.0588083,0.0423637},
{-0.0074999,-0.0888850,0.0384067},
{0.0043498,-0.0057668,0.0314548},
{-0.0030001,-0.0050617,-0.0203555},
{-0.0126623,-0.0381906,0.0594460},

Biology Open • Supplementary information

{-0.0030217,0.0097835,-0.0172079},
{-0.0043389,0.0182402,-0.0127080},
{0.0186853,-0.0943518,0.0370267},
{0.0037077,-0.0813678,0.0194045},
{0.0165284,-0.0973488,0.0455178},
{0.0096267,0.0211063,0.0054858},
{0.0117920,0.0100617,0.0148603},
{0.0011031,0.0165780,-0.0145832},
{0.0161422,-0.0128558,-0.0090200},
{0.0190099,-0.0084089,-0.0077352},
{-0.0087346,-0.0267964,0.0310198},
{-0.0109470,-0.0381053,0.0327892},
{-0.0134555,-0.0680611,0.0262295},
{-0.0014213,0.0029363,0.0204860},
{0.0041698,0.0080793,0.0176737},
{-0.0025253,-0.0031478,0.0261714},
{0.0002577,-0.0340091,0.0663817},
{0.0069253,-0.0479422,0.0610919},
{-0.0117287,-0.0219209,0.0639958},
{-0.0117016,-0.0290702,0.0632111},
{0.0090599,0.0223685,0.0027007},
{0.0171376,0.0156242,0.0014330},
{-0.0071403,-0.0120540,0.0651568},
{0.0152564,-0.0587124,0.0350561},
{-0.0124338,-0.0525350,0.0490064},
{-0.0150384,-0.0557130,0.0224477},
{0.0109779,-0.0931255,0.0516131},
{-0.0098147,-0.0278826,0.0180591},
{-0.0101164,-0.0169792,0.0173639},
{-0.0044327,0.0176680,0.0128863},
{0.0008956,-0.0752826,0.0570597},
{0.0058957,-0.0694070,0.0572351},
{-0.0022611,-0.0690629,0.0567201},
{0.0165920,-0.0813832,0.0316812},
{-0.0124692,-0.0202059,0.0499270},
{0.0106806,-0.0274539,0.0175787},
{0.0051591,-0.0579811,0.0597532},
{0.0083124,-0.0387496,0.0635286},
{0.0095481,-0.0930718,0.0202423},
{-0.0067842,-0.0584005,0.0580163},
{0.0179416,-0.0472521,0.0448404},
{-0.0119284,-0.0472327,0.0215667},
{0.0212043,-0.0227507,0.0381461},
{-0.0040318,-0.0672421,0.0206887},
{0.0166592,-0.0021959,0.0130134},
{0.0061764,0.0059577,0.0178259},

Biology Open • Supplementary information

{0.0009165,-0.0357544,0.0662616},
{-0.0148318,0.0151219,-0.0051698},
{0.0106136,0.0172730,0.0103526},
{-0.0106773,0.0176239,0.0083069},
{-0.0186194,-0.0060483,-0.0052598},
{-0.0187303,0.0005594,-0.0075449},
{-0.0049037,-0.0192430,0.0676053},
{-0.0131568,-0.0377586,0.0443264},
{0.0055901,-0.0515212,0.0610631},
{0.0124419,-0.0457572,0.0587780},
{-0.0087462,-0.0750584,0.0258513},
{0.0126392,0.0077884,0.0153033},
{0.0182435,-0.0117500,-0.0011424},
{-0.0173344,-0.0088283,0.0050922},
{-0.0127829,-0.0617579,0.0392799},
{-0.0073173,-0.0184845,-0.0037804},
{-0.0129437,-0.0302275,0.0475121},
{0.0046558,-0.0821253,0.0554826},
{0.0159841,-0.0547427,0.0523062},
{-0.0017364,-0.0382178,0.0111222},
{0.0062515,-0.0350960,0.0132571},
{0.0187856,-0.0076404,0.0058298},
{0.0181390,-0.0892677,0.0420584},
{-0.0091390,-0.0207491,0.0253212},
{-0.0008794,0.0142003,0.0162053},
{0.0074091,-0.0747980,0.0213438},
{0.0088001,-0.0683517,0.0565856},
{0.0150310,-0.0142059,-0.0094991},
{0.0005563,-0.0099727,0.0647803},
{-0.0071553,-0.0161555,-0.0142424},
{0.0159059,-0.0772208,0.0472464},
{0.0076936,-0.0201712,-0.0103955},
{-0.0106216,-0.0706050,0.0233390},
{0.0133203,-0.0430590,0.0291645},
{0.0155262,-0.0046839,0.0541312},
{0.0047469,-0.0083636,0.0394578},
{-0.0030379,-0.0676160,0.0572801},
{-0.0128107,-0.0651717,0.0382525},
{-0.0144619,-0.0125049,0.0541672},
{-0.0078637,0.0134268,-0.0146852},
{-0.0171780,0.0063314,0.0081686},
{0.0093526,-0.0745041,0.0222516},
{0.0024951,-0.0425329,0.0642430},
{-0.0135095,-0.0237088,0.0551025},
{0.0064427,-0.0067057,-0.0196962},
{0.0154119,-0.0308805,0.0566410},

{0.0119687,-0.0163625,0.0621528},
{0.0104243,0.0022594,-0.0181417},
{0.0137128,-0.0088439,0.0172285},
{-0.0129452,0.0120600,0.0117175},
{0.0103630,-0.0572188,0.0576950},
{-0.0054678,-0.0415522,0.0656820},
{0.0194496,-0.0314419,0.0501629},
{0.0158525,-0.0143119,-0.0070263},
{-0.0149265,-0.0542918,0.0365964},
{-0.0075540,-0.0797169,0.0453720},
{0.0032005,-0.0568892,0.0604941},
{-0.0044159,-0.0447634,0.0140694},
{0.0111401,0.0115522,-0.0146686},
{-0.0021439,-0.0233462,-0.0008475},
{-0.0091704,-0.0791858,0.0303455},
{-0.0103319,-0.0037825,-0.0176411},
{-0.0029171,0.0234052,-0.0046929},
{-0.0095908,-0.0234480,0.0133214},
{-0.0107450,-0.0695604,0.0231607},
{-0.0059490,-0.0806769,0.0249357},
{0.0009535,0.0127984,-0.0167812},
{0.0198530,-0.0280509,0.0493913},
{-0.0058979,-0.0626016,0.0200093},
{0.0176390,-0.0391839,0.0399166},
{0.0176135,-0.0332152,0.0355635},
{-0.0143673,-0.0129049,-0.0009856},
{0.0132790,0.0151206,0.0102064},
{-0.0133956,-0.0117695,0.0070100},
{-0.0107050,-0.0379612,0.0361861},
{0.0179713,-0.0946177,0.0429545},
{0.0176946,-0.0111412,0.0563859},
{0.0187814,-0.0167753,0.0522373},
{0.0013630,-0.0285419,0.0050847},
{0.0108016,-0.0251690,0.0636150},
{-0.0027924,-0.0102777,0.0317929},
{-0.0192987,-0.0045710,-0.0017603},
{0.0027607,-0.0226297,-0.0038575},
{-0.0096950,-0.0765823,0.0374183},
{-0.0127523,-0.0295814,0.0616836},
{0.0179110,0.0055852,-0.0108969},
{-0.0147865,-0.0670016,0.0305003},
{-0.0152489,-0.0045181,0.0129514},
{0.0120076,-0.0086463,0.0273876},
{-0.0134453,-0.0460897,0.0324397},
{0.0150640,-0.0769723,0.0291432},
{0.0007702,0.0036234,-0.0198537},

Biology Open • Supplementary information

{0.0169002,-0.0833279,0.0317986},
{0.0069496,0.0068603,0.0170804},
{-0.0143007,0.0024293,0.0131144},
{0.0058567,-0.0461345,0.0168121},
{-0.0064822,-0.0204487,0.0344565},
{0.0205731,-0.0289703,0.0435241},
{-0.0129203,-0.0672663,0.0232885},
{0.0208788,0.0054951,-0.0030845},
{-0.0138919,-0.0674444,0.0283763},
{0.0112783,-0.0240345,0.0113129},
{0.0197627,-0.0125623,0.0464317},
{0.0086730,0.0020901,-0.0191008},
{0.0147724,-0.0089213,0.0138462},
{-0.0113565,-0.0682676,0.0411192},
{0.0004639,0.0221175,0.0094276},
{0.0028461,0.0035481,-0.0201791},
{0.0377999,-0.4329396,0.0215635},
{0.0051227,0.0219159,-0.0079895},
{-0.0136876,-0.0483234,0.0382956},
{-0.0318095,-0.4285551,-0.0276310},
{0.0023480,0.0247939,-0.0000328},
{0.0145629,-0.0263425,0.0273079},
{-0.0143046,-0.0011812,-0.0149230},
{0.0167744,-0.0332640,0.0544341},
{-0.0148491,0.0144375,0.0048712},
{0.0174880,-0.0430328,0.0411366},
{0.0156074,-0.0480073,0.0352847},
{-0.0134490,-0.0378672,0.0470238},
{0.0175606,-0.0608054,0.0452570},
{-0.0169503,-0.0528391,0.0282430},
{0.0030970,-0.0603672,0.0597241},
{0.0011021,-0.0563145,0.0178364},
{0.0216970,-0.0229462,0.0452427},
{0.0080107,-0.0838514,0.0541378},
{-0.0060270,-0.0910346,0.0286964},
{-0.0192289,-0.0023625,0.0031368},
{0.0145752,-0.0147085,0.0202779},
{0.0140184,-0.0730861,0.0282154},
{-0.0094107,-0.0270948,0.0282586},
{-0.0197427,-0.4393912,0.0366655},
{-0.0087605,-0.0611913,0.0535802},
{-0.0115920,-0.4334508,-0.0105552},
{0.0108039,-0.0474409,0.0229987},
{0.0109803,0.0159232,-0.0114931},
{-0.0246509,-0.4298741,0.0296677},
{0.0158259,-0.0582816,0.0359608},

Biology Open • Supplementary information

{0.0003019,-0.0543464,0.0614576},
{-0.0111091,0.0198424,0.0029203},
{-0.0024956,-0.0183125,0.0674620},
{-0.0073777,-0.0080817,0.0247587},
{0.0195986,-0.0113015,0.0433364},
{0.0150089,-0.0268597,0.0587113},
{-0.0142915,-0.0119615,0.0534159},
{0.0200312,0.0090323,-0.0028811},
{-0.0101320,-0.0374173,0.0184003},
{-0.0003404,-0.0154887,-0.0168718},
{-0.0108903,-0.0660538,0.0453291},
{-0.0091004,0.0187801,0.0082302},
{0.0098650,0.0209930,-0.0030799},
{-0.0135372,-0.0296524,0.0584966},
{-0.0060438,-0.4304097,0.0415604},
{-0.0135723,-0.4362616,-0.0389532},
{-0.0134970,-0.4362030,-0.0381480},
{-0.0134217,-0.4361445,-0.0373429},
{-0.0133464,-0.4360860,-0.0365378},
{-0.0132712,-0.4360275,-0.0357326},
{-0.0131959,-0.4359690,-0.0349275},
{-0.0131206,-0.4359105,-0.0341224},
{-0.0130453,-0.4358519,-0.0333172},
{-0.0129700,-0.4357934,-0.0325121},
{-0.0128947,-0.4357349,-0.0317070},
{-0.0128194,-0.4356764,-0.0309018},
{-0.0127442,-0.4356179,-0.0300967},
{-0.0126689,-0.4355593,-0.0292915},
{-0.0125936,-0.4355008,-0.0284864},
{-0.0125183,-0.4354423,-0.0276813},
{-0.0124430,-0.4353838,-0.0268761},
{-0.0123677,-0.4353253,-0.0260710},
{-0.0122925,-0.4352668,-0.0252659},
{-0.0122172,-0.4352082,-0.0244607},
{-0.0121419,-0.4351497,-0.0236556},
{-0.0120666,-0.4350912,-0.0228505},
{-0.0119913,-0.4350327,-0.0220453},
{-0.0119160,-0.4349742,-0.0212402},
{-0.0118408,-0.4349156,-0.0204351},
{-0.0117655,-0.4348571,-0.0196299},
{-0.0116902,-0.4347986,-0.0188248},
{-0.0116149,-0.4347401,-0.0180197},
{-0.0115396,-0.4346816,-0.0172145},
{-0.0114643,-0.4346231,-0.0164094},
{-0.0113890,-0.4345645,-0.0156042},
{-0.0113138,-0.4345060,-0.0147991},

Biology Open • Supplementary information

{-0.0112385,-0.4344475,-0.0139940},
{-0.0111632,-0.4343890,-0.0131888},
{-0.0110879,-0.4343305,-0.0123837},
{-0.0110126,-0.4342719,-0.0115786},
{-0.0109373,-0.4342134,-0.0107734},
{-0.0108621,-0.4341549,-0.0099683},
{-0.0107868,-0.4340964,-0.0091632},
{-0.0107115,-0.4340379,-0.0083580},
{-0.0106362,-0.4339794,-0.0075529},
{-0.0105609,-0.4339208,-0.0067478},
{-0.0104856,-0.4338623,-0.0059426},
{-0.0104103,-0.4338038,-0.0051375},
{-0.0103351,-0.4337453,-0.0043324},
{-0.0102598,-0.4336868,-0.0035272},
{-0.0101845,-0.4336282,-0.0027221},
{-0.0101092,-0.4335697,-0.0019169},
{-0.0100339,-0.4335112,-0.0011118},
{-0.0099586,-0.4334527,-0.0003067},
{-0.0098834,-0.4333942,0.0004985},
{-0.0098081,-0.4333357,0.0013036},
{-0.0097328,-0.4332771,0.0021087},
{-0.0096575,-0.4332186,0.0029139},
{-0.0095822,-0.4331601,0.0037190},
{-0.0095069,-0.4331016,0.0045241},
{-0.0094316,-0.4330431,0.0053293},
{-0.0093564,-0.4329845,0.0061344},
{-0.0092811,-0.4329260,0.0069395},
{-0.0092058,-0.4328675,0.0077447},
{-0.0091305,-0.4328090,0.0085498},
{-0.0090552,-0.4327505,0.0093549},
{-0.0089799,-0.4326919,0.0101601},
{-0.0089047,-0.4326334,0.0109652},
{-0.0088294,-0.4325749,0.0117704},
{-0.0087541,-0.4325164,0.0125755},
{-0.0086788,-0.4324579,0.0133806},
{-0.0086035,-0.4323994,0.0141858},
{-0.0085282,-0.4323408,0.0149909},
{-0.0084530,-0.4322823,0.0157960},
{-0.0083777,-0.4322238,0.0166012},
{-0.0083024,-0.4321653,0.0174063},
{-0.0082271,-0.4321068,0.0182114},
{-0.0081518,-0.4320482,0.0190166},
{-0.0080765,-0.4319897,0.0198217},
{-0.0080012,-0.4319312,0.0206268},
{-0.0079260,-0.4318727,0.0214320},
{-0.0078507,-0.4318142,0.0222371},

Biology Open • Supplementary information

```
                    {-0.0077754,-0.4317557,0.0230423},
                    {-0.0077001,-0.4316971,0.0238474},
                    {-0.0076248,-0.4316386,0.0246525},
                    {-0.0075495,-0.4315801,0.0254577},
                    {-0.0074743,-0.4315216,0.0262628},
                    {-0.0073990,-0.4314631,0.0270679},
                    {-0.0073237,-0.4314045,0.0278731},
                    {-0.0072484,-0.4313460,0.0286782},
                    {-0.0071731,-0.4312875,0.0294833},
                    {-0.0070978,-0.4312290,0.0302885},
                    {-0.0070225,-0.4311705,0.0310936},
                    {-0.0069473,-0.4311120,0.0318987},
                    {-0.0068720,-0.4310534,0.0327039},
                    {-0.0067967,-0.4309949,0.0335090},
                    {-0.0067214,-0.4309364,0.0343141},
                    {-0.0066461,-0.4308779,0.0351193},
                    {-0.0065708,-0.4308194,0.0359244},
                    {-0.0064956,-0.4307608,0.0367296},
                    {-0.0064203,-0.4307023,0.0375347},
                    {-0.0063450,-0.4306438,0.0383398},
                    {-0.0062697,-0.4305853,0.0391450},
                    {-0.0061944,-0.4305268,0.0399501},
                    {-0.0061191,-0.4304683,0.0407552}
                    };
        BoundingBoxOnOff = Off;
    };
    AnyFunTransform3DIdentity ScaleFunction = {
        PreTransforms = {&.RBFTransform};
};
};

};
};
```

Biology Open • Supplementary information

ScalingFunctionTLEMLucyFemur_Mirrored_2014040

```
AnyFolder ScalingFunctionTLEMLucyFemur_Mirrored = {
  AnyFolder Left = {
          AnyFolder Thigh = {
        AnyMatrix AMirroring = {
        {1,0,0},
        {0,1,0},
        {0,0,-1}
        };
          AnyFunTransform3DRBF RBFTransform = {
                  RBFDef.Type = RBF_ThinPlate;
                  PolynomDegree = 1;
                  Points0 = ({
                          {0.0000000,0.0000000,0.0000000},
                          {-0.0000000,-0.3616821,0.0000000},
                          {-0.0097563,-0.3678799,0.0012967},
                          {-0.0000000,-0.3660632,0.0408203},
                          {-0.0000000,-0.3573010,-0.0408203},
                          {0.0161460,-0.0072838,0.0601290},
                          {0.0220217,-0.0203698,0.0463848},
                          {0.0123977,-0.0241932,0.0668573},
                          {0.0006898,0.0018121,0.0538181},
                          {0.0122809,-0.0068668,0.0414535},
                          {-0.0058991,-0.0138188,0.0648412},
                          {0.0211469,-0.0380855,0.0538111},
                          {0.0172133,-0.0317342,0.0328381},
                          {0.0177311,-0.0131946,0.0256176},
                          {-0.0110079,-0.0308867,0.0632370},
                          {0.0039435,-0.0395977,0.0638271},
                          {-0.0041683,-0.0187472,0.0413214},
                          {-0.0177999,-0.0021535,0.0496084},
                          {0.0001603,-0.0053126,0.0279593},
                          {-0.0211600,-0.0216243,0.0497147},
                          {0.0096163,-0.0568663,0.0545719},
                          {0.0162112,-0.0515013,0.0395230},
                          {0.0093533,-0.0433265,0.0187387},
                          {0.0119398,-0.0238469,0.0118403},
                          {0.0210707,-0.0048848,0.0065878},
                          {0.0125013,0.0064094,0.0177702},
                          {-0.0191523,-0.0356053,0.0382429},
                          {-0.0115821,-0.0474937,0.0537648},
                          {-0.0100073,-0.0236371,0.0267322},
                          {-0.0078925,0.0107098,0.0182833},
                          {-0.0134217,-0.0055356,0.0182000},
                          {-0.0090061,-0.0665700,0.0475484},
```

Biology Open • Supplementary information

{0.0071095,-0.0761976,0.0520035},
{0.0182119,-0.0704149,0.0386546},
{0.0073764,-0.0609481,0.0221006},
{-0.0122984,-0.0439585,0.0198428},
{-0.0032253,-0.0306792,0.0110995},
{-0.0012975,-0.0198451,-0.0052592},
{0.0140405,-0.0161125,-0.0071649},
{0.0189222,-0.0014898,-0.0127226},
{0.0181642,0.0132812,-0.0011050},
{0.0045119,0.0200001,0.0091189},
{-0.0176761,-0.0541983,0.0348587},
{-0.0136548,-0.0142015,0.0054311},
{-0.0135510,0.0182940,0.0017175},
{-0.0223865,0.0023399,0.0028018},
{-0.0086455,-0.0738247,0.0283843},
{-0.0050356,-0.0877749,0.0442195},
{0.0160135,-0.0919319,0.0465120},
{0.0128290,-0.0785810,0.0232431},
{-0.0168328,-0.0616327,0.0155917},
{-0.0172369,-0.0094567,-0.0119502},
{0.0002090,-0.0093641,-0.0209779},
{0.0064827,0.0081538,-0.0198453},
{0.0010777,0.0210422,-0.0084721},
{-0.0129485,0.0085451,-0.0162212},
{-0.0002322,-0.0924033,0.0245644},
{-0.0028175,-0.1052263,0.0418294},
{0.0172322,-0.1111444,0.0445760},
{0.0203259,-0.0957409,0.0271449},
{0.0057102,-0.1088933,0.0195113},
{-0.0010852,-0.1230776,0.0363132},
{0.0182727,-0.1303312,0.0422884},
{0.0242891,-0.1161703,0.0265054},
{0.0094531,-0.1268584,0.0162294},
{0.0014244,-0.1413293,0.0331653},
{0.0207271,-0.1496427,0.0394157},
{0.0267562,-0.1360142,0.0236798},
{0.0113839,-0.1461651,0.0136024},
{0.0030845,-0.1595094,0.0297156},
{0.0209528,-0.1691386,0.0369775},
{0.0283958,-0.1540209,0.0213795},
{0.0129499,-0.1649012,0.0113589},
{0.0016490,-0.1783108,0.0216287},
{0.0147105,-0.1880678,0.0328614},
{0.0298491,-0.1859865,0.0248551},
{0.0286645,-0.1714377,0.0169488},
{0.0165897,-0.1861006,0.0082595},

{0.0014000,-0.1973476,0.0183740},
{0.0114301,-0.2109792,0.0286157},
{0.0267422,-0.2037622,0.0296951},
{0.0288630,-0.2018335,0.0130127},
{0.0145471,-0.2083505,0.0062833},
{0.0011300,-0.2198071,0.0155602},
{0.0126610,-0.2321235,0.0275354},
{0.0275647,-0.2219268,0.0265185},
{0.0286645,-0.2230253,0.0098701},
{0.0130434,-0.2304822,0.0039177},
{0.0022984,-0.2409585,0.0149650},
{0.0101315,-0.2534740,0.0252125},
{0.0279485,-0.2428555,0.0234276},
{0.0276684,-0.2421559,0.0058691},
{0.0101250,-0.2529527,0.0018983},
{0.0021376,-0.2667831,0.0107073},
{0.0114309,-0.2734905,0.0249960},
{0.0273923,-0.2634953,0.0216324},
{0.0278429,-0.2592546,0.0037692},
{0.0159101,-0.2712985,-0.0033988},
{0.0036708,-0.2863280,0.0004644},
{0.0023115,-0.2884756,0.0187554},
{0.0231385,-0.2879051,0.0224853},
{0.0304009,-0.2799973,0.0083549},
{0.0238189,-0.2917246,-0.0052462},
{0.0064904,-0.3027687,-0.0070587},
{-0.0003492,-0.3064690,0.0093014},
{0.0122179,-0.3045269,0.0243511},
{0.0301837,-0.3030127,0.0114462},
{0.0234182,-0.3107336,-0.0085198},
{0.0046893,-0.3214995,-0.0123881},
{-0.0022352,-0.3264905,0.0043340},
{0.0016841,-0.3202756,0.0217839},
{0.0229722,-0.3196887,0.0215083},
{0.0292438,-0.3228042,0.0051430},
{0.0221641,-0.3296190,-0.0132361},
{0.0100459,-0.3417597,-0.0244560},
{-0.0053009,-0.3375737,-0.0137705},
{-0.0064432,-0.3487307,0.0028676},
{-0.0059346,-0.3407801,0.0202938},
{0.0105130,-0.3365976,0.0264175},
{0.0282695,-0.3386249,0.0180307},
{0.0270402,-0.3431844,-0.0001487},
{0.0257040,-0.3506950,-0.0181851},
{0.0178948,-0.3636114,-0.0302838},
{0.0043149,-0.3544109,-0.0381949},

Biology Open • Supplementary information

{-0.0095210,-0.3461482,-0.0295031},
{-0.0164526,-0.3542895,-0.0126666},
{-0.0060896,-0.3690127,-0.0033061},
{-0.0176523,-0.3612686,0.0110109},
{-0.0153310,-0.3546943,0.0299634},
{0.0021329,-0.3508021,0.0350763},
{0.0210690,-0.3548628,0.0279835},
{0.0356594,-0.3558162,0.0159356},
{0.0275718,-0.3617450,-0.0018607},
{0.0298503,-0.3741530,-0.0163721},
{0.0175320,-0.3855300,-0.0277515},
{0.0025604,-0.3740099,-0.0397137},
{-0.0136727,-0.3624016,-0.0402102},
{-0.0277647,-0.3567472,-0.0284947},
{-0.0275446,-0.3699630,-0.0120829},
{-0.0175846,-0.3859355,-0.0177922},
{0.0001572,-0.3856873,-0.0118050},
{0.0030262,-0.3834856,0.0075553},
{-0.0144114,-0.3810887,0.0140304},
{-0.0247706,-0.3701239,0.0285134},
{-0.0044804,-0.3677201,0.0404632},
{0.0133183,-0.3712274,0.0334208},
{0.0324861,-0.3723417,0.0245668},
{0.0271451,-0.3766027,0.0072441},
{0.0167985,-0.3822816,-0.0065349},
{-0.0001454,-0.3910823,-0.0303010},
{-0.0144998,-0.3834154,-0.0384137},
{-0.0301321,-0.3750841,-0.0321336},
{0.0196491,-0.3864804,0.0210853},
{0.0015726,-0.3888034,0.0276569},
{-0.0146652,-0.3847848,0.0336913},
{-0.0014588,-0.0619951,0.0219778},
{-0.0118435,-0.0550626,0.0139639},
{0.0129812,-0.0349966,0.0673128},
{-0.0162907,-0.0105666,0.0597739},
{-0.0012885,-0.0520816,0.0562989},
{-0.0090599,-0.0631298,0.0172224},
{-0.0000222,-0.0771663,0.0231897},
{-0.0068148,0.0023885,-0.0160086},
{-0.0180544,-0.0425409,0.0446585},
{0.0020525,-0.0249904,0.0691549},
{-0.0157097,-0.0662315,0.0238393},
{-0.0010518,-0.0510686,0.0199353},
{-0.0208874,-0.0152865,0.0500034},
{0.0168316,-0.0280857,0.0666680},
{-0.0187392,-0.0282333,0.0403282},

Biology Open • Supplementary information

{0.0191622,-0.0351633,0.0622707},
{0.0001216,-0.0706289,0.0524049},
{-0.0169862,-0.0399062,0.0311827},
{-0.0185737,-0.0533066,0.0151224},
{-0.0219058,-0.0528723,0.0222263},
{-0.0028271,-0.0391620,0.0148452},
{0.0117900,-0.0553425,0.0235348},
{0.0219835,-0.0106400,0.0396338},
{-0.0025366,-0.0401046,0.0625273},
{0.0141017,-0.0018767,0.0198108},
{-0.0078770,-0.0473929,0.0173542},
{-0.0065351,-0.0766431,0.0470258},
{0.0065585,0.0145593,0.0161638},
{-0.0118588,0.0172875,-0.0085064},
{-0.0074087,-0.0627500,0.0506700},
{0.0071940,-0.0780068,0.0213362},
{0.0126736,-0.0708176,0.0244108},
{0.0074694,-0.0436450,0.0604196},
{0.0154186,-0.0139471,0.0064312},
{-0.0053535,-0.0213210,0.0040094},
{-0.0088733,0.0002018,0.0205402},
{-0.0112471,-0.0542608,0.0500266},
{-0.0061625,-0.0248673,0.0653244},
{0.0093695,-0.0065826,0.0614157},
{0.0138015,-0.0467322,0.0284333},
{-0.0020999,-0.0186885,-0.0116392},
{-0.0185640,-0.0225591,0.0566779},
{0.0175798,0.0124360,-0.0068467},
{-0.0039962,0.0217127,0.0053055},
{0.0014975,-0.0784591,0.0514056},
{-0.0172997,-0.0386971,0.0525252},
{0.0196540,-0.0268271,0.0416806},
{0.0135296,-0.0406504,0.0236882},
{0.0182471,0.0095959,0.0087906},
{0.0218667,0.0016804,0.0058793},
{0.0224975,-0.0022652,-0.0022994},
{0.0166750,-0.0191391,0.0236436},
{0.0119785,-0.0328723,0.0195376},
{0.0187631,-0.0133469,0.0308741},
{-0.0187543,-0.0461463,0.0257491},
{0.0039834,-0.0373928,0.0683089},
{0.0157029,-0.0423169,0.0385226},
{0.0164003,-0.0087069,0.0131181},
{0.0008253,0.0033370,-0.0212576},
{0.0101707,-0.0009724,-0.0205688},
{0.0102948,-0.0642683,0.0524532},

Biology Open • Supplementary information

{0.0152217,-0.0557944,0.0508978},
{0.0172973,-0.0565707,0.0419468},
{0.0167723,-0.0635334,0.0369834},
{-0.0047397,-0.0695325,0.0241398},
{-0.0152913,0.0035778,0.0161461},
{0.0091697,0.0009340,0.0575160},
{0.0196686,-0.0089533,0.0500006},
{0.0183998,-0.0212943,0.0622968},
{-0.0114340,-0.0316810,0.0250940},
{-0.0206442,-0.0092599,-0.0028425},
{0.0166868,-0.0784295,0.0276925},
{-0.0069678,-0.0054521,-0.0207011},
{0.0049490,0.0151109,-0.0160093},
{-0.0084025,-0.0318937,0.0162788},
{-0.0173679,0.0095594,-0.0108509},
{-0.0218912,0.0063457,-0.0020698},
{-0.0106547,-0.0668750,0.0397749},
{0.0136098,-0.0170666,-0.0018890},
{0.0141599,-0.0441405,0.0587594},
{0.0093255,-0.0183218,-0.0083008},
{0.0106367,-0.0128276,-0.0154269},
{0.0037350,-0.0549365,0.0209786},
{0.0087662,-0.0472902,0.0195496},
{0.0016839,-0.0708213,0.0221325},
{-0.0202977,-0.0530540,0.0301270},
{-0.0068584,0.0021781,0.0530002},
{-0.0144817,-0.0297218,0.0605833},
{0.0197015,-0.0165217,0.0560654},
{0.0156609,-0.0705363,0.0477736},
{0.0207876,-0.0221433,0.0330733},
{0.0092324,-0.0300767,0.0713160},
{-0.0203392,-0.0034488,0.0097332},
{0.0156978,-0.0143438,0.0629053},
{0.0143267,-0.0563519,0.0317489},
{-0.0161685,-0.0465391,0.0188591},
{-0.0196397,-0.0309875,0.0546496},
{0.0154804,-0.0380422,0.0305433},
{-0.0077774,-0.0351303,0.0637765},
{0.0063980,-0.0086419,-0.0201654},
{0.0030146,-0.0151094,-0.0165974},
{-0.0210080,-0.0096030,0.0507855},
{-0.0105684,-0.0411377,0.0586146},
{0.0180619,-0.0498960,0.0469835},
{-0.0192318,0.0116108,0.0024070},
{-0.0165651,0.0083622,0.0126870},
{-0.0014333,-0.0627138,0.0534746},

Biology Open • Supplementary information

{0.0145127,-0.0080178,0.0353667},
{0.0148374,-0.0012228,0.0566181},
{0.0153834,-0.0062427,0.0444727},
{-0.0155164,-0.0132103,-0.0096398},
{0.0068685,-0.0221022,0.0011547},
{0.0003332,-0.0458500,0.0183716},
{0.0205582,0.0040956,-0.0082969},
{0.0175752,0.0009340,-0.0148407},
{0.0142357,-0.0063623,-0.0169332},
{-0.0147285,0.0023412,-0.0164678},
{-0.0158781,-0.0064451,-0.0151346},
{0.0175951,0.0133456,0.0037559},
{0.0117660,0.0189899,-0.0030690},
{0.0096040,0.0151827,-0.0137450},
{0.0096565,-0.0215194,0.0662734},
{-0.0006793,-0.0074494,0.0631218},
{0.0007819,-0.0004519,0.0600781},
{0.0065377,-0.0125433,0.0654199},
{0.0090461,0.0073096,0.0188711},
{0.0151418,-0.0168302,0.0146465},
{0.0025166,-0.0231863,0.0688894},
{-0.0097891,-0.0152077,0.0253086},
{-0.0109149,-0.0177935,0.0119825},
{-0.0072079,-0.0099373,0.0272725},
{-0.0183727,-0.0467429,0.0356276},
{-0.0126871,-0.0584555,0.0428141},
{-0.0057054,-0.0273042,0.0103554},
{0.0215142,-0.0159693,0.0474531},
{0.0128492,-0.0070016,0.0287645},
{0.0052134,-0.0017047,0.0247463},
{-0.0024284,-0.0707498,0.0513049},
{-0.0036140,0.0220593,-0.0040484},
{-0.0002405,-0.0121253,0.0390050},
{-0.0119811,-0.0156737,0.0015445},
{0.0142082,-0.0279792,0.0246553},
{0.0122244,-0.0262013,0.0169829},
{-0.0146264,-0.0038855,0.0549384},
{0.0207450,-0.0325771,0.0496412},
{-0.0155040,-0.0497560,0.0437087},
{-0.0081119,-0.0756450,0.0387365},
{0.0039684,-0.0062047,0.0313900},
{-0.0023726,-0.0038892,-0.0220521},
{-0.0136977,-0.0339839,0.0602619},
{-0.0035399,0.0089081,-0.0188894},
{-0.0049820,0.0176571,-0.0132254},
{0.0193902,-0.0803191,0.0360429},

Biology Open • Supplementary information

{0.0040648,-0.0697109,0.0218205},
{0.0170470,-0.0818610,0.0456892},
{0.0104175,0.0188635,0.0064965},
{0.0135206,0.0092549,0.0158079},
{0.0023933,0.0160498,-0.0157404},
{0.0164753,-0.0118197,-0.0096995},
{0.0202584,-0.0066858,-0.0082383},
{-0.0098401,-0.0226001,0.0314028},
{-0.0130937,-0.0309622,0.0320764},
{-0.0201510,-0.0613297,0.0222264},
{-0.0010389,0.0036055,0.0209003},
{0.0042025,0.0073392,0.0199393},
{-0.0021630,-0.0038531,0.0251898},
{-0.0007845,-0.0293574,0.0675788},
{0.0070027,-0.0406327,0.0624565},
{-0.0124363,-0.0183001,0.0627966},
{-0.0120485,-0.0256613,0.0628493},
{0.0101200,0.0197695,0.0039334},
{0.0167636,0.0148266,0.0014155},
{-0.0074981,-0.0106930,0.0632797},
{0.0142533,-0.0503445,0.0351954},
{-0.0146107,-0.0460953,0.0506120},
{-0.0161117,-0.0471301,0.0178900},
{0.0112149,-0.0792793,0.0501460},
{-0.0099060,-0.0230794,0.0186951},
{-0.0114543,-0.0153049,0.0183675},
{-0.0042882,0.0161879,0.0151423},
{0.0014582,-0.0638572,0.0541080},
{0.0048554,-0.0582603,0.0553815},
{-0.0017077,-0.0572302,0.0546635},
{0.0166090,-0.0697169,0.0323038},
{-0.0208812,-0.0164273,0.0474354},
{0.0130454,-0.0241864,0.0178294},
{0.0053123,-0.0483770,0.0582251},
{0.0112225,-0.0361429,0.0673300},
{0.0085729,-0.0771256,0.0216100},
{-0.0070783,-0.0488033,0.0560869},
{0.0182083,-0.0411024,0.0454236},
{-0.0119590,-0.0403809,0.0213546},
{0.0233409,-0.0185535,0.0366343},
{-0.0046347,-0.0571211,0.0208243},
{0.0183166,-0.0006966,0.0140116},
{0.0061913,0.0053512,0.0201003},
{-0.0002364,-0.0310828,0.0680351},
{-0.0170773,0.0139270,-0.0054987},
{0.0110805,0.0156034,0.0117010},

Biology Open • Supplementary information

{-0.0124628,0.0164667,0.0094550},
{-0.0209014,-0.0073407,-0.0051969},
{-0.0210131,0.0010693,-0.0086891},
{-0.0041596,-0.0172332,0.0663041},
{-0.0216595,-0.0322172,0.0451540},
{0.0051908,-0.0426014,0.0613013},
{0.0150003,-0.0416136,0.0606257},
{-0.0135090,-0.0674234,0.0236425},
{0.0146416,0.0073691,0.0160052},
{0.0195615,-0.0112412,-0.0010333},
{-0.0198115,-0.0098244,0.0044735},
{-0.0157054,-0.0521236,0.0395039},
{-0.0081381,-0.0173108,-0.0046152},
{-0.0218324,-0.0262661,0.0471115},
{0.0051446,-0.0707257,0.0530555},
{0.0179359,-0.0471493,0.0528134},
{-0.0005591,-0.0317983,0.0113632},
{0.0071707,-0.0305943,0.0121186},
{0.0206239,-0.0061367,0.0066642},
{0.0187057,-0.0745868,0.0420207},
{-0.0095784,-0.0163113,0.0259524},
{-0.0012181,0.0127661,0.0186855},
{0.0059420,-0.0630691,0.0220087},
{0.0069408,-0.0573878,0.0552614},
{0.0150782,-0.0132884,-0.0101793},
{0.0005533,-0.0078574,0.0633775},
{-0.0088284,-0.0146399,-0.0147837},
{0.0167444,-0.0651105,0.0470347},
{0.0082666,-0.0181741,-0.0095133},
{-0.0175162,-0.0637444,0.0177069},
{0.0153795,-0.0360767,0.0287608},
{0.0162363,-0.0018864,0.0548412},
{0.0052731,-0.0075919,0.0392990},
{-0.0022288,-0.0556644,0.0549909},
{-0.0156238,-0.0550038,0.0377688},
{-0.0204137,-0.0100550,0.0536429},
{-0.0093683,0.0122429,-0.0162013},
{-0.0202927,0.0049166,0.0083887},
{0.0074706,-0.0627945,0.0222133},
{0.0035034,-0.0376103,0.0672421},
{-0.0190752,-0.0194129,0.0555530},
{0.0061758,-0.0074961,-0.0207741},
{0.0192011,-0.0273063,0.0588233},
{0.0130796,-0.0134383,0.0638611},
{0.0120421,0.0019566,-0.0194140},
{0.0154152,-0.0065294,0.0179581},

Biology Open • Supplementary information

{-0.0157335,0.0101219,0.0123981},
{0.0110376,-0.0488274,0.0567911},
{-0.0055170,-0.0343676,0.0647140},
{0.0216204,-0.0273988,0.0512041},
{0.0161331,-0.0135875,-0.0075501},
{-0.0184643,-0.0455633,0.0360087},
{-0.0091978,-0.0682413,0.0460847},
{0.0034304,-0.0471677,0.0588866},
{-0.0048025,-0.0384316,0.0149400},
{0.0118505,0.0109735,-0.0159161},
{-0.0027036,-0.0204189,-0.0015094},
{-0.0117607,-0.0692759,0.0292171},
{-0.0107080,-0.0033455,-0.0190487},
{-0.0042548,0.0214947,-0.0056846},
{-0.0097315,-0.0208529,0.0131997},
{-0.0178958,-0.0627218,0.0171551},
{-0.0068692,-0.0688818,0.0233316},
{0.0012083,0.0120695,-0.0186179},
{0.0215073,-0.0236027,0.0503069},
{-0.0060262,-0.0534931,0.0189523},
{0.0179951,-0.0336785,0.0397267},
{0.0190509,-0.0284972,0.0336394},
{-0.0169188,-0.0133609,-0.0014203},
{0.0135722,0.0136491,0.0114695},
{-0.0150549,-0.0123230,0.0067722},
{-0.0148411,-0.0311945,0.0355412},
{0.0185490,-0.0794042,0.0431266},
{0.0193568,-0.0087326,0.0575704},
{0.0201026,-0.0144353,0.0531586},
{0.0025583,-0.0248211,0.0045714},
{0.0132689,-0.0214866,0.0652899},
{-0.0028669,-0.0099311,0.0309271},
{-0.0219487,-0.0054893,-0.0021853},
{0.0026180,-0.0203806,-0.0040620},
{-0.0116230,-0.0668518,0.0370152},
{-0.0133007,-0.0264159,0.0619176},
{0.0186167,0.0050491,-0.0120059},
{-0.0191359,-0.0591690,0.0288149},
{-0.0178121,-0.0041834,0.0141448},
{0.0123077,-0.0063648,0.0279743},
{-0.0157729,-0.0374010,0.0315335},
{0.0149328,-0.0659100,0.0290095},
{0.0006205,0.0021977,-0.0214613},
{0.0170484,-0.0715687,0.0325980},
{0.0068499,0.0061192,0.0196608},
{-0.0167075,0.0020024,0.0150382},

Biology Open • Supplementary information

{0.0059801,-0.0393117,0.0167489},
{-0.0072506,-0.0171067,0.0338751},
{0.0202363,-0.0251596,0.0420997},
{-0.0199311,-0.0601255,0.0171522},
{0.0216641,0.0053410,-0.0027841},
{-0.0197665,-0.0606125,0.0252972},
{0.0129986,-0.0214528,0.0098002},
{0.0201151,-0.0100929,0.0460501},
{0.0104137,0.0017765,-0.0203073},
{0.0166557,-0.0064123,0.0140197},
{-0.0124120,-0.0596549,0.0422637},
{0.0014885,0.0202590,0.0096781},
{0.0028720,0.0020207,-0.0219044},
{0.0376095,-0.3675427,0.0214537},
{0.0049949,0.0206417,-0.0079017},
{-0.0191723,-0.0407590,0.0391310},
{-0.0316406,-0.3638215,-0.0274841},
{0.0021392,0.0226920,0.0000985},
{0.0168435,-0.0229382,0.0264788},
{-0.0157304,-0.0005437,-0.0161456},
{0.0203722,-0.0299750,0.0562762},
{-0.0171077,0.0134146,0.0059615},
{0.0175519,-0.0370653,0.0413374},
{0.0154657,-0.0405127,0.0348980},
{-0.0215172,-0.0323502,0.0481925},
{0.0177998,-0.0515776,0.0444709},
{-0.0183804,-0.0448316,0.0275139},
{0.0033607,-0.0502028,0.0575894},
{0.0010325,-0.0479377,0.0191141},
{0.0224852,-0.0181821,0.0451780},
{0.0084089,-0.0720538,0.0521031},
{-0.0070880,-0.0772912,0.0288023},
{-0.0223712,-0.0028990,0.0024047},
{0.0173136,-0.0120017,0.0204612},
{0.0137772,-0.0623537,0.0276621},
{-0.0100390,-0.0231180,0.0292222},
{-0.0196395,-0.3730207,0.0364730},
{-0.0094445,-0.0526688,0.0528111},
{-0.0115309,-0.3679776,-0.0104995},
{0.0129812,-0.0410477,0.0224072},
{0.0117394,0.0154849,-0.0117191},
{-0.0245208,-0.3649412,0.0295111},
{0.0147476,-0.0502966,0.0364899},
{0.0006132,-0.0446771,0.0600524},
{-0.0118123,0.0191139,0.0031977},
{-0.0017185,-0.0163303,0.0664216},

Biology Open • Supplementary information

{-0.0085098,-0.0074564,0.0247745},
{0.0197459,-0.0086852,0.0430460},
{0.0187064,-0.0223532,0.0600966},
{-0.0206552,-0.0099712,0.0525024},
{0.0207525,0.0084646,-0.0024846},
{-0.0094915,-0.0325446,0.0184441},
{-0.0014464,-0.0140050,-0.0179819},
{-0.0122725,-0.0573444,0.0463125},
{-0.0106397,0.0177211,0.0094669},
{0.0111981,0.0191236,-0.0041491},
{-0.0162790,-0.0256279,0.0592687},
{-0.0060120,-0.3653959,0.0413411},
{-0.0135007,-0.3703638,-0.0387476},
{-0.0134258,-0.3703142,-0.0379467},
{-0.0133509,-0.3702645,-0.0371458},
{-0.0132760,-0.3702148,-0.0363450},
{-0.0132011,-0.3701651,-0.0355441},
{-0.0131262,-0.3701154,-0.0347432},
{-0.0130513,-0.3700658,-0.0339423},
{-0.0129765,-0.3700161,-0.0331414},
{-0.0129016,-0.3699664,-0.0323405},
{-0.0128267,-0.3699167,-0.0315396},
{-0.0127518,-0.3698670,-0.0307388},
{-0.0126769,-0.3698174,-0.0299379},
{-0.0126020,-0.3697677,-0.0291370},
{-0.0125271,-0.3697180,-0.0283361},
{-0.0124523,-0.3696683,-0.0275352},
{-0.0123774,-0.3696187,-0.0267343},
{-0.0123025,-0.3695690,-0.0259334},
{-0.0122276,-0.3695193,-0.0251325},
{-0.0121527,-0.3694696,-0.0243317},
{-0.0120778,-0.3694199,-0.0235308},
{-0.0120029,-0.3693703,-0.0227299},
{-0.0119280,-0.3693206,-0.0219290},
{-0.0118532,-0.3692709,-0.0211281},
{-0.0117783,-0.3692212,-0.0203272},
{-0.0117034,-0.3691715,-0.0195263},
{-0.0116285,-0.3691219,-0.0187255},
{-0.0115536,-0.3690722,-0.0179246},
{-0.0114787,-0.3690225,-0.0171237},
{-0.0114038,-0.3689728,-0.0163228},
{-0.0113289,-0.3689231,-0.0155219},
{-0.0112541,-0.3688735,-0.0147210},
{-0.0111792,-0.3688238,-0.0139201},
{-0.0111043,-0.3687741,-0.0131192},
{-0.0110294,-0.3687244,-0.0123184},

{-0.0109545,-0.3686748,-0.0115175},
{-0.0108796,-0.3686251,-0.0107166},
{-0.0108047,-0.3685754,-0.0099157},
{-0.0107298,-0.3685257,-0.0091148},
{-0.0106550,-0.3684760,-0.0083139},
{-0.0105801,-0.3684264,-0.0075130},
{-0.0105052,-0.3683767,-0.0067121},
{-0.0104303,-0.3683270,-0.0059113},
{-0.0103554,-0.3682773,-0.0051104},
{-0.0102805,-0.3682276,-0.0043095},
{-0.0102056,-0.3681780,-0.0035086},
{-0.0101307,-0.3681283,-0.0027077},
{-0.0100559,-0.3680786,-0.0019068},
{-0.0099810,-0.3680289,-0.0011059},
{-0.0099061,-0.3679792,-0.0003051},
{-0.0098312,-0.3679296,0.0004958},
{-0.0097563,-0.3678799,0.0012967},
{-0.0096814,-0.3678302,0.0020976},
{-0.0096065,-0.3677805,0.0028985},
{-0.0095317,-0.3677308,0.0036994},
{-0.0094568,-0.3676812,0.0045003},
{-0.0093819,-0.3676315,0.0053012},
{-0.0093070,-0.3675818,0.0061020},
{-0.0092321,-0.3675321,0.0069029},
{-0.0091572,-0.3674825,0.0077038},
{-0.0090823,-0.3674328,0.0085047},
{-0.0090074,-0.3673831,0.0093056},
{-0.0089326,-0.3673334,0.0101065},
{-0.0088577,-0.3672837,0.0109074},
{-0.0087828,-0.3672341,0.0117082},
{-0.0087079,-0.3671844,0.0125091},
{-0.0086330,-0.3671347,0.0133100},
{-0.0085581,-0.3670850,0.0141109},
{-0.0084832,-0.3670353,0.0149118},
{-0.0084083,-0.3669857,0.0157127},
{-0.0083335,-0.3669360,0.0165136},
{-0.0082586,-0.3668863,0.0173145},
{-0.0081837,-0.3668366,0.0181153},
{-0.0081088,-0.3667869,0.0189162},
{-0.0080339,-0.3667373,0.0197171},
{-0.0079590,-0.3666876,0.0205180},
{-0.0078841,-0.3666379,0.0213189},
{-0.0078092,-0.3665882,0.0221198},
{-0.0077344,-0.3665386,0.0229207},
{-0.0076595,-0.3664889,0.0237215},
{-0.0075846,-0.3664392,0.0245224},

Biology Open • Supplementary information

```
{-0.0075097,-0.3663895,0.0253233},
{-0.0074348,-0.3663398,0.0261242},
{-0.0073599,-0.3662902,0.0269251},
{-0.0072850,-0.3662405,0.0277260},
{-0.0072101,-0.3661908,0.0285269},
{-0.0071353,-0.3661411,0.0293278},
{-0.0070604,-0.3660914,0.0301286},
{-0.0069855,-0.3660418,0.0309295},
{-0.0069106,-0.3659921,0.0317304},
{-0.0068357,-0.3659424,0.0325313},
{-0.0067608,-0.3658927,0.0333322},
{-0.0066859,-0.3658430,0.0341331},
{-0.0066111,-0.3657934,0.0349340},
{-0.0065362,-0.3657437,0.0357348},
{-0.0064613,-0.3656940,0.0365357},
{-0.0063864,-0.3656443,0.0373366},
{-0.0063115,-0.3655947,0.0381375},
{-0.0062366,-0.3655450,0.0389384},
{-0.0061617,-0.3654953,0.0397393},
{-0.0060868,-0.3654456,0.0405402}
}* .AMirroring);
Points1 = ({
{0.0000000,0.0000000,0.0000000},
{-0.0000000,-0.3752256,0.0000000},
{-0.0085693,-0.3816555,0.0011390},
{-0.0000000,-0.3797708,0.0358540},
{-0.0000000,-0.3706805,-0.0358540},
{0.0127569,-0.0084323,0.0514324},
{0.0185102,-0.0219307,0.0404797},
{0.0088961,-0.0248749,0.0561087},
{0.0001438,-0.0003879,0.0457825},
{0.0103310,-0.0066699,0.0356789},
{-0.0054462,-0.0136607,0.0581914},
{0.0154934,-0.0377578,0.0463652},
{0.0138814,-0.0327857,0.0297933},
{0.0131119,-0.0141170,0.0225403},
{-0.0093298,-0.0313549,0.0555238},
{0.0030907,-0.0410453,0.0546077},
{-0.0028296,-0.0196543,0.0370377},
{-0.0114859,-0.0042181,0.0431362},
{-0.0000082,-0.0041377,0.0250272},
{-0.0112786,-0.0223822,0.0443424},
{0.0092663,-0.0594052,0.0487118},
{0.0147365,-0.0527748,0.0347026},
{0.0076820,-0.0445626,0.0174009},
{0.0088840,-0.0237326,0.0113519},
```

Biology Open • Supplementary information

{0.0168343,-0.0053844,0.0051890},
{0.0095259,0.0059365,0.0144813},
{-0.0112201,-0.0373344,0.0329994},
{-0.0092173,-0.0485327,0.0468854},
{-0.0084617,-0.0249827,0.0226532},
{-0.0056180,0.0104502,0.0145078},
{-0.0104260,-0.0057492,0.0143011},
{-0.0068236,-0.0686348,0.0406596},
{0.0057743,-0.0787569,0.0472332},
{0.0156927,-0.0738266,0.0334758},
{0.0076161,-0.0635767,0.0191200},
{-0.0107808,-0.0451418,0.0187907},
{-0.0038472,-0.0320878,0.0092844},
{-0.0004070,-0.0194753,-0.0043709},
{0.0117237,-0.0152638,-0.0059346},
{0.0158411,-0.0017547,-0.0101350},
{0.0155323,0.0127130,-0.0011033},
{0.0028708,0.0192927,0.0081957},
{-0.0130358,-0.0560370,0.0308336},
{-0.0108303,-0.0122450,0.0052776},
{-0.0110853,0.0166343,0.0014637},
{-0.0169107,0.0023061,0.0028877},
{-0.0062217,-0.0760229,0.0252498},
{-0.0039371,-0.0911524,0.0382865},
{0.0133589,-0.0957316,0.0408105},
{0.0114035,-0.0817930,0.0198254},
{-0.0092568,-0.0607181,0.0195177},
{-0.0132830,-0.0086658,-0.0093103},
{0.0006015,-0.0089495,-0.0170153},
{0.0054637,0.0076111,-0.0156620},
{0.0015373,0.0196876,-0.0073996},
{-0.0096161,0.0082366,-0.0132399},
{0.0004049,-0.0958962,0.0212343},
{-0.0017006,-0.1090245,0.0365077},
{0.0143866,-0.1154333,0.0389357},
{0.0171602,-0.0992357,0.0239866},
{0.0057060,-0.1126714,0.0176132},
{0.0000126,-0.1273038,0.0319684},
{0.0153989,-0.1351688,0.0369248},
{0.0204804,-0.1204499,0.0235480},
{0.0088770,-0.1311515,0.0150480},
{0.0021363,-0.1461530,0.0293557},
{0.0175222,-0.1551211,0.0344439},
{0.0226167,-0.1409515,0.0211682},
{0.0104839,-0.1511220,0.0128401},
{0.0035131,-0.1649807,0.0264163},

Biology Open • Supplementary information

{0.0177982,-0.1752963,0.0323331},
{0.0240371,-0.1595929,0.0191795},
{0.0117451,-0.1705654,0.0108652},
{0.0024149,-0.1844183,0.0195494},
{0.0128086,-0.1948284,0.0288500},
{0.0252214,-0.1928034,0.0219609},
{0.0243553,-0.1776228,0.0153757},
{0.0146761,-0.1926273,0.0080851},
{0.0021502,-0.2042005,0.0166924},
{0.0101453,-0.2185813,0.0251846},
{0.0226483,-0.2112652,0.0259975},
{0.0245873,-0.2091761,0.0118779},
{0.0129615,-0.2157436,0.0062676},
{0.0018156,-0.2275754,0.0141569},
{0.0111105,-0.2405944,0.0241550},
{0.0233802,-0.2301381,0.0232178},
{0.0244662,-0.2311974,0.0090771},
{0.0116629,-0.2387643,0.0040913},
{0.0026394,-0.2496287,0.0134901},
{0.0089806,-0.2627871,0.0221013},
{0.0237634,-0.2518906,0.0204967},
{0.0236955,-0.2510759,0.0055487},
{0.0091611,-0.2621453,0.0021945},
{0.0023529,-0.2765323,0.0096714},
{0.0100190,-0.2836367,0.0218445},
{0.0233816,-0.2733442,0.0188925},
{0.0238850,-0.2688640,0.0036303},
{0.0139608,-0.2812902,-0.0025476},
{0.0035362,-0.2968940,0.0006879},
{0.0022923,-0.2991674,0.0164840},
{0.0199384,-0.2986938,0.0196105},
{0.0260954,-0.2904759,0.0074227},
{0.0206724,-0.3025999,-0.0043678},
{0.0058273,-0.3140367,-0.0060270},
{-0.0001515,-0.3178955,0.0082215},
{0.0106884,-0.3159212,0.0213060},
{0.0261651,-0.3143846,0.0100321},
{0.0204709,-0.3223598,-0.0073997},
{0.0041188,-0.3335384,-0.0108809},
{-0.0019632,-0.3387163,0.0038067},
{0.0014792,-0.3322687,0.0191336},
{0.0201659,-0.3316617,0.0188863},
{0.0256789,-0.3348928,0.0045181},
{0.0194676,-0.3419619,-0.0116257},
{0.0088237,-0.3545572,-0.0214806},
{-0.0046560,-0.3502145,-0.0120951},

{-0.0056593,-0.3617892,0.0025187},
{-0.0052126,-0.3535410,0.0178248},
{0.0092339,-0.3492018,0.0232035},
{0.0248311,-0.3513051,0.0158371},
{0.0237513,-0.3560352,-0.0001306},
{0.0225751,-0.3638271,-0.0159718},
{0.0157177,-0.3772272,-0.0265994},
{0.0037900,-0.3676822,-0.0335453},
{-0.0083626,-0.3591100,-0.0259137},
{-0.0144509,-0.3675562,-0.0111255},
{-0.0053487,-0.3828307,-0.0029039},
{-0.0155047,-0.3747966,0.0096713},
{-0.0134658,-0.3679762,0.0263180},
{0.0018734,-0.3639382,0.0308089},
{0.0185057,-0.3681510,0.0245790},
{0.0313201,-0.3691401,0.0139968},
{0.0242183,-0.3752910,-0.0016343},
{0.0262187,-0.3881635,-0.0143802},
{0.0153999,-0.3999666,-0.0243752},
{0.0022489,-0.3880151,-0.0348821},
{-0.0120101,-0.3759721,-0.0353190},
{-0.0243868,-0.3701059,-0.0250289},
{-0.0241935,-0.3838167,-0.0106129},
{-0.0154444,-0.4003873,-0.0156275},
{0.0001381,-0.4001298,-0.0103687},
{0.0026580,-0.3978456,0.0066361},
{-0.0126581,-0.3953589,0.0123234},
{-0.0217570,-0.3839836,0.0250444},
{-0.0039353,-0.3814897,0.0355404},
{0.0116979,-0.3851284,0.0293548},
{0.0285347,-0.3862844,0.0215780},
{0.0238426,-0.3907050,0.0063627},
{0.0147548,-0.3965966,-0.0057398},
{-0.0001277,-0.4057267,-0.0266154},
{-0.0127357,-0.3977728,-0.0337402},
{-0.0264661,-0.3891295,-0.0282242},
{0.0172576,-0.4009525,0.0185200},
{0.0013813,-0.4033625,0.0242930},
{-0.0128801,-0.3991935,0.0295923},
{-0.0007430,-0.0652699,0.0170507},
{-0.0089080,-0.0568188,0.0184901},
{0.0084913,-0.0333307,0.0553352},
{-0.0122521,-0.0135434,0.0510048},
{-0.0015019,-0.0555202,0.0512765},
{-0.0054347,-0.0623479,0.0201175},
{-0.0012172,-0.0801118,0.0183707},

Biology Open • Supplementary information

{-0.0060350,0.0016551,-0.0152224},
{-0.0107588,-0.0435749,0.0368455},
{0.0028528,-0.0259741,0.0586688},
{-0.0087721,-0.0644069,0.0230101},
{-0.0009212,-0.0529352,0.0164305},
{-0.0113757,-0.0166238,0.0454629},
{0.0117544,-0.0293976,0.0538323},
{-0.0090358,-0.0291987,0.0355321},
{0.0123383,-0.0336016,0.0523252},
{-0.0007693,-0.0731265,0.0480612},
{-0.0127950,-0.0428103,0.0279753},
{-0.0120556,-0.0542860,0.0196800},
{-0.0140388,-0.0530954,0.0208173},
{-0.0021416,-0.0403502,0.0121476},
{0.0093514,-0.0565639,0.0213784},
{0.0187346,-0.0129375,0.0354035},
{-0.0026980,-0.0429838,0.0554158},
{0.0117132,-0.0027396,0.0154038},
{-0.0076533,-0.0474893,0.0169169},
{-0.0051593,-0.0789462,0.0396771},
{0.0048256,0.0144171,0.0117346},
{-0.0097813,0.0165875,-0.0053794},
{-0.0061090,-0.0655868,0.0424700},
{0.0067862,-0.0824920,0.0173488},
{0.0111915,-0.0723638,0.0217174},
{0.0067054,-0.0459381,0.0525521},
{0.0124998,-0.0122783,0.0067828},
{-0.0050026,-0.0228945,0.0040909},
{-0.0062261,-0.0009443,0.0179419},
{-0.0087473,-0.0546791,0.0437014},
{-0.0053653,-0.0254398,0.0587247},
{0.0072871,-0.0061772,0.0536349},
{0.0117869,-0.0491071,0.0264605},
{-0.0017386,-0.0176186,-0.0107643},
{-0.0119412,-0.0237607,0.0487619},
{0.0138756,0.0129272,-0.0062647},
{-0.0035153,0.0209486,0.0032638},
{0.0005469,-0.0826136,0.0466157},
{-0.0112527,-0.0384077,0.0441466},
{0.0172039,-0.0271592,0.0374864},
{0.0100356,-0.0416902,0.0213108},
{0.0154409,0.0100907,0.0063782},
{0.0177951,0.0020209,0.0059575},
{0.0186277,-0.0019234,-0.0030154},
{0.0119324,-0.0195327,0.0198803},
{0.0099047,-0.0336220,0.0189811},

Biology Open • Supplementary information

{0.0140570,-0.0142543,0.0284396},
{-0.0150322,-0.0475557,0.0233783},
{0.0026011,-0.0371753,0.0569285},
{0.0145429,-0.0437865,0.0338790},
{0.0125703,-0.0098200,0.0117007},
{0.0008877,0.0042585,-0.0171227},
{0.0075021,-0.0007399,-0.0169926},
{0.0089853,-0.0653445,0.0473385},
{0.0128327,-0.0590076,0.0449697},
{0.0150728,-0.0573314,0.0373928},
{0.0152452,-0.0663703,0.0316227},
{-0.0040599,-0.0724145,0.0216416},
{-0.0114061,0.0036156,0.0123047},
{0.0066370,-0.0010864,0.0485901},
{0.0165134,-0.0090514,0.0442912},
{0.0128747,-0.0228773,0.0529973},
{-0.0102390,-0.0345873,0.0223014},
{-0.0157825,-0.0072434,-0.0022485},
{0.0140423,-0.0799660,0.0241049},
{-0.0059942,-0.0055490,-0.0167254},
{0.0035343,0.0136192,-0.0130306},
{-0.0080250,-0.0319217,0.0140698},
{-0.0127734,0.0099606,-0.0091553},
{-0.0173366,0.0047902,-0.0014611},
{-0.0078358,-0.0676624,0.0347748},
{0.0100351,-0.0168141,-0.0008342},
{0.0108003,-0.0438498,0.0504578},
{0.0074975,-0.0178276,-0.0079040},
{0.0086888,-0.0134263,-0.0121142},
{0.0037206,-0.0573054,0.0166625},
{0.0077467,-0.0488846,0.0188788},
{0.0010587,-0.0732853,0.0176506},
{-0.0157603,-0.0540707,0.0265536},
{-0.0052394,-0.0013107,0.0458783},
{-0.0113610,-0.0296713,0.0523308},
{0.0155578,-0.0167193,0.0476038},
{0.0130164,-0.0741518,0.0424536},
{0.0163066,-0.0231283,0.0308567},
{0.0072544,-0.0306856,0.0585273},
{-0.0147750,-0.0031309,0.0087373},
{0.0125292,-0.0155507,0.0529699},
{0.0134565,-0.0590310,0.0286968},
{-0.0132173,-0.0483199,0.0198255},
{-0.0122210,-0.0321547,0.0463107},
{0.0120841,-0.0401242,0.0269073},
{-0.0068006,-0.0373335,0.0566651},

Biology Open • Supplementary information

{0.0057749,-0.0074624,-0.0165945},
{0.0036009,-0.0148693,-0.0133449},
{-0.0125334,-0.0103967,0.0453313},
{-0.0090913,-0.0431561,0.0510196},
{0.0151903,-0.0521146,0.0416269},
{-0.0147089,0.0107673,0.0013814},
{-0.0120326,0.0088431,0.0102631},
{-0.0016594,-0.0655748,0.0482843},
{0.0115746,-0.0089563,0.0300473},
{0.0121004,-0.0033791,0.0485711},
{0.0133074,-0.0055389,0.0380633},
{-0.0111510,-0.0129769,-0.0075828},
{0.0049977,-0.0211807,0.0020747},
{0.0003220,-0.0473078,0.0150301},
{0.0174780,0.0030202,-0.0066992},
{0.0149810,0.0015670,-0.0116161},
{0.0111728,-0.0074429,-0.0139320},
{-0.0114665,0.0013244,-0.0136307},
{-0.0128037,-0.0057678,-0.0116799},
{0.0154553,0.0127683,0.0028292},
{0.0092349,0.0184109,-0.0020251},
{0.0078827,0.0133937,-0.0116732},
{0.0070439,-0.0218045,0.0569605},
{-0.0006211,-0.0083291,0.0561801},
{-0.0001449,-0.0020185,0.0506306},
{0.0047035,-0.0132402,0.0574723},
{0.0073443,0.0070935,0.0146183},
{0.0107795,-0.0167963,0.0138621},
{0.0034455,-0.0244018,0.0584411},
{-0.0079826,-0.0166788,0.0215880},
{-0.0089469,-0.0168570,0.0107766},
{-0.0056707,-0.0096539,0.0241057},
{-0.0132010,-0.0489613,0.0315355},
{-0.0099463,-0.0591888,0.0365018},
{-0.0060710,-0.0280934,0.0083445},
{0.0181461,-0.0178651,0.0411941},
{0.0107227,-0.0081729,0.0246706},
{0.0037891,-0.0009137,0.0216140},
{-0.0031419,-0.0734325,0.0469957},
{-0.0021083,0.0211894,-0.0029124},
{-0.0010020,-0.0127697,0.0348488},
{-0.0095875,-0.0138778,0.0021942},
{0.0113651,-0.0278506,0.0228411},
{0.0090559,-0.0262673,0.0152618},
{-0.0096493,-0.0067219,0.0469106},
{0.0164289,-0.0320499,0.0427300},

Biology Open • Supplementary information

{-0.0105881,-0.0517948,0.0370133},
{-0.0065527,-0.0782845,0.0335560},
{0.0038004,-0.0050790,0.0274822},
{-0.0026212,-0.0044580,-0.0177846},
{-0.0110631,-0.0336360,0.0519381},
{-0.0026400,0.0086167,-0.0150346},
{-0.0037909,0.0160649,-0.0111031},
{0.0163254,-0.0830993,0.0323503},
{0.0032394,-0.0716637,0.0169537},
{0.0144409,-0.0857388,0.0397690},
{0.0084109,0.0185891,0.0047930},
{0.0103027,0.0088617,0.0129835},
{0.0009638,0.0146009,-0.0127414},
{0.0141035,-0.0113226,-0.0078808},
{0.0166090,-0.0074060,-0.0067583},
{-0.0076314,-0.0236007,0.0271021},
{-0.0095645,-0.0335608,0.0286480},
{-0.0117561,-0.0599440,0.0229168},
{-0.0012418,0.0025861,0.0178987},
{0.0036432,0.0071157,0.0154416},
{-0.0022064,-0.0027724,0.0228660},
{0.0002251,-0.0299532,0.0579978},
{0.0060507,-0.0422246,0.0533761},
{-0.0102474,-0.0193066,0.0559133},
{-0.0102237,-0.0256033,0.0552277},
{0.0079157,0.0197008,0.0023596},
{0.0149731,0.0137608,0.0012520},
{-0.0062385,-0.0106164,0.0569277},
{0.0133296,-0.0517103,0.0306286},
{-0.0108634,-0.0462696,0.0428170},
{-0.0131391,-0.0490686,0.0196127},
{0.0095914,-0.0820193,0.0450945},
{-0.0085751,-0.0245573,0.0157783},
{-0.0088387,-0.0149543,0.0151709},
{-0.0038729,0.0155609,0.0112588},
{0.0007825,-0.0663043,0.0498533},
{0.0051511,-0.0611295,0.0500065},
{-0.0019755,-0.0608263,0.0495565},
{0.0144964,-0.0716774,0.0276799},
{-0.0108944,-0.0177961,0.0436214},
{0.0093317,-0.0241797,0.0153586},
{0.0045075,-0.0510662,0.0522065},
{0.0072626,-0.0341283,0.0555051},
{0.0083422,-0.0819719,0.0176858},
{-0.0059274,-0.0514356,0.0506890},
{0.0156756,-0.0416167,0.0391771},

Biology Open • Supplementary information

{-0.0104219,-0.0415997,0.0188429},
{0.0185263,-0.0200374,0.0333284},
{-0.0035226,-0.0592227,0.0180758},
{0.0145552,-0.0019340,0.0113699},
{0.0053963,0.0052472,0.0155746},
{0.0008007,-0.0314903,0.0578930},
{-0.0129586,0.0133185,-0.0045168},
{0.0092731,0.0152130,0.0090451},
{-0.0093288,0.0155220,0.0072577},
{-0.0162678,-0.0053270,-0.0045955},
{-0.0163648,0.0004927,-0.0065920},
{-0.0042843,-0.0169480,0.0590670},
{-0.0114951,-0.0332554,0.0387281},
{0.0048841,-0.0453767,0.0533510},
{0.0108705,-0.0403001,0.0513545},
{-0.0076416,-0.0661069,0.0225864},
{0.0110429,0.0068596,0.0133705},
{0.0159394,-0.0103487,-0.0009981},
{-0.0151451,-0.0077754,0.0044490},
{-0.0111684,-0.0543926,0.0343189},
{-0.0063932,-0.0162800,-0.0033029},
{-0.0113090,-0.0266225,0.0415114},
{0.0040678,-0.0723309,0.0484753},
{0.0139654,-0.0482140,0.0457000},
{-0.0015171,-0.0336599,0.0097175},
{0.0054620,-0.0309104,0.0115828},
{0.0164131,-0.0067292,0.0050935},
{0.0158481,-0.0786215,0.0367466},
{-0.0079848,-0.0182745,0.0221232},
{-0.0007684,0.0125068,0.0141586},
{0.0064733,-0.0658775,0.0186481},
{0.0076887,-0.0602000,0.0494390},
{0.0131326,-0.0125117,-0.0082994},
{0.0004861,-0.0087834,0.0565987},
{-0.0062516,-0.0142288,-0.0124436},
{0.0138971,-0.0680114,0.0412793},
{0.0067219,-0.0177656,-0.0090826},
{-0.0092801,-0.0621846,0.0203913},
{0.0116379,-0.0379238,0.0254811},
{0.0135652,-0.0041253,0.0472946},
{0.0041474,-0.0073662,0.0344744},
{-0.0026543,-0.0595521,0.0500458},
{-0.0111927,-0.0573992,0.0334213},
{-0.0126354,-0.0110136,0.0473261},
{-0.0068705,0.0118255,-0.0128305},
{-0.0150085,0.0055763,0.0071369},

Biology Open • Supplementary information

{0.0081714,-0.0656186,0.0194413},
{0.0021800,-0.0374604,0.0561293},
{-0.0118033,-0.0208813,0.0481432},
{0.0056290,-0.0059060,-0.0172086},
{0.0134654,-0.0271977,0.0494874},
{0.0104571,-0.0144111,0.0543031},
{0.0091077,0.0019899,-0.0158505},
{0.0119809,-0.0077892,0.0150526},
{-0.0113102,0.0106217,0.0102376},
{0.0090542,-0.0503948,0.0504082},
{-0.0047772,-0.0365966,0.0573865},
{0.0169932,-0.0276921,0.0438275},
{0.0138504,-0.0126050,-0.0061389},
{-0.0130413,-0.0478169,0.0319744},
{-0.0066000,-0.0702098,0.0396417},
{0.0027963,-0.0501045,0.0528539},
{-0.0038582,-0.0394248,0.0122925},
{0.0097331,0.0101745,-0.0128160},
{-0.0018732,-0.0205619,-0.0007405},
{-0.0080122,-0.0697420,0.0265129},
{-0.0090271,-0.0033314,-0.0154131},
{-0.0025487,0.0206138,-0.0041002},
{-0.0083795,-0.0206516,0.0116389},
{-0.0093879,-0.0612645,0.0202356},
{-0.0051977,-0.0710552,0.0217864},
{0.0008331,0.0112721,-0.0146618},
{0.0173456,-0.0247055,0.0431533},
{-0.0051530,-0.0551357,0.0174822},
{0.0154112,-0.0345108,0.0348753},
{0.0153889,-0.0292539,0.0310719},
{-0.0125528,-0.0113659,-0.0008611},
{0.0116019,0.0133173,0.0089173},
{-0.0117037,-0.0103659,0.0061247},
{-0.0093530,-0.0334339,0.0316159},
{0.0157016,-0.0833335,0.0375294},
{0.0154598,-0.0098125,0.0492645},
{0.0164094,-0.0147746,0.0456399},
{0.0011909,-0.0251380,0.0044426},
{0.0094374,-0.0221673,0.0555806},
{-0.0024398,-0.0090520,0.0277775},
{-0.0168614,-0.0040259,-0.0015380},
{0.0024120,-0.0199308,-0.0033703},
{-0.0084705,-0.0674490,0.0326925},
{-0.0111417,-0.0260535,0.0538931},
{0.0156489,0.0049191,-0.0095206},
{-0.0129190,-0.0590109,0.0266482},

Biology Open • Supplementary information

{-0.0133230,-0.0039792,0.0113157},
{0.0104910,-0.0076152,0.0239286},
{-0.0117472,-0.0405930,0.0283427},
{0.0131615,-0.0677925,0.0254625},
{0.0006729,0.0031913,-0.0173462},
{0.0147657,-0.0733901,0.0277825},
{0.0060719,0.0060421,0.0149232},
{-0.0124945,0.0021396,0.0114581},
{0.0051170,-0.0406325,0.0146888},
{-0.0056635,-0.0180100,0.0301048},
{0.0179748,-0.0255153,0.0380271},
{-0.0112885,-0.0592440,0.0203472},
{0.0182419,0.0048398,-0.0026949},
{-0.0121374,-0.0594009,0.0247925},
{0.0098539,-0.0211681,0.0098841},
{0.0172667,-0.0110641,0.0405675},
{0.0075776,0.0018408,-0.0166885},
{0.0129067,-0.0078573,0.0120975},
{-0.0099222,-0.0601259,0.0359260},
{0.0004053,0.0194797,0.0082369},
{0.0024867,0.0031249,-0.0176306},
{0.0330259,-0.3813067,0.0188401},
{0.0044758,0.0193022,-0.0069805},
{-0.0119589,-0.0425603,0.0334590},
{-0.0277920,-0.3774451,-0.0241412},
{0.0020514,0.0218369,-0.0000287},
{0.0127237,-0.0232008,0.0238590},
{-0.0124979,-0.0010403,-0.0130383},
{0.0146558,-0.0292969,0.0475592},
{-0.0129737,0.0127157,0.0042560},
{0.0152793,-0.0379007,0.0359412},
{0.0136362,-0.0422819,0.0308283},
{-0.0117504,-0.0333511,0.0410849},
{0.0153427,-0.0535536,0.0395412},
{-0.0148095,-0.0465374,0.0246760},
{0.0027058,-0.0531678,0.0521811},
{0.0009629,-0.0495983,0.0155837},
{0.0189567,-0.0202096,0.0395286},
{0.0069989,-0.0738512,0.0473003},
{-0.0052658,-0.0801777,0.0250721},
{-0.0168004,-0.0020808,0.0027406},
{0.0127344,-0.0129544,0.0177169},
{0.0122479,-0.0643698,0.0246518},
{-0.0082221,-0.0238635,0.0246897},
{-0.0172492,-0.3869889,0.0320347},
{-0.0076541,-0.0538935,0.0468132},

{-0.0101280,-0.3817569,-0.0092221},
{0.0094394,-0.0417831,0.0200940},
{0.0095935,0.0140242,-0.0100416},
{-0.0215376,-0.3786068,0.0259207},
{0.0138272,-0.0513308,0.0314191},
{0.0002637,-0.0478650,0.0536957},
{-0.0097061,0.0174760,0.0025514},
{-0.0021804,-0.0161286,0.0589417},
{-0.0064460,-0.0071179,0.0216317},
{0.0171234,-0.0099537,0.0378631},
{0.0131133,-0.0236564,0.0512963},
{-0.0124865,-0.0105350,0.0466696},
{0.0175013,0.0079551,-0.0025172},
{-0.0088523,-0.0329548,0.0160764},
{-0.0002974,-0.0136415,-0.0147409},
{-0.0095149,-0.0581761,0.0396042},
{-0.0079510,0.0165404,0.0071908},
{0.0086191,0.0184894,-0.0026909},
{-0.0118275,-0.0261161,0.0511087},
{-0.0052805,-0.3790786,0.0363114},
{-0.0118582,-0.3842325,-0.0340335},
{-0.0117924,-0.3841809,-0.0333300},
{-0.0117266,-0.3841294,-0.0326266},
{-0.0116608,-0.3840779,-0.0319231},
{-0.0115950,-0.3840263,-0.0312197},
{-0.0115293,-0.3839748,-0.0305163},
{-0.0114635,-0.3839232,-0.0298128},
{-0.0113977,-0.3838717,-0.0291094},
{-0.0113319,-0.3838202,-0.0284059},
{-0.0112662,-0.3837686,-0.0277025},
{-0.0112004,-0.3837171,-0.0269990},
{-0.0111346,-0.3836655,-0.0262956},
{-0.0110688,-0.3836140,-0.0255921},
{-0.0110031,-0.3835625,-0.0248887},
{-0.0109373,-0.3835109,-0.0241852},
{-0.0108715,-0.3834594,-0.0234818},
{-0.0108057,-0.3834079,-0.0227783},
{-0.0107400,-0.3833563,-0.0220749},
{-0.0106742,-0.3833048,-0.0213714},
{-0.0106084,-0.3832532,-0.0206680},
{-0.0105426,-0.3832017,-0.0199645},
{-0.0104769,-0.3831502,-0.0192611},
{-0.0104111,-0.3830986,-0.0185576},
{-0.0103453,-0.3830471,-0.0178542},
{-0.0102795,-0.3829955,-0.0171507},
{-0.0102137,-0.3829440,-0.0164473},

Biology Open • Supplementary information

{-0.0101480,-0.3828925,-0.0157438},
{-0.0100822,-0.3828409,-0.0150404},
{-0.0100164,-0.3827894,-0.0143369},
{-0.0099506,-0.3827378,-0.0136335},
{-0.0098849,-0.3826863,-0.0129300},
{-0.0098191,-0.3826348,-0.0122266},
{-0.0097533,-0.3825832,-0.0115231},
{-0.0096875,-0.3825317,-0.0108197},
{-0.0096218,-0.3824801,-0.0101162},
{-0.0095560,-0.3824286,-0.0094128},
{-0.0094902,-0.3823771,-0.0087093},
{-0.0094244,-0.3823255,-0.0080059},
{-0.0093587,-0.3822740,-0.0073024},
{-0.0092929,-0.3822224,-0.0065990},
{-0.0092271,-0.3821709,-0.0058955},
{-0.0091613,-0.3821194,-0.0051921},
{-0.0090955,-0.3820678,-0.0044886},
{-0.0090298,-0.3820163,-0.0037852},
{-0.0089640,-0.3819648,-0.0030817},
{-0.0088982,-0.3819132,-0.0023783},
{-0.0088324,-0.3818617,-0.0016748},
{-0.0087667,-0.3818101,-0.0009714},
{-0.0087009,-0.3817586,-0.0002679},
{-0.0086351,-0.3817071,0.0004355},
{-0.0085693,-0.3816555,0.0011390},
{-0.0085036,-0.3816040,0.0018424},
{-0.0084378,-0.3815524,0.0025459},
{-0.0083720,-0.3815009,0.0032493},
{-0.0083062,-0.3814494,0.0039528},
{-0.0082405,-0.3813978,0.0046562},
{-0.0081747,-0.3813463,0.0053596},
{-0.0081089,-0.3812947,0.0060631},
{-0.0080431,-0.3812432,0.0067665},
{-0.0079774,-0.3811917,0.0074700},
{-0.0079116,-0.3811401,0.0081734},
{-0.0078458,-0.3810886,0.0088769},
{-0.0077800,-0.3810370,0.0095803},
{-0.0077142,-0.3809855,0.0102838},
{-0.0076485,-0.3809340,0.0109872},
{-0.0075827,-0.3808824,0.0116907},
{-0.0075169,-0.3808309,0.0123941},
{-0.0074511,-0.3807793,0.0130976},
{-0.0073854,-0.3807278,0.0138010},
{-0.0073196,-0.3806763,0.0145045},
{-0.0072538,-0.3806247,0.0152079},
{-0.0071880,-0.3805732,0.0159114},

Biology Open • Supplementary information

```
                    {-0.0071223,-0.3805217,0.0166148},
                    {-0.0070565,-0.3804701,0.0173183},
                    {-0.0069907,-0.3804186,0.0180217},
                    {-0.0069249,-0.3803670,0.0187252},
                    {-0.0068592,-0.3803155,0.0194286},
                    {-0.0067934,-0.3802640,0.0201321},
                    {-0.0067276,-0.3802124,0.0208355},
                    {-0.0066618,-0.3801609,0.0215390},
                    {-0.0065961,-0.3801093,0.0222424},
                    {-0.0065303,-0.3800578,0.0229459},
                    {-0.0064645,-0.3800063,0.0236493},
                    {-0.0063987,-0.3799547,0.0243528},
                    {-0.0063329,-0.3799032,0.0250562},
                    {-0.0062672,-0.3798516,0.0257597},
                    {-0.0062014,-0.3798001,0.0264631},
                    {-0.0061356,-0.3797486,0.0271666},
                    {-0.0060698,-0.3796970,0.0278700},
                    {-0.0060041,-0.3796455,0.0285735},
                    {-0.0059383,-0.3795939,0.0292769},
                    {-0.0058725,-0.3795424,0.0299804},
                    {-0.0058067,-0.3794909,0.0306838},
                    {-0.0057410,-0.3794393,0.0313873},
                    {-0.0056752,-0.3793878,0.0320907},
                    {-0.0056094,-0.3793362,0.0327942},
                    {-0.0055436,-0.3792847,0.0334976},
                    {-0.0054779,-0.3792332,0.0342011},
                    {-0.0054121,-0.3791816,0.0349045},
                    {-0.0053463,-0.3791301,0.0356080}
                    }* .AMirroring);
            BoundingBoxOnOff = Off;
        };
        AnyFunTransform3DIdentity ScaleFunction = {
            PreTransforms = {&.RBFTransform};
};
};

};
};
```

Biology Open • Supplementary information

ScalingFunctionTLEMLucyPelvis_2014040

```
AnyFolder ScalingFunctionTLEMLucyPelvis = {
AnyFolder Pelvis = {
	AnyFunTransform3DRBF RBFTransform = {
		RBFDef.Type = RBF_ThinPlate;
		PolynomDegree = 1;
		Points0 = {
			{0.0000000,0.0000000,0.1177000},
			{-0.0000000,-0.0832729,0.0191000},
			{-0.0508179,-0.0694062,0.0815920},
			{0.0000000,0.0000000,-0.1177000},
			{-0.0000000,-0.0832729,-0.0191000},
			{-0.0508179,-0.0694062,-0.0815920},
			{0.0000000,0.0000000,0.0000000},
			{-0.1164020,-0.0039449,0.0456760},
			{-0.1164020,-0.0039449,-0.0456760},
			{-0.1092909,-0.0992949,0.0515590},
			{-0.1092909,-0.0992949,-0.0515590},
			{-0.0733749,0.0757445,0.0915590},
			{-0.0733749,0.0757445,-0.0915590},
			{-0.1241738,0.0316243,0.0458050},
			{-0.1241738,0.0316243,-0.0458050},
			{-0.1108008,0.0563490,0.0555690},
			{-0.1108008,0.0563490,-0.0555690},
			{-0.0545356,0.0481653,0.1291690},
			{-0.0545356,0.0481653,-0.1291690},
			{-0.0360236,0.0456352,0.1316250},
			{-0.0360236,0.0456352,-0.1316250},
			{-0.0804664,-0.0228481,0.0672140},
			{-0.0804664,-0.0228481,-0.0672140},
			{-0.1004070,-0.0623213,0.0494990},
			{-0.1004070,-0.0623213,-0.0494990},
			{-0.0174901,-0.0357786,0.0977400},
			{-0.0174901,-0.0357786,-0.0977400},
			{-0.0665950,-0.1308418,0.0278190},
			{-0.0665950,-0.1308418,-0.0278190},
			{-0.0962739,-0.1257274,0.0551670},
			{-0.0962739,-0.1257274,-0.0551670},
			{-0.0393340,-0.1196098,0.0159820},
			{-0.0393340,-0.1196098,-0.0159820},
			{-0.0585979,-0.0148191,0.0651020},
			{-0.0585979,-0.0148191,-0.0651020},
			{-0.0238420,-0.0800479,0.0351680},
			{-0.0238420,-0.0800479,-0.0351680},
			{-0.0234553,-0.0576924,0.0715330},
```

Biology Open • Supplementary information

{-0.0234553,-0.0576924,-0.0715330},
{-0.0472319,-0.0833276,0.0513790},
{-0.0472319,-0.0833276,-0.0513790},
{-0.0327175,-0.0506481,0.0520240},
{-0.0327175,-0.0506481,-0.0520240},
{-0.0746024,-0.0627219,0.0928110},
{-0.0746024,-0.0627219,-0.0928110},
{-0.0119676,-0.0928080,0.0076900},
{-0.0119676,-0.0928080,-0.0076900},
{-0.0263299,-0.0760728,0.0667080},
{-0.0263299,-0.0760728,-0.0667080},
{-0.0498842,0.0119450,0.1070630},
{-0.0498842,0.0119450,-0.1070630},
{-0.0553874,0.0627474,0.1172390},
{-0.0553874,0.0627474,-0.1172390},
{-0.0786774,0.0073556,0.0741800},
{-0.0786774,0.0073556,-0.0741800},
{-0.0742762,0.0474272,0.0542330},
{-0.0742762,0.0474272,-0.0542330},
{-0.0634121,0.0117185,0.0618430},
{-0.0634121,0.0117185,-0.0618430},
{-0.0813084,0.0007821,0.0508130},
{-0.0813084,0.0007821,-0.0508130},
{-0.0496423,-0.0444458,0.0983920},
{-0.0496423,-0.0444458,-0.0983920},
{-0.0480005,-0.0338999,0.0625080},
{-0.0480005,-0.0338999,-0.0625080},
{-0.0428000,-0.0273188,0.0973490},
{-0.0428000,-0.0273188,-0.0973490},
{-0.0548383,-0.0518766,0.0624050},
{-0.0548383,-0.0518766,-0.0624050},
{-0.0696448,-0.1052827,0.0436200},
{-0.0696448,-0.1052827,-0.0436200},
{-0.0739608,-0.0803177,0.0785950},
{-0.0739608,-0.0803177,-0.0785950},
{-0.0586066,-0.0884764,0.0653190},
{-0.0586066,-0.0884764,-0.0653190},
{-0.1017003,0.0085215,0.0624150},
{-0.1017003,0.0085215,-0.0624150},
{-0.0126252,0.0252852,0.1292530},
{-0.0126252,0.0252852,-0.1292530},
{-0.0030963,0.0150156,0.1262640},
{-0.0030963,0.0150156,-0.1262640},
{-0.0621797,0.0048743,0.0605920},
{-0.0621797,0.0048743,-0.0605920},
{-0.0870082,-0.0684280,0.0489900},

Biology Open • Supplementary information

{-0.0870082,-0.0684280,-0.0489900},
{-0.0916211,-0.0478344,0.0575290},
{-0.0916211,-0.0478344,-0.0575290},
{-0.0428573,-0.0467026,0.0985250},
{-0.0428573,-0.0467026,-0.0985250},
{-0.0483869,-0.0455474,0.0848580},
{-0.0483869,-0.0455474,-0.0848580},
{-0.0203929,-0.0681811,0.0426070},
{-0.0203929,-0.0681811,-0.0426070},
{-0.0271973,-0.0807786,0.0491650},
{-0.0271973,-0.0807786,-0.0491650},
{-0.0325935,-0.1026813,0.0148280},
{-0.0325935,-0.1026813,-0.0148280},
{-0.0273900,-0.1059173,0.0048750},
{-0.0273900,-0.1059173,-0.0048750},
{-0.0015971,-0.0778770,0.0065530},
{-0.0015971,-0.0778770,-0.0065530},
{-0.1034983,-0.0140026,0.0521800},
{-0.1034983,-0.0140026,-0.0521800},
{-0.1141160,0.0077734,0.0497600},
{-0.1141160,0.0077734,-0.0497600},
{-0.1123797,0.0244808,0.0350160},
{-0.1123797,0.0244808,-0.0350160},
{-0.0984470,-0.0648210,0.0457100},
{-0.0984470,-0.0648210,-0.0457100},
{-0.0814332,-0.0704479,0.0806410},
{-0.0814332,-0.0704479,-0.0806410},
{-0.0540218,-0.0805070,0.0547320},
{-0.0540218,-0.0805070,-0.0547320},
{-0.0674356,-0.0763171,0.0651080},
{-0.0674356,-0.0763171,-0.0651080},
{-0.0602364,-0.0751827,0.0490600},
{-0.0602364,-0.0751827,-0.0490600},
{-0.0604272,-0.0801096,0.0617610},
{-0.0604272,-0.0801096,-0.0617610},
{-0.0655251,0.0632155,0.1093750},
{-0.0655251,0.0632155,-0.1093750},
{-0.0665046,0.0014289,0.0572170},
{-0.0665046,0.0014289,-0.0572170},
{-0.1053786,0.0347803,0.0071580},
{-0.1053786,0.0347803,-0.0071580},
{-0.1321649,-0.0069725,0.0058340},
{-0.1321649,-0.0069725,-0.0058340},
{-0.1232735,-0.0434903,0.0301110},
{-0.1232735,-0.0434903,-0.0301110},
{-0.0497024,0.0261862,0.0084640},

Biology Open • Supplementary information

```
        {-0.0497024,0.0261862,-0.0084640},
        {-0.0887827,0.0446048,0.0327030},
        {-0.0887827,0.0446048,-0.0327030},
        {-0.1153998,-0.0126010,0.0290730},
        {-0.1153998,-0.0126010,-0.0290730},
        {-0.0899939,0.0093882,0.0208440},
        {-0.0899939,0.0093882,-0.0208440},
        {-0.1312654,-0.0574898,0.0094690},
        {-0.1312654,-0.0574898,-0.0094690},
        {-0.0996029,0.0042806,0.0105620},
        {-0.0996029,0.0042806,-0.0105620},
        {-0.1226177,-0.0324069,0.0331630},
        {-0.1226177,-0.0324069,-0.0331630},
        {-0.1097808,-0.0051619,0.0102520},
        {-0.1097808,-0.0051619,-0.0102520}
        };
Points1 = {
        {0.0000000,0.0000000,0.1105234},
        {-0.0000006,-0.0811296,0.0160937},
        {-0.0277147,-0.0629993,0.0769122},
        {0.0000000,0.0000000,-0.1105234},
        {-0.0000006,-0.0811296,-0.0160937},
        {-0.0277147,-0.0629993,-0.0769122},
        {0.0000000,0.0000000,0.0000000},
        {-0.0756739,-0.0264940,0.0374381},
        {-0.0756739,-0.0264940,-0.0374381},
        {-0.0717271,-0.0891007,0.0546621},
        {-0.0717271,-0.0891007,-0.0546621},
        {-0.0492431,0.0372616,0.0806084},
        {-0.0492431,0.0372616,-0.0806084},
        {-0.0798895,-0.0066375,0.0331927},
        {-0.0798895,-0.0066375,-0.0331927},
        {-0.0725206,0.0139569,0.0440721},
        {-0.0725206,0.0139569,-0.0440721},
        {-0.0304006,0.0286002,0.1144451},
        {-0.0304006,0.0286002,-0.1144451},
        {-0.0198115,0.0288889,0.1182021},
        {-0.0198115,0.0288889,-0.1182021},
        {-0.0535569,-0.0359484,0.0606838},
        {-0.0535569,-0.0359484,-0.0606838},
        {-0.0669143,-0.0657146,0.0561354},
        {-0.0669143,-0.0657146,-0.0561354},
        {-0.0099718,-0.0352249,0.0829771},
        {-0.0099718,-0.0352249,-0.0829771},
        {-0.0463459,-0.1122080,0.0301673},
        {-0.0463459,-0.1122080,-0.0301673},
```

Biology Open • Supplementary information

{-0.0626961,-0.1040185,0.0574857},
{-0.0626961,-0.1040185,-0.0574857},
{-0.0260746,-0.1075315,0.0168306},
{-0.0260746,-0.1075315,-0.0168306},
{-0.0416725,-0.0256200,0.0586353},
{-0.0416725,-0.0256200,-0.0586353},
{-0.0132493,-0.0739734,0.0303998},
{-0.0132493,-0.0739734,-0.0303998},
{-0.0139429,-0.0525814,0.0651752},
{-0.0139429,-0.0525814,-0.0651752},
{-0.0258862,-0.0716047,0.0500070},
{-0.0258862,-0.0716047,-0.0500070},
{-0.0216700,-0.0527849,0.0507206},
{-0.0216700,-0.0527849,-0.0507206},
{-0.0439404,-0.0590908,0.0808942},
{-0.0439404,-0.0590908,-0.0808942},
{-0.0063389,-0.0900052,0.0069099},
{-0.0063389,-0.0900052,-0.0069099},
{-0.0148159,-0.0633584,0.0593778},
{-0.0148159,-0.0633584,-0.0593778},
{-0.0320942,-0.0008899,0.0941315},
{-0.0320942,-0.0008899,-0.0941315},
{-0.0342553,0.0355672,0.1044140},
{-0.0342553,0.0355672,-0.1044140},
{-0.0544137,-0.0127742,0.0614996},
{-0.0544137,-0.0127742,-0.0614996},
{-0.0521108,0.0125483,0.0443190},
{-0.0521108,0.0125483,-0.0443190},
{-0.0473154,-0.0091714,0.0498659},
{-0.0473154,-0.0091714,-0.0498659},
{-0.0569586,-0.0206773,0.0414022},
{-0.0569586,-0.0206773,-0.0414022},
{-0.0275861,-0.0452671,0.0836826},
{-0.0275861,-0.0452671,-0.0836826},
{-0.0317850,-0.0389625,0.0598029},
{-0.0317850,-0.0389625,-0.0598029},
{-0.0256633,-0.0312889,0.0840100},
{-0.0256633,-0.0312889,-0.0840100},
{-0.0328379,-0.0523813,0.0612771},
{-0.0328379,-0.0523813,-0.0612771},
{-0.0443960,-0.0919179,0.0469762},
{-0.0443960,-0.0919179,-0.0469762},
{-0.0432416,-0.0722464,0.0735819},
{-0.0432416,-0.0722464,-0.0735819},
{-0.0329703,-0.0785971,0.0655994},
{-0.0329703,-0.0785971,-0.0655994},

Biology Open • Supplementary information

{-0.0674238,-0.0154609,0.0509955},
{-0.0674238,-0.0154609,-0.0509955},
{-0.0065970,0.0170265,0.1185032},
{-0.0065970,0.0170265,-0.1185032},
{-0.0011854,0.0107354,0.1170453},
{-0.0011854,0.0107354,-0.1170453},
{-0.0470828,-0.0137559,0.0486440},
{-0.0470828,-0.0137559,-0.0486440},
{-0.0562872,-0.0681085,0.0557619},
{-0.0562872,-0.0681085,-0.0557619},
{-0.0597288,-0.0544873,0.0583051},
{-0.0597288,-0.0544873,-0.0583051},
{-0.0227641,-0.0469511,0.0832783},
{-0.0227641,-0.0469511,-0.0832783},
{-0.0278715,-0.0462768,0.0748418},
{-0.0278715,-0.0462768,-0.0748418},
{-0.0117618,-0.0655053,0.0368321},
{-0.0117618,-0.0655053,-0.0368321},
{-0.0149798,-0.0714796,0.0436407},
{-0.0149798,-0.0714796,-0.0436407},
{-0.0187464,-0.0950872,0.0153337},
{-0.0187464,-0.0950872,-0.0153337},
{-0.0139157,-0.0980832,0.0059901},
{-0.0139157,-0.0980832,-0.0059901},
{-0.0016109,-0.0799818,0.0055586},
{-0.0016109,-0.0799818,-0.0055586},
{-0.0676786,-0.0317009,0.0465267},
{-0.0676786,-0.0317009,-0.0465267},
{-0.0745054,-0.0174772,0.0394441},
{-0.0745054,-0.0174772,-0.0394441},
{-0.0731467,-0.0104996,0.0253603},
{-0.0731467,-0.0104996,-0.0253603},
{-0.0659809,-0.0674090,0.0549506},
{-0.0659809,-0.0674090,-0.0549506},
{-0.0489803,-0.0660188,0.0748906},
{-0.0489803,-0.0660188,-0.0748906},
{-0.0293108,-0.0688673,0.0543030},
{-0.0293108,-0.0688673,-0.0543030},
{-0.0389543,-0.0704232,0.0654972},
{-0.0389543,-0.0704232,-0.0654972},
{-0.0348397,-0.0685716,0.0511845},
{-0.0348397,-0.0685716,-0.0511845},
{-0.0337500,-0.0728492,0.0631809},
{-0.0337500,-0.0728492,-0.0631809},
{-0.0424263,0.0325432,0.0969958},
{-0.0424263,0.0325432,-0.0969958},

Biology Open • Supplementary information

```
                    {-0.0500656,-0.0172130,0.0451854},
                    {-0.0500656,-0.0172130,-0.0451854},
                    {-0.0692451,-0.0054420,0.0050965},
                    {-0.0692451,-0.0054420,-0.0050965},
                    {-0.0846295,-0.0343558,0.0040555},
                    {-0.0846295,-0.0343558,-0.0040555},
                    {-0.0837929,-0.0594791,0.0190383},
                    {-0.0837929,-0.0594791,-0.0190383},
                    {-0.0377736,-0.0062180,0.0069270},
                    {-0.0377736,-0.0062180,-0.0069270},
                    {-0.0605861,0.0055831,0.0250998},
                    {-0.0605861,0.0055831,-0.0250998},
                    {-0.0750570,-0.0357622,0.0229473},
                    {-0.0750570,-0.0357622,-0.0229473},
                    {-0.0605183,-0.0206807,0.0162436},
                    {-0.0605183,-0.0206807,-0.0162436},
                    {-0.0893172,-0.0695952,0.0059503},
                    {-0.0893172,-0.0695952,-0.0059503},
                    {-0.0644435,-0.0265559,0.0081507},
                    {-0.0644435,-0.0265559,-0.0081507},
                    {-0.0818466,-0.0506120,0.0242379},
                    {-0.0818466,-0.0506120,-0.0242379},
                    {-0.0695979,-0.0336955,0.0078098},
                    {-0.0695979,-0.0336955,-0.0078098}
                    };
            BoundingBoxOnOff = Off;
        };
        AnyFunTransform3DIdentity ScaleFunction = {
            PreTransforms = {&.RBFTransform};
};
};
AnyFolder Sacrum = {
        AnyFunTransform3DRBF RBFTransform = {
            RBFDef.Type = RBF_ThinPlate;
            PolynomDegree = 1;
            Points0 = {
                    {0.0000000,0.0000000,0.1177000},
                    {-0.0000000,-0.0832729,0.0191000},
                    {-0.0508179,-0.0694062,0.0815920},
                    {0.0000000,0.0000000,-0.1177000},
                    {-0.0000000,-0.0832729,-0.0191000},
                    {-0.0508179,-0.0694062,-0.0815920},
                    {0.0000000,0.0000000,0.0000000},
                    {-0.1164020,-0.0039449,0.0456760},
                    {-0.1164020,-0.0039449,-0.0456760},
                    {-0.1092909,-0.0992949,0.0515590},
```

Biology Open • Supplementary information

{-0.1092909,-0.0992949,-0.0515590},
{-0.0733749,0.0757445,0.0915590},
{-0.0733749,0.0757445,-0.0915590},
{-0.1241738,0.0316243,0.0458050},
{-0.1241738,0.0316243,-0.0458050},
{-0.1108008,0.0563490,0.0555690},
{-0.1108008,0.0563490,-0.0555690},
{-0.0545356,0.0481653,0.1291690},
{-0.0545356,0.0481653,-0.1291690},
{-0.0360236,0.0456352,0.1316250},
{-0.0360236,0.0456352,-0.1316250},
{-0.0804664,-0.0228481,0.0672140},
{-0.0804664,-0.0228481,-0.0672140},
{-0.1004070,-0.0623213,0.0494990},
{-0.1004070,-0.0623213,-0.0494990},
{-0.0174901,-0.0357786,0.0977400},
{-0.0174901,-0.0357786,-0.0977400},
{-0.0665950,-0.1308418,0.0278190},
{-0.0665950,-0.1308418,-0.0278190},
{-0.0962739,-0.1257274,0.0551670},
{-0.0962739,-0.1257274,-0.0551670},
{-0.0393340,-0.1196098,0.0159820},
{-0.0393340,-0.1196098,-0.0159820},
{-0.0585979,-0.0148191,0.0651020},
{-0.0585979,-0.0148191,-0.0651020},
{-0.0238420,-0.0800479,0.0351680},
{-0.0238420,-0.0800479,-0.0351680},
{-0.0234553,-0.0576924,0.0715330},
{-0.0234553,-0.0576924,-0.0715330},
{-0.0472319,-0.0833276,0.0513790},
{-0.0472319,-0.0833276,-0.0513790},
{-0.0327175,-0.0506481,0.0520240},
{-0.0327175,-0.0506481,-0.0520240},
{-0.0746024,-0.0627219,0.0928110},
{-0.0746024,-0.0627219,-0.0928110},
{-0.0119676,-0.0928080,0.0076900},
{-0.0119676,-0.0928080,-0.0076900},
{-0.0263299,-0.0760728,0.0667080},
{-0.0263299,-0.0760728,-0.0667080},
{-0.0498842,0.0119450,0.1070630},
{-0.0498842,0.0119450,-0.1070630},
{-0.0553874,0.0627474,0.1172390},
{-0.0553874,0.0627474,-0.1172390},
{-0.0786774,0.0073556,0.0741800},
{-0.0786774,0.0073556,-0.0741800},
{-0.0742762,0.0474272,0.0542330},

Biology Open • Supplementary information

{-0.0742762,0.0474272,-0.0542330},
{-0.0634121,0.0117185,0.0618430},
{-0.0634121,0.0117185,-0.0618430},
{-0.0813084,0.0007821,0.0508130},
{-0.0813084,0.0007821,-0.0508130},
{-0.0496423,-0.0444458,0.0983920},
{-0.0496423,-0.0444458,-0.0983920},
{-0.0480005,-0.0338999,0.0625080},
{-0.0480005,-0.0338999,-0.0625080},
{-0.0428000,-0.0273188,0.0973490},
{-0.0428000,-0.0273188,-0.0973490},
{-0.0548383,-0.0518766,0.0624050},
{-0.0548383,-0.0518766,-0.0624050},
{-0.0696448,-0.1052827,0.0436200},
{-0.0696448,-0.1052827,-0.0436200},
{-0.0739608,-0.0803177,0.0785950},
{-0.0739608,-0.0803177,-0.0785950},
{-0.0586066,-0.0884764,0.0653190},
{-0.0586066,-0.0884764,-0.0653190},
{-0.1017003,0.0085215,0.0624150},
{-0.1017003,0.0085215,-0.0624150},
{-0.0126252,0.0252852,0.1292530},
{-0.0126252,0.0252852,-0.1292530},
{-0.0030963,0.0150156,0.1262640},
{-0.0030963,0.0150156,-0.1262640},
{-0.0621797,0.0048743,0.0605920},
{-0.0621797,0.0048743,-0.0605920},
{-0.0870082,-0.0684280,0.0489900},
{-0.0870082,-0.0684280,-0.0489900},
{-0.0916211,-0.0478344,0.0575290},
{-0.0916211,-0.0478344,-0.0575290},
{-0.0428573,-0.0467026,0.0985250},
{-0.0428573,-0.0467026,-0.0985250},
{-0.0483869,-0.0455474,0.0848580},
{-0.0483869,-0.0455474,-0.0848580},
{-0.0203929,-0.0681811,0.0426070},
{-0.0203929,-0.0681811,-0.0426070},
{-0.0271973,-0.0807786,0.0491650},
{-0.0271973,-0.0807786,-0.0491650},
{-0.0325935,-0.1026813,0.0148280},
{-0.0325935,-0.1026813,-0.0148280},
{-0.0273900,-0.1059173,0.0048750},
{-0.0273900,-0.1059173,-0.0048750},
{-0.0015971,-0.0778770,0.0065530},
{-0.0015971,-0.0778770,-0.0065530},
{-0.1034983,-0.0140026,0.0521800},

Biology Open • Supplementary information

```
	{-0.1034983,-0.0140026,-0.0521800},
	{-0.1141160,0.0077734,0.0497600},
	{-0.1141160,0.0077734,-0.0497600},
	{-0.1123797,0.0244808,0.0350160},
	{-0.1123797,0.0244808,-0.0350160},
	{-0.0984470,-0.0648210,0.0457100},
	{-0.0984470,-0.0648210,-0.0457100},
	{-0.0814332,-0.0704479,0.0806410},
	{-0.0814332,-0.0704479,-0.0806410},
	{-0.0540218,-0.0805070,0.0547320},
	{-0.0540218,-0.0805070,-0.0547320},
	{-0.0674356,-0.0763171,0.0651080},
	{-0.0674356,-0.0763171,-0.0651080},
	{-0.0602364,-0.0751827,0.0490600},
	{-0.0602364,-0.0751827,-0.0490600},
	{-0.0604272,-0.0801096,0.0617610},
	{-0.0604272,-0.0801096,-0.0617610},
	{-0.0655251,0.0632155,0.1093750},
	{-0.0655251,0.0632155,-0.1093750},
	{-0.0665046,0.0014289,0.0572170},
	{-0.0665046,0.0014289,-0.0572170},
	{-0.1053786,0.0347803,0.0071580},
	{-0.1053786,0.0347803,-0.0071580},
	{-0.1321649,-0.0069725,0.0058340},
	{-0.1321649,-0.0069725,-0.0058340},
	{-0.1232735,-0.0434903,0.0301110},
	{-0.1232735,-0.0434903,-0.0301110},
	{-0.0497024,0.0261862,0.0084640},
	{-0.0497024,0.0261862,-0.0084640},
	{-0.0887827,0.0446048,0.0327030},
	{-0.0887827,0.0446048,-0.0327030},
	{-0.1153998,-0.0126010,0.0290730},
	{-0.1153998,-0.0126010,-0.0290730},
	{-0.0899939,0.0093882,0.0208440},
	{-0.0899939,0.0093882,-0.0208440},
	{-0.1312654,-0.0574898,0.0094690},
	{-0.1312654,-0.0574898,-0.0094690},
	{-0.0996029,0.0042806,0.0105620},
	{-0.0996029,0.0042806,-0.0105620},
	{-0.1226177,-0.0324069,0.0331630},
	{-0.1226177,-0.0324069,-0.0331630},
	{-0.1097808,-0.0051619,0.0102520},
	{-0.1097808,-0.0051619,-0.0102520}
	};
Points1 = {
	{0.0000000,0.0000000,0.1105234},
```

Biology Open • Supplementary information

{-0.0000006,-0.0811296,0.0160937},
{-0.0277147,-0.0629993,0.0769122},
{0.0000000,0.0000000,-0.1105234},
{-0.0000006,-0.0811296,-0.0160937},
{-0.0277147,-0.0629993,-0.0769122},
{0.0000000,0.0000000,0.0000000},
{-0.0756739,-0.0264940,0.0374381},
{-0.0756739,-0.0264940,-0.0374381},
{-0.0717271,-0.0891007,0.0546621},
{-0.0717271,-0.0891007,-0.0546621},
{-0.0492431,0.0372616,0.0806084},
{-0.0492431,0.0372616,-0.0806084},
{-0.0798895,-0.0066375,0.0331927},
{-0.0798895,-0.0066375,-0.0331927},
{-0.0725206,0.0139569,0.0440721},
{-0.0725206,0.0139569,-0.0440721},
{-0.0304006,0.0286002,0.1144451},
{-0.0304006,0.0286002,-0.1144451},
{-0.0198115,0.0288889,0.1182021},
{-0.0198115,0.0288889,-0.1182021},
{-0.0535569,-0.0359484,0.0606838},
{-0.0535569,-0.0359484,-0.0606838},
{-0.0669143,-0.0657146,0.0561354},
{-0.0669143,-0.0657146,-0.0561354},
{-0.0099718,-0.0352249,0.0829771},
{-0.0099718,-0.0352249,-0.0829771},
{-0.0463459,-0.1122080,0.0301673},
{-0.0463459,-0.1122080,-0.0301673},
{-0.0626961,-0.1040185,0.0574857},
{-0.0626961,-0.1040185,-0.0574857},
{-0.0260746,-0.1075315,0.0168306},
{-0.0260746,-0.1075315,-0.0168306},
{-0.0416725,-0.0256200,0.0586353},
{-0.0416725,-0.0256200,-0.0586353},
{-0.0132493,-0.0739734,0.0303998},
{-0.0132493,-0.0739734,-0.0303998},
{-0.0139429,-0.0525814,0.0651752},
{-0.0139429,-0.0525814,-0.0651752},
{-0.0258862,-0.0716047,0.0500070},
{-0.0258862,-0.0716047,-0.0500070},
{-0.0216700,-0.0527849,0.0507206},
{-0.0216700,-0.0527849,-0.0507206},
{-0.0439404,-0.0590908,0.0808942},
{-0.0439404,-0.0590908,-0.0808942},
{-0.0063389,-0.0900052,0.0069099},
{-0.0063389,-0.0900052,-0.0069099},

Biology Open • Supplementary information

{-0.0148159,-0.0633584,0.0593778},
{-0.0148159,-0.0633584,-0.0593778},
{-0.0320942,-0.0008899,0.0941315},
{-0.0320942,-0.0008899,-0.0941315},
{-0.0342553,0.0355672,0.1044140},
{-0.0342553,0.0355672,-0.1044140},
{-0.0544137,-0.0127742,0.0614996},
{-0.0544137,-0.0127742,-0.0614996},
{-0.0521108,0.0125483,0.0443190},
{-0.0521108,0.0125483,-0.0443190},
{-0.0473154,-0.0091714,0.0498659},
{-0.0473154,-0.0091714,-0.0498659},
{-0.0569586,-0.0206773,0.0414022},
{-0.0569586,-0.0206773,-0.0414022},
{-0.0275861,-0.0452671,0.0836826},
{-0.0275861,-0.0452671,-0.0836826},
{-0.0317850,-0.0389625,0.0598029},
{-0.0317850,-0.0389625,-0.0598029},
{-0.0256633,-0.0312889,0.0840100},
{-0.0256633,-0.0312889,-0.0840100},
{-0.0328379,-0.0523813,0.0612771},
{-0.0328379,-0.0523813,-0.0612771},
{-0.0443960,-0.0919179,0.0469762},
{-0.0443960,-0.0919179,-0.0469762},
{-0.0432416,-0.0722464,0.0735819},
{-0.0432416,-0.0722464,-0.0735819},
{-0.0329703,-0.0785971,0.0655994},
{-0.0329703,-0.0785971,-0.0655994},
{-0.0674238,-0.0154609,0.0509955},
{-0.0674238,-0.0154609,-0.0509955},
{-0.0065970,0.0170265,0.1185032},
{-0.0065970,0.0170265,-0.1185032},
{-0.0011854,0.0107354,0.1170453},
{-0.0011854,0.0107354,-0.1170453},
{-0.0470828,-0.0137559,0.0486440},
{-0.0470828,-0.0137559,-0.0486440},
{-0.0562872,-0.0681085,0.0557619},
{-0.0562872,-0.0681085,-0.0557619},
{-0.0597288,-0.0544873,0.0583051},
{-0.0597288,-0.0544873,-0.0583051},
{-0.0227641,-0.0469511,0.0832783},
{-0.0227641,-0.0469511,-0.0832783},
{-0.0278715,-0.0462768,0.0748418},
{-0.0278715,-0.0462768,-0.0748418},
{-0.0117618,-0.0655053,0.0368321},
{-0.0117618,-0.0655053,-0.0368321},

{-0.0149798,-0.0714796,0.0436407},
{-0.0149798,-0.0714796,-0.0436407},
{-0.0187464,-0.0950872,0.0153337},
{-0.0187464,-0.0950872,-0.0153337},
{-0.0139157,-0.0980832,0.0059901},
{-0.0139157,-0.0980832,-0.0059901},
{-0.0016109,-0.0799818,0.0055586},
{-0.0016109,-0.0799818,-0.0055586},
{-0.0676786,-0.0317009,0.0465267},
{-0.0676786,-0.0317009,-0.0465267},
{-0.0745054,-0.0174772,0.0394441},
{-0.0745054,-0.0174772,-0.0394441},
{-0.0731467,-0.0104996,0.0253603},
{-0.0731467,-0.0104996,-0.0253603},
{-0.0659809,-0.0674090,0.0549506},
{-0.0659809,-0.0674090,-0.0549506},
{-0.0489803,-0.0660188,0.0748906},
{-0.0489803,-0.0660188,-0.0748906},
{-0.0293108,-0.0688673,0.0543030},
{-0.0293108,-0.0688673,-0.0543030},
{-0.0389543,-0.0704232,0.0654972},
{-0.0389543,-0.0704232,-0.0654972},
{-0.0348397,-0.0685716,0.0511845},
{-0.0348397,-0.0685716,-0.0511845},
{-0.0337500,-0.0728492,0.0631809},
{-0.0337500,-0.0728492,-0.0631809},
{-0.0424263,0.0325432,0.0969958},
{-0.0424263,0.0325432,-0.0969958},
{-0.0500656,-0.0172130,0.0451854},
{-0.0500656,-0.0172130,-0.0451854},
{-0.0692451,-0.0054420,0.0050965},
{-0.0692451,-0.0054420,-0.0050965},
{-0.0846295,-0.0343558,0.0040555},
{-0.0846295,-0.0343558,-0.0040555},
{-0.0837929,-0.0594791,0.0190383},
{-0.0837929,-0.0594791,-0.0190383},
{-0.0377736,-0.0062180,0.0069270},
{-0.0377736,-0.0062180,-0.0069270},
{-0.0605861,0.0055831,0.0250998},
{-0.0605861,0.0055831,-0.0250998},
{-0.0750570,-0.0357622,0.0229473},
{-0.0750570,-0.0357622,-0.0229473},
{-0.0605183,-0.0206807,0.0162436},
{-0.0605183,-0.0206807,-0.0162436},
{-0.0893172,-0.0695952,0.0059503},
{-0.0893172,-0.0695952,-0.0059503},

Biology Open • Supplementary information

```
                    {-0.0644435,-0.0265559,0.0081507},
                    {-0.0644435,-0.0265559,-0.0081507},
                    {-0.0818466,-0.0506120,0.0242379},
                    {-0.0818466,-0.0506120,-0.0242379},
                    {-0.0695979,-0.0336955,0.0078098},
                    {-0.0695979,-0.0336955,-0.0078098}
                    };
            BoundingBoxOnOff = Off;
        };
        AnyFunTransform3DIdentity ScaleFunction = {
            PreTransforms = {&.RBFTransform};
};
};

};
```

Biology Open • Supplementary information

ScalingFunctionTLEMLucyFemur_2014040

```
AnyFolder ScalingFunctionTLEMLucyFemur = {
  AnyFolder Right = {
          AnyFolder Thigh = {
          AnyFunTransform3DRBF RBFTransform = {
                    RBFDef.Type = RBF_ThinPlate;
                    PolynomDegree = 1;
                    Points0 = {
                              {0.0000000,0.0000000,0.0000000},
                              {-0.0000000,-0.3616821,0.0000000},
                              {-0.0097563,-0.3678799,0.0012967},
                              {-0.0000000,-0.3660632,0.0408203},
                              {-0.0000000,-0.3573010,-0.0408203},
                              {0.0161460,-0.0072838,0.0601290},
                              {0.0220217,-0.0203698,0.0463848},
                              {0.0123977,-0.0241932,0.0668573},
                              {0.0006898,0.0018121,0.0538181},
                              {0.0122809,-0.0068668,0.0414535},
                              {-0.0058991,-0.0138188,0.0648412},
                              {0.0211469,-0.0380855,0.0538111},
                              {0.0172133,-0.0317342,0.0328381},
                              {0.0177311,-0.0131946,0.0256176},
                              {-0.0110079,-0.0308867,0.0632370},
                              {0.0039435,-0.0395977,0.0638271},
                              {-0.0041683,-0.0187472,0.0413214},
                              {-0.0177999,-0.0021535,0.0496084},
                              {0.0001603,-0.0053126,0.0279593},
                              {-0.0211600,-0.0216243,0.0497147},
                              {0.0096163,-0.0568663,0.0545719},
                              {0.0162112,-0.0515013,0.0395230},
                              {0.0093533,-0.0433265,0.0187387},
                              {0.0119398,-0.0238469,0.0118403},
                              {0.0210707,-0.0048848,0.0065878},
                              {0.0125013,0.0064094,0.0177702},
                              {-0.0191523,-0.0356053,0.0382429},
                              {-0.0115821,-0.0474937,0.0537648},
                              {-0.0100073,-0.0236371,0.0267322},
                              {-0.0078925,0.0107098,0.0182833},
                              {-0.0134217,-0.0055356,0.0182000},
                              {-0.0090061,-0.0665700,0.0475484},
                              {0.0071095,-0.0761976,0.0520035},
                              {0.0182119,-0.0704149,0.0386546},
                              {0.0073764,-0.0609481,0.0221006},
                              {-0.0122984,-0.0439585,0.0198428},
                              {-0.0032253,-0.0306792,0.0110995},
```

Biology Open • Supplementary information

{-0.0012975,-0.0198451,-0.0052592},
{0.0140405,-0.0161125,-0.0071649},
{0.0189222,-0.0014898,-0.0127226},
{0.0181642,0.0132812,-0.0011050},
{0.0045119,0.0200001,0.0091189},
{-0.0176761,-0.0541983,0.0348587},
{-0.0136548,-0.0142015,0.0054311},
{-0.0135510,0.0182940,0.0017175},
{-0.0223865,0.0023399,0.0028018},
{-0.0086455,-0.0738247,0.0283843},
{-0.0050356,-0.0877749,0.0442195},
{0.0160135,-0.0919319,0.0465120},
{0.0128290,-0.0785810,0.0232431},
{-0.0168328,-0.0616327,0.0155917},
{-0.0172369,-0.0094567,-0.0119502},
{0.0002090,-0.0093641,-0.0209779},
{0.0064827,0.0081538,-0.0198453},
{0.0010777,0.0210422,-0.0084721},
{-0.0129485,0.0085451,-0.0162212},
{-0.0002322,-0.0924033,0.0245644},
{-0.0028175,-0.1052263,0.0418294},
{0.0172322,-0.1111444,0.0445760},
{0.0203259,-0.0957409,0.0271449},
{0.0057102,-0.1088933,0.0195113},
{-0.0010852,-0.1230776,0.0363132},
{0.0182727,-0.1303312,0.0422884},
{0.0242891,-0.1161703,0.0265054},
{0.0094531,-0.1268584,0.0162294},
{0.0014244,-0.1413293,0.0331653},
{0.0207271,-0.1496427,0.0394157},
{0.0267562,-0.1360142,0.0236798},
{0.0113839,-0.1461651,0.0136024},
{0.0030845,-0.1595094,0.0297156},
{0.0209528,-0.1691386,0.0369775},
{0.0283958,-0.1540209,0.0213795},
{0.0129499,-0.1649012,0.0113589},
{0.0016490,-0.1783108,0.0216287},
{0.0147105,-0.1880678,0.0328614},
{0.0298491,-0.1859865,0.0248551},
{0.0286645,-0.1714377,0.0169488},
{0.0165897,-0.1861006,0.0082595},
{0.0014000,-0.1973476,0.0183740},
{0.0114301,-0.2109792,0.0286157},
{0.0267422,-0.2037622,0.0296951},
{0.0288630,-0.2018335,0.0130127},
{0.0145471,-0.2083505,0.0062833},

{0.0011300,-0.2198071,0.0155602},
{0.0126610,-0.2321235,0.0275354},
{0.0275647,-0.2219268,0.0265185},
{0.0286645,-0.2230253,0.0098701},
{0.0130434,-0.2304822,0.0039177},
{0.0022984,-0.2409585,0.0149650},
{0.0101315,-0.2534740,0.0252125},
{0.0279485,-0.2428555,0.0234276},
{0.0276684,-0.2421559,0.0058691},
{0.0101250,-0.2529527,0.0018983},
{0.0021376,-0.2667831,0.0107073},
{0.0114309,-0.2734905,0.0249960},
{0.0273923,-0.2634953,0.0216324},
{0.0278429,-0.2592546,0.0037692},
{0.0159101,-0.2712985,-0.0033988},
{0.0036708,-0.2863280,0.0004644},
{0.0023115,-0.2884756,0.0187554},
{0.0231385,-0.2879051,0.0224853},
{0.0304009,-0.2799973,0.0083549},
{0.0238189,-0.2917246,-0.0052462},
{0.0064904,-0.3027687,-0.0070587},
{-0.0003492,-0.3064690,0.0093014},
{0.0122179,-0.3045269,0.0243511},
{0.0301837,-0.3030127,0.0114462},
{0.0234182,-0.3107336,-0.0085198},
{0.0046893,-0.3214995,-0.0123881},
{-0.0022352,-0.3264905,0.0043340},
{0.0016841,-0.3202756,0.0217839},
{0.0229722,-0.3196887,0.0215083},
{0.0292438,-0.3228042,0.0051430},
{0.0221641,-0.3296190,-0.0132361},
{0.0100459,-0.3417597,-0.0244560},
{-0.0053009,-0.3375737,-0.0137705},
{-0.0064432,-0.3487307,0.0028676},
{-0.0059346,-0.3407801,0.0202938},
{0.0105130,-0.3365976,0.0264175},
{0.0282695,-0.3386249,0.0180307},
{0.0270402,-0.3431844,-0.0001487},
{0.0257040,-0.3506950,-0.0181851},
{0.0178948,-0.3636114,-0.0302838},
{0.0043149,-0.3544109,-0.0381949},
{-0.0095210,-0.3461482,-0.0295031},
{-0.0164526,-0.3542895,-0.0126666},
{-0.0060896,-0.3690127,-0.0033061},
{-0.0176523,-0.3612686,0.0110109},
{-0.0153310,-0.3546943,0.0299634},

Biology Open • Supplementary information

{0.0021329,-0.3508021,0.0350763},
{0.0210690,-0.3548628,0.0279835},
{0.0356594,-0.3558162,0.0159356},
{0.0275718,-0.3617450,-0.0018607},
{0.0298503,-0.3741530,-0.0163721},
{0.0175320,-0.3855300,-0.0277515},
{0.0025604,-0.3740099,-0.0397137},
{-0.0136727,-0.3624016,-0.0402102},
{-0.0277647,-0.3567472,-0.0284947},
{-0.0275446,-0.3699630,-0.0120829},
{-0.0175846,-0.3859355,-0.0177922},
{0.0001572,-0.3856873,-0.0118050},
{0.0030262,-0.3834856,0.0075553},
{-0.0144114,-0.3810887,0.0140304},
{-0.0247706,-0.3701239,0.0285134},
{-0.0044804,-0.3677201,0.0404632},
{0.0133183,-0.3712274,0.0334208},
{0.0324861,-0.3723417,0.0245668},
{0.0271451,-0.3766027,0.0072441},
{0.0167985,-0.3822816,-0.0065349},
{-0.0001454,-0.3910823,-0.0303010},
{-0.0144998,-0.3834154,-0.0384137},
{-0.0301321,-0.3750841,-0.0321336},
{0.0196491,-0.3864804,0.0210853},
{0.0015726,-0.3888034,0.0276569},
{-0.0146652,-0.3847848,0.0336913},
{-0.0014588,-0.0619951,0.0219778},
{-0.0118435,-0.0550626,0.0139639},
{0.0129812,-0.0349966,0.0673128},
{-0.0162907,-0.0105666,0.0597739},
{-0.0012885,-0.0520816,0.0562989},
{-0.0090599,-0.0631298,0.0172224},
{-0.0000222,-0.0771663,0.0231897},
{-0.0068148,0.0023885,-0.0160086},
{-0.0180544,-0.0425409,0.0446585},
{0.0020525,-0.0249904,0.0691549},
{-0.0157097,-0.0662315,0.0238393},
{-0.0010518,-0.0510686,0.0199353},
{-0.0208874,-0.0152865,0.0500034},
{0.0168316,-0.0280857,0.0666680},
{-0.0187392,-0.0282333,0.0403282},
{0.0191622,-0.0351633,0.0622707},
{0.0001216,-0.0706289,0.0524049},
{-0.0169862,-0.0399062,0.0311827},
{-0.0185737,-0.0533066,0.0151224},
{-0.0219058,-0.0528723,0.0222263},

Biology Open • Supplementary information

{-0.0028271,-0.0391620,0.0148452},
{0.0117900,-0.0553425,0.0235348},
{0.0219835,-0.0106400,0.0396338},
{-0.0025366,-0.0401046,0.0625273},
{0.0141017,-0.0018767,0.0198108},
{-0.0078770,-0.0473929,0.0173542},
{-0.0065351,-0.0766431,0.0470258},
{0.0065585,0.0145593,0.0161638},
{-0.0118588,0.0172875,-0.0085064},
{-0.0074087,-0.0627500,0.0506700},
{0.0071940,-0.0780068,0.0213362},
{0.0126736,-0.0708176,0.0244108},
{0.0074694,-0.0436450,0.0604196},
{0.0154186,-0.0139471,0.0064312},
{-0.0053535,-0.0213210,0.0040094},
{-0.0088733,0.0002018,0.0205402},
{-0.0112471,-0.0542608,0.0500266},
{-0.0061625,-0.0248673,0.0653244},
{0.0093695,-0.0065826,0.0614157},
{0.0138015,-0.0467322,0.0284333},
{-0.0020999,-0.0186885,-0.0116392},
{-0.0185640,-0.0225591,0.0566779},
{0.0175798,0.0124360,-0.0068467},
{-0.0039962,0.0217127,0.0053055},
{0.0014975,-0.0784591,0.0514056},
{-0.0172997,-0.0386971,0.0525252},
{0.0196540,-0.0268271,0.0416806},
{0.0135296,-0.0406504,0.0236882},
{0.0182471,0.0095959,0.0087906},
{0.0218667,0.0016804,0.0058793},
{0.0224975,-0.0022652,-0.0022994},
{0.0166750,-0.0191391,0.0236436},
{0.0119785,-0.0328723,0.0195376},
{0.0187631,-0.0133469,0.0308741},
{-0.0187543,-0.0461463,0.0257491},
{0.0039834,-0.0373928,0.0683089},
{0.0157029,-0.0423169,0.0385226},
{0.0164003,-0.0087069,0.0131181},
{0.0008253,0.0033370,-0.0212576},
{0.0101707,-0.0009724,-0.0205688},
{0.0102948,-0.0642683,0.0524532},
{0.0152217,-0.0557944,0.0508978},
{0.0172973,-0.0565707,0.0419468},
{0.0167723,-0.0635334,0.0369834},
{-0.0047397,-0.0695325,0.0241398},
{-0.0152913,0.0035778,0.0161461},

Biology Open • Supplementary information

{0.0091697,0.0009340,0.0575160},
{0.0196686,-0.0089533,0.0500006},
{0.0183998,-0.0212943,0.0622968},
{-0.0114340,-0.0316810,0.0250940},
{-0.0206442,-0.0092599,-0.0028425},
{0.0166868,-0.0784295,0.0276925},
{-0.0069678,-0.0054521,-0.0207011},
{0.0049490,0.0151109,-0.0160093},
{-0.0084025,-0.0318937,0.0162788},
{-0.0173679,0.0095594,-0.0108509},
{-0.0218912,0.0063457,-0.0020698},
{-0.0106547,-0.0668750,0.0397749},
{0.0136098,-0.0170666,-0.0018890},
{0.0141599,-0.0441405,0.0587594},
{0.0093255,-0.0183218,-0.0083008},
{0.0106367,-0.0128276,-0.0154269},
{0.0037350,-0.0549365,0.0209786},
{0.0087662,-0.0472902,0.0195496},
{0.0016839,-0.0708213,0.0221325},
{-0.0202977,-0.0530540,0.0301270},
{-0.0068584,0.0021781,0.0530002},
{-0.0144817,-0.0297218,0.0605833},
{0.0197015,-0.0165217,0.0560654},
{0.0156609,-0.0705363,0.0477736},
{0.0207876,-0.0221433,0.0330733},
{0.0092324,-0.0300767,0.0713160},
{-0.0203392,-0.0034488,0.0097332},
{0.0156978,-0.0143438,0.0629053},
{0.0143267,-0.0563519,0.0317489},
{-0.0161685,-0.0465391,0.0188591},
{-0.0196397,-0.0309875,0.0546496},
{0.0154804,-0.0380422,0.0305433},
{-0.0077774,-0.0351303,0.0637765},
{0.0063980,-0.0086419,-0.0201654},
{0.0030146,-0.0151094,-0.0165974},
{-0.0210080,-0.0096030,0.0507855},
{-0.0105684,-0.0411377,0.0586146},
{0.0180619,-0.0498960,0.0469835},
{-0.0192318,0.0116108,0.0024070},
{-0.0165651,0.0083622,0.0126870},
{-0.0014333,-0.0627138,0.0534746},
{0.0145127,-0.0080178,0.0353667},
{0.0148374,-0.0012228,0.0566181},
{0.0153834,-0.0062427,0.0444727},
{-0.0155164,-0.0132103,-0.0096398},
{0.0068685,-0.0221022,0.0011547},

Biology Open • Supplementary information

{0.0003332,-0.0458500,0.0183716},
{0.0205582,0.0040956,-0.0082969},
{0.0175752,0.0009340,-0.0148407},
{0.0142357,-0.0063623,-0.0169332},
{-0.0147285,0.0023412,-0.0164678},
{-0.0158781,-0.0064451,-0.0151346},
{0.0175951,0.0133456,0.0037559},
{0.0117660,0.0189899,-0.0030690},
{0.0096040,0.0151827,-0.0137450},
{0.0096565,-0.0215194,0.0662734},
{-0.0006793,-0.0074494,0.0631218},
{0.0007819,-0.0004519,0.0600781},
{0.0065377,-0.0125433,0.0654199},
{0.0090461,0.0073096,0.0188711},
{0.0151418,-0.0168302,0.0146465},
{0.0025166,-0.0231863,0.0688894},
{-0.0097891,-0.0152077,0.0253086},
{-0.0109149,-0.0177935,0.0119825},
{-0.0072079,-0.0099373,0.0272725},
{-0.0183727,-0.0467429,0.0356276},
{-0.0126871,-0.0584555,0.0428141},
{-0.0057054,-0.0273042,0.0103554},
{0.0215142,-0.0159693,0.0474531},
{0.0128492,-0.0070016,0.0287645},
{0.0052134,-0.0017047,0.0247463},
{-0.0024284,-0.0707498,0.0513049},
{-0.0036140,0.0220593,-0.0040484},
{-0.0002405,-0.0121253,0.0390050},
{-0.0119811,-0.0156737,0.0015445},
{0.0142082,-0.0279792,0.0246553},
{0.0122244,-0.0262013,0.0169829},
{-0.0146264,-0.0038855,0.0549384},
{0.0207450,-0.0325771,0.0496412},
{-0.0155040,-0.0497560,0.0437087},
{-0.0081119,-0.0756450,0.0387365},
{0.0039684,-0.0062047,0.0313900},
{-0.0023726,-0.0038892,-0.0220521},
{-0.0136977,-0.0339839,0.0602619},
{-0.0035399,0.0089081,-0.0188894},
{-0.0049820,0.0176571,-0.0132254},
{0.0193902,-0.0803191,0.0360429},
{0.0040648,-0.0697109,0.0218205},
{0.0170470,-0.0818610,0.0456892},
{0.0104175,0.0188635,0.0064965},
{0.0135206,0.0092549,0.0158079},
{0.0023933,0.0160498,-0.0157404},

Biology Open • Supplementary information

{0.0164753,-0.0118197,-0.0096995},
{0.0202584,-0.0066858,-0.0082383},
{-0.0098401,-0.0226001,0.0314028},
{-0.0130937,-0.0309622,0.0320764},
{-0.0201510,-0.0613297,0.0222264},
{-0.0010389,0.0036055,0.0209003},
{0.0042025,0.0073392,0.0199393},
{-0.0021630,-0.0038531,0.0251898},
{-0.0007845,-0.0293574,0.0675788},
{0.0070027,-0.0406327,0.0624565},
{-0.0124363,-0.0183001,0.0627966},
{-0.0120485,-0.0256613,0.0628493},
{0.0101200,0.0197695,0.0039334},
{0.0167636,0.0148266,0.0014155},
{-0.0074981,-0.0106930,0.0632797},
{0.0142533,-0.0503445,0.0351954},
{-0.0146107,-0.0460953,0.0506120},
{-0.0161117,-0.0471301,0.0178900},
{0.0112149,-0.0792793,0.0501460},
{-0.0099060,-0.0230794,0.0186951},
{-0.0114543,-0.0153049,0.0183675},
{-0.0042882,0.0161879,0.0151423},
{0.0014582,-0.0638572,0.0541080},
{0.0048554,-0.0582603,0.0553815},
{-0.0017077,-0.0572302,0.0546635},
{0.0166090,-0.0697169,0.0323038},
{-0.0208812,-0.0164273,0.0474354},
{0.0130454,-0.0241864,0.0178294},
{0.0053123,-0.0483770,0.0582251},
{0.0112225,-0.0361429,0.0673300},
{0.0085729,-0.0771256,0.0216100},
{-0.0070783,-0.0488033,0.0560869},
{0.0182083,-0.0411024,0.0454236},
{-0.0119590,-0.0403809,0.0213546},
{0.0233409,-0.0185535,0.0366343},
{-0.0046347,-0.0571211,0.0208243},
{0.0183166,-0.0006966,0.0140116},
{0.0061913,0.0053512,0.0201003},
{-0.0002364,-0.0310828,0.0680351},
{-0.0170773,0.0139270,-0.0054987},
{0.0110805,0.0156034,0.0117010},
{-0.0124628,0.0164667,0.0094550},
{-0.0209014,-0.0073407,-0.0051969},
{-0.0210131,0.0010693,-0.0086891},
{-0.0041596,-0.0172332,0.0663041},
{-0.0216595,-0.0322172,0.0451540},

Biology Open • Supplementary information

{0.0051908,-0.0426014,0.0613013},
{0.0150003,-0.0416136,0.0606257},
{-0.0135090,-0.0674234,0.0236425},
{0.0146416,0.0073691,0.0160052},
{0.0195615,-0.0112412,-0.0010333},
{-0.0198115,-0.0098244,0.0044735},
{-0.0157054,-0.0521236,0.0395039},
{-0.0081381,-0.0173108,-0.0046152},
{-0.0218324,-0.0262661,0.0471115},
{0.0051446,-0.0707257,0.0530555},
{0.0179359,-0.0471493,0.0528134},
{-0.0005591,-0.0317983,0.0113632},
{0.0071707,-0.0305943,0.0121186},
{0.0206239,-0.0061367,0.0066642},
{0.0187057,-0.0745868,0.0420207},
{-0.0095784,-0.0163113,0.0259524},
{-0.0012181,0.0127661,0.0186855},
{0.0059420,-0.0630691,0.0220087},
{0.0069408,-0.0573878,0.0552614},
{0.0150782,-0.0132884,-0.0101793},
{0.0005533,-0.0078574,0.0633775},
{-0.0088284,-0.0146399,-0.0147837},
{0.0167444,-0.0651105,0.0470347},
{0.0082666,-0.0181741,-0.0095133},
{-0.0175162,-0.0637444,0.0177069},
{0.0153795,-0.0360767,0.0287608},
{0.0162363,-0.0018864,0.0548412},
{0.0052731,-0.0075919,0.0392990},
{-0.0022288,-0.0556644,0.0549909},
{-0.0156238,-0.0550038,0.0377688},
{-0.0204137,-0.0100550,0.0536429},
{-0.0093683,0.0122429,-0.0162013},
{-0.0202927,0.0049166,0.0083887},
{0.0074706,-0.0627945,0.0222133},
{0.0035034,-0.0376103,0.0672421},
{-0.0190752,-0.0194129,0.0555530},
{0.0061758,-0.0074961,-0.0207741},
{0.0192011,-0.0273063,0.0588233},
{0.0130796,-0.0134383,0.0638611},
{0.0120421,0.0019566,-0.0194140},
{0.0154152,-0.0065294,0.0179581},
{-0.0157335,0.0101219,0.0123981},
{0.0110376,-0.0488274,0.0567911},
{-0.0055170,-0.0343676,0.0647140},
{0.0216204,-0.0273988,0.0512041},
{0.0161331,-0.0135875,-0.0075501},

{-0.0184643,-0.0455633,0.0360087},
{-0.0091978,-0.0682413,0.0460847},
{0.0034304,-0.0471677,0.0588866},
{-0.0048025,-0.0384316,0.0149400},
{0.0118505,0.0109735,-0.0159161},
{-0.0027036,-0.0204189,-0.0015094},
{-0.0117607,-0.0692759,0.0292171},
{-0.0107080,-0.0033455,-0.0190487},
{-0.0042548,0.0214947,-0.0056846},
{-0.0097315,-0.0208529,0.0131997},
{-0.0178958,-0.0627218,0.0171551},
{-0.0068692,-0.0688818,0.0233316},
{0.0012083,0.0120695,-0.0186179},
{0.0215073,-0.0236027,0.0503069},
{-0.0060262,-0.0534931,0.0189523},
{0.0179951,-0.0336785,0.0397267},
{0.0190509,-0.0284972,0.0336394},
{-0.0169188,-0.0133609,-0.0014203},
{0.0135722,0.0136491,0.0114695},
{-0.0150549,-0.0123230,0.0067722},
{-0.0148411,-0.0311945,0.0355412},
{0.0185490,-0.0794042,0.0431266},
{0.0193568,-0.0087326,0.0575704},
{0.0201026,-0.0144353,0.0531586},
{0.0025583,-0.0248211,0.0045714},
{0.0132689,-0.0214866,0.0652899},
{-0.0028669,-0.0099311,0.0309271},
{-0.0219487,-0.0054893,-0.0021853},
{0.0026180,-0.0203806,-0.0040620},
{-0.0116230,-0.0668518,0.0370152},
{-0.0133007,-0.0264159,0.0619176},
{0.0186167,0.0050491,-0.0120059},
{-0.0191359,-0.0591690,0.0288149},
{-0.0178121,-0.0041834,0.0141448},
{0.0123077,-0.0063648,0.0279743},
{-0.0157729,-0.0374010,0.0315335},
{0.0149328,-0.0659100,0.0290095},
{0.0006205,0.0021977,-0.0214613},
{0.0170484,-0.0715687,0.0325980},
{0.0068499,0.0061192,0.0196608},
{-0.0167075,0.0020024,0.0150382},
{0.0059801,-0.0393117,0.0167489},
{-0.0072506,-0.0171067,0.0338751},
{0.0202363,-0.0251596,0.0420997},
{-0.0199311,-0.0601255,0.0171522},
{0.0216641,0.0053410,-0.0027841},

{-0.0197665,-0.0606125,0.0252972},
{0.0129986,-0.0214528,0.0098002},
{0.0201151,-0.0100929,0.0460501},
{0.0104137,0.0017765,-0.0203073},
{0.0166557,-0.0064123,0.0140197},
{-0.0124120,-0.0596549,0.0422637},
{0.0014885,0.0202590,0.0096781},
{0.0028720,0.0020207,-0.0219044},
{0.0376095,-0.3675427,0.0214537},
{0.0049949,0.0206417,-0.0079017},
{-0.0191723,-0.0407590,0.0391310},
{-0.0316406,-0.3638215,-0.0274841},
{0.0021392,0.0226920,0.0000985},
{0.0168435,-0.0229382,0.0264788},
{-0.0157304,-0.0005437,-0.0161456},
{0.0203722,-0.0299750,0.0562762},
{-0.0171077,0.0134146,0.0059615},
{0.0175519,-0.0370653,0.0413374},
{0.0154657,-0.0405127,0.0348980},
{-0.0215172,-0.0323502,0.0481925},
{0.0177998,-0.0515776,0.0444709},
{-0.0183804,-0.0448316,0.0275139},
{0.0033607,-0.0502028,0.0575894},
{0.0010325,-0.0479377,0.0191141},
{0.0224852,-0.0181821,0.0451780},
{0.0084089,-0.0720538,0.0521031},
{-0.0070880,-0.0772912,0.0288023},
{-0.0223712,-0.0028990,0.0024047},
{0.0173136,-0.0120017,0.0204612},
{0.0137772,-0.0623537,0.0276621},
{-0.0100390,-0.0231180,0.0292222},
{-0.0196395,-0.3730207,0.0364730},
{-0.0094445,-0.0526688,0.0528111},
{-0.0115309,-0.3679776,-0.0104995},
{0.0129812,-0.0410477,0.0224072},
{0.0117394,0.0154849,-0.0117191},
{-0.0245208,-0.3649412,0.0295111},
{0.0147476,-0.0502966,0.0364899},
{0.0006132,-0.0446771,0.0600524},
{-0.0118123,0.0191139,0.0031977},
{-0.0017185,-0.0163303,0.0664216},
{-0.0085098,-0.0074564,0.0247745},
{0.0197459,-0.0086852,0.0430460},
{0.0187064,-0.0223532,0.0600966},
{-0.0206552,-0.0099712,0.0525024},
{0.0207525,0.0084646,-0.0024846},

Biology Open • Supplementary information

{-0.0094915,-0.0325446,0.0184441},
{-0.0014464,-0.0140050,-0.0179819},
{-0.0122725,-0.0573444,0.0463125},
{-0.0106397,0.0177211,0.0094669},
{0.0111981,0.0191236,-0.0041491},
{-0.0162790,-0.0256279,0.0592687},
{-0.0060120,-0.3653959,0.0413411},
{-0.0135007,-0.3703638,-0.0387476},
{-0.0134258,-0.3703142,-0.0379467},
{-0.0133509,-0.3702645,-0.0371458},
{-0.0132760,-0.3702148,-0.0363450},
{-0.0132011,-0.3701651,-0.0355441},
{-0.0131262,-0.3701154,-0.0347432},
{-0.0130513,-0.3700658,-0.0339423},
{-0.0129765,-0.3700161,-0.0331414},
{-0.0129016,-0.3699664,-0.0323405},
{-0.0128267,-0.3699167,-0.0315396},
{-0.0127518,-0.3698670,-0.0307388},
{-0.0126769,-0.3698174,-0.0299379},
{-0.0126020,-0.3697677,-0.0291370},
{-0.0125271,-0.3697180,-0.0283361},
{-0.0124523,-0.3696683,-0.0275352},
{-0.0123774,-0.3696187,-0.0267343},
{-0.0123025,-0.3695690,-0.0259334},
{-0.0122276,-0.3695193,-0.0251325},
{-0.0121527,-0.3694696,-0.0243317},
{-0.0120778,-0.3694199,-0.0235308},
{-0.0120029,-0.3693703,-0.0227299},
{-0.0119280,-0.3693206,-0.0219290},
{-0.0118532,-0.3692709,-0.0211281},
{-0.0117783,-0.3692212,-0.0203272},
{-0.0117034,-0.3691715,-0.0195263},
{-0.0116285,-0.3691219,-0.0187255},
{-0.0115536,-0.3690722,-0.0179246},
{-0.0114787,-0.3690225,-0.0171237},
{-0.0114038,-0.3689728,-0.0163228},
{-0.0113289,-0.3689231,-0.0155219},
{-0.0112541,-0.3688735,-0.0147210},
{-0.0111792,-0.3688238,-0.0139201},
{-0.0111043,-0.3687741,-0.0131192},
{-0.0110294,-0.3687244,-0.0123184},
{-0.0109545,-0.3686748,-0.0115175},
{-0.0108796,-0.3686251,-0.0107166},
{-0.0108047,-0.3685754,-0.0099157},
{-0.0107298,-0.3685257,-0.0091148},
{-0.0106550,-0.3684760,-0.0083139},

Biology Open • Supplementary information

{-0.0105801,-0.3684264,-0.0075130},
{-0.0105052,-0.3683767,-0.0067121},
{-0.0104303,-0.3683270,-0.0059113},
{-0.0103554,-0.3682773,-0.0051104},
{-0.0102805,-0.3682276,-0.0043095},
{-0.0102056,-0.3681780,-0.0035086},
{-0.0101307,-0.3681283,-0.0027077},
{-0.0100559,-0.3680786,-0.0019068},
{-0.0099810,-0.3680289,-0.0011059},
{-0.0099061,-0.3679792,-0.0003051},
{-0.0098312,-0.3679296,0.0004958},
{-0.0097563,-0.3678799,0.0012967},
{-0.0096814,-0.3678302,0.0020976},
{-0.0096065,-0.3677805,0.0028985},
{-0.0095317,-0.3677308,0.0036994},
{-0.0094568,-0.3676812,0.0045003},
{-0.0093819,-0.3676315,0.0053012},
{-0.0093070,-0.3675818,0.0061020},
{-0.0092321,-0.3675321,0.0069029},
{-0.0091572,-0.3674825,0.0077038},
{-0.0090823,-0.3674328,0.0085047},
{-0.0090074,-0.3673831,0.0093056},
{-0.0089326,-0.3673334,0.0101065},
{-0.0088577,-0.3672837,0.0109074},
{-0.0087828,-0.3672341,0.0117082},
{-0.0087079,-0.3671844,0.0125091},
{-0.0086330,-0.3671347,0.0133100},
{-0.0085581,-0.3670850,0.0141109},
{-0.0084832,-0.3670353,0.0149118},
{-0.0084083,-0.3669857,0.0157127},
{-0.0083335,-0.3669360,0.0165136},
{-0.0082586,-0.3668863,0.0173145},
{-0.0081837,-0.3668366,0.0181153},
{-0.0081088,-0.3667869,0.0189162},
{-0.0080339,-0.3667373,0.0197171},
{-0.0079590,-0.3666876,0.0205180},
{-0.0078841,-0.3666379,0.0213189},
{-0.0078092,-0.3665882,0.0221198},
{-0.0077344,-0.3665386,0.0229207},
{-0.0076595,-0.3664889,0.0237215},
{-0.0075846,-0.3664392,0.0245224},
{-0.0075097,-0.3663895,0.0253233},
{-0.0074348,-0.3663398,0.0261242},
{-0.0073599,-0.3662902,0.0269251},
{-0.0072850,-0.3662405,0.0277260},
{-0.0072101,-0.3661908,0.0285269},

Biology Open • Supplementary information

{-0.0071353,-0.3661411,0.0293278},
{-0.0070604,-0.3660914,0.0301286},
{-0.0069855,-0.3660418,0.0309295},
{-0.0069106,-0.3659921,0.0317304},
{-0.0068357,-0.3659424,0.0325313},
{-0.0067608,-0.3658927,0.0333322},
{-0.0066859,-0.3658430,0.0341331},
{-0.0066111,-0.3657934,0.0349340},
{-0.0065362,-0.3657437,0.0357348},
{-0.0064613,-0.3656940,0.0365357},
{-0.0063864,-0.3656443,0.0373366},
{-0.0063115,-0.3655947,0.0381375},
{-0.0062366,-0.3655450,0.0389384},
{-0.0061617,-0.3654953,0.0397393},
{-0.0060868,-0.3654456,0.0405402}
};
Points1 = {
{0.0000000,0.0000000,0.0000000},
{-0.0000000,-0.3752256,0.0000000},
{-0.0085693,-0.3816555,0.0011390},
{-0.0000000,-0.3797708,0.0358540},
{-0.0000000,-0.3706805,-0.0358540},
{0.0127569,-0.0084323,0.0514324},
{0.0185102,-0.0219307,0.0404797},
{0.0088961,-0.0248749,0.0561087},
{0.0001438,-0.0003879,0.0457825},
{0.0103310,-0.0066699,0.0356789},
{-0.0054462,-0.0136607,0.0581914},
{0.0154934,-0.0377578,0.0463652},
{0.0138814,-0.0327857,0.0297933},
{0.0131119,-0.0141170,0.0225403},
{-0.0093298,-0.0313549,0.0555238},
{0.0030907,-0.0410453,0.0546077},
{-0.0028296,-0.0196543,0.0370377},
{-0.0114859,-0.0042181,0.0431362},
{-0.0000082,-0.0041377,0.0250272},
{-0.0112786,-0.0223822,0.0443424},
{0.0092663,-0.0594052,0.0487118},
{0.0147365,-0.0527748,0.0347026},
{0.0076820,-0.0445626,0.0174009},
{0.0088840,-0.0237326,0.0113519},
{0.0168343,-0.0053844,0.0051890},
{0.0095259,0.0059365,0.0144813},
{-0.0112201,-0.0373344,0.0329994},
{-0.0092173,-0.0485327,0.0468854},
{-0.0084617,-0.0249827,0.0226532},

Biology Open • Supplementary information

{-0.0056180,0.0104502,0.0145078},
{-0.0104260,-0.0057492,0.0143011},
{-0.0068236,-0.0686348,0.0406596},
{0.0057743,-0.0787569,0.0472332},
{0.0156927,-0.0738266,0.0334758},
{0.0076161,-0.0635767,0.0191200},
{-0.0107808,-0.0451418,0.0187907},
{-0.0038472,-0.0320878,0.0092844},
{-0.0004070,-0.0194753,-0.0043709},
{0.0117237,-0.0152638,-0.0059346},
{0.0158411,-0.0017547,-0.0101350},
{0.0155323,0.0127130,-0.0011033},
{0.0028708,0.0192927,0.0081957},
{-0.0130358,-0.0560370,0.0308336},
{-0.0108303,-0.0122450,0.0052776},
{-0.0110853,0.0166343,0.0014637},
{-0.0169107,0.0023061,0.0028877},
{-0.0062217,-0.0760229,0.0252498},
{-0.0039371,-0.0911524,0.0382865},
{0.0133589,-0.0957316,0.0408105},
{0.0114035,-0.0817930,0.0198254},
{-0.0092568,-0.0607181,0.0195177},
{-0.0132830,-0.0086658,-0.0093103},
{0.0006015,-0.0089495,-0.0170153},
{0.0054637,0.0076111,-0.0156620},
{0.0015373,0.0196876,-0.0073996},
{-0.0096161,0.0082366,-0.0132399},
{0.0004049,-0.0958962,0.0212343},
{-0.0017006,-0.1090245,0.0365077},
{0.0143866,-0.1154333,0.0389357},
{0.0171602,-0.0992357,0.0239866},
{0.0057060,-0.1126714,0.0176132},
{0.0000126,-0.1273038,0.0319684},
{0.0153989,-0.1351688,0.0369248},
{0.0204804,-0.1204499,0.0235480},
{0.0088770,-0.1311515,0.0150480},
{0.0021363,-0.1461530,0.0293557},
{0.0175222,-0.1551211,0.0344439},
{0.0226167,-0.1409515,0.0211682},
{0.0104839,-0.1511220,0.0128401},
{0.0035131,-0.1649807,0.0264163},
{0.0177982,-0.1752963,0.0323331},
{0.0240371,-0.1595929,0.0191795},
{0.0117451,-0.1705654,0.0108652},
{0.0024149,-0.1844183,0.0195494},
{0.0128086,-0.1948284,0.0288500},

Biology Open • Supplementary information

{0.0252214,-0.1928034,0.0219609},
{0.0243553,-0.1776228,0.0153757},
{0.0146761,-0.1926273,0.0080851},
{0.0021502,-0.2042005,0.0166924},
{0.0101453,-0.2185813,0.0251846},
{0.0226483,-0.2112652,0.0259975},
{0.0245873,-0.2091761,0.0118779},
{0.0129615,-0.2157436,0.0062676},
{0.0018156,-0.2275754,0.0141569},
{0.0111105,-0.2405944,0.0241550},
{0.0233802,-0.2301381,0.0232178},
{0.0244662,-0.2311974,0.0090771},
{0.0116629,-0.2387643,0.0040913},
{0.0026394,-0.2496287,0.0134901},
{0.0089806,-0.2627871,0.0221013},
{0.0237634,-0.2518906,0.0204967},
{0.0236955,-0.2510759,0.0055487},
{0.0091611,-0.2621453,0.0021945},
{0.0023529,-0.2765323,0.0096714},
{0.0100190,-0.2836367,0.0218445},
{0.0233816,-0.2733442,0.0188925},
{0.0238850,-0.2688640,0.0036303},
{0.0139608,-0.2812902,-0.0025476},
{0.0035362,-0.2968940,0.0006879},
{0.0022923,-0.2991674,0.0164840},
{0.0199384,-0.2986938,0.0196105},
{0.0260954,-0.2904759,0.0074227},
{0.0206724,-0.3025999,-0.0043678},
{0.0058273,-0.3140367,-0.0060270},
{-0.0001515,-0.3178955,0.0082215},
{0.0106884,-0.3159212,0.0213060},
{0.0261651,-0.3143846,0.0100321},
{0.0204709,-0.3223598,-0.0073997},
{0.0041188,-0.3335384,-0.0108809},
{-0.0019632,-0.3387163,0.0038067},
{0.0014792,-0.3322687,0.0191336},
{0.0201659,-0.3316617,0.0188863},
{0.0256789,-0.3348928,0.0045181},
{0.0194676,-0.3419619,-0.0116257},
{0.0088237,-0.3545572,-0.0214806},
{-0.0046560,-0.3502145,-0.0120951},
{-0.0056593,-0.3617892,0.0025187},
{-0.0052126,-0.3535410,0.0178248},
{0.0092339,-0.3492018,0.0232035},
{0.0248311,-0.3513051,0.0158371},
{0.0237513,-0.3560352,-0.0001306},

Biology Open • Supplementary information

{0.0225751,-0.3638271,-0.0159718},
{0.0157177,-0.3772272,-0.0265994},
{0.0037900,-0.3676822,-0.0335453},
{-0.0083626,-0.3591100,-0.0259137},
{-0.0144509,-0.3675562,-0.0111255},
{-0.0053487,-0.3828307,-0.0029039},
{-0.0155047,-0.3747966,0.0096713},
{-0.0134658,-0.3679762,0.0263180},
{0.0018734,-0.3639382,0.0308089},
{0.0185057,-0.3681510,0.0245790},
{0.0313201,-0.3691401,0.0139968},
{0.0242183,-0.3752910,-0.0016343},
{0.0262187,-0.3881635,-0.0143802},
{0.0153999,-0.3999666,-0.0243752},
{0.0022489,-0.3880151,-0.0348821},
{-0.0120101,-0.3759721,-0.0353190},
{-0.0243868,-0.3701059,-0.0250289},
{-0.0241935,-0.3838167,-0.0106129},
{-0.0154444,-0.4003873,-0.0156275},
{0.0001381,-0.4001298,-0.0103687},
{0.0026580,-0.3978456,0.0066361},
{-0.0126581,-0.3953589,0.0123234},
{-0.0217570,-0.3839836,0.0250444},
{-0.0039353,-0.3814897,0.0355404},
{0.0116979,-0.3851284,0.0293548},
{0.0285347,-0.3862844,0.0215780},
{0.0238426,-0.3907050,0.0063627},
{0.0147548,-0.3965966,-0.0057398},
{-0.0001277,-0.4057267,-0.0266154},
{-0.0127357,-0.3977728,-0.0337402},
{-0.0264661,-0.3891295,-0.0282242},
{0.0172576,-0.4009525,0.0185200},
{0.0013813,-0.4033625,0.0242930},
{-0.0128801,-0.3991935,0.0295923},
{-0.0007430,-0.0652699,0.0170507},
{-0.0089080,-0.0568188,0.0184901},
{0.0084913,-0.0333307,0.0553352},
{-0.0122521,-0.0135434,0.0510048},
{-0.0015019,-0.0555202,0.0512765},
{-0.0054347,-0.0623479,0.0201175},
{-0.0012172,-0.0801118,0.0183707},
{-0.0060350,0.0016551,-0.0152224},
{-0.0107588,-0.0435749,0.0368455},
{0.0028528,-0.0259741,0.0586688},
{-0.0087721,-0.0644069,0.0230101},
{-0.0009212,-0.0529352,0.0164305},

{-0.0113757,-0.0166238,0.0454629},
{0.0117544,-0.0293976,0.0538323},
{-0.0090358,-0.0291987,0.0355321},
{0.0123383,-0.0336016,0.0523252},
{-0.0007693,-0.0731265,0.0480612},
{-0.0127950,-0.0428103,0.0279753},
{-0.0120556,-0.0542860,0.0196800},
{-0.0140388,-0.0530954,0.0208173},
{-0.0021416,-0.0403502,0.0121476},
{0.0093514,-0.0565639,0.0213784},
{0.0187346,-0.0129375,0.0354035},
{-0.0026980,-0.0429838,0.0554158},
{0.0117132,-0.0027396,0.0154038},
{-0.0076533,-0.0474893,0.0169169},
{-0.0051593,-0.0789462,0.0396771},
{0.0048256,0.0144171,0.0117346},
{-0.0097813,0.0165875,-0.0053794},
{-0.0061090,-0.0655868,0.0424700},
{0.0067862,-0.0824920,0.0173488},
{0.0111915,-0.0723638,0.0217174},
{0.0067054,-0.0459381,0.0525521},
{0.0124998,-0.0122783,0.0067828},
{-0.0050026,-0.0228945,0.0040909},
{-0.0062261,-0.0009443,0.0179419},
{-0.0087473,-0.0546791,0.0437014},
{-0.0053653,-0.0254398,0.0587247},
{0.0072871,-0.0061772,0.0536349},
{0.0117869,-0.0491071,0.0264605},
{-0.0017386,-0.0176186,-0.0107643},
{-0.0119412,-0.0237607,0.0487619},
{0.0138756,0.0129272,-0.0062647},
{-0.0035153,0.0209486,0.0032638},
{0.0005469,-0.0826136,0.0466157},
{-0.0112527,-0.0384077,0.0441466},
{0.0172039,-0.0271592,0.0374864},
{0.0100356,-0.0416902,0.0213108},
{0.0154409,0.0100907,0.0063782},
{0.0177951,0.0020209,0.0059575},
{0.0186277,-0.0019234,-0.0030154},
{0.0119324,-0.0195327,0.0198803},
{0.0099047,-0.0336220,0.0189811},
{0.0140570,-0.0142543,0.0284396},
{-0.0150322,-0.0475557,0.0233783},
{0.0026011,-0.0371753,0.0569285},
{0.0145429,-0.0437865,0.0338790},
{0.0125703,-0.0098200,0.0117007},

Biology Open • Supplementary information

{0.0008877,0.0042585,-0.0171227},
{0.0075021,-0.0007399,-0.0169926},
{0.0089853,-0.0653445,0.0473385},
{0.0128327,-0.0590076,0.0449697},
{0.0150728,-0.0573314,0.0373928},
{0.0152452,-0.0663703,0.0316227},
{-0.0040599,-0.0724145,0.0216416},
{-0.0114061,0.0036156,0.0123047},
{0.0066370,-0.0010864,0.0485901},
{0.0165134,-0.0090514,0.0442912},
{0.0128747,-0.0228773,0.0529973},
{-0.0102390,-0.0345873,0.0223014},
{-0.0157825,-0.0072434,-0.0022485},
{0.0140423,-0.0799660,0.0241049},
{-0.0059942,-0.0055490,-0.0167254},
{0.0035343,0.0136192,-0.0130306},
{-0.0080250,-0.0319217,0.0140698},
{-0.0127734,0.0099606,-0.0091553},
{-0.0173366,0.0047902,-0.0014611},
{-0.0078358,-0.0676624,0.0347748},
{0.0100351,-0.0168141,-0.0008342},
{0.0108003,-0.0438498,0.0504578},
{0.0074975,-0.0178276,-0.0079040},
{0.0086888,-0.0134263,-0.0121142},
{0.0037206,-0.0573054,0.0166625},
{0.0077467,-0.0488846,0.0188788},
{0.0010587,-0.0732853,0.0176506},
{-0.0157603,-0.0540707,0.0265536},
{-0.0052394,-0.0013107,0.0458783},
{-0.0113610,-0.0296713,0.0523308},
{0.0155578,-0.0167193,0.0476038},
{0.0130164,-0.0741518,0.0424536},
{0.0163066,-0.0231283,0.0308567},
{0.0072544,-0.0306856,0.0585273},
{-0.0147750,-0.0031309,0.0087373},
{0.0125292,-0.0155507,0.0529699},
{0.0134565,-0.0590310,0.0286968},
{-0.0132173,-0.0483199,0.0198255},
{-0.0122210,-0.0321547,0.0463107},
{0.0120841,-0.0401242,0.0269073},
{-0.0068006,-0.0373335,0.0566651},
{0.0057749,-0.0074624,-0.0165945},
{0.0036009,-0.0148693,-0.0133449},
{-0.0125334,-0.0103967,0.0453313},
{-0.0090913,-0.0431561,0.0510196},
{0.0151903,-0.0521146,0.0416269},

Biology Open • Supplementary information

{-0.0147089,0.0107673,0.0013814},
{-0.0120326,0.0088431,0.0102631},
{-0.0016594,-0.0655748,0.0482843},
{0.0115746,-0.0089563,0.0300473},
{0.0121004,-0.0033791,0.0485711},
{0.0133074,-0.0055389,0.0380633},
{-0.0111510,-0.0129769,-0.0075828},
{0.0049977,-0.0211807,0.0020747},
{0.0003220,-0.0473078,0.0150301},
{0.0174780,0.0030202,-0.0066992},
{0.0149810,0.0015670,-0.0116161},
{0.0111728,-0.0074429,-0.0139320},
{-0.0114665,0.0013244,-0.0136307},
{-0.0128037,-0.0057678,-0.0116799},
{0.0154553,0.0127683,0.0028292},
{0.0092349,0.0184109,-0.0020251},
{0.0078827,0.0133937,-0.0116732},
{0.0070439,-0.0218045,0.0569605},
{-0.0006211,-0.0083291,0.0561801},
{-0.0001449,-0.0020185,0.0506306},
{0.0047035,-0.0132402,0.0574723},
{0.0073443,0.0070935,0.0146183},
{0.0107795,-0.0167963,0.0138621},
{0.0034455,-0.0244018,0.0584411},
{-0.0079826,-0.0166788,0.0215880},
{-0.0089469,-0.0168570,0.0107766},
{-0.0056707,-0.0096539,0.0241057},
{-0.0132010,-0.0489613,0.0315355},
{-0.0099463,-0.0591888,0.0365018},
{-0.0060710,-0.0280934,0.0083445},
{0.0181461,-0.0178651,0.0411941},
{0.0107227,-0.0081729,0.0246706},
{0.0037891,-0.0009137,0.0216140},
{-0.0031419,-0.0734325,0.0469957},
{-0.0021083,0.0211894,-0.0029124},
{-0.0010020,-0.0127697,0.0348488},
{-0.0095875,-0.0138778,0.0021942},
{0.0113651,-0.0278506,0.0228411},
{0.0090559,-0.0262673,0.0152618},
{-0.0096493,-0.0067219,0.0469106},
{0.0164289,-0.0320499,0.0427300},
{-0.0105881,-0.0517948,0.0370133},
{-0.0065527,-0.0782845,0.0335560},
{0.0038004,-0.0050790,0.0274822},
{-0.0026212,-0.0044580,-0.0177846},
{-0.0110631,-0.0336360,0.0519381},

Biology Open • Supplementary information

{-0.0026400,0.0086167,-0.0150346},
{-0.0037909,0.0160649,-0.0111031},
{0.0163254,-0.0830993,0.0323503},
{0.0032394,-0.0716637,0.0169537},
{0.0144409,-0.0857388,0.0397690},
{0.0084109,0.0185891,0.0047930},
{0.0103027,0.0088617,0.0129835},
{0.0009638,0.0146009,-0.0127414},
{0.0141035,-0.0113226,-0.0078808},
{0.0166090,-0.0074060,-0.0067583},
{-0.0076314,-0.0236007,0.0271021},
{-0.0095645,-0.0335608,0.0286480},
{-0.0117561,-0.0599440,0.0229168},
{-0.0012418,0.0025861,0.0178987},
{0.0036432,0.0071157,0.0154416},
{-0.0022064,-0.0027724,0.0228660},
{0.0002251,-0.0299532,0.0579978},
{0.0060507,-0.0422246,0.0533761},
{-0.0102474,-0.0193066,0.0559133},
{-0.0102237,-0.0256033,0.0552277},
{0.0079157,0.0197008,0.0023596},
{0.0149731,0.0137608,0.0012520},
{-0.0062385,-0.0106164,0.0569277},
{0.0133296,-0.0517103,0.0306286},
{-0.0108634,-0.0462696,0.0428170},
{-0.0131391,-0.0490686,0.0196127},
{0.0095914,-0.0820193,0.0450945},
{-0.0085751,-0.0245573,0.0157783},
{-0.0088387,-0.0149543,0.0151709},
{-0.0038729,0.0155609,0.0112588},
{0.0007825,-0.0663043,0.0498533},
{0.0051511,-0.0611295,0.0500065},
{-0.0019755,-0.0608263,0.0495565},
{0.0144964,-0.0716774,0.0276799},
{-0.0108944,-0.0177961,0.0436214},
{0.0093317,-0.0241797,0.0153586},
{0.0045075,-0.0510662,0.0522065},
{0.0072626,-0.0341283,0.0555051},
{0.0083422,-0.0819719,0.0176858},
{-0.0059274,-0.0514356,0.0506890},
{0.0156756,-0.0416167,0.0391771},
{-0.0104219,-0.0415997,0.0188429},
{0.0185263,-0.0200374,0.0333284},
{-0.0035226,-0.0592227,0.0180758},
{0.0145552,-0.0019340,0.0113699},
{0.0053963,0.0052472,0.0155746},

Biology Open • Supplementary information

{0.0008007,-0.0314903,0.0578930},
{-0.0129586,0.0133185,-0.0045168},
{0.0092731,0.0152130,0.0090451},
{-0.0093288,0.0155220,0.0072577},
{-0.0162678,-0.0053270,-0.0045955},
{-0.0163648,0.0004927,-0.0065920},
{-0.0042843,-0.0169480,0.0590670},
{-0.0114951,-0.0332554,0.0387281},
{0.0048841,-0.0453767,0.0533510},
{0.0108705,-0.0403001,0.0513545},
{-0.0076416,-0.0661069,0.0225864},
{0.0110429,0.0068596,0.0133705},
{0.0159394,-0.0103487,-0.0009981},
{-0.0151451,-0.0077754,0.0044490},
{-0.0111684,-0.0543926,0.0343189},
{-0.0063932,-0.0162800,-0.0033029},
{-0.0113090,-0.0266225,0.0415114},
{0.0040678,-0.0723309,0.0484753},
{0.0139654,-0.0482140,0.0457000},
{-0.0015171,-0.0336599,0.0097175},
{0.0054620,-0.0309104,0.0115828},
{0.0164131,-0.0067292,0.0050935},
{0.0158481,-0.0786215,0.0367466},
{-0.0079848,-0.0182745,0.0221232},
{-0.0007684,0.0125068,0.0141586},
{0.0064733,-0.0658775,0.0186481},
{0.0076887,-0.0602000,0.0494390},
{0.0131326,-0.0125117,-0.0082994},
{0.0004861,-0.0087834,0.0565987},
{-0.0062516,-0.0142288,-0.0124436},
{0.0138971,-0.0680114,0.0412793},
{0.0067219,-0.0177656,-0.0090826},
{-0.0092801,-0.0621846,0.0203913},
{0.0116379,-0.0379238,0.0254811},
{0.0135652,-0.0041253,0.0472946},
{0.0041474,-0.0073662,0.0344744},
{-0.0026543,-0.0595521,0.0500458},
{-0.0111927,-0.0573992,0.0334213},
{-0.0126354,-0.0110136,0.0473261},
{-0.0068705,0.0118255,-0.0128305},
{-0.0150085,0.0055763,0.0071369},
{0.0081714,-0.0656186,0.0194413},
{0.0021800,-0.0374604,0.0561293},
{-0.0118033,-0.0208813,0.0481432},
{0.0056290,-0.0059060,-0.0172086},
{0.0134654,-0.0271977,0.0494874},

Biology Open • Supplementary information

{0.0104571,-0.0144111,0.0543031},
{0.0091077,0.0019899,-0.0158505},
{0.0119809,-0.0077892,0.0150526},
{-0.0113102,0.0106217,0.0102376},
{0.0090542,-0.0503948,0.0504082},
{-0.0047772,-0.0365966,0.0573865},
{0.0169932,-0.0276921,0.0438275},
{0.0138504,-0.0126050,-0.0061389},
{-0.0130413,-0.0478169,0.0319744},
{-0.0066000,-0.0702098,0.0396417},
{0.0027963,-0.0501045,0.0528539},
{-0.0038582,-0.0394248,0.0122925},
{0.0097331,0.0101745,-0.0128160},
{-0.0018732,-0.0205619,-0.0007405},
{-0.0080122,-0.0697420,0.0265129},
{-0.0090271,-0.0033314,-0.0154131},
{-0.0025487,0.0206138,-0.0041002},
{-0.0083795,-0.0206516,0.0116389},
{-0.0093879,-0.0612645,0.0202356},
{-0.0051977,-0.0710552,0.0217864},
{0.0008331,0.0112721,-0.0146618},
{0.0173456,-0.0247055,0.0431533},
{-0.0051530,-0.0551357,0.0174822},
{0.0154112,-0.0345108,0.0348753},
{0.0153889,-0.0292539,0.0310719},
{-0.0125528,-0.0113659,-0.0008611},
{0.0116019,0.0133173,0.0089173},
{-0.0117037,-0.0103659,0.0061247},
{-0.0093530,-0.0334339,0.0316159},
{0.0157016,-0.0833335,0.0375294},
{0.0154598,-0.0098125,0.0492645},
{0.0164094,-0.0147746,0.0456399},
{0.0011909,-0.0251380,0.0044426},
{0.0094374,-0.0221673,0.0555806},
{-0.0024398,-0.0090520,0.0277775},
{-0.0168614,-0.0040259,-0.0015380},
{0.0024120,-0.0199308,-0.0033703},
{-0.0084705,-0.0674490,0.0326925},
{-0.0111417,-0.0260535,0.0538931},
{0.0156489,0.0049191,-0.0095206},
{-0.0129190,-0.0590109,0.0266482},
{-0.0133230,-0.0039792,0.0113157},
{0.0104910,-0.0076152,0.0239286},
{-0.0117472,-0.0405930,0.0283427},
{0.0131615,-0.0677925,0.0254625},
{0.0006729,0.0031913,-0.0173462},

Biology Open • Supplementary information

{0.0147657,-0.0733901,0.0277825},
{0.0060719,0.0060421,0.0149232},
{-0.0124945,0.0021396,0.0114581},
{0.0051170,-0.0406325,0.0146888},
{-0.0056635,-0.0180100,0.0301048},
{0.0179748,-0.0255153,0.0380271},
{-0.0112885,-0.0592440,0.0203472},
{0.0182419,0.0048398,-0.0026949},
{-0.0121374,-0.0594009,0.0247925},
{0.0098539,-0.0211681,0.0098841},
{0.0172667,-0.0110641,0.0405675},
{0.0075776,0.0018408,-0.0166885},
{0.0129067,-0.0078573,0.0120975},
{-0.0099222,-0.0601259,0.0359260},
{0.0004053,0.0194797,0.0082369},
{0.0024867,0.0031249,-0.0176306},
{0.0330259,-0.3813067,0.0188401},
{0.0044758,0.0193022,-0.0069805},
{-0.0119589,-0.0425603,0.0334590},
{-0.0277920,-0.3774451,-0.0241412},
{0.0020514,0.0218369,-0.0000287},
{0.0127237,-0.0232008,0.0238590},
{-0.0124979,-0.0010403,-0.0130383},
{0.0146558,-0.0292969,0.0475592},
{-0.0129737,0.0127157,0.0042560},
{0.0152793,-0.0379007,0.0359412},
{0.0136362,-0.0422819,0.0308283},
{-0.0117504,-0.0333511,0.0410849},
{0.0153427,-0.0535536,0.0395412},
{-0.0148095,-0.0465374,0.0246760},
{0.0027058,-0.0531678,0.0521811},
{0.0009629,-0.0495983,0.0155837},
{0.0189567,-0.0202096,0.0395286},
{0.0069989,-0.0738512,0.0473003},
{-0.0052658,-0.0801777,0.0250721},
{-0.0168004,-0.0020808,0.0027406},
{0.0127344,-0.0129544,0.0177169},
{0.0122479,-0.0643698,0.0246518},
{-0.0082221,-0.0238635,0.0246897},
{-0.0172492,-0.3869889,0.0320347},
{-0.0076541,-0.0538935,0.0468132},
{-0.0101280,-0.3817569,-0.0092221},
{0.0094394,-0.0417831,0.0200940},
{0.0095935,0.0140242,-0.0100416},
{-0.0215376,-0.3786068,0.0259207},
{0.0138272,-0.0513308,0.0314191},

{0.0002637,-0.0478650,0.0536957},
{-0.0097061,0.0174760,0.0025514},
{-0.0021804,-0.0161286,0.0589417},
{-0.0064460,-0.0071179,0.0216317},
{0.0171234,-0.0099537,0.0378631},
{0.0131133,-0.0236564,0.0512963},
{-0.0124865,-0.0105350,0.0466696},
{0.0175013,0.0079551,-0.0025172},
{-0.0088523,-0.0329548,0.0160764},
{-0.0002974,-0.0136415,-0.0147409},
{-0.0095149,-0.0581761,0.0396042},
{-0.0079510,0.0165404,0.0071908},
{0.0086191,0.0184894,-0.0026909},
{-0.0118275,-0.0261161,0.0511087},
{-0.0052805,-0.3790786,0.0363114},
{-0.0118582,-0.3842325,-0.0340335},
{-0.0117924,-0.3841809,-0.0333300},
{-0.0117266,-0.3841294,-0.0326266},
{-0.0116608,-0.3840779,-0.0319231},
{-0.0115950,-0.3840263,-0.0312197},
{-0.0115293,-0.3839748,-0.0305163},
{-0.0114635,-0.3839232,-0.0298128},
{-0.0113977,-0.3838717,-0.0291094},
{-0.0113319,-0.3838202,-0.0284059},
{-0.0112662,-0.3837686,-0.0277025},
{-0.0112004,-0.3837171,-0.0269990},
{-0.0111346,-0.3836655,-0.0262956},
{-0.0110688,-0.3836140,-0.0255921},
{-0.0110031,-0.3835625,-0.0248887},
{-0.0109373,-0.3835109,-0.0241852},
{-0.0108715,-0.3834594,-0.0234818},
{-0.0108057,-0.3834079,-0.0227783},
{-0.0107400,-0.3833563,-0.0220749},
{-0.0106742,-0.3833048,-0.0213714},
{-0.0106084,-0.3832532,-0.0206680},
{-0.0105426,-0.3832017,-0.0199645},
{-0.0104769,-0.3831502,-0.0192611},
{-0.0104111,-0.3830986,-0.0185576},
{-0.0103453,-0.3830471,-0.0178542},
{-0.0102795,-0.3829955,-0.0171507},
{-0.0102137,-0.3829440,-0.0164473},
{-0.0101480,-0.3828925,-0.0157438},
{-0.0100822,-0.3828409,-0.0150404},
{-0.0100164,-0.3827894,-0.0143369},
{-0.0099506,-0.3827378,-0.0136335},
{-0.0098849,-0.3826863,-0.0129300},

Biology Open • Supplementary information

{-0.0098191,-0.3826348,-0.0122266},
{-0.0097533,-0.3825832,-0.0115231},
{-0.0096875,-0.3825317,-0.0108197},
{-0.0096218,-0.3824801,-0.0101162},
{-0.0095560,-0.3824286,-0.0094128},
{-0.0094902,-0.3823771,-0.0087093},
{-0.0094244,-0.3823255,-0.0080059},
{-0.0093587,-0.3822740,-0.0073024},
{-0.0092929,-0.3822224,-0.0065990},
{-0.0092271,-0.3821709,-0.0058955},
{-0.0091613,-0.3821194,-0.0051921},
{-0.0090955,-0.3820678,-0.0044886},
{-0.0090298,-0.3820163,-0.0037852},
{-0.0089640,-0.3819648,-0.0030817},
{-0.0088982,-0.3819132,-0.0023783},
{-0.0088324,-0.3818617,-0.0016748},
{-0.0087667,-0.3818101,-0.0009714},
{-0.0087009,-0.3817586,-0.0002679},
{-0.0086351,-0.3817071,0.0004355},
{-0.0085693,-0.3816555,0.0011390},
{-0.0085036,-0.3816040,0.0018424},
{-0.0084378,-0.3815524,0.0025459},
{-0.0083720,-0.3815009,0.0032493},
{-0.0083062,-0.3814494,0.0039528},
{-0.0082405,-0.3813978,0.0046562},
{-0.0081747,-0.3813463,0.0053596},
{-0.0081089,-0.3812947,0.0060631},
{-0.0080431,-0.3812432,0.0067665},
{-0.0079774,-0.3811917,0.0074700},
{-0.0079116,-0.3811401,0.0081734},
{-0.0078458,-0.3810886,0.0088769},
{-0.0077800,-0.3810370,0.0095803},
{-0.0077142,-0.3809855,0.0102838},
{-0.0076485,-0.3809340,0.0109872},
{-0.0075827,-0.3808824,0.0116907},
{-0.0075169,-0.3808309,0.0123941},
{-0.0074511,-0.3807793,0.0130976},
{-0.0073854,-0.3807278,0.0138010},
{-0.0073196,-0.3806763,0.0145045},
{-0.0072538,-0.3806247,0.0152079},
{-0.0071880,-0.3805732,0.0159114},
{-0.0071223,-0.3805217,0.0166148},
{-0.0070565,-0.3804701,0.0173183},
{-0.0069907,-0.3804186,0.0180217},
{-0.0069249,-0.3803670,0.0187252},
{-0.0068592,-0.3803155,0.0194286},

Biology Open • Supplementary information

```
                    {-0.0067934,-0.3802640,0.0201321},
                    {-0.0067276,-0.3802124,0.0208355},
                    {-0.0066618,-0.3801609,0.0215390},
                    {-0.0065961,-0.3801093,0.0222424},
                    {-0.0065303,-0.3800578,0.0229459},
                    {-0.0064645,-0.3800063,0.0236493},
                    {-0.0063987,-0.3799547,0.0243528},
                    {-0.0063329,-0.3799032,0.0250562},
                    {-0.0062672,-0.3798516,0.0257597},
                    {-0.0062014,-0.3798001,0.0264631},
                    {-0.0061356,-0.3797486,0.0271666},
                    {-0.0060698,-0.3796970,0.0278700},
                    {-0.0060041,-0.3796455,0.0285735},
                    {-0.0059383,-0.3795939,0.0292769},
                    {-0.0058725,-0.3795424,0.0299804},
                    {-0.0058067,-0.3794909,0.0306838},
                    {-0.0057410,-0.3794393,0.0313873},
                    {-0.0056752,-0.3793878,0.0320907},
                    {-0.0056094,-0.3793362,0.0327942},
                    {-0.0055436,-0.3792847,0.0334976},
                    {-0.0054779,-0.3792332,0.0342011},
                    {-0.0054121,-0.3791816,0.0349045},
                    {-0.0053463,-0.3791301,0.0356080}
                    };
            BoundingBoxOnOff = Off;
        };
        AnyFunTransform3DIdentity ScaleFunction = {
            PreTransforms = {&.RBFTransform};
};
};

};
};
```

Biology Open • Supplementary information

ScalingFunctionTLEMLucyFemur_Mirrored_2014048

```
AnyFolder ScalingFunctionTLEMLucyFemur_Mirrored = {
  AnyFolder Left = {
          AnyFolder Thigh = {
       AnyMatrix AMirroring = {
       {1,0,0},
       {0,1,0},
       {0,0,-1}
      };
          AnyFunTransform3DRBF RBFTransform = {
                  RBFDef.Type = RBF_ThinPlate;
                  PolynomDegree = 1;
                  Points0 = ({
                          {0.0000000,0.0000000,0.0000000},
                          {-0.0000000,-0.3616821,0.0000000},
                          {-0.0097563,-0.3678799,0.0012967},
                          {-0.0000000,-0.3660632,0.0408203},
                          {-0.0000000,-0.3573010,-0.0408203},
                          {0.0161460,-0.0072838,0.0601290},
                          {0.0220217,-0.0203698,0.0463848},
                          {0.0123977,-0.0241932,0.0668573},
                          {0.0006898,0.0018121,0.0538181},
                          {0.0122809,-0.0068668,0.0414535},
                          {-0.0058991,-0.0138188,0.0648412},
                          {0.0211469,-0.0380855,0.0538111},
                          {0.0172133,-0.0317342,0.0328381},
                          {0.0177311,-0.0131946,0.0256176},
                          {-0.0110079,-0.0308867,0.0632370},
                          {0.0039435,-0.0395977,0.0638271},
                          {-0.0041683,-0.0187472,0.0413214},
                          {-0.0177999,-0.0021535,0.0496084},
                          {0.0001603,-0.0053126,0.0279593},
                          {-0.0211600,-0.0216243,0.0497147},
                          {0.0096163,-0.0568663,0.0545719},
                          {0.0162112,-0.0515013,0.0395230},
                          {0.0093533,-0.0433265,0.0187387},
                          {0.0119398,-0.0238469,0.0118403},
                          {0.0210707,-0.0048848,0.0065878},
                          {0.0125013,0.0064094,0.0177702},
                          {-0.0191523,-0.0356053,0.0382429},
                          {-0.0115821,-0.0474937,0.0537648},
                          {-0.0100073,-0.0236371,0.0267322},
                          {-0.0078925,0.0107098,0.0182833},
                          {-0.0134217,-0.0055356,0.0182000},
                          {-0.0090061,-0.0665700,0.0475484},
```

Biology Open • Supplementary information

{0.0071095,-0.0761976,0.0520035},
{0.0182119,-0.0704149,0.0386546},
{0.0073764,-0.0609481,0.0221006},
{-0.0122984,-0.0439585,0.0198428},
{-0.0032253,-0.0306792,0.0110995},
{-0.0012975,-0.0198451,-0.0052592},
{0.0140405,-0.0161125,-0.0071649},
{0.0189222,-0.0014898,-0.0127226},
{0.0181642,0.0132812,-0.0011050},
{0.0045119,0.0200001,0.0091189},
{-0.0176761,-0.0541983,0.0348587},
{-0.0136548,-0.0142015,0.0054311},
{-0.0135510,0.0182940,0.0017175},
{-0.0223865,0.0023399,0.0028018},
{-0.0086455,-0.0738247,0.0283843},
{-0.0050356,-0.0877749,0.0442195},
{0.0160135,-0.0919319,0.0465120},
{0.0128290,-0.0785810,0.0232431},
{-0.0168328,-0.0616327,0.0155917},
{-0.0172369,-0.0094567,-0.0119502},
{0.0002090,-0.0093641,-0.0209779},
{0.0064827,0.0081538,-0.0198453},
{0.0010777,0.0210422,-0.0084721},
{-0.0129485,0.0085451,-0.0162212},
{-0.0002322,-0.0924033,0.0245644},
{-0.0028175,-0.1052263,0.0418294},
{0.0172322,-0.1111444,0.0445760},
{0.0203259,-0.0957409,0.0271449},
{0.0057102,-0.1088933,0.0195113},
{-0.0010852,-0.1230776,0.0363132},
{0.0182727,-0.1303312,0.0422884},
{0.0242891,-0.1161703,0.0265054},
{0.0094531,-0.1268584,0.0162294},
{0.0014244,-0.1413293,0.0331653},
{0.0207271,-0.1496427,0.0394157},
{0.0267562,-0.1360142,0.0236798},
{0.0113839,-0.1461651,0.0136024},
{0.0030845,-0.1595094,0.0297156},
{0.0209528,-0.1691386,0.0369775},
{0.0283958,-0.1540209,0.0213795},
{0.0129499,-0.1649012,0.0113589},
{0.0016490,-0.1783108,0.0216287},
{0.0147105,-0.1880678,0.0328614},
{0.0298491,-0.1859865,0.0248551},
{0.0286645,-0.1714377,0.0169488},
{0.0165897,-0.1861006,0.0082595},

Biology Open • Supplementary information

{0.0014000,-0.1973476,0.0183740},
{0.0114301,-0.2109792,0.0286157},
{0.0267422,-0.2037622,0.0296951},
{0.0288630,-0.2018335,0.0130127},
{0.0145471,-0.2083505,0.0062833},
{0.0011300,-0.2198071,0.0155602},
{0.0126610,-0.2321235,0.0275354},
{0.0275647,-0.2219268,0.0265185},
{0.0286645,-0.2230253,0.0098701},
{0.0130434,-0.2304822,0.0039177},
{0.0022984,-0.2409585,0.0149650},
{0.0101315,-0.2534740,0.0252125},
{0.0279485,-0.2428555,0.0234276},
{0.0276684,-0.2421559,0.0058691},
{0.0101250,-0.2529527,0.0018983},
{0.0021376,-0.2667831,0.0107073},
{0.0114309,-0.2734905,0.0249960},
{0.0273923,-0.2634953,0.0216324},
{0.0278429,-0.2592546,0.0037692},
{0.0159101,-0.2712985,-0.0033988},
{0.0036708,-0.2863280,0.0004644},
{0.0023115,-0.2884756,0.0187554},
{0.0231385,-0.2879051,0.0224853},
{0.0304009,-0.2799973,0.0083549},
{0.0238189,-0.2917246,-0.0052462},
{0.0064904,-0.3027687,-0.0070587},
{-0.0003492,-0.3064690,0.0093014},
{0.0122179,-0.3045269,0.0243511},
{0.0301837,-0.3030127,0.0114462},
{0.0234182,-0.3107336,-0.0085198},
{0.0046893,-0.3214995,-0.0123881},
{-0.0022352,-0.3264905,0.0043340},
{0.0016841,-0.3202756,0.0217839},
{0.0229722,-0.3196887,0.0215083},
{0.0292438,-0.3228042,0.0051430},
{0.0221641,-0.3296190,-0.0132361},
{0.0100459,-0.3417597,-0.0244560},
{-0.0053009,-0.3375737,-0.0137705},
{-0.0064432,-0.3487307,0.0028676},
{-0.0059346,-0.3407801,0.0202938},
{0.0105130,-0.3365976,0.0264175},
{0.0282695,-0.3386249,0.0180307},
{0.0270402,-0.3431844,-0.0001487},
{0.0257040,-0.3506950,-0.0181851},
{0.0178948,-0.3636114,-0.0302838},
{0.0043149,-0.3544109,-0.0381949},

Biology Open • Supplementary information

{-0.0095210,-0.3461482,-0.0295031},
{-0.0164526,-0.3542895,-0.0126666},
{-0.0060896,-0.3690127,-0.0033061},
{-0.0176523,-0.3612686,0.0110109},
{-0.0153310,-0.3546943,0.0299634},
{0.0021329,-0.3508021,0.0350763},
{0.0210690,-0.3548628,0.0279835},
{0.0356594,-0.3558162,0.0159356},
{0.0275718,-0.3617450,-0.0018607},
{0.0298503,-0.3741530,-0.0163721},
{0.0175320,-0.3855300,-0.0277515},
{0.0025604,-0.3740099,-0.0397137},
{-0.0136727,-0.3624016,-0.0402102},
{-0.0277647,-0.3567472,-0.0284947},
{-0.0275446,-0.3699630,-0.0120829},
{-0.0175846,-0.3859355,-0.0177922},
{0.0001572,-0.3856873,-0.0118050},
{0.0030262,-0.3834856,0.0075553},
{-0.0144114,-0.3810887,0.0140304},
{-0.0247706,-0.3701239,0.0285134},
{-0.0044804,-0.3677201,0.0404632},
{0.0133183,-0.3712274,0.0334208},
{0.0324861,-0.3723417,0.0245668},
{0.0271451,-0.3766027,0.0072441},
{0.0167985,-0.3822816,-0.0065349},
{-0.0001454,-0.3910823,-0.0303010},
{-0.0144998,-0.3834154,-0.0384137},
{-0.0301321,-0.3750841,-0.0321336},
{0.0196491,-0.3864804,0.0210853},
{0.0015726,-0.3888034,0.0276569},
{-0.0146652,-0.3847848,0.0336913},
{-0.0014588,-0.0619951,0.0219778},
{-0.0118435,-0.0550626,0.0139639},
{0.0129812,-0.0349966,0.0673128},
{-0.0162907,-0.0105666,0.0597739},
{-0.0012885,-0.0520816,0.0562989},
{-0.0090599,-0.0631298,0.0172224},
{-0.0000222,-0.0771663,0.0231897},
{-0.0068148,0.0023885,-0.0160086},
{-0.0180544,-0.0425409,0.0446585},
{0.0020525,-0.0249904,0.0691549},
{-0.0157097,-0.0662315,0.0238393},
{-0.0010518,-0.0510686,0.0199353},
{-0.0208874,-0.0152865,0.0500034},
{0.0168316,-0.0280857,0.0666680},
{-0.0187392,-0.0282333,0.0403282},

Biology Open • Supplementary information

{0.0191622,-0.0351633,0.0622707},
{0.0001216,-0.0706289,0.0524049},
{-0.0169862,-0.0399062,0.0311827},
{-0.0185737,-0.0533066,0.0151224},
{-0.0219058,-0.0528723,0.0222263},
{-0.0028271,-0.0391620,0.0148452},
{0.0117900,-0.0553425,0.0235348},
{0.0219835,-0.0106400,0.0396338},
{-0.0025366,-0.0401046,0.0625273},
{0.0141017,-0.0018767,0.0198108},
{-0.0078770,-0.0473929,0.0173542},
{-0.0065351,-0.0766431,0.0470258},
{0.0065585,0.0145593,0.0161638},
{-0.0118588,0.0172875,-0.0085064},
{-0.0074087,-0.0627500,0.0506700},
{0.0071940,-0.0780068,0.0213362},
{0.0126736,-0.0708176,0.0244108},
{0.0074694,-0.0436450,0.0604196},
{0.0154186,-0.0139471,0.0064312},
{-0.0053535,-0.0213210,0.0040094},
{-0.0088733,0.0002018,0.0205402},
{-0.0112471,-0.0542608,0.0500266},
{-0.0061625,-0.0248673,0.0653244},
{0.0093695,-0.0065826,0.0614157},
{0.0138015,-0.0467322,0.0284333},
{-0.0020999,-0.0186885,-0.0116392},
{-0.0185640,-0.0225591,0.0566779},
{0.0175798,0.0124360,-0.0068467},
{-0.0039962,0.0217127,0.0053055},
{0.0014975,-0.0784591,0.0514056},
{-0.0172997,-0.0386971,0.0525252},
{0.0196540,-0.0268271,0.0416806},
{0.0135296,-0.0406504,0.0236882},
{0.0182471,0.0095959,0.0087906},
{0.0218667,0.0016804,0.0058793},
{0.0224975,-0.0022652,-0.0022994},
{0.0166750,-0.0191391,0.0236436},
{0.0119785,-0.0328723,0.0195376},
{0.0187631,-0.0133469,0.0308741},
{-0.0187543,-0.0461463,0.0257491},
{0.0039834,-0.0373928,0.0683089},
{0.0157029,-0.0423169,0.0385226},
{0.0164003,-0.0087069,0.0131181},
{0.0008253,0.0033370,-0.0212576},
{0.0101707,-0.0009724,-0.0205688},
{0.0102948,-0.0642683,0.0524532},

Biology Open • Supplementary information

{0.0152217,-0.0557944,0.0508978},
{0.0172973,-0.0565707,0.0419468},
{0.0167723,-0.0635334,0.0369834},
{-0.0047397,-0.0695325,0.0241398},
{-0.0152913,0.0035778,0.0161461},
{0.0091697,0.0009340,0.0575160},
{0.0196686,-0.0089533,0.0500006},
{0.0183998,-0.0212943,0.0622968},
{-0.0114340,-0.0316810,0.0250940},
{-0.0206442,-0.0092599,-0.0028425},
{0.0166868,-0.0784295,0.0276925},
{-0.0069678,-0.0054521,-0.0207011},
{0.0049490,0.0151109,-0.0160093},
{-0.0084025,-0.0318937,0.0162788},
{-0.0173679,0.0095594,-0.0108509},
{-0.0218912,0.0063457,-0.0020698},
{-0.0106547,-0.0668750,0.0397749},
{0.0136098,-0.0170666,-0.0018890},
{0.0141599,-0.0441405,0.0587594},
{0.0093255,-0.0183218,-0.0083008},
{0.0106367,-0.0128276,-0.0154269},
{0.0037350,-0.0549365,0.0209786},
{0.0087662,-0.0472902,0.0195496},
{0.0016839,-0.0708213,0.0221325},
{-0.0202977,-0.0530540,0.0301270},
{-0.0068584,0.0021781,0.0530002},
{-0.0144817,-0.0297218,0.0605833},
{0.0197015,-0.0165217,0.0560654},
{0.0156609,-0.0705363,0.0477736},
{0.0207876,-0.0221433,0.0330733},
{0.0092324,-0.0300767,0.0713160},
{-0.0203392,-0.0034488,0.0097332},
{0.0156978,-0.0143438,0.0629053},
{0.0143267,-0.0563519,0.0317489},
{-0.0161685,-0.0465391,0.0188591},
{-0.0196397,-0.0309875,0.0546496},
{0.0154804,-0.0380422,0.0305433},
{-0.0077774,-0.0351303,0.0637765},
{0.0063980,-0.0086419,-0.0201654},
{0.0030146,-0.0151094,-0.0165974},
{-0.0210080,-0.0096030,0.0507855},
{-0.0105684,-0.0411377,0.0586146},
{0.0180619,-0.0498960,0.0469835},
{-0.0192318,0.0116108,0.0024070},
{-0.0165651,0.0083622,0.0126870},
{-0.0014333,-0.0627138,0.0534746},

Biology Open • Supplementary information

{0.0145127,-0.0080178,0.0353667},
{0.0148374,-0.0012228,0.0566181},
{0.0153834,-0.0062427,0.0444727},
{-0.0155164,-0.0132103,-0.0096398},
{0.0068685,-0.0221022,0.0011547},
{0.0003332,-0.0458500,0.0183716},
{0.0205582,0.0040956,-0.0082969},
{0.0175752,0.0009340,-0.0148407},
{0.0142357,-0.0063623,-0.0169332},
{-0.0147285,0.0023412,-0.0164678},
{-0.0158781,-0.0064451,-0.0151346},
{0.0175951,0.0133456,0.0037559},
{0.0117660,0.0189899,-0.0030690},
{0.0096040,0.0151827,-0.0137450},
{0.0096565,-0.0215194,0.0662734},
{-0.0006793,-0.0074494,0.0631218},
{0.0007819,-0.0004519,0.0600781},
{0.0065377,-0.0125433,0.0654199},
{0.0090461,0.0073096,0.0188711},
{0.0151418,-0.0168302,0.0146465},
{0.0025166,-0.0231863,0.0688894},
{-0.0097891,-0.0152077,0.0253086},
{-0.0109149,-0.0177935,0.0119825},
{-0.0072079,-0.0099373,0.0272725},
{-0.0183727,-0.0467429,0.0356276},
{-0.0126871,-0.0584555,0.0428141},
{-0.0057054,-0.0273042,0.0103554},
{0.0215142,-0.0159693,0.0474531},
{0.0128492,-0.0070016,0.0287645},
{0.0052134,-0.0017047,0.0247463},
{-0.0024284,-0.0707498,0.0513049},
{-0.0036140,0.0220593,-0.0040484},
{-0.0002405,-0.0121253,0.0390050},
{-0.0119811,-0.0156737,0.0015445},
{0.0142082,-0.0279792,0.0246553},
{0.0122244,-0.0262013,0.0169829},
{-0.0146264,-0.0038855,0.0549384},
{0.0207450,-0.0325771,0.0496412},
{-0.0155040,-0.0497560,0.0437087},
{-0.0081119,-0.0756450,0.0387365},
{0.0039684,-0.0062047,0.0313900},
{-0.0023726,-0.0038892,-0.0220521},
{-0.0136977,-0.0339839,0.0602619},
{-0.0035399,0.0089081,-0.0188894},
{-0.0049820,0.0176571,-0.0132254},
{0.0193902,-0.0803191,0.0360429},

Biology Open • Supplementary information

{0.0040648,-0.0697109,0.0218205},
{0.0170470,-0.0818610,0.0456892},
{0.0104175,0.0188635,0.0064965},
{0.0135206,0.0092549,0.0158079},
{0.0023933,0.0160498,-0.0157404},
{0.0164753,-0.0118197,-0.0096995},
{0.0202584,-0.0066858,-0.0082383},
{-0.0098401,-0.0226001,0.0314028},
{-0.0130937,-0.0309622,0.0320764},
{-0.0201510,-0.0613297,0.0222264},
{-0.0010389,0.0036055,0.0209003},
{0.0042025,0.0073392,0.0199393},
{-0.0021630,-0.0038531,0.0251898},
{-0.0007845,-0.0293574,0.0675788},
{0.0070027,-0.0406327,0.0624565},
{-0.0124363,-0.0183001,0.0627966},
{-0.0120485,-0.0256613,0.0628493},
{0.0101200,0.0197695,0.0039334},
{0.0167636,0.0148266,0.0014155},
{-0.0074981,-0.0106930,0.0632797},
{0.0142533,-0.0503445,0.0351954},
{-0.0146107,-0.0460953,0.0506120},
{-0.0161117,-0.0471301,0.0178900},
{0.0112149,-0.0792793,0.0501460},
{-0.0099060,-0.0230794,0.0186951},
{-0.0114543,-0.0153049,0.0183675},
{-0.0042882,0.0161879,0.0151423},
{0.0014582,-0.0638572,0.0541080},
{0.0048554,-0.0582603,0.0553815},
{-0.0017077,-0.0572302,0.0546635},
{0.0166090,-0.0697169,0.0323038},
{-0.0208812,-0.0164273,0.0474354},
{0.0130454,-0.0241864,0.0178294},
{0.0053123,-0.0483770,0.0582251},
{0.0112225,-0.0361429,0.0673300},
{0.0085729,-0.0771256,0.0216100},
{-0.0070783,-0.0488033,0.0560869},
{0.0182083,-0.0411024,0.0454236},
{-0.0119590,-0.0403809,0.0213546},
{0.0233409,-0.0185535,0.0366343},
{-0.0046347,-0.0571211,0.0208243},
{0.0183166,-0.0006966,0.0140116},
{0.0061913,0.0053512,0.0201003},
{-0.0002364,-0.0310828,0.0680351},
{-0.0170773,0.0139270,-0.0054987},
{0.0110805,0.0156034,0.0117010},

Biology Open • Supplementary information

{-0.0124628,0.0164667,0.0094550},
{-0.0209014,-0.0073407,-0.0051969},
{-0.0210131,0.0010693,-0.0086891},
{-0.0041596,-0.0172332,0.0663041},
{-0.0216595,-0.0322172,0.0451540},
{0.0051908,-0.0426014,0.0613013},
{0.0150003,-0.0416136,0.0606257},
{-0.0135090,-0.0674234,0.0236425},
{0.0146416,0.0073691,0.0160052},
{0.0195615,-0.0112412,-0.0010333},
{-0.0198115,-0.0098244,0.0044735},
{-0.0157054,-0.0521236,0.0395039},
{-0.0081381,-0.0173108,-0.0046152},
{-0.0218324,-0.0262661,0.0471115},
{0.0051446,-0.0707257,0.0530555},
{0.0179359,-0.0471493,0.0528134},
{-0.0005591,-0.0317983,0.0113632},
{0.0071707,-0.0305943,0.0121186},
{0.0206239,-0.0061367,0.0066642},
{0.0187057,-0.0745868,0.0420207},
{-0.0095784,-0.0163113,0.0259524},
{-0.0012181,0.0127661,0.0186855},
{0.0059420,-0.0630691,0.0220087},
{0.0069408,-0.0573878,0.0552614},
{0.0150782,-0.0132884,-0.0101793},
{0.0005533,-0.0078574,0.0633775},
{-0.0088284,-0.0146399,-0.0147837},
{0.0167444,-0.0651105,0.0470347},
{0.0082666,-0.0181741,-0.0095133},
{-0.0175162,-0.0637444,0.0177069},
{0.0153795,-0.0360767,0.0287608},
{0.0162363,-0.0018864,0.0548412},
{0.0052731,-0.0075919,0.0392990},
{-0.0022288,-0.0556644,0.0549909},
{-0.0156238,-0.0550038,0.0377688},
{-0.0204137,-0.0100550,0.0536429},
{-0.0093683,0.0122429,-0.0162013},
{-0.0202927,0.0049166,0.0083887},
{0.0074706,-0.0627945,0.0222133},
{0.0035034,-0.0376103,0.0672421},
{-0.0190752,-0.0194129,0.0555530},
{0.0061758,-0.0074961,-0.0207741},
{0.0192011,-0.0273063,0.0588233},
{0.0130796,-0.0134383,0.0638611},
{0.0120421,0.0019566,-0.0194140},
{0.0154152,-0.0065294,0.0179581},

{-0.0157335,0.0101219,0.0123981},
{0.0110376,-0.0488274,0.0567911},
{-0.0055170,-0.0343676,0.0647140},
{0.0216204,-0.0273988,0.0512041},
{0.0161331,-0.0135875,-0.0075501},
{-0.0184643,-0.0455633,0.0360087},
{-0.0091978,-0.0682413,0.0460847},
{0.0034304,-0.0471677,0.0588866},
{-0.0048025,-0.0384316,0.0149400},
{0.0118505,0.0109735,-0.0159161},
{-0.0027036,-0.0204189,-0.0015094},
{-0.0117607,-0.0692759,0.0292171},
{-0.0107080,-0.0033455,-0.0190487},
{-0.0042548,0.0214947,-0.0056846},
{-0.0097315,-0.0208529,0.0131997},
{-0.0178958,-0.0627218,0.0171551},
{-0.0068692,-0.0688818,0.0233316},
{0.0012083,0.0120695,-0.0186179},
{0.0215073,-0.0236027,0.0503069},
{-0.0060262,-0.0534931,0.0189523},
{0.0179951,-0.0336785,0.0397267},
{0.0190509,-0.0284972,0.0336394},
{-0.0169188,-0.0133609,-0.0014203},
{0.0135722,0.0136491,0.0114695},
{-0.0150549,-0.0123230,0.0067722},
{-0.0148411,-0.0311945,0.0355412},
{0.0185490,-0.0794042,0.0431266},
{0.0193568,-0.0087326,0.0575704},
{0.0201026,-0.0144353,0.0531586},
{0.0025583,-0.0248211,0.0045714},
{0.0132689,-0.0214866,0.0652899},
{-0.0028669,-0.0099311,0.0309271},
{-0.0219487,-0.0054893,-0.0021853},
{0.0026180,-0.0203806,-0.0040620},
{-0.0116230,-0.0668518,0.0370152},
{-0.0133007,-0.0264159,0.0619176},
{0.0186167,0.0050491,-0.0120059},
{-0.0191359,-0.0591690,0.0288149},
{-0.0178121,-0.0041834,0.0141448},
{0.0123077,-0.0063648,0.0279743},
{-0.0157729,-0.0374010,0.0315335},
{0.0149328,-0.0659100,0.0290095},
{0.0006205,0.0021977,-0.0214613},
{0.0170484,-0.0715687,0.0325980},
{0.0068499,0.0061192,0.0196608},
{-0.0167075,0.0020024,0.0150382},

Biology Open • Supplementary information

{0.0059801,-0.0393117,0.0167489},
{-0.0072506,-0.0171067,0.0338751},
{0.0202363,-0.0251596,0.0420997},
{-0.0199311,-0.0601255,0.0171522},
{0.0216641,0.0053410,-0.0027841},
{-0.0197665,-0.0606125,0.0252972},
{0.0129986,-0.0214528,0.0098002},
{0.0201151,-0.0100929,0.0460501},
{0.0104137,0.0017765,-0.0203073},
{0.0166557,-0.0064123,0.0140197},
{-0.0124120,-0.0596549,0.0422637},
{0.0014885,0.0202590,0.0096781},
{0.0028720,0.0020207,-0.0219044},
{0.0376095,-0.3675427,0.0214537},
{0.0049949,0.0206417,-0.0079017},
{-0.0191723,-0.0407590,0.0391310},
{-0.0316406,-0.3638215,-0.0274841},
{0.0021392,0.0226920,0.0000985},
{0.0168435,-0.0229382,0.0264788},
{-0.0157304,-0.0005437,-0.0161456},
{0.0203722,-0.0299750,0.0562762},
{-0.0171077,0.0134146,0.0059615},
{0.0175519,-0.0370653,0.0413374},
{0.0154657,-0.0405127,0.0348980},
{-0.0215172,-0.0323502,0.0481925},
{0.0177998,-0.0515776,0.0444709},
{-0.0183804,-0.0448316,0.0275139},
{0.0033607,-0.0502028,0.0575894},
{0.0010325,-0.0479377,0.0191141},
{0.0224852,-0.0181821,0.0451780},
{0.0084089,-0.0720538,0.0521031},
{-0.0070880,-0.0772912,0.0288023},
{-0.0223712,-0.0028990,0.0024047},
{0.0173136,-0.0120017,0.0204612},
{0.0137772,-0.0623537,0.0276621},
{-0.0100390,-0.0231180,0.0292222},
{-0.0196395,-0.3730207,0.0364730},
{-0.0094445,-0.0526688,0.0528111},
{-0.0115309,-0.3679776,-0.0104995},
{0.0129812,-0.0410477,0.0224072},
{0.0117394,0.0154849,-0.0117191},
{-0.0245208,-0.3649412,0.0295111},
{0.0147476,-0.0502966,0.0364899},
{0.0006132,-0.0446771,0.0600524},
{-0.0118123,0.0191139,0.0031977},
{-0.0017185,-0.0163303,0.0664216},

Biology Open • Supplementary information

{-0.0085098,-0.0074564,0.0247745},
{0.0197459,-0.0086852,0.0430460},
{0.0187064,-0.0223532,0.0600966},
{-0.0206552,-0.0099712,0.0525024},
{0.0207525,0.0084646,-0.0024846},
{-0.0094915,-0.0325446,0.0184441},
{-0.0014464,-0.0140050,-0.0179819},
{-0.0122725,-0.0573444,0.0463125},
{-0.0106397,0.0177211,0.0094669},
{0.0111981,0.0191236,-0.0041491},
{-0.0162790,-0.0256279,0.0592687},
{-0.0060120,-0.3653959,0.0413411},
{-0.0135007,-0.3703638,-0.0387476},
{-0.0134258,-0.3703142,-0.0379467},
{-0.0133509,-0.3702645,-0.0371458},
{-0.0132760,-0.3702148,-0.0363450},
{-0.0132011,-0.3701651,-0.0355441},
{-0.0131262,-0.3701154,-0.0347432},
{-0.0130513,-0.3700658,-0.0339423},
{-0.0129765,-0.3700161,-0.0331414},
{-0.0129016,-0.3699664,-0.0323405},
{-0.0128267,-0.3699167,-0.0315396},
{-0.0127518,-0.3698670,-0.0307388},
{-0.0126769,-0.3698174,-0.0299379},
{-0.0126020,-0.3697677,-0.0291370},
{-0.0125271,-0.3697180,-0.0283361},
{-0.0124523,-0.3696683,-0.0275352},
{-0.0123774,-0.3696187,-0.0267343},
{-0.0123025,-0.3695690,-0.0259334},
{-0.0122276,-0.3695193,-0.0251325},
{-0.0121527,-0.3694696,-0.0243317},
{-0.0120778,-0.3694199,-0.0235308},
{-0.0120029,-0.3693703,-0.0227299},
{-0.0119280,-0.3693206,-0.0219290},
{-0.0118532,-0.3692709,-0.0211281},
{-0.0117783,-0.3692212,-0.0203272},
{-0.0117034,-0.3691715,-0.0195263},
{-0.0116285,-0.3691219,-0.0187255},
{-0.0115536,-0.3690722,-0.0179246},
{-0.0114787,-0.3690225,-0.0171237},
{-0.0114038,-0.3689728,-0.0163228},
{-0.0113289,-0.3689231,-0.0155219},
{-0.0112541,-0.3688735,-0.0147210},
{-0.0111792,-0.3688238,-0.0139201},
{-0.0111043,-0.3687741,-0.0131192},
{-0.0110294,-0.3687244,-0.0123184},

Biology Open • Supplementary information

{-0.0109545,-0.3686748,-0.0115175},
{-0.0108796,-0.3686251,-0.0107166},
{-0.0108047,-0.3685754,-0.0099157},
{-0.0107298,-0.3685257,-0.0091148},
{-0.0106550,-0.3684760,-0.0083139},
{-0.0105801,-0.3684264,-0.0075130},
{-0.0105052,-0.3683767,-0.0067121},
{-0.0104303,-0.3683270,-0.0059113},
{-0.0103554,-0.3682773,-0.0051104},
{-0.0102805,-0.3682276,-0.0043095},
{-0.0102056,-0.3681780,-0.0035086},
{-0.0101307,-0.3681283,-0.0027077},
{-0.0100559,-0.3680786,-0.0019068},
{-0.0099810,-0.3680289,-0.0011059},
{-0.0099061,-0.3679792,-0.0003051},
{-0.0098312,-0.3679296,0.0004958},
{-0.0097563,-0.3678799,0.0012967},
{-0.0096814,-0.3678302,0.0020976},
{-0.0096065,-0.3677805,0.0028985},
{-0.0095317,-0.3677308,0.0036994},
{-0.0094568,-0.3676812,0.0045003},
{-0.0093819,-0.3676315,0.0053012},
{-0.0093070,-0.3675818,0.0061020},
{-0.0092321,-0.3675321,0.0069029},
{-0.0091572,-0.3674825,0.0077038},
{-0.0090823,-0.3674328,0.0085047},
{-0.0090074,-0.3673831,0.0093056},
{-0.0089326,-0.3673334,0.0101065},
{-0.0088577,-0.3672837,0.0109074},
{-0.0087828,-0.3672341,0.0117082},
{-0.0087079,-0.3671844,0.0125091},
{-0.0086330,-0.3671347,0.0133100},
{-0.0085581,-0.3670850,0.0141109},
{-0.0084832,-0.3670353,0.0149118},
{-0.0084083,-0.3669857,0.0157127},
{-0.0083335,-0.3669360,0.0165136},
{-0.0082586,-0.3668863,0.0173145},
{-0.0081837,-0.3668366,0.0181153},
{-0.0081088,-0.3667869,0.0189162},
{-0.0080339,-0.3667373,0.0197171},
{-0.0079590,-0.3666876,0.0205180},
{-0.0078841,-0.3666379,0.0213189},
{-0.0078092,-0.3665882,0.0221198},
{-0.0077344,-0.3665386,0.0229207},
{-0.0076595,-0.3664889,0.0237215},
{-0.0075846,-0.3664392,0.0245224},

{-0.0075097,-0.3663895,0.0253233},
{-0.0074348,-0.3663398,0.0261242},
{-0.0073599,-0.3662902,0.0269251},
{-0.0072850,-0.3662405,0.0277260},
{-0.0072101,-0.3661908,0.0285269},
{-0.0071353,-0.3661411,0.0293278},
{-0.0070604,-0.3660914,0.0301286},
{-0.0069855,-0.3660418,0.0309295},
{-0.0069106,-0.3659921,0.0317304},
{-0.0068357,-0.3659424,0.0325313},
{-0.0067608,-0.3658927,0.0333322},
{-0.0066859,-0.3658430,0.0341331},
{-0.0066111,-0.3657934,0.0349340},
{-0.0065362,-0.3657437,0.0357348},
{-0.0064613,-0.3656940,0.0365357},
{-0.0063864,-0.3656443,0.0373366},
{-0.0063115,-0.3655947,0.0381375},
{-0.0062366,-0.3655450,0.0389384},
{-0.0061617,-0.3654953,0.0397393},
{-0.0060868,-0.3654456,0.0405402}
}* .AMirroring);
Points1 = ({
{0.0000000,0.0000000,0.0000000},
{-0.0000000,-0.4005477,0.0000000},
{-0.0090626,-0.4074115,0.0012045},
{-0.0000000,-0.4053996,0.0379177},
{-0.0000000,-0.3956958,-0.0379177},
{0.0134912,-0.0090013,0.0543928},
{0.0195756,-0.0234107,0.0428096},
{0.0094082,-0.0265536,0.0593382},
{0.0001520,-0.0004141,0.0484176},
{0.0109257,-0.0071200,0.0377325},
{-0.0057597,-0.0145826,0.0615408},
{0.0163852,-0.0403059,0.0490339},
{0.0146804,-0.0349983,0.0315081},
{0.0138666,-0.0150697,0.0238377},
{-0.0098668,-0.0334709,0.0587196},
{0.0032686,-0.0438153,0.0577509},
{-0.0029925,-0.0209807,0.0391695},
{-0.0121470,-0.0045028,0.0456190},
{-0.0000086,-0.0044170,0.0264678},
{-0.0119278,-0.0238926,0.0468946},
{0.0097996,-0.0634141,0.0515156},
{0.0155847,-0.0563363,0.0367000},
{0.0081241,-0.0475699,0.0184024},
{0.0093953,-0.0253342,0.0120053},

Biology Open • Supplementary information

{0.0178032,-0.0057478,0.0054876},
{0.0100742,0.0063371,0.0153148},
{-0.0118659,-0.0398539,0.0348988},
{-0.0097478,-0.0518079,0.0495840},
{-0.0089488,-0.0266687,0.0239571},
{-0.0059414,0.0111555,0.0153428},
{-0.0110261,-0.0061372,0.0151242},
{-0.0072163,-0.0732666,0.0429999},
{0.0061066,-0.0840718,0.0499518},
{0.0165959,-0.0788088,0.0354027},
{0.0080545,-0.0678671,0.0202205},
{-0.0114013,-0.0481882,0.0198723},
{-0.0040686,-0.0342532,0.0098188},
{-0.0004304,-0.0207896,-0.0046225},
{0.0123984,-0.0162939,-0.0062762},
{0.0167528,-0.0018731,-0.0107184},
{0.0164263,0.0135709,-0.0011668},
{0.0030361,0.0205947,0.0086675},
{-0.0137861,-0.0598187,0.0326083},
{-0.0114537,-0.0130713,0.0055814},
{-0.0117234,0.0177569,0.0015479},
{-0.0178840,0.0024617,0.0030539},
{-0.0065798,-0.0811533,0.0267032},
{-0.0041637,-0.0973039,0.0404902},
{0.0141278,-0.1021921,0.0431595},
{0.0120599,-0.0873128,0.0209665},
{-0.0097896,-0.0648157,0.0206412},
{-0.0140476,-0.0092506,-0.0098462},
{0.0006361,-0.0095534,-0.0179947},
{0.0057782,0.0081247,-0.0165634},
{0.0016258,0.0210162,-0.0078255},
{-0.0101696,0.0087925,-0.0140020},
{0.0004282,-0.1023677,0.0224565},
{-0.0017985,-0.1163820,0.0386090},
{0.0152147,-0.1232234,0.0411768},
{0.0181479,-0.1059326,0.0253672},
{0.0060344,-0.1202750,0.0186269},
{0.0000133,-0.1358949,0.0338084},
{0.0162852,-0.1442906,0.0390501},
{0.0216592,-0.1285784,0.0249034},
{0.0093879,-0.1400022,0.0159141},
{0.0022593,-0.1560161,0.0310453},
{0.0185307,-0.1655895,0.0364265},
{0.0239185,-0.1504636,0.0223866},
{0.0110874,-0.1613205,0.0135791},
{0.0037153,-0.1761144,0.0279368},

Biology Open • Supplementary information

{0.0188227,-0.1871262,0.0341941},
{0.0254206,-0.1703630,0.0202834},
{0.0124212,-0.1820760,0.0114906},
{0.0025539,-0.1968638,0.0206747},
{0.0135458,-0.2079764,0.0305105},
{0.0266731,-0.2058147,0.0232249},
{0.0257572,-0.1896096,0.0162607},
{0.0155208,-0.2056268,0.0085505},
{0.0022740,-0.2179810,0.0176532},
{0.0107292,-0.2333322,0.0266342},
{0.0239519,-0.2255224,0.0274938},
{0.0260025,-0.2232924,0.0125616},
{0.0137075,-0.2303030,0.0066284},
{0.0019201,-0.2429333,0.0149718},
{0.0117500,-0.2568309,0.0255453},
{0.0247259,-0.2456690,0.0245542},
{0.0258744,-0.2467997,0.0095996},
{0.0123341,-0.2548773,0.0043268},
{0.0027913,-0.2664749,0.0142666},
{0.0094975,-0.2805213,0.0233734},
{0.0251312,-0.2688895,0.0216765},
{0.0250593,-0.2680198,0.0058680},
{0.0096884,-0.2798362,0.0023208},
{0.0024883,-0.2951941,0.0102281},
{0.0105957,-0.3027779,0.0231018},
{0.0247274,-0.2917908,0.0199799},
{0.0252598,-0.2870083,0.0038393},
{0.0147644,-0.3002730,-0.0026943},
{0.0037397,-0.3169299,0.0007275},
{0.0024242,-0.3193567,0.0174327},
{0.0210861,-0.3188511,0.0207393},
{0.0275974,-0.3100786,0.0078499},
{0.0218623,-0.3230209,-0.0046192},
{0.0061627,-0.3352295,-0.0063739},
{-0.0001603,-0.3393486,0.0086947},
{0.0113036,-0.3372411,0.0225323},
{0.0276712,-0.3356008,0.0106095},
{0.0216492,-0.3441143,-0.0078256},
{0.0043558,-0.3560472,-0.0115072},
{-0.0020762,-0.3615745,0.0040258},
{0.0015643,-0.3546918,0.0202349},
{0.0213266,-0.3540439,0.0199733},
{0.0271570,-0.3574930,0.0047782},
{0.0205881,-0.3650392,-0.0122949},
{0.0093316,-0.3784845,-0.0227170},
{-0.0049240,-0.3738487,-0.0127913},

Biology Open • Supplementary information

{-0.0059850,-0.3862046,0.0026637},
{-0.0055126,-0.3773997,0.0188508},
{0.0097654,-0.3727677,0.0245390},
{0.0262603,-0.3750129,0.0167486},
{0.0251184,-0.3800623,-0.0001381},
{0.0238744,-0.3883799,-0.0168911},
{0.0166224,-0.4026843,-0.0281305},
{0.0040081,-0.3924952,-0.0354762},
{-0.0088440,-0.3833446,-0.0274053},
{-0.0152827,-0.3923608,-0.0117659},
{-0.0056566,-0.4086660,-0.0030710},
{-0.0163971,-0.4000898,0.0102279},
{-0.0142409,-0.3928091,0.0278328},
{0.0019812,-0.3884986,0.0325822},
{0.0195709,-0.3929957,0.0259937},
{0.0331229,-0.3940515,0.0148024},
{0.0256122,-0.4006175,-0.0017283},
{0.0277278,-0.4143587,-0.0152079},
{0.0162863,-0.4269583,-0.0257782},
{0.0023783,-0.4142003,-0.0368898},
{-0.0127014,-0.4013446,-0.0373519},
{-0.0257905,-0.3950825,-0.0264695},
{-0.0255860,-0.4097185,-0.0112237},
{-0.0163333,-0.4274074,-0.0165270},
{0.0001460,-0.4271325,-0.0109655},
{0.0028110,-0.4246942,0.0070181},
{-0.0133866,-0.4220397,0.0130327},
{-0.0230093,-0.4098967,0.0264859},
{-0.0041618,-0.4072346,0.0375860},
{0.0123712,-0.4111188,0.0310444},
{0.0301771,-0.4123528,0.0228200},
{0.0252149,-0.4170717,0.0067290},
{0.0156040,-0.4233609,-0.0060702},
{-0.0001350,-0.4331072,-0.0281473},
{-0.0134687,-0.4246165,-0.0356822},
{-0.0279895,-0.4153899,-0.0298487},
{0.0182509,-0.4280107,0.0195860},
{0.0014608,-0.4305834,0.0256913},
{-0.0136215,-0.4261330,0.0312956},
{-0.0007858,-0.0696747,0.0180321},
{-0.0094207,-0.0606532,0.0195544},
{0.0089801,-0.0355801,0.0585202},
{-0.0129573,-0.0144574,0.0539405},
{-0.0015883,-0.0592670,0.0542278},
{-0.0057475,-0.0665554,0.0212754},
{-0.0012873,-0.0855182,0.0194281},

{-0.0063823,0.0017668,-0.0160986},
{-0.0113780,-0.0465155,0.0389663},
{0.0030170,-0.0277269,0.0620457},
{-0.0092770,-0.0687534,0.0243346},
{-0.0009743,-0.0565076,0.0173762},
{-0.0120304,-0.0177457,0.0480797},
{0.0124310,-0.0313815,0.0569308},
{-0.0095559,-0.0311692,0.0375772},
{0.0130485,-0.0358692,0.0553369},
{-0.0008136,-0.0780615,0.0508276},
{-0.0135314,-0.0456994,0.0295855},
{-0.0127495,-0.0579495,0.0208128},
{-0.0148468,-0.0566786,0.0220155},
{-0.0022649,-0.0430732,0.0128468},
{0.0098896,-0.0603811,0.0226089},
{0.0198130,-0.0138106,0.0374413},
{-0.0028533,-0.0458846,0.0586054},
{0.0123874,-0.0029245,0.0162904},
{-0.0080938,-0.0506941,0.0178906},
{-0.0054562,-0.0842739,0.0419608},
{0.0051033,0.0153900,0.0124100},
{-0.0103443,0.0177069,-0.0056891},
{-0.0064606,-0.0700129,0.0449144},
{0.0071768,-0.0880590,0.0183474},
{0.0118356,-0.0772473,0.0229674},
{0.0070913,-0.0490382,0.0555769},
{0.0132193,-0.0131069,0.0071732},
{-0.0052905,-0.0244395,0.0043263},
{-0.0065845,-0.0010081,0.0189746},
{-0.0092507,-0.0583691,0.0462168},
{-0.0056741,-0.0271566,0.0621048},
{0.0077065,-0.0065940,0.0567220},
{0.0124653,-0.0524211,0.0279836},
{-0.0018387,-0.0188076,-0.0113839},
{-0.0126285,-0.0253642,0.0515685},
{0.0146743,0.0137996,-0.0066253},
{-0.0037177,0.0223623,0.0034516},
{0.0005784,-0.0881887,0.0492988},
{-0.0119004,-0.0409996,0.0466876},
{0.0181941,-0.0289921,0.0396440},
{0.0106133,-0.0445036,0.0225374},
{0.0163296,0.0107717,0.0067453},
{0.0188193,0.0021573,0.0063004},
{0.0196999,-0.0020532,-0.0031890},
{0.0126192,-0.0208508,0.0210246},
{0.0104747,-0.0358910,0.0200737},

Biology Open • Supplementary information

{0.0148661,-0.0152162,0.0300765},
{-0.0158974,-0.0507650,0.0247239},
{0.0027508,-0.0396841,0.0602052},
{0.0153800,-0.0467414,0.0358290},
{0.0132938,-0.0104827,0.0123741},
{0.0009388,0.0045459,-0.0181083},
{0.0079339,-0.0007898,-0.0179706},
{0.0095025,-0.0697542,0.0500632},
{0.0135713,-0.0629897,0.0475581},
{0.0159403,-0.0612004,0.0395451},
{0.0161227,-0.0708493,0.0334428},
{-0.0042936,-0.0773014,0.0228872},
{-0.0120626,0.0038596,0.0130129},
{0.0070190,-0.0011597,0.0513869},
{0.0174639,-0.0096622,0.0468405},
{0.0136157,-0.0244212,0.0560477},
{-0.0108284,-0.0369215,0.0235850},
{-0.0166909,-0.0077323,-0.0023779},
{0.0148506,-0.0853625,0.0254923},
{-0.0063392,-0.0059235,-0.0176881},
{0.0037378,0.0145383,-0.0137806},
{-0.0084869,-0.0340759,0.0148797},
{-0.0135086,0.0106328,-0.0096822},
{-0.0183345,0.0051135,-0.0015452},
{-0.0082869,-0.0722286,0.0367764},
{0.0106127,-0.0179488,-0.0008822},
{0.0114220,-0.0468090,0.0533621},
{0.0079291,-0.0190307,-0.0083590},
{0.0091889,-0.0143323,-0.0128115},
{0.0039347,-0.0611727,0.0176215},
{0.0081926,-0.0521836,0.0199655},
{0.0011197,-0.0782310,0.0186665},
{-0.0166675,-0.0577196,0.0280820},
{-0.0055410,-0.0013992,0.0485190},
{-0.0120149,-0.0316737,0.0553429},
{0.0164533,-0.0178476,0.0503438},
{0.0137656,-0.0791560,0.0448971},
{0.0172452,-0.0246891,0.0326328},
{0.0076720,-0.0327565,0.0618960},
{-0.0156255,-0.0033422,0.0092402},
{0.0132504,-0.0166001,0.0560187},
{0.0142310,-0.0630147,0.0303485},
{-0.0139781,-0.0515807,0.0209667},
{-0.0129244,-0.0343247,0.0489763},
{0.0127796,-0.0428320,0.0284560},
{-0.0071920,-0.0398530,0.0599266},

Biology Open • Supplementary information

{0.0061073,-0.0079660,-0.0175496},
{0.0038081,-0.0158727,-0.0141130},
{-0.0132548,-0.0110983,0.0479405},
{-0.0096145,-0.0460685,0.0539562},
{0.0160646,-0.0556316,0.0440229},
{-0.0155555,0.0114939,0.0014609},
{-0.0127251,0.0094399,0.0108539},
{-0.0017549,-0.0700002,0.0510634},
{0.0122408,-0.0095607,0.0317768},
{0.0127969,-0.0036071,0.0513668},
{0.0140733,-0.0059126,0.0402541},
{-0.0117928,-0.0138526,-0.0080193},
{0.0052854,-0.0226100,0.0021941},
{0.0003405,-0.0505003,0.0158953},
{0.0184840,0.0032240,-0.0070848},
{0.0158432,0.0016727,-0.0122847},
{0.0118158,-0.0079451,-0.0147338},
{-0.0121265,0.0014138,-0.0144153},
{-0.0135406,-0.0061570,-0.0123522},
{0.0163449,0.0136300,0.0029920},
{0.0097665,0.0196534,-0.0021417},
{0.0083365,0.0142976,-0.0123451},
{0.0074493,-0.0232760,0.0602390},
{-0.0006568,-0.0088912,0.0594137},
{-0.0001532,-0.0021547,0.0535448},
{0.0049743,-0.0141337,0.0607803},
{0.0077671,0.0075722,0.0154597},
{0.0114000,-0.0179298,0.0146600},
{0.0036438,-0.0260485,0.0618049},
{-0.0084421,-0.0178044,0.0228305},
{-0.0094618,-0.0179946,0.0113968},
{-0.0059971,-0.0103054,0.0254932},
{-0.0139608,-0.0522654,0.0333506},
{-0.0105188,-0.0631832,0.0386028},
{-0.0064204,-0.0299893,0.0088248},
{0.0191906,-0.0190708,0.0435651},
{0.0113399,-0.0087245,0.0260906},
{0.0040072,-0.0009754,0.0228581},
{-0.0033227,-0.0783880,0.0497007},
{-0.0022297,0.0226193,-0.0030800},
{-0.0010597,-0.0136315,0.0368546},
{-0.0101394,-0.0148143,0.0023205},
{0.0120192,-0.0297301,0.0241558},
{0.0095771,-0.0280400,0.0161403},
{-0.0102047,-0.0071755,0.0496106},
{0.0173745,-0.0342128,0.0451894},

Biology Open • Supplementary information

{-0.0111976,-0.0552901,0.0391437},
{-0.0069299,-0.0835675,0.0354874},
{0.0040191,-0.0054218,0.0290640},
{-0.0027720,-0.0047589,-0.0188083},
{-0.0116999,-0.0359059,0.0549276},
{-0.0027920,0.0091982,-0.0159000},
{-0.0040091,0.0171490,-0.0117421},
{0.0172651,-0.0887072,0.0342123},
{0.0034259,-0.0765000,0.0179296},
{0.0152721,-0.0915249,0.0420580},
{0.0088950,0.0198436,0.0050689},
{0.0108957,0.0094598,0.0137308},
{0.0010193,0.0155862,-0.0134747},
{0.0149152,-0.0120867,-0.0083344},
{0.0175650,-0.0079058,-0.0071473},
{-0.0080707,-0.0251934,0.0286621},
{-0.0101150,-0.0358256,0.0302970},
{-0.0124327,-0.0639894,0.0242359},
{-0.0013133,0.0027606,0.0189289},
{0.0038529,0.0075959,0.0163304},
{-0.0023334,-0.0029595,0.0241821},
{0.0002381,-0.0319745,0.0613361},
{0.0063989,-0.0450741,0.0564484},
{-0.0108372,-0.0206095,0.0591316},
{-0.0108121,-0.0273311,0.0584065},
{0.0083713,0.0210303,0.0024954},
{0.0158350,0.0146895,0.0013241},
{-0.0065976,-0.0113329,0.0602043},
{0.0140968,-0.0552000,0.0323916},
{-0.0114887,-0.0493921,0.0452815},
{-0.0138953,-0.0523800,0.0207415},
{0.0101435,-0.0875543,0.0476901},
{-0.0090687,-0.0262145,0.0166865},
{-0.0093474,-0.0159635,0.0160441},
{-0.0040958,0.0166110,0.0119069},
{0.0008275,-0.0707788,0.0527227},
{0.0054476,-0.0652548,0.0528848},
{-0.0020892,-0.0649312,0.0524089},
{0.0153308,-0.0765145,0.0292732},
{-0.0115214,-0.0189971,0.0461321},
{0.0098688,-0.0258115,0.0162426},
{0.0047669,-0.0545124,0.0552114},
{0.0076806,-0.0364315,0.0586999},
{0.0088223,-0.0875038,0.0187037},
{-0.0062686,-0.0549067,0.0536065},
{0.0165779,-0.0444252,0.0414321},

{-0.0110218,-0.0444070,0.0199275},
{0.0195926,-0.0213896,0.0352467},
{-0.0037254,-0.0632193,0.0191162},
{0.0153930,-0.0020645,0.0120243},
{0.0057069,0.0056013,0.0164710},
{0.0008468,-0.0336154,0.0612252},
{-0.0137045,0.0142173,-0.0047768},
{0.0098069,0.0162397,0.0095657},
{-0.0098658,0.0165695,0.0076755},
{-0.0172042,-0.0056865,-0.0048600},
{-0.0173067,0.0005260,-0.0069715},
{-0.0045309,-0.0180918,0.0624668},
{-0.0121567,-0.0354997,0.0409572},
{0.0051652,-0.0484390,0.0564218},
{0.0114962,-0.0430198,0.0543104},
{-0.0080814,-0.0705681,0.0238864},
{0.0116785,0.0073225,0.0141401},
{0.0168569,-0.0110471,-0.0010556},
{-0.0160169,-0.0083001,0.0047051},
{-0.0118112,-0.0580633,0.0362943},
{-0.0067612,-0.0173787,-0.0034931},
{-0.0119599,-0.0284192,0.0439007},
{0.0043019,-0.0772122,0.0512655},
{0.0147692,-0.0514677,0.0483304},
{-0.0016044,-0.0359315,0.0102768},
{0.0057763,-0.0329964,0.0122495},
{0.0173578,-0.0071833,0.0053867},
{0.0167603,-0.0839273,0.0388616},
{-0.0084444,-0.0195078,0.0233965},
{-0.0008126,0.0133508,0.0149736},
{0.0068459,-0.0703233,0.0197215},
{0.0081312,-0.0642626,0.0522846},
{0.0138885,-0.0133560,-0.0087771},
{0.0005140,-0.0093761,0.0598564},
{-0.0066114,-0.0151890,-0.0131599},
{0.0146969,-0.0726011,0.0436552},
{0.0071088,-0.0189645,-0.0096054},
{-0.0098143,-0.0663811,0.0215650},
{0.0123078,-0.0404831,0.0269478},
{0.0143460,-0.0044037,0.0500168},
{0.0043861,-0.0078633,0.0364586},
{-0.0028070,-0.0635709,0.0529263},
{-0.0118370,-0.0612728,0.0353449},
{-0.0133627,-0.0117568,0.0500501},
{-0.0072660,0.0126236,-0.0135690},
{-0.0158723,0.0059526,0.0075477},

Biology Open • Supplementary information

{0.0086417,-0.0700469,0.0205603},
{0.0023054,-0.0399884,0.0593600},
{-0.0124827,-0.0222904,0.0509142},
{0.0059530,-0.0063045,-0.0181991},
{0.0142405,-0.0290331,0.0523358},
{0.0110590,-0.0153836,0.0574286},
{0.0096319,0.0021242,-0.0167628},
{0.0126705,-0.0083148,0.0159190},
{-0.0119612,0.0113385,0.0108269},
{0.0095753,-0.0537957,0.0533096},
{-0.0050522,-0.0390663,0.0606896},
{0.0179713,-0.0295609,0.0463501},
{0.0146476,-0.0134557,-0.0064922},
{-0.0137920,-0.0510438,0.0338148},
{-0.0069799,-0.0749479,0.0419234},
{0.0029573,-0.0534858,0.0558961},
{-0.0040802,-0.0420854,0.0130000},
{0.0102933,0.0108611,-0.0135536},
{-0.0019810,-0.0219496,-0.0007831},
{-0.0084733,-0.0744485,0.0280390},
{-0.0095466,-0.0035562,-0.0163002},
{-0.0026954,0.0220050,-0.0043362},
{-0.0088618,-0.0220452,0.0123089},
{-0.0099282,-0.0653990,0.0214003},
{-0.0054968,-0.0758504,0.0230404},
{0.0008810,0.0120327,-0.0155057},
{0.0183440,-0.0263728,0.0456372},
{-0.0054496,-0.0588565,0.0184884},
{0.0162983,-0.0368397,0.0368826},
{0.0162747,-0.0312281,0.0328604},
{-0.0132753,-0.0121329,-0.0009107},
{0.0122697,0.0142160,0.0094306},
{-0.0123774,-0.0110654,0.0064772},
{-0.0098913,-0.0356902,0.0334356},
{0.0166054,-0.0889573,0.0396896},
{0.0163496,-0.0104747,0.0521001},
{0.0173538,-0.0157717,0.0482669},
{0.0012594,-0.0268344,0.0046983},
{0.0099806,-0.0236633,0.0587797},
{-0.0025802,-0.0096629,0.0293763},
{-0.0178319,-0.0042976,-0.0016265},
{0.0025509,-0.0212759,-0.0035643},
{-0.0089581,-0.0720008,0.0345742},
{-0.0117830,-0.0278117,0.0569951},
{0.0165496,0.0052510,-0.0100686},
{-0.0136626,-0.0629933,0.0281820},

Biology Open • Supplementary information

{-0.0140899,-0.0042478,0.0119670},
{0.0110949,-0.0081291,0.0253059},
{-0.0124233,-0.0433324,0.0299740},
{0.0139190,-0.0723675,0.0269281},
{0.0007117,0.0034066,-0.0183447},
{0.0156156,-0.0783429,0.0293816},
{0.0064214,0.0064498,0.0157821},
{-0.0132137,0.0022840,0.0121176},
{0.0054116,-0.0433745,0.0155342},
{-0.0059895,-0.0192254,0.0318375},
{0.0190094,-0.0272372,0.0402159},
{-0.0119383,-0.0632421,0.0215184},
{0.0192919,0.0051664,-0.0028500},
{-0.0128360,-0.0634096,0.0262195},
{0.0104211,-0.0225967,0.0104530},
{0.0182606,-0.0118107,0.0429025},
{0.0080138,0.0019651,-0.0176490},
{0.0136496,-0.0083876,0.0127938},
{-0.0104933,-0.0641835,0.0379938},
{0.0004287,0.0207943,0.0087110},
{0.0026298,0.0033358,-0.0186453},
{0.0349268,-0.4070391,0.0199245},
{0.0047334,0.0206048,-0.0073822},
{-0.0126473,-0.0454325,0.0353848},
{-0.0293917,-0.4029170,-0.0255308},
{0.0021695,0.0233106,-0.0000303},
{0.0134560,-0.0247665,0.0252322},
{-0.0132173,-0.0011105,-0.0137887},
{0.0154994,-0.0312740,0.0502966},
{-0.0137204,0.0135738,0.0045010},
{0.0161587,-0.0404584,0.0380099},
{0.0144211,-0.0451353,0.0326028},
{-0.0124268,-0.0356018,0.0434496},
{0.0162258,-0.0571677,0.0418171},
{-0.0156619,-0.0496780,0.0260963},
{0.0028616,-0.0567558,0.0551845},
{0.0010183,-0.0529455,0.0164807},
{0.0200478,-0.0215734,0.0418038},
{0.0074018,-0.0788350,0.0500229},
{-0.0055689,-0.0855885,0.0265152},
{-0.0177674,-0.0022212,0.0028983},
{0.0134674,-0.0138286,0.0187366},
{0.0129529,-0.0687138,0.0260707},
{-0.0086954,-0.0254739,0.0261107},
{-0.0182420,-0.4131048,0.0338786},
{-0.0080946,-0.0575305,0.0495076},

Biology Open • Supplementary information

{-0.0107110,-0.4075198,-0.0097529},
{0.0099827,-0.0446028,0.0212506},
{0.0101457,0.0149706,-0.0106196},
{-0.0227772,-0.4041571,0.0274127},
{0.0146230,-0.0547949,0.0332275},
{0.0002789,-0.0510952,0.0567863},
{-0.0102647,0.0186554,0.0026983},
{-0.0023059,-0.0172170,0.0623343},
{-0.0068170,-0.0075982,0.0228768},
{0.0181090,-0.0106254,0.0400425},
{0.0138681,-0.0252528,0.0542488},
{-0.0132052,-0.0112459,0.0493558},
{0.0185086,0.0084919,-0.0026621},
{-0.0093619,-0.0351788,0.0170017},
{-0.0003146,-0.0145621,-0.0155894},
{-0.0100626,-0.0621021,0.0418837},
{-0.0084087,0.0176566,0.0076047},
{0.0091152,0.0197371,-0.0028458},
{-0.0125083,-0.0278785,0.0540504},
{-0.0055845,-0.4046607,0.0384014},
{-0.0125407,-0.4101624,-0.0359924},
{-0.0124711,-0.4101074,-0.0352485},
{-0.0124016,-0.4100524,-0.0345045},
{-0.0123320,-0.4099973,-0.0337606},
{-0.0122624,-0.4099423,-0.0330166},
{-0.0121929,-0.4098873,-0.0322727},
{-0.0121233,-0.4098323,-0.0315288},
{-0.0120537,-0.4097773,-0.0307848},
{-0.0119842,-0.4097223,-0.0300409},
{-0.0119146,-0.4096672,-0.0292970},
{-0.0118451,-0.4096122,-0.0285530},
{-0.0117755,-0.4095572,-0.0278091},
{-0.0117059,-0.4095022,-0.0270651},
{-0.0116364,-0.4094472,-0.0263212},
{-0.0115668,-0.4093922,-0.0255773},
{-0.0114973,-0.4093371,-0.0248333},
{-0.0114277,-0.4092821,-0.0240894},
{-0.0113581,-0.4092271,-0.0233455},
{-0.0112886,-0.4091721,-0.0226015},
{-0.0112190,-0.4091171,-0.0218576},
{-0.0111494,-0.4090620,-0.0211136},
{-0.0110799,-0.4090070,-0.0203697},
{-0.0110103,-0.4089520,-0.0196258},
{-0.0109408,-0.4088970,-0.0188818},
{-0.0108712,-0.4088420,-0.0181379},
{-0.0108016,-0.4087870,-0.0173939},

Biology Open • Supplementary information

{-0.0107321,-0.4087319,-0.0166500},
{-0.0106625,-0.4086769,-0.0159061},
{-0.0105929,-0.4086219,-0.0151621},
{-0.0105234,-0.4085669,-0.0144182},
{-0.0104538,-0.4085119,-0.0136743},
{-0.0103843,-0.4084569,-0.0129303},
{-0.0103147,-0.4084018,-0.0121864},
{-0.0102451,-0.4083468,-0.0114424},
{-0.0101756,-0.4082918,-0.0106985},
{-0.0101060,-0.4082368,-0.0099546},
{-0.0100364,-0.4081818,-0.0092106},
{-0.0099669,-0.4081267,-0.0084667},
{-0.0098973,-0.4080717,-0.0077227},
{-0.0098278,-0.4080167,-0.0069788},
{-0.0097582,-0.4079617,-0.0062349},
{-0.0096886,-0.4079067,-0.0054909},
{-0.0096191,-0.4078517,-0.0047470},
{-0.0095495,-0.4077966,-0.0040031},
{-0.0094799,-0.4077416,-0.0032591},
{-0.0094104,-0.4076866,-0.0025152},
{-0.0093408,-0.4076316,-0.0017712},
{-0.0092713,-0.4075766,-0.0010273},
{-0.0092017,-0.4075216,-0.0002834},
{-0.0091321,-0.4074665,0.0004606},
{-0.0090626,-0.4074115,0.0012045},
{-0.0089930,-0.4073565,0.0019484},
{-0.0089234,-0.4073015,0.0026924},
{-0.0088539,-0.4072465,0.0034363},
{-0.0087843,-0.4071915,0.0041803},
{-0.0087148,-0.4071364,0.0049242},
{-0.0086452,-0.4070814,0.0056681},
{-0.0085756,-0.4070264,0.0064121},
{-0.0085061,-0.4069714,0.0071560},
{-0.0084365,-0.4069164,0.0079000},
{-0.0083670,-0.4068613,0.0086439},
{-0.0082974,-0.4068063,0.0093878},
{-0.0082278,-0.4067513,0.0101318},
{-0.0081583,-0.4066963,0.0108757},
{-0.0080887,-0.4066413,0.0116196},
{-0.0080191,-0.4065863,0.0123636},
{-0.0079496,-0.4065312,0.0131075},
{-0.0078800,-0.4064762,0.0138515},
{-0.0078105,-0.4064212,0.0145954},
{-0.0077409,-0.4063662,0.0153393},
{-0.0076713,-0.4063112,0.0160833},
{-0.0076018,-0.4062562,0.0168272},

Biology Open • Supplementary information

```
                    {-0.0075322,-0.4062011,0.0175712},
                    {-0.0074626,-0.4061461,0.0183151},
                    {-0.0073931,-0.4060911,0.0190590},
                    {-0.0073235,-0.4060361,0.0198030},
                    {-0.0072540,-0.4059811,0.0205469},
                    {-0.0071844,-0.4059261,0.0212908},
                    {-0.0071148,-0.4058710,0.0220348},
                    {-0.0070453,-0.4058160,0.0227787},
                    {-0.0069757,-0.4057610,0.0235227},
                    {-0.0069061,-0.4057060,0.0242666},
                    {-0.0068366,-0.4056510,0.0250105},
                    {-0.0067670,-0.4055959,0.0257545},
                    {-0.0066975,-0.4055409,0.0264984},
                    {-0.0066279,-0.4054859,0.0272424},
                    {-0.0065583,-0.4054309,0.0279863},
                    {-0.0064888,-0.4053759,0.0287302},
                    {-0.0064192,-0.4053209,0.0294742},
                    {-0.0063496,-0.4052658,0.0302181},
                    {-0.0062801,-0.4052108,0.0309620},
                    {-0.0062105,-0.4051558,0.0317060},
                    {-0.0061410,-0.4051008,0.0324499},
                    {-0.0060714,-0.4050458,0.0331939},
                    {-0.0060018,-0.4049908,0.0339378},
                    {-0.0059323,-0.4049357,0.0346817},
                    {-0.0058627,-0.4048807,0.0354257},
                    {-0.0057932,-0.4048257,0.0361696},
                    {-0.0057236,-0.4047707,0.0369135},
                    {-0.0056540,-0.4047157,0.0376575}
                    }* .AMirroring);
            BoundingBoxOnOff = Off;
        };
        AnyFunTransform3DIdentity ScaleFunction = {
            PreTransforms = {&.RBFTransform};
};
};

};
};
```

Biology Open • Supplementary information

ScalingFunctionTLEMLucyPelvis_2014048

```
AnyFolder ScalingFunctionTLEMLucyPelvis = {
AnyFolder Pelvis = {
	AnyFunTransform3DRBF RBFTransform = {
		RBFDef.Type = RBF_ThinPlate;
		PolynomDegree = 1;
		Points0 = {
			{0.0000000,0.0000000,0.1177000},
			{-0.0000000,-0.0832729,0.0191000},
			{-0.0508179,-0.0694062,0.0815920},
			{0.0000000,0.0000000,-0.1177000},
			{-0.0000000,-0.0832729,-0.0191000},
			{-0.0508179,-0.0694062,-0.0815920},
			{0.0000000,0.0000000,0.0000000},
			{-0.1164020,-0.0039449,0.0456760},
			{-0.1164020,-0.0039449,-0.0456760},
			{-0.1092909,-0.0992949,0.0515590},
			{-0.1092909,-0.0992949,-0.0515590},
			{-0.0733749,0.0757445,0.0915590},
			{-0.0733749,0.0757445,-0.0915590},
			{-0.1241738,0.0316243,0.0458050},
			{-0.1241738,0.0316243,-0.0458050},
			{-0.1108008,0.0563490,0.0555690},
			{-0.1108008,0.0563490,-0.0555690},
			{-0.0545356,0.0481653,0.1291690},
			{-0.0545356,0.0481653,-0.1291690},
			{-0.0360236,0.0456352,0.1316250},
			{-0.0360236,0.0456352,-0.1316250},
			{-0.0804664,-0.0228481,0.0672140},
			{-0.0804664,-0.0228481,-0.0672140},
			{-0.1004070,-0.0623213,0.0494990},
			{-0.1004070,-0.0623213,-0.0494990},
			{-0.0174901,-0.0357786,0.0977400},
			{-0.0174901,-0.0357786,-0.0977400},
			{-0.0665950,-0.1308418,0.0278190},
			{-0.0665950,-0.1308418,-0.0278190},
			{-0.0962739,-0.1257274,0.0551670},
			{-0.0962739,-0.1257274,-0.0551670},
			{-0.0393340,-0.1196098,0.0159820},
			{-0.0393340,-0.1196098,-0.0159820},
			{-0.0585979,-0.0148191,0.0651020},
			{-0.0585979,-0.0148191,-0.0651020},
			{-0.0238420,-0.0800479,0.0351680},
			{-0.0238420,-0.0800479,-0.0351680},
			{-0.0234553,-0.0576924,0.0715330},
```

Biology Open • Supplementary information

{-0.0234553,-0.0576924,-0.0715330},
{-0.0472319,-0.0833276,0.0513790},
{-0.0472319,-0.0833276,-0.0513790},
{-0.0327175,-0.0506481,0.0520240},
{-0.0327175,-0.0506481,-0.0520240},
{-0.0746024,-0.0627219,0.0928110},
{-0.0746024,-0.0627219,-0.0928110},
{-0.0119676,-0.0928080,0.0076900},
{-0.0119676,-0.0928080,-0.0076900},
{-0.0263299,-0.0760728,0.0667080},
{-0.0263299,-0.0760728,-0.0667080},
{-0.0498842,0.0119450,0.1070630},
{-0.0498842,0.0119450,-0.1070630},
{-0.0553874,0.0627474,0.1172390},
{-0.0553874,0.0627474,-0.1172390},
{-0.0786774,0.0073556,0.0741800},
{-0.0786774,0.0073556,-0.0741800},
{-0.0742762,0.0474272,0.0542330},
{-0.0742762,0.0474272,-0.0542330},
{-0.0634121,0.0117185,0.0618430},
{-0.0634121,0.0117185,-0.0618430},
{-0.0813084,0.0007821,0.0508130},
{-0.0813084,0.0007821,-0.0508130},
{-0.0496423,-0.0444458,0.0983920},
{-0.0496423,-0.0444458,-0.0983920},
{-0.0480005,-0.0338999,0.0625080},
{-0.0480005,-0.0338999,-0.0625080},
{-0.0428000,-0.0273188,0.0973490},
{-0.0428000,-0.0273188,-0.0973490},
{-0.0548383,-0.0518766,0.0624050},
{-0.0548383,-0.0518766,-0.0624050},
{-0.0696448,-0.1052827,0.0436200},
{-0.0696448,-0.1052827,-0.0436200},
{-0.0739608,-0.0803177,0.0785950},
{-0.0739608,-0.0803177,-0.0785950},
{-0.0586066,-0.0884764,0.0653190},
{-0.0586066,-0.0884764,-0.0653190},
{-0.1017003,0.0085215,0.0624150},
{-0.1017003,0.0085215,-0.0624150},
{-0.0126252,0.0252852,0.1292530},
{-0.0126252,0.0252852,-0.1292530},
{-0.0030963,0.0150156,0.1262640},
{-0.0030963,0.0150156,-0.1262640},
{-0.0621797,0.0048743,0.0605920},
{-0.0621797,0.0048743,-0.0605920},
{-0.0870082,-0.0684280,0.0489900},

Biology Open • Supplementary information

{-0.0870082,-0.0684280,-0.0489900},
{-0.0916211,-0.0478344,0.0575290},
{-0.0916211,-0.0478344,-0.0575290},
{-0.0428573,-0.0467026,0.0985250},
{-0.0428573,-0.0467026,-0.0985250},
{-0.0483869,-0.0455474,0.0848580},
{-0.0483869,-0.0455474,-0.0848580},
{-0.0203929,-0.0681811,0.0426070},
{-0.0203929,-0.0681811,-0.0426070},
{-0.0271973,-0.0807786,0.0491650},
{-0.0271973,-0.0807786,-0.0491650},
{-0.0325935,-0.1026813,0.0148280},
{-0.0325935,-0.1026813,-0.0148280},
{-0.0273900,-0.1059173,0.0048750},
{-0.0273900,-0.1059173,-0.0048750},
{-0.0015971,-0.0778770,0.0065530},
{-0.0015971,-0.0778770,-0.0065530},
{-0.1034983,-0.0140026,0.0521800},
{-0.1034983,-0.0140026,-0.0521800},
{-0.1141160,0.0077734,0.0497600},
{-0.1141160,0.0077734,-0.0497600},
{-0.1123797,0.0244808,0.0350160},
{-0.1123797,0.0244808,-0.0350160},
{-0.0984470,-0.0648210,0.0457100},
{-0.0984470,-0.0648210,-0.0457100},
{-0.0814332,-0.0704479,0.0806410},
{-0.0814332,-0.0704479,-0.0806410},
{-0.0540218,-0.0805070,0.0547320},
{-0.0540218,-0.0805070,-0.0547320},
{-0.0674356,-0.0763171,0.0651080},
{-0.0674356,-0.0763171,-0.0651080},
{-0.0602364,-0.0751827,0.0490600},
{-0.0602364,-0.0751827,-0.0490600},
{-0.0604272,-0.0801096,0.0617610},
{-0.0604272,-0.0801096,-0.0617610},
{-0.0655251,0.0632155,0.1093750},
{-0.0655251,0.0632155,-0.1093750},
{-0.0665046,0.0014289,0.0572170},
{-0.0665046,0.0014289,-0.0572170},
{-0.1053786,0.0347803,0.0071580},
{-0.1053786,0.0347803,-0.0071580},
{-0.1321649,-0.0069725,0.0058340},
{-0.1321649,-0.0069725,-0.0058340},
{-0.1232735,-0.0434903,0.0301110},
{-0.1232735,-0.0434903,-0.0301110},
{-0.0497024,0.0261862,0.0084640},

Biology Open • Supplementary information

{-0.0497024,0.0261862,-0.0084640},
{-0.0887827,0.0446048,0.0327030},
{-0.0887827,0.0446048,-0.0327030},
{-0.1153998,-0.0126010,0.0290730},
{-0.1153998,-0.0126010,-0.0290730},
{-0.0899939,0.0093882,0.0208440},
{-0.0899939,0.0093882,-0.0208440},
{-0.1312654,-0.0574898,0.0094690},
{-0.1312654,-0.0574898,-0.0094690},
{-0.0996029,0.0042806,0.0105620},
{-0.0996029,0.0042806,-0.0105620},
{-0.1226177,-0.0324069,0.0331630},
{-0.1226177,-0.0324069,-0.0331630},
{-0.1097808,-0.0051619,0.0102520},
{-0.1097808,-0.0051619,-0.0102520}
};
Points1 = {
{0.0000000,0.0000000,0.1189982},
{-0.0000007,-0.0873505,0.0173277},
{-0.0298398,-0.0678300,0.0828097},
{0.0000000,0.0000000,-0.1189982},
{-0.0000007,-0.0873505,-0.0173277},
{-0.0298398,-0.0678300,-0.0828097},
{0.0000000,0.0000000,0.0000000},
{-0.0814765,-0.0285255,0.0403088},
{-0.0814765,-0.0285255,-0.0403088},
{-0.0772270,-0.0959328,0.0588535},
{-0.0772270,-0.0959328,-0.0588535},
{-0.0530190,0.0401187,0.0867894},
{-0.0530190,0.0401187,-0.0867894},
{-0.0860153,-0.0071465,0.0357378},
{-0.0860153,-0.0071465,-0.0357378},
{-0.0780814,0.0150271,0.0474515},
{-0.0780814,0.0150271,-0.0474515},
{-0.0327316,0.0307932,0.1232205},
{-0.0327316,0.0307932,-0.1232205},
{-0.0213306,0.0311041,0.1272656},
{-0.0213306,0.0311041,-0.1272656},
{-0.0576636,-0.0387048,0.0653369},
{-0.0576636,-0.0387048,-0.0653369},
{-0.0720452,-0.0707535,0.0604398},
{-0.0720452,-0.0707535,-0.0604398},
{-0.0107364,-0.0379259,0.0893397},
{-0.0107364,-0.0379259,-0.0893397},
{-0.0498997,-0.1208119,0.0324805},
{-0.0498997,-0.1208119,-0.0324805},

{-0.0675036,-0.1119944,0.0618936},
{-0.0675036,-0.1119944,-0.0618936},
{-0.0280740,-0.1157768,0.0181211},
{-0.0280740,-0.1157768,-0.0181211},
{-0.0448679,-0.0275844,0.0631313},
{-0.0448679,-0.0275844,-0.0631313},
{-0.0142652,-0.0796456,0.0327308},
{-0.0142652,-0.0796456,-0.0327308},
{-0.0150120,-0.0566132,0.0701727},
{-0.0150120,-0.0566132,-0.0701727},
{-0.0278712,-0.0770952,0.0538414},
{-0.0278712,-0.0770952,-0.0538414},
{-0.0233316,-0.0568324,0.0546097},
{-0.0233316,-0.0568324,-0.0546097},
{-0.0473097,-0.0636218,0.0870971},
{-0.0473097,-0.0636218,-0.0870971},
{-0.0068250,-0.0969066,0.0074397},
{-0.0068250,-0.0969066,-0.0074397},
{-0.0159519,-0.0682167,0.0639308},
{-0.0159519,-0.0682167,-0.0639308},
{-0.0345551,-0.0009581,0.1013494},
{-0.0345551,-0.0009581,-0.1013494},
{-0.0368819,0.0382944,0.1124203},
{-0.0368819,0.0382944,-0.1124203},
{-0.0585860,-0.0137537,0.0662153},
{-0.0585860,-0.0137537,-0.0662153},
{-0.0561066,0.0135105,0.0477173},
{-0.0561066,0.0135105,-0.0477173},
{-0.0509435,-0.0098746,0.0536895},
{-0.0509435,-0.0098746,-0.0536895},
{-0.0613261,-0.0222628,0.0445768},
{-0.0613261,-0.0222628,-0.0445768},
{-0.0297013,-0.0487381,0.0900992},
{-0.0297013,-0.0487381,-0.0900992},
{-0.0342222,-0.0419501,0.0643885},
{-0.0342222,-0.0419501,-0.0643885},
{-0.0276311,-0.0336881,0.0904517},
{-0.0276311,-0.0336881,-0.0904517},
{-0.0353558,-0.0563978,0.0659757},
{-0.0353558,-0.0563978,-0.0659757},
{-0.0478002,-0.0989660,0.0505783},
{-0.0478002,-0.0989660,-0.0505783},
{-0.0465573,-0.0777861,0.0792240},
{-0.0465573,-0.0777861,-0.0792240},
{-0.0354984,-0.0846238,0.0706294},
{-0.0354984,-0.0846238,-0.0706294},

Biology Open • Supplementary information

{-0.0725937,-0.0166464,0.0549058},
{-0.0725937,-0.0166464,-0.0549058},
{-0.0071029,0.0183320,0.1275899},
{-0.0071029,0.0183320,-0.1275899},
{-0.0012763,0.0115586,0.1260201},
{-0.0012763,0.0115586,-0.1260201},
{-0.0506930,-0.0148107,0.0523739},
{-0.0506930,-0.0148107,-0.0523739},
{-0.0606032,-0.0733309,0.0600376},
{-0.0606032,-0.0733309,-0.0600376},
{-0.0643087,-0.0586653,0.0627759},
{-0.0643087,-0.0586653,-0.0627759},
{-0.0245096,-0.0505513,0.0896640},
{-0.0245096,-0.0505513,-0.0896640},
{-0.0300086,-0.0498253,0.0805805},
{-0.0300086,-0.0498253,-0.0805805},
{-0.0126637,-0.0705281,0.0396563},
{-0.0126637,-0.0705281,-0.0396563},
{-0.0161285,-0.0769605,0.0469870},
{-0.0161285,-0.0769605,-0.0469870},
{-0.0201839,-0.1023783,0.0165095},
{-0.0201839,-0.1023783,-0.0165095},
{-0.0149827,-0.1056040,0.0064494},
{-0.0149827,-0.1056040,-0.0064494},
{-0.0017344,-0.0861147,0.0059849},
{-0.0017344,-0.0861147,-0.0059849},
{-0.0728681,-0.0341316,0.0500943},
{-0.0728681,-0.0341316,-0.0500943},
{-0.0802183,-0.0188173,0.0424686},
{-0.0802183,-0.0188173,-0.0424686},
{-0.0787555,-0.0113047,0.0273049},
{-0.0787555,-0.0113047,-0.0273049},
{-0.0710402,-0.0725779,0.0591641},
{-0.0710402,-0.0725779,-0.0591641},
{-0.0527360,-0.0710810,0.0806331},
{-0.0527360,-0.0710810,-0.0806331},
{-0.0315583,-0.0741479,0.0584669},
{-0.0315583,-0.0741479,-0.0584669},
{-0.0419413,-0.0758231,0.0705194},
{-0.0419413,-0.0758231,-0.0705194},
{-0.0375111,-0.0738295,0.0551093},
{-0.0375111,-0.0738295,-0.0551093},
{-0.0363379,-0.0784351,0.0680255},
{-0.0363379,-0.0784351,-0.0680255},
{-0.0456795,0.0350386,0.1044333},
{-0.0456795,0.0350386,-0.1044333},

Biology Open • Supplementary information

```
				{-0.0539046,-0.0185329,0.0486502},
				{-0.0539046,-0.0185329,-0.0486502},
				{-0.0745547,-0.0058593,0.0054873},
				{-0.0745547,-0.0058593,-0.0054873},
				{-0.0911188,-0.0369901,0.0043664},
				{-0.0911188,-0.0369901,-0.0043664},
				{-0.0902180,-0.0640398,0.0204981},
				{-0.0902180,-0.0640398,-0.0204981},
				{-0.0406700,-0.0066948,0.0074582},
				{-0.0406700,-0.0066948,-0.0074582},
				{-0.0652318,0.0060112,0.0270244},
				{-0.0652318,0.0060112,-0.0270244},
				{-0.0808123,-0.0385043,0.0247068},
				{-0.0808123,-0.0385043,-0.0247068},
				{-0.0651587,-0.0222665,0.0174891},
				{-0.0651587,-0.0222665,-0.0174891},
				{-0.0961659,-0.0749316,0.0064065},
				{-0.0961659,-0.0749316,-0.0064065},
				{-0.0693849,-0.0285922,0.0087757},
				{-0.0693849,-0.0285922,-0.0087757},
				{-0.0881225,-0.0544929,0.0260964},
				{-0.0881225,-0.0544929,-0.0260964},
				{-0.0749345,-0.0362792,0.0084086},
				{-0.0749345,-0.0362792,-0.0084086}
				};
			BoundingBoxOnOff = Off;
		};
		AnyFunTransform3DIdentity ScaleFunction = {
			PreTransforms = {&.RBFTransform};
};
};
AnyFolder Sacrum = {
		AnyFunTransform3DRBF RBFTransform = {
			RBFDef.Type = RBF_ThinPlate;
			PolynomDegree = 1;
			Points0 = {
				{0.0000000,0.0000000,0.1177000},
				{-0.0000000,-0.0832729,0.0191000},
				{-0.0508179,-0.0694062,0.0815920},
				{0.0000000,0.0000000,-0.1177000},
				{-0.0000000,-0.0832729,-0.0191000},
				{-0.0508179,-0.0694062,-0.0815920},
				{0.0000000,0.0000000,0.0000000},
				{-0.1164020,-0.0039449,0.0456760},
				{-0.1164020,-0.0039449,-0.0456760},
				{-0.1092909,-0.0992949,0.0515590},
```

Biology Open • Supplementary information

{-0.1092909,-0.0992949,-0.0515590},
{-0.0733749,0.0757445,0.0915590},
{-0.0733749,0.0757445,-0.0915590},
{-0.1241738,0.0316243,0.0458050},
{-0.1241738,0.0316243,-0.0458050},
{-0.1108008,0.0563490,0.0555690},
{-0.1108008,0.0563490,-0.0555690},
{-0.0545356,0.0481653,0.1291690},
{-0.0545356,0.0481653,-0.1291690},
{-0.0360236,0.0456352,0.1316250},
{-0.0360236,0.0456352,-0.1316250},
{-0.0804664,-0.0228481,0.0672140},
{-0.0804664,-0.0228481,-0.0672140},
{-0.1004070,-0.0623213,0.0494990},
{-0.1004070,-0.0623213,-0.0494990},
{-0.0174901,-0.0357786,0.0977400},
{-0.0174901,-0.0357786,-0.0977400},
{-0.0665950,-0.1308418,0.0278190},
{-0.0665950,-0.1308418,-0.0278190},
{-0.0962739,-0.1257274,0.0551670},
{-0.0962739,-0.1257274,-0.0551670},
{-0.0393340,-0.1196098,0.0159820},
{-0.0393340,-0.1196098,-0.0159820},
{-0.0585979,-0.0148191,0.0651020},
{-0.0585979,-0.0148191,-0.0651020},
{-0.0238420,-0.0800479,0.0351680},
{-0.0238420,-0.0800479,-0.0351680},
{-0.0234553,-0.0576924,0.0715330},
{-0.0234553,-0.0576924,-0.0715330},
{-0.0472319,-0.0833276,0.0513790},
{-0.0472319,-0.0833276,-0.0513790},
{-0.0327175,-0.0506481,0.0520240},
{-0.0327175,-0.0506481,-0.0520240},
{-0.0746024,-0.0627219,0.0928110},
{-0.0746024,-0.0627219,-0.0928110},
{-0.0119676,-0.0928080,0.0076900},
{-0.0119676,-0.0928080,-0.0076900},
{-0.0263299,-0.0760728,0.0667080},
{-0.0263299,-0.0760728,-0.0667080},
{-0.0498842,0.0119450,0.1070630},
{-0.0498842,0.0119450,-0.1070630},
{-0.0553874,0.0627474,0.1172390},
{-0.0553874,0.0627474,-0.1172390},
{-0.0786774,0.0073556,0.0741800},
{-0.0786774,0.0073556,-0.0741800},
{-0.0742762,0.0474272,0.0542330},

Biology Open • Supplementary information

{-0.0742762,0.0474272,-0.0542330},
{-0.0634121,0.0117185,0.0618430},
{-0.0634121,0.0117185,-0.0618430},
{-0.0813084,0.0007821,0.0508130},
{-0.0813084,0.0007821,-0.0508130},
{-0.0496423,-0.0444458,0.0983920},
{-0.0496423,-0.0444458,-0.0983920},
{-0.0480005,-0.0338999,0.0625080},
{-0.0480005,-0.0338999,-0.0625080},
{-0.0428000,-0.0273188,0.0973490},
{-0.0428000,-0.0273188,-0.0973490},
{-0.0548383,-0.0518766,0.0624050},
{-0.0548383,-0.0518766,-0.0624050},
{-0.0696448,-0.1052827,0.0436200},
{-0.0696448,-0.1052827,-0.0436200},
{-0.0739608,-0.0803177,0.0785950},
{-0.0739608,-0.0803177,-0.0785950},
{-0.0586066,-0.0884764,0.0653190},
{-0.0586066,-0.0884764,-0.0653190},
{-0.1017003,0.0085215,0.0624150},
{-0.1017003,0.0085215,-0.0624150},
{-0.0126252,0.0252852,0.1292530},
{-0.0126252,0.0252852,-0.1292530},
{-0.0030963,0.0150156,0.1262640},
{-0.0030963,0.0150156,-0.1262640},
{-0.0621797,0.0048743,0.0605920},
{-0.0621797,0.0048743,-0.0605920},
{-0.0870082,-0.0684280,0.0489900},
{-0.0870082,-0.0684280,-0.0489900},
{-0.0916211,-0.0478344,0.0575290},
{-0.0916211,-0.0478344,-0.0575290},
{-0.0428573,-0.0467026,0.0985250},
{-0.0428573,-0.0467026,-0.0985250},
{-0.0483869,-0.0455474,0.0848580},
{-0.0483869,-0.0455474,-0.0848580},
{-0.0203929,-0.0681811,0.0426070},
{-0.0203929,-0.0681811,-0.0426070},
{-0.0271973,-0.0807786,0.0491650},
{-0.0271973,-0.0807786,-0.0491650},
{-0.0325935,-0.1026813,0.0148280},
{-0.0325935,-0.1026813,-0.0148280},
{-0.0273900,-0.1059173,0.0048750},
{-0.0273900,-0.1059173,-0.0048750},
{-0.0015971,-0.0778770,0.0065530},
{-0.0015971,-0.0778770,-0.0065530},
{-0.1034983,-0.0140026,0.0521800},

```
        {-0.1034983,-0.0140026,-0.0521800},
        {-0.1141160,0.0077734,0.0497600},
        {-0.1141160,0.0077734,-0.0497600},
        {-0.1123797,0.0244808,0.0350160},
        {-0.1123797,0.0244808,-0.0350160},
        {-0.0984470,-0.0648210,0.0457100},
        {-0.0984470,-0.0648210,-0.0457100},
        {-0.0814332,-0.0704479,0.0806410},
        {-0.0814332,-0.0704479,-0.0806410},
        {-0.0540218,-0.0805070,0.0547320},
        {-0.0540218,-0.0805070,-0.0547320},
        {-0.0674356,-0.0763171,0.0651080},
        {-0.0674356,-0.0763171,-0.0651080},
        {-0.0602364,-0.0751827,0.0490600},
        {-0.0602364,-0.0751827,-0.0490600},
        {-0.0604272,-0.0801096,0.0617610},
        {-0.0604272,-0.0801096,-0.0617610},
        {-0.0655251,0.0632155,0.1093750},
        {-0.0655251,0.0632155,-0.1093750},
        {-0.0665046,0.0014289,0.0572170},
        {-0.0665046,0.0014289,-0.0572170},
        {-0.1053786,0.0347803,0.0071580},
        {-0.1053786,0.0347803,-0.0071580},
        {-0.1321649,-0.0069725,0.0058340},
        {-0.1321649,-0.0069725,-0.0058340},
        {-0.1232735,-0.0434903,0.0301110},
        {-0.1232735,-0.0434903,-0.0301110},
        {-0.0497024,0.0261862,0.0084640},
        {-0.0497024,0.0261862,-0.0084640},
        {-0.0887827,0.0446048,0.0327030},
        {-0.0887827,0.0446048,-0.0327030},
        {-0.1153998,-0.0126010,0.0290730},
        {-0.1153998,-0.0126010,-0.0290730},
        {-0.0899939,0.0093882,0.0208440},
        {-0.0899939,0.0093882,-0.0208440},
        {-0.1312654,-0.0574898,0.0094690},
        {-0.1312654,-0.0574898,-0.0094690},
        {-0.0996029,0.0042806,0.0105620},
        {-0.0996029,0.0042806,-0.0105620},
        {-0.1226177,-0.0324069,0.0331630},
        {-0.1226177,-0.0324069,-0.0331630},
        {-0.1097808,-0.0051619,0.0102520},
        {-0.1097808,-0.0051619,-0.0102520}
        };
Points1 = {
        {0.0000000,0.0000000,0.1189982},
```

{-0.0000007,-0.0873505,0.0173277},
{-0.0298398,-0.0678300,0.0828097},
{0.0000000,0.0000000,-0.1189982},
{-0.0000007,-0.0873505,-0.0173277},
{-0.0298398,-0.0678300,-0.0828097},
{0.0000000,0.0000000,0.0000000},
{-0.0814765,-0.0285255,0.0403088},
{-0.0814765,-0.0285255,-0.0403088},
{-0.0772270,-0.0959328,0.0588535},
{-0.0772270,-0.0959328,-0.0588535},
{-0.0530190,0.0401187,0.0867894},
{-0.0530190,0.0401187,-0.0867894},
{-0.0860153,-0.0071465,0.0357378},
{-0.0860153,-0.0071465,-0.0357378},
{-0.0780814,0.0150271,0.0474515},
{-0.0780814,0.0150271,-0.0474515},
{-0.0327316,0.0307932,0.1232205},
{-0.0327316,0.0307932,-0.1232205},
{-0.0213306,0.0311041,0.1272656},
{-0.0213306,0.0311041,-0.1272656},
{-0.0576636,-0.0387048,0.0653369},
{-0.0576636,-0.0387048,-0.0653369},
{-0.0720452,-0.0707535,0.0604398},
{-0.0720452,-0.0707535,-0.0604398},
{-0.0107364,-0.0379259,0.0893397},
{-0.0107364,-0.0379259,-0.0893397},
{-0.0498997,-0.1208119,0.0324805},
{-0.0498997,-0.1208119,-0.0324805},
{-0.0675036,-0.1119944,0.0618936},
{-0.0675036,-0.1119944,-0.0618936},
{-0.0280740,-0.1157768,0.0181211},
{-0.0280740,-0.1157768,-0.0181211},
{-0.0448679,-0.0275844,0.0631313},
{-0.0448679,-0.0275844,-0.0631313},
{-0.0142652,-0.0796456,0.0327308},
{-0.0142652,-0.0796456,-0.0327308},
{-0.0150120,-0.0566132,0.0701727},
{-0.0150120,-0.0566132,-0.0701727},
{-0.0278712,-0.0770952,0.0538414},
{-0.0278712,-0.0770952,-0.0538414},
{-0.0233316,-0.0568324,0.0546097},
{-0.0233316,-0.0568324,-0.0546097},
{-0.0473097,-0.0636218,0.0870971},
{-0.0473097,-0.0636218,-0.0870971},
{-0.0068250,-0.0969066,0.0074397},
{-0.0068250,-0.0969066,-0.0074397},

Biology Open • Supplementary information

{-0.0159519,-0.0682167,0.0639308},
{-0.0159519,-0.0682167,-0.0639308},
{-0.0345551,-0.0009581,0.1013494},
{-0.0345551,-0.0009581,-0.1013494},
{-0.0368819,0.0382944,0.1124203},
{-0.0368819,0.0382944,-0.1124203},
{-0.0585860,-0.0137537,0.0662153},
{-0.0585860,-0.0137537,-0.0662153},
{-0.0561066,0.0135105,0.0477173},
{-0.0561066,0.0135105,-0.0477173},
{-0.0509435,-0.0098746,0.0536895},
{-0.0509435,-0.0098746,-0.0536895},
{-0.0613261,-0.0222628,0.0445768},
{-0.0613261,-0.0222628,-0.0445768},
{-0.0297013,-0.0487381,0.0900992},
{-0.0297013,-0.0487381,-0.0900992},
{-0.0342222,-0.0419501,0.0643885},
{-0.0342222,-0.0419501,-0.0643885},
{-0.0276311,-0.0336881,0.0904517},
{-0.0276311,-0.0336881,-0.0904517},
{-0.0353558,-0.0563978,0.0659757},
{-0.0353558,-0.0563978,-0.0659757},
{-0.0478002,-0.0989660,0.0505783},
{-0.0478002,-0.0989660,-0.0505783},
{-0.0465573,-0.0777861,0.0792240},
{-0.0465573,-0.0777861,-0.0792240},
{-0.0354984,-0.0846238,0.0706294},
{-0.0354984,-0.0846238,-0.0706294},
{-0.0725937,-0.0166464,0.0549058},
{-0.0725937,-0.0166464,-0.0549058},
{-0.0071029,0.0183320,0.1275899},
{-0.0071029,0.0183320,-0.1275899},
{-0.0012763,0.0115586,0.1260201},
{-0.0012763,0.0115586,-0.1260201},
{-0.0506930,-0.0148107,0.0523739},
{-0.0506930,-0.0148107,-0.0523739},
{-0.0606032,-0.0733309,0.0600376},
{-0.0606032,-0.0733309,-0.0600376},
{-0.0643087,-0.0586653,0.0627759},
{-0.0643087,-0.0586653,-0.0627759},
{-0.0245096,-0.0505513,0.0896640},
{-0.0245096,-0.0505513,-0.0896640},
{-0.0300086,-0.0498253,0.0805805},
{-0.0300086,-0.0498253,-0.0805805},
{-0.0126637,-0.0705281,0.0396563},
{-0.0126637,-0.0705281,-0.0396563},

Biology Open • Supplementary information

{-0.0161285,-0.0769605,0.0469870},
{-0.0161285,-0.0769605,-0.0469870},
{-0.0201839,-0.1023783,0.0165095},
{-0.0201839,-0.1023783,-0.0165095},
{-0.0149827,-0.1056040,0.0064494},
{-0.0149827,-0.1056040,-0.0064494},
{-0.0017344,-0.0861147,0.0059849},
{-0.0017344,-0.0861147,-0.0059849},
{-0.0728681,-0.0341316,0.0500943},
{-0.0728681,-0.0341316,-0.0500943},
{-0.0802183,-0.0188173,0.0424686},
{-0.0802183,-0.0188173,-0.0424686},
{-0.0787555,-0.0113047,0.0273049},
{-0.0787555,-0.0113047,-0.0273049},
{-0.0710402,-0.0725779,0.0591641},
{-0.0710402,-0.0725779,-0.0591641},
{-0.0527360,-0.0710810,0.0806331},
{-0.0527360,-0.0710810,-0.0806331},
{-0.0315583,-0.0741479,0.0584669},
{-0.0315583,-0.0741479,-0.0584669},
{-0.0419413,-0.0758231,0.0705194},
{-0.0419413,-0.0758231,-0.0705194},
{-0.0375111,-0.0738295,0.0551093},
{-0.0375111,-0.0738295,-0.0551093},
{-0.0363379,-0.0784351,0.0680255},
{-0.0363379,-0.0784351,-0.0680255},
{-0.0456795,0.0350386,0.1044333},
{-0.0456795,0.0350386,-0.1044333},
{-0.0539046,-0.0185329,0.0486502},
{-0.0539046,-0.0185329,-0.0486502},
{-0.0745547,-0.0058593,0.0054873},
{-0.0745547,-0.0058593,-0.0054873},
{-0.0911188,-0.0369901,0.0043664},
{-0.0911188,-0.0369901,-0.0043664},
{-0.0902180,-0.0640398,0.0204981},
{-0.0902180,-0.0640398,-0.0204981},
{-0.0406700,-0.0066948,0.0074582},
{-0.0406700,-0.0066948,-0.0074582},
{-0.0652318,0.0060112,0.0270244},
{-0.0652318,0.0060112,-0.0270244},
{-0.0808123,-0.0385043,0.0247068},
{-0.0808123,-0.0385043,-0.0247068},
{-0.0651587,-0.0222665,0.0174891},
{-0.0651587,-0.0222665,-0.0174891},
{-0.0961659,-0.0749316,0.0064065},
{-0.0961659,-0.0749316,-0.0064065},

Biology Open • Supplementary information

```
                    {-0.0693849,-0.0285922,0.0087757},
                    {-0.0693849,-0.0285922,-0.0087757},
                    {-0.0881225,-0.0544929,0.0260964},
                    {-0.0881225,-0.0544929,-0.0260964},
                    {-0.0749345,-0.0362792,0.0084086},
                    {-0.0749345,-0.0362792,-0.0084086}
                    };
            BoundingBoxOnOff = Off;
        };
        AnyFunTransform3DIdentity ScaleFunction = {
            PreTransforms = {&.RBFTransform};
};
};

};
```

Biology Open • Supplementary information

ScalingFunctionTLEMLucyFemur_2014048

```
AnyFolder ScalingFunctionTLEMLucyFemur = {
  AnyFolder Right = {
        AnyFolder Thigh = {
        AnyFunTransform3DRBF RBFTransform = {
                RBFDef.Type = RBF_ThinPlate;
                PolynomDegree = 1;
                Points0 = {
                        {0.0000000,0.0000000,0.0000000},
                        {-0.0000000,-0.3616821,0.0000000},
                        {-0.0097563,-0.3678799,0.0012967},
                        {-0.0000000,-0.3660632,0.0408203},
                        {-0.0000000,-0.3573010,-0.0408203},
                        {0.0161460,-0.0072838,0.0601290},
                        {0.0220217,-0.0203698,0.0463848},
                        {0.0123977,-0.0241932,0.0668573},
                        {0.0006898,0.0018121,0.0538181},
                        {0.0122809,-0.0068668,0.0414535},
                        {-0.0058991,-0.0138188,0.0648412},
                        {0.0211469,-0.0380855,0.0538111},
                        {0.0172133,-0.0317342,0.0328381},
                        {0.0177311,-0.0131946,0.0256176},
                        {-0.0110079,-0.0308867,0.0632370},
                        {0.0039435,-0.0395977,0.0638271},
                        {-0.0041683,-0.0187472,0.0413214},
                        {-0.0177999,-0.0021535,0.0496084},
                        {0.0001603,-0.0053126,0.0279593},
                        {-0.0211600,-0.0216243,0.0497147},
                        {0.0096163,-0.0568663,0.0545719},
                        {0.0162112,-0.0515013,0.0395230},
                        {0.0093533,-0.0433265,0.0187387},
                        {0.0119398,-0.0238469,0.0118403},
                        {0.0210707,-0.0048848,0.0065878},
                        {0.0125013,0.0064094,0.0177702},
                        {-0.0191523,-0.0356053,0.0382429},
                        {-0.0115821,-0.0474937,0.0537648},
                        {-0.0100073,-0.0236371,0.0267322},
                        {-0.0078925,0.0107098,0.0182833},
                        {-0.0134217,-0.0055356,0.0182000},
                        {-0.0090061,-0.0665700,0.0475484},
                        {0.0071095,-0.0761976,0.0520035},
                        {0.0182119,-0.0704149,0.0386546},
                        {0.0073764,-0.0609481,0.0221006},
                        {-0.0122984,-0.0439585,0.0198428},
                        {-0.0032253,-0.0306792,0.0110995},
```

Biology Open • Supplementary information

{-0.0012975,-0.0198451,-0.0052592},
{0.0140405,-0.0161125,-0.0071649},
{0.0189222,-0.0014898,-0.0127226},
{0.0181642,0.0132812,-0.0011050},
{0.0045119,0.0200001,0.0091189},
{-0.0176761,-0.0541983,0.0348587},
{-0.0136548,-0.0142015,0.0054311},
{-0.0135510,0.0182940,0.0017175},
{-0.0223865,0.0023399,0.0028018},
{-0.0086455,-0.0738247,0.0283843},
{-0.0050356,-0.0877749,0.0442195},
{0.0160135,-0.0919319,0.0465120},
{0.0128290,-0.0785810,0.0232431},
{-0.0168328,-0.0616327,0.0155917},
{-0.0172369,-0.0094567,-0.0119502},
{0.0002090,-0.0093641,-0.0209779},
{0.0064827,0.0081538,-0.0198453},
{0.0010777,0.0210422,-0.0084721},
{-0.0129485,0.0085451,-0.0162212},
{-0.0002322,-0.0924033,0.0245644},
{-0.0028175,-0.1052263,0.0418294},
{0.0172322,-0.1111444,0.0445760},
{0.0203259,-0.0957409,0.0271449},
{0.0057102,-0.1088933,0.0195113},
{-0.0010852,-0.1230776,0.0363132},
{0.0182727,-0.1303312,0.0422884},
{0.0242891,-0.1161703,0.0265054},
{0.0094531,-0.1268584,0.0162294},
{0.0014244,-0.1413293,0.0331653},
{0.0207271,-0.1496427,0.0394157},
{0.0267562,-0.1360142,0.0236798},
{0.0113839,-0.1461651,0.0136024},
{0.0030845,-0.1595094,0.0297156},
{0.0209528,-0.1691386,0.0369775},
{0.0283958,-0.1540209,0.0213795},
{0.0129499,-0.1649012,0.0113589},
{0.0016490,-0.1783108,0.0216287},
{0.0147105,-0.1880678,0.0328614},
{0.0298491,-0.1859865,0.0248551},
{0.0286645,-0.1714377,0.0169488},
{0.0165897,-0.1861006,0.0082595},
{0.0014000,-0.1973476,0.0183740},
{0.0114301,-0.2109792,0.0286157},
{0.0267422,-0.2037622,0.0296951},
{0.0288630,-0.2018335,0.0130127},
{0.0145471,-0.2083505,0.0062833},

Biology Open • Supplementary information

{0.0011300,-0.2198071,0.0155602},
{0.0126610,-0.2321235,0.0275354},
{0.0275647,-0.2219268,0.0265185},
{0.0286645,-0.2230253,0.0098701},
{0.0130434,-0.2304822,0.0039177},
{0.0022984,-0.2409585,0.0149650},
{0.0101315,-0.2534740,0.0252125},
{0.0279485,-0.2428555,0.0234276},
{0.0276684,-0.2421559,0.0058691},
{0.0101250,-0.2529527,0.0018983},
{0.0021376,-0.2667831,0.0107073},
{0.0114309,-0.2734905,0.0249960},
{0.0273923,-0.2634953,0.0216324},
{0.0278429,-0.2592546,0.0037692},
{0.0159101,-0.2712985,-0.0033988},
{0.0036708,-0.2863280,0.0004644},
{0.0023115,-0.2884756,0.0187554},
{0.0231385,-0.2879051,0.0224853},
{0.0304009,-0.2799973,0.0083549},
{0.0238189,-0.2917246,-0.0052462},
{0.0064904,-0.3027687,-0.0070587},
{-0.0003492,-0.3064690,0.0093014},
{0.0122179,-0.3045269,0.0243511},
{0.0301837,-0.3030127,0.0114462},
{0.0234182,-0.3107336,-0.0085198},
{0.0046893,-0.3214995,-0.0123881},
{-0.0022352,-0.3264905,0.0043340},
{0.0016841,-0.3202756,0.0217839},
{0.0229722,-0.3196887,0.0215083},
{0.0292438,-0.3228042,0.0051430},
{0.0221641,-0.3296190,-0.0132361},
{0.0100459,-0.3417597,-0.0244560},
{-0.0053009,-0.3375737,-0.0137705},
{-0.0064432,-0.3487307,0.0028676},
{-0.0059346,-0.3407801,0.0202938},
{0.0105130,-0.3365976,0.0264175},
{0.0282695,-0.3386249,0.0180307},
{0.0270402,-0.3431844,-0.0001487},
{0.0257040,-0.3506950,-0.0181851},
{0.0178948,-0.3636114,-0.0302838},
{0.0043149,-0.3544109,-0.0381949},
{-0.0095210,-0.3461482,-0.0295031},
{-0.0164526,-0.3542895,-0.0126666},
{-0.0060896,-0.3690127,-0.0033061},
{-0.0176523,-0.3612686,0.0110109},
{-0.0153310,-0.3546943,0.0299634},

{0.0021329,-0.3508021,0.0350763},
{0.0210690,-0.3548628,0.0279835},
{0.0356594,-0.3558162,0.0159356},
{0.0275718,-0.3617450,-0.0018607},
{0.0298503,-0.3741530,-0.0163721},
{0.0175320,-0.3855300,-0.0277515},
{0.0025604,-0.3740099,-0.0397137},
{-0.0136727,-0.3624016,-0.0402102},
{-0.0277647,-0.3567472,-0.0284947},
{-0.0275446,-0.3699630,-0.0120829},
{-0.0175846,-0.3859355,-0.0177922},
{0.0001572,-0.3856873,-0.0118050},
{0.0030262,-0.3834856,0.0075553},
{-0.0144114,-0.3810887,0.0140304},
{-0.0247706,-0.3701239,0.0285134},
{-0.0044804,-0.3677201,0.0404632},
{0.0133183,-0.3712274,0.0334208},
{0.0324861,-0.3723417,0.0245668},
{0.0271451,-0.3766027,0.0072441},
{0.0167985,-0.3822816,-0.0065349},
{-0.0001454,-0.3910823,-0.0303010},
{-0.0144998,-0.3834154,-0.0384137},
{-0.0301321,-0.3750841,-0.0321336},
{0.0196491,-0.3864804,0.0210853},
{0.0015726,-0.3888034,0.0276569},
{-0.0146652,-0.3847848,0.0336913},
{-0.0014588,-0.0619951,0.0219778},
{-0.0118435,-0.0550626,0.0139639},
{0.0129812,-0.0349966,0.0673128},
{-0.0162907,-0.0105666,0.0597739},
{-0.0012885,-0.0520816,0.0562989},
{-0.0090599,-0.0631298,0.0172224},
{-0.0000222,-0.0771663,0.0231897},
{-0.0068148,0.0023885,-0.0160086},
{-0.0180544,-0.0425409,0.0446585},
{0.0020525,-0.0249904,0.0691549},
{-0.0157097,-0.0662315,0.0238393},
{-0.0010518,-0.0510686,0.0199353},
{-0.0208874,-0.0152865,0.0500034},
{0.0168316,-0.0280857,0.0666680},
{-0.0187392,-0.0282333,0.0403282},
{0.0191622,-0.0351633,0.0622707},
{0.0001216,-0.0706289,0.0524049},
{-0.0169862,-0.0399062,0.0311827},
{-0.0185737,-0.0533066,0.0151224},
{-0.0219058,-0.0528723,0.0222263},

Biology Open • Supplementary information

{-0.0028271,-0.0391620,0.0148452},
{0.0117900,-0.0553425,0.0235348},
{0.0219835,-0.0106400,0.0396338},
{-0.0025366,-0.0401046,0.0625273},
{0.0141017,-0.0018767,0.0198108},
{-0.0078770,-0.0473929,0.0173542},
{-0.0065351,-0.0766431,0.0470258},
{0.0065585,0.0145593,0.0161638},
{-0.0118588,0.0172875,-0.0085064},
{-0.0074087,-0.0627500,0.0506700},
{0.0071940,-0.0780068,0.0213362},
{0.0126736,-0.0708176,0.0244108},
{0.0074694,-0.0436450,0.0604196},
{0.0154186,-0.0139471,0.0064312},
{-0.0053535,-0.0213210,0.0040094},
{-0.0088733,0.0002018,0.0205402},
{-0.0112471,-0.0542608,0.0500266},
{-0.0061625,-0.0248673,0.0653244},
{0.0093695,-0.0065826,0.0614157},
{0.0138015,-0.0467322,0.0284333},
{-0.0020999,-0.0186885,-0.0116392},
{-0.0185640,-0.0225591,0.0566779},
{0.0175798,0.0124360,-0.0068467},
{-0.0039962,0.0217127,0.0053055},
{0.0014975,-0.0784591,0.0514056},
{-0.0172997,-0.0386971,0.0525252},
{0.0196540,-0.0268271,0.0416806},
{0.0135296,-0.0406504,0.0236882},
{0.0182471,0.0095959,0.0087906},
{0.0218667,0.0016804,0.0058793},
{0.0224975,-0.0022652,-0.0022994},
{0.0166750,-0.0191391,0.0236436},
{0.0119785,-0.0328723,0.0195376},
{0.0187631,-0.0133469,0.0308741},
{-0.0187543,-0.0461463,0.0257491},
{0.0039834,-0.0373928,0.0683089},
{0.0157029,-0.0423169,0.0385226},
{0.0164003,-0.0087069,0.0131181},
{0.0008253,0.0033370,-0.0212576},
{0.0101707,-0.0009724,-0.0205688},
{0.0102948,-0.0642683,0.0524532},
{0.0152217,-0.0557944,0.0508978},
{0.0172973,-0.0565707,0.0419468},
{0.0167723,-0.0635334,0.0369834},
{-0.0047397,-0.0695325,0.0241398},
{-0.0152913,0.0035778,0.0161461},

{0.0091697,0.0009340,0.0575160},
{0.0196686,-0.0089533,0.0500006},
{0.0183998,-0.0212943,0.0622968},
{-0.0114340,-0.0316810,0.0250940},
{-0.0206442,-0.0092599,-0.0028425},
{0.0166868,-0.0784295,0.0276925},
{-0.0069678,-0.0054521,-0.0207011},
{0.0049490,0.0151109,-0.0160093},
{-0.0084025,-0.0318937,0.0162788},
{-0.0173679,0.0095594,-0.0108509},
{-0.0218912,0.0063457,-0.0020698},
{-0.0106547,-0.0668750,0.0397749},
{0.0136098,-0.0170666,-0.0018890},
{0.0141599,-0.0441405,0.0587594},
{0.0093255,-0.0183218,-0.0083008},
{0.0106367,-0.0128276,-0.0154269},
{0.0037350,-0.0549365,0.0209786},
{0.0087662,-0.0472902,0.0195496},
{0.0016839,-0.0708213,0.0221325},
{-0.0202977,-0.0530540,0.0301270},
{-0.0068584,0.0021781,0.0530002},
{-0.0144817,-0.0297218,0.0605833},
{0.0197015,-0.0165217,0.0560654},
{0.0156609,-0.0705363,0.0477736},
{0.0207876,-0.0221433,0.0330733},
{0.0092324,-0.0300767,0.0713160},
{-0.0203392,-0.0034488,0.0097332},
{0.0156978,-0.0143438,0.0629053},
{0.0143267,-0.0563519,0.0317489},
{-0.0161685,-0.0465391,0.0188591},
{-0.0196397,-0.0309875,0.0546496},
{0.0154804,-0.0380422,0.0305433},
{-0.0077774,-0.0351303,0.0637765},
{0.0063980,-0.0086419,-0.0201654},
{0.0030146,-0.0151094,-0.0165974},
{-0.0210080,-0.0096030,0.0507855},
{-0.0105684,-0.0411377,0.0586146},
{0.0180619,-0.0498960,0.0469835},
{-0.0192318,0.0116108,0.0024070},
{-0.0165651,0.0083622,0.0126870},
{-0.0014333,-0.0627138,0.0534746},
{0.0145127,-0.0080178,0.0353667},
{0.0148374,-0.0012228,0.0566181},
{0.0153834,-0.0062427,0.0444727},
{-0.0155164,-0.0132103,-0.0096398},
{0.0068685,-0.0221022,0.0011547},

Biology Open • Supplementary information

{0.0003332,-0.0458500,0.0183716},
{0.0205582,0.0040956,-0.0082969},
{0.0175752,0.0009340,-0.0148407},
{0.0142357,-0.0063623,-0.0169332},
{-0.0147285,0.0023412,-0.0164678},
{-0.0158781,-0.0064451,-0.0151346},
{0.0175951,0.0133456,0.0037559},
{0.0117660,0.0189899,-0.0030690},
{0.0096040,0.0151827,-0.0137450},
{0.0096565,-0.0215194,0.0662734},
{-0.0006793,-0.0074494,0.0631218},
{0.0007819,-0.0004519,0.0600781},
{0.0065377,-0.0125433,0.0654199},
{0.0090461,0.0073096,0.0188711},
{0.0151418,-0.0168302,0.0146465},
{0.0025166,-0.0231863,0.0688894},
{-0.0097891,-0.0152077,0.0253086},
{-0.0109149,-0.0177935,0.0119825},
{-0.0072079,-0.0099373,0.0272725},
{-0.0183727,-0.0467429,0.0356276},
{-0.0126871,-0.0584555,0.0428141},
{-0.0057054,-0.0273042,0.0103554},
{0.0215142,-0.0159693,0.0474531},
{0.0128492,-0.0070016,0.0287645},
{0.0052134,-0.0017047,0.0247463},
{-0.0024284,-0.0707498,0.0513049},
{-0.0036140,0.0220593,-0.0040484},
{-0.0002405,-0.0121253,0.0390050},
{-0.0119811,-0.0156737,0.0015445},
{0.0142082,-0.0279792,0.0246553},
{0.0122244,-0.0262013,0.0169829},
{-0.0146264,-0.0038855,0.0549384},
{0.0207450,-0.0325771,0.0496412},
{-0.0155040,-0.0497560,0.0437087},
{-0.0081119,-0.0756450,0.0387365},
{0.0039684,-0.0062047,0.0313900},
{-0.0023726,-0.0038892,-0.0220521},
{-0.0136977,-0.0339839,0.0602619},
{-0.0035399,0.0089081,-0.0188894},
{-0.0049820,0.0176571,-0.0132254},
{0.0193902,-0.0803191,0.0360429},
{0.0040648,-0.0697109,0.0218205},
{0.0170470,-0.0818610,0.0456892},
{0.0104175,0.0188635,0.0064965},
{0.0135206,0.0092549,0.0158079},
{0.0023933,0.0160498,-0.0157404},

Biology Open • Supplementary information

{0.0164753,-0.0118197,-0.0096995},
{0.0202584,-0.0066858,-0.0082383},
{-0.0098401,-0.0226001,0.0314028},
{-0.0130937,-0.0309622,0.0320764},
{-0.0201510,-0.0613297,0.0222264},
{-0.0010389,0.0036055,0.0209003},
{0.0042025,0.0073392,0.0199393},
{-0.0021630,-0.0038531,0.0251898},
{-0.0007845,-0.0293574,0.0675788},
{0.0070027,-0.0406327,0.0624565},
{-0.0124363,-0.0183001,0.0627966},
{-0.0120485,-0.0256613,0.0628493},
{0.0101200,0.0197695,0.0039334},
{0.0167636,0.0148266,0.0014155},
{-0.0074981,-0.0106930,0.0632797},
{0.0142533,-0.0503445,0.0351954},
{-0.0146107,-0.0460953,0.0506120},
{-0.0161117,-0.0471301,0.0178900},
{0.0112149,-0.0792793,0.0501460},
{-0.0099060,-0.0230794,0.0186951},
{-0.0114543,-0.0153049,0.0183675},
{-0.0042882,0.0161879,0.0151423},
{0.0014582,-0.0638572,0.0541080},
{0.0048554,-0.0582603,0.0553815},
{-0.0017077,-0.0572302,0.0546635},
{0.0166090,-0.0697169,0.0323038},
{-0.0208812,-0.0164273,0.0474354},
{0.0130454,-0.0241864,0.0178294},
{0.0053123,-0.0483770,0.0582251},
{0.0112225,-0.0361429,0.0673300},
{0.0085729,-0.0771256,0.0216100},
{-0.0070783,-0.0488033,0.0560869},
{0.0182083,-0.0411024,0.0454236},
{-0.0119590,-0.0403809,0.0213546},
{0.0233409,-0.0185535,0.0366343},
{-0.0046347,-0.0571211,0.0208243},
{0.0183166,-0.0006966,0.0140116},
{0.0061913,0.0053512,0.0201003},
{-0.0002364,-0.0310828,0.0680351},
{-0.0170773,0.0139270,-0.0054987},
{0.0110805,0.0156034,0.0117010},
{-0.0124628,0.0164667,0.0094550},
{-0.0209014,-0.0073407,-0.0051969},
{-0.0210131,0.0010693,-0.0086891},
{-0.0041596,-0.0172332,0.0663041},
{-0.0216595,-0.0322172,0.0451540},

{0.0051908,-0.0426014,0.0613013},
{0.0150003,-0.0416136,0.0606257},
{-0.0135090,-0.0674234,0.0236425},
{0.0146416,0.0073691,0.0160052},
{0.0195615,-0.0112412,-0.0010333},
{-0.0198115,-0.0098244,0.0044735},
{-0.0157054,-0.0521236,0.0395039},
{-0.0081381,-0.0173108,-0.0046152},
{-0.0218324,-0.0262661,0.0471115},
{0.0051446,-0.0707257,0.0530555},
{0.0179359,-0.0471493,0.0528134},
{-0.0005591,-0.0317983,0.0113632},
{0.0071707,-0.0305943,0.0121186},
{0.0206239,-0.0061367,0.0066642},
{0.0187057,-0.0745868,0.0420207},
{-0.0095784,-0.0163113,0.0259524},
{-0.0012181,0.0127661,0.0186855},
{0.0059420,-0.0630691,0.0220087},
{0.0069408,-0.0573878,0.0552614},
{0.0150782,-0.0132884,-0.0101793},
{0.0005533,-0.0078574,0.0633775},
{-0.0088284,-0.0146399,-0.0147837},
{0.0167444,-0.0651105,0.0470347},
{0.0082666,-0.0181741,-0.0095133},
{-0.0175162,-0.0637444,0.0177069},
{0.0153795,-0.0360767,0.0287608},
{0.0162363,-0.0018864,0.0548412},
{0.0052731,-0.0075919,0.0392990},
{-0.0022288,-0.0556644,0.0549909},
{-0.0156238,-0.0550038,0.0377688},
{-0.0204137,-0.0100550,0.0536429},
{-0.0093683,0.0122429,-0.0162013},
{-0.0202927,0.0049166,0.0083887},
{0.0074706,-0.0627945,0.0222133},
{0.0035034,-0.0376103,0.0672421},
{-0.0190752,-0.0194129,0.0555530},
{0.0061758,-0.0074961,-0.0207741},
{0.0192011,-0.0273063,0.0588233},
{0.0130796,-0.0134383,0.0638611},
{0.0120421,0.0019566,-0.0194140},
{0.0154152,-0.0065294,0.0179581},
{-0.0157335,0.0101219,0.0123981},
{0.0110376,-0.0488274,0.0567911},
{-0.0055170,-0.0343676,0.0647140},
{0.0216204,-0.0273988,0.0512041},
{0.0161331,-0.0135875,-0.0075501},

Biology Open • Supplementary information

{-0.0184643,-0.0455633,0.0360087},
{-0.0091978,-0.0682413,0.0460847},
{0.0034304,-0.0471677,0.0588866},
{-0.0048025,-0.0384316,0.0149400},
{0.0118505,0.0109735,-0.0159161},
{-0.0027036,-0.0204189,-0.0015094},
{-0.0117607,-0.0692759,0.0292171},
{-0.0107080,-0.0033455,-0.0190487},
{-0.0042548,0.0214947,-0.0056846},
{-0.0097315,-0.0208529,0.0131997},
{-0.0178958,-0.0627218,0.0171551},
{-0.0068692,-0.0688818,0.0233316},
{0.0012083,0.0120695,-0.0186179},
{0.0215073,-0.0236027,0.0503069},
{-0.0060262,-0.0534931,0.0189523},
{0.0179951,-0.0336785,0.0397267},
{0.0190509,-0.0284972,0.0336394},
{-0.0169188,-0.0133609,-0.0014203},
{0.0135722,0.0136491,0.0114695},
{-0.0150549,-0.0123230,0.0067722},
{-0.0148411,-0.0311945,0.0355412},
{0.0185490,-0.0794042,0.0431266},
{0.0193568,-0.0087326,0.0575704},
{0.0201026,-0.0144353,0.0531586},
{0.0025583,-0.0248211,0.0045714},
{0.0132689,-0.0214866,0.0652899},
{-0.0028669,-0.0099311,0.0309271},
{-0.0219487,-0.0054893,-0.0021853},
{0.0026180,-0.0203806,-0.0040620},
{-0.0116230,-0.0668518,0.0370152},
{-0.0133007,-0.0264159,0.0619176},
{0.0186167,0.0050491,-0.0120059},
{-0.0191359,-0.0591690,0.0288149},
{-0.0178121,-0.0041834,0.0141448},
{0.0123077,-0.0063648,0.0279743},
{-0.0157729,-0.0374010,0.0315335},
{0.0149328,-0.0659100,0.0290095},
{0.0006205,0.0021977,-0.0214613},
{0.0170484,-0.0715687,0.0325980},
{0.0068499,0.0061192,0.0196608},
{-0.0167075,0.0020024,0.0150382},
{0.0059801,-0.0393117,0.0167489},
{-0.0072506,-0.0171067,0.0338751},
{0.0202363,-0.0251596,0.0420997},
{-0.0199311,-0.0601255,0.0171522},
{0.0216641,0.0053410,-0.0027841},

{-0.0197665,-0.0606125,0.0252972},
{0.0129986,-0.0214528,0.0098002},
{0.0201151,-0.0100929,0.0460501},
{0.0104137,0.0017765,-0.0203073},
{0.0166557,-0.0064123,0.0140197},
{-0.0124120,-0.0596549,0.0422637},
{0.0014885,0.0202590,0.0096781},
{0.0028720,0.0020207,-0.0219044},
{0.0376095,-0.3675427,0.0214537},
{0.0049949,0.0206417,-0.0079017},
{-0.0191723,-0.0407590,0.0391310},
{-0.0316406,-0.3638215,-0.0274841},
{0.0021392,0.0226920,0.0000985},
{0.0168435,-0.0229382,0.0264788},
{-0.0157304,-0.0005437,-0.0161456},
{0.0203722,-0.0299750,0.0562762},
{-0.0171077,0.0134146,0.0059615},
{0.0175519,-0.0370653,0.0413374},
{0.0154657,-0.0405127,0.0348980},
{-0.0215172,-0.0323502,0.0481925},
{0.0177998,-0.0515776,0.0444709},
{-0.0183804,-0.0448316,0.0275139},
{0.0033607,-0.0502028,0.0575894},
{0.0010325,-0.0479377,0.0191141},
{0.0224852,-0.0181821,0.0451780},
{0.0084089,-0.0720538,0.0521031},
{-0.0070880,-0.0772912,0.0288023},
{-0.0223712,-0.0028990,0.0024047},
{0.0173136,-0.0120017,0.0204612},
{0.0137772,-0.0623537,0.0276621},
{-0.0100390,-0.0231180,0.0292222},
{-0.0196395,-0.3730207,0.0364730},
{-0.0094445,-0.0526688,0.0528111},
{-0.0115309,-0.3679776,-0.0104995},
{0.0129812,-0.0410477,0.0224072},
{0.0117394,0.0154849,-0.0117191},
{-0.0245208,-0.3649412,0.0295111},
{0.0147476,-0.0502966,0.0364899},
{0.0006132,-0.0446771,0.0600524},
{-0.0118123,0.0191139,0.0031977},
{-0.0017185,-0.0163303,0.0664216},
{-0.0085098,-0.0074564,0.0247745},
{0.0197459,-0.0086852,0.0430460},
{0.0187064,-0.0223532,0.0600966},
{-0.0206552,-0.0099712,0.0525024},
{0.0207525,0.0084646,-0.0024846},

Biology Open • Supplementary information

{-0.0094915,-0.0325446,0.0184441},
{-0.0014464,-0.0140050,-0.0179819},
{-0.0122725,-0.0573444,0.0463125},
{-0.0106397,0.0177211,0.0094669},
{0.0111981,0.0191236,-0.0041491},
{-0.0162790,-0.0256279,0.0592687},
{-0.0060120,-0.3653959,0.0413411},
{-0.0135007,-0.3703638,-0.0387476},
{-0.0134258,-0.3703142,-0.0379467},
{-0.0133509,-0.3702645,-0.0371458},
{-0.0132760,-0.3702148,-0.0363450},
{-0.0132011,-0.3701651,-0.0355441},
{-0.0131262,-0.3701154,-0.0347432},
{-0.0130513,-0.3700658,-0.0339423},
{-0.0129765,-0.3700161,-0.0331414},
{-0.0129016,-0.3699664,-0.0323405},
{-0.0128267,-0.3699167,-0.0315396},
{-0.0127518,-0.3698670,-0.0307388},
{-0.0126769,-0.3698174,-0.0299379},
{-0.0126020,-0.3697677,-0.0291370},
{-0.0125271,-0.3697180,-0.0283361},
{-0.0124523,-0.3696683,-0.0275352},
{-0.0123774,-0.3696187,-0.0267343},
{-0.0123025,-0.3695690,-0.0259334},
{-0.0122276,-0.3695193,-0.0251325},
{-0.0121527,-0.3694696,-0.0243317},
{-0.0120778,-0.3694199,-0.0235308},
{-0.0120029,-0.3693703,-0.0227299},
{-0.0119280,-0.3693206,-0.0219290},
{-0.0118532,-0.3692709,-0.0211281},
{-0.0117783,-0.3692212,-0.0203272},
{-0.0117034,-0.3691715,-0.0195263},
{-0.0116285,-0.3691219,-0.0187255},
{-0.0115536,-0.3690722,-0.0179246},
{-0.0114787,-0.3690225,-0.0171237},
{-0.0114038,-0.3689728,-0.0163228},
{-0.0113289,-0.3689231,-0.0155219},
{-0.0112541,-0.3688735,-0.0147210},
{-0.0111792,-0.3688238,-0.0139201},
{-0.0111043,-0.3687741,-0.0131192},
{-0.0110294,-0.3687244,-0.0123184},
{-0.0109545,-0.3686748,-0.0115175},
{-0.0108796,-0.3686251,-0.0107166},
{-0.0108047,-0.3685754,-0.0099157},
{-0.0107298,-0.3685257,-0.0091148},
{-0.0106550,-0.3684760,-0.0083139},

Biology Open • Supplementary information

{-0.0105801,-0.3684264,-0.0075130},
{-0.0105052,-0.3683767,-0.0067121},
{-0.0104303,-0.3683270,-0.0059113},
{-0.0103554,-0.3682773,-0.0051104},
{-0.0102805,-0.3682276,-0.0043095},
{-0.0102056,-0.3681780,-0.0035086},
{-0.0101307,-0.3681283,-0.0027077},
{-0.0100559,-0.3680786,-0.0019068},
{-0.0099810,-0.3680289,-0.0011059},
{-0.0099061,-0.3679792,-0.0003051},
{-0.0098312,-0.3679296,0.0004958},
{-0.0097563,-0.3678799,0.0012967},
{-0.0096814,-0.3678302,0.0020976},
{-0.0096065,-0.3677805,0.0028985},
{-0.0095317,-0.3677308,0.0036994},
{-0.0094568,-0.3676812,0.0045003},
{-0.0093819,-0.3676315,0.0053012},
{-0.0093070,-0.3675818,0.0061020},
{-0.0092321,-0.3675321,0.0069029},
{-0.0091572,-0.3674825,0.0077038},
{-0.0090823,-0.3674328,0.0085047},
{-0.0090074,-0.3673831,0.0093056},
{-0.0089326,-0.3673334,0.0101065},
{-0.0088577,-0.3672837,0.0109074},
{-0.0087828,-0.3672341,0.0117082},
{-0.0087079,-0.3671844,0.0125091},
{-0.0086330,-0.3671347,0.0133100},
{-0.0085581,-0.3670850,0.0141109},
{-0.0084832,-0.3670353,0.0149118},
{-0.0084083,-0.3669857,0.0157127},
{-0.0083335,-0.3669360,0.0165136},
{-0.0082586,-0.3668863,0.0173145},
{-0.0081837,-0.3668366,0.0181153},
{-0.0081088,-0.3667869,0.0189162},
{-0.0080339,-0.3667373,0.0197171},
{-0.0079590,-0.3666876,0.0205180},
{-0.0078841,-0.3666379,0.0213189},
{-0.0078092,-0.3665882,0.0221198},
{-0.0077344,-0.3665386,0.0229207},
{-0.0076595,-0.3664889,0.0237215},
{-0.0075846,-0.3664392,0.0245224},
{-0.0075097,-0.3663895,0.0253233},
{-0.0074348,-0.3663398,0.0261242},
{-0.0073599,-0.3662902,0.0269251},
{-0.0072850,-0.3662405,0.0277260},
{-0.0072101,-0.3661908,0.0285269},

{-0.0071353,-0.3661411,0.0293278},
{-0.0070604,-0.3660914,0.0301286},
{-0.0069855,-0.3660418,0.0309295},
{-0.0069106,-0.3659921,0.0317304},
{-0.0068357,-0.3659424,0.0325313},
{-0.0067608,-0.3658927,0.0333322},
{-0.0066859,-0.3658430,0.0341331},
{-0.0066111,-0.3657934,0.0349340},
{-0.0065362,-0.3657437,0.0357348},
{-0.0064613,-0.3656940,0.0365357},
{-0.0063864,-0.3656443,0.0373366},
{-0.0063115,-0.3655947,0.0381375},
{-0.0062366,-0.3655450,0.0389384},
{-0.0061617,-0.3654953,0.0397393},
{-0.0060868,-0.3654456,0.0405402}
};
Points1 = {
{0.0000000,0.0000000,0.0000000},
{-0.0000000,-0.4005477,0.0000000},
{-0.0090626,-0.4074115,0.0012045},
{-0.0000000,-0.4053996,0.0379177},
{-0.0000000,-0.3956958,-0.0379177},
{0.0134912,-0.0090013,0.0543928},
{0.0195756,-0.0234107,0.0428096},
{0.0094082,-0.0265536,0.0593382},
{0.0001520,-0.0004141,0.0484176},
{0.0109257,-0.0071200,0.0377325},
{-0.0057597,-0.0145826,0.0615408},
{0.0163852,-0.0403059,0.0490339},
{0.0146804,-0.0349983,0.0315081},
{0.0138666,-0.0150697,0.0238377},
{-0.0098668,-0.0334709,0.0587196},
{0.0032686,-0.0438153,0.0577509},
{-0.0029925,-0.0209807,0.0391695},
{-0.0121470,-0.0045028,0.0456190},
{-0.0000086,-0.0044170,0.0264678},
{-0.0119278,-0.0238926,0.0468946},
{0.0097996,-0.0634141,0.0515156},
{0.0155847,-0.0563363,0.0367000},
{0.0081241,-0.0475699,0.0184024},
{0.0093953,-0.0253342,0.0120053},
{0.0178032,-0.0057478,0.0054876},
{0.0100742,0.0063371,0.0153148},
{-0.0118659,-0.0398539,0.0348988},
{-0.0097478,-0.0518079,0.0495840},
{-0.0089488,-0.0266687,0.0239571},

Biology Open • Supplementary information

{-0.0059414,0.0111555,0.0153428},
{-0.0110261,-0.0061372,0.0151242},
{-0.0072163,-0.0732666,0.0429999},
{0.0061066,-0.0840718,0.0499518},
{0.0165959,-0.0788088,0.0354027},
{0.0080545,-0.0678671,0.0202205},
{-0.0114013,-0.0481882,0.0198723},
{-0.0040686,-0.0342532,0.0098188},
{-0.0004304,-0.0207896,-0.0046225},
{0.0123984,-0.0162939,-0.0062762},
{0.0167528,-0.0018731,-0.0107184},
{0.0164263,0.0135709,-0.0011668},
{0.0030361,0.0205947,0.0086675},
{-0.0137861,-0.0598187,0.0326083},
{-0.0114537,-0.0130713,0.0055814},
{-0.0117234,0.0177569,0.0015479},
{-0.0178840,0.0024617,0.0030539},
{-0.0065798,-0.0811533,0.0267032},
{-0.0041637,-0.0973039,0.0404902},
{0.0141278,-0.1021921,0.0431595},
{0.0120599,-0.0873128,0.0209665},
{-0.0097896,-0.0648157,0.0206412},
{-0.0140476,-0.0092506,-0.0098462},
{0.0006361,-0.0095534,-0.0179947},
{0.0057782,0.0081247,-0.0165634},
{0.0016258,0.0210162,-0.0078255},
{-0.0101696,0.0087925,-0.0140020},
{0.0004282,-0.1023677,0.0224565},
{-0.0017985,-0.1163820,0.0386090},
{0.0152147,-0.1232234,0.0411768},
{0.0181479,-0.1059326,0.0253672},
{0.0060344,-0.1202750,0.0186269},
{0.0000133,-0.1358949,0.0338084},
{0.0162852,-0.1442906,0.0390501},
{0.0216592,-0.1285784,0.0249034},
{0.0093879,-0.1400022,0.0159141},
{0.0022593,-0.1560161,0.0310453},
{0.0185307,-0.1655895,0.0364265},
{0.0239185,-0.1504636,0.0223866},
{0.0110874,-0.1613205,0.0135791},
{0.0037153,-0.1761144,0.0279368},
{0.0188227,-0.1871262,0.0341941},
{0.0254206,-0.1703630,0.0202834},
{0.0124212,-0.1820760,0.0114906},
{0.0025539,-0.1968638,0.0206747},
{0.0135458,-0.2079764,0.0305105},

Biology Open • Supplementary information

{0.0266731,-0.2058147,0.0232249},
{0.0257572,-0.1896096,0.0162607},
{0.0155208,-0.2056268,0.0085505},
{0.0022740,-0.2179810,0.0176532},
{0.0107292,-0.2333322,0.0266342},
{0.0239519,-0.2255224,0.0274938},
{0.0260025,-0.2232924,0.0125616},
{0.0137075,-0.2303030,0.0066284},
{0.0019201,-0.2429333,0.0149718},
{0.0117500,-0.2568309,0.0255453},
{0.0247259,-0.2456690,0.0245542},
{0.0258744,-0.2467997,0.0095996},
{0.0123341,-0.2548773,0.0043268},
{0.0027913,-0.2664749,0.0142666},
{0.0094975,-0.2805213,0.0233734},
{0.0251312,-0.2688895,0.0216765},
{0.0250593,-0.2680198,0.0058680},
{0.0096884,-0.2798362,0.0023208},
{0.0024883,-0.2951941,0.0102281},
{0.0105957,-0.3027779,0.0231018},
{0.0247274,-0.2917908,0.0199799},
{0.0252598,-0.2870083,0.0038393},
{0.0147644,-0.3002730,-0.0026943},
{0.0037397,-0.3169299,0.0007275},
{0.0024242,-0.3193567,0.0174327},
{0.0210861,-0.3188511,0.0207393},
{0.0275974,-0.3100786,0.0078499},
{0.0218623,-0.3230209,-0.0046192},
{0.0061627,-0.3352295,-0.0063739},
{-0.0001603,-0.3393486,0.0086947},
{0.0113036,-0.3372411,0.0225323},
{0.0276712,-0.3356008,0.0106095},
{0.0216492,-0.3441143,-0.0078256},
{0.0043558,-0.3560472,-0.0115072},
{-0.0020762,-0.3615745,0.0040258},
{0.0015643,-0.3546918,0.0202349},
{0.0213266,-0.3540439,0.0199733},
{0.0271570,-0.3574930,0.0047782},
{0.0205881,-0.3650392,-0.0122949},
{0.0093316,-0.3784845,-0.0227170},
{-0.0049240,-0.3738487,-0.0127913},
{-0.0059850,-0.3862046,0.0026637},
{-0.0055126,-0.3773997,0.0188508},
{0.0097654,-0.3727677,0.0245390},
{0.0262603,-0.3750129,0.0167486},
{0.0251184,-0.3800623,-0.0001381},

Biology Open • Supplementary information

{0.0238744,-0.3883799,-0.0168911},
{0.0166224,-0.4026843,-0.0281305},
{0.0040081,-0.3924952,-0.0354762},
{-0.0088440,-0.3833446,-0.0274053},
{-0.0152827,-0.3923608,-0.0117659},
{-0.0056566,-0.4086660,-0.0030710},
{-0.0163971,-0.4000898,0.0102279},
{-0.0142409,-0.3928091,0.0278328},
{0.0019812,-0.3884986,0.0325822},
{0.0195709,-0.3929957,0.0259937},
{0.0331229,-0.3940515,0.0148024},
{0.0256122,-0.4006175,-0.0017283},
{0.0277278,-0.4143587,-0.0152079},
{0.0162863,-0.4269583,-0.0257782},
{0.0023783,-0.4142003,-0.0368898},
{-0.0127014,-0.4013446,-0.0373519},
{-0.0257905,-0.3950825,-0.0264695},
{-0.0255860,-0.4097185,-0.0112237},
{-0.0163333,-0.4274074,-0.0165270},
{0.0001460,-0.4271325,-0.0109655},
{0.0028110,-0.4246942,0.0070181},
{-0.0133866,-0.4220397,0.0130327},
{-0.0230093,-0.4098967,0.0264859},
{-0.0041618,-0.4072346,0.0375860},
{0.0123712,-0.4111188,0.0310444},
{0.0301771,-0.4123528,0.0228200},
{0.0252149,-0.4170717,0.0067290},
{0.0156040,-0.4233609,-0.0060702},
{-0.0001350,-0.4331072,-0.0281473},
{-0.0134687,-0.4246165,-0.0356822},
{-0.0279895,-0.4153899,-0.0298487},
{0.0182509,-0.4280107,0.0195860},
{0.0014608,-0.4305834,0.0256913},
{-0.0136215,-0.4261330,0.0312956},
{-0.0007858,-0.0696747,0.0180321},
{-0.0094207,-0.0606532,0.0195544},
{0.0089801,-0.0355801,0.0585202},
{-0.0129573,-0.0144574,0.0539405},
{-0.0015883,-0.0592670,0.0542278},
{-0.0057475,-0.0665554,0.0212754},
{-0.0012873,-0.0855182,0.0194281},
{-0.0063823,0.0017668,-0.0160986},
{-0.0113780,-0.0465155,0.0389663},
{0.0030170,-0.0277269,0.0620457},
{-0.0092770,-0.0687534,0.0243346},
{-0.0009743,-0.0565076,0.0173762},

Biology Open • Supplementary information

{-0.0120304,-0.0177457,0.0480797},
{0.0124310,-0.0313815,0.0569308},
{-0.0095559,-0.0311692,0.0375772},
{0.0130485,-0.0358692,0.0553369},
{-0.0008136,-0.0780615,0.0508276},
{-0.0135314,-0.0456994,0.0295855},
{-0.0127495,-0.0579495,0.0208128},
{-0.0148468,-0.0566786,0.0220155},
{-0.0022649,-0.0430732,0.0128468},
{0.0098896,-0.0603811,0.0226089},
{0.0198130,-0.0138106,0.0374413},
{-0.0028533,-0.0458846,0.0586054},
{0.0123874,-0.0029245,0.0162904},
{-0.0080938,-0.0506941,0.0178906},
{-0.0054562,-0.0842739,0.0419608},
{0.0051033,0.0153900,0.0124100},
{-0.0103443,0.0177069,-0.0056891},
{-0.0064606,-0.0700129,0.0449144},
{0.0071768,-0.0880590,0.0183474},
{0.0118356,-0.0772473,0.0229674},
{0.0070913,-0.0490382,0.0555769},
{0.0132193,-0.0131069,0.0071732},
{-0.0052905,-0.0244395,0.0043263},
{-0.0065845,-0.0010081,0.0189746},
{-0.0092507,-0.0583691,0.0462168},
{-0.0056741,-0.0271566,0.0621048},
{0.0077065,-0.0065940,0.0567220},
{0.0124653,-0.0524211,0.0279836},
{-0.0018387,-0.0188076,-0.0113839},
{-0.0126285,-0.0253642,0.0515685},
{0.0146743,0.0137996,-0.0066253},
{-0.0037177,0.0223623,0.0034516},
{0.0005784,-0.0881887,0.0492988},
{-0.0119004,-0.0409996,0.0466876},
{0.0181941,-0.0289921,0.0396440},
{0.0106133,-0.0445036,0.0225374},
{0.0163296,0.0107717,0.0067453},
{0.0188193,0.0021573,0.0063004},
{0.0196999,-0.0020532,-0.0031890},
{0.0126192,-0.0208508,0.0210246},
{0.0104747,-0.0358910,0.0200737},
{0.0148661,-0.0152162,0.0300765},
{-0.0158974,-0.0507650,0.0247239},
{0.0027508,-0.0396841,0.0602052},
{0.0153800,-0.0467414,0.0358290},
{0.0132938,-0.0104827,0.0123741},

Biology Open • Supplementary information

{0.0009388,0.0045459,-0.0181083},
{0.0079339,-0.0007898,-0.0179706},
{0.0095025,-0.0697542,0.0500632},
{0.0135713,-0.0629897,0.0475581},
{0.0159403,-0.0612004,0.0395451},
{0.0161227,-0.0708493,0.0334428},
{-0.0042936,-0.0773014,0.0228872},
{-0.0120626,0.0038596,0.0130129},
{0.0070190,-0.0011597,0.0513869},
{0.0174639,-0.0096622,0.0468405},
{0.0136157,-0.0244212,0.0560477},
{-0.0108284,-0.0369215,0.0235850},
{-0.0166909,-0.0077323,-0.0023779},
{0.0148506,-0.0853625,0.0254923},
{-0.0063392,-0.0059235,-0.0176881},
{0.0037378,0.0145383,-0.0137806},
{-0.0084869,-0.0340759,0.0148797},
{-0.0135086,0.0106328,-0.0096822},
{-0.0183345,0.0051135,-0.0015452},
{-0.0082869,-0.0722286,0.0367764},
{0.0106127,-0.0179488,-0.0008822},
{0.0114220,-0.0468090,0.0533621},
{0.0079291,-0.0190307,-0.0083590},
{0.0091889,-0.0143323,-0.0128115},
{0.0039347,-0.0611727,0.0176215},
{0.0081926,-0.0521836,0.0199655},
{0.0011197,-0.0782310,0.0186665},
{-0.0166675,-0.0577196,0.0280820},
{-0.0055410,-0.0013992,0.0485190},
{-0.0120149,-0.0316737,0.0553429},
{0.0164533,-0.0178476,0.0503438},
{0.0137656,-0.0791560,0.0448971},
{0.0172452,-0.0246891,0.0326328},
{0.0076720,-0.0327565,0.0618960},
{-0.0156255,-0.0033422,0.0092402},
{0.0132504,-0.0166001,0.0560187},
{0.0142310,-0.0630147,0.0303485},
{-0.0139781,-0.0515807,0.0209667},
{-0.0129244,-0.0343247,0.0489763},
{0.0127796,-0.0428320,0.0284560},
{-0.0071920,-0.0398530,0.0599266},
{0.0061073,-0.0079660,-0.0175496},
{0.0038081,-0.0158727,-0.0141130},
{-0.0132548,-0.0110983,0.0479405},
{-0.0096145,-0.0460685,0.0539562},
{0.0160646,-0.0556316,0.0440229},

Biology Open • Supplementary information

{-0.0155555,0.0114939,0.0014609},
{-0.0127251,0.0094399,0.0108539},
{-0.0017549,-0.0700002,0.0510634},
{0.0122408,-0.0095607,0.0317768},
{0.0127969,-0.0036071,0.0513668},
{0.0140733,-0.0059126,0.0402541},
{-0.0117928,-0.0138526,-0.0080193},
{0.0052854,-0.0226100,0.0021941},
{0.0003405,-0.0505003,0.0158953},
{0.0184840,0.0032240,-0.0070848},
{0.0158432,0.0016727,-0.0122847},
{0.0118158,-0.0079451,-0.0147338},
{-0.0121265,0.0014138,-0.0144153},
{-0.0135406,-0.0061570,-0.0123522},
{0.0163449,0.0136300,0.0029920},
{0.0097665,0.0196534,-0.0021417},
{0.0083365,0.0142976,-0.0123451},
{0.0074493,-0.0232760,0.0602390},
{-0.0006568,-0.0088912,0.0594137},
{-0.0001532,-0.0021547,0.0535448},
{0.0049743,-0.0141337,0.0607803},
{0.0077671,0.0075722,0.0154597},
{0.0114000,-0.0179298,0.0146600},
{0.0036438,-0.0260485,0.0618049},
{-0.0084421,-0.0178044,0.0228305},
{-0.0094618,-0.0179946,0.0113968},
{-0.0059971,-0.0103054,0.0254932},
{-0.0139608,-0.0522654,0.0333506},
{-0.0105188,-0.0631832,0.0386028},
{-0.0064204,-0.0299893,0.0088248},
{0.0191906,-0.0190708,0.0435651},
{0.0113399,-0.0087245,0.0260906},
{0.0040072,-0.0009754,0.0228581},
{-0.0033227,-0.0783880,0.0497007},
{-0.0022297,0.0226193,-0.0030800},
{-0.0010597,-0.0136315,0.0368546},
{-0.0101394,-0.0148143,0.0023205},
{0.0120192,-0.0297301,0.0241558},
{0.0095771,-0.0280400,0.0161403},
{-0.0102047,-0.0071755,0.0496106},
{0.0173745,-0.0342128,0.0451894},
{-0.0111976,-0.0552901,0.0391437},
{-0.0069299,-0.0835675,0.0354874},
{0.0040191,-0.0054218,0.0290640},
{-0.0027720,-0.0047589,-0.0188083},
{-0.0116999,-0.0359059,0.0549276},

{-0.0027920,0.0091982,-0.0159000},
{-0.0040091,0.0171490,-0.0117421},
{0.0172651,-0.0887072,0.0342123},
{0.0034259,-0.0765000,0.0179296},
{0.0152721,-0.0915249,0.0420580},
{0.0088950,0.0198436,0.0050689},
{0.0108957,0.0094598,0.0137308},
{0.0010193,0.0155862,-0.0134747},
{0.0149152,-0.0120867,-0.0083344},
{0.0175650,-0.0079058,-0.0071473},
{-0.0080707,-0.0251934,0.0286621},
{-0.0101150,-0.0358256,0.0302970},
{-0.0124327,-0.0639894,0.0242359},
{-0.0013133,0.0027606,0.0189289},
{0.0038529,0.0075959,0.0163304},
{-0.0023334,-0.0029595,0.0241821},
{0.0002381,-0.0319745,0.0613361},
{0.0063989,-0.0450741,0.0564484},
{-0.0108372,-0.0206095,0.0591316},
{-0.0108121,-0.0273311,0.0584065},
{0.0083713,0.0210303,0.0024954},
{0.0158350,0.0146895,0.0013241},
{-0.0065976,-0.0113329,0.0602043},
{0.0140968,-0.0552000,0.0323916},
{-0.0114887,-0.0493921,0.0452815},
{-0.0138953,-0.0523800,0.0207415},
{0.0101435,-0.0875543,0.0476901},
{-0.0090687,-0.0262145,0.0166865},
{-0.0093474,-0.0159635,0.0160441},
{-0.0040958,0.0166110,0.0119069},
{0.0008275,-0.0707788,0.0527227},
{0.0054476,-0.0652548,0.0528848},
{-0.0020892,-0.0649312,0.0524089},
{0.0153308,-0.0765145,0.0292732},
{-0.0115214,-0.0189971,0.0461321},
{0.0098688,-0.0258115,0.0162426},
{0.0047669,-0.0545124,0.0552114},
{0.0076806,-0.0364315,0.0586999},
{0.0088223,-0.0875038,0.0187037},
{-0.0062686,-0.0549067,0.0536065},
{0.0165779,-0.0444252,0.0414321},
{-0.0110218,-0.0444070,0.0199275},
{0.0195926,-0.0213896,0.0352467},
{-0.0037254,-0.0632193,0.0191162},
{0.0153930,-0.0020645,0.0120243},
{0.0057069,0.0056013,0.0164710},

Biology Open • Supplementary information

{0.0008468,-0.0336154,0.0612252},
{-0.0137045,0.0142173,-0.0047768},
{0.0098069,0.0162397,0.0095657},
{-0.0098658,0.0165695,0.0076755},
{-0.0172042,-0.0056865,-0.0048600},
{-0.0173067,0.0005260,-0.0069715},
{-0.0045309,-0.0180918,0.0624668},
{-0.0121567,-0.0354997,0.0409572},
{0.0051652,-0.0484390,0.0564218},
{0.0114962,-0.0430198,0.0543104},
{-0.0080814,-0.0705681,0.0238864},
{0.0116785,0.0073225,0.0141401},
{0.0168569,-0.0110471,-0.0010556},
{-0.0160169,-0.0083001,0.0047051},
{-0.0118112,-0.0580633,0.0362943},
{-0.0067612,-0.0173787,-0.0034931},
{-0.0119599,-0.0284192,0.0439007},
{0.0043019,-0.0772122,0.0512655},
{0.0147692,-0.0514677,0.0483304},
{-0.0016044,-0.0359315,0.0102768},
{0.0057763,-0.0329964,0.0122495},
{0.0173578,-0.0071833,0.0053867},
{0.0167603,-0.0839273,0.0388616},
{-0.0084444,-0.0195078,0.0233965},
{-0.0008126,0.0133508,0.0149736},
{0.0068459,-0.0703233,0.0197215},
{0.0081312,-0.0642626,0.0522846},
{0.0138885,-0.0133560,-0.0087771},
{0.0005140,-0.0093761,0.0598564},
{-0.0066114,-0.0151890,-0.0131599},
{0.0146969,-0.0726011,0.0436552},
{0.0071088,-0.0189645,-0.0096054},
{-0.0098143,-0.0663811,0.0215650},
{0.0123078,-0.0404831,0.0269478},
{0.0143460,-0.0044037,0.0500168},
{0.0043861,-0.0078633,0.0364586},
{-0.0028070,-0.0635709,0.0529263},
{-0.0118370,-0.0612728,0.0353449},
{-0.0133627,-0.0117568,0.0500501},
{-0.0072660,0.0126236,-0.0135690},
{-0.0158723,0.0059526,0.0075477},
{0.0086417,-0.0700469,0.0205603},
{0.0023054,-0.0399884,0.0593600},
{-0.0124827,-0.0222904,0.0509142},
{0.0059530,-0.0063045,-0.0181991},
{0.0142405,-0.0290331,0.0523358},

Biology Open • Supplementary information

{0.0110590,-0.0153836,0.0574286},
{0.0096319,0.0021242,-0.0167628},
{0.0126705,-0.0083148,0.0159190},
{-0.0119612,0.0113385,0.0108269},
{0.0095753,-0.0537957,0.0533096},
{-0.0050522,-0.0390663,0.0606896},
{0.0179713,-0.0295609,0.0463501},
{0.0146476,-0.0134557,-0.0064922},
{-0.0137920,-0.0510438,0.0338148},
{-0.0069799,-0.0749479,0.0419234},
{0.0029573,-0.0534858,0.0558961},
{-0.0040802,-0.0420854,0.0130000},
{0.0102933,0.0108611,-0.0135536},
{-0.0019810,-0.0219496,-0.0007831},
{-0.0084733,-0.0744485,0.0280390},
{-0.0095466,-0.0035562,-0.0163002},
{-0.0026954,0.0220050,-0.0043362},
{-0.0088618,-0.0220452,0.0123089},
{-0.0099282,-0.0653990,0.0214003},
{-0.0054968,-0.0758504,0.0230404},
{0.0008810,0.0120327,-0.0155057},
{0.0183440,-0.0263728,0.0456372},
{-0.0054496,-0.0588565,0.0184884},
{0.0162983,-0.0368397,0.0368826},
{0.0162747,-0.0312281,0.0328604},
{-0.0132753,-0.0121329,-0.0009107},
{0.0122697,0.0142160,0.0094306},
{-0.0123774,-0.0110654,0.0064772},
{-0.0098913,-0.0356902,0.0334356},
{0.0166054,-0.0889573,0.0396896},
{0.0163496,-0.0104747,0.0521001},
{0.0173538,-0.0157717,0.0482669},
{0.0012594,-0.0268344,0.0046983},
{0.0099806,-0.0236633,0.0587797},
{-0.0025802,-0.0096629,0.0293763},
{-0.0178319,-0.0042976,-0.0016265},
{0.0025509,-0.0212759,-0.0035643},
{-0.0089581,-0.0720008,0.0345742},
{-0.0117830,-0.0278117,0.0569951},
{0.0165496,0.0052510,-0.0100686},
{-0.0136626,-0.0629933,0.0281820},
{-0.0140899,-0.0042478,0.0119670},
{0.0110949,-0.0081291,0.0253059},
{-0.0124233,-0.0433324,0.0299740},
{0.0139190,-0.0723675,0.0269281},
{0.0007117,0.0034066,-0.0183447},

{0.0156156,-0.0783429,0.0293816},
{0.0064214,0.0064498,0.0157821},
{-0.0132137,0.0022840,0.0121176},
{0.0054116,-0.0433745,0.0155342},
{-0.0059895,-0.0192254,0.0318375},
{0.0190094,-0.0272372,0.0402159},
{-0.0119383,-0.0632421,0.0215184},
{0.0192919,0.0051664,-0.0028500},
{-0.0128360,-0.0634096,0.0262195},
{0.0104211,-0.0225967,0.0104530},
{0.0182606,-0.0118107,0.0429025},
{0.0080138,0.0019651,-0.0176490},
{0.0136496,-0.0083876,0.0127938},
{-0.0104933,-0.0641835,0.0379938},
{0.0004287,0.0207943,0.0087110},
{0.0026298,0.0033358,-0.0186453},
{0.0349268,-0.4070391,0.0199245},
{0.0047334,0.0206048,-0.0073822},
{-0.0126473,-0.0454325,0.0353848},
{-0.0293917,-0.4029170,-0.0255308},
{0.0021695,0.0233106,-0.0000303},
{0.0134560,-0.0247665,0.0252322},
{-0.0132173,-0.0011105,-0.0137887},
{0.0154994,-0.0312740,0.0502966},
{-0.0137204,0.0135738,0.0045010},
{0.0161587,-0.0404584,0.0380099},
{0.0144211,-0.0451353,0.0326028},
{-0.0124268,-0.0356018,0.0434496},
{0.0162258,-0.0571677,0.0418171},
{-0.0156619,-0.0496780,0.0260963},
{0.0028616,-0.0567558,0.0551845},
{0.0010183,-0.0529455,0.0164807},
{0.0200478,-0.0215734,0.0418038},
{0.0074018,-0.0788350,0.0500229},
{-0.0055689,-0.0855885,0.0265152},
{-0.0177674,-0.0022212,0.0028983},
{0.0134674,-0.0138286,0.0187366},
{0.0129529,-0.0687138,0.0260707},
{-0.0086954,-0.0254739,0.0261107},
{-0.0182420,-0.4131048,0.0338786},
{-0.0080946,-0.0575305,0.0495076},
{-0.0107110,-0.4075198,-0.0097529},
{0.0099827,-0.0446028,0.0212506},
{0.0101457,0.0149706,-0.0106196},
{-0.0227772,-0.4041571,0.0274127},
{0.0146230,-0.0547949,0.0332275},

{0.0002789,-0.0510952,0.0567863},
{-0.0102647,0.0186554,0.0026983},
{-0.0023059,-0.0172170,0.0623343},
{-0.0068170,-0.0075982,0.0228768},
{0.0181090,-0.0106254,0.0400425},
{0.0138681,-0.0252528,0.0542488},
{-0.0132052,-0.0112459,0.0493558},
{0.0185086,0.0084919,-0.0026621},
{-0.0093619,-0.0351788,0.0170017},
{-0.0003146,-0.0145621,-0.0155894},
{-0.0100626,-0.0621021,0.0418837},
{-0.0084087,0.0176566,0.0076047},
{0.0091152,0.0197371,-0.0028458},
{-0.0125083,-0.0278785,0.0540504},
{-0.0055845,-0.4046607,0.0384014},
{-0.0125407,-0.4101624,-0.0359924},
{-0.0124711,-0.4101074,-0.0352485},
{-0.0124016,-0.4100524,-0.0345045},
{-0.0123320,-0.4099973,-0.0337606},
{-0.0122624,-0.4099423,-0.0330166},
{-0.0121929,-0.4098873,-0.0322727},
{-0.0121233,-0.4098323,-0.0315288},
{-0.0120537,-0.4097773,-0.0307848},
{-0.0119842,-0.4097223,-0.0300409},
{-0.0119146,-0.4096672,-0.0292970},
{-0.0118451,-0.4096122,-0.0285530},
{-0.0117755,-0.4095572,-0.0278091},
{-0.0117059,-0.4095022,-0.0270651},
{-0.0116364,-0.4094472,-0.0263212},
{-0.0115668,-0.4093922,-0.0255773},
{-0.0114973,-0.4093371,-0.0248333},
{-0.0114277,-0.4092821,-0.0240894},
{-0.0113581,-0.4092271,-0.0233455},
{-0.0112886,-0.4091721,-0.0226015},
{-0.0112190,-0.4091171,-0.0218576},
{-0.0111494,-0.4090620,-0.0211136},
{-0.0110799,-0.4090070,-0.0203697},
{-0.0110103,-0.4089520,-0.0196258},
{-0.0109408,-0.4088970,-0.0188818},
{-0.0108712,-0.4088420,-0.0181379},
{-0.0108016,-0.4087870,-0.0173939},
{-0.0107321,-0.4087319,-0.0166500},
{-0.0106625,-0.4086769,-0.0159061},
{-0.0105929,-0.4086219,-0.0151621},
{-0.0105234,-0.4085669,-0.0144182},
{-0.0104538,-0.4085119,-0.0136743},

Biology Open • Supplementary information

{-0.0103843,-0.4084569,-0.0129303},
{-0.0103147,-0.4084018,-0.0121864},
{-0.0102451,-0.4083468,-0.0114424},
{-0.0101756,-0.4082918,-0.0106985},
{-0.0101060,-0.4082368,-0.0099546},
{-0.0100364,-0.4081818,-0.0092106},
{-0.0099669,-0.4081267,-0.0084667},
{-0.0098973,-0.4080717,-0.0077227},
{-0.0098278,-0.4080167,-0.0069788},
{-0.0097582,-0.4079617,-0.0062349},
{-0.0096886,-0.4079067,-0.0054909},
{-0.0096191,-0.4078517,-0.0047470},
{-0.0095495,-0.4077966,-0.0040031},
{-0.0094799,-0.4077416,-0.0032591},
{-0.0094104,-0.4076866,-0.0025152},
{-0.0093408,-0.4076316,-0.0017712},
{-0.0092713,-0.4075766,-0.0010273},
{-0.0092017,-0.4075216,-0.0002834},
{-0.0091321,-0.4074665,0.0004606},
{-0.0090626,-0.4074115,0.0012045},
{-0.0089930,-0.4073565,0.0019484},
{-0.0089234,-0.4073015,0.0026924},
{-0.0088539,-0.4072465,0.0034363},
{-0.0087843,-0.4071915,0.0041803},
{-0.0087148,-0.4071364,0.0049242},
{-0.0086452,-0.4070814,0.0056681},
{-0.0085756,-0.4070264,0.0064121},
{-0.0085061,-0.4069714,0.0071560},
{-0.0084365,-0.4069164,0.0079000},
{-0.0083670,-0.4068613,0.0086439},
{-0.0082974,-0.4068063,0.0093878},
{-0.0082278,-0.4067513,0.0101318},
{-0.0081583,-0.4066963,0.0108757},
{-0.0080887,-0.4066413,0.0116196},
{-0.0080191,-0.4065863,0.0123636},
{-0.0079496,-0.4065312,0.0131075},
{-0.0078800,-0.4064762,0.0138515},
{-0.0078105,-0.4064212,0.0145954},
{-0.0077409,-0.4063662,0.0153393},
{-0.0076713,-0.4063112,0.0160833},
{-0.0076018,-0.4062562,0.0168272},
{-0.0075322,-0.4062011,0.0175712},
{-0.0074626,-0.4061461,0.0183151},
{-0.0073931,-0.4060911,0.0190590},
{-0.0073235,-0.4060361,0.0198030},
{-0.0072540,-0.4059811,0.0205469},

```
                    {-0.0071844,-0.4059261,0.0212908},
                    {-0.0071148,-0.4058710,0.0220348},
                    {-0.0070453,-0.4058160,0.0227787},
                    {-0.0069757,-0.4057610,0.0235227},
                    {-0.0069061,-0.4057060,0.0242666},
                    {-0.0068366,-0.4056510,0.0250105},
                    {-0.0067670,-0.4055959,0.0257545},
                    {-0.0066975,-0.4055409,0.0264984},
                    {-0.0066279,-0.4054859,0.0272424},
                    {-0.0065583,-0.4054309,0.0279863},
                    {-0.0064888,-0.4053759,0.0287302},
                    {-0.0064192,-0.4053209,0.0294742},
                    {-0.0063496,-0.4052658,0.0302181},
                    {-0.0062801,-0.4052108,0.0309620},
                    {-0.0062105,-0.4051558,0.0317060},
                    {-0.0061410,-0.4051008,0.0324499},
                    {-0.0060714,-0.4050458,0.0331939},
                    {-0.0060018,-0.4049908,0.0339378},
                    {-0.0059323,-0.4049357,0.0346817},
                    {-0.0058627,-0.4048807,0.0354257},
                    {-0.0057932,-0.4048257,0.0361696},
                    {-0.0057236,-0.4047707,0.0369135},
                    {-0.0056540,-0.4047157,0.0376575}
                    };
        BoundingBoxOnOff = Off;
    };
    AnyFunTransform3DIdentity ScaleFunction = {
        PreTransforms = {&.RBFTransform};
};
};

};
};
```

Biology Open • Supplementary information

ScalingFunctionTLEMLucyFemur_Mirrored_2014051

```
AnyFolder ScalingFunctionTLEMLucyFemur_Mirrored = {
  AnyFolder Left = {
            AnyFolder Thigh = {
        AnyMatrix AMirroring = {
        {1,0,0},
        {0,1,0},
        {0,0,-1}
        };
          AnyFunTransform3DRBF RBFTransform = {
                    RBFDef.Type = RBF_ThinPlate;
                    PolynomDegree = 1;
                    Points0 = ({
                              {0.0000000,0.0000000,0.0000000},
                              {-0.0000000,-0.3616821,0.0000000},
                              {-0.0097563,-0.3678799,0.0012967},
                              {-0.0000000,-0.3660632,0.0408203},
                              {-0.0000000,-0.3573010,-0.0408203},
                              {0.0161460,-0.0072838,0.0601290},
                              {0.0220217,-0.0203698,0.0463848},
                              {0.0123977,-0.0241932,0.0668573},
                              {0.0006898,0.0018121,0.0538181},
                              {0.0122809,-0.0068668,0.0414535},
                              {-0.0058991,-0.0138188,0.0648412},
                              {0.0211469,-0.0380855,0.0538111},
                              {0.0172133,-0.0317342,0.0328381},
                              {0.0177311,-0.0131946,0.0256176},
                              {-0.0110079,-0.0308867,0.0632370},
                              {0.0039435,-0.0395977,0.0638271},
                              {-0.0041683,-0.0187472,0.0413214},
                              {-0.0177999,-0.0021535,0.0496084},
                              {0.0001603,-0.0053126,0.0279593},
                              {-0.0211600,-0.0216243,0.0497147},
                              {0.0096163,-0.0568663,0.0545719},
                              {0.0162112,-0.0515013,0.0395230},
                              {0.0093533,-0.0433265,0.0187387},
                              {0.0119398,-0.0238469,0.0118403},
                              {0.0210707,-0.0048848,0.0065878},
                              {0.0125013,0.0064094,0.0177702},
                              {-0.0191523,-0.0356053,0.0382429},
                              {-0.0115821,-0.0474937,0.0537648},
                              {-0.0100073,-0.0236371,0.0267322},
                              {-0.0078925,0.0107098,0.0182833},
                              {-0.0134217,-0.0055356,0.0182000},
                              {-0.0090061,-0.0665700,0.0475484},
```

Biology Open • Supplementary information

{0.0071095,-0.0761976,0.0520035},
{0.0182119,-0.0704149,0.0386546},
{0.0073764,-0.0609481,0.0221006},
{-0.0122984,-0.0439585,0.0198428},
{-0.0032253,-0.0306792,0.0110995},
{-0.0012975,-0.0198451,-0.0052592},
{0.0140405,-0.0161125,-0.0071649},
{0.0189222,-0.0014898,-0.0127226},
{0.0181642,0.0132812,-0.0011050},
{0.0045119,0.0200001,0.0091189},
{-0.0176761,-0.0541983,0.0348587},
{-0.0136548,-0.0142015,0.0054311},
{-0.0135510,0.0182940,0.0017175},
{-0.0223865,0.0023399,0.0028018},
{-0.0086455,-0.0738247,0.0283843},
{-0.0050356,-0.0877749,0.0442195},
{0.0160135,-0.0919319,0.0465120},
{0.0128290,-0.0785810,0.0232431},
{-0.0168328,-0.0616327,0.0155917},
{-0.0172369,-0.0094567,-0.0119502},
{0.0002090,-0.0093641,-0.0209779},
{0.0064827,0.0081538,-0.0198453},
{0.0010777,0.0210422,-0.0084721},
{-0.0129485,0.0085451,-0.0162212},
{-0.0002322,-0.0924033,0.0245644},
{-0.0028175,-0.1052263,0.0418294},
{0.0172322,-0.1111444,0.0445760},
{0.0203259,-0.0957409,0.0271449},
{0.0057102,-0.1088933,0.0195113},
{-0.0010852,-0.1230776,0.0363132},
{0.0182727,-0.1303312,0.0422884},
{0.0242891,-0.1161703,0.0265054},
{0.0094531,-0.1268584,0.0162294},
{0.0014244,-0.1413293,0.0331653},
{0.0207271,-0.1496427,0.0394157},
{0.0267562,-0.1360142,0.0236798},
{0.0113839,-0.1461651,0.0136024},
{0.0030845,-0.1595094,0.0297156},
{0.0209528,-0.1691386,0.0369775},
{0.0283958,-0.1540209,0.0213795},
{0.0129499,-0.1649012,0.0113589},
{0.0016490,-0.1783108,0.0216287},
{0.0147105,-0.1880678,0.0328614},
{0.0298491,-0.1859865,0.0248551},
{0.0286645,-0.1714377,0.0169488},
{0.0165897,-0.1861006,0.0082595},

Biology Open • Supplementary information

{0.0014000,-0.1973476,0.0183740},
{0.0114301,-0.2109792,0.0286157},
{0.0267422,-0.2037622,0.0296951},
{0.0288630,-0.2018335,0.0130127},
{0.0145471,-0.2083505,0.0062833},
{0.0011300,-0.2198071,0.0155602},
{0.0126610,-0.2321235,0.0275354},
{0.0275647,-0.2219268,0.0265185},
{0.0286645,-0.2230253,0.0098701},
{0.0130434,-0.2304822,0.0039177},
{0.0022984,-0.2409585,0.0149650},
{0.0101315,-0.2534740,0.0252125},
{0.0279485,-0.2428555,0.0234276},
{0.0276684,-0.2421559,0.0058691},
{0.0101250,-0.2529527,0.0018983},
{0.0021376,-0.2667831,0.0107073},
{0.0114309,-0.2734905,0.0249960},
{0.0273923,-0.2634953,0.0216324},
{0.0278429,-0.2592546,0.0037692},
{0.0159101,-0.2712985,-0.0033988},
{0.0036708,-0.2863280,0.0004644},
{0.0023115,-0.2884756,0.0187554},
{0.0231385,-0.2879051,0.0224853},
{0.0304009,-0.2799973,0.0083549},
{0.0238189,-0.2917246,-0.0052462},
{0.0064904,-0.3027687,-0.0070587},
{-0.0003492,-0.3064690,0.0093014},
{0.0122179,-0.3045269,0.0243511},
{0.0301837,-0.3030127,0.0114462},
{0.0234182,-0.3107336,-0.0085198},
{0.0046893,-0.3214995,-0.0123881},
{-0.0022352,-0.3264905,0.0043340},
{0.0016841,-0.3202756,0.0217839},
{0.0229722,-0.3196887,0.0215083},
{0.0292438,-0.3228042,0.0051430},
{0.0221641,-0.3296190,-0.0132361},
{0.0100459,-0.3417597,-0.0244560},
{-0.0053009,-0.3375737,-0.0137705},
{-0.0064432,-0.3487307,0.0028676},
{-0.0059346,-0.3407801,0.0202938},
{0.0105130,-0.3365976,0.0264175},
{0.0282695,-0.3386249,0.0180307},
{0.0270402,-0.3431844,-0.0001487},
{0.0257040,-0.3506950,-0.0181851},
{0.0178948,-0.3636114,-0.0302838},
{0.0043149,-0.3544109,-0.0381949},

Biology Open • Supplementary information

{-0.0095210,-0.3461482,-0.0295031},
{-0.0164526,-0.3542895,-0.0126666},
{-0.0060896,-0.3690127,-0.0033061},
{-0.0176523,-0.3612686,0.0110109},
{-0.0153310,-0.3546943,0.0299634},
{0.0021329,-0.3508021,0.0350763},
{0.0210690,-0.3548628,0.0279835},
{0.0356594,-0.3558162,0.0159356},
{0.0275718,-0.3617450,-0.0018607},
{0.0298503,-0.3741530,-0.0163721},
{0.0175320,-0.3855300,-0.0277515},
{0.0025604,-0.3740099,-0.0397137},
{-0.0136727,-0.3624016,-0.0402102},
{-0.0277647,-0.3567472,-0.0284947},
{-0.0275446,-0.3699630,-0.0120829},
{-0.0175846,-0.3859355,-0.0177922},
{0.0001572,-0.3856873,-0.0118050},
{0.0030262,-0.3834856,0.0075553},
{-0.0144114,-0.3810887,0.0140304},
{-0.0247706,-0.3701239,0.0285134},
{-0.0044804,-0.3677201,0.0404632},
{0.0133183,-0.3712274,0.0334208},
{0.0324861,-0.3723417,0.0245668},
{0.0271451,-0.3766027,0.0072441},
{0.0167985,-0.3822816,-0.0065349},
{-0.0001454,-0.3910823,-0.0303010},
{-0.0144998,-0.3834154,-0.0384137},
{-0.0301321,-0.3750841,-0.0321336},
{0.0196491,-0.3864804,0.0210853},
{0.0015726,-0.3888034,0.0276569},
{-0.0146652,-0.3847848,0.0336913},
{-0.0014588,-0.0619951,0.0219778},
{-0.0118435,-0.0550626,0.0139639},
{0.0129812,-0.0349966,0.0673128},
{-0.0162907,-0.0105666,0.0597739},
{-0.0012885,-0.0520816,0.0562989},
{-0.0090599,-0.0631298,0.0172224},
{-0.0000222,-0.0771663,0.0231897},
{-0.0068148,0.0023885,-0.0160086},
{-0.0180544,-0.0425409,0.0446585},
{0.0020525,-0.0249904,0.0691549},
{-0.0157097,-0.0662315,0.0238393},
{-0.0010518,-0.0510686,0.0199353},
{-0.0208874,-0.0152865,0.0500034},
{0.0168316,-0.0280857,0.0666680},
{-0.0187392,-0.0282333,0.0403282},

{0.0191622,-0.0351633,0.0622707},
{0.0001216,-0.0706289,0.0524049},
{-0.0169862,-0.0399062,0.0311827},
{-0.0185737,-0.0533066,0.0151224},
{-0.0219058,-0.0528723,0.0222263},
{-0.0028271,-0.0391620,0.0148452},
{0.0117900,-0.0553425,0.0235348},
{0.0219835,-0.0106400,0.0396338},
{-0.0025366,-0.0401046,0.0625273},
{0.0141017,-0.0018767,0.0198108},
{-0.0078770,-0.0473929,0.0173542},
{-0.0065351,-0.0766431,0.0470258},
{0.0065585,0.0145593,0.0161638},
{-0.0118588,0.0172875,-0.0085064},
{-0.0074087,-0.0627500,0.0506700},
{0.0071940,-0.0780068,0.0213362},
{0.0126736,-0.0708176,0.0244108},
{0.0074694,-0.0436450,0.0604196},
{0.0154186,-0.0139471,0.0064312},
{-0.0053535,-0.0213210,0.0040094},
{-0.0088733,0.0002018,0.0205402},
{-0.0112471,-0.0542608,0.0500266},
{-0.0061625,-0.0248673,0.0653244},
{0.0093695,-0.0065826,0.0614157},
{0.0138015,-0.0467322,0.0284333},
{-0.0020999,-0.0186885,-0.0116392},
{-0.0185640,-0.0225591,0.0566779},
{0.0175798,0.0124360,-0.0068467},
{-0.0039962,0.0217127,0.0053055},
{0.0014975,-0.0784591,0.0514056},
{-0.0172997,-0.0386971,0.0525252},
{0.0196540,-0.0268271,0.0416806},
{0.0135296,-0.0406504,0.0236882},
{0.0182471,0.0095959,0.0087906},
{0.0218667,0.0016804,0.0058793},
{0.0224975,-0.0022652,-0.0022994},
{0.0166750,-0.0191391,0.0236436},
{0.0119785,-0.0328723,0.0195376},
{0.0187631,-0.0133469,0.0308741},
{-0.0187543,-0.0461463,0.0257491},
{0.0039834,-0.0373928,0.0683089},
{0.0157029,-0.0423169,0.0385226},
{0.0164003,-0.0087069,0.0131181},
{0.0008253,0.0033370,-0.0212576},
{0.0101707,-0.0009724,-0.0205688},
{0.0102948,-0.0642683,0.0524532},

Biology Open • Supplementary information

{0.0152217,-0.0557944,0.0508978},
{0.0172973,-0.0565707,0.0419468},
{0.0167723,-0.0635334,0.0369834},
{-0.0047397,-0.0695325,0.0241398},
{-0.0152913,0.0035778,0.0161461},
{0.0091697,0.0009340,0.0575160},
{0.0196686,-0.0089533,0.0500006},
{0.0183998,-0.0212943,0.0622968},
{-0.0114340,-0.0316810,0.0250940},
{-0.0206442,-0.0092599,-0.0028425},
{0.0166868,-0.0784295,0.0276925},
{-0.0069678,-0.0054521,-0.0207011},
{0.0049490,0.0151109,-0.0160093},
{-0.0084025,-0.0318937,0.0162788},
{-0.0173679,0.0095594,-0.0108509},
{-0.0218912,0.0063457,-0.0020698},
{-0.0106547,-0.0668750,0.0397749},
{0.0136098,-0.0170666,-0.0018890},
{0.0141599,-0.0441405,0.0587594},
{0.0093255,-0.0183218,-0.0083008},
{0.0106367,-0.0128276,-0.0154269},
{0.0037350,-0.0549365,0.0209786},
{0.0087662,-0.0472902,0.0195496},
{0.0016839,-0.0708213,0.0221325},
{-0.0202977,-0.0530540,0.0301270},
{-0.0068584,0.0021781,0.0530002},
{-0.0144817,-0.0297218,0.0605833},
{0.0197015,-0.0165217,0.0560654},
{0.0156609,-0.0705363,0.0477736},
{0.0207876,-0.0221433,0.0330733},
{0.0092324,-0.0300767,0.0713160},
{-0.0203392,-0.0034488,0.0097332},
{0.0156978,-0.0143438,0.0629053},
{0.0143267,-0.0563519,0.0317489},
{-0.0161685,-0.0465391,0.0188591},
{-0.0196397,-0.0309875,0.0546496},
{0.0154804,-0.0380422,0.0305433},
{-0.0077774,-0.0351303,0.0637765},
{0.0063980,-0.0086419,-0.0201654},
{0.0030146,-0.0151094,-0.0165974},
{-0.0210080,-0.0096030,0.0507855},
{-0.0105684,-0.0411377,0.0586146},
{0.0180619,-0.0498960,0.0469835},
{-0.0192318,0.0116108,0.0024070},
{-0.0165651,0.0083622,0.0126870},
{-0.0014333,-0.0627138,0.0534746},

Biology Open • Supplementary information

{0.0145127,-0.0080178,0.0353667},
{0.0148374,-0.0012228,0.0566181},
{0.0153834,-0.0062427,0.0444727},
{-0.0155164,-0.0132103,-0.0096398},
{0.0068685,-0.0221022,0.0011547},
{0.0003332,-0.0458500,0.0183716},
{0.0205582,0.0040956,-0.0082969},
{0.0175752,0.0009340,-0.0148407},
{0.0142357,-0.0063623,-0.0169332},
{-0.0147285,0.0023412,-0.0164678},
{-0.0158781,-0.0064451,-0.0151346},
{0.0175951,0.0133456,0.0037559},
{0.0117660,0.0189899,-0.0030690},
{0.0096040,0.0151827,-0.0137450},
{0.0096565,-0.0215194,0.0662734},
{-0.0006793,-0.0074494,0.0631218},
{0.0007819,-0.0004519,0.0600781},
{0.0065377,-0.0125433,0.0654199},
{0.0090461,0.0073096,0.0188711},
{0.0151418,-0.0168302,0.0146465},
{0.0025166,-0.0231863,0.0688894},
{-0.0097891,-0.0152077,0.0253086},
{-0.0109149,-0.0177935,0.0119825},
{-0.0072079,-0.0099373,0.0272725},
{-0.0183727,-0.0467429,0.0356276},
{-0.0126871,-0.0584555,0.0428141},
{-0.0057054,-0.0273042,0.0103554},
{0.0215142,-0.0159693,0.0474531},
{0.0128492,-0.0070016,0.0287645},
{0.0052134,-0.0017047,0.0247463},
{-0.0024284,-0.0707498,0.0513049},
{-0.0036140,0.0220593,-0.0040484},
{-0.0002405,-0.0121253,0.0390050},
{-0.0119811,-0.0156737,0.0015445},
{0.0142082,-0.0279792,0.0246553},
{0.0122244,-0.0262013,0.0169829},
{-0.0146264,-0.0038855,0.0549384},
{0.0207450,-0.0325771,0.0496412},
{-0.0155040,-0.0497560,0.0437087},
{-0.0081119,-0.0756450,0.0387365},
{0.0039684,-0.0062047,0.0313900},
{-0.0023726,-0.0038892,-0.0220521},
{-0.0136977,-0.0339839,0.0602619},
{-0.0035399,0.0089081,-0.0188894},
{-0.0049820,0.0176571,-0.0132254},
{0.0193902,-0.0803191,0.0360429},

{0.0040648,-0.0697109,0.0218205},
{0.0170470,-0.0818610,0.0456892},
{0.0104175,0.0188635,0.0064965},
{0.0135206,0.0092549,0.0158079},
{0.0023933,0.0160498,-0.0157404},
{0.0164753,-0.0118197,-0.0096995},
{0.0202584,-0.0066858,-0.0082383},
{-0.0098401,-0.0226001,0.0314028},
{-0.0130937,-0.0309622,0.0320764},
{-0.0201510,-0.0613297,0.0222264},
{-0.0010389,0.0036055,0.0209003},
{0.0042025,0.0073392,0.0199393},
{-0.0021630,-0.0038531,0.0251898},
{-0.0007845,-0.0293574,0.0675788},
{0.0070027,-0.0406327,0.0624565},
{-0.0124363,-0.0183001,0.0627966},
{-0.0120485,-0.0256613,0.0628493},
{0.0101200,0.0197695,0.0039334},
{0.0167636,0.0148266,0.0014155},
{-0.0074981,-0.0106930,0.0632797},
{0.0142533,-0.0503445,0.0351954},
{-0.0146107,-0.0460953,0.0506120},
{-0.0161117,-0.0471301,0.0178900},
{0.0112149,-0.0792793,0.0501460},
{-0.0099060,-0.0230794,0.0186951},
{-0.0114543,-0.0153049,0.0183675},
{-0.0042882,0.0161879,0.0151423},
{0.0014582,-0.0638572,0.0541080},
{0.0048554,-0.0582603,0.0553815},
{-0.0017077,-0.0572302,0.0546635},
{0.0166090,-0.0697169,0.0323038},
{-0.0208812,-0.0164273,0.0474354},
{0.0130454,-0.0241864,0.0178294},
{0.0053123,-0.0483770,0.0582251},
{0.0112225,-0.0361429,0.0673300},
{0.0085729,-0.0771256,0.0216100},
{-0.0070783,-0.0488033,0.0560869},
{0.0182083,-0.0411024,0.0454236},
{-0.0119590,-0.0403809,0.0213546},
{0.0233409,-0.0185535,0.0366343},
{-0.0046347,-0.0571211,0.0208243},
{0.0183166,-0.0006966,0.0140116},
{0.0061913,0.0053512,0.0201003},
{-0.0002364,-0.0310828,0.0680351},
{-0.0170773,0.0139270,-0.0054987},
{0.0110805,0.0156034,0.0117010},

Biology Open • Supplementary information

{-0.0124628,0.0164667,0.0094550},
{-0.0209014,-0.0073407,-0.0051969},
{-0.0210131,0.0010693,-0.0086891},
{-0.0041596,-0.0172332,0.0663041},
{-0.0216595,-0.0322172,0.0451540},
{0.0051908,-0.0426014,0.0613013},
{0.0150003,-0.0416136,0.0606257},
{-0.0135090,-0.0674234,0.0236425},
{0.0146416,0.0073691,0.0160052},
{0.0195615,-0.0112412,-0.0010333},
{-0.0198115,-0.0098244,0.0044735},
{-0.0157054,-0.0521236,0.0395039},
{-0.0081381,-0.0173108,-0.0046152},
{-0.0218324,-0.0262661,0.0471115},
{0.0051446,-0.0707257,0.0530555},
{0.0179359,-0.0471493,0.0528134},
{-0.0005591,-0.0317983,0.0113632},
{0.0071707,-0.0305943,0.0121186},
{0.0206239,-0.0061367,0.0066642},
{0.0187057,-0.0745868,0.0420207},
{-0.0095784,-0.0163113,0.0259524},
{-0.0012181,0.0127661,0.0186855},
{0.0059420,-0.0630691,0.0220087},
{0.0069408,-0.0573878,0.0552614},
{0.0150782,-0.0132884,-0.0101793},
{0.0005533,-0.0078574,0.0633775},
{-0.0088284,-0.0146399,-0.0147837},
{0.0167444,-0.0651105,0.0470347},
{0.0082666,-0.0181741,-0.0095133},
{-0.0175162,-0.0637444,0.0177069},
{0.0153795,-0.0360767,0.0287608},
{0.0162363,-0.0018864,0.0548412},
{0.0052731,-0.0075919,0.0392990},
{-0.0022288,-0.0556644,0.0549909},
{-0.0156238,-0.0550038,0.0377688},
{-0.0204137,-0.0100550,0.0536429},
{-0.0093683,0.0122429,-0.0162013},
{-0.0202927,0.0049166,0.0083887},
{0.0074706,-0.0627945,0.0222133},
{0.0035034,-0.0376103,0.0672421},
{-0.0190752,-0.0194129,0.0555530},
{0.0061758,-0.0074961,-0.0207741},
{0.0192011,-0.0273063,0.0588233},
{0.0130796,-0.0134383,0.0638611},
{0.0120421,0.0019566,-0.0194140},
{0.0154152,-0.0065294,0.0179581},

Biology Open • Supplementary information

{-0.0157335,0.0101219,0.0123981},
{0.0110376,-0.0488274,0.0567911},
{-0.0055170,-0.0343676,0.0647140},
{0.0216204,-0.0273988,0.0512041},
{0.0161331,-0.0135875,-0.0075501},
{-0.0184643,-0.0455633,0.0360087},
{-0.0091978,-0.0682413,0.0460847},
{0.0034304,-0.0471677,0.0588866},
{-0.0048025,-0.0384316,0.0149400},
{0.0118505,0.0109735,-0.0159161},
{-0.0027036,-0.0204189,-0.0015094},
{-0.0117607,-0.0692759,0.0292171},
{-0.0107080,-0.0033455,-0.0190487},
{-0.0042548,0.0214947,-0.0056846},
{-0.0097315,-0.0208529,0.0131997},
{-0.0178958,-0.0627218,0.0171551},
{-0.0068692,-0.0688818,0.0233316},
{0.0012083,0.0120695,-0.0186179},
{0.0215073,-0.0236027,0.0503069},
{-0.0060262,-0.0534931,0.0189523},
{0.0179951,-0.0336785,0.0397267},
{0.0190509,-0.0284972,0.0336394},
{-0.0169188,-0.0133609,-0.0014203},
{0.0135722,0.0136491,0.0114695},
{-0.0150549,-0.0123230,0.0067722},
{-0.0148411,-0.0311945,0.0355412},
{0.0185490,-0.0794042,0.0431266},
{0.0193568,-0.0087326,0.0575704},
{0.0201026,-0.0144353,0.0531586},
{0.0025583,-0.0248211,0.0045714},
{0.0132689,-0.0214866,0.0652899},
{-0.0028669,-0.0099311,0.0309271},
{-0.0219487,-0.0054893,-0.0021853},
{0.0026180,-0.0203806,-0.0040620},
{-0.0116230,-0.0668518,0.0370152},
{-0.0133007,-0.0264159,0.0619176},
{0.0186167,0.0050491,-0.0120059},
{-0.0191359,-0.0591690,0.0288149},
{-0.0178121,-0.0041834,0.0141448},
{0.0123077,-0.0063648,0.0279743},
{-0.0157729,-0.0374010,0.0315335},
{0.0149328,-0.0659100,0.0290095},
{0.0006205,0.0021977,-0.0214613},
{0.0170484,-0.0715687,0.0325980},
{0.0068499,0.0061192,0.0196608},
{-0.0167075,0.0020024,0.0150382},

{0.0059801,-0.0393117,0.0167489},
{-0.0072506,-0.0171067,0.0338751},
{0.0202363,-0.0251596,0.0420997},
{-0.0199311,-0.0601255,0.0171522},
{0.0216641,0.0053410,-0.0027841},
{-0.0197665,-0.0606125,0.0252972},
{0.0129986,-0.0214528,0.0098002},
{0.0201151,-0.0100929,0.0460501},
{0.0104137,0.0017765,-0.0203073},
{0.0166557,-0.0064123,0.0140197},
{-0.0124120,-0.0596549,0.0422637},
{0.0014885,0.0202590,0.0096781},
{0.0028720,0.0020207,-0.0219044},
{0.0376095,-0.3675427,0.0214537},
{0.0049949,0.0206417,-0.0079017},
{-0.0191723,-0.0407590,0.0391310},
{-0.0316406,-0.3638215,-0.0274841},
{0.0021392,0.0226920,0.0000985},
{0.0168435,-0.0229382,0.0264788},
{-0.0157304,-0.0005437,-0.0161456},
{0.0203722,-0.0299750,0.0562762},
{-0.0171077,0.0134146,0.0059615},
{0.0175519,-0.0370653,0.0413374},
{0.0154657,-0.0405127,0.0348980},
{-0.0215172,-0.0323502,0.0481925},
{0.0177998,-0.0515776,0.0444709},
{-0.0183804,-0.0448316,0.0275139},
{0.0033607,-0.0502028,0.0575894},
{0.0010325,-0.0479377,0.0191141},
{0.0224852,-0.0181821,0.0451780},
{0.0084089,-0.0720538,0.0521031},
{-0.0070880,-0.0772912,0.0288023},
{-0.0223712,-0.0028990,0.0024047},
{0.0173136,-0.0120017,0.0204612},
{0.0137772,-0.0623537,0.0276621},
{-0.0100390,-0.0231180,0.0292222},
{-0.0196395,-0.3730207,0.0364730},
{-0.0094445,-0.0526688,0.0528111},
{-0.0115309,-0.3679776,-0.0104995},
{0.0129812,-0.0410477,0.0224072},
{0.0117394,0.0154849,-0.0117191},
{-0.0245208,-0.3649412,0.0295111},
{0.0147476,-0.0502966,0.0364899},
{0.0006132,-0.0446771,0.0600524},
{-0.0118123,0.0191139,0.0031977},
{-0.0017185,-0.0163303,0.0664216},

Biology Open • Supplementary information

{-0.0085098,-0.0074564,0.0247745},
{0.0197459,-0.0086852,0.0430460},
{0.0187064,-0.0223532,0.0600966},
{-0.0206552,-0.0099712,0.0525024},
{0.0207525,0.0084646,-0.0024846},
{-0.0094915,-0.0325446,0.0184441},
{-0.0014464,-0.0140050,-0.0179819},
{-0.0122725,-0.0573444,0.0463125},
{-0.0106397,0.0177211,0.0094669},
{0.0111981,0.0191236,-0.0041491},
{-0.0162790,-0.0256279,0.0592687},
{-0.0060120,-0.3653959,0.0413411},
{-0.0135007,-0.3703638,-0.0387476},
{-0.0134258,-0.3703142,-0.0379467},
{-0.0133509,-0.3702645,-0.0371458},
{-0.0132760,-0.3702148,-0.0363450},
{-0.0132011,-0.3701651,-0.0355441},
{-0.0131262,-0.3701154,-0.0347432},
{-0.0130513,-0.3700658,-0.0339423},
{-0.0129765,-0.3700161,-0.0331414},
{-0.0129016,-0.3699664,-0.0323405},
{-0.0128267,-0.3699167,-0.0315396},
{-0.0127518,-0.3698670,-0.0307388},
{-0.0126769,-0.3698174,-0.0299379},
{-0.0126020,-0.3697677,-0.0291370},
{-0.0125271,-0.3697180,-0.0283361},
{-0.0124523,-0.3696683,-0.0275352},
{-0.0123774,-0.3696187,-0.0267343},
{-0.0123025,-0.3695690,-0.0259334},
{-0.0122276,-0.3695193,-0.0251325},
{-0.0121527,-0.3694696,-0.0243317},
{-0.0120778,-0.3694199,-0.0235308},
{-0.0120029,-0.3693703,-0.0227299},
{-0.0119280,-0.3693206,-0.0219290},
{-0.0118532,-0.3692709,-0.0211281},
{-0.0117783,-0.3692212,-0.0203272},
{-0.0117034,-0.3691715,-0.0195263},
{-0.0116285,-0.3691219,-0.0187255},
{-0.0115536,-0.3690722,-0.0179246},
{-0.0114787,-0.3690225,-0.0171237},
{-0.0114038,-0.3689728,-0.0163228},
{-0.0113289,-0.3689231,-0.0155219},
{-0.0112541,-0.3688735,-0.0147210},
{-0.0111792,-0.3688238,-0.0139201},
{-0.0111043,-0.3687741,-0.0131192},
{-0.0110294,-0.3687244,-0.0123184},

Biology Open • Supplementary information

{-0.0109545,-0.3686748,-0.0115175},
{-0.0108796,-0.3686251,-0.0107166},
{-0.0108047,-0.3685754,-0.0099157},
{-0.0107298,-0.3685257,-0.0091148},
{-0.0106550,-0.3684760,-0.0083139},
{-0.0105801,-0.3684264,-0.0075130},
{-0.0105052,-0.3683767,-0.0067121},
{-0.0104303,-0.3683270,-0.0059113},
{-0.0103554,-0.3682773,-0.0051104},
{-0.0102805,-0.3682276,-0.0043095},
{-0.0102056,-0.3681780,-0.0035086},
{-0.0101307,-0.3681283,-0.0027077},
{-0.0100559,-0.3680786,-0.0019068},
{-0.0099810,-0.3680289,-0.0011059},
{-0.0099061,-0.3679792,-0.0003051},
{-0.0098312,-0.3679296,0.0004958},
{-0.0097563,-0.3678799,0.0012967},
{-0.0096814,-0.3678302,0.0020976},
{-0.0096065,-0.3677805,0.0028985},
{-0.0095317,-0.3677308,0.0036994},
{-0.0094568,-0.3676812,0.0045003},
{-0.0093819,-0.3676315,0.0053012},
{-0.0093070,-0.3675818,0.0061020},
{-0.0092321,-0.3675321,0.0069029},
{-0.0091572,-0.3674825,0.0077038},
{-0.0090823,-0.3674328,0.0085047},
{-0.0090074,-0.3673831,0.0093056},
{-0.0089326,-0.3673334,0.0101065},
{-0.0088577,-0.3672837,0.0109074},
{-0.0087828,-0.3672341,0.0117082},
{-0.0087079,-0.3671844,0.0125091},
{-0.0086330,-0.3671347,0.0133100},
{-0.0085581,-0.3670850,0.0141109},
{-0.0084832,-0.3670353,0.0149118},
{-0.0084083,-0.3669857,0.0157127},
{-0.0083335,-0.3669360,0.0165136},
{-0.0082586,-0.3668863,0.0173145},
{-0.0081837,-0.3668366,0.0181153},
{-0.0081088,-0.3667869,0.0189162},
{-0.0080339,-0.3667373,0.0197171},
{-0.0079590,-0.3666876,0.0205180},
{-0.0078841,-0.3666379,0.0213189},
{-0.0078092,-0.3665882,0.0221198},
{-0.0077344,-0.3665386,0.0229207},
{-0.0076595,-0.3664889,0.0237215},
{-0.0075846,-0.3664392,0.0245224},

Biology Open • Supplementary information

```
{-0.0075097,-0.3663895,0.0253233},
{-0.0074348,-0.3663398,0.0261242},
{-0.0073599,-0.3662902,0.0269251},
{-0.0072850,-0.3662405,0.0277260},
{-0.0072101,-0.3661908,0.0285269},
{-0.0071353,-0.3661411,0.0293278},
{-0.0070604,-0.3660914,0.0301286},
{-0.0069855,-0.3660418,0.0309295},
{-0.0069106,-0.3659921,0.0317304},
{-0.0068357,-0.3659424,0.0325313},
{-0.0067608,-0.3658927,0.0333322},
{-0.0066859,-0.3658430,0.0341331},
{-0.0066111,-0.3657934,0.0349340},
{-0.0065362,-0.3657437,0.0357348},
{-0.0064613,-0.3656940,0.0365357},
{-0.0063864,-0.3656443,0.0373366},
{-0.0063115,-0.3655947,0.0381375},
{-0.0062366,-0.3655450,0.0389384},
{-0.0061617,-0.3654953,0.0397393},
{-0.0060868,-0.3654456,0.0405402}
}* .AMirroring);
Points1 = ({
{0.0000000,0.0000000,0.0000000},
{-0.0000000,-0.4956311,0.0000000},
{-0.0105515,-0.5041242,0.0014024},
{-0.0000000,-0.5016347,0.0441474},
{-0.0000000,-0.4896274,-0.0441474},
{0.0157077,-0.0111381,0.0633292},
{0.0227918,-0.0289680,0.0498430},
{0.0109539,-0.0328570,0.0690871},
{0.0001770,-0.0005124,0.0563724},
{0.0127207,-0.0088102,0.0439317},
{-0.0067059,-0.0180443,0.0716517},
{0.0190772,-0.0498739,0.0570900},
{0.0170923,-0.0433063,0.0366848},
{0.0161448,-0.0186469,0.0277541},
{-0.0114878,-0.0414163,0.0683670},
{0.0038056,-0.0542163,0.0672390},
{-0.0034841,-0.0259612,0.0456049},
{-0.0141427,-0.0055717,0.0531140},
{-0.0000101,-0.0054655,0.0308163},
{-0.0138874,-0.0295643,0.0545992},
{0.0114097,-0.0784676,0.0599793},
{0.0181452,-0.0697096,0.0427296},
{0.0094589,-0.0588622,0.0214259},
{0.0109389,-0.0313482,0.0139777},
```

Biology Open • Supplementary information

{0.0207282,-0.0071122,0.0063892},
{0.0117294,0.0078414,0.0178309},
{-0.0138154,-0.0493146,0.0406325},
{-0.0113493,-0.0641063,0.0577304},
{-0.0104190,-0.0329994,0.0278931},
{-0.0069175,0.0138036,0.0178635},
{-0.0128376,-0.0075941,0.0176091},
{-0.0084019,-0.0906589,0.0500646},
{0.0071099,-0.1040291,0.0581587},
{0.0193225,-0.0975167,0.0412191},
{0.0093778,-0.0839777,0.0235426},
{-0.0132745,-0.0596273,0.0231372},
{-0.0047370,-0.0423843,0.0114320},
{-0.0005011,-0.0257247,-0.0053819},
{0.0144355,-0.0201618,-0.0073073},
{0.0195053,-0.0023177,-0.0124793},
{0.0191251,0.0167924,-0.0013585},
{0.0035349,0.0254835,0.0100915},
{-0.0160511,-0.0740186,0.0379657},
{-0.0133355,-0.0161742,0.0064984},
{-0.0136495,0.0219721,0.0018022},
{-0.0208223,0.0030461,0.0035557},
{-0.0076608,-0.1004177,0.0310904},
{-0.0048478,-0.1204022,0.0471425},
{0.0164489,-0.1264508,0.0502504},
{0.0140413,-0.1080394,0.0244112},
{-0.0113980,-0.0802019,0.0240324},
{-0.0163555,-0.0114465,-0.0114638},
{0.0007406,-0.0118213,-0.0209511},
{0.0067275,0.0100534,-0.0192847},
{0.0018929,0.0260052,-0.0091112},
{-0.0118404,0.0108797,-0.0163024},
{0.0004985,-0.1266681,0.0261460},
{-0.0020940,-0.1440091,0.0449523},
{0.0177144,-0.1524745,0.0479420},
{0.0211295,-0.1310793,0.0295349},
{0.0070258,-0.1488262,0.0216873},
{0.0000155,-0.1681540,0.0393630},
{0.0189608,-0.1785428,0.0454658},
{0.0252177,-0.1591008,0.0289949},
{0.0109303,-0.1732364,0.0185287},
{0.0026304,-0.1930517,0.0361459},
{0.0215753,-0.2048977,0.0424111},
{0.0278482,-0.1861811,0.0260647},
{0.0129090,-0.1996153,0.0158101},
{0.0043258,-0.2179210,0.0325267},

Biology Open • Supplementary information

{0.0219151,-0.2315468,0.0398121},
{0.0295971,-0.2108044,0.0236158},
{0.0144619,-0.2252977,0.0133784},
{0.0029735,-0.2435960,0.0240714},
{0.0157713,-0.2573466,0.0355232},
{0.0310553,-0.2546717,0.0270406},
{0.0299889,-0.2346198,0.0189322},
{0.0180708,-0.2544391,0.0099553},
{0.0026476,-0.2697261,0.0205535},
{0.0124920,-0.2887214,0.0310101},
{0.0278871,-0.2790577,0.0320109},
{0.0302746,-0.2762983,0.0146254},
{0.0159596,-0.2849731,0.0077174},
{0.0022355,-0.3006016,0.0174315},
{0.0136804,-0.3177982,0.0297423},
{0.0287882,-0.3039867,0.0285883},
{0.0301255,-0.3053858,0.0111768},
{0.0143606,-0.3153809,0.0050377},
{0.0032499,-0.3297316,0.0166105},
{0.0110579,-0.3471124,0.0272136},
{0.0292601,-0.3327193,0.0252378},
{0.0291765,-0.3316432,0.0068321},
{0.0112801,-0.3462646,0.0027021},
{0.0028971,-0.3652682,0.0119085},
{0.0123365,-0.3746523,0.0268973},
{0.0287900,-0.3610570,0.0232626},
{0.0294099,-0.3551392,0.0044700},
{0.0171901,-0.3715528,-0.0031369},
{0.0043542,-0.3921637,0.0008470},
{0.0028225,-0.3951666,0.0202969},
{0.0245504,-0.3945411,0.0241466},
{0.0321315,-0.3836861,0.0091396},
{0.0254541,-0.3997006,-0.0053781},
{0.0071752,-0.4148073,-0.0074211},
{-0.0001866,-0.4199043,0.0101232},
{0.0131607,-0.4172965,0.0262343},
{0.0322174,-0.4152668,0.0123526},
{0.0252061,-0.4258013,-0.0091113},
{0.0050715,-0.4405668,-0.0133978},
{-0.0024173,-0.4474063,0.0046872},
{0.0018213,-0.4388897,0.0235594},
{0.0248305,-0.4380879,0.0232548},
{0.0316187,-0.4423559,0.0055632},
{0.0239706,-0.4516933,-0.0143149},
{0.0108647,-0.4683304,-0.0264493},
{-0.0057330,-0.4625942,-0.0148929},

{-0.0069683,-0.4778830,0.0031013},
{-0.0064183,-0.4669881,0.0219479},
{0.0113698,-0.4612565,0.0285707},
{0.0305747,-0.4640347,0.0195003},
{0.0292452,-0.4702827,-0.0001608},
{0.0277969,-0.4805748,-0.0196663},
{0.0193534,-0.4982748,-0.0327522},
{0.0046666,-0.4856670,-0.0413047},
{-0.0102970,-0.4743441,-0.0319078},
{-0.0177936,-0.4855006,-0.0136990},
{-0.0065859,-0.5056765,-0.0035756},
{-0.0190911,-0.4950644,0.0119083},
{-0.0165806,-0.4860554,0.0324056},
{0.0023067,-0.4807216,0.0379353},
{0.0227863,-0.4862863,0.0302644},
{0.0385648,-0.4875928,0.0172344},
{0.0298202,-0.4957173,-0.0020123},
{0.0322833,-0.5127205,-0.0177065},
{0.0189620,-0.5283111,-0.0300134},
{0.0027691,-0.5125245,-0.0429506},
{-0.0147882,-0.4966170,-0.0434887},
{-0.0300277,-0.4888685,-0.0308183},
{-0.0297896,-0.5069788,-0.0130677},
{-0.0190168,-0.5288668,-0.0192423},
{0.0001700,-0.5285266,-0.0127671},
{0.0032728,-0.5255095,0.0081711},
{-0.0155860,-0.5222249,0.0151739},
{-0.0267896,-0.5071993,0.0308374},
{-0.0048456,-0.5039052,0.0437612},
{0.0144038,-0.5087115,0.0361448},
{0.0351350,-0.5102384,0.0265692},
{0.0293576,-0.5160776,0.0078345},
{0.0181677,-0.5238596,-0.0070675},
{-0.0001572,-0.5359196,-0.0327718},
{-0.0156816,-0.5254133,-0.0415447},
{-0.0325880,-0.5139966,-0.0347527},
{0.0212495,-0.5296133,0.0228039},
{0.0017008,-0.5327967,0.0299122},
{-0.0158594,-0.5272899,0.0364373},
{-0.0009149,-0.0862143,0.0209947},
{-0.0109685,-0.0750513,0.0227671},
{0.0104555,-0.0440262,0.0681348},
{-0.0150861,-0.0178894,0.0628027},
{-0.0018493,-0.0733360,0.0631372},
{-0.0066918,-0.0823546,0.0247709},
{-0.0014988,-0.1058188,0.0226200},

Biology Open • Supplementary information

{-0.0074309,0.0021863,-0.0187435},
{-0.0132474,-0.0575575,0.0453683},
{0.0035127,-0.0343088,0.0722395},
{-0.0108011,-0.0850744,0.0283326},
{-0.0011343,-0.0699215,0.0202311},
{-0.0140070,-0.0219582,0.0559790},
{0.0144733,-0.0388310,0.0662842},
{-0.0111259,-0.0385682,0.0437510},
{0.0151923,-0.0443840,0.0644285},
{-0.0009472,-0.0965920,0.0591783},
{-0.0157546,-0.0565476,0.0344463},
{-0.0148442,-0.0717058,0.0242322},
{-0.0172861,-0.0701331,0.0256326},
{-0.0026370,-0.0532981,0.0149574},
{0.0115144,-0.0747146,0.0263235},
{0.0230681,-0.0170890,0.0435927},
{-0.0033220,-0.0567768,0.0682340},
{0.0144226,-0.0036187,0.0189669},
{-0.0094236,-0.0627280,0.0208299},
{-0.0063527,-0.1042791,0.0488548},
{0.0059418,0.0190433,0.0144489},
{-0.0120438,0.0219102,-0.0066237},
{-0.0075221,-0.0866328,0.0522937},
{0.0083559,-0.1089627,0.0213618},
{0.0137802,-0.0955845,0.0267408},
{0.0082564,-0.0606791,0.0647079},
{0.0153911,-0.0162183,0.0083518},
{-0.0061598,-0.0302411,0.0050371},
{-0.0076663,-0.0012474,0.0220920},
{-0.0107706,-0.0722249,0.0538100},
{-0.0066063,-0.0336032,0.0723083},
{0.0089727,-0.0081593,0.0660412},
{0.0145133,-0.0648650,0.0325811},
{-0.0021408,-0.0232723,-0.0132542},
{-0.0147033,-0.0313853,0.0600410},
{0.0170852,0.0170754,-0.0077138},
{-0.0043285,0.0276707,0.0040187},
{0.0006734,-0.1091233,0.0573984},
{-0.0138556,-0.0507322,0.0543581},
{0.0211834,-0.0358743,0.0461573},
{0.0123570,-0.0550680,0.0262402},
{0.0190125,0.0133287,0.0078535},
{0.0219112,0.0026694,0.0073356},
{0.0229365,-0.0025406,-0.0037129},
{0.0146924,-0.0258005,0.0244788},
{0.0121957,-0.0444110,0.0233717},

{0.0173085,-0.0188283,0.0350179},
{-0.0185093,-0.0628157,0.0287859},
{0.0032027,-0.0491044,0.0700966},
{0.0179069,-0.0578371,0.0417155},
{0.0154779,-0.0129711,0.0144071},
{0.0010931,0.0056250,-0.0210834},
{0.0092374,-0.0009773,-0.0209231},
{0.0110637,-0.0863127,0.0582883},
{0.0158010,-0.0779425,0.0553717},
{0.0185593,-0.0757284,0.0460421},
{0.0187716,-0.0876677,0.0389373},
{-0.0049990,-0.0956514,0.0266475},
{-0.0140444,0.0047758,0.0151509},
{0.0081722,-0.0014350,0.0598295},
{0.0203332,-0.0119559,0.0545361},
{0.0158527,-0.0302184,0.0652561},
{-0.0126074,-0.0456860,0.0274599},
{-0.0194332,-0.0095678,-0.0027686},
{0.0172905,-0.1056261,0.0296806},
{-0.0073807,-0.0073296,-0.0205941},
{0.0043518,0.0179895,-0.0160447},
{-0.0098812,-0.0421650,0.0173243},
{-0.0157280,0.0131569,-0.0112730},
{-0.0213468,0.0063273,-0.0017991},
{-0.0096483,-0.0893744,0.0428186},
{0.0123563,-0.0222095,-0.0010272},
{0.0132986,-0.0579206,0.0621292},
{0.0092318,-0.0235482,-0.0097323},
{0.0106986,-0.0177346,-0.0149163},
{0.0045812,-0.0756941,0.0205166},
{0.0095386,-0.0645711,0.0232457},
{0.0013036,-0.0968017,0.0217334},
{-0.0194058,-0.0714213,0.0326957},
{-0.0064514,-0.0017313,0.0564904},
{-0.0139889,-0.0391925,0.0644354},
{0.0191565,-0.0220843,0.0586151},
{0.0160273,-0.0979463,0.0522735},
{0.0200785,-0.0305499,0.0379942},
{0.0089325,-0.0405323,0.0720652},
{-0.0181927,-0.0041355,0.0107584},
{0.0154274,-0.0205407,0.0652223},
{0.0165691,-0.0779733,0.0353346},
{-0.0162746,-0.0638251,0.0244114},
{-0.0150479,-0.0424728,0.0570228},
{0.0148793,-0.0529995,0.0331312},
{-0.0083736,-0.0493134,0.0697723},

Biology Open • Supplementary information

{0.0071107,-0.0098570,-0.0204330},
{0.0044338,-0.0196407,-0.0164317},
{-0.0154325,-0.0137329,0.0558168},
{-0.0111942,-0.0570043,0.0628209},
{0.0187040,-0.0688376,0.0512556},
{-0.0181112,0.0142224,0.0017009},
{-0.0148158,0.0116808,0.0126371},
{-0.0020432,-0.0866170,0.0594529},
{0.0142519,-0.0118303,0.0369975},
{0.0148994,-0.0044634,0.0598061},
{0.0163855,-0.0073162,0.0468677},
{-0.0137303,-0.0171410,-0.0093368},
{0.0061537,-0.0279773,0.0025546},
{0.0003965,-0.0624883,0.0185068},
{0.0215209,0.0039893,-0.0082488},
{0.0184462,0.0020698,-0.0143030},
{0.0137571,-0.0098312,-0.0171546},
{-0.0141188,0.0017494,-0.0167836},
{-0.0157653,-0.0076186,-0.0143816},
{0.0190303,0.0168655,0.0034836},
{0.0113710,0.0243188,-0.0024935},
{0.0097061,0.0176916,-0.0143733},
{0.0086732,-0.0288014,0.0701360},
{-0.0007647,-0.0110019,0.0691751},
{-0.0001784,-0.0026662,0.0623420},
{0.0057915,-0.0174888,0.0707662},
{0.0090432,0.0093698,0.0179997},
{0.0132729,-0.0221861,0.0170686},
{0.0042424,-0.0322320,0.0719592},
{-0.0098291,-0.0220308,0.0265815},
{-0.0110164,-0.0222662,0.0132693},
{-0.0069824,-0.0127517,0.0296816},
{-0.0162545,-0.0646723,0.0388299},
{-0.0122470,-0.0781818,0.0449450},
{-0.0074753,-0.0371083,0.0102747},
{0.0223435,-0.0235978,0.0507226},
{0.0132029,-0.0107955,0.0303771},
{0.0046655,-0.0012069,0.0266135},
{-0.0038686,-0.0969960,0.0578663},
{-0.0025960,0.0279888,-0.0035860},
{-0.0012338,-0.0168674,0.0429097},
{-0.0118052,-0.0183310,0.0027018},
{0.0139939,-0.0367876,0.0281245},
{0.0111506,-0.0346962,0.0187921},
{-0.0118813,-0.0088788,0.0577614},
{0.0202291,-0.0423344,0.0526139},

Biology Open • Supplementary information

{-0.0130373,-0.0684151,0.0455748},
{-0.0080684,-0.1034050,0.0413178},
{0.0046795,-0.0067088,0.0338391},
{-0.0032275,-0.0058885,-0.0218984},
{-0.0136221,-0.0444293,0.0639519},
{-0.0032507,0.0113817,-0.0185123},
{-0.0046677,0.0212199,-0.0136713},
{0.0201016,-0.1097649,0.0398332},
{0.0039887,-0.0946598,0.0208753},
{0.0177812,-0.1132514,0.0489680},
{0.0103564,0.0245542,0.0059017},
{0.0126858,0.0117053,0.0159867},
{0.0011868,0.0192862,-0.0156886},
{0.0173657,-0.0149559,-0.0097037},
{0.0204508,-0.0097826,-0.0083215},
{-0.0093966,-0.0311738,0.0333711},
{-0.0117768,-0.0443300,0.0352746},
{-0.0144754,-0.0791794,0.0282177},
{-0.0015291,0.0034159,0.0220388},
{0.0044859,0.0093991,0.0190134},
{-0.0027167,-0.0036621,0.0281551},
{0.0002772,-0.0395648,0.0714133},
{0.0074502,-0.0557739,0.0657226},
{-0.0126177,-0.0255018,0.0688466},
{-0.0125885,-0.0338190,0.0680024},
{0.0097466,0.0260225,0.0029054},
{0.0184366,0.0181765,0.0015416},
{-0.0076815,-0.0140231,0.0700956},
{0.0164128,-0.0683035,0.0377133},
{-0.0133762,-0.0611170,0.0527210},
{-0.0161782,-0.0648141,0.0241493},
{0.0118100,-0.1083383,0.0555253},
{-0.0105586,-0.0324374,0.0194280},
{-0.0108832,-0.0197529,0.0186801},
{-0.0047687,0.0205542,0.0138631},
{0.0009635,-0.0875805,0.0613848},
{0.0063426,-0.0807452,0.0615735},
{-0.0024325,-0.0803448,0.0610194},
{0.0178496,-0.0946778,0.0340826},
{-0.0134144,-0.0235066,0.0537114},
{0.0114902,-0.0319387,0.0189111},
{0.0055501,-0.0674527,0.0642824},
{0.0089425,-0.0450797,0.0683440},
{0.0102718,-0.1082757,0.0217767},
{-0.0072985,-0.0679406,0.0624138},
{0.0193015,-0.0549711,0.0482392},

Biology Open • Supplementary information

{-0.0128326,-0.0549485,0.0232015},
{0.0228116,-0.0264671,0.0410375},
{-0.0043374,-0.0782265,0.0222569},
{0.0179219,-0.0025546,0.0139998},
{0.0066445,0.0069310,0.0191771},
{0.0009859,-0.0415952,0.0712842},
{-0.0159561,0.0175922,-0.0055616},
{0.0114181,0.0200947,0.0111374},
{-0.0114867,0.0205028,0.0089365},
{-0.0200307,-0.0070363,-0.0056585},
{-0.0201501,0.0006508,-0.0081168},
{-0.0052754,-0.0223864,0.0727297},
{-0.0141540,-0.0439267,0.0476863},
{0.0060138,-0.0599376,0.0656916},
{0.0133850,-0.0532319,0.0632333},
{-0.0094092,-0.0873197,0.0278108},
{0.0135972,0.0090607,0.0164632},
{0.0196264,-0.0136695,-0.0012290},
{-0.0186484,-0.0102705,0.0054782},
{-0.0137518,-0.0718465,0.0422572},
{-0.0078720,-0.0215041,-0.0040670},
{-0.0139249,-0.0351654,0.0511134},
{0.0050087,-0.0955410,0.0596882},
{0.0171957,-0.0636853,0.0562709},
{-0.0018680,-0.0444610,0.0119652},
{0.0067254,-0.0408292,0.0142620},
{0.0202096,-0.0088885,0.0062717},
{0.0195139,-0.1038502,0.0452464},
{-0.0098318,-0.0241386,0.0272405},
{-0.0009461,0.0165201,0.0174337},
{0.0079707,-0.0870168,0.0229616},
{0.0094671,-0.0795175,0.0608747},
{0.0161704,-0.0165265,-0.0102191},
{0.0005985,-0.0116018,0.0696906},
{-0.0076976,-0.0187946,-0.0153220},
{0.0171116,-0.0898354,0.0508276},
{0.0082767,-0.0234663,-0.0111835},
{-0.0114267,-0.0821388,0.0251080},
{0.0143299,-0.0500931,0.0313752},
{0.0167030,-0.0054491,0.0582343},
{0.0051067,-0.0097299,0.0424486},
{-0.0032682,-0.0786616,0.0616219},
{-0.0137817,-0.0758180,0.0411519},
{-0.0155581,-0.0145477,0.0582731},
{-0.0084597,0.0156202,-0.0157983},
{-0.0184801,0.0073657,0.0087878},

Biology Open • Supplementary information

{0.0100615,-0.0866749,0.0239382},
{0.0026842,-0.0494810,0.0691125},
{-0.0145335,-0.0275818,0.0592792},
{0.0069311,-0.0078011,-0.0211891},
{0.0165801,-0.0359251,0.0609343},
{0.0128759,-0.0190354,0.0668639},
{0.0112144,0.0026285,-0.0195168},
{0.0147522,-0.0102886,0.0185344},
{-0.0139264,0.0140301,0.0126057},
{0.0111485,-0.0665659,0.0620682},
{-0.0058822,-0.0483400,0.0706606},
{0.0209239,-0.0365782,0.0539652},
{0.0170541,-0.0166498,-0.0075588},
{-0.0160579,-0.0631608,0.0393704},
{-0.0081266,-0.0927392,0.0488112},
{0.0034431,-0.0661825,0.0650795},
{-0.0047506,-0.0520758,0.0151359},
{0.0119845,0.0134394,-0.0157804},
{-0.0023064,-0.0271600,-0.0009118},
{-0.0098655,-0.0921214,0.0326456},
{-0.0111151,-0.0044004,-0.0189783},
{-0.0031382,0.0272286,-0.0050486},
{-0.0103178,-0.0272784,0.0143311},
{-0.0115594,-0.0809236,0.0249163},
{-0.0063999,-0.0938560,0.0268258},
{0.0010258,0.0148891,-0.0180532},
{0.0213578,-0.0326332,0.0531351},
{-0.0063450,-0.0728281,0.0215260},
{0.0189760,-0.0455848,0.0429423},
{0.0189485,-0.0386411,0.0382592},
{-0.0154563,-0.0150130,-0.0010603},
{0.0142855,0.0175906,0.0109800},
{-0.0144109,-0.0136922,0.0075414},
{-0.0115164,-0.0441624,0.0389289},
{0.0193335,-0.1100742,0.0462104},
{0.0190358,-0.0129612,0.0606599},
{0.0202050,-0.0195156,0.0561969},
{0.0014663,-0.0332045,0.0054702},
{0.0116203,-0.0292805,0.0684369},
{-0.0030041,-0.0119567,0.0342027},
{-0.0207616,-0.0053178,-0.0018937},
{0.0029700,-0.0263264,-0.0041499},
{-0.0104299,-0.0890926,0.0402546},
{-0.0137189,-0.0344138,0.0663591},
{0.0192686,0.0064975,-0.0117229},
{-0.0159073,-0.0779468,0.0328122},

Biology Open • Supplementary information

{-0.0164048,-0.0052561,0.0139331},
{0.0129177,-0.0100588,0.0294635},
{-0.0144644,-0.0536188,0.0348986},
{0.0162058,-0.0895463,0.0313522},
{0.0008286,0.0042153,-0.0213586},
{0.0181812,-0.0969402,0.0342089},
{0.0074764,0.0079809,0.0183750},
{-0.0153846,0.0028261,0.0141084},
{0.0063007,-0.0536709,0.0180864},
{-0.0069736,-0.0237891,0.0370683},
{0.0221325,-0.0337028,0.0468232},
{-0.0138997,-0.0782547,0.0250537},
{0.0224614,0.0063928,-0.0033183},
{-0.0149449,-0.0784620,0.0305272},
{0.0121332,-0.0279607,0.0121704},
{0.0212607,-0.0146144,0.0499511},
{0.0093304,0.0024316,-0.0205487},
{0.0158922,-0.0103787,0.0148958},
{-0.0122173,-0.0794196,0.0442360},
{0.0004991,0.0257305,0.0101421},
{0.0030619,0.0041277,-0.0217087},
{0.0406651,-0.5036634,0.0231980},
{0.0055110,0.0254960,-0.0085951},
{-0.0147251,-0.0562174,0.0411984},
{-0.0342206,-0.4985627,-0.0297253},
{0.0025259,0.0288442,-0.0000353},
{0.0156668,-0.0306457,0.0293778},
{-0.0153888,-0.0013741,-0.0160541},
{0.0180459,-0.0386979,0.0585601},
{-0.0159746,0.0167960,0.0052405},
{0.0188135,-0.0500625,0.0442547},
{0.0167904,-0.0558496,0.0379592},
{-0.0144684,-0.0440530,0.0505882},
{0.0188917,-0.0707384,0.0486875},
{-0.0182351,-0.0614707,0.0303838},
{0.0033317,-0.0702287,0.0642511},
{0.0011856,-0.0655139,0.0191884},
{0.0233416,-0.0266946,0.0486720},
{0.0086179,-0.0975491,0.0582414},
{-0.0064838,-0.1059057,0.0308715},
{-0.0206864,-0.0027485,0.0033745},
{0.0156800,-0.0171113,0.0218149},
{0.0150809,-0.0850253,0.0303540},
{-0.0101240,-0.0315210,0.0304006},
{-0.0212391,-0.5111690,0.0394447},
{-0.0094246,-0.0711873,0.0576415},

{-0.0124707,-0.5042581,-0.0113553},
{0.0116228,-0.0551908,0.0247419},
{0.0118126,0.0185244,-0.0123643},
{-0.0265194,-0.5000972,0.0319165},
{0.0170255,-0.0678023,0.0386866},
{0.0003247,-0.0632243,0.0661160},
{-0.0119512,0.0230839,0.0031416},
{-0.0026848,-0.0213040,0.0725755},
{-0.0079370,-0.0094019,0.0266354},
{0.0210842,-0.0131477,0.0466213},
{0.0161465,-0.0312474,0.0631616},
{-0.0153748,-0.0139155,0.0574647},
{0.0215495,0.0105078,-0.0030995},
{-0.0109000,-0.0435296,0.0197950},
{-0.0003662,-0.0180189,-0.0181506},
{-0.0117158,-0.0768442,0.0487650},
{-0.0097902,0.0218480,0.0088541},
{0.0106128,0.0244224,-0.0033133},
{-0.0145633,-0.0344964,0.0629306},
{-0.0065020,-0.5007203,0.0447106},
{-0.0146011,-0.5075281,-0.0419058},
{-0.0145201,-0.5074600,-0.0410396},
{-0.0144391,-0.5073919,-0.0401735},
{-0.0143581,-0.5073239,-0.0393073},
{-0.0142771,-0.5072558,-0.0384411},
{-0.0141961,-0.5071877,-0.0375750},
{-0.0141151,-0.5071196,-0.0367088},
{-0.0140341,-0.5070515,-0.0358426},
{-0.0139531,-0.5069835,-0.0349765},
{-0.0138721,-0.5069154,-0.0341103},
{-0.0137911,-0.5068473,-0.0332441},
{-0.0137102,-0.5067792,-0.0323780},
{-0.0136292,-0.5067112,-0.0315118},
{-0.0135482,-0.5066431,-0.0306457},
{-0.0134672,-0.5065750,-0.0297795},
{-0.0133862,-0.5065069,-0.0289133},
{-0.0133052,-0.5064388,-0.0280472},
{-0.0132242,-0.5063708,-0.0271810},
{-0.0131432,-0.5063027,-0.0263148},
{-0.0130622,-0.5062346,-0.0254487},
{-0.0129812,-0.5061665,-0.0245825},
{-0.0129002,-0.5060985,-0.0237163},
{-0.0128193,-0.5060304,-0.0228502},
{-0.0127383,-0.5059623,-0.0219840},
{-0.0126573,-0.5058942,-0.0211179},
{-0.0125763,-0.5058261,-0.0202517},

Biology Open • Supplementary information

{-0.0124953,-0.5057581,-0.0193855},
{-0.0124143,-0.5056900,-0.0185194},
{-0.0123333,-0.5056219,-0.0176532},
{-0.0122523,-0.5055538,-0.0167870},
{-0.0121713,-0.5054858,-0.0159209},
{-0.0120903,-0.5054177,-0.0150547},
{-0.0120093,-0.5053496,-0.0141885},
{-0.0119284,-0.5052815,-0.0133224},
{-0.0118474,-0.5052134,-0.0124562},
{-0.0117664,-0.5051454,-0.0115900},
{-0.0116854,-0.5050773,-0.0107239},
{-0.0116044,-0.5050092,-0.0098577},
{-0.0115234,-0.5049411,-0.0089916},
{-0.0114424,-0.5048731,-0.0081254},
{-0.0113614,-0.5048050,-0.0072592},
{-0.0112804,-0.5047369,-0.0063931},
{-0.0111994,-0.5046688,-0.0055269},
{-0.0111184,-0.5046007,-0.0046607},
{-0.0110375,-0.5045327,-0.0037946},
{-0.0109565,-0.5044646,-0.0029284},
{-0.0108755,-0.5043965,-0.0020622},
{-0.0107945,-0.5043284,-0.0011961},
{-0.0107135,-0.5042604,-0.0003299},
{-0.0106325,-0.5041923,0.0005362},
{-0.0105515,-0.5041242,0.0014024},
{-0.0104705,-0.5040561,0.0022686},
{-0.0103895,-0.5039880,0.0031347},
{-0.0103085,-0.5039200,0.0040009},
{-0.0102275,-0.5038519,0.0048671},
{-0.0101466,-0.5037838,0.0057332},
{-0.0100656,-0.5037157,0.0065994},
{-0.0099846,-0.5036477,0.0074656},
{-0.0099036,-0.5035796,0.0083317},
{-0.0098226,-0.5035115,0.0091979},
{-0.0097416,-0.5034434,0.0100640},
{-0.0096606,-0.5033753,0.0109302},
{-0.0095796,-0.5033073,0.0117964},
{-0.0094986,-0.5032392,0.0126625},
{-0.0094176,-0.5031711,0.0135287},
{-0.0093366,-0.5031030,0.0143949},
{-0.0092557,-0.5030350,0.0152610},
{-0.0091747,-0.5029669,0.0161272},
{-0.0090937,-0.5028988,0.0169934},
{-0.0090127,-0.5028307,0.0178595},
{-0.0089317,-0.5027626,0.0187257},
{-0.0088507,-0.5026946,0.0195918},

Biology Open • Supplementary information

```
                    {-0.0087697,-0.5026265,0.0204580},
                    {-0.0086887,-0.5025584,0.0213242},
                    {-0.0086077,-0.5024903,0.0221903},
                    {-0.0085267,-0.5024223,0.0230565},
                    {-0.0084457,-0.5023542,0.0239227},
                    {-0.0083648,-0.5022861,0.0247888},
                    {-0.0082838,-0.5022180,0.0256550},
                    {-0.0082028,-0.5021500,0.0265212},
                    {-0.0081218,-0.5020819,0.0273873},
                    {-0.0080408,-0.5020138,0.0282535},
                    {-0.0079598,-0.5019457,0.0291196},
                    {-0.0078788,-0.5018776,0.0299858},
                    {-0.0077978,-0.5018096,0.0308520},
                    {-0.0077168,-0.5017415,0.0317181},
                    {-0.0076358,-0.5016734,0.0325843},
                    {-0.0075548,-0.5016053,0.0334505},
                    {-0.0074739,-0.5015373,0.0343166},
                    {-0.0073929,-0.5014692,0.0351828},
                    {-0.0073119,-0.5014011,0.0360490},
                    {-0.0072309,-0.5013330,0.0369151},
                    {-0.0071499,-0.5012649,0.0377813},
                    {-0.0070689,-0.5011969,0.0386474},
                    {-0.0069879,-0.5011288,0.0395136},
                    {-0.0069069,-0.5010607,0.0403798},
                    {-0.0068259,-0.5009926,0.0412459},
                    {-0.0067449,-0.5009246,0.0421121},
                    {-0.0066639,-0.5008565,0.0429783},
                    {-0.0065830,-0.5007884,0.0438444}
                    }* .AMirroring);
                BoundingBoxOnOff = Off;
            };
            AnyFunTransform3DIdentity ScaleFunction = {
                PreTransforms = {&.RBFTransform};
};
};

};
};
```

Biology Open • Supplementary information

ScalingFunctionTLEMLucyPelvis_2014051

```
AnyFolder ScalingFunctionTLEMLucyPelvis = {
AnyFolder Pelvis = {
	AnyFunTransform3DRBF RBFTransform = {
		RBFDef.Type = RBF_ThinPlate;
		PolynomDegree = 1;
		Points0 = {
			{0.0000000,0.0000000,0.1177000},
			{-0.0000000,-0.0832729,0.0191000},
			{-0.0508179,-0.0694062,0.0815920},
			{0.0000000,0.0000000,-0.1177000},
			{-0.0000000,-0.0832729,-0.0191000},
			{-0.0508179,-0.0694062,-0.0815920},
			{0.0000000,0.0000000,0.0000000},
			{-0.1164020,-0.0039449,0.0456760},
			{-0.1164020,-0.0039449,-0.0456760},
			{-0.1092909,-0.0992949,0.0515590},
			{-0.1092909,-0.0992949,-0.0515590},
			{-0.0733749,0.0757445,0.0915590},
			{-0.0733749,0.0757445,-0.0915590},
			{-0.1241738,0.0316243,0.0458050},
			{-0.1241738,0.0316243,-0.0458050},
			{-0.1108008,0.0563490,0.0555690},
			{-0.1108008,0.0563490,-0.0555690},
			{-0.0545356,0.0481653,0.1291690},
			{-0.0545356,0.0481653,-0.1291690},
			{-0.0360236,0.0456352,0.1316250},
			{-0.0360236,0.0456352,-0.1316250},
			{-0.0804664,-0.0228481,0.0672140},
			{-0.0804664,-0.0228481,-0.0672140},
			{-0.1004070,-0.0623213,0.0494990},
			{-0.1004070,-0.0623213,-0.0494990},
			{-0.0174901,-0.0357786,0.0977400},
			{-0.0174901,-0.0357786,-0.0977400},
			{-0.0665950,-0.1308418,0.0278190},
			{-0.0665950,-0.1308418,-0.0278190},
			{-0.0962739,-0.1257274,0.0551670},
			{-0.0962739,-0.1257274,-0.0551670},
			{-0.0393340,-0.1196098,0.0159820},
			{-0.0393340,-0.1196098,-0.0159820},
			{-0.0585979,-0.0148191,0.0651020},
			{-0.0585979,-0.0148191,-0.0651020},
			{-0.0238420,-0.0800479,0.0351680},
			{-0.0238420,-0.0800479,-0.0351680},
			{-0.0234553,-0.0576924,0.0715330},
```

Biology Open • Supplementary information

{-0.0234553,-0.0576924,-0.0715330},
{-0.0472319,-0.0833276,0.0513790},
{-0.0472319,-0.0833276,-0.0513790},
{-0.0327175,-0.0506481,0.0520240},
{-0.0327175,-0.0506481,-0.0520240},
{-0.0746024,-0.0627219,0.0928110},
{-0.0746024,-0.0627219,-0.0928110},
{-0.0119676,-0.0928080,0.0076900},
{-0.0119676,-0.0928080,-0.0076900},
{-0.0263299,-0.0760728,0.0667080},
{-0.0263299,-0.0760728,-0.0667080},
{-0.0498842,0.0119450,0.1070630},
{-0.0498842,0.0119450,-0.1070630},
{-0.0553874,0.0627474,0.1172390},
{-0.0553874,0.0627474,-0.1172390},
{-0.0786774,0.0073556,0.0741800},
{-0.0786774,0.0073556,-0.0741800},
{-0.0742762,0.0474272,0.0542330},
{-0.0742762,0.0474272,-0.0542330},
{-0.0634121,0.0117185,0.0618430},
{-0.0634121,0.0117185,-0.0618430},
{-0.0813084,0.0007821,0.0508130},
{-0.0813084,0.0007821,-0.0508130},
{-0.0496423,-0.0444458,0.0983920},
{-0.0496423,-0.0444458,-0.0983920},
{-0.0480005,-0.0338999,0.0625080},
{-0.0480005,-0.0338999,-0.0625080},
{-0.0428000,-0.0273188,0.0973490},
{-0.0428000,-0.0273188,-0.0973490},
{-0.0548383,-0.0518766,0.0624050},
{-0.0548383,-0.0518766,-0.0624050},
{-0.0696448,-0.1052827,0.0436200},
{-0.0696448,-0.1052827,-0.0436200},
{-0.0739608,-0.0803177,0.0785950},
{-0.0739608,-0.0803177,-0.0785950},
{-0.0586066,-0.0884764,0.0653190},
{-0.0586066,-0.0884764,-0.0653190},
{-0.1017003,0.0085215,0.0624150},
{-0.1017003,0.0085215,-0.0624150},
{-0.0126252,0.0252852,0.1292530},
{-0.0126252,0.0252852,-0.1292530},
{-0.0030963,0.0150156,0.1262640},
{-0.0030963,0.0150156,-0.1262640},
{-0.0621797,0.0048743,0.0605920},
{-0.0621797,0.0048743,-0.0605920},
{-0.0870082,-0.0684280,0.0489900},

Biology Open • Supplementary information

{-0.0870082,-0.0684280,-0.0489900},
{-0.0916211,-0.0478344,0.0575290},
{-0.0916211,-0.0478344,-0.0575290},
{-0.0428573,-0.0467026,0.0985250},
{-0.0428573,-0.0467026,-0.0985250},
{-0.0483869,-0.0455474,0.0848580},
{-0.0483869,-0.0455474,-0.0848580},
{-0.0203929,-0.0681811,0.0426070},
{-0.0203929,-0.0681811,-0.0426070},
{-0.0271973,-0.0807786,0.0491650},
{-0.0271973,-0.0807786,-0.0491650},
{-0.0325935,-0.1026813,0.0148280},
{-0.0325935,-0.1026813,-0.0148280},
{-0.0273900,-0.1059173,0.0048750},
{-0.0273900,-0.1059173,-0.0048750},
{-0.0015971,-0.0778770,0.0065530},
{-0.0015971,-0.0778770,-0.0065530},
{-0.1034983,-0.0140026,0.0521800},
{-0.1034983,-0.0140026,-0.0521800},
{-0.1141160,0.0077734,0.0497600},
{-0.1141160,0.0077734,-0.0497600},
{-0.1123797,0.0244808,0.0350160},
{-0.1123797,0.0244808,-0.0350160},
{-0.0984470,-0.0648210,0.0457100},
{-0.0984470,-0.0648210,-0.0457100},
{-0.0814332,-0.0704479,0.0806410},
{-0.0814332,-0.0704479,-0.0806410},
{-0.0540218,-0.0805070,0.0547320},
{-0.0540218,-0.0805070,-0.0547320},
{-0.0674356,-0.0763171,0.0651080},
{-0.0674356,-0.0763171,-0.0651080},
{-0.0602364,-0.0751827,0.0490600},
{-0.0602364,-0.0751827,-0.0490600},
{-0.0604272,-0.0801096,0.0617610},
{-0.0604272,-0.0801096,-0.0617610},
{-0.0655251,0.0632155,0.1093750},
{-0.0655251,0.0632155,-0.1093750},
{-0.0665046,0.0014289,0.0572170},
{-0.0665046,0.0014289,-0.0572170},
{-0.1053786,0.0347803,0.0071580},
{-0.1053786,0.0347803,-0.0071580},
{-0.1321649,-0.0069725,0.0058340},
{-0.1321649,-0.0069725,-0.0058340},
{-0.1232735,-0.0434903,0.0301110},
{-0.1232735,-0.0434903,-0.0301110},
{-0.0497024,0.0261862,0.0084640},

```
        {-0.0497024,0.0261862,-0.0084640},
        {-0.0887827,0.0446048,0.0327030},
        {-0.0887827,0.0446048,-0.0327030},
        {-0.1153998,-0.0126010,0.0290730},
        {-0.1153998,-0.0126010,-0.0290730},
        {-0.0899939,0.0093882,0.0208440},
        {-0.0899939,0.0093882,-0.0208440},
        {-0.1312654,-0.0574898,0.0094690},
        {-0.1312654,-0.0574898,-0.0094690},
        {-0.0996029,0.0042806,0.0105620},
        {-0.0996029,0.0042806,-0.0105620},
        {-0.1226177,-0.0324069,0.0331630},
        {-0.1226177,-0.0324069,-0.0331630},
        {-0.1097808,-0.0051619,0.0102520},
        {-0.1097808,-0.0051619,-0.0102520}
        };
Points1 = {
        {0.0000000,0.0000000,0.1330193},
        {-0.0000008,-0.0976427,0.0193694},
        {-0.0333557,-0.0758221,0.0925669},
        {0.0000000,0.0000000,-0.1330193},
        {-0.0000008,-0.0976427,-0.0193694},
        {-0.0333557,-0.0758221,-0.0925669},
        {0.0000000,0.0000000,0.0000000},
        {-0.0910766,-0.0318866,0.0450582},
        {-0.0910766,-0.0318866,-0.0450582},
        {-0.0863264,-0.1072362,0.0657880},
        {-0.0863264,-0.1072362,-0.0657880},
        {-0.0592660,0.0448458,0.0970154},
        {-0.0592660,0.0448458,-0.0970154},
        {-0.0961502,-0.0079885,0.0399487},
        {-0.0961502,-0.0079885,-0.0399487},
        {-0.0872815,0.0167976,0.0530425},
        {-0.0872815,0.0167976,-0.0530425},
        {-0.0365883,0.0344214,0.1377392},
        {-0.0365883,0.0344214,-0.1377392},
        {-0.0238439,0.0347690,0.1422609},
        {-0.0238439,0.0347690,-0.1422609},
        {-0.0644579,-0.0432653,0.0730353},
        {-0.0644579,-0.0432653,-0.0730353},
        {-0.0805341,-0.0790901,0.0675612},
        {-0.0805341,-0.0790901,-0.0675612},
        {-0.0120014,-0.0423946,0.0998663},
        {-0.0120014,-0.0423946,-0.0998663},
        {-0.0557792,-0.1350468,0.0363076},
        {-0.0557792,-0.1350468,-0.0363076},
```

{-0.0754573,-0.1251903,0.0691863},
{-0.0754573,-0.1251903,-0.0691863},
{-0.0313819,-0.1294184,0.0202563},
{-0.0313819,-0.1294184,-0.0202563},
{-0.0501545,-0.0308346,0.0705698},
{-0.0501545,-0.0308346,-0.0705698},
{-0.0159461,-0.0890300,0.0365873},
{-0.0159461,-0.0890300,-0.0365873},
{-0.0167808,-0.0632838,0.0784409},
{-0.0167808,-0.0632838,-0.0784409},
{-0.0311551,-0.0861790,0.0601854},
{-0.0311551,-0.0861790,-0.0601854},
{-0.0260807,-0.0635288,0.0610442},
{-0.0260807,-0.0635288,-0.0610442},
{-0.0528840,-0.0711181,0.0973594},
{-0.0528840,-0.0711181,-0.0973594},
{-0.0076291,-0.1083248,0.0083163},
{-0.0076291,-0.1083248,-0.0083163},
{-0.0178315,-0.0762544,0.0714635},
{-0.0178315,-0.0762544,-0.0714635},
{-0.0386267,-0.0010710,0.1132910},
{-0.0386267,-0.0010710,-0.1132910},
{-0.0412276,0.0428065,0.1256664},
{-0.0412276,0.0428065,-0.1256664},
{-0.0654890,-0.0153743,0.0740172},
{-0.0654890,-0.0153743,-0.0740172},
{-0.0627174,0.0151024,0.0533397},
{-0.0627174,0.0151024,-0.0533397},
{-0.0569459,-0.0110381,0.0600156},
{-0.0569459,-0.0110381,-0.0600156},
{-0.0685519,-0.0248859,0.0498292},
{-0.0685519,-0.0248859,-0.0498292},
{-0.0332009,-0.0544807,0.1007153},
{-0.0332009,-0.0544807,-0.1007153},
{-0.0382545,-0.0468930,0.0719751},
{-0.0382545,-0.0468930,-0.0719751},
{-0.0308867,-0.0376574,0.1011094},
{-0.0308867,-0.0376574,-0.1011094},
{-0.0395217,-0.0630430,0.0737494},
{-0.0395217,-0.0630430,-0.0737494},
{-0.0534323,-0.1106268,0.0565377},
{-0.0534323,-0.1106268,-0.0565377},
{-0.0520430,-0.0869514,0.0885587},
{-0.0520430,-0.0869514,-0.0885587},
{-0.0396810,-0.0945948,0.0789515},
{-0.0396810,-0.0945948,-0.0789515},

Biology Open • Supplementary information

{-0.0811472,-0.0186078,0.0613751},
{-0.0811472,-0.0186078,-0.0613751},
{-0.0079398,0.0204920,0.1426233},
{-0.0079398,0.0204920,-0.1426233},
{-0.0014267,0.0129205,0.1408687},
{-0.0014267,0.0129205,-0.1408687},
{-0.0566660,-0.0165558,0.0585450},
{-0.0566660,-0.0165558,-0.0585450},
{-0.0677439,-0.0819713,0.0671116},
{-0.0677439,-0.0819713,-0.0671116},
{-0.0718860,-0.0655776,0.0701725},
{-0.0718860,-0.0655776,-0.0701725},
{-0.0273974,-0.0565075,0.1002288},
{-0.0273974,-0.0565075,-0.1002288},
{-0.0335444,-0.0556960,0.0900750},
{-0.0335444,-0.0556960,-0.0900750},
{-0.0141558,-0.0788382,0.0443289},
{-0.0141558,-0.0788382,-0.0443289},
{-0.0180288,-0.0860285,0.0525233},
{-0.0180288,-0.0860285,-0.0525233},
{-0.0225621,-0.1144412,0.0184548},
{-0.0225621,-0.1144412,-0.0184548},
{-0.0167480,-0.1180470,0.0072093},
{-0.0167480,-0.1180470,-0.0072093},
{-0.0019388,-0.0962613,0.0066900},
{-0.0019388,-0.0962613,-0.0066900},
{-0.0814539,-0.0381532,0.0559967},
{-0.0814539,-0.0381532,-0.0559967},
{-0.0896701,-0.0210344,0.0474725},
{-0.0896701,-0.0210344,-0.0474725},
{-0.0880350,-0.0126367,0.0305221},
{-0.0880350,-0.0126367,-0.0305221},
{-0.0794106,-0.0811295,0.0661352},
{-0.0794106,-0.0811295,-0.0661352},
{-0.0589497,-0.0794563,0.0901338},
{-0.0589497,-0.0794563,-0.0901338},
{-0.0352767,-0.0828845,0.0653558},
{-0.0352767,-0.0828845,-0.0653558},
{-0.0468831,-0.0847571,0.0788284},
{-0.0468831,-0.0847571,-0.0788284},
{-0.0419310,-0.0825286,0.0616026},
{-0.0419310,-0.0825286,-0.0616026},
{-0.0406195,-0.0876769,0.0760407},
{-0.0406195,-0.0876769,-0.0760407},
{-0.0510618,0.0391671,0.1167383},
{-0.0510618,0.0391671,-0.1167383},

```
				{-0.0602560,-0.0207165,0.0543825},
				{-0.0602560,-0.0207165,-0.0543825},
				{-0.0833393,-0.0065496,0.0061338},
				{-0.0833393,-0.0065496,-0.0061338},
				{-0.1018550,-0.0413485,0.0048809},
				{-0.1018550,-0.0413485,-0.0048809},
				{-0.1008481,-0.0715854,0.0229133},
				{-0.1008481,-0.0715854,-0.0229133},
				{-0.0454620,-0.0074836,0.0083370},
				{-0.0454620,-0.0074836,-0.0083370},
				{-0.0729178,0.0067194,0.0302086},
				{-0.0729178,0.0067194,-0.0302086},
				{-0.0903341,-0.0430412,0.0276179},
				{-0.0903341,-0.0430412,-0.0276179},
				{-0.0728361,-0.0248901,0.0195498},
				{-0.0728361,-0.0248901,-0.0195498},
				{-0.1074968,-0.0837605,0.0071614},
				{-0.1074968,-0.0837605,-0.0071614},
				{-0.0775603,-0.0319611,0.0098097},
				{-0.0775603,-0.0319611,-0.0098097},
				{-0.0985056,-0.0609136,0.0291713},
				{-0.0985056,-0.0609136,-0.0291713},
				{-0.0837638,-0.0405539,0.0093994},
				{-0.0837638,-0.0405539,-0.0093994}
				};
			BoundingBoxOnOff = Off;
		};
		AnyFunTransform3DIdentity ScaleFunction = {
			PreTransforms = {&.RBFTransform};
};
};
AnyFolder Sacrum = {
		AnyFunTransform3DRBF RBFTransform = {
			RBFDef.Type = RBF_ThinPlate;
			PolynomDegree = 1;
			Points0 = {
				{0.0000000,0.0000000,0.1177000},
				{-0.0000000,-0.0832729,0.0191000},
				{-0.0508179,-0.0694062,0.0815920},
				{0.0000000,0.0000000,-0.1177000},
				{-0.0000000,-0.0832729,-0.0191000},
				{-0.0508179,-0.0694062,-0.0815920},
				{0.0000000,0.0000000,0.0000000},
				{-0.1164020,-0.0039449,0.0456760},
				{-0.1164020,-0.0039449,-0.0456760},
				{-0.1092909,-0.0992949,0.0515590},
```

Biology Open • Supplementary information

{-0.1092909,-0.0992949,-0.0515590},
{-0.0733749,0.0757445,0.0915590},
{-0.0733749,0.0757445,-0.0915590},
{-0.1241738,0.0316243,0.0458050},
{-0.1241738,0.0316243,-0.0458050},
{-0.1108008,0.0563490,0.0555690},
{-0.1108008,0.0563490,-0.0555690},
{-0.0545356,0.0481653,0.1291690},
{-0.0545356,0.0481653,-0.1291690},
{-0.0360236,0.0456352,0.1316250},
{-0.0360236,0.0456352,-0.1316250},
{-0.0804664,-0.0228481,0.0672140},
{-0.0804664,-0.0228481,-0.0672140},
{-0.1004070,-0.0623213,0.0494990},
{-0.1004070,-0.0623213,-0.0494990},
{-0.0174901,-0.0357786,0.0977400},
{-0.0174901,-0.0357786,-0.0977400},
{-0.0665950,-0.1308418,0.0278190},
{-0.0665950,-0.1308418,-0.0278190},
{-0.0962739,-0.1257274,0.0551670},
{-0.0962739,-0.1257274,-0.0551670},
{-0.0393340,-0.1196098,0.0159820},
{-0.0393340,-0.1196098,-0.0159820},
{-0.0585979,-0.0148191,0.0651020},
{-0.0585979,-0.0148191,-0.0651020},
{-0.0238420,-0.0800479,0.0351680},
{-0.0238420,-0.0800479,-0.0351680},
{-0.0234553,-0.0576924,0.0715330},
{-0.0234553,-0.0576924,-0.0715330},
{-0.0472319,-0.0833276,0.0513790},
{-0.0472319,-0.0833276,-0.0513790},
{-0.0327175,-0.0506481,0.0520240},
{-0.0327175,-0.0506481,-0.0520240},
{-0.0746024,-0.0627219,0.0928110},
{-0.0746024,-0.0627219,-0.0928110},
{-0.0119676,-0.0928080,0.0076900},
{-0.0119676,-0.0928080,-0.0076900},
{-0.0263299,-0.0760728,0.0667080},
{-0.0263299,-0.0760728,-0.0667080},
{-0.0498842,0.0119450,0.1070630},
{-0.0498842,0.0119450,-0.1070630},
{-0.0553874,0.0627474,0.1172390},
{-0.0553874,0.0627474,-0.1172390},
{-0.0786774,0.0073556,0.0741800},
{-0.0786774,0.0073556,-0.0741800},
{-0.0742762,0.0474272,0.0542330},

Biology Open • Supplementary information

{-0.0742762,0.0474272,-0.0542330},
{-0.0634121,0.0117185,0.0618430},
{-0.0634121,0.0117185,-0.0618430},
{-0.0813084,0.0007821,0.0508130},
{-0.0813084,0.0007821,-0.0508130},
{-0.0496423,-0.0444458,0.0983920},
{-0.0496423,-0.0444458,-0.0983920},
{-0.0480005,-0.0338999,0.0625080},
{-0.0480005,-0.0338999,-0.0625080},
{-0.0428000,-0.0273188,0.0973490},
{-0.0428000,-0.0273188,-0.0973490},
{-0.0548383,-0.0518766,0.0624050},
{-0.0548383,-0.0518766,-0.0624050},
{-0.0696448,-0.1052827,0.0436200},
{-0.0696448,-0.1052827,-0.0436200},
{-0.0739608,-0.0803177,0.0785950},
{-0.0739608,-0.0803177,-0.0785950},
{-0.0586066,-0.0884764,0.0653190},
{-0.0586066,-0.0884764,-0.0653190},
{-0.1017003,0.0085215,0.0624150},
{-0.1017003,0.0085215,-0.0624150},
{-0.0126252,0.0252852,0.1292530},
{-0.0126252,0.0252852,-0.1292530},
{-0.0030963,0.0150156,0.1262640},
{-0.0030963,0.0150156,-0.1262640},
{-0.0621797,0.0048743,0.0605920},
{-0.0621797,0.0048743,-0.0605920},
{-0.0870082,-0.0684280,0.0489900},
{-0.0870082,-0.0684280,-0.0489900},
{-0.0916211,-0.0478344,0.0575290},
{-0.0916211,-0.0478344,-0.0575290},
{-0.0428573,-0.0467026,0.0985250},
{-0.0428573,-0.0467026,-0.0985250},
{-0.0483869,-0.0455474,0.0848580},
{-0.0483869,-0.0455474,-0.0848580},
{-0.0203929,-0.0681811,0.0426070},
{-0.0203929,-0.0681811,-0.0426070},
{-0.0271973,-0.0807786,0.0491650},
{-0.0271973,-0.0807786,-0.0491650},
{-0.0325935,-0.1026813,0.0148280},
{-0.0325935,-0.1026813,-0.0148280},
{-0.0273900,-0.1059173,0.0048750},
{-0.0273900,-0.1059173,-0.0048750},
{-0.0015971,-0.0778770,0.0065530},
{-0.0015971,-0.0778770,-0.0065530},
{-0.1034983,-0.0140026,0.0521800},

{-0.1034983,-0.0140026,-0.0521800},
{-0.1141160,0.0077734,0.0497600},
{-0.1141160,0.0077734,-0.0497600},
{-0.1123797,0.0244808,0.0350160},
{-0.1123797,0.0244808,-0.0350160},
{-0.0984470,-0.0648210,0.0457100},
{-0.0984470,-0.0648210,-0.0457100},
{-0.0814332,-0.0704479,0.0806410},
{-0.0814332,-0.0704479,-0.0806410},
{-0.0540218,-0.0805070,0.0547320},
{-0.0540218,-0.0805070,-0.0547320},
{-0.0674356,-0.0763171,0.0651080},
{-0.0674356,-0.0763171,-0.0651080},
{-0.0602364,-0.0751827,0.0490600},
{-0.0602364,-0.0751827,-0.0490600},
{-0.0604272,-0.0801096,0.0617610},
{-0.0604272,-0.0801096,-0.0617610},
{-0.0655251,0.0632155,0.1093750},
{-0.0655251,0.0632155,-0.1093750},
{-0.0665046,0.0014289,0.0572170},
{-0.0665046,0.0014289,-0.0572170},
{-0.1053786,0.0347803,0.0071580},
{-0.1053786,0.0347803,-0.0071580},
{-0.1321649,-0.0069725,0.0058340},
{-0.1321649,-0.0069725,-0.0058340},
{-0.1232735,-0.0434903,0.0301110},
{-0.1232735,-0.0434903,-0.0301110},
{-0.0497024,0.0261862,0.0084640},
{-0.0497024,0.0261862,-0.0084640},
{-0.0887827,0.0446048,0.0327030},
{-0.0887827,0.0446048,-0.0327030},
{-0.1153998,-0.0126010,0.0290730},
{-0.1153998,-0.0126010,-0.0290730},
{-0.0899939,0.0093882,0.0208440},
{-0.0899939,0.0093882,-0.0208440},
{-0.1312654,-0.0574898,0.0094690},
{-0.1312654,-0.0574898,-0.0094690},
{-0.0996029,0.0042806,0.0105620},
{-0.0996029,0.0042806,-0.0105620},
{-0.1226177,-0.0324069,0.0331630},
{-0.1226177,-0.0324069,-0.0331630},
{-0.1097808,-0.0051619,0.0102520},
{-0.1097808,-0.0051619,-0.0102520}
};
Points1 = {
{0.0000000,0.0000000,0.1330193},

Biology Open • Supplementary information

{-0.0000008,-0.0976427,0.0193694},
{-0.0333557,-0.0758221,0.0925669},
{0.0000000,0.0000000,-0.1330193},
{-0.0000008,-0.0976427,-0.0193694},
{-0.0333557,-0.0758221,-0.0925669},
{0.0000000,0.0000000,0.0000000},
{-0.0910766,-0.0318866,0.0450582},
{-0.0910766,-0.0318866,-0.0450582},
{-0.0863264,-0.1072362,0.0657880},
{-0.0863264,-0.1072362,-0.0657880},
{-0.0592660,0.0448458,0.0970154},
{-0.0592660,0.0448458,-0.0970154},
{-0.0961502,-0.0079885,0.0399487},
{-0.0961502,-0.0079885,-0.0399487},
{-0.0872815,0.0167976,0.0530425},
{-0.0872815,0.0167976,-0.0530425},
{-0.0365883,0.0344214,0.1377392},
{-0.0365883,0.0344214,-0.1377392},
{-0.0238439,0.0347690,0.1422609},
{-0.0238439,0.0347690,-0.1422609},
{-0.0644579,-0.0432653,0.0730353},
{-0.0644579,-0.0432653,-0.0730353},
{-0.0805341,-0.0790901,0.0675612},
{-0.0805341,-0.0790901,-0.0675612},
{-0.0120014,-0.0423946,0.0998663},
{-0.0120014,-0.0423946,-0.0998663},
{-0.0557792,-0.1350468,0.0363076},
{-0.0557792,-0.1350468,-0.0363076},
{-0.0754573,-0.1251903,0.0691863},
{-0.0754573,-0.1251903,-0.0691863},
{-0.0313819,-0.1294184,0.0202563},
{-0.0313819,-0.1294184,-0.0202563},
{-0.0501545,-0.0308346,0.0705698},
{-0.0501545,-0.0308346,-0.0705698},
{-0.0159461,-0.0890300,0.0365873},
{-0.0159461,-0.0890300,-0.0365873},
{-0.0167808,-0.0632838,0.0784409},
{-0.0167808,-0.0632838,-0.0784409},
{-0.0311551,-0.0861790,0.0601854},
{-0.0311551,-0.0861790,-0.0601854},
{-0.0260807,-0.0635288,0.0610442},
{-0.0260807,-0.0635288,-0.0610442},
{-0.0528840,-0.0711181,0.0973594},
{-0.0528840,-0.0711181,-0.0973594},
{-0.0076291,-0.1083248,0.0083163},
{-0.0076291,-0.1083248,-0.0083163},

Biology Open • Supplementary information

{-0.0178315,-0.0762544,0.0714635},
{-0.0178315,-0.0762544,-0.0714635},
{-0.0386267,-0.0010710,0.1132910},
{-0.0386267,-0.0010710,-0.1132910},
{-0.0412276,0.0428065,0.1256664},
{-0.0412276,0.0428065,-0.1256664},
{-0.0654890,-0.0153743,0.0740172},
{-0.0654890,-0.0153743,-0.0740172},
{-0.0627174,0.0151024,0.0533397},
{-0.0627174,0.0151024,-0.0533397},
{-0.0569459,-0.0110381,0.0600156},
{-0.0569459,-0.0110381,-0.0600156},
{-0.0685519,-0.0248859,0.0498292},
{-0.0685519,-0.0248859,-0.0498292},
{-0.0332009,-0.0544807,0.1007153},
{-0.0332009,-0.0544807,-0.1007153},
{-0.0382545,-0.0468930,0.0719751},
{-0.0382545,-0.0468930,-0.0719751},
{-0.0308867,-0.0376574,0.1011094},
{-0.0308867,-0.0376574,-0.1011094},
{-0.0395217,-0.0630430,0.0737494},
{-0.0395217,-0.0630430,-0.0737494},
{-0.0534323,-0.1106268,0.0565377},
{-0.0534323,-0.1106268,-0.0565377},
{-0.0520430,-0.0869514,0.0885587},
{-0.0520430,-0.0869514,-0.0885587},
{-0.0396810,-0.0945948,0.0789515},
{-0.0396810,-0.0945948,-0.0789515},
{-0.0811472,-0.0186078,0.0613751},
{-0.0811472,-0.0186078,-0.0613751},
{-0.0079398,0.0204920,0.1426233},
{-0.0079398,0.0204920,-0.1426233},
{-0.0014267,0.0129205,0.1408687},
{-0.0014267,0.0129205,-0.1408687},
{-0.0566660,-0.0165558,0.0585450},
{-0.0566660,-0.0165558,-0.0585450},
{-0.0677439,-0.0819713,0.0671116},
{-0.0677439,-0.0819713,-0.0671116},
{-0.0718860,-0.0655776,0.0701725},
{-0.0718860,-0.0655776,-0.0701725},
{-0.0273974,-0.0565075,0.1002288},
{-0.0273974,-0.0565075,-0.1002288},
{-0.0335444,-0.0556960,0.0900750},
{-0.0335444,-0.0556960,-0.0900750},
{-0.0141558,-0.0788382,0.0443289},
{-0.0141558,-0.0788382,-0.0443289},

Biology Open • Supplementary information

{-0.0180288,-0.0860285,0.0525233},
{-0.0180288,-0.0860285,-0.0525233},
{-0.0225621,-0.1144412,0.0184548},
{-0.0225621,-0.1144412,-0.0184548},
{-0.0167480,-0.1180470,0.0072093},
{-0.0167480,-0.1180470,-0.0072093},
{-0.0019388,-0.0962613,0.0066900},
{-0.0019388,-0.0962613,-0.0066900},
{-0.0814539,-0.0381532,0.0559967},
{-0.0814539,-0.0381532,-0.0559967},
{-0.0896701,-0.0210344,0.0474725},
{-0.0896701,-0.0210344,-0.0474725},
{-0.0880350,-0.0126367,0.0305221},
{-0.0880350,-0.0126367,-0.0305221},
{-0.0794106,-0.0811295,0.0661352},
{-0.0794106,-0.0811295,-0.0661352},
{-0.0589497,-0.0794563,0.0901338},
{-0.0589497,-0.0794563,-0.0901338},
{-0.0352767,-0.0828845,0.0653558},
{-0.0352767,-0.0828845,-0.0653558},
{-0.0468831,-0.0847571,0.0788284},
{-0.0468831,-0.0847571,-0.0788284},
{-0.0419310,-0.0825286,0.0616026},
{-0.0419310,-0.0825286,-0.0616026},
{-0.0406195,-0.0876769,0.0760407},
{-0.0406195,-0.0876769,-0.0760407},
{-0.0510618,0.0391671,0.1167383},
{-0.0510618,0.0391671,-0.1167383},
{-0.0602560,-0.0207165,0.0543825},
{-0.0602560,-0.0207165,-0.0543825},
{-0.0833393,-0.0065496,0.0061338},
{-0.0833393,-0.0065496,-0.0061338},
{-0.1018550,-0.0413485,0.0048809},
{-0.1018550,-0.0413485,-0.0048809},
{-0.1008481,-0.0715854,0.0229133},
{-0.1008481,-0.0715854,-0.0229133},
{-0.0454620,-0.0074836,0.0083370},
{-0.0454620,-0.0074836,-0.0083370},
{-0.0729178,0.0067194,0.0302086},
{-0.0729178,0.0067194,-0.0302086},
{-0.0903341,-0.0430412,0.0276179},
{-0.0903341,-0.0430412,-0.0276179},
{-0.0728361,-0.0248901,0.0195498},
{-0.0728361,-0.0248901,-0.0195498},
{-0.1074968,-0.0837605,0.0071614},
{-0.1074968,-0.0837605,-0.0071614},

Biology Open • Supplementary information

```
                    {-0.0775603,-0.0319611,0.0098097},
                    {-0.0775603,-0.0319611,-0.0098097},
                    {-0.0985056,-0.0609136,0.0291713},
                    {-0.0985056,-0.0609136,-0.0291713},
                    {-0.0837638,-0.0405539,0.0093994},
                    {-0.0837638,-0.0405539,-0.0093994}
                    };
            BoundingBoxOnOff = Off;
        };
        AnyFunTransform3DIdentity ScaleFunction = {
            PreTransforms = {&.RBFTransform};
};
};

};
```

Biology Open • Supplementary information

ScalingFunctionTLEMLucyFemur_2014051

```
AnyFolder ScalingFunctionTLEMLucyFemur = {
  AnyFolder Right = {
        AnyFolder Thigh = {
        AnyFunTransform3DRBF RBFTransform = {
                RBFDef.Type = RBF_ThinPlate;
                PolynomDegree = 1;
                Points0 = {
                        {0.0000000,0.0000000,0.0000000},
                        {-0.0000000,-0.3616821,0.0000000},
                        {-0.0097563,-0.3678799,0.0012967},
                        {-0.0000000,-0.3660632,0.0408203},
                        {-0.0000000,-0.3573010,-0.0408203},
                        {0.0161460,-0.0072838,0.0601290},
                        {0.0220217,-0.0203698,0.0463848},
                        {0.0123977,-0.0241932,0.0668573},
                        {0.0006898,0.0018121,0.0538181},
                        {0.0122809,-0.0068668,0.0414535},
                        {-0.0058991,-0.0138188,0.0648412},
                        {0.0211469,-0.0380855,0.0538111},
                        {0.0172133,-0.0317342,0.0328381},
                        {0.0177311,-0.0131946,0.0256176},
                        {-0.0110079,-0.0308867,0.0632370},
                        {0.0039435,-0.0395977,0.0638271},
                        {-0.0041683,-0.0187472,0.0413214},
                        {-0.0177999,-0.0021535,0.0496084},
                        {0.0001603,-0.0053126,0.0279593},
                        {-0.0211600,-0.0216243,0.0497147},
                        {0.0096163,-0.0568663,0.0545719},
                        {0.0162112,-0.0515013,0.0395230},
                        {0.0093533,-0.0433265,0.0187387},
                        {0.0119398,-0.0238469,0.0118403},
                        {0.0210707,-0.0048848,0.0065878},
                        {0.0125013,0.0064094,0.0177702},
                        {-0.0191523,-0.0356053,0.0382429},
                        {-0.0115821,-0.0474937,0.0537648},
                        {-0.0100073,-0.0236371,0.0267322},
                        {-0.0078925,0.0107098,0.0182833},
                        {-0.0134217,-0.0055356,0.0182000},
                        {-0.0090061,-0.0665700,0.0475484},
                        {0.0071095,-0.0761976,0.0520035},
                        {0.0182119,-0.0704149,0.0386546},
                        {0.0073764,-0.0609481,0.0221006},
                        {-0.0122984,-0.0439585,0.0198428},
                        {-0.0032253,-0.0306792,0.0110995},
```

{-0.0012975,-0.0198451,-0.0052592},
{0.0140405,-0.0161125,-0.0071649},
{0.0189222,-0.0014898,-0.0127226},
{0.0181642,0.0132812,-0.0011050},
{0.0045119,0.0200001,0.0091189},
{-0.0176761,-0.0541983,0.0348587},
{-0.0136548,-0.0142015,0.0054311},
{-0.0135510,0.0182940,0.0017175},
{-0.0223865,0.0023399,0.0028018},
{-0.0086455,-0.0738247,0.0283843},
{-0.0050356,-0.0877749,0.0442195},
{0.0160135,-0.0919319,0.0465120},
{0.0128290,-0.0785810,0.0232431},
{-0.0168328,-0.0616327,0.0155917},
{-0.0172369,-0.0094567,-0.0119502},
{0.0002090,-0.0093641,-0.0209779},
{0.0064827,0.0081538,-0.0198453},
{0.0010777,0.0210422,-0.0084721},
{-0.0129485,0.0085451,-0.0162212},
{-0.0002322,-0.0924033,0.0245644},
{-0.0028175,-0.1052263,0.0418294},
{0.0172322,-0.1111444,0.0445760},
{0.0203259,-0.0957409,0.0271449},
{0.0057102,-0.1088933,0.0195113},
{-0.0010852,-0.1230776,0.0363132},
{0.0182727,-0.1303312,0.0422884},
{0.0242891,-0.1161703,0.0265054},
{0.0094531,-0.1268584,0.0162294},
{0.0014244,-0.1413293,0.0331653},
{0.0207271,-0.1496427,0.0394157},
{0.0267562,-0.1360142,0.0236798},
{0.0113839,-0.1461651,0.0136024},
{0.0030845,-0.1595094,0.0297156},
{0.0209528,-0.1691386,0.0369775},
{0.0283958,-0.1540209,0.0213795},
{0.0129499,-0.1649012,0.0113589},
{0.0016490,-0.1783108,0.0216287},
{0.0147105,-0.1880678,0.0328614},
{0.0298491,-0.1859865,0.0248551},
{0.0286645,-0.1714377,0.0169488},
{0.0165897,-0.1861006,0.0082595},
{0.0014000,-0.1973476,0.0183740},
{0.0114301,-0.2109792,0.0286157},
{0.0267422,-0.2037622,0.0296951},
{0.0288630,-0.2018335,0.0130127},
{0.0145471,-0.2083505,0.0062833},

Biology Open • Supplementary information

{0.0011300,-0.2198071,0.0155602},
{0.0126610,-0.2321235,0.0275354},
{0.0275647,-0.2219268,0.0265185},
{0.0286645,-0.2230253,0.0098701},
{0.0130434,-0.2304822,0.0039177},
{0.0022984,-0.2409585,0.0149650},
{0.0101315,-0.2534740,0.0252125},
{0.0279485,-0.2428555,0.0234276},
{0.0276684,-0.2421559,0.0058691},
{0.0101250,-0.2529527,0.0018983},
{0.0021376,-0.2667831,0.0107073},
{0.0114309,-0.2734905,0.0249960},
{0.0273923,-0.2634953,0.0216324},
{0.0278429,-0.2592546,0.0037692},
{0.0159101,-0.2712985,-0.0033988},
{0.0036708,-0.2863280,0.0004644},
{0.0023115,-0.2884756,0.0187554},
{0.0231385,-0.2879051,0.0224853},
{0.0304009,-0.2799973,0.0083549},
{0.0238189,-0.2917246,-0.0052462},
{0.0064904,-0.3027687,-0.0070587},
{-0.0003492,-0.3064690,0.0093014},
{0.0122179,-0.3045269,0.0243511},
{0.0301837,-0.3030127,0.0114462},
{0.0234182,-0.3107336,-0.0085198},
{0.0046893,-0.3214995,-0.0123881},
{-0.0022352,-0.3264905,0.0043340},
{0.0016841,-0.3202756,0.0217839},
{0.0229722,-0.3196887,0.0215083},
{0.0292438,-0.3228042,0.0051430},
{0.0221641,-0.3296190,-0.0132361},
{0.0100459,-0.3417597,-0.0244560},
{-0.0053009,-0.3375737,-0.0137705},
{-0.0064432,-0.3487307,0.0028676},
{-0.0059346,-0.3407801,0.0202938},
{0.0105130,-0.3365976,0.0264175},
{0.0282695,-0.3386249,0.0180307},
{0.0270402,-0.3431844,-0.0001487},
{0.0257040,-0.3506950,-0.0181851},
{0.0178948,-0.3636114,-0.0302838},
{0.0043149,-0.3544109,-0.0381949},
{-0.0095210,-0.3461482,-0.0295031},
{-0.0164526,-0.3542895,-0.0126666},
{-0.0060896,-0.3690127,-0.0033061},
{-0.0176523,-0.3612686,0.0110109},
{-0.0153310,-0.3546943,0.0299634},

Biology Open • Supplementary information

{0.0021329,-0.3508021,0.0350763},
{0.0210690,-0.3548628,0.0279835},
{0.0356594,-0.3558162,0.0159356},
{0.0275718,-0.3617450,-0.0018607},
{0.0298503,-0.3741530,-0.0163721},
{0.0175320,-0.3855300,-0.0277515},
{0.0025604,-0.3740099,-0.0397137},
{-0.0136727,-0.3624016,-0.0402102},
{-0.0277647,-0.3567472,-0.0284947},
{-0.0275446,-0.3699630,-0.0120829},
{-0.0175846,-0.3859355,-0.0177922},
{0.0001572,-0.3856873,-0.0118050},
{0.0030262,-0.3834856,0.0075553},
{-0.0144114,-0.3810887,0.0140304},
{-0.0247706,-0.3701239,0.0285134},
{-0.0044804,-0.3677201,0.0404632},
{0.0133183,-0.3712274,0.0334208},
{0.0324861,-0.3723417,0.0245668},
{0.0271451,-0.3766027,0.0072441},
{0.0167985,-0.3822816,-0.0065349},
{-0.0001454,-0.3910823,-0.0303010},
{-0.0144998,-0.3834154,-0.0384137},
{-0.0301321,-0.3750841,-0.0321336},
{0.0196491,-0.3864804,0.0210853},
{0.0015726,-0.3888034,0.0276569},
{-0.0146652,-0.3847848,0.0336913},
{-0.0014588,-0.0619951,0.0219778},
{-0.0118435,-0.0550626,0.0139639},
{0.0129812,-0.0349966,0.0673128},
{-0.0162907,-0.0105666,0.0597739},
{-0.0012885,-0.0520816,0.0562989},
{-0.0090599,-0.0631298,0.0172224},
{-0.0000222,-0.0771663,0.0231897},
{-0.0068148,0.0023885,-0.0160086},
{-0.0180544,-0.0425409,0.0446585},
{0.0020525,-0.0249904,0.0691549},
{-0.0157097,-0.0662315,0.0238393},
{-0.0010518,-0.0510686,0.0199353},
{-0.0208874,-0.0152865,0.0500034},
{0.0168316,-0.0280857,0.0666680},
{-0.0187392,-0.0282333,0.0403282},
{0.0191622,-0.0351633,0.0622707},
{0.0001216,-0.0706289,0.0524049},
{-0.0169862,-0.0399062,0.0311827},
{-0.0185737,-0.0533066,0.0151224},
{-0.0219058,-0.0528723,0.0222263},

Biology Open • Supplementary information

{-0.0028271,-0.0391620,0.0148452},
{0.0117900,-0.0553425,0.0235348},
{0.0219835,-0.0106400,0.0396338},
{-0.0025366,-0.0401046,0.0625273},
{0.0141017,-0.0018767,0.0198108},
{-0.0078770,-0.0473929,0.0173542},
{-0.0065351,-0.0766431,0.0470258},
{0.0065585,0.0145593,0.0161638},
{-0.0118588,0.0172875,-0.0085064},
{-0.0074087,-0.0627500,0.0506700},
{0.0071940,-0.0780068,0.0213362},
{0.0126736,-0.0708176,0.0244108},
{0.0074694,-0.0436450,0.0604196},
{0.0154186,-0.0139471,0.0064312},
{-0.0053535,-0.0213210,0.0040094},
{-0.0088733,0.0002018,0.0205402},
{-0.0112471,-0.0542608,0.0500266},
{-0.0061625,-0.0248673,0.0653244},
{0.0093695,-0.0065826,0.0614157},
{0.0138015,-0.0467322,0.0284333},
{-0.0020999,-0.0186885,-0.0116392},
{-0.0185640,-0.0225591,0.0566779},
{0.0175798,0.0124360,-0.0068467},
{-0.0039962,0.0217127,0.0053055},
{0.0014975,-0.0784591,0.0514056},
{-0.0172997,-0.0386971,0.0525252},
{0.0196540,-0.0268271,0.0416806},
{0.0135296,-0.0406504,0.0236882},
{0.0182471,0.0095959,0.0087906},
{0.0218667,0.0016804,0.0058793},
{0.0224975,-0.0022652,-0.0022994},
{0.0166750,-0.0191391,0.0236436},
{0.0119785,-0.0328723,0.0195376},
{0.0187631,-0.0133469,0.0308741},
{-0.0187543,-0.0461463,0.0257491},
{0.0039834,-0.0373928,0.0683089},
{0.0157029,-0.0423169,0.0385226},
{0.0164003,-0.0087069,0.0131181},
{0.0008253,0.0033370,-0.0212576},
{0.0101707,-0.0009724,-0.0205688},
{0.0102948,-0.0642683,0.0524532},
{0.0152217,-0.0557944,0.0508978},
{0.0172973,-0.0565707,0.0419468},
{0.0167723,-0.0635334,0.0369834},
{-0.0047397,-0.0695325,0.0241398},
{-0.0152913,0.0035778,0.0161461},

Biology Open • Supplementary information

{0.0091697,0.0009340,0.0575160},
{0.0196686,-0.0089533,0.0500006},
{0.0183998,-0.0212943,0.0622968},
{-0.0114340,-0.0316810,0.0250940},
{-0.0206442,-0.0092599,-0.0028425},
{0.0166868,-0.0784295,0.0276925},
{-0.0069678,-0.0054521,-0.0207011},
{0.0049490,0.0151109,-0.0160093},
{-0.0084025,-0.0318937,0.0162788},
{-0.0173679,0.0095594,-0.0108509},
{-0.0218912,0.0063457,-0.0020698},
{-0.0106547,-0.0668750,0.0397749},
{0.0136098,-0.0170666,-0.0018890},
{0.0141599,-0.0441405,0.0587594},
{0.0093255,-0.0183218,-0.0083008},
{0.0106367,-0.0128276,-0.0154269},
{0.0037350,-0.0549365,0.0209786},
{0.0087662,-0.0472902,0.0195496},
{0.0016839,-0.0708213,0.0221325},
{-0.0202977,-0.0530540,0.0301270},
{-0.0068584,0.0021781,0.0530002},
{-0.0144817,-0.0297218,0.0605833},
{0.0197015,-0.0165217,0.0560654},
{0.0156609,-0.0705363,0.0477736},
{0.0207876,-0.0221433,0.0330733},
{0.0092324,-0.0300767,0.0713160},
{-0.0203392,-0.0034488,0.0097332},
{0.0156978,-0.0143438,0.0629053},
{0.0143267,-0.0563519,0.0317489},
{-0.0161685,-0.0465391,0.0188591},
{-0.0196397,-0.0309875,0.0546496},
{0.0154804,-0.0380422,0.0305433},
{-0.0077774,-0.0351303,0.0637765},
{0.0063980,-0.0086419,-0.0201654},
{0.0030146,-0.0151094,-0.0165974},
{-0.0210080,-0.0096030,0.0507855},
{-0.0105684,-0.0411377,0.0586146},
{0.0180619,-0.0498960,0.0469835},
{-0.0192318,0.0116108,0.0024070},
{-0.0165651,0.0083622,0.0126870},
{-0.0014333,-0.0627138,0.0534746},
{0.0145127,-0.0080178,0.0353667},
{0.0148374,-0.0012228,0.0566181},
{0.0153834,-0.0062427,0.0444727},
{-0.0155164,-0.0132103,-0.0096398},
{0.0068685,-0.0221022,0.0011547},

{0.0003332,-0.0458500,0.0183716},
{0.0205582,0.0040956,-0.0082969},
{0.0175752,0.0009340,-0.0148407},
{0.0142357,-0.0063623,-0.0169332},
{-0.0147285,0.0023412,-0.0164678},
{-0.0158781,-0.0064451,-0.0151346},
{0.0175951,0.0133456,0.0037559},
{0.0117660,0.0189899,-0.0030690},
{0.0096040,0.0151827,-0.0137450},
{0.0096565,-0.0215194,0.0662734},
{-0.0006793,-0.0074494,0.0631218},
{0.0007819,-0.0004519,0.0600781},
{0.0065377,-0.0125433,0.0654199},
{0.0090461,0.0073096,0.0188711},
{0.0151418,-0.0168302,0.0146465},
{0.0025166,-0.0231863,0.0688894},
{-0.0097891,-0.0152077,0.0253086},
{-0.0109149,-0.0177935,0.0119825},
{-0.0072079,-0.0099373,0.0272725},
{-0.0183727,-0.0467429,0.0356276},
{-0.0126871,-0.0584555,0.0428141},
{-0.0057054,-0.0273042,0.0103554},
{0.0215142,-0.0159693,0.0474531},
{0.0128492,-0.0070016,0.0287645},
{0.0052134,-0.0017047,0.0247463},
{-0.0024284,-0.0707498,0.0513049},
{-0.0036140,0.0220593,-0.0040484},
{-0.0002405,-0.0121253,0.0390050},
{-0.0119811,-0.0156737,0.0015445},
{0.0142082,-0.0279792,0.0246553},
{0.0122244,-0.0262013,0.0169829},
{-0.0146264,-0.0038855,0.0549384},
{0.0207450,-0.0325771,0.0496412},
{-0.0155040,-0.0497560,0.0437087},
{-0.0081119,-0.0756450,0.0387365},
{0.0039684,-0.0062047,0.0313900},
{-0.0023726,-0.0038892,-0.0220521},
{-0.0136977,-0.0339839,0.0602619},
{-0.0035399,0.0089081,-0.0188894},
{-0.0049820,0.0176571,-0.0132254},
{0.0193902,-0.0803191,0.0360429},
{0.0040648,-0.0697109,0.0218205},
{0.0170470,-0.0818610,0.0456892},
{0.0104175,0.0188635,0.0064965},
{0.0135206,0.0092549,0.0158079},
{0.0023933,0.0160498,-0.0157404},

{0.0164753,-0.0118197,-0.0096995},
{0.0202584,-0.0066858,-0.0082383},
{-0.0098401,-0.0226001,0.0314028},
{-0.0130937,-0.0309622,0.0320764},
{-0.0201510,-0.0613297,0.0222264},
{-0.0010389,0.0036055,0.0209003},
{0.0042025,0.0073392,0.0199393},
{-0.0021630,-0.0038531,0.0251898},
{-0.0007845,-0.0293574,0.0675788},
{0.0070027,-0.0406327,0.0624565},
{-0.0124363,-0.0183001,0.0627966},
{-0.0120485,-0.0256613,0.0628493},
{0.0101200,0.0197695,0.0039334},
{0.0167636,0.0148266,0.0014155},
{-0.0074981,-0.0106930,0.0632797},
{0.0142533,-0.0503445,0.0351954},
{-0.0146107,-0.0460953,0.0506120},
{-0.0161117,-0.0471301,0.0178900},
{0.0112149,-0.0792793,0.0501460},
{-0.0099060,-0.0230794,0.0186951},
{-0.0114543,-0.0153049,0.0183675},
{-0.0042882,0.0161879,0.0151423},
{0.0014582,-0.0638572,0.0541080},
{0.0048554,-0.0582603,0.0553815},
{-0.0017077,-0.0572302,0.0546635},
{0.0166090,-0.0697169,0.0323038},
{-0.0208812,-0.0164273,0.0474354},
{0.0130454,-0.0241864,0.0178294},
{0.0053123,-0.0483770,0.0582251},
{0.0112225,-0.0361429,0.0673300},
{0.0085729,-0.0771256,0.0216100},
{-0.0070783,-0.0488033,0.0560869},
{0.0182083,-0.0411024,0.0454236},
{-0.0119590,-0.0403809,0.0213546},
{0.0233409,-0.0185535,0.0366343},
{-0.0046347,-0.0571211,0.0208243},
{0.0183166,-0.0006966,0.0140116},
{0.0061913,0.0053512,0.0201003},
{-0.0002364,-0.0310828,0.0680351},
{-0.0170773,0.0139270,-0.0054987},
{0.0110805,0.0156034,0.0117010},
{-0.0124628,0.0164667,0.0094550},
{-0.0209014,-0.0073407,-0.0051969},
{-0.0210131,0.0010693,-0.0086891},
{-0.0041596,-0.0172332,0.0663041},
{-0.0216595,-0.0322172,0.0451540},

Biology Open • Supplementary information

{0.0051908,-0.0426014,0.0613013},
{0.0150003,-0.0416136,0.0606257},
{-0.0135090,-0.0674234,0.0236425},
{0.0146416,0.0073691,0.0160052},
{0.0195615,-0.0112412,-0.0010333},
{-0.0198115,-0.0098244,0.0044735},
{-0.0157054,-0.0521236,0.0395039},
{-0.0081381,-0.0173108,-0.0046152},
{-0.0218324,-0.0262661,0.0471115},
{0.0051446,-0.0707257,0.0530555},
{0.0179359,-0.0471493,0.0528134},
{-0.0005591,-0.0317983,0.0113632},
{0.0071707,-0.0305943,0.0121186},
{0.0206239,-0.0061367,0.0066642},
{0.0187057,-0.0745868,0.0420207},
{-0.0095784,-0.0163113,0.0259524},
{-0.0012181,0.0127661,0.0186855},
{0.0059420,-0.0630691,0.0220087},
{0.0069408,-0.0573878,0.0552614},
{0.0150782,-0.0132884,-0.0101793},
{0.0005533,-0.0078574,0.0633775},
{-0.0088284,-0.0146399,-0.0147837},
{0.0167444,-0.0651105,0.0470347},
{0.0082666,-0.0181741,-0.0095133},
{-0.0175162,-0.0637444,0.0177069},
{0.0153795,-0.0360767,0.0287608},
{0.0162363,-0.0018864,0.0548412},
{0.0052731,-0.0075919,0.0392990},
{-0.0022288,-0.0556644,0.0549909},
{-0.0156238,-0.0550038,0.0377688},
{-0.0204137,-0.0100550,0.0536429},
{-0.0093683,0.0122429,-0.0162013},
{-0.0202927,0.0049166,0.0083887},
{0.0074706,-0.0627945,0.0222133},
{0.0035034,-0.0376103,0.0672421},
{-0.0190752,-0.0194129,0.0555530},
{0.0061758,-0.0074961,-0.0207741},
{0.0192011,-0.0273063,0.0588233},
{0.0130796,-0.0134383,0.0638611},
{0.0120421,0.0019566,-0.0194140},
{0.0154152,-0.0065294,0.0179581},
{-0.0157335,0.0101219,0.0123981},
{0.0110376,-0.0488274,0.0567911},
{-0.0055170,-0.0343676,0.0647140},
{0.0216204,-0.0273988,0.0512041},
{0.0161331,-0.0135875,-0.0075501},

Biology Open • Supplementary information

{-0.0184643,-0.0455633,0.0360087},
{-0.0091978,-0.0682413,0.0460847},
{0.0034304,-0.0471677,0.0588866},
{-0.0048025,-0.0384316,0.0149400},
{0.0118505,0.0109735,-0.0159161},
{-0.0027036,-0.0204189,-0.0015094},
{-0.0117607,-0.0692759,0.0292171},
{-0.0107080,-0.0033455,-0.0190487},
{-0.0042548,0.0214947,-0.0056846},
{-0.0097315,-0.0208529,0.0131997},
{-0.0178958,-0.0627218,0.0171551},
{-0.0068692,-0.0688818,0.0233316},
{0.0012083,0.0120695,-0.0186179},
{0.0215073,-0.0236027,0.0503069},
{-0.0060262,-0.0534931,0.0189523},
{0.0179951,-0.0336785,0.0397267},
{0.0190509,-0.0284972,0.0336394},
{-0.0169188,-0.0133609,-0.0014203},
{0.0135722,0.0136491,0.0114695},
{-0.0150549,-0.0123230,0.0067722},
{-0.0148411,-0.0311945,0.0355412},
{0.0185490,-0.0794042,0.0431266},
{0.0193568,-0.0087326,0.0575704},
{0.0201026,-0.0144353,0.0531586},
{0.0025583,-0.0248211,0.0045714},
{0.0132689,-0.0214866,0.0652899},
{-0.0028669,-0.0099311,0.0309271},
{-0.0219487,-0.0054893,-0.0021853},
{0.0026180,-0.0203806,-0.0040620},
{-0.0116230,-0.0668518,0.0370152},
{-0.0133007,-0.0264159,0.0619176},
{0.0186167,0.0050491,-0.0120059},
{-0.0191359,-0.0591690,0.0288149},
{-0.0178121,-0.0041834,0.0141448},
{0.0123077,-0.0063648,0.0279743},
{-0.0157729,-0.0374010,0.0315335},
{0.0149328,-0.0659100,0.0290095},
{0.0006205,0.0021977,-0.0214613},
{0.0170484,-0.0715687,0.0325980},
{0.0068499,0.0061192,0.0196608},
{-0.0167075,0.0020024,0.0150382},
{0.0059801,-0.0393117,0.0167489},
{-0.0072506,-0.0171067,0.0338751},
{0.0202363,-0.0251596,0.0420997},
{-0.0199311,-0.0601255,0.0171522},
{0.0216641,0.0053410,-0.0027841},

Biology Open • Supplementary information

{-0.0197665,-0.0606125,0.0252972},
{0.0129986,-0.0214528,0.0098002},
{0.0201151,-0.0100929,0.0460501},
{0.0104137,0.0017765,-0.0203073},
{0.0166557,-0.0064123,0.0140197},
{-0.0124120,-0.0596549,0.0422637},
{0.0014885,0.0202590,0.0096781},
{0.0028720,0.0020207,-0.0219044},
{0.0376095,-0.3675427,0.0214537},
{0.0049949,0.0206417,-0.0079017},
{-0.0191723,-0.0407590,0.0391310},
{-0.0316406,-0.3638215,-0.0274841},
{0.0021392,0.0226920,0.0000985},
{0.0168435,-0.0229382,0.0264788},
{-0.0157304,-0.0005437,-0.0161456},
{0.0203722,-0.0299750,0.0562762},
{-0.0171077,0.0134146,0.0059615},
{0.0175519,-0.0370653,0.0413374},
{0.0154657,-0.0405127,0.0348980},
{-0.0215172,-0.0323502,0.0481925},
{0.0177998,-0.0515776,0.0444709},
{-0.0183804,-0.0448316,0.0275139},
{0.0033607,-0.0502028,0.0575894},
{0.0010325,-0.0479377,0.0191141},
{0.0224852,-0.0181821,0.0451780},
{0.0084089,-0.0720538,0.0521031},
{-0.0070880,-0.0772912,0.0288023},
{-0.0223712,-0.0028990,0.0024047},
{0.0173136,-0.0120017,0.0204612},
{0.0137772,-0.0623537,0.0276621},
{-0.0100390,-0.0231180,0.0292222},
{-0.0196395,-0.3730207,0.0364730},
{-0.0094445,-0.0526688,0.0528111},
{-0.0115309,-0.3679776,-0.0104995},
{0.0129812,-0.0410477,0.0224072},
{0.0117394,0.0154849,-0.0117191},
{-0.0245208,-0.3649412,0.0295111},
{0.0147476,-0.0502966,0.0364899},
{0.0006132,-0.0446771,0.0600524},
{-0.0118123,0.0191139,0.0031977},
{-0.0017185,-0.0163303,0.0664216},
{-0.0085098,-0.0074564,0.0247745},
{0.0197459,-0.0086852,0.0430460},
{0.0187064,-0.0223532,0.0600966},
{-0.0206552,-0.0099712,0.0525024},
{0.0207525,0.0084646,-0.0024846},

{-0.0094915,-0.0325446,0.0184441},
{-0.0014464,-0.0140050,-0.0179819},
{-0.0122725,-0.0573444,0.0463125},
{-0.0106397,0.0177211,0.0094669},
{0.0111981,0.0191236,-0.0041491},
{-0.0162790,-0.0256279,0.0592687},
{-0.0060120,-0.3653959,0.0413411},
{-0.0135007,-0.3703638,-0.0387476},
{-0.0134258,-0.3703142,-0.0379467},
{-0.0133509,-0.3702645,-0.0371458},
{-0.0132760,-0.3702148,-0.0363450},
{-0.0132011,-0.3701651,-0.0355441},
{-0.0131262,-0.3701154,-0.0347432},
{-0.0130513,-0.3700658,-0.0339423},
{-0.0129765,-0.3700161,-0.0331414},
{-0.0129016,-0.3699664,-0.0323405},
{-0.0128267,-0.3699167,-0.0315396},
{-0.0127518,-0.3698670,-0.0307388},
{-0.0126769,-0.3698174,-0.0299379},
{-0.0126020,-0.3697677,-0.0291370},
{-0.0125271,-0.3697180,-0.0283361},
{-0.0124523,-0.3696683,-0.0275352},
{-0.0123774,-0.3696187,-0.0267343},
{-0.0123025,-0.3695690,-0.0259334},
{-0.0122276,-0.3695193,-0.0251325},
{-0.0121527,-0.3694696,-0.0243317},
{-0.0120778,-0.3694199,-0.0235308},
{-0.0120029,-0.3693703,-0.0227299},
{-0.0119280,-0.3693206,-0.0219290},
{-0.0118532,-0.3692709,-0.0211281},
{-0.0117783,-0.3692212,-0.0203272},
{-0.0117034,-0.3691715,-0.0195263},
{-0.0116285,-0.3691219,-0.0187255},
{-0.0115536,-0.3690722,-0.0179246},
{-0.0114787,-0.3690225,-0.0171237},
{-0.0114038,-0.3689728,-0.0163228},
{-0.0113289,-0.3689231,-0.0155219},
{-0.0112541,-0.3688735,-0.0147210},
{-0.0111792,-0.3688238,-0.0139201},
{-0.0111043,-0.3687741,-0.0131192},
{-0.0110294,-0.3687244,-0.0123184},
{-0.0109545,-0.3686748,-0.0115175},
{-0.0108796,-0.3686251,-0.0107166},
{-0.0108047,-0.3685754,-0.0099157},
{-0.0107298,-0.3685257,-0.0091148},
{-0.0106550,-0.3684760,-0.0083139},

Biology Open • Supplementary information

{-0.0105801,-0.3684264,-0.0075130},
{-0.0105052,-0.3683767,-0.0067121},
{-0.0104303,-0.3683270,-0.0059113},
{-0.0103554,-0.3682773,-0.0051104},
{-0.0102805,-0.3682276,-0.0043095},
{-0.0102056,-0.3681780,-0.0035086},
{-0.0101307,-0.3681283,-0.0027077},
{-0.0100559,-0.3680786,-0.0019068},
{-0.0099810,-0.3680289,-0.0011059},
{-0.0099061,-0.3679792,-0.0003051},
{-0.0098312,-0.3679296,0.0004958},
{-0.0097563,-0.3678799,0.0012967},
{-0.0096814,-0.3678302,0.0020976},
{-0.0096065,-0.3677805,0.0028985},
{-0.0095317,-0.3677308,0.0036994},
{-0.0094568,-0.3676812,0.0045003},
{-0.0093819,-0.3676315,0.0053012},
{-0.0093070,-0.3675818,0.0061020},
{-0.0092321,-0.3675321,0.0069029},
{-0.0091572,-0.3674825,0.0077038},
{-0.0090823,-0.3674328,0.0085047},
{-0.0090074,-0.3673831,0.0093056},
{-0.0089326,-0.3673334,0.0101065},
{-0.0088577,-0.3672837,0.0109074},
{-0.0087828,-0.3672341,0.0117082},
{-0.0087079,-0.3671844,0.0125091},
{-0.0086330,-0.3671347,0.0133100},
{-0.0085581,-0.3670850,0.0141109},
{-0.0084832,-0.3670353,0.0149118},
{-0.0084083,-0.3669857,0.0157127},
{-0.0083335,-0.3669360,0.0165136},
{-0.0082586,-0.3668863,0.0173145},
{-0.0081837,-0.3668366,0.0181153},
{-0.0081088,-0.3667869,0.0189162},
{-0.0080339,-0.3667373,0.0197171},
{-0.0079590,-0.3666876,0.0205180},
{-0.0078841,-0.3666379,0.0213189},
{-0.0078092,-0.3665882,0.0221198},
{-0.0077344,-0.3665386,0.0229207},
{-0.0076595,-0.3664889,0.0237215},
{-0.0075846,-0.3664392,0.0245224},
{-0.0075097,-0.3663895,0.0253233},
{-0.0074348,-0.3663398,0.0261242},
{-0.0073599,-0.3662902,0.0269251},
{-0.0072850,-0.3662405,0.0277260},
{-0.0072101,-0.3661908,0.0285269},

{-0.0071353,-0.3661411,0.0293278},
{-0.0070604,-0.3660914,0.0301286},
{-0.0069855,-0.3660418,0.0309295},
{-0.0069106,-0.3659921,0.0317304},
{-0.0068357,-0.3659424,0.0325313},
{-0.0067608,-0.3658927,0.0333322},
{-0.0066859,-0.3658430,0.0341331},
{-0.0066111,-0.3657934,0.0349340},
{-0.0065362,-0.3657437,0.0357348},
{-0.0064613,-0.3656940,0.0365357},
{-0.0063864,-0.3656443,0.0373366},
{-0.0063115,-0.3655947,0.0381375},
{-0.0062366,-0.3655450,0.0389384},
{-0.0061617,-0.3654953,0.0397393},
{-0.0060868,-0.3654456,0.0405402}
};
Points1 = {
{0.0000000,0.0000000,0.0000000},
{-0.0000000,-0.4956311,0.0000000},
{-0.0105515,-0.5041242,0.0014024},
{-0.0000000,-0.5016347,0.0441474},
{-0.0000000,-0.4896274,-0.0441474},
{0.0157077,-0.0111381,0.0633292},
{0.0227918,-0.0289680,0.0498430},
{0.0109539,-0.0328570,0.0690871},
{0.0001770,-0.0005124,0.0563724},
{0.0127207,-0.0088102,0.0439317},
{-0.0067059,-0.0180443,0.0716517},
{0.0190772,-0.0498739,0.0570900},
{0.0170923,-0.0433063,0.0366848},
{0.0161448,-0.0186469,0.0277541},
{-0.0114878,-0.0414163,0.0683670},
{0.0038056,-0.0542163,0.0672390},
{-0.0034841,-0.0259612,0.0456049},
{-0.0141427,-0.0055717,0.0531140},
{-0.0000101,-0.0054655,0.0308163},
{-0.0138874,-0.0295643,0.0545992},
{0.0114097,-0.0784676,0.0599793},
{0.0181452,-0.0697096,0.0427296},
{0.0094589,-0.0588622,0.0214259},
{0.0109389,-0.0313482,0.0139777},
{0.0207282,-0.0071122,0.0063892},
{0.0117294,0.0078414,0.0178309},
{-0.0138154,-0.0493146,0.0406325},
{-0.0113493,-0.0641063,0.0577304},
{-0.0104190,-0.0329994,0.0278931},

Biology Open • Supplementary information

{-0.0069175,0.0138036,0.0178635},
{-0.0128376,-0.0075941,0.0176091},
{-0.0084019,-0.0906589,0.0500646},
{0.0071099,-0.1040291,0.0581587},
{0.0193225,-0.0975167,0.0412191},
{0.0093778,-0.0839777,0.0235426},
{-0.0132745,-0.0596273,0.0231372},
{-0.0047370,-0.0423843,0.0114320},
{-0.0005011,-0.0257247,-0.0053819},
{0.0144355,-0.0201618,-0.0073073},
{0.0195053,-0.0023177,-0.0124793},
{0.0191251,0.0167924,-0.0013585},
{0.0035349,0.0254835,0.0100915},
{-0.0160511,-0.0740186,0.0379657},
{-0.0133355,-0.0161742,0.0064984},
{-0.0136495,0.0219721,0.0018022},
{-0.0208223,0.0030461,0.0035557},
{-0.0076608,-0.1004177,0.0310904},
{-0.0048478,-0.1204022,0.0471425},
{0.0164489,-0.1264508,0.0502504},
{0.0140413,-0.1080394,0.0244112},
{-0.0113980,-0.0802019,0.0240324},
{-0.0163555,-0.0114465,-0.0114638},
{0.0007406,-0.0118213,-0.0209511},
{0.0067275,0.0100534,-0.0192847},
{0.0018929,0.0260052,-0.0091112},
{-0.0118404,0.0108797,-0.0163024},
{0.0004985,-0.1266681,0.0261460},
{-0.0020940,-0.1440091,0.0449523},
{0.0177144,-0.1524745,0.0479420},
{0.0211295,-0.1310793,0.0295349},
{0.0070258,-0.1488262,0.0216873},
{0.0000155,-0.1681540,0.0393630},
{0.0189608,-0.1785428,0.0454658},
{0.0252177,-0.1591008,0.0289949},
{0.0109303,-0.1732364,0.0185287},
{0.0026304,-0.1930517,0.0361459},
{0.0215753,-0.2048977,0.0424111},
{0.0278482,-0.1861811,0.0260647},
{0.0129090,-0.1996153,0.0158101},
{0.0043258,-0.2179210,0.0325267},
{0.0219151,-0.2315468,0.0398121},
{0.0295971,-0.2108044,0.0236158},
{0.0144619,-0.2252977,0.0133784},
{0.0029735,-0.2435960,0.0240714},
{0.0157713,-0.2573466,0.0355232},

{0.0310553,-0.2546717,0.0270406},
{0.0299889,-0.2346198,0.0189322},
{0.0180708,-0.2544391,0.0099553},
{0.0026476,-0.2697261,0.0205535},
{0.0124920,-0.2887214,0.0310101},
{0.0278871,-0.2790577,0.0320109},
{0.0302746,-0.2762983,0.0146254},
{0.0159596,-0.2849731,0.0077174},
{0.0022355,-0.3006016,0.0174315},
{0.0136804,-0.3177982,0.0297423},
{0.0287882,-0.3039867,0.0285883},
{0.0301255,-0.3053858,0.0111768},
{0.0143606,-0.3153809,0.0050377},
{0.0032499,-0.3297316,0.0166105},
{0.0110579,-0.3471124,0.0272136},
{0.0292601,-0.3327193,0.0252378},
{0.0291765,-0.3316432,0.0068321},
{0.0112801,-0.3462646,0.0027021},
{0.0028971,-0.3652682,0.0119085},
{0.0123365,-0.3746523,0.0268973},
{0.0287900,-0.3610570,0.0232626},
{0.0294099,-0.3551392,0.0044700},
{0.0171901,-0.3715528,-0.0031369},
{0.0043542,-0.3921637,0.0008470},
{0.0028225,-0.3951666,0.0202969},
{0.0245504,-0.3945411,0.0241466},
{0.0321315,-0.3836861,0.0091396},
{0.0254541,-0.3997006,-0.0053781},
{0.0071752,-0.4148073,-0.0074211},
{-0.0001866,-0.4199043,0.0101232},
{0.0131607,-0.4172965,0.0262343},
{0.0322174,-0.4152668,0.0123526},
{0.0252061,-0.4258013,-0.0091113},
{0.0050715,-0.4405668,-0.0133978},
{-0.0024173,-0.4474063,0.0046872},
{0.0018213,-0.4388897,0.0235594},
{0.0248305,-0.4380879,0.0232548},
{0.0316187,-0.4423559,0.0055632},
{0.0239706,-0.4516933,-0.0143149},
{0.0108647,-0.4683304,-0.0264493},
{-0.0057330,-0.4625942,-0.0148929},
{-0.0069683,-0.4778830,0.0031013},
{-0.0064183,-0.4669881,0.0219479},
{0.0113698,-0.4612565,0.0285707},
{0.0305747,-0.4640347,0.0195003},
{0.0292452,-0.4702827,-0.0001608},

Biology Open • Supplementary information

{0.0277969,-0.4805748,-0.0196663},
{0.0193534,-0.4982748,-0.0327522},
{0.0046666,-0.4856670,-0.0413047},
{-0.0102970,-0.4743441,-0.0319078},
{-0.0177936,-0.4855006,-0.0136990},
{-0.0065859,-0.5056765,-0.0035756},
{-0.0190911,-0.4950644,0.0119083},
{-0.0165806,-0.4860554,0.0324056},
{0.0023067,-0.4807216,0.0379353},
{0.0227863,-0.4862863,0.0302644},
{0.0385648,-0.4875928,0.0172344},
{0.0298202,-0.4957173,-0.0020123},
{0.0322833,-0.5127205,-0.0177065},
{0.0189620,-0.5283111,-0.0300134},
{0.0027691,-0.5125245,-0.0429506},
{-0.0147882,-0.4966170,-0.0434887},
{-0.0300277,-0.4888685,-0.0308183},
{-0.0297896,-0.5069788,-0.0130677},
{-0.0190168,-0.5288668,-0.0192423},
{0.0001700,-0.5285266,-0.0127671},
{0.0032728,-0.5255095,0.0081711},
{-0.0155860,-0.5222249,0.0151739},
{-0.0267896,-0.5071993,0.0308374},
{-0.0048456,-0.5039052,0.0437612},
{0.0144038,-0.5087115,0.0361448},
{0.0351350,-0.5102384,0.0265692},
{0.0293576,-0.5160776,0.0078345},
{0.0181677,-0.5238596,-0.0070675},
{-0.0001572,-0.5359196,-0.0327718},
{-0.0156816,-0.5254133,-0.0415447},
{-0.0325880,-0.5139966,-0.0347527},
{0.0212495,-0.5296133,0.0228039},
{0.0017008,-0.5327967,0.0299122},
{-0.0158594,-0.5272899,0.0364373},
{-0.0009149,-0.0862143,0.0209947},
{-0.0109685,-0.0750513,0.0227671},
{0.0104555,-0.0440262,0.0681348},
{-0.0150861,-0.0178894,0.0628027},
{-0.0018493,-0.0733360,0.0631372},
{-0.0066918,-0.0823546,0.0247709},
{-0.0014988,-0.1058188,0.0226200},
{-0.0074309,0.0021863,-0.0187435},
{-0.0132474,-0.0575575,0.0453683},
{0.0035127,-0.0343088,0.0722395},
{-0.0108011,-0.0850744,0.0283326},
{-0.0011343,-0.0699215,0.0202311},

Biology Open • Supplementary information

{-0.0140070,-0.0219582,0.0559790},
{0.0144733,-0.0388310,0.0662842},
{-0.0111259,-0.0385682,0.0437510},
{0.0151923,-0.0443840,0.0644285},
{-0.0009472,-0.0965920,0.0591783},
{-0.0157546,-0.0565476,0.0344463},
{-0.0148442,-0.0717058,0.0242322},
{-0.0172861,-0.0701331,0.0256326},
{-0.0026370,-0.0532981,0.0149574},
{0.0115144,-0.0747146,0.0263235},
{0.0230681,-0.0170890,0.0435927},
{-0.0033220,-0.0567768,0.0682340},
{0.0144226,-0.0036187,0.0189669},
{-0.0094236,-0.0627280,0.0208299},
{-0.0063527,-0.1042791,0.0488548},
{0.0059418,0.0190433,0.0144489},
{-0.0120438,0.0219102,-0.0066237},
{-0.0075221,-0.0866328,0.0522937},
{0.0083559,-0.1089627,0.0213618},
{0.0137802,-0.0955845,0.0267408},
{0.0082564,-0.0606791,0.0647079},
{0.0153911,-0.0162183,0.0083518},
{-0.0061598,-0.0302411,0.0050371},
{-0.0076663,-0.0012474,0.0220920},
{-0.0107706,-0.0722249,0.0538100},
{-0.0066063,-0.0336032,0.0723083},
{0.0089727,-0.0081593,0.0660412},
{0.0145133,-0.0648650,0.0325811},
{-0.0021408,-0.0232723,-0.0132542},
{-0.0147033,-0.0313853,0.0600410},
{0.0170852,0.0170754,-0.0077138},
{-0.0043285,0.0276707,0.0040187},
{0.0006734,-0.1091233,0.0573984},
{-0.0138556,-0.0507322,0.0543581},
{0.0211834,-0.0358743,0.0461573},
{0.0123570,-0.0550680,0.0262402},
{0.0190125,0.0133287,0.0078535},
{0.0219112,0.0026694,0.0073356},
{0.0229365,-0.0025406,-0.0037129},
{0.0146924,-0.0258005,0.0244788},
{0.0121957,-0.0444110,0.0233717},
{0.0173085,-0.0188283,0.0350179},
{-0.0185093,-0.0628157,0.0287859},
{0.0032027,-0.0491044,0.0700966},
{0.0179069,-0.0578371,0.0417155},
{0.0154779,-0.0129711,0.0144071},

Biology Open • Supplementary information

{0.0010931,0.0056250,-0.0210834},
{0.0092374,-0.0009773,-0.0209231},
{0.0110637,-0.0863127,0.0582883},
{0.0158010,-0.0779425,0.0553717},
{0.0185593,-0.0757284,0.0460421},
{0.0187716,-0.0876677,0.0389373},
{-0.0049990,-0.0956514,0.0266475},
{-0.0140444,0.0047758,0.0151509},
{0.0081722,-0.0014350,0.0598295},
{0.0203332,-0.0119559,0.0545361},
{0.0158527,-0.0302184,0.0652561},
{-0.0126074,-0.0456860,0.0274599},
{-0.0194332,-0.0095678,-0.0027686},
{0.0172905,-0.1056261,0.0296806},
{-0.0073807,-0.0073296,-0.0205941},
{0.0043518,0.0179895,-0.0160447},
{-0.0098812,-0.0421650,0.0173243},
{-0.0157280,0.0131569,-0.0112730},
{-0.0213468,0.0063273,-0.0017991},
{-0.0096483,-0.0893744,0.0428186},
{0.0123563,-0.0222095,-0.0010272},
{0.0132986,-0.0579206,0.0621292},
{0.0092318,-0.0235482,-0.0097323},
{0.0106986,-0.0177346,-0.0149163},
{0.0045812,-0.0756941,0.0205166},
{0.0095386,-0.0645711,0.0232457},
{0.0013036,-0.0968017,0.0217334},
{-0.0194058,-0.0714213,0.0326957},
{-0.0064514,-0.0017313,0.0564904},
{-0.0139889,-0.0391925,0.0644354},
{0.0191565,-0.0220843,0.0586151},
{0.0160273,-0.0979463,0.0522735},
{0.0200785,-0.0305499,0.0379942},
{0.0089325,-0.0405323,0.0720652},
{-0.0181927,-0.0041355,0.0107584},
{0.0154274,-0.0205407,0.0652223},
{0.0165691,-0.0779733,0.0353346},
{-0.0162746,-0.0638251,0.0244114},
{-0.0150479,-0.0424728,0.0570228},
{0.0148793,-0.0529995,0.0331312},
{-0.0083736,-0.0493134,0.0697723},
{0.0071107,-0.0098570,-0.0204330},
{0.0044338,-0.0196407,-0.0164317},
{-0.0154325,-0.0137329,0.0558168},
{-0.0111942,-0.0570043,0.0628209},
{0.0187040,-0.0688376,0.0512556},

{-0.0181112,0.0142224,0.0017009},
{-0.0148158,0.0116808,0.0126371},
{-0.0020432,-0.0866170,0.0594529},
{0.0142519,-0.0118303,0.0369975},
{0.0148994,-0.0044634,0.0598061},
{0.0163855,-0.0073162,0.0468677},
{-0.0137303,-0.0171410,-0.0093368},
{0.0061537,-0.0279773,0.0025546},
{0.0003965,-0.0624883,0.0185068},
{0.0215209,0.0039893,-0.0082488},
{0.0184462,0.0020698,-0.0143030},
{0.0137571,-0.0098312,-0.0171546},
{-0.0141188,0.0017494,-0.0167836},
{-0.0157653,-0.0076186,-0.0143816},
{0.0190303,0.0168655,0.0034836},
{0.0113710,0.0243188,-0.0024935},
{0.0097061,0.0176916,-0.0143733},
{0.0086732,-0.0288014,0.0701360},
{-0.0007647,-0.0110019,0.0691751},
{-0.0001784,-0.0026662,0.0623420},
{0.0057915,-0.0174888,0.0707662},
{0.0090432,0.0093698,0.0179997},
{0.0132729,-0.0221861,0.0170686},
{0.0042424,-0.0322320,0.0719592},
{-0.0098291,-0.0220308,0.0265815},
{-0.0110164,-0.0222662,0.0132693},
{-0.0069824,-0.0127517,0.0296816},
{-0.0162545,-0.0646723,0.0388299},
{-0.0122470,-0.0781818,0.0449450},
{-0.0074753,-0.0371083,0.0102747},
{0.0223435,-0.0235978,0.0507226},
{0.0132029,-0.0107955,0.0303771},
{0.0046655,-0.0012069,0.0266135},
{-0.0038686,-0.0969960,0.0578663},
{-0.0025960,0.0279888,-0.0035860},
{-0.0012338,-0.0168674,0.0429097},
{-0.0118052,-0.0183310,0.0027018},
{0.0139939,-0.0367876,0.0281245},
{0.0111506,-0.0346962,0.0187921},
{-0.0118813,-0.0088788,0.0577614},
{0.0202291,-0.0423344,0.0526139},
{-0.0130373,-0.0684151,0.0455748},
{-0.0080684,-0.1034050,0.0413178},
{0.0046795,-0.0067088,0.0338391},
{-0.0032275,-0.0058885,-0.0218984},
{-0.0136221,-0.0444293,0.0639519},

{-0.0032507,0.0113817,-0.0185123},
{-0.0046677,0.0212199,-0.0136713},
{0.0201016,-0.1097649,0.0398332},
{0.0039887,-0.0946598,0.0208753},
{0.0177812,-0.1132514,0.0489680},
{0.0103564,0.0245542,0.0059017},
{0.0126858,0.0117053,0.0159867},
{0.0011868,0.0192862,-0.0156886},
{0.0173657,-0.0149559,-0.0097037},
{0.0204508,-0.0097826,-0.0083215},
{-0.0093966,-0.0311738,0.0333711},
{-0.0117768,-0.0443300,0.0352746},
{-0.0144754,-0.0791794,0.0282177},
{-0.0015291,0.0034159,0.0220388},
{0.0044859,0.0093991,0.0190134},
{-0.0027167,-0.0036621,0.0281551},
{0.0002772,-0.0395648,0.0714133},
{0.0074502,-0.0557739,0.0657226},
{-0.0126177,-0.0255018,0.0688466},
{-0.0125885,-0.0338190,0.0680024},
{0.0097466,0.0260225,0.0029054},
{0.0184366,0.0181765,0.0015416},
{-0.0076815,-0.0140231,0.0700956},
{0.0164128,-0.0683035,0.0377133},
{-0.0133762,-0.0611170,0.0527210},
{-0.0161782,-0.0648141,0.0241493},
{0.0118100,-0.1083383,0.0555253},
{-0.0105586,-0.0324374,0.0194280},
{-0.0108832,-0.0197529,0.0186801},
{-0.0047687,0.0205542,0.0138631},
{0.0009635,-0.0875805,0.0613848},
{0.0063426,-0.0807452,0.0615735},
{-0.0024325,-0.0803448,0.0610194},
{0.0178496,-0.0946778,0.0340826},
{-0.0134144,-0.0235066,0.0537114},
{0.0114902,-0.0319387,0.0189111},
{0.0055501,-0.0674527,0.0642824},
{0.0089425,-0.0450797,0.0683440},
{0.0102718,-0.1082757,0.0217767},
{-0.0072985,-0.0679406,0.0624138},
{0.0193015,-0.0549711,0.0482392},
{-0.0128326,-0.0549485,0.0232015},
{0.0228116,-0.0264671,0.0410375},
{-0.0043374,-0.0782265,0.0222569},
{0.0179219,-0.0025546,0.0139998},
{0.0066445,0.0069310,0.0191771},

{0.0009859,-0.0415952,0.0712842},
{-0.0159561,0.0175922,-0.0055616},
{0.0114181,0.0200947,0.0111374},
{-0.0114867,0.0205028,0.0089365},
{-0.0200307,-0.0070363,-0.0056585},
{-0.0201501,0.0006508,-0.0081168},
{-0.0052754,-0.0223864,0.0727297},
{-0.0141540,-0.0439267,0.0476863},
{0.0060138,-0.0599376,0.0656916},
{0.0133850,-0.0532319,0.0632333},
{-0.0094092,-0.0873197,0.0278108},
{0.0135972,0.0090607,0.0164632},
{0.0196264,-0.0136695,-0.0012290},
{-0.0186484,-0.0102705,0.0054782},
{-0.0137518,-0.0718465,0.0422572},
{-0.0078720,-0.0215041,-0.0040670},
{-0.0139249,-0.0351654,0.0511134},
{0.0050087,-0.0955410,0.0596882},
{0.0171957,-0.0636853,0.0562709},
{-0.0018680,-0.0444610,0.0119652},
{0.0067254,-0.0408292,0.0142620},
{0.0202096,-0.0088885,0.0062717},
{0.0195139,-0.1038502,0.0452464},
{-0.0098318,-0.0241386,0.0272405},
{-0.0009461,0.0165201,0.0174337},
{0.0079707,-0.0870168,0.0229616},
{0.0094671,-0.0795175,0.0608747},
{0.0161704,-0.0165265,-0.0102191},
{0.0005985,-0.0116018,0.0696906},
{-0.0076976,-0.0187946,-0.0153220},
{0.0171116,-0.0898354,0.0508276},
{0.0082767,-0.0234663,-0.0111835},
{-0.0114267,-0.0821388,0.0251080},
{0.0143299,-0.0500931,0.0313752},
{0.0167030,-0.0054491,0.0582343},
{0.0051067,-0.0097299,0.0424486},
{-0.0032682,-0.0786616,0.0616219},
{-0.0137817,-0.0758180,0.0411519},
{-0.0155581,-0.0145477,0.0582731},
{-0.0084597,0.0156202,-0.0157983},
{-0.0184801,0.0073657,0.0087878},
{0.0100615,-0.0866749,0.0239382},
{0.0026842,-0.0494810,0.0691125},
{-0.0145335,-0.0275818,0.0592792},
{0.0069311,-0.0078011,-0.0211891},
{0.0165801,-0.0359251,0.0609343},

Biology Open • Supplementary information

{0.0128759,-0.0190354,0.0668639},
{0.0112144,0.0026285,-0.0195168},
{0.0147522,-0.0102886,0.0185344},
{-0.0139264,0.0140301,0.0126057},
{0.0111485,-0.0665659,0.0620682},
{-0.0058822,-0.0483400,0.0706606},
{0.0209239,-0.0365782,0.0539652},
{0.0170541,-0.0166498,-0.0075588},
{-0.0160579,-0.0631608,0.0393704},
{-0.0081266,-0.0927392,0.0488112},
{0.0034431,-0.0661825,0.0650795},
{-0.0047506,-0.0520758,0.0151359},
{0.0119845,0.0134394,-0.0157804},
{-0.0023064,-0.0271600,-0.0009118},
{-0.0098655,-0.0921214,0.0326456},
{-0.0111151,-0.0044004,-0.0189783},
{-0.0031382,0.0272286,-0.0050486},
{-0.0103178,-0.0272784,0.0143311},
{-0.0115594,-0.0809236,0.0249163},
{-0.0063999,-0.0938560,0.0268258},
{0.0010258,0.0148891,-0.0180532},
{0.0213578,-0.0326332,0.0531351},
{-0.0063450,-0.0728281,0.0215260},
{0.0189760,-0.0455848,0.0429423},
{0.0189485,-0.0386411,0.0382592},
{-0.0154563,-0.0150130,-0.0010603},
{0.0142855,0.0175906,0.0109800},
{-0.0144109,-0.0136922,0.0075414},
{-0.0115164,-0.0441624,0.0389289},
{0.0193335,-0.1100742,0.0462104},
{0.0190358,-0.0129612,0.0606599},
{0.0202050,-0.0195156,0.0561969},
{0.0014663,-0.0332045,0.0054702},
{0.0116203,-0.0292805,0.0684369},
{-0.0030041,-0.0119567,0.0342027},
{-0.0207616,-0.0053178,-0.0018937},
{0.0029700,-0.0263264,-0.0041499},
{-0.0104299,-0.0890926,0.0402546},
{-0.0137189,-0.0344138,0.0663591},
{0.0192686,0.0064975,-0.0117229},
{-0.0159073,-0.0779468,0.0328122},
{-0.0164048,-0.0052561,0.0139331},
{0.0129177,-0.0100588,0.0294635},
{-0.0144644,-0.0536188,0.0348986},
{0.0162058,-0.0895463,0.0313522},
{0.0008286,0.0042153,-0.0213586},

{0.0181812,-0.0969402,0.0342089},
{0.0074764,0.0079809,0.0183750},
{-0.0153846,0.0028261,0.0141084},
{0.0063007,-0.0536709,0.0180864},
{-0.0069736,-0.0237891,0.0370683},
{0.0221325,-0.0337028,0.0468232},
{-0.0138997,-0.0782547,0.0250537},
{0.0224614,0.0063928,-0.0033183},
{-0.0149449,-0.0784620,0.0305272},
{0.0121332,-0.0279607,0.0121704},
{0.0212607,-0.0146144,0.0499511},
{0.0093304,0.0024316,-0.0205487},
{0.0158922,-0.0103787,0.0148958},
{-0.0122173,-0.0794196,0.0442360},
{0.0004991,0.0257305,0.0101421},
{0.0030619,0.0041277,-0.0217087},
{0.0406651,-0.5036634,0.0231980},
{0.0055110,0.0254960,-0.0085951},
{-0.0147251,-0.0562174,0.0411984},
{-0.0342206,-0.4985627,-0.0297253},
{0.0025259,0.0288442,-0.0000353},
{0.0156668,-0.0306457,0.0293778},
{-0.0153888,-0.0013741,-0.0160541},
{0.0180459,-0.0386979,0.0585601},
{-0.0159746,0.0167960,0.0052405},
{0.0188135,-0.0500625,0.0442547},
{0.0167904,-0.0558496,0.0379592},
{-0.0144684,-0.0440530,0.0505882},
{0.0188917,-0.0707384,0.0486875},
{-0.0182351,-0.0614707,0.0303838},
{0.0033317,-0.0702287,0.0642511},
{0.0011856,-0.0655139,0.0191884},
{0.0233416,-0.0266946,0.0486720},
{0.0086179,-0.0975491,0.0582414},
{-0.0064838,-0.1059057,0.0308715},
{-0.0206864,-0.0027485,0.0033745},
{0.0156800,-0.0171113,0.0218149},
{0.0150809,-0.0850253,0.0303540},
{-0.0101240,-0.0315210,0.0304006},
{-0.0212391,-0.5111690,0.0394447},
{-0.0094246,-0.0711873,0.0576415},
{-0.0124707,-0.5042581,-0.0113553},
{0.0116228,-0.0551908,0.0247419},
{0.0118126,0.0185244,-0.0123643},
{-0.0265194,-0.5000972,0.0319165},
{0.0170255,-0.0678023,0.0386866},

{0.0003247,-0.0632243,0.0661160},
{-0.0119512,0.0230839,0.0031416},
{-0.0026848,-0.0213040,0.0725755},
{-0.0079370,-0.0094019,0.0266354},
{0.0210842,-0.0131477,0.0466213},
{0.0161465,-0.0312474,0.0631616},
{-0.0153748,-0.0139155,0.0574647},
{0.0215495,0.0105078,-0.0030995},
{-0.0109000,-0.0435296,0.0197950},
{-0.0003662,-0.0180189,-0.0181506},
{-0.0117158,-0.0768442,0.0487650},
{-0.0097902,0.0218480,0.0088541},
{0.0106128,0.0244224,-0.0033133},
{-0.0145633,-0.0344964,0.0629306},
{-0.0065020,-0.5007203,0.0447106},
{-0.0146011,-0.5075281,-0.0419058},
{-0.0145201,-0.5074600,-0.0410396},
{-0.0144391,-0.5073919,-0.0401735},
{-0.0143581,-0.5073239,-0.0393073},
{-0.0142771,-0.5072558,-0.0384411},
{-0.0141961,-0.5071877,-0.0375750},
{-0.0141151,-0.5071196,-0.0367088},
{-0.0140341,-0.5070515,-0.0358426},
{-0.0139531,-0.5069835,-0.0349765},
{-0.0138721,-0.5069154,-0.0341103},
{-0.0137911,-0.5068473,-0.0332441},
{-0.0137102,-0.5067792,-0.0323780},
{-0.0136292,-0.5067112,-0.0315118},
{-0.0135482,-0.5066431,-0.0306457},
{-0.0134672,-0.5065750,-0.0297795},
{-0.0133862,-0.5065069,-0.0289133},
{-0.0133052,-0.5064388,-0.0280472},
{-0.0132242,-0.5063708,-0.0271810},
{-0.0131432,-0.5063027,-0.0263148},
{-0.0130622,-0.5062346,-0.0254487},
{-0.0129812,-0.5061665,-0.0245825},
{-0.0129002,-0.5060985,-0.0237163},
{-0.0128193,-0.5060304,-0.0228502},
{-0.0127383,-0.5059623,-0.0219840},
{-0.0126573,-0.5058942,-0.0211179},
{-0.0125763,-0.5058261,-0.0202517},
{-0.0124953,-0.5057581,-0.0193855},
{-0.0124143,-0.5056900,-0.0185194},
{-0.0123333,-0.5056219,-0.0176532},
{-0.0122523,-0.5055538,-0.0167870},
{-0.0121713,-0.5054858,-0.0159209},

Biology Open • Supplementary information

{-0.0120903,-0.5054177,-0.0150547},
{-0.0120093,-0.5053496,-0.0141885},
{-0.0119284,-0.5052815,-0.0133224},
{-0.0118474,-0.5052134,-0.0124562},
{-0.0117664,-0.5051454,-0.0115900},
{-0.0116854,-0.5050773,-0.0107239},
{-0.0116044,-0.5050092,-0.0098577},
{-0.0115234,-0.5049411,-0.0089916},
{-0.0114424,-0.5048731,-0.0081254},
{-0.0113614,-0.5048050,-0.0072592},
{-0.0112804,-0.5047369,-0.0063931},
{-0.0111994,-0.5046688,-0.0055269},
{-0.0111184,-0.5046007,-0.0046607},
{-0.0110375,-0.5045327,-0.0037946},
{-0.0109565,-0.5044646,-0.0029284},
{-0.0108755,-0.5043965,-0.0020622},
{-0.0107945,-0.5043284,-0.0011961},
{-0.0107135,-0.5042604,-0.0003299},
{-0.0106325,-0.5041923,0.0005362},
{-0.0105515,-0.5041242,0.0014024},
{-0.0104705,-0.5040561,0.0022686},
{-0.0103895,-0.5039880,0.0031347},
{-0.0103085,-0.5039200,0.0040009},
{-0.0102275,-0.5038519,0.0048671},
{-0.0101466,-0.5037838,0.0057332},
{-0.0100656,-0.5037157,0.0065994},
{-0.0099846,-0.5036477,0.0074656},
{-0.0099036,-0.5035796,0.0083317},
{-0.0098226,-0.5035115,0.0091979},
{-0.0097416,-0.5034434,0.0100640},
{-0.0096606,-0.5033753,0.0109302},
{-0.0095796,-0.5033073,0.0117964},
{-0.0094986,-0.5032392,0.0126625},
{-0.0094176,-0.5031711,0.0135287},
{-0.0093366,-0.5031030,0.0143949},
{-0.0092557,-0.5030350,0.0152610},
{-0.0091747,-0.5029669,0.0161272},
{-0.0090937,-0.5028988,0.0169934},
{-0.0090127,-0.5028307,0.0178595},
{-0.0089317,-0.5027626,0.0187257},
{-0.0088507,-0.5026946,0.0195918},
{-0.0087697,-0.5026265,0.0204580},
{-0.0086887,-0.5025584,0.0213242},
{-0.0086077,-0.5024903,0.0221903},
{-0.0085267,-0.5024223,0.0230565},
{-0.0084457,-0.5023542,0.0239227},

```
                    {-0.0083648,-0.5022861,0.0247888},
                    {-0.0082838,-0.5022180,0.0256550},
                    {-0.0082028,-0.5021500,0.0265212},
                    {-0.0081218,-0.5020819,0.0273873},
                    {-0.0080408,-0.5020138,0.0282535},
                    {-0.0079598,-0.5019457,0.0291196},
                    {-0.0078788,-0.5018776,0.0299858},
                    {-0.0077978,-0.5018096,0.0308520},
                    {-0.0077168,-0.5017415,0.0317181},
                    {-0.0076358,-0.5016734,0.0325843},
                    {-0.0075548,-0.5016053,0.0334505},
                    {-0.0074739,-0.5015373,0.0343166},
                    {-0.0073929,-0.5014692,0.0351828},
                    {-0.0073119,-0.5014011,0.0360490},
                    {-0.0072309,-0.5013330,0.0369151},
                    {-0.0071499,-0.5012649,0.0377813},
                    {-0.0070689,-0.5011969,0.0386474},
                    {-0.0069879,-0.5011288,0.0395136},
                    {-0.0069069,-0.5010607,0.0403798},
                    {-0.0068259,-0.5009926,0.0412459},
                    {-0.0067449,-0.5009246,0.0421121},
                    {-0.0066639,-0.5008565,0.0429783},
                    {-0.0065830,-0.5007884,0.0438444}
                    };
            BoundingBoxOnOff = Off;
        };
        AnyFunTransform3DIdentity ScaleFunction = {
            PreTransforms = {&.RBFTransform};
};
};

};
};
```

Biology Open • Supplementary information
